# The flounder effect: disparities in taxonomic and ecological study intensity across extant and fossil marine organisms hamper conservation

**DOI:** 10.1038/s44185-025-00118-1

**Published:** 2026-01-31

**Authors:** Brendan M. Anderson, James C. Lamsdell, Amanda R. Falk, Curtis R. Congreve, Jonathan R. Hendricks

**Affiliations:** 1 Department of Geosciences, Union College, F.W. Olin Center, Schenectady, USA; 2 Department of Geology and Geography, West Virginia University, Morgantown, WV USA; 3 Department of Biology, Centre College, Danville, KY USA; 4 Marine, Earth & Atmospheric Sciences Department, North Carolina State University, Raleigh, NC USA; 5 Milwaukee Public Museum, Milwaukee, WI USA; 6 Present Address: Department of Earth and Atmospheric Sciences, SUNY Oneonta, Oneonta, NY USA

**Keywords:** Research data, Palaeoecology, Conservation biology, Zoology, Biodiversity, Palaeoecology, Careers

## Abstract

Large datasets have allowed biologists and palaeontologists to investigate a multitude of ecological processes. They have also obfuscated the ways in which our limited knowledge of ecology can affect our results. We focus on how our biased understanding of organismal natural history and taxonomy can have significant impacts on our perspective of ecological and evolutionary processes across multiple temporal and hierarchical scales, and suggest broad structural solutions to this problem.

## Introduction

A key goal of modern biology is to determine general patterns of organismal response to environmental or ecological changes, a goal with direct implications for shaping our efforts to preserve biodiversity and ecosystem functioning under an anthropogenically altered climate. There is an associated need to expand the taxonomic scope of ecological studies. However, the magnitude of the task (with some 8.7 million eukaryotic species estimated to exist^1^) in combination with budgetary and temporal constraints renders a complete understanding of ecological interactions across all life beyond our ability to document. Therefore, to conduct any analyses of the interrelationship between evolutionary history and ecology, we must make approximations based on the data that are currently available. It is our perspective that there are disparities in past and present taxonomic effort (e.g. work to identify specimens, diagnose species, communicate about organisms and determine phylogenetic relationships) that bias the data available in existing databases, resulting in significant impacts on our understanding of biodiversity and ecology. These biases have potential downstream effects on how we interpret and understand the reaction of the biosphere to current and future environmental changes.

While we have entered a period of advancement in ‘big data’ biology and palaeontology, our ecological, taxonomic and physiological datasets still represent a limited and potentially biased sampling of the natural world. Therefore, we caution against considering our present understanding of organismal natural history and taxonomic sampling sufficiently complete to focus research support solely on meta-analyses^2,3^. For example, the spatial distribution of fossil sampling has been impacted by a wide variety of geologic and historically contingent factors and this structures our understanding of fossil ecosystems in important and potentially misleading ways^4–6^. Modern biodiversity data are impacted by similar concerns—a relatively small portion of the globe (<7%) has been sampled for animals in large databases, with marine, high-altitude, tropical, and deep-sea regions severely undersampled^7^. Vertebrates are known to be overrepresented in large datasets^8^, and greater data availability has increased the degree to which vertebrates are overrepresented when compared to invertebrates^7^. For marine animal taxa, these limitations may be reflected in patterns of citation; studies of terrestrial ecologies are frequently cited by studies of marine systems, but the reverse is often not the case^9,10^. This can exacerbate the unconscious assumption that terrestrial ecological systems function as a default normal system^9,10^. Basing our understanding on a limited subset of the world’s biota does not necessarily mean our data are unrepresentative, however, the non-random assignment of research intensity suggests our ability to properly extrapolate natural history information from well-studied systems is likely to vary substantially among taxa.

The use of limited natural history observations causes propagation of errors into ecological and evolutionary theories and conservation efforts^11^. Benthic marine invertebrates are some of the most important components of the fossil record, but extant examples are frequently understudied. While marine communities are currently undergoing rapid compositional changes compared with terrestrial counterparts^12^, the first comprehensive red list assessment of any marine gastropod genus did not occur until the 2010s^13^. Even commentaries highlighting the need for additional effort in studying invertebrate organisms may not mention marine systems at all (e.g. Eisenhauer et al.^14^). Understanding basic life history parameters (e.g. lifespan, growth rates, reproductive age) is important for devising appropriate conservation strategies (e.g. Abhijith and Mukherjee^15^; Herbert et al.^16^), but these data can be time and effort-intensive to collect and are presently taxonomically biased^17,18^.

To compensate for this lack of species-specific information, we tend to assume that closely related taxa are more likely to share similar ecological and environmental preferences^19–22^, however the accuracy of our estimations based on niche conservatism is only as good as the depth of our sampling. Generalisation of data across broader phylogenetic distances can be prominently observed in the way we use ecological information from a few well-studied organisms to infer the ecology or environmental tolerances of whole clades (e.g. Hughes et al.^7^). The assumption that ecological traits do not vary among similar taxa may impact our interpretation of palaeocological patterns or macroevolutionary trends^23,24^. However, general rules may not apply across higher taxonomic ranks when derived from a limited subset of taxa^25^. The degree of extrapolation, and our confidence in such extrapolations, should be stated explicitly when utilising these estimates. Until we determine the veracity of these estimates with additional studies within closely related taxa, we do not know the extent to which our estimates are likely to be accurate.

Studies of the Earth’s modern and ancient biota are known to be unevenly distributed taxonomically, however, the extent and potential consequences of this taxonomic worker-effort bias have not been previously quantified. In this perspective paper, we conduct a preliminary investigation of datasets using both modern and fossil biological data and show that similar biases impact these disparate datasets, which illustrates that these biases are due to structural issues within science and society, rather than concerns unique to specific biological data. Instead, these biases are related to a broader issue with how biological sciences in general treat taxonomic data and the people who work to acquire said data. Since these are largely biases based on human systems, there are reasonable solutions that can ameliorate many of these concerns, and we highlight these here. Addressing these concerns soon is vital if we wish to be able to generate the appropriate data required to properly plan and adapt conservation efforts for a changing Earth system.

### A few good species and the ecological flounder effect

The number of scientific publications on different higher taxa does not reflect their diversity. For example, the volume of papers on threatened large mammal species overwhelmingly eclipses those on other threatened vertebrates^26^. Focusing research effort on a small number of species could be problematic if the ecological or physiological information gained is incorrectly presumed to be widely applicable across a clade (e.g. Cope’s rule doesn’t apply to many invertebrate groups^27–29^).

**Fig. 1:**
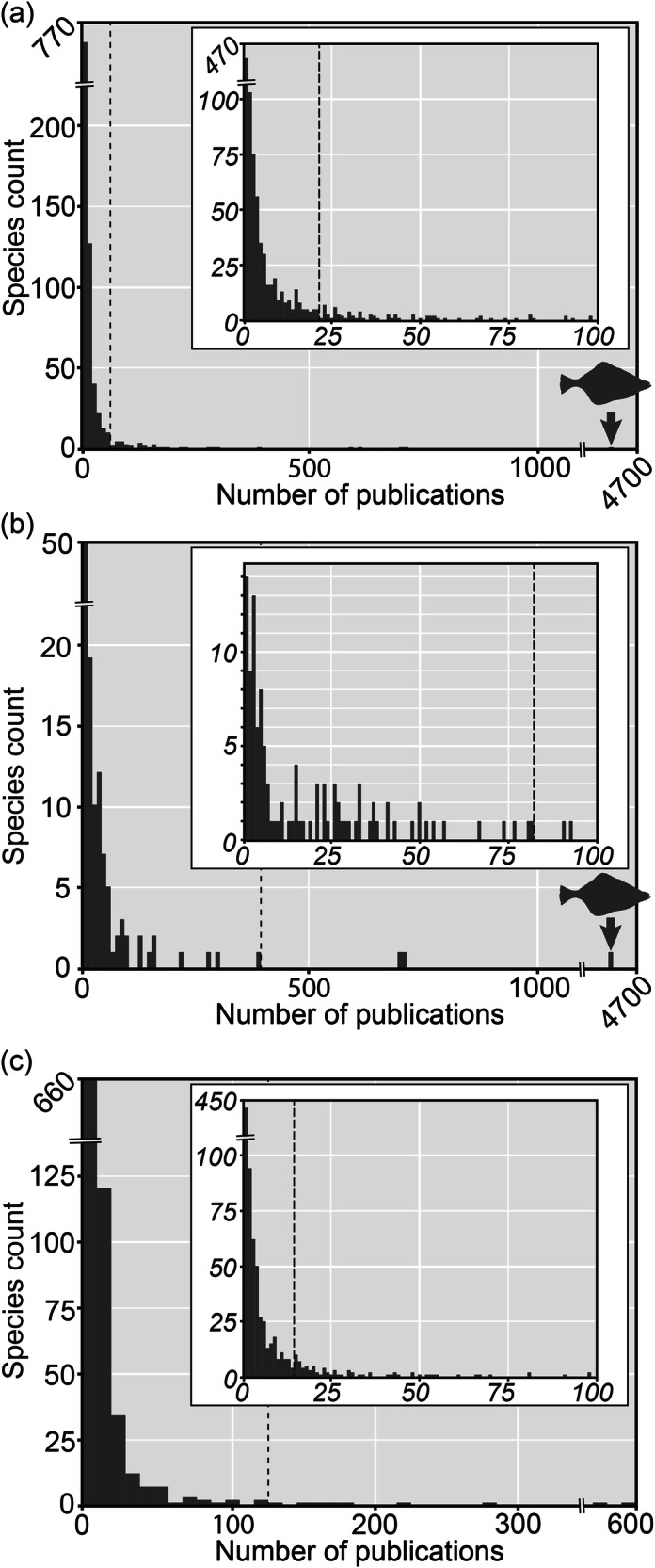
Histograms showing the number of papers published per species of a random sampling of marine taxa. Data presented in this figure are shown as **a** the whole dataset, **b** data for fisheries taxa only, and **c** data for non-fisheries taxa only. The main histogram in each panel displays all species in the respective subsample, with a bin size of ten publications. The dashed line represents the 99th percentile of the data. The smaller inset within each panel shows the species within the relevant dataset receiving fewer than 100 citations, with a bin size of one publication and the dashed line indicating the 95th percentile for the data. A major outlier within the dataset, the English Sole (*Parophrys vetulus*, Gerard 1854), is indicated. Note the break in both axes in all graphs and the difference in scale between (**b**, **c**).

To illustrate the possible impact of worker effort bias on ecological studies, we conducted a random sampling of over 1000 animal genera from the World Register of Marine Species (WoRMS)^30^ and assessed the number of publications associated with constituent species. WoRMS is an authoritative list of taxonomic names and synonymies rather than a version-controlled database. In order to compile a randomly sampled list, species were randomly pulled through random number generators, and then we searched every English language article that mentioned these species through Google Scholar to determine how many papers have been published and verified that these articles contained ecological information (i.e. range, environment, physiology, etc.). This resulted in sampling 126 genera, containing a total of 1026 species. Of the species sampled in this analysis, the modal number of papers per species is one (Fig. 1; Supplemental datasets 1 and 2). There is a sharp decline in the number of species that have more than 2 publications; over 80% of species have fewer than 10 papers. Less than 5% of our sample has over 50 papers published for a given species. Even for these, the primary ecological information in these publications may only be occurrence data rather than new ecological information.

The taxa that do have larger numbers of associated publications are those that are commercially traded, are congeneric with targeted species, or are parasites to fished species. These taxa have a proportionally higher rate of study when compared to taxa that are not associated with fisheries (Fig. 1b, c). Only one species in our sample has been the focus of more than 1000 publications: *Parophrys vetulus* Girard, 1854^31^, the English Sole. This is a commercially important flatfish with major fisheries in Washington, Oregon and California, with over 4600 publications to its name (the second-most published taxon was *Gerres filamentosus* Cuvier, 1829^32^, another species caught by humans). Among the sampled species not commercially fished (either for food or the aquarium trade), the majority with a publication count greater than 20 are either congeners of commercially fished species or are potentially hazardous to human life or deleterious to commercially important species (e.g. parasites). This relationship makes intuitive sense, as there are socioeconomic incentives to studying food sources and economically valuable ornamental taxa. We refer to this phenomenon, including the tendency to continue to focus efforts on previously researched species, as the ‘flounder effect’, named in part due to our first trial of this experiment becoming inundated with papers after randomly sampling the economically important flatfish genus *Arnoglossus* Bleeker, 1862^33^. The 12% of species in our study that were considered economically relevant made up 47% of the species with over 50 publications. Our sample dataset not only suggests that a majority of the biosphere is poorly studied, but also that there are significant biasing factors for the few species that have been strongly represented in our ecological literature.

Our collective research output is preferentially focused on a small number of species, meaning that few species have been examined sufficiently to generate representative natural history data. As most species have not been studied for a wide range of evolutionary, ecological, or physiological parameters, scientists often need to extrapolate information from the available data using the few well-sampled species as model organisms or exemplars for phylogenetically distant relatives. Sampling effort tends to be focused towards species that are commercially viable (e.g. Jarić et al.^34^; Pimental et al.^35^), publicly appealing/charismatic^36–38^, or medically important^39,40^. Focusing intensely on only a few species to act as exemplars may have significant impacts on our conservation efforts, as well as our general understanding of the anatomy and physiology of taxa. Different commercially important fisheries have exhibited varying responses to historical warming in both direction and magnitude of response^41^. While tropical marine animals are sometimes considered especially vulnerable to climate warming^42^, this is not always the case^43^. This represents a key area where broadening the taxonomic and physiologic sampling of our studies may have significant consequences^43,44^.

### The trouble with turrids: understudied, underidentified, unincluded

Taxonomic effort is unevenly distributed among species and the downstream consequences of such disparities in human attention are seldom explicitly stated as areas of concern in big-data studies or conservation assessments^45^. A frequent first-pass form of data cleaning when using community resources like the Paleobiology Database (PBDB) is the removal of occurrences that are not identified to species level (e.g. Janevski and Baumiller^46^, Xue et al.^47^, Plotnick and Wagner^48^). We agree that this is a good policy under most circumstances (even when analyses are conducted at the genus level, accepting occurrences with species-level identification may exclude material which is of poor quality or which may be more likely to have been improperly identified), but wished to explore whether the failure to assign a name (accurately or not) is randomly or evenly distributed across clades. We focused on marine gastropods and bivalves from the richly fossiliferous Plio-Pleistocene of the southeastern United States. We first examined the PBDB to establish whether some taxa were disproportionately under-determined. This is not a measure of the accuracy of these identifications, just a measure of how frequently a species-level assignment has been attempted. We then examined neogastropod specimen records in the online database collections of the Florida Museum of Natural History to compare the rates at which species assignments were made therein.

The PBDB study sample included 51 gastropod families with 15 or more occurrences (records), representing 4529 total occurrences across 1092 species epithets. The average gastropod family had 85% of records identified to the species level. The gastropod families with the lowest species-level identification rate were the Pyramidellidae (54%), Turridae (56%, 11 known only to the family), Marginellidae (65%), Pseudomelaniidae (68%), and Trochidae (69%).

**Fig. 2:**
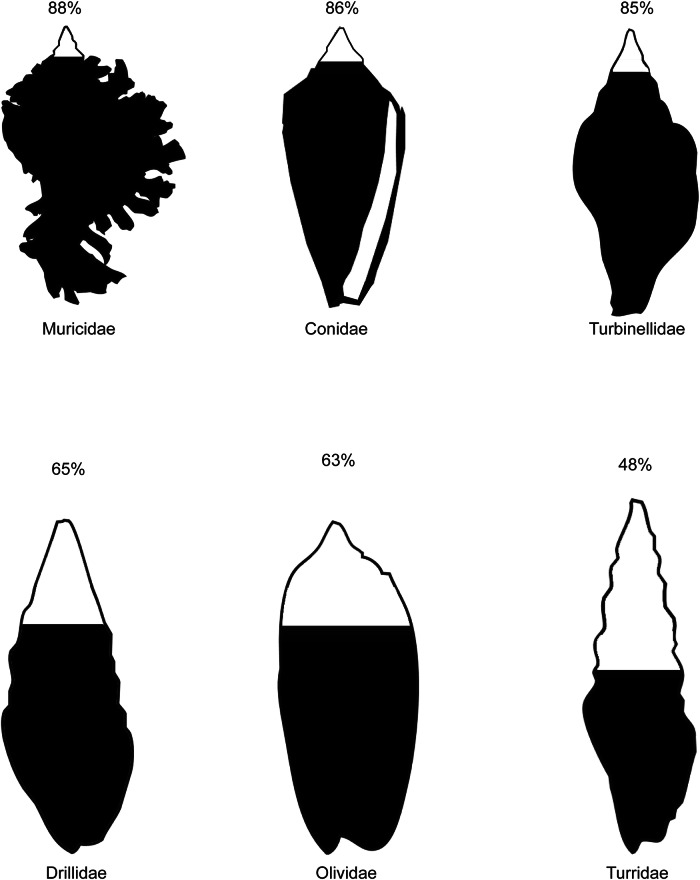
Percent of lots assigned a species-level identification for the three neogastropod families with the highest percentages of species identifications and lowest percentages of species identification in the online Specify database of the Florida Museum of Natural History. Graphical representations show the percent identified to species level as the portion filled vertically (not as a filled area).

In the UF Specify database, 76% of lots are assigned to a species, however, only 48% of 1,986 lots assigned to ‘Turridae’ are confidently identified to a species, with only 69% receiving even a generic assignment. This represents the lowest identification rate among neogastropods, followed by Olividae, Costellariidae, Drillidae, Terebridae, and Mangellidae. (Fig. 2 and Supplemental dataset 3). Notably, the Drillidae, Terebridae, and Mangellidae are all members of the venomous Conoidea clade, and both Mangellidae and Drillidae have also classically been considered ‘turrids’^49^.

The species-ID rates of neogastropod families in the PBDB and FLMNH datasets are significantly correlated (Spearman’s *ρ* = 0.59, *p* < 0.013), indicating that the same groups have been consistently problematic (Supplemental dataset 4). Further, Turridae has the lowest species identification rate in both datasets. Species richness in each family is also correlated (Spearman’s *ρ* = 0.57, *p* = 0.014), however the number of species assigned to Turridae in the PBDB and FLMNH databases differs substantially, with only 12 and 87 species, respectively, in the PBDB and FLMNH datasets. Removing Turridae from the analysis greatly affects the correlation and level of significance, increasing *ρ* to 0.72 (*p* = 0.001).

Depending on whether diversity estimates are made based on synonymy rates from local monographed Conidae^50^ or turrids from other depositional settings (e.g. Panama^51,52^), the approximately 170 named turrid species present in the Plio-Pleistocene of Florida could be estimated to potentially represent anywhere from 19 to 15,000. Our inability to determine the order of magnitude or even direction in which our diversity estimates are likely skewed must be considered when attempting to determine ecological changes or extinction and diversification rates. The exclusion of records not identified to species level is not a clade-independent decision^53^ and is likely to disproportionately affect groups like turrid snails, which are frequently small in size (see McClain et al.^54^) and have been considered extremely taxonomically challenging^55–57^. We encourage those conducting big-data analyses to consider whether the disproportionate removal of occurrences in some groups over others may have downstream consequences, or consider statistical adjustments for the disproportionate removal of some records due to taxonomic uncertainty.

### Broader impacts of extrapolating from limited data

Both the limited ecological study of species which are not presently viewed as commercially important (the flounder effect) and the neglect of some clades with respect to taxonomic work (for a variety of causes) lead to limitations on our understanding of the natural world, either due to human favouritism or aversion. The two examples described above represent extreme ends of a spectrum of worker effort that are often caused by a multitude of social and economic factors. In addition to the biases described above, taxonomic understanding of extant and fossil taxa is known to be biased towards regions with long histories of such study, high GDP countries, towards charismatic vertebrate taxa, and towards terrestrial taxa^7,58,59^. Ecological knowledge of extant marine taxa is also more limited for practical reasons as well related to sampling logistics^7,10^. Assessment of extinction risk in extant organisms is biased towards groups that are best described taxonomically, which also tend to be large species^54,60^. This leads to relatively low rates of assessment for IUCN red list status for marine invertebrates, primarily focused on economically or medically important groups like cuttlefish, lobsters, abalone, scleractinian corals, and cone snails^13,61,62^. In contrast, a wide variety of bony fish and elasmobranchs have been assessed^61,62^. Chen^63^ found 11,000 marine vertebrate species had been assessed, with around 20% rated as data deficient, compared with only 3000 invertebrates, one-third of which were rated as data deficient, despite invertebrates making up over 90% of marine species. From an ecological perspective, it is notable that while molluscs are the most diverse marine animal group, many modern marine mollusc species have been described from empty shells and so all ecological information is necessarily being extrapolated from relatives, and even their status as extant must be inferred^59,64–66^.

While no dataset will ever be truly ‘complete’, we stress that meta-analyses and big-data studies should still be supplemented with continued work to improve our current data through smaller taxonomic studies (e.g. identifying and describing new species), as well as refinement of our existing online datasets. This does not mean that meta-analyses should not be undertaken or that such studies should be called in question, but rather that we must not systemically favour meta-analysis over the very important smaller works that build those datasets^2^. As discussed above, there are known gaps in our current understanding of the natural world (both modern and fossil), which in turn could have unintended consequences on our understanding not just of the groups being studied, but potentially broader evolutionary and ecological theory and conservation efforts.

Although it is recognised that niche conservatism is intrinsically tied to phylogenetic distance, there remains a phenomenon in the literature of assuming that human-defined higher taxa maintain some form of inherent ecology (see Hendricks et al.^67^). Part of this tendency can be attributed to an averaging of ecologies across a clade, and part of this can be explained by a long tradition of strongly linking ecology and higher taxa^68^. Modern environmental climate tolerances of organisms, often inferred from presently occupied ranges, can also prove to be unrepresentative of the fundamental niche of organisms or conditions once occupied by members of a species, as inferred from taxonomy-independent environmental data. Increasingly, we are beginning to recognise distinct, non-analogous ecologies and life habits among stem lineages of clades whose modern descendants show minimal variation in morphology and ecology (e.g. horseshoe crabs^69–71^ and edible whelks^72–75^). This further underlines the importance of proper taxonomic sampling, as improving our taxonomic sampling will, in turn, improve the quality of these types of environmental models.

Biodiversity conservation efforts based on data obtained in relatives of well-studied species, which have fundamentally different ecological requirements and life history traits, will not succeed in meeting their goals. Broad generalisation about environmental tolerances or life history strategies of related taxa are often subject to exceptions (e.g. turritellid gastropods are frequently considered to prefer siliciclastic substrates and fully marine environments, and to live only one or two years, but there are species which are exceptions to each of these ecological parameters^76–81^). Species may even exhibit differing ecological requirements throughout ontogeny^82,83^. Further, generalising from ‘typical’ ecologies or environmental tolerances derived from a limited understanding of the diversity of ecologies represented by a clade can lead to forcibly grouping species of concern into incorrect or unsuitable ecological categories, or improperly assuming other life history characteristics such as incubation or maturation times^16,81,84–86^, interfering with conservation or fisheries management efforts. For example, Greenland sharks (*Somniosus microcephalus* Bloch and Schneider, 1801^87^) are caught for human consumption, but their age of reproductive maturity (~134 years) was only determined in 2016^84,88^. Without an understanding of population sizes, environmental requirements, and reproductive parameters for a species, we cannot properly evaluate what would constitute sustainable fishing practices.

### Floundering forward

Calls for supporting natural history and taxonomic study have remained fairly consistent in their suggestions for improving the state of taxonomic work^45,53,58,89–91^ and focusing on under-researched taxa and regions^7^, but these have not yet been operationalised. It is no surprise that natural history and taxonomic information are structured significantly for historically contingent reasons or differences in accessibility of organisms, as well as vagaries of human interest (e.g. favouring visually appealing birds^38^), and there have been other recent calls to recognise the importance of gathering new natural history information^92–94^ and making museum specimen data more available^95^. There are, however, structural changes within academic systems that can be made to create an environment more favourable towards the primary research that builds our natural history datasets.

### 1) Identify our unknowns

In meta-analyses, identify sources of data structuring and what uncollected data would be most valuable to supplement the included data, as the absence of information may be significant^3,96^. Greater understanding of the gaps and biases in the fossil and modern datasets we currently have can help to (a) identify the key areas that need further research and (b) allow us to determine what groups are best suited to use for current meta-analyses.

### 2) Ensure citations for the work

Publications should require methods sections to clarify how species were identified and cite works used for identification. These citations could appear either in the main text or in supplemental references/authorities. This should also include references for data that have been pulled from online databases (e.g. PBDB). This would more accurately reflect their utility to the scientific community^2,97,98^. This would, in turn, allow for statistics like the h-index of taxonomic authors to more accurately reflect their impact in the field. When journals一especially those with higher impact factors一require that authors both limit the number of references used and disincentivise citation of publications older than five years, this leads to under-utilisation of foundational taxonomic work, which often takes longer to produce but can remain authoritative for periods of time spanning decades or more^99^.

### 3) Shift funding/incentivisation structures

Government and private agencies should fund more taxonomically based grants because it (a) will ensure a greater chance at tenure for those in the field, and (b) will also make sure that primary research focusing on poorly studied groups will get the funding necessary to achieve that work^53^. The data we have are demonstrably gappy in spatial, temporal, and taxonomic coverage^5–7,17,54,100,101^. Without more work being done and incentivised at a systemic level, these gaps will continue to cause indefinite problems over the long term. While we tend to focus currently on the charismatic and economically important taxa for conservation (the flounder effect), the success or failure of those taxa is often bound to the less charismatic taxa they co-occur with. Ecosystems both support commercially important species and provide a wide range of services, many of which are not appreciated until they are already degraded. In the absence of a broad range of natural history information for community members, our understanding of the factors which impact commercially important species and vital ecosystem services remains incomplete, and this potentially puts these systems at heightened risk. A wider sampling of organismal natural history information would improve both theoretical and applied ecological endeavours^93,102–104^, as well as potentially yielding unanticipated benefits^104,105^. State-level agencies and private institutions, such as fisheries, benefit from conservation of the entire community structure, but this cannot be done effectively in the absence of data for these less charismatic components of the community. We cannot continue meta-analysis without current updated data, and we cannot update data without people to do that work. Taxonomic knowledge and expertise are foundational, but the work is generally underfunded relative to its impact and sometimes authors are uncredited and unrewarded. Tenure is a vital career milestone for researchers who wish to remain working in academia, and our current reward structures devalue building natural history data through actions such as taxonomic monographs.

### 4) Promotion and tenure review restructuring

Tenure requirements should allow for longer taxonomic studies to count for more than short turnaround publications (h-index/impact factor do not reflect societal value). Taxonomic monographs, in particular, are time-consuming endeavours that function more similarly to books in terms of scope and impact and are critical assessments of our understanding of biodiversity. As such, these works should be assessed and weighted differently from standard scientific publications. While there is concern that h-indexes may be artificially bolstered via unwarranted self-citation and predatory journals, tenure committees should still be encouraged not to remove self-citations to taxonomic literature, as many taxonomists often must self-cite since they may be one of the only experts in their fields.

### 5) Fund museums and collections

Funding agencies must continue to provide support for museums that serve as repositories for specimens and training centres for taxonomic experts^2,53,95,106^. Natural history and taxonomy as scientific enterprises rely on these institutions for reproducibility and as records of species’ occurrences and features; the loss of collections—especially type, voucher and other research specimens—represents a breach of prior institutional commitment to these sciences and scientists. While not every small collection is likely to continue at its host institution in perpetuity, institutions should make every effort to rehouse important natural history specimen collections, and ideally, financial support should be provided for the destination institutions to support this trend of consolidation.

### 6) Actively engage with and fund historically excluded communities/peoples

Taxonomic and ecological data are highly structured, favouring high GDP countries/past colonial powers^5,7,44,99^. This means that supporting the development of infrastructure and generating research capacity in historically understudied regions would be disproportionately beneficial to our understanding of the natural world^5,107,108^. The training of persons from regions that have been historically excluded from natural sciences research would also aid the development of environmental perspectives less dictated by colonialist thought^109^. International collaborations and collaborations with indigenous peoples should be encouraged, but the development of local talent and local research capacity should also be encouraged as a broader impact by granting agencies in high-GDP countries, including work with, and the training of, local experts^5^.

### Summary

Meta-analyses, modelling, and conservation plans depend on accurate information from diverse species^45,110^ yet support for the collection of taxonomic and natural history data has not yet risen to even the level of lip service in most high-profile analyses. Explicitly identifying the ways existing datasets are structured can clarify where new natural history information needs to be collected, without hindering the publication of studies based on the best available data. Continued support for organism or community-based projects is necessary to ground-truth ecological theory and conservation efforts, as well as to better assess the rate of anthropogenic extinctions, with most marine and invertebrate taxa in particular being too data-deficient to properly assess or declare extinct at present^59,111^. In a rapidly changing world, with anthropogenic climate change and widespread habitat destruction, the changes proposed in this prospectus are vital to provide an accurate foundation on which to develop successful and effective conservation practices. Furthermore, the development and support of human expertise on natural history (from a wide range of backgrounds and experiences) is a critical but neglected component of our biodiversity conservation strategy. The authors of this perspective believe that these changes could help to ensure that we not only actively build a more robust understanding of fossil and modern taxa, but that this work would be done in an equitable and sustainable fashion.

## Supplementary information

Supplementary Information

## Data Availability

All data used to generate this manuscript are available as supplemental datasets (Supplemental datasets 1–4 on Dryad at 10.5061/dryad.v6wwpzh61.
