# Supplementary material for: The flounder effect: disparities in taxonomic and ecological study intensity across extant and fossil marine organisms hamper conservation
Source: NPJ Biodivers. 2026 Jan 31;5:4. doi: 10.1038/s44185-025-00118-1 (PMC12860799; doi:10.1038/s44185-025-00118-1)
Supplement: Supplementary file 1 — Supplementary Information [file 44185_2025_118_MOESM1_ESM.pdf]

Supplemental Datasets

Supplemental datasets are available on dryad at: https://doi.org/10.5061/dryad.v6wwpzh61

Reviewer sharing link to dryad: http://datadryad.org/share/LINK_NOT_FOR_PUBLICATION/

KPQ27ZNOEloSFcQ6qFISGdfsAVVhEwbZpDOA4b_Kpv4

Supplemental dataset 1. Number of publications per species.

Supplemental dataset 2. Species sampled for publication numbers.

Supplemental dataset 3. UF Neogastropod species identification rates dataset.

Supplemental dataset 4. PBDB Florida and Atlantic coastal plain species identification rates dataset and comparison to UF data.

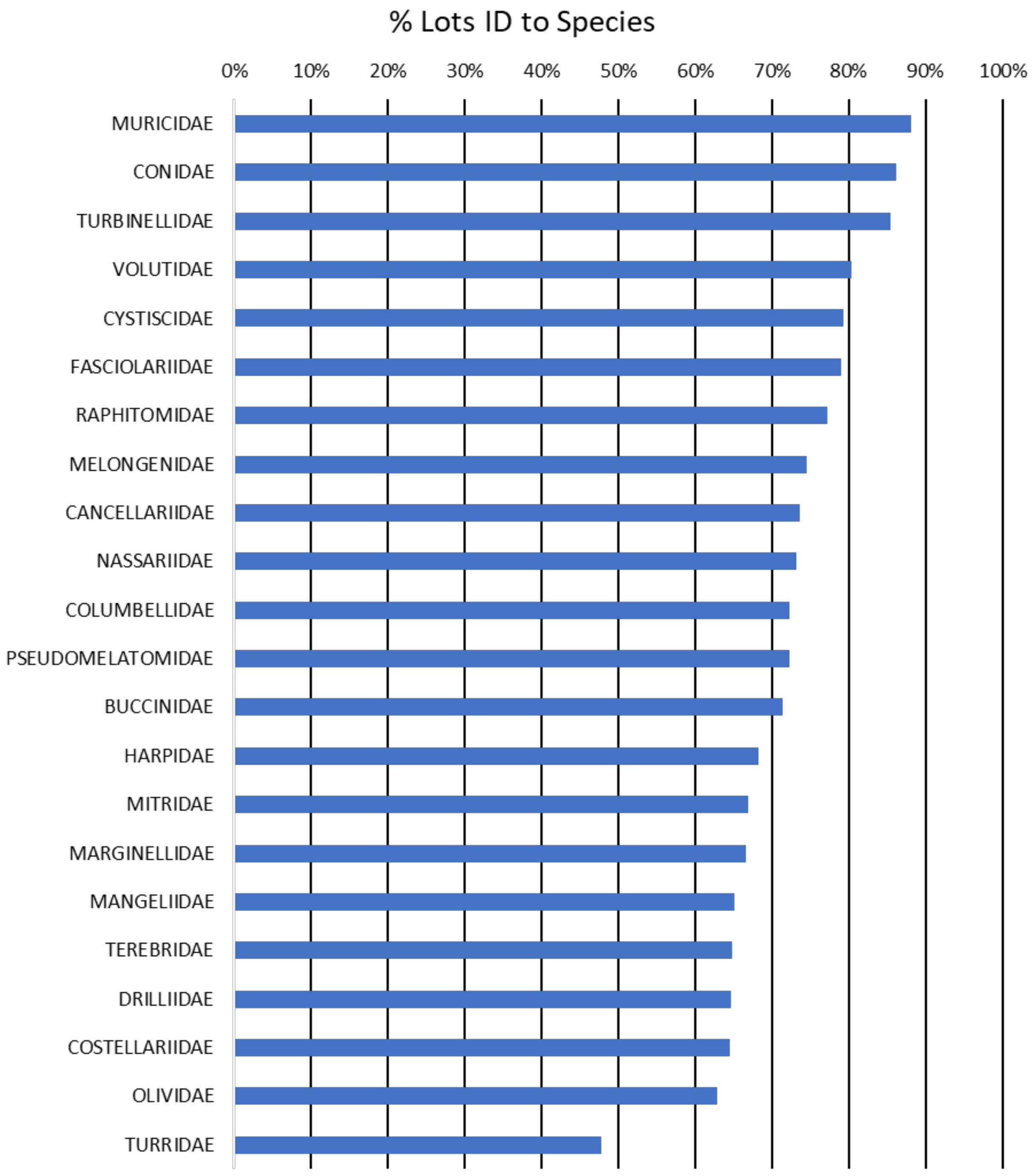

Supplemental fig 1. Percent of neogastropod lots assigned a species identification in the Specify database of the Florida Museum of Natural History by family.

Count
1
1
1
1
1
1
1
1
1
1
1
1
1
1
1
1
1
1
1
1
1
1
1
1
1
1
1
1
1
1
1
1
1
1
1
1
1
1
1
1
1
1

1
1
1
1
1
1
1
1
1
1
1
1
1
1
1
1
1
1
1
1
1
1
1
1
1
1
1
1
1
1
1
1
1
1
1
1
1
1
1
1
1
1
1

1
1
1
1
1
1
1
1
1
1
1
1
1
1
1
1
1
1
1
1
1
1
1
1
1
1
1
1
1
1
1
1
1
1
1
1
1
1
1
1
1
1
1

1
1
1
1
1
1
1
1
1
1
1
1
1
1
1
1
1
1
1
1
1
1
1
1
1
1
1
1
1
1
1
1
1
1
1
1
1
1
1
1
1
1
1

1
1
1
1
1
1
1
1
1
1
1
1
1
1
1
1
1
1
1
1
1
1
1
1
1
1
1
1
1
1
1
1
1
1
1
1
1
1
1
1
1
1
1

1
1
1
1
1
1
1
1
1
1
1
1
1
1
1
1
1
1
1
1
1
1
1
1
1
1
1
1
1
1
1
1
1
1
1
1
1
1
1
1
1
1
1

1
1
1
1
1
1
1
1
1
1
1
1
1
1
1
1
1
1
1
1
1
1
1
1
1
1
1
1
1
1
1
1
1
1
1
1
1
1
1
1
1
1
1

1
1
1
1
1
1
1
1
1
1
1
1
1
1
1
1
1
1
1
1
1
1
1
1
1
1
1
1
1
1
1
1
1
1
1
1
1
1
1
1
1
1
1

1
1
1
1
1
1
1
1
1
1
1
1
1
1
1
1
1
1
1
1
1
1
1
1
1
1
1
1
1
1
1
1
1
1
1
1
1
1
1
1
1
1
1

1
1
1
1
1
1
1
1
1
1
1
1
1
1
1
1
1
1
1
1
1
1
1
1
1
1
1
1
1
1
1
1
1
1
1
1
1
1
1
1
1
1
1

1
1
1
1
1
1
1
1
1
1
1
1
1
1
1
1
1
1
1
1
1
1
1
1
1
1
1
1
1
1
1
1
1
1
1
1
1
1
1
1
1
1
1

1
1
1
1
1
1
1
1
1
1
1
1
2
2
2
2
2
2
2
2
2
2
2
2
2
2
2
2
2
2
2
2
2
2
2
2
2
2
2
2
2
2
2

2
2
2
2
2
2
2
2
2
2
2
2
2
2
2
2
2
2
2
2
2
2
2
2
2
2
2
2
2
2
2
2
2
2
2
2
2
2
2
2
2
2
2

2
2
2
2
2
2
2
2
2
2
2
2
2
2
2
2
2
2
2
2
2
2
2
2
2
2
2
2
2
3
3
3
3
3
3
3
3
3
3
3
3
3
3

3
3
3
3
3
3
3
3
3
3
3
3
3
3
3
3
3
3
3
3
3
3
3
3
3
3
3
3
3
3
3
3
3
3
3
3
3
3
3
3
3
3
3

3
3
3
3
3
3
3
3
3
3
3
3
3
3
3
3
3
3
4
4
4
4
4
4
4
4
4
4
4
4
4
4
4
4
4
4
4
4
4
4
4
4
4

4
4
4
4
4
4
4
4
4
4
4
4
4
4
4
4
4
4
4
4
4
4
4
4
4
4
4
4
4
4
4
5
5
5
5
5
5
5
5
5
5
5
5

5
5
5
5
5
5
5
5
5
5
5
5
5
5
5
5
5
5
5
5
5
5
5
6
6
6
6
6
6
6
6
6
6
6
6
6
6
6
6
6
6
6
6

6
6
6
6
6
6
6
6
6
6
7
7
7
7
7
7
7
7
7
7
7
7
7
7
7
7
8
8
8
8
8
8
8
8
8
8
8
8
8
8
8
8
9

9
9
9
9
9
9
9
9
9
9
9
9
9
9
9
9
9
9
10
10
10
10
10
10
10
10
10
11
11
11
11
11
11
11
11
11
11
11
11
11
12
12
12

12
12
12
12
12
13
13
13
13
13
13
13
13
13
14
14
14
14
14
15
15
15
15
15
15
15
15
15
15
15
15
15
15
16
16
16
16
16
16
16
16
17
17

17
17
17
18
18
18
18
18
19
19
19
19
20
20
20
20
20
21
21
21
21
21
22
23
23
23
23
23
23
23
24
24
24
25
26
26
26
26
26
26
27
27
27

28
28
29
30
30
30
30
31
31
32
33
33
33
33
34
36
36
36
37
37
38
41
41
41
42
43
43
43
44
48
48
48
50
52
52
53
53
54
54
55
57
61
66

67
67
70
74
77
81
81
81
82
91
91
93
98
98
116
120
122
122
126
133
144
146
147
152
160
166
182
212
224
270
277
288
376
580
602
689
700
4628

Count
1
1
1
1
1
1
1
1
1
1
1
1
1
1
2
2
2
2
2
2
2
2
2
3
3
3
3
3
3
3
3
3
3
3
3
3
4
4
4
4
4
4

5
5
5
5
5
5
5
5
6
6
6
6
6
7
7
7
8
9
10
11
11
13
14
15
15
15
15
16
17
19
21
21
21
23
23
23
24
26
26
26
27
27
28

29
30
32
33
33
33
36
37
37
38
41
41
43
48
50
50
52
54
57
67
74
77
81
82
91
93
120
122
144
146
152
212
270
288
376
689
700
4628

Count
1
1
1
1
1
1
1
1
1
1
1
1
1
1
1
1
1
1
1
1
1
1
1
1
1
1
1
1
1
1
1
1
1
1
1
1
1
1
1
1
1
1

1
1
1
1
1
1
1
1
1
1
1
1
1
1
1
1
1
1
1
1
1
1
1
1
1
1
1
1
1
1
1
1
1
1
1
1
1
1
1
1
1
1
1

1
1
1
1
1
1
1
1
1
1
1
1
1
1
1
1
1
1
1
1
1
1
1
1
1
1
1
1
1
1
1
1
1
1
1
1
1
1
1
1
1
1
1

1
1
1
1
1
1
1
1
1
1
1
1
1
1
1
1
1
1
1
1
1
1
1
1
1
1
1
1
1
1
1
1
1
1
1
1
1
1
1
1
1
1
1

1
1
1
1
1
1
1
1
1
1
1
1
1
1
1
1
1
1
1
1
1
1
1
1
1
1
1
1
1
1
1
1
1
1
1
1
1
1
1
1
1
1
1

1
1
1
1
1
1
1
1
1
1
1
1
1
1
1
1
1
1
1
1
1
1
1
1
1
1
1
1
1
1
1
1
1
1
1
1
1
1
1
1
1
1
1

1
1
1
1
1
1
1
1
1
1
1
1
1
1
1
1
1
1
1
1
1
1
1
1
1
1
1
1
1
1
1
1
1
1
1
1
1
1
1
1
1
1
1

1
1
1
1
1
1
1
1
1
1
1
1
1
1
1
1
1
1
1
1
1
1
1
1
1
1
1
1
1
1
1
1
1
1
1
1
1
1
1
1
1
1
1

1
1
1
1
1
1
1
1
1
1
1
1
1
1
1
1
1
1
1
1
1
1
1
1
1
1
1
1
1
1
1
1
1
1
1
1
1
1
1
1
1
1
1

1
1
1
1
1
1
1
1
1
1
1
1
1
1
1
1
1
1
1
1
1
1
1
1
1
1
1
1
1
1
1
1
1
1
1
1
1
1
1
1
1
1
1

1
1
1
1
1
1
1
1
1
1
1
1
1
1
1
1
1
1
1
1
1
1
1
1
1
1
1
1
1
1
1
1
1
1
1
1
1
1
1
1
1
2
2

2
2
2
2
2
2
2
2
2
2
2
2
2
2
2
2
2
2
2
2
2
2
2
2
2
2
2
2
2
2
2
2
2
2
2
2
2
2
2
2
2
2
2

2
2
2
2
2
2
2
2
2
2
2
2
2
2
2
2
2
2
2
2
2
2
2
2
2
2
2
2
2
2
2
2
2
2
2
2
2
2
2
2
2
2
2

2
2
2
2
2
2
3
3
3
3
3
3
3
3
3
3
3
3
3
3
3
3
3
3
3
3
3
3
3
3
3
3
3
3
3
3
3
3
3
3
3
3
3

3
3
3
3
3
3
3
3
3
3
3
3
3
3
3
3
3
3
3
3
3
3
3
3
3
4
4
4
4
4
4
4
4
4
4
4
4
4
4
4
4
4
4

4
4
4
4
4
4
4
4
4
4
4
4
4
4
4
4
4
4
4
4
4
4
4
4
4
4
4
4
4
4
4
4
5
5
5
5
5
5
5
5
5
5
5

5
5
5
5
5
5
5
5
5
5
5
5
5
5
5
5
6
6
6
6
6
6
6
6
6
6
6
6
6
6
6
6
6
6
6
6
6
6
6
6
6
7
7

7
7
7
7
7
7
7
7
7
7
7
8
8
8
8
8
8
8
8
8
8
8
8
8
8
8
9
9
9
9
9
9
9
9
9
9
9
9
9
9
9
9
9

9
10
10
10
10
10
10
10
10
11
11
11
11
11
11
11
11
11
11
11
12
12
12
12
12
12
12
12
13
13
13
13
13
13
13
13
14
14
14
14
15
15
15

15
15
15
15
15
15
15
16
16
16
16
16
16
16
17
17
17
17
18
18
18
18
18
19
19
19
20
20
20
20
20
21
21
22
23
23
23
23
24
24
25
26
26

26
27
28
30
30
30
31
31
33
34
36
36
41
42
43
43
44
48
48
50
52
53
54
55
61
66
67
70
81
81
91
98
98
116
122
126
147
160
166
182
224
277
580

Species	Number of English Google Scholar returns (50=50 or greater)	Genus is used for human Consumption/other fishery/is a parasite of such species?	Fossil only
Arhynchobatis asperrimus	24		
Arielia cancellata	1		
Ariomma bondi	91	1	
Ariomma brevimanum	19	1	
Ariomma dollfusi	3	1	
Ariomma indicum	52	1	
Ariomma luridum	8	1	
Ariomma melanum	23	1	
Ariomma parini	1	1	
Ariomma regulus	37	1	
Ariosoma anago	54	1	
Ariosoma anagoides	29	1	
Ariosoma anale	36	1	
Ariosoma balearicum	288	1	
Ariosoma bauchotae	2	1	
Ariosoma coquettei	4	1	
Ariosoma fasciatum	15	1	
Ariosoma gilberti	41	1	
Ariosoma howensis	2	1	
Ariosoma major	41	1	
Ariosoma marginatum	26	1	
Ariosoma mauritianum	14	1	
Ariosoma meeki	26	1	
Ariosoma megalops	4	1	
Ariosoma mellissii	4	1	
Ariosoma multivertebratum	2	1	
Ariosoma nigrimanum	1	1	
Ariosoma obud	1	1	
Ariosoma ophidiophthalmus	1	1	
Ariosoma opistophthalmum	5	1	
Ariosoma prorigerum	5	1	
Ariosoma sanzoi	3	1	
Ariosoma sazonovi	2	1	
Ariosoma scheelei	38	1	
Ariosoma selenops	15	1	
Ariosoma sereti	1	1	
Ariosoma shiroanago	24	1	

Ariosoma sokotranum	2	1	
Aristostomias grimaldii	28	1	
Aristostomias lunifer	21	1	
Aristostomias polydactylus	11	1	
Aristostomias titmanni	5	1	
Aristostomias xenostoma	23	1	
Arites eocaenicus	1		1
Arites minor	1		1
Arites sibiricus	1		1
Arkonips topororum	4		1
Armatognathia birsteini	3		
Arnaudia arnaudi	1		1
Arnoglossus andrewsi	5	1	
Arnoglossus arabicus	6	1	
Arnoglossus armstrongi	4	1	
Arnoglossus aspilos	30	1	
Arnoglossus bassensis	6	1	
Arnoglossus boops	3	1	
Arnoglossus brunneus	3	1	
Arnoglossus capensis	37	1	
Arnoglossus coeruleosticta	1	1	
Arnoglossus dalgleishi	7	1	
Arnoglossus debilis	11	1	
Arnoglossus elongatus	15	1	
Arnoglossus fisoni	6	1	
Arnoglossus grohmanni	32	1	
Arnoglossus imperialis	152	1	
Arnoglossus japonicus	27	1	
Arnoglossus kessleri	57	1	
Arnoglossus laterna	689	1	
Arnoglossus macrolophus	21	1	
Arnoglossus marisrubri	5	1	
Arnoglossus micrommatus	2	1	
Arnoglossus muelleri	9	1	
Arnoglossus multirastris	1	1	
Arnoglossus nigrifrons	2	1	
Arnoglossus nigrofilamentosus	3	1	
Arnoglossus oxyrhynchus	4	1	
Arnoglossus polyspilus	27	1	
Arnoglossus rueppelii	43	1	
Arnoglossus sayaensis	5	1	
Arnoglossus scapha	67	1	
Arnoglossus septemventralis	1	1	

Arnoglossus tapeinosoma	33	1	
Arnoglossus tenuis	33	1	
Arnoglossus thori	270	1	
Arnoglossus waitei	26	1	
Arnoglossus yamanakai	3	1	
Aroapyrgus alleei	13	1	
Aroapyrgus chagresensis	2		
Aroapyrgus chefinesnoel	1		
Aroapyrgus cisterninus	13		
Aroapyrgus clenchi	3		
Aroapyrgus conchensensis	1		
Aroapyrgus costaricensis	11		
Aroapyrgus ernesti	3		
Aroapyrgus guatemalensis	4		
Aroapyrgus hinkleyi	1		
Aroapyrgus joseanus	1		
Aroapyrgus mexicanus	1		
Aroapyrgus orizabensis	1		
Aroapyrgus panamensis	3		
Aroapyrgus panzosensis	1		
Aroapyrgus pasionensis	3		
Aroapyrgus petenensis	1		
Aroapyrgus rhegoides	1		
Aroapyrgus stolli	1		
Aroapyrgus subangulatus	2		
Aroapyrgus tryoni	2		
Aroapyrgus vivens	2		
Arrhamphus sclerolepis	212	1	
Artedius corallinus	43		
Artedius fenestralis	160		
Artedius harringtoni	166		
Artedius lateralis	147		
Artedius notospilotus	44		
Arthochordeumium asteromorphe	1		
Arthritica ambotruncata	1		1
Arthritica bifurca	116		
Arthritica crassiformis	27		
Arthritica crassitesta	1		1
Arthritica dispar	1		1
Arthritica elongata	1		1
Arthritica finlayi	1		1
Arthritica hulmei	1		

Arthritica japonica	11		
Arthritica perfragilis	1		1
Arthritica reikoae	5		
Arthritica semen	91		
Arthritica whitechurchi	1		
Arthrochordeumium appendiculosum	2		
Bathyaethiops atercrinis	3	1	
Bathyaethiops baka	1	1	
Bathyaethiops breuseghemi	6	1	
Bathyaethiops caudomaculatus	16	1	
Bathyaethiops flammeus	1	1	
Bathyaethiops greeni	6	1	
Bathybelos typhlops	6		
Bathycalliax geomar	21		
Bathycalliax mediterranea	3		1
Bathycallisoma schellenbergi	23		
Bathyceradocus hawkingi	2		
Bathyceradocus iberiensis	1		
Bathyceradocus stephenseni	8		
Bathyceradocus wuzzae	4		
Bathycongrus aequoreus	11		
Bathycongrus bertini	7		
Bathycongrus bleekeri	9		
Bathycongrus bullisi	8		
Bathycongrus dubius	13		
Bathycongrus guttulatus	21		
Bathycongrus longicavis	3		
Bathycongrus macrocercus	5		
Bathycongrus macrurus	19		
Bathycongrus nasicus	6		
Bathycongrus odontostomus	9		
Bathycongrus parapolyporus	2		
Bathycongrus parviporus	2		
Bathycongrus polyporus	3		
Bathycongrus retrotinctus	16		
Bathycongrus thysanochilus	12		
Bathycongrus trilineatus	3		
Bathycongrus trimaculatus	7		
Bathycongrus unimaculatus	2		
Bathycongrus varidens	11		
Bathycongrus vicinalis	31		

Bathycongrus wallacei	18		
Bathycrinicola curta	8		
Bathycrinicola fernandinae	1		
Bathycrinicola macrapex	2		
Bathycrinicola media	1		
Bathycrinicola micrapex	2		
Bathycrinicola nacraensis	1		
Bathycrinicola talaena	1		
Bathycrinicola tumidula	18		
Bathydorus echinus	3		
Bathydorus fimbriatus	3		
Bathydorus laevis	9		
Bathydorus laniger	12		
Bathydorus servatus	1		
Bathydorus spinosissimus	1		
Bathydorus spinosus	18		
Bathydorus uncifer	1		
Bathyepsilonema anulosum	10		
Bathyepsilonema brachycephalum	4		
Bathyepsilonema cobbi	1		
Bathyepsilonema compactum	4		
Bathyepsilonema dermoglyphum	1		
Bathyepsilonema drygalskii	2		
Bathyepsilonema lissum	4		
Bathyepsilonema lopheliae	5		
Bathyepsilonema monniotorum	1		
Bathyepsilonema salvati	1		
Bathyepsilonema spongiosum	2		
Bathyepsilonema vulgare	1		
Fabella constricta	8		
Fabella pilsbryi	3		
Fabella stearnsii	7		
Fabienna oligonema (Pochella oligonema)	10		
Fabienna sphaerica	17		
Fabifenestella ajensis	1		1
Fabifenestella almazani	1		1
Fabifenestella arcitubulata	1		1
Fabifenestella binodosa	1		1
Fabifenestella completa	2		1

Fabifenestella congruens	1		1
Fabifenestella dubia	1		1
Fabifenestella edzhekalensis	1		1
Fabifenestella fabalis	2		1
Fabifenestella huascatayana	1		1
Fabifenestella irregularis	1		1
Fabifenestella irsuensis	1		1
Fabifenestella jabensis	1		1
Fabifenestella jakutica	1		1
Fabifenestella joachimi	1		1
Fabifenestella khozhainovi	1		1
Fabifenestella macrofenestrata	1		1
Fabifenestella mirifica	1		1
Fabifenestella mirificina	1		1
Fabifenestella neoforaminosa	1		1
Fabifenestella paratuberculifera	1		1
Fabifenestella permiana	1		1
Fabifenestella plummerae	1		1
Fabifenestella praevirgosa	2		1
Fabifenestella procera	1		1
Fabifenestella pseudovirgosa	1		1
Fabifenestella sibirica	1		1
Fabifenestella subvirgosa	1		1
Fabifenestella symmetrica	1		1
Fabifenestella tortuosa	3		1
Fabifenestella tsherepetiensis	1		1
Fabifenestella tumulosa	1		1
Fabifenestella vediensis	1		1
Fabifenestella virgosa	2		1
Fabifenestella vulgaris	1		1
Fabricinuda bikinii	8		
Fabricinuda limnicola	15		
Fabricinuda longilabrum	2		
Fabricinuda mossambica	6		
Fabricinuda pseudocollaris	5		
Fabricinuda pseudopalpa	4		
Fabricinuda rosaelenae	1		
Fabricinuda trilobata	48		
Fabrisabella similis	6		
Fabrisabella vasculosa	2		
Fairfaxia cribbi	12		

Fairfaxia lethrini	10		
Falkla bacillata	1		
Fallacihowella caligo	3		
Fallacihowella sol	5		
Falsanathelges mariae	1		
Falsanathelges muelleri	6		
Falsapseudes bowmani	4		
Falsifusus apicalis	3		1
Falsifusus dissimilis	1		1
Falsifusus erectus	1		1
Falsifusus londini	3		1
Falsifusus multispiratus	1		1
Falsifusus ottonis	3		1
Falsifusus regnorum	3		1
Falsifusus unicarinatus	9		1
Falsifusus ytenae	1		1
Falsilunatia ambigua	11		
Falsilunatia amphiala	4		
Falsilunatia benthicola	2		
Falsilunatia carcellesi	8		
Falsilunatia eltanini	4		
Falsilunatia falklandica	7		
Falsilunatia fartilis	3		
Falsilunatia joubini	1		
Falsilunatia nigromaculata	1		
Falsilunatia notorcadensis	2		
Falsilunatia patagonica	17		
Falsilunatia pseudopsila	1		
Falsilunatia scotiana	1		
Falsilunatia subperforata	1		
Falsilunatia xantha	3		
Geomonhystera aenariensis	3		
Geomonhystera altaica	1		
Geomonhystera antarcticola	18		
Geomonhystera australis	6		
Geomonhystera auvillis	3		
Geomonhystera breviseta	5		
Geomonhystera dubia	3		
Geomonhystera glandulata	3		
Geomonhystera japonica	2		
Geomonhystera karuni	5		
Geomonhystera longicaudata	1		
Geomonhystera media	1		

Geomonhystera mexicana	1		
Geomonhystera parvillosa	1		
Geomonhystera pervaga	4		
Geomonhystera steineri	1		
Geomonhystera tripyloides	3		
Geomonhystera villosa	48		
Geophagus abalios	34		
Geophagus altifrons	77	ornamental aquaria	
Geophagus argyrostictus	20		
Geophagus brachybranchus	13		
Geophagus brasiliensis (Acara gymnopoma,	14		
Geophagus brokopondo	5		
Geophagus camopiensis	20		
Geophagus crassilabris	23		
Geophagus crocatus	2		
Geophagus diamantinensis	6		
Geophagus dicrozoster	14		
Geophagus gottwaldi	6		
Geophagus grammepareius	11		
Geophagus harreri	15		
Geophagus megasema	15		
Geophagus mirabilis	4		
Geophagus neambi	9		
Geophagus obscurus	5	1	
Geophagus parnaibae	15	1	
Geophagus pellegrini	9		
Geophagus proximus	122	1	
Geophagus steindachneri	93	1	
Geophagus surinamensis	144	1	
Geophagus sveni	16		
Geophagus taeniopareius	7	1	
Geophagus winemilleri	5		
Gephyreaster swifti	25		
Gephyrophora bilamellaria	1		1
Gephyrophora polymorpha	6		
Gephyrophora rubra	9		
Gephyrothuria alcocki	10		
Geromura teckliformis	1		1
Geromura touertensis	1		1
Gerres akazakii	*4*		

Gerres argyreus	53	? heavy metal toxicity	
Gerres baconensis	33	toxicity?	
Gerres chrysops	23		
Gerres cinereus	602		
Gerres decacanthus	16		
Gerres equulus	52		
Gerres erythrourus	11		
Gerres filamentosus	700	human consumption	
Gerres infasciatus	18		
Gerres japonicus	41		
Gerres limbatus	98		
Gerres longirostris	122		
Gerres macracanthus	70		
Gerres maldivensis	7		
Gerres methueni	61		
Gerres microphthalmus	15		
Gerres mozambiquensis	1		
Gerres nigri	66		
Gerres oblongus	126		
Gerres oyena	580		
Gerres phaiya	15		
Gerres ryukyuensis	4		
Gerres septemfasciatus	1		
Gerres setifer	55		
Gerres shima	6		
Gerres silaceus	2		
Gerres simillimus	26		
Gerres subfasciatus	277		
Gerulusosacculus commutabilis	1		
Giardella caissarum	2		
Giardella callianassae	9		
Giardella carinifer	1		
Giardella ricoensis	2		
Giardella thompsoni	2		
Gibberleberis elongata	1		1
Gibberula ablita	1		
Gibberula abyssicola	4		
Gibberula achenea	2		
Gibberula adyae	1		
Gibberula adzubae	1		

Gibberula aequatorialis	1		
Gibberula agapeta	1		
Gibberula agricola	1		
Gibberula albotriangularis	1		
Gibberula almadiensis	1		
Gibberula almo	1		
Gibberula amurakucensis	1		1
Gibberula andreahirundae	1		
Gibberula angelarum	1		
Gibberula aperta	1		
Gibberula aquitanensis	1		1
Gibberula ardovinii	1		
Gibberula arubagrandis	1		
Gibberula asellina	1		
Gibberula atlantidea	1		
Gibberula atwoodae	1		
Gibberula audreyae	1		
Gibberula aurata	1		
Gibberula aurelieae	1		
Gibberula baisrei	1		
Gibberula belizensis	1		
Gibberula benguelensis	1		
Gibberula bensoni	1		
Gibberula benyi	1		
Gibberula betancourtae	1		
Gibberula bezoyensis	1		1
Gibberula boettgeri	1		
Gibberula borbonica	1		
Gibberula boulmerkae	1		
Gibberula boyeri	1		
Gibberula bribri	2		
Gibberula bulbosa	1		
Gibberula burnupi	4		
Gibberula caelata	7		
Gibberula candida	1		
Gibberula caribetica	1		
Gibberula catenata	13		
Gibberula cavernicola	1		
Gibberula cavinae	1		
Gibberula ceciliae	1		
Gibberula celerae	1		
Gibberula cherubini	1		
Gibberula chiarae	1		

Gibberula chudeaui	3		
Gibberula chumi	1		
Gibberula cincta	1		
Gibberula colombiana	2		
Gibberula columnella	1		
Gibberula compressa	1		
Gibberula conejoensis	2		
Gibberula confusa	1		
Gibberula crassa	1		
Gibberula cristata	1		
Gibberula cristinae	2		
Gibberula cucullata	1		
Gibberula debilis	1		
Gibberula decorfasciata	1		
Gibberula degrangei	1		1
Gibberula delarrochae	1		
Gibberula dens	1		
Gibberula denticulata	1		
Gibberula dentiformis	1		
Gibberula diadema	1		
Gibberula differens	4		
Gibberula diplostreptus	4		
Gibberula dosmosquises	1		
Gibberula dulcis	3		
Gibberula elisae	1		
Gibberula eloinae	1		
Gibberula elvirae	1		
Gibberula epigrus	6		
Gibberula estherae	1		
Gibberula eugeniae	1		
Gibberula evadne	1		
Gibberula evandroi	1		
Gibberula farlensis	1		
Gibberula fatimae	1		
Gibberula ficula	3		
Gibberula fluctuata	6		
Gibberula fortis	1		
Gibberula fortisminor	1		
Gibberula gabryae	1		
Gibberula garzae	1		
Gibberula gironai	1		
Gibberula goodallae	1		
Gibberula gradatim	1		

Gibberula grafae	1		
Gibberula granulinaformis	1		
Gibberula gruveli	1		
Gibberula hardingae	1		
Gibberula hendricksae	1		
Gibberula hernandezi	2		
Gibberula hirami	1		
Gibberula infundibulum	1		
Gibberula inopinata	2		
Gibberula insularum	1		
Gibberula isinbayevae	1		
Gibberula jansseni	9		
Gibberula japonica	1		
Gibberula jayi	1		
Gibberula jeanae	1		
Gibberula jenphillipsi	1		
Gibberula jorgefoyoi	1		
Gibberula judithae	1		
Gibberula lachryma	1		
Gibberula lalaina	1		
Gibberula langostera	1		
Gibberula laritzae	1		
Gibberula lavalleeana	17		
Gibberula laviuda	1		
Gibberula lazaroi	2		
Gibberula leibovitzae	1		
Gibberula lessingae	1		
Gibberula lifouana	1		
Gibberula lorenziana	1		
Gibberula louisae	2		
Gibberula lucia	1		
Gibberula luglii	1		
Gibberula macarioi	1		
Gibberula macula	1		
Gibberula madbelono	1		
Gibberula maldiviana	1		
Gibberula mamillata	1		
Gibberula mandyi	1		
Gibberula manzanillera	1		
Gibberula mapipi	1		
Gibberula marcelae	1		
Gibberula marinae	1		
Gibberula marioi	1		

Gibberula mariscali	1		
Gibberula martinae	1		
Gibberula martingaiteae	1		
Gibberula mazagonica	2		
Gibberula mendacis	1		
Gibberula metoo	1		
Gibberula miliaria	1		
Gibberula mimetica	1		
Gibberula modica	1		
Gibberula morchii	7		
Gibberula moscatellii	1		
Gibberula navratilovae	1		
Gibberula nebulosa	2		
Gibberula nilsi	1		
Gibberula norvisae	1		
Gibberula novemprovincialis	2		
Gibberula nuryana	1		
Gibberula nussbaumae	1		
Gibberula occidentalis	1		
Gibberula ocellus	1		
Gibberula olivai	1		
Gibberula olivella	1		
Gibberula oriens	1		
Gibberula oryza	9		
Gibberula ovata	1		
Gibberula ovoides	1		
Gibberula pacifica	1		
Gibberula palazzii	1		
Gibberula pallata	1		
Gibberula palmasola	1		
Gibberula peterbonuttii	1		
Gibberula pfeifferi	5		
Gibberula phantasma	1		
Gibberula philippii	33		
Gibberula pignonae	1		
Gibberula poppei	1		
Gibberula pseudoaquitanica	1		1
Gibberula pulchella	5		
Gibberula punctillum	1		
Gibberula quadrifasciata	1		
Gibberula quatrefortis	1		
Gibberula ramsi	1		
Gibberula raquelae	1		

Gibberula rauli	2		
Gibberula recondita	7		
Gibberula robinsonae	1		
Gibberula ronchinorum	1		
Gibberula rowlingae	1		
Gibberula rufanensis	1		
Gibberula sassenae	1		
Gibberula savignyi	4		
Gibberula scalarispira	1		
Gibberula sebastiani	1		
Gibberula secreta	4		
Gibberula sierrai	1		
Gibberula silviae	1		
Gibberula simii	1		
Gibberula simonae	1		
Gibberula simplex	1		
Gibberula socoae	1		
Gibberula soniae	1		
Gibberula spiriplana	1		
Gibberula squamosa	1		
Gibberula stella	1		
Gibberula striata	1		
Gibberula subovulatoides	1		1
Gibberula subtrigona	1		
Gibberula sueziensis	2		
Gibberula tahaukuensis	4		
Gibberula tantula	1		
Gibberula teresitae	1		
Gibberula themisae	1		
Gibberula thetisae	1		
Gibberula thomensis	1		
Gibberula turbinata	1		
Gibberula turgidula	6		
Gibberula ubitaensis	2		
Gibberula ubitalta	1		
Gibberula varicosa	1		
Gibberula veilae	1		
Gibberula velox	1		
Gibberula ventricosa	1		
Gibberula vignali	2		
Gibberula vitium	1		
Gibberula vomoensis	1		
Gibberula watkinsae	1		

Gibberula yidii	1		
Gibberula yoshikoae	1		
Gibberula zambranoae	1		
Gibberula zonata	2		
Gibbula albida	67		
Gibbula anodosula	1		1
Gibbula ardens	98		
Gibbula aurantia	1		
Gibbula beckeri	5		
Gibbula benzi (Trochus benzi, Gibbula pintado, Margarita pintado, Trochus ludwigi)	4		
Gibbula brebioni	1		1
Gibbula candei	3		
Gibbula capensis (trochus capensis)	12		
Gibbula cicer	15		
Gibbula clanculiforma	1		1
Gibbula clandestina	1		
Gibbula conicomagus	1		1
Gibbula corallioides	1		
Gibbula denizi	1		
Gibbula deversa	6		
Gibbula drepanensis (Trochus drepanensis)	9		
Gibbula eikoae	1		
Gibbula euomphala (trochus euomphalus)	1		1
Gibbula fanulum	30		
Gibbula filiformis	1		1
Gibbula guishanensis	1		
Gibbula guttadauri	30		
Gibbula hisseyiana	4		
Gibbula houarti	1		
Gibbula joubini	4		
Gibbula leucophaea	13		
Gibbula loculosa	1		
Gibbula magus	182		
Gibbula marianae	1		1
Gibbula marmorea	9		
Gibbula massieri	3		
Gibbula megamagus	1		1
Gibbula mirabilis	1		1

Gibbula multicolor (Trochus multicolor, cynisca rufanensis)	9		
Gibbula parnensis	1		1
Gibbula philberti	24		
Gibbula provosti	2		1
Gibbula racketti	28		
Gibbula rosea	16		
Gibbula saeniensis	1		1
Gibbula sementis	1		
Gibbula senegalensis	1		
Gibbula spratti	10		
Gibbula spurca	1		
Gibbula tantilla	1		
Gibbula tindayaensis	1		1
Gibbula tingitana	8		
Gibbula tryoni	1		
Gibbula tumida	81		
Gibbula turbinoides	36		
Gibbula vanwalleghemi	1		
Gibbula verdensis	2		
Gibbula vimontiae	6		
Gibbula zonata	13		
Gieysztoria (Marcusiella) okugawai	4		
Gieysztoria (Marcusiella) thymara	5		
Gieysztoria acariaia	1		
Gieysztoria ashokae	2		
Gieysztoria atalaya	1		
Gieysztoria beklemischevi	1		
Gieysztoria bellis	3		
Gieysztoria beltrani	2		
Gieysztoria bergi	4		
Gieysztoria bicoronaria	1		
Gieysztoria billabongensis	1		
Gieysztoria bimaculata	1		
Gieysztoria blodgetti	1		
Gieysztoria capreola	1		
Gieysztoria caucasica	1		
Gieysztoria chiqchi	4		
Gieysztoria chlynovica	1		
Gieysztoria choctaw	1		
Gieysztoria complicata	4		

Gieysztoria coronae	2		
Gieysztoria coronaria	1		
Gieysztoria cuspidata	14		
Gieysztoria cypris	4		
Gieysztoria diadema	7		
Gieysztoria dodgei	10		
Gieysztoria donnae	3		
Gieysztoria dubitativa	1		
Gieysztoria erivanica	1		
Gieysztoria euchroa	1		
Gieysztoria evelinae	5		
Gieysztoria expedita	5		
Gieysztoria expeditoides	1		
Gieysztoria falx	3		
Gieysztoria faubeli	2		
Gieysztoria foreli	1		
Gieysztoria garudae	1		
Gieysztoria graffi	1		
Gieysztoria guangdongensis	1		
Gieysztoria huizhouensis	1		
Gieysztoria hymanae	3		
Gieysztoria iberica	1		
Gieysztoria inflata	1		
Gieysztoria infundibuliformis	3		
Gieysztoria intricata	4		
Gieysztoria isoldeae	1		
Gieysztoria italica	1		
Gieysztoria japonica	1		
Gieysztoria joannae	2		
Gieysztoria kasasapa	2		
Gieysztoria knipovici	2		
Gieysztoria koiwi	1		
Gieysztoria kolasai	4		
Gieysztoria lugubris	1		
Gieysztoria macrovariata	16		
Gieysztoria maritima	1		
Gieysztoria matilde	1		
Gieysztoria minima	31		
Gieysztoria multiovata	1		
Gieysztoria namuncurai	1		
Gieysztoria octospinosa	1		
Gieysztoria oligocentra	1		
Gieysztoria ornata	11		

Gieysztoria oryzae	1		
Gieysztoria papii	2		
Gieysztoria pavimentata	3		
Gieysztoria pisana	1		
Gieysztoria pseudoblodgetti	1		
Gieysztoria pseudodiadema	2		
Gieysztoria pulchra	5		
Gieysztoria qixingyanensis	1		
Gieysztoria quadrata	3		
Gieysztoria quadridens	2		
Gieysztoria quadridensoides	2		
Gieysztoria queenslandica	2		
Gieysztoria ramayana	1		
Gieysztoria reggae	4		
Gieysztoria rhombigera	1		
Gieysztoria rubra	12		
Gieysztoria saganae	3		
Gieysztoria santafeensis	4		
Gieysztoria sasa	2		
Gieysztoria semispinosa	1		
Gieysztoria shantouensis	1		
Gieysztoria shenzhensis	1		
Gieysztoria shiyanensis	1		
Gieysztoria sibirica	1		
Gieysztoria stokesi	1		
Gieysztoria subsalsa	1		
Gieysztoria superba	19		
Gieysztoria taurica	1		
Gieysztoria therapaina	4		
Gieysztoria thienemanni	3		
Gieysztoria thymara	2		
Gieysztoria tigrensis	2		
Gieysztoria transilvana	1		
Gieysztoria triangulata	4		
Gieysztoria tridesma	3		
Gieysztoria triquetra	6		
Gieysztoria trisolena	3		
Gieysztoria uncia	3		
Gieysztoria variata	2		
Gieysztoria virgulifera	6		
Gieysztoria wuyishanensis	1		
Gieysztoria zuluensis	2		
Gieysztoria zykovi	1		

Gigantapseudes adactylus	5		
Gigantapseudes maximus	9		
Indocotyle elegans	2		
Indocotyle hemirhamphi	2		
Indodidymozoon branchiale	10	1	
Indodidymozoon brevicolle	3	1	
Indodidymozoon ditremion	2	1	
Indodidymozoon koti (Didymozoon koti)	3	1	
Indodidymozoon lesteri	3	1	
Indodidymozoon madhaviae	2	1	
Indodidymozoon metridion	3	1	
Indodidymozoon moretonensis	1	1	
Indodidymozoon pearsoni	21	1	
Indodidymozoon platycephali	3	1	
Indodidymozoon suttiei	1	1	
Indodidyozoon musculi	1	1	
Indolaophonte gemmarum	4		
Indolaophonte ramai	5		
Indomodulus tectum	9		
Indopeniculus fryeri	4	parasite	
Indopeniculus siamensis	1	parasite	
Indothais blanfordi (Purpura blanfordi, Thais blanfordi)	11		
Indothais dubia (Cymmia dubia, thais dubia)	1		
Indothais gradata (Purpura gradata)	20		
Indothais javanica (Purpura javanica, thais javanica)	3		
Indothais lacera	30		
Indothais malayensis (Thais malayensis)	6		
Indothais pinangensis (Thais pinangensis)	1		
Indothais rufotincta (thais rufotincta)	6		
Indothais sacellum (Murex sacellum, Purpora diadema, Lataxiena taiwanica)	6		
Indothais scalaris (Purpura scalaris)	1		

Indothais wutingi (Thais wuttingi)	1		
Inella acicula	2		
Inella aculeata	1		
Inella aoteaensis	1		1
Inella apexbilirata	1		
Inella asperrima	2		
Inella blainvilli	1		
Inella carinata	1		
Inella chrysalis	1		
Inella colon	1		
Inella cordata	2		1
Inella dauciformis	1		1
Inella differens	1		
Inella faberi	1		
Inella gemmulata	1		
Inella gigas	2		
Inella harryleei	1		
Inella ile	1		
Inella intercalaris	1		
Inella intermedia	1		
Inella japonica	5		
Inella kimbiae	1		
Inella lanceolata	1		
Inella longissima	1		
Inella mariei	1		
Inella micans	1		
Inella moniliferata	1		1
Inella multitecta	1		
Inella noduloides	1		
Inella numerosa	1		
Inella obliqua	1		
Inella obtusa	1		
Inella perimensis	1		
Inella pinarena	1		
Inella planaria	1		
Inella pseudolongissima	1		
Inella pseudotorticula	1		
Inella rossiteri	1		
Inella ryosukei	1		
Inella sagamiensis	2		
Inella sarissa	1		
Inella undebermuda	1		

Inella unicornium	3		
Inella verluysi	1		
Inella verrucosa	4		
Inella vittata	1		
Inella zeattenuata	1		1
Inella zespina (Notosinister zespina)	1		1
Inermiphonte danversae (laophonte danversae)	8		
Inermiphonte drachi (laophonte drachi)	8		
Inermiphonte hamondi	1		
Ingenia communis	2		
Ingenia mirabilis	4		
Intasia amblynema	1		
Intasia lympha	1		
Intasia monohystera	11		
Intasia nojii	3		
Intercletodes interita	8		
Intermedichelia gracilis	7		
Intermedichelia jesseri	3		
Orecturus ampulus	1		
Orecturus antillensis	1		
Orecturus bahiensis	3		
Orecturus bracatus	4		
Orecturus canariensis	2		
Orecturus excavatus	6		
Orecturus finitimus	3		
Orecturus forticulus	2		
Orecturus grandisetiger	5		
Orecturus longicaudatus	1		
Orecturus ortizi	1		
Orecturus sakalavicus	8		
Orecturus similis	1		
Oregonia bifurca	23	1	
Oregonia gracilis	146	1	
Orenoqueia serrata	2		
Orientocreadium batrachoides	50	1	
Orientocreadium chaenogobii	3	1	
Orientocreadium indicum	5	1	
Orientocreadium pseudobagri	17	1	
Orithyia sinica	81	1	
Ornatoleberis botanybayensis	1		

Ornatoleberis fortii	1		
Ornatoleberis morkhoveni	19		
Ornatoleberis parva	1		
Ornithobilharzia canaliculata	74	1	
Orobitella chacei	1		
Orobitella floridana	14		
Orobitella jipijapa	1		
Orobitella limpida	3		
Orobitella margarita	1		
Orobitella oblonga	1		
Orobitella zorrita	1		
Orozoe marginata	1		1
Orozoe mira	2		1
Orphnurgus asper	6		
Orphnurgus aspersignis	1		
Orphnurgus bacillus	1		
Orphnurgus dorisae	1		
Orphnurgus glaber (syn: orphnurgus invalidus and orphnurgus rigidus)	5		
Orphnurgus insignis	4		
Orphnurgus natalasper	1		
Orphnurgus protectus	1		
Orphnurgus vitreus	1		
Orstomella faviae	3		
Orstomella lobophylliae	2		
Orstomella yaliuensis	7		
Orthagoriscicola muricatus	11		
Orthobairdia anguuta	1		1
Orthobairdia antamputata	1		1
Orthobairdia auricula	1		1
Orthobairdia bidorsalis	1		1
Orthobairdia crassa	1		1
Orthobairdia insolens	1		1
Orthobairdia oklahomaensis	6		1
Orthobairdia perincerta	1		1
Orthobairdia powersi	2		1
Orthobairdia subreniformis	1		1
Orthobairdia texana	5		1
Parinoberyx horridus	4		
Parkius karenwishnerae	13		
Parkraemeria ornata	9		
Parkraemeria rhinoceros	1		

Parkraemeria saltator	1		
Paromola bathyalis	9		
Paromola crosnieri	3		
Paromola cuvieri	224		
Paromola japonica	23		
Paromola macrochira	13		
Paromola rathbuni	6		
Paropecoelus bhabhai	1		
Paropecoelus corneliae	2		
Paropecoelus dollfusi	1		
Paropecoelus elongatus	3		
Paropecoelus filiformis	1		
Paropecoelus indicus	2		
Paropecoelus lanceolatus	3		
Paropecoelus leonae	2		
Paropecoelus manteri	1		
Paropecoelus overstreeti	1		
Paropecoelus palawanensis	3		
Paropecoelus parupenei	2		
Paropecoelus pritchardae	1		
Paropecoelus quadratus	1		
Paropecoelus sacculatus	3		
Paropecoelus sciaeni	1		
Paropecoelus sogandaresi	3		
Paropecoelus thapari	2		
Paropecoelus theraponi	1		
Paropecoelus upenoidis	2		
Parophiopsyllus ligatus	*4*		
Parophrys vetulus (Parophrys hubbardii, Parophrys ischyrus, Parophrys vetula, Pleuronectes digrammus, pleuronectes vetulus)	4628	1	
Paroriza grevei	4		
Paroriza pallens	36		
Paroriza prouhoi	43		
Paroriza verrucosa	2		
Parulrichia bispinosa	1		1
Parulrichia diversa	1		1
Parvaplustrum cadieni	1		
Parvaplustrum japonicum	1		
Parvaplustrum tenerum	5		
Parvidrilus camachoi	2		

Parvidrilus gianii	2		
Parvidrilus jugeti	1		
Parvidrilus meyssonnieri	2		
Parvidrilus spelaeus	16		
Parvidrilus stochi	1		
Parvidrilus strayeri	16		
Parvidrilus tomasini	1		
Parvilucina crenella	22		
Parvilucina ephraimi	1		1
Parvilucina latens	2		
Parvilucina mazatlanica	15		
Parvilucina pectinella	12		
Parvilucina tenuisculpta	133		
Parvilucina yamakawai	1		
Qianlabeo striatus	15		
Quadricolina initialis	1		1
Quadritestis almehensis	1		
Quasibuntonia radiatopora	20		1
Quasibuntonia sculpta	2		1
Quasibuntonia seguenziana	3		1
Quasibuntonia triquetra	1		1
Quasiknoxiella sp.	4		1
Quidnipagus palatam	48	1	
Quoiecchia aliciae	*4*		1
Quoiecchia sigeli	1		1
Waiparacythereis joanae	15		1
Wallerconcha sarae	3		1
Walvisteuthis jeremiahi	9		
Walvisteuthis rancureli	10		
Walvisteuthis virilis	8		
Walvisteuthis youngorum	8		
Watatsumi grubei	3		
Wattsia mossambicus	12		
Weddellaophonte anyae	2		
Weitschatopsis sp.	2		1
Wherlina sp.	0		1
Yamaguchiella coerulescens	8		
Yangella cuneatiformis	1		1
Yangella sichuanensis	1		1
Yaninella belbekensis	1		1
Yaquinabyssia careyi	4		

Yarica hyalosoma (Apogon hyalosoma; Mionorus bombonensis)	81		
Yemenicaris trullicauda	5		
Yezocythere hayashii	20		1
Yiduella sp.	1		1
Yokoyamaia orientalis	1		
Yokoyamaia ornatissima	12		
Yorkeria chiloscyllii	5		
Yorkeria chonburiensis	2		
Yorkeria garneri	4		
Yorkeria hilli	8		
Yorkeria indica	1		
Yorkeria izardi	6		
Yorkeria kelleyae	5		
Yorkeria longstaffae	4		
Yorkeria moretonensis	1		
Yorkeria parva	10		
Yorkeria pusillulus	3		
Yorkeria saliputium	7		
Yorkeria southwelli	3		
Yorkeria teeveeyi	3		
Yorkeria williamsi	1		
Yorkeria xiamenensis	4		
Yorkeria yubodohensis	5		
Ypsilothuria bitentaculata (sphaerothuria bitentaculata)	54		
Ypsilothuria talismani (Sphaerothuria talismani)	26		
Ysideria hastata	1		
Yuebeipotamon calciatile	7	1 (aquarium)	
Yukonibolbina plicata	1		1
Yunnanocopia grandis	1		1
Yunnanocopia longicauda	1		1
Zacatrophon beebei	1		
Zacatrophon skoglundae	1		
Zaletrigonia hoepeni	1		1
Zaletrigonia inconstans	2		1
Zaletrigonia normanbyia	1		1
Zaletrigonia telefominiana	1		1
Zalieutes elater (Malthe elater)	53		
Zalieutes mcgintyi	15		

Zaniolepis frenata	82	1	
Zaniolepis latipinnis	120	1	
Zanobatus maculatus	4		
Zanobatus schoenleinii	42		
Zaops angelae	17		
Zaops geddesi	7		
Zaops ostreus (Pinnotheres ostreus; Pinnotheres depressum)	376	1	
Zazaranus fungicolus	2		
Zelithophaga truncata	26		

Neogastropod family	Conoidean?	Number of lots (no aff etc)	Raw Number of lots in family	percent culled for no species ID
TURRIDAE	Yes	1986	2001	52.57%
COSTELLARIIDAE	No	217	232	39.66%
OLIVIDAE	No	2313	2360	38.43%
DRILLIIDAE	Yes	1659	1731	38.07%
TEREBRIDAE	Yes	1503	1551	37.14%
MANGELIIDAE	Yes	86	87	35.63%
MARGINELLIDAE	No	2872	2944	35.05%
MITRIDAE	No	1087	1104	34.15%
PSEUDOMELATOMIDAE	Yes	54	59	33.90%
HARPIDAE	No	63	63	31.75%
COLUMBELLIDAE	No	1857	1910	29.69%
BUCCINIDAE	No	1957	1983	29.50%
NASSARIIDAE	No	2277	2332	28.52%
CANCELLARIIDAE	No	2587	2622	27.35%
MELONGENIDAE	No	5545	5611	26.38%
RAPHITOMIDAE	Yes	79	79	22.78%
FASCIOLARIIDAE	No	4600	4633	21.50%
CYSTISCIDAE	No	58	58	20.69%
VOLUTIDAE	No	1200	1214	20.51%
CONIDAE	Yes	4332	4429	15.69%
TURBINELLIDAE	No	2054	2067	15.09%
MURICIDAE	No	8280	8409	13.21%

Number of specimens	Number lots ID genus	Number lots ID Species	Number of genera	Number of species	% Lots ID to Genus	% Lots ID to Species	Notes
7969	1377	949	32	87	69.3%	47.8%	
1171	217	140	5	14	100.0%	64.5%	
31143	2303	1453	7	53	99.6%	62.8%	
8926	1650	1072	13	59	99.5%	64.6%	
10701	1439	975	7	28	95.7%	64.9%	
345	83	56	3	10	96.5%	65.1%	
31503	2790	1912	13	66	97.1%	66.6%	
8020	1080	727	5	29	99.4%	66.9%	
300	53	39	3	5	98.1%	72.2%	
206	63	43	1	4	100.0%	68.3%	
16527	1769	1343	26	59	95.3%	72.3%	
14875	1856	1398	19	63	94.8%	71.4%	
23545	2248	1667	9	58	98.7%	73.2%	
15723	2581	1905	12	55	99.8%	73.6%	
22976	5477	4131	9	93	98.8%	74.5%	
592	79	61	2	4	100.0%	77.2%	
22956	4530	3637	16	67	98.5%	79.1%	
1355	57	46	2	3	98.3%	79.3%	753 lots assigned to [illegible]
5956	1194	965	3	25	99.5%	80.4%	
37005	4325	3734	3	66	99.8%	86.2%	
7269	2051	1755	3	24	99.9%	85.4%	
56257	8197	7298	47	133	99.0%	88.1%	

Cymatosyrinx

Class	Family	number of species epithets	Total number occcs	number kr
Bivalvia	Anatinellidae	2	23	0
Bivalvia	Anomiidae	6	85	0
Bivalvia	Arcidae	38	341	0
Bivalvia	Astartidae	14	105	0
Gastropod	Batillariidae	13	28	0
Gastropod	Buccinidae	32	71	0
Gastropod	Bullidae	3	27	0
Gastropod	Caecidae	18	58	0
Gastropod	Calyptraeidae	32	312	0
Gastropod	Cancellariidae	29	86	1
Bivalvia	Cardiidae	33	200	3
Bivalvia	Carditidae	19	263	1
Gastropod	Cassidae	9	20	0
Gastropod	Cerithiidae	20	95	0
Gastropod	Cerithiopsidae	12	55	0
Bivalvia	Chamidae	11	96	0
Gastropod	Clathurellidae	11	17	0
Gastropod	Columbellidae	39	171	0
Gastropod	Conidae	33	142	0
Bivalvia	Corbulidae	14	142	0
Bivalvia	Crassatellidae	12	135	0
Gastropod	Cylichnidae	8	68	0
Gastropod	Cypraeidae	52	80	0
Gastropod	Cystiscidae	4	18	0
Bivalvia	Donacidae	6	50	0
Gastropod	Drilliidae	20	66	0
Gastropod	Epitoniidae	34	90	0
Gastropod	Eulimidae	25	71	0
Gastropod	Fasciolariidae	50	159	0
Gastropod	Ficidae	4	15	0
Gastropod	Fissurellidae	20	76	0
Bivalvia	Glycymerididae	10	152	0
Bivalvia	Gryphaeidae	3	42	0
Bivalvia	Hiatellidae	6	37	0
Bivalvia	Kelliidae	5	21	0
Bivalvia	Limidae	9	21	0
Gastropod	Littorinidae	7	27	0
Bivalvia	Lucinidae	46	407	3
Bivalvia	Mactridae	16	271	5
Gastropod	Mangeliidae	22	55	0
Gastropod	Marginellidae	30	147	0
Gastropod	Melongenidae	46	265	1
Gastropod	Mitridae	9	19	1
Gastropod	Modulidae	7	26	0
Bivalvia	Montacutidae	7	20	0
Gastropod	Muricidae	54	549	0

Bivalvia	Myidae	4	29	0
Bivalvia	Mytilidae	16	79	0
Gastropod	Nassariidae	40	240	0
Gastropod	Naticidae	24	178	2
Bivalvia	Noetiidae	8	114	0
Bivalvia	Nuculanidae	4	78	0
Bivalvia	Nuculidae	2	85	0
Gastropod	Olividae	43	195	0
Bivalvia	Ostreidae	12	221	0
Gastropod	Ovulidae	12	19	0
Bivalvia	Pandoridae		41	0
Bivalvia	Pectinidae	52	344	0
Bivalvia	Pharidae	5	58	0
Bivalvia	Pholadidae	9	31	0
Bivalvia	Pinnidae	4	18	0
Gastropod	Planorbidae	14	57	0
Bivalvia	Plicatulidae	2	80	0
Gastropod	Polygyridae	9	33	0
Gastropod	Potamididae	11	17	0
Gastropod	Pseudomelaniidae	10	22	0
Bivalvia	Pteriidae	3	16	0
Gastropod	Pyramidellidae	21	122	0
Gastropod	Rissoidae	18	27	0
Bivalvia	Semelidae	22	193	0
Bivalvia	Solecurtidae	5	32	0
Bivalvia	Sportellidae	15	43	0
Gastropod	Strombidae	28	72	0
Bivalvia	Tellinidae	41	265	6
Gastropod	Terebridae	20	107	0
Gastropod	Tornidae	24	47	0
Gastropod	Triviidae	9	29	0
Gastropod	Trochidae	21	51	0
Gastropod	Turbinellidae	31	57	0
Gastropod	Turbinidae	16	45	0
Gastropod	Turridae	12	34	11
Gastropod	Turritellidae	34	210	1
Bivalvia	Ungulinidae	14	68	0
Bivalvia	Veneridae	69	755	4
Bivalvia	Veneridae listed as Pet	7	26	0
Gastropod	Vermetidae	8	46	1
Gastropod	Vitrinellidae	20	46	0
Gastropod	Viviparidae	1	21	0
Gastropod	Volutidae	23	41	0
Bivalvia	Yoldiidae	4	21	0
**TOTALS**	**ALL**	1647	9537	40
	**Bivalve**	725	4143	
	**Gastropods**	922	5394	

**Notes**	Removed 81 families, 461 occurrences for having less than 15 occcs;
	spelling errors removed, existing PBDB corrections and subjective synor
	identified as "sp. 1/A" not counted as ID to species
	misidentification for turritellids was high (species not present in region/
	c.f., aff. And known to subgenus treated as known to genus

number kr	number known t	Fraction kr	less than 9	less than 80
1	22	96%	0	0
12	73	86%	1	0
12	329	96%	0	0
11	94	90%	1	0
1	27	96%	0	0
6	65	92%	0	0
1	26	96%	0	0
4	54	93%	0	0
17	295	95%	0	0
19	66	77%	1	1
20	177	89%	1	0
10	252	96%	0	0
0	20	100%	0	0
28	67	71%	1	1
3	52	95%	0	0
3	93	97%	0	0
2	15	88%	1	0
20	151	88%	1	0
21	121	85%	1	0
13	129	91%	0	0
19	116	86%	1	0
5	63	93%	0	0
0	80	100%	0	0
0	18	100%	0	0
4	46	92%	0	0
17	49	74%	1	1
21	69	77%	1	1
18	53	75%	1	1
19	140	88%	1	0
2	13	87%	1	0
17	59	78%	1	1
15	137	90%	0	0
1	41	98%	0	0
1	36	97%	0	0
2	19	90%	0	0
5	16	76%	1	1
1	26	96%	0	0
20	384	94%	0	0
30	236	87%	1	0
9	46	84%	1	0
51	96	65%	1	1
77	187	71%	1	1
2	16	84%	1	0
0	26	100%	0	0
1	19	95%	0	0
27	522	95%	0	0

7	22	76%	1	1	
29	50	63%	1	1	
31	209	87%	1	0	
17	159	89%	1	0	
11	103	90%	0	0	
18	60	77%	1	1	
1	84	99%	0	0	
25	170	87%	1	0	
39	182	82%	1	0	
0	19	100%	0	0	
7	34	83%	1	0	
46	298	87%	1	0	
1	57	98%	0	0	
3	28	90%	0	0	
11	7	39%	1	1	
3	54	95%	0	0	
6	74	93%	0	0	
10	23	70%	1	1	
0	17	100%	0	0	
7	15	68%	1	1	
2	14	88%	1	0	
56	66	54%	1	1	
1	26	96%	0	0	
10	183	95%	0	0	
4	28	88%	1	0	
6	37	86%	1	0	
7	65	90%	0	0	
84	175	66%	1	1	
21	86	80%	1	0	
9	38	81%	1	0	
3	26	90%	1	0	
16	35	69%	1	1	
3	54	95%	0	0	
3	42	93%	0	0	
4	19	56%	1	1	
29	180	86%	1	0	
6	62	91%	0	0	
67	684	91%	0	0	
0	26	100%	0	0	
2	43	93%	0	0	
6	40	87%	1	0	
0	21	100%	0	0	
5	36	88%	1	0	
3	18	86%	1	0	
	8310	87%	1	0	
	3729	90%	0	0	
	4581	85%	1	0	

ıymes removed, species entered in multiple genera only counted once

/at the time period)

Family	number of FMNH lots	Number of OCCS	FMNH Species richness
MANGELIIDAE	86	147	10
CYSTISCIDAE	58	18	3
TURBINELLIDAE	2054	57	24
VOLUTIDAE	1200	41	25
OLIVIDAE	2313	195	53
FASCIOLARIIDAE	4600	159	67
TEREBRIDAE	1503	107	28
NASSARIIDAE	2277	240	58
COLUMBELLIDAE	1857	171	59
CANCELLARIIDAE	2587	86	55
BUCCINIDAE	1957	71	63
CONIDAE	4332	142	66
MELONGENIDAE	5545	265	93
MARGINELLIDAE	2872	147	66
MURICIDAE	8280	549	133
DRILLIIDAE	1659	66	59
MITRIDAE	1087	19	29
TURRIDAE	1986	34	87

(Less than 50 removed)

The families Costellariidae, Harpidae, Pseudomelatomidae, Raphitomida

spearman rho for occs is .6722, p value < 0.01; (0.0022)

PBDB species richness	
22	-0.375
4	-0.14286
31	-0.12727
23	0.041667
43	0.104167
50	0.145299
20	0.166667
40	0.183673
39	0.204082
29	0.309524
32	0.326316
33	0.333333
46	0.338129
30	0.375
54	0.42246
20	0.493671
9	0.526316
12	0.757576

ae were removed (no overlap)

Class	Family	number of species epithets	Total number occs	number known to family only
Gastropoda	Acteocinidae	2	4	0
Gastropoda	Amathinidae	2	2	0
Gastropoda	Ampullariidae	1	7	0
Gastropoda	Ampullinidae	1	1	0
Bivalvia	Anatinellidae	2	23	0
Gastropoda	Architectonicidae	3	11	0
Bivalvia	Arcidae	38	341	0
Gastropoda	Areneidae	1	11	0
Bivalvia	Aviculidae	1	1	0
Gastropoda	Batillariidae	13	28	0
Gastropoda	Buccinidae	32	71	0
Gastropoda	Bullidae	3	27	0
Gastropoda	Bursidae	2	3	0
Gastropoda	Caecidae	18	58	0
Gastropoda	Calyptraeidae	32	312	0
Gastropoda	Campanilidae	6	13	0
Bivalvia	Carditidae	19	263	1
Gastropoda	Cassidae	9	20	0
Gastropoda	Cerithiopsidae	12	55	0
Bivalvia	Chamidae	11	96	0
Gastropoda	Clavatulidae	1	3	0
Gastropoda	Cochlespiridae	3	7	0
Bivalvia	Condylocardiidae	1	2	0
Bivalvia	Corbiculidae	2	8	0
Bivalvia	Corbulidae	14	142	0
Gastropoda	Costellariidae	7	12	0
Bivalvia	Crenellidae	3	6	0
Bivalvia	Cuspidariidae	6	8	0
Gastropoda	Cylichnidae	8	68	0
Gastropoda	Cypraeidae	52	80	0
Bivalvia	Cyrenoididae	1	1	0
Gastropoda	Cystiscidae	4	18	0
Gastropoda	Discidae	2	3	0
Bivalvia	Donacidae	6	50	0
Gastropoda	Ellobiidae	4	7	0
Gastropoda	Endodontidae	3	4	0
Bivalvia	Gastrochaenidae	5	11	0
Gastropoda	Gastrocoptidae	1	8	0
Bivalvia	Glossidae	1	8	0
Bivalvia	Glycymerididae	10	152	0
Bivalvia	Gryphaeidae	3	42	0
Gastropoda	Helicinidae	1	2	0
Bivalvia	Hiatellidae	6	37	0
Gastropoda	Hipponicidae	2	2	0
Bivalvia	Kelliidae	5	21	0

Bivalvia	Lasaeidae	4	9	0
Gastropoda	Littorinidae	7	27	0
Bivalvia	Lucinidae	46	407	3
Gastropoda	Lymnaeidae	4	8	0
Bivalvia	Lyonsiidae	1	1	0
Gastropoda	Magilidae	1	1	0
Bivalvia	Margaritariidae	1	3	0
Gastropoda	Mitromorphidae	2	3	0
Gastropoda	Modulidae	7	26	0
Bivalvia	Montacutidae	7	20	0
Gastropoda	Muricidae	54	549	0
Bivalvia	Noetiidae	8	114	0
Bivalvia	Nucinellidae	1	2	0
Bivalvia	Nuculidae	2	85	0
Gastropoda	Ovulidae	12	19	0
Bivalvia	Periplomatidae	2	3	0
Bivalvia	Pharidae	5	58	0
Gastropoda	Phasianellidae	2	2	0
Bivalvia	Pholadidae	9	31	0
Bivalvia	Pichleriidae	1	1	0
Gastropoda	Planaxidae	2	5	0
Gastropoda	Planorbidae	14	57	0
Bivalvia	Plicatulidae	2	80	0
Gastropoda	Potamididae	11	17	0
Gastropoda	Ranellidae	3	6	0
Gastropoda	Raphitomidae	1	1	0
Gastropoda	Retusidae	3	4	0
Gastropoda	Rissoidae	18	27	0
Gastropoda	Scaliolidae	2	14	0
Bivalvia	Semelidae	22	193	0
Gastropoda	Skeneidae	7	14	0
Bivalvia	Solemyidae	1	1	0
Gastropoda	Spiraxidae	1	6	0
Gastropoda	Strobilopsidae	4	4	0
Gastropoda	Strombidae	28	72	0
Gastropoda	Tegulidae	4	7	0
Bivalvia	Trapezidae	1	1	0
Gastropoda	Turbinellidae	31	57	0
Gastropoda	Turbinidae	16	45	0
Bivalvia	Ungulinidae	14	68	0
Bivalvia	Unionidae	0	1	0
Gastropoda	Valloniidae	1	1	0
Bivalvia	Veneridae	69	755	4
Bivalvia	Veneridae listed as Petrico	7	26	0
Gastropoda	Vermetidae	8	46	1
Bivalvia	Verticordiidae	2	7	0
Gastropoda	Viviparidae	1	21	0

Gastropoda	Xenophoridae	2	6	0
Gastropoda	Zonitidae	5	8	0
Bivalvia	Anomiidae	6	85	0
Bivalvia	Astartidae	14	105	0
Bivalvia	Cardiidae	33	200	3
Gastropoda	Clathurellidae	11	17	0
Gastropoda	Columbellidae	39	171	0
Gastropoda	Conidae	33	142	0
Bivalvia	Crassatellidae	12	135	0
Bivalvia	Erycinidae	2	8	0
Gastropoda	Fasciolariidae	50	159	0
Gastropoda	Ficidae	4	15	0
Gastropoda	Liotiidae	5	11	0
Bivalvia	Mactridae	16	271	5
Bivalvia	Malleidae	3	7	0
Gastropoda	Mangeliidae	22	55	0
Gastropoda	Mitridae	9	19	1
Gastropoda	Nassariidae	40	240	0
Gastropoda	Naticidae	24	178	2
Gastropoda	Olividae	43	195	0
Bivalvia	Ostreidae	12	221	0
Bivalvia	Pandoridae	6	41	0
Bivalvia	Pectinidae	52	344	0
Bivalvia	Pteriidae	3	16	0
Gastropoda	Pupillidae	5	11	0
Gastropoda	Ringiculidae	1	6	0
Bivalvia	Solecurtidae	5	32	0
Bivalvia	Sportellidae	15	43	0
Gastropoda	Terebridae	20	107	0
Bivalvia	Teredinidae	2	14	2
Gastropoda	Tornidae	24	47	0
Gastropoda	Triviidae	9	29	0
Gastropoda	Turritellidae	34	210	1
Gastropoda	Vitrinellidae	20	46	0
Gastropoda	Volutidae	23	41	0
Bivalvia	Yoldiidae	4	21	0
Gastropoda	Acteonidae	4	9	0
Gastropoda	Amnicolidae	4	10	0
Gastropoda	Cancellariidae	29	86	1
Gastropoda	Capulidae	0	1	0
Gastropoda	Cerithiidae	20	95	0
Gastropoda	Discohelicidae	1	1	0
Bivalvia	Dreissenidae	2	4	0
Gastropoda	Drilliidae	20	66	0
Gastropoda	Epitoniidae	34	90	0
Gastropoda	Eulimidae	25	71	0
Gastropoda	Fissurellidae	20	76	0

Gastropoda	Haminoeidae	3	6	0
Gastropoda	Harpidae	2	7	0
Gastropoda	Horaiclavidae	3	3	0
Gastropoda	Hydrobiidae	3	7	1
Bivalvia	Limidae	9	21	0
Gastropoda	Lottiidae	2	3	0
Gastropoda	Marginellidae	30	147	0
Gastropoda	Melongenidae	46	265	1
Bivalvia	Myidae	4	29	0
Bivalvia	Myochamidae	0	2	0
Bivalvia	Mytilidae	16	79	0
Gastropoda	Neritidae	4	9	0
Bivalvia	Nuculanidae	4	78	0
Gastropoda	Physidae	2	11	0
Bivalvia	Pinnidae	4	18	0
Gastropoda	Polygyridae	9	33	0
Bivalvia	Poromyidae	1	2	0
Bivalvia	Psammobiidae	1	5	1
Gastropoda	Pseudomelaniidae	10	22	0
Gastropoda	Pyramidellidae	21	122	0
Gastropoda	Solariellidae	2	5	0
Bivalvia	Solenidae	2	6	0
Bivalvia	Sphaeriidae	3	5	0
Bivalvia	Spondylidae	3	11	0
Bivalvia	Tellinidae	41	265	6
Bivalvia	Thraciidae	6	14	0
Bivalvia	Thyasiridae	1	2	0
Gastropoda	Tonnidae	2	6	0
Gastropoda	Triphoridae	3	10	0
Gastropoda	Trochidae	21	51	0
Gastropoda	Turridae	12	34	11
Gastropoda	Valvatidae	0	1	0

NOTES:

spelling errors removed, existing PBDB corrections and subjective sync
identified as "sp. 1/A" not counted as ID to species
misidentification for turritellids was high (species not present in region
c.f., aff. And known to subgenus treated as known to genus

number known to genus only	number known to species	Fraction known to species	less than 90	less than 80
0	4	1.00	0	0
0	2	1.00	0	0
0	7	1.00	0	0
0	1	1.00	0	0
1	22	0.96	0	0
0	11	1.00	0	0
12	329	0.96	0	0
0	11	1.00	0	0
0	1	1.00	0	0
1	27	0.96	0	0
6	65	0.92	0	0
1	26	0.96	0	0
0	3	1.00	0	0
4	54	0.93	0	0
17	295	0.95	0	0
1	12	0.92	0	0
10	252	0.96	0	0
0	20	1.00	0	0
3	52	0.95	0	0
3	93	0.97	0	0
0	3	1.00	0	0
0	7	1.00	0	0
0	2	1.00	0	0
0	8	1.00	0	0
13	129	0.91	0	0
1	11	0.92	0	0
0	6	1.00	0	0
0	8	1.00	0	0
5	63	0.93	0	0
0	80	1.00	0	0
0	1	1.00	0	0
0	18	1.00	0	0
0	3	1.00	0	0
4	46	0.92	0	0
0	7	1.00	0	0
0	4	1.00	0	0
1	10	0.91	0	0
0	8	1.00	0	0
0	8	1.00	0	0
15	137	0.90	0	0
1	41	0.98	0	0
0	2	1.00	0	0
1	36	0.97	0	0
0	2	1.00	0	0
2	19	0.90	0	0

0	9	1.00	0	0
1	26	0.96	0	0
20	384	0.94	0	0
0	8	1.00	0	0
0	1	1.00	0	0
0	1	1.00	0	0
0	3	1.00	0	0
0	3	1.00	0	0
0	26	1.00	0	0
1	19	0.95	0	0
27	522	0.95	0	0
11	103	0.90	0	0
0	2	1.00	0	0
1	84	0.99	0	0
0	19	1.00	0	0
0	3	1.00	0	0
1	57	0.98	0	0
0	2	1.00	0	0
3	28	0.90	0	0
0	1	1.00	0	0
0	5	1.00	0	0
3	54	0.95	0	0
6	74	0.93	0	0
0	17	1.00	0	0
0	6	1.00	0	0
0	1	1.00	0	0
0	4	1.00	0	0
1	26	0.96	0	0
0	14	1.00	0	0
10	183	0.95	0	0
0	14	1.00	0	0
0	1	1.00	0	0
0	6	1.00	0	0
0	4	1.00	0	0
7	65	0.90	0	0
0	7	1.00	0	0
0	1	1.00	0	0
3	54	0.95	0	0
3	42	0.93	0	0
6	62	0.91	0	0
0	1	1.00	0	0
0	1	1.00	0	0
67	684	0.91	0	0
0	26	1.00	0	0
2	43	0.93	0	0
0	7	1.00	0	0
0	21	1.00	0	0

0	6	1.00	0	0
0	8	1.00	0	0
12	73	0.86	1	0
11	94	0.90	1	0
20	177	0.89	1	0
2	15	0.88	1	0
20	151	0.88	1	0
21	121	0.85	1	0
19	116	0.86	1	0
1	7	0.88	1	0
19	140	0.88	1	0
2	13	0.87	1	0
2	9	0.82	1	0
30	236	0.87	1	0
1	6	0.86	1	0
9	46	0.84	1	0
2	16	0.84	1	0
31	209	0.87	1	0
17	159	0.89	1	0
25	170	0.87	1	0
39	182	0.82	1	0
7	34	0.83	1	0
46	298	0.87	1	0
2	14	0.88	1	0
2	9	0.82	1	0
1	5	0.83	1	0
4	28	0.88	1	0
6	37	0.86	1	0
21	86	0.80	1	0
0	12	0.86	1	0
9	38	0.81	1	0
3	26	0.90	1	0
29	180	0.86	1	0
6	40	0.87	1	0
5	36	0.88	1	0
3	18	0.86	1	0
2	7	0.78	1	1
4	6	0.60	1	1
19	66	0.77	1	1
1	0	0.00	1	1
28	67	0.71	1	1
1	0	0.00	1	1
1	3	0.75	1	1
17	49	0.74	1	1
21	69	0.77	1	1
18	53	0.75	1	1
17	59	0.78	1	1

2	4	0.67	1	1
3	4	0.57	1	1
1	2	0.67	1	1
3	3	0.43	1	1
5	16	0.76	1	1
1	2	0.67	1	1
51	96	0.65	1	1
77	187	0.71	1	1
7	22	0.76	1	1
2	0	0.00	1	1
29	50	0.63	1	1
2	7	0.78	1	1
18	60	0.77	1	1
7	4	0.36	1	1
11	7	0.39	1	1
10	23	0.70	1	1
1	1	0.50	1	1
3	1	0.20	1	1
7	15	0.68	1	1
56	66	0.54	1	1
2	3	0.60	1	1
2	4	0.67	1	1
2	3	0.60	1	1
4	7	0.64	1	1
84	175	0.66	1	1
6	8	0.57	1	1
1	1	0.50	1	1
2	4	0.67	1	1
6	4	0.40	1	1
16	35	0.69	1	1
4	19	0.56	1	1
1	0	0.00	1	1

›nymes removed, species entered in multiple genera only counted once

ı/at the time period)

Data Provi	The Paleobiology Database						
Data Sourc	The Paleobiology Database						
Data Licen:	Creative Commons CC-BY						
License UR	http://creativecommons.org/licenses/by/4.0/						
Document	http://paleobiodb.org/data1.2/occs/list_doc.html						
Data URL	http://paleobiodb.org/data1.2/occs/list.csv?datainfo&rowcount&base_name=Mollus						
Access Tim	Fri 2023-06-30 00:57:22 GMT						
Title	PBDB Data Service						
Parameters:							
	base_nam	Mollusca					
	pgm	gplates,scotese,seton					
	cc	US					
	state	Florida					
	interval	Pliocene,Pleistocene					
	timerule	major					
	taxon_stat	all					
	show	attr,class,classext,genus,pres,loc,paleoloc,lithext,refattr					
Elapsed Tir	0.488						
Records Fc	5594						
Records Re	5594						
Records:							
occurrence_no	record_type	reid_no	flags	collection_no	identified_name	identified_rank	identified_no
582636	occ			61310	Raeta plica	species	156772
582645	occ			61311	Raeta plica	species	156772
582657	occ			61309	Raeta plica	species	156772
623013	occ			67069	Labiosa an	species	91142
738358	occ			79331	Raeta cana	species	156774
747487	occ			79787	Raeta plica	species	108709
1088915	occ			96538	Raeta plica	species	108709
619728	occ			66760	Labiosa lin	species	156694
619729	occ			66760	Labiosa pli	species	108710
619730	occ			66756	Labiosa pli	species	108710
741368	occ			79707	Raeta plica	species	108709
741412	occ			79705	Raeta plica	species	108709
748119	occ			80083	Raeta plica	species	108709
564623	occ			58894	Raeta plica	species	108709
564648	occ			58895	Raeta plica	species	108709
564913	occ			58897	Raeta plica	species	108709
619727	occ			66742	Labiosa pli	species	108710
642745	occ			69045	Raeta plica	species	108709
738454	occ			79343	Raeta plica	species	108709
748534	occ			80033	Raeta sp.	genus	18175
559236	occ			57703	Raeta plica	species	108709
559249	occ			57704	Raeta plica	species	108709
737460	occ			79246	Raeta plica	species	108709
362485	occ			34269	Anomia sir	species	87561

423385	occ	41205	Anomia sir	species	87561
423394	occ	41206	Anomia sir	species	87561
425302	occ	41496	Anomia sir	species	87561
425319	occ	41497	Anomia sir	species	87561
425395	occ	41501	Anomia sir	species	87561
426076	occ	41539	Anomia sir	species	87561
427349	occ	41705	Anomia sir	species	87561
427689	occ	41718	Anomia sir	species	87561
427970	occ	41749	Anomia sir	species	87561
568622	occ	59490	Anomia sir	species	87561
568623	occ	59485	Anomia sir	species	87561
568624	occ	59484	Anomia sir	species	87561
568625	occ	59487	Anomia sir	species	87561
568626	occ	59484	Placunanoı	species	93765
568627	occ	59487	Placunanoı	species	93765
581017	occ	61155	Placunanoı	species	93765
582577	occ	61309	Anomia sir	species	87561
582591	occ	61310	Anomia sir	species	87561
582599	occ	61311	Anomia sir	species	87561
622949	occ	67069	Anomia sir	species	87561
623067	occ	67072	Anomia sir	species	87561
623080	occ	67074	Anomia sir	species	87561
623086	occ	67075	Anomia sir	species	87561
640723	occ	69087	Anomia sir	species	87561
697914	occ	75001	Anomia sp	genus	16476
697940	occ	75004	Anomia sp	genus	16476
698035	occ	75008	Anomia sp	genus	16476
738207	occ	79331	Anomia sp	genus	16476
742167	occ	79765	Placunanoı	species	93765
742218	occ	79338	Placunanoı	species	93772
742221	occ	79331	Placunanoı	species	93772
742439	occ	35026	Placunanoı	species	93772
743053	occ	79797	Anomia sir	species	87561
743054	occ	79797	Placunanoı	species	93765
745356	occ	79789	Anomia sp	genus	16476
745391	occ	79788	Anomia sp	genus	16476
745432	occ	69040	Anomia sir	species	87561
745541	occ	79962	Placunanoı	species	16925
747252	occ	69084	Anomia sir	species	87561
747486	occ	79787	Anomia sp	genus	16476
747606	occ	80037	Anomia sp	genus	16476
747607	occ	80037	Placunanoı	species	93765
747690	occ	80044	Placunanoı	species	93765
747772	occ	80046	Anomia sp	genus	16476
748126	occ	69034	Anomia sir	species	87561
748292	occ	80025	Anomia sir	species	87561
748325	occ	80024	Anomia sir	species	87561

1087891	occ		96538	Anomia sir	species	87561
256372	occ		20604	Anomia sir	species	87561
256390	occ		20606	Anomia sir	species	87561
578587	occ		60927	Anomia sir	species	87561
578648	occ		60928	Anomia sir	species	87561
578723	occ		60929	Anomia sir	species	87561
578750	occ		60930	Anomia sir	species	87561
578840	occ		60931	Anomia sir	species	87561
578892	occ		60940	Placunanoı	species	93765
578893	occ		60940	Placunanoı	species	93772
578894	occ		60940	Anomia sir	species	87561
619542	occ		66760	Anomia sir	species	87561
619547	occ		66755	Anomia sir	species	87561
619548	occ		66758	Anomia sir	species	87561
642870	occ		69046	Anomia sir	species	87561
745501	occ		79957	Anomia sir	species	87561
1457443	occ		150656	Anomia ep	species	90062
1457462	occ		203376	Anomia ep	species	90062
564319	occ		58895	Anomia sir	species	87561
564357	occ		58897	Anomia sir	species	87561
566573	occ		59315	Ostrea wa	species	120100
619543	occ		66742	Anomia sir	species	87561
619544	occ		66749	Anomia sir	species	87561
619545	occ		66751	Anomia sir	species	87561
619546	occ		66752	Anomia sir	species	87561
642724	occ		69045	Anomia sir	species	87561
701051	occ		69047	Anomia sir	species	87561
738513	occ		79339	Anomia sir	species	87561
748360	occ		80033	Anomia sir	species	87561
748570	occ		80035	Anomia sir	species	87561
557480	occ		57702	Anomia sir	species	87561
559142	occ		57705	Anomia sir	species	87561
559183	occ		57706	Anomia sir	species	87561
582214	occ		61293	Anomia ? s	genus	16476
737476	occ		79246	Anomia sp	genus	16476
741471	occ		79709	Anomia sp	genus	16476
748268	occ		80038	Anomia sir	species	87561
362462	occ		34269	Scapharca	species	101071
362463	occ		34269	Scapharca	species	85539
362464	occ		34269	Scapharca	species	85537
362480	occ		34269	Arca (Noet	species	89695
362481	occ		34269	Arca cf. ur	species	81150
362482	occ	22323	34269	Acar domiı	species	90951
423335	occ		41202	Arca (Anad	subgenus	95071
423416	occ		41209	Arca occid	species	85500
425318	occ		41497	Anadara rı	species	157125
425361	occ		41497	Barbatia ta	species	109876

425366	occ		41498	Arca wagn	species	109874
425387	occ		41501	Anadara cf	species	109482
425388	occ		41501	Anadara in	species	16201
425389	occ		41501	Anadara cf	species	16201
425390	occ		41501	Anadara lie	species	95073
425391	occ		41501	Anadara ru	species	157125
425392	occ		41501	Anadara tr	species	87549
425396	occ		41501	Arca wagn	species	109874
425398	occ		41501	Barbatia ta	species	109876
426077	occ		41539	Arca wagn	species	109874
427342	occ		41705	Anadara lie	species	95073
427343	occ		41705	Anadara m	species	16201
427344	occ		41705	Anadara sp	species	16201
427345	occ		41705	Anadara tr	species	87549
427601	occ		41715	Anadara lie	species	95073
427602	occ		41715	Anadara tr	species	87549
427681	occ		41718	Anadara cf	species	109482
427682	occ		41718	Anadara in	species	16201
427683	occ		41718	Anadara lie	species	95073
427684	occ		41718	Anadara ru	species	157125
427685	occ		41718	Anadara sc	species	109478
427686	occ		41718	Anadara sp	species	16201
427690	occ		41718	Arca wagn	species	109874
427691	occ	20480	41718	Barbatia (C	species	109878
427849	occ		41718	Anadara sp	species	16201
427902	occ		41740	Anadara tr	species	87549
427903	occ		41740	Anadara sp	species	16201
427963	occ		41749	Anadara ca	species	109482
427964	occ	22836	41749	Arca (Anad	species	120097
427965	occ		41749	Anadara lie	species	95073
427966	occ		41749	Anadara ru	species	157125
427967	occ		41749	Anadara sc	species	109478
427968	occ		41749	Anadara sp	genus	16201
427971	occ		41749	Arca aquila	species	93761
427972	occ		41749	Arca wagn	species	109874
432398	occ		41705	Anadara sp	species	16201
568567	occ		59484	Arca (Arca)	species	95725
568568	occ		59484	Barbatia (P	species	90947
568569	occ		59484	Barbatia (C	species	85504
568570	occ		59484	Barbatia (G	species	95066
568571	occ		59486	Barbatia (G	species	95066
568572	occ		59489	Barbatia (G	species	95066
568573	occ		59491	Barbatia (G	species	95066
568578	occ		59489	Arca (Anad	species	98537
568579	occ		59485	Arca (Anad	species	98537
568580	occ		59484	Arca (Anad	species	98537
568581	occ		59484	Arca (Anad	species	120097

568582	occ	59487	Arca (Anad	species	95078
568583	occ	59484	Arca (Anad	species	95078
568584	occ	59486	Arca (Anad	species	95072
568585	occ	59487	Arca (Anad	species	95072
568586	occ	59484	Arca (Anad	species	95072
568587	occ	59491	Arca (Anad	species	95072
568588	occ	59491	Arca (Anad	species	95071
568589	occ	59486	Arca (Anad	species	95071
568590	occ	59485	Arca (Anad	species	95071
568591	occ	59485	Arca (Anad	species	95071
568592	occ	59486	Arca (Anad	species	95071
568593	occ	59486	Arca (Anad	species	157130
568594	occ	59487	Arca (Anad	species	157130
568595	occ	59484	Arca (Cune	species	95090
582531	occ	61309	Anadara lie	species	95073
582532	occ	61309	Anadara tr	species	87549
582534	occ	61309	Barbatia ca	species	90947
582551	occ	61310	Anadara lie	species	95073
582552	occ	61310	Anadara tr	species	87549
582554	occ	61310	Barbatia te	species	91193
582563	occ	61311	Anadara lie	species	95073
582564	occ	61311	Anadara tr	species	87549
622881	occ	67070	Anadara ae	species	16201
622882	occ	67070	Barbatia te	species	91193
622909	occ	67069	Arca imbric	species	89693
622910	occ	67069	Arca zebra	species	90945
622911	occ	67069	Barbatia te	species	91193
622912	occ	67069	Barbatia ca	species	90947
622913	occ	67069	Barbatia do	species	90950
622915	occ	67069	Anadara ae	species	157202
622916	occ	67069	Anadara tr	species	87549
622917	occ	67069	Anadara ca	species	16201
622918	occ	67069	Anadara lie	species	95073
623057	occ	67072	Arca lienos	species	91378
623058	occ	67072	Arca (Cune	species	95090
623074	occ	67074	Navicula ur	species	61324
623075	occ	67074	Arca (Cune	species	95090
623083	occ	67075	Arca (Cune	species	95090
623092	occ	67076	Arca lienos	species	91378
623100	occ	67077	Arca (Cune	species	95090
640716	occ	69087	Anadara (R	species	160421
697888	occ	74999	Anadara sp	genus	16201
697898	occ	75009	Cunearca c	species	118865
697899	occ	75009	Anadara in	species	16201
697913	occ	75001	Anadara cf	species	87549
697923	occ	75003	Anadara tr	species	87549
697937	occ	75004	Cunearca s	genus	16227

697938	occ	75004	Anadara cf	species	16201
697968	occ	75012	Anadara in	species	16201
697969	occ	75012	Cunearca s	genus	16227
697979	occ	75013	Anadara in	species	16201
697983	occ	75014	Anadara in	species	16201
698007	occ	75016	Anadara sp	genus	16201
698032	occ	75008	Cunearca s	species	118865
738136	occ	79335	Anadara (A	species	109882
738137	occ	79335	Anadara (A	species	157202
738139	occ	79331	Arca (Arca)	species	95725
738141	occ	79331	Arca (Arca)	species	93760
738143	occ	79331	Arca (Arcop	species	109874
738147	occ	79335	Anadara (C	species	109478
738154	occ	79331	Barbatia (C	species	109878
738158	occ	79331	Anadara (A	species	109879
738159	occ	79331	Anadara (A	species	109882
738160	occ	79331	Anadara (A	species	157202
738161	occ	79331	Anadara (A	species	86518
738162	occ	79331	Anadara (A	species	109482
738163	occ	79331	Anadara (A	species	86518
738164	occ	79331	Anadara (C	species	89990
738174	occ	79336	Arca (Arcop	species	109874
738175	occ	79336	Barbatia (C	species	109877
738176	occ	79336	Barbatia (C	species	109878
738177	occ	79336	Anadara (C	species	109478
741758	occ	79731	Anadara sp	genus	16201
742039	occ	79747	Caloosarca	species	89988
742059	occ	79747	Anadara sp	genus	16201
742165	occ	79761	Arca (Arca)	species	93758
742166	occ	79762	Arca (Arca)	species	93762
742170	occ	79764	Arca (Arca)	species	93762
742494	occ	79761	Anadara cf	species	120098
742508	occ	79335	Anadara cr	species	157126
742509	occ	79335	Arca wagn	species	109874
742544	occ	35031	Anadara (A	species	167868
742545	occ	35031	Anadara (C	species	157203
743041	occ	79797	Navicula o	species	61324
743042	occ	79797	Navicula u	species	61324
743043	occ	79797	Anadara lie	species	95073
743044	occ	79797	Anadara sc	species	95091
745345	occ	79789	Anadara in	species	16201
745367	occ	79788	Anadara in	species	16201
745449	occ	69040	Anadara tr	species	59092
745744	occ	79959	Caloosarca	species	157123
747000	occ	80002	Anadara ca	species	109482
747274	occ	80027	Lunarca ov	species	91376
747296	occ	69044	Arca wagn	species	109874

747337	occ	69029	Anadara in	species	16201
747355	occ	69029	Anadara (C	species	89989
747367	occ	69029	Anadara lie	species	95073
747423	occ	79786	Arca wagn	species	109874
747440	occ	79786	Anadara lie	species	95073
747499	occ	79787	Anadara lir	species	16201
747528	occ	79786	Anadara in	species	16201
747533	occ	79786	Barbatia sp	genus	16210
747598	occ	80037	Anadara lie	species	95073
747610	occ	80037	Dallarca id	species	16232
747659	occ	80039	Dallarca al	species	16232
747699	occ	80044	Anadara ca	species	95079
747700	occ	80044	Anadara lie	species	95073
747749	occ	80046	Lunarca ov	species	91376
747758	occ	80046	Anadara lie	species	95073
747775	occ	80046	Caloosarca	species	157203
748138	occ	69034	Anadara tr	species	87549
748286	occ	80025	Cunearca r	species	16227
748289	occ	80025	Anadara lie	species	95073
748301	occ	80025	Anadara sp	genus	16201
748320	occ	80024	Arca wagn	species	109874
748349	occ	80024	Anadara in	species	16201
832775	occ	92361	Anadara (C	species	157123
832776	occ	92362	Anadara (C	species	157123
832777	occ	92363	Anadara (C	species	157123
832778	occ	92344	Anadara (C	species	157124
832783	occ	92332	Anadara (C	species	157119
832784	occ	92364	Anadara (C	species	157119
832785	occ	92331	Anadara (C	species	157119
832786	occ	92334	Anadara (C	species	157119
832787	occ	92337	Anadara (C	species	157119
832788	occ	92338	Anadara (C	species	157119
832789	occ	92366	Anadara (C	species	157119
832790	occ	92335	Anadara (C	species	157119
832791	occ	92365	Anadara (C	species	157119
832792	occ	92339	Anadara (C	species	157119
832793	occ	92336	Anadara (C	species	157119
832794	occ	92340	Anadara (C	species	157119
832795	occ	92341	Anadara (C	species	157119
832796	occ	92342	Anadara (C	species	157119
832797	occ	92333	Anadara (C	species	157119
832798	occ	92343	Anadara (C	species	157119
832799	occ	92369	Anadara (C	species	157203
832800	occ	92370	Anadara (C	species	157203
853727	occ	94809	Arca (Anad	species	98538
853728	occ	94809	Arca (Anad	species	95071
1011545	occ	123549	Arca (Anad	species	95078

1011546	occ	123549	Arca (Anad	species	98538
1011547	occ	123549	Arca (Anad	species	95072
1011548	occ	123549	Arca (Anad	species	120097
1087876	occ	96538	Anadara fl	species	116283
1087877	occ	96538	Anadara tr	species	87549
256369	occ	20604	Anadara br	species	87547
256370	occ	20604	Anadara ov	species	87545
256371	occ	20604	Anadara tr	species	87549
256387	occ	20606	Anadara br	species	87547
256388	occ	20606	Anadara ov	species	87545
256389	occ	20606	Anadara tr	species	87549
618759	occ	66724	Anadara br	species	87547
618761	occ	66725	Anadara br	species	87547
578555	occ	60923	Anadara tr	species	87549
578574	occ	60918	Anadara tr	species	87549
578585	occ	60927	Anadara ov	species	87545
578586	occ	60927	Anadara tr	species	87549
578647	occ	60928	Anadara tr	species	87549
578722	occ	60929	Anadara tr	species	87549
578749	occ	60930	Anadara tr	species	87549
578751	occ	60930	Arca umbo	species	81150
578839	occ	60931	Anadara tr	species	87549
578880	occ	60940	Anadara lie	species	95073
578935	occ	60939	Anadara in	species	16201
579012	occ	60944	Anadara tr	species	87549
619468	occ	66760	Anadara lie	species	95073
619482	occ	66760	Anadara br	species	87547
619483	occ	66758	Anadara br	species	87547
619484	occ	66757	Anadara br	species	87547
619485	occ	66755	Anadara br	species	87547
619490	occ	66760	Anadara ov	species	87545
619491	occ	66758	Anadara ov	species	87545
619492	occ	66757	Anadara ov	species	87545
619493	occ	66756	Anadara ov	species	87545
619494	occ	66755	Anadara ov	species	87545
642861	occ	69046	Anadara (R	species	160421
642875	occ	69046	Anadara ae	species	82688
741362	occ	79707	Lunarca ov	species	91376
741367	occ	79705	Lunarca ov	species	91376
741378	occ	79706	Lunarca ov	species	91376
745481	occ	79956	Lunarca ov	species	91376
745498	occ	79957	Lunarca ov	species	91376
747827	occ	80053	Lunarca ov	species	91376
748086	occ	80083	Lunarca ov	species	91376
748100	occ	80083	Anadara lie	species	95073
832779	occ	92367	Anadara (C	species	120096
832860	occ	92404	Arca sp.	genus	16203

832865	occ	92406	Arca pexat	species	157896
832924	occ	92408	Arca lienos	species	91378
832925	occ	92408	Arca aff. tr	species	87548
832998	occ	92412	Arca (Scapl	species	180894
1457465	occ	203376	Arca caelat	species	392332
564281	occ	58893	Anadara ae	species	82688
564290	occ	58894	Anadara ae	species	82688
564317	occ	58895	Anadara ae	species	82688
564355	occ	58897	Anadara ae	species	82688
564363	occ	58898	Anadara ae	species	82688
566494	occ	59327	Anadara lie	species	95073
566495	occ	59307	Anadara lie	species	95073
566496	occ	59310	Anadara lie	species	95073
566497	occ	59316	Anadara lie	species	95073
566498	occ	59315	Anadara lie	species	95073
566499	occ	59318	Anadara lie	species	95073
566504	occ	59327	Anadara ca	species	95086
566505	occ	59307	Anadara ca	species	95079
566506	occ	59307	Anadara m	species	95082
566507	occ	59307	Anadara ca	species	95086
566508	occ	59313	Anadara (C	species	95091
566509	occ	59307	Anadara (C	species	95091
578469	occ	60899	Anadara tr	species	87549
579564	occ	60986	Anadara ae	species	82688
579670	occ	60989	Anadara ae	species	82688
579735	occ	60988	Anadara ae	species	82688
579808	occ	60987	Anadara ae	species	82688
619469	occ	66754	Anadara tr	species	87549
619470	occ	66763	Anadara tr	species	87549
619471	occ	66752	Anadara tr	species	87549
619472	occ	66751	Anadara tr	species	87549
619473	occ	66750	Anadara tr	species	87549
619474	occ	66749	Anadara tr	species	87549
619475	occ	66748	Anadara tr	species	87549
619476	occ	66747	Anadara tr	species	87549
619477	occ	66746	Anadara tr	species	87549
619478	occ	66745	Anadara tr	species	87549
619479	occ	66744	Anadara tr	species	87549
619480	occ	66743	Anadara tr	species	87549
619481	occ	66742	Anadara tr	species	87549
619486	occ	66763	Anadara br	species	87547
619487	occ	66754	Anadara br	species	87547
619488	occ	66751	Anadara br	species	87547
619489	occ	66744	Anadara br	species	87547
619495	occ	66754	Anadara ov	species	87545
619496	occ	66751	Anadara ov	species	87545
619497	occ	66750	Anadara ov	species	87545

619498	occ	66749	Anadara ov	species	87545
619499	occ	66748	Anadara ov	species	87545
619500	occ	66746	Anadara ov	species	87545
619501	occ	66742	Anadara ov	species	87545
642741	occ	69045	Anadara tr	species	87549
700940	occ	69043	Lunarca ov	species	91376
701047	occ	69047	Anadara (L	species	108166
702858	occ	68979	Lunarca ov	species	91376
702891	occ	69018	Lunarca sp	genus	16264
737410	occ	79249	Lunarca sp	genus	16264
738404	occ	79342	Lunarca ov	species	91376
738434	occ	79343	Lunarca ov	species	91376
738466	occ	79341	Lunarca ov	species	91376
738538	occ	79340	Lunarca ov	species	91376
741418	occ	79708	Anadara (L	species	108166
748219	occ	80034	Anadara (A	subgenus	86518
748369	occ	80033	Anadara (R	subgenus	89995
748370	occ	80033	Anadara (R	species	160421
748371	occ	80033	Anadara ca	species	95079
748666	occ	80035	Anadara (R	subgenus	89995
748668	occ	80035	Anadara (R	species	160421
832780	occ	92368	Anadara (C	species	120096
832781	occ	80035	Anadara (C	species	120096
832782	occ	80033	Anadara (C	species	120096
1512829	occ	211029	Anadara ae	species	82688
1512838	occ	211183	Anadara ae	species	82688
1512936	occ	211181	Anadara ae	species	82688
1512974	occ	211182	Anadara ae	species	82688
94757	occ	7108	Arca transv	species	87548
94779	occ	7109	Anadara tr	species	87549
557393	occ	57704	Anadara ov	species	87545
557426	occ	57703	Anadara ov	species	87545
557479	occ	57702	Anadara tr	species	87549
559141	occ	57705	Anadara tr	species	87549
559182	occ	57706	Anadara tr	species	87549
566320	occ	59294	Arca (Barba	species	98543
566321	occ	59294	Arca idone	species	98537
640372	occ	69049	Anadara (L	species	108166
727669	occ	78271	Striarca ce	species	99232
727692	occ	78272	Striarca ce	species	99232
727695	occ	78272	Sectiarca li	species	16308
727786	occ	78269	Striarca ce	species	16314
728027	occ	78261	Striarca ce	species	99232
728066	occ	78262	Striarca ce	species	99232
728112	occ	78264	Striarca ce	species	99232
748257	occ	80038	Anadara ca	species	95079
748258	occ	80038	Striarca ce	species	16314

748427	occ	80036	Striarca ce	species	99232
749171	occ	80132	Anadara tr	species	87549
427973	occ	41749	Astarte sp.	genus	17370
568631	occ	59487	Astarte (As	species	158543
568632	occ	59486	Astarte (As	species	158543
568633	occ	59487	Astarte (As	species	98481
568634	occ	59487	Astarte aff	species	98475
640740	occ	69087	Astarte sp.	genus	17370
738193	occ	79338	Taxocardia	species	119003
738218	occ	79331	Astarte (As	species	98480
745785	occ	79959	Astarte coi	species	439681
747612	occ	80037	Astarte flo	species	158543
747642	occ	80037	Astarte gle	species	17370
747658	occ	80039	Astarte flo	species	158543
747692	occ	80044	Astarte flo	species	158543
853730	occ	94809	Astarte (As	species	98481
578752	occ	60930	Astarte sp.	genus	17370
578856	occ	60933	Astarte sp.	genus	17370
578913	occ	60940	Astarte vau	species	17370
578914	occ	60940	Astarte coi	species	439681
642872	occ	69046	Astarte coi	species	439681
748090	occ	80083	Astarte coi	species	439681
748122	occ	80083	Astarte un	species	158538
832889	occ	92407	Astarte coi	species	439681
832890	occ	92407	Astarte rac	species	17370
832930	occ	92408	Astarte coi	species	439681
833005	occ	92412	Astarte coi	species	439681
833006	occ	92412	Astarte flo	species	158543
564283	occ	58893	Astarte coi	species	439681
564291	occ	58894	Astarte bei	species	17370
564292	occ	58894	Astarte coi	species	439681
564318	occ	58895	Astarte bei	species	17370
564320	occ	58895	Astarte coi	species	439681
564358	occ	58897	Astarte bei	species	17370
564359	occ	58897	Astarte coi	species	439681
564365	occ	58898	Astarte bei	species	17370
564366	occ	58898	Astarte coi	species	439681
566334	occ	59301	Astarte un	species	158538
566582	occ	59329	Astarte syr	species	98475
566583	occ	59328	Astarte bei	species	17370
566584	occ	59309	Astarte (As	species	158540
566585	occ	59330	Astarte (As	species	158540
566586	occ	59329	Astarte (As	species	158540
566587	occ	59328	Astarte (As	species	158540
566588	occ	59331	Astarte (As	species	158540
566589	occ	59330	Astarte (As	species	439682
566590	occ	59329	Astarte (As	species	439682

566591	occ	59327	Astarte (As	species	439682
566592	occ	59307	Astarte (As	species	439682
566593	occ	59309	Astarte (As	species	439682
566594	occ	59310	Astarte (As	species	439682
566595	occ	59315	Astarte (As	species	439682
566596	occ	59316	Astarte (As	species	439682
566597	occ	59317	Astarte (As	species	439682
640555	occ	69086	Astarte cre	species	91397
659704	occ	71138	Astarte coı	species	439681
702853	occ	68979	Astarte coı	species	439681
702900	occ	69018	Astarte coı	species	439681
737386	occ	79248	Astarte coı	species	439681
737392	occ	79249	Astarte coı	species	439681
738410	occ	79342	Astarte coı	species	439681
738439	occ	79343	Astarte coı	species	439681
738461	occ	79341	Astarte coı	species	439681
738505	occ	79339	Astarte coı	species	439681
738541	occ	79340	Astarte coı	species	439681
741417	occ	79708	Astarte cre	species	91397
748374	occ	80033	Astarte coı	species	439681
748517	occ	80033	Astarte coı	species	439681
748522	occ	80033	Astarte sp.	genus	17370
748651	occ	80035	Astarte coı	species	439681
748664	occ	80035	Astarte sp.	genus	17370
559185	occ	57706	Astarte cas	species	91385
566293	occ	59297	Astarte vag	species	17370
566310	occ	59293	Astarte exa	species	17370
566311	occ	59293	Astarte un	species	158538
582213	occ	61293	Astarte coı	species	439681
582215	occ	61293	Astarte un	species	158538
664780	occ	71620	Astarte vag	species	17370
664781	occ	71620	Astarte del	species	17370
664836	occ	71624	Astarte vag	species	17370
664837	occ	71624	Astarte del	species	17370
665352	occ	60660	Astarte un	species	158538
727679	occ	78271	Astarte coı	species	439681
727699	occ	78272	Astarte coı	species	439681
727700	occ	78272	Astarte un	species	158538
727739	occ	78270	Astarte col	species	17370
727740	occ	78270	Astarte coı	species	439681
727801	occ	78269	Astarte col	species	17370
727802	occ	78269	Astarte coı	species	439681
727855	occ	78267	Astarte col	species	17370
727856	occ	78267	Astarte coı	species	439681
727920	occ	78266	Astarte coı	species	439681
727982	occ	78265	Astarte coı	species	439681
728083	occ	78262	Astarte col	species	17370

728084	occ		78262	Astarte coı	species	439681
728124	occ		78264	Astarte coı	species	439681
728125	occ		78264	Astarte col	species	17370
737459	occ		79247	Astarte sp.	genus	17370
737467	occ		79246	Astarte sp.	genus	17370
737514	occ		79245	Astarte sp.	genus	17370
741460	occ		79709	Astarte un	species	158538
741467	occ		79709	Astarte coı	species	439681
748174	occ		80040	Astarte coı	species	439681
748181	occ		80040	Astarte sp.	genus	17370
748250	occ		80038	Astarte coı	species	439681
748408	occ		80036	Astarte (As	species	439681
748409	occ		80036	Astarte sp.	genus	17370
1457463	occ		203376	Avicula atl	species	392329
362496	occ		34269	Cardium (T	species	101132
362497	occ		34269	Cardium (C	species	82716
362498	occ		34269	Cardium (T	species	94587
362499	occ		34269	Cardium (L	species	102136
362500	occ		34269	Cardium (L	species	98406
423421	occ		41209	Cardium sp	genus	17448
425336	occ		41497	Trigonioca	species	18358
425371	occ		41498	Trachycarc	genus	18347
425419	occ		41501	Dinocardiu	species	82717
425433	occ		41501	Laevicardiu	species	94599
425434	occ		41501	Laevicardiu	species	98406
425991	occ		41501	Trachycarc	species	18347
425992	occ		41501	Trachycarc	species	101259
425993	occ		41501	Trachycarc	genus	18347
425995	occ		41501	Trigonioca	species	18358
427377	occ		41705	Dinocardiu	species	82717
427388	occ		41705	Laevicardiu	species	98406
427433	occ		41705	Trachycarc	species	18347
427434	occ		41705	Trachycarc	species	101259
427435	occ		41705	Trachycarc	genus	18347
427437	occ		41705	Trigonioca	species	18358
427608	occ		41715	Laevicardiu	species	94599
427615	occ		41715	Trigonioca	species	18358
427708	occ		41718	Dinocardiu	species	82717
427715	occ		41718	Laevicardiu	species	98406
427747	occ		41718	Trachycarc	genus	18347
427907	occ		41740	Laevicardiu	species	98406
427975	occ		41749	Cardium ac	species	17448
427998	occ		41749	Laevicardiu	species	94599
428034	occ		41749	Trachycarc	species	101259
428035	occ		41749	Trachycarc	species	18347
568702	occ	22610	59485	Trachycarc	species	160965
568703	occ	22611	59486	Trachycarc	species	160965

568704	occ	59491	Cardium (T	species	85635
568705	occ	59487	Cardium (C	species	93781
568706	occ	59485	Cardium (T	species	92345
568707	occ	59484	Cardium (T	species	92345
568708	occ	59484	Cardium (L	species	102136
568709	occ	59487	Protocardi	species	18121
568710	occ	59485	Protocardi	species	18121
582617	occ	61309	Dinocardiu	species	82717
582618	occ	61309	Laevicardiu	species	94599
582619	occ	61309	Trachycarc	species	429681
582632	occ	61310	Dinocardiu	species	82717
582633	occ	61310	Laevicardiu	species	94599
582634	occ	61310	Trachycarc	species	429681
582647	occ	61311	Trachycarc	species	429681
582648	occ	61311	Dinocardiu	species	82717
622883	occ	67070	Trachycarc	species	18347
622983	occ	67069	Trachycarc	species	18347
622984	occ	67069	Trachycarc	species	18347
622985	occ	67069	Trachycarc	species	101259
622986	occ	67069	Dinocardiu	species	82717
622987	occ	67069	Americardi	species	90889
622988	occ	67069	Papyridea	species	91168
622989	occ	67069	Papyridea	species	91166
622990	occ	67069	Laevicardiu	species	94599
640530	occ	69084	Laevicardiu	species	94599
640718	occ	69087	Trachycarc	species	18347
640726	occ	69087	Dinocardiu	species	82717
640744	occ	69087	Laevicardiu	species	94599
647828	occ	70010	Laevicardiu	species	94599
647831	occ	70010	Trachycarc	species	101259
697904	occ	75009	Cardium sp	genus	17448
697929	occ	75003	Cardium sp	genus	17448
698001	occ	75015	Cardium sp	genus	17448
698072	occ	75023	Cardium (T	species	94587
698094	occ	75025	Cardium cf	species	82716
738257	occ	79338	Trigoniocar	species	248866
738259	occ	79334	Cardium (D	species	82716
738262	occ	79333	Cardium (T	species	94587
738263	occ	79333	Cardium (T	species	85635
738264	occ	79333	Trigoniocar	species	18358
738269	occ	79336	Trigoniocar	species	94688
738270	occ	79336	Cardium (D	species	82716
738271	occ	79336	Papyridea	species	91163
738284	occ	79331	Cardium (T	species	416607
738286	occ	79331	Cardium (T	species	101132
738289	occ	79335	Cardium (D	species	82716
738290	occ	79331	Trigoniocar	species	18358

738292	occ	79331	Trigoniocai	species	248866
738293	occ	79331	Papyridea .	species	91163
741763	occ	79731	Dinocardiu	species	82717
741987	occ	79748	Dinocardiu	species	17577
742169	occ	79765	Planicardiu	species	93782
742176	occ	79762	Trachycarc	species	93777
742184	occ	79761	Trachycarc	species	93777
742193	occ	79766	Trachycarc	species	93777
742550	occ	35031	Acrosterigi	species	17301
745349	occ	79789	Laevicardiu	species	94599
745377	occ	79788	Dinocardiu	species	82717
745403	occ	79788	Laevicardiu	species	94599
745414	occ	79788	Cardiidae i	family	59532
745433	occ	69040	Trachycarc	species	101259
745448	occ	69038	Laevicardiu	species	94599
745451	occ	69040	Laevicardiu	species	94599
747005	occ	80002	Laevicardiu	species	98406
747026	occ	80001	Laevicardiu	species	98406
747032	occ	80001	Trachycarc	genus	18347
747259	occ	80027	Laevicardiu	species	94599
747297	occ	69044	Trachycarc	subgenus	93777
747324	occ	69029	Americardi	species	90889
747326	occ	69029	Laevicardiu	species	94599
747391	occ	69029	Trachycarc	species	18347
747476	occ	79787	Cardiidae i	family	59532
747494	occ	79787	Dinocardiu	species	82717
747583	occ	80030	Laevicardiu	species	94599
747600	occ	80037	Chesacardi	species	99298
747652	occ	80039	Cardiidae i	family	59532
747739	occ	80046	Americardi	genus	17324
747769	occ	80046	Laevicardiu	species	94599
747779	occ	80046	Trachycarc	genus	18347
748158	occ	69034	Trachycarc	species	101259
748162	occ	69034	Laevicardiu	species	94599
748302	occ	80025	Trachycarc	genus	18347
748336	occ	80024	Trachycarc	species	18347
832803	occ	92380	Agnocardia	species	157206
1011549	occ	123549	Cardium (T	species	85635
1087918	occ	96538	Dallocardia	species	17567
1087919	occ	96538	Dinocardiu	species	82717
1087920	occ	96538	Laevicardiu	species	98406
1087921	occ	96538	Laevicardiu	species	156426
1087922	occ	96538	Trachycarc	species	429681
578660	occ	60928	Laevicardiu	species	98406
578917	occ	60940	Planicardiu	species	93778
578918	occ	60940	Dinocardiu	species	82717
579021	occ	60944	Dinocardiu	species	82717

579041	occ	60944	Trachycarc	genus	18347
619619	occ	66760	Dinocardiu	species	82717
619620	occ	66758	Dinocardiu	species	82717
619621	occ	66756	Dinocardiu	species	82717
619627	occ	66755	Trachycarc	species	101259
619628	occ	66760	Trachycarc	species	101259
619629	occ	66758	Trachycarc	species	101259
619630	occ	66758	Laevicardiu	species	94599
619633	occ	66760	Laevicardiu	species	98406
642885	occ	69046	Laevicardiu	species	94599
642886	occ	69046	Trachycarc	species	429681
642928	occ	69046	Dinocardiu	species	82717
832971	occ	92411	Cardium su	species	17448
833018	occ	92412	Cardium sp	genus	17448
1457466	occ	203376	Cardium is	species	94587
1457467	occ	203376	Laevicardiu	species	156426
1457491	occ	203378	Cardium is	species	94587
564313	occ	58894	Laevicardiu	species	17785
564404	occ	58897	Laevicardiu	species	17785
566699	occ	59330	Cerastoder	species	17455
566700	occ	59329	Cerastoder	species	17455
566701	occ	59307	Cerastoder	species	17455
566703	occ	59316	Trachycarc	species	18347
566704	occ	59313	Trachycarc	species	18347
566705	occ	59307	Trigonioca	species	243304
566706	occ	59316	Trigonioca	species	243304
566707	occ	59318	Laevicardiu	species	17785
566708	occ	59315	Laevicardiu	species	17785
566709	occ	59317	Laevicardiu	species	17785
566710	occ	59316	Laevicardiu	species	17785
566711	occ	59313	Laevicardiu	species	17785
566712	occ	59307	Laevicardiu	species	17785
619622	occ	66752	Dinocardiu	species	82717
619623	occ	66751	Dinocardiu	species	82717
619624	occ	66750	Dinocardiu	species	82717
619625	occ	66746	Dinocardiu	species	82717
619626	occ	66742	Dinocardiu	species	82717
619631	occ	66746	Laevicardiu	species	94599
619632	occ	66742	Laevicardiu	species	94599
619634	occ	66746	Laevicardiu	species	98406
619635	occ	66742	Laevicardiu	species	98406
642723	occ	69045	Dinocardiu	species	82717
701059	occ	69047	Dinocardiu	species	82717
702869	occ	68979	Chesacardi	species	99303
702908	occ	69018	Chesacardi	species	99303
738480	occ	79341	Chesacardi	species	99303
738489	occ	79339	Laevicardiu	species	94599

738520	occ	79340	Laevicardiu	species	94599
748238	occ	80034	Dinocardiu	species	82717
748375	occ	80033	Laevicardiu	species	17785
748380	occ	80033	Trachycarc	species	160941
748473	occ	80033	Dinocardiu	genus	17577
748475	occ	80033	Laevicardiu	species	17785
748530	occ	80033	Trigonioca	genus	18358
748533	occ	80033	Acrosterigi	genus	17301
748577	occ	80035	Trachycarc	species	101133
748642	occ	80035	Laevicardiu	species	17785
748654	occ	80035	Trachycarc	species	160941
829460	occ	91488	Dinocardiu	species	82717
1173576	occ	151326	Chesacardi	species	17465
559150	occ	57705	Dinocardiu	species	82717
559192	occ	57706	Dinocardiu	species	82717
559228	occ	57703	Dinocardiu	species	82717
727701	occ	78272	Chesacardi	species	99303
727731	occ	78273	Chesacardi	species	99303
727736	occ	78270	Chesacardi	species	99303
727913	occ	78266	Chesacardi	species	99303
727994	occ	78260	Chesacardi	species	99303
728128	occ	78264	Chesacardi	species	99303
737470	occ	79247	Chesacardi	species	99303
737471	occ	79246	Chesacardi	species	99303
748189	occ	80040	Chesacardi	genus	17465
748253	occ	80038	Chesacardi	genus	17465
362488	occ	34269	Cardita (Ca	species	93560
362489	occ	34269	Venericard	species	87631
362490	occ	34269	Venericard	species	93590
423386	occ	41205	Cardita (Ca	subgenus	93545
423395	occ	41206	Cardita (Ca	subgenus	93545
425401	occ	41501	Cardita ara	species	116340
425402	occ	41501	Cardita flo	species	93559
425403	occ	41501	Cardita pe	species	93589
425404	occ	41501	Cardita tric	species	87632
425405	occ	41501	Cardita sp.	genus	17441
427351	occ	41705	Cardita ara	species	116340
427352	occ	41705	Cardita flo	species	93559
427353	occ	41705	Cardita tric	species	87632
427603	occ	41715	Cardita ara	species	116340
427694	occ	41718	Cardita ara	species	116340
427695	occ	41718	Cardita flo	species	93559
427696	occ	41718	Cardita tric	species	87632
427905	occ	41740	Cardita sp.	genus	17441
427976	occ	41749	Cardita ara	species	116340
427977	occ	41749	Cardita tric	species	87632
533410	occ	20400	Carditame	species	93558

568648	occ	59487	Cardita (Ca	species	93556
568649	occ	59489	Cardita (Ca	species	93556
568650	occ	59489	Cardita (Ca	species	93555
568651	occ	59486	Cardita (Ca	species	93555
568652	occ	59491	Cardita (Ca	species	93555
568653	occ	59484	Cardita (Ca	species	93555
568654	occ	59487	Venericard	species	93582
568655	occ	59489	Venericard	species	93582
568656	occ	59486	Venericard	species	93582
568657	occ	59484	Venericard	species	93582
568658	occ	59487	Venericard	species	93590
568659	occ	59484	Venericard	species	93590
568660	occ	59487	Venericard	species	94522
568661	occ	59486	Venericard	species	94522
568662	occ	59484	Venericard	species	94522
568663	occ	59489	Venericard	species	86149
568664	occ	59487	Venericard	species	86149
582612	occ	61309	Carditamei	species	93558
582613	occ	61309	Pleuromer	species	94523
582614	occ	61309	Pteromeris	species	109774
582628	occ	61310	Carditamei	species	93558
582629	occ	61310	Pleuromer	species	94523
582630	occ	61310	Pteromeris	species	109774
582650	occ	61311	Pleuromer	species	94523
582651	occ	61311	Carditamei	species	93558
622953	occ	67069	Cardita flo	species	93559
622954	occ	67069	Venericard	species	87631
640516	occ	69085	Carditamei	species	93558
640544	occ	69084	Carditamei	species	93558
640773	occ	69087	Carditamei	species	93558
697918	occ	75001	Cardita (Ca	species	93560
697934	occ	75010	Cardita (Ca	subgenus	93545
697935	occ	75010	Venericard	subgenus	86147
697998	occ	75015	Cardita (Ca	species	93555
698009	occ	75016	Venericard	species	93590
698014	occ	75016	Cardita (Ca	subgenus	93545
698037	occ	75008	Venericard	species	93773
698050	occ	75019	Cardita (Ca	subgenus	93545
698054	occ	75020	Venericard	species	93773
698058	occ	75021	Venericard	species	93773
698063	occ	75022	Venericard	species	93773
698073	occ	75023	Venericard	species	93773
698077	occ	75024	Venericard	species	93773
698093	occ	75025	Venericard	species	93773
698100	occ	75026	Venericard	species	93773
738225	occ	79331	Cardita (Pl	species	110039
738226	occ	79331	Cardita (Pt	species	110041

738229	occ	79331	Cardita (Ca	species	93555
738230	occ	79331	Cardita (Ca	species	93561
742168	occ	79762	Cardita (Ca	species	93767
742172	occ	79761	Cardita (Ca	species	93767
742177	occ	79761	Cardita (Pl	species	93794
742183	occ	79761	Carditamer	species	93776
743056	occ	79797	Venericard	species	89177
743715	occ	79847	Carditamer	genus	17442
745342	occ	79789	Carditamer	species	93551
745380	occ	79788	Carditamer	species	93551
745441	occ	69040	Pleuromer	species	94523
745453	occ	69040	Carditamer	species	93558
745456	occ	69038	Carditamer	species	93558
745746	occ	79959	Venericard	species	89177
745801	occ	79959	Cardita ser	species	93767
745802	occ	79959	Carditamer	species	93776
745803	occ	79959	Pleuromer	species	18092
747002	occ	80002	Cardita flo	species	93559
747022	occ	80001	Cardita flo	species	93559
747256	occ	80027	Carditamer	species	93558
747286	occ	69044	Carditamer	species	93551
747302	occ	69044	Pleuromer	species	94523
747338	occ	69029	Carditamer	species	93558
747377	occ	69029	Pleuromer	species	94523
747421	occ	79786	Carditamer	species	93551
747471	occ	79787	Carditamer	species	93551
747511	occ	79787	Pleuromer	species	94523
747623	occ	80037	Carditamer	species	93551
747640	occ	80037	Cyclocardia	species	82713
747650	occ	80039	Cyclocardia	species	82713
747664	occ	80039	Carditamer	species	93551
747695	occ	80044	Cyclocardia	species	82713
748141	occ	69034	Carditamer	species	93558
748154	occ	69034	Pleuromer	species	94523
748284	occ	80025	Carditamer	species	93551
748319	occ	80024	Carditamer	species	93551
751038	occ	80357	Cardita tric	species	87632
751039	occ	80357	Cardita ara	species	116340
1087895	occ	96538	Carditamer	species	93551
1087896	occ	96538	Carditamer	species	93558
1087897	occ	96538	Pleuromer	species	94523
256378	occ	20604	Pleuromer	species	94523
256396	occ	20606	Pleuromer	species	94523
578556	occ	60923	Cardita gra	species	93579
578563	occ	60920	Cardita gra	species	93579
578575	occ	60918	Cardita gra	species	93579
578588	occ	60927	Cardita ara	species	116340

578589	occ	60927	Cardita pei	species	93589
578590	occ	60927	Cardita tric	species	87632
578649	occ	60928	Cardita gui	species	17441
578725	occ	60929	Cardita gra	species	93579
578726	occ	60929	Cardita tric	species	87632
578753	occ	60930	Cardita gra	species	93579
578841	occ	60931	Cardita gra	species	93579
578912	occ	60940	Cyclocardia	species	82713
579013	occ	60944	Cardita ara	species	116340
579014	occ	60944	Cardita tric	species	87632
619588	occ	66760	Venericard	species	87631
619589	occ	66756	Venericard	species	87631
619590	occ	66760	Cardita flo	species	93559
642883	occ	69046	Pleuromer	species	94523
745476	occ	79956	Pteromeris	species	109774
747828	occ	80053	Cyclocardia	species	82713
748087	occ	80083	Cyclocardia	species	82713
832894	occ	92407	Carditamer	species	93551
832932	occ	92408	Cardita (Ca	subgenus	93545
832933	occ	92408	Venericard	species	87631
832934	occ	92408	Venericard	species	93590
832935	occ	92408	Venericard	species	82712
832956	occ	92409	Venericard	species	93590
832957	occ	92409	Venericard	species	87631
832958	occ	92409	Venericard	species	82712
833007	occ	92412	Cardita (Ca	species	93555
833008	occ	92412	Venericard	species	82712
1457445	occ	150656	Cardita tric	species	87632
564289	occ	58893	Cyclocardia	species	82713
564298	occ	58894	Cyclocardia	species	82713
564322	occ	58895	Carditamer	species	93551
564333	occ	58895	Cyclocardia	species	82713
564361	occ	58897	Carditamer	species	93551
564367	occ	58898	Carditamer	species	93551
564372	occ	58898	Cyclocardia	species	82713
564406	occ	58897	Cyclocardia	species	82713
564603	occ	58893	Pleuromer	species	82711
564604	occ	58893	Pleuromer	species	93773
564606	occ	58893	Pteromeris	species	109774
564620	occ	58894	Pleuromer	species	82711
564621	occ	58894	Pleuromer	species	93773
564644	occ	58895	Pleuromer	species	82711
564645	occ	58895	Pleuromer	species	93773
564647	occ	58895	Pteromeris	species	109774
564894	occ	58898	Pleuromer	species	93773
564895	occ	58898	Pteromeris	species	109774
564910	occ	58897	Pleuromer	species	82711

564911	occ	58897	Pleuromer	species	93773
564912	occ	58897	Pteromeris	species	109774
566618	occ	59330	Cardita ara	species	116340
566619	occ	59316	Carditamer	species	17442
566620	occ	59317	Glans (Pleu	species	94521
566621	occ	59330	Glans (Pleu	species	94521
566622	occ	59307	Glans (Pleu	species	94521
566623	occ	59316	Glans (Pleu	species	94521
566624	occ	59327	Glans (Pleu	species	94521
566625	occ	59310	Glans (Pleu	species	94521
566626	occ	59311	Glans (Pleu	species	94521
566627	occ	59313	Glans (Pleu	species	94521
566628	occ	59318	Glans (Pleu	species	94521
566629	occ	59309	Glans (Pleu	species	94521
566630	occ	59318	Glans (Pter	species	110043
566631	occ	59327	Glans (Pter	species	110043
566632	occ	59330	Glans (Pter	species	110043
566633	occ	59307	Glans (Pter	species	110043
566634	occ	59309	Glans (Pter	species	110043
566635	occ	59313	Glans (Pter	species	110043
566636	occ	59317	Glans (Pter	species	110043
566637	occ	59315	Glans (Pter	species	110043
566638	occ	59316	Glans (Pter	species	110043
566639	occ	59310	Glans (Pter	species	110042
578470	occ	60899	Cardita tric	species	87632
619584	occ	66752	Venericard	species	87631
619585	occ	66750	Venericard	species	87631
619586	occ	66749	Venericard	species	87631
619587	occ	66742	Venericard	species	87631
619591	occ	66752	Cardita flo	species	93559
619592	occ	66749	Cardita flo	species	93559
640559	occ	69086	Cyclocardia	species	82713
640562	occ	69086	Carditamer	species	93551
642714	occ	69045	Cyclocardia	species	82713
659741	occ	71138	Cyclocardia	species	82713
700942	occ	69043	Carditamer	species	93551
702859	occ	68979	Cyclocardia	species	82713
702864	occ	68979	Pleuromer	species	94523
702875	occ	68979	Pteromeris	species	109774
702889	occ	69018	Cyclocardia	genus	17548
702907	occ	69018	Carditamer	genus	17442
702914	occ	69018	Pleuromer	species	94523
737393	occ	79248	Carditamer	species	93551
737397	occ	79249	Cyclocardia	species	82713
737407	occ	79249	Pteromeris	species	109774
737420	occ	79248	Pleuromer	species	94523
737425	occ	79248	Pteromeris	species	109774

737427	occ	79249	Carditamei	species	93551
738415	occ	79342	Cyclocardia	species	82713
738416	occ	79342	Pteromeris	species	109774
738417	occ	79342	Pleuromer	species	94523
738441	occ	79343	Pleuromer	species	94523
738444	occ	79343	Cyclocardia	species	82713
738465	occ	79341	Cyclocardia	species	82713
738468	occ	79341	Pteromeris	species	109774
738494	occ	79339	Pleuromer	species	94523
738495	occ	79339	Cyclocardia	species	82713
738536	occ	79340	Carditidae	family	59551
738537	occ	79340	Pleuromer	species	94523
741421	occ	79708	Cyclocardia	species	82713
748211	occ	80034	Cyclocardia	species	82713
748217	occ	80034	Carditamei	species	93551
748240	occ	80034	Pleuromer	species	94523
748241	occ	80034	Pteromeris	species	109774
748364	occ	80033	Carditamei	species	93551
748365	occ	80033	Pteromeris	species	109774
748366	occ	80033	Pleuromer	species	94523
748367	occ	80033	Pleuromer	genus	18092
748399	occ	80033	Carditamei	genus	17442
748658	occ	80035	Pteromeris	species	109774
1173577	occ	151326	Carditamei	species	93551
94765	occ	7108	Venericard	species	93493
559173	occ	57705	Pleuromer	species	94523
559191	occ	57706	Cyclocardia	species	102540
566292	occ	59297	Cyclocardia	species	82713
566314	occ	59293	Venericard	species	82712
582230	occ	61293	Pleuromer	genus	18092
640365	occ	69049	Pleuromer	species	94523
664779	occ	71620	Cyclocardia	species	82713
664835	occ	71624	Cyclocardia	species	82713
665351	occ	60660	Cyclocardia	species	82713
727676	occ	78271	Cyclocardia	species	82713
727703	occ	78272	Cyclocardia	species	82713
727704	occ	78272	Carditamei	species	93551
727732	occ	78273	Cyclocardia	species	82713
727769	occ	78270	Cyclocardia	species	82713
727793	occ	78269	Cyclocardia	species	82713
727839	occ	78268	Cyclocardia	species	82713
727852	occ	78267	Cyclocardia	species	82713
727911	occ	78266	Carditamei	species	93551
727972	occ	78265	Carditamei	species	93551
727973	occ	78265	Cyclocardia	species	82713
728076	occ	78262	Cyclocardia	species	82713
728130	occ	78264	Cyclocardia	species	82713

737444	occ	79246	Cyclocardi	species	82713
737461	occ	79247	Cyclocardi	species	82713
741476	occ	79709	Pteromeris	species	18162
741477	occ	79709	Cyclocardi	species	82713
748187	occ	80040	Cyclocardi	species	82713
748249	occ	80038	Cyclocardi	species	82713
748407	occ	80036	Cyclocardi	species	82713
425367	occ	41498	Chama gar	species	17459
425407	occ	41501	Chama gar	species	17459
425423	occ	41501	Echinochar	species	90898
426079	occ	41539	Chama gar	species	17459
426083	occ	41539	Echinochar	species	90898
427356	occ	41705	Chama calc	species	17459
427357	occ	41705	Chama gar	species	17459
427358	occ	41705	Chama will	species	17459
427381	occ	41705	Echinochar	species	90898
427698	occ	41718	Chama calc	species	17459
427699	occ	41718	Chama gar	species	17459
427700	occ	41718	Chama will	species	17459
427711	occ	41718	Echinochar	species	90898
427978	occ	41749	Chama gar	species	17459
427979	occ	41749	Chama hei	species	17459
427980	occ	41749	Chama will	species	17459
427994	occ	41749	Echinochar	species	90898
568665	occ	59484	Chama cor	species	90994
568666	occ	59487	Chama cor	species	90994
568667	occ	59491	Chama cor	species	90994
568669	occ	59485	Echinochar	species	91234
568670	occ	59484	Echinochar	species	91234
582578	occ	61309	Arcinella co	species	90900
582579	occ	61309	Chama cor	species	90994
582580	occ	61309	Chama ma	species	90983
582592	occ	61310	Arcinella co	species	90897
582593	occ	61310	Chama cor	species	90994
582600	occ	61311	Arcinella co	species	90897
622979	occ	67069	Chama ma	species	90983
622980	occ	67069	Chama cor	species	90994
622981	occ	67069	Pseudocha	species	90999
622982	occ	67069	Arcinella co	species	90897
738231	occ	79331	Chama will	species	17459
738232	occ	79331	Chama gar	species	17459
738233	occ	79331	Echinochar	species	91234
747334	occ	69029	Arcinella co	species	90897
747335	occ	69029	Chama cor	species	90994
747422	occ	79786	Echinochar	species	91234
747611	occ	80037	Echinochar	species	91234
747651	occ	80039	Chama cor	species	90994

747654	occ	80039	Chama spi	species	17459
747753	occ	80046	Chama sp.	genus	17459
747756	occ	80046	Echinochar	species	91234
747777	occ	80046	Chama cor	species	90994
748134	occ	69034	Arcinella c	species	90897
748153	occ	69034	Chama cor	species	90994
748307	occ	80025	Chama gar	species	17459
748330	occ	80024	Arcinella c	species	90897
853748	occ	94809	Echinochar	species	91234
1011555	occ	123549	Chama cor	species	90994
1087910	occ	96538	Arcinella c	species	90897
1087911	occ	96538	Chama cor	species	90994
1150757	occ	146873	Pseudocha	species	18144
1150758	occ	146873	Pseudocha	species	18144
578591	occ	60927	Chama gar	species	17459
578842	occ	60931	Chama gar	species	17459
578910	occ	60940	Pseudocha	species	18144
578911	occ	60940	Arcinella c	species	90897
578947	occ	60941	Arcinella c	species	90897
579017	occ	60944	Chama gar	species	17459
619583	occ	66760	Echinochar	species	91234
642864	occ	69046	Pseudocha	genus	18144
745463	occ	79956	Chama rad	species	90998
748120	occ	80083	Pseudocha	species	18144
832897	occ	92407	Chama cor	species	90994
833009	occ	92412	Chama cor	species	17459
833010	occ	92412	Echinochar	species	91234
1457444	occ	150656	Chama arc	species	91233
564376	occ	58897	Chama gar	species	17459
566336	occ	59301	Chama cor	species	90994
566696	occ	59330	Pseudocha	species	18144
566697	occ	59307	Pseudocha	species	18144
566698	occ	59309	Pseudocha	species	18144
578472	occ	60899	Chama gar	species	17459
619582	occ	66749	Chama ma	species	90983
640561	occ	69086	Chama sp.	genus	17459
737403	occ	79249	Pseudocha	species	18144
738499	occ	79339	Pseudocha	species	18144
748232	occ	80034	Arcinella c	species	90897
748376	occ	80033	Pseudocha	species	18144
748446	occ	80033	Arcinella c	species	90897
748639	occ	80035	Arcinella c	species	90897
748647	occ	80035	Pseudocha	species	18144
576308	occ	60660	Chama cor	species	90994
664792	occ	71621	Chama cor	species	90994
727702	occ	78272	Chama cor	species	90994
727770	occ	78270	Chama cor	species	90994

727771	occ	78270	Pseudocha	species	18144
727795	occ	78269	Chama cor	species	90994
727974	occ	78265	Chama cor	species	90994
737452	occ	79247	Chama cor	species	90994
737465	occ	79246	Chama cor	species	90994
741459	occ	79709	Pseudocha	species	18144
748203	occ	80040	Pseudocha	species	18144
748255	occ	80038	Pseudocha	species	18144
748415	occ	80036	Pseudocha	species	18144
564339	occ	58895	Erycinella (	species	142142
727859	occ	78267	Erycinella (	species	142142
578757	occ	60930	Corbicula c	species	17504
619559	occ	66760	Polymesoc	species	156517
642874	occ	69046	"Corbicula'	species	17504
566614	occ	59309	Corbicula c	species	17504
566615	occ	59316	Corbicula c	species	17504
566616	occ	59317	Corbicula c	species	17504
566617	occ	59318	Corbicula c	species	17504
619560	occ	66742	Polymesoc	species	156517
362524	occ	34269	Corbula cu	species	158601
425320	occ	41497	Caryocorbı	species	18437
425337	occ	41497	Varicorbul	species	18517
425372	occ	41498	Varicorbul	species	18517
425399	occ	41501	Bothrocorl	species	121465
425406	occ	41501	Caryocorbı	species	18437
425996	occ	41501	Varicorbul	species	18517
426092	occ	41539	Varicorbul	species	18517
427354	occ	41705	Caryocorbı	species	109798
427355	occ	41705	Caryocorbı	species	18437
427387	occ	41705	Juliacorbul	species	121472
427438	occ	41705	Varicorbul	species	18517
427692	occ	41718	Bothrocorl	species	121465
427697	occ	41718	Caryocorbı	species	18437
427749	occ	41718	Varicorbul	species	18517
427974	occ	41749	Bothrocorl	species	121465
428038	occ	41749	Varicorbul	species	18517
568782	occ	59487	Corbula (C	species	162185
568783	occ	59484	Corbula (C	species	162185
568784	occ	59485	Corbula (C	species	162185
568785	occ	59487	Corbula (C	species	82729
568786	occ	59484	Corbula (C	species	82729
568787	occ	59485	Corbula (C	species	82729
568788	occ	59486	Corbula (C	species	82729
568789	occ	59487	Corbula (C	species	97922
568790	occ	59487	Corbula (C	species	109797
568791	occ	59484	Corbula (C	species	109797
568792	occ	59487	Corbula (C	species	158601

582643	occ	61311	Corbula co	species	82732
582682	occ	61309	Corbula co	species	82732
582706	occ	61310	Corbula co	species	82732
582707	occ	61310	Juliacorbul	species	121472
622885	occ	67070	Corbula sc	species	121471
623047	occ	67069	Corbula sc	species	121471
623048	occ	67069	Corbula ca	species	101163
623049	occ	67069	Corbula sw	species	109344
623050	occ	67069	Corbula kr	species	146240
623051	occ	67069	Corbula sp	genus	18441
623052	occ	67069	Notocorbu	species	18477
640511	occ	69085	Caryocorbu	species	82733
647840	occ	70010	Caryocorbu	species	82733
697895	occ	74999	Corbula sp	genus	18441
697915	occ	75001	Corbula sp	genus	18441
738362	occ	79331	Caryocorbu	species	101162
738363	occ	79331	Bothrocorb	species	121465
738364	occ	79331	Varicorbula	species	18517
738365	occ	79331	Juliacorbul	species	121472
745352	occ	79789	Caryocorbu	species	18437
747359	occ	69029	Caryocorbu	species	18437
747383	occ	69029	Bothrocorb	species	121465
747521	occ	79786	Bothrocorb	species	121465
747530	occ	79786	Caryocorbu	species	18437
747531	occ	79786	Notocorbu	genus	18477
747566	occ	80030	Corbula sp	genus	18441
747568	occ	80030	Caryocorbu	species	118937
747576	occ	80030	Caryocorbu	species	82733
747588	occ	80029	Corbula sp	genus	18441
747590	occ	80029	Caryocorbu	species	118937
747624	occ	80037	Corbula cf.	species	121458
747625	occ	80037	Bothrocorb	genus	18433
747626	occ	80037	Caryocorbu	species	118937
747670	occ	80039	Caryocorbu	species	118937
747672	occ	80039	Corbula cf.	species	121458
747673	occ	80039	Caryocorbu	species	158603
747714	occ	80044	Caryocorbu	species	118937
747715	occ	80044	Bothrocorb	genus	18433
747784	occ	80046	Bothrocorb	genus	18433
747815	occ	80046	Caryocorbu	genus	18437
748161	occ	69034	Caryocorbu	species	82733
750998	occ	80359	Caryocorbu	species	18437
751028	occ	80357	Varicorbula	species	18517
751037	occ	80357	Caryocorbu	species	18437
853739	occ	94809	Corbula (Ca	species	109797
853740	occ	94809	Corbula (Ca	species	82729
853741	occ	94809	Corbula (C	species	162185

1066832	occ	35025	Corbula (B	species	121451
1066837	occ	35031	Corbula (B	species	121451
1088919	occ	96538	Caryocorbu	species	101255
1088920	occ	96538	Caryocorbu	species	82733
578650	occ	60928	Caryocorbu	species	82733
578754	occ	60930	Caryocorbu	species	109798
579015	occ	60944	Caryocorbu	species	109798
579016	occ	60944	Caryocorbu	species	82733
642940	occ	69046	Caryocorbu	species	82733
745462	occ	79956	Caryocorbu	species	82733
745490	occ	79957	Caryocorbu	species	82733
747845	occ	80053	Caryocorbu	genus	18437
832946	occ	92408	Corbula ina	species	97922
832964	occ	92409	Corbula ina	species	97922
833025	occ	92412	Corbula ina	species	97922
833026	occ	92412	Corbula cf.	species	121458
1457478	occ	203376	Corbula co	species	82732
564284	occ	58893	Caryocorbu	species	18437
564293	occ	58894	Caryocorbu	species	18437
564324	occ	58895	Caryocorbu	species	18437
564325	occ	58895	Caryocorbu	species	82733
564369	occ	58898	Caryocorbu	species	18437
564373	occ	58897	Caryocorbu	species	18437
564374	occ	58897	Caryocorbu	species	82733
566922	occ	59330	Corbula (Ca	species	82730
566923	occ	59329	Corbula (Ca	species	82730
566924	occ	59328	Corbula (Ca	species	82730
566925	occ	59307	Corbula (Ca	species	82730
566926	occ	59316	Corbula (Ca	species	121471
578471	occ	60899	Caryocorbu	species	109798
579686	occ	60989	Corbula co	species	82730
579767	occ	60988	Corbula co	species	82730
579819	occ	60987	Corbula co	species	82730
619773	occ	66749	Corbula co	species	82732
619774	occ	66750	Corbula co	species	82732
619775	occ	66742	Corbula co	species	82732
640557	occ	69086	Caryocorbu	genus	18437
642713	occ	69045	Caryocorbu	species	82733
659701	occ	71138	Caryocorbu	species	82733
700973	occ	69043	Caryocorbu	species	82733
702878	occ	68979	Caryocorbu	species	82731
702912	occ	69018	Caryocorbu	species	82731
737424	occ	79248	Caryocorbu	species	82731
738448	occ	79343	Caryocorbu	species	82731
738458	occ	79341	Caryocorbu	species	82731
738510	occ	79339	Caryocorbu	species	82733
738530	occ	79340	Caryocorbu	species	82733

741420	occ	79708	Caryocorbı	genus	18437
748213	occ	80034	Caryocorbı	species	82731
748400	occ	80033	Caryocorbı	species	82731
748499	occ	80033	Caryocorbı	species	82731
94776	occ	7109	Corbula co	species	82732
559146	occ	57705	Corbula co	species	82732
559188	occ	57706	Corbula co	species	82732
559207	occ	57702	Corbula co	species	82732
559224	occ	57703	Corbula co	species	82732
559239	occ	57704	Corbula co	species	82732
559240	occ	57704	Corbula sw	species	109344
640357	occ	69048	Caryocorbı	species	82733
727763	occ	78270	Corbula ina	species	97922
727964	occ	78265	Corbula ina	species	97922
728068	occ	78262	Corbula ina	species	97922
737466	occ	79247	Caryocorbı	species	118937
737473	occ	79246	Corbula ina	species	97922
737494	occ	79245	Corbula ina	species	97922
741448	occ	79709	Caryocorbı	species	82731
748183	occ	80040	Caryocorbı	species	82731
748267	occ	80038	Caryocorbı	species	82731
362486	occ	34269	Crassinella	species	108664
362487	occ	34269	Crassinella	genus	17523
425323	occ	41497	Crassinella	species	17523
425416	occ	41501	Crassinella	species	17523
425417	occ	41501	Crassinella	species	108664
427371	occ	41705	Crassinella	species	17523
427372	occ	41705	Crassinella	species	17523
427373	occ	41705	Crassinella	species	108664
568635	occ	59487	Crassatellit	species	99289
568636	occ	59489	Crassatellit	species	99289
568637	occ	59488	Crassatellit	species	99289
568638	occ	59484	Crassatellit	species	110035
568639	occ	59487	Crassatellit	species	110035
568640	occ	59485	Crassatellit	species	110035
568641	occ	59486	Crassinella	species	108664
568642	occ	59487	Crassinella	species	108664
568643	occ	59484	Crassinella	species	108664
568644	occ	59485	Crassinella	species	17523
568645	occ	59484	Crassinella	species	17523
568646	occ	59485	Crassinella	species	17523
568647	occ	59484	Crassinella	species	17523
582615	occ	61309	Crassinella	species	108664
582631	occ	61310	Crassinella	species	108664
582649	occ	61311	Crassinella	species	108664
622951	occ	67069	Crassinella	species	108664
622952	occ	67069	Crassinella	genus	17523

697986	occ	75014	Eucrassate	genus	17640
698019	occ	75006	Crassinella	genus	17523
738194	occ	79338	Eucrassate	species	110036
738219	occ	79331	Eucrassate	species	110036
738220	occ	79331	Crassinella	species	108664
738222	occ	79331	Crassinella	species	17523
738227	occ	79334	Eucrassate	species	110036
745411	occ	79788	Crassinella	species	108664
745631	occ	79960	Marvacras	species	17876
747621	occ	80037	Marvacras	species	17876
747647	occ	80039	Marvacras	species	17876
747660	occ	80039	Crassinella	species	17523
747704	occ	80044	Marvacras	species	17876
747813	occ	80046	Crassinella	species	108664
853731	occ	94809	Crassatelli	species	94753
1087892	occ	96538	Crassinella	species	17523
1087893	occ	96538	Crassinella	species	108664
578559	occ	60923	Crassinella	species	108664
578596	occ	60927	Crassinella	species	108664
578652	occ	60928	Crassinella	species	108664
578727	occ	60929	Crassinella	species	108664
578758	occ	60930	Crassinella	species	17523
578759	occ	60930	Crassinella	species	108664
578916	occ	60940	Eucrassate	species	17640
578942	occ	60939	Eucrassate	species	17640
579018	occ	60944	Crassinella	species	17523
579019	occ	60944	Crassinella	species	108664
619593	occ	66760	Crassinella	species	108664
619594	occ	66758	Crassinella	species	108664
619595	occ	66756	Crassinella	species	108664
642873	occ	69046	Crassinella	genus	17523
642882	occ	69046	Crassinella	species	108664
642937	occ	69046	Marvacras	genus	17876
745499	occ	79957	Crassinella	species	108664
747839	occ	80053	Crassinella	species	108664
747840	occ	80053	Marvacras	genus	17876
748110	occ	80083	Crassinella	species	108664
832931	occ	92408	Crassinella	species	108664
832955	occ	92409	Crassinella	species	108664
833003	occ	92412	Crassatelli	species	110035
564286	occ	58893	Crassinella	species	17523
564287	occ	58893	Crassinella	species	108664
564296	occ	58894	Crassinella	species	17523
564297	occ	58894	Crassinella	species	108664
564328	occ	58895	Crassinella	species	17523
564329	occ	58895	Crassinella	species	82715
564330	occ	58895	Crassinella	species	108664

564371	occ	58898	Crassinella	species	108664
564377	occ	58897	Crassinella	species	17523
564378	occ	58897	Crassinella	species	82715
564379	occ	58897	Crassinella	species	108664
564390	occ	58898	Marvacras	species	82714
564401	occ	58897	Marvacras	species	82714
566335	occ	59301	Marvacras	species	17876
566598	occ	59328	Crassinella	species	108664
566599	occ	59329	Crassinella	species	108664
566600	occ	59327	Crassinella	species	108664
566601	occ	59330	Crassinella	species	108664
566602	occ	59310	Crassinella	species	108664
566603	occ	59309	Crassinella	species	108664
566604	occ	59312	Crassinella	species	108664
566605	occ	59313	Crassinella	species	108664
566606	occ	59315	Crassinella	species	108664
566607	occ	59316	Crassinella	species	108664
566608	occ	59318	Crassinella	species	108664
566609	occ	59307	Crassinella	species	108664
566610	occ	59312	Crassinella	species	17523
566611	occ	59309	Crassinella	species	17523
566612	occ	59307	Crassinella	species	17523
566613	occ	59316	Crassinella	species	17523
619596	occ	66752	Crassinella	species	108664
619597	occ	66749	Crassinella	species	108664
619598	occ	66742	Crassinella	species	108664
702880	occ	68979	Crassinella	species	108664
738422	occ	79342	Crassinella	species	108664
738445	occ	79343	Marvacras	genus	17876
738464	occ	79341	Marvacras	genus	17876
738516	occ	79339	Crassinella	species	108664
738531	occ	79340	Eucrassate	genus	17640
748235	occ	80034	Marvacras	genus	17876
748488	occ	80033	Crassatella	genus	17518
748644	occ	80035	Crassatella	genus	17518
559147	occ	57705	Crassinella	species	108664
559189	occ	57706	Crassinella	species	108664
559208	occ	57702	Crassinella	species	108664
559225	occ	57703	Crassinella	species	108664
566294	occ	59297	Marvacras	species	17876
582217	occ	61293	Crassinella	species	108664
582218	occ	61293	Eucrassate	genus	17640
664782	occ	71620	Marvacras	species	17876
664838	occ	71624	Marvacras	species	17876
665353	occ	60660	Marvacras	species	17876
727680	occ	78271	Marvacras	species	17876
727681	occ	78271	Crassinella	species	108664

727698	occ	78272	Marvacras	species	17876
727730	occ	78273	Marvacras	species	17876
727741	occ	78270	Marvacras	species	17876
727803	occ	78269	Crassinella	species	108664
727857	occ	78267	Marvacras	species	17876
727858	occ	78267	Crassinella	species	108664
727981	occ	78265	Crassinella	species	108664
728085	occ	78262	Marvacras	species	17876
737447	occ	79246	Marvacras	species	17876
737451	occ	79247	Marvacras	species	17876
737462	occ	79246	Marvacras	species	17876
741450	occ	79709	Marvacras	genus	17876
748173	occ	80040	Marvacras	genus	17876
748280	occ	80038	Marvacras	genus	17876
748403	occ	80036	Marvacras	genus	17876
622926	occ	67069	Musculus l	species	16383
622927	occ	67069	Gregariella	species	16359
1087883	occ	96538	Musculus l	species	16383
564397	occ	58897	Musculus l	species	16383
559197	occ	57706	Musculus l	species	16383
728037	occ	78261	Musculus \	species	16383
568630	occ	59486	Cuspidaria	species	134770
632422	occ	67069	Cuspidaria	species	108203
632423	occ	67069	Cardiomya	species	134772
632424	occ	67069	Cardiomya	species	134773
632425	occ	67069	Cardiomya	species	134770
738206	occ	79336	Cuspidaria	species	152212
738214	occ	79331	Cuspidaria	species	162234
738215	occ	79336	Cuspidaria	species	108202
748139	occ	69034	Cyrenoida	species	382142
362518	occ	34269	Donax vari	species	109660
362519	occ	34269	Donax tum	species	17592
698091	occ	75025	Donax foss	species	108682
738351	occ	79331	Donax foss	species	108682
741764	occ	79731	Donax vari	species	109660
256374	occ	20604	Donax vari	species	109660
256392	occ	20606	Donax vari	species	109660
256432	occ	20613	Donax vari	species	109660
578560	occ	60923	Donax vari	species	109660
578654	occ	60928	Donax vari	species	109660
578655	occ	60928	Donax sp.	genus	17592
578729	occ	60929	Donax vari	species	109660
578761	occ	60930	Donax vari	species	109660
619780	occ	66760	Donax foss	species	108682
619782	occ	66758	Donax foss	species	108682
619783	occ	66755	Donax foss	species	108682
642934	occ	69046	Donax foss	species	108682

745483	occ	79956	Donax vari	species	109660
745492	occ	79957	Donax vari	species	109660
832869	occ	92406	Donax vari	species	109660
832888	occ	92407	Donax vari	species	109660
832944	occ	92408	Donax cf. f	species	108682
1457473	occ	203376	Serrula var	species	392354
564299	occ	58894	Donax foss	species	108682
564336	occ	58895	Donax foss	species	108682
564408	occ	58897	Donax foss	species	108682
566754	occ	59316	Donax emr	species	17592
566755	occ	59313	Donax emr	species	17592
566756	occ	59312	Donax emr	species	17592
566757	occ	59307	Donax emr	species	17592
566758	occ	59310	Donax emr	species	17592
566759	occ	59309	Donax emr	species	17592
566760	occ	59315	Donax emr	species	17592
566761	occ	59330	Donax emr	species	17592
566762	occ	59316	Donax aeq	species	17592
566763	occ	59307	Donax foss	species	108682
566764	occ	59312	Donax foss	species	108682
566765	occ	59310	Donax foss	species	108682
566766	occ	59313	Donax foss	species	108682
566767	occ	59316	Donax foss	species	108682
566768	occ	59317	Donax foss	species	108682
619776	occ	66754	Donax vari	species	109660
619777	occ	66750	Donax vari	species	109660
619778	occ	66749	Donax vari	species	109660
619779	occ	66742	Donax vari	species	109660
619781	occ	66742	Donax foss	species	108682
642712	occ	69045	"Donax" cf	species	108682
748527	occ	80033	Donax sp.	genus	17592
559153	occ	57705	Donax parv	species	17592
559154	occ	57705	Donax roer	species	156638
728079	occ	78262	Bornia tria	species	399206
738211	occ	79331	Mytilopsis	species	110032
747437	occ	79786	Mytilopsis	genus	71502
1088917	occ	96538	Mytilopsis	species	155519
619551	occ	66742	Congeria le	species	97797
622974	occ	67069	Erycina sp.	genus	17634
738275	occ	79331	Erycina pha	species	17634
747475	occ	79787	Erycina pha	species	17634
578657	occ	60928	Erycina car	species	17634
566661	occ	59317	Erycina car	species	17634
566662	occ	59307	Erycina car	species	17634
566663	occ	59316	Erycina car	species	17634
659731	occ	71138	Erycina car	species	17634
423399	occ	41206	Gastrochae	genus	18456

425458	occ		41501	Rocellaria l	species	116480
568794	occ		59484	Gastrochae	species	18456
582708	occ		61310	Gastrochae	species	156310
632412	occ		67069	Gastrochae	species	156310
632413	occ		67069	Spengleria	species	416610
738329	occ		79336	Gastrochae	species	158754
738369	occ	22286	79331	Gastrochae	species	156310
1087915	occ		96538	Lamychaer	species	251737
578929	occ		60940	Gastrochae	species	18456
619793	occ		66760	Gastrochae	species	158754
833020	occ		92412	Isocardia fr	species	98395
566327	occ		59300	Glossus fra	species	99325
566295	occ		59297	Glossus fra	species	99325
664783	occ		71620	Glossus fra	species	99325
664839	occ		71624	Glossus fra	species	99325
748171	occ		80040	Glossus fra	species	99325
748277	occ		80038	Glossus fra	species	99325
748438	occ		80036	Isocardia fr	species	98395
362460	occ		34269	Glycymeris	species	90960
362461	occ		34269	Glycymeris	species	109129
423369	occ		41205	Glycymeris	species	90960
423370	occ		41205	Glycymeris	species	109129
427384	occ		41705	Glycymeris	species	90960
427385	occ		41705	Glycymeris	species	109872
427714	occ		41718	Glycymeris	species	109872
427996	occ		41749	Glycymeris	species	90960
568560	occ		59484	Glycymeris	species	90960
568561	occ		59491	Glycymeris	species	90960
568563	occ		59487	Glycymeris	species	109129
568564	occ		59484	Glycymeris	species	109129
568565	occ		59487	Glycymeris	species	90960
568566	occ		59487	Glycymeris	species	98550
582567	occ		61309	Glycymeris	species	90960
582581	occ		61310	Glycymeris	species	109129
582594	occ		61311	Glycymeris	species	109129
622920	occ		67069	Glycymeris	species	109872
622921	occ		67069	Glycymeris	species	109129
647827	occ		70010	Tucetona p	species	90964
698042	occ		75018	Glycymeris	species	98550
698060	occ		75022	Glycymeris	genus	16243
698066	occ		75023	Glycymeris	species	98550
738132	occ		79335	Glycymeris	species	90962
738138	occ		79331	Glycymeris	species	109872
738149	occ		79338	Glycymeris	species	109872
741765	occ		79731	Glycymeris	species	119869
742547	occ		35031	Glycymeris	species	90960
745425	occ		69040	Tucetona a	species	135317

745760	occ	79959	Costaglycy	species	99238
747287	occ	69044	Tucetona a	species	135317
747348	occ	69029	Tucetona s	genus	16324
747619	occ	80037	Costaglycy	species	99238
747620	occ	80037	Glycymeris	species	90960
747638	occ	80037	Tucetona s	genus	16324
747648	occ	80039	Glycymeris	species	90960
747649	occ	80039	Costaglycy	species	99238
747709	occ	80044	Glycymeris	species	90960
747716	occ	80044	Tucetona s	genus	16324
747783	occ	80046	Tucetona s	genus	16324
748129	occ	69034	Tucetona p	species	90964
748309	occ	80025	Tucetona a	species	135317
748326	occ	80024	Tucetona a	species	135317
751031	occ	80357	Glycymeris	species	109872
751048	occ	80355	Glycymeris	species	109872
1011570	occ	123549	Glycymeris	species	90960
1087881	occ	96538	Tucetona p	species	90964
578578	occ	60918	Glycymeris	species	90960
578599	occ	60927	Glycymeris	species	109129
578659	occ	60928	Glycymeris	species	90960
578732	occ	60929	Glycymeris	species	90960
578765	occ	60930	Glycymeris	species	90960
578846	occ	60931	Glycymeris	species	90960
578872	occ	60942	Glycymeris	species	16243
578883	occ	60940	Glycymeris	species	90960
578884	occ	60940	Glycymeris	species	98550
579027	occ	60944	Glycymeris	species	109129
619514	occ	66760	Glycymeris	species	90960
619515	occ	66760	Glycymeris	species	109129
619516	occ	66758	Glycymeris	species	109129
619517	occ	66757	Glycymeris	species	109129
642866	occ	69046	Glycymeris	species	90960
642867	occ	69046	Glycymeris	species	109872
745464	occ	79956	Glycymeris	species	90960
745473	occ	79956	Tucetona p	species	90964
747824	occ	80053	Tucetona a	species	135317
747825	occ	80053	Glycymeris	species	90960
748083	occ	80083	Tucetona a	species	135317
748093	occ	80083	Glycymeris	species	90960
832861	occ	92404	Pectunculu	genus	18044
832895	occ	92407	Pectunculu	species	18044
832896	occ	92407	Pectunculu	species	18044
832921	occ	92408	Glycymeris	species	109128
832922	occ	92408	Glycymeris	species	16243
832923	occ	92408	Glycymeris	species	119869
832950	occ	92409	Glycymeris	species	90960

832951	occ	92409	Glycymeris	species	109128
832997	occ	92412	Glycymeris	species	98550
564302	occ	58893	Glycymeris	species	90960
564303	occ	58893	Glycymeris	species	82692
564310	occ	58894	Glycymeris	species	90960
564311	occ	58894	Glycymeris	species	82692
564340	occ	58895	Glycymeris	species	90960
564341	occ	58895	Glycymeris	species	109872
564342	occ	58895	Glycymeris	species	82691
564343	occ	58895	Glycymeris	species	82692
564385	occ	58898	Glycymeris	species	90960
564386	occ	58898	Glycymeris	species	82692
564402	occ	58897	Glycymeris	species	82692
564412	occ	58897	Glycymeris	species	109872
566325	occ	59300	Costaglycy	species	99238
566330	occ	59301	Costaglycy	species	99238
566515	occ	59328	Glycymeris	species	16243
566516	occ	59329	Glycymeris	species	16243
566517	occ	59330	Glycymeris	species	16243
566518	occ	59313	Glycymeris	species	16243
566519	occ	59307	Glycymeris	species	16243
566521	occ	59318	Glycymeris	species	90960
566522	occ	59316	Glycymeris	species	90960
566523	occ	59315	Glycymeris	species	90960
566524	occ	59313	Glycymeris	species	90960
566525	occ	59317	Glycymeris	species	90960
566526	occ	59311	Glycymeris	species	90960
566527	occ	59309	Glycymeris	species	90960
566528	occ	59307	Glycymeris	species	90960
566529	occ	59330	Glycymeris	species	90960
578474	occ	60899	Glycymeris	species	90960
640556	occ	69086	Glycymeris	species	90960
659692	occ	71138	Glycymeris	species	98550
659699	occ	71138	Glycymeris	species	90960
700957	occ	69043	Glycymeris	species	90960
702856	occ	68979	Glycymeris	species	90960
702884	occ	69018	Tucetona s	genus	16324
702885	occ	69018	Glycymeris	species	90960
737389	occ	79248	Glycymeris	species	16243
737399	occ	79249	Glycymeris	species	16243
738405	occ	79342	Glycymeris	species	90960
738435	occ	79343	Glycymeris	species	90960
738467	occ	79341	Glycymeris	species	90960
738488	occ	79339	Glycymeris	species	90960
738490	occ	79339	Tucetona s	genus	16324
738519	occ	79340	Glycymeris	species	90960
738522	occ	79340	Tucetona s	genus	16324

741422	occ	79708	Glycymeris	species	90960
748233	occ	80034	Glycymeris	species	90960
748397	occ	80033	Glycymeris	species	16243
748442	occ	80033	Tucetona a	species	135317
748462	occ	80033	Glycymeris	species	90960
748465	occ	80033	Glycymeris	genus	16243
748466	occ	80033	Glycymere	species	16242
748578	occ	80035	Glycymeris	species	90960
748641	occ	80035	Glycymere	species	16242
1173582	occ	151326	Costaglycy	species	99238
1173583	occ	151326	Glycymeris	species	90960
566286	occ	59297	Costaglycy	species	99238
566306	occ	59293	Glycymeris	species	98550
566319	occ	59294	Glycymeris	species	16243
582219	occ	61293	Glycymeris	species	109129
664774	occ	71620	Costaglycy	species	99238
664832	occ	71624	Costaglycy	species	99238
665349	occ	60660	Costaglycy	species	99238
727727	occ	78273	Costaglycy	species	99238
727785	occ	78269	Costaglycy	species	99238
727870	occ	78267	Glycymeris	species	90960
727871	occ	78267	Costaglycy	species	99238
727899	occ	78266	Costaglycy	species	99238
727961	occ	78265	Costaglycy	species	99238
728111	occ	78264	Costaglycy	species	99238
737464	occ	79247	Costaglycy	species	99238
741452	occ	79709	Costaglycy	species	99238
748197	occ	80040	Costaglycy	species	99238
748404	occ	80036	Glycymeris	species	90960
423302	occ	41199	Ostrea tam	species	160459
423380	occ	41205	Ostrea tam	species	160459
423392	occ	41206	Ostrea n. s	species	160459
423417	occ	41209	Ostrea tam	species	160459
428012	occ	41749	Ostrea tam	species	160459
568602	occ	59487	Ostrea hail	species	99269
568603	occ	59484	Ostrea hail	species	99269
568604	occ	59486	Ostrea hail	species	99269
568668	occ	59484	Chama stri	species	352871
738135	occ	79334	Ostrea (Ale	species	160459
742432	occ	35026	Pycnodont	species	120102
742495	occ	79761	Pycnodont	species	120102
744390	occ	79874	Hyotissa m	species	16717
747288	occ	69044	Hyotissa m	species	16717
747616	occ	80037	Chama stri	species	352871
747697	occ	80044	Chama stri	species	352871
748293	occ	80025	Chama stri	species	352871
789715	occ	85448	Ostrea hail	species	99269

834786	occ	92739	Ostrea hai	species	99269
834790	occ	92740	Ostrea hai	species	99269
578902	occ	60940	Hyotissa h	species	99270
579037	occ	60944	Pycnodont	genus	62025
566687	occ	59330	Chama stri	species	352871
566688	occ	59327	Chama stri	species	352871
566689	occ	59308	Chama stri	species	352871
566690	occ	59307	Chama stri	species	352871
566691	occ	59309	Chama stri	species	352871
566692	occ	59310	Chama stri	species	352871
566693	occ	59316	Chama stri	species	352871
566694	occ	59317	Chama stri	species	352871
566695	occ	59318	Chama stri	species	352871
737408	occ	79249	Chama stri	species	352871
737419	occ	79248	Chama stri	species	352871
738498	occ	79339	Chama stri	species	352871
738528	occ	79340	Chama stri	species	352871
748377	occ	80033	Chama stri	species	352871
748532	occ	80033	Chama stri	species	352871
748648	occ	80035	Chama stri	species	352871
741454	occ	79709	Chama stri	species	352871
748198	occ	80040	Chama stri	species	352871
748254	occ	80038	Chama stri	species	352871
748414	occ	80036	Chama stri	species	352871
427726	occ	41718	Panope flo	species	61583
568793	occ	59484	Panope ref	species	61583
582683	occ	61309	Panopea b	species	157436
582711	occ	61311	Panopea b	species	157436
632415	occ	67069	Hiatella arc	species	107596
632416	occ	67069	Panopea fl	species	18482
738322	occ	79334	Panopea fl	species	18482
738367	occ	79331	Hiatella arc	species	107596
738368	occ	79331	Panopea fl	species	18482
743723	occ	79804	Panopea sp	genus	18482
853738	occ	94809	Panope go	species	61583
1087914	occ	96538	Panopea b	species	157436
1317135	occ	176823	Panopea re	species	18482
578513	occ	60920	Panopea sp	genus	18482
578931	occ	60940	Panopea re	species	18482
833027	occ	92412	Saxicava sp	genus	82726
566338	occ	59301	Panopea re	species	18482
579687	occ	60989	Hiatella arc	species	107596
579768	occ	60988	Hiatella arc	species	107596
579820	occ	60987	Hiatella arc	species	107596
619791	occ	66747	Hiatella arc	species	107596
619792	occ	66742	Hiatella arc	species	107596
737434	occ	79248	Panopea re	species	18482

748352	occ	80033	Panopea s|	genus	18482
748579	occ	80035	Panopea s|	genus	18482
566297	occ	59297	Panopea re	species	18482
664785	occ	71620	Panopea re	species	18482
664841	occ	71624	Panopea re	species	18482
665356	occ	60660	Panopea re	species	18482
727671	occ	78271	Hiatella arc	species	107596
727694	occ	78272	Panopea re	species	18482
727734	occ	78273	Panopea re	species	18482
727876	occ	78267	Panopea s|	genus	18482
727902	occ	78266	Hiatella arc	species	107596
727965	occ	78265	Hiatella arc	species	107596
728030	occ	78261	Hiatella arc	species	107596
728069	occ	78262	Hiatella arc	species	107596
728117	occ	78264	Hiatella arc	species	107596
737472	occ	79246	Hiatella arc	species	107596
737497	occ	79245	Panopea re	species	18482
741444	occ	79709	Panopea re	species	18482
748204	occ	80040	Panopea g	species	18482
748402	occ	80036	Panopea g	species	97915
622976	occ	67069	Bornia lioic	species	17408
622977	occ	67069	Bornia maz	species	17408
738276	occ	79331	Bornia cf. l	species	17408
738277	occ	79331	Bornia cf. t	species	98425
741384	occ	79707	Bornia tria	species	98425
741399	occ	79706	Bornia tria	species	98425
564321	occ	58895	Bornia tria	species	98425
564360	occ	58897	Bornia tria	species	98425
566664	occ	59316	Bornia tria	species	98425
566665	occ	59330	Bornia tria	species	98425
566666	occ	59307	Bornia tria	species	98425
566667	occ	59313	Bornia tria	species	98425
566668	occ	59309	Bornia tria	species	98425
566669	occ	59317	Bornia tria	species	98425
566670	occ	59313	Bornia blac	species	17408
579677	occ	60989	Bornia long	species	17408
557394	occ	57704	Bornia long	species	17408
557427	occ	57703	Bornia long	species	17408
557482	occ	57702	Bornia long	species	17408
559145	occ	57705	Bornia long	species	17408
559187	occ	57706	Bornia long	species	17408
619616	occ	66760	Aligena ele	species	108655
833019	occ	92412	Aligena ae	species	98434
564316	occ	58895	Aligena str	species	293505
564354	occ	58897	Aligena str	species	293505
566686	occ	59307	Aligena rhc	species	17315
659703	occ	71138	Aligena str	species	293505

700958	occ		69043	Aligena str	species	293505
702898	occ		69018	Aligena rho	species	17315
727798	occ		78269	Aligena ae	species	98434
427389	occ		41705	Lima scabr	species	87560
568621	occ		59484	Lima (Man	species	158821
582571	occ		61309	Lima sp.	genus	16765
582585	occ		61310	Lima sp.	genus	16765
582586	occ		61310	Limea bror	species	16771
622943	occ	18734	67069	Lima cariba	species	90974
622944	occ		67069	Lima pelluc	species	156668
622945	occ		67069	Lima scabr	species	87560
622946	occ		67069	Limatula su	species	383441
623068	occ		67072	Lima (Man	species	158821
738203	occ		79336	Lima (Cten	species	87559
738204	occ		79336	Lima (Cten	species	158812
738205	occ		79331	Lima (Lima	subgenus	167897
743052	occ		79797	Lima (Man	species	158821
836329	occ		92335	Ctenoides	species	158832
836334	occ		92440	Limaria (Li	species	158823
836335	occ		92335	Limaria (Li	species	158813
1150760	occ		146873	Lima florpa	species	120103
578898	occ		60940	Limaria car	species	158823
642943	occ		69046	Limatula sp	genus	16769
748521	occ		80033	Limaria sp.	genus	16767
362491	occ		34269	Phacoides	species	95179
362492	occ		34269	Phacoides	species	54125
362493	occ		34269	Phacoides	genus	54125
362494	occ		34269	Phacoides	species	102587
362495	occ		34269	Divaricella	species	98460
423396	occ		41206	Divaricella	genus	17586
425332	occ		41497	Phacoides	species	82705
425393	occ		41501	Anodontia	species	94612
425415	occ		41501	Codakia co	species	156719
425421	occ		41501	Divaricella	species	17586
425439	occ		41501	Miltha calc	species	157933
425449	occ		41501	Phacoides	species	98449
425450	occ		41501	Phacoides	species	95179
425451	occ		41501	Phacoides	species	102586
425452	occ		41501	Phacoides	species	102583
425453	occ		41501	Phacoides	species	82705
426074	occ		41539	Anodontia	species	94612
426081	occ		41539	Divaricella	species	17586
426085	occ		41539	Miltha calc	species	157933
426088	occ		41539	Phacoides	species	95179
426089	occ		41539	Phacoides	species	82705
427346	occ		41705	Anodontia	species	94612
427369	occ		41705	Codakia co	species	156719

427370	occ		41705	Codakia or	species	94547
427379	occ		41705	Divaricella	genus	17586
427390	occ	19411	41705	Lucina pen	species	94553
427417	occ		41705	Phacoides	species	98449
427418	occ		41705	Phacoides	species	95179
427419	occ		41705	Phacoides	species	102586
427420	occ		41705	Phacoides	species	102583
427421	occ		41705	Phacoides	species	82705
427612	occ		41715	Phacoides	species	95179
427613	occ		41715	Phacoides	species	102586
427687	occ		41718	Anodontia	genus	17339
427706	occ		41718	Codakia or	species	94547
427709	occ		41718	Divaricella	species	17586
427716	occ	19412	41718	Lucina pen	species	94553
427718	occ		41718	Miltha calc	species	157933
427728	occ		41718	Phacoides	species	95179
427729	occ		41718	Phacoides	species	102586
427738	occ		41718	Phacoides	species	102583
427739	occ		41718	Phacoides	species	82705
427912	occ		41740	Phacoides	species	95179
427992	occ		41749	Divaricella	genus	17586
427999	occ	19413	41749	Lucina pen	species	94553
428023	occ		41749	Phacoides	species	54125
428024	occ		41749	Phacoides	species	102583
428025	occ		41749	Phacoides	genus	54125
428026	occ		41749	Phacoides	species	94561
428027	occ		41749	Phacoides	species	54125
568671	occ		59484	Codakia (Ja	species	95160
568672	occ		59484	Codakia (Ja	species	95158
568673	occ		59487	Codakia (Ja	species	95158
568674	occ		59487	Codakia (Ja	species	95157
568675	occ		59485	Lucina chry	species	109332
568676	occ		59489	Lucina chry	species	109332
568677	occ		59484	Lucina chry	species	109332
568678	occ		59484	Phacoides	species	95165
568679	occ		59490	Phacoides	species	95174
568680	occ		59484	Phacoides	species	95174
568681	occ		59486	Phacoides	species	95174
568682	occ		59485	Phacoides	species	95174
568683	occ		59488	Phacoides	species	158595
568684	occ		59487	Phacoides	species	158595
568685	occ		59490	Phacoides	species	158595
568686	occ		59485	Phacoides	species	158595
568687	occ		59484	Phacoides	species	158595
568688	occ		59491	Phacoides	species	95179
568689	occ		59484	Phacoides	species	95179
568690	occ		59485	Phacoides	species	95179

568691	occ		59484	Phacoides species	82704
568692	occ		59487	Phacoides species	82704
568693	occ		59485	Phacoides species	82704
568694	occ		59491	Phacoides species	82704
568695	occ		59486	Phacoides species	82704
582601	occ	22345	61309	Anodontia species	106101
582602	occ		61309	Anodontia species	94612
582603	occ		61309	Codakia or species	94547
582604	occ		61309	Linga wacc species	17817
582605	occ		61309	Lucina nas: species	97987
582606	occ		61309	Parvilucina species	156407
582607	occ		61309	Parvilucina species	95180
582608	occ		61309	Pseudomil species	156736
582621	occ		61310	Linga wacc species	17817
582622	occ		61310	Lucina nas: species	97987
582623	occ		61310	Lucina kee species	102584
582624	occ		61310	Parvilucina species	156407
582625	occ		61310	Parvilucina species	95180
582638	occ		61311	Anodontia species	94612
582639	occ		61311	Divaricella species	98460
582640	occ		61311	Linga pens species	156733
582641	occ		61311	Linga wacc species	17817
582642	occ		61311	Lucina nas: species	97987
582655	occ		61311	Parvilucina species	95180
622956	occ	19415	67069	Lucina pen species	94553
622957	occ		67069	Lucina mul species	95176
622958	occ		67069	Phacoides species	102586
622959	occ		67069	Phacoides species	82705
622960	occ		67069	Phacoides species	54125
622961	occ		67069	Phacoides species	102583
622962	occ		67069	Anodontia species	94612
622963	occ		67069	Miltha carr species	157935
622964	occ		67069	Codakia or species	101129
622965	occ		67069	Codakia or species	94547
622966	occ		67069	Codakia co species	156719
622967	occ		67069	Divalinga q species	102596
623066	occ		67072	Phacoides species	54125
640515	occ		69085	Lucina pen species	94553
640520	occ		69085	Radiolucina species	94606
640521	occ		69085	Stewartia f species	94543
640523	occ		69085	Callucina r species	154306
640535	occ		69084	Lucina pen species	94553
640542	occ		69084	Stewartia f species	94543
640758	occ		69087	Radiolucina species	94606
640761	occ		69087	Lucinoma s genus	17840
640769	occ		69087	Anodontia species	94612
647829	occ		70010	Stewartia a species	99275

697890	occ	74999	Phacoides	species	95179
697891	occ	74999	Phacoides	species	82704
697927	occ	75003	Phacoides	species	82704
697952	occ	75011	Phacoides	species	95171
697953	occ	75011	Phacoides	species	95179
697987	occ	75014	Phacoides	species	95164
697992	occ	75005	Phacoides	species	248700
698018	occ	75006	Phacoides	species	95179
698034	occ	75008	Phacoides	species	158595
698040	occ	75017	Phacoides	subgenus	95164
698045	occ	75019	Phacoides	species	94631
698046	occ	75019	Phacoides	subgenus	95164
698052	occ	75020	Phacoides	species	95165
698056	occ	75021	Phacoides	species	94631
698057	occ	75021	Phacoides	species	95165
698068	occ	75023	Phacoides	species	95165
698069	occ	75023	Divaricella	species	98460
738195	occ	79338	Codakia or	species	94547
738197	occ	79335	Codakia or	species	94547
738198	occ	79333	Phacoides	species	156710
738199	occ	79335	Loripinus (	species	422749
738200	occ	79333	Miltha (Mi	species	157933
738221	occ	79336	Codakia (C	species	101129
738223	occ	79336	Phacoides	species	54125
738224	occ	79336	Divaricella	species	17586
738228	occ	79334	Codakia or	species	94547
738234	occ	79331	Codakia (C	species	101129
738235	occ	79331	Phacoides	species	98449
738236	occ	79331	Phacoides	species	156710
738237	occ	79331	Phacoides	species	157845
738238	occ	79331	Phacoides	species	248700
738239	occ	79331	Phacoides	species	95165
738240	occ	79331	Phacoides	species	95179
738241	occ	79331	Phacoides	species	82705
738242	occ	79331	Phacoides	species	54125
738243	occ	79331	Phacoides	species	102586
738244	occ	79331	Miltha (Mi	species	157933
738245	occ	79331	Divaricella	species	17586
738273	occ	79331	Lucina (Luc	species	94553
741912	occ	79739	Pseudomil	species	156736
742052	occ	79747	Miltha carr	species	157935
742058	occ	79747	Codakia sp	genus	17488
742549	occ	35031	Miltha sp.	genus	17916
743057	occ	79797	Phacoides	species	54125
745347	occ	79789	Stewartia a	species	99275
745357	occ	79789	Lucinisca n	species	97988
745359	occ	79789	Callucina r	species	154306

745373	occ	79788	Stewartia a	species	99275
745395	occ	79788	Lucinisca n	species	97988
745458	occ	69038	Stewartia a	species	99275
747007	occ	80002	Phacoides	species	95179
747029	occ	80001	Phacoides	species	98449
747030	occ	80001	Phacoides	species	95179
747035	occ	80001	Phacoides	species	82705
747257	occ	80027	Stewartia f	species	94543
747368	occ	69029	Bellucina w	species	82706
747379	occ	69029	Lucinisca n	species	97988
747388	occ	69029	Parvilucina	species	95180
747430	occ	79786	Lucina pen	species	94553
747450	occ	79786	Radiolucina	species	94604
747457	occ	79786	Parvilucina	species	95180
747478	occ	79787	Pegophyse	species	160643
747491	occ	79787	Lucinisca n	species	97988
747512	occ	79787	Radiolucina	species	94606
747522	occ	79786	Stewartia a	species	99275
747581	occ	80030	Lucinisca n	species	97988
747778	occ	80046	Pegophyse	genus	18045
747816	occ	80046	Radiolucina	species	94604
748149	occ	69034	Radiolucina	species	94604
748315	occ	80025	Stewartia a	species	99275
748327	occ	80024	Bellucina w	species	82706
751001	occ	80359	Phacoides	species	82705
751010	occ	80358	Phacoides	species	95179
751029	occ	80357	Phacoides	species	82705
751040	occ	80356	Phacoides	species	95179
833899	occ	92331	Miltha (Mi	species	157933
833900	occ	92338	Miltha (Mi	species	157933
833901	occ	92336	Miltha (Mi	species	157933
833902	occ	92340	Miltha (Mi	species	157933
833903	occ	92342	Miltha (Mi	species	157933
833904	occ	92343	Miltha (Mi	species	157933
833905	occ	92333	Miltha (Mi	species	157933
833906	occ	92364	Miltha (Mi	species	157933
833907	occ	92498	Miltha (Mi	species	157933
833908	occ	92500	Miltha (Mi	species	157933
833909	occ	92344	Miltha (Mi	species	157933
833910	occ	92442	Miltha (Mi	species	157933
833911	occ	92361	Miltha (Mi	species	157933
833912	occ	92362	Miltha (Mi	species	157933
833913	occ	92363	Miltha (Mi	species	157933
833914	occ	92624	Miltha (Mi	species	157933
833915	occ	92656	Miltha (Mi	species	157933
833916	occ	92657	Miltha (Mi	species	157933
833917	occ	92658	Miltha (Mi	species	157933

833918	occ	92659	Miltha (Mi	species	157933
833919	occ	92660	Miltha (Mi	species	157933
833920	occ	92661	Miltha (Mi	species	157933
833921	occ	92662	Miltha (Mi	species	157933
833922	occ	92663	Miltha (Mi	species	157933
833923	occ	92664	Miltha (Mi	species	157933
833924	occ	92665	Miltha (Mi	species	157933
833925	occ	92666	Miltha (Mi	species	157933
833926	occ	92667	Miltha (Mi	species	157933
833927	occ	92668	Miltha (Mi	species	157933
833929	occ	92621	Miltha (Mi	species	157935
833930	occ	92369	Miltha (Mi	species	157935
833933	occ	92674	Miltha (Mi	species	157935
833934	occ	92673	Miltha (Mi	species	157935
833935	occ	92672	Miltha (Mi	species	157935
833936	occ	92671	Miltha (Mi	species	157935
853744	occ	94809	Phacoides	species	95179
853745	occ	94809	Phacoides	species	158595
853746	occ	94809	Phacoides	species	98451
853747	occ	94809	Phacoides	species	95174
1087898	occ	96538	Anodontia	species	94612
1087899	occ	96538	Callucina k	species	102585
1087900	occ	96538	Codakia or	species	94547
1087901	occ	96538	Divalinga q	species	102596
1087902	occ	96538	Lucina pen	species	94553
1087903	occ	96538	Lucinisca n	species	97988
1087904	occ	96538	Parvilucina	species	102589
1087906	occ	96538	Radiolucina	species	94606
1087907	occ	96538	Stewartia f	species	94543
1317134	occ	176823	Lucinoma (	species	17840
256373	occ	20604	Divaricella	species	98460
256391	occ	20606	Divaricella	species	98460
578551	occ	60923	Phacoides	species	95179
578606	occ	60927	Phacoides	species	95179
578607	occ	60927	Phacoides	species	82705
578653	occ	60928	Divaricella	species	98460
578661	occ	60928	Lucina pun	species	17835
578671	occ	60928	Phacoides	species	95179
578728	occ	60929	Divaricella	species	98460
578735	occ	60929	Phacoides	species	95179
578771	occ	60930	Phacoides	species	95179
578903	occ	60940	Linga pens	species	156733
578904	occ	60940	Lucinisca c	species	95175
578905	occ	60940	Radiolucina	species	94604
578906	occ	60940	Ctena spec	species	95159
578907	occ	60940	Pseudomil	species	98449
578908	occ	60940	Divalinga q	species	102596

578938	occ	60939	Radiolucina	species	94604
578939	occ	60939	Parvilucina	species	95180
578940	occ	60939	Ctena spec	species	95159
579033	occ	60944	Phacoides	species	98449
579034	occ	60944	Phacoides	species	95179
579035	occ	60944	Phacoides	species	82705
619561	occ	66760	Phacoides	species	155484
619565	occ	66760	Phacoides	species	102583
619566	occ	66758	Lucina mul	species	95176
619569	occ	66758	Lucina ami	species	156734
619570	occ	66760	Lucina ami	species	156734
619571	occ	66757	Phacoides	species	102588
619572	occ	66756	Phacoides	species	102588
619600	occ	66756	Codakia co	species	156719
619602	occ	66760	Divaricella	species	98460
619603	occ	66758	Divaricella	species	98460
619604	occ	66756	Divaricella	species	98460
619605	occ	66755	Divaricella	species	98460
642884	occ	69046	Linga wacc	species	17817
642950	occ	69046	Divalinga (	species	109760
741369	occ	79706	Divalinga q	species	102596
741374	occ	79705	Divalinga q	species	102596
741382	occ	79707	Radiolucina	species	94606
741385	occ	79707	Divalinga q	species	102596
741391	occ	79706	Radiolucina	species	94606
741405	occ	79705	Radiolucina	species	94606
745504	occ	79957	Parvilucina	species	95180
747830	occ	80053	Divalinga q	species	102596
747832	occ	80053	Radiolucina	species	94606
747834	occ	80053	Stewartia a	species	99275
747836	occ	80053	Parvilucina	species	95180
748101	occ	80083	Radiolucina	species	94604
748103	occ	80083	Stewartia a	species	99275
832936	occ	92408	Phacoides	species	82705
832937	occ	92408	Phacoides	species	102583
832959	occ	92409	Phacoides	species	95179
832960	occ	92409	Phacoides	species	82705
833011	occ	92412	Phacoides	species	95174
833012	occ	92412	Phacoides	species	54125
833013	occ	92412	Phacoides	species	158595
833014	occ	92412	Phacoides	species	102583
833017	occ	92412	Divaricella	species	98460
1457452	occ	79950	Lucina n. s	species	392334
1457468	occ	203376	Lucina cost	species	156717
1457469	occ	203376	Lucina crer	species	98454
564326	occ	58895	Cavilinga t	species	82708
564375	occ	58897	Cavilinga t	species	82708

564642	occ	58895	Parvilucina	species	95180
564902	occ	58898	Stewartia a	species	99275
564906	occ	58897	Parvilucina	species	95180
566640	occ	59307	Ctena mag	species	95161
566641	occ	59307	Ctena spec	species	95159
566642	occ	59307	Ctena micr	species	95162
566643	occ	59313	Phacoides	species	95165
566644	occ	59309	Phacoides	species	95165
566645	occ	59310	Phacoides	species	95165
566646	occ	59318	Phacoides	species	95165
566647	occ	59327	Phacoides	species	95174
566648	occ	59330	Phacoides	species	95179
566649	occ	59307	Phacoides	species	95179
566650	occ	59312	Phacoides	species	95179
566651	occ	59309	Phacoides	species	95179
566652	occ	59313	Phacoides	species	95179
566653	occ	59315	Phacoides	species	95179
566654	occ	59316	Phacoides	species	95179
566655	occ	59318	Phacoides	species	95179
579675	occ	60989	Parvilucina	species	95180
579812	occ	60987	Parvilucina	species	95180
619562	occ	66742	Phacoides	species	155484
619563	occ	66742	Phacoides	species	102583
619564	occ	66742	Lucina mul	species	95176
619567	occ	66742	Lucina ami	species	156734
619568	occ	66742	Phacoides	species	102586
619573	occ	66750	Phacoides	species	102588
619574	occ	66748	Phacoides	species	102588
619575	occ	66747	Phacoides	species	102588
619576	occ	66742	Phacoides	species	102588
619599	occ	66749	Anodontia	species	94612
619601	occ	66742	Codakia co	species	156719
619606	occ	66749	Divaricella	species	98460
619607	occ	66748	Divaricella	species	98460
619608	occ	66747	Divaricella	species	98460
619609	occ	66742	Divaricella	species	98460
642710	occ	69045	Divalinga (I	species	109760
642715	occ	69045	Linga wacc	species	17817
642716	occ	69045	Callucina ((	species	154306
642728	occ	69045	Cavilinga tı	species	82708
659687	occ	71138	Parvilucina	species	158594
659728	occ	71138	Stewartia a	species	99275
659732	occ	71138	Cavilinga tı	species	82708
700938	occ	69043	Stewartia a	species	99275
700962	occ	69043	Bellucina w	species	82706
701055	occ	69047	Divalinga s	genus	17583
737409	occ	79249	Lucinidae i	family	60971

737422	occ	79248	Lucinidae i	family	60971
737433	occ	79248	Stewartia a	species	99275
738398	occ	69043	Cardiolucir	species	95163
738418	occ	79342	Cardiolucir	species	95163
738491	occ	79339	Cavilucina	genus	17452
738503	occ	79339	Stewartia 1	species	99275
738527	occ	79340	Cavilucina	genus	17452
738540	occ	79340	Lucina pen	species	94553
738543	occ	79340	Lucinidae i	family	60971
748214	occ	80034	Stewartia a	species	99275
748236	occ	80034	Divalinga q	species	102596
748363	occ	80033	Stewartia (	species	99275
748395	occ	80033	Divalinga q	species	102596
748452	occ	80033	Cavilinga s	genus	17451
748453	occ	80033	Lucina sp.	genus	17835
748511	occ	80033	Linga sp.	genus	17817
748513	occ	80033	Stewartia (	species	99275
748514	occ	80033	Callucina s	genus	17428
748524	occ	80033	Bellucina s	genus	17402
748552	occ	80035	Divalinga q	species	102596
748562	occ	80035	Linga sp.	genus	17817
748563	occ	80035	Bellucina w	species	82706
748573	occ	80035	Miltha sp.	genus	17916
748656	occ	80035	Stewartia a	species	99275
559152	occ	57705	Divalinga q	species	102596
559170	occ	57705	Parvilucina	species	95180
559193	occ	57706	Divalinga q	species	102596
559211	occ	57702	Divalinga q	species	102596
566290	occ	59297	Stewartia a	species	99275
566291	occ	59297	Lucinoma (	species	99277
566313	occ	59293	Phacoides	species	98449
582222	occ	61293	Parvilucina	species	158594
582243	occ	61294	Parvilucina	species	158594
582251	occ	61295	Parvilucina	species	158594
582262	occ	61296	Parvilucina	species	158594
664778	occ	71620	Stewartia a	species	99275
664834	occ	71624	Stewartia a	species	99275
727677	occ	78271	Parvilucina	species	118933
727794	occ	78269	Parvilucina	species	118933
727853	occ	78267	Parvilucina	species	118933
727912	occ	78266	Parvilucina	species	118933
727975	occ	78265	Parvilucina	species	118933
728036	occ	78261	Parvilucina	species	118933
728077	occ	78262	Ctena spec	species	95159
728078	occ	78262	Parvilucina	species	158594
728129	occ	78264	Parvilucina	species	158594
737458	occ	79247	Stewartia a	species	99275

737463	occ	79246	Stewartia a	species	99275
737480	occ	79247	Divalinga q	species	102596
741457	occ	79709	Parvilucina	species	118933
748172	occ	80040	Divalinga q	species	102596
748179	occ	80040	Stewartia a	species	99275
748195	occ	80040	Lucinisca c	species	17839
748245	occ	80038	Stewartia a	species	99275
748434	occ	80036	Stewartia (	species	99275
559158	occ	57705	Lyonsia hya	species	107598
362520	occ	34269	Mulinia lat	species	94392
362521	occ	34269	Mactra sp.	genus	17859
362522	occ	34269	Spisula soli	species	94390
362523	occ	34269	Rangia cf. (	species	18179
423345	occ	41202	Spisula sp.	genus	18275
425328	occ	41497	Mulinia lat	species	94392
425333	occ	41497	Rangia nas	species	156284
425370	occ	41498	Mulinia lat	species	94392
425440	occ	41501	Mulinia lat	species	94392
425441	occ	41501	Mulinia sap	species	17933
425457	occ	41501	Rangia nas	species	156284
427411	occ	41705	Mulinia lat	species	94392
427609	occ	41715	Mulinia lat	species	94392
427719	occ	41718	Mulinia lat	species	94392
427720	occ	41718	Mulinia sap	species	17933
427742	occ	41718	Rangia nas	species	156284
427909	occ	41740	Mulinia lat	species	94392
428003	occ	41749	Mulinia co	species	120108
428004	occ	41749	Mulinia sap	species	17933
428028	occ	41749	Rangia nas	species	156284
568771	occ	59484	Mactra (M	species	171574
568772	occ	59486	Spisula (He	species	97975
568773	occ	59487	Spisula sp.	genus	18275
568774	occ	59484	Spisula sp.	genus	18275
568775	occ	59485	Spisula sp.	genus	18275
568776	occ	59488	Mulinia co	species	120108
568777	occ	59487	Mulinia co	species	120108
568778	occ	59490	Mulinia co	species	120108
568779	occ	59484	Mulinia co	species	120108
582620	occ	61309	Mactra fra	species	156278
582635	occ	61310	Mulinia lat	species	94392
582637	occ	61310	Rangia cun	species	94526
582644	occ	61311	Rangia cun	species	94526
582646	occ	61311	Mulinia lat	species	94392
582656	occ	61309	Mulinia lat	species	94392
582658	occ	61309	Rangia cun	species	94526
623011	occ	67069	Mactra fra	species	156278
623012	occ	67069	Mulinia sp.	genus	17933

623014	occ	67069	Rangia cun	species	94526
640549	occ	69084	Mactrotom	species	156279
640736	occ	69087	Spisula sp.	genus	18275
647836	occ	70010	Mulinia sa	species	17933
697900	occ	75009	Mulinia co	species	120108
697924	occ	75003	Mulinia cf.	species	94392
697939	occ	75004	Mulinia lat	species	94392
697970	occ	75012	Mulinia co	species	120108
697980	occ	75013	Mulinia co	species	120108
697984	occ	75014	Mulinia co	species	120108
697999	occ	75015	Mulinia sp.	genus	17933
698047	occ	75019	Mulinia cf.	species	120108
738321	occ	79333	Mulinia sa	species	17933
738328	occ	79336	Mulinia sa	species	17933
738352	occ	79331	Mactra (M	species	92356
738353	occ	79331	Mulinia co	species	120108
738354	occ	79331	Mulinia lat	species	94392
738355	occ	79331	Mulinia cal	species	17933
738356	occ	79331	Mulinia sa	species	17933
738357	occ	79331	Rangia nas	species	156284
741878	occ	79738	Rangia cun	species	94526
741973	occ	79749	Mulinia sp.	genus	17933
741993	occ	79748	Mulinia lat	species	94392
742185	occ	79761	Spisula sub	species	97983
742191	occ	79761	Spisula inc	species	18275
743717	occ	79847	Mulinia sp.	genus	17933
744344	occ	79876	Mulinia sa	species	17933
745383	occ	79788	Mulinia lat	species	94392
745419	occ	69040	Mulinia sa	species	17933
745428	occ	69040	Hemimact	species	17724
745545	occ	79962	Mulinia co	species	120108
747028	occ	80001	Mulinia cf.	species	17933
747300	occ	69044	Mactrotom	genus	120906
747350	occ	69029	Mulinia sa	species	17933
747366	occ	69029	Mactrotom	species	120906
747464	occ	79786	Mulinia lat	species	94392
747505	occ	79787	Mulinia lat	species	94392
747563	occ	80030	Mulinia co	species	120108
747577	occ	80030	Mulinia lat	species	94392
747585	occ	80029	Mulinia co	species	120108
747627	occ	80037	Mulinia co	species	120108
747655	occ	80039	Mulinia co	species	120108
747746	occ	80046	Rangia cun	species	94526
747760	occ	80046	Hemimact	genus	17724
747800	occ	80046	Mulinia lat	species	94392
748128	occ	69034	Hemimact	species	17724
748131	occ	69034	Rangia cun	species	94526

748145	occ		69034	Mulinia sa|	species	17933
748151	occ		69034	Mactroton	species	156279
748163	occ		69034	Mactridae	family	61026
748287	occ		80025	Rangia cun	species	94526
748306	occ		80025	Mulinia lat	species	94392
748321	occ		80024	Rangia cun	species	94526
748339	occ		80024	Mulinia cal	species	17933
751011	occ		80358	Rangia nas	species	156284
1088913	occ		96538	Mactroton	species	156279
1088914	occ		96538	Mulinia lat	species	94392
1088916	occ		96538	Rangia cun	species	94526
1317133	occ		176823	Mulinia co	species	120108
256375	occ		20604	Mulinia lat	species	94392
256393	occ		20606	Mulinia lat	species	94392
256433	occ		20613	Mulinia lat	species	94392
619178	occ		66724	Mulinia lat	species	94392
1248041	occ		164698	Rangia cun	species	94526
1248042	occ		164698	Mulinia lat	species	94392
1248043	occ		164698	Mulinia co	species	120108
578549	occ		60923	Mulinia lat	species	94392
578552	occ		60923	Spisula soli	species	94390
578602	occ		60927	Mulinia lat	species	94392
578609	occ		60927	Spisula soli	species	94390
578665	occ		60928	Mulinia lat	species	94392
578673	occ		60928	Spisula soli	species	94390
578734	occ		60929	Mulinia lat	species	94392
578768	occ		60930	Mulinia lat	species	94392
578773	occ		60930	Spisula soli	species	94390
578919	occ	21624	60940	Spisula soli	species	94390
578920	occ		60940	Mulinia co	species	120108
579038	occ		60944	Rangia clat	species	18179
579039	occ	21625	60944	Spisula soli	species	94390
619741	occ		66760	Mulinia lat	species	94392
619742	occ		66757	Mulinia lat	species	94392
619743	occ		66758	Mulinia lat	species	94392
619744	occ		66756	Mulinia lat	species	94392
619745	occ		66755	Mulinia lat	species	94392
619746	occ		66755	Spisula soli	species	94390
619747	occ		66756	Spisula soli	species	94390
619751	occ		66760	Mactra fra	species	156278
642862	occ		69046	Spisula sim	species	156541
642863	occ		69046	Mulinia lat	species	94392
741355	occ		79707	Mulinia lat	species	94392
741359	occ		79706	Mulinia lat	species	94392
741360	occ		79705	Mulinia lat	species	94392
745461	occ		79956	Mulinia lat	species	94392
745466	occ		79956	Hemimact	genus	17724

745485	occ		79957	Mulinia lat species	94392
745495	occ		79957	Hemimacti genus	17724
747826	occ		80053	Mulinia lat species	94392
747842	occ		80053	Hemimacti genus	17724
748085	occ		80083	Mulinia co species	120108
748111	occ		80083	Hemimacti genus	17724
832862	occ		92405	Mactra late species	94391
832866	occ		92406	Mactra late species	94391
832867	occ		92406	Mactra sim species	156540
832870	occ		92406	Gnathodor species	157897
832887	occ		92407	Mactra late species	94391
832945	occ		92408	Mulinia lat species	94392
832947	occ		92408	Rangia clat species	18179
1457471	occ		203376	Mactra sim species	156540
564649	occ	21619	58895	Spisula soli species	94390
564914	occ	21620	58897	Spisula soli species	94390
566773	occ		59316	Mactra (M species	156281
566774	occ		59310	Spisula (He species	85857
566775	occ		59330	Spisula (He species	85857
566832	occ	21621	59310	Spisula soli species	94390
566833	occ	21622	59315	Spisula soli species	94390
566834	occ	21623	59309	Spisula soli species	94390
566835	occ		59331	Mulinia co species	120108
566836	occ		59329	Mulinia co species	120108
566837	occ		59327	Mulinia co species	120108
566838	occ		59308	Mulinia co species	120108
566839	occ		59307	Mulinia co species	120108
566840	occ		59313	Mulinia co species	120108
566841	occ		59310	Mulinia co species	120108
566842	occ		59318	Mulinia co species	120108
566843	occ		59317	Mulinia co species	120108
566844	occ		59330	Mulinia co species	120108
566845	occ		59316	Mulinia co species	120108
566846	occ		59309	Mulinia co species	120108
579605	occ		60986	Mulinia lat species	94392
579679	occ		60989	Mulinia lat species	94392
579740	occ		60988	Spisula sp. genus	18275
579741	occ		60988	Mulinia lat species	94392
579814	occ		60987	Mulinia lat species	94392
619724	occ		66750	Rangia cun species	94526
619725	occ		66754	Rangia cun species	94526
619726	occ		66749	Rangia cun species	94526
619731	occ		66754	Mulinia lat species	94392
619732	occ		66752	Mulinia lat species	94392
619733	occ		66751	Mulinia lat species	94392
619734	occ		66750	Mulinia lat species	94392
619735	occ		66749	Mulinia lat species	94392

619736	occ		66748	Mulinia lat	species	94392
619737	occ		66747	Mulinia lat	species	94392
619738	occ		66746	Mulinia lat	species	94392
619739	occ		66744	Mulinia lat	species	94392
619740	occ		66742	Mulinia lat	species	94392
619748	occ		66744	Spisula soli	species	94390
619749	occ		66742	Spisula soli	species	94390
619750	occ		66747	Spisula soli	species	94390
642729	occ		69045	Spisula soli	species	94390
642742	occ		69045	Mulinia lat	species	94392
659742	occ	21626	71138	Spisula soli	species	94390
700947	occ		69043	Mulinia lat	species	94392
700961	occ		69043	Mactridae	family	61026
701056	occ		69047	Mulinia lat	species	94392
701061	occ		69047	Hemimacti	species	156539
702852	occ		68979	Mulinia co	species	120108
702883	occ		69018	Mulinia co	species	120108
702913	occ		69018	Hemimacti	species	156539
737378	occ		68979	Mactridae	family	61026
737390	occ		79249	Mulinia co	species	120108
737398	occ		79248	Mulinia co	species	120108
737435	occ		79248	Hemimacti	species	17724
738400	occ		79342	Mulinia co	species	120108
738430	occ		79343	Mulinia co	species	120108
738443	occ		79343	"Spisula" s	genus	18275
738457	occ		79341	Mulinia co	species	120108
738548	occ		79340	Mactridae	family	61026
741424	occ		79708	Rangia cun	species	94526
748212	occ		80034	Mulinia lat	species	94392
748216	occ		80034	Hemimacti	species	156539
748362	occ		80033	Hemimacti	species	17724
748386	occ		80033	Hemimacti	genus	17724
748394	occ		80033	Mulinia sp.	genus	17933
748444	occ		80033	Harvella sp	genus	111710
748485	occ		80033	Spisula soli	species	94390
748525	occ		80033	Mulinia lat	species	94392
748569	occ		80035	Hemimacti	species	17724
748576	occ		80035	Spisula cf.	species	94390
829362	occ		91458	Mulinia lat	species	94392
829363	occ		91459	Mulinia lat	species	94392
829369	occ		91461	Mulinia lat	species	94392
829370	occ		91462	Mulinia lat	species	94392
829374	occ		91463	Mulinia lat	species	94392
829459	occ		91488	Mulinia lat	species	94392
829461	occ		91489	Mulinia lat	species	94392
1173573	occ		151326	Spisula mo	species	18275
1173574	occ		151326	Mulinia co	species	120108

1173575	occ		151326	Hemimacti	species	17724
1512846	occ		211183	Mulinia lat	species	94392
1512975	occ		211182	Mulinia lat	species	94392
94758	occ		7108	Mulinia lat	species	94392
94762	occ		7108	Mactra sol	species	94388
94767	occ	19410	7108	Rangia cun	species	94526
559162	occ		57705	Mulinia lat	species	94392
559174	occ		57705	Rangia cun	species	94526
559177	occ		57705	Spisula soli	species	94390
559203	occ		57706	Rangia cun	species	94526
559204	occ		57706	Spisula soli	species	94390
559206	occ		57707	Spisula soli	species	94390
559217	occ		57702	Mulinia lat	species	94392
559231	occ		57703	Mulinia lat	species	94392
559237	occ		57703	Rangia cun	species	94526
559244	occ		57704	Mulinia lat	species	94392
559250	occ		57704	Rangia cun	species	94526
566317	occ		59293	Spisula cor	species	97981
640355	occ		69048	Spisula soli	species	94390
640373	occ		69049	Rangia cun	species	94526
640379	occ		69049	Mulinia lat	species	94392
727759	occ		78270	Leptomact	species	99315
727760	occ		78270	"Spisula" s	genus	18275
727761	occ		78270	"Spisula" n	species	18275
727762	occ		78270	Mulinia co	species	120108
727788	occ		78269	"Spisula" n	species	18275
727796	occ		78269	Mulinia co	species	120108
727877	occ		78267	Mulinia co	species	120108
727878	occ		78267	"Spisula" n	species	18275
727901	occ		78266	Rangia clat	species	18179
727963	occ		78265	"Spisula" n	species	18275
728029	occ		78261	Leptomact	species	99315
728114	occ		78264	Leptomact	species	99315
728115	occ		78264	"Spisula" n	species	18275
737468	occ		79247	Mulinia co	species	120108
737475	occ		79246	Mulinia co	species	17933
737478	occ		79246	Mactridae	family	61026
737488	occ		79245	Mulinia co	species	17933
741449	occ		79709	Mulinia co	species	120108
747720	occ		80048	Mulinia lat	species	94392
748175	occ		80040	Mulinia lat	species	94392
748252	occ		80038	Mulinia lat	species	94392
748426	occ		80036	Hemimacti	species	156539
748431	occ		80036	Mulinia lat	species	94392
425432	occ		41501	Isognomor	species	156536
428022	occ		41749	Pedalion k	species	369733
622932	occ		67069	Isognomor	species	16730

738168	occ		79331	Isognomor	species	156536
566315	occ		59293	Pedalion m	species	369733
566323	occ		59294	Pedalion m	species	369733
748208	occ		80040	Isognomor	species	16730
745543	occ		79962	Margaritar	species	98494
727851	occ		78267	Margaritar	species	98494
727909	occ		78266	Margaritar	species	98494
582610	occ	22485	61309	Neaeromy.	species	91184
582626	occ		61310	Mysella pl	species	17949
582627	occ		61310	Neaeromy.	species	91184
582652	occ		61311	Neaeromy.	species	91184
582653	occ		61311	Mysella pl	species	17949
738281	occ		79331	Montacuta	species	91183
745408	occ		79788	Montacuta	genus	17931
1087912	occ		96538	Mysella pl	species	17949
1087913	occ		96538	Orobitella	species	91185
578550	occ		60923	Mysella ve	species	17949
578666	occ		60928	Mysella ve	species	17949
564398	occ		58897	Mysella be	species	82710
566681	occ		59318	Mysella sta	species	17949
566682	occ		59316	Mysella sta	species	17949
566683	occ		59316	Mysella ve	species	17949
566684	occ		59313	Mysella ve	species	17949
566685	occ		59316	Mysella m	species	17949
619617	occ		66752	Mysella pl	species	17949
619618	occ		66751	Mysella pl	species	17949
659718	occ		71138	Mysella bl	species	17949
582705	occ		61310	Sphenia sp	genus	18504
623046	occ		67069	Sphenia tu	species	18504
738366	occ		79331	Sphenia at	species	18504
747331	occ		69029	Mya arena	species	95559
1088918	occ		96538	Sphenia tu	species	18504
619789	occ		66757	Mya arena	species	95559
619790	occ		66760	Mya arena	species	95559
832886	occ		92407	Myalina su	species	97927
1457477	occ		203376	Mya arena	species	95559
564602	occ		58893	Paramya s	species	97928
564641	occ		58895	Paramya s	species	97928
564905	occ		58897	Paramya s	species	97928
566920	occ		59316	Mya arena	species	95559
566921	occ		59317	Mya arena	species	95559
579609	occ		60986	Sphenia sp	genus	18504
579685	occ		60989	Sphenia sp	genus	18504
579766	occ		60988	Sphenia sp	genus	18504
579818	occ		60987	Sphenia sp	genus	18504
619787	occ		66746	Mya arena	species	95559
700949	occ		69043	Mya (Aren	species	101900

702874	occ	68979	Mya (Aren	species	101900
741427	occ	79708	Sphenia du	species	97926
1512840	occ	211183	Sphenia sp	genus	18504
1512977	occ	211182	Sphenia sp	genus	18504
559169	occ	57705	Paramya su	species	97928
559245	occ	57704	Mya arena	species	95559
559248	occ	57704	Paramya su	species	97928
727789	occ	78269	Sphenia du	species	97926
728116	occ	78264	Sphenia du	species	97926
737377	occ	68979	Myochama	genus	18767
737515	occ	79245	Myochama	genus	18767
425400	occ	41501	Brachiodor	species	64227
427350	occ	41705	Brachiodor	species	64227
427375	occ	41705	Crenella di	species	100455
427410	occ	41705	Modiolus ?	species	90991
427693	occ	41718	Brachiodor	species	64227
428005	occ	41749	Mytilus cor	species	16392
568628	occ	59491	Mytilus cor	species	16392
582568	occ	61309	Brachidont	species	87573
582569	occ	61309	Modiolus s	genus	16379
582582	occ	61310	Brachidont	species	87573
582583	occ	61310	Lithophaga	genus	16370
582595	occ	61311	Brachidont	species	87573
622922	occ	67069	Crenella di	species	100455
622923	occ	67069	Lithophaga	genus	16370
622924	occ	67069	Modiolus a	species	90991
622925	occ	67069	Brachidont	species	87573
622928	occ	67069	Botula fusc	species	90978
640754	occ	69087	Brachidont	genus	16347
640760	occ	69087	Modiolus c	species	90991
697932	occ	75010	Mytilus sp.	genus	16392
697971	occ	75012	Mytilus sp.	genus	16392
698006	occ	75016	Mytilus sp.	genus	16392
738208	occ	79331	Brachidont	species	110030
738209	occ	79331	Brachidont	species	110029
738210	occ	79331	Crenella di	species	100455
742171	occ	79761	Mytilocon	species	90011
742173	occ	79764	Mytilocon	species	90011
742496	occ	79761	Mytilus inc	species	16392
744369	occ	79875	Perna conr	species	16396
745330	occ	79789	Brachidont	species	87573
747021	occ	80001	Brachidont	genus	16347
747451	occ	79786	Modiolus s	genus	16379
747506	occ	79787	Crenella di	species	100455
747507	occ	79787	Brachiodor	species	64227
747519	occ	79787	Modiolus s	genus	16379
747613	occ	80037	Perna sp.	genus	16396

747694	occ	80044	Modiolus s	genus	16379
747773	occ	80046	Modiolus s	genus	16379
747808	occ	80046	Brachiodor	species	64227
748155	occ	69034	Modiolus s	genus	16379
748308	occ	80025	Brachiodor	species	64227
748333	occ	80024	Brachiodor	species	64227
750997	occ	80359	Brachidont	genus	16347
834795	occ	92741	Mytilocon	genus	90011
1087882	occ	96538	Brachidont	species	87573
1197528	occ	22712	Perna conr	species	16396
1248045	occ	164698	Modiolaria	genus	83859
578885	occ	60940	Modiolus g	species	16379
578886	occ	60940	Lithophaga	genus	16370
619549	occ	66760	Brachidont	species	87573
642941	occ	69046	Mytilus sp.	genus	16392
642942	occ	69046	Aulacomya	genus	16343
748109	occ	80083	Modiolus s	genus	16379
832983	occ	92410	Mytilus sp.	genus	16392
564288	occ	58893	Crenella de	species	132362
564306	occ	58893	Modiolus c	species	87568
564331	occ	58895	Crenella de	species	132362
564347	occ	58895	Mytilus sp.	genus	16392
564350	occ	58895	Modiolus c	species	87568
564381	occ	58897	Crenella de	species	132362
564392	occ	58898	Modiolus c	species	87568
566530	occ	59313	Brachidont	species	87575
566531	occ	59316	Brachidont	species	87575
579672	occ	60989	Modiolus s	genus	16379
579736	occ	60988	Modiolus s	genus	16379
619550	occ	66746	Brachidont	species	87575
659685	occ	71138	Modiolus c	species	158589
701065	occ	69047	Modiolus r	species	87568
738396	occ	69043	Brachidont	genus	16347
94766	occ	7108	Brachidont	species	87575
559161	occ	57705	Modiolus s	species	16379
559163	occ	57705	Mytilus ed	species	87571
559196	occ	57706	Modiolus s	species	16379
559213	occ	57702	Geukensia	species	107587
559214	occ	57702	Ischadium	species	90989
559216	occ	57702	Modiolus s	species	16379
727918	occ	78266	Modiolus c	species	16379
741478	occ	79709	Ischadium	species	90989
748437	occ	80036	Brachiodor	genus	64227
425326	occ	41497	Fossularca	species	95756
425369	occ	41498	Fossularca	species	95756
425427	occ	41501	Fossularca	species	95756
425442	occ	41501	Noetia plat	species	16277

426084	occ	41539	Fossularca	species	95756
426086	occ	41539	Noetia plat	species	16277
427383	occ	41705	Fossularca	species	95756
427610	occ	41715	Noetia por	species	87629
427713	occ	41718	Fossularca	species	95756
427721	occ	41718	Noetia plat	species	16277
428006	occ	41749	Noetia por	species	87629
568574	occ	59484	Arca (Fossu	species	82689
568575	occ	59487	Arca (Noet	species	98541
568576	occ	59485	Arca (Noet	species	98541
568577	occ	59484	Arca (Noet	species	98541
582533	occ	61309	Arcopsis ac	species	90942
582535	occ	61309	Noetia plat	species	16277
582536	occ	61309	Noetia por	species	87629
582553	occ	61310	Arcopsis ac	species	90942
582555	occ	61310	Noetia por	species	87629
582565	occ	61311	Arcopsis ac	species	90942
582566	occ	61311	Noetia por	species	87629
622914	occ	67069	Arcopsis ac	species	90942
622919	occ	67069	Eontia play	species	16238
640777	occ	69087	Arcopsis ac	species	90942
697887	occ	74999	Arcopsis ac	species	90942
697912	occ	75001	Arcopsis ac	species	90942
697931	occ	75010	Arcopsis ac	species	90942
697942	occ	75004	Eontia vari	species	16238
738157	occ	79331	Fossularca	species	95756
738165	occ	79331	Noetia (Eo	species	87629
741767	occ	79731	Noetia por	species	87629
741974	occ	79749	Noetia sp.	genus	16277
741985	occ	79748	Noetia limı	species	16277
742057	occ	79747	Noetia play	species	16277
742546	occ	35031	Noetia (Eo	species	87628
745368	occ	79788	Noetia por	species	87629
745444	occ	69040	Arcopsis ac	species	90942
747313	occ	69044	Arcopsis ac	species	90942
747385	occ	69029	Arcopsis ac	species	90942
747455	occ	79786	Arcopsis ac	species	90942
747578	occ	80030	Arcopsis ac	species	90942
747634	occ	80037	Noetia inci	species	116339
747657	occ	80039	Noetia inci	species	116339
747761	occ	80046	Noetia inci	species	116339
747774	occ	80046	Arcopsis ac	species	90942
748147	occ	69034	Arcopsis ac	species	90942
1087878	occ	96538	Arcopsis ac	species	90942
1087879	occ	96538	Noetia cf. p	species	16277
1087880	occ	96538	Noetia por	species	87629
256376	occ	20604	Noetia por	species	87629

256394	occ	20606	Noetia por	species	87629
618760	occ	66724	Noetia (Eo	species	87629
618762	occ	66725	Noetia (Eo	species	87629
578603	occ	60927	Noetia sp.	genus	16277
578724	occ	60929	Arcopsis ac	species	90942
578769	occ	60930	Noetia por	species	87629
578881	occ	60940	Noetia trig	species	16277
578882	occ	60940	Noetia inci	species	116339
619467	occ	66758	Arca adam	species	82689
619511	occ	66755	Noetia (Eo	species	87629
619512	occ	66758	Noetia (Eo	species	87629
619513	occ	66760	Noetia (Eo	species	87629
642876	occ	69046	Arcopsis ac	species	90942
642877	occ	69046	Noetia (No	species	90005
745477	occ	79956	Noetia por	species	87629
747833	occ	80053	Noetia inci	species	116339
832864	occ	92406	Arca ponde	species	87626
832926	occ	92408	Arca adam	species	82689
832952	occ	92409	Arca adam	species	82689
564348	occ	58895	Noetia lim	species	16277
564393	occ	58898	Noetia lim	species	16277
579671	occ	60989	Noetia lim	species	16277
579809	occ	60987	Noetia lim	species	16277
619502	occ	66742	Noetia (Eo	species	87629
619503	occ	66747	Noetia (Eo	species	87629
619504	occ	66744	Noetia (Eo	species	87629
619505	occ	66748	Noetia (Eo	species	87629
619506	occ	66749	Noetia (Eo	species	87629
619507	occ	66750	Noetia (Eo	species	87629
619508	occ	66751	Noetia (Eo	species	87629
619509	occ	66763	Noetia (Eo	species	87629
619510	occ	66754	Noetia (Eo	species	87629
642719	occ	69045	Noetia (No	species	90005
659738	occ	71138	Noetia car	species	16277
700941	occ	69043	Noetia lim	species	16277
700959	occ	69043	Arcopsis ac	species	90942
702881	occ	68979	Noetia sp.	genus	16277
702892	occ	69018	Noetia sp.	genus	16277
737396	occ	79248	Noetia sp.	genus	16277
737428	occ	79249	Noetia sp.	genus	16277
738539	occ	79340	Arcopsis ac	species	90942
748218	occ	80034	Noetia sp.	genus	16277
748368	occ	80033	Noetia sp.	genus	16277
748373	occ	80033	Arcopsis ac	species	90942
748506	occ	80033	Arcopsis cf	species	90942
748567	occ	80035	Noetia sp.	genus	16277
1512856	occ	211183	Noetia lim	species	16277

559164	occ	57705	Noetia (Eo	species	87629
559198	occ	57706	Noetia (Eo	species	87629
559218	occ	57702	Noetia (Eo	species	87629
727670	occ	78271	Noetia inci	species	116339
727693	occ	78272	Noetia inci	species	116339
727758	occ	78270	Noetia inci	species	116339
727787	occ	78269	Noetia inci	species	116339
727879	occ	78267	Noetia inci	species	116339
727900	occ	78266	Noetia inci	species	116339
727962	occ	78265	Noetia inci	species	116339
728028	occ	78261	Noetia inci	species	116339
728067	occ	78262	Noetia inci	species	116339
741430	occ	79709	Noetia inci	species	116339
748262	occ	80038	Noetia inci	species	116339
748406	occ	80036	Noetia inci	species	116339
749170	occ	80132	Noetia por	species	87629
568562	occ	59486	Pleurodon	species	136166
829782	occ	35025	Nucinella r	species	136167
425330	occ	41497	Nuculana a	species	87523
425444	occ	41501	Nuculana a	species	87523
427413	occ	41705	Nuculana a	species	87523
427723	occ	41718	Nuculana a	species	87523
428008	occ	41749	Nuculana a	species	87523
568552	occ	59487	Leda troch	species	66037
568553	occ	59484	Leda troch	species	66037
568554	occ	59486	Leda troch	species	66037
568555	occ	59490	Leda troch	species	66037
568556	occ	59491	Leda troch	species	66037
568557	occ	59485	Leda troch	species	66037
568558	occ	59485	Leda hamli	species	66037
582530	occ	61309	Nuculana a	species	87523
582550	occ	61310	Nuculana a	species	87523
582562	occ	61311	Nuculana a	species	87523
622908	occ	67069	Nuculana a	species	87523
623056	occ	67072	Nuculana s	genus	16083
623073	occ	67074	Nuculana s	genus	16083
623082	occ	67075	Nuculana s	genus	16083
623091	occ	67076	Nuculana s	genus	16083
697886	occ	74999	Nuculana c	species	87523
697966	occ	75012	Nuculana t	species	16083
697967	occ	75012	Nuculana a	species	87523
697997	occ	75015	Nuculana a	species	87523
738133	occ	79331	Nuculana (	species	109869
743040	occ	79797	Nuculana s	genus	16083
747500	occ	79787	Saccella ac	species	108618
747569	occ	80030	Saccella tr	species	16121
747570	occ	80030	Saccella pc	species	16121

747591	occ	80029	Saccella trc	species	16121
747592	occ	80029	Saccella pc	species	16121
751030	occ	80357	Nuculana a	species	87523
853726	occ	94809	Leda polyc	species	66037
1087875	occ	96538	Nuculana a	species	87523
578668	occ	60928	Nuculana a	species	87523
578879	occ	60940	Nuculana a	species	87523
579030	occ	60944	Nuculana a	species	87523
619463	occ	66758	Nuculana a	species	87523
642932	occ	69046	Nuculana a	species	87523
832949	occ	92409	Leda acuta	species	87522
832995	occ	92412	Leda troch	species	66037
1457455	occ	66757	Nucula acu	species	87521
1457490	occ	203378	Leda limula	species	66037
564601	occ	58893	Nuculana a	species	87523
564618	occ	58894	Nuculana a	species	87523
564639	occ	58895	Nuculana a	species	87523
564903	occ	58897	Nuculana a	species	87523
579566	occ	60986	Nuculana a	species	87523
579668	occ	60989	Nuculana a	species	87523
579733	occ	60988	Nuculana a	species	87523
579806	occ	60987	Nuculana a	species	87523
619464	occ	66752	Nuculana a	species	87523
619465	occ	66751	Nuculana a	species	87523
619466	occ	66750	Nuculana a	species	87523
640565	occ	69086	Saccella ac	species	108618
659706	occ	71138	Nuculana a	species	87523
700965	occ	69043	Saccella ac	species	108618
702861	occ	68979	Saccella ac	species	108618
738412	occ	79342	Saccella ac	species	108618
738452	occ	79343	Saccella ac	species	108618
738475	occ	79341	Saccella ac	species	108618
748384	occ	80033	Saccella sp	genus	16121
748498	occ	80033	Saccella sp	genus	16121
1512827	occ	211029	Nuculana a	species	87523
1512836	occ	211183	Nuculana a	species	87523
1512934	occ	211181	Nuculana a	species	87523
1512972	occ	211182	Nuculana a	species	87523
94777	occ	7109	Nuculana a	species	87523
559166	occ	57705	Nuculana a	species	87523
559200	occ	57706	Nuculana a	species	87523
559233	occ	57703	Nuculana a	species	87523
559247	occ	57704	Nuculana a	species	87523
582221	occ	61293	Nuculana a	species	87523
582242	occ	61294	Nuculana a	species	87523
582250	occ	61295	Nuculana a	species	87523
582261	occ	61296	Nuculana a	species	87523

727784	occ	78269	Nuculana a	species	87523
728065	occ	78262	Nuculana a	species	87523
362459	occ	34269	Nucula pro	species	87510
425329	occ	41497	Nucula pro	species	87510
425443	occ	41501	Nucula pro	species	87510
427412	occ	41705	Nucula pro	species	87510
427611	occ	41715	Nucula pro	species	87510
427722	occ	41718	Nucula pro	species	87510
427910	occ	41740	Nucula pro	species	87510
428007	occ	41749	Nucula pro	species	87510
568545	occ	59490	Nucula tap	species	98558
568546	occ	59487	Nucula tap	species	98558
568547	occ	59486	Nucula pro	species	87510
568548	occ	59485	Nucula pro	species	87510
568549	occ	59491	Nucula pro	species	87510
568550	occ	59484	Nucula pro	species	87510
568551	occ	59487	Nucula pro	species	87510
582529	occ	61309	Nucula pro	species	87510
582549	occ	61310	Nucula pro	species	87510
622907	occ	67069	Nucula pro	species	87510
697996	occ	75015	Nucula sp.	genus	16082
738130	occ	79331	Nucula pro	species	87510
745412	occ	79788	Nucula pro	species	87510
747275	occ	80027	Nucula pro	species	87510
747504	occ	79787	Nucula pro	species	87510
747646	occ	80037	Nucula tap	species	98558
751009	occ	80358	Nucula pro	species	87510
1087874	occ	96538	Nucula pro	species	87510
256377	occ	20604	Nucula pro	species	87510
256395	occ	20606	Nucula pro	species	87510
578604	occ	60927	Nucula pro	species	87510
578667	occ	60928	Nucula pro	species	87510
578847	occ	60931	Nucula pro	species	87510
578878	occ	60940	Nucula pro	species	87510
579029	occ	60944	Nucula pro	species	87510
619453	occ	66760	Nucula pro	species	87510
619454	occ	66756	Nucula pro	species	87510
642936	occ	69046	Nucula pro	species	87510
741373	occ	79705	Nucula pro	species	87510
741377	occ	79707	Nucula pro	species	87510
745470	occ	79956	Nucula pro	species	87510
745494	occ	79957	Nucula pro	species	87510
832919	occ	92408	Nucula pro	species	87510
832994	occ	92412	Nucula pro	species	87510
564307	occ	58893	Nucula pro	species	87510
564314	occ	58894	Nucula pro	species	87510
564345	occ	58895	Nucula pro	species	87510

564346	occ	58895	Nucula tap	species	98558
564394	occ	58898	Nucula pro	species	87510
564395	occ	58897	Nucula pro	species	87510
564396	occ	58897	Nucula tap	species	98558
566483	occ	59327	Nucula pro	species	87510
566484	occ	59310	Nucula pro	species	87510
566485	occ	59316	Nucula pro	species	87510
566486	occ	59317	Nucula pro	species	87510
566487	occ	59318	Nucula pro	species	87510
566488	occ	59313	Nucula pro	species	87510
566490	occ	59307	Nucula pro	species	87510
579567	occ	60986	Nucula pro	species	87510
579667	occ	60989	Nucula pro	species	87510
579732	occ	60988	Nucula pro	species	87510
579805	occ	60987	Nucula pro	species	87510
619455	occ	66752	Nucula pro	species	87510
619456	occ	66751	Nucula pro	species	87510
619457	occ	66750	Nucula pro	species	87510
619458	occ	66742	Nucula pro	species	87510
659709	occ	71138	Nucula pro	species	87510
738408	occ	79342	Nucula pro	species	87510
748509	occ	80033	Nucula pro	species	87510
748659	occ	80035	Nucula pro	species	87510
1512852	occ	211183	Nucula pro	species	16082
1512940	occ	211181	Nucula pro	species	87510
559165	occ	57705	Nucula pro	species	87510
559199	occ	57706	Nucula pro	species	87510
559219	occ	57702	Nucula pro	species	87510
559230	occ	57703	Nucula pro	species	87510
559246	occ	57704	Nucula pro	species	87510
582220	occ	61293	Nucula pro	species	87510
582241	occ	61294	Nucula pro	species	87510
582249	occ	61295	Nucula pro	species	87510
582260	occ	61296	Nucula pro	species	87510
727783	occ	78269	Nucula pro	species	87510
727869	occ	78267	Nucula pro	species	87510
727959	occ	78265	Nucula pro	species	87510
747722	occ	80048	Nucula pro	species	87510
749173	occ	80132	Nucula pro	species	87510
423300	occ	41199	Ostrea scu	species	109463
423301	occ	41199	Ostrea sp.	genus	16870
423371	occ	41205	Ostrea sp.	genus	16870
425305	occ	41496	Ostrea disp	species	16870
425306	occ	41496	Ostrea me	species	16870
425324	occ	41497	Crassostre	species	82699
425331	occ	41497	Ostrea scu	species	109463
425445	occ	41501	Ostrea scu	species	109463

427374	occ	41705	Crassostrea	species	82699
427414	occ	41705	Ostrea scu	species	109463
427707	occ	41718	Crassostrea	species	82699
427724	occ	41718	Ostrea scu	species	109463
427725	occ	41718	Ostrea sp.	genus	16870
427990	occ	41749	Crassostrea	species	82699
428009	occ	41749	Ostrea me	species	16870
428010	occ	41749	Ostrea scu	species	109463
428011	occ	41749	Ostrea sp.	genus	16870
533414	occ	20400	Ostrea sp.	genus	16870
568596	occ	59486	Ostrea disp	species	16870
568599	occ	59484	Ostrea disp	species	16870
568600	occ	59488	Ostrea disp	species	16870
568601	occ	59487	Ostrea disp	species	16870
582572	occ	61309	Crassostrea	species	82699
582573	occ	61309	Ostrea scu	species	109463
582587	occ	61310	Ostrea scu	species	109463
582597	occ	61311	Ostrea equ	species	101121
622933	occ	67069	Ostrea scu	species	109463
622934	occ	67069	Ostrea virg	species	82698
622935	occ	67069	Ostrea sp.	genus	16870
623059	occ	67072	Ostrea me	species	16870
623060	occ	67072	Ostrea disp	species	16870
623069	occ	67073	Ostrea me	species	16870
623076	occ	67074	Ostrea me	species	16870
623084	occ	67075	Ostrea disp	species	16870
623101	occ	67077	Ostrea me	species	16870
623102	occ	67077	Ostrea disp	species	16870
697889	occ	74999	Ostrea cf. s	species	109463
697926	occ	75003	Ostrea scu	species	109463
697933	occ	75010	Ostrea sp.	genus	16870
697941	occ	75004	Ostrea sp.	genus	16870
697951	occ	75011	Ostrea scu	species	109463
697973	occ	75012	Ostrea scu	species	16870
697991	occ	75005	Ostrea sp.	genus	16870
698039	occ	75017	Ostrea scu	species	109463
698061	occ	75022	Ostrea scu	species	109463
698067	occ	75023	Ostrea scu	species	109463
698090	occ	75025	Ostrea scu	species	109463
698099	occ	75026	Ostrea sp.	genus	16870
738131	occ	79334	Ostrea (Os	species	94289
738134	occ	79333	Ostrea (Ale	species	109464
738148	occ	79335	Ostrea (Os	species	94289
738151	occ	79335	Ostrea sp.	genus	16870
738155	occ	79335	Ostrea (Cra	species	109465
738169	occ	79331	Ostrea (Os	species	94289
738170	occ	79331	Ostrea (Ale	species	109464

738178	occ	79336	Ostrea (Os	species	94289
741761	occ	79731	Crassostre	species	82699
741986	occ	79748	Conradost	species	82701
742049	occ	79747	Conradost	species	118875
742428	occ	35026	Ostrea scu	species	109463
742429	occ	35026	Ostrea disp	species	16870
742430	occ	35026	Ostrea lock	species	16870
742431	occ	35026	Ostrea cox	species	16870
743045	occ	79797	Ostrea me	species	16870
743046	occ	79797	Ostrea disp	species	16870
743322	occ	79804	Crassostre	species	16578
743714	occ	79847	Conradost	species	16563
745338	occ	79789	Ostrea sub	species	109883
745421	occ	69040	Ostrea sp.	genus	16870
745536	occ	79961	Ostrea con	species	99271
747290	occ	69044	Ostrea sp.	genus	16870
747361	occ	69029	Crassostre	genus	16578
747432	occ	79786	Ostrea sub	species	109883
747482	occ	79787	Ostrea sub	species	109883
747527	occ	79786	Crassostre	species	82699
747617	occ	80037	Ostrea disp	species	16870
747618	occ	80037	Conradost	species	118875
747666	occ	80039	Ostrea disp	species	16870
747667	occ	80039	Conradost	species	118875
747752	occ	80046	Ostrea sp.	genus	16870
748123	occ	69034	Ostrea sp.	genus	16870
748124	occ	69034	Crassostre	species	82699
748291	occ	80025	Ostrea disp	species	16870
748322	occ	80024	Crassostre	species	82699
853729	occ	94809	Ostrea disp	species	16870
1011552	occ	123549	Ostrea scu	species	109463
1011553	occ	123549	Ostrea disp	species	16870
1087885	occ	96538	Crassostre	species	82699
1087886	occ	96538	Ostrea equ	species	101121
1317126	occ	176821	Ostrea sp.	genus	16870
618802	occ	66724	Crassostre	species	82699
618803	occ	66725	Crassostre	species	82699
618804	occ	66726	Crassostre	species	82699
618805	occ	66727	Crassostre	species	82699
578510	occ	60917	Ostrea scu	species	109463
578515	occ	60921	Ostrea scu	species	109463
578518	occ	60922	Ostrea scu	species	109463
578566	occ	60920	Ostrea disp	species	16870
578567	occ	60920	Ostrea scu	species	109463
578579	occ	60918	Ostrea scu	species	109463
578581	occ	60924	Crassostre	species	82699
578582	occ	60925	Crassostre	species	82699

578583	occ	60926	Crassostre	species	82699
578597	occ	60927	Crassostre	species	82699
578605	occ	60927	Ostrea scu	species	109463
578760	occ	60930	Crassostre	species	82699
578770	occ	60930	Ostrea scu	species	109463
578848	occ	60931	Ostrea disp	species	16870
578849	occ	60931	Ostrea scu	species	109463
578899	occ	60940	Ostrea con	species	16870
578900	occ	60940	Ostrea scu	species	109463
578901	occ	60940	Ostrea lock	species	16870
578937	occ	60939	Ostrea con	species	16870
579020	occ	60944	Crassostre	species	82699
579031	occ	60944	Ostrea scu	species	109463
619529	occ	66762	Crassostre	species	82699
619530	occ	66760	Crassostre	species	82699
619531	occ	66758	Crassostre	species	82699
619532	occ	66756	Crassostre	species	82699
642865	occ	69046	Conradostr	genus	16563
642927	occ	69046	Crassostre	species	82699
745487	occ	79957	Ostrea sp.	genus	16870
747838	occ	80053	Conradostr	genus	16563
748088	occ	80083	Crassostre	species	82699
832858	occ	92404	Ostrea virg	species	82698
832898	occ	92407	Ostrea virg	species	87556
832927	occ	92408	Ostrea virg	species	82698
832953	occ	92409	Ostrea scu	species	109463
832980	occ	92410	Ostrea scu	species	109463
832981	occ	92410	Ostrea sp.	genus	16870
832999	occ	92412	Ostrea sp.	genus	16870
833000	occ	92412	Ostrea disp	species	16870
1457451	occ	79950	Ostrea virg	species	87556
1457453	occ	79767	Ostrea virg	species	87556
564285	occ	58893	Conradostr	species	82701
564295	occ	58894	Conradostr	species	82701
564327	occ	58895	Conradostr	species	82701
564370	occ	58898	Conradostr	species	82701
564380	occ	58897	Crassostre	species	82699
566329	occ	59300	Ostrea con	species	99271
566332	occ	59301	Ostrea con	species	99271
566333	occ	59301	Conradostr	species	118875
578487	occ	60902	Ostrea disp	species	16870
578489	occ	60903	Ostrea disp	species	16870
579562	occ	60986	Ostrea scu	species	109463
579674	occ	60989	Ostrea scu	species	109463
579738	occ	60988	Ostrea scu	species	109463
579811	occ	60987	Ostrea scu	species	109463
619533	occ	66763	Crassostre	species	82699

619534	occ	66754	Crassostrea	species	82699
619535	occ	66751	Crassostrea	species	82699
619536	occ	66750	Crassostrea	species	82699
619537	occ	66749	Crassostrea	species	82699
619538	occ	66748	Crassostrea	species	82699
619539	occ	66747	Crassostrea	species	82699
619540	occ	66746	Crassostrea	species	82699
619541	occ	66742	Crassostrea	species	82699
640554	occ	69086	Ostrea disp	species	16870
640563	occ	69086	Conradostr	species	118875
642726	occ	69045	Crassostrea	species	82699
642736	occ	69045	Ostrea sp.	genus	16870
659743	occ	71138	Ostrea scu	species	109463
700970	occ	69043	Conradostr	genus	16563
701045	occ	69047	Crassostrea	species	82699
702876	occ	68979	Conradostr	genus	16563
702895	occ	69018	Conradostr	genus	16563
737387	occ	79248	Conradostr	species	118875
737404	occ	79249	Ostrea con	species	16870
738403	occ	79342	Conradostr	genus	16563
738433	occ	79343	Conradostr	genus	16563
738462	occ	79341	Conradostr	genus	16563
738497	occ	79339	Conradostr	genus	16563
738534	occ	79340	Conradostr	genus	16563
741429	occ	79708	Crassostrea	species	82699
748220	occ	80034	Ostrea sp.	genus	16870
748221	occ	80034	Crassostrea	species	82699
748449	occ	80033	Crassostrea	species	82699
748474	occ	80033	Ostrea sp.	genus	16870
748582	occ	80035	Crassostrea	species	82699
748583	occ	80035	"Ostrea" sp	genus	16870
1512828	occ	211029	Ostrea scu	species	109463
1512835	occ	211183	Ostrea scu	species	109463
1512935	occ	211181	Ostrea scu	species	109463
1512973	occ	211182	Ostrea scu	species	109463
94763	occ	7108	Crassostrea	species	109465
559148	occ	57705	Crassostrea	species	82699
559190	occ	57706	Crassostrea	species	82699
559209	occ	57702	Crassostrea	species	82699
559226	occ	57703	Crassostrea	species	82699
559241	occ	57704	Crassostrea	species	82699
566289	occ	59297	Ostrea con	species	99271
566303	occ	59298	Ostrea con	species	99271
566304	occ	59298	Conradostr	species	118875
576307	occ	60660	Ostrea scu	species	109463
640353	occ	69048	Crassostrea	species	82699
640370	occ	69049	Crassostrea	species	82699

664777	occ	71620	Ostrea con	species	99271
664790	occ	71621	Ostrea con	species	99271
664791	occ	71621	Conradostı	species	118875
664833	occ	71624	Ostrea con	species	99271
665350	occ	60660	Conradostı	species	118875
727682	occ	78271	Conradostı	species	118875
727697	occ	78272	Conradostı	species	118875
727729	occ	78273	Ostrea con	species	99271
727742	occ	78270	Conradostı	species	118875
727804	occ	78269	Conradostı	species	118875
727860	occ	78267	Conradostı	species	118875
727861	occ	78267	Ostrea rav	species	160470
727921	occ	78266	Conradostı	species	118875
727979	occ	78265	Conradostı	species	118875
727995	occ	78260	Conradostı	species	118875
728086	occ	78262	Conradostı	species	118875
728123	occ	78264	Conradostı	species	118875
737443	occ	79246	Conradostı	species	118875
737456	occ	79247	Conradostı	species	118875
737496	occ	79245	Conradostı	species	118875
741451	occ	79709	Conradostı	genus	16563
748182	occ	80040	Ostrea sp.	genus	16870
748275	occ	80038	Ostrea sp.	genus	16870
748276	occ	80038	Crassostre	species	82699
748420	occ	80036	"Ostrea" sı	genus	16870
427415	occ	41705	Pandora cf	species	98486
568629	occ	59486	Pandora (K	species	162224
632418	occ	67069	Pandora bı	species	18783
632419	occ	67069	Pandora tr	species	108687
632420	occ	67069	Pandora tı	species	18783
738212	occ	79331	Pandora (C	species	98487
747333	occ	69029	Pandora (C	subgenus	98485
747516	occ	79787	Pandora sı	genus	18783
579032	occ	60944	Pandora tr	species	108687
619552	occ	66760	Pandora tr	species	156303
619557	occ	66760	Pandora tr	species	108687
564619	occ	58894	Pandora tı	species	18783
564640	occ	58895	Pandora tı	species	18783
564904	occ	58897	Pandora tı	species	18783
566576	occ	59315	Pandora (K	species	375831
566577	occ	59316	Pandora (K	species	375831
566578	occ	59313	Pandora (C	species	98487
566579	occ	59313	Pandora (C	species	98485
619553	occ	66751	Pandora tr	species	156303
619554	occ	66752	Pandora tr	species	156303
619555	occ	66751	Pandora tr	species	108687
619556	occ	66752	Pandora tr	species	108687

619558	occ	66742	Pandora ar	species	162224
642725	occ	69045	Pandora (P	species	90384
659749	occ	71138	Pandora tu	species	18783
738419	occ	79342	Pandora sp	genus	18783
748501	occ	80033	Pandora (C	species	98485
748564	occ	80035	Pandora (C	species	98485
559167	occ	57705	Pandora gc	species	108685
559168	occ	57705	Pandora tr	species	108687
559201	occ	57706	Pandora gc	species	108685
559220	occ	57702	Pandora gc	species	108685
559221	occ	57702	Pandora tr	species	108687
727738	occ	78270	Pandora ar	species	162224
727800	occ	78269	Pandora cr	species	98486
727919	occ	78266	Pandora ar	species	162224
727983	occ	78265	Pandora ar	species	162224
728038	occ	78261	Pandora ar	species	162224
728082	occ	78262	Pandora cr	species	98486
728126	occ	78264	Pandora cr	species	98486
748206	occ	80040	Pandora (C	subgenus	18783
362483	occ	34269	Pecten gibl	species	109885
423303	occ	41199	Pecten (Pla	species	87088
423304	occ	41199	Pecten (No	species	102113
423305	occ	41199	Pecten (Pe	subgenus	85852
423337	occ	41202	Pecten (Pla	species	87088
423338	occ	41202	Pecten (No	species	102113
423339	occ	41202	Pecten (Lyı	species	416602
423381	occ	41205	Pecten (Pla	species	87088
423382	occ	41205	Pecten (No	species	102113
423383	occ	41205	Pecten (Pe	subgenus	85852
423393	occ	41206	Pecten (Pla	species	87088
423418	occ	41209	Pecten (Pla	species	87088
423419	occ	41209	Pecten (Lyı	species	416602
425303	occ	41496	Chlamys bu	species	16549
425386	occ	41501	Amusium r	species	95154
425410	occ	41501	Chlamys ar	species	156308
425411	occ	41501	Chlamys cf	species	95132
425412	occ	41501	Chlamys fu	species	109884
425414	occ	41501	Chlamys sp	genus	16549
425448	occ	41501	Pecten zicz	species	102590
426073	occ	41539	Amusium r	species	95154
426080	occ	41539	Chlamys sp	genus	16549
427363	occ	41705	Chlamys ar	species	156308
427364	occ	41705	Chlamys ca	species	109897
427365	occ	41705	Chlamys fu	species	109884
427366	occ	41705	Chlamys no	species	109895
427368	occ	41705	Chlamys sp	genus	16549
427605	occ	41715	Chlamys fu	species	109884

427606	occ	41715	Chlamys iri	species	156779
427680	occ	41718	Amusium r	species	95154
427704	occ	41718	Chlamys ar	species	156308
427705	occ	41718	Chlamys sp	genus	16549
427985	occ	41749	Chlamys cc	species	95132
427986	occ	41749	Chlamys iri	species	156779
427987	occ	41749	Chlamys je	species	95111
427989	occ	41749	Chlamys sp	genus	16549
428021	occ	41749	Pecten rav	species	95097
568605	occ	59489	Pecten (Pe	species	85852
568606	occ	59487	Chlamys (P	species	95127
568607	occ	59484	Chlamys (P	species	95127
568608	occ	59491	Chlamys (P	species	95127
568609	occ	59490	Chlamys (P	species	95127
568610	occ	59486	Chlamys (P	species	95127
568611	occ	59484	Chlamys (P	species	95132
568612	occ	59491	Chlamys (P	species	95132
568613	occ	59486	Chlamys (P	species	95132
568614	occ	59489	Chlamys (P	species	95132
568615	occ	59487	Chlamys (P	species	95132
568616	occ	59487	Amusium r	species	95154
581014	occ	61155	Pecten (Pe	species	85852
581015	occ	61155	Chlamys (P	species	95132
581016	occ	61155	Amusium r	species	95154
582575	occ	61309	Argopecte	genus	16490
582576	occ	61309	Argopecte	species	102541
582589	occ	61310	Aequipecte	genus	16446
582590	occ	61310	Argopecte	species	102541
591052	occ	62462	Pecten zicz	species	102590
622936	occ	67069	Pecten rav	species	95097
622937	occ	67069	Lyropecter	species	91151
622938	occ	67069	Lyropecter	species	91148
622939	occ	67069	Aequipecte	species	87554
622940	occ	67069	Aequipecte	species	90976
622941	occ	67069	Aequipecte	species	16446
622942	occ	67069	Aequipecte	species	16446
623061	occ	67072	Pecten (Pe	species	85852
623062	occ	67072	Pecten (Nc	species	109892
623063	occ	67072	Pecten (Ch	species	89174
623064	occ	67072	Pecten (Ch	species	82959
623070	occ	67073	Pecten (Ch	species	89174
623071	occ	67073	Pecten (Ch	species	82959
623077	occ	67074	Pecten (Ch	species	82959
623085	occ	67075	Pecten (Ch	species	82959
623093	occ	67076	Pecten (Ch	species	82959
623103	occ	67077	Pecten (Pe	species	85852
623104	occ	67077	Pecten ebc	species	82298

623105	occ	67077	Pecten cal	species	89174
640730	occ	69087	Argopecte	genus	16490
697901	occ	75009	Pecten sp.	genus	16904
697925	occ	75003	Pecten sp.	genus	16904
697972	occ	75012	Pecten cf.	species	82298
697985	occ	75014	Pecten ebo	species	82298
698008	occ	75016	Pecten cf.	species	82298
698017	occ	75006	Pecten (N	subgenus	16904
698043	occ	75018	Pecten cf.	species	82298
698044	occ	75019	Pecten sp.	genus	16904
698089	occ	75025	Pecten sp.	genus	16904
738140	occ	79333	Chlamys (P	species	109887
738142	occ	79333	Chlamys (P	species	95126
738144	occ	79334	Chlamys (P	species	95132
738145	occ	79333	Chlamys (P	species	95132
738146	occ	79333	Chlamys (P	species	95126
738152	occ	79337	Chlamys (P	species	95132
738153	occ	79338	Chlamys (P	species	109887
738156	occ	79335	Chlamys (P	species	95132
738171	occ	79331	Chlamys (C	species	109884
738172	occ	79331	Chlamys (P	species	109887
738179	occ	79336	Chlamys (C	species	109884
741769	occ	79731	Pecten zicz	species	102590
741876	occ	79738	Argopecte	species	102541
741877	occ	79738	Euvola zicz	species	87035
741940	occ	79740	Nodipecte	species	16853
741971	occ	79749	Carolinape	species	16539
741992	occ	79748	Carolinape	species	119966
742050	occ	79747	Carolinape	species	119961
742178	occ	79764	Aequipecte	species	95125
742434	occ	35026	Pecten (Pe	species	85852
742435	occ	35026	Aequipecte	species	93764
742436	occ	35026	Aequipecte	species	93764
742437	occ	35026	Nodipecte	species	16853
742438	occ	35026	Lyropecter	species	16786
743047	occ	79797	Pecten och	species	16904
743048	occ	79797	Pecten we	species	160239
743049	occ	79797	Chlamys ca	species	89174
743051	occ	79797	Chlamys no	species	109895
743319	occ	79804	Leptopecte	species	16762
743320	occ	79804	Lindapecte	species	97218
743321	occ	79804	Stralopecte	species	17052
743729	occ	79804	Argopecte	species	16490
743730	occ	79804	Carolinape	species	119966
744299	occ	79876	Carolinape	species	16539
744403	occ	79874	Nodipecte	species	16853
744404	occ	79874	Stralopecte	species	17052

745320	occ	79789	Carolinape	species	119966
745384	occ	79788	Carolinape	species	119966
745427	occ	69040	Carolinape	species	82694
745530	occ	79961	Argopecte	species	16490
745531	occ	79961	Carolinape	species	16539
745532	occ	79961	Chesapect	species	108717
745533	occ	79961	Chesapect	species	16548
745534	occ	79961	Euvola smi	species	99246
745535	occ	79961	Leptopect	species	16762
745540	occ	79962	Chesapect	species	108717
745556	occ	79960	Carolinape	species	16539
745557	occ	79960	Nodipecte	species	16853
745589	occ	79960	Euvola och	species	95095
745590	occ	79960	Argopecte	species	16490
745745	occ	79959	Nodipecte	species	16853
745756	occ	79959	Euvola her	species	95095
745757	occ	79959	Argopecte	species	16490
745758	occ	79959	Christinap	species	16551
745759	occ	79959	Carolinape	species	82694
745800	occ	79959	Carolinape	species	16539
747004	occ	80002	Chlamys cf	species	109158
747024	occ	80001	Chlamys sp	genus	16549
747389	occ	69029	Chlamys fu	species	109884
747466	occ	79786	Aequipect	genus	16446
747493	occ	79787	Argopecte	genus	16490
747513	occ	79787	Aequipect	genus	16446
747526	occ	79786	Amussium	genus	16466
747529	occ	79786	Argopecte	genus	16490
747615	occ	80037	Amusium r	species	59066
747630	occ	80037	Aequipect	species	16446
747661	occ	80039	Pecten och	species	16904
747706	occ	80044	Aequipect	species	16446
747768	occ	80046	Pecten zicz	species	102590
747770	occ	80046	Argopecte	genus	16490
748142	occ	69034	Aequipect	genus	16446
748288	occ	80025	Aequipect	species	16446
748310	occ	80025	Chlamys fu	species	109884
751000	occ	80359	Chlamys fu	species	109884
751033	occ	80357	Chlamys ar	species	156308
751049	occ	80355	Chlamys cf	species	109884
789714	occ	85448	Pecten sp.	genus	16904
789717	occ	85448	Pecten ebo	species	82298
834785	occ	92739	Pecten sp.	genus	16904
834791	occ	92740	Pecten ebo	species	82298
853732	occ	94809	Chlamys (P	species	95126
1011550	occ	123549	Chlamys (P	species	95127
1011551	occ	123549	Chlamys (P	species	95132

1067689	occ	35029	Spathochla	species	244803
1067690	occ	35026	Spathochla	species	244803
1068697	occ	79960	Nodipecter	species	16853
1068698	occ	35025	Nodipecter	species	16853
1068704	occ	35031	Antillipecte	species	244886
1087887	occ	96538	Argopecter	species	90997
1087888	occ	96538	Argopecter	species	102541
1087889	occ	96538	Euvola zicz	species	87035
1317140	occ	176822	Chesapecte	species	289066
1317141	occ	176822	Chesapecte	species	82646
1087106	occ	96681	Placopecte	species	82638
578509	occ	60917	Chlamys el	species	95127
578512	occ	60919	Chlamys el	species	95127
578558	occ	60923	Chlamys sp	genus	16549
578562	occ	60920	Amusium r	species	95154
578564	occ	60920	Chlamys el	species	95127
578565	occ	60920	Chlamys sp	genus	16549
578570	occ	60919	Chlamys sp	genus	16549
578576	occ	60918	Chlamys el	species	95127
578577	occ	60918	Chlamys sp	genus	16549
578651	occ	60928	Chlamys (A	species	109158
578755	occ	60930	Chlamys (A	species	109158
578794	occ	60932	Amusium r	species	95154
578795	occ	60932	Chlamys el	species	95127
578796	occ	60932	Chlamys sp	genus	16549
578844	occ	60931	Chlamys el	species	95127
578845	occ	60931	Chlamys sp	genus	16549
578853	occ	60933	Chesapecte	species	108717
578854	occ	60933	Leptopecte	species	16762
578855	occ	60933	Chesapecte	species	82646
578857	occ	60934	Chesapecte	species	289066
578858	occ	60935	Chesapecte	species	289066
578863	occ	60938	Pecten her	species	416604
578864	occ	60938	Chesapecte	species	289066
578865	occ	60941	Pecten her	species	416604
578867	occ	60940	Pecten her	species	416604
578868	occ	60940	Leptopecte	species	16762
578869	occ	60940	Chesapecte	species	82646
578870	occ	60940	Chesapecte	species	289066
578871	occ	60940	Nodipecter	species	16853
578873	occ	60942	Chesapecte	species	289066
578877	occ	60939	Pecten her	species	416604
578888	occ	60940	Pecten sp.	genus	16904
578889	occ	60940	Amusium r	species	95154
578890	occ	60940	Carolinape	species	82694
578891	occ	60940	Argopecter	species	16490
578897	occ	60940	Chlamys de	species	82640

578936	occ	60939	Carolinape	species	82694
578946	occ	60941	Amusium r	species	95154
590468	occ	62355	Argopecter	species	82299
619527	occ	66760	Aequipecte	species	87552
642871	occ	69046	Argopecter	species	90997
747818	occ	80053	Carolinape	species	82694
748084	occ	80083	Carolinape	species	82694
832928	occ	92408	Pecten ebc	species	82298
832954	occ	92409	Pecten ebc	species	82298
832969	occ	92411	Amusium r	species	95154
832970	occ	92411	Pecten ebc	species	82298
832978	occ	92410	Pecten her	species	416604
832979	occ	92410	Amusium r	species	95154
833001	occ	92412	Pecten jeff	species	82641
564305	occ	58893	Leptopecte	species	82693
564323	occ	58895	Carolinape	species	82694
564352	occ	58895	Leptopecte	species	82693
564362	occ	58897	Carolinape	species	82694
564368	occ	58898	Carolinape	species	82694
564388	occ	58898	Leptopecte	species	82693
564405	occ	58897	Leptopecte	species	82693
566326	occ	59300	Placopecte	species	95572
566328	occ	59300	Chesapecte	species	108717
566331	occ	59301	Chesapecte	species	82646
566535	occ	59310	Pecten (Eu	species	95098
566536	occ	59318	Pecten (Pla	species	95100
566537	occ	59317	Pecten (Pla	species	95100
566541	occ	59331	Chlamys (L	species	95112
566542	occ	59329	Chlamys (L	species	95112
566543	occ	59313	Chlamys (L	species	95124
566544	occ	59316	Chlamys (L	species	95124
566545	occ	59310	Chlamys (A	species	95129
566546	occ	59309	Chlamys (A	species	95129
566547	occ	59327	Chlamys (A	species	95129
566548	occ	59308	Chlamys (A	species	95129
566549	occ	59307	Chlamys (A	species	95129
566550	occ	59330	Chlamys (A	species	95129
566551	occ	59331	Chlamys (A	species	95129
566552	occ	59328	Chlamys (A	species	95129
566553	occ	59329	Chlamys (A	species	95129
566554	occ	59318	Chlamys (A	species	95129
566555	occ	59316	Chlamys (A	species	95129
566556	occ	59312	Chlamys (A	species	95129
566557	occ	59313	Chlamys (A	species	95129
566558	occ	59314	Chlamys (A	species	95129
566559	occ	59314	Amusium r	species	95154
566560	occ	59317	Amusium r	species	95154

566561	occ	59307	Amusium r	species	95154
578473	occ	60899	Chlamys er	species	95124
578483	occ	60900	Amusium r	species	95154
578486	occ	60902	Chlamys el	species	95127
578490	occ	60903	Chlamys el	species	95127
579563	occ	60986	Argopecter	species	82299
579673	occ	60989	Argopecter	species	82299
579737	occ	60988	Argopecter	species	82299
579810	occ	60987	Argopecter	species	82299
590475	occ	62357	Argopecter	species	82299
590476	occ	52582	Argopecter	species	82299
590477	occ	52582	Placopecte	species	95572
590488	occ	52582	Placopecte	species	82638
590489	occ	52582	Chlamys de	species	16549
590490	occ	52582	Chesapecte	species	108717
590491	occ	52582	Chesapecte	species	82646
619523	occ	66749	Aequipecte	species	87552
619524	occ	66747	Aequipecte	species	87552
619525	occ	66745	Aequipecte	species	87552
619526	occ	66742	Aequipecte	species	87552
642720	occ	69045	Argopecter	species	90997
659697	occ	71138	Argopecter	species	82299
700952	occ	69043	Aequipecte	species	95568
700955	occ	69043	Chlamys de	species	16549
701066	occ	69047	Argopecter	species	102541
702882	occ	68979	Aequipecte	species	95568
702897	occ	69018	Aequipecte	species	95568
737437	occ	79248	Aequipecte	species	95568
738545	occ	79340	Aequipecte	species	16446
741428	occ	79708	Carolinape	species	82694
748210	occ	80034	Carolinape	species	82694
748361	occ	80033	Amussium	species	95153
748476	occ	80033	Aequipecte	species	95568
748584	occ	80035	Amusium r	species	95154
748585	occ	80035	Argopecter	species	16490
748586	occ	80035	Chesapecte	species	108717
1173580	occ	151326	Carolinape	species	82694
559143	occ	57705	Argopecter	species	90997
559184	occ	57706	Argopecter	species	90997
566287	occ	59297	Chesapecte	species	108717
566288	occ	59297	Chesapecte	species	16548
566302	occ	59298	Chesapecte	species	82646
566307	occ	59293	Pecten clin	species	82303
566308	occ	59293	Pecten jeff	species	82641
566309	occ	59293	Pecten ebc	species	82298
566322	occ	59294	Pecten san	species	99250
566324	occ	59294	Pecten ma	species	82645

576306	occ	60660	Chesapect	species	82646
576542	occ	60667	Chesapect	species	82646
582258	occ	61296	Chesapect	species	82646
590466	occ	59155	Argopecte	species	82299
590474	occ	62356	Argopecte	species	82299
664775	occ	71620	Placopecte	species	95572
664776	occ	71620	Chesapect	species	108717
664789	occ	71621	Chesapect	species	82646
664830	occ	71624	Placopecte	species	95572
664831	occ	71624	Chesapect	species	108717
727696	occ	78272	Chesapect	species	82646
727728	occ	78273	Chesapect	species	108717
727743	occ	78270	Chesapect	species	16548
727841	occ	78268	Chesapect	species	82646
727862	occ	78267	Chesapect	species	82646
727916	occ	78266	Carolinape	species	82694
727922	occ	78266	Chesapect	species	82646
728087	occ	78262	Chesapect	species	82646
728122	occ	78264	Chesapect	species	82646
737445	occ	79246	Chesapect	species	108717
737450	occ	79247	Chesapect	species	108717
737485	occ	79247	Placopecte	species	95572
737486	occ	79247	Chlamys de	species	16549
741434	occ	79709	Chesapect	species	82646
741435	occ	79709	Chesapect	species	108717
741443	occ	79709	Placopecte	species	95572
748177	occ	80040	Chesapect	species	82646
748180	occ	80040	Chesapect	species	108717
748200	occ	80040	Chlamys de	species	16549
748201	occ	80040	Placopecte	species	95572
748202	occ	80040	Carolinape	species	82694
748248	occ	80038	Placopecte	species	95572
748264	occ	80038	Euvola sp.	genus	95095
748269	occ	80038	Chesapect	species	82648
748270	occ	80038	Chlamys de	species	16549
748271	occ	80038	Chesapect	species	82646
748272	occ	80038	Chesapect	species	108717
748418	occ	80036	Chesapect	species	108717
748419	occ	80036	Chesapect	species	16548
578669	occ	60928	Periploma	species	18791
564294	occ	58894	Cochlodesı	species	82736
559171	occ	57705	Periploma	species	156455
623009	occ	67069	Rupellaria	species	91181
738307	occ	79331	Petricola (I	species	158760
745944	occ	79958	Petricoxen	species	158757
835959	occ	92380	Petricola (I	species	158757
1150761	occ	146873	Rupellaria	species	18205

618993	occ	66724	Petricola p	species	61694
578670	occ	60928	Petricola p	species	61694
619678	occ	66760	Petricola p	species	61694
619679	occ	66758	Petricola p	species	61694
619680	occ	66755	Petricola p	species	61694
619682	occ	66760	Petricola la	species	156572
619683	occ	66760	Rupellaria	species	91181
564907	occ	58897	Petricola p	species	82728
566850	occ	59313	Petricola (I	species	85862
566851	occ	59318	Petricola (I	species	85862
566852	occ	59313	Petricola (I	species	61694
566853	occ	59316	Petricola (I	species	61694
566854	occ	59310	Petricola (I	species	99351
566855	occ	59309	Petricola (I	species	99351
619681	occ	66746	Petricola p	species	61694
559172	occ	57705	Petricola p	species	61694
559202	occ	57706	Petricola p	species	61694
559234	occ	57703	Petricola p	species	61694
727705	occ	78272	Pleiorytis c	species	18086
727733	occ	78273	Pleiorytis c	species	18086
727768	occ	78270	Pleiorytis c	species	18086
727910	occ	78266	Pleiorytis c	species	18086
728035	occ	78261	Pleiorytis c	species	18086
728075	occ	78262	Pleiorytis c	species	18086
582659	occ	61309	Ensis mino	species	158641
623045	occ	67069	Ensis mino	species	158641
745455	occ	69040	Ensis direc	species	94528
747635	occ	80037	Ensis direc	species	94528
578762	occ	60930	Ensis direc	species	94528
578921	occ	60940	Ensis direc	species	94528
578943	occ	60939	Ensis direc	species	94528
579024	occ	60944	Ensis direc	species	94528
619761	occ	66758	Ensis mino	species	158641
619762	occ	66760	Ensis mino	species	158641
619771	occ	66758	Ensis direc	species	94528
619772	occ	66760	Ensis direc	species	94528
642903	occ	69046	Ensis direc	species	94528
741383	occ	79707	Ensis direc	species	94528
741400	occ	79706	Ensis direc	species	94528
741406	occ	79705	Ensis direc	species	94528
745480	occ	79956	Ensis direc	species	94528
745502	occ	79957	Ensis direc	species	94528
747831	occ	80053	Ensis direc	species	94528
564301	occ	58893	Ensis direc	species	94528
564309	occ	58894	Ensis direc	species	94528
564337	occ	58895	Ensis direc	species	94528
564409	occ	58897	Ensis direc	species	94528

579742	occ	60988	Ensis direc	species	94528
619759	occ	66742	Siliqua cos	species	18250
619763	occ	66742	Ensis direc	species	94528
619764	occ	66746	Ensis direc	species	94528
619765	occ	66748	Ensis direc	species	94528
619766	occ	66749	Ensis direc	species	94528
619767	occ	66750	Ensis direc	species	94528
619768	occ	66751	Ensis direc	species	94528
619769	occ	66752	Ensis direc	species	94528
619770	occ	66754	Ensis direc	species	94528
659698	occ	71138	Ensis direc	species	94528
700950	occ	69043	Ensis direc	species	94528
701069	occ	69047	Ensis direc	species	94528
702866	occ	68979	Ensis direc	species	94528
702910	occ	69018	Ensis sp.	genus	17612
737423	occ	79248	Ensis direc	species	94528
737429	occ	79249	Ensis direc	species	94528
738401	occ	79342	Ensis direc	species	94528
738431	occ	79343	Ensis direc	species	94528
738479	occ	79341	Ensis direc	species	94528
738512	occ	79339	Ensis direc	species	94528
738526	occ	79340	Ensis direc	species	94528
748239	occ	80034	Ensis direc	species	94528
748574	occ	80035	Ensis veriti	species	17612
829366	occ	91459	Ensis direc	species	94528
829371	occ	91462	Ensis direc	species	94528
94759	occ	7108	Ensis direc	species	94528
559155	occ	57705	Ensis direc	species	94528
559194	occ	57706	Ensis direc	species	94528
559212	occ	57702	Ensis direc	species	94528
640369	occ	69049	Ensis direc	species	94528
727971	occ	78265	Ensis ensif	species	98319
728034	occ	78261	Ensis ensif	species	98319
728074	occ	78262	Ensis ensif	species	98319
749169	occ	80132	Ensis direc	species	94528
632417	occ	67069	Diplothyra	genus	158643
738370	occ	79331	Barnea (Sc	species	251665
743721	occ	79804	Cyrtopleur	species	158642
747329	occ	69029	Cyrtopleur	species	158642
1088921	occ	96538	Cyrtopleur	species	158642
1088922	occ	96538	Martesia s	species	61061
578930	occ	60940	Teredina fi	species	18508
578949	occ	60941	Teredina fi	species	18508
619794	occ	66760	Barnea cos	species	18431
619795	occ	66756	Barnea cos	species	18431
619796	occ	66755	Barnea cos	species	18431
619800	occ	66760	Pholas can	species	109672

619801	occ	66760	Pholas trur	species	108683
619802	occ	66760	Martesia c	species	392371
619803	occ	66758	Martesia c	species	392371
642929	occ	69046	Pholas can	species	109672
832884	occ	92407	Pholas turg	species	18491
564334	occ	58895	Cyrtopleur	genus	18448
566927	occ	59316	Barnea (Sc	species	18431
566928	occ	59317	Barnea (Sc	species	18431
566929	occ	59329	Barnea (Sc	species	18431
619797	occ	66750	Barnea cos	species	18431
619798	occ	66746	Barnea cos	species	18431
619799	occ	66742	Barnea cos	species	18431
829367	occ	91460	Cyrtopleur	genus	18448
94756	occ	7108	Barnea cos	species	18431
557481	occ	57702	Barnea tru	species	156793
559144	occ	57705	Barnea tru	species	156793
559186	occ	57706	Barnea tru	species	156793
559227	occ	57703	Cyrtopleur	species	158642
727903	occ	78266	Cyrtopleur	species	18448
743318	occ	79804	Arcoptera	species	383263
423336	occ	41202	Pinna sp.	genus	16400
423391	occ	41206	Pinna sp.	genus	16400
425397	occ	41501	Atrina calo	species	16342
426078	occ	41539	Atrina calo	species	16342
568597	occ	59484	Atrina n. sp	genus	16342
582570	occ	61309	Atrina sp.	genus	16342
582584	occ	61310	Atrina sp.	genus	16342
622931	occ	67069	Pinna sp.	genus	16400
698038	occ	75008	Atrina sp.	genus	16342
738166	occ	79331	Pinna sp.	genus	16400
748323	occ	80024	Pinna sp.	genus	16400
619520	occ	66758	Atrina rigic	species	16342
619521	occ	66760	Atrina rigic	species	16342
619522	occ	66760	Atrina serr	species	158634
832863	occ	92405	Pinna semi	species	157898
1457464	occ	203376	Pinna semi	species	157898
748353	occ	80033	Atrina sp.	genus	16342
748510	occ	80033	Atrina sp.	genus	16342
362484	occ	34269	Plicatula rc	species	16936
423306	occ	41199	Plicatula m	species	109467
423341	occ	41202	Plicatula m	species	109467
423384	occ	41205	Plicatula m	species	109467
425455	occ	41501	Plicatula m	species	109467
427423	occ	41705	Plicatula m	species	109467
427424	occ	41705	Plicatula sp	species	16936
427614	occ	41715	Plicatula m	species	109467
427741	occ	41718	Plicatula m	species	109467

427913	occ	41740	Plicatula sp	genus	16936
568617	occ	59484	Plicatula m	species	109467
568618	occ	59487	Plicatula m	species	109467
568619	occ	59491	Plicatula m	species	109467
568620	occ	59486	Plicatula m	species	109467
582574	occ	61309	Plicatula cf	species	87555
582588	occ	61310	Plicatula cf	species	87555
582598	occ	61311	Plicatula cf	species	87555
622948	occ	67069	Plicatula gi	species	91000
640743	occ	69087	Plicatula gi	species	87555
738202	occ	79331	Plicatula cf	species	109467
747289	occ	69044	Plicatula m	species	109467
747322	occ	69029	Plicatula m	species	109467
747456	occ	79786	Plicatula m	species	109467
747601	occ	80037	Plicatula m	species	109467
747665	occ	80039	Plicatula m	species	109467
747701	occ	80044	Plicatula m	species	109467
747748	occ	80046	Plicatula m	species	109467
748324	occ	80024	Plicatula m	species	109467
1011554	occ	123549	Plicatula m	species	109467
1087890	occ	96538	Plicatula gi	species	87555
578568	occ	60920	Plicatula m	species	109467
578571	occ	60919	Plicatula m	species	109467
578608	occ	60927	Plicatula m	species	109467
578850	occ	60931	Plicatula m	species	109467
578896	occ	60940	Plicatula m	species	109467
619528	occ	66760	Plicatula gi	species	87555
642868	occ	69046	Plicatula gi	species	87555
747819	occ	80053	Plicatula m	species	109467
748089	occ	80083	Plicatula m	species	109467
832929	occ	92408	Plicatula sp	genus	16936
832982	occ	92410	Plicatula m	species	109467
833002	occ	92412	Plicatula m	species	109467
564605	occ	58893	Plicatula m	species	109467
564622	occ	58894	Plicatula m	species	109467
564646	occ	58895	Plicatula m	species	109467
566562	occ	59328	Plicatula m	species	109467
566563	occ	59329	Plicatula m	species	109467
566564	occ	59330	Plicatula m	species	109467
566565	occ	59327	Plicatula m	species	109467
566566	occ	59308	Plicatula m	species	109467
566567	occ	59307	Plicatula m	species	109467
566568	occ	59309	Plicatula m	species	109467
566569	occ	59310	Plicatula m	species	109467
566570	occ	59317	Plicatula m	species	109467
566571	occ	59316	Plicatula m	species	109467
566572	occ	59318	Plicatula m	species	109467

578475	occ	60899	Plicatula m	species	109467
640558	occ	69086	Plicatula m	species	109467
659747	occ	71138	Plicatula m	species	109467
737382	occ	68979	Plicatula m	species	109467
737388	occ	79248	Plicatula m	species	109467
737400	occ	79249	Plicatula m	species	109467
738411	occ	79342	Plicatula m	species	109467
738447	occ	79343	Plicatula m	species	109467
738500	occ	79339	Plicatula m	species	109467
738535	occ	79340	Plicatula m	species	109467
741416	occ	79708	Plicatula m	species	109467
748215	occ	80034	Plicatula m	species	109467
748401	occ	80033	Plicatula m	species	109467
748505	occ	80033	Plicatula m	species	109467
748581	occ	80035	Plicatula m	species	109467
1173579	occ	151326	Plicatula m	species	109467
727737	occ	78270	Plicatula m	species	109467
727799	occ	78269	Plicatula m	species	109467
727854	occ	78267	Plicatula m	species	109467
727917	occ	78266	Plicatula m	species	109467
727977	occ	78265	Plicatula m	species	109467
728081	occ	78262	Plicatula m	species	109467
748188	occ	80040	Plicatula m	species	109467
748281	occ	80038	Plicatula m	species	109467
738213	occ	79331	Poromya fl	species	18808
745329	occ	79789	Poromya fl	species	18808
568769	occ	59486	Asaphis ce	species	98347
748396	occ	80033	Gari (Gobr	subgenus	161374
737455	occ	79247	Gari sp.	genus	17675
737474	occ	79246	Gari sp.	genus	17675
741461	occ	79709	Psammobi	family	61938
425456	occ	41501	Pteria coly	species	90967
568598	occ	59487	Pteria mult	species	16981
582596	occ	61311	Pteria coly	species	90967
622929	occ	67069	Pteria coly	species	90967
622930	occ	67069	Pinctada ra	species	90970
640772	occ	69087	Pinctada sp	genus	16921
738150	occ	79338	Pteria coly	species	90967
738167	occ	79331	Pteria coly	species	90967
747515	occ	79787	Pteria coly	species	90967
747807	occ	80046	Pteria coly	species	90967
1087884	occ	96538	Pteria coly	species	90967
578887	occ	60940	Pteria coly	species	90967
619518	occ	66758	Pteria coly	species	90967
619519	occ	66760	Pteria coly	species	90967
748657	occ	80035	Pteria sp.	genus	16981
727980	occ	78265	Pteria coly	species	90967

362516	occ	34269	Semele prc	species	109796
362517	occ	34269	Semele bel	species	101157
425385	occ	41501	Abra aequa	species	101161
425418	occ	41501	Cumingia a	species	17537
425425	occ	41501	Ervilia polit	species	77232
425459	occ	41501	Semele bel	species	101157
427341	occ	41705	Abra aequa	species	101161
427376	occ	41705	Cumingia a	species	17537
427425	occ	41705	Semele bel	species	101157
427426	occ	41705	Semele pe	species	161493
427607	occ	41715	Ervilia polit	species	77232
427901	occ	41740	Abra aequa	species	101161
428029	occ	41749	Semele bel	species	101157
568760	occ	59487	Semele alu	species	18239
568761	occ	59487	Semele car	species	98350
568762	occ	59487	Semele bel	species	101157
568763	occ	59487	Semele pu	species	109781
568764	occ	59484	Semele prc	species	109796
568765	occ	59486	Semele (Se	species	18239
568766	occ	59487	Abra subre	species	17298
568767	occ	59484	Abra aequa	species	101161
568768	occ	59486	Abra aequa	species	101161
568780	occ	59484	Ervilia lata	species	77232
568781	occ	59486	Ervilia lata	species	77232
582668	occ	61309	Abra aequa	species	101161
582669	occ	61309	Semele bel	species	101157
582670	occ	61309	Semele pe	species	161493
582671	occ	61309	Semele prc	species	109796
582693	occ	61310	Cumingia s	genus	17537
582694	occ	61310	Semele bel	species	101157
622884	occ	67070	Semele pe	species	161493
623038	occ	67069	Semele prc	species	109796
623039	occ	67069	Semele pu	species	109781
623040	occ	67069	Semele pe	species	161493
623041	occ	67069	Semele bel	species	101157
623042	occ	67069	Semele nu	species	18239
623043	occ	67069	Cumingia t	species	94394
623044	occ	67069	Abra aequa	species	101161
632414	occ	67069	Ervilia conc	species	77232
697990	occ	75005	Abra aequa	species	101161
738325	occ	79336	Semele pu	species	109781
738326	occ	79336	Cumingia a	species	17537
738343	occ	79331	Semele pe	species	161493
738344	occ	79331	Semele pu	species	109781
738345	occ	79331	Semele ebl	species	18239
738346	occ	79331	Semelina n	species	18240
738347	occ	79331	Abra aequa	species	101161

738348	occ		79331	Cumingia c	species	249439
738359	occ		79331	Ervilia conc	species	109782
738360	occ		79331	Ervilia lata	species	77232
738361	occ		79331	Ervilia polit	species	77232
742053	occ		79747	Semele aff	species	161493
747532	occ		79786	Semele bel	species	101157
747639	occ		80037	Abra subre	species	17298
747641	occ		80037	Semele alu	species	18239
747669	occ		80039	Semele alu	species	18239
747707	occ		80044	Semele alu	species	18239
747803	occ		80046	Semele bel	species	101157
747814	occ		80046	Abra aequa	species	101161
748148	occ		69034	Abra aequa	species	101161
748164	occ		69034	Semele bel	species	101157
1087939	occ		96538	Abra aequa	species	101161
1087940	occ		96538	Cumingia t	species	94394
1087941	occ		96538	Ervilia polit	species	77232
1087942	occ		96538	Semele bel	species	101157
1087943	occ		96538	Semele pro	species	109796
256368	occ		20604	Abra aequa	species	101161
256386	occ		20606	Abra aequa	species	101161
578561	occ	18761	60923	Ervilia conc	species	109782
578584	occ		60927	Abra aequa	species	101161
578646	occ		60928	Abra aequa	species	101161
578656	occ	18762	60928	Ervilia conc	species	109782
578672	occ		60928	Semele pu	species	18239
578730	occ	18763	60929	Ervilia conc	species	109782
578763	occ	18764	60930	Ervilia conc	species	109782
578772	occ		60930	Semele pro	species	109796
578923	occ		60940	Semele car	species	98350
579025	occ	18765	60944	Ervilia lata	species	77232
619709	occ		66758	Abra angul	species	392355
619712	occ		66760	Semele bel	species	101157
619714	occ		66760	Abra aequa	species	101161
619715	occ		66758	Semele bel	species	101157
619716	occ		66756	Semele bel	species	101157
619718	occ		66760	Semele pro	species	109796
619719	occ		66758	Semele pro	species	109796
619720	occ		66756	Semele pro	species	109796
619722	occ		66760	Cumingia t	species	94394
619723	occ		66758	Cumingia t	species	94394
619784	occ	18767	66758	Ervilia conc	species	109782
619785	occ	18768	66760	Ervilia conc	species	109782
619786	occ	18769	66760	Ervilia polit	species	77232
619788	occ		66757	Mesodesm	species	91254
642869	occ		69046	Semele pa	species	18239
642878	occ		69046	Cumingia t	species	94394

642901	occ	69046	Semele bel	species	101157
642933	occ	69046	Abra aequa	species	101161
642947	occ	69046	Semele sp.	genus	18239
745459	occ	79956	Semele pu	species	109781
745469	occ	79956	Abra aequa	species	101161
745471	occ	79956	Semele prc	species	109796
745472	occ	79956	Cumingia t	species	94394
745484	occ	79957	Semele pu	species	109781
745489	occ	79957	Abra aequa	species	101161
745496	occ	79957	Cumingia t	species	94394
748102	occ	80083	Abra aequa	species	101161
833024	occ	92412	Semele sul	species	98354
1457474	occ	203376	Abra angul	species	392355
1457475	occ	203376	Semele tra	species	392359
564280	occ	58893	Abra aequa	species	101161
564315	occ	58895	Abra aequa	species	101161
564332	occ	58895	Cumingia t	species	17537
564353	occ	58897	Abra aequa	species	101161
564382	occ	58897	Cumingia t	species	94394
564607	occ	58893	Semele bel	species	101157
564624	occ	58894	Semele bel	species	101157
564896	occ	58898	Semele bel	species	101157
566734	occ	59310	Semele sul	species	98354
566735	occ	59329	Semele sul	species	98354
566736	occ	59318	Semele sul	species	98354
566737	occ	59316	Semele bel	species	101157
566738	occ	59313	Semele bel	species	101157
566739	occ	59315	Semele bel	species	101157
566740	occ	59318	Semele (Se	species	18239
566741	occ	59313	Semele (Se	species	18239
566742	occ	59317	Semele (Se	species	18239
566743	occ	59327	Semele (Se	species	18239
566744	occ	59307	Semele (Se	species	18239
566745	occ	59307	Abra aequa	species	101161
566746	occ	59330	Abra aequa	species	101161
566747	occ	59329	Abra aequa	species	101161
566748	occ	59309	Abra aequa	species	101161
566749	occ	59310	Abra aequa	species	101161
566750	occ	59313	Abra aequa	species	101161
566751	occ	59317	Abra aequa	species	101161
566752	occ	59316	Abra aequa	species	101161
566753	occ	59315	Abra aequa	species	101161
566847	occ	59307	Ervilia lata	species	77232
566848	occ	59315	Ervilia lata	species	77232
566849	occ	59316	Ervilia lata	species	77232
579606	occ	60986	Cumingia t	species	94394
579607	occ	60986	Abra aequa	species	101161

579681	occ	60989	Cumingia t	species	94394
579682	occ	60989	Abra aequa	species	101161
579762	occ	60988	Cumingia t	species	94394
579763	occ	60988	Abra aequa	species	101161
579815	occ	60987	Cumingia t	species	94394
579816	occ	60987	Abra aequa	species	101161
619710	occ	66751	Semele bel	species	101157
619711	occ	66752	Abra aequa	species	101161
619713	occ	66751	Abra aequa	species	101161
619717	occ	66742	Semele bel	species	101157
619721	occ	66742	Semele pro	species	109796
642727	occ	69045	Abra aequa	species	101161
642731	occ	69045	Ervilia sp.	genus	77232
659688	occ	71138	Cumingia t	species	94394
659740	occ	71138	Abra aequa	species	101161
659748	occ	71138	Abra subre	species	17298
700945	occ	69043	Cumingia t	species	94394
701050	occ	69047	Cumingia t	species	94394
702879	occ	68979	Semele sul	species	98354
702888	occ	69018	Semele (Se	subgenus	101158
737379	occ	68979	Ervilia lata	species	77232
737385	occ	79248	Semele sul	species	98354
737391	occ	79249	Semele sul	species	98354
738423	occ	79342	Semele (Se	species	18239
738424	occ	79342	Ervilia lata	species	77232
738450	occ	79343	Ervilia lata	species	77232
738481	occ	79341	Semele (Se	species	18239
738504	occ	79339	Semele (Se	species	101159
738525	occ	79340	Semele (Se	species	101159
748379	occ	80033	Abra (Abra	species	101161
748495	occ	80033	Abra aequa	species	101161
748520	occ	80033	Semele bel	species	101157
748526	occ	80033	Ervilia sp.	genus	77232
748565	occ	80035	Ervilia sp.	genus	77232
748655	occ	80035	Semele bel	species	101157
748660	occ	80035	Ervilia sp.	genus	77232
1512839	occ	211183	Abra aequa	species	101161
1512844	occ	211183	Cumingia t	species	94394
1512982	occ	211182	Abra aequa	species	101161
94761	occ	7108	Abra aequa	species	101161
557392	occ	57704	Abra aequa	species	101161
559140	occ	57705	Abra aequa	species	101161
559149	occ	57705	Cumingia t	species	94394
559175	occ	57705	Semele cf.	species	109781
559210	occ	57702	Cumingia t	species	94394
640359	occ	69048	Cumingia t	species	94394
727767	occ	78270	Cumingia t	species	94394

727792	occ	78269	Cumingia t	species	94394
727970	occ	78265	Semele sul	species	98354
728121	occ	78264	Semele sul	species	98354
741472	occ	79709	Semele sul	species	98354
748429	occ	80036	Semele bel	species	101157
425460	occ	41501	Solecurtus	species	18263
427427	occ	41705	Solecurtus	species	18263
568770	occ	59486	Tagelus (M	species	161437
582666	occ	61309	Solecurtus	species	18263
623036	occ	67069	Solecurtus	species	18263
623037	occ	67069	Solecurtus	species	18263
640751	occ	69087	Tagelus sp.	genus	18299
738314	occ	79335	Solecurtus	species	18263
738319	occ	79333	Solecurtus	species	18263
738320	occ	79333	Tagelus (M	species	161437
738327	occ	79336	Solecurtus	species	18263
738349	occ	79331	Solecurtus	species	18263
738350	occ	79331	Tagelus (M	species	161437
619754	occ	66760	Tagelus gib	species	108175
619755	occ	66757	Tagelus gib	species	108175
619756	occ	66756	Tagelus gib	species	108175
619757	occ	66755	Tagelus gib	species	108175
642948	occ	69046	Tagelus (Ta	species	108173
832885	occ	92407	Solecurtus	species	157297
566769	occ	59316	Tagelus gib	species	108175
566770	occ	59313	Tagelus gib	species	108175
566771	occ	59318	Tagelus gib	species	108175
619752	occ	66744	Tagelus gib	species	108175
619753	occ	66742	Tagelus gib	species	108175
700948	occ	69043	Tagelus ple	species	108173
702919	occ	69018	Tagelus sp.	genus	18299
741425	occ	79708	Tagelus ple	species	108173
748381	occ	80033	Tagelus car	species	18299
559238	occ	57703	Tagelus ple	species	108173
559251	occ	57704	Tagelus ple	species	108173
737454	occ	79247	Tagelus sp.	genus	18299
748274	occ	80038	Tagelus sp.	genus	18299
559176	occ	57705	Solemya ve	species	136155
619760	occ	66760	Solen viridi	species	18264
832972	occ	92411	Solen ensis	species	143258
566772	occ	59313	Solen viridi	species	18264
748388	occ	80033	Solen viridi	species	18264
582229	occ	61293	Solen sp.	genus	18264
582264	occ	61296	Solen sp.	genus	18264
1087917	occ	96538	Musculium	species	251736
1607539	occ	227396	Pisidium sp	genus	61772
1607541	occ	227396	Musculium	genus	251736

915138	occ	103991	Sphaerium	species	62302
915139	occ	103991	Pisidium d	species	61772
423340	occ	41202	Spondylus	genus	17044
423420	occ	41209	Spondylus	genus	17044
425461	occ	41501	Spondylus	species	109898
427428	occ	41705	Spondylus	species	109898
622947	occ	67069	Spondylus	species	90965
738196	occ	79335	Spondylus	species	109898
738201	occ	79331	Spondylus	species	109898
742433	occ	35026	Spondylus	species	86295
747767	occ	80046	Spondylus	genus	17044
1150759	occ	146873	Spondylus	species	86295
578895	occ	60940	Spondylus	genus	17044
425424	occ	41501	Ensitellops	species	17614
568700	occ	59484	Sportella c	species	18277
568701	occ	59484	Sportella p	species	18277
622975	occ	67069	Basterotia	genus	17399
622978	occ	67069	Fabella dal	species	17647
738256	occ	79338	Anisodonta	species	17338
738267	occ	79336	Ensitellops	species	17614
738268	occ	79336	Ensitellops	species	156639
738278	occ	79331	Fabella dal	species	17647
738279	occ	79331	Fabella nav	species	17647
738280	occ	79331	Ensitellops	species	17614
738282	occ	79331	Basterotia	species	158523
738283	occ	79331	Anisodonta	species	17338
1087916	occ	96538	Ensitellops	species	144830
578674	occ	60928	Sportella s	genus	18277
619610	occ	66758	Sportella p	species	18277
619611	occ	66760	Sportella c	species	18277
619612	occ	66760	Sportella p	species	18277
619615	occ	66760	Anisodonta	species	156314
642935	occ	69046	Basterotia	genus	17399
741393	occ	79706	Sportella s	genus	18277
564282	occ	58893	Anisodonta	species	146437
564338	occ	58895	Ensitellops	species	17614
564356	occ	58897	Anisodonta	species	146437
564364	occ	58898	Anisodonta	species	146437
564410	occ	58897	Ensitellops	species	17614
566671	occ	59313	Sportella c	species	18277
566672	occ	59307	Sportella c	species	18277
566673	occ	59313	Sportella c	species	18277
566674	occ	59310	Sportella c	species	18277
566675	occ	59313	Sportella g	species	18277
566676	occ	59316	Sportella g	species	18277
566677	occ	59316	Sportella w	species	18277
566678	occ	59313	Sportella w	species	18277

566679	occ	59316	Sportella c	species	18277
566680	occ	59313	Sportella c	species	18277
579561	occ	60986	Ensitellops	species	17614
579678	occ	60989	Ensitellops	species	17614
579739	occ	60988	Ensitellops	species	17614
579813	occ	60987	Ensitellops	species	17614
619613	occ	66752	Anisodonta	species	156314
619614	occ	66751	Anisodonta	species	156314
659707	occ	71138	Fabella cal	species	17647
659721	occ	71138	Ensitellops	species	17614
582257	occ	61296	Anisodonta	genus	17338
362514	occ	34269	Strigilla fle:	species	136203
362515	occ	34269	Strigilla sp.	genus	18287
423344	occ	41202	Metis mag	species	118881
425334	occ	41497	Tellina sp.	genus	18314
425335	occ	41497	Tellina tayl	species	102580
425463	occ	41501	Tellidora lu	species	392351
425464	occ	41501	Tellina cf. a	species	102570
425465	occ	41501	Tellina alte	species	102568
425466	occ	41501	Tellina call	species	18314
425984	occ	41501	Tellina sim	species	94613
425985	occ	41501	Tellina sub	species	18314
425986	occ	41501	Tellina sp.	species	18314
425987	occ	41501	Tellina sp.	species	18314
425988	occ	41501	Tellina sp.	species	18314
425989	occ	41501	Tellina sp.	species	18314
425990	occ	41501	Tellina sp.	genus	18314
427406	occ	41705	Macoma sp	species	17852
427429	occ	41705	Tellidora lu	species	392351
427430	occ	41705	Tellina cf. a	species	102570
427431	occ	41705	Tellina sp.	genus	18314
427432	occ	41705	Tellina tayl	species	102580
427744	occ	41718	Tellidora lu	species	392351
427745	occ	41718	Tellina sp.	species	18314
427746	occ	41718	Tellina tayl	species	102580
427850	occ	41718	Tellina sp.	species	18314
427915	occ	41740	Tellidora cı	species	91170
427916	occ	41740	Tellina sayi	species	95184
427997	occ	41749	Hemimetis	species	60619
428030	occ	41749	Tellidora lu	species	392351
428031	occ	41749	Tellina cf. c	species	18314
428032	occ	41749	Tellina cf. s	species	95184
428033	occ	41749	Tellina sp.	genus	18314
568747	occ	59484	Tellina (Eu	species	161299
568748	occ	59486	Tellina (Me	species	95004
568749	occ	59486	Tellina (Mc	species	95189
568750	occ	59486	Tellina (Mc	species	95185

568751	occ	59484	Tellina (Mo	species	95185
568752	occ	59484	Metis mag	species	118881
568753	occ	59487	Macoma v	species	95195
568754	occ	59485	Macoma v	species	95195
568755	occ	59486	Macoma v	species	95195
568756	occ	59491	Macoma (I	species	94746
568757	occ	59484	Macoma (I	species	94746
568758	occ	59486	Macoma (I	species	94746
568759	occ	59487	Macoma (I	species	94746
582660	occ	61309	Psammotr	species	156514
582661	occ	61309	Tellidora c	species	91170
582662	occ	61309	Tellina sp.	genus	18314
582663	occ	61309	Tellina alte	species	102568
582664	occ	61309	Tellina ma	species	102558
582665	occ	61309	Tellina nite	species	91154
582687	occ	61310	Psammotr	species	156514
582688	occ	61310	Strigilla mi	species	136201
582689	occ	61310	Tellidora c	species	91170
582690	occ	61310	Tellina sp.	genus	18314
582691	occ	61310	Tellina aeq	species	95003
582714	occ	61311	Tellidora c	species	91170
582715	occ	61311	Tellina sp.	genus	18314
623016	occ	67069	Tellina sim	species	94613
623017	occ	67069	Tellina cf. r	species	102556
623018	occ	67069	Tellina sub	species	18314
623019	occ	67069	Tellina line	species	102562
623021	occ	67069	Tellina aeq	species	95003
623022	occ	67069	Tellina ver	species	102578
623023	occ	67069	Tellina sp.	genus	18314
623024	occ	67069	Tellina gou	species	102566
623025	occ	67069	Arcopagia	species	109136
623026	occ	67069	Strigilla cf.	species	136201
623027	occ	67069	Macoma b	species	17852
623028	occ	67069	Macoma c	species	17852
623029	occ	67069	Macoma t	species	158646
623030	occ	67069	Macoma c	species	116271
623031	occ	67069	Macoma t	species	161340
623032	occ	67069	Macoma o	species	156831
623033	occ	67069	Apolymeti	species	90891
623034	occ	67069	Tellidora c	species	91170
623107	occ	67077	Metis bipli	species	98368
640519	occ	69085	Moerella s	genus	17928
640522	occ	69085	Eurytellina	genus	17645
640539	occ	69084	Moerella s	genus	17928
640731	occ	69087	Macoma s	genus	17852
640732	occ	69087	Angulus sp	genus	17335
640737	occ	69087	Macoma (I	subgenus	95033

640746	occ	69087	Tellidora cı	species	91170
640764	occ	69087	Macoma (I	subgenus	95033
640765	occ	69087	Angulus sp	genus	17335
640767	occ	69087	Angulus sp	genus	17335
640770	occ	69087	Scissula sir	species	94614
647835	occ	70010	Tellinella s	genus	18315
647844	occ	70010	Eurytellina	genus	17645
697993	occ	75005	Tellina sp.	genus	18314
738261	occ	79338	Tellina (Sci	species	18228
738266	occ	79333	Tellina (Eu	species	161295
738288	occ	79336	Tellina (Eu	species	161299
738291	occ	79336	Tellina (Mc	species	95014
738294	occ	79336	Tellina (Sci	species	18228
738309	occ	79331	Tellina (Eu	species	91190
738310	occ	79331	Tellina (Mc	species	102578
738311	occ	79331	Tellina (Mc	species	95014
738312	occ	79331	Tellina (Sci	species	94613
738313	occ	79338	Tellidora lu	species	392351
738318	occ	79333	Tellidora lu	species	392351
738323	occ	79336	Hemimetis	species	161356
738324	occ	79336	Tellidora lu	species	392351
738339	occ	79331	Tellina (Ac	species	18314
738340	occ	79331	Hemimetis	species	118882
738341	occ	79331	Strigilla fle	species	136203
738342	occ	79331	Tellidora lu	species	392351
745325	occ	79789	Moerella s	genus	17928
745327	occ	79789	Macoma s	genus	17852
745354	occ	79789	Tellidora lu	species	392351
745374	occ	79788	Tellidora lu	species	392351
745406	occ	79788	Moerella s	genus	17928
745415	occ	79788	Macoma s	genus	17852
745429	occ	69040	Angulus sp	genus	17335
745445	occ	69038	Eurytellina	genus	17645
745446	occ	69040	Eurytellina	genus	17645
747009	occ	80002	Tellina sp.	genus	18314
747033	occ	80001	Tellina sp.	genus	18314
747265	occ	80027	Eurytellina	genus	17645
747266	occ	80027	Moerella s	genus	17928
747303	occ	69044	Tellina (Tel	subgenus	18314
747314	occ	69044	Tellinidae i	family	62468
747354	occ	69029	Moerella s	genus	17928
747360	occ	69029	Scissula ca	species	95012
747452	occ	79786	Moerella s	genus	17928
747453	occ	79786	Tellidora lu	species	392351
747488	occ	79787	Tellidora lu	species	392351
747520	occ	79787	Macoma s	genus	17852
747572	occ	80030	Macoma v	species	17852

747575	occ	80030	Moerella s	genus	17928
747579	occ	80030	Tellidora lu	species	392351
747587	occ	80029	Moerella s	genus	17928
747594	occ	80029	Macoma v	species	17852
747743	occ	80046	Moerella s	genus	17928
748157	occ	69034	Moerella s	genus	17928
834613	occ	92369	Arcopagia	species	158308
834614	occ	92621	Arcopagia	species	158308
834615	occ	92622	Arcopagia	species	158308
834616	occ	92333	Arcopagia	species	158308
834617	occ	92623	Arcopagia	species	158308
834618	occ	92334	Arcopagia	species	158308
834619	occ	92362	Arcopagia	species	158308
834620	occ	92363	Arcopagia	species	158308
834621	occ	92624	Arcopagia	species	158308
1087935	occ	96538	Angulus te	species	102574
1087938	occ	96538	Tellidora cı	species	91170
578553	occ	60923	Tellina texa	species	102573
578610	occ	60927	Tellina texa	species	102573
578662	occ	60928	Macoma sp	genus	17852
578675	occ	60928	Tellidora cı	species	91170
578676	occ	60928	Tellina alte	species	102568
578677	occ	60928	Tellina texa	species	102573
578736	occ	60929	Tellina texa	species	102573
578922	occ	60940	Hemimetis	species	60619
578944	occ	60939	Macoma v	species	95195
579040	occ	60944	Tellina cf. t	species	102573
619684	occ	66760	Tellina alte	species	102568
619685	occ	66758	Tellina alte	species	102568
619686	occ	66757	Tellina alte	species	102568
619687	occ	66756	Tellina alte	species	102568
619691	occ	66760	Tellina texa	species	102573
619692	occ	66758	Tellina texa	species	102573
619699	occ	66755	Macoma b	species	107606
619700	occ	66755	Macoma ca	species	98000
619701	occ	66755	Macoma co	species	116271
619702	occ	66758	Strigilla mi	species	136201
619703	occ	66760	Macoma b	species	107606
619704	occ	66760	Macoma te	species	158646
619705	occ	66760	Strigilla mi	species	136201
619706	occ	66760	Tellidora cı	species	91170
619707	occ	66758	Tellidora cı	species	91170
642880	occ	69046	Angulus sp	genus	17335
642881	occ	69046	Angulus sp	genus	17335
741379	occ	79705	Angulus te	species	102574
741380	occ	79706	Angulus te	species	102574
741386	occ	79707	Angulus te	species	102574

741387	occ	79705	Macoma sp	genus	17852
741388	occ	79706	Macoma sp	genus	17852
741394	occ	79707	Macoma sp	genus	17852
741403	occ	79707	Scissula sp	genus	95012
745500	occ	79957	Tellinidae i	family	62468
747841	occ	80053	Moerella s	genus	17928
748098	occ	80083	Moerella s	genus	17928
748113	occ	80083	Macoma sp	genus	17852
833022	occ	92412	Tellina (Me	species	95004
833023	occ	92412	Tellina (An	species	95182
1457472	occ	203376	Peronaeod	species	392347
564608	occ	58893	Tellina agil	species	102570
564650	occ	58895	Tellina agil	species	102570
564901	occ	58898	Tellina agil	species	102570
564915	occ	58897	Tellina agil	species	102570
566714	occ	59313	Tellina (Mo	species	95185
566715	occ	59316	Tellina (Mo	species	95185
566716	occ	59327	Tellina (Mo	species	95187
566717	occ	59327	Tellina sp.	genus	18314
566718	occ	59330	Tellina (Mo	species	95187
566719	occ	59309	Tellina (Mo	species	95187
566720	occ	59307	Tellina (Mo	species	95187
566721	occ	59312	Tellina (Mo	species	95187
566722	occ	59310	Tellina (Mo	species	95187
566723	occ	59316	Tellina (Mo	species	95187
566724	occ	59313	Tellina (Mo	species	95187
566725	occ	59317	Tellina (Mo	species	95187
566726	occ	59315	Tellina (Mo	species	95187
566727	occ	59318	Tellina (Mo	species	95187
566728	occ	59329	Tellina (Mo	species	95189
566729	occ	59309	Tellina (Mo	species	95189
566730	occ	59307	Tellina (Mo	species	95189
566731	occ	59313	Tellina (Mo	species	95189
566732	occ	59310	Tellina (Mo	species	95189
566733	occ	59309	Macoma ca	species	95199
579680	occ	60989	Tellina aff.	species	102573
619688	occ	66742	Tellina alte	species	102568
619689	occ	66744	Tellina agil	species	102570
619690	occ	66742	Tellina agil	species	102570
619693	occ	66752	Tellina texa	species	102573
619694	occ	66751	Tellina texa	species	102573
619695	occ	66749	Tellina texa	species	102573
619708	occ	66742	Tellidora cr	species	91170
659750	occ	71138	Tellina agil	species	102570
700946	occ	69043	Strigilla (St	subgenus	161310
700968	occ	69043	Eurytellina	genus	17645
700969	occ	69043	Moerella n	species	95582

701067	occ		69047	Psammaco	subgenus	94615
702855	occ		68979	Moerella s	genus	17928
702862	occ		68979	Moerella d	species	95583
702863	occ		68979	Moerella n	species	95582
702886	occ		69018	Moerella d	species	95583
702887	occ		69018	Moerella n	species	95582
737380	occ		68979	Tellinidae i	family	62468
738453	occ		79343	Moerella s	genus	17928
738482	occ		79341	Moerella s	genus	17928
741423	occ		79708	Angulus sp	genus	17335
748439	occ		80033	Tellinidae i	family	62468
748441	occ		80033	Strigilla sp.	genus	18287
748486	occ		80033	Psammaco	genus	18126
748528	occ		80033	Moerella s	genus	17928
748529	occ		80033	Macoma sp	genus	17852
748662	occ		80035	Strigilla sp.	genus	18287
94760	occ	19409	7108	Tellina agil	species	102570
559178	occ		57705	Tellina agil	species	102570
559179	occ		57705	Tellina texa	species	102573
559205	occ		57706	Tellina texa	species	102573
559223	occ		57702	Tellina texa	species	102573
559242	occ		57704	Macoma b	species	107606
559243	occ		57704	Macoma c	species	116271
582228	occ		61293	Tellina sp.	genus	18314
582244	occ		61294	Tellina sp.	genus	18314
582255	occ		61295	Tellina sp.	genus	18314
582265	occ		61296	Tellina sp.	genus	18314
727765	occ		78270	Tellina pro	species	18314
727766	occ		78270	Tellina dec	species	95181
727791	occ		78269	Tellina sp.	genus	18314
727872	occ		78267	Tellina sp.	genus	18314
727873	occ		78267	Tellina dec	species	95181
727969	occ		78265	Tellina pro	species	18314
728120	occ		78264	Tellina sp.	genus	18314
741462	occ		79709	Moerella d	species	95584
741475	occ		79709	Tellinidae i	family	62468
747724	occ		80048	Macoma ca	species	98000
747738	occ		80047	Macoma te	species	158646
748263	occ		80038	Moerella s	genus	17928
748436	occ		80036	Tellinidae i	family	62468
745434	occ		69040	Teredinida	family	62496
747341	occ		69029	Teredinida	family	62496
833028	occ		92412	Teredo cal	species	18510
566930	occ		59331	Kuphus cal	species	98761
566298	occ		59297	Kuphus fist	species	99358
566316	occ		59293	Kuphus cal	species	98761
664786	occ		71620	Kuphus fist	species	99358

664842	occ	71624	Kuphus fist	species	99358
727672	occ	78271	Kuphus fist	species	99358
727838	occ	78268	Kuphus fist	species	99358
728031	occ	78261	Teredo fist	species	99357
728070	occ	78262	Kuphus fist	species	99358
741447	occ	79709	Kuphus fist	species	99358
748207	occ	80040	Kuphus cal	species	98761
748246	occ	80038	Kuphus cal	species	98761
423342	occ	41202	Thracia (Cy	species	114420
623106	occ	67077	Thracia sp.	genus	18836
623112	occ	67078	Thracia sp.	genus	18836
632421	occ	67069	Cyathodon	species	18728
743055	occ	79797	Thracia (Cy	subgenus	114420
578932	occ	60940	Thracia ma	species	18836
578933	occ	60940	Cyathodon	species	18728
564609	occ	58893	Thracia bri	species	82737
564651	occ	58895	Thracia bri	species	82737
564916	occ	58897	Thracia bri	species	82737
566574	occ	59310	Thracia co	species	108651
566575	occ	59316	Thracia tra	species	18836
737495	occ	79245	Thracia sp.	genus	18836
741463	occ	79709	"Thracia" s	genus	18836
622972	occ	67069	Thyasira tr	species	18336
640735	occ	69087	Thyasira sp	genus	18336
623010	occ	67069	Coralliopha	species	59853
425420	occ	41501	Diplodonta	species	98441
427378	occ	41705	Diplodonta	species	98441
427991	occ	41749	Diplodonta	species	98441
568696	occ	59484	Diplodonta	species	98441
568697	occ	59484	Diplodonta	species	17578
568698	occ	59486	Diplodonta	species	17578
568699	occ	59487	Diplodonta	species	17578
582609	occ	61309	Diplodonta	genus	17578
582654	occ	61311	Diplodonta	genus	17578
622969	occ	67069	Diplodonta	species	17578
622970	occ	67069	Diplodonta	species	147914
622971	occ	67069	Diplodonta	species	135918
640543	occ	69084	Diplodonta	species	90985
640739	occ	69087	Diplodonta	subgenus	94616
738246	occ	79331	Taras (Tara	species	100560
738274	occ	79331	Taras (Phly	species	146393
745326	occ	79789	Diplodonta	species	98441
745407	occ	79788	Diplodonta	species	17578
745544	occ	79962	Diplodonta	species	98445
747574	occ	80030	Diplodonta	genus	17578
853743	occ	94809	Diplodonta	species	98445
1087908	occ	96538	Diplodonta	species	90985

1087909	occ	96538	Phlyctiderr	species	147915
1317136	occ	176823	Diplodonta	species	98445
578909	occ	60940	Diplodonta	species	17578
579022	occ	60944	Diplodonta	species	98441
619579	occ	66758	Diplodonta	species	147914
619580	occ	66760	Diplodonta	species	146392
619581	occ	66758	Diplodonta	species	146392
642951	occ	69046	Diplodonta	species	90985
741357	occ	79707	Phlyctiderr	species	146393
741361	occ	79705	Phlyctiderr	species	146393
741364	occ	79706	Phlyctiderr	species	146393
745503	occ	79957	Diplodonta	species	90985
748091	occ	80083	Diplodonta	species	98441
833015	occ	92412	Diplodonta	species	135918
833016	occ	92412	Diplodonta	species	17578
564300	occ	58893	Diplodonta	species	98441
564335	occ	58895	Diplodonta	species	98441
564383	occ	58898	Diplodonta	species	17578
564384	occ	58898	Diplodonta	species	98441
564407	occ	58897	Diplodonta	species	98441
564908	occ	58897	Phlyctiderr	species	82709
566656	occ	59316	Diplodonta	species	17578
566657	occ	59313	Diplodonta	species	17578
566658	occ	59310	Diplodonta	species	17578
566659	occ	59307	Diplodonta	species	146392
566660	occ	59316	Diplodonta	species	146392
579676	occ	60989	Diplodonta	genus	17578
619577	occ	66752	Diplodonta	species	90985
619578	occ	66751	Diplodonta	species	90985
700943	occ	69043	Diplodonta	species	98441
701068	occ	69047	Diplodonta	species	98441
702893	occ	69018	Diplodonta	subgenus	94616
702894	occ	69018	Diplodonta	species	98441
738492	occ	79339	Diplodonta	species	98441
738547	occ	79340	Diplodonta	species	98441
748222	occ	80034	Phlyctiderr	species	146393
748354	occ	80033	Phlyctiderr	species	146393
748445	occ	80033	Diplodonta	genus	17578
748497	occ	80033	Diplodonta	species	17578
748571	occ	80035	Phlyctiderr	genus	18068
559151	occ	57705	Diplodonta	species	90985
727735	occ	78270	Diplodonta	species	98441
727797	occ	78269	Diplodonta	species	17578
727914	occ	78266	Diplodonta	species	98441
741473	occ	79709	Diplodonta	species	98441
748433	occ	80036	Diplodonta	subgenus	94616
745369	occ	79788	Anodonta	subgenus	83658

362501	occ	34269	Dosinia dis	species	17595
362502	occ	34269	Dosinia sp.	genus	17595
362503	occ	34269	Chione (Lir	species	145058
362504	occ	34269	Chione car	species	94441
362505	occ	34269	Chione inte	species	17468
362506	occ	34269	Chione (Tir	species	95522
362507	occ	34269	Chione sp.	genus	17468
362508	occ	34269	Anomaloca	species	94549
362509	occ	34269	Venus sp.	genus	18391
362510	occ	34269	Venus mor	species	102577
362511	occ	34269	Venus cam	species	94395
362513	occ	34269	Transenne	species	162009
423307	occ	41199	Chione inta	species	108667
423343	occ	41202	Chione inta	species	108667
423387	occ	41205	Chione inta	species	108667
423397	occ	41206	Chione inta	species	108667
423398	occ	41206	Chione car	species	94441
423422	occ	41209	Chione car	species	94441
423423	occ	41209	Venus ? sp	genus	18391
425304	occ	41496	Mercenaria	species	94396
425321	occ	41497	Chione car	species	94441
425322	occ	41497	Chione gru	species	248728
425325	occ	41497	Dosinia ele	species	94559
425327	occ	41497	Gouldia cf.	species	17708
425368	occ	41498	Chione car	species	94441
425381	occ	41498	Anomaloca	species	17341
425394	occ	41501	Anomaloca	species	17341
425408	occ	41501	Chione car	species	94441
425409	occ	41501	Chione gru	species	248728
425422	occ	41501	Dosinia ele	species	94559
425428	occ	41501	Gouldia cf.	species	101136
425429	occ	41501	Gouldia flo	species	17708
425430	occ	41501	Gouldia me	species	17708
425431	occ	41501	Gouldia sp	genus	17708
425436	occ	41501	Macrocallis	species	161775
425437	occ	41501	Macrocallis	species	109469
425438	occ	41501	Mercenaria	species	94396
425447	occ	41501	Parastarte	species	162147
425454	occ	41501	Pitar cf. sa	species	146429
425994	occ	41501	Transenne	species	162010
426075	occ	41539	Anomaloca	species	17341
426082	occ	41539	Dosinia dis	species	94592
426087	occ	41539	Parastarte	species	162147
426090	occ	41539	Pitar cf. sa	species	146429
426091	occ	41539	Transenne	species	162010
426114	occ	41539	Chione car	species	94441
427348	occ	41705	Anomaloca	species	17341

427359	occ	41705	Chione car	species	94441
427360	occ	41705	Chione gru	species	248728
427361	occ	41705	Chione lati	species	98378
427362	occ	41705	Chione mo	species	17468
427380	occ	41705	Dosinia ele	species	94559
427386	occ	41705	Gouldia cf.	species	101136
427407	occ	41705	Macrocallis	species	161775
427408	occ	41705	Macrocallis	species	109469
427409	occ	41705	Mercenaria	species	94396
427416	occ	41705	Parastarte	species	162147
427422	occ	41705	Pitar cf. sa	species	146429
427604	occ	41715	Chione car	species	94441
427688	occ	41718	Anomaloca	species	17341
427701	occ	41718	Chione car	species	94441
427702	occ	41718	Chione gru	species	248728
427703	occ	41718	Chione lati	species	98378
427710	occ	41718	Dosinia ele	species	94559
427717	occ	41718	Mercenaria	species	94396
427727	occ	41718	Parastarte	species	162147
427740	occ	41718	Pitar cf. sa	species	146429
427748	occ	41718	Transenne	species	162010
427904	occ	41740	Anomaloca	species	17341
427906	occ	41740	Chione car	species	94441
427908	occ	41740	Macrocallis	species	109469
427911	occ	41740	Parastarte	species	162147
427918	occ	41740	Transenne	species	162010
427919	occ	41740	Transenne	species	94533
427920	occ	41740	Venus cam	species	94395
427969	occ	41749	Anomaloca	species	17341
427981	occ	41749	Chione car	species	94441
427982	occ	41749	Chione crib	species	17468
427983	occ	41749	Chione lati	species	98378
427984	occ	41749	Chione cf.	species	248724
427993	occ	41749	Dosinia ele	species	94559
428000	occ	41749	Macrocallis	genus	17856
428001	occ	41749	Macrocallis	species	109469
428002	occ	41749	Mercenaria	species	94396
428020	occ	41749	Parastarte	species	162147
428036	occ	41749	Transenne	genus	162009
533409	occ	20400	Chione car	species	94441
568711	occ	59486	Dosinia (Do	species	95242
568712	occ	59491	Dosinia (Do	species	95243
568713	occ	59484	Dosinia (Do	species	95243
568714	occ	59484	Transenne	species	162009
568715	occ	59485	Transenne	species	162009
568716	occ	59487	Gafrarium	species	100932
568717	occ	59484	Gafrarium	species	100932

568718	occ	59491	Gafrarium	species	100932
568719	occ	59486	Gafrarium	species	100932
568720	occ	59486	Macrocalli:	species	95248
568721	occ	59484	Macrocalli:	species	95248
568722	occ	59485	Callocardia	species	95264
568723	occ	59487	Callocardia	species	95264
568724	occ	59486	Cyclinella c	species	158650
568725	occ	59485	Cyclinella c	species	158650
568726	occ	59484	Chione (Ch	species	95479
568727	occ	59484	Chione (Ch	species	95479
568728	occ	59485	Chione (Ch	species	95479
568729	occ	59487	Chione (Ch	species	95479
568730	occ	59487	Chione (Ch	species	95484
568731	occ	59487	Chione (Lir	species	92348
568732	occ	59487	Chione (Lir	species	161769
568733	occ	59484	Chione (Lir	species	161769
568734	occ	59486	Chione (Lir	species	161769
568735	occ	59491	Chione (Lir	species	161769
568736	occ	59484	Chione (Lir	species	145058
568737	occ	59485	Chione (Lir	subgenus	92348
568738	occ	59487	Chione (Lir	subgenus	92348
568739	occ	59484	Chione (Tir	species	95522
568740	occ	59486	Venus cam	species	94395
568741	occ	59484	Venus cam	species	94395
568742	occ	59485	Venus cam	species	94395
568743	occ	59487	Venus trida	species	95542
568744	occ	59487	Gemma ma	species	95553
568746	occ	59484	Parastarte	species	162147
582672	occ	61309	Anomaloca	species	146388
582673	occ	61309	Anomaloca	species	101147
582674	occ	61309	Chione car	species	94441
582675	occ	61309	Chione gru	species	248728
582676	occ	61309	Dosinia ele	species	94559
582677	occ	61309	Macrocalli:	species	161775
582678	occ	61309	Macrocalli:	species	109469
582679	occ	61309	Mercenari	species	94396
582680	occ	61309	Pitar sp.	genus	18075
582681	occ	61309	Pitar simps	species	146434
582695	occ	61310	Anomaloca	species	146388
582696	occ	61310	Chione car	species	94441
582697	occ	61310	Chione gru	species	248728
582698	occ	61310	Dosinia ele	species	94559
582699	occ	61310	Gouldia ce	species	101136
582700	occ	61310	Macrocalli:	species	109469
582701	occ	61310	Mercenari	species	94396
582702	occ	61310	Parastarte	species	162147
582703	occ	61310	Pitar simps	species	146434

582704	occ	61310	Transenne	species	162009
582717	occ	61311	Anomaloca	genus	17341
582718	occ	61311	Chione car	species	94441
582719	occ	61311	Chione gru	species	248728
582720	occ	61311	Dosinia ele	species	94559
582721	occ	61311	Macrocallis	species	109469
582722	occ	61311	Mercenaria	species	94396
582723	occ	61311	Parastarte	species	162147
582724	occ	61311	Pitar simps	species	146434
587890	occ	61924	Mercenaria	species	17892
587891	occ	61924	Mclelannia	species	461847
622991	occ	67069	Antigona li	species	17345
622992	occ	67069	Antigona r	species	416608
622993	occ	67069	Chione inta	species	108667
622994	occ	67069	Chione car	species	94441
622995	occ	67069	Chione lati	species	98378
622996	occ	67069	Chione gru	species	248728
622997	occ	67069	Anomaloca	species	17341
622998	occ	67069	Anomaloca	species	101147
622999	occ	67069	Pitar corda	species	146428
623000	occ	67069	Pitar sayan	species	146429
623001	occ	67069	Pitar (Hyph	subgenus	90367
623002	occ	67069	Gouldia ce	species	101136
623003	occ	67069	Macrocallis	species	161775
623004	occ	67069	Mercenaria	species	94396
623005	occ	67069	Transenne	species	135798
623006	occ	67069	Transenne	species	162010
623007	occ	67069	Dosinia ele	species	94559
623008	occ	67069	Cyclinella t	species	158650
623065	occ	67072	Chione ulo	species	248724
623078	occ	67074	Chione ulo	species	248724
623079	occ	67074	Dosinia ele	species	94559
623094	occ	67076	Chione ulo	species	248724
623108	occ	67077	Chione lati	species	98378
623109	occ	67077	Chione ulo	species	248724
623113	occ	67078	Chione ulo	species	248724
640509	occ	69085	Chione car	species	94441
640510	occ	69085	Transenne	species	94533
640512	occ	69085	Dosinia ele	species	94559
640525	occ	69085	Anomaloca	species	146388
640528	occ	69084	Chione car	species	94441
640529	occ	69084	Transenne	species	94533
640550	occ	69084	Anomaloca	species	146388
640552	occ	69084	Gemma ge	species	108168
640553	occ	69084	Dosinia ele	species	94559
640722	occ	69087	Chione car	species	94441
640725	occ	69087	Dosinia (Do	species	94592

640727	occ	69087	Pitar sp.	genus	18075
640728	occ	69087	Anomaloca	species	94549
640729	occ	69087	Transenne	species	94533
647825	occ	70010	Chione car	species	94441
647830	occ	70010	Anomaloca	species	146388
647834	occ	70010	Pitar simps	species	146434
647837	occ	70010	Transenne	species	94533
697892	occ	74999	Gemma m	species	95553
697893	occ	74999	Venus sp.	genus	18391
697894	occ	74999	Chione car	species	94441
697902	occ	75009	Chione (Lir	species	145058
697903	occ	75009	Chione car	species	94441
697916	occ	75001	Chione cf.	species	95487
697917	occ	75001	Chione car	species	94441
697921	occ	75002	Chione car	species	94441
697928	occ	75003	Dosinia sp.	genus	17595
697943	occ	75004	Chione car	species	94441
697954	occ	75011	Chione car	species	94441
697974	occ	75012	Dosinia sp.	genus	17595
697975	occ	75012	Chione car	species	94441
697981	occ	75013	Chione car	species	94441
697988	occ	75014	Chione car	species	94441
698000	occ	75015	Chione sp.	genus	17468
698010	occ	75016	Gemma m	species	95553
698011	occ	75016	Chione (At	subgenus	17468
698012	occ	75016	Chione (Lir	species	145058
698013	occ	75016	Chione car	species	94441
698020	occ	75006	Venus sp.	genus	18391
698021	occ	75006	Chione sp.	genus	17468
698022	occ	75006	Parastarte	genus	18033
698026	occ	75007	Chione sp.	genus	17468
698033	occ	75008	Parastarte	genus	18033
698036	occ	75008	Chione car	species	94441
698041	occ	75017	Chione sp.	genus	17468
698048	occ	75019	Transenella	genus	91157
698049	occ	75019	Chione sp.	genus	17468
698053	occ	75020	Transenella	genus	91157
698055	occ	75021	Parastarte	genus	18033
698062	occ	75022	Transenella	genus	91157
698070	occ	75023	Macrocallis	genus	17856
698071	occ	75023	Transenella	genus	91157
698076	occ	75024	Transenella	genus	91157
698092	occ	75025	Transenella	genus	91157
738258	occ	79338	Gemma m	species	95553
738260	occ	79338	Gemma (P	species	162147
738265	occ	79333	Chione (Ch	species	109475
738272	occ	79336	Chione (Ch	species	109475

738285	occ	79336	Anomaloca	species	17341
738287	occ	79336	Anomaloca	species	17341
738295	occ	79331	Mercenari	species	108656
738296	occ	79331	Macrocallis	species	109471
738297	occ	79331	Macrocallis	species	109338
738298	occ	79331	Pitar (Pitar	species	146429
738299	occ	79331	Antigona (\	species	156716
738300	occ	79331	Chione (Ch	species	109474
738301	occ	79331	Chione (Tir	species	95522
738302	occ	79331	Anomaloca	species	17341
738303	occ	79331	Transenne	species	162010
738304	occ	79331	Dosinia (Do	species	95243
738305	occ	79331	Gouldia flo	species	17708
738306	occ	79331	Gemma ma	species	95553
741760	occ	79731	Chione car	species	94441
741766	occ	79731	Mercenari	species	108656
741879	occ	79738	Mercenari	species	94396
741913	occ	79739	Anomaloca	species	17341
741914	occ	79739	Chione ele	species	146243
741915	occ	79739	Mercenari	species	94396
741920	occ	79739	Anomaloca	species	17341
741972	occ	79749	Mercenari	species	82725
742054	occ	79747	Chione ele	species	146243
742190	occ	79761	Macrocallis	species	95248
742497	occ	79761	Chione ero	species	17468
743058	occ	79797	Chione ulo	species	248724
743059	occ	79797	Chione lati	species	98378
743716	occ	79847	Anomaloca	genus	17341
745319	occ	79789	Chione car	species	94441
745321	occ	79789	Mercenari	species	108656
745328	occ	79789	Pitar morrl	species	146424
745331	occ	79789	Anomaloca	species	17341
745351	occ	79789	Macrocallis	species	109471
745375	occ	79788	Dosinia (Do	species	95243
745376	occ	79788	Macrocallis	species	109471
745378	occ	79788	Mercenari	species	108656
745386	occ	79788	Chione car	species	94441
745397	occ	79788	Anomaloca	species	17341
745400	occ	79788	Pitar morrl	species	146424
745410	occ	79788	Transenne	species	162010
745420	occ	69040	Chione car	species	94441
745436	occ	69038	Chione car	species	94441
745437	occ	69040	Gemma ge	species	108168
745439	occ	69038	Transenne	species	94533
745440	occ	69038	Anomaloca	species	146388
745442	occ	69040	Transenne	species	94533
745447	occ	69040	Anomaloca	species	146388

745452	occ	69038	Pitar simps	species	146434
745553	occ	79960	Mercenaria	species	95543
745554	occ	79960	Mercenaria	species	95536
745555	occ	79960	Mercenaria	species	17892
745566	occ	79960	Lirophora u	species	162137
745753	occ	79959	Chione pro	species	17468
745754	occ	79959	Mercenaria	species	95538
745755	occ	79959	Chione crib	species	95487
745784	occ	79959	Macrocallis	species	95248
747001	occ	80002	Anomaloca	species	17341
747003	occ	80002	Chione car	species	94441
747006	occ	80002	Macrocallis	species	109469
747008	occ	80002	Transenne	species	162009
747010	occ	80002	Pitar sayan	species	146429
747020	occ	80001	Anomaloca	species	17341
747023	occ	80001	Chione car	species	94441
747025	occ	80001	Gemma tri	species	17681
747027	occ	80001	Macrocallis	species	109469
747031	occ	80001	Transenella	species	91161
747034	occ	80001	Pitar sayan	species	146429
747254	occ	80027	Dosinia ele	species	94559
747255	occ	80027	Transenne	species	94533
747261	occ	80027	Chione car	species	94441
747291	occ	69044	Macrocallis	species	95251
747292	occ	69044	Transenne	species	162010
747293	occ	69044	Lirophora l	species	99343
747295	occ	69044	Chione crib	species	95487
747319	occ	69029	Chione car	species	94441
747321	occ	69029	Anomaloca	species	17341
747351	occ	69029	Transenne	species	162009
747356	occ	69029	Gemma ge	species	108168
747387	occ	69029	Pitar morrl	species	146424
747420	occ	79786	Chione car	species	94441
747427	occ	79786	Dosinia ele	species	94559
747435	occ	79786	Anomaloca	species	17341
747444	occ	79786	Lirophora s	genus	17824
747458	occ	79786	Transenne	species	162010
747460	occ	79786	Pitar morrl	species	146424
747461	occ	79786	Mercenaria	species	108656
747463	occ	79786	Macrocallis	species	109469
747468	occ	79786	Macrocallis	species	161775
747470	occ	79787	Chione car	species	94441
747472	occ	79787	Mercenaria	species	108656
747473	occ	79787	Macrocallis	species	109469
747474	occ	79787	Anomaloca	species	17341
747495	occ	79787	Dosinia ele	species	94559
747496	occ	79787	Transenne	species	162010

747510	occ	79787	Callocardia	subgenus	82721
747534	occ	79786	Gemma ma	species	95553
747603	occ	80037	Lirophora u	species	162137
747604	occ	80037	Chione ero	species	17468
747605	occ	80037	Lirophora l	species	99343
747628	occ	80037	Mercenaria	species	95536
747629	occ	80037	Macrocallis	species	95248
747633	occ	80037	Transenella	species	91157
747653	occ	80039	Lirophora l	species	99343
747656	occ	80039	Lirophora u	species	162137
747663	occ	80039	Chione ero	species	17468
747671	occ	80039	Pitar sp.	genus	18075
747674	occ	80039	Transenella	species	91157
747689	occ	80044	Mercenaria	species	95536
747691	occ	80044	Lirophora u	species	162137
747698	occ	80044	Lirophora l	species	99343
747702	occ	80044	Chione ero	species	17468
747710	occ	80044	Transenella	species	91157
747740	occ	80046	Pitar sp.	genus	18075
747741	occ	80046	Transenne	genus	162009
747744	occ	80046	Dosinia ele	species	94559
747747	occ	80046	Chione ele	species	146243
747755	occ	80046	Lirophora l	species	99343
747757	occ	80046	Mercenaria	genus	17892
747766	occ	80046	Macrocallis	species	161775
747801	occ	80046	Transenne	species	94533
747806	occ	80046	Anomaloca	genus	17341
748125	occ	69034	Chione car	species	94441
748132	occ	69034	Anomaloca	species	146388
748135	occ	69034	Globivenus	species	135221
748152	occ	69034	Gemma ge	species	108168
748283	occ	80025	Chione (Ch	species	146243
748303	occ	80025	Chione (Ch	species	95488
748314	occ	80025	Macrocallis	species	109471
748318	occ	80024	Chione (Ch	species	146243
748337	occ	80024	Dosinia ele	species	94559
748338	occ	80024	Transenne	species	162010
748345	occ	80024	Macrocallis	species	95248
748347	occ	80024	Chione (Ch	species	95488
748348	occ	80024	Anomaloca	species	17341
750999	occ	80359	Chione car	species	94441
751007	occ	80358	Chione car	species	94441
751008	occ	80358	Chione gru	species	248728
751034	occ	80357	Chione gru	species	248728
751035	occ	80357	Chione car	species	94441
751036	occ	80357	Chione gar	species	17468
751041	occ	80356	Chione car	species	94441

751046	occ		80355	Anomaloca	species	17341
751047	occ		80355	Chione car	species	94441
853742	occ		94809	Venus cam	species	94395
1011571	occ		123549	Chione (Lir	species	161769
1087923	occ		96538	Anomaloca	species	94549
1087924	occ		96538	Chione ele	species	146243
1087925	occ		96538	Cyclinella t	species	158650
1087926	occ		96538	Dosinia dis	species	94592
1087927	occ		96538	Dosinia ele	species	94559
1087928	occ		96538	Macrocallis	species	161775
1087929	occ		96538	Mercenaria	species	94396
1087930	occ		96538	Parastarte	species	162147
1087931	occ		96538	Pitar morrl	species	146424
1087932	occ		96538	Pitar simps	species	146434
1087933	occ		96538	Timoclea g	species	109498
1087934	occ		96538	Transenne	species	162010
256443	occ		20606	Mercenaria	species	108656
618909	occ		66724	Mercenaria	species	108656
1248044	occ		164698	Dosinia sp.	genus	17595
1248046	occ		164698	Gemma pu	species	17681
578548	occ		60923	Gemma ma	species	95553
578557	occ		60923	Chione lati	species	98378
578592	occ		60927	Chione car	species	94441
578593	occ	21707	60927	Chione (Ch	species	95488
578594	occ		60927	Chione lati	species	98378
578600	occ		60927	Gouldia me	species	17708
578601	occ		60927	Mercenaria	species	94396
578658	occ		60928	Gemma ma	species	95553
578663	occ		60928	Macrocallis	species	109469
578664	occ		60928	Mercenaria	species	94396
578678	occ		60928	Transenella	species	91157
578731	occ		60929	Gemma ma	species	95553
578733	occ		60929	Mercenaria	species	94396
578764	occ		60930	Gemma ma	species	95553
578766	occ		60930	Macrocallis	species	109469
578767	occ		60930	Mercenaria	species	94396
578774	occ		60930	Transenella	species	91157
578843	occ		60931	Chione lati	species	98378
578876	occ		60942	Mercenaria	species	95543
578924	occ		60940	Mercenaria	species	17892
578925	occ		60940	Chione ero	species	17468
578926	occ		60940	Chione (Lir	species	145058
578927	occ		60940	Macrocallis	species	170492
578928	occ		60940	Dosinia ace	species	95241
578945	occ		60939	Pitar sayan	species	146429
578948	occ		60941	Chione ero	species	17468
579023	occ		60944	Dosinia ele	species	94559

579026	occ	60944	Gemma m	species	95553
579028	occ	60944	Macrocalli	species	109469
579036	occ	60944	Pitar sayan	species	146429
619636	occ	66759	Dosinia ele	species	94559
619640	occ	66760	Macrocalli	species	109469
619643	occ	66760	Chione car	species	94441
619644	occ	66758	Chione car	species	94441
619645	occ	66756	Chione car	species	94441
619652	occ	66757	Chione int	species	108667
619653	occ	66760	Chione int	species	108667
619655	occ	66758	Chione gru	species	248728
619656	occ	66760	Chione gru	species	248728
619660	occ	66760	Mercenari	species	108656
619661	occ	66758	Mercenari	species	108656
619662	occ	66756	Mercenari	species	108656
619672	occ	66760	Mercenari	species	94396
619673	occ	66758	Mercenari	species	94396
619674	occ	66756	Mercenari	species	94396
642859	occ	69046	Lirophora (	subgenus	90364
642931	occ	69046	Gouldia m	species	17708
642938	occ	69046	Chione sp.	genus	17468
642944	occ	69046	Mercenari	genus	17892
642946	occ	69046	Pitar (Pitar	species	100951
642949	occ	69046	Transenne	species	94533
745479	occ	79956	Macrocalli	species	109338
745482	occ	79956	Veneridae	family	62779
745486	occ	79957	Pitar morrl	species	146424
747821	occ	80053	Macrocalli	species	109471
747823	occ	80053	Mercenari	species	94396
747835	occ	80053	Gemma m	species	95553
747843	occ	80053	Lirophora l	species	99343
747844	occ	80053	Dosinia ele	species	94559
748095	occ	80083	Lirophora l	species	99343
748105	occ	80083	Gemma m	species	95553
748106	occ	80083	Transenell	species	91157
748114	occ	80083	Macrocalli	species	95251
832857	occ	92404	Venus mer	species	95528
832891	occ	92407	Cytherea s	species	95259
832892	occ	92407	Artemis ac	species	95240
832893	occ	92407	Venus crib	species	95485
832938	occ	92408	Gouldia m	species	17708
832939	occ	92408	Gemma m	species	95553
832940	occ	92408	Chione gru	species	248728
832941	occ	92408	Chione crib	species	95487
832942	occ	92408	Chione lati	species	98378
832943	occ	92408	Venus riley	species	95535
832961	occ	92409	Gouldia m	species	17708

832962	occ		92409	Gemma ma	species	95553
832963	occ	22482	92409	Mercenaria	genus	17892
832974	occ		92411	Venus crib	species	95485
833021	occ		92412	Chione lati	species	98378
1457470	occ		203376	Chione crib	species	95487
564304	occ		58893	Gouldia me	species	17708
564312	occ		58894	Gouldia me	species	17708
564344	occ		58895	Gouldia me	species	17708
564349	occ		58895	Mercenaria	species	82725
564351	occ		58895	Macrocallis	species	82723
564387	occ		58898	Gouldia me	species	17708
564389	occ		58898	Macrocallis	species	82723
564391	occ		58898	Mercenaria	species	82725
564399	occ		58897	Mercenaria	species	82725
564400	occ		58897	Macrocallis	species	82723
564403	occ		58897	Gouldia me	species	17708
564411	occ		58897	Gemma ma	species	95553
564610	occ		58893	Transenne	species	94533
564643	occ		58895	Pitar chion	species	18075
564652	occ		58895	Transenne	species	94533
564893	occ		58898	Pitar chion	species	18075
564909	occ		58897	Pitar chion	species	18075
564917	occ		58897	Transenne	species	94533
564918	occ		58897	Ventricolar	species	18389
566337	occ		59301	Mercenaria	species	95536
566856	occ		59307	Cooperella	species	17500
566857	occ		59329	Dosinia ace	species	95241
566859	occ		59309	Dosinia (Do	species	95243
566860	occ		59313	Dosinia (Do	species	95243
566861	occ		59316	Dosinia (Do	species	95243
566862	occ		59307	Macrocallis	species	95248
566863	occ		59327	Macrocallis	species	95248
566864	occ		59309	Macrocallis	species	95248
566865	occ		59313	Macrocallis	species	95248
566866	occ		59310	Macrocallis	species	95248
566867	occ		59318	Macrocallis	species	95248
566868	occ		59316	Macrocallis	species	95248
566869	occ		59307	Macrocallis	species	95263
566871	occ		59318	Callocardia	species	95264
566872	occ		59313	Callocardia	species	95264
566873	occ		59316	Callocardia	species	95264
566874	occ		59312	Callocardia	species	95264
566875	occ		59309	Callocardia	species	95264
566876	occ		59310	Callocardia	species	95264
566877	occ		59328	Callocardia	species	95478
566878	occ		59318	Callocardia	species	82722
566879	occ		59307	Chione (Ch	species	95488

566880	occ	59330	Chione (Ch	species	95488
566881	occ	59317	Chione (Ch	species	95488
566882	occ	59313	Chione (Ch	species	95488
566883	occ	59309	Chione (Ch	species	95488
566884	occ	59316	Chione (Ch	species	95488
566885	occ	59315	Chione (Ch	species	95488
566886	occ	59318	Chione (Ch	species	95488
566887	occ	59307	Chione (Ch	species	95523
566888	occ	59309	Chione (Ch	species	95523
566889	occ	59313	Chione (Ch	species	95523
566890	occ	59316	Chione (Ch	species	95523
566891	occ	59318	Chione (Ch	species	95523
566892	occ	59330	Chione (Ch	species	95523
566893	occ	59318	Venus (Me	species	95534
566894	occ	59328	Venus (Me	species	95534
566895	occ	59330	Venus (Me	species	95534
566896	occ	59327	Venus (Me	species	95534
566897	occ	59309	Venus (Me	species	95534
566898	occ	59307	Venus (Me	species	95534
566899	occ	59311	Venus (Me	species	95534
566900	occ	59310	Venus (Me	species	95534
566901	occ	59313	Venus (Me	species	95534
566902	occ	59312	Venus (Me	species	95534
566903	occ	59316	Venus (Me	species	95534
566904	occ	59317	Venus (Me	species	95534
566905	occ	59329	Venus (Me	species	95534
566906	occ	59318	Gemma ma	species	95553
566907	occ	59317	Gemma ma	species	95553
566908	occ	59316	Gemma ma	species	95553
566909	occ	59312	Gemma ma	species	95553
566910	occ	59314	Gemma ma	species	95553
566911	occ	59310	Gemma ma	species	95553
566912	occ	59309	Gemma ma	species	95553
566913	occ	59307	Gemma ma	species	95553
566914	occ	59330	Gemma ma	species	95553
566915	occ	59329	Gemma ma	species	95553
566916	occ	59328	Gemma ma	species	95553
566917	occ	59313	Gemma ma	species	95553
566918	occ	59315	Gemma ma	species	95553
566919	occ	59330	Gemma cra	species	95557
579608	occ	60986	Mercenaria	species	82725
579683	occ	60989	Mercenaria	species	82725
579684	occ	60989	Callocardia	species	95264
579764	occ	60988	Mercenaria	species	82725
579765	occ	60988	Callocardia	species	95264
579817	occ	60987	Mercenaria	species	82725
587879	occ	61919	Mclellania	species	82264

587880	occ	52582	Mclelannia	species	461847
587881	occ	49983	Mclellania	species	82264
587882	occ	61920	Mclelannia	species	461847
587883	occ	61920	Mercenaria	species	17892
587884	occ	61921	Mercenaria	species	17892
587885	occ	61921	Mclelannia	species	461847
587886	occ	61922	Mercenaria	species	17892
587887	occ	61922	Mclelannia	species	461847
587888	occ	61923	Mercenaria	species	17892
587889	occ	61923	Mclelannia	species	461847
619637	occ	66742	Dosinia ele	species	94559
619638	occ	66742	Dosinia dis	species	94592
619639	occ	66742	Pitar morrl	species	94531
619641	occ	66750	Macrocallis	species	109469
619642	occ	66742	Macrocallis	species	109469
619646	occ	66754	Chione car	species	94441
619647	occ	66750	Chione car	species	94441
619648	occ	66749	Chione car	species	94441
619649	occ	66748	Chione car	species	94441
619650	occ	66747	Chione car	species	94441
619651	occ	66742	Chione car	species	94441
619654	occ	66742	Chione lati	species	98378
619657	occ	66750	Gemma ge	species	108168
619658	occ	66749	Gemma ge	species	108168
619659	occ	66742	Gemma ge	species	108168
619663	occ	66754	Mercenaria	species	108656
619664	occ	66751	Mercenaria	species	108656
619665	occ	66750	Mercenaria	species	108656
619666	occ	66749	Mercenaria	species	108656
619667	occ	66748	Mercenaria	species	108656
619668	occ	66747	Mercenaria	species	108656
619669	occ	66745	Mercenaria	species	108656
619670	occ	66744	Mercenaria	species	108656
619671	occ	66742	Mercenaria	species	108656
619675	occ	66750	Mercenaria	species	94396
619676	occ	66748	Mercenaria	species	94396
619677	occ	66742	Mercenaria	species	94396
640560	occ	69086	Pitar chion	species	18075
642711	occ	69045	Macrocallis	species	109471
642722	occ	69045	Mercenaria	species	108656
642746	occ	69045	Chione sp.	genus	17468
642747	occ	69045	Transenella	genus	91157
659686	occ	71138	Mercenaria	species	95538
659700	occ	71138	Pitar sayan	species	146429
659729	occ	71138	Gouldia me	species	17708
659745	occ	71138	Gemma ma	species	95553
700939	occ	69043	Mercenaria	species	94396

700951	occ	69043	Chione crik	species	95487
700953	occ	69043	Macrocallis	species	95248
700956	occ	69043	Transenne	species	94533
700963	occ	69043	Gouldia me	species	17708
701046	occ	69047	Mercenaria	species	108656
701054	occ	69047	Ventricolar	genus	18389
701070	occ	69047	Veneridae	family	62779
702854	occ	68979	Macrocallis	species	95251
702857	occ	68979	Chione crik	species	95487
702860	occ	68979	Mercenaria	species	94396
702865	occ	68979	Transenella	species	91157
702896	occ	69018	Macrocallis	species	95251
702909	occ	69018	Transenella	genus	91157
702917	occ	69018	Chione sp.	genus	17468
702918	occ	69018	Lirophora (	subgenus	90364
737381	occ	68979	Gemma ma	species	95553
737394	occ	79249	Gemma ma	species	95553
737395	occ	79249	Transenella	species	91157
737402	occ	79249	Mercenaria	species	94396
737432	occ	79248	Veneridae	family	62779
738402	occ	79342	Transenella	species	91157
738407	occ	79342	Macrocallis	species	95251
738409	occ	79342	Mercenaria	species	94396
738432	occ	79343	Transenella	species	91157
738437	occ	79343	Macrocallis	species	95251
738440	occ	79343	Gemma ma	species	95553
738451	occ	79343	Chione crik	species	95487
738459	occ	79341	Transenella	species	91157
738463	occ	79341	Chione crik	species	95487
738501	occ	79339	Gouldia me	species	17708
738502	occ	79339	Transenne	species	94533
738507	occ	79339	Chione crik	species	95487
738517	occ	79339	Macrocallis	species	95248
738529	occ	79340	Chione crik	species	95487
738532	occ	79340	Lirophora l	species	99343
738533	occ	79340	Macrocallis	species	95248
738542	occ	79340	Gemma ma	species	95553
741419	occ	79708	Pitar chion	species	18075
748224	occ	80034	Mercenaria	species	94396
748228	occ	80034	Chione (Ch	species	109474
748229	occ	80034	Chione (Ch	species	95484
748230	occ	80034	Lirophora l	species	99343
748231	occ	80034	Pitar casto	species	18075
748372	occ	80033	Lirophora s	genus	17824
748378	occ	80033	Dosinia (Do	species	95242
748385	occ	80033	Macrocallis	genus	17856
748398	occ	80033	Gouldia sp	genus	17708

748448	occ		80033	Mercenari	genus	17892
748451	occ		80033	Chione gru	species	248728
748463	occ		80033	Mercenari	species	95545
748500	occ		80033	Macrocalli	subgenus	94610
748502	occ		80033	Lirophora s	genus	17824
748515	occ		80033	Gouldia sp	genus	17708
748518	occ		80033	Chione cf.	species	94441
748519	occ		80033	Timoclea c	species	109498
748531	occ		80033	Mercenari	genus	17892
748554	occ		80035	Gouldia sp	genus	17708
748555	occ		80035	Transenell	genus	91157
748568	occ		80035	Chione car	species	94441
748572	occ		80035	Dosinia ac	species	95241
748575	occ		80035	Macrocalli	species	95251
748580	occ		80035	Mercenari	genus	17892
748650	occ		80035	Lirophora s	genus	17824
748661	occ		80035	Chione gru	species	248728
829462	occ		91489	Gouldia (P	species	155061
1173572	occ		151326	Mercenari	species	95536
1512845	occ		211183	Mercenari	species	82725
1512981	occ		211182	Mercenari	species	82725
94764	occ		7108	Mercenari	species	94396
94768	occ		7108	Mercenari	species	108656
94778	occ		7109	Pitar morrl	species	146423
94780	occ		7109	Mercenari	species	108656
559156	occ		57705	Gemma pu	species	17681
559159	occ		57705	Mercenari	species	94396
559160	occ		57705	Mercenari	species	108656
559195	occ		57706	Mercenari	species	94396
559215	occ		57702	Mercenari	species	94396
559222	occ		57702	Pitar morrl	species	94531
559229	occ		57703	Dosinia dis	species	94592
559232	occ		57703	Mercenari	species	108656
559235	occ		57703	Pitar morrl	species	94531
566296	occ		59297	Mercenari	species	17892
566318	occ		59293	Venus riley	species	95535
576543	occ		60667	Mercenari	species	95543
582212	occ		61293	Amiantis ?	genus	17326
582216	occ	20509	61293	Chione crib	species	95487
582231	occ		61293	Pitar sayan	species	146429
582256	occ		61295	Pitar sayan	species	146429
582259	occ		61296	Macrocalli	genus	17856
582263	occ		61296	Pitar sayan	species	146429
587892	occ		61925	Mercenari	species	17892
587893	occ		61925	Mclelannia	species	461847
587894	occ		61926	Mercenari	species	17892
587895	occ		61926	Mclelannia	species	461847

587896	occ	61927	Mercenari	species	17892
587897	occ	61927	Mclelannia	species	461847
587898	occ	61928	Mercenari	species	17892
587899	occ	61928	Mclelannia	species	461847
587900	occ	59155	Mercenari	species	17892
587901	occ	59155	Mclelannia	species	461847
587902	occ	61929	Mercenari	species	17892
587903	occ	61929	Mclelannia	species	461847
640358	occ	69048	Chione sp.	genus	17468
640362	occ	69048	Mercenari	species	94396
640364	occ	69049	Mercenari	species	94396
664784	occ	71620	Mercenari	species	17892
664840	occ	71624	Mercenari	species	17892
665354	occ	60660	Dosinia ace	species	95241
665355	occ	60660	Mercenari	species	95543
727673	occ	78271	Pitar sayan	species	146429
727674	occ	78271	Mercenari	species	95543
727675	occ	78271	Gemma ma	species	95553
727678	occ	78271	Dosinia ace	species	95241
727706	occ	78272	Mercenari	species	95543
727764	occ	78270	Mercenari	genus	17892
727790	occ	78269	Gemma ma	species	95553
727840	occ	78268	Dosinia ace	species	95241
727874	occ	78267	Gemma ma	species	95553
727875	occ	78267	Mercenari	genus	17892
727904	occ	78266	Transenella	species	91157
727905	occ	78266	Macrocallis	species	95248
727906	occ	78266	Pitar sayan	species	146429
727907	occ	78266	Mercenari	genus	17892
727908	occ	78266	Gemma ma	species	95553
727915	occ	78266	Dosinia ace	species	95241
727966	occ	78265	Gouldia me	species	17708
727967	occ	78265	Macrocallis	species	95248
727968	occ	78265	Mercenari	genus	17892
727976	occ	78265	Dosinia ace	species	95241
728032	occ	78261	Pitar sayan	species	146429
728033	occ	78261	Mercenari	genus	17892
728071	occ	78262	Gouldia me	species	17708
728072	occ	78262	Mercenari	genus	17892
728073	occ	78262	Gemma ma	species	95553
728080	occ	78262	Dosinia ace	species	95241
728118	occ	78264	Macrocallis	species	95248
728119	occ	78264	Mercenari	genus	17892
728127	occ	78264	Dosinia ace	species	95241
737477	occ	79246	Veneridae	family	62779
737487	occ	79247	Lirophora l	species	99343
737490	occ	79245	Dosinia (Do	species	95242

737491	occ		79245	Chione car	species	94441
737492	occ		79245	Agriopoma	species	95264
741431	occ		79709	Gouldia me	species	17708
741438	occ		79709	Mercenaria	species	94396
741439	occ		79709	Mercenaria	genus	17892
741468	occ		79709	Chione crib	species	95487
741474	occ		79709	Agriopoma	species	95264
747721	occ		80048	Pitar morrl	species	146424
747736	occ		80047	Mercenaria	species	108656
748170	occ		80040	Dosinia ace	species	17595
748176	occ		80040	Mercenaria	species	94396
748273	occ		80038	Lirophora l	species	99343
748279	occ		80038	Mercenaria	species	94396
748405	occ		80036	Mercenaria	species	94396
748432	occ		80036	Dosinia (Do	species	95242
942735	occ		103358	Cryptograr	species	437839
632426	occ		67069	Verticordia	species	412248
738216	occ		79331	Verticordia	species	108208
738217	occ		79336	Verticordia	species	108208
578934	occ		60940	Verticordia	species	18843
564653	occ		58895	Verticordia	species	18843
566580	occ		59318	Verticordia	species	108208
566581	occ		59307	Verticordia	species	108208
568559	occ		59486	Yoldia (Adr	species	16145
747564	occ		80030	Yoldia sp.	genus	16145
747586	occ		80029	Yoldia sp.	genus	16145
579042	occ		60944	Yoldia laev	species	98556
619459	occ		66760	Yoldia lima	species	87532
832920	occ		92408	Yoldia sp.	genus	16145
832996	occ		92412	Yoldia tarp	species	16145
566489	occ		59329	Yoldia laev	species	98556
566491	occ		59312	Yoldia laev	species	98556
566492	occ		59313	Yoldia laev	species	98556
566493	occ		59310	Yoldia laev	species	98556
579565	occ	21089	60986	Yoldia limu	species	16145
579669	occ		60989	Yoldia lima	species	87532
579734	occ		60988	Yoldia lima	species	87532
579807	occ		60987	Yoldia lima	species	87532
619460	occ		66751	Yoldia lima	species	87532
619461	occ		66744	Yoldia lima	species	87532
619462	occ		66742	Yoldia lima	species	87532
659691	occ		71138	Yoldia laev	species	98556
748523	occ		80033	Yoldia laev	species	98556
582227	occ		61293	Yoldia laev	species	98556
582245	occ		61294	Yoldia laev	species	98556
582254	occ		61295	Yoldia laev	species	98556
582266	occ		61296	Yoldia laev	species	98556

727668	occ	78271	Yoldia laev	species	98556
727837	occ	78268	Yoldia laev	species	98556
727898	occ	78266	Yoldia laev	species	98556
727960	occ	78265	Yoldia laev	species	98556
728026	occ	78261	Yoldia laev	species	98556
737493	occ	79245	Yoldia laev	species	98556
747723	occ	80048	Yoldia lima	species	87532
427347	occ	41705	Bivalvia inc	class	16005
640524	occ	69085	Cyrenoidea	species	244401
640548	occ	69084	Bivalvia inc	class	16005
747268	occ	80027	Bivalvia inc	class	16005
747270	occ	80027	Cyrenoidea	species	244401
747301	occ	69044	Bivalvia inc	class	16005
747503	occ	79787	Bivalvia inc	class	16005
748313	occ	80025	Bivalvia inc	class	16005
748329	occ	80024	Bivalvia inc	class	16005
741392	occ	79706	Bivalvia inc	class	16005
748121	occ	80083	Bivalvia inc	class	16005
700974	occ	69043	Bivalvia inc	class	16005
737436	occ	79248	Bivalvia inc	class	16005
738425	occ	79342	Bivalvia inc	class	16005
738477	occ	79341	Bivalvia inc	class	16005
738493	occ	79339	Bivalvia inc	class	16005
582240	occ	61294	Leptonacea	superfamil	93685
737483	occ	79247	Bivalvia inc	class	16005
737516	occ	79245	Bivalvia inc	class	16005
426219	occ	41501	Sulcularia p	species	71753
567324	occ	59486	Sulcularia s	species	71753
567337	occ	59484	Sulcularia s	species	71753
738384	occ	79331	Sulcularia p	species	71753
425997	occ	41501	Acteon pu	species	156427
427921	occ	41740	Actaeon sp	genus	11895
567318	occ	59485	Acteon va	species	58568
567319	occ	59484	Acteon va	species	58568
697950	occ	75011	Acteon sp.	genus	58568
738381	occ	79331	Acteon pu	species	156427
738392	occ	79336	Rictaxis my	species	11995
738395	occ	79338	Rictaxis my	species	11995
1089062	occ	96538	Japonactae	species	156428
728090	occ	78264	Actaeon nc	species	11895
637119	occ	67069	Aorotrema	species	116503
750985	occ	79331	Aorotrema	species	116503
426149	occ	41501	"Fontigens	genus	121479
427776	occ	41718	"Fontigens	genus	121479
568440	occ	59484	Amnicola a	species	81489
747040	occ	80001	"Fontigens	genus	121479
751016	occ	80358	"Fontigens	genus	121479

1607544	occ	227396	Amnicola c	species	81489
1607588	occ	227400	Amnicola c	species	81489
1248051	occ	164698	Amnicola s	species	311057
1248052	occ	164698	Amnicola g	species	311058
1248053	occ	164698	Amnicola e	species	311059
1088935	occ	96538	Pomacea p	species	251916
1607473	occ	227384	Pomacea p	species	251916
1607490	occ	227388	Pomacea p	species	251916
1607492	occ	227389	Pomacea p	species	251916
1607498	occ	227390	Pomacea p	species	251916
1607513	occ	227392	Pomacea p	species	251916
1607542	occ	227396	Pomacea p	species	251916
745954	occ	79958	Pachycrom	species	95682
428040	occ	41749	Architecto	species	90235
568153	occ	59487	Architecto	species	90239
568154	occ	59486	Architecto	species	90239
568155	occ	59484	Architecto	species	90239
1089056	occ	96538	Architecto	species	90235
578957	occ	60940	Architecto	species	90235
619872	occ	66760	Architecto	species	90235
1457440	occ	79745	Architecto	species	392478
1457488	occ	203377	Architecto	species	392478
567044	occ	59309	Architecto	species	109082
727842	occ	78269	Architecto	species	119754
637088	occ	67069	Arene trica	species	156288
749679	occ	79331	Arene (Ma	species	156288
564611	occ	58893	Arene perg	species	82743
564625	occ	58894	Arene perg	species	82743
564659	occ	58895	Arene perg	species	82743
564923	occ	58897	Arene perg	species	82743
567029	occ	59317	Liotia (Are	species	82742
567030	occ	59316	Liotia (Are	species	82742
567031	occ	59315	Liotia (Are	species	82742
659736	occ	71138	Arene perg	species	82743
748184	occ	80040	Arene trica	species	156288
426201	occ	41501	Pyrazus sca	species	10393
427807	occ	41718	Pyrazus sca	species	10393
428105	occ	41749	Pyrazus sca	species	10393
590997	occ	62458	Pyrazisinus	species	82782
622886	occ	67070	Pyrazus sca	species	10393
741866	occ	79738	Pyrazisinus	species	10392
741881	occ	79739	Batillaria m	species	156622
742001	occ	79747	Pyrazisinus	species	10392
742002	occ	79747	Pyrazisinus	species	10392
742003	occ	79747	Pyrazisinus	species	10392
742220	occ	79335	Pyrazus (Py	species	93965
742564	occ	79782	Pyrazisinus	species	10392

743218	occ	79804	Pyrazisinus	species	10392
743219	occ	79804	Pyrazisinus	species	10392
744289	occ	79876	Pyrazisinus	species	10392
744290	occ	79876	Pyrazisinus	species	10392
744291	occ	79876	Pyrazisinus	species	93966
744365	occ	79875	Pyrazisinus	species	10392
749429	occ	79331	Pyrazus (Py	species	93965
749462	occ	79333	Pyrazus (Py	species	93965
870234	occ	79960	Pyrazisinus	species	93966
870258	occ	35025	Pyrazisinus	species	10392
870265	occ	35031	Pyrazisinus	genus	10392
870275	occ	35031	Pyrazisinus	species	10392
1088942	occ	96538	Batillaria m	species	156622
1088945	occ	96538	Pyrazisinus	species	10392
1088946	occ	96538	Pyrazisinus	species	10392
1088947	occ	96538	Pyrazisinus	species	82782
426155	occ	41501	Hanetia mo	species	164465
427759	occ	41718	Cantharus	genus	10894
428052	occ	41749	Cantharus	species	10894
428053	occ	41749	Cantharus	species	10894
428083	occ	41749	Hanetia mo	species	164465
428084	occ	41749	Hanetia va	species	11175
567630	occ	59484	Phos sloan	species	188145
582433	occ	61309	Antillophos	species	91532
582452	occ	61310	Cantharus	species	91546
582453	occ	61310	Cantharus	species	91554
582467	occ	61311	Cantharus	species	91545
622898	occ	67070	Bailya intri	species	91136
622899	occ	67070	Monostiolu	species	93959
697959	occ	75012	"Nassa" cf.	species	11401
741344	occ	79331	Gemophos	species	81260
741345	occ	79331	Gemophos	species	156667
741346	occ	79331	Hanetia (Sc	species	164465
741347	occ	79331	Engina flor	species	11064
741903	occ	79739	Solenostei	species	91543
742182	occ	79762	Metula rob	species	93958
742189	occ	79762	Hindsia pyt	species	81261
742209	occ	79765	Hindsia pyt	species	81261
744402	occ	79874	Monostiolu	species	93960
745651	occ	79331	Strombino	species	104866
745683	occ	79331	Bailya royc	species	10823
745726	occ	79959	Cymatopho	species	11009
745928	occ	79958	Solenostei	species	11681
745930	occ	79958	Hesperiste	species	188171
745931	occ	79958	Hesperiste	species	188176
745932	occ	79958	Hesperiste	species	188175
747016	occ	80002	Hanetia mo	species	164465

747357	occ	69029	Solenostei	species	11681
747445	occ	79786	Buccinum (	species	10866
747602	occ	80037	Ptychosalp	species	11599
748159	occ	69034	Cantharus	genus	10894
748351	occ	80024	Gemophos	genus	81260
870202	occ	79960	Celatoconu	species	10904
1089010	occ	96538	Bailya intri	species	91136
1089011	occ	96538	Cantharus	species	91554
1089012	occ	96538	Gemophos	species	91540
1089013	occ	96538	Solenostei	species	91543
1150739	occ	146873	Strombino	genus	11715
1150745	occ	146873	Ptychosalp	genus	11599
578681	occ	60928	Cantharus	species	91541
578776	occ	60930	Cantharus	species	91541
578861	occ	60937	Phos sloan	species	188145
619951	occ	66760	Cantharus	species	91547
619952	occ	66758	Cantharus	species	91547
619953	occ	66756	Cantharus	species	91547
619954	occ	66755	Cantharus	species	91547
619956	occ	66760	Cantharus	species	91541
567085	occ	59307	Colubraria	species	9673
567132	occ	59307	Ptychosalp	species	11599
567133	occ	59329	Ptychosalp	species	11599
567134	occ	59309	Ptychosalp	species	11599
567135	occ	59310	Ptychosalp	species	11599
567136	occ	59307	Pisania (Ce	species	97504
567137	occ	59316	Pisania (Ce	species	97504
619955	occ	66742	Cantharus	species	91547
559477	occ	57706	Buccinulun	species	91534
559481	occ	57706	Colus pygn	species	91559
559838	occ	57704	Cantharus	species	91545
727847	occ	78269	Ptychosalp	species	11599
728016	occ	78261	Pisania nu›	species	11526
728052	occ	78262	Ptychosalp	species	11599
737508	occ	79245	Ptychosalp	species	11599
737511	occ	79245	Ptychosalp	species	11599
737512	occ	79245	Ptychosalp	species	11599
741442	occ	79709	Ptychosalp	genus	11599
741453	occ	79709	Ptychosalp	species	11599
870225	occ	96535	Celatoconu	species	10904
426011	occ	41501	Bulla occid	species	119892
426012	occ	41501	Bulla striat	species	94556
427447	occ	41705	Bulla occid	species	119892
427617	occ	41715	Bulla striat	species	94556
427752	occ	41718	Bulla cf. oc	species	119892
428042	occ	41749	Bulla occid	species	119892
428043	occ	41749	Bulla striat	species	94556

582524	occ		61309	Bulla striat	species	94556
582540	occ		61310	Bulla striat	species	94556
582556	occ		61311	Bulla striat	species	94556
640538	occ		69084	Bulla striat	species	94556
640745	occ		69087	Bulla sp.	genus	11910
738387	occ		79331	Bulla striat	species	94556
738388	occ		79331	Bulla occid	species	119892
741911	occ		79739	Bulla occid	species	119892
744300	occ		79876	Bulla saras	species	11910
745355	occ		79789	Bulla occid	species	119892
745382	occ		79788	Bulla occid	species	119892
745438	occ		69038	Bulla striat	species	94556
747011	occ		80002	Bulla occid	species	119892
747253	occ		80027	Bulla striat	species	94556
747365	occ		69029	Bulla striat	species	94556
747480	occ		79787	Bulla striat	species	94556
747535	occ		79786	Bulla occid	species	119892
748340	occ		80024	Bulla striat	species	94556
870296	occ		96538	Bulla umbi	species	11910
1089065	occ		96538	Bulla occid	species	119892
745951	occ	21786	79958	Bursa rugo	species	187876
745952	occ		79958	Bursa (Mar	species	159054
1087964	occ		79959	Marsupina	species	117921
426017	occ		41501	Caecum co	species	9577
426018	occ		41501	Caecum flo	species	9577
426019	occ		41501	Caecum im	species	101217
426020	occ		41501	Caecum re	species	116029
426172	occ		41501	Meioceras	species	156531
426173	occ		41501	Meioceras	species	10110
427450	occ		41705	Caecum cf.	species	9577
427451	occ		41705	Caecum co	species	9577
427452	occ		41705	Caecum flo	species	9577
427453	occ		41705	Caecum im	species	101217
427454	occ		41705	Caecum re	species	116029
427455	occ		41705	Caecum sp	genus	9577
427463	occ		41705	Caecum to	species	9577
427755	occ		41718	Caecum flo	species	9577
427756	occ		41718	Caecum im	species	101217
427785	occ		41718	Meioceras	species	156531
568129	occ		59484	Caecum co	species	157307
568130	occ		59484	Caecum flo	species	9577
568131	occ		59484	Caecum co	species	248851
568132	occ		59484	Caecum re	species	116029
568133	occ		59484	Meioceras	species	156530
568134	occ		59484	Meioceras	species	156531
582357	occ		61310	Caecum flo	species	9577
582358	occ		61310	Caecum im	species	101217

582359	occ	61310	Caecum pu	species	9577
582360	occ	61310	Caecum ve	species	156712
749426	occ	79338	Caecum (C	species	116030
749427	occ	79338	Caecum (C	species	116030
749428	occ	79338	Caecum (C	species	116028
749431	occ	79338	Caecum (C	species	116028
749452	occ	79331	Caecum (C	species	116030
749453	occ	79331	Caecum (C	species	116028
749454	occ	79331	Caecum (C	species	116028
749456	occ	79331	Caecum (C	species	116028
749464	occ	79331	Caecum (E	species	101218
749643	occ	79331	Caecum (B	species	248852
749644	occ	79331	Meioceras	species	156530
749645	occ	79331	Meioceras	species	156531
1088963	occ	96538	Caecum im	species	101217
1088964	occ	96538	Caecum pu	species	9577
1088965	occ	96538	Caecum st	species	9577
1088966	occ	96538	Meioceras	species	156530
579044	occ	60944	Caecum im	species	101217
832912	occ	92408	Caecum co	species	157307
832913	occ	92408	Caecum ib	species	9577
564626	occ	58894	Caecum pu	species	9577
564662	occ	58895	Caecum fle	species	116033
564663	occ	58895	Caecum im	species	101217
564664	occ	58895	Caecum pu	species	9577
564929	occ	58897	Caecum be	species	82747
564930	occ	58897	Caecum fle	species	116033
564931	occ	58897	Caecum im	species	101217
567052	occ	59316	Caecum fle	species	116033
567053	occ	59316	Caecum re	species	116029
567054	occ	59330	Caecum re	species	116029
567055	occ	59330	Caecum st	species	9577
567056	occ	59307	Caecum st	species	9577
559480	occ	57706	Caecum co	species	157307
362445	occ	34269	Crepidula f	species	94398
362446	occ	34269	Crepidula p	species	81481
423334	occ	41202	Calyptraea	genus	9583
423368	occ	41205	Crucibulum	genus	9704
425342	occ	41497	Crepidula r	species	95417
425343	occ	41497	Crucibulum	species	156291
425362	occ	41497	Crepidula a	species	81485
425373	occ	41498	Crepidula a	species	81485
425374	occ	41498	Crucibulum	species	156291
426024	occ	41501	Calyptraea	species	81411
426097	occ	41539	Crepidula a	species	81485
426098	occ	41539	Crucibulum	species	156291
426131	occ	41501	Crepidula a	species	81485

426132	occ	41501	Crepidula r	species	95417
426133	occ	41501	Crepidula p	species	81481
426134	occ	41501	Crucibulum	species	156291
426135	occ	41501	Crucibulum	species	9704
426136	occ	41501	Crucibulum	genus	9704
427474	occ	41705	Crepidula a	species	81485
427475	occ	41705	Crepidula r	species	95417
427476	occ	41705	Crepidula p	species	81481
427477	occ	41705	Crucibulum	species	156291
427621	occ	41715	Crepidula r	species	95417
427766	occ	41718	Crepidula a	species	81485
427767	occ	41718	Crepidula r	species	95417
427768	occ	41718	Crucibulum	species	156291
427927	occ	41740	Crepidula p	species	81481
428067	occ	41749	Crepidula a	species	81485
428068	occ	41749	Crepidula f	species	94398
428069	occ	41749	Crepidula p	species	81481
428070	occ	41749	Crucibulum	species	9704
428071	occ	41749	Crucibulum	species	97657
568422	occ	59489	Crepidula a	species	81485
568423	occ	59487	Crepidula a	species	81485
568424	occ	59487	Crepidula p	species	81481
568425	occ	59484	Crepidula p	species	81481
568426	occ	59487	Crepidula f	species	94398
568427	occ	59485	Crepidula a	species	9697
568428	occ	59484	Crepidula a	species	9697
568429	occ	59486	Crucibulum	species	156291
568430	occ	59487	Crucibulum	species	156291
568431	occ	59484	Crucibulum	species	156291
568432	occ	59489	Crucibulum	species	94742
568433	occ	59484	Crucibulum	species	97657
568434	occ	59485	Crucibulum	species	97657
568435	occ	59487	Calyptraea	species	81411
568436	occ	59485	Calyptraea	species	81411
568437	occ	59484	Calyptraea	species	81411
582375	occ	61311	Crepidula a	species	81485
582376	occ	61311	Crepidula f	species	94398
582377	occ	61311	Crepidula r	species	95417
582378	occ	61311	Crepidula p	species	81481
582395	occ	61309	Crepidula a	species	81485
582396	occ	61309	Crepidula f	species	94398
582397	occ	61309	Crepidula p	species	81481
582398	occ	61309	Crepidula r	species	95417
582415	occ	61310	Calyptraea	species	81411
582416	occ	61310	Crepidula a	species	81485
582417	occ	61310	Crepidula f	species	94398
582418	occ	61310	Crepidula r	species	95417

582419	occ	61310	Crepidula p	species	81481
640533	occ	69084	Crepidula p	species	81481
640551	occ	69084	Crepidula f	species	94398
640747	occ	69087	Crepidula f	species	94398
640759	occ	69087	Crepidula p	species	81481
697877	occ	74999	Calyptraea	species	81411
697944	occ	75011	Crepidula f	species	94398
741762	occ	79731	Crepidula f	species	94398
744336	occ	79875	Crepidula c	species	9697
745335	occ	79789	Crepidula f	species	94398
745358	occ	79789	Crucibulum	subgenus	92422
745389	occ	79788	Crepidula f	species	94398
745396	occ	79788	Crepidula p	species	81481
745418	occ	69040	Bostrycapu	species	116296
745422	occ	69040	Crucibulum	species	92421
745423	occ	69040	Crepidula f	species	94398
745701	occ	79331	Crucibulum	species	92422
745702	occ	79331	Crucibulum	species	97657
747014	occ	80002	Crepidula f	species	94398
747039	occ	80001	Crepidula f	species	94398
747316	occ	69044	Calyptraea	species	81411
747349	occ	69029	Crucibulum	subgenus	92421
747380	occ	69029	Crepidula p	species	81481
747448	occ	79786	Crucibulum	species	9704
747508	occ	79787	Crepidula f	species	94398
747509	occ	79787	Crepidula p	species	81481
747536	occ	79786	Crepidula f	species	94398
747614	occ	80037	Crepidula f	species	94398
747622	occ	80037	Crepidula s	species	156292
747643	occ	80037	Crucibulum	genus	9704
747703	occ	80044	Crepidula f	species	94398
747711	occ	80044	Crepidula s	species	156292
747750	occ	80046	Crepidula f	species	94398
747751	occ	80046	Crepidula p	species	81481
747795	occ	80046	Crucibulum	genus	9704
747798	occ	80046	Crepidula s	species	156292
748127	occ	69034	Crepidula f	species	9697
748136	occ	69034	Crepidula p	species	81481
748294	occ	80025	Crepidula a	species	81485
748298	occ	80025	Crucibulum	species	92421
748300	occ	80025	Crucibulum	species	9704
748331	occ	80024	Crucibulum	species	92421
748332	occ	80024	Calyptraea	species	81411
748341	occ	80024	Crepidula a	species	81485
748344	occ	80024	Crepidula p	species	81481
749388	occ	79331	Calyptraea	species	81411
749389	occ	79331	Crepidula c	species	95417

749390	occ	79331	Crepidula (	species	95456
749391	occ	79331	Crepidula (	species	95456
749392	occ	79331	Crepidula (	species	81482
749393	occ	79331	Crepidula (	species	81485
749466	occ	79336	Crepidula (	species	95456
751003	occ	80359	Calyptraea	species	81411
751015	occ	80358	Crepidula f	species	94398
751026	occ	80357	Crepidula c	species	94398
835861	occ	92693	Calyptraea	species	158723
853735	occ	94809	Crucibulum	species	156291
1088971	occ	96538	Bostrycapu	species	116296
1088972	occ	96538	Calyptraea	species	81411
1088973	occ	96538	Crepidula c	species	94519
1088974	occ	96538	Crepidula f	species	94398
1088975	occ	96538	Crepidula r	species	95417
1088976	occ	96538	Crepidula p	species	81481
1088977	occ	96538	Crucibulum	species	95428
1150709	occ	146872	Trochita flo	species	158723
1150710	occ	146872	Crucibulum	species	92421
1150712	occ	146872	Crucibulum	species	402832
1150713	occ	146872	Crucibulum	species	97657
1150714	occ	146872	Crepidula p	species	9697
1150715	occ	146872	Crepidula r	species	9697
1150716	occ	146872	Crepidula c	species	90749
1150717	occ	146872	Crepidula c	species	95440
1150718	occ	146872	Crepidula a	species	9697
1150719	occ	146872	Crepidula r	species	9697
1150720	occ	146872	Calyptraea	species	81411
1197529	occ	22712	Crepidula c	species	94398
256380	occ	20606	Crepidula f	species	94398
256427	occ	20613	Crepidula f	species	94398
578543	occ	60923	Crepidula f	species	94398
578612	occ	60927	Crepidula f	species	94398
578613	occ	60927	Crepidula p	species	81481
578683	occ	60928	Crepidula f	species	94398
578684	occ	60928	Crepidula p	species	81481
578777	occ	60930	Crepidula a	species	81485
578959	occ	60940	Calyptraea	species	81411
578960	occ	60940	Crucibulum	species	9704
578961	occ	60940	Crepidula f	species	94398
579047	occ	60944	Crepidula f	species	94398
579048	occ	60944	Crepidula p	species	81481
619853	occ	66760	Calyptraea	species	81411
619854	occ	66760	Crepidula f	species	94398
619855	occ	66758	Crepidula f	species	94398
619860	occ	66760	Crepidula p	species	81481
619865	occ	66760	Crepidula c	species	94519

619871	occ	66758	Crepidula a	species	81485
642858	occ	69046	Crepidula p	species	81481
642860	occ	69046	Crepidula f	species	94398
642902	occ	69046	Crepidula s	genus	9697
642945	occ	69046	Crucibulun	subgenus	92422
741381	occ	79707	Crepidula p	species	81481
745468	occ	79956	Crepidula p	species	81481
745488	occ	79957	Crepidula f	species	94398
745491	occ	79957	Calyptraea	species	81411
747820	occ	80053	Crepidula f	species	94398
747829	occ	80053	Crepidula a	species	81485
748099	occ	80083	Crepidula f	species	94398
748108	occ	80083	Calyptraea	species	81411
832872	occ	92407	Crepidula f	species	94398
1457454	occ	79767	Crypta forr	species	392481
1457459	occ	66757	Crypta acu	species	392482
564665	occ	58895	Calyptraea	species	81411
564666	occ	58895	Crepidula a	species	81485
564667	occ	58895	Crepidula f	species	94398
564668	occ	58895	Crepidula p	species	81481
564669	occ	58895	Crucibulun	species	82760
564897	occ	58898	Crepidula f	species	94398
564933	occ	58897	Crepidula a	species	81485
564934	occ	58897	Crepidula f	species	94398
564935	occ	58897	Crepidula p	species	81481
564936	occ	58897	Crepidula l	species	9697
578485	occ	60901	Crepidula f	species	94398
578488	occ	60903	Crepidula f	species	94398
579552	occ	60986	Crepidula f	species	94398
579553	occ	60986	Crepidula c	species	94519
579554	occ	60986	Crepidula p	species	81481
579647	occ	60989	Crepidula f	species	94398
579648	occ	60989	Crepidula c	species	94519
579649	occ	60989	Crepidula p	species	81481
579650	occ	60989	Crepidula s	genus	9697
579710	occ	60988	Crepidula f	species	94398
579711	occ	60988	Crepidula c	species	94519
579712	occ	60988	Crepidula p	species	81481
579785	occ	60987	Crepidula f	species	94398
579786	occ	60987	Crepidula c	species	94519
579787	occ	60987	Crepidula p	species	81481
619856	occ	66751	Crepidula f	species	94398
619857	occ	66749	Crepidula f	species	94398
619858	occ	66748	Crepidula f	species	94398
619859	occ	66742	Crepidula f	species	94398
619861	occ	66754	Crepidula p	species	81481
619862	occ	66751	Crepidula p	species	81481

619863	occ	66749	Crepidula p	species	81481
619864	occ	66742	Crepidula p	species	81481
619866	occ	66754	Crepidula c	species	94519
619867	occ	66750	Crepidula c	species	94519
619868	occ	66747	Crepidula c	species	94519
619869	occ	66744	Crepidula c	species	94519
619870	occ	66742	Crepidula c	species	94519
642709	occ	69045	Crepidula f	species	94398
642732	occ	69045	Crepidula p	species	81481
659689	occ	71138	Crepidula f	species	94398
659690	occ	71138	Crepidula p	species	81481
659730	occ	71138	Crucibulum	species	97657
659734	occ	71138	Crepidula a	species	81485
700975	occ	69043	Crepidula f	species	94398
701048	occ	69047	Crepidula p	species	81481
701049	occ	69047	Crepidula f	species	94398
702871	occ	68979	Calyptraea	genus	9583
702873	occ	68979	Crucibulum	genus	9704
702890	occ	69018	Calyptraea	genus	9583
702904	occ	69018	Crepidula f	species	94398
702911	occ	69018	Crepidula p	species	81481
737418	occ	79248	Crepidula s	species	9697
737430	occ	79248	Crucibulum	species	94757
737440	occ	79249	Crepidula s	species	156292
738420	occ	79342	Calyptraea	species	81411
738438	occ	79343	Crepidula f	species	94398
738446	occ	79343	Crucibulum	genus	9704
738470	occ	79341	Crucibulum	genus	9704
738471	occ	79341	Calyptraea	species	81411
738473	occ	79341	Crepidula a	species	81485
738496	occ	79339	Crepidula f	species	94398
738521	occ	79340	Crepidula f	species	94398
738544	occ	79340	Crucibulum	genus	9704
748223	occ	80034	Crepidula f	species	94398
748227	occ	80034	Crepidula p	species	81481
748234	occ	80034	Bostrycapu	species	116296
748244	occ	80034	Crucibulum	subgenus	92422
748382	occ	80033	Crepidula f	species	94398
748383	occ	80033	Crepidula p	species	81481
748387	occ	80033	Bostrycapu	species	116296
748450	occ	80033	Crucibulum	genus	9704
748559	occ	80035	Crepidula f	species	94398
748592	occ	80035	Bostrycapu	species	116296
748653	occ	80035	Crepidula p	species	81481
1512824	occ	211029	Crepidula f	species	94398
1512833	occ	211183	Crepidula f	species	94398
1512931	occ	211181	Crepidula f	species	94398

1512969	occ	211182	Crepidula f	species	94398
94775	occ	7109	Crepidula c	species	94519
559482	occ	57706	Crepidula c	species	94519
559483	occ	57706	Crepidula f	species	94398
559484	occ	57706	Crepidula p	species	81481
559824	occ	57705	Crepidula c	species	94519
559825	occ	57705	Crepidula f	species	94398
559826	occ	57705	Crepidula p	species	81481
559840	occ	57704	Crepidula f	species	94398
559841	occ	57704	Crepidula p	species	81481
559853	occ	57702	Crepidula c	species	94519
559854	occ	57702	Crepidula p	species	81481
582269	occ	61296	Crepidula f	species	94398
640361	occ	69048	Crepidula f	species	94398
640371	occ	69049	Crepidula c	species	94519
727661	occ	78271	Crepidula s	species	156292
727662	occ	78271	Crepidula p	species	81481
727663	occ	78271	Crepidula f	species	94398
727667	occ	78271	Crucibulum	species	94742
727747	occ	78270	Crepidula p	species	81481
727748	occ	78270	Crepidula f	species	94398
727816	occ	78269	Crepidula s	species	156292
727817	occ	78269	Crepidula p	species	81481
727818	occ	78269	Crepidula f	species	94398
727827	occ	78268	Crepidula s	species	156292
727832	occ	78268	Crucibulum	species	94742
727844	occ	78269	Crucibulum	species	94742
727864	occ	78267	Crepidula s	species	156292
727865	occ	78267	Crepidula f	species	94398
727880	occ	78266	Crepidula p	species	81481
727881	occ	78266	Crepidula f	species	94398
727886	occ	78266	Crucibulum	species	94742
727930	occ	78266	Crepidula s	species	156292
727941	occ	78265	Crepidula s	species	156292
727942	occ	78265	Crepidula p	species	81481
727943	occ	78265	Crepidula f	species	94398
727950	occ	78265	Crucibulum	species	94742
727986	occ	78260	Crepidula s	species	156292
727989	occ	78260	Crucibulum	species	94742
728006	occ	78261	Crepidula s	species	156292
728007	occ	78261	Crepidula p	species	81481
728008	occ	78261	Crepidula f	species	94398
728013	occ	78261	Crucibulum	species	9704
728014	occ	78261	Crucibulum	species	94742
728043	occ	78262	Crepidula s	species	156292
728044	occ	78262	Crepidula p	species	81481
728045	occ	78262	Crepidula f	species	94398

728050	occ	78262	Crucibulum	species	94742
728096	occ	78264	Crepidula s	species	156292
728097	occ	78264	Crepidula f	species	94398
728103	occ	78264	Crucibulum	species	94742
737449	occ	79246	Crepidula s	species	156292
737453	occ	79247	Crepidula s	species	156292
737499	occ	79245	Crucibulum	species	94757
737500	occ	79245	Crepidula s	species	9697
741436	occ	79709	Crucibulum	species	94757
741465	occ	79709	Crepidula s	species	156292
741469	occ	79709	Crepidula f	species	94398
748185	occ	80040	Bostrycapu	species	116296
748186	occ	80040	Crepidula r	species	9697
748251	occ	80038	Crepidula a	species	81485
748266	occ	80038	Crucibulum	subgenus	92422
748416	occ	80036	Crepidula a	species	81485
748417	occ	80036	Crucibulum	genus	9704
747386	occ	69029	Cerithium	species	96763
748305	occ	80025	Thericium	species	10574
749396	occ	79331	Cerithium	species	96763
749397	occ	79331	Cerithium	species	96763
749400	occ	79331	Cerithium	species	96763
749401	occ	79338	Cerithium	species	96763
749402	occ	79331	Cerithium	species	96763
749406	occ	79338	Cerithium	species	96763
749455	occ	79333	Cerithium	species	96763
749457	occ	79333	Cerithium	species	96763
749459	occ	79333	Cerithium	species	96763
749467	occ	79336	Cerithium	species	96763
1150803	occ	146875	Thericium	genus	10574
426117	occ	41501	Cancellaria	species	107354
426125	occ	41501	Colubraria	species	91557
427466	occ	41705	Cancellaria	species	107354
427757	occ	41718	Cancellaria	species	107354
428049	occ	41749	Cancellaria	genus	10889
428050	occ	41749	Cancellaria	species	120336
428051	occ	41749	Cancellaria	species	93811
428060	occ	41749	Colubraria	species	91557
567357	occ	59485	Cancellaria	species	120336
567358	occ	59484	Cancellaria	species	120336
567359	occ	59490	Cancellaria	species	120336
567360	occ	59485	Cancellaria	species	107271
567361	occ	59484	Cancellaria	species	107749
567362	occ	59487	Cancellaria	species	120365
568801	occ	59484	Cancellaria	species	107271
568802	occ	59486	Cancellaria	species	97423
622901	occ	67070	Trigonosto	species	91138

623089	occ	67076	Cancellaria	species	120336
623090	occ	67076	Cancellaria	species	93811
623095	occ	67077	Cancellaria	species	120336
697883	occ	74999	Cancellaria	species	107354
697963	occ	75012	Cancellaria	species	93811
741269	occ	79331	Cancellaria	species	107355
741270	occ	79331	Cancellaria	species	93812
741271	occ	79331	Perplicaria	species	250430
741272	occ	79331	Trigonosto	species	120368
741273	occ	79331	Trigonosto	species	11783
741311	occ	79334	Cancellaria	species	107355
741333	occ	79333	Cancellaria	species	107355
741334	occ	79333	Cancellaria	species	120332
741991	occ	79748	Ventrilia ru	species	167563
742181	occ	79765	Cancellaria	species	93810
742188	occ	79765	Trigonosto	species	93815
742194	occ	79765	Trigonosto	species	93818
742196	occ	79765	Perplicaria	species	250430
742200	occ	79766	Trigonosto	species	93815
742219	occ	79335	Trigonosto	species	93819
742476	occ	79762	Cancellaria	species	107750
742493	occ	79765	Trigonosto	species	11783
742504	occ	79761	Cancellaria	species	107750
742582	occ	79782	Cancellaria	species	10889
743280	occ	79805	Cancellaria	species	10889
743303	occ	79804	Cancellaria	species	107354
743304	occ	79804	Perplicaria	species	250430
743720	occ	79847	Cancellaria	species	107354
744358	occ	79875	Cancellaria	species	10889
744407	occ	79874	Cancellaria	species	10889
744418	occ	79874	Ventrilia ki	species	167564
745430	occ	69040	Cancellaria	species	107271
745593	occ	79960	Ventrilia al	species	11834
745638	occ	79960	Ventrilia sr	species	11834
745684	occ	79331	Colubraria	species	91557
745738	occ	79959	Ventrilia se	species	11834
745739	occ	79959	Trigonosto	species	11783
745762	occ	79959	Ventrilia ca	species	11834
745763	occ	79959	Extractrix I	species	93813
745764	occ	79959	Cancellaria	species	108725
747327	occ	69029	Cancellaria	genus	10889
747328	occ	69029	Agatrix sp.	genus	10731
747384	occ	69029	Cancellaria	genus	10889
747431	occ	79786	Cancellaria	species	108725
747541	occ	79786	Cancellaria	species	108725
747632	occ	80037	Trigonosto	species	11783
789718	occ	85448	Cancellaria	species	107749

834792	occ		92740	Cancellaria	species	107749
870204	occ		79960	Trigonosto	species	11783
870206	occ		79960	Trigonosto	genus	11783
1089044	occ		96538	Cancellaria	species	107271
1150740	occ		146873	Extractrix s	genus	93813
1150741	occ		146873	Massyla sp	genus	11328
1150775	occ		146875	Cancellaria	genus	10889
1150776	occ		146875	Extractrix s	genus	93813
1150777	occ		146875	Massyla sp	genus	11328
1150779	occ		146875	Emmonsel	genus	93816
1150780	occ		146875	Trigonosto	genus	11783
1331697	occ		179337	Cancellaria	species	108725
578680	occ		60928	Cancellaria	species	107271
620031	occ		66760	Cancellaria	species	107271
1457435	occ		79745	Cancellaria	species	107271
567215	occ		59307	Cancellaria	species	108725
737426	occ		79249	Trigonosto	genus	11783
748443	occ		80033	Cancellaria	species	108725
728064	occ		78262	Trigonosto	genus	11783
737506	occ		79245	Trigonosto	genus	11783
748422	occ		80036	Cancellaria	genus	10889
748425	occ		80036	Cancellarii	family	59518
747424	occ		79786	Capulus sp	genus	9594
567681	occ		59486	Sconsia ho	species	250603
622897	occ		67070	Phalium in	species	10270
741891	occ	27805	79739	Cassis mad	species	134864
742013	occ		79747	Cassis jame	species	9604
742014	occ		79747	Cassis schr	species	9604
742015	occ		79747	Phalium lo	species	159038
744394	occ		79874	Cassis kett	species	251416
745573	occ		79960	Sconsia me	species	250604
745749	occ		79959	Cassis flori	species	251404
745782	occ		79959	Sconsia ho	species	250603
836647	occ		92624	Cassis kett	species	251416
870253	occ		79960	Cassis flori	species	251404
1085509	occ		35031	Sconsia pro	species	250632
1086459	occ		35025	Semicassis	species	156568
1086461	occ		35031	Semicassis	species	156568
1088990	occ		96538	Cassis mad	species	134864
1088991	occ		96538	Semicassis	species	250755
578967	occ		60940	Sconsia ho	species	250603
567083	occ		59307	Sconsia ho	species	250603
567084	occ		59310	Sconsia ho	species	250603
362438	occ		34269	Cerithium	species	109117
362439	occ		34269	Cerithium	genus	9632
362440	occ		34269	Cerithium	species	94534
425341	occ		41497	Cerithium	species	109148

426000	occ	41501	Alabina sp.	species	9441
426006	occ	41501	Bittium po	species	9553
426007	occ	41501	Bittium sp.	species	9553
426008	occ	41501	Bittium vai	species	156816
426009	occ	41501	"Bittium" s	genus	9553
426120	occ	41501	Cerithium (	species	9632
426121	occ	41501	Cerithium l	species	9632
426122	occ	41501	Cerithium :	genus	9632
427445	occ	41705	Bittium vai	species	156816
427446	occ	41705	"Bittium" s	genus	9553
427468	occ	41705	Cerithium l	species	9632
427469	occ	41705	Cerithium :	genus	9632
427470	occ	41705	Cerithium '	species	134876
427530	occ	41705	Rhinoclavis	species	62110
427751	occ	41718	Bittium vai	species	156816
427760	occ	41718	Cerithium (	species	9632
427761	occ	41718	Cerithium l	species	9632
427762	occ	41718	Cerithium :	genus	9632
427926	occ	41740	Cerithium ı	species	94534
428055	occ	41749	Cerithium (	species	9632
428056	occ	41749	Cerithium (	species	9632
428057	occ	41749	Cerithium '	species	134876
428111	occ	41749	Rhinoclavis	species	62110
428112	occ	41749	Rhinoclavis	genus	62110
432399	occ	41705	Cerithium :	species	9632
568121	occ	59484	Cerithium '	species	109117
568122	occ	59484	"Cerithium	species	9632
582369	occ	61311	Cerithium :	genus	9632
582370	occ	61311	Diastoma v	species	156527
582405	occ	61310	Cerithium :	genus	9632
582406	occ	61310	Cerithium ı	species	94534
640514	occ	69085	Cerithium ı	species	94534
640541	occ	69084	Cerithium ı	species	94534
640734	occ	69087	Cerithium :	genus	9632
640741	occ	69087	Cerithium :	genus	9632
647833	occ	70010	Cerithium ı	species	94534
697879	occ	74999	Cerithium (	species	9632
697907	occ	75001	Cerithium :	genus	9632
697919	occ	75002	Cerithium ;	species	9632
697976	occ	75013	Cerithium ;	species	9632
698003	occ	75016	Cerithium :	genus	9632
698023	occ	75007	Cerithium ;	species	9632
698028	occ	75008	Cerithium ;	species	9632
741882	occ	79739	Cerithium ı	species	94534
741883	occ	79739	Cerithium ;	species	109148
741884	occ	79739	Cerithium '	species	109117
743220	occ	79804	Cerithiocla	species	9623

743719	occ		79847	Cerithiocla	species	9623
745336	occ		79789	Cerithium	species	94534
745413	occ		79788	Cerithium	species	94534
745435	occ		69040	Cerithium	species	94534
745844	occ		79958	Cerithiocla	species	9623
745946	occ		79958	Bayericerit	species	120722
745947	occ		79958	Cerithium	species	250661
745948	occ		79958	Cerithium	species	9632
747012	occ		80002	Cerithium	species	94534
747037	occ		80001	Cerithium	species	94534
747258	occ		80027	Cerithium	species	94534
747298	occ		69044	Rhinoclavis	genus	62110
747309	occ		69044	Cerithium	genus	9632
747442	occ		79786	Cerithium	species	109116
747443	occ		79786	Cerithiocla	species	9623
747582	occ		80030	Cerithium	genus	9632
747796	occ		80046	Cerithium	species	109116
747797	occ		80046	Cerithium	species	109148
747799	occ		80046	Cerithium	genus	9632
748146	occ		69034	Cerithium	species	109149
749395	occ		79331	Cerithium	species	134876
749398	occ		79331	Cerithium	species	94534
749399	occ		79338	Cerithium	species	94534
749403	occ		79338	Cerithium	species	94534
749404	occ		79338	Cerithium	species	9632
749405	occ		79331	Cerithium	species	9632
749407	occ		79338	Cerithium	species	9632
749408	occ		79338	Rhinoclava	species	10203
749412	occ		79331	Bittium (Bi	species	116068
749413	occ		79338	Rhinoclava	species	10203
749458	occ		79333	Cerithium	species	94534
749460	occ		79333	Cerithium	species	9632
1088936	occ		96538	Bittiolum v	species	156526
1088937	occ		96538	Cerithium	species	109116
1088938	occ		96538	Cerithium	species	109149
1088939	occ		96538	Cerithium	species	109318
1088940	occ		96538	Cerithium	species	109324
1088941	occ		96538	Cerithium	species	94534
1150801	occ		146875	Bittium sp.	genus	9553
1150802	occ		146875	Ochetoclav	genus	10203
1150804	occ		146875	Cerithiocla	genus	9623
564927	occ		58897	Bittium po	species	9553
578477	occ		60899	Bittium cf.	species	9553
642734	occ		69045	Cerithium	species	109149
559855	occ		57702	Diastoma a	species	156637
426119	occ	21062	41501	Retilaskeya	species	134872
427630	occ		41715	Seila adam	species	108707

427809	occ		41718	Seila adam	species	108707
568116	occ		59491	Cerithiopsi	genus	9630
568117	occ		59484	Cerithiopsi	genus	9630
568118	occ		59484	Cerithiopsi	genus	9630
568119	occ		59487	Seila clavul	species	10445
568120	occ		59484	Seila clavul	species	10445
582371	occ		61311	Seila adam	species	108707
582386	occ		61309	Seila adam	species	108707
582404	occ	21067	61310	Retilaskeya	species	134872
582407	occ		61310	Seila adam	species	108707
749410	occ		79338	Seila adam	species	108707
749415	occ		79338	Seila adam	species	108707
749436	occ		79331	Cerithiopsi	species	90926
749437	occ		79331	Cerithiopsi	species	90927
749438	occ		79331	Cerithiopsi	species	157328
749439	occ		79331	Cerithiopsi	species	157327
749440	occ		79331	Cerithiopsi	species	157327
749441	occ		79331	Cerithiopsi	species	157327
749442	occ	21071	79331	Retilaskeya	species	134872
749443	occ		79331	Seila adam	species	108707
1088994	occ		96538	Cerithiopsi	species	134875
1088995	occ		96538	Seila adam	species	90921
578546	occ		60923	Seila adam	species	108707
578691	occ		60928	Seila adam	species	108707
578692	occ		60928	Triphora pı	species	109786
619883	occ	21075	66756	Cerithiopsi	species	90924
619884	occ		66758	Seila adam	species	108707
832911	occ		92408	Seila adam	species	108707
564632	occ		58894	Seila adam	species	108707
564687	occ		58895	Seila adam	species	108707
564889	occ		58898	Seila adam	species	108707
564955	occ		58897	Seila adam	species	108707
567057	occ	21069	59315	Retilaskeya	species	134872
567058	occ	21070	59307	Retilaskeya	species	134872
579489	occ		60986	Seila adam	species	108707
579641	occ		60989	Cerithiopsi	species	90928
579642	occ		60989	Seila adam	species	108707
579702	occ	21063	60988	Retilaskeya	species	134872
579703	occ		60988	Cerithiopsi	species	90928
579704	occ		60988	Seila adam	species	108707
579781	occ	18720	60987	Cerithiopsi	species	90920
579782	occ		60987	Seila adam	species	108707
619882	occ	21074	66742	Retilaskeya	species	134872
659723	occ		71138	Seila adam	species	90921
559821	occ		57705	Cerithiopsi	species	134875
559839	occ		57704	Cerithiopsi	species	134875
559852	occ		57702	Cerithiopsi	species	134875

727779	occ	78269	Seila adam	species	108707
727895	occ	78266	Seila adam	species	90921
727957	occ	78265	Seila adam	species	90921
728023	occ	78261	Seila adam	species	90921
728060	occ	78262	Seila adam	species	90921
728109	occ	78264	Seila adam	species	90921
426153	occ	41501	Glyphostoı	species	11160
426154	occ	41501	Glyphostoı	species	11160
426184	occ	41501	Nannodiell	genus	107559
567355	occ	59485	Glyphostoı	species	11160
567356	occ	59484	Glyphostoı	species	11160
750874	occ	79331	Glyphostoı	species	11160
750875	occ	79331	Glyphostoı	species	11160
750876	occ	79331	Glyphostoı	species	11160
750877	occ	79331	Glyphostoı	species	11160
750878	occ	79331	Glyphostoı	species	11160
750880	occ	79331	Lioglyphos	species	162807
750881	occ	79331	Glyphostoı	species	121447
750882	occ	79331	Nannodiell	species	107559
1150791	occ	146875	Glyphostoı	genus	11160
567238	occ	59307	Glyphostoı	species	11160
567239	occ	59310	Glyphostoı	species	11160
567240	occ	59316	Glyphostoı	species	11160
567217	occ	59316	Clavatula c	species	108733
567218	occ	59313	Clavatula c	species	108733
567219	occ	59307	Clavatula c	species	108733
426124	occ	41501	Clathrodril	species	120665
427472	occ	41705	Clathrodril	species	120665
427763	occ	41718	Clathrodril	species	120665
582484	occ	61309	Crassispira	species	156523
582485	occ	61309	Drillia acur	species	91226
582515	occ	61311	Crassispira	species	156523
1089052	occ	96538	Pyrgospira	species	156524
362430	occ	34269	Columbella	species	135663
426001	occ	41501	Anachis ca	species	10755
426002	occ	41501	Anachis ga	species	82768
427440	occ	41705	Anachis ca	species	10755
427441	occ	41705	Anachis ga	species	82768
427442	occ	41705	Anachis sp	genus	10755
427616	occ	41715	Anachis ga	species	82768
427750	occ	41718	Anachis ca	species	10755
427922	occ	41740	Anachis ob	species	10755
427923	occ	41740	Anachis ? s	species	10755
428039	occ	41749	Anachis ca	species	10755
428061	occ	41749	Columbella	genus	10947
567651	occ	59484	Anachis ca	species	10755
567652	occ	59484	Anachis wi	species	10755

567653	occ	59484	Strombina	species	11712
568815	occ	59486	Anachis ca	species	10755
568816	occ	59484	Strombina	species	11712
582430	occ	61309	Mitrella lu	species	94415
582431	occ	61309	Anachis ob	species	94412
582432	occ	61309	Anachis sp	genus	10755
582454	occ	61310	Anachis sp	genus	10755
582455	occ	61310	Mitrella lu	species	94415
582468	occ	61311	Anachis ob	species	94412
582469	occ	61311	Mitrella lu	species	94415
640749	occ	69087	Strombina	subgenus	89604
647838	occ	70010	Costoanacl	genus	10978
697881	occ	74999	Mitrella sp	genus	11368
697946	occ	75011	Mitrella sp	genus	11368
741348	occ	79331	Columbella	species	88864
741349	occ	79331	Strombina	species	237709
741350	occ	79331	Strombina	species	11712
741902	occ	79739	Columbella	species	88864
741953	occ	79740	Eurypyrene	species	88870
745643	occ	79331	Anachis (C	species	94411
745644	occ	79331	Anachis (C	species	94409
745645	occ	79331	Anachis (C	species	94409
745646	occ	79331	Anachis (C	species	94409
745647	occ	79331	Anachis (M	species	10755
745648	occ	79331	Anachis (A	species	82768
745649	occ	79331	Anachis (A	species	134955
745650	occ	79331	Anachis (A	species	134954
745652	occ	79331	Nassarina (	species	156599
745653	occ	79331	Aesopus (G	species	10724
745654	occ	79331	Aesopus (I	species	10724
745668	occ	79333	Anachis (M	species	10755
745669	occ	79333	Aesopus (G	species	10724
745732	occ	79959	Parametar	species	11481
745929	occ	79958	Parametar	species	11481
745956	occ	79958	Columbella	species	88863
747013	occ	80002	Columbella	species	88864
747038	occ	80001	Columbella	species	88864
747312	occ	69044	Costoanacl	genus	10978
747548	occ	79786	Costoanacl	genus	10978
747573	occ	80030	Anachis da	species	10755
747595	occ	80029	Anachis da	species	10755
747805	occ	80046	Columbella	species	88864
870272	occ	35031	Eurypyrene	genus	88870
1066564	occ	35025	Columbella	species	88864
1068778	occ	35025	Sincola (Sir	species	11670
1089028	occ	96538	Astyris luna	species	94414
1089029	occ	96538	Columbella	species	109114

1089030	occ	96538	Columbella	species	88864
1089031	occ	96538	Costoanacl	species	108675
1089032	occ	96538	Nitidella ni	species	88922
1089033	occ	96538	Parvanachi	species	94438
1150737	occ	146873	Parametar	species	11481
578544	occ	60923	Mitrella lu	species	94415
578554	occ	60923	Anachis av	species	108676
578611	occ	60927	Anachis av	species	108676
578679	occ	60928	Anachis av	species	108676
578685	occ	60928	Mitrella lu	species	94415
578775	occ	60930	Anachis av	species	108676
578784	occ	60930	Mitrella lu	species	94415
579045	occ	60944	Columbella	species	135663
579055	occ	60944	Mitrella lu	species	11368
619902	occ	66760	Columbella	species	94411
619903	occ	66758	Columbella	species	94411
619904	occ	66756	Columbella	species	94411
619910	occ	66758	Columbella	species	135663
619911	occ	66760	Columbella	species	135663
619913	occ	66760	Columbella	species	94417
619914	occ	66758	Columbella	species	94417
741414	occ	79705	Mitrella sp	genus	11368
832881	occ	92407	Columbella	species	135663
1457456	occ	66757	Columbella	species	392387
1457479	occ	203377	Columbella	species	392386
564656	occ	58895	Aesopus ga	species	82800
564657	occ	58895	Aesopus st	species	10724
564658	occ	58895	Anachis mi	species	10755
564920	occ	58897	Aesopus ga	species	82800
564921	occ	58897	Aesopus st	species	10724
564922	occ	58897	Anachis mi	species	10755
564948	occ	58897	Mitrella wa	species	11368
567107	occ	59330	Mitrella lu	species	94415
567108	occ	59307	Mitrella lu	species	94415
567109	occ	59310	Mitrella lu	species	94415
567110	occ	59313	Mitrella lu	species	94415
567111	occ	59316	Mitrella lu	species	94415
567112	occ	59317	Mitrella lu	species	94415
567113	occ	59316	Mitrella wa	species	11368
567114	occ	59307	Anachis (Tl	species	116650
567115	occ	59309	Anachis (Tl	species	116650
567116	occ	59313	Anachis (C	species	108676
567117	occ	59307	Anachis (C	species	108676
567118	occ	59317	Anachis (C	species	108676
567119	occ	59316	Anachis (C	species	108676
567120	occ	59318	Anachis (C	species	108676
567121	occ	59330	Anachis (C	species	108676

567127	occ	59316	Anachis (C	species	94409
567128	occ	59307	Strombina	species	237707
567129	occ	59315	Aesopus st	species	10724
567130	occ	59316	Aesopus st	species	10724
567131	occ	59307	Aesopus st	species	10724
578476	occ	60899	Anachis ga	species	82768
579556	occ	60986	Anachis laf	species	156800
579557	occ	60986	Anachis ob	species	94412
579558	occ	60986	Anachis sp	genus	10755
579559	occ	60986	Mitrella lu	species	94415
579653	occ	60989	Anachis laf	species	156800
579654	occ	60989	Anachis ob	species	94412
579655	occ	60989	Mitrella lu	species	94415
579715	occ	60988	Anachis laf	species	156800
579716	occ	60988	Anachis ob	species	94412
579717	occ	60988	Mitrella lu	species	94415
579790	occ	60987	Anachis laf	species	156800
579791	occ	60987	Anachis ob	species	94412
579792	occ	60987	Mitrella lu	species	94415
619905	occ	66752	Columbella	species	94411
619906	occ	66751	Columbella	species	94411
619907	occ	66751	Columbella	species	135663
619908	occ	66750	Columbella	species	135663
619909	occ	66742	Columbella	species	135663
619912	occ	66742	Columbella	species	94417
619915	occ	66752	Columbella	species	94417
619916	occ	66751	Columbella	species	94417
619917	occ	66750	Columbella	species	94417
659714	occ	71138	Anachis cf.	species	108676
738428	occ	79342	Anachis (Tl	species	10755
748460	occ	80033	Strombina	genus	11712
748477	occ	80033	Anachis (C	species	94409
748550	occ	80035	Strombina	genus	11712
1512830	occ	211029	Anachis laf	species	156800
1512831	occ	211029	Anachis ob	species	94412
1512842	occ	211183	Anachis laf	species	156800
1512848	occ	211183	Anachis ob	species	94412
1512937	occ	211181	Anachis ob	species	94412
1512938	occ	211181	Anachis laf	species	156800
1512979	occ	211182	Anachis ob	species	94412
1512980	occ	211182	Anachis laf	species	156800
559490	occ	57706	Mitrella lu	species	94415
559817	occ	57705	Anachis laf	species	156800
559837	occ	57704	Anachis ob	species	94412
559848	occ	57702	Anachis laf	species	156800
640348	occ	69048	Astyris lun	species	94414
640360	occ	69048	Parvanachi	species	94438

640374	occ		69049	Astyris lun	species	94414
727772	occ		78269	Mitrella sp	genus	11368
727849	occ		78269	Anachis cf.	species	10755
727850	occ		78269	Mitrella co	species	116690
727888	occ		78266	Anachis sp	genus	10755
727889	occ		78266	Mitrella co	species	116690
727890	occ		78266	Mitrella sp	genus	11368
727953	occ		78265	Mitrella co	species	116690
727954	occ		78265	Mitrella sp	genus	11368
727990	occ		78260	Mitrella co	species	11368
728018	occ		78261	Mitrella co	species	116690
728054	occ		78262	Anachis ha	species	10755
728055	occ		78262	Mitrella co	species	116690
728105	occ		78264	Mitrella sp	genus	11368
741456	occ		79709	Astyris con	species	108019
741458	occ		79709	Costoanacl	species	108675
426096	occ		41539	Conus stea	species	119995
426126	occ		41501	Conus adv	species	116721
426127	occ		41501	Conus dau	species	147375
426128	occ		41501	Conus flori	species	107791
426129	occ		41501	Conus stea	species	119995
427473	occ		41705	Conus stea	species	119995
427620	occ		41715	Conus flori	species	107791
427765	occ		41718	Conus stea	species	119995
428062	occ		41749	Conus adv	species	116721
428063	occ		41749	Conus cf. d	species	147375
428064	occ		41749	Conus flori	species	107791
428065	occ		41749	Conus spu	species	188685
428066	occ		41749	Conus wac	species	10963
567334	occ		59485	Conus adv	species	116721
567335	occ		59485	Conus flori	species	107791
567336	occ		59487	Conus adv	species	116721
567338	occ		59484	Conus flori	species	107791
568796	occ		59484	Conus adv	species	116721
568797	occ	21779	59484	Conus patr	species	107767
568798	occ		59484	Conus harv	species	119971
582483	occ		61309	Conus jasp	species	107770
582499	occ		61310	Conus jasp	species	107770
582514	occ		61311	Conus jasp	species	107770
591050	occ		62465	Jaspidiconu	species	82798
640540	occ		69084	Conus jasp	species	107770
640756	occ		69087	Conus sp.	genus	10963
697964	occ		75012	Conus sp.	genus	10963
698031	occ		75008	Conus adv	species	116721
741263	occ		79331	Conus (Cor	species	116722
741264	occ		79331	Conus (Lith	species	95697
741265	occ		79331	Conus (Lith	species	147377

741266	occ	79331	Conus (Lep	species	93793
741267	occ	79331	Conus (Lep	species	120662
741268	occ	79331	Conus (Lep	species	95705
741305	occ	79334	Conus (Cor	species	116722
741308	occ	79334	Conus (Lep	species	93793
741309	occ	79334	Conus (Lep	species	120662
741310	occ	79334	Conus (Lep	species	95705
741313	occ	79335	Conus (Cor	species	116722
741314	occ	79335	Conus (Lith	species	95697
741316	occ	79335	Conus (Lep	species	95705
741328	occ	79333	Conus (Lith	species	95697
741329	occ	79333	Conus (Lep	species	93793
741330	occ	79333	Conus (Lep	species	120662
741331	occ	79333	Conus (Lep	species	95705
741332	occ	79333	Conus (Lep	species	95705
741875	occ	79738	Jaspidiconu	species	82797
741898	occ	79739	Gradiconus	species	120716
741907	occ	79739	Jaspidiconu	species	120016
741908	occ	79739	Spuriconus	species	188684
741943	occ	79740	Spuriconus	species	147356
741944	occ	79740	Spuriconus	species	147353
741970	occ	79749	Ximenicon	species	147389
742034	occ	79747	Cariboconu	species	147379
742035	occ	79747	Gradiconus	species	147514
742036	occ	79747	Gradiconus	species	147512
742037	occ	79747	Jaspidiconu	species	119998
742038	occ	79747	Jaspidiconu	species	120001
742048	occ	79747	Ximenicon	species	147389
742197	occ	79766	Conus drui	species	93795
742578	occ	79782	Ximenicon	species	147389
742579	occ	79782	Contraconu	species	147420
742580	occ	79782	Contraconu	species	147424
742581	occ	79782	Contraconu	species	147426
743289	occ	79805	Contraconu	species	147423
743290	occ	79805	Dauciconus	species	147385
743291	occ	79805	Gradiconus	species	120716
743310	occ	79804	Contraconu	species	147423
743311	occ	79804	Contraconu	species	147419
743312	occ	79804	Gradiconus	species	120716
743314	occ	79804	Seminoleco	species	119968
743315	occ	79804	Ximenicon	species	147389
743709	occ	79847	Contraconu	species	147419
743713	occ	79847	Ximenicon	species	147389
744301	occ	79876	Contraconu	species	147427
744315	occ	79876	Jaspidiconu	species	120000
744359	occ	79875	Spuriconus	species	147374
744405	occ	79874	Spuriconus	species	119969

744406	occ	79874	Spuriconus	species	119969
745340	occ	79789	Conus wac	species	10963
745402	occ	79788	Conus wac	species	10963
745560	occ	79960	Contraconu	genus	147416
745578	occ	79960	Seminoleco	species	119968
745587	occ	79960	Contraconu	species	147425
745588	occ	79960	Jaspidiconu	species	119976
745735	occ	79959	Jaspidiconu	species	82797
745736	occ	79959	Eugeniconu	species	119970
745737	occ	79959	Dauciconus	species	147384
745741	occ	79959	Lithoconus	species	11285
745742	occ	79959	Spuriconus	species	147349
745752	occ	79959	Seminoleco	species	147517
745761	occ	79959	Contraconu	species	147417
745795	occ	79959	Jaspidiconu	species	119997
745796	occ	79959	Gradiconus	species	147431
745939	occ	79958	Jaspidiconu	species	119996
745940	occ	79958	Contraconu	species	147421
745941	occ	79958	Eugeniconu	species	119970
745942	occ	79958	Spuriconus	species	119969
745943	occ	79958	Virgiconus	species	120720
747260	occ	80027	Conus jasp	species	107770
747363	occ	69029	Conus jasp	species	107770
747481	occ	79787	Conus (Cor	species	95696
747537	occ	79786	Conus (Cor	species	95696
747782	occ	80046	Conus ever	species	10963
747794	occ	80046	Conus sp.	genus	10963
748144	occ	69034	Conus jasp	species	107770
751014	occ	80358	Conus stea	species	120662
751027	occ	80357	Conus stea	species	120662
789716	occ	85448	Conus sp.	genus	10963
789720	occ	85448	Conus adve	species	116721
834787	occ	92739	Conus adve	species	116721
834794	occ	92740	Conus adve	species	116721
870178	occ	79960	Contraconu	species	147417
870240	occ	79960	Conus (Lith	species	147522
870256	occ	35025	Contraconu	species	147419
870263	occ	35031	Conus (Lith	species	147347
870264	occ	35031	Conus cf. e	species	10963
870270	occ	35031	Conus (Lep	species	147430
870273	occ	35031	Conus (Lep	species	95705
870285	occ	35031	Conus (Cor	species	147394
1011562	occ	123549	Conus adve	species	116721
1089053	occ	96538	Conus cf. la	species	147507
1089054	occ	96538	Conus jasp	species	107770
1089055	occ	96538	Conus spur	species	107772
1150765	occ	146874	Conus (Virg	species	120719

1150781	occ	146875	Conus (Lith	species	147519
1150782	occ	146875	Conus (Lith	species	147348
1150783	occ	146875	Contracon	species	147417
578682	occ	60928	Conus sp.	genus	10963
578981	occ	60940	Conus adv	species	116721
578982	occ	60940	Conus mar	species	93793
578983	occ	60940	Conus pres	species	120118
579046	occ	60944	Conus jasp	species	107770
642889	occ	69046	Conus sp.	genus	10963
832880	occ	92407	Conus adv	species	116721
737411	occ	79249	Conus sp.	genus	10963
748487	occ	80033	Conus sp.	genus	10963
748643	occ	80035	Conus sp.	genus	10963
566301	occ	59297	Conus mar	species	93793
727809	occ	78269	Conus mar	species	93793
727926	occ	78266	Conus mar	species	93793
870229	occ	96535	Contracon	species	147417
567625	occ	59484	Mitra wan	species	157305
741282	occ	79331	Vexillum (U	species	107873
741283	occ	79331	Vexillum (U	species	107873
741284	occ	79331	Vexillum (U	species	107873
741285	occ	79331	Vexillum (U	species	164664
741286	occ	79331	Vexillum (U	species	107873
743317	occ	79804	Knefastia li	species	188699
832908	occ	92408	Mitra wan	species	157305
1457436	occ	79745	Volutomitr	species	157303
564695	occ	58895	Vexillum w	species	157304
564967	occ	58897	Vexillum w	species	157304
748543	occ	80033	Uromitra s	genus	11823
426137	occ	41501	Cylichnella	species	11924
426138	occ	41501	Cylichnella	species	11924
426204	occ	41501	Retusa can	species	108177
427478	occ	41705	Cylichnella	species	11924
427529	occ	41705	Retusa can	species	108177
427629	occ	41715	Retusa can	species	108177
427808	occ	41718	Retusa can	species	108177
427941	occ	41740	Retusa can	species	108177
567320	occ	59490	Acteocina	species	91361
567321	occ	59484	Acteocina	species	91361
567322	occ	59487	Acteocina	species	91361
567323	occ	59485	Acteocina	species	91361
567325	occ	59484	Cylichnella	species	11924
567326	occ	59485	Cylichnella	species	11924
567327	occ	59487	Cylichnella	species	11924
568795	occ	59484	Bullaria sp.	genus	98733
582489	occ	61309	Acteocina	species	91363
582516	occ	61311	Acteocina	species	91363

582538	occ	61310	Acteocina	species	91363
582539	occ	61310	Cylichnella	species	90940
697911	occ	75001	Acteocina	genus	11900
738382	occ	79331	Acteocina	species	91363
738383	occ	79331	Acteocina	species	11900
738393	occ	79336	Acteocina	species	91361
738394	occ	79336	Cylichnella	species	11924
747036	occ	80001	Acteocina	species	91361
751002	occ	80359	Acteocina	species	91361
751012	occ	80358	Acteocina	species	91361
751044	occ	80356	Acteocina	species	91361
751045	occ	80355	Acteocina	species	91361
1089063	occ	96538	Acteocina	species	91361
1089064	occ	96538	Acteocina	species	91363
619440	occ	66724	Retusa can	species	108177
578690	occ	60928	Retusa can	species	108177
578741	occ	60929	Retusa can	species	108177
579061	occ	60944	Retusa can	species	108177
620032	occ	66760	Retusa can	species	108177
620033	occ	66758	Retusa can	species	108177
741371	occ	79705	Acteocina	species	91361
741389	occ	79706	Acteocina	species	91361
741395	occ	79707	Acteocina	species	91361
832901	occ	92408	Acteocina	species	91361
564655	occ	58895	Acteocina	species	91363
564919	occ	58897	Acteocina	species	91363
567251	occ	59316	Acteocina	species	91361
567252	occ	59307	Cylichna du	species	11921
579568	occ	60986	Acteocina	species	91363
620034	occ	66752	Retusa can	species	108177
620035	occ	66751	Retusa can	species	108177
620036	occ	66750	Retusa can	species	108177
620037	occ	66748	Retusa can	species	108177
620038	occ	66747	Retusa can	species	108177
620039	occ	66742	Retusa can	species	108177
659737	occ	71138	Acteocina	species	91361
559475	occ	57706	Acteocina	species	91361
559816	occ	57705	Acteocina	species	91361
559836	occ	57704	Acteocina	species	91361
559847	occ	57702	Acteocina	species	91361
582225	occ	61293	Acteocina	species	91363
727745	occ	78270	Acteocina	species	91363
727807	occ	78269	Acteocina	species	91363
727924	occ	78266	Acteocina	species	91363
727935	occ	78265	Acteocina	species	91363
727984	occ	78260	Cylichna cy	species	11921
727997	occ	78261	Acteocina	species	91363

727998	occ	78261	Cylichna cy	species	11921
728091	occ	78264	Acteocina	species	91363
728092	occ	78264	Cylichna sp	genus	11921
426141	occ	41501	Cypraea pr	species	342576
427770	occ	41718	Cypraea pr	species	342576
428076	occ	41749	Cypraea ca	species	335331
567683	occ	59484	Cypraea ca	species	335331
622887	occ	67070	Cypraea ce	species	156624
623096	occ	67077	Cypraea ca	species	335331
741890	occ	79739	Macrocypr	species	156625
742005	occ	79747	Luria volek	species	342051
742006	occ	79747	Macrocypr	species	343131
742007	occ	79747	Macrocypr	species	343176
742008	occ	79747	Pseudozon	species	249773
742520	occ	79335	Siphocypra	species	111875
742565	occ	79782	Siphocypra	species	342628
743221	occ	79804	Siphocypra	species	111875
743222	occ	79804	Siphocypra	species	343470
743223	occ	79804	Siphocypra	species	343123
743241	occ	79805	Siphocypra	species	111875
743242	occ	79805	Siphocypra	species	343149
743243	occ	79805	Siphocypra	species	343146
743700	occ	79847	Siphocypra	species	343062
743701	occ	79847	Siphocypra	species	343074
744293	occ	79876	Akleistostc	species	343120
744294	occ	79876	Akleistostc	species	343156
744295	occ	79876	Calusacypr	species	121276
744296	occ	79876	Calusacypr	species	82795
744297	occ	79876	Pseudadus	species	343716
744311	occ	79876	Siphocypra	species	343136
744337	occ	79875	Akleistostc	species	343181
744338	occ	79875	Calusacypr	species	121277
744339	occ	79875	Calusacypr	species	343129
744340	occ	79875	Pseudadus	species	342988
744341	occ	79875	Siphocypra	species	342009
744342	occ	79875	Siphocypra	species	343711
744360	occ	79875	Akleistostc	species	82791
744361	occ	79875	Siphocypra	species	343147
744391	occ	79874	Calusacypr	species	82796
744392	occ	79874	Pseudadus	species	343718
744393	occ	79874	Siphocypra	species	343143
744410	occ	79874	Akleistostc	species	342581
744411	occ	79874	Siphocypra	species	343133
744412	occ	79874	Siphocypra	species	343713
744413	occ	79874	Siphocypra	species	343714
745561	occ	79960	Akleistostc	species	341764
745562	occ	79960	Akleistostc	species	341756

745570	occ		79960	Akleistostc	species	343154
745571	occ		79960	Siphocypra	species	343134
745572	occ		79960	Calusacypr	species	121274
745685	occ		79331	Cypraea (S	species	342576
745711	occ		79959	Akleistostc	species	343118
745712	occ		79959	Siphocypra	species	343709
745713	occ		79959	Calusacypr	species	82793
745714	occ		79959	Calusacypr	species	121273
745715	occ		79959	Pseudadus	species	342964
745716	occ		79959	Siphocypra	species	342082
745787	occ		79959	Akleistostc	species	342632
745851	occ		79958	Pseudadus	species	343140
745852	occ		79958	Pseudadus	species	342726
745926	occ		79958	Siphocypra	species	342625
749711	occ		79761	Calusacypr	species	121273
749712	occ		79761	Calusacypr	species	121274
749713	occ		79761	Calusacypr	species	121276
749714	occ		79761	Calusacypr	species	121277
870190	occ		79960	Siphocypra	species	343117
870191	occ		79960	Siphocypra	species	342082
870192	occ		79960	Siphocypra	species	341763
870193	occ		79960	Siphocypra	species	342631
870257	occ		35025	Siphocypra	species	111875
870282	occ		35031	Cypraea (M	species	156624
1088980	occ		96538	Macrocypr	species	156625
1150764	occ		146874	Cypraea lir	species	342724
1150797	occ		146875	Siphocypra	species	342644
1331696	occ	32410	179337	Siphocypra	species	342631
1332759	occ		179591	Akleistostc	species	343180
567076	occ		59330	Cypraea (C	species	335332
567077	occ		59327	Cypraea (C	species	335332
567078	occ		59308	Cypraea (C	species	335332
567079	occ		59307	Cypraea (C	species	335332
567080	occ		59309	Cypraea (C	species	335332
567081	occ		59310	Cypraea (C	species	335332
1330580	occ		179144	Cypraea n.	species	341755
425348	occ		41497	Persicula o	species	120680
426152	occ		41501	Gibberulina	species	91269
426196	occ		41501	Persicula o	species	120680
427487	occ		41705	Gibberulina	species	91269
567532	occ		59484	Cypraeolin	species	107859
741290	occ		79331	Persicula o	species	120680
741291	occ		79331	Persicula (	species	107687
741292	occ		79331	Cypraeolin	species	156629
751019	occ		80358	Persicula o	species	120680
1089038	occ		96538	Granulina (	species	91270
564678	occ		58895	Granulina (	species	91270

564946	occ	58897	Granulina (	species	91270
567214	occ	59307	Cypraeolin	species	156629
659725	occ	71138	Granulina (	species	91270
559861	occ	57702	Granulina (	species	91270
727815	occ	78269	Granulina (	species	91270
727933	occ	78266	Granulina (	species	91270
727939	occ	78265	Granulina (	species	91270
915126	occ	103991	Anguispira	species	384101
915127	occ	103991	Discus cats	species	135550
929562	occ	82258	Anguispira	species	384101
426144	occ	41501	Discohelix	genus	8448
425376	occ	41498	"Drillia" sp	genus	11043
426099	occ	41539	Cymatosyr	species	93806
426123	occ	41501	Clathrodril	species	10923
426139	occ	41501	Cymatosyr	species	93806
426140	occ	41501	Cymatosyr	species	120675
427769	occ	41718	Cymatosyr	species	93806
427772	occ	41718	"Drillia" sp	species	11043
428058	occ	41749	Clathrodril	species	10923
428059	occ	41749	Clathrodril	species	10923
428074	occ	41749	Cymatosyr	species	93806
428075	occ	41749	Cymatosyr	genus	11010
567339	occ	59484	Cymatosyr	species	93806
567340	occ	59485	Cymatosyr	species	93806
567341	occ	59486	Cymatosyr	species	93806
567342	occ	59484	Clathrodril	species	108739
567343	occ	59485	Clathrodril	species	10923
567344	occ	59484	Clathrodril	species	10923
567345	occ	59485	Clathrodril	species	10923
567346	occ	59484	Crassispira	species	164125
568799	occ	59486	Clathrodril	species	108739
568800	occ	59486	Crassispira	species	164125
582501	occ	61310	Cymatosyr	genus	11010
697884	occ	74999	Drillia pogo	species	11043
697965	occ	75012	Drillia luna	species	11043
741942	occ	79740	Neodrillia l	species	119967
742175	occ	79765	Cymatosyr	species	93808
742199	occ	79766	Cymatosyr	species	93808
742203	occ	79761	Cymatosyr	species	93808
742477	occ	79762	Cymatosyr	species	93808
743316	occ	79804	Hindsiclava	species	164124
745743	occ	79959	Cymatosyr	species	93808
747393	occ	69029	Cymatosyr	genus	11010
747441	occ	79786	Clathrodril	genus	10923
747547	occ	79786	Syntomodı	genus	11737
748168	occ	69034	Splendrillia	genus	11692
750842	occ	79331	Cymatosyr	species	93806

750844	occ	79331	Cymatosyr	species	11010
750845	occ	79331	Cymatosyr	species	11010
750860	occ	79331	Cerodrillia	species	120688
1011565	occ	123549	Cymatosyr	species	93806
1150787	occ	146875	Cymatosyr	genus	11010
1150790	occ	146875	Cerodrillia	genus	108414
1150793	occ	146875	Hindsiclava	genus	107032
1150794	occ	146875	Syntomodı	genus	11737
564671	occ	58895	Cymatosyr	species	93806
564938	occ	58897	Cymatosyr	species	93806
567220	occ	59316	"Drillia" en	species	108738
567221	occ	59313	"Drillia" en	species	108738
567222	occ	59307	"Drillia" en	species	108738
567223	occ	59307	"Drillia" dr	species	108746
567224	occ	59307	Cymatosyr	species	108750
567225	occ	59307	Cymatosyr	species	108751
567226	occ	59316	Syntomodı	species	107424
567227	occ	59307	"Drillia" po	species	108764
567228	occ	59313	"Drillia" sir	species	108766
567229	occ	59316	"Drillia" sir	species	108766
567230	occ	59307	"Drillia" sir	species	108766
659693	occ	71138	Cymatosyr	species	93806
748537	occ	80033	Cymatosyr	genus	11010
727810	occ	78269	"Drillia" eb	species	108742
727863	occ	78267	"Drillia" sp	genus	11043
727927	occ	78266	"Drillia" eb	species	108742
727999	occ	78261	Cymatosyr	species	93806
728040	occ	78262	Cymatosyr	species	93806
728094	occ	78264	"Drillia" eb	species	108742
741464	occ	79709	"Drillia" eb	species	108742
741909	occ	79739	Melampus	species	155529
741910	occ	79739	Melampus	species	155532
1089066	occ	96538	Melampus	species	108704
1089067	occ	96538	Melampus	species	155528
1089068	occ	96538	Melampus	species	155531
1089083	occ	96538	Carychium	species	114476
620040	occ	66756	Melampus	species	108705
915128	occ	103991	Helicodiscu	species	162066
915129	occ	103991	Helicodiscu	species	162066
915130	occ	103991	Helicodiscu	species	162066
929563	occ	82258	Helicodiscu	species	162066
362435	occ	34269	Epitonium	species	9821
362436	occ	34269	Epitonium	genus	9821
426145	occ	41501	Epitonium	species	9821
426210	occ	41501	Scala sayar	species	97564
427764	occ	41718	Clathrus sp	genus	9656
567663	occ	59484	Epitonium	species	9821

582372	occ	61311	Epitonium	genus	9821
582391	occ	61309	Epitonium	genus	9821
582412	occ	61310	Epitonium	genus	9821
637097	occ	67069	Epitonium	species	108694
637098	occ	67069	Epitonium	species	9821
637099	occ	67069	Epitonium	species	109792
637100	occ	67069	Epitonium	species	9821
637101	occ	67069	Epitonium	species	156461
637102	occ	67069	Epitonium	species	9821
637103	occ	67069	Opalia deb	species	10213
637104	occ	67069	Depressisc	species	9744
637105	occ	67069	Cirsotrema	species	120557
697936	occ	75004	Epitonium	genus	9821
698016	occ	75006	Epitonium	genus	9821
745584	occ	79960	Epitonium	species	9821
747580	occ	80030	Epitonium	genus	9821
749664	occ	79331	Epitonium	species	9821
749665	occ	79331	Epitonium	species	9821
749666	occ	79331	Epitonium	species	9821
749667	occ	79331	Clathrus (C	species	9656
749668	occ	79331	Clathrus ju	species	9656
749669	occ	79331	Clathrus ol	species	9656
749670	occ	79331	Clathrus (P	species	9656
749671	occ	79331	Opalia (De	species	93659
1088997	occ	96538	Epitonium	species	109674
256363	occ	20604	Epitonium	species	108693
256381	occ	20606	Epitonium	species	108693
578779	occ	60930	Epitonium	species	109681
578780	occ	60930	Epitonium	species	109794
578958	occ	60940	Epitonium	species	9821
579051	occ	60944	Epitonium	species	9821
579053	occ	60944	Epitonium	species	108694
579054	occ	60944	Epitonium	genus	9821
619816	occ	66760	Epitonium	species	108694
619817	occ	66758	Epitonium	species	108694
619818	occ	66760	Epitonium	species	109674
619820	occ	66760	Epitonium	species	108696
619821	occ	66758	Epitonium	species	108696
619822	occ	66760	Epitonium	species	109676
619823	occ	66760	Epitonium	species	109681
619824	occ	66758	Epitonium	species	109681
741401	occ	79706	Epitonium	species	109681
832990	occ	92412	Epitonium	genus	9821
1457486	occ	203377	Scalaria cla	species	109678
564675	occ	58895	Epitonium	species	9821
564676	occ	58895	Epitonium	species	82751
564677	occ	58895	Epitonium	species	82752

564942	occ	58897	Epitonium	species	9821
567060	occ	59307	Epitonium	species	116536
567061	occ	59307	Epitonium	species	116536
567062	occ	59307	Epitonium	species	9821
567063	occ	59310	Epitonium	species	9821
567064	occ	59318	Epitonium	species	116533
567065	occ	59310	Epitonium	species	116533
567066	occ	59309	Epitonium	species	116533
579550	occ	60986	Epitonium	species	9821
579644	occ	60989	Epitonium	species	9821
579707	occ	60988	Epitonium	species	9821
579783	occ	60987	Epitonium	species	9821
619813	occ	66751	Epitonium	species	108694
619814	occ	66750	Epitonium	species	108694
619815	occ	66742	Epitonium	species	108694
619819	occ	66742	Epitonium	species	108696
659720	occ	71138	Clathrus ar	species	9656
748541	occ	80033	Epitonium	genus	9821
559829	occ	57705	Epitonium	species	109681
559830	occ	57705	Epitonium	species	9821
559831	occ	57705	Epitonium	species	108696
559832	occ	57705	Epitonium	species	109676
559842	occ	57704	Epitonium	species	109681
559843	occ	57704	Epitonium	species	109676
559844	occ	57704	Epitonium	species	108694
559856	occ	57702	Epitonium	species	109681
559857	occ	57702	Epitonium	species	108696
559858	occ	57702	Epitonium	species	109676
559859	occ	57702	Epitonium	species	108694
727757	occ	78270	Epitonium	genus	9821
727775	occ	78269	Epitonium	genus	9821
727892	occ	78266	Epitonium	genus	9821
727955	occ	78265	Epitonium	genus	9821
727992	occ	78260	Epitonium	genus	9821
728021	occ	78261	Epitonium	genus	9821
728058	occ	78262	Epitonium	genus	9821
728107	occ	78264	Epitonium	genus	9821
425346	occ	41497	Melanella :	genus	10112
426174	occ	41501	Melanella :	genus	10112
427495	occ	41705	Melanella (	species	10112
427496	occ	41705	Melanella :	genus	10112
567664	occ	59484	Melanella (	species	442834
567665	occ	59485	Melanella (	species	442834
567666	occ	59487	Melanella (	species	442834
567667	occ	59484	Melanella (	species	442834
567668	occ	59484	Strombifor	species	10516
567669	occ	59485	Strombifor	species	10516

567670	occ		59484	Niso wilcox	species	10181
582374	occ		61311	Melanella :	genus	10112
582393	occ		61309	Melanella :	genus	10112
582394	occ		61309	Melanella :	genus	10112
582414	occ		61310	Melanella :	genus	10112
637092	occ		67069	Niso interr	species	10181
637093	occ		67069	Eulima bifa	species	12094
637094	occ	22351	67069	Melanella _	species	156316
637095	occ		67069	Balcis sp.	genus	9524
749653	occ		79331	Niso willco	species	10181
749654	occ		79331	Strombifor	species	10516
749655	occ		79331	Strombifor	species	82754
749656	occ		79331	Melanella	species	442834
749657	occ		79331	Melanella	species	349339
749658	occ		79331	Melanella	species	109686
749659	occ		79331	Melanella	species	349339
749660	occ		79331	Melanella	species	349339
749661	occ		79331	Melanella	species	349339
749662	occ		79331	Melanella	species	349339
749663	occ		79331	Melanella	species	349339
749697	occ		79338	Strombifor	species	10516
578783	occ		60930	Melanella	species	109686
578859	occ		60937	Melanella	species	10112
619826	occ		66760	Melanella	species	109686
619827	occ		66758	Melanella	species	109686
564660	occ		58895	Balcis bico	species	82755
564924	occ		58897	Balcis beuf	species	9524
564925	occ		58897	Balcis ebor	species	82757
564926	occ		58897	Balcis ? sp.	genus	9524
564943	occ		58897	Eulima jun	species	82759
567067	occ		59307	Strombifor	species	10516
567068	occ		59316	Strombifor	species	82758
567069	occ		59316	Strombifor	species	82754
567070	occ		59307	Strombifor	species	82754
567071	occ		59307	Strombifor	species	10516
567072	occ		59330	Strombifor	species	10516
567073	occ		59307	Strombifor	species	10516
567074	occ		59307	Strombifor	species	116654
567075	occ		59316	Niso dalli	species	10181
579551	occ		60986	Melanella	species	109686
579645	occ		60989	Melanella	species	109686
579646	occ	22287	60989	Melanella _	species	156316
579708	occ		60988	Melanella	species	109686
579709	occ	22288	60988	Melanella _	species	156316
579784	occ		60987	Melanella	genus	10112
619825	occ	22289	66742	Melanella _	species	156316
642748	occ		69045	Niso sp.	genus	10181

659724	occ	71138	Balcis ebor	species	82757
748458	occ	80033	Niso dalli	species	10181
1512850	occ	211183	Melanella (	species	109686
582239	occ	61293	Eulima dall	species	12094
582248	occ	61294	Eulima dall	species	12094
727776	occ	78269	Strombifor	genus	10516
727777	occ	78269	Eulima sp.	genus	12094
727778	occ	78269	Eulima (Lic	species	12094
727893	occ	78266	Strombifor	genus	10516
727956	occ	78265	Strombifor	genus	10516
727993	occ	78260	Eulima sp.	genus	12094
728022	occ	78261	Eulima (Lic	species	12094
728059	occ	78262	Eulima sp.	genus	12094
728108	occ	78264	Strombifor	genus	10516
362426	occ	34269	Fasciolaria	species	156286
423299	occ	41199	Fasciolaria	genus	11116
423332	occ	41202	Fasciolaria	genus	11116
425344	occ	41497	Fasciolaria	species	120699
426100	occ	41539	Fasciolaria	species	120697
426147	occ	41501	Fasciolaria	species	120697
426148	occ	41501	Fasciolaria	species	120699
426150	occ	41501	Fusinus cal	species	120701
427484	occ	41705	Fasciolaria	species	120697
427485	occ	41705	Fasciolaria	species	116299
427774	occ	41718	Fasciolaria	species	120697
427775	occ	41718	Fasciolaria	species	156286
427928	occ	41740	Fasciolaria	species	120697
427929	occ	41740	Fasciolaria	species	156286
428078	occ	41749	Fasciolaria	species	120697
428079	occ	41749	Fasciolaria	species	156286
428080	occ	41749	Fasciolaria	species	116740
428081	occ	41749	Fasciolaria	species	120699
428082	occ	41749	Fusinus cal	species	120701
567626	occ	59488	Peristernia	species	11504
567627	occ	59484	Peristernia	species	11504
567628	occ	59488	Fusinus da	species	167739
568807	occ	59484	Fasciolaria	species	116740
568808	occ	59484	Fasciolaria	species	11116
568809	occ	59486	Peristernia	species	11504
568810	occ	59484	Fusinus cal	species	120701
582423	occ	61309	Fasciolaria	genus	11116
582424	occ	61309	Pleuroploc	species	120712
582490	occ	61310	Fasciolaria	genus	11116
582491	occ	61310	Pleuroploc	species	120712
622903	occ	67070	Fusinus wa	species	11138
622904	occ	67070	Latirus ma:	species	11251
622905	occ	67070	Latirus jucı	species	156695

622906	occ	67070	Fasciolaria	species	11116
640738	occ	69087	Fusinus sp.	genus	11138
741320	occ	79331	Latirus (Po	species	89559
741321	occ	79331	Latirus (Po	species	89559
741322	occ	79331	Latirus (Po	species	89559
741323	occ	79331	Fasciolaria	species	120698
741324	occ	79331	Fasciolaria	species	116741
741325	occ	79331	Fasciolaria	species	120700
741326	occ	79331	Fusinus (H	species	120701
741894	occ	79739	Fasciolaria	species	116299
741895	occ	79739	Fasciolaria	species	262616
741896	occ	79739	Triplofusus	species	135775
741933	occ	79740	Fasciolaria	species	156617
741934	occ	79740	Fusinus wa	species	11138
741975	occ	79748	Fasciolaria	species	156617
741982	occ	79748	Triplofusus	species	135774
741997	occ	79747	Fasciolaria	species	11116
742017	occ	79747	Fasciolaria	species	156617
742018	occ	79747	Fasciolaria	species	156617
742019	occ	79747	Fusinus ca	species	11138
742060	occ	79747	Fasciolaria	species	11116
742511	occ	79335	Liochlamys	species	11271
742519	occ	79335	Fasciolaria	species	120699
742552	occ	35031	Fasciolaria	species	120697
742553	occ	35031	Fasciolaria	species	11116
742568	occ	79782	Liochlamys	species	11271
743249	occ	79804	Terebraspi	species	136782
743250	occ	79804	Liochlamys	species	11271
743257	occ	79804	Heilprinia (	species	82815
743270	occ	79804	Fasciolaria	species	11116
743271	occ	79804	Fasciolaria	species	11116
743272	occ	79804	Fasciolaria	species	120697
743277	occ	79805	Terebraspi	species	136782
743278	occ	79805	Fasciolaria	species	156617
743279	occ	79805	Fasciolaria	species	11116
743704	occ	79847	Terebraspi	species	136782
743712	occ	79847	Terebraspi	species	136782
743728	occ	79804	Triplofusus	species	135774
744308	occ	79876	Fasciolara	species	136783
744312	occ	79876	Terebraspi	species	136782
744352	occ	79875	Heilprinia l	species	11186
744367	occ	79875	Terebraspi	species	136782
744400	occ	79874	Dolicholati	species	11038
744401	occ	79874	Latirus due	species	11251
744414	occ	79874	Terebraspi	species	136782
744415	occ	79874	Terebraspi	species	136782
745322	occ	79789	Fasciolaria	species	120697

745390	occ	79788	Triplofusus	species	116741
745404	occ	79788	Fasciolaria	species	120697
745426	occ	69040	Latirus sp.	genus	11251
745558	occ	79960	Terebraspi	genus	136782
745581	occ	79960	Fusinus dia	species	11138
745609	occ	79960	Heilprinia (	species	167743
745611	occ	79960	Heilprinia g	species	11186
745772	occ	79959	Heilprinia (	species	11186
745773	occ	79959	Triplofusus	species	135774
745774	occ	79959	Terebraspi	species	136782
745775	occ	79959	Fasciolaria	species	156617
745791	occ	79959	Terebraspi	species	136782
745792	occ	79959	Terebraspi	species	136782
745793	occ	79959	Terebraspi	species	136782
745936	occ	79958	Pleuroploc	species	11537
745937	occ	79958	Latirus mia	species	11251
747277	occ	80027	Latirus sp.	genus	11251
747364	occ	69029	Latirus sp.	genus	11251
747390	occ	69029	Fusinus (H	species	120701
747429	occ	79786	Heilprinia (	species	82815
747438	occ	79786	Fasciolaria	species	120697
747596	occ	80029	Peristernia	species	11504
747599	occ	80037	Fusinus da	species	167739
747734	occ	80046	Fusinus sp.	genus	11138
747780	occ	80046	Fasciolaria	species	11116
747810	occ	80046	Fusinus wa	species	11138
748169	occ	69034	Latirus sp.	genus	11251
748311	occ	80025	Latirus sp.	genus	11251
870208	occ	79960	Heilprinia (	species	11186
870219	occ	35029	Heilprinia (	species	167743
870260	occ	35025	Terebraspi	species	136782
870261	occ	35025	Liochlamys	species	11271
870267	occ	35031	Fasciolaria	species	156285
870280	occ	35031	Latirus juc	species	156695
1089024	occ	96538	Fasciolaria	species	156285
1089025	occ	96538	Fasciolaria	species	116299
1089026	occ	96538	Polygona a	species	156696
1089027	occ	96538	Triplofusus	species	135775
1150754	occ	146873	Latirus mia	species	11251
578875	occ	60942	Terebraspi	species	136782
578977	occ	60940	Triplofusus	species	116741
578978	occ	60940	Fasciolaria	species	11116
578990	occ	60940	Terebraspi	species	136782
579007	occ	60939	Terebraspi	species	136782
579008	occ	60939	Fusinus eq	species	11138
619978	occ	66758	Fasciolaria	species	156286
619979	occ	66760	Fasciolaria	species	156286

619980	occ	66758	Fasciolaria	species	116740
619981	occ	66760	Fasciolaria	species	116740
619982	occ	66760	Fasciolaria	species	116299
642888	occ	69046	Fasciolaria	genus	11116
642894	occ	69046	Fusinus sp.	genus	11138
567200	occ	59307	Fasciolaria	species	11116
567201	occ	59316	Fasciolaria	species	11116
567202	occ	59307	Fusinus he	species	11138
701063	occ	69047	Fasciolaria	species	116299
702916	occ	69018	Fasciolaria	species	11116
748480	occ	80033	Fasciolaria	genus	11116
748481	occ	80033	Fusinus exi	species	81355
748547	occ	80035	Fusinus exi	species	81355
748588	occ	80035	Fasciolaria	genus	11116
870215	occ	96536	Heilprinia (	species	11186
1173585	occ	151326	Fasciolaria	species	11116
727688	occ	78272	Peristernia	species	11504
727773	occ	78269	Peristernia	species	11504
727825	occ	78268	Lirosoma s	species	11281
727826	occ	78268	Lirosoma s	genus	11281
727845	occ	78269	Fusinus exi	species	81355
727891	occ	78266	Peristernia	species	11504
727991	occ	78260	Peristernia	species	11504
728004	occ	78261	Lirosoma s	species	85846
728005	occ	78261	Lirosoma s	genus	11281
728019	occ	78261	Peristernia	species	11504
728051	occ	78262	Fusinus exi	species	81355
728056	occ	78262	Peristernia	species	11504
737507	occ	79245	Lirosoma s	species	85846
737510	occ	79245	Fusinus sp.	genus	11138
741437	occ	79709	Lirosoma s	species	85846
748428	occ	80036	Lirosoma s	species	85846
427486	occ	41705	Ficus papy	species	147912
567682	occ	59484	Ficus aff. p	species	9857
582438	occ	61309	Ficus comr	species	147913
745622	occ	79960	Ficus holm	species	9857
745670	occ	79333	Ficus florid	species	9857
745681	occ	79331	Ficus papy	species	147912
745682	occ	79331	Ficus florid	species	9857
745703	occ	79334	Ficus papy	species	147912
745751	occ	79959	Ficus jacks	species	9857
747811	occ	80046	Ficus sp.	genus	9857
1088993	occ	96538	Ficus comr	species	147913
578968	occ	60940	Ficus jacks	species	9857
567086	occ	59316	Ficus papy	species	147912
748447	occ	80033	Ficus comr	species	147913
748566	occ	80035	Ficus comr	species	147913

362458	occ	34269	Fissurella a	species	91264
425375	occ	41498	Diodora sp	genus	8748
426143	occ	41501	Diodora ca	species	91263
427480	occ	41705	Diodora cf.	species	8748
427481	occ	41705	Diodora ca	species	91263
427771	occ	41718	Diodora ca	species	91263
568464	occ	59484	Fissuridea	species	77250
582349	occ	61310	Diodora ca	species	91263
582350	occ	61310	Diodora m	species	109210
582351	occ	61310	Diodora sa	species	8748
582352	occ	61310	Lucapina te	species	91312
582353	occ	61310	Lucapinella	species	91321
582365	occ	61311	Diodora ca	species	91263
582380	occ	61309	Diodora ca	species	91263
632436	occ	67069	Emarginula	species	8773
632437	occ	67069	Diodora ca	species	8748
632438	occ	67069	Diodora ca	species	91263
632439	occ	67069	Lucapina s	species	109109
632440	occ	67069	Lucapina s	species	91314
632441	occ	67069	Lucapinella	species	91321
632442	occ	67069	Lucapina te	species	91312
745401	occ	79788	Diodora sp	genus	8748
745615	occ	79960	Diodora (G	species	8748
747299	occ	69044	Fissurella s	genus	8815
747490	occ	79787	Diodora co	species	8748
748137	occ	69034	Fissurella s	genus	8815
749688	occ	79331	Lucapina te	species	91312
749690	occ	79331	Lucapina ta	species	8947
749691	occ	79331	Diodora flo	species	8748
749692	occ	79331	Diodora ca	species	8748
749693	occ	79331	Diodora ca	species	8748
749694	occ	79331	Diodora ca	species	91263
749701	occ	79338	Diodora ca	species	8748
749702	occ	79338	Diodora ca	species	8748
749707	occ	79336	Lucapina ta	species	8947
749708	occ	79336	Diodora ca	species	8748
749709	occ	79336	Emarginula	species	8773
1088924	occ	96538	Diodora ca	species	91263
1088925	occ	96538	Lucapina s	species	91314
1150721	occ	146872	Diodora al	species	8748
1150722	occ	146872	Diodora ca	species	8748
1150723	occ	146872	Diodora pe	species	8748
1150724	occ	146872	Diodora sp	genus	8748
578614	occ	60927	Diodora sp	genus	8748
578778	occ	60930	Diodora sp	genus	8748
578950	occ	60940	Diodora au	species	82739
579050	occ	60944	Diodora ca	species	91263

619805	occ	66760	Diodora ca	species	91263
619806	occ	66758	Diodora ca	species	91263
619807	occ	66756	Diodora ca	species	91263
619808	occ	66755	Diodora ca	species	91263
642930	occ	69046	Diodora pa	species	82740
832917	occ	92408	Fissuridea	species	109306
832992	occ	92412	Fissuridea	species	77250
1457458	occ	66757	Fissurella a	species	91264
564612	occ	58893	Diodora nu	species	8748
564629	occ	58894	Diodora nu	species	8748
564674	occ	58895	Diodora nu	species	8748
564941	occ	58897	Diodora nu	species	8748
567022	occ	59329	Diodora re	species	116542
567023	occ	59328	Diodora re	species	116542
567024	occ	59330	Diodora ca	species	8748
567025	occ	59307	Diodora ca	species	8748
702906	occ	69018	Diodora ca	species	8748
748455	occ	80033	Diodora ca	species	8748
748593	occ	80035	Diodora sp	genus	8748
748635	occ	80033	Diodora sp	genus	8748
582268	occ	61296	Diodora cf.	species	82739
727685	occ	78272	Diodora re	species	116542
727752	occ	78270	Diodora re	species	116542
727946	occ	78265	Diodora re	species	116542
728100	occ	78264	Diodora re	species	116542
741432	occ	79709	Diodora sp	genus	8748
748178	occ	80040	Diodora sp	genus	8748
748247	occ	80038	Diodora sp	genus	8748
748412	occ	80036	Diodora sp	genus	8748
1089084	occ	96538	Gastrocopt	species	162062
1089085	occ	96538	Gastrocopt	species	162062
1089086	occ	96538	Gastrocopt	species	162062
1607557	occ	227396	Gastrocopt	species	162062
1607558	occ	227396	Gastrocopt	species	162062
1607600	occ	227400	Gastrocopt	species	162062
915120	occ	103991	Gastrocopt	species	162062
929564	occ	82258	Gastrocopt	species	162062
426004	occ	41501	Atys subsc	species	11904
427443	occ	41705	Atys obscu	species	134956
427444	occ	41705	Atys sande	species	11904
640771	occ	69087	Atys sp.	genus	11904
738386	occ	79331	Atys (Alicu	species	134957
747311	occ	69044	Haminoea	genus	11946
622891	occ	67070	Morum on	species	86363
743251	occ	79805	Cancellom	species	86369
743301	occ	79804	Cancellom	species	86369
743302	occ	79804	Morum flo	species	10151

870262	occ	35025	Morum (Ca	species	115959
1066574	occ	35031	Morum (M	species	86367
1066575	occ	35025	Morum (M	species	86367
915118	occ	103991	Henderson	species	116329
927255	occ	82258	Henderson	species	116329
745950	occ	79958	Hipponix c	species	105945
749394	occ	79331	Hipponix fl	species	105945
750843	occ	79331	Cymatosyr	species	120669
750865	occ	79331	Thelecytha	species	11768
1150789	occ	146875	Buchema s	genus	108409
426158	occ	41501	"Hydrobia'	genus	85011
427489	occ	41705	"Hydrobia'	genus	85011
427780	occ	41718	"Hydrobia'	genus	85011
582543	occ	61310	Hydrobiida	family	60680
1088957	occ	96538	Tyronia ae	species	251917
1248055	occ	164698	Paludestrir	species	311056
559834	occ	57705	Hydrobia t	species	91300
568454	occ	59484	Molleria du	species	97700
637124	occ	67069	Didianema	species	8745
750967	occ	79338	Didianema	genus	8745
750970	occ	41718	Didianema	species	8745
750995	occ	79331	Didianema	species	8745
832976	occ	92410	Liotia aff. r	species	8928
564628	occ	58894	Didianema	species	8745
564673	occ	58895	Didianema	species	8745
564940	occ	58897	Didianema	species	8745
567032	occ	59313	Didianema	species	8745
578479	occ	60899	Didianema	species	8745
362444	occ	34269	Littorina ir	species	97643
742213	occ	79765	Littorina sh	species	93976
743214	occ	79804	Littorinops	species	10071
743215	occ	79804	Littorinops	species	10071
743710	occ	79847	Littorinops	species	10071
744362	occ	79875	Littorinops	species	10071
749652	occ	79331	Littorina ir	species	97643
1088954	occ	96538	Littorina ar	species	156702
619304	occ	66725	Littorina ir	species	97643
619874	occ	66756	Littorina ir	species	97643
619876	occ	66755	Littorina ir	species	97643
619878	occ	66760	Littorina ir	species	97643
619880	occ	66758	Littorina ir	species	97643
745493	occ	79957	Littoraria i	species	109671
832916	occ	92408	Littorina ir	species	97643
1457449	occ	150656	Littorina ir	species	97643
1457487	occ	203377	Littorina ir	species	97643
567033	occ	59318	Littorina ir	species	97643
567034	occ	59316	Littorina ir	species	97643

567035	occ	59317	Littorina ir	species	97643
567036	occ	59309	Littorina ir	species	97643
567037	occ	59313	Littorina ir	species	97643
619875	occ	66746	Littorina ir	species	97643
619877	occ	66742	Littorina ir	species	97643
738567	occ	69043	Littorina sp	genus	10068
748634	occ	80033	Littorina ir	species	97643
559846	occ	57704	Littorina ir	species	97643
632435	occ	67069	Acmaea pu	species	109078
745945	occ	79958	Acmaea ac	species	144943
748265	occ	80038	Acmaea sp	genus	8395
1089069	occ	96538	Fossaria cu	species	162060
1089070	occ	96538	Pseudosuc	species	251923
1607555	occ	227396	Fossaria cu	species	162060
1607556	occ	227396	Fossaria m	species	162060
1607583	occ	227398	Pseudosuc	species	251923
1607598	occ	227400	Fossaria cu	species	162060
1607599	occ	227400	Fossaria m	species	162060
929553	occ	82258	Lymnaea c	species	83642
745924	occ	79958	Magilus str	species	11308
425340	occ	41497	Brachycyth	species	10854
426010	occ	41501	Brachycyth	species	10854
426142	occ	41501	"Cythara" s	species	11016
426159	occ	41501	Ithycythara	species	91303
426160	occ	41501	Kurtziella li	species	11237
426161	occ	41501	"Kurtziella'	genus	11237
427781	occ	41718	Kurtziella li	species	11237
428073	occ	41749	"Cythara" s	genus	11016
432394	occ	41501	"Cythara" s	species	11016
432395	occ	41501	Kurtziella s	species	11237
567347	occ	59485	Mangilia ha	species	91203
567348	occ	59484	Mangilia ha	species	91203
567349	occ	59487	Mangilia ga	species	91203
567350	occ	59490	Mangilia ga	species	91203
567351	occ	59484	Mangilia af	species	91203
567353	occ	59484	"Cythara" l	species	157374
567354	occ	59484	Acmaturris	species	10713
582502	occ	61310	Pyrgocytha	species	91345
582523	occ	61311	Pyrgocytha	species	91345
750839	occ	79338	Ithycythara	species	91303
750840	occ	79338	Vitricythar	species	82944
750854	occ	41718	Brachycyth	species	162773
750861	occ	79331	Ithycythara	species	107471
750862	occ	79331	Ithycythara	species	91303
750863	occ	79331	Pyrgocytha	species	11616
750864	occ	79331	Ithycythara	species	11222
750866	occ	79331	Brachycyth	species	162773

750867	occ	79331	Brachycyth	species	10854
750869	occ	79331	Kurtziella li	species	91309
750870	occ	79331	Cryoturris :	species	91310
750871	occ	79331	Granoturri	species	121448
750872	occ	79331	Vitricythar	species	82944
750873	occ	79331	Vitricythar	species	82944
750885	occ	79336	Cryoturris :	species	91310
750886	occ	79338	Cryoturris :	species	91310
750889	occ	41718	Cryoturris :	species	91310
1089049	occ	96538	Kurtziella c	species	91307
1089051	occ	96538	Pyrgocytha	species	91345
1150788	occ	146875	Brachycyth	genus	10854
620026	occ	66758	Mangilia ce	species	108711
620027	occ	66760	Mangilia ce	species	108711
1457480	occ	203377	Pleurotom	species	162783
567231	occ	59316	Kurtziella e	species	108758
567232	occ	59307	Kurtziella e	species	108758
567233	occ	59316	Ithycythara	species	91302
567234	occ	59316	Cryoturris	species	10992
567235	occ	59316	Cryoturris	species	10992
567236	occ	59316	"Mangelia'	species	108713
567237	occ	59313	"Mangelia'	species	108713
620028	occ	66752	Mangilia ce	species	108711
620029	occ	66751	Mangilia ce	species	108711
620030	occ	66742	Mangilia ce	species	108711
659735	occ	71138	Cryoturris	species	10992
559488	occ	57706	Kurtziella c	species	91307
559835	occ	57705	Kurtziella c	species	91307
362425	occ	34269	Marginella	genus	11317
362441	occ	34269	Marginella	species	116743
425345	occ	41497	Marginella	species	11317
425363	occ	41497	Marginella	species	120677
426102	occ	41539	Marginella	species	116743
426115	occ	41539	Marginella	species	11317
426165	occ	41501	Marginella	species	116743
426166	occ	41501	Marginella	species	11317
426167	occ	41501	Marginella	species	11317
426168	occ	41501	Marginella	species	120677
426169	occ	41501	Marginella	species	82820
426170	occ	41501	Marginella	species	120691
426171	occ	41501	Marginella	genus	11317
427491	occ	41705	Marginella	species	116743
427492	occ	41705	Marginella	species	120677
427493	occ	41705	Marginella	species	11317
427494	occ	41705	Marginella	genus	11317
427623	occ	41715	Marginella	species	116743
427624	occ	41715	Marginella	species	11317

427782	occ	41718	Marginella	species	11317
427783	occ	41718	Marginella	species	116743
427784	occ	41718	Marginella	species	11317
427931	occ	41740	Marginella	species	116743
428087	occ	41749	Marginella	species	116743
428088	occ	41749	Marginella	species	120677
428089	occ	41749	Marginella	species	11317
428090	occ	41749	Marginella	species	11317
428091	occ	41749	Marginella	species	120691
428092	occ	41749	Marginella	genus	11317
567523	occ	59487	Marginella	species	11317
567524	occ	59487	Marginella	species	11317
567527	occ	59485	Marginella	species	97471
567528	occ	59484	Marginella	species	97471
567529	occ	59484	Marginella	species	11317
567530	occ	59484	Marginella	species	155515
567531	occ	59485	Marginella	species	155515
582477	occ	61309	Hyalina alb	species	91293
582478	occ	61309	Hyalina av	species	91296
582479	occ	61309	Marginella	genus	11317
582480	occ	61309	Marginella	species	116728
582481	occ	61309	Marginella	species	11317
582496	occ	61310	Hyalina alb	species	91293
582497	occ	61310	Marginella	genus	11317
582498	occ	61310	Marginella	species	116728
582513	occ	61311	Marginella	species	116728
640518	occ	69085	Marginella	genus	11317
640547	occ	69084	Marginella	genus	11317
640748	occ	69087	Prunum cf.	species	94426
647832	occ	70010	"Marginell	genus	11317
697882	occ	74999	Marginella	species	120691
697920	occ	75002	Marginella	species	82817
697922	occ	75003	Marginella	genus	11317
697960	occ	75012	Marginella	species	82817
697961	occ	75012	Marginella	species	97472
697977	occ	75013	Marginella	species	82817
697995	occ	75015	Marginella	genus	11317
698004	occ	75016	Marginella	species	82817
698029	occ	75008	Marginella	species	82817
741293	occ	79331	Marginella	species	82820
741294	occ	79331	Marginella	species	155515
741295	occ	79331	Marginella	species	11857
741296	occ	79331	Marginella	species	11857
741297	occ	79331	Marginella	species	11317
741298	occ	79331	Marginella	species	120677
741299	occ	79331	Marginella	species	116744
741300	occ	79331	Marginella	species	156670

741301	occ	79331	Marginella	species	120676
741302	occ	79331	Marginella	species	120691
741303	occ	79331	Marginella	species	92710
741317	occ	79338	Marginella	species	11857
741352	occ	79336	Marginella	species	82819
742179	occ	79762	Microspira	species	93837
742180	occ	79762	Microspira	species	93839
745348	occ	79789	Marginella	genus	11317
745388	occ	79788	Marginella	genus	11317
747015	occ	80002	Marginella	species	11317
747042	occ	80001	Marginella	species	116743
747264	occ	80027	Marginella	genus	11317
747340	occ	69029	Marginella	genus	11317
747449	occ	79786	Marginella	genus	11317
747538	occ	79786	Marginella	genus	11317
747812	occ	80046	Marginella	genus	11317
748160	occ	69034	Marginella	genus	11317
748304	occ	80025	Marginella	genus	11317
748335	occ	80024	Marginella	genus	11317
751025	occ	80357	Marginella	species	11317
1089039	occ	96538	Dentimarg	species	11026
1089040	occ	96538	Prunum ap	species	146384
1089041	occ	96538	Prunum be	species	11564
1089042	occ	96538	Prunum gu	species	11564
578782	occ	60930	Marginella	genus	11317
579060	occ	60944	Prunum co	species	11564
619988	occ	66760	Prunum ro	species	94426
619989	occ	66758	Prunum ro	species	94426
619990	occ	66757	Prunum ro	species	94426
642892	occ	69046	Prunum lin	species	82818
748116	occ	80083	Marginella	genus	11317
832882	occ	92407	Marginella	species	82817
832903	occ	92408	Marginella	species	155515
832904	occ	92408	Marginella	genus	11317
564627	occ	58894	Dentimarg	species	11026
564631	occ	58894	Prunum lin	species	82818
564672	occ	58895	Dentimarg	species	82821
564684	occ	58895	Prunum lin	species	82818
564694	occ	58895	Volvarina a	species	82823
564888	occ	58898	Prunum lin	species	82818
564939	occ	58897	Dentimarg	species	82821
564953	occ	58897	Prunum lin	species	82818
567210	occ	59307	Marginella	species	92784
567211	occ	59307	Marginella	species	97472
567212	occ	59307	Marginella	species	92647
579601	occ	60986	Prunum sp	genus	11564
579659	occ	60989	Prunum sp	genus	11564

579660	occ	60989	Hyalina sp.	genus	11209
579720	occ	60988	Prunum sp	genus	11564
579721	occ	60988	Hyalina sp.	genus	11209
579795	occ	60987	Prunum sp	genus	11564
619991	occ	66742	Prunum ro	species	94426
640567	occ	69086	Prunum lin	species	82818
642735	occ	69045	Prunum lin	species	82818
659733	occ	71138	Prunum lin	species	82818
659744	occ	71138	Prunum be	species	11564
702870	occ	68979	Marginella	genus	11317
702902	occ	69018	Marginella	genus	11317
737415	occ	79249	Marginella	genus	11317
738414	occ	79342	Marginella	genus	11317
738472	occ	79341	Marginella	genus	11317
738508	occ	79339	Marginella	genus	11317
738524	occ	79340	Marginella	genus	11317
748549	occ	80035	Marginella	subgenus	92671
1512855	occ	211183	Prunum sp	genus	11564
94753	occ	7108	Prunum ro	species	94426
559486	occ	57706	Dentimarg	species	155517
559828	occ	57705	Dentimarg	species	155517
640347	occ	69048	Prunum ro	species	94426
640363	occ	69049	Prunum ro	species	94426
727812	occ	78269	Marginella	species	82817
727813	occ	78269	Marginella	species	11317
727814	occ	78269	Marginella	genus	11317
727929	occ	78266	Marginella	species	82817
727934	occ	78266	Marginella	genus	11317
727938	occ	78265	Marginella	genus	11317
728001	occ	78261	Marginella	species	82817
728002	occ	78261	Marginella	species	11317
728003	occ	78261	Marginella	species	97472
728041	occ	78262	Marginella	species	11317
741455	occ	79709	Marginella	genus	11317
362427	occ	34269	Busycon ca	species	93862
362428	occ	34269	Busycon pe	species	108698
362429	occ	34269	Melongena	species	109659
425364	occ	41497	Melongena	genus	11341
426013	occ	41501	Busycon co	species	94555
426014	occ	41501	Busycon py	species	156465
426015	occ	41501	Busycon ra	species	120694
426016	occ	41501	Busycon sp	genus	10874
426093	occ	41539	Busycon co	species	94555
426175	occ	41501	Melongena	species	109659
427448	occ	41705	Busycon co	species	94555
427449	occ	41705	Busycon py	species	156465
427497	occ	41705	Melongena	species	109659

427618	occ	41715	Busycon sp	genus	10874
427753	occ	41718	Busycon cc	species	94555
427754	occ	41718	Busycon ra	species	120694
427786	occ	41718	Melongena	species	109659
427924	occ	41740	Busycon cc	species	94555
427925	occ	41740	Busycon py	species	156465
427932	occ	41740	Melongena	species	109659
428044	occ	41749	Busycon cc	species	94555
428046	occ	41749	Busycon pl	species	10874
428047	occ	41749	Busycon py	species	156465
428048	occ	41749	Busycon ra	species	120694
428093	occ	41749	Melongena	species	109659
568811	occ	59484	Busycon m	species	10874
568812	occ	59484	Busycon pe	species	108698
568813	occ	59486	Busycon pe	species	108698
582425	occ	61309	Melongena	species	108030
582426	occ	61309	Busycon sp	species	116295
582427	occ	61309	Busycon cc	species	94555
582459	occ	61310	Busycon cc	species	94555
582460	occ	61310	Busycon sp	species	116295
582461	occ	61310	Melongena	species	109659
582472	occ	61311	Busycon cc	species	94555
622900	occ	67070	Melongena	species	119901
640527	occ	69085	Busycon cc	species	94555
640537	occ	69084	Busycon cc	species	94555
640717	occ	69087	Busycon sp	genus	10874
640742	occ	69087	Busycon cc	species	94555
697908	occ	75001	Busycon py	species	156465
741304	occ	79331	Busycon (B	species	120694
741306	occ	79331	Busycon (B	species	94555
741307	occ	79331	Busycon pl	species	10874
741312	occ	79331	Busycon (B	species	156465
741318	occ	79331	Busycon (E	species	93857
741319	occ	79331	Melongena	species	116736
741353	occ	79336	Busycon pl	species	10874
741759	occ	79731	Busycon cc	species	94555
741871	occ	79738	Fulguropsi	species	119890
741872	occ	79738	Sinistrofulg	species	120024
741873	occ	79738	Melongena	species	116745
741905	occ	79739	Melongena	species	116745
741906	occ	79739	Sinistrofulg	species	120023
741916	occ	79739	Melongena	species	111214
741935	occ	79740	Melongena	species	11341
741936	occ	79740	Melongena	species	119902
741937	occ	79740	Melongena	species	116734
741939	occ	79740	Sinistrofulg	species	120023
741967	occ	79749	Busycon du	species	10874

741968	occ	79749	Sinistrofulg	species	120023
741976	occ	79748	Busycon ru	species	156797
741977	occ	79748	Busycotypu	species	10875
741978	occ	79748	Sinistrofulg	species	120023
741981	occ	79748	Pyruella bl	species	120732
742020	occ	79747	Melongena	species	11341
742021	occ	79747	Melongena	species	11341
742022	occ	79747	Fulguropsis	species	120025
742023	occ	79747	Fulguropsis	species	116293
742024	occ	79747	Sinistrofulg	species	120023
742045	occ	79747	Pyruella to	species	120732
742206	occ	79765	Busycon (E	species	93857
742210	occ	79761	Busycon (E	species	93857
742211	occ	79761	Tropochas	species	93954
742555	occ	35031	Melongena	species	119901
742569	occ	79782	Melongena	species	11341
742570	occ	79782	Echinofulg	species	93854
742571	occ	79782	Pyruella ei	species	120732
742572	occ	79782	Pyruella ov	species	120732
742573	occ	79782	Fulguropsis	species	116293
743238	occ	79804	Busycoarct	species	116290
743239	occ	79804	Melongena	species	11341
743246	occ	79804	Echinofulg	species	93857
743261	occ	79805	Fulguropsis	species	116293
743275	occ	79805	Sinistrofulg	species	120023
743276	occ	79805	Echinofulg	species	93854
743281	occ	79804	Sinistrofulg	species	120023
743282	occ	79804	Sinistrofulg	species	120023
743283	occ	79804	Pyruella pl	species	120732
743284	occ	79804	Pyruella so	species	120732
743285	occ	79804	Fulguropsis	species	116293
743705	occ	79847	Melongena	genus	11341
744303	occ	79876	Pyruella sc	species	120732
744304	occ	79876	Pyruella la	species	120732
744305	occ	79876	Tropochas	species	93953
744306	occ	79876	Echinofulg	species	93854
744307	occ	79876	Melongena	species	11341
744348	occ	79875	Echinofulg	species	93854
744349	occ	79875	Melongena	species	11341
744350	occ	79875	Pyruella fe	species	120732
744351	occ	79875	Pyruella tu	species	120732
744399	occ	79874	Melongena	species	11341
744416	occ	79874	Pyruella ca	species	120732
745323	occ	79789	Busycon cc	species	94555
745337	occ	79789	Busycon py	species	156465
745379	occ	79788	Busycon cc	species	94555
745393	occ	79788	Busycon py	species	156465

745564	occ	79960	Echinofulg	species	93854
745574	occ	79960	Busycon pa	species	10874
745575	occ	79960	Lindafulgu	species	120721
745576	occ	79960	Pyruella wa	species	120732
745577	occ	79960	Sinistrofulg	species	120023
745582	occ	79960	Tropochas	species	93954
745602	occ	79960	Sinistrofulg	species	120023
745603	occ	79960	Pyruella ha	species	120732
745605	occ	79960	Busycotyp	species	10875
745607	occ	79960	Busycoarct	species	116290
745629	occ	79960	Fulguropsi	species	116293
745630	occ	79960	Busycon fil	species	167640
745723	occ	79959	Echinofulg	species	93854
745724	occ	79959	Tropochas	species	93953
745725	occ	79959	Melongena	species	11341
745727	occ	79959	Busycon tit	species	10874
745728	occ	79959	Busycotyp	species	10875
745729	occ	79959	Busycoarct	species	116290
745730	occ	79959	Pyruella de	species	120732
745731	occ	79959	Pyruella ru	species	120732
745769	occ	79959	Sinistrofulg	species	120023
745770	occ	79959	Fulguropsi	species	116293
745794	occ	79959	Pyruella ba	species	120732
745933	occ	79958	Lindafulgu	species	120721
745934	occ	79958	Fulguropsi	species	116293
745935	occ	79958	Busycoarct	species	116290
747276	occ	80027	Busycon co	species	94555
747344	occ	69029	Busycon sp	genus	10874
747425	occ	79786	Busycon py	species	156465
747498	occ	79787	Busycon co	species	94555
747542	occ	79786	Busycon co	species	94555
747735	occ	80046	Busycon sp	genus	10874
747745	occ	80046	Busycon co	species	94555
747781	occ	80046	Melongena	species	11341
748130	occ	69034	Busycon co	species	94555
748296	occ	80025	Busycon sp	genus	10874
870176	occ	79960	Busycotyp	species	10875
870179	occ	79960	Busycon m	species	10874
870180	occ	79960	Sinistrofulg	species	120023
870181	occ	79960	Sinistrofulg	species	120023
870183	occ	79960	Busycoarct	genus	116290
870184	occ	79960	Fulguropsi	species	116293
870185	occ	79960	Busycon ch	species	10874
870186	occ	79960	Pyruella la	species	120732
870187	occ	79960	Busycotyp	species	10875
870198	occ	79960	Pyruella ru	species	120732
870218	occ	35025	Sinistrofulg	species	120023

870233	occ	79960	Tropochas	species	93954
870271	occ	35031	Melongena	species	116734
870274	occ	35031	Sinistrofulg	species	120023
870277	occ	35031	Melongeni	family	61090
870278	occ	35031	Melongena	species	119902
870279	occ	35031	Melongena	subgenus	116734
870292	occ	96538	Melongena	species	116745
1089014	occ	96538	Busycotypı	species	94437
1089015	occ	96538	Busycotypı	species	109669
1089017	occ	96538	Melongena	species	111214
1089018	occ	96538	Sinistrofulg	species	120023
1089019	occ	96538	Sinistrofulg	species	120024
256362	occ	20604	Busycon ca	species	93862
256379	occ	20606	Busycon ca	species	93862
256428	occ	20613	Melongena	species	109659
578972	occ	60940	Busycon cc	species	94555
578973	occ	60940	Busycon m	species	10874
578974	occ	60940	Busycon in	species	10874
578975	occ	60940	Busycon py	species	156465
579006	occ	60939	Busycon m	species	10874
579043	occ	60944	Busycon sp	genus	10874
619957	occ	66760	Busycon ca	species	94436
619958	occ	66756	Busycon ca	species	94436
619959	occ	66755	Busycon ca	species	94436
619967	occ	66760	Busycon ca	species	93862
619968	occ	66758	Busycon ca	species	93862
619969	occ	66759	Busycon ca	species	93862
619974	occ	66760	Busycon pe	species	108698
619975	occ	66758	Busycon pe	species	108698
619977	occ	66760	Busycon sp	species	116295
832878	occ	92407	Pyrula cari	species	157288
1457446	occ	150656	Busycon pe	species	108698
1457447	occ	150656	Busycon ca	species	94436
564661	occ	58895	Busycon ac	species	10874
564899	occ	58898	Busycon sp	species	109670
564900	occ	58898	Busycon cc	species	167644
564928	occ	58897	Busycon ac	species	10874
567138	occ	59313	Busycon ar	species	10874
567139	occ	59309	Busycon ar	species	10874
567140	occ	59316	Busycon ar	species	10874
567141	occ	59310	Busycon pe	species	108698
567142	occ	59309	Busycon pe	species	108698
567143	occ	59309	Busycon bl	species	10874
567144	occ	59313	Busycon bl	species	10874
567145	occ	59316	Busycon bl	species	10874
567146	occ	59313	Busycon w	species	10874
567147	occ	59316	Busycon w	species	10874

567148	occ	59316	Busycon (S	species	167644
567149	occ	59313	Busycon (S	species	167644
579560	occ	60986	Busycon sp	genus	10874
579656	occ	60989	Busycon sp	genus	10874
579718	occ	60988	Busycon sp	genus	10874
579793	occ	60987	Busycon sp	genus	10874
619960	occ	66754	Busycon ca	species	94436
619961	occ	66751	Busycon ca	species	94436
619962	occ	66750	Busycon ca	species	94436
619963	occ	66749	Busycon ca	species	94436
619964	occ	66744	Busycon ca	species	94436
619965	occ	66742	Busycon ca	species	94436
619966	occ	66742	Busycon ca	species	93862
619970	occ	66751	Busycon ca	species	93862
619971	occ	66750	Busycon ca	species	93862
619972	occ	66749	Busycon ca	species	93862
619973	occ	66744	Busycon ca	species	93862
619976	occ	66742	Busycon pe	species	108698
642733	occ	69045	Busycon sp	species	116295
659711	occ	71138	Busycon ar	species	10874
659717	occ	71138	Busycotypu	species	94437
701052	occ	69047	Busycon ca	species	93862
702915	occ	69018	Busycon sp	genus	10874
737431	occ	79248	Busycon sp	genus	10874
748225	occ	80034	Busycon ca	species	93862
748226	occ	80034	Busycon co	species	94555
748468	occ	80033	Busycon sp	genus	10874
748553	occ	80035	Busycon sp	genus	10874
748595	occ	80035	Busycon co	species	94555
829365	occ	91459	Busycon sp	genus	10874
829372	occ	91462	Busycon sp	genus	10874
870220	occ	96537	Busycon ch	species	10874
1173586	occ	151326	Busycon sp	genus	10874
1512984	occ	211182	Busycon sp	genus	10874
94749	occ	7108	Busycon ca	species	10874
94752	occ	7108	Busycon ca	species	94436
94771	occ	7109	Busycon ca	species	94436
94774	occ	7109	Busycon ca	species	93862
559478	occ	57706	Busycon ca	species	94436
559479	occ	57706	Busycon ca	species	93862
559819	occ	57705	Busycon ca	species	94436
559820	occ	57705	Busycon ca	species	93862
559849	occ	57702	Busycon ca	species	94436
559850	occ	57702	Busycon ca	species	93862
559851	occ	57702	Busycon co	species	94555
640351	occ	69048	Busycotypu	species	94437
640356	occ	69048	Busycon ca	species	93862

727690	occ	78272	Busycotypı	species	10875
727691	occ	78272	Busycon m	species	10874
727725	occ	78273	Busycotypı	species	10875
727726	occ	78273	Busycon m	species	10874
727781	occ	78269	Busycotypı	species	10875
727782	occ	78269	Busycon m	species	10874
727835	occ	78268	Busycotypı	species	10875
727836	occ	78268	Busycon m	species	10874
727897	occ	78266	Busycon m	species	10874
727958	occ	78265	Busycon sp	genus	10874
728025	occ	78261	Busycon in	species	10874
728062	occ	78262	Busycotypı	species	10875
728063	occ	78262	Busycon sp	genus	10874
728110	occ	78264	Busycon sp	genus	10874
870226	occ	96535	Busycon m	species	10874
870227	occ	96535	Sinistrofulg	species	120023
870231	occ	96535	Busycotypı	species	10875
426176	occ	41501	Mitra heilp	species	120366
428094	occ	41749	Mitra heilp	species	120366
568804	occ	59486	Mitra stepl	species	11365
568805	occ	59486	Mitra hosf	species	11365
697880	occ	74999	Mitra sp.	genus	11365
741280	occ	79331	Mitra (Plec	species	120366
741281	occ	79331	Mitra (Sub	species	115408
741339	occ	79333	Mitra (Sub	species	115408
743305	occ	79804	Pleioptygn	species	11532
745450	occ	69040	Mitridae ir	family	61164
745740	occ	79959	Pleioptygn	species	11532
745927	occ	79958	Mitra (Scra	species	11365
747394	occ	69029	Subcancilla	genus	11719
747428	occ	79786	Pleioptygn	species	11532
870174	occ	79960	Pleioptygn	species	164627
1089043	occ	96538	Mitra semi	species	11365
1150753	occ	146873	Mitra (Scal	species	11365
832977	occ	92410	Mitra aff. c	species	107864
870222	occ	96536	Pleioptygn	species	164627
426177	occ	41501	Mitramorp	species	120690
427498	occ	41705	Mitramorp	species	120690
750883	occ	79331	Mitromorp	species	107892
362442	occ	34269	Modulus fl	species	109167
426103	occ	41539	Modulus ca	species	187460
426178	occ	41501	Modulus ca	species	187460
427787	occ	41718	Modulus ca	species	187460
428095	occ	41749	Modulus ca	species	187460
568128	occ	59484	Modulus w	species	10140
582403	occ	61310	Modulus m	species	94596
640513	occ	69085	Modulus m	species	94596

640733	occ	69087	Modulus m	species	94596
647846	occ	70010	Modulus m	species	94596
741867	occ	79738	Modulus ca	species	157098
741885	occ	79739	Modulus p	species	10140
743213	occ	79804	Modulus ca	species	10140
745339	occ	79789	Modulus m	species	94596
745949	occ	79958	Modulus b	species	187452
747273	occ	80027	Modulus m	species	94596
747358	occ	69029	Modulus m	species	94596
747543	occ	79786	Modulus m	species	94596
749411	occ	79338	Modulus m	species	94596
749416	occ	79338	Modulus m	species	94596
749444	occ	79331	Modulus m	species	94596
749445	occ	79331	Modulus ca	species	187460
870294	occ	96538	Modulus m	species	94596
1088948	occ	96538	Modulus ca	species	157098
1088950	occ	96538	Modulus p	species	10140
1150800	occ	146875	Modulus w	species	10140
362432	occ	34269	Urosalpinx	genus	11824
362433	occ	34269	Eupleura ca	species	94513
362434	occ	34269	Murex fulv	species	11385
362437	occ	34269	Purpura ha	species	11605
425354	occ	41497	Trophon le	species	11793
426095	occ	41539	Cantharus	species	91538
426104	occ	41539	Murex pon	species	107994
426118	occ	41501	Cantharus	species	91538
426180	occ	41501	Murex bre	species	81537
426181	occ	41501	Murex pon	species	107994
426183	occ	41501	Murex sp.	genus	11385
426243	occ	41501	Trophon le	species	11793
426252	occ	41501	Typhis flori	species	135226
426253	occ	41501	Urosalpinx	species	11824
427467	occ	41705	Cantharus	species	91538
427483	occ	41705	Eupleura ca	species	94513
427499	occ	41705	Murex pon	species	107994
427551	occ	41705	Urosalpinx	species	135223
427758	occ	41718	Cantharus	species	91538
427799	occ	41718	Murex bre	species	81537
428054	occ	41749	Cantharus	species	91538
428096	occ	41749	Murex ma	species	82761
428097	occ	41749	Murex pon	species	107994
428110	occ	41749	Pseudosalp	genus	158732
428125	occ	41749	Trophon le	species	11793
428133	occ	41749	Urosalpinx	species	11824
567659	occ	59484	Eupleura ca	species	94513
567660	occ	59484	Muricidea	species	158736
567661	occ	59486	Purpura m	species	11605

567662	occ	59484	Urosalpinx	species	11824
582434	occ	61309	Urosalpinx	species	135224
582435	occ	61309	Phyllonotu	species	107997
582436	occ	61309	Muricanth	species	156534
582437	occ	61309	Chicoreus :	genus	10913
582445	occ	61310	Calotropho	species	91537
582446	occ	61310	Eupleura s	species	11099
582447	occ	61310	Favartia ce	species	156294
582448	occ	61310	Urosalpinx	species	135224
582449	occ	61310	Phyllonotu	species	107997
582450	occ	61310	Muricanth	species	156534
582451	occ	61310	Murexiella	species	82762
582464	occ	61311	Eupleura s	species	11099
582465	occ	61311	Phyllonotu	species	107997
582466	occ	61311	Urosalpinx	species	135224
622892	occ	67070	Tripteroty	species	135415
622893	occ	67070	Murexiella	species	158896
622895	occ	67070	Murex bell	species	119896
622896	occ	67070	Murex ann	species	144744
640532	occ	69084	Urosalpinx	genus	11824
640776	occ	69087	Urosalpinx	genus	11824
741892	occ	79739	Eupleura s	species	11099
741893	occ	79739	Vokesinotu	species	157090
741928	occ	79740	Chicoreus	species	10913
741929	occ	79740	Chicoreus	species	10913
741930	occ	79740	Vokesimur	species	119897
741931	occ	79740	Vokesimur	species	119898
741932	occ	79740	Murexiella	species	158896
741965	occ	79749	Eupleura c	species	11099
741988	occ	79748	Urosalpinx	species	11824
742016	occ	79747	Vokesimur	species	119897
742470	occ	79764	Ecphora qu	species	397350
742501	occ	79761	Ecphora qu	species	397350
742567	occ	79782	Phyllonotu	species	11521
743229	occ	79804	Chicoreus	species	10913
743230	occ	79804	Chicoreus :	species	10913
743231	occ	79804	Phyllonotu	species	11521
743232	occ	79804	Pterorhytis	species	82765
743235	occ	79804	Subpteryn	species	109406
743245	occ	79805	Chicoreus :	species	10913
743247	occ	79805	Phyllonotu	species	11521
743273	occ	79804	Eupleura c	species	11099
743274	occ	79804	Eupleura ir	species	11099
743702	occ	79847	Hexaplex t	species	11199
743703	occ	79847	Pterorhytis	species	82765
744309	occ	79876	Thais (Stra	species	89850
744310	occ	79876	Calotropho	species	10884

744343	occ	79875	Chicoreus ı	species	10913
744345	occ	79875	Pterorhytis	species	82765
744346	occ	79875	Pterorhytis	species	82765
744347	occ	79875	Hexaplex ja	species	11199
744395	occ	79874	Aspella pet	species	10792
744396	occ	79874	Murexiella	species	11386
744397	occ	79874	Chicoreus j	species	10913
745283	occ	79337	Stramonita	species	120561
745372	occ	79788	Eupleura c	species	94513
745399	occ	79788	Cantharus	species	91538
745457	occ	69038	Urosalpinx	genus	11824
745528	occ	79961	Ecphora pa	species	11052
745529	occ	79961	Ecphora ro	species	11052
745537	occ	79962	Ecphora qu	species	397350
745539	occ	79962	Pterorhytis	species	82765
745565	occ	79960	Pterorhytis	genus	82765
745613	occ	79960	Pterorhytis	species	82765
745618	occ	79960	Ecphora qu	species	397350
745621	occ	79960	Phyllonotu	species	11521
745656	occ	79331	Murex (Ph	species	107996
745657	occ	79331	Murex (Fav	species	156296
745659	occ	79338	Urosalpinx	species	11824
745660	occ	79331	Murex (Fav	species	82761
745661	occ	79331	Murex (Sul	species	109406
745662	occ	79331	Muricopsis	species	156601
745663	occ	79331	Trophon le	species	11793
745664	occ	79331	Typhis (Typ	species	135227
745665	occ	79331	Aspella (As	species	109493
745666	occ	79331	Aspella (As	species	109494
745671	occ	79331	Pterorhytis	species	82765
745672	occ	79331	Urosalpinx	species	11824
745676	occ	79331	Cantharus	species	91538
745677	occ	79331	Cantharus	species	158740
745678	occ	79336	Aspella (As	species	109493
745679	occ	79336	Cantharus	species	158740
745719	occ	79959	Chicoreus :	species	10913
745720	occ	79959	Chicoreus :	species	158671
745721	occ	79959	Hexaplex h	species	58249
745722	occ	79959	Pterorhytis	species	82765
745777	occ	79959	Ecphora qu	species	397350
745778	occ	79959	Vokesinotu	species	157092
745780	occ	79959	Phyllonotu	species	11521
745781	occ	79959	Chicoreus 1	species	81534
745788	occ	79959	Chicoreus :	species	10913
745789	occ	79959	Pterorhytis	species	82765
745853	occ	79958	Subpteryn	species	109403
745925	occ	79958	Babelomur	species	157088

745955	occ	79958	Coralliophi	species	108438
747317	occ	69044	Urosalpinx	genus	11824
747318	occ	69044	Calotrophc	genus	10884
747330	occ	69029	Typhinellu:	species	11816
747347	occ	69029	Eupleura c	species	94513
747501	occ	79787	Cantharus	species	91538
747518	occ	79787	Eupleura s	genus	11099
747525	occ	79786	Stramonita	species	92619
747597	occ	80029	Eupleura n	species	11099
747776	occ	80046	Chicoreus	species	10913
747809	occ	80046	Eupleura s	genus	11099
748140	occ	69034	Coralliophi	genus	10970
748156	occ	69034	Typhinellu:	species	11816
748165	occ	69034	Eupleura c	species	94513
748317	occ	80025	Eupleura c	species	94513
789719	occ	85448	Murex glol	species	11385
833602	occ	92440	Chicoreus	species	81534
833603	occ	92334	Chicoreus	species	81534
833604	occ	92510	Chicoreus	species	81534
833605	occ	92331	Chicoreus	species	81534
833606	occ	92511	Chicoreus	species	81534
833607	occ	92338	Chicoreus	species	81534
833608	occ	92520	Chicoreus	species	81534
833609	occ	92516	Chicoreus	species	81534
833610	occ	92337	Chicoreus	species	81534
833611	occ	92518	Chicoreus	species	81534
833612	occ	92498	Chicoreus	species	81534
833613	occ	92339	Chicoreus	species	81534
833614	occ	92441	Chicoreus	species	81534
833615	occ	92366	Chicoreus	species	81534
833618	occ	92519	Chicoreus	species	81534
833620	occ	92499	Chicoreus	species	81534
833621	occ	92500	Chicoreus	species	81534
833622	occ	92336	Chicoreus	species	81534
833623	occ	92340	Chicoreus	species	81534
833624	occ	92365	Chicoreus	species	81534
833625	occ	92335	Chicoreus	species	81534
833626	occ	92342	Chicoreus	species	81534
833628	occ	92343	Chicoreus	species	81534
833629	occ	92341	Chicoreus	species	81534
833632	occ	92369	Chicoreus	species	81536
833633	occ	92514	Chicoreus	species	81536
833634	occ	92370	Chicoreus	species	81536
833635	occ	92515	Chicoreus	species	81536
833728	occ	92369	Murex (Mu	species	157718
833729	occ	92334	Murex (Mu	species	157718
833730	occ	92337	Murex (Mu	species	157718

833731	occ	92338	Murex (Mu	species	157718
833732	occ	92366	Murex (Mu	species	157718
833733	occ	92339	Murex (Mu	species	157718
833734	occ	92335	Murex (Mu	species	157718
833735	occ	92340	Murex (Mu	species	157718
833736	occ	92500	Murex (Mu	species	157718
833737	occ	92496	Murex (Mu	species	157718
833738	occ	92331	Murex (Mu	species	157718
833739	occ	92498	Murex (Mu	species	157718
833740	occ	92499	Murex (Mu	species	157720
833741	occ	92369	Murex (Mu	species	157720
833994	occ	92344	Typhis (Ru	species	158000
833995	occ	92442	Typhis (Ru	species	158000
833997	occ	92443	Typhis (Ru	species	158000
833998	occ	92440	Typhis (Ru	species	158000
833999	occ	92576	Typhis (Ru	species	158000
834001	occ	92364	Typhis (Ru	species	158004
834002	occ	92443	Typhis (Ru	species	158004
834003	occ	92362	Typhis (Ru	species	158004
834004	occ	92664	Typhis (Ru	species	158004
834005	occ	92333	Typhis (Ru	species	158004
834006	occ	92332	Typhis (Ru	species	158004
834007	occ	92656	Typhis (Ru	species	158004
834008	occ	92659	Typhis (Ru	species	158004
834009	occ	92442	Typhis (Ru	species	158004
834010	occ	92344	Typhis (Ru	species	158004
834011	occ	92658	Typhis (Ru	species	158004
834012	occ	92343	Typhis (Ru	species	158004
834013	occ	92342	Typhis (Ru	species	158004
834014	occ	92500	Typhis (Ru	species	158004
834015	occ	92340	Typhis (Ru	species	158004
834016	occ	92365	Typhis (Ru	species	158004
834017	occ	92335	Typhis (Ru	species	158004
834018	occ	92339	Typhis (Ru	species	158004
834019	occ	92366	Typhis (Ru	species	158004
834020	occ	92338	Typhis (Ru	species	158004
834021	occ	92337	Typhis (Ru	species	158004
834022	occ	92334	Typhis (Ru	species	158004
834023	occ	92441	Typhis (Ru	species	158004
834024	occ	92331	Typhis (Ru	species	158004
834026	occ	92336	Typhis (Ru	species	158004
834027	occ	92692	Typhis (Ru	species	158004
834028	occ	92693	Typhis (Ru	species	158004
834098	occ	92623	Pterotyphi	species	158089
834099	occ	92622	Pterotyphi	species	158089
834233	occ	92440	Poirieria (P	species	158215
834234	occ	92362	Poirieria (P	species	158215

834235	occ	92510	Poirieria (P	species	158215
834236	occ	92576	Poirieria (P	species	158215
834793	occ	92740	Murex glob	species	11385
835769	occ	92624	Chicoreus (	species	158671
835787	occ	92344	Aspella ser	species	109491
835788	occ	92624	Aspella ser	species	109491
835789	occ	92514	Aspella n. s	species	109491
835790	occ	92692	Aspella ser	species	109491
835791	occ	92622	Aspella ser	species	109491
835792	occ	92369	Aspella ser	species	109491
835793	occ	92334	Aspella ser	species	109491
835794	occ	92337	Aspella ser	species	109491
835795	occ	92335	Aspella ser	species	109491
835796	occ	92336	Aspella ser	species	109491
835797	occ	92621	Aspella ser	species	109491
835798	occ	92623	Aspella ser	species	109491
835799	occ	92333	Aspella ser	species	109491
835800	occ	92332	Aspella ser	species	109491
835807	occ	92362	Dermomur	species	158684
835808	occ	92333	Dermomur	species	158684
835809	occ	92334	Dermomur	species	158684
835810	occ	92337	Dermomur	species	158684
835811	occ	92498	Dermomur	species	158684
835812	occ	92335	Dermomur	species	158684
835813	occ	92336	Dermomur	species	158684
835814	occ	92340	Dermomur	species	158684
835815	occ	92342	Dermomur	species	158684
835816	occ	92692	Dermomur	species	158684
835817	occ	92344	Dermomur	species	158684
835818	occ	92332	Dermomur	species	158684
835819	occ	92664	Dermomur	species	158684
835820	occ	92693	Dermomur	species	158684
835821	occ	92363	Dermomur	species	158684
835822	occ	92657	Dermomur	species	158684
835823	occ	92364	Dermomur	species	158684
835824	occ	92674	Dermomur	species	158684
835825	occ	92624	Dermomur	species	158684
835827	occ	92622	Dermomur	species	158688
835828	occ	92623	Dermomur	species	158688
835848	occ	92369	Dermomur	species	158693
835880	occ	92331	Calotropho	species	91537
835881	occ	92441	Calotropho	species	91537
835882	occ	92334	Calotropho	species	91537
835883	occ	92337	Calotropho	species	91537
835884	occ	92498	Calotropho	species	91537
835885	occ	92366	Calotropho	species	91537
835886	occ	92516	Calotropho	species	91537

835887	occ	92518	Calotropho	species	91537
835888	occ	92339	Calotropho	species	91537
835889	occ	92335	Calotropho	species	91537
835890	occ	92365	Calotropho	species	91537
835891	occ	92336	Calotropho	species	91537
835892	occ	92514	Calotropho	species	91537
835893	occ	92500	Calotropho	species	91537
835894	occ	92342	Calotropho	species	91537
835895	occ	92515	Calotropho	species	91537
835896	occ	92343	Calotropho	species	91537
835897	occ	92658	Calotropho	species	91537
835898	occ	92344	Calotropho	species	91537
835899	occ	92671	Calotropho	species	91537
835900	occ	92659	Calotropho	species	91537
835901	occ	92660	Calotropho	species	91537
835902	occ	92656	Calotropho	species	91537
835903	occ	92333	Calotropho	species	91537
835904	occ	92664	Calotropho	species	91537
835905	occ	92693	Calotropho	species	91537
835906	occ	92361	Calotropho	species	91537
835907	occ	92362	Calotropho	species	91537
835908	occ	92623	Calotropho	species	91537
835910	occ	92363	Calotropho	species	91537
835911	occ	92666	Calotropho	species	91537
835912	occ	92667	Calotropho	species	91537
835913	occ	92668	Calotropho	species	91537
835914	occ	92672	Calotropho	species	91537
835915	occ	92673	Calotropho	species	91537
835916	occ	92380	Calotropho	species	91537
835917	occ	92443	Calotropho	species	91537
835918	occ	92442	Calotropho	species	91537
835919	occ	92338	Calotropho	species	91537
835920	occ	92624	Calotropho	species	91537
835921	occ	92340	Calotropho	species	91537
835922	occ	92692	Calotropho	species	91537
835923	occ	92332	Calotropho	species	91537
835924	occ	92621	Calotropho	species	91537
835925	occ	92622	Calotropho	species	91537
835926	occ	92674	Calotropho	species	91537
835928	occ	92370	Calotropho	species	91537
835929	occ	92369	Calotropho	species	91537
835930	occ	92510	Calotropho	species	91537
835931	occ	92440	Calotropho	species	91537
835933	occ	92621	Attiliosa st	species	158747
835934	occ	92692	Attiliosa st	species	158747
835935	occ	92622	Attiliosa st	species	158747
835937	occ	92369	Attiliosa st	species	158747

835938	occ	92363	Attiliosa st species	158747
835939	occ	92380	Attiliosa st species	158747
836363	occ	92362	Hexaplex (species	158864
836364	occ	92658	Hexaplex (species	158864
836365	occ	92497	Hexaplex (species	158864
836366	occ	92361	Hexaplex (species	158864
836367	occ	92363	Hexaplex (species	158864
836368	occ	92664	Hexaplex (species	158864
836369	occ	92442	Hexaplex (species	158864
836370	occ	92344	Hexaplex (species	158864
836373	occ	92440	Murexiella species	109412
836374	occ	92496	Murexiella species	109412
836375	occ	92331	Murexiella species	109412
836376	occ	92334	Murexiella species	109412
836377	occ	92337	Murexiella species	109412
836378	occ	92498	Murexiella species	109412
836379	occ	92336	Murexiella species	109412
836380	occ	92342	Murexiella species	109412
836381	occ	92343	Murexiella species	109412
836382	occ	92692	Murexiella species	109412
836383	occ	92658	Murexiella species	109412
836384	occ	92344	Murexiella species	109412
836385	occ	92442	Murexiella species	109412
836386	occ	92656	Murexiella species	109412
836387	occ	92332	Murexiella species	109412
836388	occ	92664	Murexiella species	109412
836389	occ	92693	Murexiella species	109412
836390	occ	92362	Murexiella species	109412
836391	occ	92338	Murexiella species	109412
836392	occ	92621	Murexiella species	158896
836393	occ	92370	Murexiella species	158892
836394	occ	92335	Murexiella species	158892
836395	occ	92336	Murexiella species	158892
836397	occ	92621	Murexiella species	158892
836398	occ	92622	Murexiella species	158892
836399	occ	92332	Murexiella species	158892
836400	occ	92623	Murexiella species	158892
836401	occ	92369	Murexiella species	158892
836404	occ	92361	Murexiella species	158889
836405	occ	92664	Murexiella species	158889
836406	occ	92663	Murexiella species	158889
836407	occ	92659	Murexiella species	158889
836408	occ	92442	Murexiella species	158889
836409	occ	92344	Murexiella species	158889
836410	occ	92658	Murexiella species	158889
836412	occ	92518	Murexiella species	158889
836413	occ	92366	Murexiella species	158889

836414	occ	92441	Murexiella	species	158889
836415	occ	92440	Murexiella	species	158889
836416	occ	92498	Murexiella	species	158889
836417	occ	92496	Murexiella	species	158889
836418	occ	92331	Murexiella	species	158889
836419	occ	92334	Murexiella	species	158889
836420	occ	92337	Murexiella	species	158889
836421	occ	92338	Murexiella	species	158889
836422	occ	92339	Murexiella	species	158889
836423	occ	92335	Murexiella	species	158889
836424	occ	92336	Murexiella	species	158889
836425	occ	92340	Murexiella	species	158889
836426	occ	92341	Murexiella	species	158889
836428	occ	92342	Murexiella	species	158889
836429	occ	92692	Murexiella	species	158889
836430	occ	92332	Murexiella	species	158889
836431	occ	92693	Murexiella	species	158889
836432	occ	92344	Murexiella	species	158888
836433	occ	92331	Murexiella	species	158888
836434	occ	92441	Murexiella	species	158888
836435	occ	92334	Murexiella	species	158888
836437	occ	92338	Murexiella	species	158888
836438	occ	92518	Murexiella	species	158888
836439	occ	92339	Murexiella	species	158888
836440	occ	92335	Murexiella	species	158888
836444	occ	92692	Murexiella	species	158888
836445	occ	92658	Murexiella	species	158888
836446	occ	92659	Murexiella	species	158888
836447	occ	92656	Murexiella	species	158888
836448	occ	92332	Murexiella	species	158888
836449	occ	92693	Murexiella	species	158888
836450	occ	92362	Murexiella	species	158888
870172	occ	79960	Ecphora flo	species	11052
870173	occ	79960	Chicoreus s	species	10913
870188	occ	79960	Muricanthu	species	11388
870196	occ	79960	Chicoreus f	species	10913
870197	occ	79960	Pterorhytis	species	82765
870199	occ	79960	Urosalpinx	species	11824
870201	occ	79960	Ecphora vi	species	11052
870207	occ	79960	Vokesinotu	species	157092
870217	occ	35025	Vokesinotu	species	157092
870237	occ	79960	Ecphora br	species	11052
870269	occ	35031	Murex bell	species	11385
870283	occ	35031	Vokesinotu	genus	157090
870295	occ	96538	Eupleura s	species	11099
1011566	occ	123549	Muricidea	species	158736
1066593	occ	35025	Chicoreus (	species	107995

1066594	occ	35025	Pterynotus	species	89766
1066607	occ	35031	Chicoreus (	species	107995
1066608	occ	35031	Attiliosa al	species	158752
1066609	occ	35031	Pterotyphi	species	158089
1066612	occ	35025	Murexiella	species	158889
1066613	occ	35025	Murexiella	species	109412
1066619	occ	35029	Murexiella	species	109412
1088998	occ	96538	Calotropho	species	91537
1088999	occ	96538	Eupleura c	species	94513
1089001	occ	96538	Eupleura ta	species	11099
1089002	occ	96538	Favartia ce	species	156294
1089004	occ	96538	Phyllonotu	species	107997
1089005	occ	96538	Stramonita	species	92619
1089006	occ	96538	Urosalpinx	species	108171
1089007	occ	96538	Urosalpinx	species	135224
1150730	occ	146873	Coralliophi	species	10970
1150736	occ	146873	Vitularia lig	species	11843
1150746	occ	146873	Ecphora st	species	11052
1150748	occ	146873	Attiliosa vi	species	10802
1150749	occ	146873	Muricopsis	species	11389
1150750	occ	146873	Dermomur	species	158684
1150751	occ	146873	Urosalpinx	species	11824
1150752	occ	146873	Urosalpinx	species	11824
1332755	occ	179591	Ecphora qu	species	397350
1332758	occ	179591	Pterorhytis	species	82765
256364	occ	20604	Eupleura c	species	94513
256382	occ	20606	Eupleura c	species	94513
619384	occ	66725	Murex (Mu	species	109407
578615	occ	60927	Eupleura c	species	94513
578695	occ	60928	Urosalpinx	species	108171
578781	occ	60930	Eupleura c	species	94513
578860	occ	60937	Coralliophi	species	10970
578874	occ	60942	Ecphora br	species	11052
578969	occ	60940	Urosalpinx	species	11824
578970	occ	60940	Calotropho	species	91537
578971	occ	60940	Ecphora qu	species	397350
619889	occ	66757	Murex pon	species	107994
619890	occ	66758	Murex pon	species	107994
619894	occ	66760	Eupleura c	species	94513
619895	occ	66758	Eupleura c	species	94513
619896	occ	66755	Eupleura c	species	94513
619897	occ	66758	Urosalpinx	species	108171
619898	occ	66756	Urosalpinx	species	108171
619899	occ	66755	Urosalpinx	species	108171
642887	occ	69046	Urosalpinx	species	108171
642896	occ	69046	Eupleura c	species	94513
832877	occ	92407	Fusus cine	species	97548

833627	occ	92367	Chicoreus (	species	81534
834025	occ	92367	Typhis (Ru	species	158004
836396	occ	92367	Murexiella	species	158892
836402	occ	92367	Murexiella	species	158890
836427	occ	92367	Murexiella	species	158889
836443	occ	92367	Murexiella	species	158888
564697	occ	58895	Urosalpinx	species	135224
564944	occ	58897	Eupleura c	species	94513
564966	occ	58897	Urosalpinx	species	135224
567087	occ	59316	Murex (Ph	species	107996
567088	occ	59307	Tritonalia (	species	11787
567090	occ	59313	Eupleura c	species	94513
567091	occ	59316	Eupleura c	species	94513
567092	occ	59330	Eupleura c	species	94513
567093	occ	59309	Urosalpinx	species	11824
567094	occ	59310	Urosalpinx	species	11824
567095	occ	59317	Urosalpinx	species	11824
567096	occ	59318	Urosalpinx	species	11824
567097	occ	59307	Urosalpinx	species	11824
567098	occ	59310	Urosalpinx	species	11824
567099	occ	59309	Urosalpinx	species	11824
567100	occ	59313	Urosalpinx	species	11824
567101	occ	59309	Urosalpinx	species	11824
567102	occ	59316	Urosalpinx	species	11824
567103	occ	59313	Urosalpinx	species	135224
567104	occ	59310	Urosalpinx	species	135224
567105	occ	59316	Urosalpinx	species	135224
567106	occ	59307	Urosalpinx	species	11824
579555	occ	60986	Urosalpinx	species	108171
579652	occ	60989	Urosalpinx	species	108171
579714	occ	60988	Urosalpinx	species	108171
579789	occ	60987	Urosalpinx	species	108171
619891	occ	66750	Eupleura c	species	94513
619892	occ	66751	Eupleura c	species	94513
619893	occ	66742	Eupleura c	species	94513
619900	occ	66752	Urosalpinx	species	108171
619901	occ	66742	Urosalpinx	species	108171
642721	occ	69045	Eupleura c	species	94513
642740	occ	69045	Urosalpinx	species	11824
659713	occ	71138	Eupleura c	species	94513
659716	occ	71138	Urosalpinx	species	135224
659719	occ	71138	Urosalpinx	species	11824
700972	occ	69043	Urosalpinx	genus	11824
701057	occ	69047	Urosalpinx	species	108171
737417	occ	79249	"Trophon"	species	97544
737438	occ	79248	"Trophon"	species	97544
738485	occ	79341	Urosalpinx	genus	11824

738515	occ	79339	Eupleura c	species	94513
748389	occ	80033	Calotrophc	species	91537
748390	occ	80033	Thais sp.	genus	11763
748548	occ	80035	Urosalpinx	genus	11824
834000	occ	92447	Typhis (Ru	species	158000
835909	occ	80033	Calotrophc	species	91537
1512851	occ	211183	Urosalpinx	species	135224
1512983	occ	211182	Urosalpinx	species	135224
94754	occ	7108	Eupleura c	species	94513
94772	occ	7109	Eupleura c	species	94513
559476	occ	57706	Atractodor	species	136229
559487	occ	57706	Eupleura c	species	94513
559501	occ	57706	Urosalpinx	species	108171
559818	occ	57705	Atractodor	species	136229
559833	occ	57705	Eupleura c	species	94513
559845	occ	57704	Eupleura c	species	94513
559860	occ	57702	Eupleura c	species	94513
566300	occ	59297	Ecphora qu	species	397350
640366	occ	69049	Eupleura c	species	94513
664788	occ	71620	Ecphora qu	species	397350
664844	occ	71624	Ecphora qu	species	397350
665357	occ	60660	Ecphora qu	species	397350
727684	occ	78272	Ecphora qu	species	397350
727687	occ	78272	Trophon te	species	97544
727723	occ	78273	Ecphora qu	species	397350
727746	occ	78270	Ecphora qu	species	397350
727774	occ	78269	Urosalpinx	species	11824
727824	occ	78268	Ecphora qu	species	397350
727846	occ	78269	Trophon te	species	97544
727931	occ	78266	Ecphora qu	species	397350
727940	occ	78265	Ecphora qu	species	397350
728015	occ	78261	Trophon te	species	97544
728020	occ	78261	Urosalpinx	species	11824
728042	occ	78262	Ecphora qu	species	397350
728057	occ	78262	Urosalpinx	species	11824
728106	occ	78264	Urosalpinx	species	11824
737501	occ	79245	Ecphora qu	species	397350
737509	occ	79245	"Trophon"	species	97544
737513	occ	79245	Urosalpinx	genus	11824
741440	occ	79709	Urosalpinx	species	11824
741446	occ	79709	Ecphora qu	species	397350
748190	occ	80040	Ecphora sp	genus	11052
748278	occ	80038	Ecphora sp	genus	11052
748413	occ	80036	Ecphora sp	genus	11052
748424	occ	80036	Urosalpinx	genus	11824
749172	occ	80132	Eupleura c	species	94513
749174	occ	80132	Urosalpinx	species	11824

870223	occ		96535	Vokesinotu	species	157092
870224	occ		96535	Urosalpinx	species	11824
870228	occ		96535	Ecphora qu	species	397350
362431	occ		34269	Nassa acut	species	108668
425365	occ		41497	Nassarius k	species	11404
426185	occ		41501	Nassarius k	species	11404
426186	occ		41501	Nassarius c	species	109317
426187	occ		41501	Nassarius f	species	156802
426188	occ		41501	Nassarius l	species	11404
426189	occ		41501	Nassarius s	genus	11404
426190	occ		41501	Nassarius v	species	108179
427501	occ		41705	Nassarius k	species	11404
427502	occ		41705	Nassarius f	species	156802
427503	occ		41705	Nassarius l	species	11404
427504	occ		41705	Nassarius s	species	11404
427505	occ		41705	Nassarius v	species	108179
427801	occ		41718	Nassarius s	genus	11404
427802	occ		41718	Nassarius v	species	108179
427933	occ		41740	Nassarius k	species	11404
427934	occ		41740	Nassarius v	species	108179
427935	occ		41740	Nassarius s	genus	11404
428077	occ	24944	41749	Calophos v	species	188186
428100	occ		41749	Nassarius v	species	108179
567629	occ	24945	59484	Calophos v	species	188186
567643	occ		59484	Alectrion c	species	10738
567644	occ		59484	Alectrion fl	species	10738
567645	occ		59485	Alectrion fl	species	10738
567646	occ		59485	Alectrion q	species	10738
567647	occ		59484	Alectrion q	species	10738
567648	occ		59484	Alectrion c	species	10738
567649	occ		59491	Alectrion c	species	10738
567650	occ		59485	Alectrion c	species	10738
568814	occ	24946	59486	Calophos v	species	188186
582428	occ		61309	Nassarius a	species	146385
582429	occ		61309	Nassarius v	species	108179
582456	occ		61310	Nassarius a	species	108670
582457	occ		61310	Nassarius a	species	146385
582458	occ		61310	Nassarius v	species	108179
582470	occ		61311	Nassarius a	species	108670
582471	occ		61311	Nassarius v	species	108179
623053	occ	24947	67072	Calophos v	species	188186
640752	occ		69087	Nassarius s	genus	11404
741327	occ		79331	Nassarius (	species	108180
741336	occ		79331	Nassarius (	species	89569
741338	occ		79331	Nassarius (	species	89569
741340	occ		79331	Nassarius (	species	89569
741341	occ		79331	Nassarius (	species	89569

741342	occ		79331	Nassarius (	species	89569
741343	occ		79331	Nassarius (	species	156802
741354	occ		79336	Nassarius (	species	89569
741904	occ		79739	Phrontis vi	species	11520
742186	occ		79764	Ilyanassa fl	species	58245
742187	occ		79764	Ilyanassa c	species	93956
742207	occ		79765	Ilyanassa fl	species	58245
742208	occ		79765	Ilyanassa n	species	93955
742506	occ	24948	79766	Calophos v	species	188186
743292	occ		79804	Ilyanassa g	species	95627
743293	occ		79804	Ilyanassa p	species	58245
743294	occ		79804	Ilyanassa w	species	95620
744353	occ		79875	Calophos r	species	10882
744366	occ		79875	Ilyanassa n	species	93955
745353	occ		79789	Nassarius (	species	108180
745394	occ		79788	Nassarius (	species	108180
745443	occ		69038	Nassarius (	species	108180
747017	occ		80002	Nassarius \	species	108179
747045	occ		80001	Nassarius \	species	108179
747046	occ		80001	Nassarius s	genus	11404
747269	occ		80027	Nassarius (	species	108180
747310	occ		69044	Nassarius s	genus	11404
747323	occ		69029	Nassarius s	genus	11404
747381	occ		69029	Nassarius (	species	108180
747465	occ		79786	Uzita florid	species	11827
747517	occ		79787	Nassarius (	species	108180
747544	occ		79786	Nassarius (	species	108180
747637	occ		80037	Nassarius (	species	96883
748166	occ		69034	Nassarius (	species	108180
751023	occ		80357	Nassarius c	species	156802
751024	occ		80357	Nassarius b	species	11404
833242	occ		92440	Trajana (Tr	species	157445
833243	occ		92366	Trajana (Tr	species	157445
833244	occ		92344	Trajana (Tr	species	157445
833245	occ		92442	Trajana (Tr	species	157445
833246	occ		92443	Trajana (Tr	species	157445
833247	occ		92363	Trajana (Tr	species	157445
833249	occ		92441	Trajana (Tr	species	157439
870175	occ		79960	Paranassa	species	95617
870177	occ		79960	Calophos p	species	10882
870195	occ		79960	Ilyanassa s	species	95632
870255	occ		35025	Ilyanassa s	genus	58245
1089020	occ		96538	Nassarius a	species	108670
1089021	occ		96538	Nassarius a	species	146385
1089022	occ		96538	Nassarius b	species	11404
1089023	occ		96538	Nassarius \	species	108179
1150738	occ		146873	Calophos s	genus	10882

619319	occ	66724	Nassarius a	species	108670
578976	occ	60940	Nassarius c	species	11404
579056	occ	60944	Nassarius (	species	11404
619918	occ	66758	Nassarius c	species	94421
619919	occ	66760	Nassarius c	species	94421
619920	occ	66756	Nassarius c	species	94421
619921	occ	66755	Nassarius c	species	94421
619928	occ	66755	Nassarius v	species	108179
619929	occ	66756	Nassarius v	species	108179
619940	occ	66760	Nassarius t	species	94424
619941	occ	66760	Nassarius a	species	108670
619942	occ	66758	Nassarius a	species	108670
619943	occ	66756	Nassarius a	species	108670
741402	occ	79706	Nassarius a	species	108670
741408	occ	79706	Uzita sp.	genus	11827
745475	occ	79956	Ilyanassa s	genus	58245
748097	occ	80083	Ilyanassa o	species	81607
832909	occ	92408	Ilyanassa ir	species	95619
564679	occ	58895	Nassarius c	species	82802
564680	occ	58895	Nassarius c	species	11404
564949	occ	58897	Nassarius c	species	82802
564950	occ	58897	Nassarius s	species	82810
567089	occ	59307	Trajana py	species	157438
567150	occ	59307	Ilyanassa a	species	95638
567151	occ	59317	Ilyanassa ir	species	95619
567152	occ	59309	Ilyanassa ir	species	95619
567153	occ	59313	Ilyanassa ir	species	95619
567154	occ	59309	Ilyanassa is	species	95622
567155	occ	59313	Ilyanassa is	species	95622
567156	occ	59316	Ilyanassa is	species	95622
567157	occ	59318	Ilyanassa is	species	95622
567158	occ	59329	Ilyanassa s	species	95626
567159	occ	59318	Ilyanassa s	species	95626
567160	occ	59316	Ilyanassa s	species	95626
567161	occ	59309	Ilyanassa s	species	95626
567162	occ	59313	Ilyanassa s	species	95626
567163	occ	59307	Ilyanassa s	species	95626
567164	occ	59318	Ilyanassa w	species	95620
567165	occ	59330	Ilyanassa g	species	95627
567166	occ	59327	Ilyanassa g	species	95627
567167	occ	59307	Ilyanassa g	species	95627
567168	occ	59309	Ilyanassa g	species	95627
567169	occ	59309	Ilyanassa s	species	95628
567170	occ	59316	Ilyanassa s	species	95628
567171	occ	59310	Ilyanassa s	species	95628
567172	occ	59313	Ilyanassa s	species	95628
567173	occ	59316	Ilyanassa jo	species	95630

567174	occ	59313	Ilyanassa jc	species	95630
567175	occ	59310	Ilyanassa jc	species	95630
567176	occ	59307	Ilyanassa jc	species	95630
567177	occ	59309	Ilyanassa jc	species	95630
567178	occ	59307	Ilyanassa s	species	95632
567179	occ	59316	Ilyanassa s	species	95632
567180	occ	59318	Ilyanassa s	species	95632
567181	occ	59313	Ilyanassa s	species	95632
567182	occ	59309	Ilyanassa s	species	95632
567183	occ	59310	Ilyanassa s	species	95632
567184	occ	59307	Ilyanassa h	species	95637
567185	occ	59307	Uzita smith	species	95952
567186	occ	59309	Uzita neog	species	95954
567187	occ	59316	Uzita neog	species	95954
567188	occ	59307	Uzita chow	species	82801
567189	occ	59307	Uzita bider	species	95958
567190	occ	59313	Uzita laper	species	11827
567191	occ	59316	Uzita laper	species	11827
567192	occ	59316	Uzita conse	species	11827
567193	occ	59307	Uzita conse	species	11827
567194	occ	59307	Uzita caloc	species	11827
567195	occ	59316	Uzita caloc	species	11827
567196	occ	59313	Uzita caloc	species	11827
567197	occ	59316	Uzita zeta	species	11827
567198	occ	59309	Uzita vibex	species	11827
567199	occ	59307	Uzita vibex	species	11827
578481	occ	60899	Nassarius z	species	11404
579600	occ	60986	Nassarius a	species	146385
579657	occ	60989	Nassarius a	species	146385
579719	occ	60988	Nassarius a	species	146385
579794	occ	60987	Nassarius a	species	146385
619922	occ	66754	Nassarius c	species	94421
619923	occ	66752	Nassarius c	species	94421
619924	occ	66750	Nassarius c	species	94421
619925	occ	66744	Nassarius c	species	94421
619926	occ	66742	Nassarius c	species	94421
619927	occ	66742	Nassarius v	species	108179
619930	occ	66754	Nassarius t	species	94424
619931	occ	66752	Nassarius t	species	94424
619932	occ	66751	Nassarius t	species	94424
619933	occ	66750	Nassarius t	species	94424
619934	occ	66749	Nassarius t	species	94424
619935	occ	66748	Nassarius t	species	94424
619936	occ	66747	Nassarius t	species	94424
619937	occ	66746	Nassarius t	species	94424
619938	occ	66744	Nassarius t	species	94424
619939	occ	66742	Nassarius t	species	94424

619944	occ	66754	Nassarius a	species	108670
619945	occ	66752	Nassarius a	species	108670
619946	occ	66751	Nassarius a	species	108670
619947	occ	66750	Nassarius a	species	108670
619948	occ	66747	Nassarius a	species	108670
619949	occ	66748	Nassarius a	species	108670
619950	occ	66742	Nassarius a	species	108670
642737	occ	69045	Nassarius (	subgenus	89570
642739	occ	69045	Ilyanassa g	species	95627
659710	occ	71138	Nassarius (	species	82802
659712	occ	71138	Ilyanassa g	species	95627
659715	occ	71138	Ilyanassa c	species	58245
701060	occ	69047	Nassarius (	species	108669
701064	occ	69047	Ilyanassa t	species	58245
702867	occ	68979	Uzita sp.	genus	11827
738514	occ	79339	Ilyanassa s	genus	58245
741426	occ	79708	Nassarius s	genus	11404
748243	occ	80034	Ilyanassa s	species	95626
748391	occ	80033	Ilyanassa s	genus	58245
748392	occ	80033	Ilyanassa h	species	58245
748459	occ	80033	Uzita sp.	genus	11827
748482	occ	80033	Ilyanassa s	species	95632
748496	occ	80033	Ilyanassa s	genus	58245
748542	occ	80033	Ilyanassa ir	species	58245
748545	occ	80035	Ilyanassa s	species	58245
748558	occ	80035	Uzita cf. su	species	11827
748633	occ	80033	Uzita cf. su	species	11827
833241	occ	92447	Trajana (Tr	species	157445
1512832	occ	211029	Nassarius a	species	146385
1512853	occ	211183	Nassarius a	species	146385
1512939	occ	211181	Nassarius a	species	146385
1512985	occ	211182	Nassarius a	species	146385
94750	occ	7108	Nassarius t	species	94424
94770	occ	7109	Nassarius (	species	94421
94773	occ	7109	Nassarius t	species	94424
559491	occ	57706	Nassarius (	species	94420
559492	occ	57706	Nassarius (	species	94423
582238	occ	61293	Nassarius ?	genus	11404
640349	occ	69048	Ilyanassa o	species	81607
640350	occ	69048	Ilyanassa t	species	94439
640378	occ	69049	Ilyanassa t	species	94439
727756	occ	78270	Ilyanassa g	species	95627
727833	occ	78268	Nassarius s	genus	11404
727848	occ	78269	Nassarius s	genus	11404
727868	occ	78267	Ilyanassa g	species	95627
727887	occ	78266	Ilyanassa p	species	58245
727951	occ	78265	Ilyanassa g	species	95627

727952	occ		78265	Nassarius s	genus	11404
728017	occ		78261	Nassarius s	genus	11404
728053	occ		78262	Nassarius s	genus	11404
728104	occ		78264	Nassarius s	genus	11404
748421	occ		80036	Ilyanassa s	genus	58245
870230	occ		96535	Calophos p	species	10882
870232	occ		96535	Paranassa	species	95617
362447	occ		34269	Neverita d	species	108603
362455	occ		34269	Sigaretus p	species	108671
425347	occ		41497	Natica can	species	107010
425352	occ		41497	Tectonatic	species	95662
426105	occ		41539	Natica can	species	107010
426106	occ		41539	Natica sp.	genus	10166
426191	occ		41501	Natica can	species	107010
426192	occ		41501	Natica plic	species	156596
426193	occ		41501	Natica sp.	genus	10166
426199	occ		41501	Polinices d	species	94402
426221	occ		41501	Tectonatic	species	95662
427510	occ		41705	Polinices d	species	94402
427537	occ		41705	Tectonatic	species	95662
427627	occ		41715	Polinices d	species	94402
427631	occ		41715	Tectonatic	species	95662
427803	occ		41718	Natica can	species	107010
427937	occ		41740	Polinices d	species	94402
428101	occ		41749	Natica can	species	107010
428102	occ		41749	Natica gup	species	161754
428108	occ	18023	41749	Polinices c	species	94402
428109	occ		41749	Polinices s	species	95666
568441	occ		59485	Natica can	species	107010
568442	occ		59490	Natica can	species	107010
568443	occ		59484	Natica can	species	107010
568444	occ		59484	Tectonatic	species	95662
568445	occ		59485	Tectonatic	species	95662
568446	occ		59487	Tectonatic	species	95662
568447	occ		59485	Polinices (I	species	187709
568448	occ		59484	Polinices (I	species	187709
568449	occ		59484	Polinices (I	species	94401
568450	occ		59485	Polinices (I	species	94401
568451	occ		59486	Polinices (I	species	10291
582439	occ		61309	Tectonatic	species	95662
582440	occ		61309	Sinum pers	species	108672
582441	occ		61309	Polinices d	species	94402
582442	occ		61310	Polinices d	species	94402
582443	occ		61310	Sinum pers	species	108672
582444	occ		61310	Tectonatic	species	95662
582462	occ		61311	Polinices d	species	94402
582463	occ		61311	Tectonatic	species	95662

640517	occ	69085	Stigmaulax	genus	10506
640534	occ	69084	Natica (Na	species	95654
640774	occ	69087	Natica (Na	subgenus	91873
697896	occ	75009	Natica cf. c	species	107010
697905	occ	75001	Natica can	species	107010
697955	occ	75012	Neverita cf	species	108603
743727	occ	79804	Sinum mul	species	10470
745392	occ	79788	Polinices d	species	94402
745398	occ	79788	Sinum pers	species	108672
745673	occ	79333	Polinices (I	species	109423
745674	occ	79333	Sinum pola	species	10470
745680	occ	79331	Euspira cau	species	9839
745691	occ	79336	Euspira cau	species	9839
745692	occ	79331	Natica (Na	species	156596
745693	occ	79331	Polinices (I	species	114858
745694	occ	79331	Polinices (I	species	109423
745695	occ	79331	Tectonatic	species	95662
745696	occ	79331	Stigmaulax	species	10506
745697	occ	79331	Sigatica se	species	95671
745698	occ	79331	Sinum pers	species	108672
745704	occ	79334	Polinices (I	species	114858
745748	occ	79959	Dallitesta c	species	9738
747362	occ	69029	Naticarius	species	116602
747426	occ	79786	Stigmaulax	species	10506
747436	occ	79786	Neverita d	species	108603
747479	occ	79787	Neverita d	species	94431
747539	occ	79786	Neverita d	species	108603
748167	occ	69034	Naticarius	species	10167
751018	occ	80358	Natica sp.	genus	10166
1088984	occ	96538	Neverita d	species	108603
1088985	occ	96538	Polinices la	species	109160
1088986	occ	96538	Polinices p	species	10291
1088987	occ	96538	Polinices u	species	167813
1088988	occ	96538	Sinum pers	species	108672
1088989	occ	96538	Tectonatic	species	95662
1317139	occ	176823	Polinices s	genus	10291
256366	occ	20604	Polinices d	species	94402
256384	occ	20606	Polinices d	species	94402
256430	occ	20613	Polinices d	species	94402
619263	occ	66725	Polinices d	species	94402
1248048	occ	164698	Neverita s	genus	10177
578618	occ	60927	Polinices d	species	94402
578689	occ	60928	Polinices d	species	94402
578740	occ	60929	Polinices d	species	94402
578789	occ	60930	Polinices d	species	94402
578963	occ	60940	Euspira say	species	9839
578964	occ	60940	Euspira int	species	9839

578965	occ	60940	Sinum pers	species	108672
578966	occ	60940	Sinum ches	species	158564
579059	occ	60944	Polinices d	species	94402
579062	occ	60944	Tectonatic	species	95662
619833	occ	66758	Natica pusi	species	10166
619834	occ	66760	Natica pusi	species	95660
619835	occ	66757	Polinices h	species	94406
619837	occ	66760	Polinices d	species	94402
619838	occ	66758	Polinices d	species	94402
619839	occ	66756	Polinices d	species	94402
619840	occ	66755	Polinices d	species	94402
619848	occ	66760	Sinum pers	species	108672
619852	occ	66758	Sinum pers	species	108672
642898	occ	69046	Neverita d	species	94431
642939	occ	69046	Natica (Na	subgenus	91873
741397	occ	79705	Neverita d	species	94431
741398	occ	79706	Neverita d	species	94431
741404	occ	79705	Tectonatic	species	95662
748118	occ	80083	Naticidae i	family	61317
832873	occ	92407	Natica dup	species	157290
832874	occ	92407	Natica carc	species	157295
564613	occ	58893	Lunatia he	species	94407
564683	occ	58895	Polinices d	species	108605
564690	occ	58895	Tectonatic	species	95662
564887	occ	58898	Polinices d	species	108605
564957	occ	58897	Tectonatic	species	95662
579651	occ	60989	Polinices sp	genus	10291
579713	occ	60988	Polinices sp	genus	10291
579788	occ	60987	Polinices sp	genus	10291
619836	occ	66742	Polinices h	species	94406
619841	occ	66752	Polinices d	species	94402
619842	occ	66751	Polinices d	species	94402
619843	occ	66750	Polinices d	species	94402
619844	occ	66749	Polinices d	species	94402
619845	occ	66748	Polinices d	species	94402
619846	occ	66747	Polinices d	species	94402
619847	occ	66742	Polinices d	species	94402
619849	occ	66742	Sinum pers	species	108672
619850	occ	66749	Sinum pers	species	108672
619851	occ	66751	Sinum pers	species	108672
640566	occ	69086	Naticidae i	family	61317
642717	occ	69045	Neverita (N	species	94431
642744	occ	69045	Sinum mac	species	10470
659702	occ	71138	Polinices d	species	94402
659705	occ	71138	Lunatia he	species	94407
700960	occ	69043	Euspira he	species	94430
701053	occ	69047	Neverita d	species	94431

737442	occ	79249	Euspira he	species	94430
738427	occ	79342	Tectonatic	species	95662
738449	occ	79343	Euspira he	species	94430
738483	occ	79341	Euspira he	species	94430
738484	occ	79341	Tectonatic	species	95662
748237	occ	80034	Neverita d	species	94431
748456	occ	80033	Natica (Na	subgenus	95653
748483	occ	80033	Neverita d	species	94431
748484	occ	80033	Euspira he	species	94430
748544	occ	80035	Euspira he	species	94430
748557	occ	80035	Naticarius	genus	10167
748638	occ	80035	Neverita d	species	94431
829364	occ	91459	Polinices d	species	94402
829373	occ	91462	Polinices d	species	94402
1512854	occ	211183	Polinices s	genus	10291
94751	occ	7108	Polinices d	species	94402
94769	occ	7109	Polinices d	species	94402
559489	occ	57706	Lunatia he	species	94407
559495	occ	57706	Polinices d	species	94402
582235	occ	61293	Tectonatic	species	95662
582247	occ	61294	Tectonatic	species	95662
582253	occ	61295	Tectonatic	species	95662
640346	occ	69048	Euspira he	species	94430
640354	occ	69048	Neverita d	species	94431
640368	occ	69049	Neverita d	species	94431
727664	occ	78271	Lunatia he	species	94407
727749	occ	78270	Polinices d	species	108605
727819	occ	78269	Lunatia he	species	94407
727828	occ	78268	Lunatia int	species	10076
727829	occ	78268	Lunatia he	species	94407
727830	occ	78268	Sinum frag	species	95609
727882	occ	78266	Lunatia he	species	171691
727944	occ	78265	Lunatia he	species	171691
727987	occ	78260	Lunatia he	species	94407
728009	occ	78261	Polinices d	species	108605
728010	occ	78261	Lunatia he	species	94407
728046	occ	78262	Lunatia he	species	94407
728098	occ	78264	Lunatia int	species	10076
728099	occ	78264	Lunatia he	species	94407
737482	occ	79247	Euspira he	species	94430
741433	occ	79709	Euspira he	species	94430
748193	occ	80040	Natica (Na	subgenus	95653
748261	occ	80038	Natica (Na	subgenus	95653
748410	occ	80036	Lunatia he	species	94407
637091	occ	67069	Smaragdia	species	116051
744287	occ	79876	Neritina sp	species	9015
749672	occ	79331	Neritina sp	species	9015

749673	occ		79331	Smaragdia	species	116051
870205	occ		79960	Neritina cf.	species	167650
1088931	occ		96538	Neritina us	species	158616
1088932	occ		96538	Neritina vi	species	109162
1088933	occ		96538	Smaragdia	species	116051
1248047	occ		164698	Neritina sp	genus	9015
659696	occ		71138	Tortifusus	species	98746
700944	occ		69043	Illyanassa s	genus	11211
362422	occ		34269	Olivella mu	species	94511
362423	occ		34269	Olivella jas	species	109169
362424	occ		34269	Oliva litera	species	109348
426107	occ		41539	Olivella mu	species	94511
426108	occ		41539	Olivella sp.	species	11446
426194	occ		41501	Oliva sayar	species	93850
426195	occ		41501	Olivella mu	species	94511
427507	occ		41705	Oliva sayar	species	93850
427508	occ		41705	Olivella mu	species	94511
427626	occ		41715	Olivella mu	species	94511
427804	occ		41718	Oliva sayar	species	93850
427805	occ		41718	Olivella mu	species	94511
427936	occ		41740	Olivella mu	species	94511
428103	occ	24973	41749	Oliva imm	species	188352
428104	occ		41749	Oliva sp.	genus	11444
567515	occ		59485	Oliva sayar	species	93850
567516	occ		59490	Oliva sayar	species	93850
567517	occ		59484	Oliva sayar	species	93850
567518	occ		59491	Oliva sayar	species	93850
567519	occ		59484	Olivella mu	species	94511
567520	occ		59485	Olivella mu	species	94511
567521	occ		59484	Olivella (D	species	248905
567522	occ		59487	Olivella jac	species	11446
582473	occ		61309	Oliva sayar	species	93850
582474	occ		61309	Olivella flo	species	11446
582475	occ		61309	Olivella mu	species	94511
582476	occ		61309	Olivella pu	species	11446
582492	occ		61310	Oliva sayar	species	93850
582493	occ		61310	Olivella de	species	11446
582494	occ		61310	Olivella mu	species	94511
582495	occ		61310	Olivella pu	species	11446
582510	occ		61311	Oliva sayar	species	93850
582511	occ		61311	Olivella mu	species	94511
582512	occ		61311	Olivella pu	species	11446
640545	occ		69084	Olivella mu	species	94511
640775	occ		69087	Olivella (Ol	subgenus	89859
647839	occ		70010	Olivella mu	species	94511
697909	occ		75001	Oliva cf. ca	species	11444
697948	occ		75011	Olivella sp.	genus	11446

697962	occ	75012	Olivella sp.	genus	11446
697978	occ	75013	Olivella sp.	genus	11446
697982	occ	75014	Oliva cf. ca	species	11444
697989	occ	75005	Oliva sp.	genus	11444
698005	occ	75016	Oliva sp.	genus	11444
698024	occ	75007	Oliva sp.	genus	11444
698030	occ	75008	Oliva sp.	genus	11444
698097	occ	75025	Oliva muti	species	94510
698098	occ	75025	Oliva aff. s	species	93850
698103	occ	75026	Oliva muti	species	94510
741274	occ	79331	Oliva (Ispic	species	107831
741275	occ	79331	Olivella (Ol	species	89859
741276	occ	79331	Olivella (Ol	species	89859
741277	occ	79331	Olivella (Da	species	107850
741278	occ	79331	Olivella (To	species	89861
741279	occ	79331	Olivella (M	species	116726
741335	occ	79333	Olivella (Da	species	89868
741337	occ	79333	Olivella (To	species	89861
741351	occ	79336	Olivella (Da	species	107850
741768	occ	79731	Oliva sp.	genus	11444
741899	occ	79739	Olivella mu	species	94511
741946	occ	79740	Oliva (Stre	species	11701
741947	occ	79740	Oliva (Stre	species	93847
741948	occ	79740	Oliva (Stre	species	11701
741949	occ	79740	Oliva (Stre	species	11701
741950	occ	79740	Oliva (Stre	species	11701
741951	occ	79740	Lindoliva d	species	157096
741979	occ	79748	Oliva (Stre	species	11701
741989	occ	79748	Oliva (Porp	species	11444
741990	occ	79748	Oliva (Stre	species	11701
742028	occ	79747	Lindoliva g	species	157095
742029	occ	79747	Lindoliva s	species	157094
742030	occ	79747	Oliva (Stre	species	11701
742031	occ	79747	Oliva (Stre	species	11701
742032	occ	79747	Oliva (Stre	species	11701
742033	occ	79747	Oliva (Stre	species	11701
742046	occ	79747	Oliva (Porp	species	11444
742502	occ	79761	Olivella tar	species	11446
742503	occ	79761	Olivella gla	species	11446
742507	occ	79766	Olivella tar	species	11446
742551	occ	35031	Oliva sp.	genus	11444
742583	occ	79782	Oliva (Stre	species	11701
743306	occ	79804	Oliva (Porp	species	11444
743307	occ	79804	Oliva (Stre	species	11701
743308	occ	79804	Oliva (Stre	species	11701
743708	occ	79847	Oliva (Stre	species	11701
745366	occ	79788	Oliva (Oliva	species	107831

745580	occ	79960	Oliva (Strep	species	11701
745595	occ	79960	Oliva (Strep	species	11701
745597	occ	79960	Oliva (Strep	species	11701
745765	occ	79959	Oliva carol	species	11444
747018	occ	80002	Olivella mu	species	94511
747047	occ	80001	Olivella mu	species	94511
747214	occ	69085	Olivella mu	species	94511
747263	occ	80027	Olivella mu	species	94511
747307	occ	69044	Oliva sayar	species	93850
747308	occ	69044	Olivella sp.	genus	11446
747378	occ	69029	Oliva sayar	species	93850
747382	occ	69029	Olivella mu	species	94511
747433	occ	79786	Oliva (Ispic	species	107831
747762	occ	80046	Oliva sayar	species	93850
747765	occ	80046	Oliva linda	species	11444
835168	occ	92333	Oliva (Omo	species	158485
870203	occ	79960	Mansfielde	genus	116725
870281	occ	35031	Lindoliva g	species	157095
870284	occ	35031	Oliva (Strep	species	93847
870286	occ	35031	Lindoliva sp	species	157094
870287	occ	35031	Lindoliva d	species	157096
1011563	occ	123549	Oliva sayar	species	93850
1089034	occ	96538	Oliva bollir	species	11444
1089035	occ	96538	Oliva sayar	species	93850
1089036	occ	96538	Olivella mu	species	94511
1089037	occ	96538	Olivella pu	species	11446
256365	occ	20604	Oliva sayar	species	93850
256383	occ	20606	Oliva sayar	species	93850
256429	occ	20613	Oliva sayar	species	93850
578545	occ	60923	Olivella mu	species	94511
578616	occ	60927	Oliva sayar	species	93850
578617	occ	60927	Olivella mu	species	94511
578688	occ	60928	Olivella mu	species	94511
578738	occ	60929	Oliva sayar	species	93850
578739	occ	60929	Olivella mu	species	94511
578787	occ	60930	Oliva sayar	species	93850
578788	occ	60930	Olivella mu	species	94511
578979	occ	60940	Oliva canal	species	11444
579057	occ	60944	Oliva sayar	species	93850
619992	occ	66760	Oliva sayar	species	93850
619993	occ	66758	Oliva sayar	species	93850
619994	occ	66756	Oliva sayar	species	93850
620002	occ	66760	Olivella mu	species	94511
620003	occ	66758	Olivella mu	species	94511
642890	occ	69046	Oliva (Oliva	species	89857
642891	occ	69046	Olivella (Ol	species	94511
741365	occ	79706	Oliva (Oliva	species	93851

741372	occ	79705	Oliva (Oliva	species	93851
745467	occ	79956	Oliva (Ispic	species	116724
745474	occ	79956	Olivella mu	species	94511
745497	occ	79957	Olivella mu	species	94511
747822	occ	80053	Oliva carol	species	11444
748096	occ	80083	Oliva (Oliva	species	89857
748115	occ	80083	Olivella car	species	248899
832868	occ	92406	Oliva litter	species	97469
832879	occ	92407	Oliva litter	species	97469
832905	occ	92408	Oliva litter	species	97469
832906	occ	92408	Olivella mu	species	94511
832975	occ	92411	Oliva litter	species	97469
564614	occ	58893	Olivella mu	species	94511
564630	occ	58894	Olivella mu	species	94511
564681	occ	58895	Oliva carol	species	11444
564682	occ	58895	Olivella mu	species	94511
564952	occ	58897	Olivella mu	species	94511
567203	occ	59309	Oliva robes	species	11444
567204	occ	59309	Olivella car	species	248899
579658	occ	60989	Olivella sp.	genus	11446
619995	occ	66754	Oliva sayar	species	93850
619996	occ	66750	Oliva sayar	species	93850
619997	occ	66749	Oliva sayar	species	93850
619998	occ	66748	Oliva sayar	species	93850
619999	occ	66747	Oliva sayar	species	93850
620000	occ	66745	Oliva sayar	species	93850
620001	occ	66742	Oliva sayar	species	93850
620004	occ	66752	Olivella mu	species	94511
620005	occ	66750	Olivella mu	species	94511
620006	occ	66749	Olivella mu	species	94511
620007	occ	66747	Olivella mu	species	94511
620008	occ	66746	Olivella mu	species	94511
620009	occ	66742	Olivella mu	species	94511
642730	occ	69045	Oliva (Oliva	species	89857
642738	occ	69045	Olivella (Ol	species	94511
659746	occ	71138	Olivella mu	species	94511
701062	occ	69047	Olivella mu	species	94511
702868	occ	68979	Olivella car	species	248899
702899	occ	69018	Oliva robes	species	11444
702901	occ	69018	Olivella car	species	248899
737416	occ	79249	Olivella car	species	248899
737421	occ	79248	Olivella car	species	248899
738413	occ	79342	Olivella car	species	248899
738442	occ	79343	Olivella car	species	248899
738460	occ	79341	Olivella car	species	248899
748393	occ	80033	Olivella sp.	genus	11446
748535	occ	80033	Olivella car	species	248899

94755	occ	7108	Olivella mu	species	94511
559493	occ	57706	Oliva sayar	species	93850
559494	occ	57706	Olivella mu	species	94511
582236	occ	61293	Olivella sp.	genus	11446
640367	occ	69049	Olivella mu	species	94511
727660	occ	78271	Olivella sp.	genus	11446
727811	occ	78269	Olivella sp.	genus	11446
727928	occ	78266	Olivella sp.	genus	11446
727936	occ	78265	Oliva canal	species	11444
727937	occ	78265	Olivella sp.	genus	11446
727985	occ	78260	Oliva canal	species	11444
728000	occ	78261	Olivella sp.	genus	11446
728095	occ	78264	Olivella sp.	genus	11446
747737	occ	80047	Olivella mu	species	94511
748259	occ	80038	Oliva carol	species	11444
622888	occ	67070	Cyphoma i	species	91130
742042	occ	79747	Jenneria he	species	342958
742043	occ	79747	Jenneria lo	species	342635
743224	occ	79804	Jenneria ri	species	157085
743699	occ	79847	Jenneria lir	species	157084
745845	occ	79958	Cyphoma f	species	342623
745846	occ	79958	Cyphoma r	species	343161
745847	occ	79958	Cyphoma v	species	342053
745848	occ	79958	Pseudocyp	species	341766
745849	occ	79958	Jenneria vi	species	342052
870268	occ	35031	Jenneria lo	species	342635
1088949	occ	96538	Cyphoma g	species	335372
1088981	occ	96538	Cymbovula	species	91209
1150731	occ	146873	Cyphoma v	species	342053
1150732	occ	146873	Cyphoma c	species	341765
1150733	occ	146873	Cyphoma f	species	342623
1333530	occ	92342	Simnia (Spi	species	343488
619886	occ	66760	Simnia acic	species	109666
619887	occ	66758	Simnia acic	species	109666
637087	occ	67069	Tricolia tha	species	135794
749681	occ	79331	Tricolia (Tr	species	9322
426198	occ	41501	Physa sp.	genus	91741
427806	occ	41718	Physa meig	species	91741
428107	occ	41749	Physa meig	species	91741
533412	occ	20400	Physella sp	genus	83647
582544	occ	61310	Physella sp	genus	83647
582559	occ	61311	Physella sp	genus	83647
751021	occ	80357	Physa sp.	genus	91741
751043	occ	80356	Physa sp.	genus	91741
1089071	occ	96538	Physella he	species	83647
832900	occ	92408	Physa aff. ı	species	91741
929557	occ	82258	Physa hete	species	91741

568156	occ	59484	Fossarus (I	species	97645
727821	occ	78269	Fossarus ly	species	60387
727867	occ	78267	Fossarus lu	species	60387
727948	occ	78265	Fossarus ly	species	60387
728048	occ	78262	Fossarus ly	species	60387
426156	occ	41501	Helisoma c	species	83652
426157	occ	41501	Helisoma c	species	83652
427488	occ	41705	Helisoma c	species	83652
427777	occ	41718	Helisoma c	species	83652
427778	occ	41718	Helisoma c	species	83652
427779	occ	41718	Helisoma s	genus	83652
428085	occ	41749	Helisoma c	species	83652
533411	occ	20400	Planorbella	species	83653
582526	occ	61309	Planorbella	species	83653
582527	occ	61309	Planorbella	species	83653
582545	occ	61310	Micromen	species	251930
582546	occ	61310	Planorbella	species	83653
582547	occ	61310	Planorbella	species	83653
582560	occ	61311	Planorbella	species	83653
582561	occ	61311	Planorbella	species	83653
747041	occ	80001	Helisoma s	species	83652
747454	occ	79786	Planorbis s	genus	61805
751017	occ	80358	Helisoma s	species	83652
751042	occ	80356	Helisoma s	species	83652
751050	occ	80355	Helisoma s	species	83652
1089072	occ	96538	Biomphala	species	251927
1089073	occ	96538	Gyraulus p	species	70675
1089074	occ	96538	Micromen	species	251930
1089075	occ	96538	Micromen	species	251930
1089076	occ	96538	Planorbella	species	83653
1089077	occ	96538	Planorbella	species	83653
1089078	occ	96538	Planorbella	species	83653
1089079	occ	96538	Planorbella	species	83653
1089080	occ	96538	Ferrissia he	species	83656
1089081	occ	96538	Hebetoncy	species	251928
1089082	occ	96538	Laevapex p	species	251929
1607453	occ	227375	Planorbella	species	83653
1607469	occ	227381	Planorbella	species	83653
1607471	occ	227382	Planorbella	species	83653
1607476	occ	227384	Planorbella	species	83653
1607481	occ	227386	Planorbella	species	83653
1607483	occ	227387	Planorbella	species	83653
1607491	occ	227388	Planorbella	species	83653
1607494	occ	227389	Planorbella	species	83653
1607500	occ	227390	Planorbella	species	83653
1607505	occ	227391	Planorbella	species	83653
1607506	occ	227391	Planorbella	species	83653

1607516	occ	227392	Planorbella	species	83653
1607522	occ	227393	Gyraulus p	species	70675
1607523	occ	227393	Micromen	species	251930
1607531	occ	227395	Planorbella	species	83653
1607550	occ	227396	Laevapex c	species	251929
1607551	occ	227396	Gyraulus p	species	70675
1607552	occ	227396	Micromen	species	251930
1607553	occ	227396	Planorbella	species	83653
1607572	occ	227397	Gyraulus p	species	70675
1607593	occ	227400	Laevapex c	species	251929
1607594	occ	227400	Micromen	species	251930
1607595	occ	227400	Micromen	species	251930
1607596	occ	227400	Planorbella	species	83653
1248054	occ	164698	Planorbis a	species	61805
832899	occ	92408	Planorbis s	genus	61805
582548	occ	61310	Polygyra ce	species	251926
1089097	occ	96538	Daedaloch	species	251924
1089098	occ	96538	Polygyra ce	species	251926
1089099	occ	96538	Polygyra se	species	251926
1607454	occ	227375	Daedaloch	species	251924
1607479	occ	227385	Daedaloch	species	251924
1607480	occ	227385	Triodopsis	species	384100
1607484	occ	227387	Polygyra se	species	251926
1607485	occ	227387	Triodopsis	species	384100
1607489	occ	227388	Polygyra se	species	251926
1607496	occ	227389	Daedaloch	species	251924
1607497	occ	227389	Polygyra se	species	251926
1607502	occ	227390	Daedaloch	species	251924
1607503	occ	227390	Polygyra se	species	251926
1607509	occ	227391	Daedaloch	species	251924
1607510	occ	227391	Polygyra se	species	251926
1607518	occ	227392	Daedaloch	species	251924
1607519	occ	227392	Triodopsis	species	384100
1607535	occ	227395	Daedaloch	species	251924
1607536	occ	227395	Polygyra se	species	251926
1607538	occ	227395	Triodopsis	species	384100
1607566	occ	227396	Daedaloch	species	251924
1607567	occ	227396	Polygyra se	species	251926
1607568	occ	227396	Triodopsis	species	384100
1607575	occ	227397	Daedaloch	species	251924
1607576	occ	227397	Triodopsis	species	384100
1607584	occ	227398	Daedaloch	species	251924
1607586	occ	227399	Polygyra se	species	251926
1607605	occ	227400	Triodopsis	species	384100
915133	occ	103991	Triodopsis	species	384100
915134	occ	103991	Triodopsis	species	384100
915135	occ	103991	Triodopsis	species	384100

929558	occ	82258	Triodopsis	species	384100
929559	occ	82258	Triodopsis	species	384100
590995	occ	62458	Cerithidea	species	134870
590996	occ	62458	Cerithidea	species	82780
741880	occ	79739	Cerithidea	species	134869
741917	occ	79739	Cerithidea	species	134870
741918	occ	79739	Cerithidea	species	82780
742000	occ	79747	Cerithidea	species	9615
743216	occ	79804	Cerithidea	species	9615
743217	occ	79804	Cerithidea	species	9615
744288	occ	79876	Cerithidea	species	9615
744363	occ	79875	Cerithidea	species	9615
744364	occ	79875	Cerithidea	species	9615
749421	occ	79331	Cerithidea	species	337633
749461	occ	79333	Cerithidea	species	337633
1088943	occ	96538	Cerithidea	species	134869
1088944	occ	96538	Cerithidea	species	82780
1248049	occ	164698	Potamides	species	311061
1248050	occ	164698	Potamides	species	311055
426116	occ	41501	Compsodri	species	10958
426130	occ	41501	Crassispira	species	108731
426179	occ	41501	Monilispira	species	121445
426211	occ	41501	Sedilia acu	species	120708
582500	occ	61310	Crassispira	genus	10983
750853	occ	41718	Carinodrilli	species	248862
750855	occ	79331	Carinodrilli	species	248862
750856	occ	79331	Compsodri	species	10958
750857	occ	79331	Compsodri	species	10958
750858	occ	79331	Compsodri	species	10958
750859	occ	79331	Monilispira	species	121445
750879	occ	79331	Lioglyphos	species	11272
750884	occ	79336	Compsodri	species	10958
750887	occ	79338	Compsodri	species	10958
750890	occ	41718	Compsodri	species	10958
1089050	occ	96538	Pilsbryspira	species	107921
1150792	occ	146875	Carinodrilli	genus	107399
567216	occ	59316	Crassispira	species	108731
748461	occ	80033	Crassispira	genus	10983
748560	occ	80035	Compsodri	species	108749
748561	occ	80035	Crassispira	genus	10983
748637	occ	80033	Compsodri	genus	10958
1089087	occ	96538	Pupoides a	species	162063
1089088	occ	96538	Vertigo mil	species	162064
1089089	occ	96538	Vertigo ova	species	162064
1607524	occ	227393	Pupoides a	species	162063
1607560	occ	227396	Pupoides s	genus	162063
1607561	occ	227396	Pupoides a	species	162063

1607562	occ	227396	Vertigo ova	species	162064
1607601	occ	227400	Pupoides s	genus	162063
1607602	occ	227400	Pupoides a	species	162063
915122	occ	103991	Vertigo tric	species	162064
915123	occ	103991	Vertigo gor	species	162064
425377	occ	41498	Odostomia	species	12153
426005	occ	41501	Bartschella	species	12046
426163	occ	41501	Longchaeu	species	91319
426164	occ	41501	Longchaeu	genus	12130
426246	occ	41501	Turbonilla	species	12221
427471	occ	41705	Chrysallida	species	12064
427490	occ	41705	Longchaeu	species	12130
427506	occ	41705	Odostomia	genus	12153
427509	occ	41705	Oscilla bise	species	414220
427548	occ	41705	Turbonilla	species	97612
427549	occ	41705	Turbonilla	genus	12221
427619	occ	41715	Chrysallida	species	12064
427622	occ	41715	Longchaeu	species	91319
427625	occ	41715	Mormula s	genus	12139
427633	occ	41715	Turbonilla	species	135314
427634	occ	41715	Turbonilla	genus	12221
427814	occ	41718	Turbonilla	genus	12221
427930	occ	41740	Longchaeu	species	91319
427944	occ	41740	Turbonilla	species	135311
427945	occ	41740	Turbonilla	species	12221
432397	occ	41501	Turbonilla	genus	12221
567671	occ	59486	Pyramidell	genus	12182
567672	occ	59484	Pyramidell	genus	12182
567673	occ	59484	Turbonilla	subgenus	97612
567674	occ	59486	Turbonilla	subgenus	97612
567675	occ	59484	Turbonilla	subgenus	85655
567676	occ	59486	Turbonilla	subgenus	85655
567677	occ	59486	Turbonilla	subgenus	85655
567678	occ	59484	Turbonilla	subgenus	12221
567679	occ	59486	Turbonilla	subgenus	85655
567680	occ	59484	Turbonilla	subgenus	85655
582487	occ	61309	Odostomia	species	109789
582488	occ	61309	Turbonilla	genus	12221
582506	occ	61310	Odostomia	genus	12153
582507	occ	61310	Odostomia	species	12153
582508	occ	61310	Odostomia	species	109789
582509	occ	61310	Pyramidell	species	109662
582517	occ	61311	Turbonilla	genus	12221
582518	occ	61311	Pyramidell	species	109662
582519	occ	61311	Odostomia	species	109789
582520	occ	61311	Odostomia	species	12153
582537	occ	61310	Turbonilla	genus	12221

747019	occ	80002	"Turbonilla	genus	12221
747044	occ	80001	"Turbonilla	genus	12221
747514	occ	79787	Pyramidell	genus	12182
1089057	occ	96538	Boonea se	species	109790
1089058	occ	96538	Longchaeu	species	12130
1089059	occ	96538	Odostomia	species	156561
1089060	occ	96538	Odostomia	species	12153
1089061	occ	96538	Turbonilla	species	135313
578686	occ	60928	Odostomia	species	109771
578687	occ	60928	Odostomia	species	12153
578693	occ	60928	Turbonilla	species	12221
578694	occ	60928	Turbonilla	species	135974
578737	occ	60929	Odostomia	species	109789
578742	occ	60929	Turbonilla	species	135974
578785	occ	60930	Odostomia	species	109771
578786	occ	60930	Odostomia	species	109789
578790	occ	60930	Turbonilla	species	135974
579066	occ	60944	Turbonilla	species	135974
579067	occ	60944	Turbonilla	genus	12221
619828	occ	66760	Pyramidell	species	109662
619829	occ	66758	Pyramidell	species	12182
741396	occ	79707	Turbonilla	genus	12221
741407	occ	79705	Turbonilla	genus	12221
1457437	occ	79745	Turbonilla	species	392401
1457438	occ	79745	Turbonilla	species	392402
1457439	occ	79745	Odostomia	species	392405
1457448	occ	150656	Turbonilla	species	392393
1457481	occ	203377	Turbonilla	species	392393
1457482	occ	203377	Turbonilla	species	392397
1457483	occ	203377	Turbonilla	species	392401
1457484	occ	203377	Turbonilla	species	392402
1457485	occ	203377	Odostomia	species	392405
564616	occ	58893	Turbonilla	species	12221
564635	occ	58894	Turbonilla	species	12221
564685	occ	58895	Pyrgiscus c	species	12185
564686	occ	58895	Pyrgiscus s	genus	12185
564692	occ	58895	Turbonilla	species	12221
564951	occ	58897	Odostomia	species	82826
564963	occ	58897	Turbonilla	species	12221
579569	occ	60986	Turbonilla	subgenus	85655
579602	occ	60986	Boonea se	species	109790
579603	occ	60986	Boonea im	species	108660
579604	occ	60986	Odostomia	genus	12153
579661	occ	60989	Boonea se	species	109790
579662	occ	60989	Boonea im	species	108660
579663	occ	60989	Odostomia	genus	12153
579664	occ	60989	Turbonilla	subgenus	97612

579665	occ		60989	Turbonilla	subgenus	85655
579666	occ		60989	Turbonilla	subgenus	85655
579724	occ		60988	Boonea se	species	109790
579725	occ		60988	Boonea im	species	108660
579726	occ	22327	60988	Odostomia	species	156561
579727	occ		60988	Odostomia	genus	12153
579728	occ		60988	Turbonilla	subgenus	97612
579729	occ		60988	Turbonilla	subgenus	85655
579730	occ		60988	Turbonilla	subgenus	85655
579797	occ		60987	Boonea se	species	109790
579798	occ		60987	Boonea im	species	108660
579799	occ		60987	Turbonilla	subgenus	97612
579800	occ		60987	Turbonilla	subgenus	85655
579804	occ		60987	Turbonilla	subgenus	85655
619830	occ		66752	Pyramidell	species	12182
619831	occ		66751	Pyramidell	species	12182
619832	occ		66746	Pyramidell	species	12182
659695	occ		71138	Turbonilla	species	135974
659726	occ		71138	Turbonilla	species	392397
738486	occ		79341	Turbonilla	genus	12221
1512825	occ		211029	Boonea se	species	109790
1512834	occ		211183	Boonea se	species	109790
1512843	occ		211183	Boonea im	species	108660
1512847	occ		211183	Pyrgiscus s	genus	12185
1512849	occ		211183	Chemnitzia	genus	12063
1512932	occ		211181	Boonea se	species	109790
1512970	occ		211182	Boonea se	species	109790
1512976	occ		211182	Chemnitzia	genus	12063
1512978	occ		211182	Boonea im	species	108660
559500	occ		57706	Turbonilla	species	135974
582233	occ		61293	Turbonilla	genus	12221
582237	occ		61293	Odostomia	genus	12153
582267	occ		61296	Odostomia	genus	12153
567685	occ		59486	Cymatium	species	159059
745750	occ		79959	Cymatium	species	159059
836616	occ		92693	Monoplex	species	159070
836617	occ		92344	Monoplex	species	159070
1086715	occ		35025	Monoplex	species	159062
1086716	occ		35031	Monoplex	species	159062
427479	occ		41705	Daphnella	species	11020
426257	occ	22321	41501	Volvulella t	species	12027
567328	occ		59484	Volvula ox	species	107150
738385	occ	22322	79331	Volvulella t	species	12027
640376	occ		69049	Retusa obt	species	94509
426205	occ		41501	Ringicula fl	species	120660
427531	occ		41705	Ringicula fl	species	120660
567329	occ		59486	Ringicula fl	species	120660

567330	occ	59484	Ringicula fl	species	120660
738389	occ	79331	Ringicula (I	species	120661
579731	occ	60988	Ringicula s	genus	11997
425350	occ	41497	Rissoina bu	species	10421
426206	occ	41501	Rissoina bu	species	10421
426207	occ	41501	Rissoina flo	species	91281
426208	occ	41501	Rissoina la	species	91276
426209	occ	41501	Rissoina sp	genus	10421
427532	occ	41705	Rissoina lir	species	10421
568162	occ	59487	Rissoina (Z	species	123130
568163	occ	59484	Rissoina (Z	species	123130
568421	occ	59484	Rissoa lipe	species	10419
582356	occ	61310	Rissoina ca	species	91188
582367	occ	61311	Rissoina ca	species	91188
749646	occ	79331	Rissoina (Z	species	100866
749647	occ	79331	Rissoina (Z	species	100866
749648	occ	79331	Rissoina (Z	species	100866
749649	occ	79331	Rissoina (S	species	91281
749650	occ	79331	Rissoina (C	species	117248
749651	occ	79331	Rissoina (C	species	116019
749682	occ	79338	Rissoina (Z	species	100866
749689	occ	79338	Rissoina (P	species	248848
749695	occ	79338	Rissoina (S	species	91281
749696	occ	79338	Rissoina pa	species	10421
1088955	occ	96538	Schwartzie	species	91189
1088956	occ	96538	Schwartzie	species	10440
564954	occ	58897	Rissoa gera	species	116530
567038	occ	59316	Rissoa gera	species	116530
567039	occ	59313	Rissoina (S	species	91280
559822	occ	57705	Cingula no	species	9647
425338	occ	41497	Alabina ad	species	90881
425339	occ	41497	Alabina cer	species	157388
425998	occ	41501	Alabina ad	species	90881
425999	occ	41501	Alabina cer	species	157388
427439	occ	41705	Alabina ad	species	90881
568123	occ	59485	Alabina ad	species	90881
568124	occ	59491	Alabina ad	species	90881
568125	occ	59484	Alabina ad	species	90881
568127	occ	59490	Alabina ad	species	90881
749409	occ	79338	Alabina (Ca	species	90881
749414	occ	79338	Alabina (Ca	species	90881
749430	occ	79331	Alabina cer	species	157388
749432	occ	79331	Alabina (Ca	species	90881
619888	occ	66760	Bittium cer	species	156801
426215	occ	41501	Solariorbis	species	10479
568461	occ	59484	Circulus ? l	species	158631
637110	occ	67069	Solariorbis	species	156789

637111	occ	67069	Solariorbis	species	156786
637112	occ	67069	Solariorbis	species	10479
637118	occ	67069	Vitrinella b	species	135103
749680	occ	79331	Parviturbo	species	117454
750969	occ	41718	Solariorbis	species	10479
750977	occ	79331	Solariorbis	species	10479
750978	occ	79331	Solariorbis	species	10479
750979	occ	79331	Solariorbis	species	10479
750980	occ	79331	Solariorbis	species	156789
750981	occ	79331	Solariorbis	species	156786
1088960	occ	96538	Solariorbis	species	156785
568456	occ	59487	Solariella v	species	167649
637085	occ	67069	Solariella s	genus	9254
697930	occ	75010	"Solariella'	genus	9254
579009	occ	60941	Solariella g	species	156290
748556	occ	80035	Solariella g	species	156290
582528	occ	61309	Euglandina	species	251918
1089092	occ	96538	Euglandina	species	251918
1607488	occ	227388	Euglandina	species	251918
1607517	occ	227392	Euglandina	species	251918
1607532	occ	227395	Euglandina	species	251918
1607564	occ	227396	Euglandina	species	251918
1089090	occ	96538	Strobilops	species	114481
1089091	occ	96538	Strobilops	species	114481
1607563	occ	227396	Strobilops	species	114481
915136	occ	103991	Strobilops	species	114481
423333	occ	41202	Strombus s	genus	10520
425351	occ	41497	Strombus a	species	82785
426109	occ	41539	Strombus a	species	82785
426216	occ	41501	Strombus a	species	82785
426217	occ	41501	Strombus l	species	10520
426218	occ	41501	Strombus p	species	94554
427536	occ	41705	Strombus a	species	82785
427810	occ	41718	Strombus a	species	82785
427942	occ	41740	Strombus a	species	82785
428115	occ	41749	Strombus a	species	82785
567687	occ	59485	Strombus p	species	94554
567688	occ	59484	Strombus p	species	94554
582373	occ	61311	Strombus a	species	82785
582392	occ	61309	Strombus a	species	82785
582413	occ	61310	Strombus a	species	82785
591020	occ	62462	Strombus a	species	82785
591049	occ	62465	Strombus l	species	82787
622889	occ	67070	Strombus r	species	10520
640536	occ	69084	Strombus a	species	82785
640719	occ	69087	Strombus s	genus	10520
640720	occ	69087	Strombus s	genus	10520

647826	occ	70010	Strombus a	species	82785
741868	occ	79738	Strombus a	species	82785
741869	occ	79738	Strombus l	species	82787
741870	occ	79738	Macrostro	species	120030
741886	occ	79739	Strombus a	species	82785
741887	occ	79739	Strombus (	species	122144
741888	occ	79739	Eustrombu	species	109145
741889	occ	79739	Macrostro	species	120030
741925	occ	79740	Strombus e	species	10520
741926	occ	79740	Strombus l	species	82787
741927	occ	79740	Macrostro	species	120029
741995	occ	79747	Strombus e	species	10520
741996	occ	79747	Macrostro	species	120029
742009	occ	79747	Macrostro	species	120029
742010	occ	79747	Macrostro	species	120029
742011	occ	79747	Eustrombu	species	109145
742012	occ	79747	Titanostro	species	146700
742521	occ	79335	Strombus l	species	10520
742554	occ	35031	Fusus wate	species	11143
742557	occ	35031	Strombus r	species	10520
742566	occ	79782	Macrostro	species	120029
743227	occ	79804	Macrostro	species	120029
743228	occ	79804	Strombus l	species	10520
743244	occ	79805	Macrostro	species	120029
744292	occ	79876	Strombus s	species	187626
745324	occ	79789	Strombus p	species	109157
745675	occ	79333	Strombus p	species	109157
745699	occ	79331	Strombus p	species	109157
745717	occ	79959	Macrostro	species	120029
745747	occ	79959	Strombus f	species	187625
745953	occ	79958	Eustrombu	species	111499
747524	occ	79786	Strombus p	species	94554
747733	occ	80046	Strombus e	species	10520
748295	occ	80025	Strombus p	species	94554
870254	occ	79960	Strombus (	subgenus	9842
870288	occ	35031	Strombus s	genus	10520
870289	occ	35031	Strombus (	species	9842
870290	occ	35031	Strombus (	subgenus	9842
870291	occ	35031	Strombus a	species	82785
1088967	occ	96538	Lobatus co	species	146702
1088968	occ	96538	Lobatus gi	species	146697
1088969	occ	96538	Lobatus ra	species	146670
1088970	occ	96538	Strombus a	species	82785
1150798	occ	146875	Strombus f	species	187625
1150799	occ	146875	Strombus (	subgenus	105938
619885	occ	66760	Strombus p	species	94554
619983	occ	66760	"Fusus" mi	species	392380

619984	occ	66760	"Fusus" ru	species	392384
619985	occ	66758	"Fusus" bu	species	392383
619986	occ	66758	"Fusus" co	species	392381
619987	occ	66760	"Fusus" fili	species	392382
428116	occ	41749	Tegula fas	species	9297
637084	occ	67069	Tegula fas	species	9297
743210	occ	79804	Tegula calu	species	9297
745705	occ	79959	Tegula lind	species	9297
745783	occ	79959	Tegula exo	species	9297
749683	occ	79331	Tegula (Ag	species	109206
1088928	occ	96538	Tegula fas	species	9297
362419	occ	34269	Terebra cir	species	101228
362420	occ	34269	Terebra co	species	94428
362421	occ	34269	Terebra dis	species	108678
423331	occ	41202	Terebra dis	species	108678
426239	occ	41501	Terebra dis	species	108678
426240	occ	41501	Terebra sp	genus	11754
427541	occ	41705	Terebra co	species	94428
427542	occ	41705	Terebra dis	species	108678
427543	occ	41705	Terebra sp	species	11754
427544	occ	41705	Terebra sp	genus	11754
427632	occ	41715	Terebra dis	species	108678
427811	occ	41718	Terebra co	species	94428
427812	occ	41718	Terebra dis	species	108678
427813	occ	41718	Terebra sp	genus	11754
427943	occ	41740	Terebra ? s	genus	11754
428117	occ	41749	Terebra dis	species	108678
428118	occ	41749	Terebra sp	genus	11754
428124	occ	41749	Terebra un	species	11754
428476	occ	41749	Terebra sp	genus	11754
567331	occ	59486	Myurella b	species	11395
567332	occ	59484	Myurella d	species	11395
567333	occ	59484	Myurella p	species	11395
582486	occ	61309	Terebra sp	genus	11754
582503	occ	61310	Terebra co	species	94428
582504	occ	61310	Terebra dis	species	108678
582505	occ	61310	Terebra pr	species	107184
582521	occ	61311	Terebra dis	species	108678
582522	occ	61311	Terebra sp	genus	11754
647842	occ	70010	Terebra dis	species	108678
697885	occ	74999	Terebra co	species	94428
697910	occ	75001	Terebra af	species	107184
697949	occ	75011	Terebra dis	species	108678
738390	occ	79331	Terebra (St	species	107306
738391	occ	79331	Terebra (St	species	107183
741261	occ	79331	Terebra (St	species	94429
741262	occ	79331	Terebra (St	species	92443

741984	occ	79748	Strioterebr	species	93791
742174	occ	79765	Terebra (M	species	11396
742192	occ	79761	Terebra (M	species	93786
742195	occ	79766	Terebra (M	species	93787
742201	occ	79761	Strioterebr	species	93790
742202	occ	79761	Strioterebr	species	93792
745637	occ	79960	Myurellina	species	90106
747272	occ	80027	Terebra dis	species	108678
870182	occ	79960	Myurellina	species	93786
870297	occ	96538	Terebra (St	species	92443
870298	occ	96538	Terebra (St	species	92443
870299	occ	96538	Terebra (St	species	107306
1089046	occ	96538	Hastula cin	species	101229
1089047	occ	96538	Terebra co	species	94428
1089048	occ	96538	Terebra pr	species	107184
1150784	occ	146875	Hastula sp.	genus	11182
1150785	occ	146875	Strioterebr	genus	11711
1150786	occ	146875	Myurellina	genus	90106
256367	occ	20604	Terebra dis	species	108678
256385	occ	20606	Terebra dis	species	108678
256431	occ	20613	Terebra dis	species	108678
579064	occ	60944	Terebra co	species	94428
579065	occ	60944	Terebra dis	species	108678
620010	occ	66760	Terebra co	species	94428
620015	occ	66760	Terebra dis	species	108678
620016	occ	66758	Terebra dis	species	108678
620017	occ	66755	Terebra dis	species	108678
620025	occ	66760	Terebra pr	species	107184
642899	occ	69046	Strioterebr	genus	11711
832902	occ	92408	Terebra dis	species	108678
564689	occ	58895	Strioterebr	genus	11711
567241	occ	59310	Terebra (Pa	species	90112
567242	occ	59309	Terebra (Pa	species	90112
567243	occ	59307	Terebra (Pa	species	90112
567244	occ	59310	Terebra (St	species	92443
567245	occ	59307	Terebra (St	species	92443
567246	occ	59329	Terebra (St	species	92443
567247	occ	59309	Terebra (St	species	92443
567248	occ	59309	Terebra (St	species	92443
567249	occ	59313	Terebra (St	species	107183
567250	occ	59316	Terebra (St	species	107183
620011	occ	66752	Terebra co	species	94428
620012	occ	66751	Terebra co	species	94428
620013	occ	66749	Terebra co	species	94428
620014	occ	66742	Terebra co	species	94428
620018	occ	66752	Terebra dis	species	108678
620019	occ	66751	Terebra dis	species	108678

620020	occ		66750	Terebra dis	species	108678
620021	occ		66749	Terebra dis	species	108678
620022	occ		66748	Terebra dis	species	108678
620023	occ		66747	Terebra dis	species	108678
620024	occ		66742	Terebra dis	species	108678
642718	occ		69045	Strioterebr	genus	11711
702903	occ		69018	Strioterebr	species	11711
737412	occ		79248	Strioterebr	species	11711
737413	occ		79249	Strioterebr	species	11711
738426	occ		79342	Strioterebr	species	11711
738509	occ		79339	Strioterebr	species	11711
748242	occ		80034	Strioterebr	genus	11711
748355	occ		80033	Strioterebr	species	11711
748356	occ		80033	Strioterebr	species	120663
748587	occ		80035	Strioterebr	genus	11711
559498	occ		57706	Terebra co	species	94428
559499	occ		57706	Terebra dis	species	108678
582234	occ		61293	Terebra ? s	genus	11754
640352	occ		69048	Terebra co	species	94428
640377	occ		69049	Terebra sp	genus	11754
727808	occ		78269	Terebra sp	genus	11754
727925	occ		78266	Terebra sp	genus	11754
728093	occ		78264	Terebra sp	genus	11754
741445	occ		79709	Strioterebr	species	11711
622890	occ		67070	Tonna gale	species	91197
742044	occ	27756	79747	Malea spri	species	250577
743226	occ		79804	Malea spri	species	250577
870259	occ		35025	Malea sp.	genus	10086
1088992	occ		96538	Tonna gale	species	91197
1150742	occ		146873	Malea sp.	genus	10086
425353	occ		41497	Teinostom	species	10554
426235	occ		41501	Teinostom	species	135872
426236	occ		41501	Teinostom	species	156720
426237	occ		41501	Teinostom	species	10554
426238	occ		41501	Teinostom	species	10554
427538	occ		41705	Teinostom	species	135876
427539	occ		41705	Teinostom	species	10554
427540	occ		41705	Teinostom	species	10554
568462	occ		59486	Teinostom	species	10554
568463	occ		59484	Teinostom	species	107015
582363	occ		61310	Teinostom	species	135863
637113	occ		67069	Teinostom	species	135867
637114	occ		67069	Teinostom	species	135873
637115	occ		67069	Teinostom	species	135865
637116	occ		67069	Teinostom	species	10554
637117	occ		67069	Teinostom	species	10554
637121	occ		67069	Cochliolepi	species	109665

637122	occ	67069	Cochliolepi	species	97698
747392	occ	69029	Teinostom	genus	10554
750971	occ	79331	Teinostom	species	10554
750972	occ	79331	Teinostom	species	157319
750973	occ	79331	Teinostom	species	157377
750974	occ	79331	Teinostom	species	156822
750975	occ	79331	Teinostom	species	107015
750976	occ	79331	Teinostom	species	114944
750991	occ	79331	Cochliolepi	species	97698
750992	occ	79331	Cochliolepi	species	97697
750993	occ	79331	Cochliolepi	species	109665
579063	occ	60944	Teinostom	genus	10554
619810	occ	66758	Cochliolepi	species	156619
619811	occ	66760	Adeorbis h	species	109664
1457441	occ	79745	Adeorbis n	species	156618
1457489	occ	203377	Adeorbis n	species	156618
564615	occ	58893	Teinostom	species	10554
564634	occ	58894	Teinostom	species	82744
564691	occ	58895	Teinostom	species	10554
564947	occ	58897	Macromph	species	82746
564958	occ	58897	Teinostom	species	82744
564959	occ	58897	Teinostom	species	10554
564960	occ	58897	Teinostom	species	10554
567026	occ	59316	Teinostom	species	116529
567040	occ	59316	Macromph	species	82745
567041	occ	59307	Macromph	genus	82745
619812	occ	66751	Adeorbis h	species	109664
659694	occ	71138	Teinostom	species	10554
659727	occ	71138	Cochliolepi	genus	97697
559497	occ	57706	Teinostom	species	156720
427545	occ	41705	Triphora b	species	135784
567684	occ	59484	Triphora sp	genus	10609
567686	occ	59486	Triphora sp	genus	10609
640526	occ	69085	Triphora sp	genus	10609
749434	occ	79331	Triphora (C	species	135784
749435	occ	79331	Triphora (C	species	10609
1088996	occ	96538	Marshallor	species	108703
832910	occ	92408	Triforis sp.	genus	10608
579706	occ	60988	Triphora sp	genus	10609
727894	occ	78266	Triphora sp	genus	10609
426146	occ	41501	Erato mau	species	91240
426242	occ	41501	Trivia floric	species	135322
427482	occ	41705	Erato mau	species	91240
427546	occ	41705	Trivia pedi	species	167159
427773	occ	41718	Erato mau	species	91240
582400	occ	61309	Erato mau	species	91240
582401	occ	61309	Trivia pedi	species	167159

582402	occ	61309	Trivia quac	species	135320
582421	occ	61310	Trivia pedi	species	167159
582422	occ	61310	Trivia quac	species	135320
742004	occ	79747	Trivia (Nive	species	343151
742040	occ	79747	Pusula lind	species	342727
742041	occ	79747	Pusula dad	species	343063
742214	occ	79761	Pusula (Pu	species	93962
743225	occ	79804	Trivia incer	species	342695
743698	occ	79847	Trivia perm	species	156592
745686	occ	79331	Trivia islah	species	71691
745687	occ	79331	Trivia suffu	species	109041
745688	occ	79331	Trivia floric	species	135322
745689	occ	79331	Trivia (Triv	species	167159
745690	occ	79331	Erato mau	species	91240
745850	occ	79958	Decoriatriv	species	343042
1088982	occ	96538	Hesperera	species	91241
1088983	occ	96538	Niveria cf.	species	342093
1150734	occ	146873	Trivia sp.	genus	10611
1150735	occ	146873	Pusula sp.	genus	10388
832883	occ	92407	Cypraea pe	species	109039
832914	occ	92408	Trivia sp.	genus	10611
564962	occ	58897	Trivia floric	species	135322
567082	occ	59316	Trivia pedi	species	167159
426021	occ	41501	Calliostom	species	8628
426022	occ	41501	Calliostom	species	8628
426023	occ	41501	Calliostom	genus	8628
426094	occ	41539	Calliostom	species	187405
427464	occ	41705	Calliostom	species	8628
427465	occ	41705	Calliostom	genus	8628
568455	occ	59484	Calliostom	species	97673
568457	occ	59491	Tegula (On	species	384559
568458	occ	59484	Tegula (On	species	384559
582354	occ	61310	Calliostom	genus	8628
582366	occ	61311	Calliostom	genus	8628
582381	occ	61309	Calliostom	genus	8628
637080	occ	67069	Calliostom	species	187405
637081	occ	67069	Calliostom	species	8628
637083	occ	67069	Calliostom	species	8628
698074	occ	75023	Omphalius	species	9041
741923	occ	79740	Calliostom	species	8628
742216	occ	79765	Calliostom	species	116531
747304	occ	69044	Calliostom	genus	8628
747489	occ	79787	Calliostom	species	8628
747668	occ	80039	Calliostom	species	8628
747712	occ	80044	Calliostom	species	8628
748297	occ	80025	Calliostom	genus	8628
749684	occ	79331	Calliostom	species	8628

749685	occ	79331	Calliostom	species	8628
749686	occ	79331	Calliostom	species	8628
749687	occ	79331	Calliostom	species	8628
749698	occ	79338	Calliostom	species	8628
749699	occ	79338	Calliostom	species	8628
749700	occ	79338	Calliostom	species	8628
751013	occ	80358	Calliostom	genus	8628
1011544	occ	123549	Tegula (On	species	384559
1088929	occ	96538	Calliostom	species	187405
1088930	occ	96538	Calliostom	species	8628
832871	occ	92407	Infundibulu	species	157291
832875	occ	92407	Trochus ph	species	9336
564898	occ	58898	Calliostom	species	8628
564932	occ	58897	Calliostom	species	8628
578478	occ	60899	Calliostom	species	8628
748457	occ	80033	Calliostom	genus	8628
748540	occ	80033	Calliostom	species	97676
748590	occ	80035	Calliostom	species	97676
727750	occ	78270	Calliostom	species	97673
727751	occ	78270	Calliostom	genus	8628
727820	occ	78269	Calliostom	genus	8628
727883	occ	78266	Calliostom	genus	8628
727945	occ	78265	Calliostom	genus	8628
728011	occ	78261	Calliostom	genus	8628
737502	occ	79245	Calliostom	species	8628
748205	occ	80040	Calliostom	genus	8628
748411	occ	80036	Calliostom	species	97676
426254	occ	41501	Vasum hor	species	120369
426258	occ	41501	Xancus reg	species	120685
426259	occ	41501	Xancus sco	species	120686
428136	occ	41749	Xancus sco	species	120686
568806	occ	59484	Vasum ? sp	genus	11832
591051	occ	62462	Turbinella	species	82799
622902	occ	67070	Vasum flor	species	11832
741287	occ	79331	Vasum hor	species	120369
741288	occ	79331	Xancus reg	species	120685
741315	occ	79338	Vasum locl	species	120727
741897	occ	79739	Fulgurofus	species	11132
741901	occ	79739	Vasum mu	species	11832
741938	occ	79740	Fulgurofus	species	11132
741952	occ	79740	Turbinella	species	11806
741998	occ	79747	Vasum flor	species	11832
742556	occ	35031	Vasum flor	species	11832
742577	occ	79782	Turbinella	species	11806
742584	occ	79782	Hystrivasu	species	120725
743252	occ	79805	Turbinella	species	11806
743253	occ	79805	Hystrivasu	species	120730

743297	occ	79804	Turbinella	species	81571
743298	occ	79804	Turbinella	species	120684
743299	occ	79804	Hystrivasu	species	120730
743706	occ	79847	Hystrivasu	species	120730
743707	occ	79847	Hystrivasu	species	120725
744302	occ	79876	Hystrivasu	species	120725
744314	occ	79876	Hystrivasu	species	120725
744354	occ	79875	Hystrivasu	species	120725
744355	occ	79875	Hystrivasu	species	120726
744368	occ	79875	Hystrivasu	species	120725
744408	occ	79874	Hystrivasu	species	120725
744409	occ	79874	Hystrivasu	species	120725
744417	occ	79874	Hystrivasu	species	120725
745344	occ	79789	Vasum mu	species	11832
745563	occ	79960	Hystrivasu	species	120725
745568	occ	79960	Hystrivasu	species	120725
745585	occ	79960	Hystrivasu	species	120725
745586	occ	79960	Hystrivasu	species	120725
745733	occ	79959	Turbinella	species	11806
745734	occ	79959	Hystrivasu	species	120729
745797	occ	79959	Hystrivasu	species	120725
745798	occ	79959	Hystrivasu	species	120725
745799	occ	79959	Hystrivasu	species	120728
747523	occ	79786	Xancus reg	species	120685
747545	occ	79786	Vasum hor	species	120369
835679	occ	92338	Turbinella	species	120684
835680	occ	92369	Turbinella	species	11806
870209	occ	79960	Hystrivasu	species	120725
870210	occ	79960	Hystrivasu	genus	120725
870211	occ	79960	Hystrivasu	species	120725
870212	occ	79960	Hystrivasu	species	120729
870213	occ	79960	Hystrivasu	species	120725
870214	occ	79960	Hystrivasu	species	120728
1089008	occ	96538	Turbinella	species	82799
1089009	occ	96538	Vasum mu	species	11832
1150756	occ	146873	Hystrivasu	genus	120725
870216	occ	96536	Fulgurofus	species	11132
426151	occ	41501	Gelasinost	species	8827
426244	occ	41501	Turbo cast	species	135225
426245	occ	41501	Turbo rhec	species	9347
427547	occ	41705	Turbo cast	species	135225
428041	occ	41749	Astraea sc	species	8573
428126	occ	41749	Turbo rhec	species	9347
568452	occ	59486	Turbo cast	species	135225
568453	occ	59484	Turbo cast	species	135225
582355	occ	61310	Turbo cast	species	119763
582382	occ	61309	Turbo cast	species	119763

637086	occ	67069	Turbo cast	species	135225
637089	occ	67069	Astraea ph	species	149122
637090	occ	67069	Astraea an	species	109176
741921	occ	79740	Astraea (Li	species	119895
741922	occ	79740	Turbo (Ma	species	93981
741999	occ	79747	Astraea (Li	species	109200
742217	occ	79761	Turbo (Ma	species	155278
743208	occ	79804	Turbo (Tae	species	9347
743209	occ	79804	Astraea (Li	species	109200
743718	occ	79847	Turbo rhec	species	9347
745706	occ	79959	Turbo (Ma	species	93981
745843	occ	79958	Astraea (Li	species	109200
747342	occ	69029	Turbo sp.	genus	9347
748133	occ	69034	Turbo (Tae	subgenus	107067
749674	occ	79331	Turbo (Tae	species	107067
749675	occ	79331	Turbo (Tae	species	107067
749676	occ	79331	Turbo (Ma	species	93981
749677	occ	79331	Astraea (Li	species	109200
749678	occ	79331	Gelasinost	species	8827
749703	occ	79336	Turbo (Tae	species	107067
749704	occ	79336	Turbo (Ma	species	93981
749705	occ	79336	Astraea (A	species	115454
749706	occ	79336	Astraea (Li	species	109200
1088926	occ	96538	Astralium	species	149123
1088927	occ	96538	Turbo cast	species	119763
1150755	occ	146873	Astraea te	species	8573
1150766	occ	146874	Turbo ayer	species	187432
1150767	occ	146874	Turbo well	species	9347
1150768	occ	146874	Turbo cren	species	155282
1150769	occ	146874	Turbo (Tae	subgenus	9347
619873	occ	66760	"Angaria"	species	392479
619879	occ	66758	"Angaria"	species	392479
1457450	occ	150656	Angaria cra	species	392479
1457457	occ	66757	Angaria cra	species	392479
642743	occ	69045	Turbo cast	species	119763
425349	occ	41497	Polystira al	species	91341
426200	occ	41501	Polystira al	species	91341
426212	occ	41501	Sedilia ? ap	species	120711
426213	occ	41501	Sedilia cf. l	species	82943
426214	occ	41501	Sedilia sp.	species	82943
427533	occ	41705	Sedilia cf. l	species	82943
427534	occ	41705	Sedilia cf. c	species	82943
427535	occ	41705	Sedilia ? sp	genus	82943
428114	occ	41749	Sedilia sp.	genus	82943
567352	occ	59484	Brachycyth	species	161162
640753	occ	69087	Turridae in	family	62680
697947	occ	75011	Pleuroliria	genus	107810

747549	occ	79786	Turridae in	family	62680
747804	occ	80046	Turridae in	family	62680
750841	occ	79331	Polystira al	species	91341
750846	occ	79331	Fenimorea	species	120673
750847	occ	79331	Fenimorea	species	120673
750848	occ	79331	Fenimorea	species	120674
750850	occ	79331	Sedilia och	species	82943
750851	occ	79331	Sedilia sap	species	82943
750852	occ	79331	Sedilia trar	species	82943
750868	occ	79331	Glabrocyth	species	82945
750888	occ	41718	Sedilia ond	species	82943
870200	occ	79960	Sedilia sap	species	82943
1150795	occ	146875	Sedilia sp.	genus	82943
748117	occ	80083	Turridae in	family	62680
578480	occ	60899	Fenimorea	species	120673
579796	occ	60987	Turridae ?	family	62680
738397	occ	69043	Turridae in	family	62680
738429	occ	79342	Turridae in	family	62680
738511	occ	79339	Turridae in	family	62680
748539	occ	80033	Turridae in	family	62680
737505	occ	79245	Turridae in	family	62680
748196	occ	80040	Turridae in	family	62680
362443	occ	34269	Turritella s	genus	10637
362456	occ	34269	Vermicular	species	10661
423367	occ	41205	Turritella a	species	107007
425307	occ	41496	Turritella p	species	10637
425308	occ	41496	Turritella s	species	10637
425355	occ	41497	Turritella a	species	10637
425356	occ	41497	Turritella p	species	107007
425357	occ	41497	Turritella s	species	10637
425358	occ	41497	Vermicular	genus	10661
425379	occ	41498	Turritella a	species	10637
426110	occ	41539	Turritella a	species	10637
426111	occ	41539	Turritella p	species	107007
426247	occ	41501	Turritella a	species	10637
426248	occ	41501	Turritella p	species	107007
426249	occ	41501	Turritella p	species	10637
426250	occ	41501	Turritella s	species	10637
426251	occ	41501	Turritella v	species	10637
426255	occ	41501	Vermicular	species	10661
426256	occ	41501	Vermicular	genus	10661
427550	occ	41705	Turritella s	species	10637
427552	occ	41705	Vermicular	species	109778
427815	occ	41718	Turritella s	species	10637
427816	occ	41718	Vermicular	species	10661
428127	occ	41749	Turritella a	species	10637
428128	occ	41749	Turritella c	species	10637

428129	occ	41749	Turritella p	species	10637
428130	occ	41749	Turritella s	genus	10637
428131	occ	41749	Turritella s	species	10637
428132	occ	41749	Turritella c	species	10637
428134	occ	41749	Vermicular	species	10661
568136	occ	59484	Vermicular	species	109778
568137	occ	59491	Vermicular	species	109778
568141	occ	59487	Turritella a	species	10637
568142	occ	59484	Turritella e	species	10637
568143	occ	59485	Turritella e	species	10637
568144	occ	59489	Turritella e	species	10637
568145	occ	59490	Turritella e	species	10637
568146	occ	59488	Turritella e	species	10637
568147	occ	59484	Turritella c	species	10637
568148	occ	59486	Turritella d	species	95601
568149	occ	59491	Turritella d	species	95601
568150	occ	59484	Turritella d	species	95601
568151	occ	59487	Turritella s	species	10637
582387	occ	61309	Turritella s	genus	10637
582388	occ	61309	Turritella s	species	10637
582389	occ	61309	Vermicular	species	10661
582408	occ	61310	Turritella s	genus	10637
582409	occ	61310	Turritella s	species	10637
582410	occ	61310	Vermicular	species	10661
623054	occ	67072	Turritella c	species	10637
623055	occ	67072	Turritella b	species	89172
623072	occ	67074	Turritella b	species	89172
623081	occ	67075	Turritella a	species	144976
623087	occ	67076	Turritella b	species	89172
623088	occ	67076	Turritella a	species	144976
623097	occ	67077	Turritella b	species	89172
623098	occ	67077	Turritella a	species	144976
623099	occ	67077	Turritella c	species	10637
623110	occ	67078	Turritella b	species	89172
623111	occ	67078	Turritella a	species	144976
647843	occ	70010	Vermicular	species	109778
697878	occ	74999	Turritella s	species	10637
697897	occ	75009	Turritella c	species	97639
697906	occ	75001	Turritella s	species	10637
697945	occ	75011	Turritella s	genus	10637
697956	occ	75012	Turritella s	genus	10637
697957	occ	75012	Turritella v	species	97639
697994	occ	75015	Turritella c	species	10637
698002	occ	75016	Turritella s	genus	10637
698027	occ	75008	Turritella c	species	107007
698064	occ	75022	Turritella p	species	10637
698065	occ	75022	Turritella c	species	10637

698075	occ	75023	Turritella c	species	10637
698078	occ	75024	Turritella p	species	10637
698095	occ	75025	Turritella c	species	10637
698096	occ	75025	Turritella p	species	10637
698101	occ	75026	Turritella c	species	10637
698102	occ	75026	Turritella p	species	10637
742212	occ	79765	Turritella (I	species	93964
742215	occ	79761	Turritella (I	species	93964
742469	occ	79764	Turritella fl	species	10637
742473	occ	79762	Turritella g	species	10637
742499	occ	79761	Turritella g	species	10637
742500	occ	79761	Turritella p	species	10637
742510	occ	79335	Turritella p	species	107007
743212	occ	79804	Bactrospira	species	115371
743722	occ	79804	Vermicular	genus	10661
745424	occ	69040	Turritella a	species	10637
745431	occ	69040	Turritella s	genus	10637
745583	occ	79960	Eichwaldie	species	114779
745617	occ	79960	Torcula alu	species	10588
745708	occ	79959	Eichwaldie	species	114779
745709	occ	79959	Eichwaldie	species	114779
745710	occ	79959	Vermicular	species	10661
747305	occ	69044	Turritella (T	subgenus	94618
747306	occ	69044	Turritella s	genus	10637
747320	occ	69029	Turritella a	species	10637
747325	occ	69029	Turritella (I	species	9796
747336	occ	69029	Turritella (I	species	115371
747343	occ	69029	Vermicular	species	10661
747434	occ	79786	Turritella (T	species	94618
747546	occ	79786	Turritella (I	species	115371
747562	occ	80030	Torcula alu	species	10588
747584	occ	80029	Torcula alu	species	10588
747631	occ	80037	Turritella a	species	10637
747636	occ	80037	Vermicular	species	109778
747696	occ	80044	Torcula alu	species	10588
747771	occ	80046	Vermicular	species	109778
747785	occ	80046	Turritella s	genus	10637
748143	occ	69034	Turritella s	genus	10637
748285	occ	80025	Turritella (T	species	94618
748290	occ	80025	Vermicular	species	109778
748328	occ	80024	Turritella (I	species	115371
748334	occ	80024	Turritella (T	species	94618
748343	occ	80024	Vermicular	species	109778
749386	occ	79334	Vermicular	species	10661
749387	occ	79334	Vermicular	species	10661
749418	occ	79338	Vermicular	species	10661
749422	occ	79338	Vermicular	species	10661

749424	occ	79338	Turritella (⁻	species	94618
749425	occ	79338	Turritella (⁻	species	94618
749448	occ	79331	Vermicular	species	119771
749449	occ	79331	Turritella (I	species	10637
749450	occ	79331	Turritella (⁻	species	94618
749451	occ	79331	Turritella (⁻	species	94618
749463	occ	79333	Turritella (⁻	species	94618
749465	occ	79333	Turritella (I	species	115371
751004	occ	80359	Turritella a	species	10637
751005	occ	80359	Turritella s	species	10637
751006	occ	80359	Vermicular	genus	10661
751020	occ	80358	Turritella p	species	107007
751022	occ	80357	Turritella s	species	10637
853736	occ	94809	Turritella c	species	10637
853737	occ	94809	Turritella s	species	10637
1011564	occ	123549	Turritella a	species	10637
1088951	occ	96538	Torcula acı	species	96457
1088952	occ	96538	Turritella s	species	10637
1088953	occ	96538	Vermicular	species	109778
1150770	occ	146875	Turritella s	genus	10637
1150771	occ	146875	Turritella (I	species	9796
1150772	occ	146875	Turritella (I	species	93964
1150773	occ	146875	Turritella b	species	89172
1150774	occ	146875	Turritella n	species	96466
1317137	occ	176823	Turritella s	genus	10637
578619	occ	60927	Vermicular	species	109778
578791	occ	60930	Turritella s	species	10637
578951	occ	60940	Turritella e	species	10637
578952	occ	60940	Turritella h	species	10637
578953	occ	60940	Turritella b	species	10637
578954	occ	60940	Turritella p	species	10637
579004	occ	60939	Turritella p	species	10637
579068	occ	60944	Vermicular	species	10661
642895	occ	69046	Turritella s	genus	10637
748112	occ	80083	Turritellida	family	55363
832876	occ	92407	Turritella a	species	157289
832915	occ	92408	Vermicular	species	109778
832948	occ	92409	Turritella s	genus	10637
832991	occ	92412	Turritella v	species	97639
564617	occ	58893	Turritella p	species	10637
564636	occ	58894	Turritella p	species	10637
564637	occ	58894	Vermicular	species	109778
564696	occ	58895	Vermicular	species	109778
564698	occ	58895	Turritella p	species	10637
564699	occ	58895	Turritella b	species	82748
564890	occ	58898	Turritella b	species	82748
564891	occ	58898	Turritella p	species	10637

564964	occ	58897	Turritella b	species	82748
564965	occ	58897	Turritella p	species	10637
567042	occ	59316	Turritella ('	species	95602
567043	occ	59307	Turritella ('	species	95602
567049	occ	59316	Vermicular	species	109778
567050	occ	59307	Vermicular	species	109778
567051	occ	59310	Vermicular	species	109778
578482	occ	60899	Turritella s	species	10637
579640	occ	60989	Vermicular	species	187603
619881	occ	66752	Vermicular	species	109778
640564	occ	69086	Turritella p	species	10637
659708	occ	71138	Turritella s	genus	10637
702877	occ	68979	Turritella s	genus	10637
737401	occ	79248	Turritella d	species	95601
737414	occ	79249	Turritella d	species	95601
738421	occ	79342	Turritella d	species	95601
738474	occ	79341	Turritella d	species	95601
748357	occ	80033	Turritella p	species	10637
748358	occ	80033	Torcula sp.	genus	10588
748551	occ	80035	Turritella s	genus	10637
1173584	occ	151326	Turritella s	genus	10637
1512857	occ	211183	Vermicular	species	187603
566299	occ	59297	Turritella p	species	95597
566305	occ	59293	Turritella v	species	97639
582232	occ	61293	Turritella a	species	116540
582252	occ	61295	Turritella a	species	116540
664787	occ	71620	Turritella p	species	95597
664843	occ	71624	Turritella p	species	95597
727666	occ	78271	Turritella c	species	116540
727686	occ	78272	Turritella a	species	116540
727755	occ	78270	Turritella c	species	116540
727831	occ	78268	Turritella a	species	116540
727866	occ	78267	Turritella c	species	116540
727884	occ	78266	Turritella a	species	116540
727947	occ	78265	Turritella c	species	116540
727988	occ	78260	Turritella a	species	116540
728012	occ	78261	Turritella a	species	116540
728047	occ	78262	Turritella a	species	116540
728102	occ	78264	Turritella c	species	116540
737469	occ	79246	Turritella p	species	95597
737481	occ	79247	Turritella p	species	95597
737503	occ	79245	Turritella a	species	116540
741466	occ	79709	Turritella ('	species	116541
748191	occ	80040	Turritella p	species	95598
748192	occ	80040	Turritella ir	species	10637
748256	occ	80038	Turritella p	species	95598
748423	occ	80036	Turritella p	species	95598

915119	occ	103991	Vallonia co	species	114478
927257	occ	82258	Valvata sp.	genus	82075
362457	occ	34269	Petaloconc	species	109122
425378	occ	41498	Petaloconc	species	89539
426101	occ	41539	Lemintina :	genus	10042
426162	occ	41501	Lemintina (	species	156699
426197	occ	41501	Petaloconc	species	89539
428086	occ	41749	Lemintina :	genus	10042
428106	occ	41749	Petaloconc	species	89540
568135	occ	59484	Serpulorbi:	species	82750
568138	occ	59491	Petaloconc	species	89540
568139	occ	59487	Petaloconc	species	89540
568140	occ	59484	Petaloconc	species	89540
640750	occ	69087	Vermetida	family	62787
747294	occ	69044	Petaloconc	species	445956
747609	occ	80037	Petaloconc	species	89540
747705	occ	80044	Petaloconc	species	445956
747708	occ	80044	Petaloconc	species	89540
749417	occ	79338	Lemintina	species	10042
749419	occ	79338	Lemintina	species	10042
749446	occ	79331	Petaloconc	species	89539
749447	occ	79331	Lemintina (	species	156699
1011543	occ	123549	Petaloconc	species	89540
1150711	occ	146872	Petaloconc	species	89539
578955	occ	60940	Petaloconc	species	89540
578956	occ	60940	Serpulorbi:	species	10457
747837	occ	80053	Petaloconc	species	26115
748094	occ	80083	Petaloconc	species	445956
564633	occ	58894	Serpulorbi:	species	82750
564688	occ	58895	Serpulorbi:	species	10457
564956	occ	58897	Serpulorbi:	species	82750
567046	occ	59307	Lemintina ;	species	10042
567047	occ	59318	Lemintina ;	species	10042
567048	occ	59310	Lemintina ;	species	10042
737383	occ	79248	Serpulorbi:	species	82750
737441	occ	79249	Serpulorbi:	species	82750
748359	occ	80033	Petaloconc	species	26115
748478	occ	80033	Lemintina ;	species	10042
748546	occ	80035	Petaloconc	species	26115
727689	occ	78272	Serpulorbi:	species	82750
727724	occ	78273	Serpulorbi:	species	82750
727780	occ	78269	Serpulorbi:	species	10457
727834	occ	78268	Serpulorbi:	species	82750
727896	occ	78266	Serpulorbi:	species	82750
728024	occ	78261	Serpulorbi:	species	82750
728061	occ	78262	Serpulorbi:	species	10457
748282	occ	80038	Petaloconc	species	445956

748430	occ	80036	Petaloconc	species	26115
568459	occ	59484	Cyclostrem	species	248855
568460	occ	59484	Circulus gu	species	8665
582361	occ	61310	Cyclostrem	species	115224
582362	occ	61310	Episcynia n	species	91237
582364	occ	61310	Vitrinella s	genus	10670
582368	occ	61311	Episcynia n	species	91237
582383	occ	61309	Cyclostrem	species	115224
582384	occ	61309	Episcynia n	species	91237
582385	occ	61309	Vitrinella s	genus	10670
637106	occ	67069	Episcynia n	species	91237
637107	occ	67069	Cyclostrem	species	156724
637108	occ	67069	Cyclostrem	species	9715
637109	occ	67069	Cyclostrem	genus	9715
637120	occ	67069	Anticlimax	species	9483
637123	occ	67069	Parviturbo	species	117497
750962	occ	79338	Anticlimax	species	9483
750963	occ	79338	Anticlimax	species	9483
750964	occ	79338	Cyclostrem	species	248856
750965	occ	79338	Cyclostrem	species	9715
750966	occ	79338	Cyclostrem	species	114956
750968	occ	79338	Parviturbo	species	117497
750982	occ	79331	Anticlimax	species	9483
750983	occ	79331	Anticlimax	species	9483
750984	occ	79331	Anticlimax	species	9483
750986	occ	79331	Cyclostrem	species	9715
750987	occ	79331	Cyclostrem	species	248856
750988	occ	79331	Cyclostrem	species	114956
750989	occ	79331	Cyclostrem	species	9715
750990	occ	79331	Episcynia n	species	91237
750994	occ	79331	Parviturbo	species	117497
750996	occ	79333	Cyclostrem	species	114956
1088958	occ	96538	Anticlimax	species	9483
1088959	occ	96538	Cyclostrem	species	115224
1088961	occ	96538	Vitrinella fl	species	10670
1088962	occ	96538	Episcynia i	species	91239
619809	occ	66757	Vitrinella n	species	91236
564670	occ	58895	Cyclostrem	species	9715
564937	occ	58897	Cyclostrem	species	9715
567027	occ	59318	"Circulus"	species	8665
567028	occ	59307	"Circulus"	species	8665
579639	occ	60989	Cyclostrem	species	9715
559823	occ	57705	Circulus lir	species	90933
727843	occ	78269	Adeorbis s	genus	9430
727885	occ	78266	Adeorbis s	genus	9430
727949	occ	78265	Vitrinella s	genus	10670
728049	occ	78262	Cyclostrem	species	9715

289921	occ		19764	Viviparus g	species	68006
428135	occ		41749	Viviparus g	species	68006
582525	occ		61309	Viviparus g	species	68006
582541	occ		61310	Viviparus g	species	68006
582557	occ		61311	Viviparus g	species	68006
1088934	occ		96538	Viviparus g	species	68006
1607450	occ		227375	Viviparus g	species	68006
1607466	occ		227381	Viviparus g	species	68006
1607470	occ		227382	Viviparus g	species	68006
1607472	occ		227383	Viviparus g	species	68006
1607474	occ		227384	Viviparus g	species	68006
1607477	occ		227385	Viviparus g	species	68006
1607482	occ		227387	Viviparus g	species	68006
1607514	occ		227392	Viviparus g	species	68006
1607525	occ		227394	Viviparus g	species	68006
1607527	occ		227395	Viviparus g	species	68006
1607543	occ		227396	Viviparus g	species	68006
1607569	occ		227397	Viviparus g	species	68006
1607578	occ		227398	Viviparus g	species	68006
1607585	occ		227399	Viviparus g	species	68006
1607587	occ		227400	Viviparus g	species	68006
428113	occ		41749	Scaphella f	species	93822
567525	occ		59484	Marginella	species	93841
567526	occ		59490	Marginella	species	93841
567533	occ	18937	59487	Aurinia mu	species	93831
568803	occ		59486	Marginella	species	93841
741289	occ		79331	Scaphella f	species	93822
741969	occ		79749	Scaphella k	species	11654
741983	occ		79748	Volutifusus	species	82940
742026	occ		79747	Scaphella c	species	156792
742027	occ		79747	Scaphella s	species	11654
742198	occ		79765	Scaphella t	species	11654
742204	occ		79765	Aurinia (Vo	species	93834
742205	occ		79761	Aurinia (Vo	species	93834
742574	occ		79782	Scaphella g	species	11654
742575	occ		79782	Scaphella c	species	11654
742576	occ		79782	Scaphella t	species	11654
743300	occ		79804	Scaphella f	species	93822
744356	occ		79875	Scaphella g	species	11654
744357	occ		79875	Scaphella r	species	11654
745579	occ		79960	Scaphella r	species	11654
745639	occ		79960	Bullata ant	species	10870
745642	occ		79960	Volutifusus	species	82940
745766	occ		79959	Volutifusus	species	82940
745767	occ		79959	Volutifusus	species	82940
745768	occ		79959	Scaphella t	species	11654
747439	occ		79786	Scaphella f	species	93822

578980	occ	60940	Aurinia mu	species	93831
642893	occ	69046	Scaphella s	genus	11654
832973	occ	92411	Voluta mu	species	11845
564892	occ	58898	Volutifusus	species	82940
567205	occ	59307	Scaphella t	species	93821
567206	occ	59316	Scaphella p	species	86316
567207	occ	59316	Aurinia ob	species	93834
567208	occ	59318	Aurinia ob	species	93834
567209	occ	59307	Aurinia ob	species	93834
567213	occ	59307	Marginella	species	116680
748472	occ	80033	Scaphella s	genus	11654
727822	occ	78268	Scaphella c	species	93833
727823	occ	78268	Scaphella s	genus	11654
727932	occ	78266	Scaphella s	genus	11654
737498	occ	79245	Scaphella s	genus	11654
428137	occ	41749	Xenophora	species	81731
568438	occ	59486	Xenophora	species	187630
568439	occ	59484	Xenophora	species	187630
745700	occ	79331	Xenophora	species	81731
1088979	occ	96538	Xenophora	species	81731
579005	occ	60939	Xenophora	species	81731
1089093	occ	96538	Glyphyalin	species	251920
1089094	occ	96538	Hawaiia m	species	162067
1089095	occ	96538	Nesovitrea	species	251921
1089096	occ	96538	Zonitoides	species	251922
1607533	occ	227395	Glyphyalin	species	251920
1607565	occ	227396	Hawaiia m	species	162067
1607603	occ	227400	Glyphyalin	species	251920
1607604	occ	227400	Hawaiia m	species	162067
640757	occ	69087	Gastropod	class	8304
640766	occ	69087	Gastropod	class	8304
640768	occ	69087	Gastropod	class	8304
748312	occ	80025	Gastropod	class	8304
1317131	occ	176822	Gastropod	class	8304
642879	occ	69046	Gastropod	class	8304
748107	occ	80083	Gastropod	class	8304
738455	occ	79343	Gastropod	class	8304
738487	occ	79341	Gastropod	class	8304
737484	occ	79247	Gastropod	class	8304
915121	occ	103991	Gastropod	species	8304

sca&interval=Pliocene,Pleistocene&cc=US&state=Florida&pgm=gplates,scotese,seton&show=attr,class,classext,g

difference	accepted_name	accepted_attr	accepted_rank	accepted_no
recombined as	Raeta plicatella		species	108708
recombined as	Raeta plicatella		species	108708
recombined as	Raeta plicatella		species	108708
recombined as	Anatina anatina		species	91140
subjective synonym of	Raeta plicatella		species	108708
	Raeta plicatella		species	108708
	Raeta plicatella		species	108708
subjective synonym of	Anatina anatina		species	91140
recombined as	Raeta plicatella		species	108708
recombined as	Raeta plicatella		species	108708
	Raeta plicatella		species	108708
	Raeta plicatella		species	108708
	Raeta plicatella		species	108708
	Raeta plicatella		species	108708
	Raeta plicatella		species	108708
	Raeta plicatella		species	108708
recombined as	Raeta plicatella		species	108708
	Raeta plicatella		species	108708
	Raeta plicatella		species	108708
	Raeta		genus	18175
	Raeta plicatella		species	108708
	Raeta plicatella		species	108708
	Raeta plicatella		species	108708
	Anomia simplex		species	87561

	Anomia simplex	species	87561
	Anomia simplex	species	87561
	Anomia simplex	species	87561
	Anomia simplex	species	87561
	Anomia simplex	species	87561
	Anomia simplex	species	87561
	Anomia simplex	species	87561
	Anomia simplex	species	87561
	Anomia simplex	species	87561
	Anomia simplex	species	87561
	Anomia simplex	species	87561
	Anomia simplex	species	87561
	Anomia simplex	species	87561
	Placunanomia plicata	species	93765
	Placunanomia plicata	species	93765
	Placunanomia plicata	species	93765
	Anomia simplex	species	87561
	Anomia simplex	species	87561
	Anomia simplex	species	87561
	Anomia simplex	species	87561
	Anomia simplex	species	87561
	Anomia simplex	species	87561
	Anomia simplex	species	87561
	Anomia simplex	species	87561
	Anomia	genus	16476
	Anomia	genus	16476
	Anomia	genus	16476
	Anomia	genus	16476
	Placunanomia plicata	species	93765
	Placunanomia (Tamiai	species	89176
	Placunanomia (Tamiai	species	89176
recombined as	Placunanomia (Tamiai	species	89176
	Anomia simplex	species	87561
	Placunanomia plicata	species	93765
	Anomia	genus	16476
	Anomia	genus	16476
	Anomia simplex	species	87561
species not entered	Placunanomia	genus	16925
	Anomia simplex	species	87561
	Anomia	genus	16476
	Anomia	genus	16476
	Placunanomia plicata	species	93765
	Placunanomia plicata	species	93765
	Anomia	genus	16476
	Anomia simplex	species	87561
	Anomia simplex	species	87561
	Anomia simplex	species	87561

	Anomia simplex	species	87561
	Anomia simplex	species	87561
	Anomia simplex	species	87561
	Anomia simplex	species	87561
	Anomia simplex	species	87561
	Anomia simplex	species	87561
	Anomia simplex	species	87561
	Anomia simplex	species	87561
	Placunanomia plicata	species	93765
recombined as	Placunanomia (Tamiar	species	89176
	Anomia simplex	species	87561
	Anomia simplex	species	87561
	Anomia simplex	species	87561
	Anomia simplex	species	87561
	Anomia simplex	species	87561
	Anomia simplex	species	87561
	Anomia ephippium	species	90062
	Anomia ephippium	species	90062
	Anomia simplex	species	87561
	Anomia simplex	species	87561
recombined as	Pododesmus waccama	species	120100
	Anomia simplex	species	87561
	Anomia simplex	species	87561
	Anomia simplex	species	87561
	Anomia simplex	species	87561
	Anomia simplex	species	87561
	Anomia simplex	species	87561
	Anomia simplex	species	87561
	Anomia simplex	species	87561
	Anomia simplex	species	87561
	Anomia simplex	species	87561
	Anomia simplex	species	87561
	Anomia simplex	species	87561
	Anomia	genus	16476
	Anomia	genus	16476
	Anomia	genus	16476
	Anomia simplex	species	87561
recombined as	Anadara (Larkinia) tra	species	87548
species not entered	Anadara (Cunearca)	subgenus	16227
species not entered	Arca (Argina)	subgenus	71496
obsolete variant of	Arca limula	species	89695
subjective synonym of	Arca (Arca) imbricata	species	89693
	Acar domingensis	species	90949
obsolete variant of	Anadara	genus	16201
subjective synonym of	Byssoarca zebra	species	90944
recombined as	Anadara (Caloosarca)	species	109880
recombined as	Calloarca (Taeniarca) t	species	109876

	Arca wagneriana	species	109874
recombined as	Anadara (Anadara) ca	species	109479
species not entered	Anadara	genus	16201
species not entered	Anadara	genus	16201
recombined as	Anadara (Rasia) lienos	species	91378
recombined as	Anadara (Caloosarca)	species	109880
recombined as	Anadara (Larkinia) tra	species	87548
	Arca wagneriana	species	109874
recombined as	Calloarca (Taeniarca) t	species	109876
	Arca wagneriana	species	109874
recombined as	Anadara (Rasia) lienos	species	91378
species not entered	Anadara	genus	16201
species not entered	Anadara	genus	16201
recombined as	Anadara (Larkinia) tra	species	87548
recombined as	Anadara (Rasia) lienos	species	91378
recombined as	Anadara (Larkinia) tra	species	87548
recombined as	Anadara (Anadara) ca	species	109479
species not entered	Anadara	genus	16201
recombined as	Anadara (Rasia) lienos	species	91378
recombined as	Anadara (Caloosarca)	species	109880
recombined as	Cunearca scalarina	species	109476
species not entered	Anadara	genus	16201
	Arca wagneriana	species	109874
	Barbatia (Calloarca) ir	species	109878
species not entered	Anadara	genus	16201
recombined as	Anadara (Larkinia) tra	species	87548
species not entered	Anadara	genus	16201
recombined as	Anadara (Anadara) ca	species	109479
recombined as	Anadara (Larkinia) sell	species	120097
recombined as	Anadara (Rasia) lienos	species	91378
recombined as	Anadara (Caloosarca)	species	109880
recombined as	Cunearca scalarina	species	109476
obsolete variant of	Anadara	genus	16201
recombined as	Arca (Arca) aquila	species	93761
	Arca wagneriana	species	109874
species not entered	Anadara	genus	16201
subjective synonym of	Byssoarca zebra	species	90944
recombined as	Barbatia (Cucullaearca	species	90946
species not entered	Calloarca	genus	16214
recombined as	Barbatia (Barbatia) pr	species	95063
recombined as	Barbatia (Barbatia) pr	species	95063
recombined as	Barbatia (Barbatia) pr	species	95063
recombined as	Barbatia (Barbatia) pr	species	95063
recombined as	Dallarca idonea	species	98537
recombined as	Dallarca idonea	species	98537
recombined as	Dallarca idonea	species	98537
recombined as	Anadara (Larkinia) sell	species	120097

recombined as	Anadara callicestosa	species	95076
recombined as	Anadara callicestosa	species	95076
recombined as	Anadara (Rasia) lienos	species	91378
recombined as	Anadara (Rasia) lienos	species	91378
recombined as	Anadara (Rasia) lienos	species	91378
recombined as	Anadara (Rasia) lienos	species	91378
species not entered	Anadara	genus	16201
species not entered	Anadara	genus	16201
species not entered	Anadara	genus	16201
species not entered	Anadara	genus	16201
species not entered	Anadara	genus	16201
recombined as	Anadara campsa	species	157130
recombined as	Anadara campsa	species	157130
recombined as	Cunearca scalaris	species	95087
recombined as	Anadara (Rasia) lienos	species	91378
recombined as	Anadara (Larkinia) tra	species	87548
recombined as	Barbatia (Cucullaearca	species	90946
recombined as	Anadara (Rasia) lienos	species	91378
recombined as	Anadara (Larkinia) tra	species	87548
recombined as	Fugleria tenera	species	91192
recombined as	Anadara (Rasia) lienos	species	91378
recombined as	Anadara (Larkinia) tra	species	87548
species not entered	Anadara	genus	16201
recombined as	Fugleria tenera	species	91192
recombined as	Arca (Arca) imbricata	species	89693
	Arca zebra	species	90945
recombined as	Fugleria tenera	species	91192
recombined as	Barbatia (Cucullaearca	species	90946
recombined as	Acar domingensis	species	90949
recombined as	Anadara (Caloosarca)	species	157127
recombined as	Anadara (Larkinia) tra	species	87548
species not entered	Anadara	genus	16201
recombined as	Anadara (Rasia) lienos	species	91378
recombined as	Anadara (Rasia) lienos	species	91378
recombined as	Cunearca scalaris	species	95087
subjective synonym of, species not entered	Arca	genus	16203
recombined as	Cunearca scalaris	species	95087
recombined as	Cunearca scalaris	species	95087
recombined as	Anadara (Rasia) lienos	species	91378
recombined as	Cunearca scalaris	species	95087
	Anadara (Rasia) lienos	species	91378
	Anadara	genus	16201
	Cunearca scalaris	species	95087
species not entered	Anadara	genus	16201
recombined as	Anadara (Larkinia) tra	species	87548
recombined as	Anadara (Larkinia) tra	species	87548
obsolete variant of	Anadara (Cunearca)	subgenus	16227

species not entered	Anadara	genus	16201
species not entered	Anadara	genus	16201
obsolete variant of	Anadara (Cunearca)	subgenus	16227
species not entered	Anadara	genus	16201
species not entered	Anadara	genus	16201
	Anadara	genus	16201
	Cunearca scalaris	species	95087
recombined as	Anadara (Caloosarca)	species	109880
recombined as	Anadara (Caloosarca)	species	157127
subjective synonym of	Byssoarca zebra	species	90944
subjective synonym of	Arca (Arca) imbricata	species	89693
obsolete variant of	Arca wagneriana	species	109874
recombined as	Cunearca scalarina	species	109476
	Barbatia (Calloarca) ir	species	109878
recombined as	Anadara (Rasia) lienos	species	91378
recombined as	Anadara (Caloosarca)	species	109880
recombined as	Anadara (Caloosarca)	species	157127
species not entered	Anadara (Anadara)	subgenus	86518
	Anadara (Anadara) ca	species	109479
species not entered	Anadara (Anadara)	subgenus	86518
species not entered	Anadara (Cunearca)	subgenus	16227
obsolete variant of	Arca wagneriana	species	109874
recombined as	Calloarca (Taeniarca) t	species	109876
	Barbatia (Calloarca) ir	species	109878
recombined as	Cunearca scalarina	species	109476
	Anadara	genus	16201
species not entered	Anadara (Caloosarca)	subgenus	89988
	Anadara	genus	16201
	Arca (Arca) williamsor	species	93758
	Arca (Arca) aquila	species	93761
	Arca (Arca) aquila	species	93761
recombined as	Anadara (Larkinia) sell	species	120097
recombined as	Anadara (Caloosarca)	species	157118
	Arca wagneriana	species	109874
recombined as	Anadara tuberculosa	species	167867
	Anadara (Caloosarca)	species	157127
subjective synonym of, species not entered	Arca	genus	16203
subjective synonym of, species not entered	Arca	genus	16203
recombined as	Anadara (Rasia) lienos	species	91378
recombined as	Cunearca scalaris	species	95087
species not entered	Anadara	genus	16201
species not entered	Anadara	genus	16201
	Anadara trapezia	species	59092
obsolete variant of	Anadara (Caloosarca)	species	157123
recombined as	Anadara (Anadara) ca	species	109479
	Lunarca ovalis	species	87544
	Arca wagneriana	species	109874

species not entered	Anadara	genus	16201
species not entered	Anadara (Caloosarca)	subgenus	89988
recombined as	Anadara (Rasia) lienos	species	91378
	Arca wagneriana	species	109874
recombined as	Anadara (Rasia) lienos	species	91378
species not entered	Anadara	genus	16201
species not entered	Anadara	genus	16201
	Barbatia	genus	16210
recombined as	Anadara (Rasia) lienos	species	91378
species not entered	Dallarca	genus	16232
species not entered	Dallarca	genus	16232
	Anadara callicestosa	species	95076
recombined as	Anadara (Rasia) lienos	species	91378
	Lunarca ovalis	species	87544
recombined as	Anadara (Rasia) lienos	species	91378
recombined as	Anadara (Caloosarca)	species	157127
recombined as	Anadara (Larkinia) tra	species	87548
species not entered	Anadara (Cunearca)	subgenus	16227
recombined as	Anadara (Rasia) lienos	species	91378
	Anadara	genus	16201
	Arca wagneriana	species	109874
species not entered	Anadara	genus	16201
	Anadara (Caloosarca)	species	157123
	Anadara (Caloosarca)	species	157123
	Anadara (Caloosarca)	species	157123
	Anadara (Caloosarca)	species	157124
	Anadara (Caloosarca)	species	157118
	Anadara (Caloosarca)	species	157118
	Anadara (Caloosarca)	species	157118
	Anadara (Caloosarca)	species	157118
	Anadara (Caloosarca)	species	157118
	Anadara (Caloosarca)	species	157118
	Anadara (Caloosarca)	species	157118
	Anadara (Caloosarca)	species	157118
	Anadara (Caloosarca)	species	157118
	Anadara (Caloosarca)	species	157118
	Anadara (Caloosarca)	species	157118
	Anadara (Caloosarca)	species	157118
	Anadara (Caloosarca)	species	157118
	Anadara (Caloosarca)	species	157118
	Anadara (Caloosarca)	species	157118
	Anadara (Caloosarca)	species	157118
	Anadara (Caloosarca)	species	157127
	Anadara (Caloosarca)	species	157127
recombined as	Dallarca idonea	species	98537
species not entered	Anadara	genus	16201
recombined as	Anadara callicestosa	species	95076

recombined as	Dallarca idonea	species	98537
recombined as	Anadara (Rasia) lienos	species	91378
recombined as	Anadara (Larkinia) sell	species	120097
recombined as	Anadara (Sectiarca) flo	species	99335
recombined as	Anadara (Larkinia) tra	species	87548
recombined as	Anadara (Cunearca) b	species	87546
recombined as	Lunarca ovalis	species	87544
recombined as	Anadara (Larkinia) tra	species	87548
recombined as	Anadara (Cunearca) b	species	87546
recombined as	Lunarca ovalis	species	87544
recombined as	Anadara (Larkinia) tra	species	87548
recombined as	Anadara (Cunearca) b	species	87546
recombined as	Anadara (Cunearca) b	species	87546
recombined as	Anadara (Larkinia) tra	species	87548
recombined as	Anadara (Larkinia) tra	species	87548
recombined as	Lunarca ovalis	species	87544
recombined as	Anadara (Larkinia) tra	species	87548
recombined as	Anadara (Larkinia) tra	species	87548
recombined as	Anadara (Larkinia) tra	species	87548
recombined as	Anadara (Larkinia) tra	species	87548
subjective synonym of	Arca (Arca) imbricata	species	89693
recombined as	Anadara (Larkinia) tra	species	87548
recombined as	Anadara (Rasia) lienos	species	91378
species not entered	Anadara	genus	16201
recombined as	Anadara (Larkinia) tra	species	87548
recombined as	Anadara (Rasia) lienos	species	91378
recombined as	Anadara (Cunearca) b	species	87546
recombined as	Anadara (Cunearca) b	species	87546
recombined as	Anadara (Cunearca) b	species	87546
recombined as	Anadara (Cunearca) b	species	87546
recombined as	Lunarca ovalis	species	87544
recombined as	Lunarca ovalis	species	87544
recombined as	Lunarca ovalis	species	87544
recombined as	Lunarca ovalis	species	87544
recombined as	Lunarca ovalis	species	87544
	Anadara (Rasia) lienos	species	91378
	Anadara aequicostata	species	82687
	Lunarca ovalis	species	87544
	Lunarca ovalis	species	87544
	Lunarca ovalis	species	87544
	Lunarca ovalis	species	87544
	Lunarca ovalis	species	87544
	Lunarca ovalis	species	87544
	Lunarca ovalis	species	87544
recombined as	Anadara (Rasia) lienos	species	91378
	Anadara (Caloosarca)	species	109880
	Arca	genus	16203

subjective synonym of	Lunarca ovalis	species	87544
recombined as	Anadara (Rasia) lienos	species	91378
recombined as	Anadara (Larkinia) tra	species	87548
recombined as	Anadara (Rasia) lienos	species	91378
	Arca caelata	species	392332
	Anadara aequicostata	species	82687
	Anadara aequicostata	species	82687
	Anadara aequicostata	species	82687
	Anadara aequicostata	species	82687
	Anadara aequicostata	species	82687
recombined as	Anadara (Rasia) lienos	species	91378
recombined as	Anadara (Rasia) lienos	species	91378
recombined as	Anadara (Rasia) lienos	species	91378
recombined as	Anadara (Rasia) lienos	species	91378
recombined as	Anadara (Rasia) lienos	species	91378
recombined as	Anadara (Rasia) lienos	species	91378
recombined as	Barbatia carolinensis	species	59162
	Anadara callicestosa	species	95076
	Anadara magnoliana	species	95082
recombined as	Barbatia carolinensis	species	59162
recombined as	Cunearca scalaris	species	95087
recombined as	Cunearca scalaris	species	95087
recombined as	Anadara (Larkinia) tra	species	87548
	Anadara aequicostata	species	82687
	Anadara aequicostata	species	82687
	Anadara aequicostata	species	82687
	Anadara aequicostata	species	82687
recombined as	Anadara (Larkinia) tra	species	87548
recombined as	Anadara (Larkinia) tra	species	87548
recombined as	Anadara (Larkinia) tra	species	87548
recombined as	Anadara (Larkinia) tra	species	87548
recombined as	Anadara (Larkinia) tra	species	87548
recombined as	Anadara (Larkinia) tra	species	87548
recombined as	Anadara (Larkinia) tra	species	87548
recombined as	Anadara (Larkinia) tra	species	87548
recombined as	Anadara (Larkinia) tra	species	87548
recombined as	Anadara (Larkinia) tra	species	87548
recombined as	Anadara (Larkinia) tra	species	87548
recombined as	Anadara (Larkinia) tra	species	87548
recombined as	Anadara (Larkinia) tra	species	87548
recombined as	Anadara (Cunearca) b	species	87546
recombined as	Anadara (Cunearca) b	species	87546
recombined as	Anadara (Cunearca) b	species	87546
recombined as	Anadara (Cunearca) b	species	87546
recombined as	Lunarca ovalis	species	87544
recombined as	Lunarca ovalis	species	87544
recombined as	Lunarca ovalis	species	87544

recombined as	Lunarca ovalis	species	87544
recombined as	Lunarca ovalis	species	87544
recombined as	Lunarca ovalis	species	87544
recombined as	Lunarca ovalis	species	87544
recombined as	Anadara (Larkinia) tra	species	87548
	Lunarca ovalis	species	87544
	Anadara (Larkinia) tra	species	87548
	Lunarca ovalis	species	87544
obsolete variant of	Anadara (Lunarca)	subgenus	16264
obsolete variant of	Anadara (Lunarca)	subgenus	16264
	Lunarca ovalis	species	87544
	Lunarca ovalis	species	87544
	Lunarca ovalis	species	87544
	Lunarca ovalis	species	87544
	Anadara (Larkinia) tra	species	87548
	Anadara (Anadara)	subgenus	86518
	Anadara (Rasia)	subgenus	16305
	Anadara (Rasia) lienos	species	91378
	Anadara callicestosa	species	95076
	Anadara (Rasia)	subgenus	16305
	Anadara (Rasia) lienos	species	91378
	Anadara (Caloosarca)	species	109880
	Anadara (Caloosarca)	species	109880
	Anadara (Caloosarca)	species	109880
	Anadara aequicostata	species	82687
	Anadara aequicostata	species	82687
	Anadara aequicostata	species	82687
	Anadara aequicostata	species	82687
recombined as	Anadara (Larkinia) tra	species	87548
recombined as	Anadara (Larkinia) tra	species	87548
recombined as	Lunarca ovalis	species	87544
recombined as	Lunarca ovalis	species	87544
recombined as	Anadara (Larkinia) tra	species	87548
recombined as	Anadara (Larkinia) tra	species	87548
recombined as	Anadara (Larkinia) tra	species	87548
recombined as	Striarca centenaria	species	98542
recombined as	Dallarca idonea	species	98537
	Anadara (Larkinia) tra	species	87548
	Striarca centenaria	species	98542
	Striarca centenaria	species	98542
species not entered	Anadara (Sectiarca)	subgenus	16308
species not entered	Striarca	genus	16314
	Striarca centenaria	species	98542
	Striarca centenaria	species	98542
	Striarca centenaria	species	98542
	Anadara callicestosa	species	95076
species not entered	Striarca	genus	16314

	Striarca centenaria	species	98542
recombined as	Anadara (Larkinia) tra	species	87548
	Astarte	genus	17370
	Astarte (Ashtarotha) f	species	158542
	Astarte (Ashtarotha) f	species	158542
species not entered	Astarte (Ashtarotha)	subgenus	17369
	Astarte symmetrica	species	98475
	Astarte	genus	17370
	Taxocardia floridana	species	119003
obsolete variant of	Astarte perplana	species	98480
	Astarte concentrica	species	439681
recombined as	Astarte (Ashtarotha) f	species	158542
species not entered	Astarte	genus	17370
recombined as	Astarte (Ashtarotha) f	species	158542
recombined as	Astarte (Ashtarotha) f	species	158542
species not entered	Astarte (Ashtarotha)	subgenus	17369
	Astarte	genus	17370
	Astarte	genus	17370
species not entered	Astarte	genus	17370
	Astarte concentrica	species	439681
	Astarte concentrica	species	439681
	Astarte concentrica	species	439681
recombined as	Astarte (Ashtarotha) u	species	158538
	Astarte concentrica	species	439681
species not entered	Astarte	genus	17370
	Astarte concentrica	species	439681
	Astarte concentrica	species	439681
recombined as	Astarte (Ashtarotha) f	species	158542
	Astarte concentrica	species	439681
species not entered	Astarte	genus	17370
	Astarte concentrica	species	439681
species not entered	Astarte	genus	17370
	Astarte concentrica	species	439681
species not entered	Astarte	genus	17370
	Astarte concentrica	species	439681
species not entered	Astarte	genus	17370
	Astarte concentrica	species	439681
recombined as	Astarte (Ashtarotha) u	species	158538
	Astarte symmetrica	species	98475
species not entered	Astarte	genus	17370
	Astarte (Ashtarotha) u	species	158538
	Astarte (Ashtarotha) u	species	158538
	Astarte (Ashtarotha) u	species	158538
	Astarte (Ashtarotha) u	species	158538
	Astarte (Ashtarotha) u	species	158538
recombined as	Astarte concentrica	species	439681
recombined as	Astarte concentrica	species	439681

recombined as	Astarte concentrica	species	439681
recombined as	Astarte concentrica	species	439681
recombined as	Astarte concentrica	species	439681
recombined as	Astarte concentrica	species	439681
recombined as	Astarte concentrica	species	439681
recombined as	Astarte concentrica	species	439681
recombined as	Astarte concentrica	species	439681
	Astarte crenata	species	91396
	Astarte concentrica	species	439681
	Astarte concentrica	species	439681
	Astarte concentrica	species	439681
	Astarte concentrica	species	439681
	Astarte concentrica	species	439681
	Astarte concentrica	species	439681
	Astarte concentrica	species	439681
	Astarte concentrica	species	439681
	Astarte concentrica	species	439681
	Astarte concentrica	species	439681
	Astarte crenata	species	91396
	Astarte concentrica	species	439681
	Astarte concentrica	species	439681
	Astarte	genus	17370
	Astarte concentrica	species	439681
	Astarte	genus	17370
	Astarte castanea	species	91384
species not entered	Astarte	genus	17370
species not entered	Astarte	genus	17370
recombined as	Astarte (Ashtarotha) u	species	158538
	Astarte concentrica	species	439681
recombined as	Astarte (Ashtarotha) u	species	158538
species not entered	Astarte	genus	17370
species not entered	Astarte	genus	17370
species not entered	Astarte	genus	17370
species not entered	Astarte	genus	17370
recombined as	Astarte (Ashtarotha) u	species	158538
	Astarte concentrica	species	439681
	Astarte concentrica	species	439681
recombined as	Astarte (Ashtarotha) u	species	158538
species not entered	Astarte	genus	17370
	Astarte concentrica	species	439681
species not entered	Astarte	genus	17370
	Astarte concentrica	species	439681
species not entered	Astarte	genus	17370
	Astarte concentrica	species	439681
	Astarte concentrica	species	439681
	Astarte concentrica	species	439681
species not entered	Astarte	genus	17370

	Astarte concentrica	species	439681
	Astarte concentrica	species	439681
species not entered	Astarte	genus	17370
	Astarte	genus	17370
	Astarte	genus	17370
	Astarte	genus	17370
recombined as	Astarte (Ashtarotha) ι	species	158538
	Astarte concentrica	species	439681
	Astarte concentrica	species	439681
	Astarte	genus	17370
	Astarte concentrica	species	439681
obsolete variant of	Astarte concentrica	species	439681
	Astarte	genus	17370
	Avicula atlantica	species	392329
recombined as	Trachycardium murica	species	101131
recombined as	Dinocardium robustur	species	82716
recombined as	Trachycardium (Trach	species	94587
recombined as	Laevicardium serratur	species	102135
	Cardium (Laevicardiur	species	98405
	Cardium	genus	17448
species not entered	Trigoniocardia	genus	18358
	Trachycardium	genus	18347
	Dinocardium robustur	species	82716
replaced by	Laevicardium serratur	species	102135
recombined as	Cardium (Laevicardiur	species	98405
species not entered	Trachycardium	genus	18347
	Trachycardium murica	species	101131
	Trachycardium	genus	18347
species not entered	Trigoniocardia	genus	18358
	Dinocardium robustur	species	82716
recombined as	Cardium (Laevicardiur	species	98405
species not entered	Trachycardium	genus	18347
	Trachycardium murica	species	101131
	Trachycardium	genus	18347
species not entered	Trigoniocardia	genus	18358
replaced by	Laevicardium serratur	species	102135
species not entered	Trigoniocardia	genus	18358
	Dinocardium robustur	species	82716
recombined as	Cardium (Laevicardiur	species	98405
	Trachycardium	genus	18347
recombined as	Cardium (Laevicardiur	species	98405
species not entered	Cardium	genus	17448
replaced by	Laevicardium serratur	species	102135
	Trachycardium murica	species	101131
species not entered	Trachycardium	genus	18347
	Trachycardium (Phlog	species	160962
	Trachycardium (Phlog	species	160962

species not entered	Trachycardium	genus	18347
recombined as	Planicardium virginian	species	93779
species not entered	Trigoniocardia	genus	18358
species not entered	Trigoniocardia	genus	18358
recombined as	Laevicardium serratur	species	102135
species not entered	Protocardia	genus	61928
species not entered	Protocardia	genus	61928
	Dinocardium robustur	species	82716
replaced by	Laevicardium serratur	species	102135
	Trachycardium egmor	species	429680
	Dinocardium robustur	species	82716
replaced by	Laevicardium serratur	species	102135
	Trachycardium egmor	species	429680
	Trachycardium egmor	species	429680
	Dinocardium robustur	species	82716
species not entered	Trachycardium	genus	18347
species not entered	Trachycardium	genus	18347
species not entered	Trachycardium	genus	18347
	Trachycardium murica	species	101131
	Dinocardium robustur	species	82716
	Americardia media	species	90888
	Papyridea soleniformi	species	91167
	Papyridea semisulcata	species	91162
replaced by	Laevicardium serratur	species	102135
replaced by	Laevicardium serratur	species	102135
species not entered	Trachycardium	genus	18347
	Dinocardium robustur	species	82716
replaced by	Laevicardium serratur	species	102135
replaced by	Laevicardium serratur	species	102135
	Trachycardium murica	species	101131
	Cardium	genus	17448
	Cardium	genus	17448
	Cardium	genus	17448
recombined as	Trachycardium (Trach	species	94587
recombined as	Dinocardium robustur	species	82716
subjective synonym of	Americardia media	species	90888
recombined as	Dinocardium robustur	species	82716
recombined as	Trachycardium (Trach	species	94587
species not entered	Trachycardium	genus	18347
species not entered	Trigoniocardia	genus	18358
species not entered	Trigoniocardia (Trigon	subgenus	94688
recombined as	Dinocardium robustur	species	82716
recombined as	Papyridea semisulcata	species	91162
obsolete variant of	Cardium emmonsi	species	416607
recombined as	Trachycardium murica	species	101131
recombined as	Dinocardium robustur	species	82716
species not entered	Trigoniocardia	genus	18358

subjective synonym of	Americardia media	species	90888
recombined as	Papyridea semisulcata	species	91162
	Dinocardium robustur	species	82716
species not entered	Dinocardium	genus	17577
	Planicardium virginian	species	93779
species not entered	Trachycardium (Trach	subgenus	93777
species not entered	Trachycardium (Trach	subgenus	93777
species not entered	Trachycardium (Trach	subgenus	93777
species not entered	Acrosterigma	genus	17301
replaced by	Laevicardium serratur	species	102135
	Dinocardium robustur	species	82716
replaced by	Laevicardium serratur	species	102135
	Cardiidae	family	59532
	Trachycardium murica	species	101131
replaced by	Laevicardium serratur	species	102135
replaced by	Laevicardium serratur	species	102135
recombined as	Cardium (Laevicardiur	species	98405
recombined as	Cardium (Laevicardiur	species	98405
	Trachycardium	genus	18347
replaced by	Laevicardium serratur	species	102135
	Trachycardium (Trach	subgenus	93777
	Americardia media	species	90888
replaced by	Laevicardium serratur	species	102135
species not entered	Trachycardium	genus	18347
	Cardiidae	family	59532
	Dinocardium robustur	species	82716
replaced by	Laevicardium serratur	species	102135
	Chesacardium laqueat	species	99297
	Cardiidae	family	59532
	Americardia	genus	17324
replaced by	Laevicardium serratur	species	102135
	Trachycardium	genus	18347
	Trachycardium murica	species	101131
replaced by	Laevicardium serratur	species	102135
	Trachycardium	genus	18347
species not entered	Trachycardium	genus	18347
	Agnocardia spinosifro	species	157206
species not entered	Trachycardium	genus	18347
species not entered	Dallocardia	genus	17567
	Dinocardium robustur	species	82716
recombined as	Cardium (Laevicardiur	species	98405
	Laevicardium serratur	species	102135
	Trachycardium egmor	species	429680
recombined as	Cardium (Laevicardiur	species	98405
species not entered	Planicardium	genus	93778
	Dinocardium robustur	species	82716
	Dinocardium robustur	species	82716

	Trachycardium	genus	18347
	Dinocardium robustur	species	82716
	Dinocardium robustur	species	82716
	Dinocardium robustur	species	82716
	Trachycardium murica	species	101131
	Trachycardium murica	species	101131
	Trachycardium murica	species	101131
replaced by	Laevicardium serratur	species	102135
recombined as	Cardium (Laevicardiur	species	98405
replaced by	Laevicardium serratur	species	102135
recombined as	Trachycardium egmor	species	429680
	Dinocardium robustur	species	82716
species not entered	Cardium	genus	17448
	Cardium	genus	17448
recombined as	Trachycardium (Trach	species	94587
	Laevicardium serratur	species	102135
recombined as	Trachycardium (Trach	species	94587
species not entered	Laevicardium	genus	17785
species not entered	Laevicardium	genus	17785
misspelling of, species not entered	Cerastoderma	genus	17455
misspelling of, species not entered	Cerastoderma	genus	17455
misspelling of, species not entered	Cerastoderma	genus	17455
species not entered	Trachycardium	genus	18347
species not entered	Trachycardium	genus	18347
recombined as	Americardia media	species	90888
recombined as	Americardia media	species	90888
species not entered	Laevicardium	genus	17785
species not entered	Laevicardium	genus	17785
species not entered	Laevicardium	genus	17785
species not entered	Laevicardium	genus	17785
species not entered	Laevicardium	genus	17785
species not entered	Laevicardium	genus	17785
	Dinocardium robustur	species	82716
	Dinocardium robustur	species	82716
	Dinocardium robustur	species	82716
	Dinocardium robustur	species	82716
	Dinocardium robustur	species	82716
replaced by	Laevicardium serratur	species	102135
replaced by	Laevicardium serratur	species	102135
recombined as	Cardium (Laevicardiur	species	98405
recombined as	Cardium (Laevicardiur	species	98405
	Dinocardium robustur	species	82716
	Dinocardium robustur	species	82716
	Chesacardium acutila	species	99302
	Chesacardium acutila	species	99302
	Chesacardium acutila	species	99302
replaced by	Laevicardium serratur	species	102135

replaced by	Laevicardium serratur	species	102135
	Dinocardium robustur	species	82716
species not entered	Laevicardium	genus	17785
	Trachycardium (Trach	species	94587
	Dinocardium	genus	17577
species not entered	Laevicardium	genus	17785
	Trigoniocardia	genus	18358
	Acrosterigma	genus	17301
recombined as	Trachycardium murica	species	101131
species not entered	Laevicardium	genus	17785
	Trachycardium (Trach	species	94587
	Dinocardium robustur	species	82716
species not entered	Chesacardium	genus	17465
	Dinocardium robustur	species	82716
	Dinocardium robustur	species	82716
	Dinocardium robustur	species	82716
	Chesacardium acutila	species	99302
	Chesacardium acutila	species	99302
	Chesacardium acutila	species	99302
	Chesacardium acutila	species	99302
	Chesacardium acutila	species	99302
	Chesacardium acutila	species	99302
	Chesacardium acutila	species	99302
	Chesacardium acutila	species	99302
	Chesacardium	genus	17465
	Chesacardium	genus	17465
recombined as	Carditamera floridana	species	93558
recombined as	Pleuromeris tridentat	species	87631
recombined as	Pteromeris perplana	species	93589
obsolete variant of	Carditamera	genus	17442
obsolete variant of	Carditamera	genus	17442
recombined as	Carditamera arata	species	93550
recombined as	Carditamera floridana	species	93558
recombined as	Pteromeris perplana	species	93589
recombined as	Pleuromeris tridentat	species	87631
	Cardita	genus	17441
recombined as	Carditamera arata	species	93550
recombined as	Carditamera floridana	species	93558
recombined as	Pleuromeris tridentat	species	87631
recombined as	Carditamera arata	species	93550
recombined as	Carditamera arata	species	93550
recombined as	Carditamera floridana	species	93558
recombined as	Pleuromeris tridentat	species	87631
	Cardita	genus	17441
recombined as	Carditamera arata	species	93550
recombined as	Pleuromeris tridentat	species	87631
	Carditamera floridana	species	93558

	Cardita (Carditamera)	species	93556
	Cardita (Carditamera)	species	93556
recombined as	Carditamera arata	species	93550
recombined as	Carditamera arata	species	93550
recombined as	Carditamera arata	species	93550
recombined as	Carditamera arata	species	93550
recombined as	Cyclocardia granulata	species	82712
recombined as	Cyclocardia granulata	species	82712
recombined as	Cyclocardia granulata	species	82712
recombined as	Cyclocardia granulata	species	82712
recombined as	Pteromeris perplana	species	93589
recombined as	Pteromeris perplana	species	93589
recombined as	Pleuromeris tridentata	species	87631
recombined as	Pleuromeris tridentata	species	87631
recombined as	Pleuromeris tridentata	species	87631
	Venericardia (Pleurom	species	86149
	Venericardia (Pleurom	species	86149
	Carditamera floridana	species	93558
	Pleuromeris tridentata	species	87631
	Pteromeris perplana	species	93589
	Carditamera floridana	species	93558
	Pleuromeris tridentata	species	87631
	Pteromeris perplana	species	93589
	Pleuromeris tridentata	species	87631
	Carditamera floridana	species	93558
recombined as	Carditamera floridana	species	93558
recombined as	Pleuromeris tridentata	species	87631
	Carditamera floridana	species	93558
	Carditamera floridana	species	93558
	Carditamera floridana	species	93558
recombined as	Carditamera floridana	species	93558
obsolete variant of	Carditamera	genus	17442
obsolete variant of	Pleuromeris	genus	18092
recombined as	Carditamera arata	species	93550
recombined as	Pteromeris perplana	species	93589
obsolete variant of	Carditamera	genus	17442
obsolete variant of	Pleuromeris decemco	species	93773
obsolete variant of	Carditamera	genus	17442
obsolete variant of	Pleuromeris decemco	species	93773
obsolete variant of	Pleuromeris decemco	species	93773
obsolete variant of	Pleuromeris decemco	species	93773
obsolete variant of	Pleuromeris decemco	species	93773
obsolete variant of	Pleuromeris decemco	species	93773
obsolete variant of	Pleuromeris decemco	species	93773
obsolete variant of	Pleuromeris decemco	species	93773
recombined as	Pleuromeris tridentata	species	87631
recombined as	Pteromeris perplana	species	93589

recombined as	Carditamera arata	species	93550
recombined as	Carditamera catharia	species	93561
	Cardita (Cardita) semi	species	93767
	Cardita (Cardita) semi	species	93767
recombined as	Pleuromeris decemco	species	93773
	Carditamera (Carditan	species	93776
	Venericardia olga	species	89177
	Carditamera	genus	17442
	Carditamera arata	species	93550
	Carditamera arata	species	93550
	Pleuromeris tridentata	species	87631
	Carditamera floridana	species	93558
	Carditamera floridana	species	93558
	Venericardia olga	species	89177
obsolete variant of	Cardita (Cardita) semi	species	93767
obsolete variant of	Carditamera (Carditan	species	93776
species not entered	Pleuromeris	genus	18092
recombined as	Carditamera floridana	species	93558
recombined as	Carditamera floridana	species	93558
	Carditamera floridana	species	93558
	Carditamera arata	species	93550
	Pleuromeris tridentata	species	87631
	Carditamera floridana	species	93558
	Pleuromeris tridentata	species	87631
	Carditamera arata	species	93550
	Carditamera arata	species	93550
	Pleuromeris tridentata	species	87631
	Carditamera arata	species	93550
	Cyclocardia granulata	species	82712
	Cyclocardia granulata	species	82712
	Carditamera arata	species	93550
	Cyclocardia granulata	species	82712
	Carditamera floridana	species	93558
	Pleuromeris tridentata	species	87631
	Carditamera arata	species	93550
	Carditamera arata	species	93550
recombined as	Pleuromeris tridentata	species	87631
recombined as	Carditamera arata	species	93550
	Carditamera arata	species	93550
	Carditamera floridana	species	93558
	Pleuromeris tridentata	species	87631
	Pleuromeris tridentata	species	87631
	Pleuromeris tridentata	species	87631
recombined as	Cyclocardia granulata	species	82712
recombined as	Cyclocardia granulata	species	82712
recombined as	Cyclocardia granulata	species	82712
recombined as	Carditamera arata	species	93550

recombined as	Pteromeris perplana	species	93589
recombined as	Pleuromeris tridentat	species	87631
species not entered	Cardita	genus	17441
recombined as	Cyclocardia granulata	species	82712
recombined as	Pleuromeris tridentat	species	87631
recombined as	Cyclocardia granulata	species	82712
recombined as	Cyclocardia granulata	species	82712
	Cyclocardia granulata	species	82712
recombined as	Carditamera arata	species	93550
recombined as	Pleuromeris tridentat	species	87631
recombined as	Pleuromeris tridentat	species	87631
recombined as	Pleuromeris tridentat	species	87631
recombined as	Carditamera floridana	species	93558
	Pleuromeris tridentat	species	87631
	Pteromeris perplana	species	93589
	Cyclocardia granulata	species	82712
	Cyclocardia granulata	species	82712
	Carditamera arata	species	93550
obsolete variant of	Carditamera	genus	17442
recombined as	Pleuromeris tridentat	species	87631
recombined as	Pteromeris perplana	species	93589
recombined as	Cyclocardia granulata	species	82712
recombined as	Pteromeris perplana	species	93589
recombined as	Pleuromeris tridentat	species	87631
recombined as	Cyclocardia granulata	species	82712
recombined as	Carditamera arata	species	93550
recombined as	Cyclocardia granulata	species	82712
recombined as	Pleuromeris tridentat	species	87631
	Cyclocardia granulata	species	82712
	Cyclocardia granulata	species	82712
	Carditamera arata	species	93550
	Cyclocardia granulata	species	82712
	Carditamera arata	species	93550
	Carditamera arata	species	93550
	Cyclocardia granulata	species	82712
	Cyclocardia granulata	species	82712
	Pleuromeris auroraen	species	82711
	Pleuromeris decemco	species	93773
	Pteromeris perplana	species	93589
	Pleuromeris auroraen	species	82711
	Pleuromeris decemco	species	93773
	Pleuromeris auroraen	species	82711
	Pleuromeris decemco	species	93773
	Pteromeris perplana	species	93589
	Pleuromeris decemco	species	93773
	Pteromeris perplana	species	93589
	Pleuromeris auroraen	species	82711

	Pleuromeris decemco	species	93773
	Pteromeris perplana	species	93589
recombined as	Carditamera arata	species	93550
species not entered	Carditamera	genus	17442
recombined as	Pleuromeris tridentat	species	87631
recombined as	Pleuromeris tridentat	species	87631
recombined as	Pleuromeris tridentat	species	87631
recombined as	Pleuromeris tridentat	species	87631
recombined as	Pleuromeris tridentat	species	87631
recombined as	Pleuromeris tridentat	species	87631
recombined as	Pleuromeris tridentat	species	87631
recombined as	Pleuromeris tridentat	species	87631
recombined as	Pleuromeris tridentat	species	87631
recombined as	Pleuromeris tridentat	species	87631
recombined as	Pteromeris perplana	species	93589
recombined as	Pteromeris perplana	species	93589
recombined as	Pteromeris perplana	species	93589
recombined as	Pteromeris perplana	species	93589
recombined as	Pteromeris perplana	species	93589
recombined as	Pteromeris perplana	species	93589
recombined as	Pteromeris perplana	species	93589
recombined as	Pteromeris perplana	species	93589
recombined as	Pteromeris perplana	species	93589
species not entered	Pteromeris	genus	18162
recombined as	Pleuromeris tridentat	species	87631
recombined as	Pleuromeris tridentat	species	87631
recombined as	Pleuromeris tridentat	species	87631
recombined as	Pleuromeris tridentat	species	87631
recombined as	Pleuromeris tridentat	species	87631
recombined as	Carditamera floridana	species	93558
recombined as	Carditamera floridana	species	93558
	Cyclocardia granulata	species	82712
	Carditamera arata	species	93550
	Cyclocardia granulata	species	82712
	Cyclocardia granulata	species	82712
	Carditamera arata	species	93550
	Cyclocardia granulata	species	82712
	Pleuromeris tridentat	species	87631
	Pteromeris perplana	species	93589
	Cyclocardia	genus	17548
	Carditamera	genus	17442
	Pleuromeris tridentat	species	87631
	Carditamera arata	species	93550
	Cyclocardia granulata	species	82712
	Pteromeris perplana	species	93589
	Pleuromeris tridentat	species	87631
	Pteromeris perplana	species	93589

	Carditamera arata	species	93550
	Cyclocardia granulata	species	82712
	Pteromeris perplana	species	93589
	Pleuromeris tridentata	species	87631
	Pleuromeris tridentata	species	87631
	Cyclocardia granulata	species	82712
	Cyclocardia granulata	species	82712
	Pteromeris perplana	species	93589
	Pleuromeris tridentata	species	87631
	Cyclocardia granulata	species	82712
	Carditidae	family	59551
	Pleuromeris tridentata	species	87631
	Cyclocardia granulata	species	82712
	Cyclocardia granulata	species	82712
	Carditamera arata	species	93550
	Pleuromeris tridentata	species	87631
	Pteromeris perplana	species	93589
	Carditamera arata	species	93550
	Pteromeris perplana	species	93589
	Pleuromeris tridentata	species	87631
	Pleuromeris	genus	18092
	Carditamera	genus	17442
	Pteromeris perplana	species	93589
	Carditamera arata	species	93550
recombined as	Cyclocardia borealis	species	93493
	Pleuromeris tridentata	species	87631
	Cyclocardia borealis	species	93493
	Cyclocardia granulata	species	82712
recombined as	Cyclocardia granulata	species	82712
	Pleuromeris	genus	18092
	Pleuromeris tridentata	species	87631
	Cyclocardia granulata	species	82712
	Cyclocardia granulata	species	82712
	Cyclocardia granulata	species	82712
	Cyclocardia granulata	species	82712
	Cyclocardia granulata	species	82712
	Carditamera arata	species	93550
	Cyclocardia granulata	species	82712
	Cyclocardia granulata	species	82712
	Cyclocardia granulata	species	82712
	Cyclocardia granulata	species	82712
	Cyclocardia granulata	species	82712
	Carditamera arata	species	93550
	Carditamera arata	species	93550
	Cyclocardia granulata	species	82712
	Cyclocardia granulata	species	82712
	Cyclocardia granulata	species	82712

	Cyclocardia granulata	species	82712
	Cyclocardia granulata	species	82712
species not entered	Pteromeris	genus	18162
	Cyclocardia granulata	species	82712
	Cyclocardia granulata	species	82712
	Cyclocardia granulata	species	82712
	Cyclocardia granulata	species	82712
species not entered	Chama	genus	17459
species not entered	Chama	genus	17459
recombined as	Arcinella cornuta	species	90897
species not entered	Chama	genus	17459
recombined as	Arcinella cornuta	species	90897
species not entered	Chama	genus	17459
species not entered	Chama	genus	17459
species not entered	Chama	genus	17459
recombined as	Arcinella cornuta	species	90897
species not entered	Chama	genus	17459
species not entered	Chama	genus	17459
species not entered	Chama	genus	17459
recombined as	Arcinella cornuta	species	90897
species not entered	Chama	genus	17459
species not entered	Chama	genus	17459
species not entered	Chama	genus	17459
recombined as	Arcinella cornuta	species	90897
	Chama congregata	species	90994
	Chama congregata	species	90994
	Chama congregata	species	90994
recombined as	Arcinella arcinella	species	91233
recombined as	Arcinella arcinella	species	91233
misspelling of, obsolete variant of	Arcinella cornuta	species	90897
	Chama congregata	species	90994
	Chama macerophylla	species	90983
	Arcinella cornuta	species	90897
	Chama congregata	species	90994
	Arcinella cornuta	species	90897
	Chama macerophylla	species	90983
	Chama congregata	species	90994
recombined as	Chama radians	species	90998
	Arcinella cornuta	species	90897
species not entered	Chama	genus	17459
species not entered	Chama	genus	17459
recombined as	Arcinella arcinella	species	91233
	Arcinella cornuta	species	90897
	Chama congregata	species	90994
recombined as	Arcinella arcinella	species	91233
recombined as	Arcinella arcinella	species	91233
	Chama congregata	species	90994

species not entered	Chama	genus	17459
	Chama	genus	17459
recombined as	Arcinella arcinella	species	91233
	Chama congregata	species	90994
	Arcinella cornuta	species	90897
	Chama congregata	species	90994
species not entered	Chama	genus	17459
	Arcinella cornuta	species	90897
recombined as	Arcinella arcinella	species	91233
	Chama congregata	species	90994
	Arcinella cornuta	species	90897
	Chama congregata	species	90994
species not entered	Pseudochama	genus	18144
species not entered	Pseudochama	genus	18144
species not entered	Chama	genus	17459
species not entered	Chama	genus	17459
species not entered	Pseudochama	genus	18144
	Arcinella cornuta	species	90897
	Arcinella cornuta	species	90897
species not entered	Chama	genus	17459
recombined as	Arcinella arcinella	species	91233
	Pseudochama	genus	18144
	Chama radians	species	90998
species not entered	Pseudochama	genus	18144
	Chama congregata	species	90994
species not entered	Chama	genus	17459
recombined as	Arcinella arcinella	species	91233
recombined as	Arcinella arcinella	species	91233
species not entered	Chama	genus	17459
	Chama congregata	species	90994
species not entered	Pseudochama	genus	18144
species not entered	Pseudochama	genus	18144
species not entered	Pseudochama	genus	18144
species not entered	Chama	genus	17459
	Chama macerophylla	species	90983
	Chama	genus	17459
species not entered	Pseudochama	genus	18144
species not entered	Pseudochama	genus	18144
	Arcinella cornuta	species	90897
species not entered	Pseudochama	genus	18144
	Arcinella cornuta	species	90897
	Arcinella cornuta	species	90897
species not entered	Pseudochama	genus	18144
	Chama congregata	species	90994
	Chama congregata	species	90994
	Chama congregata	species	90994
	Chama congregata	species	90994

species not entered	Pseudochama	genus	18144
	Chama congregata	species	90994
	Chama congregata	species	90994
	Chama congregata	species	90994
	Chama congregata	species	90994
species not entered	Pseudochama	genus	18144
species not entered	Pseudochama	genus	18144
species not entered	Pseudochama	genus	18144
species not entered	Pseudochama	genus	18144
	Erycinella ovalis	species	142142
	Erycinella ovalis	species	142142
species not entered	Corbicula	genus	17504
recombined as	Polymesoda caroliniar	species	156515
species not entered	Corbicula	genus	17504
species not entered	Corbicula	genus	17504
species not entered	Corbicula	genus	17504
species not entered	Corbicula	genus	17504
species not entered	Corbicula	genus	17504
recombined as	Polymesoda caroliniar	species	156515
recombined as	Caryocorbula cuneata	species	158601
species not entered	Caryocorbula	genus	18437
species not entered	Corbula (Varicorbula)	subgenus	18517
species not entered	Corbula (Varicorbula)	subgenus	18517
	Bothrocorbula wilcoxi	species	121451
species not entered	Caryocorbula	genus	18437
species not entered	Corbula (Varicorbula)	subgenus	18517
species not entered	Corbula (Varicorbula)	subgenus	18517
subjective synonym of	Caryocorbula caribaea	species	101163
species not entered	Caryocorbula	genus	18437
	Juliacorbula scutata	species	121471
species not entered	Corbula (Varicorbula)	subgenus	18517
	Bothrocorbula wilcoxi	species	121451
species not entered	Caryocorbula	genus	18437
species not entered	Corbula (Varicorbula)	subgenus	18517
	Bothrocorbula wilcoxi	species	121451
species not entered	Corbula (Varicorbula)	subgenus	18517
subjective synonym of	Varicorbula disparilis	species	162183
subjective synonym of	Varicorbula disparilis	species	162183
subjective synonym of	Varicorbula disparilis	species	162183
species not entered	Corbula (Caryocorbula	subgenus	82729
species not entered	Corbula (Caryocorbula	subgenus	82729
species not entered	Corbula (Caryocorbula	subgenus	82729
species not entered	Corbula (Caryocorbula	subgenus	82729
obsolete variant of	Corbula inaequalis	species	97922
subjective synonym of	Caryocorbula caribaea	species	101163
subjective synonym of	Caryocorbula caribaea	species	101163
recombined as	Caryocorbula cuneata	species	158601

recombined as	Caryocorbula contract	species	82732
recombined as	Caryocorbula contract	species	82732
recombined as	Caryocorbula contract	species	82732
	Juliacorbula scutata	species	121471
recombined as	Juliacorbula scutata	species	121471
recombined as	Juliacorbula scutata	species	121471
recombined as	Caryocorbula caribaea	species	101163
subjective synonym of	Caryocorbula caribaea	species	101163
recombined as	Varicorbula krebsiana	species	146240
	Corbula	genus	18441
species not entered	Corbula (Notocorbula)	subgenus	18477
	Caryocorbula contract	species	82732
	Caryocorbula contract	species	82732
	Corbula	genus	18441
	Corbula	genus	18441
species not entered	Caryocorbula (Caryoc	subgenus	101162
	Bothrocorbula wilcoxi	species	121451
species not entered	Corbula (Varicorbula)	subgenus	18517
	Juliacorbula scutata	species	121471
species not entered	Caryocorbula	genus	18437
species not entered	Caryocorbula	genus	18437
	Bothrocorbula wilcoxi	species	121451
	Bothrocorbula wilcoxi	species	121451
species not entered	Caryocorbula	genus	18437
obsolete variant of	Corbula (Notocorbula)	subgenus	18477
	Corbula	genus	18441
recombined as	Corbula inaequalis	species	97922
	Caryocorbula contract	species	82732
	Corbula	genus	18441
recombined as	Corbula inaequalis	species	97922
recombined as	Varicorbula chipolana	species	121458
	Bothrocorbula	genus	18433
recombined as	Corbula inaequalis	species	97922
recombined as	Corbula inaequalis	species	97922
recombined as	Varicorbula chipolana	species	121458
	Caryocorbula cuneata	species	158601
recombined as	Corbula inaequalis	species	97922
	Bothrocorbula	genus	18433
	Bothrocorbula	genus	18433
	Caryocorbula	genus	18437
	Caryocorbula contract	species	82732
species not entered	Caryocorbula	genus	18437
species not entered	Corbula (Varicorbula)	subgenus	18517
species not entered	Caryocorbula	genus	18437
subjective synonym of	Caryocorbula caribaea	species	101163
species not entered	Corbula (Caryocorbula	subgenus	82729
subjective synonym of	Varicorbula disparilis	species	162183

recombined as	Bothrocorbula wilcoxi	species	121451
recombined as	Bothrocorbula wilcoxi	species	121451
	Caryocorbula caribaea	species	101163
	Caryocorbula contract	species	82732
	Caryocorbula contract	species	82732
subjective synonym of	Caryocorbula caribaea	species	101163
subjective synonym of	Caryocorbula caribaea	species	101163
	Caryocorbula contract	species	82732
	Caryocorbula contract	species	82732
	Caryocorbula contract	species	82732
	Caryocorbula contract	species	82732
	Caryocorbula	genus	18437
	Corbula inaequalis	species	97922
	Corbula inaequalis	species	97922
	Corbula inaequalis	species	97922
recombined as	Varicorbula chipolana	species	121458
recombined as	Caryocorbula contract	species	82732
species not entered	Caryocorbula	genus	18437
species not entered	Caryocorbula	genus	18437
species not entered	Caryocorbula	genus	18437
	Caryocorbula contract	species	82732
species not entered	Caryocorbula	genus	18437
species not entered	Caryocorbula	genus	18437
	Caryocorbula contract	species	82732
recombined as	Caryocorbula conradi	species	82730
recombined as	Caryocorbula conradi	species	82730
recombined as	Caryocorbula conradi	species	82730
recombined as	Caryocorbula conradi	species	82730
recombined as	Juliacorbula scutata	species	121471
subjective synonym of	Caryocorbula caribaea	species	101163
recombined as	Caryocorbula conradi	species	82730
recombined as	Caryocorbula conradi	species	82730
recombined as	Caryocorbula conradi	species	82730
recombined as	Caryocorbula contract	species	82732
recombined as	Caryocorbula contract	species	82732
recombined as	Caryocorbula contract	species	82732
	Caryocorbula	genus	18437
	Caryocorbula contract	species	82732
	Caryocorbula contract	species	82732
	Caryocorbula contract	species	82732
	Caryocorbula conradi	species	82730
	Caryocorbula conradi	species	82730
	Caryocorbula conradi	species	82730
	Caryocorbula conradi	species	82730
	Caryocorbula conradi	species	82730
	Caryocorbula contract	species	82732
	Caryocorbula contract	species	82732

	Caryocorbula	genus	18437
	Caryocorbula conradi	species	82730
	Caryocorbula conradi	species	82730
	Caryocorbula conradi	species	82730
recombined as	Caryocorbula contract	species	82732
recombined as	Caryocorbula contract	species	82732
recombined as	Caryocorbula contract	species	82732
recombined as	Caryocorbula contract	species	82732
recombined as	Caryocorbula contract	species	82732
recombined as	Caryocorbula contract	species	82732
subjective synonym of	Caryocorbula caribaea	species	101163
	Caryocorbula contract	species	82732
	Corbula inaequalis	species	97922
	Corbula inaequalis	species	97922
	Corbula inaequalis	species	97922
recombined as	Corbula inaequalis	species	97922
	Corbula inaequalis	species	97922
	Corbula inaequalis	species	97922
	Caryocorbula conradi	species	82730
	Caryocorbula conradi	species	82730
	Caryocorbula conradi	species	82730
	Crassinella lunulata	species	108663
	Crassinella	genus	17523
species not entered	Crassinella	genus	17523
species not entered	Crassinella	genus	17523
	Crassinella lunulata	species	108663
species not entered	Crassinella	genus	17523
species not entered	Crassinella	genus	17523
	Crassinella lunulata	species	108663
	Crassatellites (Crassat	species	99289
	Crassatellites (Crassat	species	99289
	Crassatellites (Crassat	species	99289
subjective synonym of	Eucrassatella (Eucrass	species	109775
subjective synonym of	Eucrassatella (Eucrass	species	109775
subjective synonym of	Eucrassatella (Eucrass	species	109775
	Crassinella lunulata	species	108663
	Crassinella lunulata	species	108663
	Crassinella lunulata	species	108663
species not entered	Crassinella	genus	17523
species not entered	Crassinella	genus	17523
species not entered	Crassinella	genus	17523
species not entered	Crassinella	genus	17523
	Crassinella lunulata	species	108663
	Crassinella lunulata	species	108663
	Crassinella lunulata	species	108663
	Crassinella lunulata	species	108663
	Crassinella	genus	17523

	Eucrassatella	genus	17640
	Crassinella	genus	17523
subjective synonym of	Eucrassatella (Eucrass	species	109775
subjective synonym of	Eucrassatella (Eucrass	species	109775
	Crassinella lunulata	species	108663
species not entered	Crassinella	genus	17523
subjective synonym of	Eucrassatella (Eucrass	species	109775
	Crassinella lunulata	species	108663
species not entered	Marvacrassatella	genus	17876
species not entered	Marvacrassatella	genus	17876
species not entered	Marvacrassatella	genus	17876
species not entered	Crassinella	genus	17523
species not entered	Marvacrassatella	genus	17876
	Crassinella lunulata	species	108663
species not entered	Scambula	genus	18221
species not entered	Crassinella	genus	17523
	Crassinella lunulata	species	108663
	Crassinella lunulata	species	108663
	Crassinella lunulata	species	108663
	Crassinella lunulata	species	108663
	Crassinella lunulata	species	108663
species not entered	Crassinella	genus	17523
	Crassinella lunulata	species	108663
species not entered	Eucrassatella	genus	17640
species not entered	Eucrassatella	genus	17640
species not entered	Crassinella	genus	17523
	Crassinella lunulata	species	108663
	Crassinella lunulata	species	108663
	Crassinella lunulata	species	108663
	Crassinella lunulata	species	108663
	Crassinella	genus	17523
	Crassinella lunulata	species	108663
	Marvacrassatella	genus	17876
	Crassinella lunulata	species	108663
	Crassinella lunulata	species	108663
	Marvacrassatella	genus	17876
	Crassinella lunulata	species	108663
	Crassinella lunulata	species	108663
	Crassinella lunulata	species	108663
subjective synonym of	Eucrassatella (Eucrass	species	109775
species not entered	Crassinella	genus	17523
	Crassinella lunulata	species	108663
species not entered	Crassinella	genus	17523
	Crassinella lunulata	species	108663
species not entered	Crassinella	genus	17523
	Crassinella johnsoni	species	82715
	Crassinella lunulata	species	108663

	Crassinella lunulata	species	108663
species not entered	Crassinella	genus	17523
	Crassinella johnsoni	species	82715
	Crassinella lunulata	species	108663
	Marvacrassatella kauf	species	82714
	Marvacrassatella kauf	species	82714
species not entered	Marvacrassatella	genus	17876
	Crassinella lunulata	species	108663
	Crassinella lunulata	species	108663
	Crassinella lunulata	species	108663
	Crassinella lunulata	species	108663
	Crassinella lunulata	species	108663
	Crassinella lunulata	species	108663
	Crassinella lunulata	species	108663
	Crassinella lunulata	species	108663
	Crassinella lunulata	species	108663
	Crassinella lunulata	species	108663
	Crassinella lunulata	species	108663
	Crassinella lunulata	species	108663
species not entered	Crassinella	genus	17523
species not entered	Crassinella	genus	17523
species not entered	Crassinella	genus	17523
species not entered	Crassinella	genus	17523
	Crassinella lunulata	species	108663
	Crassinella lunulata	species	108663
	Crassinella lunulata	species	108663
	Crassinella lunulata	species	108663
	Crassinella lunulata	species	108663
	Marvacrassatella	genus	17876
	Marvacrassatella	genus	17876
	Crassinella lunulata	species	108663
	Eucrassatella	genus	17640
	Marvacrassatella	genus	17876
	Crassatella	genus	17518
	Crassatella	genus	17518
	Crassinella lunulata	species	108663
	Crassinella lunulata	species	108663
	Crassinella lunulata	species	108663
	Crassinella lunulata	species	108663
species not entered	Marvacrassatella	genus	17876
	Crassinella lunulata	species	108663
	Eucrassatella	genus	17640
species not entered	Marvacrassatella	genus	17876
species not entered	Marvacrassatella	genus	17876
species not entered	Marvacrassatella	genus	17876
species not entered	Marvacrassatella	genus	17876
	Crassinella lunulata	species	108663

species not entered	Marvacrassatella	genus	17876
species not entered	Marvacrassatella	genus	17876
species not entered	Marvacrassatella	genus	17876
	Crassinella lunulata	species	108663
species not entered	Marvacrassatella	genus	17876
	Crassinella lunulata	species	108663
	Crassinella lunulata	species	108663
species not entered	Marvacrassatella	genus	17876
species not entered	Marvacrassatella	genus	17876
species not entered	Marvacrassatella	genus	17876
species not entered	Marvacrassatella	genus	17876
	Marvacrassatella	genus	17876
	Marvacrassatella	genus	17876
	Marvacrassatella	genus	17876
	Marvacrassatella	genus	17876
species not entered	Musculus	genus	16383
species not entered	Gregariella	genus	16359
species not entered	Musculus	genus	16383
species not entered	Musculus	genus	16383
species not entered	Musculus	genus	16383
species not entered	Musculus	genus	16383
recombined as	Cardiomya ornatissim	species	134769
recombined as	Plectodon granulatus	species	108200
	Cardiomya costellata	species	134771
subjective synonym of	Cardiomya costellata	species	134771
	Cardiomya ornatissim	species	134769
species not entered	Cardiomya	genus	18711
recombined as	Cardiomya mansfieldi	species	162234
species not entered	Plectodon	genus	18800
species not entered	Cyrenoida	genus	382142
recombined as	Serrula variabilis	species	109660
species not entered	Donax	genus	17592
	Donax fossor	species	108682
	Donax fossor	species	108682
recombined as	Serrula variabilis	species	109660
recombined as	Serrula variabilis	species	109660
recombined as	Serrula variabilis	species	109660
recombined as	Serrula variabilis	species	109660
recombined as	Serrula variabilis	species	109660
recombined as	Serrula variabilis	species	109660
	Donax	genus	17592
recombined as	Serrula variabilis	species	109660
recombined as	Serrula variabilis	species	109660
	Donax fossor	species	108682
	Donax fossor	species	108682
	Donax fossor	species	108682
	Donax fossor	species	108682

recombined as	Serrula variabilis	species	109660
recombined as	Serrula variabilis	species	109660
recombined as	Serrula variabilis	species	109660
recombined as	Serrula variabilis	species	109660
	Donax fossor	species	108682
	Serrula variabilis	species	109660
	Donax fossor	species	108682
	Donax fossor	species	108682
	Donax fossor	species	108682
species not entered	Donax	genus	17592
species not entered	Donax	genus	17592
species not entered	Donax	genus	17592
species not entered	Donax	genus	17592
species not entered	Donax	genus	17592
species not entered	Donax	genus	17592
species not entered	Donax	genus	17592
species not entered	Donax	genus	17592
species not entered	Donax	genus	17592
	Donax fossor	species	108682
	Donax fossor	species	108682
	Donax fossor	species	108682
	Donax fossor	species	108682
	Donax fossor	species	108682
	Donax fossor	species	108682
recombined as	Serrula variabilis	species	109660
recombined as	Serrula variabilis	species	109660
recombined as	Serrula variabilis	species	109660
recombined as	Serrula variabilis	species	109660
	Donax fossor	species	108682
	Donax fossor	species	108682
	Donax	genus	17592
species not entered	Donax	genus	17592
subjective synonym of	Serrula variabilis	species	109660
recombined as	Donax triangulata	species	160677
	Mytilopsis lamellata	species	110031
	Mytilopsis	genus	71502
	Mytilopsis leucophaea	species	155518
species not entered	Congeria	genus	97797
	Erycina	genus	17634
species not entered	Erycina	genus	17634
species not entered	Erycina	genus	17634
species not entered	Erycina	genus	17634
species not entered	Erycina	genus	17634
species not entered	Erycina	genus	17634
species not entered	Erycina	genus	17634
species not entered	Erycina	genus	17634
	Gastrochaena	genus	18456

species not entered	Gastrochaena (Rocella	subgenus	116480
species not entered	Gastrochaena	genus	18456
	Gastrochaena hians	species	156309
	Gastrochaena hians	species	156309
	Spengleria rostrata	species	416609
	Gastrochaena cuneifo	species	158754
	Gastrochaena hians	species	156309
species not entered	Lamychaena	genus	251737
species not entered	Gastrochaena	genus	18456
	Gastrochaena cuneifo	species	158754
recombined as	Glossus fraterna	species	98395
	Glossus fraterna	species	98395
	Glossus fraterna	species	98395
	Glossus fraterna	species	98395
	Glossus fraterna	species	98395
	Glossus fraterna	species	98395
	Glossus fraterna	species	98395
recombined as	Glossus fraterna	species	98395
	Glycymeris americana	species	90959
recombined as	Tucetona pectinata	species	90963
	Glycymeris americana	species	90959
recombined as	Tucetona pectinata	species	90963
	Glycymeris americana	species	90959
recombined as	Tucetona arata	species	109870
recombined as	Tucetona arata	species	109870
	Glycymeris americana	species	90959
	Glycymeris americana	species	90959
	Glycymeris americana	species	90959
recombined as	Tucetona pectinata	species	90963
recombined as	Tucetona pectinata	species	90963
	Glycymeris americana	species	90959
recombined as	Costaglycymeris subo	species	98549
	Glycymeris americana	species	90959
recombined as	Tucetona pectinata	species	90963
recombined as	Tucetona pectinata	species	90963
recombined as	Tucetona arata	species	109870
recombined as	Tucetona pectinata	species	90963
	Tucetona pectinata	species	90963
recombined as	Costaglycymeris subo	species	98549
	Glycymeris	genus	16243
recombined as	Costaglycymeris subo	species	98549
recombined as	Glycymeris decussata	species	90961
recombined as	Tucetona arata	species	109870
recombined as	Tucetona arata	species	109870
recombined as	Glycymeris americana	species	90959
	Glycymeris americana	species	90959
	Tucetona arata	species	109870

	Costaglycymeris subo	species	98549
	Tucetona arata	species	109870
	Tucetona	genus	16324
	Costaglycymeris subo	species	98549
	Glycymeris americana	species	90959
	Tucetona	genus	16324
	Glycymeris americana	species	90959
	Costaglycymeris subo	species	98549
	Glycymeris americana	species	90959
	Tucetona	genus	16324
	Tucetona	genus	16324
	Tucetona pectinata	species	90963
	Tucetona arata	species	109870
	Tucetona arata	species	109870
recombined as	Tucetona arata	species	109870
recombined as	Tucetona arata	species	109870
	Glycymeris americana	species	90959
	Tucetona pectinata	species	90963
	Glycymeris americana	species	90959
recombined as	Tucetona pectinata	species	90963
	Glycymeris americana	species	90959
	Glycymeris americana	species	90959
	Glycymeris americana	species	90959
	Glycymeris americana	species	90959
species not entered	Glycymeris	genus	16243
	Glycymeris americana	species	90959
recombined as	Costaglycymeris subo	species	98549
recombined as	Tucetona pectinata	species	90963
	Glycymeris americana	species	90959
recombined as	Tucetona pectinata	species	90963
recombined as	Tucetona pectinata	species	90963
recombined as	Tucetona pectinata	species	90963
	Glycymeris americana	species	90959
recombined as	Tucetona arata	species	109870
	Glycymeris americana	species	90959
	Tucetona pectinata	species	90963
	Tucetona arata	species	109870
	Glycymeris americana	species	90959
	Tucetona arata	species	109870
	Glycymeris americana	species	90959
subjective synonym of	Glycymeris	genus	16243
subjective synonym of, species not entered	Glycymeris	genus	16243
subjective synonym of, species not entered	Glycymeris	genus	16243
subjective synonym of	Glycymeris decussata	species	90961
species not entered	Glycymeris	genus	16243
recombined as	Glycymeris americana	species	90959
	Glycymeris americana	species	90959

subjective synonym of	Glycymeris decussata	species	90961
recombined as	Costaglycymeris subo	species	98549
	Glycymeris americana	species	90959
	Glycymeris sloani	species	82692
	Glycymeris americana	species	90959
	Glycymeris sloani	species	82692
	Glycymeris americana	species	90959
recombined as	Tucetona arata	species	109870
	Glycymeris hummi	species	82691
	Glycymeris sloani	species	82692
	Glycymeris americana	species	90959
	Glycymeris sloani	species	82692
	Glycymeris sloani	species	82692
recombined as	Tucetona arata	species	109870
	Costaglycymeris subo	species	98549
	Costaglycymeris subo	species	98549
species not entered	Glycymeris	genus	16243
species not entered	Glycymeris	genus	16243
species not entered	Glycymeris	genus	16243
species not entered	Glycymeris	genus	16243
species not entered	Glycymeris	genus	16243
	Glycymeris americana	species	90959
	Glycymeris americana	species	90959
	Glycymeris americana	species	90959
	Glycymeris americana	species	90959
	Glycymeris americana	species	90959
	Glycymeris americana	species	90959
	Glycymeris americana	species	90959
	Glycymeris americana	species	90959
	Glycymeris americana	species	90959
	Glycymeris americana	species	90959
	Glycymeris americana	species	90959
recombined as	Costaglycymeris subo	species	98549
	Glycymeris americana	species	90959
	Glycymeris americana	species	90959
	Glycymeris americana	species	90959
	Tucetona	genus	16324
	Glycymeris americana	species	90959
species not entered	Glycymeris	genus	16243
species not entered	Glycymeris	genus	16243
	Glycymeris americana	species	90959
	Glycymeris americana	species	90959
	Glycymeris americana	species	90959
	Glycymeris americana	species	90959
	Tucetona	genus	16324
	Glycymeris americana	species	90959
	Tucetona	genus	16324

	Glycymeris americana	species	90959
	Glycymeris americana	species	90959
species not entered	Glycymeris	genus	16243
	Tucetona arata	species	109870
	Glycymeris americana	species	90959
	Glycymeris	genus	16243
species not entered	Axinactis (Glycymerell	subgenus	16242
	Glycymeris americana	species	90959
species not entered	Axinactis (Glycymerell	subgenus	16242
	Costaglycymeris subo'	species	98549
	Glycymeris americana	species	90959
	Costaglycymeris subo'	species	98549
recombined as	Costaglycymeris subo'	species	98549
species not entered	Glycymeris	genus	16243
recombined as	Tucetona pectinata	species	90963
	Costaglycymeris subo'	species	98549
	Costaglycymeris subo'	species	98549
	Costaglycymeris subo'	species	98549
	Costaglycymeris subo'	species	98549
	Costaglycymeris subo'	species	98549
	Glycymeris americana	species	90959
	Costaglycymeris subo'	species	98549
	Costaglycymeris subo'	species	98549
	Costaglycymeris subo'	species	98549
	Costaglycymeris subo'	species	98549
	Costaglycymeris subo'	species	98549
	Costaglycymeris subo'	species	98549
	Costaglycymeris subo'	species	98549
	Glycymeris americana	species	90959
subjective synonym of	Hyotissa haitensis	species	99269
subjective synonym of	Hyotissa haitensis	species	99269
subjective synonym of	Hyotissa haitensis	species	99269
subjective synonym of	Hyotissa haitensis	species	99269
subjective synonym of	Hyotissa haitensis	species	99269
recombined as	Hyotissa haitensis	species	99269
recombined as	Hyotissa haitensis	species	99269
recombined as	Hyotissa haitensis	species	99269
subjective synonym of	Nanogyra (Palaeogyra	species	352868
subjective synonym of	Hyotissa haitensis	species	99269
recombined as	Hyotissa haitensis	species	99269
recombined as	Hyotissa haitensis	species	99269
species not entered	Hyotissa	genus	16717
species not entered	Hyotissa	genus	16717
subjective synonym of	Nanogyra (Palaeogyra	species	352868
subjective synonym of	Nanogyra (Palaeogyra	species	352868
subjective synonym of	Nanogyra (Palaeogyra	species	352868
recombined as	Hyotissa haitensis	species	99269

recombined as	Hyotissa haitensis	species	99269
recombined as	Hyotissa haitensis	species	99269
	Hyotissa haitensis	species	99269
corrected to	Pycnodonte	genus	62025
subjective synonym of	Nanogyra (Palaeogyra	species	352868
subjective synonym of	Nanogyra (Palaeogyra	species	352868
subjective synonym of	Nanogyra (Palaeogyra	species	352868
subjective synonym of	Nanogyra (Palaeogyra	species	352868
subjective synonym of	Nanogyra (Palaeogyra	species	352868
subjective synonym of	Nanogyra (Palaeogyra	species	352868
subjective synonym of	Nanogyra (Palaeogyra	species	352868
subjective synonym of	Nanogyra (Palaeogyra	species	352868
subjective synonym of	Nanogyra (Palaeogyra	species	352868
subjective synonym of	Nanogyra (Palaeogyra	species	352868
subjective synonym of	Nanogyra (Palaeogyra	species	352868
subjective synonym of	Nanogyra (Palaeogyra	species	352868
subjective synonym of	Nanogyra (Palaeogyra	species	352868
subjective synonym of	Nanogyra (Palaeogyra	species	352868
subjective synonym of	Nanogyra (Palaeogyra	species	352868
subjective synonym of	Nanogyra (Palaeogyra	species	352868
subjective synonym of	Nanogyra (Palaeogyra	species	352868
subjective synonym of	Nanogyra (Palaeogyra	species	352868
subjective synonym of	Nanogyra (Palaeogyra	species	352868
subjective synonym of	Nanogyra (Palaeogyra	species	352868
species not entered	Panopea	genus	61583
species not entered	Panopea	genus	61583
	Panopea bitruncata	species	157435
	Panopea bitruncata	species	157435
	Hiatella arctica	species	97917
species not entered	Panopea	genus	61583
species not entered	Panopea	genus	61583
	Hiatella arctica	species	97917
species not entered	Panopea	genus	61583
	Panopea	genus	61583
species not entered	Panopea	genus	61583
	Panopea bitruncata	species	157435
species not entered	Panopea	genus	61583
	Panopea	genus	61583
species not entered	Panopea	genus	61583
subjective synonym of	Hiatella	genus	18462
species not entered	Panopea	genus	61583
	Hiatella arctica	species	97917
	Hiatella arctica	species	97917
	Hiatella arctica	species	97917
	Hiatella arctica	species	97917
	Hiatella arctica	species	97917
species not entered	Panopea	genus	61583

	Panopea	genus	61583
	Panopea	genus	61583
species not entered	Panopea	genus	61583
species not entered	Panopea	genus	61583
species not entered	Panopea	genus	61583
species not entered	Panopea	genus	61583
	Hiatella arctica	species	97917
species not entered	Panopea	genus	61583
species not entered	Panopea	genus	61583
	Panopea	genus	61583
	Hiatella arctica	species	97917
	Hiatella arctica	species	97917
	Hiatella arctica	species	97917
	Hiatella arctica	species	97917
	Hiatella arctica	species	97917
	Hiatella arctica	species	97917
species not entered	Panopea	genus	61583
species not entered	Panopea	genus	61583
species not entered	Panopea	genus	61583
	Panopea goldfussii	species	97915
species not entered	Bornia	genus	17408
species not entered	Bornia	genus	17408
species not entered	Bornia	genus	17408
	Bornia triangula	species	98424
	Bornia triangula	species	98424
	Bornia triangula	species	98424
	Bornia triangula	species	98424
	Bornia triangula	species	98424
	Bornia triangula	species	98424
	Bornia triangula	species	98424
	Bornia triangula	species	98424
	Bornia triangula	species	98424
	Bornia triangula	species	98424
	Bornia triangula	species	98424
species not entered	Bornia	genus	17408
species not entered	Bornia	genus	17408
species not entered	Bornia	genus	17408
species not entered	Bornia	genus	17408
species not entered	Bornia	genus	17408
species not entered	Bornia	genus	17408
species not entered	Bornia	genus	17408
	Aligena elevata	species	108654
	Aligena aequata	species	98433
	Aligena striata	species	293505
	Aligena striata	species	293505
species not entered	Aligena	genus	17315
	Aligena striata	species	293505

	Aligena striata	species	293505
species not entered	Aligena	genus	17315
	Aligena aequata	species	98433
recombined as	Ctenoides (Ctenoides)	species	87557
recombined as	Limaria (Limaria) caro	species	158821
	Lima	genus	16765
	Lima	genus	16765
species not entered	Limea	genus	16771
	Lima caribaea	species	90974
recombined as	Limaria pellucida	species	156668
recombined as	Ctenoides (Ctenoides)	species	87557
recombined as	Lima subauriculata	species	140655
recombined as	Limaria (Limaria) caro	species	158821
recombined as	Ctenoides (Ctenoides)	species	87557
recombined as	Ctenoides (Ctenoides)	species	158812
obsolete variant of	Limaria	genus	16767
recombined as	Limaria (Limaria) caro	species	158821
	Ctenoides (Ctenoides)	species	158812
	Limaria (Limaria) caro	species	158821
	Limaria (Limaria) chip	species	158813
obsolete variant of	Lima (Promantellum)	species	120103
recombined as	Limaria (Limaria) caro	species	158821
	Limatula	genus	16769
	Limaria	genus	16767
recombined as	Parvilucina multistriat	species	95166
species not entered	Phacoides	genus	54125
	Phacoides	genus	54125
recombined as	Stewartia floridana	species	82702
recombined as	Divalinga quadrisulcat	species	98459
	Divaricella	genus	17586
recombined as	Bellucina waccamawe	species	82705
	Anodontia alba	species	94612
recombined as	Parvilucina costata	species	156717
species not entered	Divaricella	genus	17586
recombined as	Miltha (Miltha) caloos	species	157931
recombined as	Stewartia anodonta	species	98448
recombined as	Parvilucina multistriat	species	95166
recombined as	Lucinisca nassula	species	97987
replaced by	Callucina keenae	species	102585
recombined as	Bellucina waccamawe	species	82705
	Anodontia alba	species	94612
species not entered	Divaricella	genus	17586
recombined as	Miltha (Miltha) caloos	species	157931
recombined as	Parvilucina multistriat	species	95166
recombined as	Bellucina waccamawe	species	82705
	Anodontia alba	species	94612
recombined as	Parvilucina costata	species	156717

recombined as	Codakia (Codakia) orb	species	94546
	Divaricella	genus	17586
	Lucina pensylvanica	species	94535
recombined as	Stewartia anodonta	species	98448
recombined as	Parvilucina multistriat	species	95166
recombined as	Lucinisca nassula	species	97987
replaced by	Callucina keenae	species	102585
recombined as	Bellucina waccamawe	species	82705
recombined as	Parvilucina multistriat	species	95166
recombined as	Lucinisca nassula	species	97987
	Anodontia	genus	17339
recombined as	Codakia (Codakia) orb	species	94546
species not entered	Divaricella	genus	17586
	Lucina pensylvanica	species	94535
recombined as	Miltha (Miltha) caloos	species	157931
recombined as	Parvilucina multistriat	species	95166
recombined as	Lucinisca nassula	species	97987
replaced by	Callucina keenae	species	102585
recombined as	Bellucina waccamawe	species	82705
recombined as	Parvilucina multistriat	species	95166
	Divaricella	genus	17586
	Lucina pensylvanica	species	94535
species not entered	Phacoides	genus	54125
replaced by	Callucina keenae	species	102585
	Phacoides	genus	54125
recombined as	Cavilinga trisulcata	species	82707
species not entered	Phacoides	genus	54125
recombined as	Ctena (Ctena) magnol	species	95160
recombined as	Ctena (Ctena) specios	species	95155
recombined as	Ctena (Ctena) specios	species	95155
subjective synonym of, species not entered	Ctena	genus	17533
subjective synonym of	Anodontia alba	species	94612
subjective synonym of	Anodontia alba	species	94612
subjective synonym of	Anodontia alba	species	94612
recombined as	Cavilinga trisulcata	species	82707
recombined as	Lucinisca cribrarius	species	95172
recombined as	Lucinisca cribrarius	species	95172
recombined as	Lucinisca cribrarius	species	95172
recombined as	Lucinisca cribrarius	species	95172
recombined as	Parvilucina crenulata	species	98454
recombined as	Parvilucina crenulata	species	98454
recombined as	Parvilucina crenulata	species	98454
recombined as	Parvilucina crenulata	species	98454
recombined as	Parvilucina crenulata	species	98454
recombined as	Parvilucina multistriat	species	95166
recombined as	Parvilucina multistriat	species	95166
recombined as	Parvilucina multistriat	species	95166

species not entered	Phacoides (Bellucina)	subgenus	82704
species not entered	Phacoides (Bellucina)	subgenus	82704
species not entered	Phacoides (Bellucina)	subgenus	82704
species not entered	Phacoides (Bellucina)	subgenus	82704
species not entered	Phacoides (Bellucina)	subgenus	82704
	Anodontia (Pegophys	species	106100
	Anodontia alba	species	94612
recombined as	Codakia (Codakia) orb	species	94546
species not entered	Linga	genus	17817
recombined as	Lucinisca nassula	species	97987
recombined as	Cavilinga blanda	species	94562
recombined as	Parvilucina multistriat	species	95166
recombined as	Stewartia floridana	species	82702
species not entered	Linga	genus	17817
recombined as	Lucinisca nassula	species	97987
recombined as	Callucina (Callucina) k	species	102584
recombined as	Cavilinga blanda	species	94562
recombined as	Parvilucina multistriat	species	95166
	Anodontia alba	species	94612
recombined as	Divalinga quadrisulcat	species	98459
recombined as	Lucina pensylvanica	species	94535
species not entered	Linga	genus	17817
recombined as	Lucinisca nassula	species	97987
recombined as	Parvilucina multistriat	species	95166
	Lucina pensylvanica	species	94535
recombined as	Parvilucina multistriat	species	95166
recombined as	Lucinisca nassula	species	97987
recombined as	Bellucina waccamawe	species	82705
species not entered	Phacoides	genus	54125
replaced by	Callucina keenae	species	102585
	Anodontia alba	species	94612
obsolete variant of	Miltha (Miltha) carme	species	157935
recombined as	Ctena orbiculata	species	94551
recombined as	Codakia (Codakia) orb	species	94546
recombined as	Parvilucina costata	species	156717
	Divalinga quadrisulcat	species	98459
species not entered	Phacoides	genus	54125
	Lucina pensylvanica	species	94535
	Radiolucina amianta	species	94603
	Stewartia floridana	species	82702
replaced by	Callucina keenae	species	102585
	Lucina pensylvanica	species	94535
	Stewartia floridana	species	82702
	Radiolucina amianta	species	94603
	Lucinoma	genus	17840
	Anodontia alba	species	94612
	Stewartia anodonta	species	98448

recombined as	Parvilucina multistriat	species	95166
species not entered	Phacoides (Bellucina)	subgenus	82704
species not entered	Phacoides (Bellucina)	subgenus	82704
species not entered	Lucina (Lucinisca)	subgenus	17839
recombined as	Parvilucina multistriat	species	95166
species not entered	Cardiolucina	genus	95163
replaced by	Callucina keenae	species	102585
recombined as	Parvilucina multistriat	species	95166
recombined as	Parvilucina crenulata	species	98454
obsolete variant of	Cardiolucina	genus	95163
species not entered	Here	genus	17726
obsolete variant of	Cardiolucina	genus	95163
recombined as	Cavilinga trisulcata	species	82707
species not entered	Here	genus	17726
recombined as	Cavilinga trisulcata	species	82707
recombined as	Cavilinga trisulcata	species	82707
recombined as	Divalinga quadrisulcat	species	98459
recombined as	Codakia (Codakia) orb	species	94546
recombined as	Codakia (Codakia) orb	species	94546
recombined as	Phacoides pectinatus	species	156708
recombined as	Anodontia (Pegophyse	species	106100
	Miltha (Miltha) caloos	species	157931
recombined as	Ctena orbiculata	species	94551
species not entered	Phacoides	genus	54125
species not entered	Divaricella	genus	17586
recombined as	Codakia (Codakia) orb	species	94546
recombined as	Ctena orbiculata	species	94551
recombined as	Stewartia anodonta	species	98448
recombined as	Phacoides pectinatus	species	156708
recombined as	Armimiltha disciformi:	species	157844
replaced by	Callucina keenae	species	102585
recombined as	Cavilinga trisulcata	species	82707
recombined as	Parvilucina multistriat	species	95166
recombined as	Bellucina waccamawe	species	82705
species not entered	Phacoides	genus	54125
recombined as	Lucinisca nassula	species	97987
	Miltha (Miltha) caloos	species	157931
species not entered	Divaricella	genus	17586
recombined as	Lucina pensylvanica	species	94535
recombined as	Stewartia floridana	species	82702
obsolete variant of	Miltha (Miltha) carme	species	157935
	Codakia	genus	17488
	Miltha	genus	100542
species not entered	Phacoides	genus	54125
	Stewartia anodonta	species	98448
	Lucinisca nassula	species	97987
replaced by	Callucina keenae	species	102585

	Stewartia anodonta	species	98448
	Lucinisca nassula	species	97987
	Stewartia anodonta	species	98448
recombined as	Parvilucina multistriat	species	95166
recombined as	Stewartia anodonta	species	98448
recombined as	Parvilucina multistriat	species	95166
recombined as	Bellucina waccamawe	species	82705
	Stewartia floridana	species	82702
	Bellucina waccamawe	species	82705
	Lucinisca nassula	species	97987
recombined as	Parvilucina multistriat	species	95166
	Lucina pensylvanica	species	94535
species not entered	Radiolucina	genus	94604
recombined as	Parvilucina multistriat	species	95166
recombined as	Anodontia (Pegophyse	species	106100
	Lucinisca nassula	species	97987
	Radiolucina amianta	species	94603
	Stewartia anodonta	species	98448
	Lucinisca nassula	species	97987
reassigned as	Anodontia (Pegophyse	subgenus	18045
species not entered	Radiolucina	genus	94604
species not entered	Radiolucina	genus	94604
	Stewartia anodonta	species	98448
	Bellucina waccamawe	species	82705
recombined as	Bellucina waccamawe	species	82705
recombined as	Parvilucina multistriat	species	95166
recombined as	Bellucina waccamawe	species	82705
recombined as	Parvilucina multistriat	species	95166
	Miltha (Miltha) caloos	species	157931
	Miltha (Miltha) caloos	species	157931
	Miltha (Miltha) caloos	species	157931
	Miltha (Miltha) caloos	species	157931
	Miltha (Miltha) caloos	species	157931
	Miltha (Miltha) caloos	species	157931
	Miltha (Miltha) caloos	species	157931
	Miltha (Miltha) caloos	species	157931
	Miltha (Miltha) caloos	species	157931
	Miltha (Miltha) caloos	species	157931
	Miltha (Miltha) caloos	species	157931
	Miltha (Miltha) caloos	species	157931
	Miltha (Miltha) caloos	species	157931
	Miltha (Miltha) caloos	species	157931
	Miltha (Miltha) caloos	species	157931
	Miltha (Miltha) caloos	species	157931
	Miltha (Miltha) caloos	species	157931
	Miltha (Miltha) caloos	species	157931
	Miltha (Miltha) caloos	species	157931

	Miltha (Miltha) caloos	species	157931
	Miltha (Miltha) caloos	species	157931
	Miltha (Miltha) caloos	species	157931
	Miltha (Miltha) caloos	species	157931
	Miltha (Miltha) caloos	species	157931
	Miltha (Miltha) caloos	species	157931
	Miltha (Miltha) caloos	species	157931
	Miltha (Miltha) caloos	species	157931
	Miltha (Miltha) caloos	species	157931
	Miltha (Miltha) caloos	species	157931
	Miltha (Miltha) carme	species	157935
	Miltha (Miltha) carme	species	157935
	Miltha (Miltha) carme	species	157935
	Miltha (Miltha) carme	species	157935
	Miltha (Miltha) carme	species	157935
	Miltha (Miltha) carme	species	157935
recombined as	Parvilucina multistriat	species	95166
recombined as	Parvilucina crenulata	species	98454
species not entered	Lucinoma	genus	17840
recombined as	Lucinisca cribrarius	species	95172
	Anodontia alba	species	94612
	Callucina keenae	species	102585
recombined as	Codakia (Codakia) orb	species	94546
	Divalinga quadrisulcat	species	98459
	Lucina pensylvanica	species	94535
	Lucinisca nassula	species	97987
	Parvilucina crenella	species	102588
	Radiolucina amianta	species	94603
	Stewartia floridana	species	82702
species not entered	Lucinoma	genus	17840
recombined as	Divalinga quadrisulcat	species	98459
recombined as	Divalinga quadrisulcat	species	98459
recombined as	Parvilucina multistriat	species	95166
recombined as	Parvilucina multistriat	species	95166
recombined as	Bellucina waccamawe	species	82705
recombined as	Divalinga quadrisulcat	species	98459
species not entered	Lucina	genus	17835
recombined as	Parvilucina multistriat	species	95166
recombined as	Divalinga quadrisulcat	species	98459
recombined as	Parvilucina multistriat	species	95166
recombined as	Parvilucina multistriat	species	95166
recombined as	Lucina pensylvanica	species	94535
recombined as	Lucinisca cribrarius	species	95172
species not entered	Radiolucina	genus	94604
recombined as	Ctena (Ctena) specios	species	95155
recombined as	Stewartia anodonta	species	98448
	Divalinga quadrisulcat	species	98459

species not entered	Radiolucina	genus	94604
recombined as	Parvilucina multistriat	species	95166
recombined as	Ctena (Ctena) specios	species	95155
recombined as	Stewartia anodonta	species	98448
recombined as	Parvilucina multistriat	species	95166
recombined as	Bellucina waccamawe	species	82705
recombined as	Cavilinga trisulcata	species	82707
replaced by	Callucina keenae	species	102585
recombined as	Parvilucina multistriat	species	95166
recombined as	Radiolucina amianta	species	94603
recombined as	Radiolucina amianta	species	94603
recombined as	Parvilucina crenella	species	102588
recombined as	Parvilucina crenella	species	102588
recombined as	Parvilucina costata	species	156717
recombined as	Divalinga quadrisulcat	species	98459
recombined as	Divalinga quadrisulcat	species	98459
recombined as	Divalinga quadrisulcat	species	98459
recombined as	Divalinga quadrisulcat	species	98459
species not entered	Linga	genus	17817
species not entered	Divalinga (Divalinga)	subgenus	109760
	Divalinga quadrisulcat	species	98459
	Divalinga quadrisulcat	species	98459
	Radiolucina amianta	species	94603
	Divalinga quadrisulcat	species	98459
	Radiolucina amianta	species	94603
	Radiolucina amianta	species	94603
recombined as	Parvilucina multistriat	species	95166
	Divalinga quadrisulcat	species	98459
	Radiolucina amianta	species	94603
	Stewartia anodonta	species	98448
recombined as	Parvilucina multistriat	species	95166
species not entered	Radiolucina	genus	94604
	Stewartia anodonta	species	98448
recombined as	Bellucina waccamawe	species	82705
replaced by	Callucina keenae	species	102585
recombined as	Parvilucina multistriat	species	95166
recombined as	Bellucina waccamawe	species	82705
recombined as	Lucinisca cribrarius	species	95172
species not entered	Phacoides	genus	54125
recombined as	Parvilucina crenulata	species	98454
replaced by	Callucina keenae	species	102585
recombined as	Divalinga quadrisulcat	species	98459
	Lucina kiawahensis	species	392334
recombined as	Parvilucina costata	species	156717
recombined as	Parvilucina crenulata	species	98454
	Cavilinga trisulcata	species	82707
	Cavilinga trisulcata	species	82707

recombined as	Parvilucina multistriat	species	95166
	Stewartia anodonta	species	98448
recombined as	Parvilucina multistriat	species	95166
recombined as	Ctena (Ctena) magnol	species	95160
recombined as	Ctena (Ctena) specios	species	95155
recombined as	Ctena (Ctena) microin	species	95162
recombined as	Cavilinga trisulcata	species	82707
recombined as	Cavilinga trisulcata	species	82707
recombined as	Cavilinga trisulcata	species	82707
recombined as	Cavilinga trisulcata	species	82707
recombined as	Lucinisca cribrarius	species	95172
recombined as	Parvilucina multistriat	species	95166
recombined as	Parvilucina multistriat	species	95166
recombined as	Parvilucina multistriat	species	95166
recombined as	Parvilucina multistriat	species	95166
recombined as	Parvilucina multistriat	species	95166
recombined as	Parvilucina multistriat	species	95166
recombined as	Parvilucina multistriat	species	95166
recombined as	Parvilucina multistriat	species	95166
recombined as	Parvilucina multistriat	species	95166
recombined as	Parvilucina multistriat	species	95166
recombined as	Cavilinga trisulcata	species	82707
replaced by	Callucina keenae	species	102585
recombined as	Parvilucina multistriat	species	95166
recombined as	Radiolucina amianta	species	94603
recombined as	Lucinisca nassula	species	97987
recombined as	Parvilucina crenella	species	102588
recombined as	Parvilucina crenella	species	102588
recombined as	Parvilucina crenella	species	102588
recombined as	Parvilucina crenella	species	102588
	Anodontia alba	species	94612
recombined as	Parvilucina costata	species	156717
recombined as	Divalinga quadrisulcat	species	98459
recombined as	Divalinga quadrisulcat	species	98459
recombined as	Divalinga quadrisulcat	species	98459
recombined as	Divalinga quadrisulcat	species	98459
species not entered	Divalinga (Divalinga)	subgenus	109760
species not entered	Linga	genus	17817
replaced by	Callucina keenae	species	102585
	Cavilinga trisulcata	species	82707
	Parvilucina crenulata	species	98454
	Stewartia anodonta	species	98448
	Cavilinga trisulcata	species	82707
	Stewartia anodonta	species	98448
	Bellucina waccamawe	species	82705
	Divalinga	genus	17583
	Lucinidae	family	60971

	Lucinidae	family	60971
	Stewartia anodonta	species	98448
species not entered	Cardiolucina	genus	95163
species not entered	Cardiolucina	genus	95163
	Cavilucina	genus	17452
	Stewartia anodonta	species	98448
	Cavilucina	genus	17452
	Lucina pensylvanica	species	94535
	Lucinidae	family	60971
	Stewartia anodonta	species	98448
	Divalinga quadrisulcat	species	98459
	Stewartia anodonta	species	98448
	Divalinga quadrisulcat	species	98459
	Cavilinga	genus	17451
	Lucina	genus	17835
	Linga	genus	17817
	Stewartia anodonta	species	98448
	Callucina	genus	17428
subjective synonym of	Cardiolucina	genus	95163
	Divalinga quadrisulcat	species	98459
	Linga	genus	17817
	Bellucina waccamawe	species	82705
	Miltha	genus	100542
	Stewartia anodonta	species	98448
	Divalinga quadrisulcat	species	98459
recombined as	Parvilucina multistriat	species	95166
	Divalinga quadrisulcat	species	98459
	Divalinga quadrisulcat	species	98459
	Stewartia anodonta	species	98448
	Lucinoma contracta	species	98452
recombined as	Stewartia anodonta	species	98448
	Parvilucina crenulata	species	98454
	Parvilucina crenulata	species	98454
	Parvilucina crenulata	species	98454
	Parvilucina crenulata	species	98454
	Stewartia anodonta	species	98448
	Stewartia anodonta	species	98448
recombined as	Parvilucina crenulata	species	98454
recombined as	Parvilucina crenulata	species	98454
recombined as	Parvilucina crenulata	species	98454
recombined as	Parvilucina crenulata	species	98454
recombined as	Parvilucina crenulata	species	98454
recombined as	Parvilucina crenulata	species	98454
recombined as	Ctena (Ctena) specios	species	95155
	Parvilucina crenulata	species	98454
	Parvilucina crenulata	species	98454
	Stewartia anodonta	species	98448

	Stewartia anodonta	species	98448
	Divalinga quadrisulcat	species	98459
recombined as	Parvilucina crenulata	species	98454
	Divalinga quadrisulcat	species	98459
	Stewartia anodonta	species	98448
species not entered	Lucina (Lucinisca)	subgenus	17839
	Stewartia anodonta	species	98448
	Stewartia anodonta	species	98448
	Lyonsia hyalina	species	107597
	Mulinia lateralis	species	94391
	Mactra	genus	17859
recombined as	Hemimactra solidissin	species	94388
species not entered	Rangia	genus	18179
	Spisula	genus	18275
	Mulinia lateralis	species	94391
subjective synonym of	Rangia cuneata	species	156282
	Mulinia lateralis	species	94391
	Mulinia lateralis	species	94391
species not entered	Mulinia	genus	17933
subjective synonym of	Rangia cuneata	species	156282
	Mulinia lateralis	species	94391
	Mulinia lateralis	species	94391
	Mulinia lateralis	species	94391
species not entered	Mulinia	genus	17933
subjective synonym of	Rangia cuneata	species	156282
	Mulinia lateralis	species	94391
	Mulinia congesta	species	120107
species not entered	Mulinia	genus	17933
subjective synonym of	Rangia cuneata	species	156282
species not entered	Mactrotoma	genus	120906
recombined as	Leptomactra delumbis	species	97974
obsolete variant of	Spisula	genus	18275
obsolete variant of	Spisula	genus	18275
obsolete variant of	Spisula	genus	18275
	Mulinia congesta	species	120107
	Mulinia congesta	species	120107
	Mulinia congesta	species	120107
	Mulinia congesta	species	120107
recombined as	Mactrotoma fragilis	species	156278
	Mulinia lateralis	species	94391
	Rangia cuneata	species	156282
	Rangia cuneata	species	156282
	Mulinia lateralis	species	94391
	Mulinia lateralis	species	94391
	Rangia cuneata	species	156282
recombined as	Mactrotoma fragilis	species	156278
	Mulinia	genus	17933

	Rangia cuneata	species	156282
	Mactrotoma fragilis	species	156278
	Spisula	genus	18275
species not entered	Mulinia	genus	17933
	Mulinia congesta	species	120107
	Mulinia lateralis	species	94391
	Mulinia lateralis	species	94391
	Mulinia congesta	species	120107
	Mulinia congesta	species	120107
	Mulinia congesta	species	120107
	Mulinia	genus	17933
	Mulinia congesta	species	120107
species not entered	Mulinia	genus	17933
species not entered	Mulinia	genus	17933
subjective synonym of, species not entered	Mactrotoma	genus	120906
	Mulinia congesta	species	120107
	Mulinia lateralis	species	94391
species not entered	Mulinia	genus	17933
species not entered	Mulinia	genus	17933
subjective synonym of	Rangia cuneata	species	156282
	Rangia cuneata	species	156282
	Mulinia	genus	17933
	Mulinia lateralis	species	94391
recombined as	Spisula (Hemimactra)	species	97982
species not entered	Spisula	genus	18275
	Mulinia	genus	17933
species not entered	Mulinia	genus	17933
	Mulinia lateralis	species	94391
species not entered	Mulinia	genus	17933
species not entered	Hemimactra	genus	17724
	Mulinia congesta	species	120107
species not entered	Mulinia	genus	17933
	Mactrotoma	genus	120906
species not entered	Mulinia	genus	17933
species not entered	Mactrotoma	genus	120906
	Mulinia lateralis	species	94391
	Mulinia lateralis	species	94391
	Mulinia congesta	species	120107
	Mulinia lateralis	species	94391
	Mulinia congesta	species	120107
	Mulinia congesta	species	120107
	Mulinia congesta	species	120107
	Rangia cuneata	species	156282
	Hemimactra	genus	17724
	Mulinia lateralis	species	94391
species not entered	Hemimactra	genus	17724
	Rangia cuneata	species	156282

species not entered	Mulinia	genus	17933
	Mactrotoma fragilis	species	156278
	Mactridae	family	61026
	Rangia cuneata	species	156282
	Mulinia lateralis	species	94391
	Rangia cuneata	species	156282
species not entered	Mulinia	genus	17933
subjective synonym of	Rangia cuneata	species	156282
	Mactrotoma fragilis	species	156278
	Mulinia lateralis	species	94391
	Rangia cuneata	species	156282
	Mulinia congesta	species	120107
	Mulinia lateralis	species	94391
	Mulinia lateralis	species	94391
	Mulinia lateralis	species	94391
	Mulinia lateralis	species	94391
	Rangia cuneata	species	156282
	Mulinia lateralis	species	94391
	Mulinia congesta	species	120107
	Mulinia lateralis	species	94391
recombined as	Hemimactra solidissin	species	94388
	Mulinia lateralis	species	94391
recombined as	Hemimactra solidissin	species	94388
	Mulinia lateralis	species	94391
recombined as	Hemimactra solidissin	species	94388
	Mulinia lateralis	species	94391
	Mulinia lateralis	species	94391
recombined as	Hemimactra solidissin	species	94388
recombined as	Hemimactra solidissin	species	94388
	Mulinia congesta	species	120107
species not entered	Rangia	genus	18179
recombined as	Hemimactra solidissin	species	94388
	Mulinia lateralis	species	94391
	Mulinia lateralis	species	94391
	Mulinia lateralis	species	94391
	Mulinia lateralis	species	94391
	Mulinia lateralis	species	94391
recombined as	Hemimactra solidissin	species	94388
recombined as	Hemimactra solidissin	species	94388
recombined as	Mactrotoma fragilis	species	156278
subjective synonym of	Hemimactra solidissin	species	94388
	Mulinia lateralis	species	94391
	Mulinia lateralis	species	94391
	Mulinia lateralis	species	94391
	Mulinia lateralis	species	94391
	Mulinia lateralis	species	94391
	Hemimactra	genus	17724

	Mulinia lateralis	species	94391
	Hemimactra	genus	17724
	Mulinia lateralis	species	94391
	Hemimactra	genus	17724
	Mulinia congesta	species	120107
	Hemimactra	genus	17724
recombined as	Mulinia lateralis	species	94391
recombined as	Mulinia lateralis	species	94391
subjective synonym of	Hemimactra solidissin	species	94388
misspelling of, recombined as	Rangia cuneata	species	156282
recombined as	Mulinia lateralis	species	94391
	Mulinia lateralis	species	94391
species not entered	Rangia	genus	18179
subjective synonym of	Hemimactra solidissin	species	94388
recombined as	Hemimactra solidissin	species	94388
recombined as	Hemimactra solidissin	species	94388
recombined as	Mactrotoma fragilis	species	156278
species not entered	Hemimactra	genus	17724
species not entered	Hemimactra	genus	17724
recombined as	Hemimactra solidissin	species	94388
recombined as	Hemimactra solidissin	species	94388
recombined as	Hemimactra solidissin	species	94388
	Mulinia congesta	species	120107
	Mulinia congesta	species	120107
	Mulinia congesta	species	120107
	Mulinia congesta	species	120107
	Mulinia congesta	species	120107
	Mulinia congesta	species	120107
	Mulinia congesta	species	120107
	Mulinia congesta	species	120107
	Mulinia congesta	species	120107
	Mulinia congesta	species	120107
	Mulinia congesta	species	120107
	Mulinia congesta	species	120107
	Mulinia lateralis	species	94391
	Mulinia lateralis	species	94391
	Spisula	genus	18275
	Mulinia lateralis	species	94391
	Mulinia lateralis	species	94391
	Rangia cuneata	species	156282
	Rangia cuneata	species	156282
	Rangia cuneata	species	156282
	Mulinia lateralis	species	94391
	Mulinia lateralis	species	94391
	Mulinia lateralis	species	94391
	Mulinia lateralis	species	94391
	Mulinia lateralis	species	94391

	Mulinia lateralis	species	94391
	Mulinia lateralis	species	94391
	Mulinia lateralis	species	94391
	Mulinia lateralis	species	94391
	Mulinia lateralis	species	94391
recombined as	Hemimactra solidissin	species	94388
recombined as	Hemimactra solidissin	species	94388
recombined as	Hemimactra solidissin	species	94388
recombined as	Hemimactra solidissin	species	94388
	Mulinia lateralis	species	94391
recombined as	Hemimactra solidissin	species	94388
	Mulinia lateralis	species	94391
	Mactridae	family	61026
	Mulinia lateralis	species	94391
	Hemimactra solidissin	species	94388
	Mulinia congesta	species	120107
	Mulinia congesta	species	120107
	Hemimactra solidissin	species	94388
	Mactridae	family	61026
	Mulinia congesta	species	120107
	Mulinia congesta	species	120107
species not entered	Hemimactra	genus	17724
	Mulinia congesta	species	120107
	Mulinia congesta	species	120107
	Spisula	genus	18275
	Mulinia congesta	species	120107
	Mactridae	family	61026
	Rangia cuneata	species	156282
	Mulinia lateralis	species	94391
	Hemimactra solidissin	species	94388
species not entered	Hemimactra	genus	17724
	Hemimactra	genus	17724
	Mulinia	genus	17933
	Harvella	genus	81259
recombined as	Hemimactra solidissin	species	94388
	Mulinia lateralis	species	94391
species not entered	Hemimactra	genus	17724
recombined as	Hemimactra solidissin	species	94388
	Mulinia lateralis	species	94391
	Mulinia lateralis	species	94391
	Mulinia lateralis	species	94391
	Mulinia lateralis	species	94391
	Mulinia lateralis	species	94391
	Mulinia lateralis	species	94391
	Mulinia lateralis	species	94391
species not entered	Spisula	genus	18275
	Mulinia congesta	species	120107

species not entered	Hemimactra	genus	17724
	Mulinia lateralis	species	94391
	Mulinia lateralis	species	94391
	Mulinia lateralis	species	94391
recombined as	Hemimactra solidissin	species	94388
	Rangia cuneata	species	156282
	Mulinia lateralis	species	94391
	Rangia cuneata	species	156282
recombined as	Hemimactra solidissin	species	94388
	Rangia cuneata	species	156282
recombined as	Hemimactra solidissin	species	94388
recombined as	Hemimactra solidissin	species	94388
	Mulinia lateralis	species	94391
	Mulinia lateralis	species	94391
	Rangia cuneata	species	156282
	Mulinia lateralis	species	94391
	Rangia cuneata	species	156282
recombined as	Spisula (Hemimactra)	species	97980
recombined as	Hemimactra solidissin	species	94388
	Rangia cuneata	species	156282
	Mulinia lateralis	species	94391
	Leptomactra delumbis	species	97974
	Spisula	genus	18275
species not entered	Spisula	genus	18275
	Mulinia congesta	species	120107
species not entered	Spisula	genus	18275
	Mulinia congesta	species	120107
	Mulinia congesta	species	120107
species not entered	Spisula	genus	18275
species not entered	Rangia	genus	18179
species not entered	Spisula	genus	18275
	Leptomactra delumbis	species	97974
	Leptomactra delumbis	species	97974
species not entered	Spisula	genus	18275
	Mulinia congesta	species	120107
species not entered	Mulinia	genus	17933
	Mactridae	family	61026
species not entered	Mulinia	genus	17933
	Mulinia congesta	species	120107
	Mulinia lateralis	species	94391
	Mulinia lateralis	species	94391
	Mulinia lateralis	species	94391
	Hemimactra solidissin	species	94388
	Mulinia lateralis	species	94391
	Isognomon alatus	species	156535
species not entered	Pedalion	genus	369733
species not entered	Isognomon	genus	16730

	Isognomon alatus	species	156535
species not entered	Pedalion	genus	369733
species not entered	Pedalion	genus	369733
species not entered	Isognomon	genus	16730
	Margaritaria abrupta	species	98493
	Margaritaria abrupta	species	98493
	Margaritaria abrupta	species	98493
recombined as	Montacuta floridana	species	91183
species not entered	Mysella	genus	17949
recombined as	Montacuta floridana	species	91183
recombined as	Montacuta floridana	species	91183
species not entered	Mysella	genus	17949
	Montacuta floridana	species	91183
	Montacuta	genus	17931
species not entered	Mysella	genus	17949
obsolete variant of	Montacuta floridana	species	91183
species not entered	Mysella	genus	17949
species not entered	Mysella	genus	17949
	Mysella beaufortensis	species	82710
species not entered	Mysella	genus	17949
species not entered	Mysella	genus	17949
species not entered	Mysella	genus	17949
species not entered	Mysella	genus	17949
species not entered	Mysella	genus	17949
species not entered	Mysella	genus	17949
species not entered	Mysella	genus	17949
species not entered	Mysella	genus	17949
	Sphenia	genus	18504
species not entered	Sphenia	genus	18504
species not entered	Sphenia	genus	18504
recombined as	Mya (Arenomya) aren	species	95559
species not entered	Sphenia	genus	18504
recombined as	Mya (Arenomya) aren	species	95559
recombined as	Mya (Arenomya) aren	species	95559
recombined as	Paramya subovata	species	97927
recombined as	Mya (Arenomya) aren	species	95559
	Paramya subovata	species	97927
	Paramya subovata	species	97927
	Paramya subovata	species	97927
recombined as	Mya (Arenomya) aren	species	95559
recombined as	Mya (Arenomya) aren	species	95559
	Sphenia	genus	18504
	Sphenia	genus	18504
	Sphenia	genus	18504
	Sphenia	genus	18504
recombined as	Mya (Arenomya) aren	species	95559
	Mya (Arenomya) aren	species	95559

	Mya (Arenomya) aren	species	95559
	Sphenia dubia	species	97925
	Sphenia	genus	18504
	Sphenia	genus	18504
	Paramya subovata	species	97927
recombined as	Mya (Arenomya) aren	species	95559
	Paramya subovata	species	97927
	Sphenia dubia	species	97925
	Sphenia dubia	species	97925
	Myochama	genus	18767
	Myochama	genus	18767
species not entered	Brachiodontes	genus	64227
species not entered	Brachiodontes	genus	64227
subjective synonym of	Crenella decussata	species	132361
	Modiolus americanus	species	90990
species not entered	Brachiodontes	genus	64227
species not entered	Mytilus	genus	16392
species not entered	Mytilus	genus	16392
	Brachidontes exustus	species	87572
	Modiolus	genus	77219
	Brachidontes exustus	species	87572
	Lithophaga	genus	16370
	Brachidontes exustus	species	87572
subjective synonym of	Crenella decussata	species	132361
	Lithophaga	genus	16370
	Modiolus americanus	species	90990
	Brachidontes exustus	species	87572
	Botula fusca	species	90977
	Brachidontes	genus	16347
	Modiolus americanus	species	90990
	Mytilus	genus	16392
	Mytilus	genus	16392
	Mytilus	genus	16392
recombined as	Brachidontes exustus	species	87572
species not entered	Brachidontes (Brachid	subgenus	110029
subjective synonym of	Crenella decussata	species	132361
species not entered	Mytiloconcha	genus	90011
species not entered	Mytiloconcha	genus	90011
species not entered	Mytilus	genus	16392
species not entered	Perna	genus	16396
	Brachidontes exustus	species	87572
	Brachidontes	genus	16347
	Modiolus	genus	77219
subjective synonym of	Crenella decussata	species	132361
species not entered	Brachiodontes	genus	64227
	Modiolus	genus	77219
	Perna	genus	16396

	Modiolus	genus	77219
	Modiolus	genus	77219
species not entered	Brachiodontes	genus	64227
	Modiolus	genus	77219
species not entered	Brachiodontes	genus	64227
species not entered	Brachiodontes	genus	64227
	Brachidontes	genus	16347
	Mytiloconcha	genus	90011
	Brachidontes exustus	species	87572
species not entered	Perna	genus	16396
	Modiolaria	genus	83859
species not entered	Modiolus	genus	77219
	Lithophaga	genus	16370
	Brachidontes exustus	species	87572
	Mytilus	genus	16392
	Aulacomya	genus	16343
	Modiolus	genus	77219
	Mytilus	genus	16392
	Crenella decussata	species	132361
recombined as	Modiolus (Modiolus) r	species	87564
	Crenella decussata	species	132361
	Mytilus	genus	16392
recombined as	Modiolus (Modiolus) r	species	87564
	Crenella decussata	species	132361
recombined as	Modiolus (Modiolus) r	species	87564
recombined as	Ischadium recurvum	species	87574
recombined as	Ischadium recurvum	species	87574
	Modiolus	genus	77219
	Modiolus	genus	77219
recombined as	Ischadium recurvum	species	87574
	Modiolus ducatellii	species	158588
recombined as	Modiolus (Modiolus) r	species	87564
	Brachidontes	genus	16347
recombined as	Ischadium recurvum	species	87574
species not entered	Modiolus	genus	77219
	Mytilus edulis	species	87571
species not entered	Modiolus	genus	77219
	Geukensia demissa	species	87569
	Ischadium recurvum	species	87574
species not entered	Modiolus	genus	77219
species not entered	Modiolus	genus	77219
	Ischadium recurvum	species	87574
	Brachiodontes	genus	64227
recombined as	Arcopsis adamsi	species	82689
recombined as	Arcopsis adamsi	species	82689
recombined as	Arcopsis adamsi	species	82689
species not entered	Noetia	genus	16277

recombined as	Arcopsis adamsi	species	82689
species not entered	Noetia	genus	16277
recombined as	Arcopsis adamsi	species	82689
recombined as	Eontia ponderosa	species	87626
recombined as	Arcopsis adamsi	species	82689
species not entered	Noetia	genus	16277
recombined as	Eontia ponderosa	species	87626
recombined as	Arcopsis adamsi	species	82689
recombined as	Noetia incile	species	98540
recombined as	Noetia incile	species	98540
recombined as	Noetia incile	species	98540
	Arcopsis adamsi	species	82689
species not entered	Noetia	genus	16277
recombined as	Eontia ponderosa	species	87626
	Arcopsis adamsi	species	82689
recombined as	Eontia ponderosa	species	87626
	Arcopsis adamsi	species	82689
recombined as	Eontia ponderosa	species	87626
	Arcopsis adamsi	species	82689
species not entered	Eontia	genus	16238
	Arcopsis adamsi	species	82689
	Arcopsis adamsi	species	82689
	Arcopsis adamsi	species	82689
	Arcopsis adamsi	species	82689
species not entered	Eontia	genus	16238
recombined as	Arcopsis adamsi	species	82689
recombined as	Eontia ponderosa	species	87626
recombined as	Eontia ponderosa	species	87626
	Noetia	genus	16277
species not entered	Noetia	genus	16277
species not entered	Noetia	genus	16277
species not entered	Eontia	genus	16238
recombined as	Eontia ponderosa	species	87626
	Arcopsis adamsi	species	82689
	Arcopsis adamsi	species	82689
	Arcopsis adamsi	species	82689
	Arcopsis adamsi	species	82689
	Arcopsis adamsi	species	82689
	Noetia incile	species	98540
	Noetia incile	species	98540
	Noetia incile	species	98540
	Arcopsis adamsi	species	82689
	Arcopsis adamsi	species	82689
	Arcopsis adamsi	species	82689
species not entered	Noetia	genus	16277
recombined as	Eontia ponderosa	species	87626
recombined as	Eontia ponderosa	species	87626

recombined as	Eontia ponderosa	species	87626
recombined as	Eontia ponderosa	species	87626
recombined as	Eontia ponderosa	species	87626
	Noetia	genus	16277
	Arcopsis adamsi	species	82689
recombined as	Eontia ponderosa	species	87626
species not entered	Noetia	genus	16277
	Noetia incile	species	98540
recombined as	Arcopsis adamsi	species	82689
recombined as	Eontia ponderosa	species	87626
recombined as	Eontia ponderosa	species	87626
recombined as	Eontia ponderosa	species	87626
	Arcopsis adamsi	species	82689
species not entered	Noetia (Noetia)	subgenus	90005
recombined as	Eontia ponderosa	species	87626
	Noetia incile	species	98540
recombined as	Eontia ponderosa	species	87626
recombined as	Arcopsis adamsi	species	82689
recombined as	Arcopsis adamsi	species	82689
species not entered	Noetia	genus	16277
species not entered	Noetia	genus	16277
species not entered	Noetia	genus	16277
species not entered	Noetia	genus	16277
recombined as	Eontia ponderosa	species	87626
recombined as	Eontia ponderosa	species	87626
recombined as	Eontia ponderosa	species	87626
recombined as	Eontia ponderosa	species	87626
recombined as	Eontia ponderosa	species	87626
recombined as	Eontia ponderosa	species	87626
recombined as	Eontia ponderosa	species	87626
recombined as	Eontia ponderosa	species	87626
recombined as	Eontia ponderosa	species	87626
species not entered	Noetia (Noetia)	subgenus	90005
species not entered	Noetia	genus	16277
species not entered	Noetia	genus	16277
	Arcopsis adamsi	species	82689
	Noetia	genus	16277
	Noetia	genus	16277
	Noetia	genus	16277
	Noetia	genus	16277
	Arcopsis adamsi	species	82689
	Noetia	genus	16277
	Noetia	genus	16277
	Arcopsis adamsi	species	82689
	Arcopsis adamsi	species	82689
	Noetia	genus	16277
species not entered	Noetia	genus	16277

recombined as	Eontia ponderosa	species	87626
recombined as	Eontia ponderosa	species	87626
recombined as	Eontia ponderosa	species	87626
	Noetia incile	species	98540
	Noetia incile	species	98540
	Noetia incile	species	98540
	Noetia incile	species	98540
	Noetia incile	species	98540
	Noetia incile	species	98540
	Noetia incile	species	98540
	Noetia incile	species	98540
	Noetia incile	species	98540
	Noetia incile	species	98540
	Noetia incile	species	98540
	Noetia incile	species	98540
recombined as	Eontia ponderosa	species	87626
recombined as	Nucinella woodii	species	136166
	Nucinella woodii	species	136166
	Nuculana acuta	species	87521
	Nuculana acuta	species	87521
	Nuculana acuta	species	87521
	Nuculana acuta	species	87521
	Nuculana acuta	species	87521
subjective synonym of, species not entered	Nuculana	genus	16083
subjective synonym of, species not entered	Nuculana	genus	16083
subjective synonym of, species not entered	Nuculana	genus	16083
subjective synonym of, species not entered	Nuculana	genus	16083
subjective synonym of, species not entered	Nuculana	genus	16083
subjective synonym of, species not entered	Nuculana	genus	16083
subjective synonym of, species not entered	Nuculana	genus	16083
	Nuculana acuta	species	87521
	Nuculana acuta	species	87521
	Nuculana acuta	species	87521
	Nuculana acuta	species	87521
	Nuculana	genus	16083
	Nuculana	genus	16083
	Nuculana	genus	16083
	Nuculana	genus	16083
	Nuculana acuta	species	87521
species not entered	Nuculana	genus	16083
	Nuculana acuta	species	87521
	Nuculana acuta	species	87521
recombined as	Nuculana acuta	species	87521
	Nuculana	genus	16083
recombined as	Nuculana acuta	species	87521
species not entered	Saccella	genus	16121
species not entered	Saccella	genus	16121

species not entered	Saccella	genus	16121
species not entered	Saccella	genus	16121
	Nuculana acuta	species	87521
subjective synonym of, species not entered	Nuculana	genus	16083
	Nuculana acuta	species	87521
	Nuculana acuta	species	87521
	Nuculana acuta	species	87521
	Nuculana acuta	species	87521
	Nuculana acuta	species	87521
	Nuculana acuta	species	87521
recombined as	Nuculana acuta	species	87521
subjective synonym of, species not entered	Nuculana	genus	16083
recombined as	Nuculana acuta	species	87521
subjective synonym of, species not entered	Nuculana	genus	16083
	Nuculana acuta	species	87521
	Nuculana acuta	species	87521
	Nuculana acuta	species	87521
	Nuculana acuta	species	87521
	Nuculana acuta	species	87521
	Nuculana acuta	species	87521
	Nuculana acuta	species	87521
	Nuculana acuta	species	87521
	Nuculana acuta	species	87521
	Nuculana acuta	species	87521
	Nuculana acuta	species	87521
recombined as	Nuculana acuta	species	87521
	Nuculana acuta	species	87521
recombined as	Nuculana acuta	species	87521
recombined as	Nuculana acuta	species	87521
recombined as	Nuculana acuta	species	87521
recombined as	Nuculana acuta	species	87521
recombined as	Nuculana acuta	species	87521
	Saccella	genus	16121
	Saccella	genus	16121
	Nuculana acuta	species	87521
	Nuculana acuta	species	87521
	Nuculana acuta	species	87521
	Nuculana acuta	species	87521
	Nuculana acuta	species	87521
	Nuculana acuta	species	87521
	Nuculana acuta	species	87521
	Nuculana acuta	species	87521
	Nuculana acuta	species	87521
	Nuculana acuta	species	87521
	Nuculana acuta	species	87521
	Nuculana acuta	species	87521
	Nuculana acuta	species	87521

	Nuculana acuta	species	87521
	Nuculana acuta	species	87521
recombined as	Nucula (Nucula) proxi	species	87510
recombined as	Nucula (Nucula) proxi	species	87510
recombined as	Nucula (Nucula) proxi	species	87510
recombined as	Nucula (Nucula) proxi	species	87510
recombined as	Nucula (Nucula) proxi	species	87510
recombined as	Nucula (Nucula) proxi	species	87510
recombined as	Nucula (Nucula) proxi	species	87510
recombined as	Nucula (Nucula) proxi	species	87510
	Nucula taphria	species	98558
	Nucula taphria	species	98558
recombined as	Nucula (Nucula) proxi	species	87510
recombined as	Nucula (Nucula) proxi	species	87510
recombined as	Nucula (Nucula) proxi	species	87510
recombined as	Nucula (Nucula) proxi	species	87510
recombined as	Nucula (Nucula) proxi	species	87510
recombined as	Nucula (Nucula) proxi	species	87510
recombined as	Nucula (Nucula) proxi	species	87510
recombined as	Nucula (Nucula) proxi	species	87510
	Nucula	genus	16082
recombined as	Nucula (Nucula) proxi	species	87510
recombined as	Nucula (Nucula) proxi	species	87510
recombined as	Nucula (Nucula) proxi	species	87510
recombined as	Nucula (Nucula) proxi	species	87510
	Nucula taphria	species	98558
recombined as	Nucula (Nucula) proxi	species	87510
recombined as	Nucula (Nucula) proxi	species	87510
recombined as	Nucula (Nucula) proxi	species	87510
recombined as	Nucula (Nucula) proxi	species	87510
recombined as	Nucula (Nucula) proxi	species	87510
recombined as	Nucula (Nucula) proxi	species	87510
recombined as	Nucula (Nucula) proxi	species	87510
recombined as	Nucula (Nucula) proxi	species	87510
recombined as	Nucula (Nucula) proxi	species	87510
recombined as	Nucula (Nucula) proxi	species	87510
recombined as	Nucula (Nucula) proxi	species	87510
recombined as	Nucula (Nucula) proxi	species	87510
recombined as	Nucula (Nucula) proxi	species	87510
recombined as	Nucula (Nucula) proxi	species	87510
recombined as	Nucula (Nucula) proxi	species	87510
recombined as	Nucula (Nucula) proxi	species	87510
recombined as	Nucula (Nucula) proxi	species	87510
recombined as	Nucula (Nucula) proxi	species	87510
recombined as	Nucula (Nucula) proxi	species	87510
recombined as	Nucula (Nucula) proxi	species	87510
recombined as	Nucula (Nucula) proxi	species	87510

	Nucula taphria	species	98558
recombined as	Nucula (Nucula) proxi	species	87510
recombined as	Nucula (Nucula) proxi	species	87510
	Nucula taphria	species	98558
recombined as	Nucula (Nucula) proxi	species	87510
recombined as	Nucula (Nucula) proxi	species	87510
recombined as	Nucula (Nucula) proxi	species	87510
recombined as	Nucula (Nucula) proxi	species	87510
recombined as	Nucula (Nucula) proxi	species	87510
recombined as	Nucula (Nucula) proxi	species	87510
recombined as	Nucula (Nucula) proxi	species	87510
recombined as	Nucula (Nucula) proxi	species	87510
recombined as	Nucula (Nucula) proxi	species	87510
recombined as	Nucula (Nucula) proxi	species	87510
recombined as	Nucula (Nucula) proxi	species	87510
recombined as	Nucula (Nucula) proxi	species	87510
recombined as	Nucula (Nucula) proxi	species	87510
recombined as	Nucula (Nucula) proxi	species	87510
recombined as	Nucula (Nucula) proxi	species	87510
recombined as	Nucula (Nucula) proxi	species	87510
recombined as	Nucula (Nucula) proxi	species	87510
recombined as	Nucula (Nucula) proxi	species	87510
recombined as	Nucula (Nucula) proxi	species	87510
species not entered	Nucula	genus	16082
recombined as	Nucula (Nucula) proxi	species	87510
recombined as	Nucula (Nucula) proxi	species	87510
recombined as	Nucula (Nucula) proxi	species	87510
recombined as	Nucula (Nucula) proxi	species	87510
recombined as	Nucula (Nucula) proxi	species	87510
recombined as	Nucula (Nucula) proxi	species	87510
recombined as	Nucula (Nucula) proxi	species	87510
recombined as	Nucula (Nucula) proxi	species	87510
recombined as	Nucula (Nucula) proxi	species	87510
recombined as	Nucula (Nucula) proxi	species	87510
recombined as	Nucula (Nucula) proxi	species	87510
recombined as	Nucula (Nucula) proxi	species	87510
recombined as	Nucula (Nucula) proxi	species	87510
recombined as	Nucula (Nucula) proxi	species	87510
recombined as	Nucula (Nucula) proxi	species	87510
recombined as	Conradostrea sculptui	species	109463
	Ostrea	genus	16870
	Ostrea	genus	16870
species not entered	Ostrea	genus	16870
species not entered	Ostrea	genus	16870
	Crassostrea virginica	species	82698
recombined as	Conradostrea sculptui	species	109463
recombined as	Conradostrea sculptui	species	109463

	Crassostrea virginica	species	82698
recombined as	Conradostrea sculptur	species	109463
	Crassostrea virginica	species	82698
recombined as	Conradostrea sculptur	species	109463
	Ostrea	genus	16870
	Crassostrea virginica	species	82698
species not entered	Ostrea	genus	16870
recombined as	Conradostrea sculptur	species	109463
obsolete variant of	Ostrea	genus	16870
	Ostrea	genus	16870
species not entered	Ostrea	genus	16870
species not entered	Ostrea	genus	16870
species not entered	Ostrea	genus	16870
species not entered	Ostrea	genus	16870
	Crassostrea virginica	species	82698
recombined as	Conradostrea sculptur	species	109463
recombined as	Conradostrea sculptur	species	109463
	Ostrea equestris	species	101121
recombined as	Conradostrea sculptur	species	109463
recombined as	Crassostrea virginica	species	82698
	Ostrea	genus	16870
species not entered	Ostrea	genus	16870
species not entered	Ostrea	genus	16870
species not entered	Ostrea	genus	16870
species not entered	Ostrea	genus	16870
species not entered	Ostrea	genus	16870
species not entered	Ostrea	genus	16870
species not entered	Ostrea	genus	16870
recombined as	Conradostrea sculptur	species	109463
recombined as	Conradostrea sculptur	species	109463
	Ostrea	genus	16870
	Ostrea	genus	16870
recombined as	Conradostrea sculptur	species	109463
species not entered	Ostrea	genus	16870
	Ostrea	genus	16870
recombined as	Conradostrea sculptur	species	109463
recombined as	Conradostrea sculptur	species	109463
recombined as	Conradostrea sculptur	species	109463
recombined as	Conradostrea sculptur	species	109463
	Ostrea	genus	16870
species not entered	Ostrea (Ostrea)	subgenus	94289
recombined as	Conradostrea sculptur	species	109463
species not entered	Ostrea (Ostrea)	subgenus	94289
	Ostrea	genus	16870
recombined as	Crassostrea virginica	species	82698
species not entered	Ostrea (Ostrea)	subgenus	94289
recombined as	Conradostrea sculptur	species	109463

species not entered	Ostrea (Ostrea)	subgenus	94289
	Crassostrea virginica	species	82698
	Conradostrea lawrenc	species	82701
	Conradostrea sculptur	species	109463
recombined as	Conradostrea sculptur	species	109463
species not entered	Ostrea	genus	16870
species not entered	Ostrea	genus	16870
species not entered	Ostrea	genus	16870
species not entered	Ostrea	genus	16870
species not entered	Ostrea	genus	16870
species not entered	Crassostrea	genus	16578
species not entered	Conradostrea	genus	16563
obsolete variant of	Ostrea (Ostrea) subdig	species	109883
	Ostrea	genus	16870
	Ostrea compressirostr	species	99271
	Ostrea	genus	16870
	Crassostrea	genus	16578
obsolete variant of	Ostrea (Ostrea) subdig	species	109883
obsolete variant of	Ostrea (Ostrea) subdig	species	109883
	Crassostrea virginica	species	82698
species not entered	Ostrea	genus	16870
	Conradostrea sculptur	species	109463
species not entered	Ostrea	genus	16870
	Conradostrea sculptur	species	109463
	Ostrea	genus	16870
	Ostrea	genus	16870
	Crassostrea virginica	species	82698
species not entered	Ostrea	genus	16870
	Crassostrea virginica	species	82698
species not entered	Ostrea	genus	16870
recombined as	Conradostrea sculptur	species	109463
species not entered	Ostrea	genus	16870
	Crassostrea virginica	species	82698
	Ostrea equestris	species	101121
	Ostrea	genus	16870
	Crassostrea virginica	species	82698
	Crassostrea virginica	species	82698
	Crassostrea virginica	species	82698
	Crassostrea virginica	species	82698
recombined as	Conradostrea sculptur	species	109463
recombined as	Conradostrea sculptur	species	109463
recombined as	Conradostrea sculptur	species	109463
species not entered	Ostrea	genus	16870
recombined as	Conradostrea sculptur	species	109463
recombined as	Conradostrea sculptur	species	109463
	Crassostrea virginica	species	82698
	Crassostrea virginica	species	82698

	Crassostrea virginica	species	82698
	Crassostrea virginica	species	82698
recombined as	Conradostrea sculptu	species	109463
	Crassostrea virginica	species	82698
recombined as	Conradostrea sculptu	species	109463
species not entered	Ostrea	genus	16870
recombined as	Conradostrea sculptu	species	109463
species not entered	Ostrea	genus	16870
recombined as	Conradostrea sculptu	species	109463
species not entered	Ostrea	genus	16870
species not entered	Ostrea	genus	16870
	Crassostrea virginica	species	82698
recombined as	Conradostrea sculptu	species	109463
	Crassostrea virginica	species	82698
	Crassostrea virginica	species	82698
	Crassostrea virginica	species	82698
	Crassostrea virginica	species	82698
	Conradostrea	genus	16563
	Crassostrea virginica	species	82698
	Ostrea	genus	16870
	Conradostrea	genus	16563
	Crassostrea virginica	species	82698
recombined as	Crassostrea virginica	species	82698
recombined as	Crassostrea virginica	species	82698
recombined as	Crassostrea virginica	species	82698
recombined as	Conradostrea sculptu	species	109463
recombined as	Conradostrea sculptu	species	109463
	Ostrea	genus	16870
	Ostrea	genus	16870
species not entered	Ostrea	genus	16870
recombined as	Crassostrea virginica	species	82698
recombined as	Crassostrea virginica	species	82698
	Conradostrea lawrenc	species	82701
	Conradostrea lawrenc	species	82701
	Conradostrea lawrenc	species	82701
	Conradostrea lawrenc	species	82701
	Crassostrea virginica	species	82698
	Ostrea compressirost	species	99271
	Ostrea compressirost	species	99271
	Conradostrea sculptu	species	109463
species not entered	Ostrea	genus	16870
species not entered	Ostrea	genus	16870
recombined as	Conradostrea sculptu	species	109463
recombined as	Conradostrea sculptu	species	109463
recombined as	Conradostrea sculptu	species	109463
recombined as	Conradostrea sculptu	species	109463
	Crassostrea virginica	species	82698

	Crassostrea virginica	species	82698
	Crassostrea virginica	species	82698
	Crassostrea virginica	species	82698
	Crassostrea virginica	species	82698
	Crassostrea virginica	species	82698
	Crassostrea virginica	species	82698
	Crassostrea virginica	species	82698
	Crassostrea virginica	species	82698
species not entered	Ostrea	genus	16870
	Conradostrea sculptur	species	109463
	Crassostrea virginica	species	82698
	Ostrea	genus	16870
recombined as	Conradostrea sculptur	species	109463
	Conradostrea	genus	16563
	Crassostrea virginica	species	82698
	Conradostrea	genus	16563
	Conradostrea	genus	16563
	Conradostrea sculptur	species	109463
species not entered	Ostrea	genus	16870
	Conradostrea	genus	16563
	Conradostrea	genus	16563
	Conradostrea	genus	16563
	Conradostrea	genus	16563
	Conradostrea	genus	16563
	Crassostrea virginica	species	82698
	Ostrea	genus	16870
	Crassostrea virginica	species	82698
	Crassostrea virginica	species	82698
	Ostrea	genus	16870
	Crassostrea virginica	species	82698
	Ostrea	genus	16870
recombined as	Conradostrea sculptur	species	109463
recombined as	Conradostrea sculptur	species	109463
recombined as	Conradostrea sculptur	species	109463
recombined as	Conradostrea sculptur	species	109463
	Crassostrea virginica	species	82698
	Crassostrea virginica	species	82698
	Crassostrea virginica	species	82698
	Crassostrea virginica	species	82698
	Crassostrea virginica	species	82698
	Crassostrea virginica	species	82698
	Ostrea compressirostr	species	99271
	Ostrea compressirostr	species	99271
	Conradostrea sculptur	species	109463
recombined as	Conradostrea sculptur	species	109463
	Crassostrea virginica	species	82698
	Crassostrea virginica	species	82698

	Ostrea compressirostı	species	99271
	Ostrea compressirostı	species	99271
	Conradostrea sculptuı	species	109463
	Ostrea compressirostı	species	99271
	Conradostrea sculptuı	species	109463
	Conradostrea sculptuı	species	109463
	Conradostrea sculptuı	species	109463
	Ostrea compressirostı	species	99271
	Conradostrea sculptuı	species	109463
	Conradostrea sculptuı	species	109463
	Conradostrea sculptuı	species	109463
	Ostrea raveneliana	species	160470
	Conradostrea sculptuı	species	109463
	Conradostrea sculptuı	species	109463
	Conradostrea sculptuı	species	109463
	Conradostrea sculptuı	species	109463
	Conradostrea sculptuı	species	109463
	Conradostrea sculptuı	species	109463
	Conradostrea sculptuı	species	109463
	Conradostrea sculptuı	species	109463
	Conradostrea	genus	16563
	Ostrea	genus	16870
	Ostrea	genus	16870
	Crassostrea virginica	species	82698
	Ostrea	genus	16870
recombined as	Pandora (Clidiophora)	species	98486
recombined as	Pandora (Pandorella)	species	162224
species not entered	Pandora	genus	18783
recombined as	Pandora (Pandora) tril	species	108687
species not entered	Pandora	genus	18783
	Pandora (Clidiophora)	species	98486
obsolete variant of	Clidiophora	genus	18720
	Pandora	genus	18783
recombined as	Pandora (Pandora) tril	species	108687
misspelling of, recombined as	Pandora (Pandora) tril	species	108687
recombined as	Pandora (Pandora) tril	species	108687
species not entered	Pandora	genus	18783
species not entered	Pandora	genus	18783
species not entered	Pandora	genus	18783
subjective synonym of, species not entered	Pandora (Pandorella)	subgenus	18784
subjective synonym of, species not entered	Pandora (Pandorella)	subgenus	18784
	Pandora (Clidiophora)	species	98486
species not entered	Clidiophora	genus	18720
misspelling of, recombined as	Pandora (Pandora) tril	species	108687
misspelling of, recombined as	Pandora (Pandora) tril	species	108687
recombined as	Pandora (Pandora) tril	species	108687
recombined as	Pandora (Pandora) tril	species	108687

recombined as	Pandora (Pandorella)	species	162224
species not entered	Pandora (Pandora)	subgenus	90384
species not entered	Pandora	genus	18783
	Pandora	genus	18783
species not entered	Clidiophora	genus	18720
species not entered	Clidiophora	genus	18720
recombined as	Pandora (Pandora) go	species	108685
recombined as	Pandora (Pandora) tril	species	108687
recombined as	Pandora (Pandora) go	species	108685
recombined as	Pandora (Pandora) go	species	108685
recombined as	Pandora (Pandora) tril	species	108687
recombined as	Pandora (Pandorella)	species	162224
recombined as	Pandora (Clidiophora)	species	98486
recombined as	Pandora (Pandorella)	species	162224
recombined as	Pandora (Pandorella)	species	162224
recombined as	Pandora (Pandorella)	species	162224
recombined as	Pandora (Clidiophora)	species	98486
recombined as	Pandora (Clidiophora)	species	98486
obsolete variant of	Pandora	genus	18783
recombined as	Argopecten gibbus	species	109885
subjective synonym of, species not entered	Argopecten	genus	16490
recombined as	Lyropecten pittieri	species	102113
	Pecten (Pecten)	subgenus	85852
subjective synonym of, species not entered	Argopecten	genus	16490
recombined as	Lyropecten pittieri	species	102113
recombined as	Lyropecten tamiamiem	species	416602
subjective synonym of, species not entered	Argopecten	genus	16490
recombined as	Lyropecten pittieri	species	102113
	Pecten (Pecten)	subgenus	85852
subjective synonym of, species not entered	Argopecten	genus	16490
subjective synonym of, species not entered	Argopecten	genus	16490
recombined as	Lyropecten tamiamiem	species	416602
species not entered	Chlamys	genus	16549
	Amusium mortoni	species	95150
recombined as	Argopecten anteampl	species	109886
recombined as	Aequipecten comparil	species	95130
subjective synonym of	Lindapecten muscosus	species	115506
	Chlamys	genus	16549
recombined as	Euvola ziczac	species	87034
	Amusium mortoni	species	95150
	Chlamys	genus	16549
recombined as	Argopecten anteampl	species	109886
recombined as	Chlamys (Nodipecten)	species	109896
subjective synonym of	Lindapecten muscosus	species	115506
recombined as	Nodipecten nodosus	species	87175
	Chlamys	genus	16549
subjective synonym of	Lindapecten muscosus	species	115506

recombined as	Argopecten irradians	species	87551
	Amusium mortoni	species	95150
recombined as	Argopecten anteampl	species	109886
	Chlamys	genus	16549
recombined as	Aequipecten comparil	species	95130
recombined as	Argopecten irradians	species	87551
recombined as	Chesapecten jeffersor	species	82641
obsolete variant of	Chlamys	genus	16549
recombined as	Euvola raveneli	species	95096
species not entered	Pecten (Pecten)	subgenus	85852
recombined as	Carolinapecten ebore	species	95568
recombined as	Carolinapecten ebore	species	95568
recombined as	Carolinapecten ebore	species	95568
recombined as	Carolinapecten ebore	species	95568
recombined as	Carolinapecten ebore	species	95568
recombined as	Aequipecten comparil	species	95130
recombined as	Aequipecten comparil	species	95130
recombined as	Aequipecten comparil	species	95130
recombined as	Aequipecten comparil	species	95130
recombined as	Aequipecten comparil	species	95130
	Amusium mortoni	species	95150
species not entered	Pecten (Pecten)	subgenus	85852
recombined as	Aequipecten comparil	species	95130
	Amusium mortoni	species	95150
	Argopecten	genus	16490
	Argopecten irradians	species	87551
	Aequipecten	genus	16446
	Argopecten irradians	species	87551
recombined as	Euvola ziczac	species	87034
recombined as	Euvola raveneli	species	95096
recombined as	Nodipecten nodosus	species	87175
recombined as	Antillipecten antillaru	species	91147
recombined as	Lindapecten muscosu	species	115506
recombined as	Argopecten gibbus	species	109885
species not entered	Aequipecten	genus	16446
species not entered	Aequipecten	genus	16446
species not entered	Pecten (Pecten)	subgenus	85852
recombined as	Nodipecten nodosus	species	87175
	Pecten (Chlamys) calo	species	89174
	Pecten (Chlamys) ebo	species	82959
	Pecten (Chlamys) calo	species	89174
	Pecten (Chlamys) ebo	species	82959
	Pecten (Chlamys) ebo	species	82959
	Pecten (Chlamys) ebo	species	82959
	Pecten (Chlamys) ebo	species	82959
species not entered	Pecten (Pecten)	subgenus	85852
recombined as	Carolinapecten ebore	species	95568

obsolete variant of	Pecten (Chlamys) calo	species	89174
	Argopecten	genus	16490
	Pecten	genus	16904
	Pecten	genus	16904
recombined as	Carolinapecten ebore	species	95568
recombined as	Carolinapecten ebore	species	95568
recombined as	Carolinapecten ebore	species	95568
obsolete variant of	Pecten	genus	16904
recombined as	Carolinapecten ebore	species	95568
	Pecten	genus	16904
	Pecten	genus	16904
recombined as	Argopecten anteampl	species	109886
subjective synonym of, species not entered	Argopecten	genus	16490
recombined as	Aequipecten comparil	species	95130
recombined as	Aequipecten comparil	species	95130
subjective synonym of, species not entered	Argopecten	genus	16490
recombined as	Aequipecten comparil	species	95130
recombined as	Argopecten anteampl	species	109886
recombined as	Aequipecten comparil	species	95130
subjective synonym of	Lindapecten muscosu	species	115506
recombined as	Argopecten anteampl	species	109886
subjective synonym of	Lindapecten muscosu	species	115506
recombined as	Euvola ziczac	species	87034
	Argopecten irradians	species	87551
	Euvola ziczac	species	87034
species not entered	Nodipecten	genus	16853
species not entered	Carolinapecten	genus	16539
	Carolinapecten solaro	species	82301
	Carolinapecten jamiea	species	119961
recombined as	Carolinapecten ebore	species	95568
species not entered	Pecten (Pecten)	subgenus	85852
subjective synonym of, species not entered	Argopecten	genus	16490
subjective synonym of, species not entered	Argopecten	genus	16490
species not entered	Nodipecten	genus	16853
species not entered	Lyropecten	genus	16786
species not entered	Pecten	genus	16904
	Pecten wendelli	species	160239
obsolete variant of	Pecten (Chlamys) calo	species	89174
recombined as	Nodipecten nodosus	species	87175
species not entered	Leptopecten	genus	16762
species not entered	Lindapecten	genus	97218
species not entered	Stralopecten	genus	17052
species not entered	Argopecten	genus	16490
	Carolinapecten solaro	species	82301
species not entered	Carolinapecten	genus	16539
species not entered	Nodipecten	genus	16853
species not entered	Stralopecten	genus	17052

	Carolinapecten solaro	species	82301
	Carolinapecten solaro	species	82301
	Carolinapecten ebore	species	95568
species not entered	Argopecten	genus	16490
species not entered	Carolinapecten	genus	16539
	Chesapecten jefferso	species	82641
species not entered	Chesapecten	genus	16548
	Euvola smithi	species	85853
species not entered	Leptopecten	genus	16762
	Chesapecten jefferso	species	82641
species not entered	Carolinapecten	genus	16539
species not entered	Nodipecten	genus	16853
species not entered	Euvola	genus	16652
species not entered	Argopecten	genus	16490
species not entered	Nodipecten	genus	16853
species not entered	Euvola	genus	16652
species not entered	Argopecten	genus	16490
species not entered	Chesapecten (Christin	subgenus	16551
	Carolinapecten ebore	species	95568
species not entered	Carolinapecten	genus	16539
recombined as	Argopecten gibbus	species	109885
	Chlamys	genus	16549
subjective synonym of	Lindapecten muscosu	species	115506
	Aequipecten	genus	16446
	Argopecten	genus	16490
	Aequipecten	genus	16446
corrected to	Amusium	genus	16466
	Argopecten	genus	16490
species not entered	Amusium	genus	16466
species not entered	Aequipecten	genus	16446
species not entered	Pecten	genus	16904
species not entered	Aequipecten	genus	16446
recombined as	Euvola ziczac	species	87034
	Argopecten	genus	16490
	Aequipecten	genus	16446
species not entered	Aequipecten	genus	16446
subjective synonym of	Lindapecten muscosu	species	115506
subjective synonym of	Lindapecten muscosu	species	115506
recombined as	Argopecten anteampl	species	109886
subjective synonym of	Lindapecten muscosu	species	115506
	Pecten	genus	16904
recombined as	Carolinapecten ebore	species	95568
	Pecten	genus	16904
recombined as	Carolinapecten ebore	species	95568
subjective synonym of, species not entered	Argopecten	genus	16490
recombined as	Carolinapecten ebore	species	95568
recombined as	Aequipecten comparil	species	95130

	Spathochlamys vaginu	species	100388
	Spathochlamys vaginu	species	100388
species not entered	Nodipecten	genus	16853
species not entered	Nodipecten	genus	16853
	Antillipecten antillaru	species	91147
	Argopecten gibbus	species	109885
	Argopecten irradians	species	87551
	Euvola ziczac	species	87034
recombined as	Chesapecten jefferson	subspecies	82643
	Chesapecten madison	species	82645
	Placopecten magellan	species	82637
recombined as	Carolinapecten ebore	species	95568
recombined as	Carolinapecten ebore	species	95568
	Chlamys	genus	16549
	Amusium mortoni	species	95150
recombined as	Carolinapecten ebore	species	95568
	Chlamys	genus	16549
	Chlamys	genus	16549
recombined as	Carolinapecten ebore	species	95568
	Chlamys	genus	16549
recombined as	Argopecten gibbus	species	109885
recombined as	Argopecten gibbus	species	109885
	Amusium mortoni	species	95150
recombined as	Carolinapecten ebore	species	95568
	Chlamys	genus	16549
recombined as	Carolinapecten ebore	species	95568
	Chlamys	genus	16549
	Chesapecten jefferson	species	82641
species not entered	Leptopecten	genus	16762
	Chesapecten madison	species	82645
recombined as	Chesapecten jefferson	subspecies	82643
recombined as	Chesapecten jefferson	subspecies	82643
	Pecten hemicyclicus	species	416604
recombined as	Chesapecten jefferson	subspecies	82643
	Pecten hemicyclicus	species	416604
	Pecten hemicyclicus	species	416604
species not entered	Leptopecten	genus	16762
	Chesapecten madison	species	82645
recombined as	Chesapecten jefferson	subspecies	82643
species not entered	Nodipecten	genus	16853
recombined as	Chesapecten jefferson	subspecies	82643
	Pecten hemicyclicus	species	416604
	Pecten	genus	16904
	Amusium mortoni	species	95150
	Carolinapecten ebore	species	95568
species not entered	Argopecten	genus	16490
	Chlamys decemnarius	species	82639

	Carolinapecten ebore	species	95568
	Amusium mortoni	species	95150
recombined as	Carolinapecten ebore	species	95568
recombined as	Argopecten irradians	species	87551
	Argopecten gibbus	species	109885
	Carolinapecten ebore	species	95568
	Carolinapecten ebore	species	95568
recombined as	Carolinapecten ebore	species	95568
recombined as	Carolinapecten ebore	species	95568
	Amusium mortoni	species	95150
recombined as	Carolinapecten ebore	species	95568
	Pecten hemicyclicus	species	416604
	Amusium mortoni	species	95150
recombined as	Chesapecten jeffersor	species	82641
	Leptopecten auroraer	species	82693
	Carolinapecten ebore	species	95568
	Leptopecten auroraer	species	82693
	Carolinapecten ebore	species	95568
	Carolinapecten ebore	species	95568
	Leptopecten auroraer	species	82693
	Leptopecten auroraer	species	82693
	Placopecten clintoniu	species	82303
	Chesapecten jeffersor	species	82641
	Chesapecten madison	species	82645
recombined as	Euvola raveneli	species	95096
recombined as	Argopecten gibbus	species	109885
recombined as	Argopecten gibbus	species	109885
recombined as	Chesapecten jeffersor	species	82641
recombined as	Chesapecten jeffersor	species	82641
	Chlamys (Lyropecten)	species	95122
	Chlamys (Lyropecten)	species	95122
recombined as	Carolinapecten ebore	species	95568
recombined as	Carolinapecten ebore	species	95568
recombined as	Carolinapecten ebore	species	95568
recombined as	Carolinapecten ebore	species	95568
recombined as	Carolinapecten ebore	species	95568
recombined as	Carolinapecten ebore	species	95568
recombined as	Carolinapecten ebore	species	95568
recombined as	Carolinapecten ebore	species	95568
recombined as	Carolinapecten ebore	species	95568
recombined as	Carolinapecten ebore	species	95568
recombined as	Carolinapecten ebore	species	95568
recombined as	Carolinapecten ebore	species	95568
recombined as	Carolinapecten ebore	species	95568
recombined as	Carolinapecten ebore	species	95568
	Amusium mortoni	species	95150
	Amusium mortoni	species	95150

	Amusium mortoni	species	95150
recombined as	Chlamys (Lyropecten)	species	95122
	Amusium mortoni	species	95150
recombined as	Carolinapecten ebore	species	95568
recombined as	Carolinapecten ebore	species	95568
recombined as	Carolinapecten ebore	species	95568
recombined as	Carolinapecten ebore	species	95568
recombined as	Carolinapecten ebore	species	95568
recombined as	Carolinapecten ebore	species	95568
recombined as	Carolinapecten ebore	species	95568
recombined as	Carolinapecten ebore	species	95568
	Placopecten clintoniu	species	82303
	Placopecten magellan	species	82637
species not entered	Chlamys	genus	16549
	Chesapecten jeffersor	species	82641
	Chesapecten madison	species	82645
recombined as	Argopecten irradians	species	87551
recombined as	Argopecten irradians	species	87551
recombined as	Argopecten irradians	species	87551
recombined as	Argopecten irradians	species	87551
	Argopecten gibbus	species	109885
recombined as	Carolinapecten ebore	species	95568
recombined as	Carolinapecten ebore	species	95568
species not entered	Chlamys	genus	16549
	Argopecten irradians	species	87551
recombined as	Carolinapecten ebore	species	95568
recombined as	Carolinapecten ebore	species	95568
recombined as	Carolinapecten ebore	species	95568
species not entered	Aequipecten	genus	16446
	Carolinapecten ebore	species	95568
	Carolinapecten ebore	species	95568
recombined as	Amusium mortoni	species	95150
recombined as	Carolinapecten ebore	species	95568
	Amusium mortoni	species	95150
species not entered	Argopecten	genus	16490
	Chesapecten jeffersor	species	82641
	Carolinapecten ebore	species	95568
	Argopecten gibbus	species	109885
	Argopecten gibbus	species	109885
	Chesapecten jeffersor	species	82641
species not entered	Chesapecten	genus	16548
	Chesapecten madison	species	82645
recombined as	Placopecten clintoniu	species	82303
recombined as	Chesapecten jeffersor	species	82641
recombined as	Carolinapecten ebore	species	95568
recombined as	Chlamys santamaria	species	99250
recombined as	Chesapecten madison	species	82645

	Chesapecten madison	species	82645
	Chesapecten madison	species	82645
	Chesapecten madison	species	82645
recombined as	Carolinapecten ebore	species	95568
recombined as	Carolinapecten ebore	species	95568
	Placopecten clintonius	species	82303
	Chesapecten jefferson	species	82641
	Chesapecten madison	species	82645
	Placopecten clintonius	species	82303
	Chesapecten jefferson	species	82641
	Chesapecten madison	species	82645
	Chesapecten jefferson	species	82641
species not entered	Chesapecten	genus	16548
	Chesapecten madison	species	82645
	Chesapecten madison	species	82645
	Carolinapecten ebore	species	95568
	Chesapecten madison	species	82645
	Chesapecten madison	species	82645
	Chesapecten madison	species	82645
	Chesapecten jefferson	species	82641
	Chesapecten jefferson	species	82641
	Placopecten clintonius	species	82303
species not entered	Chlamys	genus	16549
	Chesapecten madison	species	82645
	Chesapecten jefferson	species	82641
	Placopecten clintonius	species	82303
	Chesapecten madison	species	82645
	Chesapecten jefferson	species	82641
species not entered	Chlamys	genus	16549
	Placopecten clintonius	species	82303
	Carolinapecten ebore	species	95568
	Placopecten clintonius	species	82303
	Euvola	genus	16652
	Chesapecten coccyme	species	82647
species not entered	Chlamys	genus	16549
	Chesapecten madison	species	82645
	Chesapecten jefferson	species	82641
	Chesapecten jefferson	species	82641
species not entered	Chesapecten	genus	16548
species not entered	Periploma	genus	18791
	Cochlodesma emmon	species	82736
recombined as	Aperiploma lena	species	156453
subjective synonym of	Choristodon robustus	species	91171
recombined as	Petricola lapicida	species	158759
obsolete variant of	Petricola (Petricoxeni	species	158757
	Petricola (Petricoxeni	species	158757
species not entered	Rupellaria	genus	18205

recombined as	Petricolaria pholadifoı	species	61694
recombined as	Petricolaria pholadifoı	species	61694
recombined as	Petricolaria pholadifoı	species	61694
recombined as	Petricolaria pholadifoı	species	61694
recombined as	Petricolaria pholadifoı	species	61694
subjective synonym of	Petricolaria pholadifoı	species	61694
subjective synonym of	Choristodon robustus	species	91171
	Petricola pectorosa	species	82727
obsolete variant of	Petricola (Claudiconch	species	85862
obsolete variant of	Petricola (Claudiconch	species	85862
recombined as	Petricolaria pholadifoı	species	61694
recombined as	Petricolaria pholadifoı	species	61694
species not entered	Petricola (Petricolaria)	subgenus	18057
species not entered	Petricola (Petricolaria)	subgenus	18057
recombined as	Petricolaria pholadifoı	species	61694
recombined as	Petricolaria pholadifoı	species	61694
recombined as	Petricolaria pholadifoı	species	61694
recombined as	Petricolaria pholadifoı	species	61694
species not entered	Pleiorytis	genus	18086
species not entered	Pleiorytis	genus	18086
species not entered	Pleiorytis	genus	18086
species not entered	Pleiorytis	genus	18086
species not entered	Pleiorytis	genus	18086
species not entered	Pleiorytis	genus	18086
	Ensis minor	species	158641
	Ensis minor	species	158641
	Ensis directus	species	94527
	Ensis directus	species	94527
	Ensis directus	species	94527
	Ensis directus	species	94527
	Ensis directus	species	94527
	Ensis directus	species	94527
	Ensis minor	species	158641
	Ensis minor	species	158641
	Ensis directus	species	94527
	Ensis directus	species	94527
	Ensis directus	species	94527
	Ensis directus	species	94527
	Ensis directus	species	94527
	Ensis directus	species	94527
	Ensis directus	species	94527
	Ensis directus	species	94527
	Ensis directus	species	94527
	Ensis directus	species	94527
	Ensis directus	species	94527
	Ensis directus	species	94527
	Ensis directus	species	94527

	Ensis directus	species	94527
species not entered	Siliqua	genus	18250
	Ensis directus	species	94527
	Ensis directus	species	94527
	Ensis directus	species	94527
	Ensis directus	species	94527
	Ensis directus	species	94527
	Ensis directus	species	94527
	Ensis directus	species	94527
	Ensis directus	species	94527
	Ensis directus	species	94527
	Ensis directus	species	94527
	Ensis directus	species	94527
	Ensis directus	species	94527
	Ensis	genus	17612
	Ensis directus	species	94527
	Ensis directus	species	94527
	Ensis directus	species	94527
	Ensis directus	species	94527
	Ensis directus	species	94527
	Ensis directus	species	94527
	Ensis directus	species	94527
	Ensis directus	species	94527
species not entered	Ensis	genus	17612
	Ensis directus	species	94527
	Ensis directus	species	94527
	Ensis directus	species	94527
	Ensis directus	species	94527
	Ensis directus	species	94527
	Ensis directus	species	94527
	Ensis directus	species	94527
	Ensis ensiformis	species	98318
	Ensis ensiformis	species	98318
	Ensis ensiformis	species	98318
	Ensis directus	species	94527
	Diplothyra	genus	158643
recombined as	Cyrtopleura costata	species	156794
	Cyrtopleura costata	species	156794
	Cyrtopleura costata	species	156794
	Cyrtopleura costata	species	156794
	Martesia striata	species	117548
species not entered	Teredina	genus	18508
species not entered	Teredina	genus	18508
species not entered	Barnea	genus	18431
species not entered	Barnea	genus	18431
species not entered	Barnea	genus	18431
recombined as	Pholas (Thovana) cam	species	109672

recombined as	Barnea truncata	species	108683
	Martesia cuneiformis	species	392370
	Martesia cuneiformis	species	392370
recombined as	Pholas (Thovana) cam	species	109672
species not entered	Pholas	genus	18491
	Cyrtopleura	genus	18448
species not entered	Barnea	genus	18431
species not entered	Barnea	genus	18431
species not entered	Barnea	genus	18431
species not entered	Barnea	genus	18431
species not entered	Barnea	genus	18431
species not entered	Barnea	genus	18431
	Cyrtopleura	genus	18448
species not entered	Barnea	genus	18431
	Barnea truncata	species	108683
	Barnea truncata	species	108683
	Barnea truncata	species	108683
	Cyrtopleura costata	species	156794
species not entered	Cyrtopleura	genus	18448
replaced by, species not entered	Elegantarca	genus	89497
	Pinna	genus	16400
	Pinna	genus	16400
species not entered	Atrina	genus	16342
species not entered	Atrina	genus	16342
	Atrina	genus	16342
	Atrina	genus	16342
	Atrina	genus	16342
	Pinna	genus	16400
	Atrina	genus	16342
	Pinna	genus	16400
	Pinna	genus	16400
species not entered	Atrina	genus	16342
species not entered	Atrina	genus	16342
	Atrina serrata	species	158633
recombined as	Atrina seminuda	species	157898
recombined as	Atrina seminuda	species	157898
	Atrina	genus	16342
	Atrina	genus	16342
species not entered	Plicatula	genus	16936
subjective synonym of	Plicatula gibbosa	species	87555
subjective synonym of	Plicatula gibbosa	species	87555
subjective synonym of	Plicatula gibbosa	species	87555
subjective synonym of	Plicatula gibbosa	species	87555
subjective synonym of	Plicatula gibbosa	species	87555
species not entered	Plicatula	genus	16936
subjective synonym of	Plicatula gibbosa	species	87555
subjective synonym of	Plicatula gibbosa	species	87555

	Plicatula	genus	16936
subjective synonym of	Plicatula gibbosa	species	87555
subjective synonym of	Plicatula gibbosa	species	87555
subjective synonym of	Plicatula gibbosa	species	87555
subjective synonym of	Plicatula gibbosa	species	87555
	Plicatula gibbosa	species	87555
	Plicatula gibbosa	species	87555
	Plicatula gibbosa	species	87555
misspelling of, obsolete variant of	Plicatula gibbosa	species	87555
	Plicatula gibbosa	species	87555
subjective synonym of	Plicatula gibbosa	species	87555
subjective synonym of	Plicatula gibbosa	species	87555
subjective synonym of	Plicatula gibbosa	species	87555
subjective synonym of	Plicatula gibbosa	species	87555
subjective synonym of	Plicatula gibbosa	species	87555
subjective synonym of	Plicatula gibbosa	species	87555
subjective synonym of	Plicatula gibbosa	species	87555
subjective synonym of	Plicatula gibbosa	species	87555
subjective synonym of	Plicatula gibbosa	species	87555
subjective synonym of	Plicatula gibbosa	species	87555
	Plicatula gibbosa	species	87555
subjective synonym of	Plicatula gibbosa	species	87555
subjective synonym of	Plicatula gibbosa	species	87555
subjective synonym of	Plicatula gibbosa	species	87555
subjective synonym of	Plicatula gibbosa	species	87555
subjective synonym of	Plicatula gibbosa	species	87555
	Plicatula gibbosa	species	87555
	Plicatula gibbosa	species	87555
subjective synonym of	Plicatula gibbosa	species	87555
subjective synonym of	Plicatula gibbosa	species	87555
	Plicatula	genus	16936
subjective synonym of	Plicatula gibbosa	species	87555
subjective synonym of	Plicatula gibbosa	species	87555
subjective synonym of	Plicatula gibbosa	species	87555
subjective synonym of	Plicatula gibbosa	species	87555
subjective synonym of	Plicatula gibbosa	species	87555
subjective synonym of	Plicatula gibbosa	species	87555
subjective synonym of	Plicatula gibbosa	species	87555
subjective synonym of	Plicatula gibbosa	species	87555
subjective synonym of	Plicatula gibbosa	species	87555
subjective synonym of	Plicatula gibbosa	species	87555
subjective synonym of	Plicatula gibbosa	species	87555
subjective synonym of	Plicatula gibbosa	species	87555
subjective synonym of	Plicatula gibbosa	species	87555
subjective synonym of	Plicatula gibbosa	species	87555
subjective synonym of	Plicatula gibbosa	species	87555
subjective synonym of	Plicatula gibbosa	species	87555

subjective synonym of	Plicatula gibbosa	species	87555
subjective synonym of	Plicatula gibbosa	species	87555
subjective synonym of	Plicatula gibbosa	species	87555
subjective synonym of	Plicatula gibbosa	species	87555
subjective synonym of	Plicatula gibbosa	species	87555
subjective synonym of	Plicatula gibbosa	species	87555
subjective synonym of	Plicatula gibbosa	species	87555
subjective synonym of	Plicatula gibbosa	species	87555
subjective synonym of	Plicatula gibbosa	species	87555
subjective synonym of	Plicatula gibbosa	species	87555
subjective synonym of	Plicatula gibbosa	species	87555
subjective synonym of	Plicatula gibbosa	species	87555
subjective synonym of	Plicatula gibbosa	species	87555
subjective synonym of	Plicatula gibbosa	species	87555
subjective synonym of	Plicatula gibbosa	species	87555
subjective synonym of	Plicatula gibbosa	species	87555
subjective synonym of	Plicatula gibbosa	species	87555
subjective synonym of	Plicatula gibbosa	species	87555
subjective synonym of	Plicatula gibbosa	species	87555
subjective synonym of	Plicatula gibbosa	species	87555
subjective synonym of	Plicatula gibbosa	species	87555
subjective synonym of	Plicatula gibbosa	species	87555
subjective synonym of	Plicatula gibbosa	species	87555
subjective synonym of	Plicatula gibbosa	species	87555
species not entered	Poromya	genus	18808
species not entered	Poromya	genus	18808
	Asaphis centenaria	species	98321
	Gari (Gobraeus)	subgenus	17695
	Gari	genus	17675
	Gari	genus	17675
	Psammobiidae	family	61938
	Pteria colymbus	species	90966
species not entered	Pteria	genus	16981
	Pteria colymbus	species	90966
	Pteria colymbus	species	90966
subjective synonym of	Pinctada imbricata	species	90968
	Pinctada	genus	16921
	Pteria colymbus	species	90966
	Pteria colymbus	species	90966
	Pteria colymbus	species	90966
	Pteria colymbus	species	90966
	Pteria colymbus	species	90966
	Pteria colymbus	species	90966
	Pteria colymbus	species	90966
	Pteria colymbus	species	90966
	Pteria	genus	16981
	Pteria colymbus	species	90966

	Semele proficua	species	109795
	Semele bellastriata	species	101156
	Abra aequalis	species	101160
species not entered	Cumingia	genus	17537
species not entered	Ervilia	genus	77232
	Semele bellastriata	species	101156
	Abra aequalis	species	101160
species not entered	Cumingia	genus	17537
	Semele bellastriata	species	101156
	Semele perlamellosa	species	161493
species not entered	Ervilia	genus	77232
	Abra aequalis	species	101160
	Semele bellastriata	species	101156
species not entered	Semele	genus	18239
	Semele carinata	species	98349
	Semele bellastriata	species	101156
	Semele purpurascens	species	109780
	Semele proficua	species	109795
species not entered	Semele	genus	18239
species not entered	Abra	genus	17298
	Abra aequalis	species	101160
	Abra aequalis	species	101160
species not entered	Ervilia	genus	77232
species not entered	Ervilia	genus	77232
	Abra aequalis	species	101160
	Semele bellastriata	species	101156
	Semele perlamellosa	species	161493
	Semele proficua	species	109795
	Cumingia	genus	17537
	Semele bellastriata	species	101156
	Semele perlamellosa	species	161493
	Semele proficua	species	109795
	Semele purpurascens	species	109780
	Semele perlamellosa	species	161493
	Semele bellastriata	species	101156
species not entered	Semele	genus	18239
	Cumingia tellinoides	species	94393
	Abra aequalis	species	101160
species not entered	Ervilia	genus	77232
	Abra aequalis	species	101160
	Semele purpurascens	species	109780
species not entered	Cumingia	genus	17537
	Semele perlamellosa	species	161493
	Semele purpurascens	species	109780
species not entered	Semele	genus	18239
species not entered	Semelina	genus	18240
	Abra aequalis	species	101160

subjective synonym of	Cumingia lamellosa	species	249438
	Ervilia concentrica	species	91254
species not entered	Ervilia	genus	77232
species not entered	Ervilia	genus	77232
	Semele perlamellosa	species	161493
	Semele bellastriata	species	101156
species not entered	Abra	genus	17298
species not entered	Semele	genus	18239
species not entered	Semele	genus	18239
species not entered	Semele	genus	18239
	Semele bellastriata	species	101156
	Abra aequalis	species	101160
	Abra aequalis	species	101160
	Semele bellastriata	species	101156
	Abra aequalis	species	101160
	Cumingia tellinoides	species	94393
species not entered	Ervilia	genus	77232
	Semele bellastriata	species	101156
	Semele proficua	species	109795
	Abra aequalis	species	101160
	Abra aequalis	species	101160
	Ervilia concentrica	species	91254
	Abra aequalis	species	101160
	Abra aequalis	species	101160
	Ervilia concentrica	species	91254
species not entered	Semele	genus	18239
	Ervilia concentrica	species	91254
	Ervilia concentrica	species	91254
	Semele proficua	species	109795
	Semele carinata	species	98349
species not entered	Ervilia	genus	77232
	Abra angulata	species	392355
	Semele bellastriata	species	101156
	Abra aequalis	species	101160
	Semele bellastriata	species	101156
	Semele bellastriata	species	101156
	Semele proficua	species	109795
	Semele proficua	species	109795
	Semele proficua	species	109795
	Cumingia tellinoides	species	94393
	Cumingia tellinoides	species	94393
	Ervilia concentrica	species	91254
	Ervilia concentrica	species	91254
species not entered	Ervilia	genus	77232
recombined as	Ervilia concentrica	species	91254
species not entered	Semele	genus	18239
	Cumingia tellinoides	species	94393

	Semele bellastriata	species	101156
	Abra aequalis	species	101160
	Semele	genus	18239
	Semele purpurascens	species	109780
	Abra aequalis	species	101160
	Semele proficua	species	109795
	Cumingia tellinoides	species	94393
	Semele purpurascens	species	109780
	Abra aequalis	species	101160
	Cumingia tellinoides	species	94393
	Abra aequalis	species	101160
	Semele subovata	species	98352
	Abra angulata	species	392355
	Semele transversa	species	392358
	Abra aequalis	species	101160
	Abra aequalis	species	101160
species not entered	Cumingia	genus	17537
	Abra aequalis	species	101160
	Cumingia tellinoides	species	94393
	Semele bellastriata	species	101156
	Semele bellastriata	species	101156
	Semele bellastriata	species	101156
	Semele subovata	species	98352
	Semele subovata	species	98352
	Semele subovata	species	98352
	Semele bellastriata	species	101156
	Semele bellastriata	species	101156
	Semele bellastriata	species	101156
species not entered	Semele	genus	18239
species not entered	Semele	genus	18239
species not entered	Semele	genus	18239
species not entered	Semele	genus	18239
species not entered	Semele	genus	18239
	Abra aequalis	species	101160
	Abra aequalis	species	101160
	Abra aequalis	species	101160
	Abra aequalis	species	101160
	Abra aequalis	species	101160
	Abra aequalis	species	101160
	Abra aequalis	species	101160
	Abra aequalis	species	101160
	Abra aequalis	species	101160
species not entered	Ervilia	genus	77232
species not entered	Ervilia	genus	77232
species not entered	Ervilia	genus	77232
	Cumingia tellinoides	species	94393
	Abra aequalis	species	101160

	Cumingia tellinoides	species	94393
	Abra aequalis	species	101160
	Cumingia tellinoides	species	94393
	Abra aequalis	species	101160
	Cumingia tellinoides	species	94393
	Abra aequalis	species	101160
	Semele bellastriata	species	101156
	Abra aequalis	species	101160
	Abra aequalis	species	101160
	Semele bellastriata	species	101156
	Semele proficua	species	109795
	Abra aequalis	species	101160
	Ervilia	genus	77232
	Cumingia tellinoides	species	94393
	Abra aequalis	species	101160
species not entered	Abra	genus	17298
	Cumingia tellinoides	species	94393
	Cumingia tellinoides	species	94393
	Semele subovata	species	98352
	Semele (Semele)	subgenus	101158
species not entered	Ervilia	genus	77232
	Semele subovata	species	98352
	Semele subovata	species	98352
species not entered	Semele	genus	18239
species not entered	Ervilia	genus	77232
species not entered	Ervilia	genus	77232
species not entered	Semele	genus	18239
recombined as	Semele bellastriata	species	101156
recombined as	Semele bellastriata	species	101156
recombined as	Abra aequalis	species	101160
	Abra aequalis	species	101160
	Semele bellastriata	species	101156
	Ervilia	genus	77232
	Ervilia	genus	77232
	Semele bellastriata	species	101156
	Ervilia	genus	77232
	Abra aequalis	species	101160
	Cumingia tellinoides	species	94393
	Abra aequalis	species	101160
	Abra aequalis	species	101160
	Abra aequalis	species	101160
	Abra aequalis	species	101160
	Cumingia tellinoides	species	94393
	Semele purpurascens	species	109780
	Cumingia tellinoides	species	94393
	Cumingia tellinoides	species	94393
	Cumingia tellinoides	species	94393

	Cumingia tellinoides	species	94393
	Semele subovata	species	98352
	Semele subovata	species	98352
	Semele subovata	species	98352
	Semele bellastriata	species	101156
species not entered	Solecurtus	genus	18263
species not entered	Solecurtus	genus	18263
	Tagelus (Mesopleura)	species	156532
species not entered	Solecurtus	genus	18263
species not entered	Solecurtus	genus	18263
species not entered	Solecurtus	genus	18263
	Tagelus	genus	18299
species not entered	Solecurtus	genus	18263
species not entered	Solecurtus	genus	18263
	Tagelus (Mesopleura)	species	156532
species not entered	Solecurtus	genus	18263
species not entered	Solecurtus	genus	18263
	Tagelus (Mesopleura)	species	156532
subjective synonym of	Tagelus plebeius	species	108172
subjective synonym of	Tagelus plebeius	species	108172
subjective synonym of	Tagelus plebeius	species	108172
subjective synonym of	Tagelus plebeius	species	108172
recombined as	Tagelus plebeius	species	108172
subjective synonym of	Tagelus plebeius	species	108172
subjective synonym of	Tagelus plebeius	species	108172
subjective synonym of	Tagelus plebeius	species	108172
subjective synonym of	Tagelus plebeius	species	108172
subjective synonym of	Tagelus plebeius	species	108172
subjective synonym of	Tagelus plebeius	species	108172
	Tagelus plebeius	species	108172
	Tagelus	genus	18299
	Tagelus plebeius	species	108172
species not entered	Tagelus	genus	18299
	Tagelus plebeius	species	108172
	Tagelus plebeius	species	108172
	Tagelus	genus	18299
	Tagelus	genus	18299
recombined as	Solemya (Petrasma) v	species	136155
species not entered	Solen	genus	18264
	Solen ensis	species	143258
species not entered	Solen	genus	18264
species not entered	Solen	genus	18264
	Solen	genus	18264
	Solen	genus	18264
species not entered	Musculium	genus	251736
	Pisidium	genus	61772
	Musculium	genus	251736

species not entered	Sphaerium	genus	62302
species not entered	Pisidium	genus	61772
	Spondylus	genus	17044
	Spondylus	genus	17044
	Spondylus rotundatus	species	109898
	Spondylus rotundatus	species	109898
	Spondylus americanus	species	90965
	Spondylus rotundatus	species	109898
	Spondylus rotundatus	species	109898
	Spondylus bostrychite	species	86295
	Spondylus	genus	17044
	Spondylus bostrychite	species	86295
	Spondylus	genus	17044
species not entered	Ensitellops	genus	17614
species not entered	Sportella	genus	18277
species not entered	Sportella	genus	18277
	Basterotia	genus	17399
species not entered	Fabella	genus	17647
species not entered	Anisodonta	genus	17338
species not entered	Ensitellops	genus	17614
recombined as	Basterotia (Basterotel	species	156312
species not entered	Fabella	genus	17647
species not entered	Fabella	genus	17647
species not entered	Ensitellops	genus	17614
	Basterotia (Basterotel	species	158520
species not entered	Anisodonta	genus	17338
	Ensitellops protextus	species	144829
	Sportella	genus	18277
species not entered	Sportella	genus	18277
species not entered	Sportella	genus	18277
species not entered	Sportella	genus	18277
recombined as	Basterotia (Basterotel	species	156312
	Basterotia	genus	17399
	Sportella	genus	18277
recombined as	Basterotia carolina	species	146437
species not entered	Ensitellops	genus	17614
recombined as	Basterotia carolina	species	146437
recombined as	Basterotia carolina	species	146437
species not entered	Ensitellops	genus	17614
species not entered	Sportella	genus	18277
species not entered	Sportella	genus	18277
species not entered	Sportella	genus	18277
species not entered	Sportella	genus	18277
species not entered	Sportella	genus	18277
species not entered	Sportella	genus	18277
species not entered	Sportella	genus	18277
species not entered	Sportella	genus	18277

species not entered	Sportella	genus	18277
species not entered	Sportella	genus	18277
species not entered	Ensitellops	genus	17614
species not entered	Ensitellops	genus	17614
species not entered	Ensitellops	genus	17614
species not entered	Ensitellops	genus	17614
recombined as	Basterotia (Basterotel	species	156312
recombined as	Basterotia (Basterotel	species	156312
species not entered	Fabella	genus	17647
species not entered	Ensitellops	genus	17614
	Anisodonta	genus	17338
replaced by	Strigilla mirabilis	species	136199
	Strigilla	genus	18287
recombined as	Florimetis magnoliana	species	118881
	Tellina	genus	18314
recombined as	Tellina (Eurytellina) ta	species	102580
	Tellidora lunulata	species	392351
recombined as	Angulus agilis	species	102570
recombined as	Tellina (Eurytellina) al	species	102568
species not entered	Tellina	genus	18314
recombined as	Scissula similis	species	94613
species not entered	Tellina	genus	18314
species not entered	Tellina	genus	18314
species not entered	Tellina	genus	18314
species not entered	Tellina	genus	18314
species not entered	Tellina	genus	18314
	Tellina	genus	18314
species not entered	Macoma	genus	17852
	Tellidora lunulata	species	392351
recombined as	Angulus agilis	species	102570
	Tellina	genus	18314
recombined as	Tellina (Eurytellina) ta	species	102580
	Tellidora lunulata	species	392351
species not entered	Tellina	genus	18314
recombined as	Tellina (Eurytellina) ta	species	102580
species not entered	Tellina	genus	18314
	Tellidora cristata	species	91169
subjective synonym of	Angulus texanus	species	102573
species not entered	Hemimetis	genus	60620
	Tellidora lunulata	species	392351
species not entered	Tellina	genus	18314
subjective synonym of	Angulus texanus	species	102573
	Tellina	genus	18314
	Tellina (Eurytellina) al	species	102568
	Tellina (Merisca) aequ	species	95003
recombined as	Moerella macilenta	species	95188
subjective synonym of	Angulus texanus	species	102573

subjective synonym of	Angulus texanus	species	102573
recombined as	Florimetis magnoliana	species	118881
recombined as	Macoma (Macoma) vi	species	95195
recombined as	Macoma (Macoma) vi	species	95195
recombined as	Macoma (Macoma) vi	species	95195
species not entered	Macoma (Psammacor	subgenus	18126
species not entered	Macoma (Psammacor	subgenus	18126
species not entered	Macoma (Psammacor	subgenus	18126
species not entered	Macoma (Psammacor	subgenus	18126
recombined as	Leporimetis intastriata	species	90890
	Tellidora cristata	species	91169
	Tellina	genus	18314
recombined as	Tellina (Eurytellina) al	species	102568
recombined as	Laciolina magna	species	102558
recombined as	Tellina (Eurytellina) ni	species	91154
recombined as	Leporimetis intastriata	species	90890
	Strigilla mirabilis	species	136199
	Tellidora cristata	species	91169
	Tellina	genus	18314
recombined as	Tellina (Merisca) aequ	species	95003
	Tellidora cristata	species	91169
	Tellina	genus	18314
recombined as	Scissula similis	species	94613
recombined as	Angulus merus	species	102556
species not entered	Tellina	genus	18314
recombined as	Eurytellina lineata	species	102562
recombined as	Tellina (Merisca) aequ	species	95003
recombined as	Angulus versicolor	species	102578
	Tellina	genus	18314
recombined as	Acorylus gouldii	species	102566
	Arcopagia fausta	species	109135
	Strigilla mirabilis	species	136199
species not entered	Macoma	genus	17852
species not entered	Macoma	genus	17852
	Macoma tenta	species	158645
	Macoma constricta	species	116270
recombined as	Psammacoma tagelifo	species	161340
recombined as	Cymatoica orientalis	species	156831
recombined as	Leporimetis intastriata	species	90890
	Tellidora cristata	species	91169
recombined as	Florimetis biplicata	species	98367
obsolete variant of	Tellina (Moerella)	subgenus	17928
obsolete variant of	Tellina (Eurytellina)	subgenus	17645
obsolete variant of	Tellina (Moerella)	subgenus	17928
	Macoma	genus	17852
	Angulus	genus	17335
obsolete variant of	Macoma (Macoma)	subgenus	95033

	Tellidora cristata	species	91169
obsolete variant of	Macoma (Macoma)	subgenus	95033
obsolete variant of	Angulus	genus	17335
obsolete variant of	Angulus	genus	17335
	Scissula similis	species	94613
obsolete variant of	Tellina (Tellinella)	subgenus	18315
obsolete variant of	Tellina (Eurytellina)	subgenus	17645
	Tellina	genus	18314
species not entered	Moerella (Scissula)	subgenus	18228
	Tellina (Eurytellina) ta	species	102580
	Tellina (Eurytellina) al	species	102568
species not entered	Tellina (Moerella)	subgenus	17928
species not entered	Moerella (Scissula)	subgenus	18228
species not entered	Tellina (Eurytellina)	subgenus	17645
recombined as	Angulus versicolor	species	102578
species not entered	Tellina (Moerella)	subgenus	17928
recombined as	Scissula similis	species	94613
	Tellidora lunulata	species	392351
	Tellidora lunulata	species	392351
recombined as	Leporimetis intastriata	species	90890
	Tellidora lunulata	species	392351
species not entered	Tellina	genus	18314
recombined as	Florimetis magnoliana	species	118881
replaced by	Strigilla mirabilis	species	136199
	Tellidora lunulata	species	392351
obsolete variant of	Tellina (Moerella)	subgenus	17928
	Macoma	genus	17852
	Tellidora lunulata	species	392351
	Tellidora lunulata	species	392351
obsolete variant of	Tellina (Moerella)	subgenus	17928
	Macoma	genus	17852
	Angulus	genus	17335
obsolete variant of	Tellina (Eurytellina)	subgenus	17645
obsolete variant of	Tellina (Eurytellina)	subgenus	17645
obsolete variant of	Tellina	genus	18314
	Tellina	genus	18314
obsolete variant of	Tellina (Eurytellina)	subgenus	17645
obsolete variant of	Tellina (Moerella)	subgenus	17928
obsolete variant of	Tellina	genus	18314
	Tellinidae	family	62468
obsolete variant of	Tellina (Moerella)	subgenus	17928
species not entered	Moerella (Scissula)	subgenus	18228
obsolete variant of	Tellina (Moerella)	subgenus	17928
	Tellidora lunulata	species	392351
	Tellidora lunulata	species	392351
	Macoma	genus	17852
species not entered	Macoma	genus	17852

obsolete variant of	Tellina (Moerella)	subgenus	17928
	Tellidora lunulata	species	392351
obsolete variant of	Tellina (Moerella)	subgenus	17928
species not entered	Macoma	genus	17852
obsolete variant of	Tellina (Moerella)	subgenus	17928
obsolete variant of	Tellina (Moerella)	subgenus	17928
recombined as	Arcopagia fausta	species	109135
recombined as	Arcopagia fausta	species	109135
recombined as	Arcopagia fausta	species	109135
recombined as	Arcopagia fausta	species	109135
recombined as	Arcopagia fausta	species	109135
recombined as	Arcopagia fausta	species	109135
recombined as	Arcopagia fausta	species	109135
recombined as	Arcopagia fausta	species	109135
recombined as	Arcopagia fausta	species	109135
	Angulus texanus	species	102573
	Tellidora cristata	species	91169
recombined as	Angulus texanus	species	102573
recombined as	Angulus texanus	species	102573
	Macoma	genus	17852
	Tellidora cristata	species	91169
recombined as	Tellina (Eurytellina) al	species	102568
recombined as	Angulus texanus	species	102573
recombined as	Angulus texanus	species	102573
species not entered	Hemimetis	genus	60620
recombined as	Macoma (Macoma) vi	species	95195
recombined as	Angulus texanus	species	102573
recombined as	Tellina (Eurytellina) al	species	102568
recombined as	Tellina (Eurytellina) al	species	102568
recombined as	Tellina (Eurytellina) al	species	102568
recombined as	Tellina (Eurytellina) al	species	102568
recombined as	Angulus texanus	species	102573
recombined as	Angulus texanus	species	102573
recombined as	Macoma (Macoma) ba	species	107603
recombined as	Macoma (Macoma) ca	species	97999
	Macoma constricta	species	116270
	Strigilla mirabilis	species	136199
recombined as	Macoma (Macoma) ba	species	107603
	Macoma tenta	species	158645
	Strigilla mirabilis	species	136199
	Tellidora cristata	species	91169
	Tellidora cristata	species	91169
obsolete variant of	Angulus	genus	17335
obsolete variant of	Angulus	genus	17335
	Angulus texanus	species	102573
	Angulus texanus	species	102573
	Angulus texanus	species	102573

	Macoma	genus	17852
	Macoma	genus	17852
	Macoma	genus	17852
reassigned as	Moerella (Scissula)	subgenus	18228
	Tellinidae	family	62468
obsolete variant of	Tellina (Moerella)	subgenus	17928
obsolete variant of	Tellina (Moerella)	subgenus	17928
	Macoma	genus	17852
	Tellina (Merisca) aequ	species	95003
recombined as	Moerella declivis	species	95181
recombined as	Tellina (Eurytellina) al	species	102568
recombined as	Angulus agilis	species	102570
recombined as	Angulus agilis	species	102570
recombined as	Angulus agilis	species	102570
recombined as	Angulus agilis	species	102570
subjective synonym of	Angulus texanus	species	102573
subjective synonym of	Angulus texanus	species	102573
recombined as	Moerella dupliniana	species	95186
	Tellina	genus	18314
recombined as	Moerella dupliniana	species	95186
recombined as	Moerella dupliniana	species	95186
recombined as	Moerella dupliniana	species	95186
recombined as	Moerella dupliniana	species	95186
recombined as	Moerella dupliniana	species	95186
recombined as	Moerella dupliniana	species	95186
recombined as	Moerella dupliniana	species	95186
recombined as	Moerella dupliniana	species	95186
recombined as	Moerella dupliniana	species	95186
recombined as	Moerella dupliniana	species	95186
recombined as	Moerella macilenta	species	95188
recombined as	Moerella macilenta	species	95188
recombined as	Moerella macilenta	species	95188
recombined as	Moerella macilenta	species	95188
recombined as	Moerella macilenta	species	95188
recombined as	Macoma (Macoma) ca	species	95199
recombined as	Angulus texanus	species	102573
recombined as	Tellina (Eurytellina) al	species	102568
recombined as	Angulus agilis	species	102570
recombined as	Angulus agilis	species	102570
recombined as	Angulus texanus	species	102573
recombined as	Angulus texanus	species	102573
recombined as	Angulus texanus	species	102573
	Tellidora cristata	species	91169
recombined as	Angulus agilis	species	102570
	Strigilla (Strigilla)	subgenus	161310
obsolete variant of	Tellina (Eurytellina)	subgenus	17645
	Moerella macilenta	species	95188

	Psammacoma (Psamn	subgenus	94615
obsolete variant of	Tellina (Moerella)	subgenus	17928
	Moerella dupliniana	species	95186
	Moerella macilenta	species	95188
	Moerella dupliniana	species	95186
	Moerella macilenta	species	95188
	Tellinidae	family	62468
obsolete variant of	Tellina (Moerella)	subgenus	17928
obsolete variant of	Tellina (Moerella)	subgenus	17928
	Angulus	genus	17335
	Tellinidae	family	62468
	Strigilla	genus	18287
obsolete variant of	Macoma (Psammacor	subgenus	18126
obsolete variant of	Tellina (Moerella)	subgenus	17928
	Macoma	genus	17852
	Strigilla	genus	18287
recombined as	Angulus agilis	species	102570
recombined as	Angulus agilis	species	102570
recombined as	Angulus texanus	species	102573
recombined as	Angulus texanus	species	102573
recombined as	Angulus texanus	species	102573
recombined as	Macoma (Macoma) ba	species	107603
	Macoma constricta	species	116270
	Tellina	genus	18314
	Tellina	genus	18314
	Tellina	genus	18314
	Tellina	genus	18314
species not entered	Tellina	genus	18314
recombined as	Moerella declivis	species	95181
	Tellina	genus	18314
	Tellina	genus	18314
recombined as	Moerella declivis	species	95181
species not entered	Tellina	genus	18314
	Tellina	genus	18314
	Moerella declivis	species	95181
	Tellinidae	family	62468
recombined as	Macoma (Macoma) ca	species	97999
	Macoma tenta	species	158645
obsolete variant of	Tellina (Moerella)	subgenus	17928
	Tellinidae	family	62468
	Teredinidae	family	62496
	Teredinidae	family	62496
species not entered	Teredo	genus	18510
species not entered	Kuphus	genus	98761
	Kuphus fistula	species	99357
species not entered	Kuphus	genus	98761
	Kuphus fistula	species	99357

	Kuphus fistula	species	99357
	Kuphus fistula	species	99357
	Kuphus fistula	species	99357
recombined as	Kuphus fistula	species	99357
	Kuphus fistula	species	99357
	Kuphus fistula	species	99357
species not entered	Kuphus	genus	98761
species not entered	Kuphus	genus	98761
species not entered	Thracia (Cyathodonta)	subgenus	18728
	Thracia	genus	18836
	Thracia	genus	18836
species not entered	Thracia (Cyathodonta)	subgenus	18728
	Thracia (Cyathodonta)	subgenus	18728
species not entered	Thracia	genus	18836
species not entered	Thracia (Cyathodonta)	subgenus	18728
	Thracia brioni	species	82737
	Thracia brioni	species	82737
	Thracia brioni	species	82737
recombined as	Thracia (Homoeodesn	species	108651
species not entered	Thracia	genus	18836
	Thracia	genus	18836
	Thracia	genus	18836
species not entered	Thyasira	genus	18336
	Thyasira	genus	18336
	Coralliophaga corallio	species	59853
	Diplodonta acclinis	species	98440
	Diplodonta acclinis	species	98440
	Diplodonta acclinis	species	98440
	Diplodonta acclinis	species	98440
species not entered	Diplodonta	genus	17578
species not entered	Diplodonta	genus	17578
species not entered	Diplodonta	genus	17578
	Diplodonta	genus	17578
	Diplodonta	genus	17578
species not entered	Diplodonta	genus	17578
recombined as	Diplodonta (Phlyctide	species	147914
	Diplodonta nucleiform	species	135917
	Diplodonta punctata	species	90984
	Diplodonta (Diplodont	subgenus	94616
species not entered	Taras (Taras)	subgenus	100560
recombined as	Diplodonta soror	species	146391
	Diplodonta acclinis	species	98440
species not entered	Diplodonta	genus	17578
	Diplodonta subvexa	species	98444
	Diplodonta	genus	17578
recombined as	Diplodonta subvexa	species	98444
	Diplodonta punctata	species	90984

recombined as	Diplodonta (Phlyctider	species	147914
	Diplodonta subvexa	species	98444
species not entered	Diplodonta	genus	17578
	Diplodonta acclinis	species	98440
recombined as	Diplodonta (Phlyctider	species	147914
	Diplodonta soror	species	146391
	Diplodonta soror	species	146391
recombined as	Diplodonta punctata	species	90984
recombined as	Diplodonta soror	species	146391
recombined as	Diplodonta soror	species	146391
recombined as	Diplodonta soror	species	146391
	Diplodonta punctata	species	90984
	Diplodonta acclinis	species	98440
	Diplodonta nucleiform	species	135917
species not entered	Diplodonta	genus	17578
	Diplodonta acclinis	species	98440
	Diplodonta acclinis	species	98440
species not entered	Diplodonta	genus	17578
	Diplodonta acclinis	species	98440
	Diplodonta acclinis	species	98440
	Phlyctiderma heroni	species	82709
species not entered	Diplodonta	genus	17578
species not entered	Diplodonta	genus	17578
species not entered	Diplodonta	genus	17578
recombined as	Diplodonta soror	species	146391
recombined as	Diplodonta soror	species	146391
	Diplodonta	genus	17578
	Diplodonta punctata	species	90984
	Diplodonta punctata	species	90984
	Diplodonta acclinis	species	98440
	Diplodonta acclinis	species	98440
	Diplodonta (Diplodont	subgenus	94616
recombined as	Diplodonta acclinis	species	98440
	Diplodonta acclinis	species	98440
	Diplodonta acclinis	species	98440
recombined as	Diplodonta soror	species	146391
recombined as	Diplodonta soror	species	146391
	Diplodonta	genus	17578
species not entered	Diplodonta	genus	17578
obsolete variant of	Diplodonta (Phlyctider	subgenus	18068
	Diplodonta punctata	species	90984
	Diplodonta acclinis	species	98440
species not entered	Diplodonta	genus	17578
	Diplodonta acclinis	species	98440
	Diplodonta acclinis	species	98440
	Diplodonta (Diplodont	subgenus	94616
obsolete variant of	Anodonta	genus	83658

species not entered	Dosinia	genus	17595
	Dosinia	genus	17595
recombined as	Lirophora (Lirophora)	species	98377
	Chione cancellata	species	94440
species not entered	Chione	genus	17468
recombined as	Timoclea grus	species	95520
	Chione	genus	17468
	Anomalocardia cuneir	species	94548
	Venus	genus	18391
subjective synonym of	Mercenaria campechi	species	94395
recombined as	Mercenaria campechi	species	94395
species not entered	Transennella	genus	18348
recombined as	Puberella intapurpure	species	108665
recombined as	Puberella intapurpure	species	108665
recombined as	Puberella intapurpure	species	108665
recombined as	Puberella intapurpure	species	108665
	Chione cancellata	species	94440
	Chione cancellata	species	94440
	Venus	genus	18391
	Mercenaria campechi	species	94395
	Chione cancellata	species	94440
recombined as	Timoclea grus	species	95520
recombined as	Dosinia (Dosinidia) ele	species	94558
species not entered	Gouldia	genus	17708
	Chione cancellata	species	94440
species not entered	Anomalocardia	genus	17341
species not entered	Anomalocardia	genus	17341
	Chione cancellata	species	94440
recombined as	Timoclea grus	species	95520
recombined as	Dosinia (Dosinidia) ele	species	94558
	Gouldia cerina	species	101134
species not entered	Gouldia	genus	17708
species not entered	Gouldia	genus	17708
	Gouldia	genus	17708
	Macrocallista maculat	species	109337
recombined as	Macrocallista (Macroc	species	109468
	Mercenaria campechi	species	94395
	Parastarte triquetra	species	162146
subjective synonym of	Pitar morrhuanus	species	94532
	Transennella conradin	species	91160
species not entered	Anomalocardia	genus	17341
	Dosinia discus	species	94591
	Parastarte triquetra	species	162146
subjective synonym of	Pitar morrhuanus	species	94532
	Transennella conradin	species	91160
	Chione cancellata	species	94440
species not entered	Anomalocardia	genus	17341

	Chione cancellata	species	94440
recombined as	Timoclea grus	species	95520
recombined as	Lirophora (Lirophora)	species	98377
species not entered	Chione	genus	17468
recombined as	Dosinia (Dosinidia) ele	species	94558
	Gouldia cerina	species	101134
	Macrocallista maculat	species	109337
recombined as	Macrocallista (Macroc	species	109468
	Mercenaria campechi	species	94395
	Parastarte triquetra	species	162146
subjective synonym of	Pitar morrhuanus	species	94532
	Chione cancellata	species	94440
species not entered	Anomalocardia	genus	17341
	Chione cancellata	species	94440
recombined as	Timoclea grus	species	95520
recombined as	Lirophora (Lirophora)	species	98377
recombined as	Dosinia (Dosinidia) ele	species	94558
	Mercenaria campechi	species	94395
	Parastarte triquetra	species	162146
subjective synonym of	Pitar morrhuanus	species	94532
	Transennella conradin	species	91160
species not entered	Anomalocardia	genus	17341
	Chione cancellata	species	94440
recombined as	Macrocallista (Macroc	species	109468
	Parastarte triquetra	species	162146
	Transennella conradin	species	91160
	Transennella stimpsor	species	94533
recombined as	Mercenaria campechi	species	94395
species not entered	Anomalocardia	genus	17341
	Chione cancellata	species	94440
species not entered	Chione	genus	17468
recombined as	Lirophora (Lirophora)	species	98377
recombined as	Lirophora (Panchione)	species	161768
recombined as	Dosinia (Dosinidia) ele	species	94558
obsolete variant of	Macrocallista	genus	17856
recombined as	Macrocallista (Macroc	species	109468
	Mercenaria campechi	species	94395
	Parastarte triquetra	species	162146
	Transennella	genus	18348
	Chione cancellata	species	94440
recombined as	Dosinia acetabulum	species	95240
	Dosinia (Dosinidia) ele	species	94558
	Dosinia (Dosinidia) ele	species	94558
species not entered	Transennella	genus	18348
species not entered	Transennella	genus	18348
species not entered	Gouldia	genus	17708
species not entered	Gouldia	genus	17708

species not entered	Gouldia	genus	17708
species not entered	Gouldia	genus	17708
recombined as	Macrocallista (Macroc	species	95245
recombined as	Macrocallista (Macroc	species	95245
subjective synonym of	Pitar morrhuanus	species	94532
subjective synonym of	Pitar morrhuanus	species	94532
	Cyclinella tenuis	species	158649
	Cyclinella tenuis	species	158649
species not entered	Chione (Chione)	subgenus	95479
species not entered	Chione (Chione)	subgenus	95479
species not entered	Chione (Chione)	subgenus	95479
species not entered	Chione (Chione)	subgenus	95479
	Chione (Chione) cortir	species	95480
species not entered	Lirophora	genus	17824
recombined as	Lirophora (Panchione)	species	161768
recombined as	Lirophora (Panchione)	species	161768
recombined as	Lirophora (Panchione)	species	161768
recombined as	Lirophora (Panchione)	species	161768
recombined as	Lirophora (Lirophora)	species	98377
obsolete variant of	Lirophora	genus	17824
obsolete variant of	Lirophora	genus	17824
recombined as	Timoclea grus	species	95520
recombined as	Mercenaria campechi	species	94395
recombined as	Mercenaria campechi	species	94395
recombined as	Mercenaria campechi	species	94395
recombined as	Mercenaria campechi	subspecies	95541
	Gemma magna	species	95553
	Parastarte triquetra	species	162146
subjective synonym of	Anomalocardia cuneir	species	94548
	Anomalocardia brasili	species	101146
	Chione cancellata	species	94440
recombined as	Timoclea grus	species	95520
recombined as	Dosinia (Dosinidia) ele	species	94558
	Macrocallista maculat	species	109337
recombined as	Macrocallista (Macroc	species	109468
	Mercenaria campechi	species	94395
	Pitar	genus	18075
	Pitar simpsoni	species	146433
subjective synonym of	Anomalocardia cuneir	species	94548
	Chione cancellata	species	94440
recombined as	Timoclea grus	species	95520
recombined as	Dosinia (Dosinidia) ele	species	94558
	Gouldia cerina	species	101134
recombined as	Macrocallista (Macroc	species	109468
	Mercenaria campechi	species	94395
	Parastarte triquetra	species	162146
	Pitar simpsoni	species	146433

species not entered	Transennella	genus	18348
	Anomalocardia	genus	17341
	Chione cancellata	species	94440
recombined as	Timoclea grus	species	95520
recombined as	Dosinia (Dosinidia) ele	species	94558
recombined as	Macrocallista (Macroc	species	109468
	Mercenaria campechi	species	94395
	Parastarte triquetra	species	162146
	Pitar simpsoni	species	146433
species not entered	Mercenaria	genus	17892
misspelling of, subjective synonym of, species not entered	Mercenaria	genus	17892
species not entered	Antigona	genus	17345
recombined as	Globivenus rugatina	species	135220
recombined as	Puberella intapurpure	species	108665
	Chione cancellata	species	94440
recombined as	Lirophora (Lirophora)	species	98377
recombined as	Timoclea grus	species	95520
species not entered	Anomalocardia	genus	17341
	Anomalocardia brasili	species	101146
recombined as	Pitar cordatus	species	146426
subjective synonym of	Pitar morrhuanus	species	94532
	Pitar (Hyphantosoma)	subgenus	17745
	Gouldia cerina	species	101134
	Macrocallista maculat	species	109337
	Mercenaria campechi	species	94395
	Transennella cubaniar	species	91158
	Transennella conradin	species	91160
recombined as	Dosinia (Dosinidia) ele	species	94558
	Cyclinella tenuis	species	158649
recombined as	Lirophora (Panchione)	species	161768
recombined as	Lirophora (Panchione)	species	161768
recombined as	Dosinia (Dosinidia) ele	species	94558
recombined as	Lirophora (Panchione)	species	161768
recombined as	Lirophora (Lirophora)	species	98377
recombined as	Lirophora (Panchione)	species	161768
recombined as	Lirophora (Panchione)	species	161768
	Chione cancellata	species	94440
	Transennella stimpsor	species	94533
recombined as	Dosinia (Dosinidia) ele	species	94558
subjective synonym of	Anomalocardia cuneir	species	94548
	Chione cancellata	species	94440
	Transennella stimpsor	species	94533
subjective synonym of	Anomalocardia cuneir	species	94548
	Gemma gemma	species	108167
recombined as	Dosinia (Dosinidia) ele	species	94558
	Chione cancellata	species	94440
recombined as	Dosinia discus	species	94591

	Pitar	genus	18075
	Anomalocardia cuneir	species	94548
	Transennella stimpsor	species	94533
	Chione cancellata	species	94440
subjective synonym of	Anomalocardia cuneir	species	94548
	Pitar simpsoni	species	146433
	Transennella stimpsor	species	94533
	Gemma magna	species	95553
	Venus	genus	18391
	Chione cancellata	species	94440
recombined as	Lirophora (Lirophora)	species	98377
	Chione cancellata	species	94440
	Chione cribraria	species	95485
	Chione cancellata	species	94440
	Chione cancellata	species	94440
	Dosinia	genus	17595
	Chione cancellata	species	94440
	Chione cancellata	species	94440
	Dosinia	genus	17595
	Chione cancellata	species	94440
	Chione cancellata	species	94440
	Chione cancellata	species	94440
	Chione	genus	17468
	Gemma magna	species	95553
obsolete variant of	Chione	genus	17468
recombined as	Lirophora (Lirophora)	species	98377
	Chione cancellata	species	94440
	Venus	genus	18391
	Chione	genus	17468
	Parastarte	genus	18033
	Chione	genus	17468
	Parastarte	genus	18033
	Chione cancellata	species	94440
	Chione	genus	17468
misspelling of, obsolete variant of	Transennella	genus	18348
	Chione	genus	17468
misspelling of, obsolete variant of	Transennella	genus	18348
	Parastarte	genus	18033
misspelling of, obsolete variant of	Transennella	genus	18348
	Macrocallista	genus	17856
misspelling of, obsolete variant of	Transennella	genus	18348
misspelling of, obsolete variant of	Transennella	genus	18348
misspelling of, obsolete variant of	Transennella	genus	18348
	Gemma magna	species	95553
recombined as	Parastarte triquetra	species	162146
recombined as	Chione moristans	species	109475
recombined as	Chione moristans	species	109475

species not entered	Anomalocardia	genus	17341
species not entered	Anomalocardia	genus	17341
	Mercenaria mercenar	species	95528
	Macrocallista (Macroc	species	109468
recombined as	Macrocallista maculat	species	109337
subjective synonym of	Pitar morrhuanus	species	94532
recombined as	Globivenus rugatina	species	135220
recombined as	Chione cancellata	species	94440
recombined as	Timoclea grus	species	95520
species not entered	Anomalocardia	genus	17341
	Transennella conradin	species	91160
	Dosinia (Dosinidia) ele	species	94558
species not entered	Gouldia	genus	17708
	Gemma magna	species	95553
	Chione cancellata	species	94440
	Mercenaria mercenar	species	95528
	Mercenaria campechi	species	94395
species not entered	Anomalocardia	genus	17341
	Chione elevata	species	146242
	Mercenaria campechi	species	94395
species not entered	Anomalocardia	genus	17341
	Mercenaria permagna	species	82724
	Chione elevata	species	146242
recombined as	Macrocallista (Macroc	species	95245
species not entered	Chione	genus	17468
recombined as	Lirophora (Panchione)	species	161768
recombined as	Lirophora (Lirophora)	species	98377
	Anomalocardia	genus	17341
	Chione cancellata	species	94440
	Mercenaria mercenar	species	95528
	Pitar morrhuanus	species	94532
species not entered	Anomalocardia	genus	17341
	Macrocallista (Macroc	species	109468
	Dosinia (Dosinidia) ele	species	94558
	Macrocallista (Macroc	species	109468
	Mercenaria mercenar	species	95528
	Chione cancellata	species	94440
species not entered	Anomalocardia	genus	17341
	Pitar morrhuanus	species	94532
	Transennella conradin	species	91160
	Chione cancellata	species	94440
	Chione cancellata	species	94440
	Gemma gemma	species	108167
	Transennella stimpsor	species	94533
subjective synonym of	Anomalocardia cuneir	species	94548
	Transennella stimpsor	species	94533
subjective synonym of	Anomalocardia cuneir	species	94548

	Pitar simpsoni	species	146433
recombined as	Mercenaria campechi	subspecies	95541
recombined as	Mercenaria campechi	subspecies	95535
species not entered	Mercenaria	genus	17892
recombined as	Lirophora (Panchione)	species	161768
species not entered	Chione	genus	17468
recombined as	Venus (Mercenaria) ca	subspecies	95538
	Chione cribraria	species	95485
recombined as	Macrocallista (Macroc	species	95245
species not entered	Anomalocardia	genus	17341
	Chione cancellata	species	94440
recombined as	Macrocallista (Macroc	species	109468
species not entered	Transennella	genus	18348
subjective synonym of	Pitar morrhuanus	species	94532
species not entered	Anomalocardia	genus	17341
	Chione cancellata	species	94440
species not entered	Gemma	genus	17681
recombined as	Macrocallista (Macroc	species	109468
recombined as	Transennella conradin	species	91160
subjective synonym of	Pitar morrhuanus	species	94532
recombined as	Dosinia (Dosinidia) ele	species	94558
	Transennella stimpsor	species	94533
	Chione cancellata	species	94440
	Macrocallista (Macroc	species	95245
	Transennella conradin	species	91160
recombined as	Lirophora (Lirophora)	species	98377
	Chione cribraria	species	95485
	Chione cancellata	species	94440
species not entered	Anomalocardia	genus	17341
species not entered	Transennella	genus	18348
	Gemma gemma	species	108167
	Pitar morrhuanus	species	94532
	Chione cancellata	species	94440
recombined as	Dosinia (Dosinidia) ele	species	94558
species not entered	Anomalocardia	genus	17341
	Lirophora	genus	17824
	Transennella conradin	species	91160
	Pitar morrhuanus	species	94532
	Mercenaria mercenar	species	95528
recombined as	Macrocallista (Macroc	species	109468
	Macrocallista maculat	species	109337
	Chione cancellata	species	94440
	Mercenaria mercenar	species	95528
recombined as	Macrocallista (Macroc	species	109468
species not entered	Anomalocardia	genus	17341
recombined as	Dosinia (Dosinidia) ele	species	94558
	Transennella conradin	species	91160

	Callocardia (Agriopom	subgenus	82721
	Gemma magna	species	95553
recombined as	Lirophora (Panchione)	species	161768
species not entered	Chione	genus	17468
recombined as	Lirophora (Lirophora)	species	98377
recombined as	Mercenaria campechi	subspecies	95535
recombined as	Macrocallista (Macroc	species	95245
misspelling of, species not entered	Transennella	genus	18348
recombined as	Lirophora (Lirophora)	species	98377
recombined as	Lirophora (Panchione)	species	161768
species not entered	Chione	genus	17468
	Pitar	genus	18075
misspelling of, species not entered	Transennella	genus	18348
recombined as	Mercenaria campechi	subspecies	95535
recombined as	Lirophora (Panchione)	species	161768
recombined as	Lirophora (Lirophora)	species	98377
species not entered	Chione	genus	17468
misspelling of, species not entered	Transennella	genus	18348
	Pitar	genus	18075
	Transennella	genus	18348
recombined as	Dosinia (Dosinidia) ele	species	94558
	Chione elevata	species	146242
recombined as	Lirophora (Lirophora)	species	98377
	Mercenaria	genus	17892
	Macrocallista maculat	species	109337
	Transennella stimpsor	species	94533
	Anomalocardia	genus	17341
	Chione cancellata	species	94440
subjective synonym of	Anomalocardia cuneir	species	94548
	Globivenus rugatina	species	135220
	Gemma gemma	species	108167
recombined as	Chione elevata	species	146242
recombined as	Chione cribraria	species	95485
	Macrocallista (Macroc	species	109468
recombined as	Chione elevata	species	146242
recombined as	Dosinia (Dosinidia) ele	species	94558
	Transennella conradin	species	91160
recombined as	Macrocallista (Macroc	species	95245
recombined as	Chione cribraria	species	95485
species not entered	Anomalocardia	genus	17341
	Chione cancellata	species	94440
	Chione cancellata	species	94440
recombined as	Timoclea grus	species	95520
recombined as	Timoclea grus	species	95520
	Chione cancellata	species	94440
species not entered	Chione	genus	17468
	Chione cancellata	species	94440

species not entered	Anomalocardia	genus	17341
	Chione cancellata	species	94440
recombined as	Mercenaria campechi	species	94395
recombined as	Lirophora (Panchione)	species	161768
	Anomalocardia cuneir	species	94548
	Chione elevata	species	146242
	Cyclinella tenuis	species	158649
	Dosinia discus	species	94591
recombined as	Dosinia (Dosinidia) ele	species	94558
	Macrocallista maculat	species	109337
	Mercenaria campechi	species	94395
	Parastarte triquetra	species	162146
	Pitar morrhuanus	species	94532
	Pitar simpsoni	species	146433
	Timoclea grus	species	95520
	Transennella conradin	species	91160
	Mercenaria mercenar	species	95528
	Mercenaria mercenar	species	95528
	Dosinia	genus	17595
species not entered	Gemma	genus	17681
	Gemma magna	species	95553
recombined as	Lirophora (Lirophora)	species	98377
	Chione cancellata	species	94440
recombined as	Chione cribraria	species	95485
recombined as	Lirophora (Lirophora)	species	98377
species not entered	Gouldia	genus	17708
	Mercenaria campechi	species	94395
	Gemma magna	species	95553
recombined as	Macrocallista (Macroc	species	109468
	Mercenaria campechi	species	94395
misspelling of, species not entered	Transennella	genus	18348
	Gemma magna	species	95553
	Mercenaria campechi	species	94395
	Gemma magna	species	95553
recombined as	Macrocallista (Macroc	species	109468
	Mercenaria campechi	species	94395
misspelling of, species not entered	Transennella	genus	18348
recombined as	Lirophora (Lirophora)	species	98377
recombined as	Mercenaria campechi	subspecies	95541
species not entered	Mercenaria	genus	17892
species not entered	Chione	genus	17468
recombined as	Lirophora (Lirophora)	species	98377
recombined as	Macrocallista (Megapi	species	170446
	Dosinia acetabulum	species	95240
subjective synonym of	Pitar morrhuanus	species	94532
species not entered	Chione	genus	17468
recombined as	Dosinia (Dosinidia) ele	species	94558

	Gemma magna	species	95553
recombined as	Macrocallista (Macroc	species	109468
subjective synonym of	Pitar morrhuanus	species	94532
recombined as	Dosinia (Dosinidia) ele	species	94558
recombined as	Macrocallista (Macroc	species	109468
	Chione cancellata	species	94440
	Chione cancellata	species	94440
	Chione cancellata	species	94440
recombined as	Puberella intapurpure	species	108665
recombined as	Puberella intapurpure	species	108665
recombined as	Timoclea grus	species	95520
recombined as	Timoclea grus	species	95520
	Mercenaria mercenar	species	95528
	Mercenaria mercenar	species	95528
	Mercenaria mercenar	species	95528
	Mercenaria campechi	species	94395
	Mercenaria campechi	species	94395
	Mercenaria campechi	species	94395
	Lirophora (Lirophora)	subgenus	90364
species not entered	Gouldia	genus	17708
	Chione	genus	17468
	Mercenaria	genus	17892
species not entered	Pitar (Pitar)	subgenus	100951
	Transennella stimpsor	species	94533
recombined as	Macrocallista maculat	species	109337
	Veneridae	family	62779
	Pitar morrhuanus	species	94532
	Macrocallista (Macroc	species	109468
	Mercenaria campechi	species	94395
	Gemma magna	species	95553
recombined as	Lirophora (Lirophora)	species	98377
recombined as	Dosinia (Dosinidia) ele	species	94558
recombined as	Lirophora (Lirophora)	species	98377
	Gemma magna	species	95553
misspelling of, species not entered	Transennella	genus	18348
	Macrocallista (Macroc	species	95245
recombined as	Mercenaria mercenar	species	95528
subjective synonym of	Pitar morrhuanus	species	94532
recombined as	Dosinia acetabulum	species	95240
recombined as	Chione cribraria	species	95485
species not entered	Gouldia	genus	17708
	Gemma magna	species	95553
recombined as	Timoclea grus	species	95520
	Chione cribraria	species	95485
recombined as	Lirophora (Lirophora)	species	98377
recombined as	Mercenaria campechi	subspecies	95535
species not entered	Gouldia	genus	17708

	Gemma magna	species	95553
	Mercenaria	genus	17892
recombined as	Chione cribraria	species	95485
recombined as	Lirophora (Lirophora)	species	98377
	Chione cribraria	species	95485
species not entered	Gouldia	genus	17708
species not entered	Gouldia	genus	17708
species not entered	Gouldia	genus	17708
	Mercenaria permagna	species	82724
	Macrocallista greeni	species	82723
species not entered	Gouldia	genus	17708
	Macrocallista greeni	species	82723
	Mercenaria permagna	species	82724
	Mercenaria permagna	species	82724
	Macrocallista greeni	species	82723
species not entered	Gouldia	genus	17708
	Gemma magna	species	95553
	Transennella stimpsor	species	94533
species not entered	Pitar	genus	18075
	Transennella stimpsor	species	94533
species not entered	Pitar	genus	18075
species not entered	Pitar	genus	18075
	Transennella stimpsor	species	94533
subjective synonym of, species not entered	Globivenus	genus	17689
recombined as	Mercenaria campechi	subspecies	95535
species not entered	Cooperella	genus	17500
	Dosinia acetabulum	species	95240
	Dosinia (Dosinidia) ele	species	94558
	Dosinia (Dosinidia) ele	species	94558
	Dosinia (Dosinidia) ele	species	94558
recombined as	Macrocallista (Macroc	species	95245
recombined as	Macrocallista (Macroc	species	95245
recombined as	Macrocallista (Macroc	species	95245
recombined as	Macrocallista (Macroc	species	95245
recombined as	Macrocallista (Macroc	species	95245
recombined as	Macrocallista (Macroc	species	95245
recombined as	Macrocallista (Macroc	species	95245
	Macrocallista (Costaca	species	95263
subjective synonym of	Pitar morrhuanus	species	94532
subjective synonym of	Pitar morrhuanus	species	94532
subjective synonym of	Pitar morrhuanus	species	94532
subjective synonym of	Pitar morrhuanus	species	94532
subjective synonym of	Pitar morrhuanus	species	94532
subjective synonym of	Pitar morrhuanus	species	94532
	Callocardia (Agriopom	species	95478
	Callocardia (Agriopom	species	82722
recombined as	Chione cribraria	species	95485

recombined as	Chione cribraria	species	95485
recombined as	Chione cribraria	species	95485
recombined as	Chione cribraria	species	95485
recombined as	Chione cribraria	species	95485
recombined as	Chione cribraria	species	95485
recombined as	Chione cribraria	species	95485
recombined as	Chione cribraria	species	95485
recombined as	Timoclea grus	species	95520
recombined as	Timoclea grus	species	95520
recombined as	Timoclea grus	species	95520
recombined as	Timoclea grus	species	95520
recombined as	Timoclea grus	species	95520
recombined as	Timoclea grus	species	95520
recombined as	Mercenaria campechi	species	94395
recombined as	Mercenaria campechi	species	94395
recombined as	Mercenaria campechi	species	94395
recombined as	Mercenaria campechi	species	94395
recombined as	Mercenaria campechi	species	94395
recombined as	Mercenaria campechi	species	94395
recombined as	Mercenaria campechi	species	94395
recombined as	Mercenaria campechi	species	94395
recombined as	Mercenaria campechi	species	94395
recombined as	Mercenaria campechi	species	94395
recombined as	Mercenaria campechi	species	94395
recombined as	Mercenaria campechi	species	94395
recombined as	Mercenaria campechi	species	94395
	Gemma magna	species	95553
	Gemma magna	species	95553
	Gemma magna	species	95553
	Gemma magna	species	95553
	Gemma magna	species	95553
	Gemma magna	species	95553
	Gemma magna	species	95553
	Gemma magna	species	95553
	Gemma magna	species	95553
	Gemma magna	species	95553
	Gemma magna	species	95553
	Gemma magna	species	95553
	Gemma magna	species	95553
	Gemma cravenensis	species	95557
	Mercenaria permagna	species	82724
	Mercenaria permagna	species	82724
subjective synonym of	Pitar morrhuanus	species	94532
	Mercenaria permagna	species	82724
subjective synonym of	Pitar morrhuanus	species	94532
	Mercenaria permagna	species	82724
	Mclellania aenigma	species	82264

misspelling of, subjective synonym of, species not entered	Mercenaria	genus	17892
	Mclellania aenigma	species	82264
misspelling of, subjective synonym of, species not entered	Mercenaria	genus	17892
species not entered	Mercenaria	genus	17892
species not entered	Mercenaria	genus	17892
misspelling of, subjective synonym of, species not entered	Mercenaria	genus	17892
species not entered	Mercenaria	genus	17892
misspelling of, subjective synonym of, species not entered	Mercenaria	genus	17892
species not entered	Mercenaria	genus	17892
misspelling of, subjective synonym of, species not entered	Mercenaria	genus	17892
recombined as	Dosinia (Dosinidia) ele	species	94558
	Dosinia discus	species	94591
recombined as	Pitar morrhuanus	species	94532
recombined as	Macrocallista (Macroc	species	109468
recombined as	Macrocallista (Macroc	species	109468
	Chione cancellata	species	94440
	Chione cancellata	species	94440
	Chione cancellata	species	94440
	Chione cancellata	species	94440
	Chione cancellata	species	94440
	Chione cancellata	species	94440
recombined as	Lirophora (Lirophora)	species	98377
	Gemma gemma	species	108167
	Gemma gemma	species	108167
	Gemma gemma	species	108167
	Mercenaria mercenar	species	95528
	Mercenaria mercenar	species	95528
	Mercenaria mercenar	species	95528
	Mercenaria mercenar	species	95528
	Mercenaria mercenar	species	95528
	Mercenaria mercenar	species	95528
	Mercenaria mercenar	species	95528
	Mercenaria mercenar	species	95528
	Mercenaria mercenar	species	95528
	Mercenaria campechi	species	94395
	Mercenaria campechi	species	94395
	Mercenaria campechi	species	94395
species not entered	Pitar	genus	18075
	Macrocallista (Macroc	species	109468
	Mercenaria mercenar	species	95528
	Chione	genus	17468
misspelling of, obsolete variant of	Transennella	genus	18348
recombined as	Venus (Mercenaria) ca	subspecies	95538
subjective synonym of	Pitar morrhuanus	species	94532
species not entered	Gouldia	genus	17708
	Gemma magna	species	95553
	Mercenaria campechi	species	94395

	Chione cribraria	species	95485
recombined as	Macrocallista (Macroc	species	95245
	Transennella stimpsor	species	94533
species not entered	Gouldia	genus	17708
	Mercenaria mercenar	species	95528
subjective synonym of	Globivenus	genus	17689
	Veneridae	family	62779
	Macrocallista (Macroc	species	95245
	Chione cribraria	species	95485
	Mercenaria campechi	species	94395
misspelling of, species not entered	Transennella	genus	18348
	Macrocallista (Macroc	species	95245
misspelling of, obsolete variant of	Transennella	genus	18348
	Chione	genus	17468
	Lirophora (Lirophora)	subgenus	90364
	Gemma magna	species	95553
	Gemma magna	species	95553
misspelling of, species not entered	Transennella	genus	18348
	Mercenaria campechi	species	94395
	Veneridae	family	62779
misspelling of, species not entered	Transennella	genus	18348
	Macrocallista (Macroc	species	95245
	Mercenaria campechi	species	94395
misspelling of, species not entered	Transennella	genus	18348
	Macrocallista (Macroc	species	95245
	Gemma magna	species	95553
	Chione cribraria	species	95485
misspelling of, species not entered	Transennella	genus	18348
	Chione cribraria	species	95485
species not entered	Gouldia	genus	17708
	Transennella stimpsor	species	94533
	Chione cribraria	species	95485
recombined as	Macrocallista (Macroc	species	95245
	Chione cribraria	species	95485
recombined as	Lirophora (Lirophora)	species	98377
recombined as	Macrocallista (Macroc	species	95245
	Gemma magna	species	95553
species not entered	Pitar	genus	18075
	Mercenaria campechi	species	94395
recombined as	Chione cancellata	species	94440
	Chione (Chione) cortir	species	95480
recombined as	Lirophora (Lirophora)	species	98377
species not entered	Pitar	genus	18075
	Lirophora	genus	17824
recombined as	Dosinia acetabulum	species	95240
	Macrocallista	genus	17856
	Gouldia	genus	17708

	Mercenaria	genus	17892
recombined as	Timoclea grus	species	95520
replaced by	Mercenaria druidi	species	99344
	Macrocallista (Macroc	subgenus	94610
	Lirophora	genus	17824
	Gouldia	genus	17708
	Chione cancellata	species	94440
	Timoclea grus	species	95520
	Mercenaria	genus	17892
	Gouldia	genus	17708
misspelling of, obsolete variant of	Transennella	genus	18348
	Chione cancellata	species	94440
	Dosinia acetabulum	species	95240
	Macrocallista (Macroc	species	95245
	Mercenaria	genus	17892
	Lirophora	genus	17824
recombined as	Timoclea grus	species	95520
	Gouldia (Paragouldia)	species	155061
recombined as	Mercenaria campechi	subspecies	95535
	Mercenaria permagna	species	82724
	Mercenaria permagna	species	82724
	Mercenaria campechi	species	94395
	Mercenaria mercenar	species	95528
misspelling of, recombined as	Pitar morrhuanus	species	94532
	Mercenaria mercenar	species	95528
species not entered	Gemma	genus	17681
	Mercenaria campechi	species	94395
	Mercenaria mercenar	species	95528
	Mercenaria campechi	species	94395
	Mercenaria campechi	species	94395
recombined as	Pitar morrhuanus	species	94532
	Dosinia discus	species	94591
	Mercenaria mercenar	species	95528
recombined as	Pitar morrhuanus	species	94532
species not entered	Mercenaria	genus	17892
recombined as	Mercenaria campechi	subspecies	95535
recombined as	Mercenaria campechi	subspecies	95541
	Amiantis	genus	17326
	Chione cribraria	species	95485
subjective synonym of	Pitar morrhuanus	species	94532
subjective synonym of	Pitar morrhuanus	species	94532
	Macrocallista	genus	17856
subjective synonym of	Pitar morrhuanus	species	94532
species not entered	Mercenaria	genus	17892
misspelling of, subjective synonym of, species not entered	Mercenaria	genus	17892
species not entered	Mercenaria	genus	17892
misspelling of, subjective synonym of, species not entered	Mercenaria	genus	17892

species not entered	Mercenaria	genus	17892
misspelling of, subjective synonym of, species not entered	Mercenaria	genus	17892
species not entered	Mercenaria	genus	17892
misspelling of, subjective synonym of, species not entered	Mercenaria	genus	17892
species not entered	Mercenaria	genus	17892
misspelling of, subjective synonym of, species not entered	Mercenaria	genus	17892
species not entered	Mercenaria	genus	17892
misspelling of, subjective synonym of, species not entered	Mercenaria	genus	17892
	Chione	genus	17468
	Mercenaria campechi	species	94395
	Mercenaria campechi	species	94395
species not entered	Mercenaria	genus	17892
species not entered	Mercenaria	genus	17892
	Dosinia acetabulum	species	95240
recombined as	Mercenaria campechi	subspecies	95541
subjective synonym of	Pitar morrhuanus	species	94532
recombined as	Mercenaria campechi	subspecies	95541
	Gemma magna	species	95553
	Dosinia acetabulum	species	95240
recombined as	Mercenaria campechi	subspecies	95541
	Mercenaria	genus	17892
	Gemma magna	species	95553
	Dosinia acetabulum	species	95240
	Gemma magna	species	95553
	Mercenaria	genus	17892
misspelling of, species not entered	Transennella	genus	18348
recombined as	Macrocallista (Macroc	species	95245
subjective synonym of	Pitar morrhuanus	species	94532
	Mercenaria	genus	17892
	Gemma magna	species	95553
	Dosinia acetabulum	species	95240
species not entered	Gouldia	genus	17708
recombined as	Macrocallista (Macroc	species	95245
	Mercenaria	genus	17892
	Dosinia acetabulum	species	95240
subjective synonym of	Pitar morrhuanus	species	94532
	Mercenaria	genus	17892
species not entered	Gouldia	genus	17708
	Mercenaria	genus	17892
	Gemma magna	species	95553
	Dosinia acetabulum	species	95240
recombined as	Macrocallista (Macroc	species	95245
	Mercenaria	genus	17892
	Dosinia acetabulum	species	95240
	Veneridae	family	62779
recombined as	Lirophora (Lirophora)	species	98377
recombined as	Dosinia acetabulum	species	95240

	Chione cancellata	species	94440
subjective synonym of	Pitar morrhuanus	species	94532
species not entered	Gouldia	genus	17708
	Mercenaria campechi	species	94395
	Mercenaria	genus	17892
	Chione cribraria	species	95485
subjective synonym of	Pitar morrhuanus	species	94532
	Pitar morrhuanus	species	94532
	Mercenaria mercenar	species	95528
species not entered	Dosinia	genus	17595
	Mercenaria campechi	species	94395
recombined as	Lirophora (Lirophora)	species	98377
	Mercenaria campechi	species	94395
	Mercenaria campechi	species	94395
recombined as	Dosinia acetabulum	species	95240
subjective synonym of, species not entered	Anomalocardia	genus	17341
recombined as	Trigonulina ornata	species	108209
species not entered	Trigonulina	genus	18838
species not entered	Trigonulina	genus	18838
species not entered	Verticordia	genus	18843
species not entered	Verticordia	genus	18843
species not entered	Trigonulina	genus	18838
species not entered	Trigonulina	genus	18838
species not entered	Yoldia	genus	16145
	Yoldia	genus	16145
	Yoldia	genus	16145
	Yoldia laevis	species	98555
	Yoldia limatula	species	87530
	Yoldia	genus	16145
species not entered	Yoldia	genus	16145
	Yoldia laevis	species	98555
	Yoldia laevis	species	98555
	Yoldia laevis	species	98555
	Yoldia laevis	species	98555
species not entered	Yoldia	genus	16145
	Yoldia limatula	species	87530
	Yoldia limatula	species	87530
	Yoldia limatula	species	87530
	Yoldia limatula	species	87530
	Yoldia limatula	species	87530
	Yoldia limatula	species	87530
	Yoldia laevis	species	98555
	Yoldia laevis	species	98555
	Yoldia laevis	species	98555
	Yoldia laevis	species	98555
	Yoldia laevis	species	98555
	Yoldia laevis	species	98555

	Yoldia laevis	species	98555
	Yoldia laevis	species	98555
	Yoldia laevis	species	98555
	Yoldia laevis	species	98555
	Yoldia laevis	species	98555
	Yoldia laevis	species	98555
	Yoldia limatula	species	87530
	Bivalvia	class	16005
species not entered	Cyrenoidea	superfamil	71675
	Bivalvia	class	16005
	Bivalvia	class	16005
species not entered	Cyrenoidea	superfamil	71675
	Bivalvia	class	16005
	Bivalvia	class	16005
	Bivalvia	class	16005
	Bivalvia	class	16005
	Bivalvia	class	16005
	Bivalvia	class	16005
	Bivalvia	class	16005
	Bivalvia	class	16005
	Bivalvia	class	16005
	Bivalvia	class	16005
	Bivalvia	class	16005
corrected to	Leptonoidea	superfamil	93685
	Bivalvia	class	16005
	Bivalvia	class	16005
species not entered	Sulcularia	genus	71753
species not entered	Sulcularia	genus	71753
species not entered	Sulcularia	genus	71753
species not entered	Sulcularia	genus	71753
recombined as	Japonactaeon punctos	species	90877
replaced by	Acteon	genus	58568
species not entered	Acteon	genus	58568
species not entered	Acteon	genus	58568
	Acteon	genus	58568
recombined as	Japonactaeon punctos	species	90877
species not entered	Rictaxis	genus	11995
species not entered	Rictaxis	genus	11995
species not entered	Japonactaeon	genus	156428
replaced by, species not entered	Acteon	genus	58568
species not entered	Aorotrema	genus	116503
species not entered	Aorotrema	genus	116503
	Fontigens	genus	121479
	Fontigens	genus	121479
species not entered	Amnicola	genus	81489
	Fontigens	genus	121479
	Fontigens	genus	121479

species not entered	Amnicola	genus	81489
species not entered	Amnicola	genus	81489
	Amnicola saltillensis	species	311057
	Amnicola georgiensis	species	311058
	Amnicola expansilabri	species	311059
species not entered	Pomacea	genus	251916
species not entered	Pomacea	genus	251916
species not entered	Pomacea	genus	251916
species not entered	Pomacea	genus	251916
species not entered	Pomacea	genus	251916
species not entered	Pomacea	genus	251916
species not entered	Pomacea	genus	251916
	Pachycrommium gupp	species	95679
	Architectonica nobilis	species	90235
subjective synonym of	Architectonica nobilis	species	90235
subjective synonym of	Architectonica nobilis	species	90235
subjective synonym of	Architectonica nobilis	species	90235
	Architectonica nobilis	species	90235
	Architectonica nobilis	species	90235
	Architectonica nobilis	species	90235
	Architectonica gemma	species	392478
	Architectonica gemma	species	392478
	Architectonica (Pseud	species	109079
recombined as	Architectonica (Pseud	species	109079
recombined as	Marevalvata tricarinat	species	156287
recombined as	Marevalvata tricarinat	species	156287
subjective synonym of	Marevalvata tricarinat	species	156287
subjective synonym of	Marevalvata tricarinat	species	156287
subjective synonym of	Marevalvata tricarinat	species	156287
subjective synonym of	Marevalvata tricarinat	species	156287
subjective synonym of	Marevalvata tricarinat	species	156287
subjective synonym of	Marevalvata tricarinat	species	156287
subjective synonym of	Marevalvata tricarinat	species	156287
subjective synonym of	Marevalvata tricarinat	species	156287
recombined as	Marevalvata tricarinat	species	156287
species not entered	Pyrazus	genus	10393
species not entered	Pyrazus	genus	10393
species not entered	Pyrazus	genus	10393
	Pyrazisinus ultimus	species	82782
species not entered	Pyrazus	genus	10393
species not entered	Pyrazisinus	genus	10392
	Batillaria minima	species	156621
species not entered	Pyrazisinus	genus	10392
species not entered	Pyrazisinus	genus	10392
species not entered	Pyrazisinus	genus	10392
species not entered	Pyrazisinus	genus	10392
species not entered	Pyrazisinus	genus	10392

species not entered	Pyrazisinus	genus	10392
species not entered	Pyrazisinus	genus	10392
species not entered	Pyrazisinus	genus	10392
species not entered	Pyrazisinus	genus	10392
obsolete variant of	Pyrazus (Pyrazisinus) s	species	93966
species not entered	Pyrazisinus	genus	10392
species not entered	Pyrazisinus	genus	10392
species not entered	Pyrazisinus	genus	10392
obsolete variant of	Pyrazus (Pyrazisinus) s	species	93966
species not entered	Pyrazisinus	genus	10392
	Pyrazisinus	genus	10392
species not entered	Pyrazisinus	genus	10392
	Batillaria minima	species	156621
species not entered	Pyrazisinus	genus	10392
species not entered	Pyrazisinus	genus	10392
	Pyrazisinus ultimus	species	82782
	Hanetia mengeana	species	164464
	Cantharus	genus	10894
species not entered	Cantharus	genus	10894
species not entered	Cantharus	genus	10894
	Hanetia mengeana	species	164464
species not entered	Hanetia	genus	11175
	Phos sloani	species	188145
recombined as	Antillophos candeanu	species	91530
recombined as	Solenosteira cancellar	species	91542
recombined as	Hesperisternia multar	species	91551
recombined as	Solenosteira cancellar	species	91542
	Bailya intricata	species	91135
	Monostiolum thomasi	species	93959
species not entered	Nassa	genus	11401
species not entered	Gemophos	genus	81260
subjective synonym of	Solenosteira cancellar	species	91542
recombined as	Hanetia mengeana	species	164464
species not entered	Engina	genus	11064
	Solenosteira cancellar	species	91542
	Metula roberti	species	93958
species not entered	Hindsia	genus	81261
species not entered	Hindsia	genus	81261
	Monostiolum petiti	species	93960
	Strombinophos maxw	species	104866
species not entered	Bailya	genus	10823
species not entered	Cymatophos	genus	11009
species not entered	Solenosteira	genus	11681
	Hesperisternia miamie	species	188171
subjective synonym of	Hesperisternia multar	species	91551
subjective synonym of	Hesperisternia multar	species	91551
	Hanetia mengeana	species	164464

species not entered	Solenosteira	genus	11681
species not entered	Buccinum	genus	10866
species not entered	Ptychosalpinx	genus	11599
	Cantharus	genus	10894
	Gemophos	genus	81260
species not entered	Pisania (Celatoconus)	subgenus	10904
	Bailya intricata	species	91135
recombined as	Hesperisternia multar	species	91551
	Gemophos tinctus	species	91539
	Solenosteira cancellar	species	91542
	Strombinophos	genus	11715
	Ptychosalpinx	genus	11599
recombined as	Gemophos tinctus	species	91539
recombined as	Gemophos tinctus	species	91539
	Phos sloani	species	188145
recombined as	Solenosteira cancellar	species	91542
recombined as	Solenosteira cancellar	species	91542
recombined as	Solenosteira cancellar	species	91542
recombined as	Solenosteira cancellar	species	91542
recombined as	Gemophos tinctus	species	91539
species not entered	Colubraria	genus	9673
species not entered	Ptychosalpinx	genus	11599
species not entered	Ptychosalpinx	genus	11599
species not entered	Ptychosalpinx	genus	11599
species not entered	Ptychosalpinx	genus	11599
species not entered	Pisania (Celatoconus)	subgenus	10904
species not entered	Pisania (Celatoconus)	subgenus	10904
recombined as	Solenosteira cancellar	species	91542
recombined as	Buccinum undatum	species	90912
	Colus pygmaeus	species	91558
recombined as	Solenosteira cancellar	species	91542
species not entered	Ptychosalpinx	genus	11599
species not entered	Pisania	genus	11526
species not entered	Ptychosalpinx	genus	11599
species not entered	Ptychosalpinx	genus	11599
species not entered	Ptychosalpinx	genus	11599
species not entered	Ptychosalpinx	genus	11599
	Ptychosalpinx	genus	11599
species not entered	Ptychosalpinx	genus	11599
species not entered	Pisania (Celatoconus)	subgenus	10904
subjective synonym of	Bulla striata	species	94556
	Bulla striata	species	94556
subjective synonym of	Bulla striata	species	94556
	Bulla striata	species	94556
subjective synonym of	Bulla striata	species	94556
subjective synonym of	Bulla striata	species	94556
	Bulla striata	species	94556

	Bulla striata	species	94556
	Bulla striata	species	94556
	Bulla striata	species	94556
	Bulla striata	species	94556
	Bulla	genus	11910
	Bulla striata	species	94556
subjective synonym of	Bulla striata	species	94556
subjective synonym of	Bulla striata	species	94556
species not entered	Bulla	genus	11910
subjective synonym of	Bulla striata	species	94556
subjective synonym of	Bulla striata	species	94556
	Bulla striata	species	94556
subjective synonym of	Bulla striata	species	94556
	Bulla striata	species	94556
	Bulla striata	species	94556
	Bulla striata	species	94556
subjective synonym of	Bulla striata	species	94556
	Bulla striata	species	94556
species not entered	Bulla	genus	11910
subjective synonym of	Bulla striata	species	94556
	Bursa rugosa	species	187875
subjective synonym of	Marsupina bufo	species	156556
	Marsupina bufo	species	156556
species not entered	Caecum	genus	9577
species not entered	Caecum	genus	9577
	Caecum imbricatum	species	101217
recombined as	Caecum (Caecum) reg	species	116029
subjective synonym of	Meioceras nitidum	species	156529
species not entered	Meioceras	genus	10110
species not entered	Caecum	genus	9577
species not entered	Caecum	genus	9577
species not entered	Caecum	genus	9577
	Caecum imbricatum	species	101217
recombined as	Caecum (Caecum) reg	species	116029
	Caecum	genus	9577
species not entered	Caecum	genus	9577
species not entered	Caecum	genus	9577
	Caecum imbricatum	species	101217
subjective synonym of	Meioceras nitidum	species	156529
	Caecum cooperi	species	157307
species not entered	Caecum	genus	9577
subjective synonym of	Caecum cycloferum	species	248850
recombined as	Caecum (Caecum) reg	species	116029
	Meioceras nitidum	species	156529
subjective synonym of	Meioceras nitidum	species	156529
species not entered	Caecum	genus	9577
	Caecum imbricatum	species	101217

species not entered	Caecum	genus	9577
subjective synonym of	Caecum circumvolutu	species	156713
	Caecum (Caecum) reg	species	116029
	Caecum (Caecum) reg	species	116029
species not entered	Caecum (Caecum)	subgenus	116028
species not entered	Caecum (Caecum)	subgenus	116028
	Caecum (Caecum) reg	species	116029
species not entered	Caecum (Caecum)	subgenus	116028
species not entered	Caecum (Caecum)	subgenus	116028
species not entered	Caecum (Caecum)	subgenus	116028
recombined as	Caecum imbricatum	species	101217
species not entered	Bambusum	genus	117488
	Meioceras nitidum	species	156529
subjective synonym of	Meioceras nitidum	species	156529
	Caecum imbricatum	species	101217
species not entered	Caecum	genus	9577
species not entered	Caecum	genus	9577
	Meioceras nitidum	species	156529
	Caecum imbricatum	species	101217
	Caecum cooperi	species	157307
species not entered	Caecum	genus	9577
species not entered	Caecum	genus	9577
recombined as	Caecum (Defolinia) fle	species	116033
	Caecum imbricatum	species	101217
species not entered	Caecum	genus	9577
	Caecum beaufortensis	species	82747
recombined as	Caecum (Defolinia) fle	species	116033
	Caecum imbricatum	species	101217
recombined as	Caecum (Defolinia) fle	species	116033
recombined as	Caecum (Caecum) reg	species	116029
recombined as	Caecum (Caecum) reg	species	116029
species not entered	Caecum	genus	9577
species not entered	Caecum	genus	9577
	Caecum cooperi	species	157307
	Crepidula fornicata	species	94397
	Crepidula plana	species	81481
	Calyptraea	genus	9583
	Crucibulum	genus	9704
	Crepidula maculosa	species	95417
recombined as	Crucibulum (Crucibulu	species	95427
recombined as	Bostrycapulus aculeat	species	81484
recombined as	Bostrycapulus aculeat	species	81484
recombined as	Crucibulum (Crucibulu	species	95427
	Calyptraea centralis	species	81409
recombined as	Bostrycapulus aculeat	species	81484
recombined as	Crucibulum (Crucibulu	species	95427
recombined as	Bostrycapulus aculeat	species	81484

	Crepidula maculosa	species	95417
	Crepidula plana	species	81481
recombined as	Crucibulum (Crucibulu	species	95427
species not entered	Crucibulum	genus	9704
	Crucibulum	genus	9704
recombined as	Bostrycapulus aculeat	species	81484
	Crepidula maculosa	species	95417
	Crepidula plana	species	81481
recombined as	Crucibulum (Crucibulu	species	95427
	Crepidula maculosa	species	95417
recombined as	Bostrycapulus aculeat	species	81484
	Crepidula maculosa	species	95417
recombined as	Crucibulum (Crucibulu	species	95427
	Crepidula plana	species	81481
recombined as	Bostrycapulus aculeat	species	81484
	Crepidula fornicata	species	94397
	Crepidula plana	species	81481
species not entered	Crucibulum	genus	9704
	Crucibulum multilinea	species	97656
recombined as	Bostrycapulus aculeat	species	81484
recombined as	Bostrycapulus aculeat	species	81484
	Crepidula plana	species	81481
	Crepidula plana	species	81481
	Crepidula fornicata	species	94397
species not entered	Crepidula	genus	9697
species not entered	Crepidula	genus	9697
recombined as	Crucibulum (Crucibulu	species	95427
recombined as	Crucibulum (Crucibulu	species	95427
recombined as	Crucibulum (Crucibulu	species	95427
recombined as	Crucibulum (Dispotae	species	94740
	Crucibulum multilinea	species	97656
	Crucibulum multilinea	species	97656
	Calyptraea centralis	species	81409
	Calyptraea centralis	species	81409
	Calyptraea centralis	species	81409
recombined as	Bostrycapulus aculeat	species	81484
	Crepidula fornicata	species	94397
	Crepidula maculosa	species	95417
	Crepidula plana	species	81481
recombined as	Bostrycapulus aculeat	species	81484
	Crepidula fornicata	species	94397
	Crepidula plana	species	81481
	Crepidula maculosa	species	95417
	Calyptraea centralis	species	81409
recombined as	Bostrycapulus aculeat	species	81484
	Crepidula fornicata	species	94397
	Crepidula maculosa	species	95417

	Crepidula plana	species	81481
	Crepidula plana	species	81481
	Crepidula fornicata	species	94397
	Crepidula fornicata	species	94397
	Crepidula plana	species	81481
	Calyptraea centralis	species	81409
	Crepidula fornicata	species	94397
	Crepidula fornicata	species	94397
species not entered	Crepidula	genus	9697
	Crepidula fornicata	species	94397
	Crucibulum (Crucibulu	subgenus	92422
	Crepidula fornicata	species	94397
	Crepidula plana	species	81481
	Bostrycapulus aculeat	species	81484
species not entered	Crucibulum (Dispotae	subgenus	9772
	Crepidula fornicata	species	94397
species not entered	Crucibulum (Crucibulu	subgenus	92422
recombined as	Crucibulum multilinea	species	97656
	Crepidula fornicata	species	94397
	Crepidula fornicata	species	94397
	Calyptraea centralis	species	81409
	Crucibulum (Dispotae	subgenus	9772
	Crepidula plana	species	81481
species not entered	Crucibulum	genus	9704
	Crepidula fornicata	species	94397
	Crepidula plana	species	81481
	Crepidula fornicata	species	94397
	Crepidula fornicata	species	94397
subjective synonym of	Bostrycapulus aculeat	species	81484
	Crucibulum	genus	9704
	Crepidula fornicata	species	94397
subjective synonym of	Bostrycapulus aculeat	species	81484
	Crepidula fornicata	species	94397
	Crepidula plana	species	81481
	Crucibulum	genus	9704
subjective synonym of	Bostrycapulus aculeat	species	81484
species not entered	Crepidula	genus	9697
	Crepidula plana	species	81481
recombined as	Bostrycapulus aculeat	species	81484
species not entered	Crucibulum (Dispotae	subgenus	9772
species not entered	Crucibulum	genus	9704
species not entered	Crucibulum (Dispotae	subgenus	9772
	Calyptraea centralis	species	81409
recombined as	Bostrycapulus aculeat	species	81484
	Crepidula plana	species	81481
	Calyptraea centralis	species	81409
	Crepidula maculosa	species	95417

species not entered	Crepidula (Crepidula)	subgenus	95456
species not entered	Crepidula (Crepidula)	subgenus	95456
recombined as	Crepidula plana	species	81481
recombined as	Bostrycapulus aculeat	species	81484
species not entered	Crepidula (Crepidula)	subgenus	95456
	Calyptraea centralis	species	81409
	Crepidula fornicata	species	94397
	Crepidula fornicata	species	94397
	Calyptraea (Trochita)	species	158722
recombined as	Crucibulum (Crucibulu	species	95427
	Bostrycapulus aculeat	species	81484
	Calyptraea centralis	species	81409
	Crepidula convexa	species	94519
	Crepidula fornicata	species	94397
	Crepidula maculosa	species	95417
	Crepidula plana	species	81481
recombined as	Crucibulum (Crucibulu	species	95427
recombined as	Calyptraea (Trochita)	species	158722
species not entered	Crucibulum (Dispotae	subgenus	9772
	Crucibulum spinosum	species	402832
	Crucibulum multilinea	species	97656
species not entered	Crepidula	genus	9697
species not entered	Crepidula	genus	9697
recombined as	Maoricrypta (Maoricry	species	90749
	Crepidula cymbaeforn	species	95440
species not entered	Crepidula	genus	9697
species not entered	Crepidula	genus	9697
	Calyptraea centralis	species	81409
	Crepidula fornicata	species	94397
	Crepidula fornicata	species	94397
	Crepidula fornicata	species	94397
	Crepidula fornicata	species	94397
	Crepidula fornicata	species	94397
	Crepidula plana	species	81481
	Crepidula fornicata	species	94397
	Crepidula plana	species	81481
recombined as	Bostrycapulus aculeat	species	81484
	Calyptraea centralis	species	81409
species not entered	Crucibulum	genus	9704
	Crepidula fornicata	species	94397
	Crepidula fornicata	species	94397
	Crepidula plana	species	81481
	Calyptraea centralis	species	81409
	Crepidula fornicata	species	94397
	Crepidula fornicata	species	94397
	Crepidula plana	species	81481
	Crepidula convexa	species	94519

recombined as	Bostrycapulus aculeat	species	81484
	Crepidula plana	species	81481
	Crepidula fornicata	species	94397
	Crepidula	genus	9697
	Crucibulum (Crucibulu	subgenus	92422
	Crepidula plana	species	81481
	Crepidula plana	species	81481
	Crepidula fornicata	species	94397
	Calyptraea centralis	species	81409
	Crepidula fornicata	species	94397
recombined as	Bostrycapulus aculeat	species	81484
	Crepidula fornicata	species	94397
	Calyptraea centralis	species	81409
	Crepidula fornicata	species	94397
recombined as	Crepidula fornicata	species	94397
recombined as	Bostrycapulus aculeat	species	81484
	Calyptraea centralis	species	81409
recombined as	Bostrycapulus aculeat	species	81484
	Crepidula fornicata	species	94397
	Crepidula plana	species	81481
	Crucibulum lawrencei	species	82760
	Crepidula fornicata	species	94397
recombined as	Bostrycapulus aculeat	species	81484
	Crepidula fornicata	species	94397
	Crepidula plana	species	81481
species not entered	Crepidula	genus	9697
	Crepidula fornicata	species	94397
	Crepidula fornicata	species	94397
	Crepidula fornicata	species	94397
	Crepidula convexa	species	94519
	Crepidula plana	species	81481
	Crepidula fornicata	species	94397
	Crepidula convexa	species	94519
	Crepidula plana	species	81481
	Crepidula	genus	9697
	Crepidula fornicata	species	94397
	Crepidula convexa	species	94519
	Crepidula plana	species	81481
	Crepidula fornicata	species	94397
	Crepidula convexa	species	94519
	Crepidula plana	species	81481
	Crepidula fornicata	species	94397
	Crepidula fornicata	species	94397
	Crepidula fornicata	species	94397
	Crepidula fornicata	species	94397
	Crepidula plana	species	81481
	Crepidula plana	species	81481

	Crepidula plana	species	81481
	Crepidula plana	species	81481
	Crepidula convexa	species	94519
	Crepidula convexa	species	94519
	Crepidula convexa	species	94519
	Crepidula convexa	species	94519
	Crepidula convexa	species	94519
	Crepidula fornicata	species	94397
	Crepidula plana	species	81481
	Crepidula fornicata	species	94397
	Crepidula plana	species	81481
	Crucibulum multilinea	species	97656
recombined as	Bostrycapulus aculeat	species	81484
	Crepidula fornicata	species	94397
	Crepidula plana	species	81481
	Crepidula fornicata	species	94397
	Calyptraea	genus	9583
	Crucibulum	genus	9704
	Calyptraea	genus	9583
	Crepidula fornicata	species	94397
	Crepidula plana	species	81481
species not entered	Crepidula	genus	9697
	Crucibulum (Dispotae	species	94740
subjective synonym of	Bostrycapulus aculeat	species	81484
	Calyptraea centralis	species	81409
	Crepidula fornicata	species	94397
	Crucibulum	genus	9704
	Crucibulum	genus	9704
	Calyptraea centralis	species	81409
recombined as	Bostrycapulus aculeat	species	81484
	Crepidula fornicata	species	94397
	Crepidula fornicata	species	94397
	Crucibulum	genus	9704
	Crepidula fornicata	species	94397
	Crepidula plana	species	81481
	Bostrycapulus aculeat	species	81484
	Crucibulum (Crucibulu	subgenus	92422
	Crepidula fornicata	species	94397
	Crepidula plana	species	81481
	Bostrycapulus aculeat	species	81484
	Crucibulum	genus	9704
	Crepidula fornicata	species	94397
	Bostrycapulus aculeat	species	81484
	Crepidula plana	species	81481
	Crepidula fornicata	species	94397
	Crepidula fornicata	species	94397
	Crepidula fornicata	species	94397

	Crepidula fornicata	species	94397
	Crepidula convexa	species	94519
	Crepidula convexa	species	94519
	Crepidula fornicata	species	94397
	Crepidula plana	species	81481
	Crepidula convexa	species	94519
	Crepidula fornicata	species	94397
	Crepidula plana	species	81481
	Crepidula fornicata	species	94397
	Crepidula plana	species	81481
	Crepidula convexa	species	94519
	Crepidula plana	species	81481
	Crepidula fornicata	species	94397
	Crepidula fornicata	species	94397
	Crepidula convexa	species	94519
subjective synonym of	Bostrycapulus aculeat	species	81484
	Crepidula plana	species	81481
	Crepidula fornicata	species	94397
recombined as	Crucibulum (Dispotae	species	94740
	Crepidula plana	species	81481
	Crepidula fornicata	species	94397
subjective synonym of	Bostrycapulus aculeat	species	81484
	Crepidula plana	species	81481
	Crepidula fornicata	species	94397
subjective synonym of	Bostrycapulus aculeat	species	81484
recombined as	Crucibulum (Dispotae	species	94740
recombined as	Crucibulum (Dispotae	species	94740
subjective synonym of	Bostrycapulus aculeat	species	81484
	Crepidula fornicata	species	94397
	Crepidula plana	species	81481
	Crepidula fornicata	species	94397
recombined as	Crucibulum (Dispotae	species	94740
subjective synonym of	Bostrycapulus aculeat	species	81484
subjective synonym of	Bostrycapulus aculeat	species	81484
	Crepidula plana	species	81481
	Crepidula fornicata	species	94397
recombined as	Crucibulum (Dispotae	species	94740
subjective synonym of	Bostrycapulus aculeat	species	81484
recombined as	Crucibulum (Dispotae	species	94740
subjective synonym of	Bostrycapulus aculeat	species	81484
	Crepidula plana	species	81481
	Crepidula fornicata	species	94397
species not entered	Crucibulum	genus	9704
recombined as	Crucibulum (Dispotae	species	94740
subjective synonym of	Bostrycapulus aculeat	species	81484
	Crepidula plana	species	81481
	Crepidula fornicata	species	94397

recombined as	Crucibulum (Dispotae	species	94740
subjective synonym of	Bostrycapulus aculeat	species	81484
	Crepidula fornicata	species	94397
recombined as	Crucibulum (Dispotae	species	94740
subjective synonym of	Bostrycapulus aculeat	species	81484
subjective synonym of	Bostrycapulus aculeat	species	81484
	Crucibulum (Dispotae	species	94740
species not entered	Crepidula	genus	9697
	Crucibulum (Dispotae	species	94740
subjective synonym of	Bostrycapulus aculeat	species	81484
	Crepidula fornicata	species	94397
	Bostrycapulus aculeat	species	81484
species not entered	Crepidula	genus	9697
recombined as	Bostrycapulus aculeat	species	81484
	Crucibulum (Crucibulu	subgenus	92422
recombined as	Bostrycapulus aculeat	species	81484
	Crucibulum	genus	9704
species not entered	Thericium	genus	10574
species not entered	Thericium	genus	10574
species not entered	Thericium	genus	10574
species not entered	Thericium	genus	10574
species not entered	Thericium	genus	10574
species not entered	Thericium	genus	10574
species not entered	Thericium	genus	10574
species not entered	Thericium	genus	10574
species not entered	Thericium	genus	10574
species not entered	Thericium	genus	10574
species not entered	Thericium	genus	10574
species not entered	Thericium	genus	10574
	Thericium	genus	10574
	Cancellaria conradiana	species	107354
recombined as	Tritonoharpa lanceola	species	91555
	Cancellaria conradiana	species	107354
	Cancellaria conradiana	species	107354
	Cancellaria	genus	10889
	Cancellaria tabulata	species	120336
recombined as	Massyla venusta	species	93811
recombined as	Tritonoharpa lanceola	species	91555
	Cancellaria tabulata	species	120336
	Cancellaria tabulata	species	120336
	Cancellaria tabulata	species	120336
	Cancellaria reticulata	species	107351
recombined as	Massyla propevenusta	species	107749
recombined as	Ventrilia tenerum	species	120364
	Cancellaria reticulata	species	107351
	Cancellaria (Trigonost	species	97422
recombined as	Bivetopsia rugosa	species	91137

	Cancellaria tabulata	species	120336
recombined as	Massyla venusta	species	93811
	Cancellaria tabulata	species	120336
	Cancellaria conradiana	species	107354
recombined as	Massyla venusta	species	93811
recombined as	Cancellaria conradiana	species	107354
recombined as	Massyla venusta	species	93811
	Perplicaria perplexa	species	250430
recombined as	Ventrilia tenerum	species	120364
species not entered	Trigonostoma	genus	11783
recombined as	Cancellaria conradiana	species	107354
recombined as	Cancellaria conradiana	species	107354
obsolete variant of	Cancellaria amoena	species	120332
	Ventrilia rucksorum	species	167563
	Cancellaria (Massyla) :	species	93810
	Trigonostoma (Extract	species	93815
	Trigonostoma (Emmo	species	93817
	Perplicaria perplexa	species	250430
	Trigonostoma (Extract	species	93815
	Trigonostoma (Emmo	species	93819
recombined as	Massyla propevenusta	species	107749
species not entered	Trigonostoma	genus	11783
recombined as	Massyla propevenusta	species	107749
species not entered	Cancellaria	genus	10889
species not entered	Cancellaria	genus	10889
	Cancellaria conradiana	species	107354
	Perplicaria perplexa	species	250430
	Cancellaria conradiana	species	107354
species not entered	Cancellaria	genus	10889
species not entered	Cancellaria	genus	10889
subjective synonym of	Ventrilia rucksorum	species	167563
	Cancellaria reticulata	species	107351
species not entered	Ventrilia	genus	11834
species not entered	Ventrilia	genus	11834
recombined as	Tritonoharpa lanceola	species	91555
species not entered	Ventrilia	genus	11834
species not entered	Trigonostoma	genus	11783
species not entered	Ventrilia	genus	11834
species not entered	Trigonostoma (Extract	subgenus	93813
	Cancellaria rotunda	species	108726
	Cancellaria	genus	10889
	Agatrix	genus	10731
	Cancellaria	genus	10889
	Cancellaria rotunda	species	108726
	Cancellaria rotunda	species	108726
species not entered	Trigonostoma	genus	11783
recombined as	Massyla propevenusta	species	107749

recombined as	Massyla propevenusta	species	107749
species not entered	Trigonostoma	genus	11783
	Trigonostoma	genus	11783
	Cancellaria reticulata	species	107351
reassigned as	Trigonostoma (Extract	subgenus	93813
	Massyla	genus	11328
	Cancellaria	genus	10889
reassigned as	Trigonostoma (Extract	subgenus	93813
	Massyla	genus	11328
subjective synonym of	Ventrilia	genus	11834
	Trigonostoma	genus	11783
recombined as	Cancellaria rotunda	species	108726
	Cancellaria reticulata	species	107351
	Cancellaria reticulata	species	107351
	Cancellaria reticulata	species	107351
recombined as	Cancellaria rotunda	species	108726
	Trigonostoma	genus	11783
	Cancellaria rotunda	species	108726
	Trigonostoma	genus	11783
	Trigonostoma	genus	11783
	Cancellaria	genus	10889
	Cancellariidae	family	59518
	Capulus	genus	9594
misspelling of, recombined as	Sconsia hodgii	species	250598
species not entered	Phalium	genus	10270
	Cassis madagascariens	species	134864
species not entered	Cassis	genus	9604
species not entered	Cassis	genus	9604
subjective synonym of	Semicassis granulata	species	156567
	Cassis ketteri	species	251416
subjective synonym of	Sconsia hodgii	species	250598
	Cassis floridensis	species	251404
misspelling of, recombined as	Sconsia hodgii	species	250598
	Cassis ketteri	species	251416
	Cassis floridensis	species	251404
	Sconsia prolongata	species	250632
	Semicassis granulata	species	156567
	Semicassis granulata	species	156567
	Cassis madagascariens	species	134864
recombined as	Semicassis granulata	species	156567
misspelling of, recombined as	Sconsia hodgii	species	250598
misspelling of, recombined as	Sconsia hodgii	species	250598
misspelling of, recombined as	Sconsia hodgii	species	250598
subjective synonym of	Cerithium atratum	species	109115
	Cerithium	genus	9632
	Cerithium muscarum	species	94534
subjective synonym of	Cerithium eburneum	species	109149

species not entered	Alabina	genus	9441
species not entered	Bittium	genus	9553
species not entered	Bittium	genus	9553
recombined as	Bittiolum varium	species	156525
	Bittium	genus	9553
species not entered	Cerithium	genus	9632
species not entered	Cerithium	genus	9632
	Cerithium	genus	9632
recombined as	Bittiolum varium	species	156525
	Bittium	genus	9553
species not entered	Cerithium	genus	9632
	Cerithium	genus	9632
subjective synonym of	Cerithium atratum	species	109115
species not entered	Rhinoclavis	genus	62110
recombined as	Bittiolum varium	species	156525
species not entered	Cerithium	genus	9632
species not entered	Cerithium	genus	9632
	Cerithium	genus	9632
	Cerithium muscarum	species	94534
species not entered	Cerithium	genus	9632
species not entered	Cerithium	genus	9632
subjective synonym of	Cerithium atratum	species	109115
species not entered	Rhinoclavis	genus	62110
	Rhinoclavis	genus	62110
species not entered	Cerithium	genus	9632
subjective synonym of	Cerithium atratum	species	109115
species not entered	Cerithium	genus	9632
	Cerithium	genus	9632
recombined as	Bittiolum varium	species	156525
	Cerithium	genus	9632
	Cerithium muscarum	species	94534
	Cerithium muscarum	species	94534
	Cerithium muscarum	species	94534
obsolete variant of	Cerithium	genus	9632
obsolete variant of	Cerithium	genus	9632
	Cerithium muscarum	species	94534
species not entered	Cerithium	genus	9632
	Cerithium	genus	9632
species not entered	Cerithium	genus	9632
species not entered	Cerithium	genus	9632
	Cerithium	genus	9632
species not entered	Cerithium	genus	9632
species not entered	Cerithium	genus	9632
	Cerithium muscarum	species	94534
subjective synonym of	Cerithium eburneum	species	109149
subjective synonym of	Cerithium atratum	species	109115
species not entered	Cerithioclava	genus	9623

species not entered	Cerithioclava	genus	9623
obsolete variant of	Cerithium muscarum	species	94534
obsolete variant of	Cerithium muscarum	species	94534
obsolete variant of	Cerithium muscarum	species	94534
species not entered	Cerithioclava	genus	9623
subjective synonym of, species not entered	Cerithium	genus	9632
	Cerithium dominicens	species	250661
species not entered	Cerithium	genus	9632
	Cerithium muscarum	species	94534
	Cerithium muscarum	species	94534
	Cerithium muscarum	species	94534
	Rhinoclavis	genus	62110
	Cerithium	genus	9632
recombined as	Cerithium atratum	species	109115
species not entered	Cerithioclava	genus	9623
	Cerithium	genus	9632
recombined as	Cerithium atratum	species	109115
subjective synonym of	Cerithium eburneum	species	109149
	Cerithium	genus	9632
	Cerithium eburneum	species	109149
subjective synonym of	Cerithium atratum	species	109115
obsolete variant of	Cerithium muscarum	species	94534
obsolete variant of	Cerithium muscarum	species	94534
obsolete variant of	Cerithium muscarum	species	94534
species not entered	Cerithium	genus	9632
species not entered	Cerithium	genus	9632
species not entered	Cerithium	genus	9632
species not entered	Rhinoclavis (Ochetocl:	subgenus	10203
species not entered	Bittiolum	genus	9551
species not entered	Rhinoclavis (Ochetocl:	subgenus	10203
obsolete variant of	Cerithium muscarum	species	94534
species not entered	Cerithium	genus	9632
	Bittiolum varium	species	156525
	Cerithium atratum	species	109115
	Cerithium eburneum	species	109149
	Cerithium guinaicum	species	109318
	Cerithium lutosum	species	109324
	Cerithium muscarum	species	94534
	Bittium	genus	9553
obsolete variant of	Rhinoclavis (Ochetocl:	subgenus	10203
	Cerithioclava	genus	9623
species not entered	Bittium	genus	9553
species not entered	Bittium	genus	9553
	Cerithium eburneum	species	109149
recombined as	Bittiolum alternatum	species	109165
	Retilaskeya bicolor	species	134871
recombined as	Seila adamsii	species	90919

recombined as	Seila adamsii	species	90919
obsolete variant of	Cerithiopsis	genus	9630
obsolete variant of	Cerithiopsis	genus	9630
obsolete variant of	Cerithiopsis	genus	9630
species not entered	Seila	genus	10445
species not entered	Seila	genus	10445
recombined as	Seila adamsii	species	90919
recombined as	Seila adamsii	species	90919
	Retilaskeya bicolor	species	134871
recombined as	Seila adamsii	species	90919
recombined as	Seila adamsii	species	90919
recombined as	Seila adamsii	species	90919
species not entered	Cerithiopsis (Cerithiop	subgenus	90926
recombined as	Cerithiopsis vinca	species	90927
recombined as	Cerithiopsis aralia	species	157328
species not entered	Cyrbasia (Joculator)	subgenus	60743
species not entered	Cyrbasia (Joculator)	subgenus	60743
species not entered	Cyrbasia (Joculator)	subgenus	60743
	Retilaskeya bicolor	species	134871
recombined as	Seila adamsii	species	90919
misspelling of, recombined as	Cerithiopsis greenii	species	90925
	Seila adamsii	species	90919
recombined as	Seila adamsii	species	90919
recombined as	Seila adamsii	species	90919
subjective synonym of	Horologica pupa	species	109783
	Cerithiopsis emersoni	species	90923
recombined as	Seila adamsii	species	90919
recombined as	Seila adamsii	species	90919
recombined as	Seila adamsii	species	90919
recombined as	Seila adamsii	species	90919
recombined as	Seila adamsii	species	90919
recombined as	Seila adamsii	species	90919
	Retilaskeya bicolor	species	134871
	Retilaskeya bicolor	species	134871
recombined as	Seila adamsii	species	90919
	Cerithiopsis vinca	species	90927
recombined as	Seila adamsii	species	90919
	Retilaskeya bicolor	species	134871
	Cerithiopsis vinca	species	90927
recombined as	Seila adamsii	species	90919
recombined as	Seila adamsii	species	90919
recombined as	Seila adamsii	species	90919
	Retilaskeya bicolor	species	134871
	Seila adamsii	species	90919
misspelling of, recombined as	Cerithiopsis greenii	species	90925
misspelling of, recombined as	Cerithiopsis greenii	species	90925
misspelling of, recombined as	Cerithiopsis greenii	species	90925

recombined as	Seila adamsii	species	90919
	Seila adamsii	species	90919
	Seila adamsii	species	90919
	Seila adamsii	species	90919
	Seila adamsii	species	90919
	Seila adamsii	species	90919
species not entered	Glyphostoma	genus	11160
species not entered	Glyphostoma	genus	11160
	Nannodiella	genus	11398
species not entered	Glyphostoma	genus	11160
species not entered	Glyphostoma	genus	11160
species not entered	Glyphostoma	genus	11160
species not entered	Glyphostoma	genus	11160
species not entered	Glyphostoma	genus	11160
species not entered	Glyphostoma	genus	11160
species not entered	Glyphostoma	genus	11160
recombined as	Glyphostoma (Rhiglyp	species	162807
species not entered	Glyphostomops	genus	121446
species not entered	Nannodiella	genus	11398
	Glyphostoma	genus	11160
species not entered	Glyphostoma	genus	11160
species not entered	Glyphostoma	genus	11160
species not entered	Glyphostoma	genus	11160
	Clavatula cornelliana	species	108732
	Clavatula cornelliana	species	108732
	Clavatula cornelliana	species	108732
recombined as	Pyrgospira ostrearum	species	120664
recombined as	Pyrgospira ostrearum	species	120664
recombined as	Pyrgospira ostrearum	species	120664
recombined as	Pyrgospira tampaensis	species	156523
recombined as	Pyrgospira acurugata	species	91225
recombined as	Pyrgospira tampaensis	species	156523
	Pyrgospira tampaensis	species	156523
recombined as	Costoanachis avara	species	135663
species not entered	Anachis	genus	10755
recombined as	Mitrella gardnerae	species	82768
species not entered	Anachis	genus	10755
recombined as	Mitrella gardnerae	species	82768
	Anachis	genus	10755
recombined as	Mitrella gardnerae	species	82768
species not entered	Anachis	genus	10755
species not entered	Anachis	genus	10755
species not entered	Anachis	genus	10755
species not entered	Anachis	genus	10755
obsolete variant of	Columbella	genus	10947
species not entered	Anachis	genus	10755
species not entered	Anachis	genus	10755

species not entered	Strombina	genus	11712
species not entered	Anachis	genus	10755
species not entered	Strombina	genus	11712
recombined as	Astyris lunata	species	94413
recombined as	Parvanachis obesa	species	94408
	Anachis	genus	10755
	Anachis	genus	10755
recombined as	Astyris lunata	species	94413
recombined as	Parvanachis obesa	species	94408
recombined as	Astyris lunata	species	94413
	Strombina (Strombina	subgenus	89604
obsolete variant of	Anachis (Costoanachis	subgenus	10978
	Mitrella	genus	11368
	Mitrella	genus	11368
	Columbella rusticoide	species	88864
species not entered	Sincola	genus	11670
species not entered	Strombina	genus	11712
	Columbella rusticoide	species	88864
species not entered	Eurypyrene	genus	11105
recombined as	Parvanachis obesa	species	94408
species not entered	Anachis (Costoanachis	subgenus	10978
species not entered	Anachis (Costoanachis	subgenus	10978
species not entered	Anachis (Costoanachis	subgenus	10978
species not entered	Anachis	genus	10755
recombined as	Mitrella gardnerae	species	82768
recombined as	Astyris lunata	species	94413
subjective synonym of	Columbellopsis nyctei	species	134952
subjective synonym of	Nassarina glypta	species	156597
species not entered	Aesopus	genus	10724
species not entered	Aesopus	genus	10724
species not entered	Anachis	genus	10755
species not entered	Aesopus	genus	10724
species not entered	Parametaria	genus	11481
species not entered	Parametaria	genus	11481
	Columbella submercat	species	88863
	Columbella rusticoide	species	88864
	Columbella rusticoide	species	88864
obsolete variant of	Anachis (Costoanachis	subgenus	10978
obsolete variant of	Anachis (Costoanachis	subgenus	10978
species not entered	Anachis	genus	10755
species not entered	Anachis	genus	10755
	Columbella rusticoide	species	88864
	Eurypyrene	genus	11105
	Columbella rusticoide	species	88864
species not entered	Sincola	genus	11670
	Astyris lunata	species	94413
	Columbella mercatori	species	109113

	Columbella rusticoide	species	88864
	Costoanachis avara	species	135663
	Nitidella nitida	species	88921
	Parvanachis obesa	species	94408
species not entered	Parametaria	genus	11481
recombined as	Astyris lunata	species	94413
recombined as	Costoanachis avara	species	135663
recombined as	Costoanachis avara	species	135663
recombined as	Costoanachis avara	species	135663
recombined as	Astyris lunata	species	94413
recombined as	Costoanachis avara	species	135663
recombined as	Astyris lunata	species	94413
recombined as	Costoanachis avara	species	135663
species not entered	Mitrella	genus	11368
recombined as	Parvanachis obesa	species	94408
recombined as	Parvanachis obesa	species	94408
recombined as	Parvanachis obesa	species	94408
recombined as	Costoanachis avara	species	135663
recombined as	Costoanachis avara	species	135663
recombined as	Astyris lunata	species	94413
recombined as	Astyris lunata	species	94413
	Mitrella	genus	11368
recombined as	Costoanachis avara	species	135663
recombined as	Astyris lunata	species	94413
	Columbella ornata	species	392386
	Aesopus gardnerae	species	82800
species not entered	Aesopus	genus	10724
species not entered	Anachis	genus	10755
	Aesopus gardnerae	species	82800
species not entered	Aesopus	genus	10724
species not entered	Anachis	genus	10755
species not entered	Mitrella	genus	11368
recombined as	Astyris lunata	species	94413
recombined as	Astyris lunata	species	94413
recombined as	Astyris lunata	species	94413
recombined as	Astyris lunata	species	94413
recombined as	Astyris lunata	species	94413
recombined as	Astyris lunata	species	94413
species not entered	Mitrella	genus	11368
species not entered	Metulella (Thiarinella)	subgenus	11770
species not entered	Metulella (Thiarinella)	subgenus	11770
recombined as	Costoanachis avara	species	135663
recombined as	Costoanachis avara	species	135663
recombined as	Costoanachis avara	species	135663
recombined as	Costoanachis avara	species	135663
recombined as	Costoanachis avara	species	135663
recombined as	Costoanachis avara	species	135663

species not entered	Anachis (Costoanachis	subgenus	10978
recombined as	Sincola anomala	species	237706
species not entered	Aesopus	genus	10724
species not entered	Aesopus	genus	10724
species not entered	Aesopus	genus	10724
recombined as	Mitrella gardnerae	species	82768
recombined as	Cotonopsis (Cotonops	species	156798
recombined as	Parvanachis obesa	species	94408
	Anachis	genus	10755
recombined as	Astyris lunata	species	94413
recombined as	Cotonopsis (Cotonops	species	156798
recombined as	Parvanachis obesa	species	94408
recombined as	Astyris lunata	species	94413
recombined as	Cotonopsis (Cotonops	species	156798
recombined as	Parvanachis obesa	species	94408
recombined as	Astyris lunata	species	94413
recombined as	Cotonopsis (Cotonops	species	156798
recombined as	Parvanachis obesa	species	94408
recombined as	Astyris lunata	species	94413
recombined as	Parvanachis obesa	species	94408
recombined as	Parvanachis obesa	species	94408
recombined as	Costoanachis avara	species	135663
recombined as	Costoanachis avara	species	135663
recombined as	Costoanachis avara	species	135663
recombined as	Astyris lunata	species	94413
recombined as	Astyris lunata	species	94413
recombined as	Astyris lunata	species	94413
recombined as	Astyris lunata	species	94413
recombined as	Costoanachis avara	species	135663
species not entered	Anachis	genus	10755
	Strombina	genus	11712
species not entered	Anachis (Costoanachis	subgenus	10978
	Strombina	genus	11712
recombined as	Cotonopsis (Cotonops	species	156798
recombined as	Parvanachis obesa	species	94408
recombined as	Cotonopsis (Cotonops	species	156798
recombined as	Parvanachis obesa	species	94408
recombined as	Parvanachis obesa	species	94408
recombined as	Cotonopsis (Cotonops	species	156798
recombined as	Parvanachis obesa	species	94408
recombined as	Cotonopsis (Cotonops	species	156798
recombined as	Astyris lunata	species	94413
recombined as	Cotonopsis (Cotonops	species	156798
recombined as	Parvanachis obesa	species	94408
recombined as	Cotonopsis (Cotonops	species	156798
	Astyris lunata	species	94413
	Parvanachis obesa	species	94408

	Astyris lunata	species	94413
	Mitrella	genus	11368
species not entered	Anachis	genus	10755
recombined as	Astyris communis	species	97536
	Anachis	genus	10755
recombined as	Astyris communis	species	97536
	Mitrella	genus	11368
recombined as	Astyris communis	species	97536
	Mitrella	genus	11368
species not entered	Mitrella	genus	11368
recombined as	Astyris communis	species	97536
species not entered	Anachis	genus	10755
recombined as	Astyris communis	species	97536
	Mitrella	genus	11368
	Astyris communis	species	97536
	Costoanachis avara	species	135663
subjective synonym of	Conus jaspideus	species	107770
recombined as	Conus (Contraconus) a	species	116721
	Conus daucus	species	147375
subjective synonym of	Conus anabathrum	species	146239
subjective synonym of	Conus jaspideus	species	107770
subjective synonym of	Conus jaspideus	species	107770
subjective synonym of	Conus anabathrum	species	146239
subjective synonym of	Conus jaspideus	species	107770
recombined as	Conus (Contraconus) a	species	116721
	Conus daucus	species	147375
subjective synonym of	Conus anabathrum	species	146239
	Conus spuroides	species	188685
species not entered	Conus	genus	10963
recombined as	Conus (Contraconus) a	species	116721
subjective synonym of	Conus anabathrum	species	146239
recombined as	Conus (Contraconus) a	species	116721
subjective synonym of	Conus anabathrum	species	146239
recombined as	Conus (Contraconus) a	species	116721
	Conus patricius	species	107767
	Conus harveyensis	species	119971
	Conus jaspideus	species	107770
	Conus jaspideus	species	107770
	Conus jaspideus	species	107770
subjective synonym of	Conus jaspideus	species	107770
	Conus jaspideus	species	107770
	Conus	genus	10963
	Conus	genus	10963
recombined as	Conus (Contraconus) a	species	116721
	Conus (Contraconus) a	species	116721
species not entered	Conus (Lithoconus)	subgenus	95697
recombined as	Conus daucus	species	147375

obsolete variant of	Conus marylandicus	species	93793
subjective synonym of	Conus jaspideus	species	107770
species not entered	Conus (Leptoconus)	subgenus	11261
	Conus (Contraconus) a	species	116721
obsolete variant of	Conus marylandicus	species	93793
subjective synonym of	Conus jaspideus	species	107770
species not entered	Conus (Leptoconus)	subgenus	11261
	Conus (Contraconus) a	species	116721
species not entered	Conus (Lithoconus)	subgenus	95697
species not entered	Conus (Leptoconus)	subgenus	11261
species not entered	Conus (Lithoconus)	subgenus	95697
obsolete variant of	Conus marylandicus	species	93793
subjective synonym of	Conus jaspideus	species	107770
species not entered	Conus (Leptoconus)	subgenus	11261
species not entered	Conus (Leptoconus)	subgenus	11261
subjective synonym of, species not entered	Conus	genus	10963
subjective synonym of, species not entered	Conus	genus	10963
subjective synonym of	Conus jaspideus	species	107770
recombined as	Conus spurius	species	107772
subjective synonym of	Conus spurius	species	107772
subjective synonym of	Conus spurius	species	107772
species not entered	Conus (Ximeniconus)	subgenus	119760
subjective synonym of	Conus daucus	species	147375
subjective synonym of	Conus largillierti	species	147507
subjective synonym of	Conus largillierti	species	147507
recombined as	Conus hyshugari	species	119982
recombined as	Conus maureenae	species	119985
species not entered	Conus (Ximeniconus)	subgenus	119760
subjective synonym of	Conus (Pyruconus) ha	species	333868
species not entered	Conus (Ximeniconus)	subgenus	119760
subjective synonym of	Conus (Contraconus) a	species	116721
subjective synonym of	Conus (Contraconus) a	species	116721
subjective synonym of	Conus (Contraconus) a	species	116721
subjective synonym of	Conus (Contraconus) a	species	116721
subjective synonym of	Conus daucus	species	147375
subjective synonym of, species not entered	Conus	genus	10963
subjective synonym of	Conus (Contraconus) a	species	116721
subjective synonym of	Conus (Contraconus) a	species	116721
subjective synonym of, species not entered	Conus	genus	10963
species not entered	Seminoleconus	genus	119968
species not entered	Conus (Ximeniconus)	subgenus	119760
subjective synonym of	Conus (Contraconus) a	species	116721
species not entered	Conus (Ximeniconus)	subgenus	119760
subjective synonym of	Conus (Contraconus) a	species	116721
recombined as	Conus laurenae	species	119984
subjective synonym of	Conus spurius	species	107772
subjective synonym of, species not entered	Conus	genus	10963

subjective synonym of, species not entered	Conus	genus	10963
species not entered	Conus	genus	10963
species not entered	Conus	genus	10963
obsolete variant of	Conus (Contraconus)	subgenus	116720
species not entered	Seminoleconus	genus	119968
subjective synonym of	Conus (Contraconus) a	species	116721
recombined as	Conus harveyensis	species	119971
subjective synonym of, species not entered	Conus	genus	10963
species not entered	Eugeniconus	genus	119970
species not entered	Dauciconus	genus	147384
species not entered	Conus (Lithoconus)	subgenus	95697
subjective synonym of	Conus spurius	species	107772
subjective synonym of	Conus largillierti	species	147507
recombined as	Conus (Contraconus) a	species	116721
recombined as	Conus jaclynae	species	119981
subjective synonym of	Conus delessertii	species	147428
recombined as	Conus hertwecki	species	119980
subjective synonym of	Conus (Contraconus) a	species	116721
species not entered	Eugeniconus	genus	119970
subjective synonym of, species not entered	Conus	genus	10963
species not entered	Conus (Virgiconus)	subgenus	120719
	Conus jaspideus	species	107770
	Conus jaspideus	species	107770
species not entered	Conus (Conus)	subgenus	95696
species not entered	Conus (Conus)	subgenus	95696
species not entered	Conus	genus	10963
	Conus	genus	10963
	Conus jaspideus	species	107770
subjective synonym of	Conus jaspideus	species	107770
subjective synonym of	Conus jaspideus	species	107770
	Conus	genus	10963
recombined as	Conus (Contraconus) a	species	116721
recombined as	Conus (Contraconus) a	species	116721
recombined as	Conus (Contraconus) a	species	116721
recombined as	Conus (Contraconus) a	species	116721
recombined as	Conus yaquensis	species	147521
subjective synonym of	Conus (Contraconus) a	species	116721
recombined as	Conus spurius	species	107772
species not entered	Conus	genus	10963
recombined as	Conus delessertii	species	147428
species not entered	Conus (Leptoconus)	subgenus	11261
recombined as	Conus jaspideus	species	107770
recombined as	Conus (Contraconus) a	species	116721
	Conus largillierti	species	147507
	Conus jaspideus	species	107770
	Conus spurius	species	107772
species not entered	Conus (Virgiconus)	subgenus	120719

subjective synonym of	Conus (Pyruconus) ha	species	333868
subjective synonym of	Conus spurius	species	107772
recombined as	Conus (Contraconus) a	species	116721
	Conus	genus	10963
recombined as	Conus (Contraconus) a	species	116721
	Conus marylandicus	species	93793
subjective synonym of	Conus delessertii	species	147428
	Conus jaspideus	species	107770
	Conus	genus	10963
recombined as	Conus (Contraconus) a	species	116721
	Conus	genus	10963
	Conus	genus	10963
	Conus	genus	10963
	Conus marylandicus	species	93793
	Conus marylandicus	species	93793
	Conus marylandicus	species	93793
recombined as	Conus (Contraconus) a	species	116721
recombined as	Vexillum wandoense	species	157303
species not entered	Uromitra	genus	11823
species not entered	Uromitra	genus	11823
species not entered	Uromitra	genus	11823
recombined as	Uromitra holmesii	species	164664
species not entered	Uromitra	genus	11823
	Knefastia lindae	species	188699
recombined as	Vexillum wandoense	species	157303
recombined as	Vexillum wandoense	species	157303
	Vexillum wandoense	species	157303
	Vexillum wandoense	species	157303
	Uromitra	genus	11823
species not entered	Cylichnella	genus	11924
species not entered	Cylichnella	genus	11924
recombined as	Acteocina canaliculata	species	91360
species not entered	Cylichnella	genus	11924
recombined as	Acteocina canaliculata	species	91360
recombined as	Acteocina canaliculata	species	91360
recombined as	Acteocina canaliculata	species	91360
recombined as	Acteocina canaliculata	species	91360
	Acteocina canaliculata	species	91360
	Acteocina canaliculata	species	91360
	Acteocina canaliculata	species	91360
	Acteocina canaliculata	species	91360
species not entered	Cylichnella	genus	11924
species not entered	Cylichnella	genus	11924
species not entered	Cylichnella	genus	11924
	Bullaria	genus	98733
	Acteocina candei	species	91362
	Acteocina candei	species	91362

	Acteocina candei	species	91362
	Cylichnella bidentata	species	90939
	Acteocina	genus	11900
	Acteocina candei	species	91362
species not entered	Acteocina	genus	11900
	Acteocina canaliculata	species	91360
species not entered	Cylichnella	genus	11924
	Acteocina canaliculata	species	91360
	Acteocina canaliculata	species	91360
	Acteocina canaliculata	species	91360
	Acteocina canaliculata	species	91360
	Acteocina canaliculata	species	91360
	Acteocina canaliculata	species	91360
	Acteocina candei	species	91362
recombined as	Acteocina canaliculata	species	91360
recombined as	Acteocina canaliculata	species	91360
recombined as	Acteocina canaliculata	species	91360
recombined as	Acteocina canaliculata	species	91360
recombined as	Acteocina canaliculata	species	91360
recombined as	Acteocina canaliculata	species	91360
	Acteocina canaliculata	species	91360
	Acteocina canaliculata	species	91360
	Acteocina canaliculata	species	91360
	Acteocina canaliculata	species	91360
	Acteocina candei	species	91362
	Acteocina candei	species	91362
	Acteocina canaliculata	species	91360
species not entered	Cylichna	genus	11921
	Acteocina candei	species	91362
recombined as	Acteocina canaliculata	species	91360
recombined as	Acteocina canaliculata	species	91360
recombined as	Acteocina canaliculata	species	91360
recombined as	Acteocina canaliculata	species	91360
recombined as	Acteocina canaliculata	species	91360
recombined as	Acteocina canaliculata	species	91360
	Acteocina canaliculata	species	91360
	Acteocina canaliculata	species	91360
	Acteocina canaliculata	species	91360
	Acteocina canaliculata	species	91360
	Acteocina canaliculata	species	91360
	Acteocina candei	species	91362
	Acteocina candei	species	91362
	Acteocina candei	species	91362
	Acteocina candei	species	91362
	Acteocina candei	species	91362
species not entered	Cylichna	genus	11921
	Acteocina candei	species	91362

species not entered	Cylichna	genus	11921
	Acteocina candei	species	91362
	Cylichna	genus	11921
	Cypraea problematica	species	342576
	Cypraea problematica	species	342576
recombined as	Akleistostoma carolin	species	335331
recombined as	Akleistostoma carolin	species	335331
recombined as	Macrocypraea cervus	species	156624
recombined as	Akleistostoma carolin	species	335331
	Macrocypraea cervus	species	156624
	Luria voleki	species	342051
	Macrocypraea joanne	species	343131
recombined as	Cypraea (Macrocypra	species	343175
	Pseudozonaria portell	species	187658
	Siphocypraea problem	species	111875
	Siphocypraea griffini	species	342628
	Siphocypraea problem	species	111875
recombined as	Siphocypraea philemo	species	343469
	Siphocypraea (Pahayo	species	343122
	Siphocypraea problem	species	111875
	Siphocypraea (Okeech	species	343149
	Siphocypraea (Pahayo	species	343146
	Siphocypraea dimasi	species	343062
	Siphocypraea mulepe	species	343074
	Akleistostoma jennife	species	343119
	Akleistostoma macbri	species	343155
	Calusacypraea teques	species	121276
	Calusacypraea (Myakk	species	82795
recombined as	Siphocypraea metae	species	343715
	Siphocypraea (Pahayo	species	343135
	Akleistostoma rilkoi	species	343179
	Calusacypraea sarasot	species	121275
	Calusacypraea (Myakk	species	343126
	Pseudadusta kalafuti	species	342988
	Siphocypraea cannoni	species	342009
	Siphocypraea (Pahayo	species	343710
	Akleistostoma bairdi	species	82791
	Siphocypraea (Pahayo	species	343147
	Calusacypraea (Myakk	species	82796
	Pseudadusta marilyna	species	343717
	Siphocypraea (Pahayo	species	343142
	Akleistostoma diegela	species	342580
	Siphocypraea (Pahayo	species	343133
	Siphocypraea (Pahayo	species	343712
	Siphocypraea (Pahayo	species	343714
	Akleistostoma carolin	species	335331
	Akleistostoma pilsbryi	species	341755

	Akleistostoma crocodi	species	343153
	Siphocypraea (Pahayo	species	343134
recombined as	Siphocypraea duerri	species	121274
obsolete variant of	Cypraea problematica	species	342576
	Akleistostoma floridar	species	343114
	Siphocypraea (Pahayo	species	343708
	Calusacypraea globuli	species	82793
obsolete variant of	Calusacypraea briani	species	121273
	Pseudadusta hertwecl	species	342963
	Siphocypraea trippear	species	342082
	Akleistostoma hughes	species	342629
	Pseudadusta ketteri	species	343139
	Pseudadusta lindae	species	342724
	Siphocypraea grovesi	species	342625
	Calusacypraea briani	species	121273
recombined as	Siphocypraea duerri	species	121274
	Calusacypraea teques	species	121276
	Calusacypraea sarasot	species	121275
recombined as	Akleistostoma floridar	species	343114
	Siphocypraea trippear	species	342082
	Siphocypraea transito	species	341759
recombined as	Akleistostoma hughes	species	342629
	Siphocypraea problem	species	111875
recombined as	Macrocypraea cervus	species	156624
	Macrocypraea cervus	species	156624
recombined as	Pseudadusta lindae	species	342724
recombined as	Akleistostoma pilsbryi	species	341755
recombined as	Akleistostoma hughes	species	342629
	Akleistostoma erici	species	343178
recombined as	Akleistostoma caroline	species	335331
recombined as	Akleistostoma caroline	species	335331
recombined as	Akleistostoma caroline	species	335331
recombined as	Akleistostoma caroline	species	335331
recombined as	Akleistostoma caroline	species	335331
recombined as	Akleistostoma caroline	species	335331
recombined as	Akleistostoma pilsbryi	species	341755
	Persicula ovula	species	120680
recombined as	Granulina ovuliformis	species	91268
	Persicula ovula	species	120680
recombined as	Granulina ovuliformis	species	91268
recombined as	Granulina ovuliformis	species	91268
	Persicula ovula	species	120680
species not entered	Gibberula	genus	11154
recombined as	Pugnus lachrimula	species	156626
	Persicula ovula	species	120680
	Granulina ovuliformis	species	91268
	Granulina ovuliformis	species	91268

		Granulina ovuliformis	species	91268
recombined as		Pugnus lachrimula	species	156626
		Granulina ovuliformis	species	91268
		Granulina ovuliformis	species	91268
		Granulina ovuliformis	species	91268
		Granulina ovuliformis	species	91268
		Granulina ovuliformis	species	91268
species not entered		Anguispira	genus	384101
species not entered		Discus	genus	135550
species not entered		Anguispira	genus	384101
		Discohelix	genus	8448
		Drillia	genus	11043
		Cymatosyrinx lunata	species	93805
species not entered		Clathrodrillia	genus	10923
		Cymatosyrinx lunata	species	93805
		Cymatosyrinx perpolit	species	120670
		Cymatosyrinx lunata	species	93805
species not entered		Drillia	genus	11043
species not entered		Clathrodrillia	genus	10923
species not entered		Clathrodrillia	genus	10923
		Cymatosyrinx lunata	species	93805
obsolete variant of		Cymatosyrinx	genus	11010
		Cymatosyrinx lunata	species	93805
		Cymatosyrinx lunata	species	93805
		Cymatosyrinx lunata	species	93805
		Clathrodrillia emmons	species	108738
species not entered		Clathrodrillia	genus	10923
species not entered		Clathrodrillia	genus	10923
species not entered		Clathrodrillia	genus	10923
recombined as		Crassispira (Hindsiclav	species	164125
		Clathrodrillia emmons	species	108738
recombined as		Crassispira (Hindsiclav	species	164125
		Cymatosyrinx	genus	11010
species not entered		Drillia	genus	11043
species not entered		Drillia	genus	11043
recombined as		Drillia blacki	species	119967
		Cymatosyrinx aclinica	species	93807
		Cymatosyrinx aclinica	species	93807
		Cymatosyrinx aclinica	species	93807
		Cymatosyrinx aclinica	species	93807
recombined as		Crassispira (Hindsiclav	species	164123
		Cymatosyrinx aclinica	species	93807
		Cymatosyrinx	genus	11010
		Clathrodrillia	genus	10923
		Syntomodrillia	genus	11737
		Splendrillia	genus	11692
		Cymatosyrinx lunata	species	93805

species not entered	Cymatosyrinx	genus	11010
species not entered	Cymatosyrinx	genus	11010
recombined as	Cerodrillia simpsoni	species	107430
	Cymatosyrinx lunata	species	93805
	Cymatosyrinx	genus	11010
	Cerodrillia	genus	108414
	Hindsiclava	genus	107032
	Syntomodrillia	genus	11737
	Cymatosyrinx lunata	species	93805
	Cymatosyrinx lunata	species	93805
recombined as	Clathrodrillia emmons	species	108738
recombined as	Clathrodrillia emmons	species	108738
recombined as	Clathrodrillia emmons	species	108738
	Drillia drewi	species	108746
	Cymatosyrinx ziczac	species	108750
	Cymatosyrinx tiara	species	108751
	Syntomodrillia lissotro	species	91205
	Drillia polygonalis	species	108764
recombined as	Cerodrillia simpsoni	species	107430
recombined as	Cerodrillia simpsoni	species	107430
recombined as	Cerodrillia simpsoni	species	107430
	Cymatosyrinx lunata	species	93805
	Cymatosyrinx	genus	11010
	Drillia eburnea	species	108742
	Drillia	genus	11043
	Drillia eburnea	species	108742
	Cymatosyrinx lunata	species	93805
	Cymatosyrinx lunata	species	93805
	Drillia eburnea	species	108742
	Drillia eburnea	species	108742
misspelling of, recombined as	Melampus coffea	species	155527
misspelling of, recombined as	Melampus monile	species	155530
	Melampus bidentatus	species	108704
	Melampus coffea	species	155527
	Melampus monile	species	155530
species not entered	Carychium	genus	114476
subjective synonym of	Melampus bidentatus	species	108704
species not entered	Helicodiscus	genus	162066
species not entered	Helicodiscus	genus	162066
species not entered	Helicodiscus	genus	162066
species not entered	Helicodiscus	genus	162066
species not entered	Epitonium	genus	9821
	Epitonium	genus	9821
species not entered	Epitonium	genus	9821
subjective synonym of	Epitonium humphreys	species	108695
	Clathrus	genus	9656
species not entered	Epitonium	genus	9821

	Epitonium	genus	9821
	Epitonium	genus	9821
	Epitonium	genus	9821
recombined as	Epitonium rupicola	species	108692
species not entered	Epitonium	genus	9821
	Epitonium candeanum	species	109791
species not entered	Epitonium	genus	9821
recombined as	Epitonium echinaticos	species	156459
species not entered	Epitonium	genus	9821
species not entered	Opalia	genus	10213
species not entered	Depressiscala	genus	9744
	Cirsotrema dalli	species	120555
	Epitonium	genus	9821
	Epitonium	genus	9821
species not entered	Epitonium	genus	9821
	Epitonium	genus	9821
species not entered	Epitonium	genus	9821
species not entered	Epitonium	genus	9821
species not entered	Epitonium	genus	9821
species not entered	Clathrus	genus	9656
species not entered	Clathrus	genus	9656
species not entered	Clathrus	genus	9656
species not entered	Clathrus	genus	9656
species not entered	Opalia (Dentiscala)	subgenus	9743
	Epitonium lamellosum	species	109673
	Epitonium rupicola	species	108692
	Epitonium rupicola	species	108692
	Epitonium angulatum	species	109680
subjective synonym of	Epitonium candeanum	species	109791
species not entered	Epitonium	genus	9821
species not entered	Epitonium	genus	9821
recombined as	Epitonium rupicola	species	108692
	Epitonium	genus	9821
recombined as	Epitonium rupicola	species	108692
recombined as	Epitonium rupicola	species	108692
	Epitonium lamellosum	species	109673
	Epitonium humphreys	species	108695
	Epitonium humphreys	species	108695
	Epitonium multistriatu	species	109675
	Epitonium angulatum	species	109680
	Epitonium angulatum	species	109680
	Epitonium angulatum	species	109680
	Epitonium	genus	9821
	Scalaria clathrus	species	109677
species not entered	Epitonium	genus	9821
	Epitonium leai	species	82751
	Epitonium sohli	species	82752

species not entered	Epitonium	genus	9821
species not entered	Epitonium (Hyaloscala	subgenus	9973
species not entered	Epitonium (Hyaloscala	subgenus	9973
species not entered	Epitonium	genus	9821
species not entered	Epitonium	genus	9821
species not entered	Epitonium (Pictoscala)	subgenus	10275
species not entered	Epitonium (Pictoscala)	subgenus	10275
species not entered	Epitonium (Pictoscala)	subgenus	10275
species not entered	Epitonium	genus	9821
species not entered	Epitonium	genus	9821
species not entered	Epitonium	genus	9821
species not entered	Epitonium	genus	9821
recombined as	Epitonium rupicola	species	108692
recombined as	Epitonium rupicola	species	108692
recombined as	Epitonium rupicola	species	108692
	Epitonium humphreys	species	108695
species not entered	Clathrus	genus	9656
	Epitonium	genus	9821
	Epitonium angulatum	species	109680
species not entered	Epitonium	genus	9821
	Epitonium humphreys	species	108695
	Epitonium multistriatu	species	109675
	Epitonium angulatum	species	109680
	Epitonium multistriatu	species	109675
recombined as	Epitonium rupicola	species	108692
	Epitonium angulatum	species	109680
	Epitonium humphreys	species	108695
	Epitonium multistriatu	species	109675
recombined as	Epitonium rupicola	species	108692
	Epitonium	genus	9821
	Epitonium	genus	9821
	Epitonium	genus	9821
	Epitonium	genus	9821
	Epitonium	genus	9821
	Epitonium	genus	9821
	Epitonium	genus	9821
	Epitonium	genus	9821
	Melanella	genus	10112
	Melanella	genus	10112
species not entered	Melanella	genus	10112
	Melanella	genus	10112
species not entered	Melanella (Melanella)	subgenus	442834
species not entered	Melanella (Melanella)	subgenus	442834
species not entered	Melanella (Melanella)	subgenus	442834
species not entered	Melanella (Melanella)	subgenus	442834
species not entered	Strombiformis	genus	10516
species not entered	Strombiformis	genus	10516

species not entered	Niso	genus	10181
obsolete variant of	Melanella	genus	10112
obsolete variant of	Melanella	genus	10112
obsolete variant of	Melanella	genus	10112
obsolete variant of	Melanella	genus	10112
species not entered	Niso	genus	10181
species not entered	Eulima	genus	12094
	Melanella jamaicensis	species	156315
	Balcis	genus	9524
species not entered	Niso	genus	10181
species not entered	Strombiformis	genus	10516
recombined as	Balcis biconica	species	82754
species not entered	Melanella (Melanella)	subgenus	442834
species not entered	Polygireulima	genus	98859
recombined as	Melanella conoidea	species	109683
species not entered	Polygireulima	genus	98859
species not entered	Polygireulima	genus	98859
species not entered	Polygireulima	genus	98859
species not entered	Polygireulima	genus	98859
species not entered	Polygireulima	genus	98859
species not entered	Strombiformis	genus	10516
	Melanella conoidea	species	109683
species not entered	Melanella	genus	10112
	Melanella conoidea	species	109683
	Melanella conoidea	species	109683
	Balcis biconica	species	82754
species not entered	Balcis	genus	9524
recombined as	Melanella eborea	species	82756
	Balcis	genus	9524
	Eulima juncea	species	82758
species not entered	Strombiformis	genus	10516
recombined as	Eulima juncea	species	82758
recombined as	Balcis biconica	species	82754
recombined as	Balcis biconica	species	82754
species not entered	Strombiformis	genus	10516
species not entered	Strombiformis	genus	10516
species not entered	Strombiformis	genus	10516
species not entered	Polygireulima	genus	98859
species not entered	Niso	genus	10181
	Melanella conoidea	species	109683
	Melanella conoidea	species	109683
	Melanella jamaicensis	species	156315
	Melanella conoidea	species	109683
	Melanella jamaicensis	species	156315
	Melanella	genus	10112
	Melanella jamaicensis	species	156315
	Niso	genus	10181

recombined as	Melanella eborea	species	82756
species not entered	Niso	genus	10181
	Melanella conoidea	species	109683
species not entered	Eulima	genus	12094
species not entered	Eulima	genus	12094
	Strombiformis	genus	10516
	Eulima	genus	12094
species not entered	Eulima	genus	12094
	Strombiformis	genus	10516
	Strombiformis	genus	10516
	Eulima	genus	12094
species not entered	Eulima	genus	12094
	Eulima	genus	12094
	Strombiformis	genus	10516
subjective synonym of	Cinctura lilium	species	156285
	Fasciolaria	genus	11116
	Fasciolaria	genus	11116
recombined as	Fasciolaria (Triplofusu	species	120699
recombined as	Fasciolaria (Fasciolaria	species	120697
recombined as	Fasciolaria (Fasciolaria	species	120697
recombined as	Fasciolaria (Triplofusu	species	120699
recombined as	Heilprinia caloosaensi	species	82814
recombined as	Fasciolaria (Fasciolaria	species	120697
	Fasciolaria tulipa	species	116298
recombined as	Fasciolaria (Fasciolaria	species	120697
subjective synonym of	Cinctura lilium	species	156285
recombined as	Fasciolaria (Fasciolaria	species	120697
subjective synonym of	Cinctura lilium	species	156285
recombined as	Fasciolaria (Fasciolaria	species	120697
subjective synonym of	Cinctura lilium	species	156285
recombined as	Triplofusus giganteus	species	116740
recombined as	Fasciolaria (Triplofusu	species	120699
recombined as	Heilprinia caloosaensi	species	82814
species not entered	Peristernia	genus	11504
species not entered	Peristernia	genus	11504
recombined as	Heilprinia dalli	species	167739
recombined as	Triplofusus giganteus	species	116740
species not entered	Fasciolaria	genus	11116
species not entered	Peristernia	genus	11504
recombined as	Heilprinia caloosaensi	species	82814
	Fasciolaria	genus	11116
recombined as	Triplofusus giganteus	species	116740
	Fasciolaria	genus	11116
recombined as	Triplofusus giganteus	species	116740
species not entered	Fusinus	genus	11138
species not entered	Latirus	genus	11251
recombined as	Polygona jucunda	species	156695

species not entered	Fasciolaria	genus	11116
	Fusinus	genus	11138
species not entered	Latirus (Polygona)	subgenus	156696
species not entered	Latirus (Polygona)	subgenus	156696
species not entered	Latirus (Polygona)	subgenus	156696
	Fasciolaria (Fasciolaria	species	120697
recombined as	Triplofusus giganteus	species	116740
	Fasciolaria (Triplofusu	species	120699
recombined as	Heilprinia caloosaensi	species	82814
	Fasciolaria tulipa	species	116298
recombined as	Cinctura hunteria	species	262609
	Triplofusus giganteus	species	116740
species not entered	Cinctura	genus	136783
species not entered	Fusinus	genus	11138
species not entered	Cinctura	genus	136783
species not entered	Triplofusus	genus	135774
species not entered	Fasciolaria	genus	11116
species not entered	Cinctura	genus	136783
species not entered	Cinctura	genus	136783
species not entered	Fusinus	genus	11138
species not entered	Fasciolaria	genus	11116
species not entered	Liochlamys	genus	11271
recombined as	Fasciolaria (Triplofusu	species	120699
recombined as	Fasciolaria (Fasciolaria	species	120697
species not entered	Fasciolaria	genus	11116
species not entered	Liochlamys	genus	11271
species not entered	Terebraspira	genus	116738
species not entered	Liochlamys	genus	11271
	Heilprinia caloosaensi	species	82814
species not entered	Fasciolaria	genus	11116
species not entered	Fasciolaria	genus	11116
recombined as	Fasciolaria (Fasciolaria	species	120697
species not entered	Terebraspira	genus	116738
species not entered	Cinctura	genus	136783
species not entered	Fasciolaria	genus	11116
species not entered	Terebraspira	genus	116738
species not entered	Terebraspira	genus	116738
species not entered	Triplofusus	genus	135774
species not entered	Cinctura	genus	136783
species not entered	Terebraspira	genus	116738
species not entered	Heilprinia	genus	11186
species not entered	Terebraspira	genus	116738
species not entered	Dolicholatirus	genus	11038
species not entered	Latirus	genus	11251
species not entered	Terebraspira	genus	116738
species not entered	Terebraspira	genus	116738
recombined as	Fasciolaria (Fasciolaria	species	120697

recombined as	Triplofusus giganteus	species	116740
recombined as	Fasciolaria (Fasciolaria	species	120697
	Latirus	genus	11251
	Terebraspira	genus	116738
species not entered	Fusinus	genus	11138
	Heilprinia dalli	species	167739
species not entered	Heilprinia	genus	11186
species not entered	Heilprinia	genus	11186
species not entered	Triplofusus	genus	135774
species not entered	Terebraspira	genus	116738
species not entered	Cinctura	genus	136783
species not entered	Terebraspira	genus	116738
species not entered	Terebraspira	genus	116738
species not entered	Terebraspira	genus	116738
species not entered	Pleuroploca	genus	11537
species not entered	Latirus	genus	11251
	Latirus	genus	11251
	Latirus	genus	11251
recombined as	Heilprinia caloosaensi	species	82814
	Heilprinia caloosaensi	species	82814
recombined as	Fasciolaria (Fasciolaria	species	120697
species not entered	Peristernia	genus	11504
recombined as	Heilprinia dalli	species	167739
	Fusinus	genus	11138
species not entered	Fasciolaria	genus	11116
species not entered	Fusinus	genus	11138
	Latirus	genus	11251
	Latirus	genus	11251
species not entered	Heilprinia	genus	11186
	Heilprinia dalli	species	167739
species not entered	Terebraspira	genus	116738
species not entered	Liochlamys	genus	11271
recombined as	Cinctura lilium	species	156285
recombined as	Polygona jucunda	species	156695
recombined as	Cinctura lilium	species	156285
	Fasciolaria tulipa	species	116298
species not entered	Latirus (Polygona)	subgenus	156696
	Triplofusus giganteus	species	116740
species not entered	Latirus	genus	11251
species not entered	Terebraspira	genus	116738
recombined as	Triplofusus giganteus	species	116740
species not entered	Fasciolaria	genus	11116
species not entered	Terebraspira	genus	116738
species not entered	Terebraspira	genus	116738
species not entered	Fusinus	genus	11138
subjective synonym of	Cinctura lilium	species	156285
subjective synonym of	Cinctura lilium	species	156285

recombined as	Triplofusus giganteus	species	116740
recombined as	Triplofusus giganteus	species	116740
	Fasciolaria tulipa	species	116298
	Fasciolaria	genus	11116
	Fusinus	genus	11138
species not entered	Fasciolaria	genus	11116
species not entered	Fasciolaria	genus	11116
species not entered	Fusinus	genus	11138
	Fasciolaria tulipa	species	116298
species not entered	Fasciolaria	genus	11116
	Fasciolaria	genus	11116
	Fusinus exilis	species	81355
	Fusinus exilis	species	81355
	Fasciolaria	genus	11116
species not entered	Heilprinia	genus	11186
species not entered	Fasciolaria	genus	11116
species not entered	Peristernia	genus	11504
species not entered	Peristernia	genus	11504
species not entered	Lirosoma	genus	11281
	Lirosoma	genus	11281
	Fusinus exilis	species	81355
species not entered	Peristernia	genus	11504
species not entered	Peristernia	genus	11504
	Lirosoma sulcosa	species	97494
	Lirosoma	genus	11281
species not entered	Peristernia	genus	11504
	Fusinus exilis	species	81355
species not entered	Peristernia	genus	11504
	Lirosoma sulcosa	species	97494
	Fusinus	genus	11138
	Lirosoma sulcosa	species	97494
	Lirosoma sulcosa	species	97494
subjective synonym of	Ficus communis	species	147913
species not entered	Ficus	genus	9857
	Ficus communis	species	147913
species not entered	Ficus	genus	9857
species not entered	Ficus	genus	9857
subjective synonym of	Ficus communis	species	147913
species not entered	Ficus	genus	9857
subjective synonym of	Ficus communis	species	147913
species not entered	Ficus	genus	9857
	Ficus	genus	9857
	Ficus communis	species	147913
species not entered	Ficus	genus	9857
subjective synonym of	Ficus communis	species	147913
	Ficus communis	species	147913
	Ficus communis	species	147913

subjective synonym of	Diodora cayenensis	species	91262
	Diodora	genus	8748
	Diodora cayenensis	species	91262
species not entered	Diodora	genus	8748
	Diodora cayenensis	species	91262
	Diodora cayenensis	species	91262
subjective synonym of, species not entered	Diodora	genus	8748
	Diodora cayenensis	species	91262
	Diodora meta	species	109209
species not entered	Diodora	genus	8748
subjective synonym of	Lucapina suffusa	species	91313
	Lucapinella limatula	species	91320
	Diodora cayenensis	species	91262
	Diodora cayenensis	species	91262
species not entered	Emarginula	genus	8773
species not entered	Diodora	genus	8748
	Diodora cayenensis	species	91262
	Lucapina sowerbii	species	109108
	Lucapina suffusa	species	91313
	Lucapinella limatula	species	91320
subjective synonym of	Lucapina suffusa	species	91313
	Diodora	genus	8748
species not entered	Diodora	genus	8748
	Fissurella	genus	8815
species not entered	Diodora	genus	8748
	Fissurella	genus	8815
subjective synonym of	Lucapina suffusa	species	91313
species not entered	Lucapina	genus	8947
species not entered	Diodora	genus	8748
species not entered	Diodora	genus	8748
species not entered	Diodora	genus	8748
	Diodora cayenensis	species	91262
species not entered	Diodora	genus	8748
species not entered	Diodora	genus	8748
species not entered	Lucapina	genus	8947
species not entered	Diodora	genus	8748
species not entered	Emarginula	genus	8773
	Diodora cayenensis	species	91262
	Lucapina suffusa	species	91313
species not entered	Diodora	genus	8748
species not entered	Diodora	genus	8748
species not entered	Diodora	genus	8748
	Diodora	genus	8748
	Diodora	genus	8748
	Diodora	genus	8748
	Diodora auroraensis	species	82739
	Diodora cayenensis	species	91262

	Diodora cayenensis	species	91262
	Diodora cayenensis	species	91262
	Diodora cayenensis	species	91262
	Diodora cayenensis	species	91262
	Diodora pamlicoensis	species	82740
subjective synonym of	Diodora cayenensis	species	91262
subjective synonym of, species not entered	Diodora	genus	8748
subjective synonym of	Diodora cayenensis	species	91262
species not entered	Diodora	genus	8748
species not entered	Diodora	genus	8748
species not entered	Diodora	genus	8748
species not entered	Diodora	genus	8748
	Diodora redimicula	species	97711
	Diodora redimicula	species	97711
species not entered	Diodora	genus	8748
species not entered	Diodora	genus	8748
species not entered	Diodora	genus	8748
species not entered	Diodora	genus	8748
	Diodora	genus	8748
	Diodora	genus	8748
	Diodora auroraensis	species	82739
	Diodora redimicula	species	97711
	Diodora redimicula	species	97711
	Diodora redimicula	species	97711
	Diodora redimicula	species	97711
	Diodora	genus	8748
	Diodora	genus	8748
	Diodora	genus	8748
	Diodora	genus	8748
species not entered	Gastrocopta	genus	162062
species not entered	Gastrocopta	genus	162062
species not entered	Gastrocopta	genus	162062
species not entered	Gastrocopta	genus	162062
species not entered	Gastrocopta	genus	162062
species not entered	Gastrocopta	genus	162062
species not entered	Gastrocopta	genus	162062
species not entered	Gastrocopta	genus	162062
species not entered	Atys	genus	11904
	Atys obscuratus	species	134956
species not entered	Atys	genus	11904
	Atys	genus	11904
recombined as	Atys obscuratus	species	134956
	Haminoea	genus	11946
recombined as	Morum (Morum) onis	species	86361
subjective synonym of, species not entered	Oniscidia	genus	10208
subjective synonym of, species not entered	Oniscidia	genus	10208
species not entered	Morum	genus	10151

subjective synonym of, species not entered	Oniscidia	genus	10208
	Morum (Morum) onis	species	86361
	Morum (Morum) onis	species	86361
species not entered	Hendersonia	genus	116329
species not entered	Hendersonia	genus	116329
species not entered	Hipponix	genus	9963
species not entered	Hipponix	genus	9963
recombined as	Inodrillia aepynota	species	120668
species not entered	Thelecythara	genus	11768
	Buchema	genus	108409
	Hydrobia	genus	85011
	Hydrobia	genus	85011
	Hydrobia	genus	85011
	Hydrobiidae	family	60680
species not entered	Tyronia	genus	251917
	Paludestrina plana	species	311056
subjective synonym of	Hydrobia truncata	species	91298
species not entered	Molleria	genus	97700
species not entered	Didianema	genus	8745
	Didianema	genus	8745
species not entered	Didianema	genus	8745
species not entered	Didianema	genus	8745
species not entered	Liotia	genus	8928
species not entered	Didianema	genus	8745
species not entered	Didianema	genus	8745
species not entered	Didianema	genus	8745
species not entered	Didianema	genus	8745
species not entered	Didianema	genus	8745
recombined as	Littoraria irrorata	species	97642
	Littorina sheaferi	species	93976
species not entered	Littoraria (Littorinopsi	subgenus	10071
species not entered	Littoraria (Littorinopsi	subgenus	10071
species not entered	Littoraria (Littorinopsi	subgenus	10071
species not entered	Littoraria (Littorinopsi	subgenus	10071
recombined as	Littoraria irrorata	species	97642
recombined as	Littoraria angulifera	species	156700
recombined as	Littoraria irrorata	species	97642
recombined as	Littoraria irrorata	species	97642
recombined as	Littoraria irrorata	species	97642
recombined as	Littoraria irrorata	species	97642
recombined as	Littoraria irrorata	species	97642
	Littoraria irrorata	species	97642
recombined as	Littoraria irrorata	species	97642
recombined as	Littoraria irrorata	species	97642
recombined as	Littoraria irrorata	species	97642
recombined as	Littoraria irrorata	species	97642
recombined as	Littoraria irrorata	species	97642

recombined as	Littoraria irrorata	species	97642
recombined as	Littoraria irrorata	species	97642
recombined as	Littoraria irrorata	species	97642
recombined as	Littoraria irrorata	species	97642
recombined as	Littoraria irrorata	species	97642
	Littorina	genus	10068
recombined as	Littoraria irrorata	species	97642
recombined as	Littoraria irrorata	species	97642
recombined as	Patelloida pustulata	species	109075
recombined as	Lottia actina	species	144943
	Acmaea	genus	8395
species not entered	Fossaria	genus	162060
species not entered	Pseudosuccinea	genus	251923
species not entered	Fossaria	genus	162060
species not entered	Fossaria	genus	162060
species not entered	Pseudosuccinea	genus	251923
species not entered	Fossaria	genus	162060
species not entered	Fossaria	genus	162060
species not entered	Lymnaea	genus	83642
species not entered	Magilus	genus	11308
species not entered	Brachycythara	genus	10854
species not entered	Brachycythara	genus	10854
species not entered	Cythara	genus	11016
subjective synonym of	Ithycythara psila	species	91301
species not entered	Kurtziella	genus	11237
	Kurtziella	genus	11237
species not entered	Kurtziella	genus	11237
obsolete variant of	Cythara	genus	11016
species not entered	Cythara	genus	11016
species not entered	Kurtziella	genus	11237
species not entered	Cythara (Mangelia)	subgenus	91203
species not entered	Cythara (Mangelia)	subgenus	91203
species not entered	Cythara (Mangelia)	subgenus	91203
species not entered	Cythara (Mangelia)	subgenus	91203
species not entered	Cythara (Mangelia)	subgenus	91203
replaced by	Ithycythara lanceolata	species	157375
species not entered	Acmaturris	genus	10713
	Pyrgocythara plicosa	species	91344
	Pyrgocythara plicosa	species	91344
subjective synonym of	Ithycythara psila	species	91301
species not entered	Vitricythara	genus	82944
recombined as	Cytharella galae	species	162773
	Ithycythara maera	species	107471
subjective synonym of	Ithycythara psila	species	91301
species not entered	Pyrgocythara	genus	11616
species not entered	Ithycythara	genus	11222
recombined as	Cytharella galae	species	162773

species not entered	Brachycythara	genus	10854
	Kurtziella limonitella	species	91308
recombined as	Kurtziella (Cryoturris)	species	91310
species not entered	Granoturris	genus	121448
species not entered	Vitricythara	genus	82944
species not entered	Vitricythara	genus	82944
recombined as	Kurtziella (Cryoturris)	species	91310
recombined as	Kurtziella (Cryoturris)	species	91310
recombined as	Kurtziella (Cryoturris)	species	91310
	Kurtziella cerina	species	162783
	Pyrgocythara plicosa	species	91344
	Brachycythara	genus	10854
recombined as	Kurtziella cerina	species	162783
recombined as	Kurtziella cerina	species	162783
recombined as	Kurtziella cerina	species	162783
recombined as	Glyphoturris eritima	species	107423
recombined as	Glyphoturris eritima	species	107423
	Ithycythara psila	species	91301
species not entered	Kurtziella (Cryoturris)	subgenus	10992
species not entered	Kurtziella (Cryoturris)	subgenus	10992
recombined as	Stellatoma stellata	species	108713
recombined as	Stellatoma stellata	species	108713
recombined as	Kurtziella cerina	species	162783
recombined as	Kurtziella cerina	species	162783
recombined as	Kurtziella cerina	species	162783
species not entered	Kurtziella (Cryoturris)	subgenus	10992
	Kurtziella cerina	species	162783
	Kurtziella cerina	species	162783
	Marginella	genus	11317
recombined as	Prunum apicinum	species	116743
species not entered	Marginella	genus	11317
subjective synonym of	Prunum roscidum	species	94425
recombined as	Prunum apicinum	species	116743
species not entered	Marginella	genus	11317
recombined as	Prunum apicinum	species	116743
species not entered	Marginella	genus	11317
species not entered	Marginella	genus	11317
subjective synonym of	Prunum roscidum	species	94425
recombined as	Dentimargo polyspira	species	82820
recombined as	Marginella (Leptegona	species	120691
	Marginella	genus	11317
recombined as	Prunum apicinum	species	116743
subjective synonym of	Prunum roscidum	species	94425
species not entered	Marginella	genus	11317
	Marginella	genus	11317
recombined as	Prunum apicinum	species	116743
species not entered	Marginella	genus	11317

species not entered	Marginella	genus	11317
recombined as	Prunum apicinum	species	116743
species not entered	Marginella	genus	11317
recombined as	Prunum apicinum	species	116743
recombined as	Prunum apicinum	species	116743
subjective synonym of	Prunum roscidum	species	94425
species not entered	Marginella	genus	11317
species not entered	Marginella	genus	11317
recombined as	Marginella (Leptegona	species	120691
obsolete variant of	Marginella	genus	11317
species not entered	Marginella	genus	11317
species not entered	Marginella	genus	11317
	Marginella minuta	species	97471
	Marginella minuta	species	97471
species not entered	Marginella	genus	11317
recombined as	Dentimargo aureocinc	species	155515
recombined as	Dentimargo aureocinc	species	155515
recombined as	Volvarina albolineata	species	91292
recombined as	Volvarina avena	species	82822
	Marginella	genus	11317
recombined as	Dentimargo eburneola	species	116728
species not entered	Marginella	genus	11317
recombined as	Volvarina albolineata	species	91292
	Marginella	genus	11317
recombined as	Dentimargo eburneola	species	116728
recombined as	Dentimargo eburneola	species	116728
	Marginella	genus	11317
	Marginella	genus	11317
	Prunum roscidum	species	94425
	Marginella	genus	11317
recombined as	Marginella (Leptegona	species	120691
recombined as	Prunum limatulum	species	82817
	Marginella	genus	11317
recombined as	Prunum limatulum	species	82817
	Marginella denticulata	species	97472
recombined as	Prunum limatulum	species	82817
	Marginella	genus	11317
recombined as	Prunum limatulum	species	82817
recombined as	Prunum limatulum	species	82817
recombined as	Dentimargo polyspira	species	82820
recombined as	Dentimargo aureocinc	species	155515
species not entered	Volvarina	genus	11857
species not entered	Volvarina	genus	11857
species not entered	Marginella	genus	11317
subjective synonym of	Prunum roscidum	species	94425
recombined as	Prunum apicinum	species	116743
recombined as	Prunum amabile	species	156670

recombined as	Marginella (Leptegona	species	120676
recombined as	Marginella (Leptegona	species	120691
species not entered	Eratoidea	genus	11085
species not entered	Volvarina	genus	11857
subjective synonym of, species not entered	Dentimargo	genus	11026
species not entered	Prunum (Microspira)	subgenus	93836
	Microspira donovani	species	93839
	Marginella	genus	11317
	Marginella	genus	11317
species not entered	Marginella	genus	11317
recombined as	Prunum apicinum	species	116743
	Marginella	genus	11317
	Marginella	genus	11317
	Marginella	genus	11317
	Marginella	genus	11317
	Marginella	genus	11317
	Marginella	genus	11317
	Marginella	genus	11317
	Marginella	genus	11317
species not entered	Marginella	genus	11317
species not entered	Dentimargo	genus	11026
	Prunum apicinum	species	116743
species not entered	Prunum	genus	11564
species not entered	Prunum	genus	11564
	Marginella	genus	11317
species not entered	Prunum	genus	11564
	Prunum roscidum	species	94425
	Prunum roscidum	species	94425
	Prunum roscidum	species	94425
	Prunum limatulum	species	82817
	Marginella	genus	11317
recombined as	Prunum limatulum	species	82817
recombined as	Dentimargo aureocinc	species	155515
	Marginella	genus	11317
species not entered	Dentimargo	genus	11026
	Prunum limatulum	species	82817
	Dentimargo polyspira	species	82820
	Prunum limatulum	species	82817
	Volvarina avena	species	82822
	Prunum limatulum	species	82817
	Dentimargo polyspira	species	82820
	Prunum limatulum	species	82817
recombined as	Volvarina avena	species	82822
obsolete variant of	Marginella denticulata	species	97472
species not entered	Serrata	genus	11665
	Prunum	genus	11564
	Prunum	genus	11564

	Hyalina	genus	11209
	Prunum	genus	11564
	Hyalina	genus	11209
	Prunum	genus	11564
	Prunum roscidum	species	94425
	Prunum limatulum	species	82817
	Prunum limatulum	species	82817
	Prunum limatulum	species	82817
species not entered	Prunum	genus	11564
	Marginella	genus	11317
	Marginella	genus	11317
	Marginella	genus	11317
	Marginella	genus	11317
	Marginella	genus	11317
	Marginella	genus	11317
	Marginella	genus	11317
subjective synonym of	Prunum (Microspira)	subgenus	93836
	Prunum	genus	11564
	Prunum roscidum	species	94425
recombined as	Dentimargo aureocinc	species	155515
recombined as	Dentimargo aureocinc	species	155515
	Prunum roscidum	species	94425
	Prunum roscidum	species	94425
recombined as	Prunum limatulum	species	82817
species not entered	Marginella	genus	11317
	Marginella	genus	11317
recombined as	Prunum limatulum	species	82817
	Marginella	genus	11317
	Marginella	genus	11317
recombined as	Prunum limatulum	species	82817
species not entered	Marginella	genus	11317
	Marginella denticulata	species	97472
species not entered	Marginella	genus	11317
	Marginella	genus	11317
	Busycon carica	species	93861
	Busycon perversum	species	108697
	Melongena corona	species	109658
	Melongena	genus	11341
	Busycon contrarium	species	94555
subjective synonym of	Busycotypus spiratus	species	109668
recombined as	Busycon (Busycon) rap	species	120693
	Busycon	genus	10874
	Busycon contrarium	species	94555
	Melongena corona	species	109658
	Busycon contrarium	species	94555
subjective synonym of	Busycotypus spiratus	species	109668
	Melongena corona	species	109658

	Busycon	genus	10874
	Busycon contrarium	species	94555
recombined as	Busycon (Busycon) rap	species	120693
	Melongena corona	species	109658
	Busycon contrarium	species	94555
subjective synonym of	Busycotypus spiratus	species	109668
	Melongena corona	species	109658
	Busycon contrarium	species	94555
species not entered	Busycon	genus	10874
subjective synonym of	Busycotypus spiratus	species	109668
recombined as	Busycon (Busycon) rap	species	120693
	Melongena corona	species	109658
species not entered	Busycon	genus	10874
	Busycon perversum	species	108697
	Busycon perversum	species	108697
	Melongena consors	species	108029
recombined as	Busycotypus spiratus	species	109668
	Busycon contrarium	species	94555
	Busycon contrarium	species	94555
recombined as	Busycotypus spiratus	species	109668
	Melongena corona	species	109658
	Busycon contrarium	species	94555
	Melongena bispinosa	species	119900
	Busycon contrarium	species	94555
	Busycon contrarium	species	94555
	Busycon	genus	10874
	Busycon contrarium	species	94555
subjective synonym of	Busycotypus spiratus	species	109668
	Busycon (Busycon) rap	species	120693
obsolete variant of	Busycon contrarium	species	94555
species not entered	Busycon	genus	10874
subjective synonym of	Busycotypus spiratus	species	109668
	Busycon (Echinofulgur	species	116733
subjective synonym of	Melongena corona	species	109658
species not entered	Busycon	genus	10874
	Busycon contrarium	species	94555
recombined as	Busycotypus spiratus	species	109668
recombined as	Busycon perversum	species	108697
recombined as	Melongena corona	species	109658
recombined as	Melongena corona	species	109658
subjective synonym of, species not entered	Busycon	genus	10874
	Melongena melongen	species	111213
species not entered	Melongena	genus	11341
recombined as	Melongena bispinosa	species	119900
species not entered	Melongena (Rexmela)	subgenus	116734
subjective synonym of, species not entered	Busycon	genus	10874
species not entered	Busycon	genus	10874

subjective synonym of, species not entered	Busycon	genus	10874
subjective synonym of	Busycon carica	species	93861
species not entered	Busycotypus	genus	10875
subjective synonym of, species not entered	Busycon	genus	10874
subjective synonym of, species not entered	Busycon	genus	10874
species not entered	Melongena	genus	11341
species not entered	Melongena	genus	11341
	Fulguropsis feldmanni	species	120025
subjective synonym of, species not entered	Busycon	genus	10874
subjective synonym of, species not entered	Busycon	genus	10874
subjective synonym of, species not entered	Busycon	genus	10874
	Busycon (Echinofulgur	species	116733
	Busycon (Echinofulgur	species	116733
	Tropochasca petiti	species	93954
	Melongena bispinosa	species	119900
species not entered	Melongena	genus	11341
subjective synonym of, species not entered	Busycon	genus	10874
subjective synonym of, species not entered	Busycon	genus	10874
subjective synonym of, species not entered	Busycon	genus	10874
subjective synonym of, species not entered	Busycon	genus	10874
species not entered	Busycon (Busycoarctu	subgenus	116291
species not entered	Melongena	genus	11341
recombined as	Busycon (Echinofulgur	species	116733
subjective synonym of, species not entered	Busycon	genus	10874
subjective synonym of, species not entered	Busycon	genus	10874
subjective synonym of, species not entered	Busycon	genus	10874
subjective synonym of, species not entered	Busycon	genus	10874
subjective synonym of, species not entered	Busycon	genus	10874
subjective synonym of, species not entered	Busycon	genus	10874
subjective synonym of, species not entered	Busycon	genus	10874
subjective synonym of, species not entered	Busycon	genus	10874
	Melongena	genus	11341
subjective synonym of, species not entered	Busycon	genus	10874
subjective synonym of, species not entered	Busycon	genus	10874
species not entered	Tropochasca	genus	93953
subjective synonym of, species not entered	Busycon	genus	10874
species not entered	Melongena	genus	11341
subjective synonym of, species not entered	Busycon	genus	10874
species not entered	Melongena	genus	11341
subjective synonym of, species not entered	Busycon	genus	10874
subjective synonym of, species not entered	Busycon	genus	10874
species not entered	Melongena	genus	11341
subjective synonym of, species not entered	Busycon	genus	10874
	Busycon contrarium	species	94555
subjective synonym of	Busycotypus spiratus	species	109668
	Busycon contrarium	species	94555
subjective synonym of	Busycotypus spiratus	species	109668

subjective synonym of, species not entered	Busycon	genus	10874
species not entered	Busycon	genus	10874
subjective synonym of, species not entered	Busycon	genus	10874
subjective synonym of, species not entered	Busycon	genus	10874
subjective synonym of, species not entered	Busycon	genus	10874
	Tropochasca petiti	species	93954
subjective synonym of, species not entered	Busycon	genus	10874
subjective synonym of, species not entered	Busycon	genus	10874
species not entered	Busycotypus	genus	10875
species not entered	Busycon (Busycoarctu	subgenus	116291
subjective synonym of, species not entered	Busycon	genus	10874
	Busycon filosum	species	167640
subjective synonym of, species not entered	Busycon	genus	10874
species not entered	Tropochasca	genus	93953
species not entered	Melongena	genus	11341
species not entered	Busycon	genus	10874
species not entered	Busycotypus	genus	10875
species not entered	Busycon (Busycoarctu	subgenus	116291
subjective synonym of, species not entered	Busycon	genus	10874
subjective synonym of, species not entered	Busycon	genus	10874
subjective synonym of, species not entered	Busycon	genus	10874
subjective synonym of, species not entered	Busycon	genus	10874
subjective synonym of, species not entered	Busycon	genus	10874
subjective synonym of, species not entered	Busycon	genus	10874
subjective synonym of, species not entered	Busycon	genus	10874
species not entered	Busycon (Busycoarctu	subgenus	116291
	Busycon contrarium	species	94555
	Busycon	genus	10874
subjective synonym of	Busycotypus spiratus	species	109668
	Busycon contrarium	species	94555
	Busycon contrarium	species	94555
	Busycon	genus	10874
	Busycon contrarium	species	94555
species not entered	Melongena	genus	11341
	Busycon contrarium	species	94555
	Busycon	genus	10874
species not entered	Busycotypus	genus	10875
species not entered	Busycon	genus	10874
subjective synonym of, species not entered	Busycon	genus	10874
subjective synonym of, species not entered	Busycon	genus	10874
obsolete variant of	Busycon (Busycoarctu	subgenus	116291
subjective synonym of, species not entered	Busycon	genus	10874
species not entered	Busycon	genus	10874
subjective synonym of, species not entered	Busycon	genus	10874
species not entered	Busycotypus	genus	10875
subjective synonym of, species not entered	Busycon	genus	10874
subjective synonym of, species not entered	Busycon	genus	10874

	Tropochasca petiti	species	93954
species not entered	Melongena (Rexmela)	subgenus	116734
subjective synonym of, species not entered	Busycon	genus	10874
	Melongenidae	family	61090
recombined as	Melongena bispinosa	species	119900
	Melongena (Rexmela)	subgenus	116734
recombined as	Melongena corona	species	109658
	Busycotypus canalicul	species	94432
	Busycotypus spiratus	species	109668
	Melongena melongen	species	111213
subjective synonym of, species not entered	Busycon	genus	10874
recombined as	Busycon perversum	species	108697
	Busycon carica	species	93861
	Busycon carica	species	93861
	Melongena corona	species	109658
	Busycon contrarium	species	94555
species not entered	Busycon	genus	10874
species not entered	Busycon	genus	10874
subjective synonym of	Busycotypus spiratus	species	109668
species not entered	Busycon	genus	10874
	Busycon	genus	10874
recombined as	Busycotypus canalicul	species	94432
recombined as	Busycotypus canalicul	species	94432
recombined as	Busycotypus canalicul	species	94432
	Busycon carica	species	93861
	Busycon carica	species	93861
	Busycon carica	species	93861
	Busycon perversum	species	108697
	Busycon perversum	species	108697
recombined as	Busycotypus spiratus	species	109668
recombined as	Busycon carica	species	93861
	Busycon perversum	species	108697
recombined as	Busycotypus canalicul	species	94432
species not entered	Busycon	genus	10874
recombined as	Busycotypus spiratus	species	109668
recombined as	Busycotypus concinnu	species	167644
species not entered	Busycon	genus	10874
species not entered	Busycon	genus	10874
species not entered	Busycon	genus	10874
species not entered	Busycon	genus	10874
	Busycon perversum	species	108697
	Busycon perversum	species	108697
species not entered	Busycon	genus	10874
species not entered	Busycon	genus	10874
species not entered	Busycon	genus	10874
species not entered	Busycon	genus	10874
species not entered	Busycon	genus	10874

recombined as	Busycotypus concinnu	species	167644
recombined as	Busycotypus concinnu	species	167644
	Busycon	genus	10874
	Busycon	genus	10874
	Busycon	genus	10874
	Busycon	genus	10874
recombined as	Busycotypus canalicul	species	94432
recombined as	Busycotypus canalicul	species	94432
recombined as	Busycotypus canalicul	species	94432
recombined as	Busycotypus canalicul	species	94432
recombined as	Busycotypus canalicul	species	94432
recombined as	Busycotypus canalicul	species	94432
	Busycon carica	species	93861
	Busycon carica	species	93861
	Busycon carica	species	93861
	Busycon carica	species	93861
	Busycon carica	species	93861
	Busycon perversum	species	108697
recombined as	Busycotypus spiratus	species	109668
species not entered	Busycon	genus	10874
	Busycotypus canalicul	species	94432
	Busycon carica	species	93861
	Busycon	genus	10874
	Busycon	genus	10874
	Busycon carica	species	93861
	Busycon contrarium	species	94555
	Busycon	genus	10874
	Busycon	genus	10874
	Busycon contrarium	species	94555
	Busycon	genus	10874
	Busycon	genus	10874
species not entered	Busycon	genus	10874
	Busycon	genus	10874
	Busycon	genus	10874
species not entered	Busycon	genus	10874
recombined as	Busycotypus canalicul	species	94432
recombined as	Busycotypus canalicul	species	94432
	Busycon carica	species	93861
recombined as	Busycotypus canalicul	species	94432
	Busycon carica	species	93861
recombined as	Busycotypus canalicul	species	94432
	Busycon carica	species	93861
recombined as	Busycotypus canalicul	species	94432
	Busycon carica	species	93861
	Busycon contrarium	species	94555
	Busycotypus canalicul	species	94432
	Busycon carica	species	93861

species not entered	Busycotypus	genus	10875
species not entered	Busycon	genus	10874
species not entered	Busycotypus	genus	10875
species not entered	Busycon	genus	10874
species not entered	Busycotypus	genus	10875
species not entered	Busycon	genus	10874
species not entered	Busycotypus	genus	10875
species not entered	Busycon	genus	10874
species not entered	Busycon	genus	10874
	Busycon	genus	10874
species not entered	Busycon	genus	10874
species not entered	Busycotypus	genus	10875
	Busycon	genus	10874
	Busycon	genus	10874
species not entered	Busycon	genus	10874
subjective synonym of, species not entered	Busycon	genus	10874
species not entered	Busycotypus	genus	10875
recombined as	Mitra (Pleioptygma) h	species	120366
recombined as	Mitra (Pleioptygma) h	species	120366
species not entered	Mitra	genus	11365
species not entered	Mitra	genus	11365
	Mitra	genus	11365
recombined as	Mitra (Pleioptygma) h	species	120366
species not entered	Subcancilla	genus	11719
species not entered	Subcancilla	genus	11719
species not entered	Mitra (Pleioptygma)	subgenus	11532
	Mitridae	family	61164
species not entered	Mitra (Pleioptygma)	subgenus	11532
species not entered	Mitra	genus	11365
	Subcancilla	genus	11719
species not entered	Mitra (Pleioptygma)	subgenus	11532
recombined as	Mitra (Pleioptygma) c	species	164623
species not entered	Mitra	genus	11365
species not entered	Mitra	genus	11365
recombined as	Subcancilla dalli	species	107864
recombined as	Mitra (Pleioptygma) c	species	164623
misspelling of, subjective synonym of	Mitromorpha dormitc	species	107893
misspelling of, subjective synonym of	Mitromorpha dormitc	species	107893
subjective synonym of	Mitromorpha dormitc	species	107893
subjective synonym of	Modulus modulus	species	94595
	Modulus carchedoniu	species	187453
	Modulus carchedoniu	species	187453
	Modulus carchedoniu	species	187453
	Modulus carchedoniu	species	187453
species not entered	Modulus	genus	10140
	Modulus modulus	species	94595
	Modulus modulus	species	94595

	Modulus modulus	species	94595
	Modulus modulus	species	94595
	Modulus calusa	species	157098
species not entered	Modulus	genus	10140
species not entered	Modulus	genus	10140
	Modulus modulus	species	94595
	Modulus basileus	species	187450
	Modulus modulus	species	94595
	Modulus modulus	species	94595
	Modulus modulus	species	94595
	Modulus modulus	species	94595
	Modulus modulus	species	94595
	Modulus modulus	species	94595
	Modulus carchedoniu	species	187453
	Modulus modulus	species	94595
	Modulus calusa	species	157098
species not entered	Modulus	genus	10140
species not entered	Modulus	genus	10140
	Urosalpinx	genus	11824
	Eupleura caudata	species	94512
species not entered	Murex	genus	11385
species not entered	Purpura	genus	11605
species not entered	Trophon	genus	11793
subjective synonym of	Calotrophon ostrearu	species	91536
recombined as	Chicoreus (Phyllonotu	species	107994
subjective synonym of	Calotrophon ostrearu	species	91536
recombined as	Chicoreus (Chicoreus)	species	81537
recombined as	Chicoreus (Phyllonotu	species	107994
	Murex	genus	11385
species not entered	Trophon	genus	11793
recombined as	Typhis (Rugotyphis) fl	species	135226
species not entered	Urosalpinx	genus	11824
subjective synonym of	Calotrophon ostrearu	species	91536
	Eupleura caudata	species	94512
recombined as	Chicoreus (Phyllonotu	species	107994
recombined as	Urosalpinx perrugata	species	135222
subjective synonym of	Calotrophon ostrearu	species	91536
recombined as	Chicoreus (Chicoreus)	species	81537
subjective synonym of	Calotrophon ostrearu	species	91536
recombined as	Murexiella macgintyi	species	82761
recombined as	Chicoreus (Phyllonotu	species	107994
subjective synonym of	Calotrophon	genus	10884
species not entered	Trophon	genus	11793
species not entered	Urosalpinx	genus	11824
	Eupleura caudata	species	94512
subjective synonym of	Calotrophon ostrearu	species	91536
species not entered	Purpura	genus	11605

species not entered	Urosalpinx	genus	11824
	Urosalpinx perrugata	species	135222
recombined as	Chicoreus (Phyllonotu	species	107994
recombined as	Hexaplex (Hexaplex) f	species	109407
	Chicoreus	genus	10913
	Calotrophon ostrearu	species	91536
species not entered	Eupleura	genus	11099
	Favartia cellulosa	species	156293
	Urosalpinx perrugata	species	135222
recombined as	Chicoreus (Phyllonotu	species	107994
recombined as	Hexaplex (Hexaplex) f	species	109407
	Murexiella macgintyi	species	82761
species not entered	Eupleura	genus	11099
recombined as	Chicoreus (Phyllonotu	species	107994
	Urosalpinx perrugata	species	135222
recombined as	Pterotyphis (Tripterot	species	135413
recombined as	Murexiella (Murexiella	species	158894
recombined as	Vokesimurex belleglac	species	119896
recombined as	Vokesimurex anniae	species	144744
	Urosalpinx	genus	11824
	Urosalpinx	genus	11824
species not entered	Eupleura	genus	11099
species not entered	Vokesinotus	genus	157090
species not entered	Chicoreus	genus	10913
species not entered	Chicoreus	genus	10913
species not entered	Vokesimurex	genus	119897
	Vokesimurex belleglac	species	119896
recombined as	Murexiella (Murexiella	species	158894
species not entered	Eupleura	genus	11099
species not entered	Urosalpinx	genus	11824
species not entered	Vokesimurex	genus	119897
	Ecphora quadricostata	species	97553
	Ecphora quadricostata	species	97553
species not entered	Phyllonotus	genus	11521
species not entered	Chicoreus	genus	10913
species not entered	Chicoreus	genus	10913
species not entered	Phyllonotus	genus	11521
species not entered	Pterorhytis	genus	82765
recombined as	Murexiella (Subpteryn	species	109402
species not entered	Chicoreus	genus	10913
species not entered	Phyllonotus	genus	11521
species not entered	Eupleura	genus	11099
species not entered	Eupleura	genus	11099
species not entered	Hexaplex	genus	11199
species not entered	Pterorhytis	genus	82765
species not entered	Stramonita	genus	11700
species not entered	Calotrophon	genus	10884

species not entered	Chicoreus	genus	10913
species not entered	Pterorhytis	genus	82765
species not entered	Pterorhytis	genus	82765
species not entered	Hexaplex	genus	11199
species not entered	Aspella	genus	10792
species not entered	Murexiella	genus	11386
species not entered	Chicoreus	genus	10913
	Stramonita penelaevis	species	120561
	Eupleura caudata	species	94512
subjective synonym of	Calotrophon ostrearu	species	91536
	Urosalpinx	genus	11824
species not entered	Ecphora	genus	11052
species not entered	Ecphora	genus	11052
	Ecphora quadricostata	species	97553
species not entered	Pterorhytis	genus	82765
	Pterorhytis	genus	82765
species not entered	Pterorhytis	genus	82765
	Ecphora quadricostata	species	97553
species not entered	Phyllonotus	genus	11521
recombined as	Chicoreus (Phyllonotu	species	107994
recombined as	Favartia cellulosa	species	156293
species not entered	Urosalpinx	genus	11824
recombined as	Murexiella macgintyi	species	82761
recombined as	Murexiella (Subpteryr	species	109402
recombined as	Murexsul hexagonus	species	156600
species not entered	Trophon	genus	11793
recombined as	Typhis (Rugotyphis) fl	species	135226
recombined as	Aspella senex	species	109491
recombined as	Dermomurex (Dermo	species	109489
species not entered	Pterorhytis	genus	82765
species not entered	Urosalpinx	genus	11824
subjective synonym of	Calotrophon ostrearu	species	91536
subjective synonym of, species not entered	Calotrophon	genus	10884
recombined as	Aspella senex	species	109491
subjective synonym of, species not entered	Calotrophon	genus	10884
species not entered	Chicoreus	genus	10913
obsolete variant of	Chicoreus (Chicoreus)	species	158671
	Hexaplex hertweckoru	species	58249
species not entered	Pterorhytis	genus	82765
	Ecphora quadricostata	species	97553
	Vokesinotus lepidotus	species	157091
species not entered	Phyllonotus	genus	11521
obsolete variant of	Chicoreus (Chicoreus)	species	81534
species not entered	Chicoreus	genus	10913
species not entered	Pterorhytis	genus	82765
species not entered	Murexiella (Subpteryr	subgenus	11720
	Babelomurex lindae	species	157088

	Coralliophila miocenic	species	108437
	Urosalpinx	genus	11824
	Calotrophon	genus	10884
species not entered	Typhinellus	genus	11816
	Eupleura caudata	species	94512
subjective synonym of	Calotrophon ostrearu	species	91536
	Eupleura	genus	11099
recombined as	Stramonita haemosto	species	92618
species not entered	Eupleura	genus	11099
species not entered	Chicoreus	genus	10913
	Eupleura	genus	11099
	Coralliophila	genus	10970
species not entered	Typhinellus	genus	11816
	Eupleura caudata	species	94512
	Eupleura caudata	species	94512
species not entered	Murex	genus	11385
	Chicoreus (Chicoreus)	species	81534
	Chicoreus (Chicoreus)	species	81534
	Chicoreus (Chicoreus)	species	81534
	Chicoreus (Chicoreus)	species	81534
	Chicoreus (Chicoreus)	species	81534
	Chicoreus (Chicoreus)	species	81534
	Chicoreus (Chicoreus)	species	81534
	Chicoreus (Chicoreus)	species	81534
	Chicoreus (Chicoreus)	species	81534
	Chicoreus (Chicoreus)	species	81534
	Chicoreus (Chicoreus)	species	81534
	Chicoreus (Chicoreus)	species	81534
	Chicoreus (Chicoreus)	species	81534
	Chicoreus (Chicoreus)	species	81534
	Chicoreus (Chicoreus)	species	81534
	Chicoreus (Chicoreus)	species	81534
	Chicoreus (Chicoreus)	species	81534
	Chicoreus (Chicoreus)	species	81534
	Chicoreus (Chicoreus)	species	81534
	Chicoreus (Chicoreus)	species	81534
	Chicoreus (Chicoreus)	species	81534
	Chicoreus (Chicoreus)	species	81534
	Chicoreus (Chicoreus)	species	81534
	Chicoreus (Chicoreus)	species	81534
	Chicoreus (Chicoreus)	species	81535
	Chicoreus (Chicoreus)	species	81535
	Chicoreus (Chicoreus)	species	81535
	Chicoreus (Chicoreus)	species	81535
recombined as	Vokesimurex rubidus	species	144746
recombined as	Vokesimurex rubidus	species	144746
recombined as	Vokesimurex rubidus	species	144746

recombined as	Vokesimurex rubidus	species	144746
recombined as	Vokesimurex rubidus	species	144746
recombined as	Vokesimurex rubidus	species	144746
recombined as	Vokesimurex rubidus	species	144746
recombined as	Vokesimurex rubidus	species	144746
recombined as	Vokesimurex rubidus	species	144746
recombined as	Vokesimurex rubidus	species	144746
recombined as	Vokesimurex rubidus	species	144746
recombined as	Vokesimurex rubidus	species	144746
recombined as	Vokesimurex anniae	species	144744
recombined as	Vokesimurex anniae	species	144744
	Typhis (Rugotyphis) ha	species	85848
	Typhis (Rugotyphis) ha	species	85848
	Typhis (Rugotyphis) ha	species	85848
	Typhis (Rugotyphis) ha	species	85848
	Typhis (Rugotyphis) ha	species	85848
	Typhis (Rugotyphis) flo	species	135226
	Typhis (Rugotyphis) flo	species	135226
	Typhis (Rugotyphis) flo	species	135226
	Typhis (Rugotyphis) flo	species	135226
	Typhis (Rugotyphis) flo	species	135226
	Typhis (Rugotyphis) flo	species	135226
	Typhis (Rugotyphis) flo	species	135226
	Typhis (Rugotyphis) flo	species	135226
	Typhis (Rugotyphis) flo	species	135226
	Typhis (Rugotyphis) flo	species	135226
	Typhis (Rugotyphis) flo	species	135226
	Typhis (Rugotyphis) flo	species	135226
	Typhis (Rugotyphis) flo	species	135226
	Typhis (Rugotyphis) flo	species	135226
	Typhis (Rugotyphis) flo	species	135226
	Typhis (Rugotyphis) flo	species	135226
	Typhis (Rugotyphis) flo	species	135226
	Typhis (Rugotyphis) flo	species	135226
	Typhis (Rugotyphis) flo	species	135226
	Typhis (Rugotyphis) flo	species	135226
	Typhis (Rugotyphis) flo	species	135226
	Typhis (Rugotyphis) flo	species	135226
	Typhis (Rugotyphis) flo	species	135226
	Typhis (Rugotyphis) flo	species	135226
	Typhis (Rugotyphis) flo	species	135226
	Typhis (Rugotyphis) flo	species	135226
	Typhis (Rugotyphis) flo	species	135226
	Pterotyphis (Tripterot	species	135413
	Pterotyphis (Tripterot	species	135413
	Poirieria (Panamurex)	species	158214
	Poirieria (Panamurex)	species	158214

	Poirieria (Panamurex)	species	158214
	Poirieria (Panamurex)	species	158214
species not entered	Murex	genus	11385
	Chicoreus (Chicoreus)	species	158671
	Aspella senex	species	109491
	Aspella senex	species	109491
	Aspella senex	species	109491
	Aspella senex	species	109491
	Aspella senex	species	109491
	Aspella senex	species	109491
	Aspella senex	species	109491
	Aspella senex	species	109491
	Aspella senex	species	109491
	Aspella senex	species	109491
	Aspella senex	species	109491
	Aspella senex	species	109491
	Aspella senex	species	109491
	Aspella senex	species	109491
	Dermomurex (Dermo	species	109489
	Dermomurex (Dermo	species	109489
	Dermomurex (Dermo	species	109489
	Dermomurex (Dermo	species	109489
	Dermomurex (Dermo	species	109489
	Dermomurex (Dermo	species	109489
	Dermomurex (Dermo	species	109489
	Dermomurex (Dermo	species	109489
	Dermomurex (Dermo	species	109489
	Dermomurex (Dermo	species	109489
	Dermomurex (Dermo	species	109489
	Dermomurex (Dermo	species	109489
	Dermomurex (Dermo	species	109489
	Dermomurex (Dermo	species	109489
	Dermomurex (Dermo	species	109489
	Dermomurex (Dermo	species	109489
	Dermomurex (Dermo	species	109489
	Dermomurex (Dermo	species	109489
	Dermomurex (Dermo	species	109489
	Dermomurex (Dermo	species	158687
	Dermomurex (Dermo	species	158687
	Dermomurex (Trialate	species	158693
	Calotrophon ostrearu	species	91536
	Calotrophon ostrearu	species	91536
	Calotrophon ostrearu	species	91536
	Calotrophon ostrearu	species	91536
	Calotrophon ostrearu	species	91536
	Calotrophon ostrearu	species	91536
	Calotrophon ostrearu	species	91536

Calotrophon ostrearu	species	91536
Calotrophon ostrearu	species	91536
Calotrophon ostrearu	species	91536
Calotrophon ostrearu	species	91536
Calotrophon ostrearu	species	91536
Calotrophon ostrearu	species	91536
Calotrophon ostrearu	species	91536
Calotrophon ostrearu	species	91536
Calotrophon ostrearu	species	91536
Calotrophon ostrearu	species	91536
Calotrophon ostrearu	species	91536
Calotrophon ostrearu	species	91536
Calotrophon ostrearu	species	91536
Calotrophon ostrearu	species	91536
Calotrophon ostrearu	species	91536
Calotrophon ostrearu	species	91536
Calotrophon ostrearu	species	91536
Calotrophon ostrearu	species	91536
Calotrophon ostrearu	species	91536
Calotrophon ostrearu	species	91536
Calotrophon ostrearu	species	91536
Calotrophon ostrearu	species	91536
Calotrophon ostrearu	species	91536
Calotrophon ostrearu	species	91536
Calotrophon ostrearu	species	91536
Calotrophon ostrearu	species	91536
Calotrophon ostrearu	species	91536
Calotrophon ostrearu	species	91536
Calotrophon ostrearu	species	91536
Calotrophon ostrearu	species	91536
Calotrophon ostrearu	species	91536
Calotrophon ostrearu	species	91536
Calotrophon ostrearu	species	91536
Calotrophon ostrearu	species	91536
Calotrophon ostrearu	species	91536
Calotrophon ostrearu	species	91536
Calotrophon ostrearu	species	91536
Calotrophon ostrearu	species	91536
Calotrophon ostrearu	species	91536
Calotrophon ostrearu	species	91536
Calotrophon ostrearu	species	91536
Calotrophon ostrearu	species	91536
Calotrophon ostrearu	species	91536
Attiliosa striata	species	158746
Attiliosa striata	species	158746
Attiliosa striata	species	158746
Attiliosa striata	species	158746

	Attiliosa striata	species	158746
	Attiliosa striata	species	158746
	Hexaplex (Hexaplex) f	species	109407
	Hexaplex (Hexaplex) f	species	109407
	Hexaplex (Hexaplex) f	species	109407
	Hexaplex (Hexaplex) f	species	109407
	Hexaplex (Hexaplex) f	species	109407
	Hexaplex (Hexaplex) f	species	109407
	Hexaplex (Hexaplex) f	species	109407
	Hexaplex (Hexaplex) f	species	109407
	Murexiella (Subpteryr	species	109402
	Murexiella (Subpteryr	species	109402
	Murexiella (Subpteryr	species	109402
	Murexiella (Subpteryr	species	109402
	Murexiella (Subpteryr	species	109402
	Murexiella (Subpteryr	species	109402
	Murexiella (Subpteryr	species	109402
	Murexiella (Subpteryr	species	109402
	Murexiella (Subpteryr	species	109402
	Murexiella (Subpteryr	species	109402
	Murexiella (Subpteryr	species	109402
	Murexiella (Subpteryr	species	109402
	Murexiella (Subpteryr	species	109402
	Murexiella (Subpteryr	species	109402
	Murexiella (Subpteryr	species	109402
	Murexiella (Subpteryr	species	109402
	Murexiella (Subpteryr	species	109402
	Murexiella (Subpteryr	species	109402
	Murexiella (Subpteryr	species	109402
	Murexiella (Murexiella	species	158894
	Murexiella (Murexiella	species	156777
	Murexiella (Murexiella	species	156777
	Murexiella (Murexiella	species	156777
	Murexiella (Murexiella	species	156777
	Murexiella (Murexiella	species	156777
	Murexiella (Murexiella	species	156777
	Murexiella (Murexiella	species	156777
	Murexiella (Murexiella	species	156777
recombined as	Murexiella macgintyi	species	82761
recombined as	Murexiella macgintyi	species	82761
recombined as	Murexiella macgintyi	species	82761
recombined as	Murexiella macgintyi	species	82761
recombined as	Murexiella macgintyi	species	82761
recombined as	Murexiella macgintyi	species	82761
recombined as	Murexiella macgintyi	species	82761
recombined as	Murexiella macgintyi	species	82761
recombined as	Murexiella macgintyi	species	82761

recombined as	Murexiella macgintyi	species	82761
recombined as	Murexiella macgintyi	species	82761
recombined as	Murexiella macgintyi	species	82761
recombined as	Murexiella macgintyi	species	82761
recombined as	Murexiella macgintyi	species	82761
recombined as	Murexiella macgintyi	species	82761
recombined as	Murexiella macgintyi	species	82761
recombined as	Murexiella macgintyi	species	82761
recombined as	Murexiella macgintyi	species	82761
recombined as	Murexiella macgintyi	species	82761
recombined as	Murexiella macgintyi	species	82761
recombined as	Murexiella macgintyi	species	82761
recombined as	Murexiella macgintyi	species	82761
recombined as	Murexiella macgintyi	species	82761
recombined as	Murexiella macgintyi	species	82761
recombined as	Murexiella macgintyi	species	82761
recombined as	Murexiella macgintyi	species	82761
	Murexiella (Murexiella	species	97541
	Murexiella (Murexiella	species	97541
	Murexiella (Murexiella	species	97541
	Murexiella (Murexiella	species	97541
	Murexiella (Murexiella	species	97541
	Murexiella (Murexiella	species	97541
	Murexiella (Murexiella	species	97541
	Murexiella (Murexiella	species	97541
	Murexiella (Murexiella	species	97541
	Murexiella (Murexiella	species	97541
	Murexiella (Murexiella	species	97541
	Murexiella (Murexiella	species	97541
	Murexiella (Murexiella	species	97541
	Murexiella (Murexiella	species	97541
	Murexiella (Murexiella	species	97541
species not entered	Ecphora	genus	11052
species not entered	Chicoreus	genus	10913
species not entered	Muricanthus	genus	11388
species not entered	Chicoreus	genus	10913
species not entered	Pterorhytis	genus	82765
species not entered	Urosalpinx	genus	11824
species not entered	Ecphora	genus	11052
	Vokesinotus lepidotus	species	157091
	Vokesinotus lepidotus	species	157091
species not entered	Ecphora	genus	11052
species not entered	Murex	genus	11385
	Vokesinotus	genus	157090
species not entered	Eupleura	genus	11099
subjective synonym of	Calotrophon ostrearu	species	91536
	Chicoreus (Phyllonotu	species	107994

species not entered	Pterynotus (Pterynotu	subgenus	89766
	Chicoreus (Phyllonotu	species	107994
	Attiliosa aldridgei	species	158751
	Pterotyphis (Tripterot	species	135413
recombined as	Murexiella macgintyi	species	82761
	Murexiella (Subpteryr	species	109402
	Murexiella (Subpteryr	species	109402
	Calotrophon ostrearu	species	91536
	Eupleura caudata	species	94512
species not entered	Eupleura	genus	11099
	Favartia cellulosa	species	156293
recombined as	Chicoreus (Phyllonotu	species	107994
recombined as	Stramonita haemosto	species	92618
	Urosalpinx cinerea	species	97548
	Urosalpinx perrugata	species	135222
species not entered	Coralliophila	genus	10970
species not entered	Vitularia	genus	11843
species not entered	Ecphora	genus	11052
species not entered	Attiliosa	genus	10802
species not entered	Muricopsis	genus	11389
recombined as	Dermomurex (Dermoı	species	109489
species not entered	Urosalpinx	genus	11824
species not entered	Urosalpinx	genus	11824
	Ecphora quadricostata	species	97553
species not entered	Pterorhytis	genus	82765
	Eupleura caudata	species	94512
	Eupleura caudata	species	94512
recombined as	Hexaplex (Hexaplex) f	species	109407
	Eupleura caudata	species	94512
	Urosalpinx cinerea	species	97548
	Eupleura caudata	species	94512
species not entered	Coralliophila	genus	10970
species not entered	Ecphora	genus	11052
species not entered	Urosalpinx	genus	11824
	Calotrophon ostrearu	species	91536
	Ecphora quadricostata	species	97553
recombined as	Chicoreus (Phyllonotu	species	107994
recombined as	Chicoreus (Phyllonotu	species	107994
	Eupleura caudata	species	94512
	Eupleura caudata	species	94512
	Eupleura caudata	species	94512
	Urosalpinx cinerea	species	97548
	Urosalpinx cinerea	species	97548
	Urosalpinx cinerea	species	97548
	Urosalpinx cinerea	species	97548
	Eupleura caudata	species	94512
recombined as	Urosalpinx cinerea	species	97548

	Chicoreus (Chicoreus)	species	81534
	Typhis (Rugotyphis) fl	species	135226
	Murexiella (Murexiella	species	156777
	Murexiella (Murexiella	species	158890
recombined as	Murexiella macgintyi	species	82761
	Murexiella (Murexiella	species	97541
	Urosalpinx perrugata	species	135222
	Eupleura caudata	species	94512
	Urosalpinx perrugata	species	135222
recombined as	Chicoreus (Phyllonotu	species	107994
species not entered	Purpura (Tritonalia)	subgenus	11787
	Eupleura caudata	species	94512
	Eupleura caudata	species	94512
	Eupleura caudata	species	94512
species not entered	Urosalpinx	genus	11824
species not entered	Urosalpinx	genus	11824
species not entered	Urosalpinx	genus	11824
species not entered	Urosalpinx	genus	11824
species not entered	Urosalpinx	genus	11824
species not entered	Urosalpinx	genus	11824
species not entered	Urosalpinx	genus	11824
species not entered	Urosalpinx	genus	11824
species not entered	Urosalpinx	genus	11824
species not entered	Urosalpinx	genus	11824
	Urosalpinx perrugata	species	135222
	Urosalpinx perrugata	species	135222
	Urosalpinx perrugata	species	135222
species not entered	Urosalpinx	genus	11824
	Urosalpinx cinerea	species	97548
	Urosalpinx cinerea	species	97548
	Urosalpinx cinerea	species	97548
	Urosalpinx cinerea	species	97548
	Eupleura caudata	species	94512
	Eupleura caudata	species	94512
	Eupleura caudata	species	94512
	Urosalpinx cinerea	species	97548
	Urosalpinx cinerea	species	97548
	Eupleura caudata	species	94512
species not entered	Urosalpinx	genus	11824
	Eupleura caudata	species	94512
	Urosalpinx perrugata	species	135222
species not entered	Urosalpinx	genus	11824
	Urosalpinx	genus	11824
	Urosalpinx cinerea	species	97548
recombined as	Boreotrophon tetricus	species	97543
recombined as	Boreotrophon tetricus	species	97543
	Urosalpinx	genus	11824

	Eupleura caudata	species	94512
	Calotrophon ostrearu	species	91536
	Thais	genus	11763
	Urosalpinx	genus	11824
	Typhis (Rugotyphis) ha	species	85848
	Calotrophon ostrearu	species	91536
	Urosalpinx perrugata	species	135222
	Urosalpinx perrugata	species	135222
	Eupleura caudata	species	94512
	Eupleura caudata	species	94512
species not entered	Atractodon	genus	136229
	Eupleura caudata	species	94512
	Urosalpinx cinerea	species	97548
species not entered	Atractodon	genus	136229
	Eupleura caudata	species	94512
	Eupleura caudata	species	94512
	Eupleura caudata	species	94512
	Ecphora quadricostata	species	97553
	Eupleura caudata	species	94512
	Ecphora quadricostata	species	97553
	Ecphora quadricostata	species	97553
	Ecphora quadricostata	species	97553
	Ecphora quadricostata	species	97553
recombined as	Boreotrophon tetricus	species	97543
	Ecphora quadricostata	species	97553
	Ecphora quadricostata	species	97553
species not entered	Urosalpinx	genus	11824
	Ecphora quadricostata	species	97553
recombined as	Boreotrophon tetricus	species	97543
	Ecphora quadricostata	species	97553
	Ecphora quadricostata	species	97553
recombined as	Boreotrophon tetricus	species	97543
species not entered	Urosalpinx	genus	11824
	Ecphora quadricostata	species	97553
species not entered	Urosalpinx	genus	11824
species not entered	Urosalpinx	genus	11824
	Ecphora quadricostata	species	97553
recombined as	Boreotrophon tetricus	species	97543
	Urosalpinx	genus	11824
species not entered	Urosalpinx	genus	11824
	Ecphora quadricostata	species	97553
	Ecphora	genus	11052
	Ecphora	genus	11052
	Ecphora	genus	11052
	Urosalpinx	genus	11824
	Eupleura caudata	species	94512
species not entered	Urosalpinx	genus	11824

	Vokesinotus lepidotus	species	157091
species not entered	Urosalpinx	genus	11824
	Ecphora quadricostata	species	97553
recombined as	Nassarius (Nassarius) a	species	108668
species not entered	Nassarius	genus	11404
species not entered	Nassarius	genus	11404
recombined as	Nassarius (Nassarius) (	species	109315
subjective synonym of	Nassarius antillarum	species	146386
species not entered	Nassarius	genus	11404
	Nassarius	genus	11404
recombined as	Nassarius (Nassarius) '	species	108178
species not entered	Nassarius	genus	11404
subjective synonym of	Nassarius antillarum	species	146386
species not entered	Nassarius	genus	11404
species not entered	Nassarius	genus	11404
recombined as	Nassarius (Nassarius) '	species	108178
	Nassarius	genus	11404
recombined as	Nassarius (Nassarius) '	species	108178
species not entered	Nassarius	genus	11404
recombined as	Nassarius (Nassarius) '	species	108178
	Nassarius	genus	11404
	Calophos wilsoni	species	188186
recombined as	Nassarius (Nassarius) '	species	108178
	Calophos wilsoni	species	188186
species not entered	Nassarius (Alectrion)	subgenus	10738
species not entered	Nassarius (Alectrion)	subgenus	10738
species not entered	Nassarius (Alectrion)	subgenus	10738
species not entered	Nassarius (Alectrion)	subgenus	10738
species not entered	Nassarius (Alectrion)	subgenus	10738
species not entered	Nassarius (Alectrion)	subgenus	10738
species not entered	Nassarius (Alectrion)	subgenus	10738
species not entered	Nassarius (Alectrion)	subgenus	10738
	Calophos wilsoni	species	188186
replaced by	Nassarius antillarum	species	146386
recombined as	Nassarius (Nassarius) '	species	108178
recombined as	Nassarius (Nassarius) a	species	108668
replaced by	Nassarius antillarum	species	146386
recombined as	Nassarius (Nassarius) '	species	108178
recombined as	Nassarius (Nassarius) a	species	108668
recombined as	Nassarius (Nassarius) '	species	108178
	Calophos wilsoni	species	188186
	Nassarius	genus	11404
recombined as	Nassarius (Nassarius) '	species	108178
species not entered	Nassarius (Uzita)	subgenus	11827
species not entered	Nassarius (Uzita)	subgenus	11827
species not entered	Nassarius (Uzita)	subgenus	11827
species not entered	Nassarius (Uzita)	subgenus	11827

species not entered	Nassarius (Uzita)	subgenus	11827
subjective synonym of	Nassarius antillarum	species	146386
species not entered	Nassarius (Uzita)	subgenus	11827
species not entered	Phrontis	genus	11520
species not entered	Ilyanassa	genus	58245
	Ilyanassa corbis	species	93956
species not entered	Ilyanassa	genus	58245
	Ilyanassa marthae	species	93955
	Calophos wilsoni	species	188186
	Ilyanassa granifera	species	82806
species not entered	Ilyanassa	genus	58245
	Ilyanassa wilmingtone	species	95620
species not entered	Calophos	genus	10882
	Ilyanassa marthae	species	93955
	Nassarius (Nassarius)	species	108178
	Nassarius (Nassarius)	species	108178
	Nassarius (Nassarius)	species	108178
recombined as	Nassarius (Nassarius)	species	108178
recombined as	Nassarius (Nassarius)	species	108178
	Nassarius	genus	11404
	Nassarius (Nassarius)	species	108178
	Nassarius	genus	11404
	Nassarius	genus	11404
	Nassarius (Nassarius)	species	108178
species not entered	Nassarius (Uzita)	subgenus	11827
	Nassarius (Nassarius)	species	108178
	Nassarius (Nassarius)	species	108178
species not entered	Nassarius (Alectrion)	subgenus	10738
	Nassarius (Nassarius)	species	108178
subjective synonym of	Nassarius antillarum	species	146386
species not entered	Nassarius	genus	11404
recombined as	Trajana pyta	species	157438
recombined as	Trajana pyta	species	157438
recombined as	Trajana pyta	species	157438
recombined as	Trajana pyta	species	157438
recombined as	Trajana pyta	species	157438
recombined as	Trajana pyta	species	157438
	Trajana (Trajana) vera	species	157439
recombined as	Ilyanassa arata	species	95610
species not entered	Calophos	genus	10882
	Ilyanassa scalaspira	species	82811
	Ilyanassa	genus	58245
recombined as	Nassarius (Nassarius)	species	108668
replaced by	Nassarius antillarum	species	146386
species not entered	Nassarius	genus	11404
recombined as	Nassarius (Nassarius)	species	108178
	Calophos	genus	10882

recombined as	Nassarius (Nassarius) ;	species	108668
species not entered	Nassarius	genus	11404
species not entered	Nassarius	genus	11404
recombined as	Ilyanassa obsoleta	species	81604
recombined as	Ilyanassa obsoleta	species	81604
recombined as	Ilyanassa obsoleta	species	81604
recombined as	Ilyanassa obsoleta	species	81604
recombined as	Nassarius (Nassarius) '	species	108178
recombined as	Nassarius (Nassarius) '	species	108178
recombined as	Ilyanassa trivittata	species	94422
recombined as	Nassarius (Nassarius) ;	species	108668
recombined as	Nassarius (Nassarius) ;	species	108668
recombined as	Nassarius (Nassarius) ;	species	108668
recombined as	Nassarius (Nassarius) ;	species	108668
obsolete variant of	Nassarius (Uzita)	subgenus	11827
	Ilyanassa	genus	58245
	Ilyanassa obsoleta	species	81604
	Ilyanassa irrorata	species	95618
	Nassarius chowanensi	species	82801
species not entered	Nassarius	genus	11404
	Nassarius chowanensi	species	82801
	Nassarius schizopyga	species	82809
	Trajana pyta	species	157438
	Ilyanassa arata	species	95610
	Ilyanassa irrorata	species	95618
	Ilyanassa irrorata	species	95618
	Ilyanassa irrorata	species	95618
	Ilyanassa isogramma	species	95621
	Ilyanassa isogramma	species	95621
	Ilyanassa isogramma	species	95621
	Ilyanassa isogramma	species	95621
	Ilyanassa sexdentata	species	95623
	Ilyanassa sexdentata	species	95623
	Ilyanassa sexdentata	species	95623
	Ilyanassa sexdentata	species	95623
	Ilyanassa sexdentata	species	95623
	Ilyanassa sexdentata	species	95623
	Ilyanassa wilmingtone	species	95620
	Ilyanassa granifera	species	82806
	Ilyanassa granifera	species	82806
	Ilyanassa granifera	species	82806
	Ilyanassa granifera	species	82806
	Ilyanassa schizopyga	species	95628
	Ilyanassa schizopyga	species	95628
	Ilyanassa schizopyga	species	95628
	Ilyanassa schizopyga	species	95628
	Ilyanassa johnsoni	species	95629

	Ilyanassa johnsoni	species	95629
	Ilyanassa johnsoni	species	95629
	Ilyanassa johnsoni	species	95629
	Ilyanassa johnsoni	species	95629
	Ilyanassa scalaspira	species	82811
	Ilyanassa scalaspira	species	82811
	Ilyanassa scalaspira	species	82811
	Ilyanassa scalaspira	species	82811
	Ilyanassa scalaspira	species	82811
	Ilyanassa scalaspira	species	82811
	Ilyanassa harpuloides	species	95633
	Uzita smithiana	species	95951
	Uzita neogenensis	species	95953
	Uzita neogenensis	species	95953
recombined as	Nassarius chowanensi	species	82801
	Uzita bidentata	species	95955
species not entered	Nassarius (Uzita)	subgenus	11827
species not entered	Nassarius (Uzita)	subgenus	11827
species not entered	Nassarius (Uzita)	subgenus	11827
species not entered	Nassarius (Uzita)	subgenus	11827
species not entered	Nassarius (Uzita)	subgenus	11827
species not entered	Nassarius (Uzita)	subgenus	11827
species not entered	Nassarius (Uzita)	subgenus	11827
species not entered	Nassarius (Uzita)	subgenus	11827
species not entered	Nassarius (Uzita)	subgenus	11827
species not entered	Nassarius (Uzita)	subgenus	11827
species not entered	Nassarius	genus	11404
replaced by	Nassarius antillarum	species	146386
replaced by	Nassarius antillarum	species	146386
replaced by	Nassarius antillarum	species	146386
replaced by	Nassarius antillarum	species	146386
recombined as	Ilyanassa obsoleta	species	81604
recombined as	Ilyanassa obsoleta	species	81604
recombined as	Ilyanassa obsoleta	species	81604
recombined as	Ilyanassa obsoleta	species	81604
recombined as	Ilyanassa obsoleta	species	81604
recombined as	Nassarius (Nassarius) '	species	108178
recombined as	Ilyanassa trivittata	species	94422
recombined as	Ilyanassa trivittata	species	94422
recombined as	Ilyanassa trivittata	species	94422
recombined as	Ilyanassa trivittata	species	94422
recombined as	Ilyanassa trivittata	species	94422
recombined as	Ilyanassa trivittata	species	94422
recombined as	Ilyanassa trivittata	species	94422
recombined as	Ilyanassa trivittata	species	94422
recombined as	Ilyanassa trivittata	species	94422
recombined as	Ilyanassa trivittata	species	94422

recombined as	Nassarius (Nassarius)	species	108668
recombined as	Nassarius (Nassarius)	species	108668
recombined as	Nassarius (Nassarius)	species	108668
recombined as	Nassarius (Nassarius)	species	108668
recombined as	Nassarius (Nassarius)	species	108668
recombined as	Nassarius (Nassarius)	species	108668
recombined as	Nassarius (Nassarius)	species	108668
	Nassarius (Nassarius)	subgenus	89570
	Ilyanassa granifera	species	82806
	Nassarius chowanensi	species	82801
	Ilyanassa granifera	species	82806
species not entered	Ilyanassa	genus	58245
	Nassarius (Nassarius)	species	108668
species not entered	Ilyanassa	genus	58245
obsolete variant of	Nassarius (Uzita)	subgenus	11827
	Ilyanassa	genus	58245
	Nassarius	genus	11404
	Ilyanassa sexdentata	species	95623
	Ilyanassa	genus	58245
species not entered	Ilyanassa	genus	58245
obsolete variant of	Nassarius (Uzita)	subgenus	11827
	Ilyanassa scalaspira	species	82811
	Ilyanassa	genus	58245
species not entered	Ilyanassa	genus	58245
species not entered	Ilyanassa	genus	58245
species not entered	Nassarius (Uzita)	subgenus	11827
species not entered	Nassarius (Uzita)	subgenus	11827
recombined as	Trajana pyta	species	157438
replaced by	Nassarius antillarum	species	146386
replaced by	Nassarius antillarum	species	146386
replaced by	Nassarius antillarum	species	146386
replaced by	Nassarius antillarum	species	146386
recombined as	Ilyanassa trivittata	species	94422
recombined as	Ilyanassa obsoleta	species	81604
recombined as	Ilyanassa trivittata	species	94422
recombined as	Ilyanassa obsoleta	species	81604
recombined as	Ilyanassa trivittata	species	94422
	Nassarius	genus	11404
	Ilyanassa obsoleta	species	81604
	Ilyanassa trivittata	species	94422
	Ilyanassa trivittata	species	94422
	Ilyanassa granifera	species	82806
	Nassarius	genus	11404
	Nassarius	genus	11404
	Ilyanassa granifera	species	82806
species not entered	Ilyanassa	genus	58245
	Ilyanassa granifera	species	82806

	Nassarius	genus	11404
	Nassarius	genus	11404
	Nassarius	genus	11404
	Nassarius	genus	11404
	Ilyanassa	genus	58245
species not entered	Calophos	genus	10882
recombined as	Ilyanassa arata	species	95610
recombined as	Neverita duplicatus	species	94399
recombined as	Sinum perspectivum	species	108671
recombined as	Naticarius canrena	species	77092
	Tectonatica pusilla	species	95660
recombined as	Naticarius canrena	species	77092
	Natica	genus	10166
recombined as	Naticarius canrena	species	77092
recombined as	Naticarius plicatella	species	156596
	Natica	genus	10166
recombined as	Neverita duplicatus	species	94399
	Tectonatica pusilla	species	95660
recombined as	Neverita duplicatus	species	94399
	Tectonatica pusilla	species	95660
recombined as	Neverita duplicatus	species	94399
	Tectonatica pusilla	species	95660
recombined as	Naticarius canrena	species	77092
recombined as	Neverita duplicatus	species	94399
recombined as	Naticarius canrena	species	77092
recombined as	Stigmaulax guppiana	species	89522
recombined as	Neverita duplicatus	species	94399
subjective synonym of	Polinices hepaticus	species	108604
recombined as	Naticarius canrena	species	77092
recombined as	Naticarius canrena	species	77092
recombined as	Naticarius canrena	species	77092
	Tectonatica pusilla	species	95660
	Tectonatica pusilla	species	95660
	Tectonatica pusilla	species	95660
obsolete variant of	Polinices coensis	species	187709
obsolete variant of	Polinices coensis	species	187709
recombined as	Neverita duplicatus	species	94399
recombined as	Neverita duplicatus	species	94399
species not entered	Polinices	genus	10291
	Tectonatica pusilla	species	95660
	Sinum perspectivum	species	108671
recombined as	Neverita duplicatus	species	94399
recombined as	Neverita duplicatus	species	94399
	Sinum perspectivum	species	108671
	Tectonatica pusilla	species	95660
recombined as	Neverita duplicatus	species	94399
	Tectonatica pusilla	species	95660

	Stigmaulax	genus	10506
recombined as	Naticarius canrena	species	77092
	Natica (Natica)	subgenus	91873
recombined as	Naticarius canrena	species	77092
recombined as	Naticarius canrena	species	77092
recombined as	Neverita duplicatus	species	94399
species not entered	Sinum	genus	10470
recombined as	Neverita duplicatus	species	94399
	Sinum perspectivum	species	108671
recombined as	Neverita duplicatus	species	94399
species not entered	Sinum	genus	10470
species not entered	Euspira	genus	9839
species not entered	Euspira	genus	9839
recombined as	Naticarius plicatella	species	156596
species not entered	Polinices (Polinices)	subgenus	114858
recombined as	Neverita duplicatus	species	94399
	Tectonatica pusilla	species	95660
species not entered	Stigmaulax	genus	10506
	Sigatica semisulcata	species	95669
	Sinum perspectivum	species	108671
species not entered	Polinices (Polinices)	subgenus	114858
subjective synonym of, species not entered	Polinices	genus	10291
	Naticarius canrena	species	77092
species not entered	Stigmaulax	genus	10506
recombined as	Neverita duplicatus	species	94399
	Neverita duplicatus	species	94399
recombined as	Neverita duplicatus	species	94399
species not entered	Naticarius	genus	10167
	Natica	genus	10166
recombined as	Neverita duplicatus	species	94399
	Polinices lacteus	species	109159
species not entered	Polinices	genus	10291
	Polinices uber	species	167812
	Sinum perspectivum	species	108671
	Tectonatica pusilla	species	95660
	Polinices	genus	10291
recombined as	Neverita duplicatus	species	94399
recombined as	Neverita duplicatus	species	94399
recombined as	Neverita duplicatus	species	94399
recombined as	Neverita duplicatus	species	94399
	Neverita	genus	10177
recombined as	Neverita duplicatus	species	94399
recombined as	Neverita duplicatus	species	94399
recombined as	Neverita duplicatus	species	94399
recombined as	Neverita duplicatus	species	94399
species not entered	Euspira	genus	9839
species not entered	Euspira	genus	9839

	Sinum perspectivum	species	108671
	Sinum chesapeakensis	species	158564
recombined as	Neverita duplicatus	species	94399
	Tectonatica pusilla	species	95660
species not entered	Natica	genus	10166
recombined as	Tectonatica pusilla	species	95660
recombined as	Euspira heros	species	94403
recombined as	Neverita duplicatus	species	94399
recombined as	Neverita duplicatus	species	94399
recombined as	Neverita duplicatus	species	94399
recombined as	Neverita duplicatus	species	94399
	Sinum perspectivum	species	108671
	Sinum perspectivum	species	108671
	Neverita duplicatus	species	94399
	Natica (Natica)	subgenus	91873
	Neverita duplicatus	species	94399
	Neverita duplicatus	species	94399
	Tectonatica pusilla	species	95660
	Naticidae	family	61317
recombined as	Neverita duplicatus	species	94399
recombined as	Sigatica carolinensis	species	157293
recombined as	Euspira heros	species	94403
recombined as	Neverita duplicatus	species	94399
	Tectonatica pusilla	species	95660
recombined as	Neverita duplicatus	species	94399
	Tectonatica pusilla	species	95660
	Polinices	genus	10291
	Polinices	genus	10291
	Polinices	genus	10291
recombined as	Euspira heros	species	94403
recombined as	Neverita duplicatus	species	94399
recombined as	Neverita duplicatus	species	94399
recombined as	Neverita duplicatus	species	94399
recombined as	Neverita duplicatus	species	94399
recombined as	Neverita duplicatus	species	94399
recombined as	Neverita duplicatus	species	94399
recombined as	Neverita duplicatus	species	94399
	Sinum perspectivum	species	108671
	Sinum perspectivum	species	108671
	Sinum perspectivum	species	108671
	Naticidae	family	61317
recombined as	Neverita duplicatus	species	94399
species not entered	Sinum	genus	10470
recombined as	Neverita duplicatus	species	94399
recombined as	Euspira heros	species	94403
	Euspira heros	species	94403
	Neverita duplicatus	species	94399

	Euspira heros	species	94403
	Tectonatica pusilla	species	95660
	Euspira heros	species	94403
	Euspira heros	species	94403
	Tectonatica pusilla	species	95660
	Neverita duplicatus	species	94399
obsolete variant of	Naticarius	genus	10167
	Neverita duplicatus	species	94399
	Euspira heros	species	94403
	Euspira heros	species	94403
	Naticarius	genus	10167
	Neverita duplicatus	species	94399
recombined as	Neverita duplicatus	species	94399
recombined as	Neverita duplicatus	species	94399
	Polinices	genus	10291
recombined as	Neverita duplicatus	species	94399
recombined as	Neverita duplicatus	species	94399
recombined as	Euspira heros	species	94403
recombined as	Neverita duplicatus	species	94399
	Tectonatica pusilla	species	95660
	Tectonatica pusilla	species	95660
	Tectonatica pusilla	species	95660
	Euspira heros	species	94403
	Neverita duplicatus	species	94399
	Neverita duplicatus	species	94399
recombined as	Euspira heros	species	94403
recombined as	Neverita duplicatus	species	94399
recombined as	Euspira heros	species	94403
species not entered	Lunatia	genus	10076
recombined as	Euspira heros	species	94403
	Sinum fragilis	species	95605
recombined as	Euspira heros	species	94403
recombined as	Euspira heros	species	94403
recombined as	Euspira heros	species	94403
recombined as	Neverita duplicatus	species	94399
recombined as	Euspira heros	species	94403
recombined as	Euspira heros	species	94403
species not entered	Lunatia	genus	10076
recombined as	Euspira heros	species	94403
	Euspira heros	species	94403
	Euspira heros	species	94403
obsolete variant of	Naticarius	genus	10167
obsolete variant of	Naticarius	genus	10167
recombined as	Euspira heros	species	94403
	Smaragdia viridis	species	116050
species not entered	Neritina	genus	9015
species not entered	Neritina	genus	9015

	Smaragdia viridis	species	116050
	Neritina sparsilineata	species	167650
	Neritina usnea	species	158615
	Neritina virginea	species	106858
	Smaragdia viridis	species	116050
	Neritina	genus	9015
species not entered	Tortifusus	genus	98746
	Illyanassa	genus	11211
	Olivella mutica	species	94510
recombined as	Jaspidella jaspidea	species	109168
subjective synonym of	Oliva (Ispidula) sayana	species	93850
	Olivella mutica	species	94510
species not entered	Olivella	genus	11446
recombined as	Oliva (Ispidula) sayana	species	93850
	Olivella mutica	species	94510
recombined as	Oliva (Ispidula) sayana	species	93850
	Olivella mutica	species	94510
	Olivella mutica	species	94510
recombined as	Oliva (Ispidula) sayana	species	93850
	Olivella mutica	species	94510
	Olivella mutica	species	94510
	Oliva immortua	species	162423
	Oliva	genus	11444
recombined as	Oliva (Ispidula) sayana	species	93850
recombined as	Oliva (Ispidula) sayana	species	93850
recombined as	Oliva (Ispidula) sayana	species	93850
recombined as	Oliva (Ispidula) sayana	species	93850
	Olivella mutica	species	94510
	Olivella mutica	species	94510
recombined as	Olivella (Niteoliva) flo	species	248905
species not entered	Olivella	genus	11446
recombined as	Oliva (Ispidula) sayana	species	93850
species not entered	Olivella	genus	11446
	Olivella mutica	species	94510
species not entered	Olivella	genus	11446
recombined as	Oliva (Ispidula) sayana	species	93850
species not entered	Olivella	genus	11446
	Olivella mutica	species	94510
species not entered	Olivella	genus	11446
recombined as	Oliva (Ispidula) sayana	species	93850
	Olivella mutica	species	94510
species not entered	Olivella	genus	11446
	Olivella mutica	species	94510
	Olivella (Olivella)	subgenus	89859
	Olivella mutica	species	94510
species not entered	Oliva	genus	11444
	Olivella	genus	11446

	Olivella	genus	11446
	Olivella	genus	11446
species not entered	Oliva	genus	11444
	Oliva	genus	11444
	Oliva	genus	11444
	Oliva	genus	11444
	Oliva	genus	11444
recombined as	Olivella mutica	species	94510
recombined as	Oliva (Ispidula) sayana	species	93850
recombined as	Olivella mutica	species	94510
recombined as	Oliva reticularis	species	107830
species not entered	Olivella (Olivella)	subgenus	89859
species not entered	Olivella (Olivella)	subgenus	89859
recombined as	Olivella mutica	species	94510
species not entered	Olivella (Toroliva)	subgenus	89861
	Olivella (Mansfieldella	species	116726
species not entered	Olivella (Dactylidia)	subgenus	11019
species not entered	Olivella (Toroliva)	subgenus	89861
recombined as	Olivella mutica	species	94510
	Oliva	genus	11444
	Olivella mutica	species	94510
species not entered	Oliva (Strephona)	subgenus	11701
obsolete variant of	Oliva (Oliva) murielae	species	93847
species not entered	Oliva (Strephona)	subgenus	11701
species not entered	Oliva (Strephona)	subgenus	11701
species not entered	Oliva (Strephona)	subgenus	11701
	Lindoliva diegelae	species	157096
species not entered	Oliva (Strephona)	subgenus	11701
species not entered	Oliva	genus	11444
species not entered	Oliva (Strephona)	subgenus	11701
subjective synonym of	Lindoliva diegelae	species	157096
subjective synonym of	Lindoliva diegelae	species	157096
species not entered	Oliva (Strephona)	subgenus	11701
species not entered	Oliva (Strephona)	subgenus	11701
species not entered	Oliva (Strephona)	subgenus	11701
species not entered	Oliva (Strephona)	subgenus	11701
species not entered	Oliva	genus	11444
species not entered	Olivella	genus	11446
species not entered	Olivella	genus	11446
species not entered	Olivella	genus	11446
	Oliva	genus	11444
species not entered	Oliva (Strephona)	subgenus	11701
species not entered	Oliva	genus	11444
species not entered	Oliva (Strephona)	subgenus	11701
species not entered	Oliva (Strephona)	subgenus	11701
species not entered	Oliva (Strephona)	subgenus	11701
recombined as	Oliva reticularis	species	107830

species not entered	Oliva (Strephona)	subgenus	11701
species not entered	Oliva (Strephona)	subgenus	11701
species not entered	Oliva (Strephona)	subgenus	11701
species not entered	Oliva	genus	11444
	Olivella mutica	species	94510
	Olivella mutica	species	94510
	Olivella mutica	species	94510
	Olivella mutica	species	94510
recombined as	Oliva (Ispidula) sayana	species	93850
	Olivella	genus	11446
recombined as	Oliva (Ispidula) sayana	species	93850
	Olivella mutica	species	94510
recombined as	Oliva reticularis	species	107830
recombined as	Oliva (Ispidula) sayana	species	93850
species not entered	Oliva	genus	11444
	Oliva (Omogymna) lio	species	158475
obsolete variant of	Olivella (Mansfieldella	subgenus	116725
subjective synonym of	Lindoliva diegelae	species	157096
obsolete variant of	Oliva (Oliva) murielae	species	93847
subjective synonym of	Lindoliva diegelae	species	157096
	Lindoliva diegelae	species	157096
recombined as	Oliva (Ispidula) sayana	species	93850
species not entered	Oliva	genus	11444
recombined as	Oliva (Ispidula) sayana	species	93850
	Olivella mutica	species	94510
species not entered	Olivella	genus	11446
recombined as	Oliva (Ispidula) sayana	species	93850
recombined as	Oliva (Ispidula) sayana	species	93850
recombined as	Oliva (Ispidula) sayana	species	93850
	Olivella mutica	species	94510
recombined as	Oliva (Ispidula) sayana	species	93850
	Olivella mutica	species	94510
	Olivella mutica	species	94510
recombined as	Oliva (Ispidula) sayana	species	93850
	Olivella mutica	species	94510
recombined as	Oliva (Ispidula) sayana	species	93850
	Olivella mutica	species	94510
species not entered	Oliva	genus	11444
recombined as	Oliva (Ispidula) sayana	species	93850
recombined as	Oliva (Ispidula) sayana	species	93850
recombined as	Oliva (Ispidula) sayana	species	93850
recombined as	Oliva (Ispidula) sayana	species	93850
	Olivella mutica	species	94510
	Olivella mutica	species	94510
species not entered	Oliva (Oliva)	subgenus	89857
recombined as	Olivella mutica	species	94510
recombined as	Oliva (Ispidula) sayana	species	93850

recombined as	Oliva (Ispidula) sayana	species	93850
recombined as	Oliva (Ispidula) sayana	species	93850
	Olivella mutica	species	94510
	Olivella mutica	species	94510
species not entered	Oliva	genus	11444
species not entered	Oliva (Oliva)	subgenus	89857
recombined as	Olivella (Dactylidia) ca	species	248899
subjective synonym of	Oliva (Ispidula) sayana	species	93850
subjective synonym of	Oliva (Ispidula) sayana	species	93850
subjective synonym of	Oliva (Ispidula) sayana	species	93850
	Olivella mutica	species	94510
subjective synonym of	Oliva (Ispidula) sayana	species	93850
	Olivella mutica	species	94510
	Olivella mutica	species	94510
species not entered	Oliva	genus	11444
	Olivella mutica	species	94510
	Olivella mutica	species	94510
species not entered	Oliva	genus	11444
recombined as	Olivella (Dactylidia) ca	species	248899
	Olivella	genus	11446
recombined as	Oliva (Ispidula) sayana	species	93850
recombined as	Oliva (Ispidula) sayana	species	93850
recombined as	Oliva (Ispidula) sayana	species	93850
recombined as	Oliva (Ispidula) sayana	species	93850
recombined as	Oliva (Ispidula) sayana	species	93850
recombined as	Oliva (Ispidula) sayana	species	93850
recombined as	Oliva (Ispidula) sayana	species	93850
	Olivella mutica	species	94510
	Olivella mutica	species	94510
	Olivella mutica	species	94510
	Olivella mutica	species	94510
	Olivella mutica	species	94510
	Olivella mutica	species	94510
species not entered	Oliva (Oliva)	subgenus	89857
recombined as	Olivella mutica	species	94510
	Olivella mutica	species	94510
	Olivella mutica	species	94510
recombined as	Olivella (Dactylidia) ca	species	248899
species not entered	Oliva	genus	11444
recombined as	Olivella (Dactylidia) ca	species	248899
recombined as	Olivella (Dactylidia) ca	species	248899
recombined as	Olivella (Dactylidia) ca	species	248899
recombined as	Olivella (Dactylidia) ca	species	248899
recombined as	Olivella (Dactylidia) ca	species	248899
recombined as	Olivella (Dactylidia) ca	species	248899
	Olivella	genus	11446
recombined as	Olivella (Dactylidia) ca	species	248899

	Olivella mutica	species	94510
recombined as	Oliva (Ispidula) sayana	species	93850
	Olivella mutica	species	94510
	Olivella	genus	11446
	Olivella mutica	species	94510
	Olivella	genus	11446
	Olivella	genus	11446
	Olivella	genus	11446
species not entered	Oliva	genus	11444
	Olivella	genus	11446
species not entered	Oliva	genus	11444
	Olivella	genus	11446
	Olivella	genus	11446
	Olivella mutica	species	94510
species not entered	Oliva	genus	11444
recombined as	Pseudocyphoma inter	species	91129
	Jenneria hepleri	species	342958
	Jenneria loxahatchien	species	342634
	Jenneria richardsi	species	157085
subjective synonym of	Jenneria richardsi	species	157085
	Cyphoma finkli	species	342623
	Cyphoma miamiensis	species	343161
	Cyphoma viaavensis	species	342053
recombined as	Cyphoma carolae	species	341765
	Jenneria violetae	species	342052
	Jenneria loxahatchien	species	342634
	Cyphoma gibbosum	species	335370
	Cymbovula acicularis	species	91208
	Cyphoma viaavensis	species	342053
	Cyphoma carolae	species	341765
	Cyphoma finkli	species	342623
	Simnia (Spiculata) terr	species	343488
recombined as	Cymbovula acicularis	species	91208
recombined as	Cymbovula acicularis	species	91208
recombined as	Eulithidium thalassico	species	135794
species not entered	Tricolia	genus	9322
	Physa	genus	91741
species not entered	Physa	genus	91741
species not entered	Physa	genus	91741
	Physella	genus	83647
	Physella	genus	83647
	Physella	genus	83647
	Physa	genus	91741
	Physa	genus	91741
species not entered	Physella	genus	83647
species not entered	Physa	genus	91741
species not entered	Physa	genus	91741

species not entered	Fossarus (Isapsis)	subgenus	97644
species not entered	Fossarus	genus	60387
species not entered	Fossarus	genus	60387
species not entered	Fossarus	genus	60387
species not entered	Fossarus	genus	60387
species not entered	Helisoma	genus	83652
species not entered	Helisoma	genus	83652
species not entered	Helisoma	genus	83652
species not entered	Helisoma	genus	83652
species not entered	Helisoma	genus	83652
	Helisoma	genus	83652
species not entered	Helisoma	genus	83652
species not entered	Planorbella	genus	83653
species not entered	Planorbella	genus	83653
species not entered	Planorbella	genus	83653
species not entered	Micromenetus	genus	251930
species not entered	Planorbella	genus	83653
species not entered	Planorbella	genus	83653
species not entered	Planorbella	genus	83653
species not entered	Planorbella	genus	83653
species not entered	Helisoma	genus	83652
	Planorbis	genus	61805
species not entered	Helisoma	genus	83652
species not entered	Helisoma	genus	83652
species not entered	Helisoma	genus	83652
species not entered	Biomphalaria	genus	251927
species not entered	Gyraulus	genus	70675
species not entered	Micromenetus	genus	251930
species not entered	Micromenetus	genus	251930
species not entered	Planorbella	genus	83653
species not entered	Planorbella	genus	83653
species not entered	Planorbella	genus	83653
species not entered	Planorbella	genus	83653
species not entered	Ferrissia	genus	83656
species not entered	Hebetoncylus	genus	251928
species not entered	Laevapex	genus	251929
species not entered	Planorbella	genus	83653
species not entered	Planorbella	genus	83653
species not entered	Planorbella	genus	83653
species not entered	Planorbella	genus	83653
species not entered	Planorbella	genus	83653
species not entered	Planorbella	genus	83653
species not entered	Planorbella	genus	83653
species not entered	Planorbella	genus	83653
species not entered	Planorbella	genus	83653
species not entered	Planorbella	genus	83653
species not entered	Planorbella	genus	83653

species not entered	Planorbella	genus	83653
species not entered	Gyraulus	genus	70675
species not entered	Micromenetus	genus	251930
species not entered	Planorbella	genus	83653
species not entered	Laevapex	genus	251929
species not entered	Gyraulus	genus	70675
species not entered	Micromenetus	genus	251930
species not entered	Planorbella	genus	83653
species not entered	Gyraulus	genus	70675
species not entered	Laevapex	genus	251929
species not entered	Micromenetus	genus	251930
species not entered	Micromenetus	genus	251930
species not entered	Planorbella	genus	83653
species not entered	Planorbis	genus	61805
	Planorbis	genus	61805
species not entered	Polygyra	genus	251926
species not entered	Daedalochila	genus	251924
species not entered	Polygyra	genus	251926
species not entered	Polygyra	genus	251926
species not entered	Daedalochila	genus	251924
species not entered	Daedalochila	genus	251924
species not entered	Triodopsis	genus	384100
species not entered	Polygyra	genus	251926
species not entered	Triodopsis	genus	384100
species not entered	Polygyra	genus	251926
species not entered	Daedalochila	genus	251924
species not entered	Polygyra	genus	251926
species not entered	Daedalochila	genus	251924
species not entered	Polygyra	genus	251926
species not entered	Daedalochila	genus	251924
species not entered	Polygyra	genus	251926
species not entered	Daedalochila	genus	251924
species not entered	Triodopsis	genus	384100
species not entered	Daedalochila	genus	251924
species not entered	Polygyra	genus	251926
species not entered	Triodopsis	genus	384100
species not entered	Daedalochila	genus	251924
species not entered	Polygyra	genus	251926
species not entered	Triodopsis	genus	384100
species not entered	Daedalochila	genus	251924
species not entered	Triodopsis	genus	384100
species not entered	Daedalochila	genus	251924
species not entered	Polygyra	genus	251926
species not entered	Triodopsis	genus	384100
species not entered	Triodopsis	genus	384100
species not entered	Triodopsis	genus	384100
species not entered	Triodopsis	genus	384100

species not entered	Triodopsis	genus	384100
species not entered	Triodopsis	genus	384100
subjective synonym of	Cerithidea costatus	species	134867
	Cerithidea scalariform	species	82779
recombined as	Cerithidea costatus	species	134867
subjective synonym of	Cerithidea costatus	species	134867
	Cerithidea scalariform	species	82779
species not entered	Cerithidea	genus	9615
species not entered	Cerithidea	genus	9615
species not entered	Cerithidea	genus	9615
species not entered	Cerithidea	genus	9615
species not entered	Cerithidea	genus	9615
species not entered	Cerithidea	genus	9615
species not entered	Cerithidea (Cerithideo	subgenus	9618
species not entered	Cerithidea (Cerithideo	subgenus	9618
recombined as	Cerithidea costatus	species	134867
	Cerithidea scalariform	species	82779
	Potamides saltillensis	species	311061
	Potamides cancelloide	species	311055
species not entered	Compsodrillia	genus	10958
	Crassispira perrugata	species	108730
species not entered	Monilispira	genus	121445
subjective synonym of	Zonulispira crocata	species	120709
	Crassispira	genus	10983
obsolete variant of	Carinodrillia pylonia	species	248862
obsolete variant of	Carinodrillia pylonia	species	248862
species not entered	Compsodrillia	genus	10958
species not entered	Compsodrillia	genus	10958
species not entered	Compsodrillia	genus	10958
species not entered	Monilispira	genus	121445
species not entered	Lioglyphostoma	genus	11272
species not entered	Compsodrillia	genus	10958
species not entered	Compsodrillia	genus	10958
species not entered	Compsodrillia	genus	10958
species not entered	Pilsbryspira	genus	107921
	Carinodrillia	genus	10897
	Crassispira perrugata	species	108730
	Crassispira	genus	10983
	Compsodrillia chowan	species	108749
	Crassispira	genus	10983
	Compsodrillia	genus	10958
species not entered	Pupoides	genus	162063
species not entered	Vertigo	genus	162064
species not entered	Vertigo	genus	162064
species not entered	Pupoides	genus	162063
	Pupoides	genus	162063
species not entered	Pupoides	genus	162063

species not entered	Vertigo	genus	162064
	Pupoides	genus	162063
species not entered	Pupoides	genus	162063
species not entered	Vertigo	genus	162064
species not entered	Vertigo	genus	162064
species not entered	Odostomia	genus	12153
species not entered	Bartschella	genus	12046
subjective synonym of	Pyramidella suturalis	species	91318
	Longchaeus	genus	12130
species not entered	Turbonilla	genus	12221
species not entered	Chrysallida	genus	12064
species not entered	Longchaeus	genus	12130
	Odostomia	genus	12153
species not entered	Odostomia (Oscilla)	subgenus	414220
species not entered	Chemnitzia	genus	12063
	Turbonilla	genus	12221
species not entered	Chrysallida	genus	12064
subjective synonym of	Pyramidella suturalis	species	91318
obsolete variant of	Turbonilla (Mormula)	subgenus	12139
subjective synonym of	Turbonilla hemphilli	species	135313
	Turbonilla	genus	12221
	Turbonilla	genus	12221
subjective synonym of	Pyramidella suturalis	species	91318
subjective synonym of	Turbonilla levis	species	135310
species not entered	Turbonilla	genus	12221
	Turbonilla	genus	12221
obsolete variant of	Pyramidella	genus	12182
obsolete variant of	Pyramidella	genus	12182
obsolete variant of	Chemnitzia	genus	12063
obsolete variant of	Chemnitzia	genus	12063
obsolete variant of	Turbonilla (Pyrgiscus)	subgenus	12185
obsolete variant of	Turbonilla (Pyrgiscus)	subgenus	12185
obsolete variant of	Turbonilla (Pyrgiscus)	subgenus	12185
obsolete variant of	Turbonilla	genus	12221
obsolete variant of	Turbonilla (Pyrgiscus)	subgenus	12185
obsolete variant of	Turbonilla (Pyrgiscus)	subgenus	12185
recombined as	Boonea seminuda	species	109788
	Turbonilla	genus	12221
	Odostomia	genus	12153
species not entered	Odostomia	genus	12153
recombined as	Boonea seminuda	species	109788
subjective synonym of	Pyramidella suturalis	species	91318
	Turbonilla	genus	12221
subjective synonym of	Pyramidella suturalis	species	91318
recombined as	Boonea seminuda	species	109788
species not entered	Odostomia	genus	12153
	Turbonilla	genus	12221

obsolete variant of	Turbonilla	genus	12221
	Turbonilla	genus	12221
	Pyramidella	genus	12182
	Boonea seminuda	species	109788
species not entered	Longchaeus	genus	12130
	Odostomia acutidens	species	156561
species not entered	Odostomia	genus	12153
	Turbonilla hemphilli	species	135313
recombined as	Sayella fusca	species	109770
species not entered	Odostomia	genus	12153
species not entered	Turbonilla	genus	12221
	Turbonilla interrupta	species	97615
recombined as	Boonea seminuda	species	109788
	Turbonilla interrupta	species	97615
recombined as	Sayella fusca	species	109770
recombined as	Boonea seminuda	species	109788
	Turbonilla interrupta	species	97615
	Turbonilla interrupta	species	97615
obsolete variant of	Turbonilla	genus	12221
subjective synonym of	Pyramidella suturalis	species	91318
species not entered	Pyramidella	genus	12182
	Turbonilla	genus	12221
	Turbonilla	genus	12221
	Turbonilla subulata	species	392401
	Turbonilla caroliniana	species	392402
	Odostomia granulatus	species	392404
	Turbonilla speira	species	392392
	Turbonilla speira	species	392392
recombined as	Turbonilla (Chemnitzia	species	97613
	Turbonilla subulata	species	392401
	Turbonilla caroliniana	species	392402
	Odostomia granulatus	species	392404
species not entered	Turbonilla	genus	12221
species not entered	Turbonilla	genus	12221
species not entered	Turbonilla (Pyrgiscus)	subgenus	12185
obsolete variant of	Turbonilla (Pyrgiscus)	subgenus	12185
species not entered	Turbonilla	genus	12221
	Odostomia simplex	species	82825
species not entered	Turbonilla	genus	12221
obsolete variant of	Turbonilla (Pyrgiscus)	subgenus	12185
	Boonea seminuda	species	109788
	Boonea impressa	species	108657
	Odostomia	genus	12153
	Boonea seminuda	species	109788
	Boonea impressa	species	108657
	Odostomia	genus	12153
obsolete variant of	Chemnitzia	genus	12063

obsolete variant of	Turbonilla (Pyrgiscus)	subgenus	12185
obsolete variant of	Turbonilla (Pyrgiscus)	subgenus	12185
	Boonea seminuda	species	109788
	Boonea impressa	species	108657
	Odostomia acutidens	species	156561
	Odostomia	genus	12153
obsolete variant of	Chemnitzia	genus	12063
obsolete variant of	Turbonilla (Pyrgiscus)	subgenus	12185
obsolete variant of	Turbonilla (Pyrgiscus)	subgenus	12185
	Boonea seminuda	species	109788
	Boonea impressa	species	108657
obsolete variant of	Chemnitzia	genus	12063
obsolete variant of	Turbonilla (Pyrgiscus)	subgenus	12185
obsolete variant of	Turbonilla (Pyrgiscus)	subgenus	12185
species not entered	Pyramidella	genus	12182
species not entered	Pyramidella	genus	12182
species not entered	Pyramidella	genus	12182
	Turbonilla interrupta	species	97615
recombined as	Turbonilla (Chemnitzia	species	97613
	Turbonilla	genus	12221
	Boonea seminuda	species	109788
	Boonea seminuda	species	109788
	Boonea impressa	species	108657
obsolete variant of	Turbonilla (Pyrgiscus)	subgenus	12185
	Chemnitzia	genus	12063
	Boonea seminuda	species	109788
	Boonea seminuda	species	109788
	Chemnitzia	genus	12063
	Boonea impressa	species	108657
	Turbonilla interrupta	species	97615
	Turbonilla	genus	12221
	Odostomia	genus	12153
	Odostomia	genus	12153
subjective synonym of	Linatella caudata	species	159057
subjective synonym of	Linatella caudata	species	159057
	Monoplex parthenope	species	159069
	Monoplex parthenope	species	159069
	Monoplex krebsii	species	159061
	Monoplex krebsii	species	159061
species not entered	Daphnella	genus	11020
species not entered	Volvulella	genus	12027
recombined as	Volvulella (Volvulella)	species	107150
species not entered	Volvulella	genus	12027
	Retusa obtusa	species	94508
recombined as	Ringicula (Ringiculella)	species	120660
recombined as	Ringicula (Ringiculella)	species	120660
recombined as	Ringicula (Ringiculella)	species	120660

recombined as	Ringicula (Ringiculella)	species	120660
	Ringicula (Ringiculella)	species	120660
	Ringicula	genus	11997
species not entered	Rissoina	genus	10421
species not entered	Rissoina	genus	10421
subjective synonym of	Schwartziella catesbya	species	91186
recombined as	Zebina laevigata	species	91275
	Rissoina	genus	10421
species not entered	Rissoina	genus	10421
species not entered	Zebina	genus	10695
species not entered	Zebina	genus	10695
species not entered	Rissoa	genus	10419
recombined as	Schwartziella catesbya	species	91186
recombined as	Schwartziella catesbya	species	91186
species not entered	Rissoina (Zebinella)	subgenus	100865
species not entered	Rissoina (Zebinella)	subgenus	100865
species not entered	Rissoina (Zebinella)	subgenus	100865
subjective synonym of	Schwartziella catesbya	species	91186
	Rissoina (Cibdezebina)	species	117247
species not entered	Zebina (Cibdezebina)	subgenus	115770
species not entered	Rissoina (Zebinella)	subgenus	100865
subjective synonym of	Rissoina (Phosinella) s	species	248845
subjective synonym of	Schwartziella catesbya	species	91186
species not entered	Rissoina	genus	10421
	Schwartziella catesbya	species	91186
species not entered	Rissoina (Schwartziella	subgenus	10440
obsolete variant of	Rissoa (Onoba) geraea	species	116530
obsolete variant of	Rissoa (Onoba) geraea	species	116530
species not entered	Rissoina (Schwartziella	subgenus	10440
species not entered	Cingula	genus	9647
recombined as	Finella adamsi	species	90880
subjective synonym of	Finella dubia	species	90884
recombined as	Finella adamsi	species	90880
subjective synonym of	Finella dubia	species	90884
recombined as	Finella adamsi	species	90880
recombined as	Finella adamsi	species	90880
recombined as	Finella adamsi	species	90880
recombined as	Finella adamsi	species	90880
recombined as	Finella adamsi	species	90880
recombined as	Finella adamsi	species	90880
recombined as	Finella adamsi	species	90880
subjective synonym of	Finella dubia	species	90884
recombined as	Finella adamsi	species	90880
subjective synonym of	Finella dubia	species	90884
species not entered	Solariorbis	genus	10479
subjective synonym of	Solariorbis infracarina	species	156784
subjective synonym of	Solariorbis mooreanus	species	156787

subjective synonym of	Solariorbis infracarina	species	156784
species not entered	Solariorbis	genus	10479
recombined as	Solariorbis blakei	species	135103
species not entered	Parviturbo	genus	117454
species not entered	Solariorbis	genus	10479
species not entered	Solariorbis	genus	10479
species not entered	Solariorbis	genus	10479
species not entered	Solariorbis	genus	10479
subjective synonym of	Solariorbis mooreanus	species	156787
subjective synonym of	Solariorbis infracarina	species	156784
	Solariorbis infracarina	species	156784
	Solariella vaughani	species	167649
	Solariella	genus	9254
	Solariella	genus	9254
	Solariella gemma	species	156289
	Solariella gemma	species	156289
species not entered	Euglandina	genus	251918
species not entered	Euglandina	genus	251918
species not entered	Euglandina	genus	251918
species not entered	Euglandina	genus	251918
species not entered	Euglandina	genus	251918
species not entered	Euglandina	genus	251918
species not entered	Strobilops	genus	114481
species not entered	Strobilops	genus	114481
species not entered	Strobilops	genus	114481
species not entered	Strobilops	genus	114481
	Strombus	genus	10520
	Strombus alatus	species	82785
	Strombus alatus	species	82785
	Strombus alatus	species	82785
species not entered	Strombus	genus	10520
	Strombus pugilis	species	94554
	Strombus alatus	species	82785
	Strombus alatus	species	82785
	Strombus alatus	species	82785
	Strombus alatus	species	82785
	Strombus pugilis	species	94554
	Strombus pugilis	species	94554
	Strombus alatus	species	82785
	Strombus alatus	species	82785
	Strombus alatus	species	82785
	Strombus alatus	species	82785
	Strombus lindae	species	82787
species not entered	Strombus	genus	10520
	Strombus alatus	species	82785
obsolete variant of	Strombus	genus	10520
obsolete variant of	Strombus	genus	10520

	Strombus alatus	species	82785
	Strombus alatus	species	82785
	Strombus lindae	species	82787
recombined as	Lobatus costatus	species	120026
	Strombus alatus	species	82785
recombined as	Strombus (Lentigo) ra	species	122144
recombined as	Lobatus gigas	species	109144
recombined as	Lobatus costatus	species	120026
species not entered	Strombus	genus	10520
	Strombus lindae	species	82787
species not entered	Strombus (Macrostroı	subgenus	117504
species not entered	Strombus	genus	10520
species not entered	Strombus (Macrostroı	subgenus	117504
species not entered	Strombus (Macrostroı	subgenus	117504
species not entered	Strombus (Macrostroı	subgenus	117504
recombined as	Lobatus gigas	species	109144
recombined as	Lobatus williamsi	species	146699
species not entered	Strombus	genus	10520
species not entered	Rostellaria (Fusus)	subgenus	11143
species not entered	Strombus	genus	10520
species not entered	Strombus (Macrostroı	subgenus	117504
species not entered	Strombus (Macrostroı	subgenus	117504
species not entered	Strombus	genus	10520
species not entered	Strombus (Macrostroı	subgenus	117504
subjective synonym of	Strombus floridanus	species	187625
misspelling of, obsolete variant of	Strombus pugilis	species	94554
misspelling of, obsolete variant of	Strombus pugilis	species	94554
misspelling of, obsolete variant of	Strombus pugilis	species	94554
species not entered	Strombus (Macrostroı	subgenus	117504
	Strombus floridanus	species	187625
species not entered	Strombus (Eustrombu	subgenus	9842
	Strombus pugilis	species	94554
species not entered	Strombus	genus	10520
	Strombus pugilis	species	94554
	Strombus (Eustrombu	subgenus	9842
	Strombus	genus	10520
species not entered	Strombus (Eustrombu	subgenus	9842
	Strombus (Eustrombu	subgenus	9842
	Strombus alatus	species	82785
	Lobatus costatus	species	120026
	Lobatus gigas	species	109144
species not entered	Lobatus	genus	146670
	Strombus alatus	species	82785
	Strombus floridanus	species	187625
	Strombus (Tricornis)	subgenus	10606
	Strombus pugilis	species	94554
	Fusus minor	species	392380

	Fusus rudis	species	392384
	Fusus bullata	species	392383
	Fusus conus	species	392381
	Fusus filiformis	species	392382
species not entered	Tegula	genus	9297
species not entered	Tegula	genus	9297
species not entered	Tegula	genus	9297
species not entered	Tegula	genus	9297
species not entered	Tegula	genus	9297
species not entered	Agathistoma	genus	8525
species not entered	Tegula	genus	9297
recombined as	Hastula cinerea	species	101225
recombined as	Strioterebrum concav	species	94427
recombined as	Strioterebrum disloca	species	107305
recombined as	Strioterebrum disloca	species	107305
recombined as	Strioterebrum disloca	species	107305
	Terebra	genus	11754
recombined as	Strioterebrum concav	species	94427
recombined as	Strioterebrum disloca	species	107305
species not entered	Terebra	genus	11754
	Terebra	genus	11754
recombined as	Strioterebrum disloca	species	107305
recombined as	Strioterebrum concav	species	94427
recombined as	Strioterebrum disloca	species	107305
	Terebra	genus	11754
	Terebra	genus	11754
recombined as	Strioterebrum disloca	species	107305
obsolete variant of	Terebra	genus	11754
species not entered	Terebra	genus	11754
obsolete variant of	Terebra	genus	11754
subjective synonym of, species not entered	Terebra	genus	11754
subjective synonym of, species not entered	Terebra	genus	11754
subjective synonym of, species not entered	Terebra	genus	11754
	Terebra	genus	11754
recombined as	Strioterebrum concav	species	94427
recombined as	Strioterebrum disloca	species	107305
	Terebra protexta	species	107179
recombined as	Strioterebrum disloca	species	107305
	Terebra	genus	11754
recombined as	Strioterebrum disloca	species	107305
recombined as	Strioterebrum concav	species	94427
	Terebra protexta	species	107179
recombined as	Strioterebrum disloca	species	107305
recombined as	Strioterebrum disloca	species	107305
recombined as	Terebra protexta	species	107179
recombined as	Strioterebrum concav	species	94427
species not entered	Strioterebrum	genus	11711

	Strioterebrum petiti	species	93791
species not entered	Terebra (Myurellina)	subgenus	11396
	Terebra (Myurellina) l	species	93786
recombined as	Terebra aclinica	species	93787
	Strioterebrum brighto	species	93790
	Strioterebrum semino	species	93792
species not entered	Terebra (Myurellina)	subgenus	11396
recombined as	Strioterebrum disloca	species	107305
obsolete variant of	Terebra (Myurellina) l	species	93786
species not entered	Strioterebrum	genus	11711
species not entered	Strioterebrum	genus	11711
recombined as	Strioterebrum disloca	species	107305
	Hastula cinerea	species	101225
recombined as	Strioterebrum concav	species	94427
	Terebra protexta	species	107179
	Hastula	genus	11182
	Strioterebrum	genus	11711
obsolete variant of	Terebra (Myurellina)	subgenus	11396
recombined as	Strioterebrum disloca	species	107305
recombined as	Strioterebrum disloca	species	107305
recombined as	Strioterebrum disloca	species	107305
recombined as	Strioterebrum concav	species	94427
recombined as	Strioterebrum disloca	species	107305
recombined as	Strioterebrum concav	species	94427
recombined as	Strioterebrum disloca	species	107305
recombined as	Strioterebrum disloca	species	107305
recombined as	Strioterebrum disloca	species	107305
	Terebra protexta	species	107179
	Strioterebrum	genus	11711
recombined as	Strioterebrum disloca	species	107305
	Strioterebrum	genus	11711
species not entered	Terebra (Paraterebra)	subgenus	90112
species not entered	Terebra (Paraterebra)	subgenus	90112
species not entered	Terebra (Paraterebra)	subgenus	90112
species not entered	Strioterebrum	genus	11711
species not entered	Strioterebrum	genus	11711
species not entered	Strioterebrum	genus	11711
species not entered	Strioterebrum	genus	11711
species not entered	Strioterebrum	genus	11711
recombined as	Terebra protexta	species	107179
recombined as	Terebra protexta	species	107179
recombined as	Strioterebrum concav	species	94427
recombined as	Strioterebrum concav	species	94427
recombined as	Strioterebrum concav	species	94427
recombined as	Strioterebrum concav	species	94427
recombined as	Strioterebrum disloca	species	107305
recombined as	Strioterebrum disloca	species	107305

recombined as	Strioterebrum disloca	species	107305
recombined as	Strioterebrum disloca	species	107305
recombined as	Strioterebrum disloca	species	107305
recombined as	Strioterebrum disloca	species	107305
recombined as	Strioterebrum disloca	species	107305
	Strioterebrum	genus	11711
species not entered	Strioterebrum	genus	11711
species not entered	Strioterebrum	genus	11711
species not entered	Strioterebrum	genus	11711
species not entered	Strioterebrum	genus	11711
species not entered	Strioterebrum	genus	11711
	Strioterebrum	genus	11711
species not entered	Strioterebrum	genus	11711
	Strioterebrum concav	species	94427
	Strioterebrum	genus	11711
recombined as	Strioterebrum concav	species	94427
recombined as	Strioterebrum disloca	species	107305
	Terebra	genus	11754
recombined as	Strioterebrum concav	species	94427
	Terebra	genus	11754
	Terebra	genus	11754
	Terebra	genus	11754
	Terebra	genus	11754
species not entered	Strioterebrum	genus	11711
	Tonna galea	species	91196
	Malea springi	species	250577
	Malea springi	species	250577
	Malea	genus	10086
	Tonna galea	species	91196
	Malea	genus	10086
species not entered	Teinostoma	genus	10554
recombined as	Pseudorotella cocolito	species	135872
subjective synonym of	Teinostoma umbilicat	species	135869
species not entered	Teinostoma	genus	10554
species not entered	Teinostoma	genus	10554
	Teinostoma altum	species	135876
species not entered	Teinostoma	genus	10554
species not entered	Teinostoma	genus	10554
species not entered	Teinostoma	genus	10554
species not entered	Teinostoma (Pseudoro	subgenus	10356
subjective synonym of	Teinostoma biscayner	species	135864
	Teinostoma parvicallu	species	135867
recombined as	Pseudorotella carinica	species	135873
	Teinostoma goniogyru	species	135865
species not entered	Teinostoma	genus	10554
species not entered	Teinostoma	genus	10554
	Cochliolepis holmesi	species	109663

	Cochliolepis striata	species	97698
	Teinostoma	genus	10554
species not entered	Teinostoma	genus	10554
recombined as	Teinostoma altum	species	135876
recombined as	Teinostoma avunculus	species	157377
recombined as	Teinostoma goniogyru	species	135865
species not entered	Teinostoma (Pseudoro	subgenus	10356
species not entered	Teinostoma (Idioraphi	subgenus	9980
	Cochliolepis striata	species	97698
species not entered	Cochliolepis	genus	97697
	Cochliolepis holmesi	species	109663
	Teinostoma	genus	10554
subjective synonym of	Cochliolepis parasitica	species	156620
recombined as	Cochliolepis holmesi	species	109663
subjective synonym of	Cochliolepis parasitica	species	156620
subjective synonym of	Cochliolepis parasitica	species	156620
species not entered	Teinostoma	genus	10554
	Teinostoma beauforte	species	82744
species not entered	Teinostoma	genus	10554
	Macromphalina hanse	species	82746
	Teinostoma beauforte	species	82744
species not entered	Teinostoma	genus	10554
species not entered	Teinostoma	genus	10554
obsolete variant of	Teinostoma (Idiorapho	species	116529
species not entered	Macromphalina	genus	82745
	Macromphalina	genus	82745
recombined as	Cochliolepis holmesi	species	109663
species not entered	Teinostoma	genus	10554
	Cochliolepis	genus	97697
subjective synonym of	Teinostoma umbilicat	species	135869
subjective synonym of	Triphora hemphilli	species	135786
	Triphora	genus	10609
	Triphora	genus	10609
	Triphora	genus	10609
subjective synonym of	Triphora hemphilli	species	135786
species not entered	Triphora	genus	10609
	Marshallora nigrocinc	species	108699
	Triforis	genus	10608
	Triphora	genus	10609
	Triphora	genus	10609
recombined as	Hespererato maugeria	species	91240
subjective synonym of	Niveria suffusa	species	120070
recombined as	Hespererato maugeria	species	91240
recombined as	Pusula pediculus	species	109039
recombined as	Hespererato maugeria	species	91240
recombined as	Hespererato maugeria	species	91240
recombined as	Pusula pediculus	species	109039

recombined as	Niveria quadripunctat	species	135318
recombined as	Pusula pediculus	species	109039
recombined as	Niveria quadripunctat	species	135318
subjective synonym of	Pusula pediculus	species	109039
	Pusula lindajoyceae	species	342727
	Pusula dadeensis	species	343063
	Pusula (Pusula) crovoa	species	93962
subjective synonym of	Pusula pediculus	species	109039
subjective synonym of	Pusula pediculus	species	109039
	Trivia islahispaniolae	species	71691
recombined as	Niveria suffusa	species	120070
subjective synonym of	Niveria suffusa	species	120070
recombined as	Pusula pediculus	species	109039
recombined as	Hespererato maugeria	species	91240
recombined as	Pusula miccosukee	species	343042
	Hespererato maugeria	species	91240
misspelling of, recombined as	Niveria suffusa	species	120070
	Trivia	genus	10611
	Pusula	genus	10388
recombined as	Pusula pediculus	species	109039
	Trivia	genus	10611
subjective synonym of	Niveria suffusa	species	120070
recombined as	Pusula pediculus	species	109039
species not entered	Calliostoma	genus	8628
species not entered	Calliostoma	genus	8628
	Calliostoma	genus	8628
recombined as	Calliostoma (Elmerlini	species	187404
species not entered	Calliostoma	genus	8628
	Calliostoma	genus	8628
	Calliostoma philanthro	species	97672
species not entered	Chlorostoma (Omphal	subgenus	9041
species not entered	Chlorostoma (Omphal	subgenus	9041
	Calliostoma	genus	8628
	Calliostoma	genus	8628
	Calliostoma	genus	8628
recombined as	Calliostoma (Elmerlini	species	187404
species not entered	Calliostoma	genus	8628
species not entered	Calliostoma	genus	8628
species not entered	Chlorostoma (Omphal	subgenus	9041
species not entered	Calliostoma	genus	8628
species not entered	Calliostoma (Leiotroch	subgenus	8913
	Calliostoma	genus	8628
species not entered	Calliostoma	genus	8628
species not entered	Calliostoma	genus	8628
species not entered	Calliostoma	genus	8628
	Calliostoma	genus	8628
species not entered	Calliostoma	genus	8628

species not entered	Calliostoma	genus	8628
species not entered	Calliostoma	genus	8628
species not entered	Calliostoma	genus	8628
species not entered	Calliostoma	genus	8628
species not entered	Calliostoma	genus	8628
species not entered	Calliostoma	genus	8628
	Calliostoma	genus	8628
species not entered	Chlorostoma (Omphal	subgenus	9041
recombined as	Calliostoma (Elmerlini	species	187404
species not entered	Calliostoma	genus	8628
	Infundibulum depress	species	157291
species not entered	Trochus	genus	9336
species not entered	Calliostoma	genus	8628
species not entered	Calliostoma	genus	8628
species not entered	Calliostoma	genus	8628
	Calliostoma	genus	8628
	Calliostoma virginicun	species	97675
	Calliostoma virginicun	species	97675
	Calliostoma philanthr(	species	97672
	Calliostoma	genus	8628
	Calliostoma	genus	8628
	Calliostoma	genus	8628
	Calliostoma	genus	8628
	Calliostoma	genus	8628
species not entered	Calliostoma	genus	8628
	Calliostoma	genus	8628
	Calliostoma virginicun	species	97675
recombined as	Vasum (Hystrivasum)	species	120369
	Xancus regina	species	81571
recombined as	Turbinella scolymoide	species	120684
recombined as	Turbinella scolymoide	species	120684
	Vasum	genus	11832
	Turbinella wheeleri	species	82799
species not entered	Vasum	genus	11832
recombined as	Vasum (Hystrivasum)	species	120369
	Xancus regina	species	81571
recombined as	Vasum (Hystrivasum)	species	120727
species not entered	Fulgurofusus	genus	11132
species not entered	Vasum	genus	11832
species not entered	Fulgurofusus	genus	11132
species not entered	Turbinella	genus	11806
species not entered	Vasum	genus	11832
species not entered	Vasum	genus	11832
species not entered	Turbinella	genus	11806
species not entered	Vasum (Hystrivasum)	subgenus	120725
species not entered	Turbinella	genus	11806
recombined as	Vasum (Hystrivasum)	species	120369

recombined as	Xancus regina	species	81571
	Turbinella scolymoide	species	120684
recombined as	Vasum (Hystrivasum)	species	120369
recombined as	Vasum (Hystrivasum)	species	120369
species not entered	Vasum (Hystrivasum)	subgenus	120725
species not entered	Vasum (Hystrivasum)	subgenus	120725
species not entered	Vasum (Hystrivasum)	subgenus	120725
species not entered	Vasum (Hystrivasum)	subgenus	120725
obsolete variant of	Vasum (Hystrivasum)	species	120726
species not entered	Vasum (Hystrivasum)	subgenus	120725
species not entered	Vasum (Hystrivasum)	subgenus	120725
species not entered	Vasum (Hystrivasum)	subgenus	120725
species not entered	Vasum (Hystrivasum)	subgenus	120725
species not entered	Vasum	genus	11832
species not entered	Vasum (Hystrivasum)	subgenus	120725
species not entered	Vasum (Hystrivasum)	subgenus	120725
species not entered	Vasum (Hystrivasum)	subgenus	120725
species not entered	Vasum (Hystrivasum)	subgenus	120725
species not entered	Turbinella	genus	11806
recombined as	Vasum (Hystrivasum)	species	120727
species not entered	Vasum (Hystrivasum)	subgenus	120725
species not entered	Vasum (Hystrivasum)	subgenus	120725
obsolete variant of	Vasum (Hystrivasum)	species	120728
	Xancus regina	species	81571
recombined as	Vasum (Hystrivasum)	species	120369
	Turbinella scolymoide	species	120684
species not entered	Turbinella	genus	11806
species not entered	Vasum (Hystrivasum)	subgenus	120725
obsolete variant of	Vasum (Hystrivasum)	subgenus	120725
species not entered	Vasum (Hystrivasum)	subgenus	120725
recombined as	Vasum (Hystrivasum)	species	120727
species not entered	Vasum (Hystrivasum)	subgenus	120725
obsolete variant of	Vasum (Hystrivasum)	species	120728
	Turbinella wheeleri	species	82799
species not entered	Vasum	genus	11832
obsolete variant of	Vasum (Hystrivasum)	subgenus	120725
species not entered	Fulgurofusus	genus	11132
species not entered	Gelasinostoma	genus	8827
misspelling of, recombined as	Turbo (Senectus) casta	species	119763
species not entered	Turbo	genus	9347
misspelling of, recombined as	Turbo (Senectus) casta	species	119763
species not entered	Astraea	genus	8573
species not entered	Turbo	genus	9347
misspelling of, recombined as	Turbo (Senectus) casta	species	119763
misspelling of, recombined as	Turbo (Senectus) casta	species	119763
recombined as	Turbo (Senectus) casta	species	119763
recombined as	Turbo (Senectus) casta	species	119763

misspelling of, recombined as	Turbo (Senectus) casta	species	119763
recombined as	Astralium phoebium	species	149122
recombined as	Lithopoma americanu	species	109175
subjective synonym of	Lithopoma americanu	species	109175
species not entered	Turbo (Marmarostom	subgenus	8962
species not entered	Lithopoma	genus	8936
recombined as	Turbo (Senectus) casta	species	119763
species not entered	Turbo	genus	9347
species not entered	Lithopoma	genus	8936
species not entered	Turbo	genus	9347
species not entered	Turbo (Marmarostom	subgenus	8962
species not entered	Lithopoma	genus	8936
	Turbo	genus	9347
	Turbo (Taeniaturbo)	subgenus	107067
species not entered	Turbo (Taeniaturbo)	subgenus	107067
species not entered	Turbo (Taeniaturbo)	subgenus	107067
species not entered	Turbo (Marmarostom	subgenus	8962
species not entered	Lithopoma	genus	8936
species not entered	Gelasinostoma	genus	8827
species not entered	Turbo (Taeniaturbo)	subgenus	107067
species not entered	Turbo (Marmarostom	subgenus	8962
species not entered	Astralium	genus	8575
species not entered	Lithopoma	genus	8936
	Astralium phoebium	species	149122
recombined as	Turbo (Senectus) casta	species	119763
species not entered	Astraea	genus	8573
subjective synonym of	Turbo (Senectus) casta	species	119763
species not entered	Turbo	genus	9347
subjective synonym of	Turbo (Senectus) casta	species	119763
obsolete variant of	Turbo	genus	9347
	Angaria crassa	species	392479
	Angaria crassa	species	392479
	Angaria crassa	species	392479
	Angaria crassa	species	392479
recombined as	Turbo (Senectus) casta	species	119763
	Polystira albida	species	91340
	Polystira albida	species	91340
	Sedilia aphanitoma	species	120706
species not entered	Sedilia	genus	82943
species not entered	Sedilia	genus	82943
species not entered	Sedilia	genus	82943
species not entered	Sedilia	genus	82943
	Sedilia	genus	82943
obsolete variant of	Sedilia	genus	82943
recombined as	Lepicythara turrita	species	161162
	Turridae	family	62680
	Pleuroliria	genus	11536

	Turridae	family	62680
	Turridae	family	62680
	Polystira albida	species	91340
species not entered	Fenimorea	genus	120673
species not entered	Fenimorea	genus	120673
	Fenimorea pagodula	species	120672
species not entered	Sedilia	genus	82943
species not entered	Sedilia	genus	82943
species not entered	Sedilia	genus	82943
species not entered	Glabrocythara	genus	82945
species not entered	Sedilia	genus	82943
species not entered	Sedilia	genus	82943
	Sedilia	genus	82943
	Turridae	family	62680
species not entered	Fenimorea	genus	120673
	Turridae	family	62680
	Turridae	family	62680
	Turridae	family	62680
	Turridae	family	62680
	Turridae	family	62680
	Turridae	family	62680
	Turridae	family	62680
	Turritella	genus	10637
species not entered	Vermicularia	genus	10661
recombined as	Turritella (Bactrospira	species	107007
species not entered	Turritella	genus	10637
species not entered	Turritella	genus	10637
species not entered	Turritella	genus	10637
recombined as	Turritella (Bactrospira	species	107007
species not entered	Turritella	genus	10637
	Vermicularia	genus	10661
species not entered	Turritella	genus	10637
species not entered	Turritella	genus	10637
recombined as	Turritella (Bactrospira	species	107007
species not entered	Turritella	genus	10637
recombined as	Turritella (Bactrospira	species	107007
species not entered	Turritella	genus	10637
species not entered	Turritella	genus	10637
species not entered	Turritella	genus	10637
species not entered	Vermicularia	genus	10661
	Vermicularia	genus	10661
species not entered	Turritella	genus	10637
	Vermicularia spirata	species	109777
species not entered	Turritella	genus	10637
species not entered	Vermicularia	genus	10661
species not entered	Turritella	genus	10637
species not entered	Turritella	genus	10637

species not entered	Turritella	genus	10637
obsolete variant of	Turritella	genus	10637
species not entered	Turritella	genus	10637
species not entered	Turritella	genus	10637
species not entered	Vermicularia	genus	10661
	Vermicularia spirata	species	109777
	Vermicularia spirata	species	109777
species not entered	Turritella	genus	10637
species not entered	Turritella	genus	10637
species not entered	Turritella	genus	10637
species not entered	Turritella	genus	10637
species not entered	Turritella	genus	10637
species not entered	Turritella	genus	10637
species not entered	Turritella	genus	10637
recombined as	Turritella (Torculoidell	species	95601
recombined as	Turritella (Torculoidell	species	95601
recombined as	Turritella (Torculoidell	species	95601
species not entered	Turritella	genus	10637
	Turritella	genus	10637
species not entered	Turritella	genus	10637
species not entered	Vermicularia	genus	10661
	Turritella	genus	10637
species not entered	Turritella	genus	10637
species not entered	Vermicularia	genus	10661
species not entered	Turritella	genus	10637
	Turritella buckingham	species	89172
	Turritella buckingham	species	89172
recombined as	Turritella (Broderiptel	subspecies	144976
	Turritella buckingham	species	89172
recombined as	Turritella (Broderiptel	subspecies	144976
	Turritella buckingham	species	89172
recombined as	Turritella (Broderiptel	subspecies	144976
species not entered	Turritella	genus	10637
	Turritella buckingham	species	89172
recombined as	Turritella (Broderiptel	subspecies	144976
	Vermicularia spirata	species	109777
species not entered	Turritella	genus	10637
recombined as	Turritella (Torcula) vai	species	97639
species not entered	Turritella	genus	10637
	Turritella	genus	10637
	Turritella	genus	10637
recombined as	Turritella (Torcula) vai	species	97639
species not entered	Turritella	genus	10637
	Turritella	genus	10637
recombined as	Turritella (Bactrospira	species	107007
species not entered	Turritella	genus	10637
species not entered	Turritella	genus	10637

species not entered	Turritella	genus	10637
species not entered	Turritella	genus	10637
species not entered	Turritella	genus	10637
species not entered	Turritella	genus	10637
species not entered	Turritella	genus	10637
species not entered	Turritella	genus	10637
	Turritella (Broderiptel	species	93964
	Turritella (Broderiptel	species	93964
species not entered	Turritella	genus	10637
species not entered	Turritella	genus	10637
species not entered	Turritella	genus	10637
species not entered	Turritella	genus	10637
recombined as	Turritella (Bactrospira	species	107007
recombined as	Turritella (Bactrospira	species	107007
	Vermicularia	genus	10661
species not entered	Turritella	genus	10637
	Turritella	genus	10637
species not entered	Turritella (Eichwaldiel	subgenus	9796
species not entered	Torcula	genus	10588
species not entered	Turritella (Eichwaldiel	subgenus	9796
species not entered	Turritella (Eichwaldiel	subgenus	9796
species not entered	Vermicularia	genus	10661
obsolete variant of	Torcula	genus	10588
	Turritella	genus	10637
species not entered	Turritella	genus	10637
species not entered	Turritella (Eichwaldiel	subgenus	9796
	Turritella (Bactrospira	species	107007
species not entered	Vermicularia	genus	10661
species not entered	Torcula	genus	10588
	Turritella (Bactrospira	species	107007
species not entered	Torcula	genus	10588
species not entered	Torcula	genus	10588
species not entered	Turritella	genus	10637
	Vermicularia spirata	species	109777
species not entered	Torcula	genus	10588
	Vermicularia spirata	species	109777
	Turritella	genus	10637
	Turritella	genus	10637
species not entered	Torcula	genus	10588
	Vermicularia spirata	species	109777
	Turritella (Bactrospira	species	107007
species not entered	Torcula	genus	10588
	Vermicularia spirata	species	109777
species not entered	Vermicularia	genus	10661
species not entered	Vermicularia	genus	10661
species not entered	Vermicularia	genus	10661
species not entered	Vermicularia	genus	10661

species not entered	Torcula	genus	10588
species not entered	Torcula	genus	10588
	Vermicularia woodring	species	119771
species not entered	Turritella	genus	10637
species not entered	Torcula	genus	10588
species not entered	Torcula	genus	10588
species not entered	Torcula	genus	10588
	Turritella (Bactrospira	species	107007
species not entered	Turritella	genus	10637
species not entered	Turritella	genus	10637
	Vermicularia	genus	10661
recombined as	Turritella (Bactrospira	species	107007
species not entered	Turritella	genus	10637
species not entered	Turritella	genus	10637
species not entered	Turritella	genus	10637
species not entered	Turritella	genus	10637
	Torcula acropora	species	96455
species not entered	Turritella	genus	10637
	Vermicularia spirata	species	109777
	Turritella	genus	10637
species not entered	Turritella (Eichwaldiel	subgenus	9796
obsolete variant of	Turritella (Broderiptel	species	93964
	Turritella buckingham	species	89172
recombined as	Turritella (Broderiptel	species	96466
	Turritella	genus	10637
	Vermicularia spirata	species	109777
species not entered	Turritella	genus	10637
species not entered	Turritella	genus	10637
species not entered	Turritella	genus	10637
species not entered	Turritella	genus	10637
species not entered	Turritella	genus	10637
species not entered	Turritella	genus	10637
species not entered	Vermicularia	genus	10661
	Turritella	genus	10637
	Turritellidae	family	55363
misspelling of, recombined as	Turritella (Turritella) a	species	116540
	Vermicularia spirata	species	109777
	Turritella	genus	10637
recombined as	Turritella (Torcula) var	species	97639
species not entered	Turritella	genus	10637
species not entered	Turritella	genus	10637
	Vermicularia spirata	species	109777
	Vermicularia spirata	species	109777
species not entered	Turritella	genus	10637
	Turritella beaufortens	species	82748
	Turritella beaufortens	species	82748
species not entered	Turritella	genus	10637

	Turritella beaufortens	species	82748
species not entered	Turritella	genus	10637
	Turritella (Torculoidell	species	95601
	Turritella (Torculoidell	species	95601
	Vermicularia spirata	species	109777
	Vermicularia spirata	species	109777
	Vermicularia spirata	species	109777
species not entered	Turritella	genus	10637
	Vermicularia knorrii	species	187602
	Vermicularia spirata	species	109777
species not entered	Turritella	genus	10637
	Turritella	genus	10637
	Turritella	genus	10637
recombined as	Turritella (Torculoidell	species	95601
recombined as	Turritella (Torculoidell	species	95601
recombined as	Turritella (Torculoidell	species	95601
recombined as	Turritella (Torculoidell	species	95601
species not entered	Turritella	genus	10637
	Torcula	genus	10588
	Turritella	genus	10637
	Turritella	genus	10637
	Vermicularia knorrii	species	187602
	Turritella pilsbryi	species	95597
recombined as	Turritella (Torcula) var	species	97639
recombined as	Turritella (Turritella) a	species	116540
recombined as	Turritella (Turritella) a	species	116540
	Turritella pilsbryi	species	95597
	Turritella pilsbryi	species	95597
recombined as	Turritella (Turritella) a	species	116540
recombined as	Turritella (Turritella) a	species	116540
recombined as	Turritella (Turritella) a	species	116540
recombined as	Turritella (Turritella) a	species	116540
recombined as	Turritella (Turritella) a	species	116540
recombined as	Turritella (Turritella) a	species	116540
recombined as	Turritella (Turritella) a	species	116540
recombined as	Turritella (Turritella) a	species	116540
recombined as	Turritella (Turritella) a	species	116540
recombined as	Turritella (Turritella) a	species	116540
recombined as	Turritella (Turritella) a	species	116540
	Turritella pilsbryi	species	95597
	Turritella pilsbryi	species	95597
recombined as	Turritella (Turritella) a	species	116540
	Turritella (Turritella) a	species	116540
recombined as	Mariacolpus plebeia	species	95598
species not entered	Turritella	genus	10637
recombined as	Mariacolpus plebeia	species	95598
recombined as	Mariacolpus plebeia	species	95598

species not entered	Vallonia	genus	114478
	Valvata	genus	82075
recombined as	Dendropoma irregular	species	109121
	Petaloconchus florida	species	89539
	Lemintina	genus	10042
recombined as	Serpulorbis decussatu	species	135105
	Petaloconchus florida	species	89539
	Lemintina	genus	10042
	Petaloconchus sculptu	species	89540
	Serpulorbis granifera	species	82749
	Petaloconchus sculptu	species	89540
	Petaloconchus sculptu	species	89540
	Petaloconchus sculptu	species	89540
	Vermetidae	family	62787
species not entered	Petaloconchus (Petalc	subgenus	445956
	Petaloconchus sculptu	species	89540
species not entered	Petaloconchus (Petalc	subgenus	445956
	Petaloconchus sculptu	species	89540
species not entered	Lemintina	genus	10042
species not entered	Lemintina	genus	10042
	Petaloconchus florida	species	89539
recombined as	Serpulorbis decussatu	species	135105
	Petaloconchus sculptu	species	89540
	Petaloconchus florida	species	89539
	Petaloconchus sculptu	species	89540
species not entered	Serpulorbis	genus	10457
species not entered	Petaloconchus	genus	26115
species not entered	Petaloconchus (Petalc	subgenus	445956
	Serpulorbis granifera	species	82749
species not entered	Serpulorbis	genus	10457
	Serpulorbis granifera	species	82749
species not entered	Lemintina	genus	10042
species not entered	Lemintina	genus	10042
species not entered	Lemintina	genus	10042
	Serpulorbis granifera	species	82749
	Serpulorbis granifera	species	82749
species not entered	Petaloconchus	genus	26115
species not entered	Lemintina	genus	10042
species not entered	Petaloconchus	genus	26115
	Serpulorbis granifera	species	82749
	Serpulorbis granifera	species	82749
species not entered	Serpulorbis	genus	10457
	Serpulorbis granifera	species	82749
	Serpulorbis granifera	species	82749
	Serpulorbis granifera	species	82749
species not entered	Serpulorbis	genus	10457
species not entered	Petaloconchus (Petalc	subgenus	445956

species not entered	Petaloconchus	genus	26115
recombined as	Cyclostremiscus barts	species	248855
species not entered	Circulus	genus	8665
recombined as	Cyclostremiscus (Ponc	species	115220
subjective synonym of	Episcynia inornata	species	91238
	Vitrinella	genus	10670
subjective synonym of	Episcynia inornata	species	91238
recombined as	Cyclostremiscus (Ponc	species	115220
subjective synonym of	Episcynia inornata	species	91238
	Vitrinella	genus	10670
subjective synonym of	Episcynia inornata	species	91238
	Cyclostremiscus beaui	species	156723
species not entered	Cyclostremiscus	genus	9715
	Cyclostremiscus	genus	9715
species not entered	Anticlimax	genus	9483
species not entered	Parviturboides	genus	117497
species not entered	Anticlimax	genus	9483
species not entered	Anticlimax	genus	9483
	Cyclostremiscus barts	species	248855
species not entered	Cyclostremiscus	genus	9715
species not entered	Cyclostremiscus (Ponc	subgenus	10295
species not entered	Parviturboides	genus	117497
species not entered	Anticlimax	genus	9483
species not entered	Anticlimax	genus	9483
species not entered	Anticlimax	genus	9483
species not entered	Cyclostremiscus	genus	9715
	Cyclostremiscus barts	species	248855
species not entered	Cyclostremiscus (Ponc	subgenus	10295
species not entered	Cyclostremiscus	genus	9715
subjective synonym of	Episcynia inornata	species	91238
species not entered	Parviturboides	genus	117497
species not entered	Cyclostremiscus (Ponc	subgenus	10295
species not entered	Anticlimax	genus	9483
recombined as	Cyclostremiscus (Ponc	species	115220
species not entered	Vitrinella	genus	10670
	Episcynia inornata	species	91238
subjective synonym of	Episcynia inornata	species	91238
species not entered	Cyclostremiscus	genus	9715
species not entered	Cyclostremiscus	genus	9715
species not entered	Circulus	genus	8665
species not entered	Circulus	genus	8665
species not entered	Cyclostremiscus	genus	9715
	Circulus liratus	species	90932
	Adeorbis	genus	9430
	Adeorbis	genus	9430
	Vitrinella	genus	10670
species not entered	Cyclostremiscus	genus	9715

species not entered	Viviparus	genus	68006
species not entered	Viviparus	genus	68006
species not entered	Viviparus	genus	68006
species not entered	Viviparus	genus	68006
species not entered	Viviparus	genus	68006
species not entered	Viviparus	genus	68006
species not entered	Viviparus	genus	68006
species not entered	Viviparus	genus	68006
species not entered	Viviparus	genus	68006
species not entered	Viviparus	genus	68006
species not entered	Viviparus	genus	68006
species not entered	Viviparus	genus	68006
species not entered	Viviparus	genus	68006
species not entered	Viviparus	genus	68006
species not entered	Viviparus	genus	68006
species not entered	Viviparus	genus	68006
species not entered	Viviparus	genus	68006
species not entered	Viviparus	genus	68006
species not entered	Viviparus	genus	68006
species not entered	Viviparus	genus	68006
species not entered	Viviparus	genus	68006
	Scaphella floridana	species	120683
subjective synonym of	Bullata taylori	species	93843
subjective synonym of	Bullata taylori	species	93843
recombined as	Volutifusus mutabilis	species	93829
subjective synonym of	Bullata taylori	species	93843
	Scaphella floridana	species	120683
species not entered	Scaphella	genus	11654
species not entered	Volutifusus	genus	82940
subjective synonym of	Scaphella junonia	species	86312
species not entered	Scaphella	genus	11654
species not entered	Scaphella	genus	11654
	Aurinia (Volutifusus) c	species	93832
	Aurinia (Volutifusus) c	species	93832
species not entered	Scaphella	genus	11654
species not entered	Scaphella	genus	11654
species not entered	Scaphella	genus	11654
	Scaphella floridana	species	120683
species not entered	Scaphella	genus	11654
species not entered	Scaphella	genus	11654
species not entered	Scaphella	genus	11654
species not entered	Closia (Bullata)	subgenus	10870
species not entered	Volutifusus	genus	82940
species not entered	Volutifusus	genus	82940
species not entered	Volutifusus	genus	82940
species not entered	Scaphella	genus	11654
	Scaphella floridana	species	120683

recombined as	Volutifusus mutabilis	species	93829
	Scaphella	genus	11654
species not entered	Voluta	genus	11845
species not entered	Volutifusus	genus	82940
	Scaphella trenholmii	species	93820
species not entered	Scaphella (Scaphella)	subgenus	86316
recombined as	Aurinia (Volutifusus) c	species	93832
recombined as	Aurinia (Volutifusus) c	species	93832
recombined as	Aurinia (Volutifusus) c	species	93832
species not entered	Closia (Bullata)	subgenus	10870
	Scaphella	genus	11654
recombined as	Aurinia (Volutifusus) c	species	93832
	Scaphella	genus	11654
	Scaphella	genus	11654
	Scaphella	genus	11654
	Xenophora conchyliop	species	81730
	Xenophora delecta	species	187629
	Xenophora delecta	species	187629
	Xenophora conchyliop	species	81730
	Xenophora conchyliop	species	81730
	Xenophora conchyliop	species	81730
species not entered	Glyphyalinia	genus	251920
species not entered	Hawaiia	genus	162067
species not entered	Nesovitrea	genus	251921
species not entered	Zonitoides	genus	251922
species not entered	Glyphyalinia	genus	251920
species not entered	Hawaiia	genus	162067
species not entered	Glyphyalinia	genus	251920
species not entered	Hawaiia	genus	162067
	Gastropoda	class	8304
obsolete variant of	Gastropoda	class	8304
obsolete variant of	Gastropoda	class	8304
	Gastropoda	class	8304
	Gastropoda	class	8304
	Gastropoda	class	8304
	Gastropoda	class	8304
	Gastropoda	class	8304
	Gastropoda	class	8304
	Gastropoda	class	8304
species not entered	Gastropoda	class	8304

enus,pres,loc,paleoloc,lithext,refattr

early_interval	late_interval	max_ma	min_ma	ref_author	ref_pubyr	reference_no	phylum	phylum_no	class
Middle Pleistocene		0.781	0.126	Portell et a	1992	17634	Mollusca	7805	Bivalvia
Late Pleistocene		0.126	0.0117	Portell et a	1992	17634	Mollusca	7805	Bivalvia
Early Pleistocene		2.588	0.781	Portell et a	1992	17634	Mollusca	7805	Bivalvia
Middle Pleistocene		0.781	0.126	Hoerle	1970	19536	Mollusca	7805	Bivalvia
Early Pleistocene		2.588	0.781	Olsson anc	1953	25463	Mollusca	7805	Bivalvia
Early Pleistocene		2.588	0.781	Hendy et a	2008	19909	Mollusca	7805	Bivalvia
Late Pleistocene		0.126	0.0117	Kittle and I	2010	40884	Mollusca	7805	Bivalvia
Late Pleistocene		0.126	0.0117	Richards	1962	18974	Mollusca	7805	Bivalvia
Late Pleistocene		0.126	0.0117	Richards	1962	18974	Mollusca	7805	Bivalvia
Late Pleistocene		0.126	0.0117	Richards	1962	18974	Mollusca	7805	Bivalvia
Late Pleistocene		0.126	0.0117	Hendy et a	2008	19909	Mollusca	7805	Bivalvia
Late Pleistocene		0.126	0.0117	Hendy et a	2008	19909	Mollusca	7805	Bivalvia
Pleistocene		2.588	0.0117	Hendy et a	2008	19909	Mollusca	7805	Bivalvia
Late Pliocene		3.6	2.588	Ward and I	1987	16700	Mollusca	7805	Bivalvia
Late Pliocene		3.6	2.588	Ward and I	1987	16700	Mollusca	7805	Bivalvia
Early Pleistocene		2.588	0.781	Ward and I	1987	16700	Mollusca	7805	Bivalvia
Late Pleistocene		0.126	0.0117	Richards	1962	18974	Mollusca	7805	Bivalvia
Late Pleistocene		0.126	0.0117	Hendy et a	2008	19909	Mollusca	7805	Bivalvia
Late Pliocene		3.6	2.588	Hendy et a	2008	19909	Mollusca	7805	Bivalvia
Early Pleistocene		2.588	0.781	Hendy et a	2008	19909	Mollusca	7805	Bivalvia
Late Pleistocene		0.126	0.0117	Spencer ar	1987	15525	Mollusca	7805	Bivalvia
Late Pleistocene		0.126	0.0117	Spencer ar	1987	15525	Mollusca	7805	Bivalvia
Late Pliocene		3.6	2.588	Hendy et a	2008	19909	Mollusca	7805	Bivalvia
Late Pleistocene		0.126	0.0117	Sellards	1917	9011	Mollusca	7805	Bivalvia

Early Pleistocene		2.588	0.781	Mansfield	1932	11083	Mollusca	7805 Bivalvia
Early Pleistocene		2.588	0.781	Mansfield	1932	11083	Mollusca	7805 Bivalvia
Early Pliocene		5.333	3.6	DuBar	1962	11119	Mollusca	7805 Bivalvia
Early Pleistocene		2.588	0.781	DuBar	1962	11119	Mollusca	7805 Bivalvia
Early Pleistocene		2.588	0.781	DuBar	1962	11119	Mollusca	7805 Bivalvia
Early Pleistocene		2.588	0.781	DuBar	1962	11119	Mollusca	7805 Bivalvia
Calabrian		1.806	0.781	DuBar	1962	11119	Mollusca	7805 Bivalvia
Early Pleistocene		2.588	0.781	DuBar	1962	11119	Mollusca	7805 Bivalvia
Pliocene	Pleistocen	5.333	0.0117	DuBar	1962	11119	Mollusca	7805 Bivalvia
Late Pliocene		3.6	2.588	Mansfield	1932	17094	Mollusca	7805 Bivalvia
Late Pliocene		3.6	2.588	Mansfield	1932	17094	Mollusca	7805 Bivalvia
Late Pliocene		3.6	2.588	Mansfield	1932	17094	Mollusca	7805 Bivalvia
Late Pliocene		3.6	2.588	Mansfield	1932	17094	Mollusca	7805 Bivalvia
Late Pliocene		3.6	2.588	Mansfield	1932	17094	Mollusca	7805 Bivalvia
Late Pliocene		3.6	2.588	Mansfield	1932	17094	Mollusca	7805 Bivalvia
Late Pliocene		3.6	2.588	Mansfield	1932	17094	Mollusca	7805 Bivalvia
Early Pleistocene		2.588	0.781	Portell et a	1992	17634	Mollusca	7805 Bivalvia
Middle Pleistocene		0.781	0.126	Portell et a	1992	17634	Mollusca	7805 Bivalvia
Late Pleistocene		0.126	0.0117	Portell et a	1992	17634	Mollusca	7805 Bivalvia
Middle Pleistocene		0.781	0.126	McGinty	1970	19114	Mollusca	7805 Bivalvia
Late Pliocene		3.6	2.588	Mansfield	1939	19112	Mollusca	7805 Bivalvia
Late Pliocene		3.6	2.588	Mansfield	1939	19112	Mollusca	7805 Bivalvia
Late Pliocene		3.6	2.588	Mansfield	1939	19112	Mollusca	7805 Bivalvia
Late Pleistocene		0.126	0.0117	Hendy et a	2008	19909	Mollusca	7805 Bivalvia
Early Pleistocene		2.588	0.781	Schroeder	1954	25303	Mollusca	7805 Bivalvia
Early Pleistocene		2.588	0.781	Schroeder	1954	25303	Mollusca	7805 Bivalvia
Early Pleistocene		2.588	0.781	Schroeder	1954	25303	Mollusca	7805 Bivalvia
Early Pleistocene		2.588	0.781	Olsson anc	1953	25463	Mollusca	7805 Bivalvia
Late Pliocene		3.6	2.588	Olsson	1967	19826	Mollusca	7805 Bivalvia
Early Pleistocene		2.588	0.781	Olsson	1967	19826	Mollusca	7805 Bivalvia
Early Pleistocene		2.588	0.781	Olsson	1967	19826	Mollusca	7805 Bivalvia
Early Pliocene		5.333	3.6	Olsson anc	1964	26836	Mollusca	7805 Bivalvia
Late Pliocene		3.6	2.588	DuBar	1958	25514	Mollusca	7805 Bivalvia
Late Pliocene		3.6	2.588	DuBar	1958	25514	Mollusca	7805 Bivalvia
Early Pleistocene		2.588	0.781	Hendy et a	2008	19909	Mollusca	7805 Bivalvia
Early Pleistocene		2.588	0.781	Hendy et a	2008	19909	Mollusca	7805 Bivalvia
Late Pleistocene		0.126	0.0117	Hendy et a	2008	19909	Mollusca	7805 Bivalvia
Early Pliocene		5.333	3.6	Petuch anc	2007	26813	Mollusca	7805 Bivalvia
Late Pleistocene		0.126	0.0117	Hendy et a	2008	19909	Mollusca	7805 Bivalvia
Early Pleistocene		2.588	0.781	Hendy et a	2008	19909	Mollusca	7805 Bivalvia
Late Pliocene		3.6	2.588	Hendy et a	2008	19909	Mollusca	7805 Bivalvia
Late Pliocene		3.6	2.588	Hendy et a	2008	19909	Mollusca	7805 Bivalvia
Late Pliocene		3.6	2.588	Hendy et a	2008	19909	Mollusca	7805 Bivalvia
Middle Pleistocene		0.781	0.126	Hendy et a	2008	19909	Mollusca	7805 Bivalvia
Late Pleistocene		0.126	0.0117	Hendy et a	2008	19909	Mollusca	7805 Bivalvia
Early Pleistocene		2.588	0.781	Hendy et a	2008	19909	Mollusca	7805 Bivalvia
Early Pleistocene		2.588	0.781	Hendy et a	2008	19909	Mollusca	7805 Bivalvia

Late Pleistocene	0.126	0.0117	Kittle and I	2010	40884	Mollusca	7805	Bivalvia
Late Pleistocene	0.126	0.0117	Hulbert an	1998	1962	Mollusca	7805	Bivalvia
Late Pleistocene	0.126	0.0117	Hulbert an	1998	1962	Mollusca	7805	Bivalvia
Late Pleistocene	0.126	0.0117	DuBar	1971	17556	Mollusca	7805	Bivalvia
Late Pleistocene	0.126	0.0117	DuBar	1971	17556	Mollusca	7805	Bivalvia
Late Pleistocene	0.126	0.0117	DuBar	1971	17556	Mollusca	7805	Bivalvia
Late Pleistocene	0.126	0.0117	DuBar	1971	17556	Mollusca	7805	Bivalvia
Pliocene	5.333	2.588	DuBar	1971	17556	Mollusca	7805	Bivalvia
Zanclean	5.333	3.6	Campbell a	1995	17558	Mollusca	7805	Bivalvia
Zanclean	5.333	3.6	Campbell a	1995	17558	Mollusca	7805	Bivalvia
Zanclean	5.333	3.6	Campbell a	1995	17558	Mollusca	7805	Bivalvia
Late Pleistocene	0.126	0.0117	Richards	1962	18974	Mollusca	7805	Bivalvia
Late Pleistocene	0.126	0.0117	Richards	1962	18974	Mollusca	7805	Bivalvia
Late Pleistocene	0.126	0.0117	Richards	1962	18974	Mollusca	7805	Bivalvia
Late Pleistocene	0.126	0.0117	Hendy et a	2008	19909	Mollusca	7805	Bivalvia
Late Pleistocene	0.126	0.0117	Hendy et a	2008	19909	Mollusca	7805	Bivalvia
Pleistocene	2.588	0.0117	Holmes	1860	64712	Mollusca	7805	Bivalvia
Pleistocene	2.588	0.0117	Holmes	1860	64712	Mollusca	7805	Bivalvia
Late Pliocene	3.6	2.588	Ward and I	1987	16700	Mollusca	7805	Bivalvia
Early Pleistocene	2.588	0.781	Ward and I	1987	16700	Mollusca	7805	Bivalvia
Early Pleistocene	2.588	0.781	Gardner	1943	16984	Mollusca	7805	Bivalvia
Late Pleistocene	0.126	0.0117	Richards	1962	18974	Mollusca	7805	Bivalvia
Late Pleistocene	0.126	0.0117	Richards	1962	18974	Mollusca	7805	Bivalvia
Late Pleistocene	0.126	0.0117	Richards	1962	18974	Mollusca	7805	Bivalvia
Late Pleistocene	0.126	0.0117	Richards	1962	18974	Mollusca	7805	Bivalvia
Late Pleistocene	0.126	0.0117	Hendy et a	2008	19909	Mollusca	7805	Bivalvia
Late Pleistocene	0.126	0.0117	Hendy et a	2008	19909	Mollusca	7805	Bivalvia
Early Pleistocene	2.588	0.781	Hendy et a	2008	19909	Mollusca	7805	Bivalvia
Early Pleistocene	2.588	0.781	Hendy et a	2008	19909	Mollusca	7805	Bivalvia
Early Pleistocene	2.588	0.781	Hendy et a	2008	19909	Mollusca	7805	Bivalvia
Late Pleistocene	0.126	0.0117	Spencer ar	1987	15525	Mollusca	7805	Bivalvia
Late Pleistocene	0.126	0.0117	Spencer ar	1987	15525	Mollusca	7805	Bivalvia
Late Pleistocene	0.126	0.0117	Spencer ar	1987	15525	Mollusca	7805	Bivalvia
Late Pliocene	3.6	2.588	Edwards et	2005	17631	Mollusca	7805	Bivalvia
Late Pliocene	3.6	2.588	Hendy et a	2008	19909	Mollusca	7805	Bivalvia
Late Pliocene	3.6	2.588	Hendy et a	2008	19909	Mollusca	7805	Bivalvia
Late Pliocene	3.6	2.588	Hendy et a	2008	19909	Mollusca	7805	Bivalvia
Late Pleistocene	0.126	0.0117	Sellards	1917	9011	Mollusca	7805	Bivalvia
Late Pleistocene	0.126	0.0117	Sellards	1917	9011	Mollusca	7805	Bivalvia
Late Pleistocene	0.126	0.0117	Sellards	1917	9011	Mollusca	7805	Bivalvia
Late Pleistocene	0.126	0.0117	Sellards	1917	9011	Mollusca	7805	Bivalvia
Late Pleistocene	0.126	0.0117	Sellards	1917	9011	Mollusca	7805	Bivalvia
Late Pleistocene	0.126	0.0117	Hendy et a	2008	19909	Mollusca	7805	Bivalvia
Early Pleistocene	2.588	0.781	Mansfield	1932	11083	Mollusca	7805	Bivalvia
Early Pleistocene	2.588	0.781	Mansfield	1932	11083	Mollusca	7805	Bivalvia
Early Pleistocene	2.588	0.781	DuBar	1962	11119	Mollusca	7805	Bivalvia
Early Pleistocene	2.588	0.781	DuBar	1962	11119	Mollusca	7805	Bivalvia

Early Pleistocene		2.588	0.781	DuBar	1962	11119	Mollusca	7805	Bivalvia
Early Pleistocene		2.588	0.781	DuBar	1962	11119	Mollusca	7805	Bivalvia
Early Pleistocene		2.588	0.781	DuBar	1962	11119	Mollusca	7805	Bivalvia
Early Pleistocene		2.588	0.781	DuBar	1962	11119	Mollusca	7805	Bivalvia
Early Pleistocene		2.588	0.781	DuBar	1962	11119	Mollusca	7805	Bivalvia
Early Pleistocene		2.588	0.781	DuBar	1962	11119	Mollusca	7805	Bivalvia
Early Pleistocene		2.588	0.781	DuBar	1962	11119	Mollusca	7805	Bivalvia
Early Pleistocene		2.588	0.781	DuBar	1962	11119	Mollusca	7805	Bivalvia
Early Pleistocene		2.588	0.781	DuBar	1962	11119	Mollusca	7805	Bivalvia
Early Pleistocene		2.588	0.781	DuBar	1962	11119	Mollusca	7805	Bivalvia
Calabrian		1.806	0.781	DuBar	1962	11119	Mollusca	7805	Bivalvia
Calabrian		1.806	0.781	DuBar	1962	11119	Mollusca	7805	Bivalvia
Calabrian		1.806	0.781	DuBar	1962	11119	Mollusca	7805	Bivalvia
Calabrian		1.806	0.781	DuBar	1962	11119	Mollusca	7805	Bivalvia
Late Pleistocene		0.126	0.0117	DuBar	1962	11119	Mollusca	7805	Bivalvia
Late Pleistocene		0.126	0.0117	DuBar	1962	11119	Mollusca	7805	Bivalvia
Early Pleistocene		2.588	0.781	DuBar	1962	11119	Mollusca	7805	Bivalvia
Early Pleistocene		2.588	0.781	DuBar	1962	11119	Mollusca	7805	Bivalvia
Early Pleistocene		2.588	0.781	DuBar	1962	11119	Mollusca	7805	Bivalvia
Early Pleistocene		2.588	0.781	DuBar	1962	11119	Mollusca	7805	Bivalvia
Early Pleistocene		2.588	0.781	DuBar	1962	11119	Mollusca	7805	Bivalvia
Early Pleistocene		2.588	0.781	DuBar	1962	11119	Mollusca	7805	Bivalvia
Early Pleistocene		2.588	0.781	DuBar	1962	11119	Mollusca	7805	Bivalvia
Early Pleistocene		2.588	0.781	Olsson anc	1953	25463	Mollusca	7805	Bivalvia
Early Pleistocene		2.588	0.781	DuBar	1962	11119	Mollusca	7805	Bivalvia
Late Pleistocene		0.126	0.0117	DuBar	1962	11119	Mollusca	7805	Bivalvia
Late Pleistocene		0.126	0.0117	DuBar	1962	11119	Mollusca	7805	Bivalvia
Pliocene	Pleistocene	5.333	0.0117	DuBar	1962	11119	Mollusca	7805	Bivalvia
Pliocene	Pleistocene	5.333	0.0117	Woodring	1973	13235	Mollusca	7805	Bivalvia
Pliocene	Pleistocene	5.333	0.0117	DuBar	1962	11119	Mollusca	7805	Bivalvia
Pliocene	Pleistocene	5.333	0.0117	DuBar	1962	11119	Mollusca	7805	Bivalvia
Pliocene	Pleistocene	5.333	0.0117	DuBar	1962	11119	Mollusca	7805	Bivalvia
Pliocene	Pleistocene	5.333	0.0117	DuBar	1962	11119	Mollusca	7805	Bivalvia
Pliocene	Pleistocene	5.333	0.0117	DuBar	1962	11119	Mollusca	7805	Bivalvia
Pliocene	Pleistocene	5.333	0.0117	DuBar	1962	11119	Mollusca	7805	Bivalvia
Calabrian		1.806	0.781	DuBar	1962	11119	Mollusca	7805	Bivalvia
Late Pliocene		3.6	2.588	Mansfield	1932	17094	Mollusca	7805	Bivalvia
Late Pliocene		3.6	2.588	Mansfield	1932	17094	Mollusca	7805	Bivalvia
Late Pliocene		3.6	2.588	Mansfield	1932	17094	Mollusca	7805	Bivalvia
Late Pliocene		3.6	2.588	Mansfield	1932	17094	Mollusca	7805	Bivalvia
Late Pliocene		3.6	2.588	Mansfield	1932	17094	Mollusca	7805	Bivalvia
Late Pliocene		3.6	2.588	Mansfield	1932	17094	Mollusca	7805	Bivalvia
Late Pliocene		3.6	2.588	Mansfield	1932	17094	Mollusca	7805	Bivalvia
Late Pliocene		3.6	2.588	Mansfield	1932	17094	Mollusca	7805	Bivalvia
Late Pliocene		3.6	2.588	Mansfield	1932	17094	Mollusca	7805	Bivalvia
Late Pliocene		3.6	2.588	Mansfield	1932	17094	Mollusca	7805	Bivalvia
Late Pliocene		3.6	2.588	Mansfield	1932	17094	Mollusca	7805	Bivalvia

Late Pliocene	3.6	2.588	Mansfield	1932	17094	Mollusca	7805	Bivalvia
Late Pliocene	3.6	2.588	Mansfield	1932	17094	Mollusca	7805	Bivalvia
Late Pliocene	3.6	2.588	Mansfield	1932	17094	Mollusca	7805	Bivalvia
Late Pliocene	3.6	2.588	Mansfield	1932	17094	Mollusca	7805	Bivalvia
Late Pliocene	3.6	2.588	Mansfield	1932	17094	Mollusca	7805	Bivalvia
Late Pliocene	3.6	2.588	Mansfield	1932	17094	Mollusca	7805	Bivalvia
Late Pliocene	3.6	2.588	Mansfield	1932	17094	Mollusca	7805	Bivalvia
Late Pliocene	3.6	2.588	Mansfield	1932	17094	Mollusca	7805	Bivalvia
Late Pliocene	3.6	2.588	Mansfield	1932	17094	Mollusca	7805	Bivalvia
Late Pliocene	3.6	2.588	Mansfield	1932	17094	Mollusca	7805	Bivalvia
Late Pliocene	3.6	2.588	Mansfield	1932	17094	Mollusca	7805	Bivalvia
Late Pliocene	3.6	2.588	Mansfield	1932	17094	Mollusca	7805	Bivalvia
Late Pliocene	3.6	2.588	Mansfield	1932	17094	Mollusca	7805	Bivalvia
Late Pliocene	3.6	2.588	Mansfield	1932	17094	Mollusca	7805	Bivalvia
Early Pleistocene	2.588	0.781	Portell et a	1992	17634	Mollusca	7805	Bivalvia
Early Pleistocene	2.588	0.781	Portell et a	1992	17634	Mollusca	7805	Bivalvia
Early Pleistocene	2.588	0.781	Portell et a	1992	17634	Mollusca	7805	Bivalvia
Middle Pleistocene	0.781	0.126	Portell et a	1992	17634	Mollusca	7805	Bivalvia
Middle Pleistocene	0.781	0.126	Portell et a	1992	17634	Mollusca	7805	Bivalvia
Middle Pleistocene	0.781	0.126	Portell et a	1992	17634	Mollusca	7805	Bivalvia
Late Pleistocene	0.126	0.0117	Portell et a	1992	17634	Mollusca	7805	Bivalvia
Late Pleistocene	0.126	0.0117	Portell et a	1992	17634	Mollusca	7805	Bivalvia
Middle Pleistocene	0.781	0.126	McGinty	1970	19114	Mollusca	7805	Bivalvia
Middle Pleistocene	0.781	0.126	McGinty	1970	19114	Mollusca	7805	Bivalvia
Middle Pleistocene	0.781	0.126	McGinty	1970	19114	Mollusca	7805	Bivalvia
Middle Pleistocene	0.781	0.126	McGinty	1970	19114	Mollusca	7805	Bivalvia
Middle Pleistocene	0.781	0.126	McGinty	1970	19114	Mollusca	7805	Bivalvia
Middle Pleistocene	0.781	0.126	McGinty	1970	19114	Mollusca	7805	Bivalvia
Middle Pleistocene	0.781	0.126	McGinty	1970	19114	Mollusca	7805	Bivalvia
Middle Pleistocene	0.781	0.126	McGinty	1970	19114	Mollusca	7805	Bivalvia
Middle Pleistocene	0.781	0.126	McGinty	1970	19114	Mollusca	7805	Bivalvia
Middle Pleistocene	0.781	0.126	McGinty	1970	19114	Mollusca	7805	Bivalvia
Middle Pleistocene	0.781	0.126	McGinty	1970	19114	Mollusca	7805	Bivalvia
Late Pliocene	3.6	2.588	Mansfield	1939	19112	Mollusca	7805	Bivalvia
Late Pliocene	3.6	2.588	Mansfield	1939	19112	Mollusca	7805	Bivalvia
Late Pliocene	3.6	2.588	Mansfield	1939	19112	Mollusca	7805	Bivalvia
Late Pliocene	3.6	2.588	Mansfield	1939	19112	Mollusca	7805	Bivalvia
Late Pliocene	3.6	2.588	Mansfield	1939	19112	Mollusca	7805	Bivalvia
Late Pliocene	3.6	2.588	Mansfield	1939	19112	Mollusca	7805	Bivalvia
Late Pliocene	3.6	2.588	Mansfield	1939	19112	Mollusca	7805	Bivalvia
Late Pleistocene	0.126	0.0117	Hendy et a	2008	19909	Mollusca	7805	Bivalvia
Early Pleistocene	2.588	0.781	Schroeder	1954	25303	Mollusca	7805	Bivalvia
Pliocene	5.333	2.588	Schroeder	1954	25303	Mollusca	7805	Bivalvia
Pliocene	5.333	2.588	Schroeder	1954	25303	Mollusca	7805	Bivalvia
Early Pleistocene	2.588	0.781	Schroeder	1954	25303	Mollusca	7805	Bivalvia
Early Pleistocene	2.588	0.781	Schroeder	1954	25303	Mollusca	7805	Bivalvia
Early Pleistocene	2.588	0.781	Schroeder	1954	25303	Mollusca	7805	Bivalvia

Early Pleistocene	2.588	0.781	Schroeder	1954	25303	Mollusca	7805	Bivalvia
Pliocene	5.333	2.588	Schroeder	1954	25303	Mollusca	7805	Bivalvia
Pliocene	5.333	2.588	Schroeder	1954	25303	Mollusca	7805	Bivalvia
Pliocene	5.333	2.588	Schroeder	1954	25303	Mollusca	7805	Bivalvia
Pliocene	5.333	2.588	Schroeder	1954	25303	Mollusca	7805	Bivalvia
Pliocene	5.333	2.588	Schroeder	1954	25303	Mollusca	7805	Bivalvia
Early Pleistocene	2.588	0.781	Schroeder	1954	25303	Mollusca	7805	Bivalvia
Early Pleistocene	2.588	0.781	Olsson anc	1953	25463	Mollusca	7805	Bivalvia
Early Pleistocene	2.588	0.781	Olsson anc	1953	25463	Mollusca	7805	Bivalvia
Early Pleistocene	2.588	0.781	Olsson anc	1953	25463	Mollusca	7805	Bivalvia
Early Pleistocene	2.588	0.781	Olsson anc	1953	25463	Mollusca	7805	Bivalvia
Early Pleistocene	2.588	0.781	Olsson anc	1953	25463	Mollusca	7805	Bivalvia
Early Pleistocene	2.588	0.781	Olsson anc	1953	25463	Mollusca	7805	Bivalvia
Early Pleistocene	2.588	0.781	Olsson anc	1953	25463	Mollusca	7805	Bivalvia
Early Pleistocene	2.588	0.781	Olsson anc	1953	25463	Mollusca	7805	Bivalvia
Early Pleistocene	2.588	0.781	Olsson anc	1953	25463	Mollusca	7805	Bivalvia
Early Pleistocene	2.588	0.781	Olsson anc	1953	25463	Mollusca	7805	Bivalvia
Early Pleistocene	2.588	0.781	Olsson anc	1953	25463	Mollusca	7805	Bivalvia
Early Pleistocene	2.588	0.781	Olsson anc	1953	25463	Mollusca	7805	Bivalvia
Early Pleistocene	2.588	0.781	Olsson anc	1953	25463	Mollusca	7805	Bivalvia
Early Pleistocene	2.588	0.781	Olsson anc	1953	25463	Mollusca	7805	Bivalvia
Early Pleistocene	2.588	0.781	Olsson anc	1953	25463	Mollusca	7805	Bivalvia
Early Pleistocene	2.588	0.781	Olsson anc	1953	25463	Mollusca	7805	Bivalvia
Early Pleistocene	2.588	0.781	Olsson anc	1953	25463	Mollusca	7805	Bivalvia
Early Pleistocene	2.588	0.781	Olsson anc	1953	25463	Mollusca	7805	Bivalvia
Late Pleistocene	0.126	0.0117	Lovejoy	1992	26806	Mollusca	7805	Bivalvia
Middle Pleistocene	0.781	0.126	Petuch anc	2007	26813	Mollusca	7805	Bivalvia
Middle Pleistocene	0.781	0.126	Petuch anc	2007	26813	Mollusca	7805	Bivalvia
Late Pliocene	3.6	2.588	Olsson	1967	19826	Mollusca	7805	Bivalvia
Late Pliocene	3.6	2.588	Olsson	1967	19826	Mollusca	7805	Bivalvia
Late Pliocene	3.6	2.588	Olsson	1967	19826	Mollusca	7805	Bivalvia
Late Pliocene	3.6	2.588	Olsson anc	1964	26836	Mollusca	7805	Bivalvia
Early Pleistocene	2.588	0.781	Olsson anc	1964	26836	Mollusca	7805	Bivalvia
Early Pleistocene	2.588	0.781	Olsson anc	1964	26836	Mollusca	7805	Bivalvia
Middle Pleistocene	0.781	0.126	Olsson anc	1964	26836	Mollusca	7805	Bivalvia
Middle Pleistocene	0.781	0.126	Olsson anc	1964	26836	Mollusca	7805	Bivalvia
Late Pliocene	3.6	2.588	DuBar	1958	25514	Mollusca	7805	Bivalvia
Late Pliocene	3.6	2.588	DuBar	1958	25514	Mollusca	7805	Bivalvia
Late Pliocene	3.6	2.588	DuBar	1958	25514	Mollusca	7805	Bivalvia
Late Pliocene	3.6	2.588	DuBar	1958	25514	Mollusca	7805	Bivalvia
Early Pleistocene	2.588	0.781	Hendy et a	2008	19909	Mollusca	7805	Bivalvia
Early Pleistocene	2.588	0.781	Hendy et a	2008	19909	Mollusca	7805	Bivalvia
Late Pleistocene	0.126	0.0117	Hendy et a	2008	19909	Mollusca	7805	Bivalvia
Late Pliocene	3.6	2.588	Petuch anc	2007	26813	Mollusca	7805	Bivalvia
Early Pleistocene	2.588	0.781	DuBar	1958	25514	Mollusca	7805	Bivalvia
Late Pleistocene	0.126	0.0117	Hendy et a	2008	19909	Mollusca	7805	Bivalvia
Late Pliocene	3.6	2.588	Hendy et a	2008	19909	Mollusca	7805	Bivalvia

Early Pleistocene	2.588	0.781	Hendy et a	2008	19909	Mollusca	7805	Bivalvia
Early Pleistocene	2.588	0.781	Hendy et a	2008	19909	Mollusca	7805	Bivalvia
Early Pleistocene	2.588	0.781	Hendy et a	2008	19909	Mollusca	7805	Bivalvia
Early Pleistocene	2.588	0.781	Hendy et a	2008	19909	Mollusca	7805	Bivalvia
Early Pleistocene	2.588	0.781	Hendy et a	2008	19909	Mollusca	7805	Bivalvia
Early Pleistocene	2.588	0.781	Hendy et a	2008	19909	Mollusca	7805	Bivalvia
Early Pleistocene	2.588	0.781	Hendy et a	2008	19909	Mollusca	7805	Bivalvia
Early Pleistocene	2.588	0.781	Hendy et a	2008	19909	Mollusca	7805	Bivalvia
Late Pliocene	3.6	2.588	Hendy et a	2008	19909	Mollusca	7805	Bivalvia
Late Pliocene	3.6	2.588	Hendy et a	2008	19909	Mollusca	7805	Bivalvia
Late Pliocene	3.6	2.588	Hendy et a	2008	19909	Mollusca	7805	Bivalvia
Late Pliocene	3.6	2.588	Hendy et a	2008	19909	Mollusca	7805	Bivalvia
Late Pliocene	3.6	2.588	Hendy et a	2008	19909	Mollusca	7805	Bivalvia
Middle Pleistocene	0.781	0.126	Hendy et a	2008	19909	Mollusca	7805	Bivalvia
Middle Pleistocene	0.781	0.126	Hendy et a	2008	19909	Mollusca	7805	Bivalvia
Middle Pleistocene	0.781	0.126	Hendy et a	2008	19909	Mollusca	7805	Bivalvia
Late Pleistocene	0.126	0.0117	Hendy et a	2008	19909	Mollusca	7805	Bivalvia
Early Pleistocene	2.588	0.781	Hendy et a	2008	19909	Mollusca	7805	Bivalvia
Early Pleistocene	2.588	0.781	Hendy et a	2008	19909	Mollusca	7805	Bivalvia
Early Pleistocene	2.588	0.781	Hendy et a	2008	19909	Mollusca	7805	Bivalvia
Early Pleistocene	2.588	0.781	Hendy et a	2008	19909	Mollusca	7805	Bivalvia
Early Pleistocene	2.588	0.781	Hendy et a	2008	19909	Mollusca	7805	Bivalvia
Late Pliocene	3.6	2.588	Vokes	1969	31134	Mollusca	7805	Bivalvia
Late Pliocene	3.6	2.588	Vokes	1969	31134	Mollusca	7805	Bivalvia
Late Pliocene	3.6	2.588	Vokes	1969	31134	Mollusca	7805	Bivalvia
Late Pliocene	3.6	2.588	Vokes	1969	31134	Mollusca	7805	Bivalvia
Pleistocene	2.588	0.0117	Vokes	1969	31134	Mollusca	7805	Bivalvia
Late Plioce Early Pleist	3.6	0.781	Vokes	1969	31134	Mollusca	7805	Bivalvia
Pleistocene	2.588	0.0117	Vokes	1969	31134	Mollusca	7805	Bivalvia
Early Pleistocene	2.588	0.781	Vokes	1969	31134	Mollusca	7805	Bivalvia
Early Pleistocene	2.588	0.781	Vokes	1969	31134	Mollusca	7805	Bivalvia
Early Pleistocene	2.588	0.781	Vokes	1969	31134	Mollusca	7805	Bivalvia
Late Plioce Early Pleist	3.6	0.781	Vokes	1969	31134	Mollusca	7805	Bivalvia
Early Pleistocene	2.588	0.781	Vokes	1969	31134	Mollusca	7805	Bivalvia
Late Plioce Early Pleist	3.6	0.781	Vokes	1969	31134	Mollusca	7805	Bivalvia
Early Pleistocene	2.588	0.781	Vokes	1969	31134	Mollusca	7805	Bivalvia
Early Pleistocene	2.588	0.781	Vokes	1969	31134	Mollusca	7805	Bivalvia
Early Pleistocene	2.588	0.781	Vokes	1969	31134	Mollusca	7805	Bivalvia
Early Pleistocene	2.588	0.781	Vokes	1969	31134	Mollusca	7805	Bivalvia
Early Pleistocene	2.588	0.781	Vokes	1969	31134	Mollusca	7805	Bivalvia
Pleistocene	2.588	0.0117	Vokes	1969	31134	Mollusca	7805	Bivalvia
Early Pleistocene	2.588	0.781	Vokes	1969	31134	Mollusca	7805	Bivalvia
Late Pleistocene	0.126	0.0117	Vokes	1969	31134	Mollusca	7805	Bivalvia
Late Pleistocene	0.126	0.0117	Vokes	1969	31134	Mollusca	7805	Bivalvia
Late Pliocene	3.6	2.588	Smith	1941	32333	Mollusca	7805	Bivalvia
Late Pliocene	3.6	2.588	Smith	1941	32333	Mollusca	7805	Bivalvia
Late Pliocene	3.6	2.588	Weisbord	1971	39743	Mollusca	7805	Bivalvia

Late Pliocene	3.6	2.588	Weisbord	1971	39743	Mollusca	7805	Bivalvia
Late Pliocene	3.6	2.588	Weisbord	1971	39743	Mollusca	7805	Bivalvia
Late Pliocene	3.6	2.588	Weisbord	1971	39743	Mollusca	7805	Bivalvia
Late Pleistocene	0.126	0.0117	Kittle and F	2010	40884	Mollusca	7805	Bivalvia
Late Pleistocene	0.126	0.0117	Kittle and F	2010	40884	Mollusca	7805	Bivalvia
Late Pleistocene	0.126	0.0117	Hulbert an	1998	1962	Mollusca	7805	Bivalvia
Late Pleistocene	0.126	0.0117	Hulbert an	1998	1962	Mollusca	7805	Bivalvia
Late Pleistocene	0.126	0.0117	Hulbert an	1998	1962	Mollusca	7805	Bivalvia
Late Pleistocene	0.126	0.0117	Hulbert an	1998	1962	Mollusca	7805	Bivalvia
Late Pleistocene	0.126	0.0117	Hulbert an	1998	1962	Mollusca	7805	Bivalvia
Late Pleistocene	0.126	0.0117	Hulbert an	1998	1962	Mollusca	7805	Bivalvia
Late Pleistocene	0.126	0.0117	Richards	1962	18974	Mollusca	7805	Bivalvia
Late Pleistocene	0.126	0.0117	Richards	1962	18974	Mollusca	7805	Bivalvia
Late Pleistocene	0.126	0.0117	DuBar	1971	17556	Mollusca	7805	Bivalvia
Pliocene	5.333	2.588	DuBar	1971	17556	Mollusca	7805	Bivalvia
Late Pleistocene	0.126	0.0117	DuBar	1971	17556	Mollusca	7805	Bivalvia
Late Pleistocene	0.126	0.0117	DuBar	1971	17556	Mollusca	7805	Bivalvia
Late Pleistocene	0.126	0.0117	DuBar	1971	17556	Mollusca	7805	Bivalvia
Late Pleistocene	0.126	0.0117	DuBar	1971	17556	Mollusca	7805	Bivalvia
Late Pleistocene	0.126	0.0117	DuBar	1971	17556	Mollusca	7805	Bivalvia
Late Pleistocene	0.126	0.0117	DuBar	1971	17556	Mollusca	7805	Bivalvia
Pliocene	5.333	2.588	DuBar	1971	17556	Mollusca	7805	Bivalvia
Zanclean	5.333	3.6	Campbell a	1995	17558	Mollusca	7805	Bivalvia
Early Pliocene	5.333	3.6	Campbell a	1995	17558	Mollusca	7805	Bivalvia
Early Pleistocene	2.588	0.781	DuBar	1971	17556	Mollusca	7805	Bivalvia
Late Pleistocene	0.126	0.0117	Richards	1962	18974	Mollusca	7805	Bivalvia
Late Pleistocene	0.126	0.0117	Richards	1962	18974	Mollusca	7805	Bivalvia
Late Pleistocene	0.126	0.0117	Richards	1962	18974	Mollusca	7805	Bivalvia
Late Pleistocene	0.126	0.0117	Richards	1962	18974	Mollusca	7805	Bivalvia
Late Pleistocene	0.126	0.0117	Richards	1962	18974	Mollusca	7805	Bivalvia
Late Pleistocene	0.126	0.0117	Richards	1962	18974	Mollusca	7805	Bivalvia
Late Pleistocene	0.126	0.0117	Richards	1962	18974	Mollusca	7805	Bivalvia
Late Pleistocene	0.126	0.0117	Richards	1962	18974	Mollusca	7805	Bivalvia
Late Pleistocene	0.126	0.0117	Richards	1962	18974	Mollusca	7805	Bivalvia
Late Pleistocene	0.126	0.0117	Richards	1962	18974	Mollusca	7805	Bivalvia
Late Pleistocene	0.126	0.0117	Hendy et a	2008	19909	Mollusca	7805	Bivalvia
Late Pleistocene	0.126	0.0117	Hendy et a	2008	19909	Mollusca	7805	Bivalvia
Late Pleistocene	0.126	0.0117	Hendy et a	2008	19909	Mollusca	7805	Bivalvia
Late Pleistocene	0.126	0.0117	Hendy et a	2008	19909	Mollusca	7805	Bivalvia
Late Pleistocene	0.126	0.0117	Hendy et a	2008	19909	Mollusca	7805	Bivalvia
Late Pleistocene	0.126	0.0117	Hendy et a	2008	19909	Mollusca	7805	Bivalvia
Late Pleistocene	0.126	0.0117	Hendy et a	2008	19909	Mollusca	7805	Bivalvia
Pleistocene	2.588	0.0117	Hendy et a	2008	19909	Mollusca	7805	Bivalvia
Pleistocene	2.588	0.0117	Hendy et a	2008	19909	Mollusca	7805	Bivalvia
Pleistocene	2.588	0.0117	Hendy et a	2008	19909	Mollusca	7805	Bivalvia
Early Pleistocene	2.588	0.781	Vokes	1969	31134	Mollusca	7805	Bivalvia
Late Pleistocene	0.126	0.0117	Cooke	1936	31145	Mollusca	7805	Bivalvia

Late Pleistocene	0.126	0.0117	Cooke	1936	31145	Mollusca	7805	Bivalvia
Early Pleistocene	2.588	0.781	Cooke	1936	31145	Mollusca	7805	Bivalvia
Early Pleistocene	2.588	0.781	Cooke	1936	31145	Mollusca	7805	Bivalvia
Late Pliocene	3.6	2.588	Cooke	1936	31145	Mollusca	7805	Bivalvia
Pleistocene	2.588	0.0117	Holmes	1860	64712	Mollusca	7805	Bivalvia
Late Pliocene	3.6	2.588	Ward and I	1987	16700	Mollusca	7805	Bivalvia
Late Pliocene	3.6	2.588	Ward and I	1987	16700	Mollusca	7805	Bivalvia
Late Pliocene	3.6	2.588	Ward and I	1987	16700	Mollusca	7805	Bivalvia
Early Pleistocene	2.588	0.781	Ward and I	1987	16700	Mollusca	7805	Bivalvia
Early Pleistocene	2.588	0.781	Ward and I	1987	16700	Mollusca	7805	Bivalvia
Late Pliocene	3.6	2.588	Gardner	1943	16984	Mollusca	7805	Bivalvia
Late Pliocene	3.6	2.588	Gardner	1943	16984	Mollusca	7805	Bivalvia
Late Pliocene	3.6	2.588	Gardner	1943	16984	Mollusca	7805	Bivalvia
Early Pleistocene	2.588	0.781	Gardner	1943	16984	Mollusca	7805	Bivalvia
Early Pleistocene	2.588	0.781	Gardner	1943	16984	Mollusca	7805	Bivalvia
Early Pleistocene	2.588	0.781	Gardner	1943	16984	Mollusca	7805	Bivalvia
Late Pliocene	3.6	2.588	Gardner	1943	16984	Mollusca	7805	Bivalvia
Late Pliocene	3.6	2.588	Gardner	1943	16984	Mollusca	7805	Bivalvia
Late Pliocene	3.6	2.588	Gardner	1943	16984	Mollusca	7805	Bivalvia
Late Pliocene	3.6	2.588	Gardner	1943	16984	Mollusca	7805	Bivalvia
Pleistocene	2.588	0.0117	Gardner	1943	16984	Mollusca	7805	Bivalvia
Late Pliocene	3.6	2.588	Gardner	1943	16984	Mollusca	7805	Bivalvia
Early Pleistocene	2.588	0.781	DuBar	1971	17556	Mollusca	7805	Bivalvia
Early Pleistocene	2.588	0.781	Miller	1990	17561	Mollusca	7805	Bivalvia
Early Pleistocene	2.588	0.781	Miller	1990	17561	Mollusca	7805	Bivalvia
Early Pleistocene	2.588	0.781	Miller	1990	17561	Mollusca	7805	Bivalvia
Early Pleistocene	2.588	0.781	Miller	1990	17561	Mollusca	7805	Bivalvia
Late Pleistocene	0.126	0.0117	Richards	1962	18974	Mollusca	7805	Bivalvia
Late Pleistocene	0.126	0.0117	Richards	1962	18974	Mollusca	7805	Bivalvia
Late Pleistocene	0.126	0.0117	Richards	1962	18974	Mollusca	7805	Bivalvia
Late Pleistocene	0.126	0.0117	Richards	1962	18974	Mollusca	7805	Bivalvia
Late Pleistocene	0.126	0.0117	Richards	1962	18974	Mollusca	7805	Bivalvia
Late Pleistocene	0.126	0.0117	Richards	1962	18974	Mollusca	7805	Bivalvia
Late Pleistocene	0.126	0.0117	Richards	1962	18974	Mollusca	7805	Bivalvia
Late Pleistocene	0.126	0.0117	Richards	1962	18974	Mollusca	7805	Bivalvia
Late Pleistocene	0.126	0.0117	Richards	1962	18974	Mollusca	7805	Bivalvia
Late Pleistocene	0.126	0.0117	Richards	1962	18974	Mollusca	7805	Bivalvia
Late Pleistocene	0.126	0.0117	Richards	1962	18974	Mollusca	7805	Bivalvia
Late Pleistocene	0.126	0.0117	Richards	1962	18974	Mollusca	7805	Bivalvia
Late Pleistocene	0.126	0.0117	Richards	1962	18974	Mollusca	7805	Bivalvia
Late Pleistocene	0.126	0.0117	Richards	1962	18974	Mollusca	7805	Bivalvia
Late Pleistocene	0.126	0.0117	Richards	1962	18974	Mollusca	7805	Bivalvia
Late Pleistocene	0.126	0.0117	Richards	1962	18974	Mollusca	7805	Bivalvia
Late Pleistocene	0.126	0.0117	Richards	1962	18974	Mollusca	7805	Bivalvia
Late Pleistocene	0.126	0.0117	Richards	1962	18974	Mollusca	7805	Bivalvia
Late Pleistocene	0.126	0.0117	Richards	1962	18974	Mollusca	7805	Bivalvia
Late Pleistocene	0.126	0.0117	Richards	1962	18974	Mollusca	7805	Bivalvia

Late Pleistocene	0.126	0.0117	Richards	1962	18974	Mollusca	7805	Bivalvia
Late Pleistocene	0.126	0.0117	Richards	1962	18974	Mollusca	7805	Bivalvia
Late Pleistocene	0.126	0.0117	Richards	1962	18974	Mollusca	7805	Bivalvia
Late Pleistocene	0.126	0.0117	Richards	1962	18974	Mollusca	7805	Bivalvia
Late Pleistocene	0.126	0.0117	Hendy et a	2008	19909	Mollusca	7805	Bivalvia
Early Pleistocene	2.588	0.781	Hendy et a	2008	19909	Mollusca	7805	Bivalvia
Late Pleistocene	0.126	0.0117	Hendy et a	2008	19909	Mollusca	7805	Bivalvia
Late Pliocene	3.6	2.588	Hendy et a	2008	19909	Mollusca	7805	Bivalvia
Late Pliocene	3.6	2.588	Hendy et a	2008	19909	Mollusca	7805	Bivalvia
Pliocene	5.333	2.588	Hendy et a	2008	19909	Mollusca	7805	Bivalvia
Late Pliocene	3.6	2.588	Hendy et a	2008	19909	Mollusca	7805	Bivalvia
Late Pliocene	3.6	2.588	Hendy et a	2008	19909	Mollusca	7805	Bivalvia
Late Pliocene	3.6	2.588	Hendy et a	2008	19909	Mollusca	7805	Bivalvia
Early Pleistocene	2.588	0.781	Hendy et a	2008	19909	Mollusca	7805	Bivalvia
Late Pliocene	3.6	2.588	Hendy et a	2008	19909	Mollusca	7805	Bivalvia
Early Pleistocene	2.588	0.781	Hendy et a	2008	19909	Mollusca	7805	Bivalvia
Early Pleistocene	2.588	0.781	Hendy et a	2008	19909	Mollusca	7805	Bivalvia
Early Pleistocene	2.588	0.781	Hendy et a	2008	19909	Mollusca	7805	Bivalvia
Early Pleistocene	2.588	0.781	Hendy et a	2008	19909	Mollusca	7805	Bivalvia
Early Pleistocene	2.588	0.781	Hendy et a	2008	19909	Mollusca	7805	Bivalvia
Early Pleistocene	2.588	0.781	Hendy et a	2008	19909	Mollusca	7805	Bivalvia
Early Pleistocene	2.588	0.781	Vokes	1969	31134	Mollusca	7805	Bivalvia
Early Pleistocene	2.588	0.781	Vokes	1969	31134	Mollusca	7805	Bivalvia
Early Pleistocene	2.588	0.781	Vokes	1969	31134	Mollusca	7805	Bivalvia
Early Pleistocene	2.588	0.781	Miller and	1988	72890	Mollusca	7805	Bivalvia
Early Pleistocene	2.588	0.781	Miller and	1988	72890	Mollusca	7805	Bivalvia
Early Pleistocene	2.588	0.781	Miller and	1988	72890	Mollusca	7805	Bivalvia
Early Pleistocene	2.588	0.781	Miller and	1988	72890	Mollusca	7805	Bivalvia
Pleistocene	2.588	0.0117	Coch	1971	441	Mollusca	7805	Bivalvia
Pleistocene	2.588	0.0117	Coch	1971	441	Mollusca	7805	Bivalvia
Late Pleistocene	0.126	0.0117	Spencer ar	1987	15525	Mollusca	7805	Bivalvia
Late Pleistocene	0.126	0.0117	Spencer ar	1987	15525	Mollusca	7805	Bivalvia
Late Pleistocene	0.126	0.0117	Spencer ar	1987	15525	Mollusca	7805	Bivalvia
Late Pleistocene	0.126	0.0117	Spencer ar	1987	15525	Mollusca	7805	Bivalvia
Late Pleistocene	0.126	0.0117	Spencer ar	1987	15525	Mollusca	7805	Bivalvia
Pliocene	5.333	2.588	Gardner	1943	16984	Mollusca	7805	Bivalvia
Pliocene	5.333	2.588	Gardner	1943	16984	Mollusca	7805	Bivalvia
Late Pleistocene	0.126	0.0117	Hendy et a	2008	19909	Mollusca	7805	Bivalvia
Late Pliocene	3.6	2.588	Ward	1993	26351	Mollusca	7805	Bivalvia
Late Pliocene	3.6	2.588	Ward	1993	26351	Mollusca	7805	Bivalvia
Late Pliocene	3.6	2.588	Ward	1993	26351	Mollusca	7805	Bivalvia
Late Pliocene	3.6	2.588	Ward	1993	26351	Mollusca	7805	Bivalvia
Late Pliocene	3.6	2.588	Ward	1993	26351	Mollusca	7805	Bivalvia
Late Pliocene	3.6	2.588	Ward	1993	26351	Mollusca	7805	Bivalvia
Late Pliocene	3.6	2.588	Ward	1993	26351	Mollusca	7805	Bivalvia
Late Pliocene	3.6	2.588	Hendy et a	2008	19909	Mollusca	7805	Bivalvia
Late Pliocene	3.6	2.588	Hendy et a	2008	19909	Mollusca	7805	Bivalvia

Late Pliocene		3.6	2.588	Hendy et a	2008	19909	Mollusca	7805 Bivalvia
Late Pleistocene		0.126	0.0117	Hendy et a	2008	19909	Mollusca	7805 Bivalvia
Pliocene	Pleistocene	5.333	0.0117	DuBar	1962	11119	Mollusca	7805 Bivalvia
Late Pliocene		3.6	2.588	Mansfield	1932	17094	Mollusca	7805 Bivalvia
Late Pliocene		3.6	2.588	Mansfield	1932	17094	Mollusca	7805 Bivalvia
Late Pliocene		3.6	2.588	Mansfield	1932	17094	Mollusca	7805 Bivalvia
Late Pliocene		3.6	2.588	Mansfield	1932	17094	Mollusca	7805 Bivalvia
Late Pleistocene		0.126	0.0117	Hendy et a	2008	19909	Mollusca	7805 Bivalvia
Early Pleistocene		2.588	0.781	Olsson anc	1953	25463	Mollusca	7805 Bivalvia
Early Pleistocene		2.588	0.781	Olsson anc	1953	25463	Mollusca	7805 Bivalvia
Late Pliocene		3.6	2.588	Petuch anc	2007	26813	Mollusca	7805 Bivalvia
Late Pliocene		3.6	2.588	Hendy et a	2008	19909	Mollusca	7805 Bivalvia
Late Pliocene		3.6	2.588	Hendy et a	2008	19909	Mollusca	7805 Bivalvia
Late Pliocene		3.6	2.588	Hendy et a	2008	19909	Mollusca	7805 Bivalvia
Late Pliocene		3.6	2.588	Hendy et a	2008	19909	Mollusca	7805 Bivalvia
Late Pliocene		3.6	2.588	Smith	1941	32333	Mollusca	7805 Bivalvia
Late Pleistocene		0.126	0.0117	DuBar	1971	17556	Mollusca	7805 Bivalvia
Late Pliocene		3.6	2.588	Campbell a	1995	17558	Mollusca	7805 Bivalvia
Zanclean		5.333	3.6	Campbell a	1995	17558	Mollusca	7805 Bivalvia
Zanclean		5.333	3.6	Campbell a	1995	17558	Mollusca	7805 Bivalvia
Late Pleistocene		0.126	0.0117	Hendy et a	2008	19909	Mollusca	7805 Bivalvia
Pleistocene		2.588	0.0117	Hendy et a	2008	19909	Mollusca	7805 Bivalvia
Pleistocene		2.588	0.0117	Hendy et a	2008	19909	Mollusca	7805 Bivalvia
Early Pleistocene		2.588	0.781	Cooke	1936	31145	Mollusca	7805 Bivalvia
Early Pleistocene		2.588	0.781	Cooke	1936	31145	Mollusca	7805 Bivalvia
Early Pleistocene		2.588	0.781	Cooke	1936	31145	Mollusca	7805 Bivalvia
Late Pliocene		3.6	2.588	Cooke	1936	31145	Mollusca	7805 Bivalvia
Late Pliocene		3.6	2.588	Cooke	1936	31145	Mollusca	7805 Bivalvia
Late Pliocene		3.6	2.588	Ward and I	1987	16700	Mollusca	7805 Bivalvia
Late Pliocene		3.6	2.588	Ward and I	1987	16700	Mollusca	7805 Bivalvia
Late Pliocene		3.6	2.588	Ward and I	1987	16700	Mollusca	7805 Bivalvia
Late Pliocene		3.6	2.588	Ward and I	1987	16700	Mollusca	7805 Bivalvia
Late Pliocene		3.6	2.588	Ward and I	1987	16700	Mollusca	7805 Bivalvia
Early Pleistocene		2.588	0.781	Ward and I	1987	16700	Mollusca	7805 Bivalvia
Early Pleistocene		2.588	0.781	Ward and I	1987	16700	Mollusca	7805 Bivalvia
Early Pleistocene		2.588	0.781	Ward and I	1987	16700	Mollusca	7805 Bivalvia
Early Pleistocene		2.588	0.781	Ward and I	1987	16700	Mollusca	7805 Bivalvia
Late Pliocene		3.6	2.588	Ward	1992	16985	Mollusca	7805 Bivalvia
Early Plioce	Late Plioce	5.333	2.588	Gardner	1943	16984	Mollusca	7805 Bivalvia
Early Plioce	Late Plioce	5.333	2.588	Gardner	1943	16984	Mollusca	7805 Bivalvia
Late Pliocene		3.6	2.588	Gardner	1943	16984	Mollusca	7805 Bivalvia
Early Plioce	Late Plioce	5.333	2.588	Gardner	1943	16984	Mollusca	7805 Bivalvia
Early Plioce	Late Plioce	5.333	2.588	Gardner	1943	16984	Mollusca	7805 Bivalvia
Early Plioce	Late Plioce	5.333	2.588	Gardner	1943	16984	Mollusca	7805 Bivalvia
Late Pliocene		3.6	2.588	Gardner	1943	16984	Mollusca	7805 Bivalvia
Early Plioce	Late Plioce	5.333	2.588	Gardner	1943	16984	Mollusca	7805 Bivalvia
Early Plioce	Late Plioce	5.333	2.588	Gardner	1943	16984	Mollusca	7805 Bivalvia

Late Pliocene	3.6	2.588	Gardner	1943	16984	Mollusca	7805	Bivalvia
Late Pliocene	3.6	2.588	Gardner	1943	16984	Mollusca	7805	Bivalvia
Late Pliocene	3.6	2.588	Gardner	1943	16984	Mollusca	7805	Bivalvia
Late Pliocene	3.6	2.588	Gardner	1943	16984	Mollusca	7805	Bivalvia
Early Pleistocene	2.588	0.781	Gardner	1943	16984	Mollusca	7805	Bivalvia
Early Pleistocene	2.588	0.781	Gardner	1943	16984	Mollusca	7805	Bivalvia
Early Pleistocene	2.588	0.781	Gardner	1943	16984	Mollusca	7805	Bivalvia
Late Pliocene	3.6	2.588	Hendy et a	2008	19909	Mollusca	7805	Bivalvia
Gelasian	2.588	1.806	Bailey and	1986	23840	Mollusca	7805	Bivalvia
Late Pliocene	3.6	2.588	Hendy et a	2008	19909	Mollusca	7805	Bivalvia
Late Pliocene	3.6	2.588	Hendy et a	2008	19909	Mollusca	7805	Bivalvia
Pliocene	5.333	2.588	Hendy et a	2008	19909	Mollusca	7805	Bivalvia
Pliocene	5.333	2.588	Hendy et a	2008	19909	Mollusca	7805	Bivalvia
Late Pliocene	3.6	2.588	Hendy et a	2008	19909	Mollusca	7805	Bivalvia
Late Pliocene	3.6	2.588	Hendy et a	2008	19909	Mollusca	7805	Bivalvia
Late Pliocene	3.6	2.588	Hendy et a	2008	19909	Mollusca	7805	Bivalvia
Early Pleistocene	2.588	0.781	Hendy et a	2008	19909	Mollusca	7805	Bivalvia
Early Pleistocene	2.588	0.781	Hendy et a	2008	19909	Mollusca	7805	Bivalvia
Late Pliocene	3.6	2.588	Hendy et a	2008	19909	Mollusca	7805	Bivalvia
Early Pleistocene	2.588	0.781	Hendy et a	2008	19909	Mollusca	7805	Bivalvia
Early Pleistocene	2.588	0.781	Hendy et a	2008	19909	Mollusca	7805	Bivalvia
Early Pleistocene	2.588	0.781	Hendy et a	2008	19909	Mollusca	7805	Bivalvia
Early Pleistocene	2.588	0.781	Hendy et a	2008	19909	Mollusca	7805	Bivalvia
Early Pleistocene	2.588	0.781	Hendy et a	2008	19909	Mollusca	7805	Bivalvia
Late Pleistocene	0.126	0.0117	Spencer ar	1987	15525	Mollusca	7805	Bivalvia
Early Pliocene	5.333	3.6	Ward	1992	16985	Mollusca	7805	Bivalvia
Pliocene	5.333	2.588	Gardner	1943	16984	Mollusca	7805	Bivalvia
Pliocene	5.333	2.588	Gardner	1943	16984	Mollusca	7805	Bivalvia
Late Pliocene	3.6	2.588	Edwards et	2005	17631	Mollusca	7805	Bivalvia
Late Pliocene	3.6	2.588	Edwards et	2005	17631	Mollusca	7805	Bivalvia
Zanclean	5.333	3.6	Ward	1992	16985	Mollusca	7805	Bivalvia
Zanclean	5.333	3.6	Ward	1992	16985	Mollusca	7805	Bivalvia
Zanclean	5.333	3.6	Ward	1992	16985	Mollusca	7805	Bivalvia
Zanclean	5.333	3.6	Ward	1992	16985	Mollusca	7805	Bivalvia
Late Pliocene	3.6	2.588	Ward	1992	16985	Mollusca	7805	Bivalvia
Late Pliocene	3.6	2.588	Ward	1993	26351	Mollusca	7805	Bivalvia
Late Pliocene	3.6	2.588	Ward	1993	26351	Mollusca	7805	Bivalvia
Late Pliocene	3.6	2.588	Ward	1993	26351	Mollusca	7805	Bivalvia
Late Pliocene	3.6	2.588	Ward	1993	26351	Mollusca	7805	Bivalvia
Late Pliocene	3.6	2.588	Ward	1993	26351	Mollusca	7805	Bivalvia
Late Pliocene	3.6	2.588	Ward	1993	26351	Mollusca	7805	Bivalvia
Late Pliocene	3.6	2.588	Ward	1993	26351	Mollusca	7805	Bivalvia
Late Pliocene	3.6	2.588	Ward	1993	26351	Mollusca	7805	Bivalvia
Late Pliocene	3.6	2.588	Ward	1993	26351	Mollusca	7805	Bivalvia
Late Pliocene	3.6	2.588	Ward	1993	26351	Mollusca	7805	Bivalvia
Late Pliocene	3.6	2.588	Ward	1993	26351	Mollusca	7805	Bivalvia
Late Pliocene	3.6	2.588	Ward	1993	26351	Mollusca	7805	Bivalvia

Late Pliocene		3.6	2.588	Ward	1993	26351	Mollusca	7805	Bivalvia
Late Pliocene		3.6	2.588	Ward	1993	26351	Mollusca	7805	Bivalvia
Late Pliocene		3.6	2.588	Ward	1993	26351	Mollusca	7805	Bivalvia
Late Pliocene		3.6	2.588	Hendy et a	2008	19909	Mollusca	7805	Bivalvia
Late Pliocene		3.6	2.588	Hendy et a	2008	19909	Mollusca	7805	Bivalvia
Late Pliocene		3.6	2.588	Hendy et a	2008	19909	Mollusca	7805	Bivalvia
Late Pliocene		3.6	2.588	Hendy et a	2008	19909	Mollusca	7805	Bivalvia
Late Pliocene		3.6	2.588	Hendy et a	2008	19909	Mollusca	7805	Bivalvia
Late Pliocene		3.6	2.588	Hendy et a	2008	19909	Mollusca	7805	Bivalvia
Late Pliocene		3.6	2.588	Hendy et a	2008	19909	Mollusca	7805	Bivalvia
Late Pliocene		3.6	2.588	Hendy et a	2008	19909	Mollusca	7805	Bivalvia
Late Pliocene		3.6	2.588	Hendy et a	2008	19909	Mollusca	7805	Bivalvia
Late Pliocene		3.6	2.588	Hendy et a	2008	19909	Mollusca	7805	Bivalvia
Pleistocene		2.588	0.0117	Holmes	1860	64712	Mollusca	7805	Bivalvia
Late Pleistocene		0.126	0.0117	Sellards	1917	9011	Mollusca	7805	Bivalvia
Late Pleistocene		0.126	0.0117	Sellards	1917	9011	Mollusca	7805	Bivalvia
Late Pleistocene		0.126	0.0117	Sellards	1917	9011	Mollusca	7805	Bivalvia
Late Pleistocene		0.126	0.0117	Sellards	1917	9011	Mollusca	7805	Bivalvia
Late Pleistocene		0.126	0.0117	Sellards	1917	9011	Mollusca	7805	Bivalvia
Early Pleistocene		2.588	0.781	Mansfield	1932	11083	Mollusca	7805	Bivalvia
Early Pleistocene		2.588	0.781	DuBar	1962	11119	Mollusca	7805	Bivalvia
Early Pleistocene		2.588	0.781	DuBar	1962	11119	Mollusca	7805	Bivalvia
Early Pleistocene		2.588	0.781	DuBar	1962	11119	Mollusca	7805	Bivalvia
Early Pleistocene		2.588	0.781	DuBar	1962	11119	Mollusca	7805	Bivalvia
Early Pleistocene		2.588	0.781	DuBar	1962	11119	Mollusca	7805	Bivalvia
Early Pleistocene		2.588	0.781	DuBar	1962	11119	Mollusca	7805	Bivalvia
Early Pleistocene		2.588	0.781	DuBar	1962	11119	Mollusca	7805	Bivalvia
Early Pleistocene		2.588	0.781	DuBar	1962	11119	Mollusca	7805	Bivalvia
Early Pleistocene		2.588	0.781	DuBar	1962	11119	Mollusca	7805	Bivalvia
Calabrian		1.806	0.781	DuBar	1962	11119	Mollusca	7805	Bivalvia
Calabrian		1.806	0.781	DuBar	1962	11119	Mollusca	7805	Bivalvia
Calabrian		1.806	0.781	DuBar	1962	11119	Mollusca	7805	Bivalvia
Calabrian		1.806	0.781	DuBar	1962	11119	Mollusca	7805	Bivalvia
Calabrian		1.806	0.781	DuBar	1962	11119	Mollusca	7805	Bivalvia
Calabrian		1.806	0.781	DuBar	1962	11119	Mollusca	7805	Bivalvia
Late Pleistocene		0.126	0.0117	DuBar	1962	11119	Mollusca	7805	Bivalvia
Late Pleistocene		0.126	0.0117	DuBar	1962	11119	Mollusca	7805	Bivalvia
Early Pleistocene		2.588	0.781	DuBar	1962	11119	Mollusca	7805	Bivalvia
Early Pleistocene		2.588	0.781	DuBar	1962	11119	Mollusca	7805	Bivalvia
Early Pleistocene		2.588	0.781	DuBar	1962	11119	Mollusca	7805	Bivalvia
Late Pleistocene		0.126	0.0117	DuBar	1962	11119	Mollusca	7805	Bivalvia
Pliocene	Pleistocen	5.333	0.0117	DuBar	1962	11119	Mollusca	7805	Bivalvia
Pliocene	Pleistocen	5.333	0.0117	DuBar	1962	11119	Mollusca	7805	Bivalvia
Pliocene	Pleistocen	5.333	0.0117	DuBar	1962	11119	Mollusca	7805	Bivalvia
Pliocene	Pleistocen	5.333	0.0117	DuBar	1962	11119	Mollusca	7805	Bivalvia
Late Pliocene		3.6	2.588	Woodring	1982	13359	Mollusca	7805	Bivalvia
Late Pliocene		3.6	2.588	Woodring	1982	13359	Mollusca	7805	Bivalvia

Late Pliocene	3.6	2.588	Mansfield	1932	17094	Mollusca	7805	Bivalvia
Late Pliocene	3.6	2.588	Mansfield	1932	17094	Mollusca	7805	Bivalvia
Late Pliocene	3.6	2.588	Mansfield	1932	17094	Mollusca	7805	Bivalvia
Late Pliocene	3.6	2.588	Mansfield	1932	17094	Mollusca	7805	Bivalvia
Late Pliocene	3.6	2.588	Mansfield	1932	17094	Mollusca	7805	Bivalvia
Late Pliocene	3.6	2.588	Mansfield	1932	17094	Mollusca	7805	Bivalvia
Late Pliocene	3.6	2.588	Mansfield	1932	17094	Mollusca	7805	Bivalvia
Early Pleistocene	2.588	0.781	Portell et a	1992	17634	Mollusca	7805	Bivalvia
Early Pleistocene	2.588	0.781	Portell et a	1992	17634	Mollusca	7805	Bivalvia
Early Pleistocene	2.588	0.781	Portell et a	1992	17634	Mollusca	7805	Bivalvia
Middle Pleistocene	0.781	0.126	Portell et a	1992	17634	Mollusca	7805	Bivalvia
Middle Pleistocene	0.781	0.126	Portell et a	1992	17634	Mollusca	7805	Bivalvia
Middle Pleistocene	0.781	0.126	Portell et a	1992	17634	Mollusca	7805	Bivalvia
Late Pleistocene	0.126	0.0117	Portell et a	1992	17634	Mollusca	7805	Bivalvia
Late Pleistocene	0.126	0.0117	Portell et a	1992	17634	Mollusca	7805	Bivalvia
Middle Pleistocene	0.781	0.126	McGinty	1970	19114	Mollusca	7805	Bivalvia
Middle Pleistocene	0.781	0.126	McGinty	1970	19114	Mollusca	7805	Bivalvia
Middle Pleistocene	0.781	0.126	McGinty	1970	19114	Mollusca	7805	Bivalvia
Middle Pleistocene	0.781	0.126	McGinty	1970	19114	Mollusca	7805	Bivalvia
Middle Pleistocene	0.781	0.126	McGinty	1970	19114	Mollusca	7805	Bivalvia
Middle Pleistocene	0.781	0.126	McGinty	1970	19114	Mollusca	7805	Bivalvia
Middle Pleistocene	0.781	0.126	McGinty	1970	19114	Mollusca	7805	Bivalvia
Middle Pleistocene	0.781	0.126	McGinty	1970	19114	Mollusca	7805	Bivalvia
Middle Pleistocene	0.781	0.126	McGinty	1970	19114	Mollusca	7805	Bivalvia
Late Pleistocene	0.126	0.0117	Hendy et a	2008	19909	Mollusca	7805	Bivalvia
Late Pleistocene	0.126	0.0117	Hendy et a	2008	19909	Mollusca	7805	Bivalvia
Late Pleistocene	0.126	0.0117	Hendy et a	2008	19909	Mollusca	7805	Bivalvia
Late Pleistocene	0.126	0.0117	Hendy et a	2008	19909	Mollusca	7805	Bivalvia
Late Pleistocene	0.126	0.0117	Hendy et a	2008	19909	Mollusca	7805	Bivalvia
Late Pleistocene	0.126	0.0117	Hendy et a	2008	19909	Mollusca	7805	Bivalvia
Pliocene	5.333	2.588	Schroeder	1954	25303	Mollusca	7805	Bivalvia
Early Pleistocene	2.588	0.781	Schroeder	1954	25303	Mollusca	7805	Bivalvia
Pliocene	5.333	2.588	Schroeder	1954	25303	Mollusca	7805	Bivalvia
Pliocene	5.333	2.588	Schroeder	1954	25303	Mollusca	7805	Bivalvia
Pliocene	5.333	2.588	Schroeder	1954	25303	Mollusca	7805	Bivalvia
Early Pleistocene	2.588	0.781	Olsson anc	1953	25463	Mollusca	7805	Bivalvia
Early Pleistocene	2.588	0.781	Olsson anc	1953	25463	Mollusca	7805	Bivalvia
Early Pleistocene	2.588	0.781	Olsson anc	1953	25463	Mollusca	7805	Bivalvia
Early Pleistocene	2.588	0.781	Olsson anc	1953	25463	Mollusca	7805	Bivalvia
Early Pleistocene	2.588	0.781	Olsson anc	1953	25463	Mollusca	7805	Bivalvia
Early Pleistocene	2.588	0.781	Olsson anc	1953	25463	Mollusca	7805	Bivalvia
Early Pleistocene	2.588	0.781	Olsson anc	1953	25463	Mollusca	7805	Bivalvia
Early Pleistocene	2.588	0.781	Olsson anc	1953	25463	Mollusca	7805	Bivalvia
Early Pleistocene	2.588	0.781	Olsson anc	1953	25463	Mollusca	7805	Bivalvia
Early Pleistocene	2.588	0.781	Olsson anc	1953	25463	Mollusca	7805	Bivalvia
Early Pleistocene	2.588	0.781	Olsson anc	1953	25463	Mollusca	7805	Bivalvia
Early Pleistocene	2.588	0.781	Olsson anc	1953	25463	Mollusca	7805	Bivalvia

Early Pleistocene	2.588	0.781	Olsson anc	1953	25463	Mollusca	7805	Bivalvia
Early Pleistocene	2.588	0.781	Olsson anc	1953	25463	Mollusca	7805	Bivalvia
Late Pleistocene	0.126	0.0117	Lovejoy	1992	26806	Mollusca	7805	Bivalvia
Early Pleistocene	2.588	0.781	Petuch anc	2007	26813	Mollusca	7805	Bivalvia
Late Pliocene	3.6	2.588	Olsson	1967	19826	Mollusca	7805	Bivalvia
Late Pliocene	3.6	2.588	Olsson	1967	19826	Mollusca	7805	Bivalvia
Late Pliocene	3.6	2.588	Olsson	1967	19826	Mollusca	7805	Bivalvia
Late Pliocene	3.6	2.588	Olsson	1967	19826	Mollusca	7805	Bivalvia
Middle Pleistocene	0.781	0.126	Olsson anc	1964	26836	Mollusca	7805	Bivalvia
Early Pleistocene	2.588	0.781	Hendy et a	2008	19909	Mollusca	7805	Bivalvia
Early Pleistocene	2.588	0.781	Hendy et a	2008	19909	Mollusca	7805	Bivalvia
Early Pleistocene	2.588	0.781	Hendy et a	2008	19909	Mollusca	7805	Bivalvia
Early Pleistocene	2.588	0.781	Hendy et a	2008	19909	Mollusca	7805	Bivalvia
Late Pleistocene	0.126	0.0117	Hendy et a	2008	19909	Mollusca	7805	Bivalvia
Late Pleistocene	0.126	0.0117	Hendy et a	2008	19909	Mollusca	7805	Bivalvia
Late Pleistocene	0.126	0.0117	Hendy et a	2008	19909	Mollusca	7805	Bivalvia
Early Pleistocene	2.588	0.781	DuBar	1958	25514	Mollusca	7805	Bivalvia
Late Pleistocene	0.126	0.0117	DuBar	1958	25514	Mollusca	7805	Bivalvia
Late Pleistocene	0.126	0.0117	DuBar	1958	25514	Mollusca	7805	Bivalvia
Late Pleistocene	0.126	0.0117	Hendy et a	2008	19909	Mollusca	7805	Bivalvia
Late Pliocene	3.6	2.588	Hendy et a	2008	19909	Mollusca	7805	Bivalvia
Early Pleistocene	2.588	0.781	Hendy et a	2008	19909	Mollusca	7805	Bivalvia
Early Pleistocene	2.588	0.781	Hendy et a	2008	19909	Mollusca	7805	Bivalvia
Early Pleistocene	2.588	0.781	Hendy et a	2008	19909	Mollusca	7805	Bivalvia
Early Pleistocene	2.588	0.781	Hendy et a	2008	19909	Mollusca	7805	Bivalvia
Early Pleistocene	2.588	0.781	Hendy et a	2008	19909	Mollusca	7805	Bivalvia
Pleistocene	2.588	0.0117	Hendy et a	2008	19909	Mollusca	7805	Bivalvia
Late Pliocene	3.6	2.588	Hendy et a	2008	19909	Mollusca	7805	Bivalvia
Late Pliocene	3.6	2.588	Hendy et a	2008	19909	Mollusca	7805	Bivalvia
Middle Pleistocene	0.781	0.126	Hendy et a	2008	19909	Mollusca	7805	Bivalvia
Middle Pleistocene	0.781	0.126	Hendy et a	2008	19909	Mollusca	7805	Bivalvia
Middle Pleistocene	0.781	0.126	Hendy et a	2008	19909	Mollusca	7805	Bivalvia
Late Pleistocene	0.126	0.0117	Hendy et a	2008	19909	Mollusca	7805	Bivalvia
Late Pleistocene	0.126	0.0117	Hendy et a	2008	19909	Mollusca	7805	Bivalvia
Early Pleistocene	2.588	0.781	Hendy et a	2008	19909	Mollusca	7805	Bivalvia
Early Pleistocene	2.588	0.781	Hendy et a	2008	19909	Mollusca	7805	Bivalvia
Late Pliocene	3.6	2.588	Vokes	1984	31139	Mollusca	7805	Bivalvia
Late Pliocene	3.6	2.588	Weisbord	1971	39743	Mollusca	7805	Bivalvia
Late Pleistocene	0.126	0.0117	Kittle and I	2010	40884	Mollusca	7805	Bivalvia
Late Pleistocene	0.126	0.0117	Kittle and I	2010	40884	Mollusca	7805	Bivalvia
Late Pleistocene	0.126	0.0117	Kittle and I	2010	40884	Mollusca	7805	Bivalvia
Late Pleistocene	0.126	0.0117	Kittle and I	2010	40884	Mollusca	7805	Bivalvia
Late Pleistocene	0.126	0.0117	Kittle and I	2010	40884	Mollusca	7805	Bivalvia
Late Pleistocene	0.126	0.0117	DuBar	1971	17556	Mollusca	7805	Bivalvia
Zanclean	5.333	3.6	Campbell a	1995	17558	Mollusca	7805	Bivalvia
Zanclean	5.333	3.6	Campbell a	1995	17558	Mollusca	7805	Bivalvia
Early Pleistocene	2.588	0.781	DuBar	1971	17556	Mollusca	7805	Bivalvia

Early Pleistocene		2.588	0.781	DuBar	1971	17556	Mollusca	7805	Bivalvia
Late Pleistocene		0.126	0.0117	Richards	1962	18974	Mollusca	7805	Bivalvia
Late Pleistocene		0.126	0.0117	Richards	1962	18974	Mollusca	7805	Bivalvia
Late Pleistocene		0.126	0.0117	Richards	1962	18974	Mollusca	7805	Bivalvia
Late Pleistocene		0.126	0.0117	Richards	1962	18974	Mollusca	7805	Bivalvia
Late Pleistocene		0.126	0.0117	Richards	1962	18974	Mollusca	7805	Bivalvia
Late Pleistocene		0.126	0.0117	Richards	1962	18974	Mollusca	7805	Bivalvia
Late Pleistocene		0.126	0.0117	Richards	1962	18974	Mollusca	7805	Bivalvia
Late Pleistocene		0.126	0.0117	Richards	1962	18974	Mollusca	7805	Bivalvia
Late Pleistocene		0.126	0.0117	Hendy et a	2008	19909	Mollusca	7805	Bivalvia
Late Pleistocene		0.126	0.0117	Hendy et a	2008	19909	Mollusca	7805	Bivalvia
Late Pleistocene		0.126	0.0117	Hendy et a	2008	19909	Mollusca	7805	Bivalvia
Early Pleistocene		2.588	0.781	Cooke	1936	31145	Mollusca	7805	Bivalvia
Late Pliocene		3.6	2.588	Cooke	1936	31145	Mollusca	7805	Bivalvia
Pleistocene		2.588	0.0117	Holmes	1860	64712	Mollusca	7805	Bivalvia
Pleistocene		2.588	0.0117	Holmes	1860	64712	Mollusca	7805	Bivalvia
Pleistocen	Holocene	2.588	0	Holmes	1860	64712	Mollusca	7805	Bivalvia
Late Pliocene		3.6	2.588	Ward and I	1987	16700	Mollusca	7805	Bivalvia
Early Pleistocene		2.588	0.781	Ward and I	1987	16700	Mollusca	7805	Bivalvia
Early Plioc	Late Plioce	5.333	2.588	Gardner	1943	16984	Mollusca	7805	Bivalvia
Early Plioc	Late Plioce	5.333	2.588	Gardner	1943	16984	Mollusca	7805	Bivalvia
Late Pliocene		3.6	2.588	Gardner	1943	16984	Mollusca	7805	Bivalvia
Early Pleistocene		2.588	0.781	Gardner	1943	16984	Mollusca	7805	Bivalvia
Pleistocene		2.588	0.0117	Gardner	1943	16984	Mollusca	7805	Bivalvia
Late Pliocene		3.6	2.588	Gardner	1943	16984	Mollusca	7805	Bivalvia
Early Pleistocene		2.588	0.781	Gardner	1943	16984	Mollusca	7805	Bivalvia
Early Pleistocene		2.588	0.781	Gardner	1943	16984	Mollusca	7805	Bivalvia
Early Pleistocene		2.588	0.781	Gardner	1943	16984	Mollusca	7805	Bivalvia
Early Pleistocene		2.588	0.781	Gardner	1943	16984	Mollusca	7805	Bivalvia
Early Pleistocene		2.588	0.781	Gardner	1943	16984	Mollusca	7805	Bivalvia
Pleistocene		2.588	0.0117	Gardner	1943	16984	Mollusca	7805	Bivalvia
Late Pliocene		3.6	2.588	Gardner	1943	16984	Mollusca	7805	Bivalvia
Late Pleistocene		0.126	0.0117	Richards	1962	18974	Mollusca	7805	Bivalvia
Late Pleistocene		0.126	0.0117	Richards	1962	18974	Mollusca	7805	Bivalvia
Late Pleistocene		0.126	0.0117	Richards	1962	18974	Mollusca	7805	Bivalvia
Late Pleistocene		0.126	0.0117	Richards	1962	18974	Mollusca	7805	Bivalvia
Late Pleistocene		0.126	0.0117	Richards	1962	18974	Mollusca	7805	Bivalvia
Late Pleistocene		0.126	0.0117	Richards	1962	18974	Mollusca	7805	Bivalvia
Late Pleistocene		0.126	0.0117	Richards	1962	18974	Mollusca	7805	Bivalvia
Late Pleistocene		0.126	0.0117	Richards	1962	18974	Mollusca	7805	Bivalvia
Late Pleistocene		0.126	0.0117	Richards	1962	18974	Mollusca	7805	Bivalvia
Late Pleistocene		0.126	0.0117	Hendy et a	2008	19909	Mollusca	7805	Bivalvia
Late Pleistocene		0.126	0.0117	Hendy et a	2008	19909	Mollusca	7805	Bivalvia
Late Pliocene		3.6	2.588	Hendy et a	2008	19909	Mollusca	7805	Bivalvia
Late Pliocene		3.6	2.588	Hendy et a	2008	19909	Mollusca	7805	Bivalvia
Late Pliocene		3.6	2.588	Hendy et a	2008	19909	Mollusca	7805	Bivalvia
Early Pleistocene		2.588	0.781	Hendy et a	2008	19909	Mollusca	7805	Bivalvia

Early Pleistocene		2.588	0.781	Hendy et a	2008	19909	Mollusca	7805	Bivalvia
Early Pleistocene		2.588	0.781	Hendy et a	2008	19909	Mollusca	7805	Bivalvia
Early Pleistocene		2.588	0.781	Hendy et a	2008	19909	Mollusca	7805	Bivalvia
Early Pleistocene		2.588	0.781	Hendy et a	2008	19909	Mollusca	7805	Bivalvia
Early Pleistocene		2.588	0.781	Hendy et a	2008	19909	Mollusca	7805	Bivalvia
Early Pleistocene		2.588	0.781	Hendy et a	2008	19909	Mollusca	7805	Bivalvia
Early Pleistocene		2.588	0.781	Hendy et a	2008	19909	Mollusca	7805	Bivalvia
Early Pleistocene		2.588	0.781	Hendy et a	2008	19909	Mollusca	7805	Bivalvia
Early Pleistocene		2.588	0.781	Hendy et a	2008	19909	Mollusca	7805	Bivalvia
Early Pleistocene		2.588	0.781	Hendy et a	2008	19909	Mollusca	7805	Bivalvia
Early Pleistocene		2.588	0.781	Hendy et a	2008	19909	Mollusca	7805	Bivalvia
Middle Pleistocene		0.781	0.126	Miller	1985	30803	Mollusca	7805	Bivalvia
Pliocene		5.333	2.588	Ward	2007	48009	Mollusca	7805	Bivalvia
Late Pleistocene		0.126	0.0117	Spencer ar	1987	15525	Mollusca	7805	Bivalvia
Late Pleistocene		0.126	0.0117	Spencer ar	1987	15525	Mollusca	7805	Bivalvia
Late Pleistocene		0.126	0.0117	Spencer ar	1987	15525	Mollusca	7805	Bivalvia
Late Pliocene		3.6	2.588	Ward	1993	26351	Mollusca	7805	Bivalvia
Late Pliocene		3.6	2.588	Ward	1993	26351	Mollusca	7805	Bivalvia
Late Pliocene		3.6	2.588	Ward	1993	26351	Mollusca	7805	Bivalvia
Late Pliocene		3.6	2.588	Ward	1993	26351	Mollusca	7805	Bivalvia
Late Pliocene		3.6	2.588	Ward	1993	26351	Mollusca	7805	Bivalvia
Late Pliocene		3.6	2.588	Ward	1993	26351	Mollusca	7805	Bivalvia
Late Pliocene		3.6	2.588	Hendy et a	2008	19909	Mollusca	7805	Bivalvia
Late Pliocene		3.6	2.588	Hendy et a	2008	19909	Mollusca	7805	Bivalvia
Late Pliocene		3.6	2.588	Hendy et a	2008	19909	Mollusca	7805	Bivalvia
Late Pliocene		3.6	2.588	Hendy et a	2008	19909	Mollusca	7805	Bivalvia
Late Pleistocene		0.126	0.0117	Sellards	1917	9011	Mollusca	7805	Bivalvia
Late Pleistocene		0.126	0.0117	Sellards	1917	9011	Mollusca	7805	Bivalvia
Late Pleistocene		0.126	0.0117	Sellards	1917	9011	Mollusca	7805	Bivalvia
Early Pleistocene		2.588	0.781	Mansfield	1932	11083	Mollusca	7805	Bivalvia
Early Pleistocene		2.588	0.781	Mansfield	1932	11083	Mollusca	7805	Bivalvia
Early Pleistocene		2.588	0.781	DuBar	1962	11119	Mollusca	7805	Bivalvia
Early Pleistocene		2.588	0.781	DuBar	1962	11119	Mollusca	7805	Bivalvia
Early Pleistocene		2.588	0.781	DuBar	1962	11119	Mollusca	7805	Bivalvia
Early Pleistocene		2.588	0.781	DuBar	1962	11119	Mollusca	7805	Bivalvia
Early Pleistocene		2.588	0.781	DuBar	1962	11119	Mollusca	7805	Bivalvia
Calabrian		1.806	0.781	DuBar	1962	11119	Mollusca	7805	Bivalvia
Calabrian		1.806	0.781	DuBar	1962	11119	Mollusca	7805	Bivalvia
Calabrian		1.806	0.781	DuBar	1962	11119	Mollusca	7805	Bivalvia
Late Pleistocene		0.126	0.0117	DuBar	1962	11119	Mollusca	7805	Bivalvia
Early Pleistocene		2.588	0.781	DuBar	1962	11119	Mollusca	7805	Bivalvia
Early Pleistocene		2.588	0.781	DuBar	1962	11119	Mollusca	7805	Bivalvia
Early Pleistocene		2.588	0.781	DuBar	1962	11119	Mollusca	7805	Bivalvia
Late Pleistocene		0.126	0.0117	DuBar	1962	11119	Mollusca	7805	Bivalvia
Pliocene	Pleistocene	5.333	0.0117	DuBar	1962	11119	Mollusca	7805	Bivalvia
Pliocene	Pleistocene	5.333	0.0117	DuBar	1962	11119	Mollusca	7805	Bivalvia
Irvingtonian		1.8	0.3	Hulbert an	1989	1960	Mollusca	7805	Bivalvia

Late Pliocene	3.6	2.588	Mansfield	1932	17094	Mollusca	7805	Bivalvia
Late Pliocene	3.6	2.588	Mansfield	1932	17094	Mollusca	7805	Bivalvia
Late Pliocene	3.6	2.588	Mansfield	1932	17094	Mollusca	7805	Bivalvia
Late Pliocene	3.6	2.588	Mansfield	1932	17094	Mollusca	7805	Bivalvia
Late Pliocene	3.6	2.588	Mansfield	1932	17094	Mollusca	7805	Bivalvia
Late Pliocene	3.6	2.588	Mansfield	1932	17094	Mollusca	7805	Bivalvia
Late Pliocene	3.6	2.588	Mansfield	1932	17094	Mollusca	7805	Bivalvia
Late Pliocene	3.6	2.588	Mansfield	1932	17094	Mollusca	7805	Bivalvia
Late Pliocene	3.6	2.588	Mansfield	1932	17094	Mollusca	7805	Bivalvia
Late Pliocene	3.6	2.588	Mansfield	1932	17094	Mollusca	7805	Bivalvia
Late Pliocene	3.6	2.588	Mansfield	1932	17094	Mollusca	7805	Bivalvia
Late Pliocene	3.6	2.588	Mansfield	1932	17094	Mollusca	7805	Bivalvia
Late Pliocene	3.6	2.588	Mansfield	1932	17094	Mollusca	7805	Bivalvia
Late Pliocene	3.6	2.588	Mansfield	1932	17094	Mollusca	7805	Bivalvia
Late Pliocene	3.6	2.588	Mansfield	1932	17094	Mollusca	7805	Bivalvia
Late Pliocene	3.6	2.588	Mansfield	1932	17094	Mollusca	7805	Bivalvia
Late Pliocene	3.6	2.588	Mansfield	1932	17094	Mollusca	7805	Bivalvia
Early Pleistocene	2.588	0.781	Portell et a	1992	17634	Mollusca	7805	Bivalvia
Early Pleistocene	2.588	0.781	Portell et a	1992	17634	Mollusca	7805	Bivalvia
Early Pleistocene	2.588	0.781	Portell et a	1992	17634	Mollusca	7805	Bivalvia
Middle Pleistocene	0.781	0.126	Portell et a	1992	17634	Mollusca	7805	Bivalvia
Middle Pleistocene	0.781	0.126	Portell et a	1992	17634	Mollusca	7805	Bivalvia
Middle Pleistocene	0.781	0.126	Portell et a	1992	17634	Mollusca	7805	Bivalvia
Late Pleistocene	0.126	0.0117	Portell et a	1992	17634	Mollusca	7805	Bivalvia
Late Pleistocene	0.126	0.0117	Portell et a	1992	17634	Mollusca	7805	Bivalvia
Middle Pleistocene	0.781	0.126	McGinty	1970	19114	Mollusca	7805	Bivalvia
Middle Pleistocene	0.781	0.126	McGinty	1970	19114	Mollusca	7805	Bivalvia
Late Pleistocene	0.126	0.0117	Hendy et a	2008	19909	Mollusca	7805	Bivalvia
Late Pleistocene	0.126	0.0117	Hendy et a	2008	19909	Mollusca	7805	Bivalvia
Late Pleistocene	0.126	0.0117	Hendy et a	2008	19909	Mollusca	7805	Bivalvia
Early Pleistocene	2.588	0.781	Schroeder	1954	25303	Mollusca	7805	Bivalvia
Pliocene	5.333	2.588	Schroeder	1954	25303	Mollusca	7805	Bivalvia
Pliocene	5.333	2.588	Schroeder	1954	25303	Mollusca	7805	Bivalvia
Pliocene	5.333	2.588	Schroeder	1954	25303	Mollusca	7805	Bivalvia
Pliocene	5.333	2.588	Schroeder	1954	25303	Mollusca	7805	Bivalvia
Pliocene	5.333	2.588	Schroeder	1954	25303	Mollusca	7805	Bivalvia
Early Pleistocene	2.588	0.781	Schroeder	1954	25303	Mollusca	7805	Bivalvia
Pliocene	5.333	2.588	Schroeder	1954	25303	Mollusca	7805	Bivalvia
Pliocene	5.333	2.588	Schroeder	1954	25303	Mollusca	7805	Bivalvia
Pliocene	5.333	2.588	Schroeder	1954	25303	Mollusca	7805	Bivalvia
Pliocene	5.333	2.588	Schroeder	1954	25303	Mollusca	7805	Bivalvia
Pliocene	5.333	2.588	Schroeder	1954	25303	Mollusca	7805	Bivalvia
Pliocene	5.333	2.588	Schroeder	1954	25303	Mollusca	7805	Bivalvia
Pliocene	5.333	2.588	Schroeder	1954	25303	Mollusca	7805	Bivalvia
Pliocene	5.333	2.588	Schroeder	1954	25303	Mollusca	7805	Bivalvia
Early Pleistocene	2.588	0.781	Olsson anc	1953	25463	Mollusca	7805	Bivalvia
Early Pleistocene	2.588	0.781	Olsson anc	1953	25463	Mollusca	7805	Bivalvia

Early Pleistocene	2.588	0.781	Olsson anc	1953	25463	Mollusca	7805	Bivalvia
Early Pleistocene	2.588	0.781	Olsson anc	1953	25463	Mollusca	7805	Bivalvia
Late Pliocene	3.6	2.588	Olsson	1967	19826	Mollusca	7805	Bivalvia
Late Pliocene	3.6	2.588	Olsson	1967	19826	Mollusca	7805	Bivalvia
Late Pliocene	3.6	2.588	Olsson	1967	19826	Mollusca	7805	Bivalvia
Late Pliocene	3.6	2.588	Olsson	1967	19826	Mollusca	7805	Bivalvia
Late Pliocene	3.6	2.588	DuBar	1958	25514	Mollusca	7805	Bivalvia
Late Plioce Pleistocen	3.6	0.0117	Petuch anc	2007	26813	Mollusca	7805	Bivalvia
Early Pleistocene	2.588	0.781	Hendy et a	2008	19909	Mollusca	7805	Bivalvia
Early Pleistocene	2.588	0.781	Hendy et a	2008	19909	Mollusca	7805	Bivalvia
Late Pleistocene	0.126	0.0117	Hendy et a	2008	19909	Mollusca	7805	Bivalvia
Late Pleistocene	0.126	0.0117	Hendy et a	2008	19909	Mollusca	7805	Bivalvia
Late Pleistocene	0.126	0.0117	Hendy et a	2008	19909	Mollusca	7805	Bivalvia
Late Pliocene	3.6	2.588	Petuch anc	2007	26813	Mollusca	7805	Bivalvia
Late Pliocene	3.6	2.588	Petuch anc	2007	26813	Mollusca	7805	Bivalvia
Late Pliocene	3.6	2.588	Petuch anc	2007	26813	Mollusca	7805	Bivalvia
Late Pliocene	3.6	2.588	Petuch anc	2007	26813	Mollusca	7805	Bivalvia
Early Pleistocene	2.588	0.781	DuBar	1958	25514	Mollusca	7805	Bivalvia
Late Pleistocene	0.126	0.0117	DuBar	1958	25514	Mollusca	7805	Bivalvia
Late Pleistocene	0.126	0.0117	Hendy et a	2008	19909	Mollusca	7805	Bivalvia
Late Pliocene	3.6	2.588	Hendy et a	2008	19909	Mollusca	7805	Bivalvia
Late Pliocene	3.6	2.588	Hendy et a	2008	19909	Mollusca	7805	Bivalvia
Early Pleistocene	2.588	0.781	Hendy et a	2008	19909	Mollusca	7805	Bivalvia
Early Pleistocene	2.588	0.781	Hendy et a	2008	19909	Mollusca	7805	Bivalvia
Early Pleistocene	2.588	0.781	Hendy et a	2008	19909	Mollusca	7805	Bivalvia
Early Pleistocene	2.588	0.781	Hendy et a	2008	19909	Mollusca	7805	Bivalvia
Early Pleistocene	2.588	0.781	Hendy et a	2008	19909	Mollusca	7805	Bivalvia
Late Pliocene	3.6	2.588	Hendy et a	2008	19909	Mollusca	7805	Bivalvia
Late Pliocene	3.6	2.588	Hendy et a	2008	19909	Mollusca	7805	Bivalvia
Late Pliocene	3.6	2.588	Hendy et a	2008	19909	Mollusca	7805	Bivalvia
Late Pliocene	3.6	2.588	Hendy et a	2008	19909	Mollusca	7805	Bivalvia
Late Pliocene	3.6	2.588	Hendy et a	2008	19909	Mollusca	7805	Bivalvia
Late Pleistocene	0.126	0.0117	Hendy et a	2008	19909	Mollusca	7805	Bivalvia
Late Pleistocene	0.126	0.0117	Hendy et a	2008	19909	Mollusca	7805	Bivalvia
Early Pleistocene	2.588	0.781	Hendy et a	2008	19909	Mollusca	7805	Bivalvia
Early Pleistocene	2.588	0.781	Hendy et a	2008	19909	Mollusca	7805	Bivalvia
Early Pleistocene	2.588	0.781	DuBar	1958	25514	Mollusca	7805	Bivalvia
Early Pleistocene	2.588	0.781	DuBar	1958	25514	Mollusca	7805	Bivalvia
Late Pleistocene	0.126	0.0117	Kittle and I	2010	40884	Mollusca	7805	Bivalvia
Late Pleistocene	0.126	0.0117	Kittle and I	2010	40884	Mollusca	7805	Bivalvia
Late Pleistocene	0.126	0.0117	Kittle and I	2010	40884	Mollusca	7805	Bivalvia
Late Pleistocene	0.126	0.0117	Hulbert an	1998	1962	Mollusca	7805	Bivalvia
Late Pleistocene	0.126	0.0117	Hulbert an	1998	1962	Mollusca	7805	Bivalvia
Late Pleistocene	0.126	0.0117	DuBar	1971	17556	Mollusca	7805	Bivalvia
Pliocene	5.333	2.588	DuBar	1971	17556	Mollusca	7805	Bivalvia
Pliocene	5.333	2.588	DuBar	1971	17556	Mollusca	7805	Bivalvia
Late Pleistocene	0.126	0.0117	DuBar	1971	17556	Mollusca	7805	Bivalvia

Late Pleistocene	0.126	0.0117	DuBar	1971	17556	Mollusca	7805	Bivalvia
Late Pleistocene	0.126	0.0117	DuBar	1971	17556	Mollusca	7805	Bivalvia
Late Pleistocene	0.126	0.0117	DuBar	1971	17556	Mollusca	7805	Bivalvia
Late Pleistocene	0.126	0.0117	DuBar	1971	17556	Mollusca	7805	Bivalvia
Late Pleistocene	0.126	0.0117	DuBar	1971	17556	Mollusca	7805	Bivalvia
Late Pleistocene	0.126	0.0117	DuBar	1971	17556	Mollusca	7805	Bivalvia
Pliocene	5.333	2.588	DuBar	1971	17556	Mollusca	7805	Bivalvia
Zanclean	5.333	3.6	Campbell a	1995	17558	Mollusca	7805	Bivalvia
Early Pleistocene	2.588	0.781	DuBar	1971	17556	Mollusca	7805	Bivalvia
Early Pleistocene	2.588	0.781	DuBar	1971	17556	Mollusca	7805	Bivalvia
Late Pleistocene	0.126	0.0117	Richards	1962	18974	Mollusca	7805	Bivalvia
Late Pleistocene	0.126	0.0117	Richards	1962	18974	Mollusca	7805	Bivalvia
Late Pleistocene	0.126	0.0117	Richards	1962	18974	Mollusca	7805	Bivalvia
Late Pleistocene	0.126	0.0117	Hendy et a	2008	19909	Mollusca	7805	Bivalvia
Late Pleistocene	0.126	0.0117	Hendy et a	2008	19909	Mollusca	7805	Bivalvia
Pleistocene	2.588	0.0117	Hendy et a	2008	19909	Mollusca	7805	Bivalvia
Pleistocene	2.588	0.0117	Hendy et a	2008	19909	Mollusca	7805	Bivalvia
Early Pleistocene	2.588	0.781	Cooke	1936	31145	Mollusca	7805	Bivalvia
Early Pleistocene	2.588	0.781	Cooke	1936	31145	Mollusca	7805	Bivalvia
Early Pleistocene	2.588	0.781	Cooke	1936	31145	Mollusca	7805	Bivalvia
Early Pleistocene	2.588	0.781	Cooke	1936	31145	Mollusca	7805	Bivalvia
Early Pleistocene	2.588	0.781	Cooke	1936	31145	Mollusca	7805	Bivalvia
Early Pleistocene	2.588	0.781	Cooke	1936	31145	Mollusca	7805	Bivalvia
Early Pleistocene	2.588	0.781	Cooke	1936	31145	Mollusca	7805	Bivalvia
Early Pleistocene	2.588	0.781	Cooke	1936	31145	Mollusca	7805	Bivalvia
Late Pliocene	3.6	2.588	Cooke	1936	31145	Mollusca	7805	Bivalvia
Late Pliocene	3.6	2.588	Cooke	1936	31145	Mollusca	7805	Bivalvia
Pleistocene	2.588	0.0117	Holmes	1860	64712	Mollusca	7805	Bivalvia
Late Pliocene	3.6	2.588	Ward and I	1987	16700	Mollusca	7805	Bivalvia
Late Pliocene	3.6	2.588	Ward and I	1987	16700	Mollusca	7805	Bivalvia
Late Pliocene	3.6	2.588	Ward and I	1987	16700	Mollusca	7805	Bivalvia
Late Pliocene	3.6	2.588	Ward and I	1987	16700	Mollusca	7805	Bivalvia
Early Pleistocene	2.588	0.781	Ward and I	1987	16700	Mollusca	7805	Bivalvia
Early Pleistocene	2.588	0.781	Ward and I	1987	16700	Mollusca	7805	Bivalvia
Early Pleistocene	2.588	0.781	Ward and I	1987	16700	Mollusca	7805	Bivalvia
Early Pleistocene	2.588	0.781	Ward and I	1987	16700	Mollusca	7805	Bivalvia
Late Pliocene	3.6	2.588	Ward and I	1987	16700	Mollusca	7805	Bivalvia
Late Pliocene	3.6	2.588	Ward and I	1987	16700	Mollusca	7805	Bivalvia
Late Pliocene	3.6	2.588	Ward and I	1987	16700	Mollusca	7805	Bivalvia
Late Pliocene	3.6	2.588	Ward and I	1987	16700	Mollusca	7805	Bivalvia
Late Pliocene	3.6	2.588	Ward and I	1987	16700	Mollusca	7805	Bivalvia
Late Pliocene	3.6	2.588	Ward and I	1987	16700	Mollusca	7805	Bivalvia
Late Pliocene	3.6	2.588	Ward and I	1987	16700	Mollusca	7805	Bivalvia
Late Pliocene	3.6	2.588	Ward and I	1987	16700	Mollusca	7805	Bivalvia
Early Pleistocene	2.588	0.781	Ward and I	1987	16700	Mollusca	7805	Bivalvia
Early Pleistocene	2.588	0.781	Ward and I	1987	16700	Mollusca	7805	Bivalvia
Early Pleistocene	2.588	0.781	Ward and I	1987	16700	Mollusca	7805	Bivalvia

Early Pleistocene		2.588	0.781	Ward and I	1987	16700	Mollusca	7805	Bivalvia
Early Pleistocene		2.588	0.781	Ward and I	1987	16700	Mollusca	7805	Bivalvia
Early Plioce	Late Plioce	5.333	2.588	Gardner	1943	16984	Mollusca	7805	Bivalvia
Early Pleistocene		2.588	0.781	Gardner	1943	16984	Mollusca	7805	Bivalvia
Early Pleistocene		2.588	0.781	Gardner	1943	16984	Mollusca	7805	Bivalvia
Early Plioce	Late Plioce	5.333	2.588	Gardner	1943	16984	Mollusca	7805	Bivalvia
Late Pliocene		3.6	2.588	Gardner	1943	16984	Mollusca	7805	Bivalvia
Early Pleistocene		2.588	0.781	Gardner	1943	16984	Mollusca	7805	Bivalvia
Late Pliocene		3.6	2.588	Gardner	1943	16984	Mollusca	7805	Bivalvia
Late Pliocene		3.6	2.588	Gardner	1943	16984	Mollusca	7805	Bivalvia
Late Pliocene		3.6	2.588	Gardner	1943	16984	Mollusca	7805	Bivalvia
Pleistocene		2.588	0.0117	Gardner	1943	16984	Mollusca	7805	Bivalvia
Early Pleistocene		2.588	0.781	Gardner	1943	16984	Mollusca	7805	Bivalvia
Late Pliocene		3.6	2.588	Gardner	1943	16984	Mollusca	7805	Bivalvia
Early Pleistocene		2.588	0.781	Gardner	1943	16984	Mollusca	7805	Bivalvia
Late Pliocene		3.6	2.588	Gardner	1943	16984	Mollusca	7805	Bivalvia
Early Plioce	Late Plioce	5.333	2.588	Gardner	1943	16984	Mollusca	7805	Bivalvia
Late Pliocene		3.6	2.588	Gardner	1943	16984	Mollusca	7805	Bivalvia
Late Pliocene		3.6	2.588	Gardner	1943	16984	Mollusca	7805	Bivalvia
Pleistocene		2.588	0.0117	Gardner	1943	16984	Mollusca	7805	Bivalvia
Early Pleistocene		2.588	0.781	Gardner	1943	16984	Mollusca	7805	Bivalvia
Early Pleistocene		2.588	0.781	Gardner	1943	16984	Mollusca	7805	Bivalvia
Early Pleistocene		2.588	0.781	Gardner	1943	16984	Mollusca	7805	Bivalvia
Late Pliocene		3.6	2.588	Gardner	1943	16984	Mollusca	7805	Bivalvia
Early Pleistocene		2.588	0.781	DuBar	1971	17556	Mollusca	7805	Bivalvia
Late Pleistocene		0.126	0.0117	Richards	1962	18974	Mollusca	7805	Bivalvia
Late Pleistocene		0.126	0.0117	Richards	1962	18974	Mollusca	7805	Bivalvia
Late Pleistocene		0.126	0.0117	Richards	1962	18974	Mollusca	7805	Bivalvia
Late Pleistocene		0.126	0.0117	Richards	1962	18974	Mollusca	7805	Bivalvia
Late Pleistocene		0.126	0.0117	Richards	1962	18974	Mollusca	7805	Bivalvia
Late Pleistocene		0.126	0.0117	Richards	1962	18974	Mollusca	7805	Bivalvia
Late Pliocene		3.6	2.588	Hendy et a	2008	19909	Mollusca	7805	Bivalvia
Late Pliocene		3.6	2.588	Hendy et a	2008	19909	Mollusca	7805	Bivalvia
Late Pleistocene		0.126	0.0117	Hendy et a	2008	19909	Mollusca	7805	Bivalvia
Gelasian		2.588	1.806	Bailey and	1986	23840	Mollusca	7805	Bivalvia
Early Pleistocene		2.588	0.781	Hendy et a	2008	19909	Mollusca	7805	Bivalvia
Late Pliocene		3.6	2.588	Hendy et a	2008	19909	Mollusca	7805	Bivalvia
Late Pliocene		3.6	2.588	Hendy et a	2008	19909	Mollusca	7805	Bivalvia
Late Pliocene		3.6	2.588	Hendy et a	2008	19909	Mollusca	7805	Bivalvia
Late Pliocene		3.6	2.588	Hendy et a	2008	19909	Mollusca	7805	Bivalvia
Late Pliocene		3.6	2.588	Hendy et a	2008	19909	Mollusca	7805	Bivalvia
Late Pliocene		3.6	2.588	Hendy et a	2008	19909	Mollusca	7805	Bivalvia
Pliocene		5.333	2.588	Hendy et a	2008	19909	Mollusca	7805	Bivalvia
Pliocene		5.333	2.588	Hendy et a	2008	19909	Mollusca	7805	Bivalvia
Pliocene		5.333	2.588	Hendy et a	2008	19909	Mollusca	7805	Bivalvia
Pliocene		5.333	2.588	Hendy et a	2008	19909	Mollusca	7805	Bivalvia
Pliocene		5.333	2.588	Hendy et a	2008	19909	Mollusca	7805	Bivalvia

Pliocene	5.333	2.588	Hendy et a	2008	19909	Mollusca	7805	Bivalvia
Late Pliocene	3.6	2.588	Hendy et a	2008	19909	Mollusca	7805	Bivalvia
Late Pliocene	3.6	2.588	Hendy et a	2008	19909	Mollusca	7805	Bivalvia
Late Pliocene	3.6	2.588	Hendy et a	2008	19909	Mollusca	7805	Bivalvia
Late Pliocene	3.6	2.588	Hendy et a	2008	19909	Mollusca	7805	Bivalvia
Late Pliocene	3.6	2.588	Hendy et a	2008	19909	Mollusca	7805	Bivalvia
Late Pliocene	3.6	2.588	Hendy et a	2008	19909	Mollusca	7805	Bivalvia
Late Pliocene	3.6	2.588	Hendy et a	2008	19909	Mollusca	7805	Bivalvia
Early Pleistocene	2.588	0.781	Hendy et a	2008	19909	Mollusca	7805	Bivalvia
Early Pleistocene	2.588	0.781	Hendy et a	2008	19909	Mollusca	7805	Bivalvia
Early Pleistocene	2.588	0.781	Hendy et a	2008	19909	Mollusca	7805	Bivalvia
Early Pleistocene	2.588	0.781	Hendy et a	2008	19909	Mollusca	7805	Bivalvia
Late Pliocene	3.6	2.588	Hendy et a	2008	19909	Mollusca	7805	Bivalvia
Early Pleistocene	2.588	0.781	Hendy et a	2008	19909	Mollusca	7805	Bivalvia
Early Pleistocene	2.588	0.781	Hendy et a	2008	19909	Mollusca	7805	Bivalvia
Early Pleistocene	2.588	0.781	Hendy et a	2008	19909	Mollusca	7805	Bivalvia
Early Pleistocene	2.588	0.781	Hendy et a	2008	19909	Mollusca	7805	Bivalvia
Early Pleistocene	2.588	0.781	Hendy et a	2008	19909	Mollusca	7805	Bivalvia
Early Pleistocene	2.588	0.781	Hendy et a	2008	19909	Mollusca	7805	Bivalvia
Early Pleistocene	2.588	0.781	Hendy et a	2008	19909	Mollusca	7805	Bivalvia
Early Pleistocene	2.588	0.781	Hendy et a	2008	19909	Mollusca	7805	Bivalvia
Early Pleistocene	2.588	0.781	Hendy et a	2008	19909	Mollusca	7805	Bivalvia
Early Pleistocene	2.588	0.781	Hendy et a	2008	19909	Mollusca	7805	Bivalvia
Pliocene	5.333	2.588	Ward	2007	48009	Mollusca	7805	Bivalvia
Pleistocene	2.588	0.0117	Coch	1971	441	Mollusca	7805	Bivalvia
Late Pleistocene	0.126	0.0117	Spencer ar	1987	15525	Mollusca	7805	Bivalvia
Late Pleistocene	0.126	0.0117	Spencer ar	1987	15525	Mollusca	7805	Bivalvia
Early Pliocene	5.333	3.6	Ward	1992	16985	Mollusca	7805	Bivalvia
Pliocene	5.333	2.588	Gardner	1943	16984	Mollusca	7805	Bivalvia
Late Pliocene	3.6	2.588	Edwards et	2005	17631	Mollusca	7805	Bivalvia
Late Pleistocene	0.126	0.0117	Hendy et a	2008	19909	Mollusca	7805	Bivalvia
Zanclean	5.333	3.6	Ward	1992	16985	Mollusca	7805	Bivalvia
Zanclean	5.333	3.6	Ward	1992	16985	Mollusca	7805	Bivalvia
Late Pliocene	3.6	2.588	Ward	1992	16985	Mollusca	7805	Bivalvia
Late Pliocene	3.6	2.588	Ward	1993	26351	Mollusca	7805	Bivalvia
Late Pliocene	3.6	2.588	Ward	1993	26351	Mollusca	7805	Bivalvia
Late Pliocene	3.6	2.588	Ward	1993	26351	Mollusca	7805	Bivalvia
Late Pliocene	3.6	2.588	Ward	1993	26351	Mollusca	7805	Bivalvia
Late Pliocene	3.6	2.588	Ward	1993	26351	Mollusca	7805	Bivalvia
Late Pliocene	3.6	2.588	Ward	1993	26351	Mollusca	7805	Bivalvia
Late Pliocene	3.6	2.588	Ward	1993	26351	Mollusca	7805	Bivalvia
Late Pliocene	3.6	2.588	Ward	1993	26351	Mollusca	7805	Bivalvia
Late Pliocene	3.6	2.588	Ward	1993	26351	Mollusca	7805	Bivalvia
Late Pliocene	3.6	2.588	Ward	1993	26351	Mollusca	7805	Bivalvia
Late Pliocene	3.6	2.588	Ward	1993	26351	Mollusca	7805	Bivalvia
Late Pliocene	3.6	2.588	Ward	1993	26351	Mollusca	7805	Bivalvia
Late Pliocene	3.6	2.588	Ward	1993	26351	Mollusca	7805	Bivalvia

Late Pliocene		3.6	2.588	Hendy et a	2008	19909	Mollusca	7805	Bivalvia
Late Pliocene		3.6	2.588	Hendy et a	2008	19909	Mollusca	7805	Bivalvia
Late Pliocene		3.6	2.588	Hendy et a	2008	19909	Mollusca	7805	Bivalvia
Late Pliocene		3.6	2.588	Hendy et a	2008	19909	Mollusca	7805	Bivalvia
Late Pliocene		3.6	2.588	Hendy et a	2008	19909	Mollusca	7805	Bivalvia
Late Pliocene		3.6	2.588	Hendy et a	2008	19909	Mollusca	7805	Bivalvia
Late Pliocene		3.6	2.588	Hendy et a	2008	19909	Mollusca	7805	Bivalvia
Early Pleistocene		2.588	0.781	DuBar	1962	11119	Mollusca	7805	Bivalvia
Early Pleistocene		2.588	0.781	DuBar	1962	11119	Mollusca	7805	Bivalvia
Early Pleistocene		2.588	0.781	DuBar	1962	11119	Mollusca	7805	Bivalvia
Early Pleistocene		2.588	0.781	DuBar	1962	11119	Mollusca	7805	Bivalvia
Early Pleistocene		2.588	0.781	DuBar	1962	11119	Mollusca	7805	Bivalvia
Calabrian		1.806	0.781	DuBar	1962	11119	Mollusca	7805	Bivalvia
Calabrian		1.806	0.781	DuBar	1962	11119	Mollusca	7805	Bivalvia
Calabrian		1.806	0.781	DuBar	1962	11119	Mollusca	7805	Bivalvia
Calabrian		1.806	0.781	DuBar	1962	11119	Mollusca	7805	Bivalvia
Early Pleistocene		2.588	0.781	DuBar	1962	11119	Mollusca	7805	Bivalvia
Early Pleistocene		2.588	0.781	DuBar	1962	11119	Mollusca	7805	Bivalvia
Early Pleistocene		2.588	0.781	DuBar	1962	11119	Mollusca	7805	Bivalvia
Early Pleistocene		2.588	0.781	DuBar	1962	11119	Mollusca	7805	Bivalvia
Pliocene	Pleistocen	5.333	0.0117	DuBar	1962	11119	Mollusca	7805	Bivalvia
Pliocene	Pleistocen	5.333	0.0117	DuBar	1962	11119	Mollusca	7805	Bivalvia
Pliocene	Pleistocen	5.333	0.0117	DuBar	1962	11119	Mollusca	7805	Bivalvia
Pliocene	Pleistocen	5.333	0.0117	DuBar	1962	11119	Mollusca	7805	Bivalvia
Late Pliocene		3.6	2.588	Mansfield	1932	17094	Mollusca	7805	Bivalvia
Late Pliocene		3.6	2.588	Mansfield	1932	17094	Mollusca	7805	Bivalvia
Late Pliocene		3.6	2.588	Mansfield	1932	17094	Mollusca	7805	Bivalvia
Late Pliocene		3.6	2.588	Mansfield	1932	17094	Mollusca	7805	Bivalvia
Late Pliocene		3.6	2.588	Mansfield	1932	17094	Mollusca	7805	Bivalvia
Early Pleistocene		2.588	0.781	Portell et a	1992	17634	Mollusca	7805	Bivalvia
Early Pleistocene		2.588	0.781	Portell et a	1992	17634	Mollusca	7805	Bivalvia
Early Pleistocene		2.588	0.781	Portell et a	1992	17634	Mollusca	7805	Bivalvia
Middle Pleistocene		0.781	0.126	Portell et a	1992	17634	Mollusca	7805	Bivalvia
Middle Pleistocene		0.781	0.126	Portell et a	1992	17634	Mollusca	7805	Bivalvia
Late Pleistocene		0.126	0.0117	Portell et a	1992	17634	Mollusca	7805	Bivalvia
Middle Pleistocene		0.781	0.126	McGinty	1970	19114	Mollusca	7805	Bivalvia
Middle Pleistocene		0.781	0.126	McGinty	1970	19114	Mollusca	7805	Bivalvia
Middle Pleistocene		0.781	0.126	McGinty	1970	19114	Mollusca	7805	Bivalvia
Middle Pleistocene		0.781	0.126	McGinty	1970	19114	Mollusca	7805	Bivalvia
Early Pleistocene		2.588	0.781	Olsson anc	1953	25463	Mollusca	7805	Bivalvia
Early Pleistocene		2.588	0.781	Olsson anc	1953	25463	Mollusca	7805	Bivalvia
Early Pleistocene		2.588	0.781	Olsson anc	1953	25463	Mollusca	7805	Bivalvia
Early Pleistocene		2.588	0.781	Hendy et a	2008	19909	Mollusca	7805	Bivalvia
Early Pleistocene		2.588	0.781	Hendy et a	2008	19909	Mollusca	7805	Bivalvia
Early Pleistocene		2.588	0.781	Hendy et a	2008	19909	Mollusca	7805	Bivalvia
Late Pliocene		3.6	2.588	Hendy et a	2008	19909	Mollusca	7805	Bivalvia
Late Pliocene		3.6	2.588	Hendy et a	2008	19909	Mollusca	7805	Bivalvia

Late Pliocene		3.6	2.588	Hendy et a	2008	19909	Mollusca	7805 Bivalvia
Middle Pleistocene		0.781	0.126	Hendy et a	2008	19909	Mollusca	7805 Bivalvia
Middle Pleistocene		0.781	0.126	Hendy et a	2008	19909	Mollusca	7805 Bivalvia
Middle Pleistocene		0.781	0.126	Hendy et a	2008	19909	Mollusca	7805 Bivalvia
Late Pleistocene		0.126	0.0117	Hendy et a	2008	19909	Mollusca	7805 Bivalvia
Late Pleistocene		0.126	0.0117	Hendy et a	2008	19909	Mollusca	7805 Bivalvia
Early Pleistocene		2.588	0.781	Hendy et a	2008	19909	Mollusca	7805 Bivalvia
Early Pleistocene		2.588	0.781	Hendy et a	2008	19909	Mollusca	7805 Bivalvia
Late Pliocene		3.6	2.588	Smith	1941	32333	Mollusca	7805 Bivalvia
Late Pliocene		3.6	2.588	Weisbord	1971	39743	Mollusca	7805 Bivalvia
Late Pleistocene		0.126	0.0117	Kittle and I	2010	40884	Mollusca	7805 Bivalvia
Late Pleistocene		0.126	0.0117	Kittle and I	2010	40884	Mollusca	7805 Bivalvia
Pliocene		5.333	2.588	Petuch	1986	47440	Mollusca	7805 Bivalvia
Pliocene		5.333	2.588	Petuch	1986	47440	Mollusca	7805 Bivalvia
Late Pleistocene		0.126	0.0117	DuBar	1971	17556	Mollusca	7805 Bivalvia
Pliocene		5.333	2.588	DuBar	1971	17556	Mollusca	7805 Bivalvia
Zanclean		5.333	3.6	Campbell a	1995	17558	Mollusca	7805 Bivalvia
Zanclean		5.333	3.6	Campbell a	1995	17558	Mollusca	7805 Bivalvia
Late Pliocene		3.6	2.588	Campbell a	1995	17558	Mollusca	7805 Bivalvia
Early Pleistocene		2.588	0.781	DuBar	1971	17556	Mollusca	7805 Bivalvia
Late Pleistocene		0.126	0.0117	Richards	1962	18974	Mollusca	7805 Bivalvia
Late Pleistocene		0.126	0.0117	Hendy et a	2008	19909	Mollusca	7805 Bivalvia
Late Pleistocene		0.126	0.0117	Hendy et a	2008	19909	Mollusca	7805 Bivalvia
Pleistocene		2.588	0.0117	Hendy et a	2008	19909	Mollusca	7805 Bivalvia
Early Pleistocene		2.588	0.781	Cooke	1936	31145	Mollusca	7805 Bivalvia
Late Pliocene		3.6	2.588	Cooke	1936	31145	Mollusca	7805 Bivalvia
Late Pliocene		3.6	2.588	Cooke	1936	31145	Mollusca	7805 Bivalvia
Pleistocene		2.588	0.0117	Holmes	1860	64712	Mollusca	7805 Bivalvia
Early Pleistocene		2.588	0.781	Ward and I	1987	16700	Mollusca	7805 Bivalvia
Late Pliocene		3.6	2.588	Ward	1992	16985	Mollusca	7805 Bivalvia
Early Plioc	Late Plioce	5.333	2.588	Gardner	1943	16984	Mollusca	7805 Bivalvia
Late Pliocene		3.6	2.588	Gardner	1943	16984	Mollusca	7805 Bivalvia
Late Pliocene		3.6	2.588	Gardner	1943	16984	Mollusca	7805 Bivalvia
Early Pleistocene		2.588	0.781	DuBar	1971	17556	Mollusca	7805 Bivalvia
Late Pleistocene		0.126	0.0117	Richards	1962	18974	Mollusca	7805 Bivalvia
Late Pliocene		3.6	2.588	Hendy et a	2008	19909	Mollusca	7805 Bivalvia
Pliocene		5.333	2.588	Hendy et a	2008	19909	Mollusca	7805 Bivalvia
Early Pleistocene		2.588	0.781	Hendy et a	2008	19909	Mollusca	7805 Bivalvia
Early Pleistocene		2.588	0.781	Hendy et a	2008	19909	Mollusca	7805 Bivalvia
Early Pleistocene		2.588	0.781	Hendy et a	2008	19909	Mollusca	7805 Bivalvia
Early Pleistocene		2.588	0.781	Hendy et a	2008	19909	Mollusca	7805 Bivalvia
Early Pleistocene		2.588	0.781	Hendy et a	2008	19909	Mollusca	7805 Bivalvia
Early Pleistocene		2.588	0.781	Hendy et a	2008	19909	Mollusca	7805 Bivalvia
Late Pliocene		3.6	2.588	Ward	1985	17420	Mollusca	7805 Bivalvia
Late Pliocene		3.6	2.588	Ward	1992	16985	Mollusca	7805 Bivalvia
Late Pliocene		3.6	2.588	Ward	1993	26351	Mollusca	7805 Bivalvia
Late Pliocene		3.6	2.588	Ward	1993	26351	Mollusca	7805 Bivalvia

Late Pliocene		3.6	2.588	Ward	1993	26351	Mollusca	7805 Bivalvia
Late Pliocene		3.6	2.588	Ward	1993	26351	Mollusca	7805 Bivalvia
Late Pliocene		3.6	2.588	Ward	1993	26351	Mollusca	7805 Bivalvia
Late Pliocene		3.6	2.588	Hendy et a	2008	19909	Mollusca	7805 Bivalvia
Late Pliocene		3.6	2.588	Hendy et a	2008	19909	Mollusca	7805 Bivalvia
Late Pliocene		3.6	2.588	Hendy et a	2008	19909	Mollusca	7805 Bivalvia
Late Pliocene		3.6	2.588	Hendy et a	2008	19909	Mollusca	7805 Bivalvia
Late Pliocene		3.6	2.588	Hendy et a	2008	19909	Mollusca	7805 Bivalvia
Late Pliocene		3.6	2.588	Hendy et a	2008	19909	Mollusca	7805 Bivalvia
Late Pliocene		3.6	2.588	Ward and I	1987	16700	Mollusca	7805 Bivalvia
Late Pliocene		3.6	2.588	Ward	1993	26351	Mollusca	7805 Bivalvia
Late Pleistocene		0.126	0.0117	DuBar	1971	17556	Mollusca	7805 Bivalvia
Late Pleistocene		0.126	0.0117	Richards	1962	18974	Mollusca	7805 Bivalvia
Late Pleistocene		0.126	0.0117	Hendy et a	2008	19909	Mollusca	7805 Bivalvia
Late Pliocene		3.6	2.588	Gardner	1943	16984	Mollusca	7805 Bivalvia
Early Pleistocene		2.588	0.781	Gardner	1943	16984	Mollusca	7805 Bivalvia
Early Pleistocene		2.588	0.781	Gardner	1943	16984	Mollusca	7805 Bivalvia
Early Pleistocene		2.588	0.781	Gardner	1943	16984	Mollusca	7805 Bivalvia
Late Pleistocene		0.126	0.0117	Richards	1962	18974	Mollusca	7805 Bivalvia
Late Pleistocene		0.126	0.0117	Sellards	1917	9011	Mollusca	7805 Bivalvia
Early Pleistocene		2.588	0.781	DuBar	1962	11119	Mollusca	7805 Bivalvia
Early Pleistocene		2.588	0.781	DuBar	1962	11119	Mollusca	7805 Bivalvia
Early Pleistocene		2.588	0.781	DuBar	1962	11119	Mollusca	7805 Bivalvia
Early Pleistocene		2.588	0.781	DuBar	1962	11119	Mollusca	7805 Bivalvia
Early Pleistocene		2.588	0.781	DuBar	1962	11119	Mollusca	7805 Bivalvia
Early Pleistocene		2.588	0.781	DuBar	1962	11119	Mollusca	7805 Bivalvia
Early Pleistocene		2.588	0.781	DuBar	1962	11119	Mollusca	7805 Bivalvia
Calabrian		1.806	0.781	DuBar	1962	11119	Mollusca	7805 Bivalvia
Calabrian		1.806	0.781	DuBar	1962	11119	Mollusca	7805 Bivalvia
Calabrian		1.806	0.781	DuBar	1962	11119	Mollusca	7805 Bivalvia
Calabrian		1.806	0.781	DuBar	1962	11119	Mollusca	7805 Bivalvia
Early Pleistocene		2.588	0.781	DuBar	1962	11119	Mollusca	7805 Bivalvia
Early Pleistocene		2.588	0.781	DuBar	1962	11119	Mollusca	7805 Bivalvia
Early Pleistocene		2.588	0.781	DuBar	1962	11119	Mollusca	7805 Bivalvia
Pliocene	Pleistocene	5.333	0.0117	DuBar	1962	11119	Mollusca	7805 Bivalvia
Pliocene	Pleistocene	5.333	0.0117	DuBar	1962	11119	Mollusca	7805 Bivalvia
Late Pliocene		3.6	2.588	Mansfield	1932	17094	Mollusca	7805 Bivalvia
Late Pliocene		3.6	2.588	Mansfield	1932	17094	Mollusca	7805 Bivalvia
Late Pliocene		3.6	2.588	Mansfield	1932	17094	Mollusca	7805 Bivalvia
Late Pliocene		3.6	2.588	Mansfield	1932	17094	Mollusca	7805 Bivalvia
Late Pliocene		3.6	2.588	Mansfield	1932	17094	Mollusca	7805 Bivalvia
Late Pliocene		3.6	2.588	Mansfield	1932	17094	Mollusca	7805 Bivalvia
Late Pliocene		3.6	2.588	Mansfield	1932	17094	Mollusca	7805 Bivalvia
Late Pliocene		3.6	2.588	Mansfield	1932	17094	Mollusca	7805 Bivalvia
Late Pliocene		3.6	2.588	Mansfield	1932	17094	Mollusca	7805 Bivalvia
Late Pliocene		3.6	2.588	Mansfield	1932	17094	Mollusca	7805 Bivalvia
Late Pliocene		3.6	2.588	Mansfield	1932	17094	Mollusca	7805 Bivalvia

Late Pleistocene	0.126	0.0117	Portell et a	1992	17634	Mollusca	7805	Bivalvia
Early Pleistocene	2.588	0.781	Portell et a	1992	17634	Mollusca	7805	Bivalvia
Middle Pleistocene	0.781	0.126	Portell et a	1992	17634	Mollusca	7805	Bivalvia
Middle Pleistocene	0.781	0.126	Portell et a	1992	17634	Mollusca	7805	Bivalvia
Middle Pleistocene	0.781	0.126	McGinty	1970	19114	Mollusca	7805	Bivalvia
Middle Pleistocene	0.781	0.126	Hoerle	1970	19536	Mollusca	7805	Bivalvia
Middle Pleistocene	0.781	0.126	Hoerle	1970	19536	Mollusca	7805	Bivalvia
Middle Pleistocene	0.781	0.126	Hoerle	1970	19536	Mollusca	7805	Bivalvia
Middle Pleistocene	0.781	0.126	Hoerle	1970	19536	Mollusca	7805	Bivalvia
Middle Pleistocene	0.781	0.126	Hoerle	1970	19536	Mollusca	7805	Bivalvia
Middle Pleistocene	0.781	0.126	Hoerle	1970	19536	Mollusca	7805	Bivalvia
Late Pleistocene	0.126	0.0117	Hendy et a	2008	19909	Mollusca	7805	Bivalvia
Late Pleistocene	0.126	0.0117	Hendy et a	2008	19909	Mollusca	7805	Bivalvia
Early Pleistocene	2.588	0.781	Schroeder	1954	25303	Mollusca	7805	Bivalvia
Early Pleistocene	2.588	0.781	Schroeder	1954	25303	Mollusca	7805	Bivalvia
Early Pleistocene	2.588	0.781	Olsson anc	1953	25463	Mollusca	7805	Bivalvia
Early Pleistocene	2.588	0.781	Olsson anc	1953	25463	Mollusca	7805	Bivalvia
Early Pleistocene	2.588	0.781	Olsson anc	1953	25463	Mollusca	7805	Bivalvia
Early Pleistocene	2.588	0.781	Olsson anc	1953	25463	Mollusca	7805	Bivalvia
Early Pleistocene	2.588	0.781	Hendy et a	2008	19909	Mollusca	7805	Bivalvia
Early Pleistocene	2.588	0.781	Hendy et a	2008	19909	Mollusca	7805	Bivalvia
Early Pleistocene	2.588	0.781	Hendy et a	2008	19909	Mollusca	7805	Bivalvia
Early Pleistocene	2.588	0.781	Hendy et a	2008	19909	Mollusca	7805	Bivalvia
Early Pleistocene	2.588	0.781	Hendy et a	2008	19909	Mollusca	7805	Bivalvia
Early Pleistocene	2.588	0.781	Hendy et a	2008	19909	Mollusca	7805	Bivalvia
Pleistocene	2.588	0.0117	Hendy et a	2008	19909	Mollusca	7805	Bivalvia
Pleistocene	2.588	0.0117	Hendy et a	2008	19909	Mollusca	7805	Bivalvia
Pleistocene	2.588	0.0117	Hendy et a	2008	19909	Mollusca	7805	Bivalvia
Late Pliocene	3.6	2.588	Hendy et a	2008	19909	Mollusca	7805	Bivalvia
Late Pliocene	3.6	2.588	Hendy et a	2008	19909	Mollusca	7805	Bivalvia
Late Pliocene	3.6	2.588	Hendy et a	2008	19909	Mollusca	7805	Bivalvia
Late Pliocene	3.6	2.588	Hendy et a	2008	19909	Mollusca	7805	Bivalvia
Late Pliocene	3.6	2.588	Hendy et a	2008	19909	Mollusca	7805	Bivalvia
Late Pliocene	3.6	2.588	Hendy et a	2008	19909	Mollusca	7805	Bivalvia
Late Pliocene	3.6	2.588	Hendy et a	2008	19909	Mollusca	7805	Bivalvia
Late Pliocene	3.6	2.588	Hendy et a	2008	19909	Mollusca	7805	Bivalvia
Late Pliocene	3.6	2.588	Hendy et a	2008	19909	Mollusca	7805	Bivalvia
Late Pliocene	3.6	2.588	Hendy et a	2008	19909	Mollusca	7805	Bivalvia
Middle Pleistocene	0.781	0.126	Hendy et a	2008	19909	Mollusca	7805	Bivalvia
Middle Pleistocene	0.781	0.126	Hendy et a	2008	19909	Mollusca	7805	Bivalvia
Late Pleistocene	0.126	0.0117	Hendy et a	2008	19909	Mollusca	7805	Bivalvia
Early Pleistocene	2.588	0.781	DuBar	1958	25514	Mollusca	7805	Bivalvia
Early Pleistocene	2.588	0.781	DuBar	1958	25514	Mollusca	7805	Bivalvia
Early Pleistocene	2.588	0.781	DuBar	1958	25514	Mollusca	7805	Bivalvia
Late Pliocene	3.6	2.588	Smith	1941	32333	Mollusca	7805	Bivalvia
Late Pliocene	3.6	2.588	Smith	1941	32333	Mollusca	7805	Bivalvia
Late Pliocene	3.6	2.588	Smith	1941	32333	Mollusca	7805	Bivalvia

Early Pleistocene		2.588	0.781	Anderson	1996	24475	Mollusca	7805	Bivalvia
Middle Pleistocene		0.781	0.126	Anderson	1996	24475	Mollusca	7805	Bivalvia
Late Pleistocene		0.126	0.0117	Kittle and I	2010	40884	Mollusca	7805	Bivalvia
Late Pleistocene		0.126	0.0117	Kittle and I	2010	40884	Mollusca	7805	Bivalvia
Late Pleistocene		0.126	0.0117	DuBar	1971	17556	Mollusca	7805	Bivalvia
Late Pleistocene		0.126	0.0117	DuBar	1971	17556	Mollusca	7805	Bivalvia
Early Pleistocene		2.588	0.781	DuBar	1971	17556	Mollusca	7805	Bivalvia
Early Pleistocene		2.588	0.781	DuBar	1971	17556	Mollusca	7805	Bivalvia
Late Pleistocene		0.126	0.0117	Hendy et a	2008	19909	Mollusca	7805	Bivalvia
Late Pleistocene		0.126	0.0117	Hendy et a	2008	19909	Mollusca	7805	Bivalvia
Late Pleistocene		0.126	0.0117	Hendy et a	2008	19909	Mollusca	7805	Bivalvia
Pleistocene		2.588	0.0117	Hendy et a	2008	19909	Mollusca	7805	Bivalvia
Early Pleistocene		2.588	0.781	Cooke	1936	31145	Mollusca	7805	Bivalvia
Early Pleistocene		2.588	0.781	Cooke	1936	31145	Mollusca	7805	Bivalvia
Late Pliocene		3.6	2.588	Cooke	1936	31145	Mollusca	7805	Bivalvia
Late Pliocene		3.6	2.588	Cooke	1936	31145	Mollusca	7805	Bivalvia
Pleistocene		2.588	0.0117	Holmes	1860	64712	Mollusca	7805	Bivalvia
Late Pliocene		3.6	2.588	Ward and I	1987	16700	Mollusca	7805	Bivalvia
Late Pliocene		3.6	2.588	Ward and I	1987	16700	Mollusca	7805	Bivalvia
Late Pliocene		3.6	2.588	Ward and I	1987	16700	Mollusca	7805	Bivalvia
Late Pliocene		3.6	2.588	Ward and I	1987	16700	Mollusca	7805	Bivalvia
Early Pleistocene		2.588	0.781	Ward and I	1987	16700	Mollusca	7805	Bivalvia
Early Pleistocene		2.588	0.781	Ward and I	1987	16700	Mollusca	7805	Bivalvia
Early Pleistocene		2.588	0.781	Ward and I	1987	16700	Mollusca	7805	Bivalvia
Early Plioc	Late Plioce	5.333	2.588	Gardner	1943	16984	Mollusca	7805	Bivalvia
Early Plioc	Late Plioce	5.333	2.588	Gardner	1943	16984	Mollusca	7805	Bivalvia
Early Plioc	Late Plioce	5.333	2.588	Gardner	1943	16984	Mollusca	7805	Bivalvia
Late Pliocene		3.6	2.588	Gardner	1943	16984	Mollusca	7805	Bivalvia
Early Pleistocene		2.588	0.781	Gardner	1943	16984	Mollusca	7805	Bivalvia
Early Pleistocene		2.588	0.781	DuBar	1971	17556	Mollusca	7805	Bivalvia
Early Pleistocene		2.588	0.781	Miller	1990	17561	Mollusca	7805	Bivalvia
Early Pleistocene		2.588	0.781	Miller	1990	17561	Mollusca	7805	Bivalvia
Early Pleistocene		2.588	0.781	Miller	1990	17561	Mollusca	7805	Bivalvia
Late Pleistocene		0.126	0.0117	Richards	1962	18974	Mollusca	7805	Bivalvia
Late Pleistocene		0.126	0.0117	Richards	1962	18974	Mollusca	7805	Bivalvia
Late Pleistocene		0.126	0.0117	Richards	1962	18974	Mollusca	7805	Bivalvia
Late Pliocene		3.6	2.588	Hendy et a	2008	19909	Mollusca	7805	Bivalvia
Late Pleistocene		0.126	0.0117	Hendy et a	2008	19909	Mollusca	7805	Bivalvia
Gelasian		2.588	1.806	Bailey and	1986	23840	Mollusca	7805	Bivalvia
Early Pleistocene		2.588	0.781	Hendy et a	2008	19909	Mollusca	7805	Bivalvia
Late Pliocene		3.6	2.588	Hendy et a	2008	19909	Mollusca	7805	Bivalvia
Late Pliocene		3.6	2.588	Hendy et a	2008	19909	Mollusca	7805	Bivalvia
Pliocene		5.333	2.588	Hendy et a	2008	19909	Mollusca	7805	Bivalvia
Late Pliocene		3.6	2.588	Hendy et a	2008	19909	Mollusca	7805	Bivalvia
Late Pliocene		3.6	2.588	Hendy et a	2008	19909	Mollusca	7805	Bivalvia
Early Pleistocene		2.588	0.781	Hendy et a	2008	19909	Mollusca	7805	Bivalvia
Early Pleistocene		2.588	0.781	Hendy et a	2008	19909	Mollusca	7805	Bivalvia

Late Pliocene	3.6	2.588	Hendy et a	2008	19909	Mollusca	7805	Bivalvia
Early Pleistocene	2.588	0.781	Hendy et a	2008	19909	Mollusca	7805	Bivalvia
Early Pleistocene	2.588	0.781	Hendy et a	2008	19909	Mollusca	7805	Bivalvia
Early Pleistocene	2.588	0.781	Hendy et a	2008	19909	Mollusca	7805	Bivalvia
Pleistocene	2.588	0.0117	Coch	1971	441	Mollusca	7805	Bivalvia
Late Pleistocene	0.126	0.0117	Spencer ar	1987	15525	Mollusca	7805	Bivalvia
Late Pleistocene	0.126	0.0117	Spencer ar	1987	15525	Mollusca	7805	Bivalvia
Late Pleistocene	0.126	0.0117	Spencer ar	1987	15525	Mollusca	7805	Bivalvia
Late Pleistocene	0.126	0.0117	Spencer ar	1987	15525	Mollusca	7805	Bivalvia
Late Pleistocene	0.126	0.0117	Spencer ar	1987	15525	Mollusca	7805	Bivalvia
Late Pleistocene	0.126	0.0117	Spencer ar	1987	15525	Mollusca	7805	Bivalvia
Late Pleistocene	0.126	0.0117	Hendy et a	2008	19909	Mollusca	7805	Bivalvia
Late Pliocene	3.6	2.588	Ward	1993	26351	Mollusca	7805	Bivalvia
Late Pliocene	3.6	2.588	Ward	1993	26351	Mollusca	7805	Bivalvia
Late Pliocene	3.6	2.588	Ward	1993	26351	Mollusca	7805	Bivalvia
Late Pliocene	3.6	2.588	Hendy et a	2008	19909	Mollusca	7805	Bivalvia
Late Pliocene	3.6	2.588	Hendy et a	2008	19909	Mollusca	7805	Bivalvia
Late Pliocene	3.6	2.588	Hendy et a	2008	19909	Mollusca	7805	Bivalvia
Late Pliocene	3.6	2.588	Hendy et a	2008	19909	Mollusca	7805	Bivalvia
Late Pliocene	3.6	2.588	Hendy et a	2008	19909	Mollusca	7805	Bivalvia
Late Pliocene	3.6	2.588	Hendy et a	2008	19909	Mollusca	7805	Bivalvia
Late Pleistocene	0.126	0.0117	Sellards	1917	9011	Mollusca	7805	Bivalvia
Late Pleistocene	0.126	0.0117	Sellards	1917	9011	Mollusca	7805	Bivalvia
Early Pleistocene	2.588	0.781	DuBar	1962	11119	Mollusca	7805	Bivalvia
Early Pleistocene	2.588	0.781	DuBar	1962	11119	Mollusca	7805	Bivalvia
Early Pleistocene	2.588	0.781	DuBar	1962	11119	Mollusca	7805	Bivalvia
Calabrian	1.806	0.781	DuBar	1962	11119	Mollusca	7805	Bivalvia
Calabrian	1.806	0.781	DuBar	1962	11119	Mollusca	7805	Bivalvia
Calabrian	1.806	0.781	DuBar	1962	11119	Mollusca	7805	Bivalvia
Late Pliocene	3.6	2.588	Mansfield	1932	17094	Mollusca	7805	Bivalvia
Late Pliocene	3.6	2.588	Mansfield	1932	17094	Mollusca	7805	Bivalvia
Late Pliocene	3.6	2.588	Mansfield	1932	17094	Mollusca	7805	Bivalvia
Late Pliocene	3.6	2.588	Mansfield	1932	17094	Mollusca	7805	Bivalvia
Late Pliocene	3.6	2.588	Mansfield	1932	17094	Mollusca	7805	Bivalvia
Late Pliocene	3.6	2.588	Mansfield	1932	17094	Mollusca	7805	Bivalvia
Late Pliocene	3.6	2.588	Mansfield	1932	17094	Mollusca	7805	Bivalvia
Late Pliocene	3.6	2.588	Mansfield	1932	17094	Mollusca	7805	Bivalvia
Late Pliocene	3.6	2.588	Mansfield	1932	17094	Mollusca	7805	Bivalvia
Late Pliocene	3.6	2.588	Mansfield	1932	17094	Mollusca	7805	Bivalvia
Late Pliocene	3.6	2.588	Mansfield	1932	17094	Mollusca	7805	Bivalvia
Late Pliocene	3.6	2.588	Mansfield	1932	17094	Mollusca	7805	Bivalvia
Late Pliocene	3.6	2.588	Mansfield	1932	17094	Mollusca	7805	Bivalvia
Early Pleistocene	2.588	0.781	Portell et a	1992	17634	Mollusca	7805	Bivalvia
Middle Pleistocene	0.781	0.126	Portell et a	1992	17634	Mollusca	7805	Bivalvia
Late Pleistocene	0.126	0.0117	Portell et a	1992	17634	Mollusca	7805	Bivalvia
Middle Pleistocene	0.781	0.126	McGinty	1970	19114	Mollusca	7805	Bivalvia
Middle Pleistocene	0.781	0.126	McGinty	1970	19114	Mollusca	7805	Bivalvia

Pliocene	5.333	2.588	Schroeder	1954	25303	Mollusca	7805	Bivalvia
Early Pleistocene	2.588	0.781	Schroeder	1954	25303	Mollusca	7805	Bivalvia
Early Pleistocene	2.588	0.781	Olsson anc	1953	25463	Mollusca	7805	Bivalvia
Early Pleistocene	2.588	0.781	Olsson anc	1953	25463	Mollusca	7805	Bivalvia
Early Pleistocene	2.588	0.781	Olsson anc	1953	25463	Mollusca	7805	Bivalvia
Early Pleistocene	2.588	0.781	Olsson anc	1953	25463	Mollusca	7805	Bivalvia
Early Pleistocene	2.588	0.781	Olsson anc	1953	25463	Mollusca	7805	Bivalvia
Early Pleistocene	2.588	0.781	Hendy et a	2008	19909	Mollusca	7805	Bivalvia
Late Pliocene	3.6	2.588	Petuch anc	2007	26813	Mollusca	7805	Bivalvia
Late Pliocene	3.6	2.588	Hendy et a	2008	19909	Mollusca	7805	Bivalvia
Late Pliocene	3.6	2.588	Hendy et a	2008	19909	Mollusca	7805	Bivalvia
Late Pliocene	3.6	2.588	Hendy et a	2008	19909	Mollusca	7805	Bivalvia
Late Pliocene	3.6	2.588	Hendy et a	2008	19909	Mollusca	7805	Bivalvia
Middle Pleistocene	0.781	0.126	Hendy et a	2008	19909	Mollusca	7805	Bivalvia
Late Pliocene	3.6	2.588	Smith	1941	32333	Mollusca	7805	Bivalvia
Late Pleistocene	0.126	0.0117	Kittle and I	2010	40884	Mollusca	7805	Bivalvia
Late Pleistocene	0.126	0.0117	Kittle and I	2010	40884	Mollusca	7805	Bivalvia
Late Pleistocene	0.126	0.0117	DuBar	1971	17556	Mollusca	7805	Bivalvia
Late Pleistocene	0.126	0.0117	DuBar	1971	17556	Mollusca	7805	Bivalvia
Late Pleistocene	0.126	0.0117	DuBar	1971	17556	Mollusca	7805	Bivalvia
Late Pleistocene	0.126	0.0117	DuBar	1971	17556	Mollusca	7805	Bivalvia
Late Pleistocene	0.126	0.0117	DuBar	1971	17556	Mollusca	7805	Bivalvia
Late Pleistocene	0.126	0.0117	DuBar	1971	17556	Mollusca	7805	Bivalvia
Zanclean	5.333	3.6	Campbell a	1995	17558	Mollusca	7805	Bivalvia
Early Pliocene	5.333	3.6	Campbell a	1995	17558	Mollusca	7805	Bivalvia
Early Pleistocene	2.588	0.781	DuBar	1971	17556	Mollusca	7805	Bivalvia
Early Pleistocene	2.588	0.781	DuBar	1971	17556	Mollusca	7805	Bivalvia
Late Pleistocene	0.126	0.0117	Richards	1962	18974	Mollusca	7805	Bivalvia
Late Pleistocene	0.126	0.0117	Richards	1962	18974	Mollusca	7805	Bivalvia
Late Pleistocene	0.126	0.0117	Richards	1962	18974	Mollusca	7805	Bivalvia
Late Pleistocene	0.126	0.0117	Hendy et a	2008	19909	Mollusca	7805	Bivalvia
Late Pleistocene	0.126	0.0117	Hendy et a	2008	19909	Mollusca	7805	Bivalvia
Late Pleistocene	0.126	0.0117	Hendy et a	2008	19909	Mollusca	7805	Bivalvia
Late Pleistocene	0.126	0.0117	Hendy et a	2008	19909	Mollusca	7805	Bivalvia
Pleistocene	2.588	0.0117	Hendy et a	2008	19909	Mollusca	7805	Bivalvia
Pleistocene	2.588	0.0117	Hendy et a	2008	19909	Mollusca	7805	Bivalvia
Pleistocene	2.588	0.0117	Hendy et a	2008	19909	Mollusca	7805	Bivalvia
Early Pleistocene	2.588	0.781	Cooke	1936	31145	Mollusca	7805	Bivalvia
Early Pleistocene	2.588	0.781	Cooke	1936	31145	Mollusca	7805	Bivalvia
Late Pliocene	3.6	2.588	Cooke	1936	31145	Mollusca	7805	Bivalvia
Late Pliocene	3.6	2.588	Ward and I	1987	16700	Mollusca	7805	Bivalvia
Late Pliocene	3.6	2.588	Ward and I	1987	16700	Mollusca	7805	Bivalvia
Late Pliocene	3.6	2.588	Ward and I	1987	16700	Mollusca	7805	Bivalvia
Late Pliocene	3.6	2.588	Ward and I	1987	16700	Mollusca	7805	Bivalvia
Late Pliocene	3.6	2.588	Ward and I	1987	16700	Mollusca	7805	Bivalvia
Late Pliocene	3.6	2.588	Ward and I	1987	16700	Mollusca	7805	Bivalvia
Late Pliocene	3.6	2.588	Ward and I	1987	16700	Mollusca	7805	Bivalvia

Early Pleistocene		2.588	0.781	Ward and I	1987	16700	Mollusca	7805 Bivalvia
Early Pleistocene		2.588	0.781	Ward and I	1987	16700	Mollusca	7805 Bivalvia
Early Pleistocene		2.588	0.781	Ward and I	1987	16700	Mollusca	7805 Bivalvia
Early Pleistocene		2.588	0.781	Ward and I	1987	16700	Mollusca	7805 Bivalvia
Early Pleistocene		2.588	0.781	Ward and I	1987	16700	Mollusca	7805 Bivalvia
Early Pleistocene		2.588	0.781	Ward and I	1987	16700	Mollusca	7805 Bivalvia
Late Pliocene		3.6	2.588	Ward	1992	16985	Mollusca	7805 Bivalvia
Early Plioc	Late Plioce	5.333	2.588	Gardner	1943	16984	Mollusca	7805 Bivalvia
Early Plioc	Late Plioce	5.333	2.588	Gardner	1943	16984	Mollusca	7805 Bivalvia
Late Pliocene		3.6	2.588	Gardner	1943	16984	Mollusca	7805 Bivalvia
Early Plioc	Late Plioce	5.333	2.588	Gardner	1943	16984	Mollusca	7805 Bivalvia
Late Pliocene		3.6	2.588	Gardner	1943	16984	Mollusca	7805 Bivalvia
Late Pliocene		3.6	2.588	Gardner	1943	16984	Mollusca	7805 Bivalvia
Early Pleistocene		2.588	0.781	Gardner	1943	16984	Mollusca	7805 Bivalvia
Pleistocene		2.588	0.0117	Gardner	1943	16984	Mollusca	7805 Bivalvia
Early Pleistocene		2.588	0.781	Gardner	1943	16984	Mollusca	7805 Bivalvia
Early Pleistocene		2.588	0.781	Gardner	1943	16984	Mollusca	7805 Bivalvia
Early Pleistocene		2.588	0.781	Gardner	1943	16984	Mollusca	7805 Bivalvia
Late Pliocene		3.6	2.588	Gardner	1943	16984	Mollusca	7805 Bivalvia
Early Pleistocene		2.588	0.781	Gardner	1943	16984	Mollusca	7805 Bivalvia
Late Pliocene		3.6	2.588	Gardner	1943	16984	Mollusca	7805 Bivalvia
Late Pliocene		3.6	2.588	Gardner	1943	16984	Mollusca	7805 Bivalvia
Early Pleistocene		2.588	0.781	Gardner	1943	16984	Mollusca	7805 Bivalvia
Late Pleistocene		0.126	0.0117	Richards	1962	18974	Mollusca	7805 Bivalvia
Late Pleistocene		0.126	0.0117	Richards	1962	18974	Mollusca	7805 Bivalvia
Late Pleistocene		0.126	0.0117	Richards	1962	18974	Mollusca	7805 Bivalvia
Late Pliocene		3.6	2.588	Hendy et a	2008	19909	Mollusca	7805 Bivalvia
Late Pliocene		3.6	2.588	Hendy et a	2008	19909	Mollusca	7805 Bivalvia
Late Pliocene		3.6	2.588	Hendy et a	2008	19909	Mollusca	7805 Bivalvia
Late Pliocene		3.6	2.588	Hendy et a	2008	19909	Mollusca	7805 Bivalvia
Early Pleistocene		2.588	0.781	Hendy et a	2008	19909	Mollusca	7805 Bivalvia
Early Pleistocene		2.588	0.781	Hendy et a	2008	19909	Mollusca	7805 Bivalvia
Early Pleistocene		2.588	0.781	Hendy et a	2008	19909	Mollusca	7805 Bivalvia
Early Pleistocene		2.588	0.781	Hendy et a	2008	19909	Mollusca	7805 Bivalvia
Early Pleistocene		2.588	0.781	Hendy et a	2008	19909	Mollusca	7805 Bivalvia
Late Pleistocene		0.126	0.0117	Spencer ar	1987	15525	Mollusca	7805 Bivalvia
Late Pleistocene		0.126	0.0117	Spencer ar	1987	15525	Mollusca	7805 Bivalvia
Late Pleistocene		0.126	0.0117	Spencer ar	1987	15525	Mollusca	7805 Bivalvia
Late Pleistocene		0.126	0.0117	Spencer ar	1987	15525	Mollusca	7805 Bivalvia
Early Pliocene		5.333	3.6	Ward	1992	16985	Mollusca	7805 Bivalvia
Late Pliocene		3.6	2.588	Edwards et	2005	17631	Mollusca	7805 Bivalvia
Late Pliocene		3.6	2.588	Edwards et	2005	17631	Mollusca	7805 Bivalvia
Zanclean		5.333	3.6	Ward	1992	16985	Mollusca	7805 Bivalvia
Zanclean		5.333	3.6	Ward	1992	16985	Mollusca	7805 Bivalvia
Late Pliocene		3.6	2.588	Ward	1992	16985	Mollusca	7805 Bivalvia
Late Pliocene		3.6	2.588	Ward	1993	26351	Mollusca	7805 Bivalvia
Late Pliocene		3.6	2.588	Ward	1993	26351	Mollusca	7805 Bivalvia

Late Pliocene	3.6	2.588	Ward	1993	26351	Mollusca	7805	Bivalvia
Late Pliocene	3.6	2.588	Ward	1993	26351	Mollusca	7805	Bivalvia
Late Pliocene	3.6	2.588	Ward	1993	26351	Mollusca	7805	Bivalvia
Late Pliocene	3.6	2.588	Ward	1993	26351	Mollusca	7805	Bivalvia
Late Pliocene	3.6	2.588	Ward	1993	26351	Mollusca	7805	Bivalvia
Late Pliocene	3.6	2.588	Ward	1993	26351	Mollusca	7805	Bivalvia
Late Pliocene	3.6	2.588	Ward	1993	26351	Mollusca	7805	Bivalvia
Late Pliocene	3.6	2.588	Ward	1993	26351	Mollusca	7805	Bivalvia
Late Pliocene	3.6	2.588	Hendy et a	2008	19909	Mollusca	7805	Bivalvia
Late Pliocene	3.6	2.588	Hendy et a	2008	19909	Mollusca	7805	Bivalvia
Late Pliocene	3.6	2.588	Hendy et a	2008	19909	Mollusca	7805	Bivalvia
Late Pliocene	3.6	2.588	Hendy et a	2008	19909	Mollusca	7805	Bivalvia
Late Pliocene	3.6	2.588	Hendy et a	2008	19909	Mollusca	7805	Bivalvia
Late Pliocene	3.6	2.588	Hendy et a	2008	19909	Mollusca	7805	Bivalvia
Late Pliocene	3.6	2.588	Hendy et a	2008	19909	Mollusca	7805	Bivalvia
Middle Pleistocene	0.781	0.126	McGinty	1970	19114	Mollusca	7805	Bivalvia
Middle Pleistocene	0.781	0.126	McGinty	1970	19114	Mollusca	7805	Bivalvia
Late Pleistocene	0.126	0.0117	Kittle and I	2010	40884	Mollusca	7805	Bivalvia
Early Pleistocene	2.588	0.781	Ward and I	1987	16700	Mollusca	7805	Bivalvia
Late Pleistocene	0.126	0.0117	Spencer ar	1987	15525	Mollusca	7805	Bivalvia
Late Pliocene	3.6	2.588	Ward	1993	26351	Mollusca	7805	Bivalvia
Late Pliocene	3.6	2.588	Mansfield	1932	17094	Mollusca	7805	Bivalvia
Middle Pleistocene	0.781	0.126	Hoerle	1970	19536	Mollusca	7805	Bivalvia
Middle Pleistocene	0.781	0.126	Hoerle	1970	19536	Mollusca	7805	Bivalvia
Middle Pleistocene	0.781	0.126	Hoerle	1970	19536	Mollusca	7805	Bivalvia
Middle Pleistocene	0.781	0.126	Hoerle	1970	19536	Mollusca	7805	Bivalvia
Early Pleistocene	2.588	0.781	Olsson anc	1953	25463	Mollusca	7805	Bivalvia
Early Pleistocene	2.588	0.781	Olsson anc	1953	25463	Mollusca	7805	Bivalvia
Early Pleistocene	2.588	0.781	Olsson anc	1953	25463	Mollusca	7805	Bivalvia
Late Pleistocene	0.126	0.0117	Hendy et a	2008	19909	Mollusca	7805	Bivalvia
Late Pleistocene	0.126	0.0117	Sellards	1917	9011	Mollusca	7805	Bivalvia
Late Pleistocene	0.126	0.0117	Sellards	1917	9011	Mollusca	7805	Bivalvia
Pliocene	5.333	2.588	Schroeder	1954	25303	Mollusca	7805	Bivalvia
Early Pleistocene	2.588	0.781	Olsson anc	1953	25463	Mollusca	7805	Bivalvia
Late Pleistocene	0.126	0.0117	Lovejoy	1992	26806	Mollusca	7805	Bivalvia
Late Pleistocene	0.126	0.0117	Hulbert an	1998	1962	Mollusca	7805	Bivalvia
Late Pleistocene	0.126	0.0117	Hulbert an	1998	1962	Mollusca	7805	Bivalvia
Late Pleistocene	0.126	0.0117	Hulbert an	1998	1962	Mollusca	7805	Bivalvia
Late Pleistocene	0.126	0.0117	DuBar	1971	17556	Mollusca	7805	Bivalvia
Late Pleistocene	0.126	0.0117	DuBar	1971	17556	Mollusca	7805	Bivalvia
Late Pleistocene	0.126	0.0117	DuBar	1971	17556	Mollusca	7805	Bivalvia
Late Pleistocene	0.126	0.0117	DuBar	1971	17556	Mollusca	7805	Bivalvia
Late Pleistocene	0.126	0.0117	DuBar	1971	17556	Mollusca	7805	Bivalvia
Late Pleistocene	0.126	0.0117	Richards	1962	18974	Mollusca	7805	Bivalvia
Late Pleistocene	0.126	0.0117	Richards	1962	18974	Mollusca	7805	Bivalvia
Late Pleistocene	0.126	0.0117	Richards	1962	18974	Mollusca	7805	Bivalvia
Late Pleistocene	0.126	0.0117	Hendy et a	2008	19909	Mollusca	7805	Bivalvia

Late Pleistocene		0.126	0.0117	Hendy et a	2008	19909	Mollusca	7805 Bivalvia
Late Pleistocene		0.126	0.0117	Hendy et a	2008	19909	Mollusca	7805 Bivalvia
Late Pleistocene		0.126	0.0117	Cooke	1936	31145	Mollusca	7805 Bivalvia
Early Pleistocene		2.588	0.781	Cooke	1936	31145	Mollusca	7805 Bivalvia
Early Pleistocene		2.588	0.781	Cooke	1936	31145	Mollusca	7805 Bivalvia
Pleistocene		2.588	0.0117	Holmes	1860	64712	Mollusca	7805 Bivalvia
Late Pliocene		3.6	2.588	Ward and I	1987	16700	Mollusca	7805 Bivalvia
Late Pliocene		3.6	2.588	Ward and I	1987	16700	Mollusca	7805 Bivalvia
Early Pleistocene		2.588	0.781	Ward and I	1987	16700	Mollusca	7805 Bivalvia
Early Pleistocene		2.588	0.781	Gardner	1943	16984	Mollusca	7805 Bivalvia
Pleistocene		2.588	0.0117	Gardner	1943	16984	Mollusca	7805 Bivalvia
Early Pleistocene		2.588	0.781	Gardner	1943	16984	Mollusca	7805 Bivalvia
Late Pliocene		3.6	2.588	Gardner	1943	16984	Mollusca	7805 Bivalvia
Late Pliocene		3.6	2.588	Gardner	1943	16984	Mollusca	7805 Bivalvia
Late Pliocene		3.6	2.588	Gardner	1943	16984	Mollusca	7805 Bivalvia
Early Pleistocene		2.588	0.781	Gardner	1943	16984	Mollusca	7805 Bivalvia
Early Plioc	Late Plioce	5.333	2.588	Gardner	1943	16984	Mollusca	7805 Bivalvia
Early Pleistocene		2.588	0.781	Gardner	1943	16984	Mollusca	7805 Bivalvia
Late Pliocene		3.6	2.588	Gardner	1943	16984	Mollusca	7805 Bivalvia
Early Pleistocene		2.588	0.781	Gardner	1943	16984	Mollusca	7805 Bivalvia
Late Pliocene		3.6	2.588	Gardner	1943	16984	Mollusca	7805 Bivalvia
Pleistocene		2.588	0.0117	Gardner	1943	16984	Mollusca	7805 Bivalvia
Early Pleistocene		2.588	0.781	Gardner	1943	16984	Mollusca	7805 Bivalvia
Early Pleistocene		2.588	0.781	Gardner	1943	16984	Mollusca	7805 Bivalvia
Late Pleistocene		0.126	0.0117	Richards	1962	18974	Mollusca	7805 Bivalvia
Late Pleistocene		0.126	0.0117	Richards	1962	18974	Mollusca	7805 Bivalvia
Late Pleistocene		0.126	0.0117	Richards	1962	18974	Mollusca	7805 Bivalvia
Late Pleistocene		0.126	0.0117	Richards	1962	18974	Mollusca	7805 Bivalvia
Late Pleistocene		0.126	0.0117	Richards	1962	18974	Mollusca	7805 Bivalvia
Late Pleistocene		0.126	0.0117	Hendy et a	2008	19909	Mollusca	7805 Bivalvia
Early Pleistocene		2.588	0.781	Hendy et a	2008	19909	Mollusca	7805 Bivalvia
Late Pleistocene		0.126	0.0117	Spencer ar	1987	15525	Mollusca	7805 Bivalvia
Late Pleistocene		0.126	0.0117	Spencer ar	1987	15525	Mollusca	7805 Bivalvia
Late Pliocene		3.6	2.588	Ward	1993	26351	Mollusca	7805 Bivalvia
Early Pleistocene		2.588	0.781	Olsson anc	1953	25463	Mollusca	7805 Bivalvia
Early Pleistocene		2.588	0.781	Hendy et a	2008	19909	Mollusca	7805 Bivalvia
Late Pleistocene		0.126	0.0117	Kittle and I	2010	40884	Mollusca	7805 Bivalvia
Late Pleistocene		0.126	0.0117	Richards	1962	18974	Mollusca	7805 Bivalvia
Middle Pleistocene		0.781	0.126	McGinty	1970	19114	Mollusca	7805 Bivalvia
Early Pleistocene		2.588	0.781	Olsson anc	1953	25463	Mollusca	7805 Bivalvia
Early Pleistocene		2.588	0.781	Hendy et a	2008	19909	Mollusca	7805 Bivalvia
Late Pleistocene		0.126	0.0117	DuBar	1971	17556	Mollusca	7805 Bivalvia
Early Pleistocene		2.588	0.781	Gardner	1943	16984	Mollusca	7805 Bivalvia
Late Pliocene		3.6	2.588	Gardner	1943	16984	Mollusca	7805 Bivalvia
Early Pleistocene		2.588	0.781	Gardner	1943	16984	Mollusca	7805 Bivalvia
Gelasian		2.588	1.806	Bailey and	1986	23840	Mollusca	7805 Bivalvia
Early Pleistocene		2.588	0.781	Mansfield	1932	11083	Mollusca	7805 Bivalvia

Early Pleistocene		2.588	0.781	DuBar	1962	11119	Mollusca	7805 Bivalvia
Late Pliocene		3.6	2.588	Mansfield	1932	17094	Mollusca	7805 Bivalvia
Middle Pleistocene		0.781	0.126	Portell et a	1992	17634	Mollusca	7805 Bivalvia
Middle Pleistocene		0.781	0.126	Hoerle	1970	19536	Mollusca	7805 Bivalvia
Middle Pleistocene		0.781	0.126	Hoerle	1970	19536	Mollusca	7805 Bivalvia
Early Pleistocene		2.588	0.781	Olsson anc	1953	25463	Mollusca	7805 Bivalvia
Early Pleistocene		2.588	0.781	Hendy et a	2008	19909	Mollusca	7805 Bivalvia
Late Pleistocene		0.126	0.0117	Kittle and I	2010	40884	Mollusca	7805 Bivalvia
Zanclean		5.333	3.6	Campbell a	1995	17558	Mollusca	7805 Bivalvia
Late Pleistocene		0.126	0.0117	Richards	1962	18974	Mollusca	7805 Bivalvia
Late Pliocene		3.6	2.588	Cooke	1936	31145	Mollusca	7805 Bivalvia
Early Pliocene		5.333	3.6	Ward	1992	16985	Mollusca	7805 Bivalvia
Early Pliocene		5.333	3.6	Ward	1992	16985	Mollusca	7805 Bivalvia
Zanclean		5.333	3.6	Ward	1992	16985	Mollusca	7805 Bivalvia
Zanclean		5.333	3.6	Ward	1992	16985	Mollusca	7805 Bivalvia
Late Pliocene		3.6	2.588	Hendy et a	2008	19909	Mollusca	7805 Bivalvia
Late Pliocene		3.6	2.588	Hendy et a	2008	19909	Mollusca	7805 Bivalvia
Late Pliocene		3.6	2.588	Hendy et a	2008	19909	Mollusca	7805 Bivalvia
Late Pleistocene		0.126	0.0117	Sellards	1917	9011	Mollusca	7805 Bivalvia
Late Pleistocene		0.126	0.0117	Sellards	1917	9011	Mollusca	7805 Bivalvia
Early Pleistocene		2.588	0.781	Mansfield	1932	11083	Mollusca	7805 Bivalvia
Early Pleistocene		2.588	0.781	Mansfield	1932	11083	Mollusca	7805 Bivalvia
Calabrian		1.806	0.781	DuBar	1962	11119	Mollusca	7805 Bivalvia
Calabrian		1.806	0.781	DuBar	1962	11119	Mollusca	7805 Bivalvia
Early Pleistocene		2.588	0.781	DuBar	1962	11119	Mollusca	7805 Bivalvia
Pliocene	Pleistocen	5.333	0.0117	DuBar	1962	11119	Mollusca	7805 Bivalvia
Late Pliocene		3.6	2.588	Mansfield	1932	17094	Mollusca	7805 Bivalvia
Late Pliocene		3.6	2.588	Mansfield	1932	17094	Mollusca	7805 Bivalvia
Late Pliocene		3.6	2.588	Mansfield	1932	17094	Mollusca	7805 Bivalvia
Late Pliocene		3.6	2.588	Mansfield	1932	17094	Mollusca	7805 Bivalvia
Late Pliocene		3.6	2.588	Mansfield	1932	17094	Mollusca	7805 Bivalvia
Late Pliocene		3.6	2.588	Mansfield	1932	17094	Mollusca	7805 Bivalvia
Early Pleistocene		2.588	0.781	Portell et a	1992	17634	Mollusca	7805 Bivalvia
Middle Pleistocene		0.781	0.126	Portell et a	1992	17634	Mollusca	7805 Bivalvia
Late Pleistocene		0.126	0.0117	Portell et a	1992	17634	Mollusca	7805 Bivalvia
Middle Pleistocene		0.781	0.126	McGinty	1970	19114	Mollusca	7805 Bivalvia
Middle Pleistocene		0.781	0.126	McGinty	1970	19114	Mollusca	7805 Bivalvia
Late Pleistocene		0.126	0.0117	Hendy et a	2008	19909	Mollusca	7805 Bivalvia
Pliocene		5.333	2.588	Schroeder	1954	25303	Mollusca	7805 Bivalvia
Pliocene		5.333	2.588	Schroeder	1954	25303	Mollusca	7805 Bivalvia
Pliocene		5.333	2.588	Schroeder	1954	25303	Mollusca	7805 Bivalvia
Early Pleistocene		2.588	0.781	Olsson anc	1953	25463	Mollusca	7805 Bivalvia
Early Pleistocene		2.588	0.781	Olsson anc	1953	25463	Mollusca	7805 Bivalvia
Early Pleistocene		2.588	0.781	Olsson anc	1953	25463	Mollusca	7805 Bivalvia
Late Pleistocene		0.126	0.0117	Lovejoy	1992	26806	Mollusca	7805 Bivalvia
Middle Pleistocene		0.781	0.126	Olsson anc	1964	26836	Mollusca	7805 Bivalvia
Late Pleistocene		0.126	0.0117	Hendy et a	2008	19909	Mollusca	7805 Bivalvia

Late Pliocene	3.6	2.588	Petuch and	2007	26813	Mollusca	7805	Bivalvia
Late Pliocene	3.6	2.588	Hendy et a	2008	19909	Mollusca	7805	Bivalvia
Early Pleistocene	2.588	0.781	Hendy et a	2008	19909	Mollusca	7805	Bivalvia
Late Pliocene	3.6	2.588	Hendy et a	2008	19909	Mollusca	7805	Bivalvia
Late Pliocene	3.6	2.588	Hendy et a	2008	19909	Mollusca	7805	Bivalvia
Late Pliocene	3.6	2.588	Hendy et a	2008	19909	Mollusca	7805	Bivalvia
Late Pliocene	3.6	2.588	Hendy et a	2008	19909	Mollusca	7805	Bivalvia
Late Pliocene	3.6	2.588	Hendy et a	2008	19909	Mollusca	7805	Bivalvia
Late Pliocene	3.6	2.588	Hendy et a	2008	19909	Mollusca	7805	Bivalvia
Late Pliocene	3.6	2.588	Hendy et a	2008	19909	Mollusca	7805	Bivalvia
Middle Pleistocene	0.781	0.126	Hendy et a	2008	19909	Mollusca	7805	Bivalvia
Late Pleistocene	0.126	0.0117	Hendy et a	2008	19909	Mollusca	7805	Bivalvia
Early Pleistocene	2.588	0.781	Hendy et a	2008	19909	Mollusca	7805	Bivalvia
Early Pleistocene	2.588	0.781	Hendy et a	2008	19909	Mollusca	7805	Bivalvia
Early Pleistocene	2.588	0.781	DuBar	1958	25514	Mollusca	7805	Bivalvia
Early Pleistocene	2.588	0.781	DuBar	1958	25514	Mollusca	7805	Bivalvia
Late Pliocene	3.6	2.588	Weisbord	1971	39743	Mollusca	7805	Bivalvia
Late Pleistocene	0.126	0.0117	Kittle and I	2010	40884	Mollusca	7805	Bivalvia
Pliocene	5.333	2.588	DuBar	1971	17556	Mollusca	7805	Bivalvia
Late Pleistocene	0.126	0.0117	DuBar	1971	17556	Mollusca	7805	Bivalvia
Late Pleistocene	0.126	0.0117	DuBar	1971	17556	Mollusca	7805	Bivalvia
Late Pleistocene	0.126	0.0117	DuBar	1971	17556	Mollusca	7805	Bivalvia
Late Pleistocene	0.126	0.0117	DuBar	1971	17556	Mollusca	7805	Bivalvia
Pliocene	5.333	2.588	DuBar	1971	17556	Mollusca	7805	Bivalvia
Late Pliocene	3.6	2.588	Campbell a	1995	17558	Mollusca	7805	Bivalvia
Zanclean	5.333	3.6	Campbell a	1995	17558	Mollusca	7805	Bivalvia
Zanclean	5.333	3.6	Campbell a	1995	17558	Mollusca	7805	Bivalvia
Early Pleistocene	2.588	0.781	DuBar	1971	17556	Mollusca	7805	Bivalvia
Late Pleistocene	0.126	0.0117	Richards	1962	18974	Mollusca	7805	Bivalvia
Late Pleistocene	0.126	0.0117	Richards	1962	18974	Mollusca	7805	Bivalvia
Late Pleistocene	0.126	0.0117	Richards	1962	18974	Mollusca	7805	Bivalvia
Late Pleistocene	0.126	0.0117	Richards	1962	18974	Mollusca	7805	Bivalvia
Late Pleistocene	0.126	0.0117	Hendy et a	2008	19909	Mollusca	7805	Bivalvia
Late Pleistocene	0.126	0.0117	Hendy et a	2008	19909	Mollusca	7805	Bivalvia
Late Pleistocene	0.126	0.0117	Hendy et a	2008	19909	Mollusca	7805	Bivalvia
Late Pleistocene	0.126	0.0117	Hendy et a	2008	19909	Mollusca	7805	Bivalvia
Pleistocene	2.588	0.0117	Hendy et a	2008	19909	Mollusca	7805	Bivalvia
Pleistocene	2.588	0.0117	Hendy et a	2008	19909	Mollusca	7805	Bivalvia
Pleistocene	2.588	0.0117	Hendy et a	2008	19909	Mollusca	7805	Bivalvia
Pleistocene	2.588	0.0117	Hendy et a	2008	19909	Mollusca	7805	Bivalvia
Late Pleistocene	0.126	0.0117	Cooke	1936	31145	Mollusca	7805	Bivalvia
Early Pleistocene	2.588	0.781	Cooke	1936	31145	Mollusca	7805	Bivalvia
Early Pleistocene	2.588	0.781	Cooke	1936	31145	Mollusca	7805	Bivalvia
Early Pleistocene	2.588	0.781	Cooke	1936	31145	Mollusca	7805	Bivalvia
Early Pleistocene	2.588	0.781	Cooke	1936	31145	Mollusca	7805	Bivalvia
Early Pleistocene	2.588	0.781	Cooke	1936	31145	Mollusca	7805	Bivalvia
Early Pleistocene	2.588	0.781	Cooke	1936	31145	Mollusca	7805	Bivalvia

Early Pleistocene		2.588	0.781	Cooke	1936	31145	Mollusca	7805	Bivalvia
Late Pliocene		3.6	2.588	Cooke	1936	31145	Mollusca	7805	Bivalvia
Late Pliocene		3.6	2.588	Ward and I	1987	16700	Mollusca	7805	Bivalvia
Late Pliocene		3.6	2.588	Ward and I	1987	16700	Mollusca	7805	Bivalvia
Late Pliocene		3.6	2.588	Ward and I	1987	16700	Mollusca	7805	Bivalvia
Late Pliocene		3.6	2.588	Ward and I	1987	16700	Mollusca	7805	Bivalvia
Late Pliocene		3.6	2.588	Ward and I	1987	16700	Mollusca	7805	Bivalvia
Late Pliocene		3.6	2.588	Ward and I	1987	16700	Mollusca	7805	Bivalvia
Late Pliocene		3.6	2.588	Ward and I	1987	16700	Mollusca	7805	Bivalvia
Late Pliocene		3.6	2.588	Ward and I	1987	16700	Mollusca	7805	Bivalvia
Early Pleistocene		2.588	0.781	Ward and I	1987	16700	Mollusca	7805	Bivalvia
Early Pleistocene		2.588	0.781	Ward and I	1987	16700	Mollusca	7805	Bivalvia
Early Pleistocene		2.588	0.781	Ward and I	1987	16700	Mollusca	7805	Bivalvia
Early Pleistocene		2.588	0.781	Ward and I	1987	16700	Mollusca	7805	Bivalvia
Early Pliocene		5.333	3.6	Ward	1992	16985	Mollusca	7805	Bivalvia
Late Pliocene		3.6	2.588	Ward	1992	16985	Mollusca	7805	Bivalvia
Early Plioc	Late Plioce	5.333	2.588	Gardner	1943	16984	Mollusca	7805	Bivalvia
Early Plioc	Late Plioce	5.333	2.588	Gardner	1943	16984	Mollusca	7805	Bivalvia
Early Plioc	Late Plioce	5.333	2.588	Gardner	1943	16984	Mollusca	7805	Bivalvia
Pleistocene		2.588	0.0117	Gardner	1943	16984	Mollusca	7805	Bivalvia
Late Pliocene		3.6	2.588	Gardner	1943	16984	Mollusca	7805	Bivalvia
Early Pleistocene		2.588	0.781	Gardner	1943	16984	Mollusca	7805	Bivalvia
Early Pleistocene		2.588	0.781	Gardner	1943	16984	Mollusca	7805	Bivalvia
Early Pleistocene		2.588	0.781	Gardner	1943	16984	Mollusca	7805	Bivalvia
Pleistocene		2.588	0.0117	Gardner	1943	16984	Mollusca	7805	Bivalvia
Early Pleistocene		2.588	0.781	Gardner	1943	16984	Mollusca	7805	Bivalvia
Late Pliocene		3.6	2.588	Gardner	1943	16984	Mollusca	7805	Bivalvia
Late Pliocene		3.6	2.588	Gardner	1943	16984	Mollusca	7805	Bivalvia
Late Pliocene		3.6	2.588	Gardner	1943	16984	Mollusca	7805	Bivalvia
Early Plioc	Late Plioce	5.333	2.588	Gardner	1943	16984	Mollusca	7805	Bivalvia
Early Pleistocene		2.588	0.781	DuBar	1971	17556	Mollusca	7805	Bivalvia
Late Pliocene		3.6	2.588	Hendy et a	2008	19909	Mollusca	7805	Bivalvia
Gelasian		2.588	1.806	Bailey and	1986	23840	Mollusca	7805	Bivalvia
Gelasian		2.588	1.806	Bailey and	1986	23840	Mollusca	7805	Bivalvia
Early Pleistocene		2.588	0.781	Hendy et a	2008	19909	Mollusca	7805	Bivalvia
Late Pliocene		3.6	2.588	Hendy et a	2008	19909	Mollusca	7805	Bivalvia
Late Pliocene		3.6	2.588	Hendy et a	2008	19909	Mollusca	7805	Bivalvia
Late Pliocene		3.6	2.588	Hendy et a	2008	19909	Mollusca	7805	Bivalvia
Pliocene		5.333	2.588	Hendy et a	2008	19909	Mollusca	7805	Bivalvia
Pliocene		5.333	2.588	Hendy et a	2008	19909	Mollusca	7805	Bivalvia
Late Pliocene		3.6	2.588	Hendy et a	2008	19909	Mollusca	7805	Bivalvia
Late Pliocene		3.6	2.588	Hendy et a	2008	19909	Mollusca	7805	Bivalvia
Late Pliocene		3.6	2.588	Hendy et a	2008	19909	Mollusca	7805	Bivalvia
Early Pleistocene		2.588	0.781	Hendy et a	2008	19909	Mollusca	7805	Bivalvia
Early Pleistocene		2.588	0.781	Hendy et a	2008	19909	Mollusca	7805	Bivalvia
Early Pleistocene		2.588	0.781	Hendy et a	2008	19909	Mollusca	7805	Bivalvia
Early Pleistocene		2.588	0.781	Hendy et a	2008	19909	Mollusca	7805	Bivalvia

Late Pliocene		3.6	2.588	Hendy et a	2008	19909	Mollusca	7805	Bivalvia
Early Pleistocene		2.588	0.781	Hendy et a	2008	19909	Mollusca	7805	Bivalvia
Early Pleistocene		2.588	0.781	Hendy et a	2008	19909	Mollusca	7805	Bivalvia
Early Pleistocene		2.588	0.781	Hendy et a	2008	19909	Mollusca	7805	Bivalvia
Early Pleistocene		2.588	0.781	Hendy et a	2008	19909	Mollusca	7805	Bivalvia
Early Pleistocene		2.588	0.781	Hendy et a	2008	19909	Mollusca	7805	Bivalvia
Early Pleistocene		2.588	0.781	Hendy et a	2008	19909	Mollusca	7805	Bivalvia
Early Pleistocene		2.588	0.781	Hendy et a	2008	19909	Mollusca	7805	Bivalvia
Early Pleistocene		2.588	0.781	Hendy et a	2008	19909	Mollusca	7805	Bivalvia
Pliocene		5.333	2.588	Ward	2007	48009	Mollusca	7805	Bivalvia
Pliocene		5.333	2.588	Ward	2007	48009	Mollusca	7805	Bivalvia
Early Pliocene		5.333	3.6	Ward	1992	16985	Mollusca	7805	Bivalvia
Pliocene		5.333	2.588	Gardner	1943	16984	Mollusca	7805	Bivalvia
Pliocene		5.333	2.588	Gardner	1943	16984	Mollusca	7805	Bivalvia
Late Pliocene		3.6	2.588	Edwards et	2005	17631	Mollusca	7805	Bivalvia
Zanclean		5.333	3.6	Ward	1992	16985	Mollusca	7805	Bivalvia
Zanclean		5.333	3.6	Ward	1992	16985	Mollusca	7805	Bivalvia
Late Pliocene		3.6	2.588	Ward	1992	16985	Mollusca	7805	Bivalvia
Late Pliocene		3.6	2.588	Ward	1993	26351	Mollusca	7805	Bivalvia
Late Pliocene		3.6	2.588	Ward	1993	26351	Mollusca	7805	Bivalvia
Late Pliocene		3.6	2.588	Ward	1993	26351	Mollusca	7805	Bivalvia
Late Pliocene		3.6	2.588	Ward	1993	26351	Mollusca	7805	Bivalvia
Late Pliocene		3.6	2.588	Ward	1993	26351	Mollusca	7805	Bivalvia
Late Pliocene		3.6	2.588	Ward	1993	26351	Mollusca	7805	Bivalvia
Late Pliocene		3.6	2.588	Ward	1993	26351	Mollusca	7805	Bivalvia
Late Pliocene		3.6	2.588	Hendy et a	2008	19909	Mollusca	7805	Bivalvia
Late Pliocene		3.6	2.588	Hendy et a	2008	19909	Mollusca	7805	Bivalvia
Late Pliocene		3.6	2.588	Hendy et a	2008	19909	Mollusca	7805	Bivalvia
Late Pliocene		3.6	2.588	Hendy et a	2008	19909	Mollusca	7805	Bivalvia
Early Pleistocene		2.588	0.781	Mansfield	1932	11083	Mollusca	7805	Bivalvia
Early Pleistocene		2.588	0.781	Mansfield	1932	11083	Mollusca	7805	Bivalvia
Early Pleistocene		2.588	0.781	Mansfield	1932	11083	Mollusca	7805	Bivalvia
Early Pleistocene		2.588	0.781	Mansfield	1932	11083	Mollusca	7805	Bivalvia
Pliocene	Pleistocen	5.333	0.0117	DuBar	1962	11119	Mollusca	7805	Bivalvia
Late Pliocene		3.6	2.588	Mansfield	1932	17094	Mollusca	7805	Bivalvia
Late Pliocene		3.6	2.588	Mansfield	1932	17094	Mollusca	7805	Bivalvia
Late Pliocene		3.6	2.588	Mansfield	1932	17094	Mollusca	7805	Bivalvia
Late Pliocene		3.6	2.588	Mansfield	1932	17094	Mollusca	7805	Bivalvia
Early Pleistocene		2.588	0.781	Olsson anc	1953	25463	Mollusca	7805	Bivalvia
Early Pliocene		5.333	3.6	Olsson anc	1964	26836	Mollusca	7805	Bivalvia
Late Pliocene		3.6	2.588	Olsson anc	1964	26836	Mollusca	7805	Bivalvia
Late Pliocene		3.6	2.588	Petuch anc	2007	26813	Mollusca	7805	Bivalvia
Late Pliocene		3.6	2.588	Hendy et a	2008	19909	Mollusca	7805	Bivalvia
Late Pliocene		3.6	2.588	Hendy et a	2008	19909	Mollusca	7805	Bivalvia
Late Pliocene		3.6	2.588	Hendy et a	2008	19909	Mollusca	7805	Bivalvia
Early Pleistocene		2.588	0.781	Hendy et a	2008	19909	Mollusca	7805	Bivalvia
Late Pliocene		3.6	2.588	Weisbord	1974	28823	Mollusca	7805	Bivalvia

Late Pliocene		3.6	2.588	Weisbord	1972	31274	Mollusca	7805	Bivalvia
Late Pliocene		3.6	2.588	Weisbord	1972	31274	Mollusca	7805	Bivalvia
Zanclean		5.333	3.6	Campbell a	1995	17558	Mollusca	7805	Bivalvia
Early Pleistocene		2.588	0.781	DuBar	1971	17556	Mollusca	7805	Bivalvia
Early Plioc	Late Plioce	5.333	2.588	Gardner	1943	16984	Mollusca	7805	Bivalvia
Late Pliocene		3.6	2.588	Gardner	1943	16984	Mollusca	7805	Bivalvia
Late Pliocene		3.6	2.588	Gardner	1943	16984	Mollusca	7805	Bivalvia
Late Pliocene		3.6	2.588	Gardner	1943	16984	Mollusca	7805	Bivalvia
Late Pliocene		3.6	2.588	Gardner	1943	16984	Mollusca	7805	Bivalvia
Late Pliocene		3.6	2.588	Gardner	1943	16984	Mollusca	7805	Bivalvia
Early Pleistocene		2.588	0.781	Gardner	1943	16984	Mollusca	7805	Bivalvia
Early Pleistocene		2.588	0.781	Gardner	1943	16984	Mollusca	7805	Bivalvia
Early Pleistocene		2.588	0.781	Gardner	1943	16984	Mollusca	7805	Bivalvia
Pliocene		5.333	2.588	Hendy et a	2008	19909	Mollusca	7805	Bivalvia
Pliocene		5.333	2.588	Hendy et a	2008	19909	Mollusca	7805	Bivalvia
Early Pleistocene		2.588	0.781	Hendy et a	2008	19909	Mollusca	7805	Bivalvia
Early Pleistocene		2.588	0.781	Hendy et a	2008	19909	Mollusca	7805	Bivalvia
Early Pleistocene		2.588	0.781	Hendy et a	2008	19909	Mollusca	7805	Bivalvia
Early Pleistocene		2.588	0.781	Hendy et a	2008	19909	Mollusca	7805	Bivalvia
Early Pleistocene		2.588	0.781	Hendy et a	2008	19909	Mollusca	7805	Bivalvia
Late Pliocene		3.6	2.588	Hendy et a	2008	19909	Mollusca	7805	Bivalvia
Late Pliocene		3.6	2.588	Hendy et a	2008	19909	Mollusca	7805	Bivalvia
Late Pliocene		3.6	2.588	Hendy et a	2008	19909	Mollusca	7805	Bivalvia
Late Pliocene		3.6	2.588	Hendy et a	2008	19909	Mollusca	7805	Bivalvia
Early Pleistocene		2.588	0.781	DuBar	1962	11119	Mollusca	7805	Bivalvia
Late Pliocene		3.6	2.588	Mansfield	1932	17094	Mollusca	7805	Bivalvia
Early Pleistocene		2.588	0.781	Portell et a	1992	17634	Mollusca	7805	Bivalvia
Late Pleistocene		0.126	0.0117	Portell et a	1992	17634	Mollusca	7805	Bivalvia
Middle Pleistocene		0.781	0.126	Hoerle	1970	19536	Mollusca	7805	Bivalvia
Middle Pleistocene		0.781	0.126	Hoerle	1970	19536	Mollusca	7805	Bivalvia
Early Pleistocene		2.588	0.781	Olsson anc	1953	25463	Mollusca	7805	Bivalvia
Early Pleistocene		2.588	0.781	Olsson anc	1953	25463	Mollusca	7805	Bivalvia
Early Pleistocene		2.588	0.781	Olsson anc	1953	25463	Mollusca	7805	Bivalvia
Late Plioce	Early Pleist	3.6	0.781	Petuch anc	2007	26813	Mollusca	7805	Bivalvia
Late Pliocene		3.6	2.588	Smith	1941	32333	Mollusca	7805	Bivalvia
Late Pleistocene		0.126	0.0117	Kittle and I	2010	40884	Mollusca	7805	Bivalvia
Late Pliocene		3.6	2.588	Ketcher an	1993	58350	Mollusca	7805	Bivalvia
Pliocene		5.333	2.588	DuBar	1971	17556	Mollusca	7805	Bivalvia
Zanclean		5.333	3.6	Campbell a	1995	17558	Mollusca	7805	Bivalvia
Late Pliocene		3.6	2.588	Cooke	1936	31145	Mollusca	7805	Bivalvia
Late Pliocene		3.6	2.588	Ward	1992	16985	Mollusca	7805	Bivalvia
Early Pleistocene		2.588	0.781	Miller	1990	17561	Mollusca	7805	Bivalvia
Early Pleistocene		2.588	0.781	Miller	1990	17561	Mollusca	7805	Bivalvia
Early Pleistocene		2.588	0.781	Miller	1990	17561	Mollusca	7805	Bivalvia
Late Pleistocene		0.126	0.0117	Richards	1962	18974	Mollusca	7805	Bivalvia
Late Pleistocene		0.126	0.0117	Richards	1962	18974	Mollusca	7805	Bivalvia
Pliocene		5.333	2.588	Hendy et a	2008	19909	Mollusca	7805	Bivalvia

Early Pleistocene	2.588	0.781	Hendy et a	2008	19909	Mollusca	7805	Bivalvia
Early Pleistocene	2.588	0.781	Hendy et a	2008	19909	Mollusca	7805	Bivalvia
Early Pliocene	5.333	3.6	Ward	1992	16985	Mollusca	7805	Bivalvia
Zanclean	5.333	3.6	Ward	1992	16985	Mollusca	7805	Bivalvia
Zanclean	5.333	3.6	Ward	1992	16985	Mollusca	7805	Bivalvia
Late Pliocene	3.6	2.588	Ward	1992	16985	Mollusca	7805	Bivalvia
Late Pliocene	3.6	2.588	Ward	1993	26351	Mollusca	7805	Bivalvia
Late Pliocene	3.6	2.588	Ward	1993	26351	Mollusca	7805	Bivalvia
Late Pliocene	3.6	2.588	Ward	1993	26351	Mollusca	7805	Bivalvia
Late Pliocene	3.6	2.588	Ward	1993	26351	Mollusca	7805	Bivalvia
Late Pliocene	3.6	2.588	Ward	1993	26351	Mollusca	7805	Bivalvia
Late Pliocene	3.6	2.588	Ward	1993	26351	Mollusca	7805	Bivalvia
Late Pliocene	3.6	2.588	Ward	1993	26351	Mollusca	7805	Bivalvia
Late Pliocene	3.6	2.588	Ward	1993	26351	Mollusca	7805	Bivalvia
Late Pliocene	3.6	2.588	Ward	1993	26351	Mollusca	7805	Bivalvia
Late Pliocene	3.6	2.588	Hendy et a	2008	19909	Mollusca	7805	Bivalvia
Late Pliocene	3.6	2.588	Hendy et a	2008	19909	Mollusca	7805	Bivalvia
Late Pliocene	3.6	2.588	Hendy et a	2008	19909	Mollusca	7805	Bivalvia
Late Pliocene	3.6	2.588	Hendy et a	2008	19909	Mollusca	7805	Bivalvia
Late Pliocene	3.6	2.588	Hendy et a	2008	19909	Mollusca	7805	Bivalvia
Middle Pleistocene	0.781	0.126	McGinty	1970	19114	Mollusca	7805	Bivalvia
Middle Pleistocene	0.781	0.126	McGinty	1970	19114	Mollusca	7805	Bivalvia
Early Pleistocene	2.588	0.781	Olsson anc	1953	25463	Mollusca	7805	Bivalvia
Early Pleistocene	2.588	0.781	Olsson anc	1953	25463	Mollusca	7805	Bivalvia
Late Pleistocene	0.126	0.0117	Hendy et a	2008	19909	Mollusca	7805	Bivalvia
Late Pleistocene	0.126	0.0117	Hendy et a	2008	19909	Mollusca	7805	Bivalvia
Late Pliocene	3.6	2.588	Ward and I	1987	16700	Mollusca	7805	Bivalvia
Early Pleistocene	2.588	0.781	Ward and I	1987	16700	Mollusca	7805	Bivalvia
Early Pleistocene	2.588	0.781	Gardner	1943	16984	Mollusca	7805	Bivalvia
Early Plioc Late Plioce	5.333	2.588	Gardner	1943	16984	Mollusca	7805	Bivalvia
Late Pliocene	3.6	2.588	Gardner	1943	16984	Mollusca	7805	Bivalvia
Pleistocene	2.588	0.0117	Gardner	1943	16984	Mollusca	7805	Bivalvia
Late Pliocene	3.6	2.588	Gardner	1943	16984	Mollusca	7805	Bivalvia
Early Pleistocene	2.588	0.781	Gardner	1943	16984	Mollusca	7805	Bivalvia
Pleistocene	2.588	0.0117	Gardner	1943	16984	Mollusca	7805	Bivalvia
Early Pleistocene	2.588	0.781	Miller	1990	17561	Mollusca	7805	Bivalvia
Late Pleistocene	0.126	0.0117	Spencer ar	1987	15525	Mollusca	7805	Bivalvia
Late Pleistocene	0.126	0.0117	Spencer ar	1987	15525	Mollusca	7805	Bivalvia
Late Pleistocene	0.126	0.0117	Spencer ar	1987	15525	Mollusca	7805	Bivalvia
Late Pleistocene	0.126	0.0117	Spencer ar	1987	15525	Mollusca	7805	Bivalvia
Late Pleistocene	0.126	0.0117	Spencer ar	1987	15525	Mollusca	7805	Bivalvia
Late Pleistocene	0.126	0.0117	Richards	1962	18974	Mollusca	7805	Bivalvia
Late Pliocene	3.6	2.588	Cooke	1936	31145	Mollusca	7805	Bivalvia
Late Pliocene	3.6	2.588	Ward and I	1987	16700	Mollusca	7805	Bivalvia
Early Pleistocene	2.588	0.781	Ward and I	1987	16700	Mollusca	7805	Bivalvia
Late Pliocene	3.6	2.588	Gardner	1943	16984	Mollusca	7805	Bivalvia
Gelasian	2.588	1.806	Bailey and	1986	23840	Mollusca	7805	Bivalvia

Early Pleistocene	2.588	0.781	Hendy et a	2008	19909	Mollusca	7805	Bivalvia
Late Pliocene	3.6	2.588	Hendy et a	2008	19909	Mollusca	7805	Bivalvia
Late Pliocene	3.6	2.588	Ward	1993	26351	Mollusca	7805	Bivalvia
Calabrian	1.806	0.781	DuBar	1962	11119	Mollusca	7805	Bivalvia
Late Pliocene	3.6	2.588	Mansfield	1932	17094	Mollusca	7805	Bivalvia
Early Pleistocene	2.588	0.781	Portell et a	1992	17634	Mollusca	7805	Bivalvia
Middle Pleistocene	0.781	0.126	Portell et a	1992	17634	Mollusca	7805	Bivalvia
Middle Pleistocene	0.781	0.126	Portell et a	1992	17634	Mollusca	7805	Bivalvia
Middle Pleistocene	0.781	0.126	Rosenberg	2005	19419	Mollusca	7805	Bivalvia
Middle Pleistocene	0.781	0.126	McGinty	1970	19114	Mollusca	7805	Bivalvia
Middle Pleistocene	0.781	0.126	McGinty	1970	19114	Mollusca	7805	Bivalvia
Middle Pleistocene	0.781	0.126	McGinty	1970	19114	Mollusca	7805	Bivalvia
Late Pliocene	3.6	2.588	Mansfield	1939	19112	Mollusca	7805	Bivalvia
Early Pleistocene	2.588	0.781	Olsson anc	1953	25463	Mollusca	7805	Bivalvia
Early Pleistocene	2.588	0.781	Olsson anc	1953	25463	Mollusca	7805	Bivalvia
Early Pleistocene	2.588	0.781	Olsson anc	1953	25463	Mollusca	7805	Bivalvia
Late Pliocene	3.6	2.588	DuBar	1958	25514	Mollusca	7805	Bivalvia
Early Pleistocene	2.588	0.781	Vokes	1973	31313	Mollusca	7805	Bivalvia
Late Pliocene	3.6	2.588	Vokes	1973	31313	Mollusca	7805	Bivalvia
Early Pleistocene	2.588	0.781	Vokes	1973	31313	Mollusca	7805	Bivalvia
Pliocene	5.333	2.588	Petuch	1986	47440	Mollusca	7805	Bivalvia
Zanclean	5.333	3.6	Campbell a	1995	17558	Mollusca	7805	Bivalvia
Late Pleistocene	0.126	0.0117	Hendy et a	2008	19909	Mollusca	7805	Bivalvia
Early Pleistocene	2.588	0.781	Hendy et a	2008	19909	Mollusca	7805	Bivalvia
Late Pleistocene	0.126	0.0117	Sellards	1917	9011	Mollusca	7805	Bivalvia
Late Pleistocene	0.126	0.0117	Sellards	1917	9011	Mollusca	7805	Bivalvia
Late Pleistocene	0.126	0.0117	Sellards	1917	9011	Mollusca	7805	Bivalvia
Late Pleistocene	0.126	0.0117	Sellards	1917	9011	Mollusca	7805	Bivalvia
Late Pleistocene	0.126	0.0117	Sellards	1917	9011	Mollusca	7805	Bivalvia
Early Pleistocene	2.588	0.781	Mansfield	1932	11083	Mollusca	7805	Bivalvia
Early Pleistocene	2.588	0.781	DuBar	1962	11119	Mollusca	7805	Bivalvia
Early Pleistocene	2.588	0.781	DuBar	1962	11119	Mollusca	7805	Bivalvia
Early Pleistocene	2.588	0.781	DuBar	1962	11119	Mollusca	7805	Bivalvia
Early Pleistocene	2.588	0.781	DuBar	1962	11119	Mollusca	7805	Bivalvia
Early Pleistocene	2.588	0.781	DuBar	1962	11119	Mollusca	7805	Bivalvia
Early Pleistocene	2.588	0.781	DuBar	1962	11119	Mollusca	7805	Bivalvia
Early Pleistocene	2.588	0.781	DuBar	1962	11119	Mollusca	7805	Bivalvia
Early Pleistocene	2.588	0.781	DuBar	1962	11119	Mollusca	7805	Bivalvia
Early Pleistocene	2.588	0.781	DuBar	1962	11119	Mollusca	7805	Bivalvia
Early Pleistocene	2.588	0.781	DuBar	1962	11119	Mollusca	7805	Bivalvia
Early Pleistocene	2.588	0.781	DuBar	1962	11119	Mollusca	7805	Bivalvia
Early Pleistocene	2.588	0.781	DuBar	1962	11119	Mollusca	7805	Bivalvia
Early Pleistocene	2.588	0.781	DuBar	1962	11119	Mollusca	7805	Bivalvia
Early Pleistocene	2.588	0.781	DuBar	1962	11119	Mollusca	7805	Bivalvia
Early Pleistocene	2.588	0.781	DuBar	1962	11119	Mollusca	7805	Bivalvia
Calabrian	1.806	0.781	DuBar	1962	11119	Mollusca	7805	Bivalvia
Calabrian	1.806	0.781	DuBar	1962	11119	Mollusca	7805	Bivalvia

Calabrian		1.806	0.781	DuBar	1962	11119	Mollusca	7805	Bivalvia
Calabrian		1.806	0.781	DuBar	1962	11119	Mollusca	7805	Bivalvia
Calabrian		1.806	0.781	Hendy et a	2008	19909	Mollusca	7805	Bivalvia
Calabrian		1.806	0.781	DuBar	1962	11119	Mollusca	7805	Bivalvia
Calabrian		1.806	0.781	DuBar	1962	11119	Mollusca	7805	Bivalvia
Calabrian		1.806	0.781	DuBar	1962	11119	Mollusca	7805	Bivalvia
Calabrian		1.806	0.781	DuBar	1962	11119	Mollusca	7805	Bivalvia
Calabrian		1.806	0.781	DuBar	1962	11119	Mollusca	7805	Bivalvia
Late Pleistocene		0.126	0.0117	DuBar	1962	11119	Mollusca	7805	Bivalvia
Late Pleistocene		0.126	0.0117	DuBar	1962	11119	Mollusca	7805	Bivalvia
Early Pleistocene		2.588	0.781	DuBar	1962	11119	Mollusca	7805	Bivalvia
Early Pleistocene		2.588	0.781	DuBar	1962	11119	Mollusca	7805	Bivalvia
Early Pleistocene		2.588	0.781	DuBar	1962	11119	Mollusca	7805	Bivalvia
Early Pleistocene		2.588	0.781	Hendy et a	2008	19909	Mollusca	7805	Bivalvia
Early Pleistocene		2.588	0.781	DuBar	1962	11119	Mollusca	7805	Bivalvia
Early Pleistocene		2.588	0.781	DuBar	1962	11119	Mollusca	7805	Bivalvia
Early Pleistocene		2.588	0.781	DuBar	1962	11119	Mollusca	7805	Bivalvia
Early Pleistocene		2.588	0.781	DuBar	1962	11119	Mollusca	7805	Bivalvia
Early Pleistocene		2.588	0.781	DuBar	1962	11119	Mollusca	7805	Bivalvia
Late Pleistocene		0.126	0.0117	DuBar	1962	11119	Mollusca	7805	Bivalvia
Pliocene	Pleistocene	5.333	0.0117	DuBar	1962	11119	Mollusca	7805	Bivalvia
Pliocene	Pleistocene	5.333	0.0117	Hendy et a	2008	19909	Mollusca	7805	Bivalvia
Pliocene	Pleistocene	5.333	0.0117	DuBar	1962	11119	Mollusca	7805	Bivalvia
Pliocene	Pleistocene	5.333	0.0117	DuBar	1962	11119	Mollusca	7805	Bivalvia
Pliocene	Pleistocene	5.333	0.0117	DuBar	1962	11119	Mollusca	7805	Bivalvia
Pliocene	Pleistocene	5.333	0.0117	DuBar	1962	11119	Mollusca	7805	Bivalvia
Pliocene	Pleistocene	5.333	0.0117	DuBar	1962	11119	Mollusca	7805	Bivalvia
Late Pliocene		3.6	2.588	Mansfield	1932	17094	Mollusca	7805	Bivalvia
Late Pliocene		3.6	2.588	Mansfield	1932	17094	Mollusca	7805	Bivalvia
Late Pliocene		3.6	2.588	Mansfield	1932	17094	Mollusca	7805	Bivalvia
Late Pliocene		3.6	2.588	Mansfield	1932	17094	Mollusca	7805	Bivalvia
Late Pliocene		3.6	2.588	Mansfield	1932	17094	Mollusca	7805	Bivalvia
Late Pliocene		3.6	2.588	Mansfield	1932	17094	Mollusca	7805	Bivalvia
Late Pliocene		3.6	2.588	Mansfield	1932	17094	Mollusca	7805	Bivalvia
Late Pliocene		3.6	2.588	Mansfield	1932	17094	Mollusca	7805	Bivalvia
Late Pliocene		3.6	2.588	Mansfield	1932	17094	Mollusca	7805	Bivalvia
Late Pliocene		3.6	2.588	Mansfield	1932	17094	Mollusca	7805	Bivalvia
Late Pliocene		3.6	2.588	Mansfield	1932	17094	Mollusca	7805	Bivalvia
Late Pliocene		3.6	2.588	Mansfield	1932	17094	Mollusca	7805	Bivalvia
Late Pliocene		3.6	2.588	Mansfield	1932	17094	Mollusca	7805	Bivalvia
Late Pliocene		3.6	2.588	Mansfield	1932	17094	Mollusca	7805	Bivalvia
Late Pliocene		3.6	2.588	Mansfield	1932	17094	Mollusca	7805	Bivalvia
Late Pliocene		3.6	2.588	Mansfield	1932	17094	Mollusca	7805	Bivalvia
Late Pliocene		3.6	2.588	Mansfield	1932	17094	Mollusca	7805	Bivalvia
Late Pliocene		3.6	2.588	Mansfield	1932	17094	Mollusca	7805	Bivalvia
Late Pliocene		3.6	2.588	Mansfield	1932	17094	Mollusca	7805	Bivalvia
Late Pliocene		3.6	2.588	Mansfield	1932	17094	Mollusca	7805	Bivalvia

Late Pliocene	3.6	2.588	Mansfield	1932	17094	Mollusca	7805	Bivalvia
Late Pliocene	3.6	2.588	Mansfield	1932	17094	Mollusca	7805	Bivalvia
Late Pliocene	3.6	2.588	Mansfield	1932	17094	Mollusca	7805	Bivalvia
Late Pliocene	3.6	2.588	Mansfield	1932	17094	Mollusca	7805	Bivalvia
Late Pliocene	3.6	2.588	Mansfield	1932	17094	Mollusca	7805	Bivalvia
Early Pleistocene	2.588	0.781	Hendy et a	2008	19909	Mollusca	7805	Bivalvia
Early Pleistocene	2.588	0.781	Portell et a	1992	17634	Mollusca	7805	Bivalvia
Early Pleistocene	2.588	0.781	Portell et a	1992	17634	Mollusca	7805	Bivalvia
Early Pleistocene	2.588	0.781	Portell et a	1992	17634	Mollusca	7805	Bivalvia
Early Pleistocene	2.588	0.781	Portell et a	1992	17634	Mollusca	7805	Bivalvia
Early Pleistocene	2.588	0.781	Portell et a	1992	17634	Mollusca	7805	Bivalvia
Early Pleistocene	2.588	0.781	Portell et a	1992	17634	Mollusca	7805	Bivalvia
Early Pleistocene	2.588	0.781	Portell et a	1992	17634	Mollusca	7805	Bivalvia
Middle Pleistocene	0.781	0.126	Portell et a	1992	17634	Mollusca	7805	Bivalvia
Middle Pleistocene	0.781	0.126	Portell et a	1992	17634	Mollusca	7805	Bivalvia
Middle Pleistocene	0.781	0.126	Portell et a	1992	17634	Mollusca	7805	Bivalvia
Middle Pleistocene	0.781	0.126	Portell et a	1992	17634	Mollusca	7805	Bivalvia
Middle Pleistocene	0.781	0.126	Portell et a	1992	17634	Mollusca	7805	Bivalvia
Late Pleistocene	0.126	0.0117	Portell et a	1992	17634	Mollusca	7805	Bivalvia
Late Pleistocene	0.126	0.0117	Portell et a	1992	17634	Mollusca	7805	Bivalvia
Late Pleistocene	0.126	0.0117	Portell et a	1992	17634	Mollusca	7805	Bivalvia
Late Pleistocene	0.126	0.0117	Portell et a	1992	17634	Mollusca	7805	Bivalvia
Late Pleistocene	0.126	0.0117	Portell et a	1992	17634	Mollusca	7805	Bivalvia
Late Pleistocene	0.126	0.0117	Portell et a	1992	17634	Mollusca	7805	Bivalvia
Middle Pleistocene	0.781	0.126	Hendy et a	2008	19909	Mollusca	7805	Bivalvia
Middle Pleistocene	0.781	0.126	McGinty	1970	19114	Mollusca	7805	Bivalvia
Middle Pleistocene	0.781	0.126	McGinty	1970	19114	Mollusca	7805	Bivalvia
Middle Pleistocene	0.781	0.126	McGinty	1970	19114	Mollusca	7805	Bivalvia
Middle Pleistocene	0.781	0.126	McGinty	1970	19114	Mollusca	7805	Bivalvia
Middle Pleistocene	0.781	0.126	McGinty	1970	19114	Mollusca	7805	Bivalvia
Middle Pleistocene	0.781	0.126	McGinty	1970	19114	Mollusca	7805	Bivalvia
Middle Pleistocene	0.781	0.126	McGinty	1970	19114	Mollusca	7805	Bivalvia
Middle Pleistocene	0.781	0.126	McGinty	1970	19114	Mollusca	7805	Bivalvia
Middle Pleistocene	0.781	0.126	McGinty	1970	19114	Mollusca	7805	Bivalvia
Middle Pleistocene	0.781	0.126	McGinty	1970	19114	Mollusca	7805	Bivalvia
Middle Pleistocene	0.781	0.126	McGinty	1970	19114	Mollusca	7805	Bivalvia
Late Pliocene	3.6	2.588	Mansfield	1939	19112	Mollusca	7805	Bivalvia
Late Pleistocene	0.126	0.0117	Hendy et a	2008	19909	Mollusca	7805	Bivalvia
Late Pleistocene	0.126	0.0117	Hendy et a	2008	19909	Mollusca	7805	Bivalvia
Late Pleistocene	0.126	0.0117	Hendy et a	2008	19909	Mollusca	7805	Bivalvia
Late Pleistocene	0.126	0.0117	Hendy et a	2008	19909	Mollusca	7805	Bivalvia
Late Pleistocene	0.126	0.0117	Hendy et a	2008	19909	Mollusca	7805	Bivalvia
Late Pleistocene	0.126	0.0117	Hendy et a	2008	19909	Mollusca	7805	Bivalvia
Late Pleistocene	0.126	0.0117	Hendy et a	2008	19909	Mollusca	7805	Bivalvia
Late Pleistocene	0.126	0.0117	Hendy et a	2008	19909	Mollusca	7805	Bivalvia
Late Pleistocene	0.126	0.0117	Hendy et a	2008	19909	Mollusca	7805	Bivalvia
Late Pleistocene	0.126	0.0117	Hendy et a	2008	19909	Mollusca	7805	Bivalvia

Early Pleistocene	2.588	0.781	Schroeder	1954	25303	Mollusca	7805	Bivalvia
Early Pleistocene	2.588	0.781	Schroeder	1954	25303	Mollusca	7805	Bivalvia
Early Pleistocene	2.588	0.781	Schroeder	1954	25303	Mollusca	7805	Bivalvia
Pliocene	5.333	2.588	Schroeder	1954	25303	Mollusca	7805	Bivalvia
Pliocene	5.333	2.588	Schroeder	1954	25303	Mollusca	7805	Bivalvia
Pliocene	5.333	2.588	Schroeder	1954	25303	Mollusca	7805	Bivalvia
Early Pleistocene	2.588	0.781	Schroeder	1954	25303	Mollusca	7805	Bivalvia
Early Pleistocene	2.588	0.781	Schroeder	1954	25303	Mollusca	7805	Bivalvia
Early Pleistocene	2.588	0.781	Schroeder	1954	25303	Mollusca	7805	Bivalvia
Pliocene	5.333	2.588	Schroeder	1954	25303	Mollusca	7805	Bivalvia
Pliocene	5.333	2.588	Schroeder	1954	25303	Mollusca	7805	Bivalvia
Pliocene	5.333	2.588	Schroeder	1954	25303	Mollusca	7805	Bivalvia
Pliocene	5.333	2.588	Schroeder	1954	25303	Mollusca	7805	Bivalvia
Pliocene	5.333	2.588	Schroeder	1954	25303	Mollusca	7805	Bivalvia
Pliocene	5.333	2.588	Schroeder	1954	25303	Mollusca	7805	Bivalvia
Pliocene	5.333	2.588	Schroeder	1954	25303	Mollusca	7805	Bivalvia
Pliocene	5.333	2.588	Schroeder	1954	25303	Mollusca	7805	Bivalvia
Early Pleistocene	2.588	0.781	Olsson anc	1953	25463	Mollusca	7805	Bivalvia
Early Pleistocene	2.588	0.781	Olsson anc	1953	25463	Mollusca	7805	Bivalvia
Early Pleistocene	2.588	0.781	Olsson anc	1953	25463	Mollusca	7805	Bivalvia
Early Pleistocene	2.588	0.781	Olsson anc	1953	25463	Mollusca	7805	Bivalvia
Early Pleistocene	2.588	0.781	Olsson anc	1953	25463	Mollusca	7805	Bivalvia
Early Pleistocene	2.588	0.781	Olsson anc	1953	25463	Mollusca	7805	Bivalvia
Early Pleistocene	2.588	0.781	Olsson anc	1953	25463	Mollusca	7805	Bivalvia
Early Pleistocene	2.588	0.781	Olsson anc	1953	25463	Mollusca	7805	Bivalvia
Early Pleistocene	2.588	0.781	Olsson anc	1953	25463	Mollusca	7805	Bivalvia
Early Pleistocene	2.588	0.781	Olsson anc	1953	25463	Mollusca	7805	Bivalvia
Early Pleistocene	2.588	0.781	Olsson anc	1953	25463	Mollusca	7805	Bivalvia
Early Pleistocene	2.588	0.781	Olsson anc	1953	25463	Mollusca	7805	Bivalvia
Early Pleistocene	2.588	0.781	Olsson anc	1953	25463	Mollusca	7805	Bivalvia
Early Pleistocene	2.588	0.781	Olsson anc	1953	25463	Mollusca	7805	Bivalvia
Early Pleistocene	2.588	0.781	Olsson anc	1953	25463	Mollusca	7805	Bivalvia
Early Pleistocene	2.588	0.781	Olsson anc	1953	25463	Mollusca	7805	Bivalvia
Early Pleistocene	2.588	0.781	Olsson anc	1953	25463	Mollusca	7805	Bivalvia
Early Pleistocene	2.588	0.781	Olsson anc	1953	25463	Mollusca	7805	Bivalvia
Early Pleistocene	2.588	0.781	Olsson anc	1953	25463	Mollusca	7805	Bivalvia
Early Pleistocene	2.588	0.781	Olsson anc	1953	25463	Mollusca	7805	Bivalvia
Early Pleistocene	2.588	0.781	Olsson anc	1953	25463	Mollusca	7805	Bivalvia
Early Pleistocene	2.588	0.781	Olsson anc	1953	25463	Mollusca	7805	Bivalvia
Late Pleistocene	0.126	0.0117	Petuch anc	2007	26813	Mollusca	7805	Bivalvia
Middle Pleistocene	0.781	0.126	Petuch anc	2007	26813	Mollusca	7805	Bivalvia
Middle Pleistocene	0.781	0.126	Petuch anc	2007	26813	Mollusca	7805	Bivalvia
Middle Pleistocene	0.781	0.126	Olsson anc	1964	26836	Mollusca	7805	Bivalvia
Late Pliocene	3.6	2.588	DuBar	1958	25514	Mollusca	7805	Bivalvia
Early Pleistocene	2.588	0.781	Hendy et a	2008	19909	Mollusca	7805	Bivalvia
Early Pleistocene	2.588	0.781	Hendy et a	2008	19909	Mollusca	7805	Bivalvia
Early Pleistocene	2.588	0.781	Hendy et a	2008	19909	Mollusca	7805	Bivalvia

Early Pleistocene	2.588	0.781	Hendy et a	2008	19909	Mollusca	7805	Bivalvia
Early Pleistocene	2.588	0.781	Hendy et a	2008	19909	Mollusca	7805	Bivalvia
Late Pleistocene	0.126	0.0117	Hendy et a	2008	19909	Mollusca	7805	Bivalvia
Early Pleistocene	2.588	0.781	DuBar	1958	25514	Mollusca	7805	Bivalvia
Late Pleistocene	0.126	0.0117	DuBar	1958	25514	Mollusca	7805	Bivalvia
Late Pleistocene	0.126	0.0117	DuBar	1958	25514	Mollusca	7805	Bivalvia
Late Pleistocene	0.126	0.0117	DuBar	1958	25514	Mollusca	7805	Bivalvia
Late Pleistocene	0.126	0.0117	Hendy et a	2008	19909	Mollusca	7805	Bivalvia
Early Pleistocene	2.588	0.781	Hendy et a	2008	19909	Mollusca	7805	Bivalvia
Early Pleistocene	2.588	0.781	Hendy et a	2008	19909	Mollusca	7805	Bivalvia
Early Pleistocene	2.588	0.781	Hendy et a	2008	19909	Mollusca	7805	Bivalvia
Early Pleistocene	2.588	0.781	Hendy et a	2008	19909	Mollusca	7805	Bivalvia
Early Pleistocene	2.588	0.781	Hendy et a	2008	19909	Mollusca	7805	Bivalvia
Early Pleistocene	2.588	0.781	Hendy et a	2008	19909	Mollusca	7805	Bivalvia
Early Pleistocene	2.588	0.781	Hendy et a	2008	19909	Mollusca	7805	Bivalvia
Early Pleistocene	2.588	0.781	Hendy et a	2008	19909	Mollusca	7805	Bivalvia
Early Pleistocene	2.588	0.781	Hendy et a	2008	19909	Mollusca	7805	Bivalvia
Early Pleistocene	2.588	0.781	Hendy et a	2008	19909	Mollusca	7805	Bivalvia
Pleistocene	2.588	0.0117	Hendy et a	2008	19909	Mollusca	7805	Bivalvia
Middle Pleistocene	0.781	0.126	Hendy et a	2008	19909	Mollusca	7805	Bivalvia
Middle Pleistocene	0.781	0.126	Hendy et a	2008	19909	Mollusca	7805	Bivalvia
Late Pleistocene	0.126	0.0117	Hendy et a	2008	19909	Mollusca	7805	Bivalvia
Early Pleistocene	2.588	0.781	Hendy et a	2008	19909	Mollusca	7805	Bivalvia
Early Pleistocene	2.588	0.781	Hendy et a	2008	19909	Mollusca	7805	Bivalvia
Early Pleistocene	2.588	0.781	DuBar	1958	25514	Mollusca	7805	Bivalvia
Early Pleistocene	2.588	0.781	DuBar	1958	25514	Mollusca	7805	Bivalvia
Early Pleistocene	2.588	0.781	DuBar	1958	25514	Mollusca	7805	Bivalvia
Late Pleistocene	0.126	0.0117	DuBar	1958	25514	Mollusca	7805	Bivalvia
Pleistocene	2.588	0.0117	Vokes	1969	31211	Mollusca	7805	Bivalvia
Early Pleistocene	2.588	0.781	Vokes	1969	31211	Mollusca	7805	Bivalvia
Early Pleistocene	2.588	0.781	Vokes	1969	31211	Mollusca	7805	Bivalvia
Early Pleistocene	2.588	0.781	Vokes	1969	31211	Mollusca	7805	Bivalvia
Early Pleistocene	2.588	0.781	Vokes	1969	31211	Mollusca	7805	Bivalvia
Early Pleistocene	2.588	0.781	Vokes	1969	31211	Mollusca	7805	Bivalvia
Pleistocene	2.588	0.0117	Vokes	1969	31211	Mollusca	7805	Bivalvia
Late Plioce Early Pleist	3.6	0.781	Vokes	1969	31211	Mollusca	7805	Bivalvia
Pleistocene	2.588	0.0117	Vokes	1969	31211	Mollusca	7805	Bivalvia
Early Pleistocene	2.588	0.781	Vokes	1969	31211	Mollusca	7805	Bivalvia
Late Pliocene	3.6	2.588	Vokes	1969	31211	Mollusca	7805	Bivalvia
Late Pliocene	3.6	2.588	Vokes	1969	31211	Mollusca	7805	Bivalvia
Late Pliocene	3.6	2.588	Vokes	1969	31211	Mollusca	7805	Bivalvia
Late Pliocene	3.6	2.588	Vokes	1969	31211	Mollusca	7805	Bivalvia
Late Pliocene	3.6	2.588	Vokes	1969	31211	Mollusca	7805	Bivalvia
Late Pliocene	3.6	2.588	Vokes	1969	31211	Mollusca	7805	Bivalvia
Early Pleistocene	2.588	0.781	Vokes	1969	31211	Mollusca	7805	Bivalvia
Late Plioce Early Pleist	3.6	0.781	Vokes	1969	31211	Mollusca	7805	Bivalvia
Late Pliocene	3.6	2.588	Vokes	1969	31211	Mollusca	7805	Bivalvia

Late Pliocene	3.6	2.588	Vokes	1969	31211	Mollusca	7805	Bivalvia
Late Pliocene	3.6	2.588	Vokes	1969	31211	Mollusca	7805	Bivalvia
Late Pliocene	3.6	2.588	Vokes	1969	31211	Mollusca	7805	Bivalvia
Late Pliocene	3.6	2.588	Vokes	1969	31211	Mollusca	7805	Bivalvia
Late Pliocene	3.6	2.588	Vokes	1969	31211	Mollusca	7805	Bivalvia
Late Pliocene	3.6	2.588	Vokes	1969	31211	Mollusca	7805	Bivalvia
Late Pliocene	3.6	2.588	Vokes	1969	31211	Mollusca	7805	Bivalvia
Late Pliocene	3.6	2.588	Vokes	1969	31211	Mollusca	7805	Bivalvia
Late Pliocene	3.6	2.588	Vokes	1969	31211	Mollusca	7805	Bivalvia
Late Pliocene	3.6	2.588	Vokes	1969	31211	Mollusca	7805	Bivalvia
Late Pleistocene	0.126	0.0117	Vokes	1969	31211	Mollusca	7805	Bivalvia
Late Pleistocene	0.126	0.0117	Vokes	1969	31211	Mollusca	7805	Bivalvia
Late Pleistocene	0.126	0.0117	Vokes	1969	31211	Mollusca	7805	Bivalvia
Late Pleistocene	0.126	0.0117	Vokes	1969	31211	Mollusca	7805	Bivalvia
Late Pleistocene	0.126	0.0117	Vokes	1969	31211	Mollusca	7805	Bivalvia
Late Pleistocene	0.126	0.0117	Vokes	1969	31211	Mollusca	7805	Bivalvia
Late Pliocene	3.6	2.588	Smith	1941	32333	Mollusca	7805	Bivalvia
Late Pliocene	3.6	2.588	Smith	1941	32333	Mollusca	7805	Bivalvia
Late Pliocene	3.6	2.588	Smith	1941	32333	Mollusca	7805	Bivalvia
Late Pliocene	3.6	2.588	Smith	1941	32333	Mollusca	7805	Bivalvia
Late Pleistocene	0.126	0.0117	Kittle and I	2010	40884	Mollusca	7805	Bivalvia
Late Pleistocene	0.126	0.0117	Kittle and I	2010	40884	Mollusca	7805	Bivalvia
Late Pleistocene	0.126	0.0117	Kittle and I	2010	40884	Mollusca	7805	Bivalvia
Late Pleistocene	0.126	0.0117	Kittle and I	2010	40884	Mollusca	7805	Bivalvia
Late Pleistocene	0.126	0.0117	Kittle and I	2010	40884	Mollusca	7805	Bivalvia
Late Pleistocene	0.126	0.0117	Kittle and I	2010	40884	Mollusca	7805	Bivalvia
Late Pleistocene	0.126	0.0117	Kittle and I	2010	40884	Mollusca	7805	Bivalvia
Late Pleistocene	0.126	0.0117	Kittle and I	2010	40884	Mollusca	7805	Bivalvia
Late Pleistocene	0.126	0.0117	Kittle and I	2010	40884	Mollusca	7805	Bivalvia
Late Pliocene	3.6	2.588	Ketcher an	1993	58350	Mollusca	7805	Bivalvia
Late Pleistocene	0.126	0.0117	Hulbert an	1998	1962	Mollusca	7805	Bivalvia
Late Pleistocene	0.126	0.0117	Hulbert an	1998	1962	Mollusca	7805	Bivalvia
Late Pleistocene	0.126	0.0117	DuBar	1971	17556	Mollusca	7805	Bivalvia
Late Pleistocene	0.126	0.0117	DuBar	1971	17556	Mollusca	7805	Bivalvia
Late Pleistocene	0.126	0.0117	DuBar	1971	17556	Mollusca	7805	Bivalvia
Late Pleistocene	0.126	0.0117	DuBar	1971	17556	Mollusca	7805	Bivalvia
Late Pleistocene	0.126	0.0117	DuBar	1971	17556	Mollusca	7805	Bivalvia
Late Pleistocene	0.126	0.0117	DuBar	1971	17556	Mollusca	7805	Bivalvia
Late Pleistocene	0.126	0.0117	DuBar	1971	17556	Mollusca	7805	Bivalvia
Late Pleistocene	0.126	0.0117	DuBar	1971	17556	Mollusca	7805	Bivalvia
Late Pleistocene	0.126	0.0117	DuBar	1971	17556	Mollusca	7805	Bivalvia
Zanclean	5.333	3.6	Campbell a	1995	17558	Mollusca	7805	Bivalvia
Zanclean	5.333	3.6	Campbell a	1995	17558	Mollusca	7805	Bivalvia
Zanclean	5.333	3.6	Campbell a	1995	17558	Mollusca	7805	Bivalvia
Zanclean	5.333	3.6	Campbell a	1995	17558	Mollusca	7805	Bivalvia
Zanclean	5.333	3.6	Campbell a	1995	17558	Mollusca	7805	Bivalvia
Zanclean	5.333	3.6	Campbell a	1995	17558	Mollusca	7805	Bivalvia

Early Pliocene	5.333	3.6	Campbell a	1995	17558	Mollusca	7805	Bivalvia
Early Pliocene	5.333	3.6	Campbell a	1995	17558	Mollusca	7805	Bivalvia
Early Pliocene	5.333	3.6	Campbell a	1995	17558	Mollusca	7805	Bivalvia
Early Pleistocene	2.588	0.781	DuBar	1971	17556	Mollusca	7805	Bivalvia
Early Pleistocene	2.588	0.781	DuBar	1971	17556	Mollusca	7805	Bivalvia
Early Pleistocene	2.588	0.781	DuBar	1971	17556	Mollusca	7805	Bivalvia
Late Pleistocene	0.126	0.0117	Richards	1962	18974	Mollusca	7805	Bivalvia
Late Pleistocene	0.126	0.0117	Richards	1962	18974	Mollusca	7805	Bivalvia
Late Pleistocene	0.126	0.0117	Richards	1962	18974	Mollusca	7805	Bivalvia
Late Pleistocene	0.126	0.0117	Richards	1962	18974	Mollusca	7805	Bivalvia
Late Pleistocene	0.126	0.0117	Richards	1962	18974	Mollusca	7805	Bivalvia
Late Pleistocene	0.126	0.0117	Richards	1962	18974	Mollusca	7805	Bivalvia
Late Pleistocene	0.126	0.0117	Richards	1962	18974	Mollusca	7805	Bivalvia
Late Pleistocene	0.126	0.0117	Richards	1962	18974	Mollusca	7805	Bivalvia
Late Pleistocene	0.126	0.0117	Richards	1962	18974	Mollusca	7805	Bivalvia
Late Pleistocene	0.126	0.0117	Richards	1962	18974	Mollusca	7805	Bivalvia
Late Pleistocene	0.126	0.0117	Richards	1962	18974	Mollusca	7805	Bivalvia
Late Pleistocene	0.126	0.0117	Richards	1962	18974	Mollusca	7805	Bivalvia
Late Pleistocene	0.126	0.0117	Hendy et a	2008	19909	Mollusca	7805	Bivalvia
Late Pleistocene	0.126	0.0117	Hendy et a	2008	19909	Mollusca	7805	Bivalvia
Late Pleistocene	0.126	0.0117	Hendy et a	2008	19909	Mollusca	7805	Bivalvia
Late Pleistocene	0.126	0.0117	Hendy et a	2008	19909	Mollusca	7805	Bivalvia
Late Pleistocene	0.126	0.0117	Hendy et a	2008	19909	Mollusca	7805	Bivalvia
Late Pleistocene	0.126	0.0117	Hendy et a	2008	19909	Mollusca	7805	Bivalvia
Late Pleistocene	0.126	0.0117	Hendy et a	2008	19909	Mollusca	7805	Bivalvia
Late Pleistocene	0.126	0.0117	Hendy et a	2008	19909	Mollusca	7805	Bivalvia
Late Pleistocene	0.126	0.0117	Hendy et a	2008	19909	Mollusca	7805	Bivalvia
Pleistocene	2.588	0.0117	Hendy et a	2008	19909	Mollusca	7805	Bivalvia
Pleistocene	2.588	0.0117	Hendy et a	2008	19909	Mollusca	7805	Bivalvia
Pleistocene	2.588	0.0117	Hendy et a	2008	19909	Mollusca	7805	Bivalvia
Pleistocene	2.588	0.0117	Hendy et a	2008	19909	Mollusca	7805	Bivalvia
Pleistocene	2.588	0.0117	Hendy et a	2008	19909	Mollusca	7805	Bivalvia
Pleistocene	2.588	0.0117	Hendy et a	2008	19909	Mollusca	7805	Bivalvia
Early Pleistocene	2.588	0.781	Cooke	1936	31145	Mollusca	7805	Bivalvia
Early Pleistocene	2.588	0.781	Cooke	1936	31145	Mollusca	7805	Bivalvia
Early Pleistocene	2.588	0.781	Cooke	1936	31145	Mollusca	7805	Bivalvia
Early Pleistocene	2.588	0.781	Cooke	1936	31145	Mollusca	7805	Bivalvia
Late Pliocene	3.6	2.588	Cooke	1936	31145	Mollusca	7805	Bivalvia
Late Pliocene	3.6	2.588	Cooke	1936	31145	Mollusca	7805	Bivalvia
Late Pliocene	3.6	2.588	Cooke	1936	31145	Mollusca	7805	Bivalvia
Late Pliocene	3.6	2.588	Cooke	1936	31145	Mollusca	7805	Bivalvia
Late Pliocene	3.6	2.588	Cooke	1936	31145	Mollusca	7805	Bivalvia
Pleistocene	2.588	0.0117	Holmes	1860	64712	Mollusca	7805	Bivalvia
Pleistocene	2.588	0.0117	Holmes	1860	64712	Mollusca	7805	Bivalvia
Pleistocene	2.588	0.0117	Holmes	1860	64712	Mollusca	7805	Bivalvia
Late Pliocene	3.6	2.588	Ward and I	1987	16700	Mollusca	7805	Bivalvia
Early Pleistocene	2.588	0.781	Ward and I	1987	16700	Mollusca	7805	Bivalvia

Late Pliocene	3.6	2.588	Ward and I	1987	16700	Mollusca	7805	Bivalvia
Early Pleistocene	2.588	0.781	Ward and I	1987	16700	Mollusca	7805	Bivalvia
Early Pleistocene	2.588	0.781	Ward and I	1987	16700	Mollusca	7805	Bivalvia
Late Pliocene	3.6	2.588	Gardner	1943	16984	Mollusca	7805	Bivalvia
Late Pliocene	3.6	2.588	Gardner	1943	16984	Mollusca	7805	Bivalvia
Late Pliocene	3.6	2.588	Gardner	1943	16984	Mollusca	7805	Bivalvia
Pleistocene	2.588	0.0117	Gardner	1943	16984	Mollusca	7805	Bivalvia
Late Pliocene	3.6	2.588	Gardner	1943	16984	Mollusca	7805	Bivalvia
Late Pliocene	3.6	2.588	Gardner	1943	16984	Mollusca	7805	Bivalvia
Early Pleistocene	2.588	0.781	Gardner	1943	16984	Mollusca	7805	Bivalvia
Late Pliocene	3.6	2.588	Gardner	1943	16984	Mollusca	7805	Bivalvia
Early Plioc Late Plioce	5.333	2.588	Gardner	1943	16984	Mollusca	7805	Bivalvia
Late Pliocene	3.6	2.588	Gardner	1943	16984	Mollusca	7805	Bivalvia
Early Pleistocene	2.588	0.781	Gardner	1943	16984	Mollusca	7805	Bivalvia
Late Pliocene	3.6	2.588	Gardner	1943	16984	Mollusca	7805	Bivalvia
Pleistocene	2.588	0.0117	Gardner	1943	16984	Mollusca	7805	Bivalvia
Early Pleistocene	2.588	0.781	Gardner	1943	16984	Mollusca	7805	Bivalvia
Early Pleistocene	2.588	0.781	Gardner	1943	16984	Mollusca	7805	Bivalvia
Early Pleistocene	2.588	0.781	Gardner	1943	16984	Mollusca	7805	Bivalvia
Early Pleistocene	2.588	0.781	Miller	1990	17561	Mollusca	7805	Bivalvia
Early Pleistocene	2.588	0.781	Miller	1990	17561	Mollusca	7805	Bivalvia
Late Pleistocene	0.126	0.0117	Richards	1962	18974	Mollusca	7805	Bivalvia
Late Pleistocene	0.126	0.0117	Richards	1962	18974	Mollusca	7805	Bivalvia
Late Pleistocene	0.126	0.0117	Richards	1962	18974	Mollusca	7805	Bivalvia
Late Pleistocene	0.126	0.0117	Richards	1962	18974	Mollusca	7805	Bivalvia
Late Pleistocene	0.126	0.0117	Richards	1962	18974	Mollusca	7805	Bivalvia
Late Pleistocene	0.126	0.0117	Richards	1962	18974	Mollusca	7805	Bivalvia
Late Pleistocene	0.126	0.0117	Richards	1962	18974	Mollusca	7805	Bivalvia
Late Pleistocene	0.126	0.0117	Richards	1962	18974	Mollusca	7805	Bivalvia
Late Pleistocene	0.126	0.0117	Richards	1962	18974	Mollusca	7805	Bivalvia
Late Pleistocene	0.126	0.0117	Richards	1962	18974	Mollusca	7805	Bivalvia
Late Pleistocene	0.126	0.0117	Richards	1962	18974	Mollusca	7805	Bivalvia
Late Pleistocene	0.126	0.0117	Richards	1962	18974	Mollusca	7805	Bivalvia
Late Pleistocene	0.126	0.0117	Richards	1962	18974	Mollusca	7805	Bivalvia
Late Pleistocene	0.126	0.0117	Richards	1962	18974	Mollusca	7805	Bivalvia
Late Pleistocene	0.126	0.0117	Richards	1962	18974	Mollusca	7805	Bivalvia
Late Pleistocene	0.126	0.0117	Hendy et a	2008	19909	Mollusca	7805	Bivalvia
Late Pleistocene	0.126	0.0117	Hendy et a	2008	19909	Mollusca	7805	Bivalvia
Late Pleistocene	0.126	0.0117	Hendy et a	2008	19909	Mollusca	7805	Bivalvia
Late Pleistocene	0.126	0.0117	Hendy et a	2008	19909	Mollusca	7805	Bivalvia
Gelasian	2.588	1.806	Bailey and	1986	23840	Mollusca	7805	Bivalvia
Gelasian	2.588	1.806	Bailey and	1986	23840	Mollusca	7805	Bivalvia
Gelasian	2.588	1.806	Bailey and	1986	23840	Mollusca	7805	Bivalvia
Early Pleistocene	2.588	0.781	Hendy et a	2008	19909	Mollusca	7805	Bivalvia
Early Pleistocene	2.588	0.781	Hendy et a	2008	19909	Mollusca	7805	Bivalvia
Late Pleistocene	0.126	0.0117	Hendy et a	2008	19909	Mollusca	7805	Bivalvia
Pliocene	5.333	2.588	Hendy et a	2008	19909	Mollusca	7805	Bivalvia

Pliocene	5.333	2.588	Hendy et a	2008	19909	Mollusca	7805	Bivalvia
Pliocene	5.333	2.588	Hendy et a	2008	19909	Mollusca	7805	Bivalvia
Early Pleistocene	2.588	0.781	Hendy et a	2008	19909	Mollusca	7805	Bivalvia
Late Pliocene	3.6	2.588	Hendy et a	2008	19909	Mollusca	7805	Bivalvia
Early Pleistocene	2.588	0.781	Hendy et a	2008	19909	Mollusca	7805	Bivalvia
Early Pleistocene	2.588	0.781	Hendy et a	2008	19909	Mollusca	7805	Bivalvia
Early Pleistocene	2.588	0.781	Hendy et a	2008	19909	Mollusca	7805	Bivalvia
Early Pleistocene	2.588	0.781	Hendy et a	2008	19909	Mollusca	7805	Bivalvia
Early Pleistocene	2.588	0.781	Hendy et a	2008	19909	Mollusca	7805	Bivalvia
Early Pleistocene	2.588	0.781	Hendy et a	2008	19909	Mollusca	7805	Bivalvia
Early Pleistocene	2.588	0.781	Hendy et a	2008	19909	Mollusca	7805	Bivalvia
Early Pleistocene	2.588	0.781	Hendy et a	2008	19909	Mollusca	7805	Bivalvia
Early Pleistocene	2.588	0.781	Hendy et a	2008	19909	Mollusca	7805	Bivalvia
Early Pleistocene	2.588	0.781	Hendy et a	2008	19909	Mollusca	7805	Bivalvia
Early Pleistocene	2.588	0.781	Hendy et a	2008	19909	Mollusca	7805	Bivalvia
Early Pleistocene	2.588	0.781	Hendy et a	2008	19909	Mollusca	7805	Bivalvia
Early Pleistocene	2.588	0.781	Hendy et a	2008	19909	Mollusca	7805	Bivalvia
Early Pleistocene	2.588	0.781	Hendy et a	2008	19909	Mollusca	7805	Bivalvia
Early Pleistocene	2.588	0.781	Hendy et a	2008	19909	Mollusca	7805	Bivalvia
Early Pleistocene	2.588	0.781	Hendy et a	2008	19909	Mollusca	7805	Bivalvia
Early Pleistocene	2.588	0.781	Hendy et a	2008	19909	Mollusca	7805	Bivalvia
Early Pleistocene	2.588	0.781	Hendy et a	2008	19909	Mollusca	7805	Bivalvia
Early Pleistocene	2.588	0.781	Hendy et a	2008	19909	Mollusca	7805	Bivalvia
Early Pleistocene	2.588	0.781	Hendy et a	2008	19909	Mollusca	7805	Bivalvia
Late Pleistocene	0.126	0.0117	Spencer ar	1987	15525	Mollusca	7805	Bivalvia
Late Pleistocene	0.126	0.0117	Spencer ar	1987	15525	Mollusca	7805	Bivalvia
Late Pleistocene	0.126	0.0117	Spencer ar	1987	15525	Mollusca	7805	Bivalvia
Late Pleistocene	0.126	0.0117	Spencer ar	1987	15525	Mollusca	7805	Bivalvia
Early Pliocene	5.333	3.6	Ward	1992	16985	Mollusca	7805	Bivalvia
Early Pliocene	5.333	3.6	Ward	1992	16985	Mollusca	7805	Bivalvia
Pliocene	5.333	2.588	Gardner	1943	16984	Mollusca	7805	Bivalvia
Late Pliocene	3.6	2.588	Edwards et	2005	17631	Mollusca	7805	Bivalvia
Late Pliocene	3.6	2.588	Edwards et	2005	17631	Mollusca	7805	Bivalvia
Late Pliocene	3.6	2.588	Edwards et	2005	17631	Mollusca	7805	Bivalvia
Late Pliocene	3.6	2.588	Edwards et	2005	17631	Mollusca	7805	Bivalvia
Zanclean	5.333	3.6	Ward	1992	16985	Mollusca	7805	Bivalvia
Zanclean	5.333	3.6	Ward	1992	16985	Mollusca	7805	Bivalvia
Late Pliocene	3.6	2.588	Ward	1993	26351	Mollusca	7805	Bivalvia
Late Pliocene	3.6	2.588	Ward	1993	26351	Mollusca	7805	Bivalvia
Late Pliocene	3.6	2.588	Ward	1993	26351	Mollusca	7805	Bivalvia
Late Pliocene	3.6	2.588	Ward	1993	26351	Mollusca	7805	Bivalvia
Late Pliocene	3.6	2.588	Ward	1993	26351	Mollusca	7805	Bivalvia
Late Pliocene	3.6	2.588	Ward	1993	26351	Mollusca	7805	Bivalvia
Late Pliocene	3.6	2.588	Ward	1993	26351	Mollusca	7805	Bivalvia
Late Pliocene	3.6	2.588	Ward	1993	26351	Mollusca	7805	Bivalvia
Late Pliocene	3.6	2.588	Ward	1993	26351	Mollusca	7805	Bivalvia
Late Pliocene	3.6	2.588	Hendy et a	2008	19909	Mollusca	7805	Bivalvia

Late Pliocene		3.6	2.588	Hendy et a	2008	19909	Mollusca	7805 Bivalvia
Late Pliocene		3.6	2.588	Hendy et a	2008	19909	Mollusca	7805 Bivalvia
Late Pliocene		3.6	2.588	Hendy et a	2008	19909	Mollusca	7805 Bivalvia
Late Pliocene		3.6	2.588	Hendy et a	2008	19909	Mollusca	7805 Bivalvia
Late Pliocene		3.6	2.588	Hendy et a	2008	19909	Mollusca	7805 Bivalvia
Late Pliocene		3.6	2.588	Hendy et a	2008	19909	Mollusca	7805 Bivalvia
Late Pliocene		3.6	2.588	Hendy et a	2008	19909	Mollusca	7805 Bivalvia
Late Pliocene		3.6	2.588	Hendy et a	2008	19909	Mollusca	7805 Bivalvia
Late Pleistocene		0.126	0.0117	Spencer ar	1987	15525	Mollusca	7805 Bivalvia
Late Pleistocene		0.126	0.0117	Sellards	1917	9011	Mollusca	7805 Bivalvia
Late Pleistocene		0.126	0.0117	Sellards	1917	9011	Mollusca	7805 Bivalvia
Late Pleistocene		0.126	0.0117	Sellards	1917	9011	Mollusca	7805 Bivalvia
Late Pleistocene		0.126	0.0117	Sellards	1917	9011	Mollusca	7805 Bivalvia
Early Pleistocene		2.588	0.781	Mansfield	1932	11083	Mollusca	7805 Bivalvia
Early Pleistocene		2.588	0.781	DuBar	1962	11119	Mollusca	7805 Bivalvia
Early Pleistocene		2.588	0.781	DuBar	1962	11119	Mollusca	7805 Bivalvia
Early Pleistocene		2.588	0.781	DuBar	1962	11119	Mollusca	7805 Bivalvia
Early Pleistocene		2.588	0.781	DuBar	1962	11119	Mollusca	7805 Bivalvia
Early Pleistocene		2.588	0.781	DuBar	1962	11119	Mollusca	7805 Bivalvia
Early Pleistocene		2.588	0.781	DuBar	1962	11119	Mollusca	7805 Bivalvia
Calabrian		1.806	0.781	DuBar	1962	11119	Mollusca	7805 Bivalvia
Late Pleistocene		0.126	0.0117	DuBar	1962	11119	Mollusca	7805 Bivalvia
Early Pleistocene		2.588	0.781	DuBar	1962	11119	Mollusca	7805 Bivalvia
Early Pleistocene		2.588	0.781	DuBar	1962	11119	Mollusca	7805 Bivalvia
Early Pleistocene		2.588	0.781	DuBar	1962	11119	Mollusca	7805 Bivalvia
Late Pleistocene		0.126	0.0117	DuBar	1962	11119	Mollusca	7805 Bivalvia
Pliocene	Pleistocene	5.333	0.0117	DuBar	1962	11119	Mollusca	7805 Bivalvia
Pliocene	Pleistocene	5.333	0.0117	DuBar	1962	11119	Mollusca	7805 Bivalvia
Pliocene	Pleistocene	5.333	0.0117	DuBar	1962	11119	Mollusca	7805 Bivalvia
Late Pliocene		3.6	2.588	Mansfield	1932	17094	Mollusca	7805 Bivalvia
Late Pliocene		3.6	2.588	Mansfield	1932	17094	Mollusca	7805 Bivalvia
Late Pliocene		3.6	2.588	Mansfield	1932	17094	Mollusca	7805 Bivalvia
Late Pliocene		3.6	2.588	Mansfield	1932	17094	Mollusca	7805 Bivalvia
Late Pliocene		3.6	2.588	Mansfield	1932	17094	Mollusca	7805 Bivalvia
Late Pliocene		3.6	2.588	Mansfield	1932	17094	Mollusca	7805 Bivalvia
Late Pliocene		3.6	2.588	Mansfield	1932	17094	Mollusca	7805 Bivalvia
Late Pliocene		3.6	2.588	Mansfield	1932	17094	Mollusca	7805 Bivalvia
Late Pliocene		3.6	2.588	Mansfield	1932	17094	Mollusca	7805 Bivalvia
Early Pleistocene		2.588	0.781	Portell et a	1992	17634	Mollusca	7805 Bivalvia
Middle Pleistocene		0.781	0.126	Portell et a	1992	17634	Mollusca	7805 Bivalvia
Middle Pleistocene		0.781	0.126	Portell et a	1992	17634	Mollusca	7805 Bivalvia
Late Pleistocene		0.126	0.0117	Portell et a	1992	17634	Mollusca	7805 Bivalvia
Late Pleistocene		0.126	0.0117	Portell et a	1992	17634	Mollusca	7805 Bivalvia
Early Pleistocene		2.588	0.781	Portell et a	1992	17634	Mollusca	7805 Bivalvia
Early Pleistocene		2.588	0.781	Portell et a	1992	17634	Mollusca	7805 Bivalvia
Middle Pleistocene		0.781	0.126	Hoerle	1970	19536	Mollusca	7805 Bivalvia
Middle Pleistocene		0.781	0.126	Hoerle	1970	19536	Mollusca	7805 Bivalvia

Middle Pleistocene	0.781	0.126	Hoerle	1970	19536	Mollusca	7805	Bivalvia
Late Pleistocene	0.126	0.0117	Hendy et a	2008	19909	Mollusca	7805	Bivalvia
Late Pleistocene	0.126	0.0117	Hendy et a	2008	19909	Mollusca	7805	Bivalvia
Late Pleistocene	0.126	0.0117	Hendy et a	2008	19909	Mollusca	7805	Bivalvia
Pliocene	5.333	2.588	Schroeder	1954	25303	Mollusca	7805	Bivalvia
Early Pleistocene	2.588	0.781	Schroeder	1954	25303	Mollusca	7805	Bivalvia
Early Pleistocene	2.588	0.781	Schroeder	1954	25303	Mollusca	7805	Bivalvia
Pliocene	5.333	2.588	Schroeder	1954	25303	Mollusca	7805	Bivalvia
Pliocene	5.333	2.588	Schroeder	1954	25303	Mollusca	7805	Bivalvia
Pliocene	5.333	2.588	Schroeder	1954	25303	Mollusca	7805	Bivalvia
Pliocene	5.333	2.588	Schroeder	1954	25303	Mollusca	7805	Bivalvia
Pliocene	5.333	2.588	Schroeder	1954	25303	Mollusca	7805	Bivalvia
Early Pleistocene	2.588	0.781	Olsson anc	1953	25463	Mollusca	7805	Bivalvia
Early Pleistocene	2.588	0.781	Olsson anc	1953	25463	Mollusca	7805	Bivalvia
Early Pleistocene	2.588	0.781	Olsson anc	1953	25463	Mollusca	7805	Bivalvia
Early Pleistocene	2.588	0.781	Olsson anc	1953	25463	Mollusca	7805	Bivalvia
Early Pleistocene	2.588	0.781	Olsson anc	1953	25463	Mollusca	7805	Bivalvia
Early Pleistocene	2.588	0.781	Olsson anc	1953	25463	Mollusca	7805	Bivalvia
Early Pleistocene	2.588	0.781	Olsson anc	1953	25463	Mollusca	7805	Bivalvia
Early Pleistocene	2.588	0.781	Olsson anc	1953	25463	Mollusca	7805	Bivalvia
Late Pleistocene	0.126	0.0117	Petuch anc	2007	26813	Mollusca	7805	Bivalvia
Early Pleistocene	2.588	0.781	Petuch anc	2007	26813	Mollusca	7805	Bivalvia
Early Pleistocene	2.588	0.781	Petuch anc	2007	26813	Mollusca	7805	Bivalvia
Late Pliocene	3.6	2.588	Olsson	1967	19826	Mollusca	7805	Bivalvia
Late Pliocene	3.6	2.588	Olsson	1967	19826	Mollusca	7805	Bivalvia
Late Plioce Pleistocen	3.6	0.0117	Petuch anc	2007	26813	Mollusca	7805	Bivalvia
Late Pliocene	3.6	2.588	Petuch anc	2007	26813	Mollusca	7805	Bivalvia
Early Pleistocene	2.588	0.781	Hendy et a	2008	19909	Mollusca	7805	Bivalvia
Late Pleistocene	0.126	0.0117	Hendy et a	2008	19909	Mollusca	7805	Bivalvia
Late Pleistocene	0.126	0.0117	Hendy et a	2008	19909	Mollusca	7805	Bivalvia
Early Pliocene	5.333	3.6	Petuch anc	2007	26813	Mollusca	7805	Bivalvia
Late Pleistocene	0.126	0.0117	DuBar	1958	25514	Mollusca	7805	Bivalvia
Late Pliocene	3.6	2.588	Hendy et a	2008	19909	Mollusca	7805	Bivalvia
Early Pleistocene	2.588	0.781	Hendy et a	2008	19909	Mollusca	7805	Bivalvia
Early Pleistocene	2.588	0.781	Hendy et a	2008	19909	Mollusca	7805	Bivalvia
Early Pleistocene	2.588	0.781	Hendy et a	2008	19909	Mollusca	7805	Bivalvia
Early Pleistocene	2.588	0.781	Hendy et a	2008	19909	Mollusca	7805	Bivalvia
Pleistocene	2.588	0.0117	Hendy et a	2008	19909	Mollusca	7805	Bivalvia
Pleistocene	2.588	0.0117	Hendy et a	2008	19909	Mollusca	7805	Bivalvia
Late Pliocene	3.6	2.588	Hendy et a	2008	19909	Mollusca	7805	Bivalvia
Late Pliocene	3.6	2.588	Hendy et a	2008	19909	Mollusca	7805	Bivalvia
Late Pliocene	3.6	2.588	Hendy et a	2008	19909	Mollusca	7805	Bivalvia
Middle Pleistocene	0.781	0.126	Hendy et a	2008	19909	Mollusca	7805	Bivalvia
Middle Pleistocene	0.781	0.126	Hendy et a	2008	19909	Mollusca	7805	Bivalvia
Middle Pleistocene	0.781	0.126	Hendy et a	2008	19909	Mollusca	7805	Bivalvia
Late Pleistocene	0.126	0.0117	Hendy et a	2008	19909	Mollusca	7805	Bivalvia
Late Pleistocene	0.126	0.0117	Hendy et a	2008	19909	Mollusca	7805	Bivalvia

Late Pleistocene	0.126	0.0117	Hendy et a	2008	19909	Mollusca	7805	Bivalvia
Late Pleistocene	0.126	0.0117	Hendy et a	2008	19909	Mollusca	7805	Bivalvia
Late Pleistocene	0.126	0.0117	Hendy et a	2008	19909	Mollusca	7805	Bivalvia
Early Pleistocene	2.588	0.781	Hendy et a	2008	19909	Mollusca	7805	Bivalvia
Early Pleistocene	2.588	0.781	Hendy et a	2008	19909	Mollusca	7805	Bivalvia
Early Pleistocene	2.588	0.781	Hendy et a	2008	19909	Mollusca	7805	Bivalvia
Early Pleistocene	2.588	0.781	Hendy et a	2008	19909	Mollusca	7805	Bivalvia
Early Pleistocene	2.588	0.781	DuBar	1958	25514	Mollusca	7805	Bivalvia
Late Pleistocene	0.126	0.0117	Kittle and I	2010	40884	Mollusca	7805	Bivalvia
Late Pleistocene	0.126	0.0117	Kittle and I	2010	40884	Mollusca	7805	Bivalvia
Late Pleistocene	0.126	0.0117	Kittle and I	2010	40884	Mollusca	7805	Bivalvia
Late Pliocene	3.6	2.588	Ketcher an	1993	58350	Mollusca	7805	Bivalvia
Late Pleistocene	0.126	0.0117	Hulbert an	1998	1962	Mollusca	7805	Bivalvia
Late Pleistocene	0.126	0.0117	Hulbert an	1998	1962	Mollusca	7805	Bivalvia
Late Pleistocene	0.126	0.0117	Hulbert an	1998	1962	Mollusca	7805	Bivalvia
Late Pleistocene	0.126	0.0117	Richards	1962	18974	Mollusca	7805	Bivalvia
Pliocene	5.333	2.588	Aldrich	1911	53829	Mollusca	7805	Bivalvia
Pliocene	5.333	2.588	Aldrich	1911	53829	Mollusca	7805	Bivalvia
Pliocene	5.333	2.588	Aldrich	1911	53829	Mollusca	7805	Bivalvia
Late Pleistocene	0.126	0.0117	DuBar	1971	17556	Mollusca	7805	Bivalvia
Late Pleistocene	0.126	0.0117	DuBar	1971	17556	Mollusca	7805	Bivalvia
Late Pleistocene	0.126	0.0117	DuBar	1971	17556	Mollusca	7805	Bivalvia
Late Pleistocene	0.126	0.0117	DuBar	1971	17556	Mollusca	7805	Bivalvia
Late Pleistocene	0.126	0.0117	DuBar	1971	17556	Mollusca	7805	Bivalvia
Late Pleistocene	0.126	0.0117	DuBar	1971	17556	Mollusca	7805	Bivalvia
Late Pleistocene	0.126	0.0117	DuBar	1971	17556	Mollusca	7805	Bivalvia
Late Pleistocene	0.126	0.0117	DuBar	1971	17556	Mollusca	7805	Bivalvia
Late Pleistocene	0.126	0.0117	DuBar	1971	17556	Mollusca	7805	Bivalvia
Zanclean	5.333	3.6	Hendy et a	2008	19909	Mollusca	7805	Bivalvia
Zanclean	5.333	3.6	Campbell a	1995	17558	Mollusca	7805	Bivalvia
Early Pleistocene	2.588	0.781	DuBar	1971	17556	Mollusca	7805	Bivalvia
Early Pleistocene	2.588	0.781	Hendy et a	2008	19909	Mollusca	7805	Bivalvia
Late Pleistocene	0.126	0.0117	Richards	1962	18974	Mollusca	7805	Bivalvia
Late Pleistocene	0.126	0.0117	Richards	1962	18974	Mollusca	7805	Bivalvia
Late Pleistocene	0.126	0.0117	Richards	1962	18974	Mollusca	7805	Bivalvia
Late Pleistocene	0.126	0.0117	Richards	1962	18974	Mollusca	7805	Bivalvia
Late Pleistocene	0.126	0.0117	Richards	1962	18974	Mollusca	7805	Bivalvia
Late Pleistocene	0.126	0.0117	Richards	1962	18974	Mollusca	7805	Bivalvia
Late Pleistocene	0.126	0.0117	Richards	1962	18974	Mollusca	7805	Bivalvia
Late Pleistocene	0.126	0.0117	Richards	1962	18974	Mollusca	7805	Bivalvia
Late Pleistocene	0.126	0.0117	Hendy et a	2008	19909	Mollusca	7805	Bivalvia
Late Pleistocene	0.126	0.0117	Hendy et a	2008	19909	Mollusca	7805	Bivalvia
Late Pleistocene	0.126	0.0117	Hendy et a	2008	19909	Mollusca	7805	Bivalvia
Late Pleistocene	0.126	0.0117	Hendy et a	2008	19909	Mollusca	7805	Bivalvia
Late Pleistocene	0.126	0.0117	Hendy et a	2008	19909	Mollusca	7805	Bivalvia
Late Pleistocene	0.126	0.0117	Hendy et a	2008	19909	Mollusca	7805	Bivalvia
Late Pleistocene	0.126	0.0117	Hendy et a	2008	19909	Mollusca	7805	Bivalvia

Late Pleistocene		0.126	0.0117	Hendy et a	2008	19909	Mollusca	7805	Bivalvia
Late Pleistocene		0.126	0.0117	Hendy et a	2008	19909	Mollusca	7805	Bivalvia
Pleistocene		2.588	0.0117	Hendy et a	2008	19909	Mollusca	7805	Bivalvia
Pleistocene		2.588	0.0117	Hendy et a	2008	19909	Mollusca	7805	Bivalvia
Pleistocene		2.588	0.0117	Hendy et a	2008	19909	Mollusca	7805	Bivalvia
Pleistocene		2.588	0.0117	Hendy et a	2008	19909	Mollusca	7805	Bivalvia
Late Pleistocene		0.126	0.0117	Cooke	1936	31145	Mollusca	7805	Bivalvia
Late Pleistocene		0.126	0.0117	Cooke	1936	31145	Mollusca	7805	Bivalvia
Late Pleistocene		0.126	0.0117	Cooke	1936	31145	Mollusca	7805	Bivalvia
Late Pleistocene		0.126	0.0117	Cooke	1936	31145	Mollusca	7805	Bivalvia
Early Pleistocene		2.588	0.781	Cooke	1936	31145	Mollusca	7805	Bivalvia
Early Pleistocene		2.588	0.781	Cooke	1936	31145	Mollusca	7805	Bivalvia
Early Pleistocene		2.588	0.781	Cooke	1936	31145	Mollusca	7805	Bivalvia
Pleistocene		2.588	0.0117	Holmes	1860	64712	Mollusca	7805	Bivalvia
Late Pliocene		3.6	2.588	Hendy et a	2008	19909	Mollusca	7805	Bivalvia
Early Pleistocene		2.588	0.781	Hendy et a	2008	19909	Mollusca	7805	Bivalvia
Early Pleistocene		2.588	0.781	Gardner	1943	16984	Mollusca	7805	Bivalvia
Late Pliocene		3.6	2.588	Gardner	1943	16984	Mollusca	7805	Bivalvia
Early Plioc	Late Plioce	5.333	2.588	Gardner	1943	16984	Mollusca	7805	Bivalvia
Late Pliocene		3.6	2.588	Hendy et a	2008	19909	Mollusca	7805	Bivalvia
Early Pleistocene		2.588	0.781	Hendy et a	2008	19909	Mollusca	7805	Bivalvia
Late Pliocene		3.6	2.588	Hendy et a	2008	19909	Mollusca	7805	Bivalvia
Late Pliocene		3.6	2.588	Gardner	1943	16984	Mollusca	7805	Bivalvia
Early Plioc	Late Plioce	5.333	2.588	Gardner	1943	16984	Mollusca	7805	Bivalvia
Late Pliocene		3.6	2.588	Gardner	1943	16984	Mollusca	7805	Bivalvia
Late Pliocene		3.6	2.588	Gardner	1943	16984	Mollusca	7805	Bivalvia
Late Pliocene		3.6	2.588	Gardner	1943	16984	Mollusca	7805	Bivalvia
Pleistocene		2.588	0.0117	Gardner	1943	16984	Mollusca	7805	Bivalvia
Late Pliocene		3.6	2.588	Gardner	1943	16984	Mollusca	7805	Bivalvia
Early Pleistocene		2.588	0.781	Gardner	1943	16984	Mollusca	7805	Bivalvia
Early Pleistocene		2.588	0.781	Gardner	1943	16984	Mollusca	7805	Bivalvia
Early Plioc	Late Plioce	5.333	2.588	Gardner	1943	16984	Mollusca	7805	Bivalvia
Early Pleistocene		2.588	0.781	Gardner	1943	16984	Mollusca	7805	Bivalvia
Late Pliocene		3.6	2.588	Gardner	1943	16984	Mollusca	7805	Bivalvia
Early Pleistocene		2.588	0.781	Miller	1990	17561	Mollusca	7805	Bivalvia
Early Pleistocene		2.588	0.781	Miller	1990	17561	Mollusca	7805	Bivalvia
Early Pleistocene		2.588	0.781	Miller	1990	17561	Mollusca	7805	Bivalvia
Early Pleistocene		2.588	0.781	Miller	1990	17561	Mollusca	7805	Bivalvia
Early Pleistocene		2.588	0.781	Miller	1990	17561	Mollusca	7805	Bivalvia
Late Pleistocene		0.126	0.0117	Richards	1962	18974	Mollusca	7805	Bivalvia
Late Pleistocene		0.126	0.0117	Richards	1962	18974	Mollusca	7805	Bivalvia
Late Pleistocene		0.126	0.0117	Richards	1962	18974	Mollusca	7805	Bivalvia
Late Pleistocene		0.126	0.0117	Richards	1962	18974	Mollusca	7805	Bivalvia
Late Pleistocene		0.126	0.0117	Richards	1962	18974	Mollusca	7805	Bivalvia
Late Pleistocene		0.126	0.0117	Richards	1962	18974	Mollusca	7805	Bivalvia
Late Pleistocene		0.126	0.0117	Richards	1962	18974	Mollusca	7805	Bivalvia
Late Pleistocene		0.126	0.0117	Richards	1962	18974	Mollusca	7805	Bivalvia

Late Pleistocene	0.126	0.0117	Richards	1962	18974	Mollusca	7805	Bivalvia
Late Pleistocene	0.126	0.0117	Richards	1962	18974	Mollusca	7805	Bivalvia
Late Pleistocene	0.126	0.0117	Richards	1962	18974	Mollusca	7805	Bivalvia
Late Pleistocene	0.126	0.0117	Richards	1962	18974	Mollusca	7805	Bivalvia
Late Pleistocene	0.126	0.0117	Richards	1962	18974	Mollusca	7805	Bivalvia
Late Pleistocene	0.126	0.0117	Richards	1962	18974	Mollusca	7805	Bivalvia
Late Pleistocene	0.126	0.0117	Richards	1962	18974	Mollusca	7805	Bivalvia
Late Pleistocene	0.126	0.0117	Richards	1962	18974	Mollusca	7805	Bivalvia
Late Pleistocene	0.126	0.0117	Hendy et a	2008	19909	Mollusca	7805	Bivalvia
Late Pleistocene	0.126	0.0117	Hendy et a	2008	19909	Mollusca	7805	Bivalvia
Gelasian	2.588	1.806	Hendy et a	2008	19909	Mollusca	7805	Bivalvia
Early Pleistocene	2.588	0.781	Hendy et a	2008	19909	Mollusca	7805	Bivalvia
Early Pleistocene	2.588	0.781	Hendy et a	2008	19909	Mollusca	7805	Bivalvia
Late Pleistocene	0.126	0.0117	Hendy et a	2008	19909	Mollusca	7805	Bivalvia
Late Pleistocene	0.126	0.0117	Hendy et a	2008	19909	Mollusca	7805	Bivalvia
Late Pliocene	3.6	2.588	Hendy et a	2008	19909	Mollusca	7805	Bivalvia
Late Pliocene	3.6	2.588	Hendy et a	2008	19909	Mollusca	7805	Bivalvia
Late Pliocene	3.6	2.588	Hendy et a	2008	19909	Mollusca	7805	Bivalvia
Late Pliocene	3.6	2.588	Hendy et a	2008	19909	Mollusca	7805	Bivalvia
Pliocene	5.333	2.588	Hendy et a	2008	19909	Mollusca	7805	Bivalvia
Pliocene	5.333	2.588	Hendy et a	2008	19909	Mollusca	7805	Bivalvia
Pliocene	5.333	2.588	Hendy et a	2008	19909	Mollusca	7805	Bivalvia
Late Pliocene	3.6	2.588	Hendy et a	2008	19909	Mollusca	7805	Bivalvia
Late Pliocene	3.6	2.588	Hendy et a	2008	19909	Mollusca	7805	Bivalvia
Late Pliocene	3.6	2.588	Hendy et a	2008	19909	Mollusca	7805	Bivalvia
Late Pliocene	3.6	2.588	Hendy et a	2008	19909	Mollusca	7805	Bivalvia
Early Pleistocene	2.588	0.781	Hendy et a	2008	19909	Mollusca	7805	Bivalvia
Late Pliocene	3.6	2.588	Hendy et a	2008	19909	Mollusca	7805	Bivalvia
Early Pleistocene	2.588	0.781	Hendy et a	2008	19909	Mollusca	7805	Bivalvia
Early Pleistocene	2.588	0.781	Hendy et a	2008	19909	Mollusca	7805	Bivalvia
Early Pleistocene	2.588	0.781	Hendy et a	2008	19909	Mollusca	7805	Bivalvia
Early Pleistocene	2.588	0.781	Hendy et a	2008	19909	Mollusca	7805	Bivalvia
Early Pleistocene	2.588	0.781	Hendy et a	2008	19909	Mollusca	7805	Bivalvia
Early Pleistocene	2.588	0.781	Hendy et a	2008	19909	Mollusca	7805	Bivalvia
Early Pleistocene	2.588	0.781	Hendy et a	2008	19909	Mollusca	7805	Bivalvia
Early Pleistocene	2.588	0.781	Hendy et a	2008	19909	Mollusca	7805	Bivalvia
Early Pleistocene	2.588	0.781	Hendy et a	2008	19909	Mollusca	7805	Bivalvia
Early Pleistocene	2.588	0.781	Hendy et a	2008	19909	Mollusca	7805	Bivalvia
Middle Pleistocene	0.781	0.126	Miller	1985	30803	Mollusca	7805	Bivalvia
Middle Pleistocene	0.781	0.126	Miller	1985	30803	Mollusca	7805	Bivalvia
Middle Pleistocene	0.781	0.126	Miller	1985	30803	Mollusca	7805	Bivalvia
Middle Pleistocene	0.781	0.126	Miller	1985	30803	Mollusca	7805	Bivalvia
Middle Pleistocene	0.781	0.126	Miller	1985	30803	Mollusca	7805	Bivalvia
Middle Pleistocene	0.781	0.126	Miller	1985	30803	Mollusca	7805	Bivalvia
Middle Pleistocene	0.781	0.126	Miller	1985	30803	Mollusca	7805	Bivalvia
Pliocene	5.333	2.588	Ward	2007	48009	Mollusca	7805	Bivalvia
Pliocene	5.333	2.588	Ward	2007	48009	Mollusca	7805	Bivalvia

Pliocene	5.333	2.588	Ward	2007	48009	Mollusca	7805	Bivalvia
Early Pleistocene	2.588	0.781	Miller and	1988	72890	Mollusca	7805	Bivalvia
Early Pleistocene	2.588	0.781	Miller and	1988	72890	Mollusca	7805	Bivalvia
Pleistocene	2.588	0.0117	Coch	1971	441	Mollusca	7805	Bivalvia
Pleistocene	2.588	0.0117	Coch	1971	441	Mollusca	7805	Bivalvia
Pleistocene	2.588	0.0117	Hendy et a	2008	19909	Mollusca	7805	Bivalvia
Late Pleistocene	0.126	0.0117	Spencer ar	1987	15525	Mollusca	7805	Bivalvia
Late Pleistocene	0.126	0.0117	Spencer ar	1987	15525	Mollusca	7805	Bivalvia
Late Pleistocene	0.126	0.0117	Spencer ar	1987	15525	Mollusca	7805	Bivalvia
Late Pleistocene	0.126	0.0117	Spencer ar	1987	15525	Mollusca	7805	Bivalvia
Late Pleistocene	0.126	0.0117	Spencer ar	1987	15525	Mollusca	7805	Bivalvia
Late Pleistocene	0.126	0.0117	Spencer ar	1987	15525	Mollusca	7805	Bivalvia
Late Pleistocene	0.126	0.0117	Spencer ar	1987	15525	Mollusca	7805	Bivalvia
Late Pleistocene	0.126	0.0117	Spencer ar	1987	15525	Mollusca	7805	Bivalvia
Late Pleistocene	0.126	0.0117	Spencer ar	1987	15525	Mollusca	7805	Bivalvia
Late Pleistocene	0.126	0.0117	Spencer ar	1987	15525	Mollusca	7805	Bivalvia
Late Pleistocene	0.126	0.0117	Spencer ar	1987	15525	Mollusca	7805	Bivalvia
Pliocene	5.333	2.588	Gardner	1943	16984	Mollusca	7805	Bivalvia
Late Pleistocene	0.126	0.0117	Hendy et a	2008	19909	Mollusca	7805	Bivalvia
Late Pleistocene	0.126	0.0117	Hendy et a	2008	19909	Mollusca	7805	Bivalvia
Late Pleistocene	0.126	0.0117	Hendy et a	2008	19909	Mollusca	7805	Bivalvia
Late Pliocene	3.6	2.588	Ward	1993	26351	Mollusca	7805	Bivalvia
Late Pliocene	3.6	2.588	Ward	1993	26351	Mollusca	7805	Bivalvia
Late Pliocene	3.6	2.588	Ward	1993	26351	Mollusca	7805	Bivalvia
Late Pliocene	3.6	2.588	Ward	1993	26351	Mollusca	7805	Bivalvia
Late Pliocene	3.6	2.588	Ward	1993	26351	Mollusca	7805	Bivalvia
Late Pliocene	3.6	2.588	Ward	1993	26351	Mollusca	7805	Bivalvia
Late Pliocene	3.6	2.588	Ward	1993	26351	Mollusca	7805	Bivalvia
Late Pliocene	3.6	2.588	Ward	1993	26351	Mollusca	7805	Bivalvia
Late Pliocene	3.6	2.588	Ward	1993	26351	Mollusca	7805	Bivalvia
Late Pliocene	3.6	2.588	Ward	1993	26351	Mollusca	7805	Bivalvia
Late Pliocene	3.6	2.588	Ward	1993	26351	Mollusca	7805	Bivalvia
Late Pliocene	3.6	2.588	Ward	1993	26351	Mollusca	7805	Bivalvia
Late Pliocene	3.6	2.588	Ward	1993	26351	Mollusca	7805	Bivalvia
Late Pliocene	3.6	2.588	Hendy et a	2008	19909	Mollusca	7805	Bivalvia
Late Pliocene	3.6	2.588	Hendy et a	2008	19909	Mollusca	7805	Bivalvia
Late Pliocene	3.6	2.588	Hendy et a	2008	19909	Mollusca	7805	Bivalvia
Late Pliocene	3.6	2.588	Hendy et a	2008	19909	Mollusca	7805	Bivalvia
Late Pliocene	3.6	2.588	Hendy et a	2008	19909	Mollusca	7805	Bivalvia
Late Pleistocene	0.126	0.0117	Hendy et a	2008	19909	Mollusca	7805	Bivalvia
Late Pliocene	3.6	2.588	Hendy et a	2008	19909	Mollusca	7805	Bivalvia
Late Pliocene	3.6	2.588	Hendy et a	2008	19909	Mollusca	7805	Bivalvia
Late Pliocene	3.6	2.588	Hendy et a	2008	19909	Mollusca	7805	Bivalvia
Late Pliocene	3.6	2.588	Hendy et a	2008	19909	Mollusca	7805	Bivalvia
Early Pleistocene	2.588	0.781	DuBar	1962	11119	Mollusca	7805	Bivalvia
Pliocene Pleistocen	5.333	0.0117	DuBar	1962	11119	Mollusca	7805	Bivalvia
Middle Pleistocene	0.781	0.126	McGinty	1970	19114	Mollusca	7805	Bivalvia

Early Pleistocene	2.588	0.781	Olsson anc	1953	25463	Mollusca	7805	Bivalvia
Pliocene	5.333	2.588	Gardner	1943	16984	Mollusca	7805	Bivalvia
Pliocene	5.333	2.588	Gardner	1943	16984	Mollusca	7805	Bivalvia
Late Pliocene	3.6	2.588	Hendy et a	2008	19909	Mollusca	7805	Bivalvia
Early Pliocene	5.333	3.6	Petuch anc	2007	26813	Mollusca	7805	Bivalvia
Late Pliocene	3.6	2.588	Ward	1993	26351	Mollusca	7805	Bivalvia
Late Pliocene	3.6	2.588	Ward	1993	26351	Mollusca	7805	Bivalvia
Early Pleistocene	2.588	0.781	Hendy et a	2008	19909	Mollusca	7805	Bivalvia
Middle Pleistocene	0.781	0.126	Portell et a	1992	17634	Mollusca	7805	Bivalvia
Middle Pleistocene	0.781	0.126	Portell et a	1992	17634	Mollusca	7805	Bivalvia
Late Pleistocene	0.126	0.0117	Portell et a	1992	17634	Mollusca	7805	Bivalvia
Late Pleistocene	0.126	0.0117	Portell et a	1992	17634	Mollusca	7805	Bivalvia
Early Pleistocene	2.588	0.781	Olsson anc	1953	25463	Mollusca	7805	Bivalvia
Early Pleistocene	2.588	0.781	Hendy et a	2008	19909	Mollusca	7805	Bivalvia
Late Pleistocene	0.126	0.0117	Kittle and I	2010	40884	Mollusca	7805	Bivalvia
Late Pleistocene	0.126	0.0117	Kittle and I	2010	40884	Mollusca	7805	Bivalvia
Late Pleistocene	0.126	0.0117	DuBar	1971	17556	Mollusca	7805	Bivalvia
Late Pleistocene	0.126	0.0117	DuBar	1971	17556	Mollusca	7805	Bivalvia
Early Pleistocene	2.588	0.781	Ward and I	1987	16700	Mollusca	7805	Bivalvia
Early Pleistocene	2.588	0.781	Gardner	1943	16984	Mollusca	7805	Bivalvia
Early Pleistocene	2.588	0.781	Gardner	1943	16984	Mollusca	7805	Bivalvia
Early Pleistocene	2.588	0.781	Gardner	1943	16984	Mollusca	7805	Bivalvia
Pleistocene	2.588	0.0117	Gardner	1943	16984	Mollusca	7805	Bivalvia
Early Pleistocene	2.588	0.781	Gardner	1943	16984	Mollusca	7805	Bivalvia
Late Pleistocene	0.126	0.0117	Richards	1962	18974	Mollusca	7805	Bivalvia
Late Pleistocene	0.126	0.0117	Richards	1962	18974	Mollusca	7805	Bivalvia
Gelasian	2.588	1.806	Bailey and	1986	23840	Mollusca	7805	Bivalvia
Middle Pleistocene	0.781	0.126	Portell et a	1992	17634	Mollusca	7805	Bivalvia
Middle Pleistocene	0.781	0.126	Hoerle	1970	19536	Mollusca	7805	Bivalvia
Early Pleistocene	2.588	0.781	Olsson anc	1953	25463	Mollusca	7805	Bivalvia
Early Pleistocene	2.588	0.781	Hendy et a	2008	19909	Mollusca	7805	Bivalvia
Late Pleistocene	0.126	0.0117	Kittle and I	2010	40884	Mollusca	7805	Bivalvia
Late Pleistocene	0.126	0.0117	Richards	1962	18974	Mollusca	7805	Bivalvia
Late Pleistocene	0.126	0.0117	Richards	1962	18974	Mollusca	7805	Bivalvia
Early Pleistocene	2.588	0.781	Cooke	1936	31145	Mollusca	7805	Bivalvia
Pleistocene	2.588	0.0117	Holmes	1860	64712	Mollusca	7805	Bivalvia
Late Pliocene	3.6	2.588	Ward and I	1987	16700	Mollusca	7805	Bivalvia
Late Pliocene	3.6	2.588	Ward and I	1987	16700	Mollusca	7805	Bivalvia
Early Pleistocene	2.588	0.781	Ward and I	1987	16700	Mollusca	7805	Bivalvia
Early Pleistocene	2.588	0.781	Gardner	1943	16984	Mollusca	7805	Bivalvia
Early Pleistocene	2.588	0.781	Gardner	1943	16984	Mollusca	7805	Bivalvia
Early Pleistocene	2.588	0.781	Miller	1990	17561	Mollusca	7805	Bivalvia
Early Pleistocene	2.588	0.781	Miller	1990	17561	Mollusca	7805	Bivalvia
Early Pleistocene	2.588	0.781	Miller	1990	17561	Mollusca	7805	Bivalvia
Early Pleistocene	2.588	0.781	Miller	1990	17561	Mollusca	7805	Bivalvia
Late Pleistocene	0.126	0.0117	Richards	1962	18974	Mollusca	7805	Bivalvia
Early Pleistocene	2.588	0.781	Hendy et a	2008	19909	Mollusca	7805	Bivalvia

Late Pliocene		3.6	2.588	Hendy et a	2008	19909	Mollusca	7805	Bivalvia
Late Pliocene		3.6	2.588	Hendy et a	2008	19909	Mollusca	7805	Bivalvia
Early Pleistocene		2.588	0.781	Miller and	1988	72890	Mollusca	7805	Bivalvia
Early Pleistocene		2.588	0.781	Miller and	1988	72890	Mollusca	7805	Bivalvia
Late Pleistocene		0.126	0.0117	Spencer ar	1987	15525	Mollusca	7805	Bivalvia
Late Pleistocene		0.126	0.0117	Spencer ar	1987	15525	Mollusca	7805	Bivalvia
Late Pleistocene		0.126	0.0117	Spencer ar	1987	15525	Mollusca	7805	Bivalvia
Late Pliocene		3.6	2.588	Ward	1993	26351	Mollusca	7805	Bivalvia
Late Pliocene		3.6	2.588	Ward	1993	26351	Mollusca	7805	Bivalvia
Late Pliocene		3.6	2.588	Hendy et a	2008	19909	Mollusca	7805	Bivalvia
Late Pliocene		3.6	2.588	Hendy et a	2008	19909	Mollusca	7805	Bivalvia
Early Pleistocene		2.588	0.781	DuBar	1962	11119	Mollusca	7805	Bivalvia
Calabrian		1.806	0.781	DuBar	1962	11119	Mollusca	7805	Bivalvia
Calabrian		1.806	0.781	DuBar	1962	11119	Mollusca	7805	Bivalvia
Calabrian		1.806	0.781	DuBar	1962	11119	Mollusca	7805	Bivalvia
Early Pleistocene		2.588	0.781	DuBar	1962	11119	Mollusca	7805	Bivalvia
Pliocene	Pleistocen	5.333	0.0117	DuBar	1962	11119	Mollusca	7805	Bivalvia
Late Pliocene		3.6	2.588	Mansfield	1932	17094	Mollusca	7805	Bivalvia
Early Pleistocene		2.588	0.781	Portell et a	1992	17634	Mollusca	7805	Bivalvia
Early Pleistocene		2.588	0.781	Portell et a	1992	17634	Mollusca	7805	Bivalvia
Middle Pleistocene		0.781	0.126	Portell et a	1992	17634	Mollusca	7805	Bivalvia
Middle Pleistocene		0.781	0.126	Portell et a	1992	17634	Mollusca	7805	Bivalvia
Late Pleistocene		0.126	0.0117	Portell et a	1992	17634	Mollusca	7805	Bivalvia
Middle Pleistocene		0.781	0.126	McGinty	1970	19114	Mollusca	7805	Bivalvia
Middle Pleistocene		0.781	0.126	McGinty	1970	19114	Mollusca	7805	Bivalvia
Middle Pleistocene		0.781	0.126	McGinty	1970	19114	Mollusca	7805	Bivalvia
Middle Pleistocene		0.781	0.126	McGinty	1970	19114	Mollusca	7805	Bivalvia
Middle Pleistocene		0.781	0.126	McGinty	1970	19114	Mollusca	7805	Bivalvia
Late Pleistocene		0.126	0.0117	Hendy et a	2008	19909	Mollusca	7805	Bivalvia
Late Pleistocene		0.126	0.0117	Hendy et a	2008	19909	Mollusca	7805	Bivalvia
Pliocene		5.333	2.588	Schroeder	1954	25303	Mollusca	7805	Bivalvia
Pliocene		5.333	2.588	Schroeder	1954	25303	Mollusca	7805	Bivalvia
Pliocene		5.333	2.588	Schroeder	1954	25303	Mollusca	7805	Bivalvia
Early Pleistocene		2.588	0.781	Olsson anc	1953	25463	Mollusca	7805	Bivalvia
Early Pleistocene		2.588	0.781	Olsson anc	1953	25463	Mollusca	7805	Bivalvia
Early Pleistocene		2.588	0.781	Olsson anc	1953	25463	Mollusca	7805	Bivalvia
Late Pliocene		3.6	2.588	Olsson	1967	19826	Mollusca	7805	Bivalvia
Late Pliocene		3.6	2.588	Olsson	1967	19826	Mollusca	7805	Bivalvia
Late Pliocene		3.6	2.588	Olsson anc	1964	26836	Mollusca	7805	Bivalvia
Late Pliocene		3.6	2.588	Petuch anc	2007	26813	Mollusca	7805	Bivalvia
Early Pleistocene		2.588	0.781	Hendy et a	2008	19909	Mollusca	7805	Bivalvia
Late Pleistocene		0.126	0.0117	DuBar	1958	25514	Mollusca	7805	Bivalvia
Early Pleistocene		2.588	0.781	Hendy et a	2008	19909	Mollusca	7805	Bivalvia
Early Pleistocene		2.588	0.781	Hendy et a	2008	19909	Mollusca	7805	Bivalvia
Early Pleistocene		2.588	0.781	Hendy et a	2008	19909	Mollusca	7805	Bivalvia
Early Pleistocene		2.588	0.781	Hendy et a	2008	19909	Mollusca	7805	Bivalvia
Late Pliocene		3.6	2.588	Hendy et a	2008	19909	Mollusca	7805	Bivalvia

Late Pliocene	3.6	2.588	Hendy et a	2008	19909	Mollusca	7805	Bivalvia
Middle Pleistocene	0.781	0.126	Hendy et a	2008	19909	Mollusca	7805	Bivalvia
Middle Pleistocene	0.781	0.126	Hendy et a	2008	19909	Mollusca	7805	Bivalvia
Late Pleistocene	0.126	0.0117	Hendy et a	2008	19909	Mollusca	7805	Bivalvia
Early Pleistocene	2.588	0.781	Hendy et a	2008	19909	Mollusca	7805	Bivalvia
Early Pleistocene	2.588	0.781	Hendy et a	2008	19909	Mollusca	7805	Bivalvia
Early Pleistocene	2.588	0.781	DuBar	1958	25514	Mollusca	7805	Bivalvia
Late Pliocene	3.6	2.588	Weisbord	1972	31274	Mollusca	7805	Bivalvia
Late Pleistocene	0.126	0.0117	Kittle and I	2010	40884	Mollusca	7805	Bivalvia
Piacenzian	3.6	2.588	Emslie et a	1996	50409	Mollusca	7805	Bivalvia
Pliocene	5.333	2.588	Aldrich	1911	53829	Mollusca	7805	Bivalvia
Zanclean	5.333	3.6	Campbell a	1995	17558	Mollusca	7805	Bivalvia
Zanclean	5.333	3.6	Campbell a	1995	17558	Mollusca	7805	Bivalvia
Late Pleistocene	0.126	0.0117	Richards	1962	18974	Mollusca	7805	Bivalvia
Late Pleistocene	0.126	0.0117	Hendy et a	2008	19909	Mollusca	7805	Bivalvia
Late Pleistocene	0.126	0.0117	Hendy et a	2008	19909	Mollusca	7805	Bivalvia
Pleistocene	2.588	0.0117	Hendy et a	2008	19909	Mollusca	7805	Bivalvia
Early Pleistocene	2.588	0.781	Cooke	1936	31145	Mollusca	7805	Bivalvia
Late Pliocene	3.6	2.588	Ward and I	1987	16700	Mollusca	7805	Bivalvia
Late Pliocene	3.6	2.588	Ward and I	1987	16700	Mollusca	7805	Bivalvia
Late Pliocene	3.6	2.588	Ward and I	1987	16700	Mollusca	7805	Bivalvia
Late Pliocene	3.6	2.588	Ward and I	1987	16700	Mollusca	7805	Bivalvia
Late Pliocene	3.6	2.588	Ward and I	1987	16700	Mollusca	7805	Bivalvia
Early Pleistocene	2.588	0.781	Ward and I	1987	16700	Mollusca	7805	Bivalvia
Early Pleistocene	2.588	0.781	Ward and I	1987	16700	Mollusca	7805	Bivalvia
Pleistocene	2.588	0.0117	Gardner	1943	16984	Mollusca	7805	Bivalvia
Early Pleistocene	2.588	0.781	Gardner	1943	16984	Mollusca	7805	Bivalvia
Early Pleistocene	2.588	0.781	Miller	1990	17561	Mollusca	7805	Bivalvia
Early Pleistocene	2.588	0.781	Miller	1990	17561	Mollusca	7805	Bivalvia
Late Pleistocene	0.126	0.0117	Richards	1962	18974	Mollusca	7805	Bivalvia
Gelasian	2.588	1.806	Bailey and	1986	23840	Mollusca	7805	Bivalvia
Late Pleistocene	0.126	0.0117	Hendy et a	2008	19909	Mollusca	7805	Bivalvia
Early Pleistocene	2.588	0.781	Hendy et a	2008	19909	Mollusca	7805	Bivalvia
Pleistocene	2.588	0.0117	Coch	1971	441	Mollusca	7805	Bivalvia
Late Pleistocene	0.126	0.0117	Spencer ar	1987	15525	Mollusca	7805	Bivalvia
Late Pleistocene	0.126	0.0117	Spencer ar	1987	15525	Mollusca	7805	Bivalvia
Late Pleistocene	0.126	0.0117	Spencer ar	1987	15525	Mollusca	7805	Bivalvia
Late Pleistocene	0.126	0.0117	Spencer ar	1987	15525	Mollusca	7805	Bivalvia
Late Pleistocene	0.126	0.0117	Spencer ar	1987	15525	Mollusca	7805	Bivalvia
Late Pleistocene	0.126	0.0117	Spencer ar	1987	15525	Mollusca	7805	Bivalvia
Late Pliocene	3.6	2.588	Ward	1993	26351	Mollusca	7805	Bivalvia
Late Pliocene	3.6	2.588	Hendy et a	2008	19909	Mollusca	7805	Bivalvia
Late Pliocene	3.6	2.588	Hendy et a	2008	19909	Mollusca	7805	Bivalvia
Early Pleistocene	2.588	0.781	DuBar	1962	11119	Mollusca	7805	Bivalvia
Early Pleistocene	2.588	0.781	DuBar	1962	11119	Mollusca	7805	Bivalvia
Early Pleistocene	2.588	0.781	DuBar	1962	11119	Mollusca	7805	Bivalvia
Early Pleistocene	2.588	0.781	DuBar	1962	11119	Mollusca	7805	Bivalvia

Early Pleistocene	2.588	0.781	DuBar	1962	11119	Mollusca	7805	Bivalvia
Early Pleistocene	2.588	0.781	DuBar	1962	11119	Mollusca	7805	Bivalvia
Calabrian	1.806	0.781	DuBar	1962	11119	Mollusca	7805	Bivalvia
Late Pleistocene	0.126	0.0117	DuBar	1962	11119	Mollusca	7805	Bivalvia
Early Pleistocene	2.588	0.781	DuBar	1962	11119	Mollusca	7805	Bivalvia
Early Pleistocene	2.588	0.781	DuBar	1962	11119	Mollusca	7805	Bivalvia
Pliocene Pleistocen	5.333	0.0117	DuBar	1962	11119	Mollusca	7805	Bivalvia
Late Pliocene	3.6	2.588	Mansfield	1932	17094	Mollusca	7805	Bivalvia
Late Pliocene	3.6	2.588	Mansfield	1932	17094	Mollusca	7805	Bivalvia
Late Pliocene	3.6	2.588	Mansfield	1932	17094	Mollusca	7805	Bivalvia
Late Pliocene	3.6	2.588	Mansfield	1932	17094	Mollusca	7805	Bivalvia
Early Pleistocene	2.588	0.781	Portell et a	1992	17634	Mollusca	7805	Bivalvia
Early Pleistocene	2.588	0.781	Portell et a	1992	17634	Mollusca	7805	Bivalvia
Early Pleistocene	2.588	0.781	Portell et a	1992	17634	Mollusca	7805	Bivalvia
Middle Pleistocene	0.781	0.126	Portell et a	1992	17634	Mollusca	7805	Bivalvia
Middle Pleistocene	0.781	0.126	Portell et a	1992	17634	Mollusca	7805	Bivalvia
Late Pleistocene	0.126	0.0117	Portell et a	1992	17634	Mollusca	7805	Bivalvia
Late Pleistocene	0.126	0.0117	Portell et a	1992	17634	Mollusca	7805	Bivalvia
Middle Pleistocene	0.781	0.126	McGinty	1970	19114	Mollusca	7805	Bivalvia
Middle Pleistocene	0.781	0.126	McGinty	1970	19114	Mollusca	7805	Bivalvia
Late Pleistocene	0.126	0.0117	Hendy et a	2008	19909	Mollusca	7805	Bivalvia
Early Pleistocene	2.588	0.781	Schroeder	1954	25303	Mollusca	7805	Bivalvia
Early Pleistocene	2.588	0.781	Schroeder	1954	25303	Mollusca	7805	Bivalvia
Pliocene	5.333	2.588	Schroeder	1954	25303	Mollusca	7805	Bivalvia
Early Pleistocene	2.588	0.781	Schroeder	1954	25303	Mollusca	7805	Bivalvia
Early Pleistocene	2.588	0.781	Olsson anc	1953	25463	Mollusca	7805	Bivalvia
Early Pleistocene	2.588	0.781	Olsson anc	1953	25463	Mollusca	7805	Bivalvia
Late Pleistocene	0.126	0.0117	Lovejoy	1992	26806	Mollusca	7805	Bivalvia
Early Pleistocene	2.588	0.781	Petuch anc	2007	26813	Mollusca	7805	Bivalvia
Early Pleistocene	2.588	0.781	Petuch anc	2007	26813	Mollusca	7805	Bivalvia
Middle Pleistocene	0.781	0.126	Petuch anc	2007	26813	Mollusca	7805	Bivalvia
Middle Pleistocene	0.781	0.126	Olsson anc	1964	26836	Mollusca	7805	Bivalvia
Early Pleistocene	2.588	0.781	Hendy et a	2008	19909	Mollusca	7805	Bivalvia
Late Pleistocene	0.126	0.0117	Hendy et a	2008	19909	Mollusca	7805	Bivalvia
Late Pliocene	3.6	2.588	Hendy et a	2008	19909	Mollusca	7805	Bivalvia
Early Pleistocene	2.588	0.781	Hendy et a	2008	19909	Mollusca	7805	Bivalvia
Early Pleistocene	2.588	0.781	Hendy et a	2008	19909	Mollusca	7805	Bivalvia
Pleistocene	2.588	0.0117	Hendy et a	2008	19909	Mollusca	7805	Bivalvia
Late Pliocene	3.6	2.588	Hendy et a	2008	19909	Mollusca	7805	Bivalvia
Late Pliocene	3.6	2.588	Hendy et a	2008	19909	Mollusca	7805	Bivalvia
Middle Pleistocene	0.781	0.126	Hendy et a	2008	19909	Mollusca	7805	Bivalvia
Middle Pleistocene	0.781	0.126	Hendy et a	2008	19909	Mollusca	7805	Bivalvia
Late Pleistocene	0.126	0.0117	Hendy et a	2008	19909	Mollusca	7805	Bivalvia
Late Pleistocene	0.126	0.0117	Kittle and I	2010	40884	Mollusca	7805	Bivalvia
Late Pleistocene	0.126	0.0117	Kittle and I	2010	40884	Mollusca	7805	Bivalvia
Late Pleistocene	0.126	0.0117	Kittle and I	2010	40884	Mollusca	7805	Bivalvia
Late Pleistocene	0.126	0.0117	Hulbert an	1998	1962	Mollusca	7805	Bivalvia

Late Pleistocene	0.126	0.0117	Hulbert an	1998	1962	Mollusca	7805	Bivalvia
Late Pleistocene	0.126	0.0117	Richards	1962	18974	Mollusca	7805	Bivalvia
Late Pleistocene	0.126	0.0117	Richards	1962	18974	Mollusca	7805	Bivalvia
Late Pleistocene	0.126	0.0117	DuBar	1971	17556	Mollusca	7805	Bivalvia
Late Pleistocene	0.126	0.0117	DuBar	1971	17556	Mollusca	7805	Bivalvia
Late Pleistocene	0.126	0.0117	DuBar	1971	17556	Mollusca	7805	Bivalvia
Zanclean	5.333	3.6	Campbell a	1995	17558	Mollusca	7805	Bivalvia
Zanclean	5.333	3.6	Campbell a	1995	17558	Mollusca	7805	Bivalvia
Late Pleistocene	0.126	0.0117	Richards	1962	18974	Mollusca	7805	Bivalvia
Late Pleistocene	0.126	0.0117	Richards	1962	18974	Mollusca	7805	Bivalvia
Late Pleistocene	0.126	0.0117	Richards	1962	18974	Mollusca	7805	Bivalvia
Late Pleistocene	0.126	0.0117	Richards	1962	18974	Mollusca	7805	Bivalvia
Late Pleistocene	0.126	0.0117	Hendy et a	2008	19909	Mollusca	7805	Bivalvia
Late Pleistocene	0.126	0.0117	Hendy et a	2008	19909	Mollusca	7805	Bivalvia
Late Pleistocene	0.126	0.0117	Hendy et a	2008	19909	Mollusca	7805	Bivalvia
Pleistocene	2.588	0.0117	Hendy et a	2008	19909	Mollusca	7805	Bivalvia
Late Pleistocene	0.126	0.0117	Cooke	1936	31145	Mollusca	7805	Bivalvia
Early Pleistocene	2.588	0.781	Cooke	1936	31145	Mollusca	7805	Bivalvia
Early Pleistocene	2.588	0.781	Cooke	1936	31145	Mollusca	7805	Bivalvia
Late Pliocene	3.6	2.588	Ward and I	1987	16700	Mollusca	7805	Bivalvia
Early Pleistocene	2.588	0.781	Ward and I	1987	16700	Mollusca	7805	Bivalvia
Early Pleistocene	2.588	0.781	Miller	1990	17561	Mollusca	7805	Bivalvia
Early Pleistocene	2.588	0.781	Miller	1990	17561	Mollusca	7805	Bivalvia
Late Pleistocene	0.126	0.0117	Richards	1962	18974	Mollusca	7805	Bivalvia
Late Pleistocene	0.126	0.0117	Richards	1962	18974	Mollusca	7805	Bivalvia
Late Pleistocene	0.126	0.0117	Richards	1962	18974	Mollusca	7805	Bivalvia
Late Pleistocene	0.126	0.0117	Richards	1962	18974	Mollusca	7805	Bivalvia
Late Pleistocene	0.126	0.0117	Richards	1962	18974	Mollusca	7805	Bivalvia
Late Pleistocene	0.126	0.0117	Richards	1962	18974	Mollusca	7805	Bivalvia
Late Pleistocene	0.126	0.0117	Richards	1962	18974	Mollusca	7805	Bivalvia
Late Pleistocene	0.126	0.0117	Richards	1962	18974	Mollusca	7805	Bivalvia
Late Pleistocene	0.126	0.0117	Richards	1962	18974	Mollusca	7805	Bivalvia
Late Pleistocene	0.126	0.0117	Hendy et a	2008	19909	Mollusca	7805	Bivalvia
Gelasian	2.588	1.806	Bailey and	1986	23840	Mollusca	7805	Bivalvia
Early Pleistocene	2.588	0.781	Hendy et a	2008	19909	Mollusca	7805	Bivalvia
Early Pleistocene	2.588	0.781	Hendy et a	2008	19909	Mollusca	7805	Bivalvia
Late Pliocene	3.6	2.588	Hendy et a	2008	19909	Mollusca	7805	Bivalvia
Late Pliocene	3.6	2.588	Hendy et a	2008	19909	Mollusca	7805	Bivalvia
Pliocene	5.333	2.588	Hendy et a	2008	19909	Mollusca	7805	Bivalvia
Pliocene	5.333	2.588	Hendy et a	2008	19909	Mollusca	7805	Bivalvia
Early Pleistocene	2.588	0.781	Hendy et a	2008	19909	Mollusca	7805	Bivalvia
Early Pleistocene	2.588	0.781	Hendy et a	2008	19909	Mollusca	7805	Bivalvia
Early Pleistocene	2.588	0.781	Hendy et a	2008	19909	Mollusca	7805	Bivalvia
Early Pleistocene	2.588	0.781	Hendy et a	2008	19909	Mollusca	7805	Bivalvia
Early Pleistocene	2.588	0.781	Hendy et a	2008	19909	Mollusca	7805	Bivalvia
Early Pleistocene	2.588	0.781	Hendy et a	2008	19909	Mollusca	7805	Bivalvia
Early Pleistocene	2.588	0.781	Miller and	1988	72890	Mollusca	7805	Bivalvia

Late Pleistocene	0.126	0.0117	Spencer ar	1987	15525	Mollusca	7805	Bivalvia
Late Pleistocene	0.126	0.0117	Spencer ar	1987	15525	Mollusca	7805	Bivalvia
Late Pleistocene	0.126	0.0117	Spencer ar	1987	15525	Mollusca	7805	Bivalvia
Late Pliocene	3.6	2.588	Ward	1993	26351	Mollusca	7805	Bivalvia
Late Pliocene	3.6	2.588	Ward	1993	26351	Mollusca	7805	Bivalvia
Late Pliocene	3.6	2.588	Ward	1993	26351	Mollusca	7805	Bivalvia
Late Pliocene	3.6	2.588	Ward	1993	26351	Mollusca	7805	Bivalvia
Late Pliocene	3.6	2.588	Ward	1993	26351	Mollusca	7805	Bivalvia
Late Pliocene	3.6	2.588	Ward	1993	26351	Mollusca	7805	Bivalvia
Late Pliocene	3.6	2.588	Ward	1993	26351	Mollusca	7805	Bivalvia
Late Pliocene	3.6	2.588	Ward	1993	26351	Mollusca	7805	Bivalvia
Late Pliocene	3.6	2.588	Ward	1993	26351	Mollusca	7805	Bivalvia
Late Pliocene	3.6	2.588	Hendy et a	2008	19909	Mollusca	7805	Bivalvia
Late Pliocene	3.6	2.588	Hendy et a	2008	19909	Mollusca	7805	Bivalvia
Late Pliocene	3.6	2.588	Hendy et a	2008	19909	Mollusca	7805	Bivalvia
Late Pleistocene	0.126	0.0117	Hendy et a	2008	19909	Mollusca	7805	Bivalvia
Late Pliocene	3.6	2.588	Mansfield	1932	17094	Mollusca	7805	Bivalvia
Early Pleistocene	2.588	0.781	Vokes	1985	30826	Mollusca	7805	Bivalvia
Early Pleistocene	2.588	0.781	DuBar	1962	11119	Mollusca	7805	Bivalvia
Early Pleistocene	2.588	0.781	DuBar	1962	11119	Mollusca	7805	Bivalvia
Calabrian	1.806	0.781	DuBar	1962	11119	Mollusca	7805	Bivalvia
Early Pleistocene	2.588	0.781	DuBar	1962	11119	Mollusca	7805	Bivalvia
Pliocene Pleistocen	5.333	0.0117	DuBar	1962	11119	Mollusca	7805	Bivalvia
Late Pliocene	3.6	2.588	Mansfield	1932	17094	Mollusca	7805	Bivalvia
Late Pliocene	3.6	2.588	Mansfield	1932	17094	Mollusca	7805	Bivalvia
Late Pliocene	3.6	2.588	Mansfield	1932	17094	Mollusca	7805	Bivalvia
Late Pliocene	3.6	2.588	Mansfield	1932	17094	Mollusca	7805	Bivalvia
Late Pliocene	3.6	2.588	Mansfield	1932	17094	Mollusca	7805	Bivalvia
Late Pliocene	3.6	2.588	Mansfield	1932	17094	Mollusca	7805	Bivalvia
Late Pliocene	3.6	2.588	Mansfield	1932	17094	Mollusca	7805	Bivalvia
Early Pleistocene	2.588	0.781	Portell et a	1992	17634	Mollusca	7805	Bivalvia
Middle Pleistocene	0.781	0.126	Portell et a	1992	17634	Mollusca	7805	Bivalvia
Late Pleistocene	0.126	0.0117	Portell et a	1992	17634	Mollusca	7805	Bivalvia
Middle Pleistocene	0.781	0.126	McGinty	1970	19114	Mollusca	7805	Bivalvia
Late Pliocene	3.6	2.588	Mansfield	1939	19112	Mollusca	7805	Bivalvia
Late Pliocene	3.6	2.588	Mansfield	1939	19112	Mollusca	7805	Bivalvia
Late Pliocene	3.6	2.588	Mansfield	1939	19112	Mollusca	7805	Bivalvia
Late Pliocene	3.6	2.588	Mansfield	1939	19112	Mollusca	7805	Bivalvia
Early Pleistocene	2.588	0.781	Schroeder	1954	25303	Mollusca	7805	Bivalvia
Pliocene	5.333	2.588	Schroeder	1954	25303	Mollusca	7805	Bivalvia
Pliocene	5.333	2.588	Schroeder	1954	25303	Mollusca	7805	Bivalvia
Pliocene	5.333	2.588	Schroeder	1954	25303	Mollusca	7805	Bivalvia
Early Pleistocene	2.588	0.781	Olsson anc	1953	25463	Mollusca	7805	Bivalvia
Late Pliocene	3.6	2.588	DuBar	1958	25514	Mollusca	7805	Bivalvia
Early Pleistocene	2.588	0.781	Hendy et a	2008	19909	Mollusca	7805	Bivalvia
Pleistocene	2.588	0.0117	Hendy et a	2008	19909	Mollusca	7805	Bivalvia
Pleistocene	2.588	0.0117	Hendy et a	2008	19909	Mollusca	7805	Bivalvia

Late Pliocene	3.6	2.588	Hendy et a	2008	19909	Mollusca	7805	Bivalvia
Late Pliocene	3.6	2.588	Hendy et a	2008	19909	Mollusca	7805	Bivalvia
Early Pleistocene	2.588	0.781	DuBar	1958	25514	Mollusca	7805	Bivalvia
Late Pliocene	3.6	2.588	Smith	1941	32333	Mollusca	7805	Bivalvia
Late Pleistocene	0.126	0.0117	Kittle and F	2010	40884	Mollusca	7805	Bivalvia
Late Pleistocene	0.126	0.0117	DuBar	1971	17556	Mollusca	7805	Bivalvia
Zanclean	5.333	3.6	Campbell a	1995	17558	Mollusca	7805	Bivalvia
Early Pleistocene	2.588	0.781	DuBar	1971	17556	Mollusca	7805	Bivalvia
Late Pleistocene	0.126	0.0117	Richards	1962	18974	Mollusca	7805	Bivalvia
Late Pleistocene	0.126	0.0117	Hendy et a	2008	19909	Mollusca	7805	Bivalvia
Early Pleistocene	2.588	0.781	Cooke	1936	31145	Mollusca	7805	Bivalvia
Late Pliocene	3.6	2.588	Cooke	1936	31145	Mollusca	7805	Bivalvia
Late Pleistocene	0.126	0.0117	Holmes	1860	64712	Mollusca	7805	Bivalvia
Pleistocene Holocene	2.588	0	Holmes	1860	64712	Mollusca	7805	Bivalvia
Late Pliocene	3.6	2.588	Ward and I	1987	16700	Mollusca	7805	Bivalvia
Late Pliocene	3.6	2.588	Ward and I	1987	16700	Mollusca	7805	Bivalvia
Late Pliocene	3.6	2.588	Ward and I	1987	16700	Mollusca	7805	Bivalvia
Early Pleistocene	2.588	0.781	Ward and I	1987	16700	Mollusca	7805	Bivalvia
Early Pleistocene	2.588	0.781	Miller	1990	17561	Mollusca	7805	Bivalvia
Early Pleistocene	2.588	0.781	Miller	1990	17561	Mollusca	7805	Bivalvia
Early Pleistocene	2.588	0.781	Miller	1990	17561	Mollusca	7805	Bivalvia
Early Pleistocene	2.588	0.781	Miller	1990	17561	Mollusca	7805	Bivalvia
Late Pleistocene	0.126	0.0117	Richards	1962	18974	Mollusca	7805	Bivalvia
Late Pleistocene	0.126	0.0117	Richards	1962	18974	Mollusca	7805	Bivalvia
Late Pleistocene	0.126	0.0117	Richards	1962	18974	Mollusca	7805	Bivalvia
Late Pliocene	3.6	2.588	Hendy et a	2008	19909	Mollusca	7805	Bivalvia
Gelasian	2.588	1.806	Bailey and	1986	23840	Mollusca	7805	Bivalvia
Early Pleistocene	2.588	0.781	Hendy et a	2008	19909	Mollusca	7805	Bivalvia
Late Pliocene	3.6	2.588	Hendy et a	2008	19909	Mollusca	7805	Bivalvia
Late Pliocene	3.6	2.588	Hendy et a	2008	19909	Mollusca	7805	Bivalvia
Late Pliocene	3.6	2.588	Hendy et a	2008	19909	Mollusca	7805	Bivalvia
Late Pliocene	3.6	2.588	Hendy et a	2008	19909	Mollusca	7805	Bivalvia
Early Pleistocene	2.588	0.781	Hendy et a	2008	19909	Mollusca	7805	Bivalvia
Early Pleistocene	2.588	0.781	Hendy et a	2008	19909	Mollusca	7805	Bivalvia
Early Pleistocene	2.588	0.781	Miller and	1988	72890	Mollusca	7805	Bivalvia
Early Pleistocene	2.588	0.781	Miller and	1988	72890	Mollusca	7805	Bivalvia
Early Pleistocene	2.588	0.781	Miller and	1988	72890	Mollusca	7805	Bivalvia
Early Pleistocene	2.588	0.781	Miller and	1988	72890	Mollusca	7805	Bivalvia
Pleistocene	2.588	0.0117	Coch	1971	441	Mollusca	7805	Bivalvia
Late Pleistocene	0.126	0.0117	Spencer ar	1987	15525	Mollusca	7805	Bivalvia
Late Pleistocene	0.126	0.0117	Spencer ar	1987	15525	Mollusca	7805	Bivalvia
Late Pleistocene	0.126	0.0117	Spencer ar	1987	15525	Mollusca	7805	Bivalvia
Late Pleistocene	0.126	0.0117	Spencer ar	1987	15525	Mollusca	7805	Bivalvia
Late Pliocene	3.6	2.588	Edwards et	2005	17631	Mollusca	7805	Bivalvia
Late Pliocene	3.6	2.588	Edwards et	2005	17631	Mollusca	7805	Bivalvia
Late Pliocene	3.6	2.588	Edwards et	2005	17631	Mollusca	7805	Bivalvia
Late Pliocene	3.6	2.588	Edwards et	2005	17631	Mollusca	7805	Bivalvia

Late Pliocene		3.6	2.588	Ward	1993	26351	Mollusca	7805 Bivalvia
Late Pliocene		3.6	2.588	Ward	1993	26351	Mollusca	7805 Bivalvia
Late Pleistocene		0.126	0.0117	Sellards	1917	9011	Mollusca	7805 Bivalvia
Early Pleistocene		2.588	0.781	DuBar	1962	11119	Mollusca	7805 Bivalvia
Early Pleistocene		2.588	0.781	DuBar	1962	11119	Mollusca	7805 Bivalvia
Calabrian		1.806	0.781	DuBar	1962	11119	Mollusca	7805 Bivalvia
Late Pleistocene		0.126	0.0117	DuBar	1962	11119	Mollusca	7805 Bivalvia
Early Pleistocene		2.588	0.781	DuBar	1962	11119	Mollusca	7805 Bivalvia
Late Pleistocene		0.126	0.0117	DuBar	1962	11119	Mollusca	7805 Bivalvia
Pliocene	Pleistocen	5.333	0.0117	DuBar	1962	11119	Mollusca	7805 Bivalvia
Late Pliocene		3.6	2.588	Mansfield	1932	17094	Mollusca	7805 Bivalvia
Late Pliocene		3.6	2.588	Mansfield	1932	17094	Mollusca	7805 Bivalvia
Late Pliocene		3.6	2.588	Mansfield	1932	17094	Mollusca	7805 Bivalvia
Late Pliocene		3.6	2.588	Mansfield	1932	17094	Mollusca	7805 Bivalvia
Late Pliocene		3.6	2.588	Mansfield	1932	17094	Mollusca	7805 Bivalvia
Late Pliocene		3.6	2.588	Mansfield	1932	17094	Mollusca	7805 Bivalvia
Late Pliocene		3.6	2.588	Mansfield	1932	17094	Mollusca	7805 Bivalvia
Early Pleistocene		2.588	0.781	Portell et a	1992	17634	Mollusca	7805 Bivalvia
Middle Pleistocene		0.781	0.126	Portell et a	1992	17634	Mollusca	7805 Bivalvia
Middle Pleistocene		0.781	0.126	McGinty	1970	19114	Mollusca	7805 Bivalvia
Pliocene		5.333	2.588	Schroeder	1954	25303	Mollusca	7805 Bivalvia
Early Pleistocene		2.588	0.781	Olsson anc	1953	25463	Mollusca	7805 Bivalvia
Early Pleistocene		2.588	0.781	Hendy et a	2008	19909	Mollusca	7805 Bivalvia
Late Pleistocene		0.126	0.0117	Hendy et a	2008	19909	Mollusca	7805 Bivalvia
Early Pleistocene		2.588	0.781	Hendy et a	2008	19909	Mollusca	7805 Bivalvia
Late Pliocene		3.6	2.588	Hendy et a	2008	19909	Mollusca	7805 Bivalvia
Early Pleistocene		2.588	0.781	DuBar	1958	25514	Mollusca	7805 Bivalvia
Late Pleistocene		0.126	0.0117	Kittle and I	2010	40884	Mollusca	7805 Bivalvia
Late Pleistocene		0.126	0.0117	Hulbert an	1998	1962	Mollusca	7805 Bivalvia
Late Pleistocene		0.126	0.0117	Hulbert an	1998	1962	Mollusca	7805 Bivalvia
Late Pleistocene		0.126	0.0117	DuBar	1971	17556	Mollusca	7805 Bivalvia
Late Pleistocene		0.126	0.0117	DuBar	1971	17556	Mollusca	7805 Bivalvia
Pliocene		5.333	2.588	DuBar	1971	17556	Mollusca	7805 Bivalvia
Zanclean		5.333	3.6	Campbell a	1995	17558	Mollusca	7805 Bivalvia
Early Pleistocene		2.588	0.781	DuBar	1971	17556	Mollusca	7805 Bivalvia
Late Pleistocene		0.126	0.0117	Richards	1962	18974	Mollusca	7805 Bivalvia
Late Pleistocene		0.126	0.0117	Richards	1962	18974	Mollusca	7805 Bivalvia
Late Pleistocene		0.126	0.0117	Hendy et a	2008	19909	Mollusca	7805 Bivalvia
Late Pleistocene		0.126	0.0117	Hendy et a	2008	19909	Mollusca	7805 Bivalvia
Late Pleistocene		0.126	0.0117	Hendy et a	2008	19909	Mollusca	7805 Bivalvia
Late Pleistocene		0.126	0.0117	Hendy et a	2008	19909	Mollusca	7805 Bivalvia
Late Pleistocene		0.126	0.0117	Hendy et a	2008	19909	Mollusca	7805 Bivalvia
Early Pleistocene		2.588	0.781	Cooke	1936	31145	Mollusca	7805 Bivalvia
Late Pliocene		3.6	2.588	Cooke	1936	31145	Mollusca	7805 Bivalvia
Late Pliocene		3.6	2.588	Ward and I	1987	16700	Mollusca	7805 Bivalvia
Late Pliocene		3.6	2.588	Ward and I	1987	16700	Mollusca	7805 Bivalvia
Late Pliocene		3.6	2.588	Ward and I	1987	16700	Mollusca	7805 Bivalvia

Late Pliocene	3.6	2.588	Ward and I	1987	16700	Mollusca	7805	Bivalvia
Early Pleistocene	2.588	0.781	Ward and I	1987	16700	Mollusca	7805	Bivalvia
Early Pleistocene	2.588	0.781	Ward and I	1987	16700	Mollusca	7805	Bivalvia
Early Pleistocene	2.588	0.781	Ward and I	1987	16700	Mollusca	7805	Bivalvia
Late Pliocene	3.6	2.588	Gardner	1943	16984	Mollusca	7805	Bivalvia
Late Pliocene	3.6	2.588	Gardner	1943	16984	Mollusca	7805	Bivalvia
Early Pleistocene	2.588	0.781	Gardner	1943	16984	Mollusca	7805	Bivalvia
Early Pleistocene	2.588	0.781	Gardner	1943	16984	Mollusca	7805	Bivalvia
Early Pleistocene	2.588	0.781	Gardner	1943	16984	Mollusca	7805	Bivalvia
Pleistocene	2.588	0.0117	Gardner	1943	16984	Mollusca	7805	Bivalvia
Late Pliocene	3.6	2.588	Gardner	1943	16984	Mollusca	7805	Bivalvia
Early Pleistocene	2.588	0.781	Miller	1990	17561	Mollusca	7805	Bivalvia
Early Pleistocene	2.588	0.781	Miller	1990	17561	Mollusca	7805	Bivalvia
Early Pleistocene	2.588	0.781	Miller	1990	17561	Mollusca	7805	Bivalvia
Early Pleistocene	2.588	0.781	Miller	1990	17561	Mollusca	7805	Bivalvia
Late Pleistocene	0.126	0.0117	Richards	1962	18974	Mollusca	7805	Bivalvia
Late Pleistocene	0.126	0.0117	Richards	1962	18974	Mollusca	7805	Bivalvia
Late Pleistocene	0.126	0.0117	Richards	1962	18974	Mollusca	7805	Bivalvia
Late Pleistocene	0.126	0.0117	Richards	1962	18974	Mollusca	7805	Bivalvia
Gelasian	2.588	1.806	Bailey and	1986	23840	Mollusca	7805	Bivalvia
Late Pliocene	3.6	2.588	Hendy et a	2008	19909	Mollusca	7805	Bivalvia
Early Pleistocene	2.588	0.781	Hendy et a	2008	19909	Mollusca	7805	Bivalvia
Early Pleistocene	2.588	0.781	Hendy et a	2008	19909	Mollusca	7805	Bivalvia
Early Pleistocene	2.588	0.781	Miller and	1988	72890	Mollusca	7805	Bivalvia
Early Pleistocene	2.588	0.781	Miller and	1988	72890	Mollusca	7805	Bivalvia
Late Pleistocene	0.126	0.0117	Spencer ar	1987	15525	Mollusca	7805	Bivalvia
Late Pleistocene	0.126	0.0117	Spencer ar	1987	15525	Mollusca	7805	Bivalvia
Late Pleistocene	0.126	0.0117	Spencer ar	1987	15525	Mollusca	7805	Bivalvia
Late Pleistocene	0.126	0.0117	Spencer ar	1987	15525	Mollusca	7805	Bivalvia
Late Pleistocene	0.126	0.0117	Spencer ar	1987	15525	Mollusca	7805	Bivalvia
Late Pliocene	3.6	2.588	Edwards et	2005	17631	Mollusca	7805	Bivalvia
Late Pliocene	3.6	2.588	Edwards et	2005	17631	Mollusca	7805	Bivalvia
Late Pliocene	3.6	2.588	Edwards et	2005	17631	Mollusca	7805	Bivalvia
Late Pliocene	3.6	2.588	Edwards et	2005	17631	Mollusca	7805	Bivalvia
Late Pliocene	3.6	2.588	Ward	1993	26351	Mollusca	7805	Bivalvia
Late Pliocene	3.6	2.588	Ward	1993	26351	Mollusca	7805	Bivalvia
Late Pliocene	3.6	2.588	Ward	1993	26351	Mollusca	7805	Bivalvia
Late Pleistocene	0.126	0.0117	Hendy et a	2008	19909	Mollusca	7805	Bivalvia
Late Pleistocene	0.126	0.0117	Hendy et a	2008	19909	Mollusca	7805	Bivalvia
Early Pleistocene	2.588	0.781	Mansfield	1932	11083	Mollusca	7805	Bivalvia
Early Pleistocene	2.588	0.781	Mansfield	1932	11083	Mollusca	7805	Bivalvia
Early Pleistocene	2.588	0.781	Mansfield	1932	11083	Mollusca	7805	Bivalvia
Early Pliocene	5.333	3.6	DuBar	1962	11119	Mollusca	7805	Bivalvia
Early Pliocene	5.333	3.6	DuBar	1962	11119	Mollusca	7805	Bivalvia
Early Pleistocene	2.588	0.781	DuBar	1962	11119	Mollusca	7805	Bivalvia
Early Pleistocene	2.588	0.781	DuBar	1962	11119	Mollusca	7805	Bivalvia
Early Pleistocene	2.588	0.781	DuBar	1962	11119	Mollusca	7805	Bivalvia

Calabrian		1.806	0.781	DuBar	1962	11119	Mollusca	7805	Bivalvia
Calabrian		1.806	0.781	DuBar	1962	11119	Mollusca	7805	Bivalvia
Early Pleistocene		2.588	0.781	DuBar	1962	11119	Mollusca	7805	Bivalvia
Early Pleistocene		2.588	0.781	DuBar	1962	11119	Mollusca	7805	Bivalvia
Early Pleistocene		2.588	0.781	DuBar	1962	11119	Mollusca	7805	Bivalvia
Pliocene	Pleistocene	5.333	0.0117	DuBar	1962	11119	Mollusca	7805	Bivalvia
Pliocene	Pleistocene	5.333	0.0117	DuBar	1962	11119	Mollusca	7805	Bivalvia
Pliocene	Pleistocene	5.333	0.0117	DuBar	1962	11119	Mollusca	7805	Bivalvia
Pliocene	Pleistocene	5.333	0.0117	DuBar	1962	11119	Mollusca	7805	Bivalvia
Irvingtonian		1.8	0.3	Hulbert an	1989	1960	Mollusca	7805	Bivalvia
Late Pliocene		3.6	2.588	Mansfield	1932	17094	Mollusca	7805	Bivalvia
Late Pliocene		3.6	2.588	Mansfield	1932	17094	Mollusca	7805	Bivalvia
Late Pliocene		3.6	2.588	Mansfield	1932	17094	Mollusca	7805	Bivalvia
Late Pliocene		3.6	2.588	Mansfield	1932	17094	Mollusca	7805	Bivalvia
Early Pleistocene		2.588	0.781	Portell et a	1992	17634	Mollusca	7805	Bivalvia
Early Pleistocene		2.588	0.781	Portell et a	1992	17634	Mollusca	7805	Bivalvia
Middle Pleistocene		0.781	0.126	Portell et a	1992	17634	Mollusca	7805	Bivalvia
Late Pleistocene		0.126	0.0117	Portell et a	1992	17634	Mollusca	7805	Bivalvia
Middle Pleistocene		0.781	0.126	McGinty	1970	19114	Mollusca	7805	Bivalvia
Middle Pleistocene		0.781	0.126	McGinty	1970	19114	Mollusca	7805	Bivalvia
Middle Pleistocene		0.781	0.126	McGinty	1970	19114	Mollusca	7805	Bivalvia
Late Pliocene		3.6	2.588	Mansfield	1939	19112	Mollusca	7805	Bivalvia
Late Pliocene		3.6	2.588	Mansfield	1939	19112	Mollusca	7805	Bivalvia
Late Pliocene		3.6	2.588	Mansfield	1939	19112	Mollusca	7805	Bivalvia
Late Pliocene		3.6	2.588	Mansfield	1939	19112	Mollusca	7805	Bivalvia
Late Pliocene		3.6	2.588	Mansfield	1939	19112	Mollusca	7805	Bivalvia
Late Pliocene		3.6	2.588	Mansfield	1939	19112	Mollusca	7805	Bivalvia
Late Pliocene		3.6	2.588	Mansfield	1939	19112	Mollusca	7805	Bivalvia
Early Pleistocene		2.588	0.781	Schroeder	1954	25303	Mollusca	7805	Bivalvia
Early Pleistocene		2.588	0.781	Schroeder	1954	25303	Mollusca	7805	Bivalvia
Pliocene		5.333	2.588	Schroeder	1954	25303	Mollusca	7805	Bivalvia
Early Pleistocene		2.588	0.781	Schroeder	1954	25303	Mollusca	7805	Bivalvia
Pliocene		5.333	2.588	Schroeder	1954	25303	Mollusca	7805	Bivalvia
Pliocene		5.333	2.588	Schroeder	1954	25303	Mollusca	7805	Bivalvia
Early Pleistocene		2.588	0.781	Schroeder	1954	25303	Mollusca	7805	Bivalvia
Pliocene		5.333	2.588	Schroeder	1954	25303	Mollusca	7805	Bivalvia
Pliocene		5.333	2.588	Schroeder	1954	25303	Mollusca	7805	Bivalvia
Pliocene		5.333	2.588	Schroeder	1954	25303	Mollusca	7805	Bivalvia
Pliocene		5.333	2.588	Schroeder	1954	25303	Mollusca	7805	Bivalvia
Pliocene		5.333	2.588	Schroeder	1954	25303	Mollusca	7805	Bivalvia
Early Pleistocene		2.588	0.781	Olsson anc	1953	25463	Mollusca	7805	Bivalvia
Early Pleistocene		2.588	0.781	Olsson anc	1953	25463	Mollusca	7805	Bivalvia
Early Pleistocene		2.588	0.781	Olsson anc	1953	25463	Mollusca	7805	Bivalvia
Early Pleistocene		2.588	0.781	Olsson anc	1953	25463	Mollusca	7805	Bivalvia
Early Pleistocene		2.588	0.781	Olsson anc	1953	25463	Mollusca	7805	Bivalvia
Early Pleistocene		2.588	0.781	Olsson anc	1953	25463	Mollusca	7805	Bivalvia
Early Pleistocene		2.588	0.781	Olsson anc	1953	25463	Mollusca	7805	Bivalvia

Early Pleistocene		2.588	0.781	Olsson anc	1953	25463	Mollusca	7805 Bivalvia
Late Pleistocene		0.126	0.0117	Lovejoy	1992	26806	Mollusca	7805 Bivalvia
Early Pleistocene		2.588	0.781	Petuch anc	2007	26813	Mollusca	7805 Bivalvia
Middle Pleistocene		0.781	0.126	Petuch anc	2007	26813	Mollusca	7805 Bivalvia
Early Pliocene		5.333	3.6	Olsson anc	1964	26836	Mollusca	7805 Bivalvia
Early Pliocene		5.333	3.6	Olsson anc	1964	26836	Mollusca	7805 Bivalvia
Early Pliocene		5.333	3.6	Olsson anc	1964	26836	Mollusca	7805 Bivalvia
Early Pliocene		5.333	3.6	Olsson anc	1964	26836	Mollusca	7805 Bivalvia
Late Pliocene		3.6	2.588	DuBar	1958	25514	Mollusca	7805 Bivalvia
Late Pliocene		3.6	2.588	DuBar	1958	25514	Mollusca	7805 Bivalvia
Late Plioce	Early Pleist	3.6	0.781	Petuch anc	2007	26813	Mollusca	7805 Bivalvia
Late Plioce	Pleistocen	3.6	0.0117	Petuch anc	2007	26813	Mollusca	7805 Bivalvia
Early Pleistocene		2.588	0.781	Hendy et a	2008	19909	Mollusca	7805 Bivalvia
Late Pleistocene		0.126	0.0117	Hendy et a	2008	19909	Mollusca	7805 Bivalvia
Early Pliocene		5.333	3.6	Petuch anc	2007	26813	Mollusca	7805 Bivalvia
Late Pliocene		3.6	2.588	Hendy et a	2008	19909	Mollusca	7805 Bivalvia
Early Pleistocene		2.588	0.781	Hendy et a	2008	19909	Mollusca	7805 Bivalvia
Early Pleistocene		2.588	0.781	Hendy et a	2008	19909	Mollusca	7805 Bivalvia
Early Pleistocene		2.588	0.781	Hendy et a	2008	19909	Mollusca	7805 Bivalvia
Early Pleistocene		2.588	0.781	Hendy et a	2008	19909	Mollusca	7805 Bivalvia
Late Pliocene		3.6	2.588	Hendy et a	2008	19909	Mollusca	7805 Bivalvia
Late Pliocene		3.6	2.588	Hendy et a	2008	19909	Mollusca	7805 Bivalvia
Late Pliocene		3.6	2.588	Hendy et a	2008	19909	Mollusca	7805 Bivalvia
Late Pliocene		3.6	2.588	Hendy et a	2008	19909	Mollusca	7805 Bivalvia
Middle Pleistocene		0.781	0.126	Hendy et a	2008	19909	Mollusca	7805 Bivalvia
Late Pleistocene		0.126	0.0117	Hendy et a	2008	19909	Mollusca	7805 Bivalvia
Late Pleistocene		0.126	0.0117	Hendy et a	2008	19909	Mollusca	7805 Bivalvia
Early Pleistocene		2.588	0.781	Hendy et a	2008	19909	Mollusca	7805 Bivalvia
Early Pleistocene		2.588	0.781	Hendy et a	2008	19909	Mollusca	7805 Bivalvia
Late Pliocene		3.6	2.588	Smith	1941	32333	Mollusca	7805 Bivalvia
Late Pliocene		3.6	2.588	Weisbord	1971	39743	Mollusca	7805 Bivalvia
Late Pliocene		3.6	2.588	Weisbord	1971	39743	Mollusca	7805 Bivalvia
Late Pleistocene		0.126	0.0117	Kittle and F	2010	40884	Mollusca	7805 Bivalvia
Late Pleistocene		0.126	0.0117	Kittle and F	2010	40884	Mollusca	7805 Bivalvia
Late Pliocene		3.6	2.588	Ketcher an	1993	58350	Mollusca	7805 Bivalvia
Late Pleistocene		0.126	0.0117	Richards	1962	18974	Mollusca	7805 Bivalvia
Late Pleistocene		0.126	0.0117	Richards	1962	18974	Mollusca	7805 Bivalvia
Late Pleistocene		0.126	0.0117	Richards	1962	18974	Mollusca	7805 Bivalvia
Late Pleistocene		0.126	0.0117	Richards	1962	18974	Mollusca	7805 Bivalvia
Early Pleistocene		2.588	0.781	DuBar	1971	17556	Mollusca	7805 Bivalvia
Early Pleistocene		2.588	0.781	DuBar	1971	17556	Mollusca	7805 Bivalvia
Early Pleistocene		2.588	0.781	DuBar	1971	17556	Mollusca	7805 Bivalvia
Pliocene		5.333	2.588	DuBar	1971	17556	Mollusca	7805 Bivalvia
Pliocene		5.333	2.588	DuBar	1971	17556	Mollusca	7805 Bivalvia
Pliocene		5.333	2.588	DuBar	1971	17556	Mollusca	7805 Bivalvia
Late Pleistocene		0.126	0.0117	DuBar	1971	17556	Mollusca	7805 Bivalvia
Late Pleistocene		0.126	0.0117	DuBar	1971	17556	Mollusca	7805 Bivalvia

Late Pleistocene	0.126	0.0117	DuBar	1971	17556	Mollusca	7805	Bivalvia
Late Pleistocene	0.126	0.0117	DuBar	1971	17556	Mollusca	7805	Bivalvia
Late Pleistocene	0.126	0.0117	DuBar	1971	17556	Mollusca	7805	Bivalvia
Late Pleistocene	0.126	0.0117	DuBar	1971	17556	Mollusca	7805	Bivalvia
Late Pleistocene	0.126	0.0117	DuBar	1971	17556	Mollusca	7805	Bivalvia
Pliocene	5.333	2.588	DuBar	1971	17556	Mollusca	7805	Bivalvia
Pliocene	5.333	2.588	DuBar	1971	17556	Mollusca	7805	Bivalvia
Zanclean	5.333	3.6	Campbell a	1995	17558	Mollusca	7805	Bivalvia
Zanclean	5.333	3.6	Campbell a	1995	17558	Mollusca	7805	Bivalvia
Zanclean	5.333	3.6	Campbell a	1995	17558	Mollusca	7805	Bivalvia
Early Pliocene	5.333	3.6	Campbell a	1995	17558	Mollusca	7805	Bivalvia
Early Pleistocene	2.588	0.781	DuBar	1971	17556	Mollusca	7805	Bivalvia
Early Pleistocene	2.588	0.781	DuBar	1971	17556	Mollusca	7805	Bivalvia
Early Pleistocene	2.588	0.781	Richards	1962	18974	Mollusca	7805	Bivalvia
Late Pleistocene	0.126	0.0117	Richards	1962	18974	Mollusca	7805	Bivalvia
Late Pleistocene	0.126	0.0117	Richards	1962	18974	Mollusca	7805	Bivalvia
Late Pleistocene	0.126	0.0117	Richards	1962	18974	Mollusca	7805	Bivalvia
Late Pleistocene	0.126	0.0117	Hendy et a	2008	19909	Mollusca	7805	Bivalvia
Late Pleistocene	0.126	0.0117	Hendy et a	2008	19909	Mollusca	7805	Bivalvia
Late Pleistocene	0.126	0.0117	Hendy et a	2008	19909	Mollusca	7805	Bivalvia
Pleistocene	2.588	0.0117	Hendy et a	2008	19909	Mollusca	7805	Bivalvia
Pleistocene	2.588	0.0117	Hendy et a	2008	19909	Mollusca	7805	Bivalvia
Late Pleistocene	0.126	0.0117	Cooke	1936	31145	Mollusca	7805	Bivalvia
Early Pleistocene	2.588	0.781	Cooke	1936	31145	Mollusca	7805	Bivalvia
Early Pleistocene	2.588	0.781	Cooke	1936	31145	Mollusca	7805	Bivalvia
Early Pleistocene	2.588	0.781	Cooke	1936	31145	Mollusca	7805	Bivalvia
Early Pleistocene	2.588	0.781	Cooke	1936	31145	Mollusca	7805	Bivalvia
Early Pleistocene	2.588	0.781	Cooke	1936	31145	Mollusca	7805	Bivalvia
Late Pliocene	3.6	2.588	Cooke	1936	31145	Mollusca	7805	Bivalvia
Late Pliocene	3.6	2.588	Cooke	1936	31145	Mollusca	7805	Bivalvia
Pleistocene	2.588	0.0117	Holmes	1860	64712	Mollusca	7805	Bivalvia
Middle Pleistocene	0.781	0.126	Holmes	1860	64712	Mollusca	7805	Bivalvia
Late Pliocene	3.6	2.588	Ward and I	1987	16700	Mollusca	7805	Bivalvia
Late Pliocene	3.6	2.588	Ward and I	1987	16700	Mollusca	7805	Bivalvia
Late Pliocene	3.6	2.588	Ward and I	1987	16700	Mollusca	7805	Bivalvia
Early Pleistocene	2.588	0.781	Ward and I	1987	16700	Mollusca	7805	Bivalvia
Early Pleistocene	2.588	0.781	Ward and I	1987	16700	Mollusca	7805	Bivalvia
Early Pliocene	5.333	3.6	Ward	1992	16985	Mollusca	7805	Bivalvia
Late Pliocene	3.6	2.588	Ward	1992	16985	Mollusca	7805	Bivalvia
Late Pliocene	3.6	2.588	Ward	1992	16985	Mollusca	7805	Bivalvia
Pliocene	5.333	2.588	DuBar	1971	17556	Mollusca	7805	Bivalvia
Pliocene	5.333	2.588	DuBar	1971	17556	Mollusca	7805	Bivalvia
Early Pleistocene	2.588	0.781	Miller	1990	17561	Mollusca	7805	Bivalvia
Early Pleistocene	2.588	0.781	Miller	1990	17561	Mollusca	7805	Bivalvia
Early Pleistocene	2.588	0.781	Miller	1990	17561	Mollusca	7805	Bivalvia
Early Pleistocene	2.588	0.781	Miller	1990	17561	Mollusca	7805	Bivalvia
Late Pleistocene	0.126	0.0117	Richards	1962	18974	Mollusca	7805	Bivalvia

Late Pleistocene	0.126	0.0117	Richards	1962	18974	Mollusca	7805	Bivalvia
Late Pleistocene	0.126	0.0117	Richards	1962	18974	Mollusca	7805	Bivalvia
Late Pleistocene	0.126	0.0117	Richards	1962	18974	Mollusca	7805	Bivalvia
Late Pleistocene	0.126	0.0117	Richards	1962	18974	Mollusca	7805	Bivalvia
Late Pleistocene	0.126	0.0117	Richards	1962	18974	Mollusca	7805	Bivalvia
Late Pleistocene	0.126	0.0117	Richards	1962	18974	Mollusca	7805	Bivalvia
Late Pleistocene	0.126	0.0117	Richards	1962	18974	Mollusca	7805	Bivalvia
Late Pleistocene	0.126	0.0117	Richards	1962	18974	Mollusca	7805	Bivalvia
Late Pliocene	3.6	2.588	Hendy et a	2008	19909	Mollusca	7805	Bivalvia
Late Pliocene	3.6	2.588	Hendy et a	2008	19909	Mollusca	7805	Bivalvia
Late Pleistocene	0.126	0.0117	Hendy et a	2008	19909	Mollusca	7805	Bivalvia
Late Pleistocene	0.126	0.0117	Hendy et a	2008	19909	Mollusca	7805	Bivalvia
Gelasian	2.588	1.806	Bailey and	1986	23840	Mollusca	7805	Bivalvia
Early Pleistocene	2.588	0.781	Hendy et a	2008	19909	Mollusca	7805	Bivalvia
Late Pleistocene	0.126	0.0117	Hendy et a	2008	19909	Mollusca	7805	Bivalvia
Late Pliocene	3.6	2.588	Hendy et a	2008	19909	Mollusca	7805	Bivalvia
Late Pliocene	3.6	2.588	Hendy et a	2008	19909	Mollusca	7805	Bivalvia
Pliocene	5.333	2.588	Hendy et a	2008	19909	Mollusca	7805	Bivalvia
Pliocene	5.333	2.588	Hendy et a	2008	19909	Mollusca	7805	Bivalvia
Late Pliocene	3.6	2.588	Hendy et a	2008	19909	Mollusca	7805	Bivalvia
Late Pliocene	3.6	2.588	Hendy et a	2008	19909	Mollusca	7805	Bivalvia
Late Pliocene	3.6	2.588	Hendy et a	2008	19909	Mollusca	7805	Bivalvia
Early Pleistocene	2.588	0.781	Hendy et a	2008	19909	Mollusca	7805	Bivalvia
Early Pleistocene	2.588	0.781	Hendy et a	2008	19909	Mollusca	7805	Bivalvia
Late Pliocene	3.6	2.588	Hendy et a	2008	19909	Mollusca	7805	Bivalvia
Early Pleistocene	2.588	0.781	Hendy et a	2008	19909	Mollusca	7805	Bivalvia
Early Pleistocene	2.588	0.781	Hendy et a	2008	19909	Mollusca	7805	Bivalvia
Early Pleistocene	2.588	0.781	Hendy et a	2008	19909	Mollusca	7805	Bivalvia
Early Pleistocene	2.588	0.781	Hendy et a	2008	19909	Mollusca	7805	Bivalvia
Early Pleistocene	2.588	0.781	Hendy et a	2008	19909	Mollusca	7805	Bivalvia
Early Pleistocene	2.588	0.781	Hendy et a	2008	19909	Mollusca	7805	Bivalvia
Early Pleistocene	2.588	0.781	Miller and	1988	72890	Mollusca	7805	Bivalvia
Early Pleistocene	2.588	0.781	Miller and	1988	72890	Mollusca	7805	Bivalvia
Early Pleistocene	2.588	0.781	Miller and	1988	72890	Mollusca	7805	Bivalvia
Early Pleistocene	2.588	0.781	Miller and	1988	72890	Mollusca	7805	Bivalvia
Pleistocene	2.588	0.0117	Coch	1971	441	Mollusca	7805	Bivalvia
Late Pleistocene	0.126	0.0117	Spencer ar	1987	15525	Mollusca	7805	Bivalvia
Late Pleistocene	0.126	0.0117	Spencer ar	1987	15525	Mollusca	7805	Bivalvia
Late Pleistocene	0.126	0.0117	Spencer ar	1987	15525	Mollusca	7805	Bivalvia
Late Pleistocene	0.126	0.0117	Spencer ar	1987	15525	Mollusca	7805	Bivalvia
Late Pleistocene	0.126	0.0117	Spencer ar	1987	15525	Mollusca	7805	Bivalvia
Early Pliocene	5.333	3.6	Ward	1992	16985	Mollusca	7805	Bivalvia
Late Pliocene	3.6	2.588	Ward	1992	16985	Mollusca	7805	Bivalvia
Late Pliocene	3.6	2.588	Ward	1992	16985	Mollusca	7805	Bivalvia
Late Pliocene	3.6	2.588	Ward	1985	17420	Mollusca	7805	Bivalvia
Late Pleistocene	0.126	0.0117	Hendy et a	2008	19909	Mollusca	7805	Bivalvia
Late Pleistocene	0.126	0.0117	Hendy et a	2008	19909	Mollusca	7805	Bivalvia

Zanclean	5.333	3.6	Ward	1992	16985	Mollusca	7805	Bivalvia
Late Pliocene	3.6	2.588	Ward	1992	16985	Mollusca	7805	Bivalvia
Late Pliocene	3.6	2.588	Ward	1992	16985	Mollusca	7805	Bivalvia
Zanclean	5.333	3.6	Ward	1992	16985	Mollusca	7805	Bivalvia
Late Pliocene	3.6	2.588	Ward	1992	16985	Mollusca	7805	Bivalvia
Late Pliocene	3.6	2.588	Ward	1993	26351	Mollusca	7805	Bivalvia
Late Pliocene	3.6	2.588	Ward	1993	26351	Mollusca	7805	Bivalvia
Late Pliocene	3.6	2.588	Ward	1993	26351	Mollusca	7805	Bivalvia
Late Pliocene	3.6	2.588	Ward	1993	26351	Mollusca	7805	Bivalvia
Late Pliocene	3.6	2.588	Ward	1993	26351	Mollusca	7805	Bivalvia
Late Pliocene	3.6	2.588	Ward	1993	26351	Mollusca	7805	Bivalvia
Late Pliocene	3.6	2.588	Ward	1993	26351	Mollusca	7805	Bivalvia
Late Pliocene	3.6	2.588	Ward	1993	26351	Mollusca	7805	Bivalvia
Late Pliocene	3.6	2.588	Ward	1993	26351	Mollusca	7805	Bivalvia
Late Pliocene	3.6	2.588	Ward	1993	26351	Mollusca	7805	Bivalvia
Late Pliocene	3.6	2.588	Ward	1993	26351	Mollusca	7805	Bivalvia
Late Pliocene	3.6	2.588	Ward	1993	26351	Mollusca	7805	Bivalvia
Late Pliocene	3.6	2.588	Hendy et a	2008	19909	Mollusca	7805	Bivalvia
Late Pliocene	3.6	2.588	Hendy et a	2008	19909	Mollusca	7805	Bivalvia
Late Pliocene	3.6	2.588	Hendy et a	2008	19909	Mollusca	7805	Bivalvia
Late Pliocene	3.6	2.588	Hendy et a	2008	19909	Mollusca	7805	Bivalvia
Late Pliocene	3.6	2.588	Hendy et a	2008	19909	Mollusca	7805	Bivalvia
Late Pliocene	3.6	2.588	Hendy et a	2008	19909	Mollusca	7805	Bivalvia
Late Pliocene	3.6	2.588	Hendy et a	2008	19909	Mollusca	7805	Bivalvia
Late Pliocene	3.6	2.588	Hendy et a	2008	19909	Mollusca	7805	Bivalvia
Calabrian	1.806	0.781	DuBar	1962	11119	Mollusca	7805	Bivalvia
Late Pliocene	3.6	2.588	Mansfield	1932	17094	Mollusca	7805	Bivalvia
Middle Pleistocene	0.781	0.126	Hoerle	1970	19536	Mollusca	7805	Bivalvia
Middle Pleistocene	0.781	0.126	Hoerle	1970	19536	Mollusca	7805	Bivalvia
Middle Pleistocene	0.781	0.126	Hoerle	1970	19536	Mollusca	7805	Bivalvia
Early Pleistocene	2.588	0.781	Olsson anc	1953	25463	Mollusca	7805	Bivalvia
Early Pleistocene	2.588	0.781	Hendy et a	2008	19909	Mollusca	7805	Bivalvia
Early Pleistocene	2.588	0.781	Hendy et a	2008	19909	Mollusca	7805	Bivalvia
Early Pleistocene	2.588	0.781	DuBar	1971	17556	Mollusca	7805	Bivalvia
Late Pleistocene	0.126	0.0117	Richards	1962	18974	Mollusca	7805	Bivalvia
Late Pleistocene	0.126	0.0117	Richards	1962	18974	Mollusca	7805	Bivalvia
Late Pliocene	3.6	2.588	Ward and I	1987	16700	Mollusca	7805	Bivalvia
Late Pliocene	3.6	2.588	Ward and I	1987	16700	Mollusca	7805	Bivalvia
Early Pleistocene	2.588	0.781	Ward and I	1987	16700	Mollusca	7805	Bivalvia
Early Pleistocene	2.588	0.781	Gardner	1943	16984	Mollusca	7805	Bivalvia
Early Pleistocene	2.588	0.781	Gardner	1943	16984	Mollusca	7805	Bivalvia
Pleistocene	2.588	0.0117	Gardner	1943	16984	Mollusca	7805	Bivalvia
Pleistocene	2.588	0.0117	Gardner	1943	16984	Mollusca	7805	Bivalvia
Late Pleistocene	0.126	0.0117	Richards	1962	18974	Mollusca	7805	Bivalvia
Late Pleistocene	0.126	0.0117	Richards	1962	18974	Mollusca	7805	Bivalvia
Late Pleistocene	0.126	0.0117	Richards	1962	18974	Mollusca	7805	Bivalvia
Late Pleistocene	0.126	0.0117	Richards	1962	18974	Mollusca	7805	Bivalvia

Late Pleistocene	0.126	0.0117	Richards	1962	18974	Mollusca	7805	Bivalvia
Late Pleistocene	0.126	0.0117	Hendy et a	2008	19909	Mollusca	7805	Bivalvia
Gelasian	2.588	1.806	Bailey and	1986	23840	Mollusca	7805	Bivalvia
Late Pliocene	3.6	2.588	Hendy et a	2008	19909	Mollusca	7805	Bivalvia
Early Pleistocene	2.588	0.781	Hendy et a	2008	19909	Mollusca	7805	Bivalvia
Early Pleistocene	2.588	0.781	Hendy et a	2008	19909	Mollusca	7805	Bivalvia
Late Pleistocene	0.126	0.0117	Spencer ar	1987	15525	Mollusca	7805	Bivalvia
Late Pleistocene	0.126	0.0117	Spencer ar	1987	15525	Mollusca	7805	Bivalvia
Late Pleistocene	0.126	0.0117	Spencer ar	1987	15525	Mollusca	7805	Bivalvia
Late Pleistocene	0.126	0.0117	Spencer ar	1987	15525	Mollusca	7805	Bivalvia
Late Pleistocene	0.126	0.0117	Spencer ar	1987	15525	Mollusca	7805	Bivalvia
Late Pliocene	3.6	2.588	Ward	1993	26351	Mollusca	7805	Bivalvia
Late Pliocene	3.6	2.588	Ward	1993	26351	Mollusca	7805	Bivalvia
Late Pliocene	3.6	2.588	Ward	1993	26351	Mollusca	7805	Bivalvia
Late Pliocene	3.6	2.588	Ward	1993	26351	Mollusca	7805	Bivalvia
Late Pliocene	3.6	2.588	Ward	1993	26351	Mollusca	7805	Bivalvia
Late Pliocene	3.6	2.588	Ward	1993	26351	Mollusca	7805	Bivalvia
Late Pliocene	3.6	2.588	Ward	1993	26351	Mollusca	7805	Bivalvia
Late Pliocene	3.6	2.588	Hendy et a	2008	19909	Mollusca	7805	Bivalvia
Late Pleistocene	0.126	0.0117	Sellards	1917	9011	Mollusca	7805	Bivalvia
Early Pleistocene	2.588	0.781	Mansfield	1932	11083	Mollusca	7805	Bivalvia
Early Pleistocene	2.588	0.781	Mansfield	1932	11083	Mollusca	7805	Bivalvia
Early Pleistocene	2.588	0.781	Mansfield	1932	11083	Mollusca	7805	Bivalvia
Early Pleistocene	2.588	0.781	Mansfield	1932	11083	Mollusca	7805	Bivalvia
Early Pleistocene	2.588	0.781	Mansfield	1932	11083	Mollusca	7805	Bivalvia
Early Pleistocene	2.588	0.781	Mansfield	1932	11083	Mollusca	7805	Bivalvia
Early Pleistocene	2.588	0.781	Mansfield	1932	11083	Mollusca	7805	Bivalvia
Early Pleistocene	2.588	0.781	Mansfield	1932	11083	Mollusca	7805	Bivalvia
Early Pleistocene	2.588	0.781	Mansfield	1932	11083	Mollusca	7805	Bivalvia
Early Pleistocene	2.588	0.781	Mansfield	1932	11083	Mollusca	7805	Bivalvia
Early Pleistocene	2.588	0.781	Mansfield	1932	11083	Mollusca	7805	Bivalvia
Early Pleistocene	2.588	0.781	Mansfield	1932	11083	Mollusca	7805	Bivalvia
Early Pliocene	5.333	3.6	DuBar	1962	11119	Mollusca	7805	Bivalvia
Early Pleistocene	2.588	0.781	DuBar	1962	11119	Mollusca	7805	Bivalvia
Early Pleistocene	2.588	0.781	DuBar	1962	11119	Mollusca	7805	Bivalvia
Early Pleistocene	2.588	0.781	DuBar	1962	11119	Mollusca	7805	Bivalvia
Early Pleistocene	2.588	0.781	DuBar	1962	11119	Mollusca	7805	Bivalvia
Early Pleistocene	2.588	0.781	DuBar	1962	11119	Mollusca	7805	Bivalvia
Early Pleistocene	2.588	0.781	DuBar	1962	11119	Mollusca	7805	Bivalvia
Early Pleistocene	2.588	0.781	DuBar	1962	11119	Mollusca	7805	Bivalvia
Early Pleistocene	2.588	0.781	DuBar	1962	11119	Mollusca	7805	Bivalvia
Calabrian	1.806	0.781	DuBar	1962	11119	Mollusca	7805	Bivalvia
Calabrian	1.806	0.781	DuBar	1962	11119	Mollusca	7805	Bivalvia
Calabrian	1.806	0.781	DuBar	1962	11119	Mollusca	7805	Bivalvia
Calabrian	1.806	0.781	DuBar	1962	11119	Mollusca	7805	Bivalvia
Calabrian	1.806	0.781	DuBar	1962	11119	Mollusca	7805	Bivalvia
Late Pleistocene	0.126	0.0117	DuBar	1962	11119	Mollusca	7805	Bivalvia

Late Pleistocene		0.126	0.0117	DuBar	1962	11119	Mollusca	7805	Bivalvia
Early Pleistocene		2.588	0.781	DuBar	1962	11119	Mollusca	7805	Bivalvia
Early Pleistocene		2.588	0.781	DuBar	1962	11119	Mollusca	7805	Bivalvia
Early Pleistocene		2.588	0.781	DuBar	1962	11119	Mollusca	7805	Bivalvia
Pliocene	Pleistocene	5.333	0.0117	DuBar	1962	11119	Mollusca	7805	Bivalvia
Pliocene	Pleistocene	5.333	0.0117	DuBar	1962	11119	Mollusca	7805	Bivalvia
Pliocene	Pleistocene	5.333	0.0117	DuBar	1962	11119	Mollusca	7805	Bivalvia
Pliocene	Pleistocene	5.333	0.0117	DuBar	1962	11119	Mollusca	7805	Bivalvia
Pliocene	Pleistocene	5.333	0.0117	DuBar	1962	11119	Mollusca	7805	Bivalvia
Late Pliocene		3.6	2.588	Mansfield	1932	17094	Mollusca	7805	Bivalvia
Late Pliocene		3.6	2.588	Mansfield	1932	17094	Mollusca	7805	Bivalvia
Late Pliocene		3.6	2.588	Mansfield	1932	17094	Mollusca	7805	Bivalvia
Late Pliocene		3.6	2.588	Mansfield	1932	17094	Mollusca	7805	Bivalvia
Late Pliocene		3.6	2.588	Mansfield	1932	17094	Mollusca	7805	Bivalvia
Late Pliocene		3.6	2.588	Mansfield	1932	17094	Mollusca	7805	Bivalvia
Late Pliocene		3.6	2.588	Mansfield	1932	17094	Mollusca	7805	Bivalvia
Late Pliocene		3.6	2.588	Mansfield	1932	17094	Mollusca	7805	Bivalvia
Late Pliocene		3.6	2.588	Mansfield	1932	17094	Mollusca	7805	Bivalvia
Late Pliocene		3.6	2.588	Mansfield	1932	17094	Mollusca	7805	Bivalvia
Late Pliocene		3.6	2.588	Mansfield	1932	17094	Mollusca	7805	Bivalvia
Late Pliocene		3.6	2.588	Mansfield	1932	17094	Mollusca	7805	Bivalvia
Late Pliocene		3.6	2.588	Mansfield	1932	17094	Mollusca	7805	Bivalvia
Late Pliocene		3.6	2.588	Mansfield	1932	17094	Mollusca	7805	Bivalvia
Late Pliocene		3.6	2.588	Mansfield	1932	17094	Mollusca	7805	Bivalvia
Early Pleistocene		2.588	0.781	Portell et a	1992	17634	Mollusca	7805	Bivalvia
Early Pleistocene		2.588	0.781	Portell et a	1992	17634	Mollusca	7805	Bivalvia
Middle Pleistocene		0.781	0.126	Portell et a	1992	17634	Mollusca	7805	Bivalvia
Middle Pleistocene		0.781	0.126	Portell et a	1992	17634	Mollusca	7805	Bivalvia
Late Pleistocene		0.126	0.0117	Petuch	2004	18040	Mollusca	7805	Bivalvia
Middle Pleistocene		0.781	0.126	McGinty	1970	19114	Mollusca	7805	Bivalvia
Middle Pleistocene		0.781	0.126	McGinty	1970	19114	Mollusca	7805	Bivalvia
Middle Pleistocene		0.781	0.126	McGinty	1970	19114	Mollusca	7805	Bivalvia
Middle Pleistocene		0.781	0.126	McGinty	1970	19114	Mollusca	7805	Bivalvia
Middle Pleistocene		0.781	0.126	McGinty	1970	19114	Mollusca	7805	Bivalvia
Middle Pleistocene		0.781	0.126	McGinty	1970	19114	Mollusca	7805	Bivalvia
Middle Pleistocene		0.781	0.126	McGinty	1970	19114	Mollusca	7805	Bivalvia
Late Pliocene		3.6	2.588	Mansfield	1939	19112	Mollusca	7805	Bivalvia
Late Pliocene		3.6	2.588	Mansfield	1939	19112	Mollusca	7805	Bivalvia
Late Pliocene		3.6	2.588	Mansfield	1939	19112	Mollusca	7805	Bivalvia
Late Pliocene		3.6	2.588	Mansfield	1939	19112	Mollusca	7805	Bivalvia
Late Pliocene		3.6	2.588	Mansfield	1939	19112	Mollusca	7805	Bivalvia
Late Pliocene		3.6	2.588	Mansfield	1939	19112	Mollusca	7805	Bivalvia
Late Pliocene		3.6	2.588	Mansfield	1939	19112	Mollusca	7805	Bivalvia
Late Pliocene		3.6	2.588	Mansfield	1939	19112	Mollusca	7805	Bivalvia
Late Pliocene		3.6	2.588	Mansfield	1939	19112	Mollusca	7805	Bivalvia
Late Pliocene		3.6	2.588	Mansfield	1939	19112	Mollusca	7805	Bivalvia
Late Pliocene		3.6	2.588	Mansfield	1939	19112	Mollusca	7805	Bivalvia

Late Pliocene	3.6	2.588	Mansfield	1939	19112	Mollusca	7805	Bivalvia
Late Pleistocene	0.126	0.0117	Hendy et a	2008	19909	Mollusca	7805	Bivalvia
Pliocene	5.333	2.588	Schroeder	1954	25303	Mollusca	7805	Bivalvia
Early Pleistocene	2.588	0.781	Schroeder	1954	25303	Mollusca	7805	Bivalvia
Pliocene	5.333	2.588	Schroeder	1954	25303	Mollusca	7805	Bivalvia
Pliocene	5.333	2.588	Schroeder	1954	25303	Mollusca	7805	Bivalvia
Pliocene	5.333	2.588	Schroeder	1954	25303	Mollusca	7805	Bivalvia
Early Pleistocene	2.588	0.781	Schroeder	1954	25303	Mollusca	7805	Bivalvia
Pliocene	5.333	2.588	Schroeder	1954	25303	Mollusca	7805	Bivalvia
Pliocene	5.333	2.588	Schroeder	1954	25303	Mollusca	7805	Bivalvia
Pliocene	5.333	2.588	Schroeder	1954	25303	Mollusca	7805	Bivalvia
Early Pleistocene	2.588	0.781	Olsson anc	1953	25463	Mollusca	7805	Bivalvia
Early Pleistocene	2.588	0.781	Olsson anc	1953	25463	Mollusca	7805	Bivalvia
Early Pleistocene	2.588	0.781	Olsson anc	1953	25463	Mollusca	7805	Bivalvia
Early Pleistocene	2.588	0.781	Olsson anc	1953	25463	Mollusca	7805	Bivalvia
Early Pleistocene	2.588	0.781	Olsson anc	1953	25463	Mollusca	7805	Bivalvia
Early Pleistocene	2.588	0.781	Olsson anc	1953	25463	Mollusca	7805	Bivalvia
Early Pleistocene	2.588	0.781	Olsson anc	1953	25463	Mollusca	7805	Bivalvia
Early Pleistocene	2.588	0.781	Olsson anc	1953	25463	Mollusca	7805	Bivalvia
Early Pleistocene	2.588	0.781	Olsson anc	1953	25463	Mollusca	7805	Bivalvia
Early Pleistocene	2.588	0.781	Olsson anc	1953	25463	Mollusca	7805	Bivalvia
Early Pleistocene	2.588	0.781	Olsson anc	1953	25463	Mollusca	7805	Bivalvia
Late Pleistocene	0.126	0.0117	Lovejoy	1992	26806	Mollusca	7805	Bivalvia
Late Pleistocene	0.126	0.0117	Petuch anc	2007	26813	Mollusca	7805	Bivalvia
Late Pleistocene	0.126	0.0117	Petuch anc	2007	26813	Mollusca	7805	Bivalvia
Middle Pleistocene	0.781	0.126	Petuch anc	2007	26813	Mollusca	7805	Bivalvia
Early Pleistocene	2.588	0.781	Petuch anc	2007	26813	Mollusca	7805	Bivalvia
Early Pleistocene	2.588	0.781	Petuch anc	2007	26813	Mollusca	7805	Bivalvia
Middle Pleistocene	0.781	0.126	Petuch anc	2007	26813	Mollusca	7805	Bivalvia
Late Pliocene	3.6	2.588	Olsson	1967	19826	Mollusca	7805	Bivalvia
Early Pliocene	5.333	3.6	Olsson anc	1964	26836	Mollusca	7805	Bivalvia
Early Pliocene	5.333	3.6	Olsson anc	1964	26836	Mollusca	7805	Bivalvia
Early Pliocene	5.333	3.6	Olsson anc	1964	26836	Mollusca	7805	Bivalvia
Early Pliocene	5.333	3.6	Olsson anc	1964	26836	Mollusca	7805	Bivalvia
Early Pliocene	5.333	3.6	Olsson anc	1964	26836	Mollusca	7805	Bivalvia
Late Pliocene	3.6	2.588	DuBar	1958	25514	Mollusca	7805	Bivalvia
Late Pliocene	3.6	2.588	DuBar	1958	25514	Mollusca	7805	Bivalvia
Late Pliocene	3.6	2.588	DuBar	1958	25514	Mollusca	7805	Bivalvia
Late Pliocene	3.6	2.588	DuBar	1958	25514	Mollusca	7805	Bivalvia
Late Plioce Early Pleist	3.6	0.781	Petuch anc	2007	26813	Mollusca	7805	Bivalvia
Late Plioce Early Pleist	3.6	0.781	Petuch anc	2007	26813	Mollusca	7805	Bivalvia
Late Plioce Early Pleist	3.6	0.781	Petuch anc	2007	26813	Mollusca	7805	Bivalvia
Late Plioce Early Pleist	3.6	0.781	Petuch anc	2007	26813	Mollusca	7805	Bivalvia
Late Plioce Early Pleist	3.6	0.781	Petuch anc	2007	26813	Mollusca	7805	Bivalvia
Late Pliocene	3.6	2.588	Petuch anc	2007	26813	Mollusca	7805	Bivalvia
Late Pliocene	3.6	2.588	Petuch anc	2007	26813	Mollusca	7805	Bivalvia
Late Pliocene	3.6	2.588	Petuch anc	2007	26813	Mollusca	7805	Bivalvia

Early Pleistocene	2.588	0.781	Hendy et a	2008	19909	Mollusca	7805	Bivalvia
Early Pleistocene	2.588	0.781	Hendy et a	2008	19909	Mollusca	7805	Bivalvia
Late Pleistocene	0.126	0.0117	Hendy et a	2008	19909	Mollusca	7805	Bivalvia
Early Pliocene	5.333	3.6	Petuch and	2007	26813	Mollusca	7805	Bivalvia
Early Pliocene	5.333	3.6	Petuch and	2007	26813	Mollusca	7805	Bivalvia
Early Pliocene	5.333	3.6	Petuch and	2007	26813	Mollusca	7805	Bivalvia
Early Pliocene	5.333	3.6	Petuch and	2007	26813	Mollusca	7805	Bivalvia
Early Pliocene	5.333	3.6	Petuch and	2007	26813	Mollusca	7805	Bivalvia
Early Pliocene	5.333	3.6	Petuch and	2007	26813	Mollusca	7805	Bivalvia
Early Pliocene	5.333	3.6	Petuch and	2007	26813	Mollusca	7805	Bivalvia
Late Pliocene	3.6	2.588	Petuch and	2007	26813	Mollusca	7805	Bivalvia
Late Pliocene	3.6	2.588	Petuch and	2007	26813	Mollusca	7805	Bivalvia
Late Pliocene	3.6	2.588	Petuch and	2007	26813	Mollusca	7805	Bivalvia
Late Pliocene	3.6	2.588	Petuch and	2007	26813	Mollusca	7805	Bivalvia
Late Pliocene	3.6	2.588	Petuch and	2007	26813	Mollusca	7805	Bivalvia
Late Pliocene	3.6	2.588	Petuch and	2007	26813	Mollusca	7805	Bivalvia
Late Pliocene	3.6	2.588	Petuch and	2007	26813	Mollusca	7805	Bivalvia
Late Pliocene	3.6	2.588	Petuch and	2007	26813	Mollusca	7805	Bivalvia
Late Pliocene	3.6	2.588	Petuch and	2007	26813	Mollusca	7805	Bivalvia
Late Pliocene	3.6	2.588	Petuch and	2007	26813	Mollusca	7805	Bivalvia
Early Pleistocene	2.588	0.781	DuBar	1958	25514	Mollusca	7805	Bivalvia
Late Pleistocene	0.126	0.0117	DuBar	1958	25514	Mollusca	7805	Bivalvia
Early Pleistocene	2.588	0.781	Hendy et a	2008	19909	Mollusca	7805	Bivalvia
Early Pleistocene	2.588	0.781	Hendy et a	2008	19909	Mollusca	7805	Bivalvia
Early Pleistocene	2.588	0.781	Hendy et a	2008	19909	Mollusca	7805	Bivalvia
Early Pleistocene	2.588	0.781	Hendy et a	2008	19909	Mollusca	7805	Bivalvia
Early Pleistocene	2.588	0.781	Hendy et a	2008	19909	Mollusca	7805	Bivalvia
Early Pleistocene	2.588	0.781	Hendy et a	2008	19909	Mollusca	7805	Bivalvia
Late Pliocene	3.6	2.588	Hendy et a	2008	19909	Mollusca	7805	Bivalvia
Late Pliocene	3.6	2.588	Hendy et a	2008	19909	Mollusca	7805	Bivalvia
Late Pliocene	3.6	2.588	Hendy et a	2008	19909	Mollusca	7805	Bivalvia
Late Pliocene	3.6	2.588	Hendy et a	2008	19909	Mollusca	7805	Bivalvia
Middle Pleistocene	0.781	0.126	Hendy et a	2008	19909	Mollusca	7805	Bivalvia
Middle Pleistocene	0.781	0.126	Hendy et a	2008	19909	Mollusca	7805	Bivalvia
Late Pleistocene	0.126	0.0117	Hendy et a	2008	19909	Mollusca	7805	Bivalvia
Early Pleistocene	2.588	0.781	Hendy et a	2008	19909	Mollusca	7805	Bivalvia
Early Pleistocene	2.588	0.781	Hendy et a	2008	19909	Mollusca	7805	Bivalvia
Early Pleistocene	2.588	0.781	DuBar	1958	25514	Mollusca	7805	Bivalvia
Early Pleistocene	2.588	0.781	DuBar	1958	25514	Mollusca	7805	Bivalvia
Early Pleistocene	2.588	0.781	DuBar	1958	25514	Mollusca	7805	Bivalvia
Late Pliocene	3.6	2.588	Weisbord	1974	28823	Mollusca	7805	Bivalvia
Late Pliocene	3.6	2.588	Weisbord	1974	28823	Mollusca	7805	Bivalvia
Late Pliocene	3.6	2.588	Weisbord	1972	31274	Mollusca	7805	Bivalvia
Late Pliocene	3.6	2.588	Weisbord	1972	31274	Mollusca	7805	Bivalvia
Late Pliocene	3.6	2.588	Smith	1941	32333	Mollusca	7805	Bivalvia
Late Pliocene	3.6	2.588	Weisbord	1971	39743	Mollusca	7805	Bivalvia
Late Pliocene	3.6	2.588	Weisbord	1971	39743	Mollusca	7805	Bivalvia

Late Pliocene	3.6	2.588	Waller	2011	37262	Mollusca	7805	Bivalvia
Early Pliocene	5.333	3.6	Waller	2011	37262	Mollusca	7805	Bivalvia
Late Pliocene	3.6	2.588	Waller	2011	37262	Mollusca	7805	Bivalvia
Early Pleistocene	2.588	0.781	Waller	2011	37262	Mollusca	7805	Bivalvia
Middle Pleistocene	0.781	0.126	Waller	2011	37262	Mollusca	7805	Bivalvia
Late Pleistocene	0.126	0.0117	Kittle and I	2010	40884	Mollusca	7805	Bivalvia
Late Pleistocene	0.126	0.0117	Kittle and I	2010	40884	Mollusca	7805	Bivalvia
Late Pleistocene	0.126	0.0117	Kittle and I	2010	40884	Mollusca	7805	Bivalvia
Late Pliocene	3.6	2.588	Ketcher an	1993	58350	Mollusca	7805	Bivalvia
Late Pliocene	3.6	2.588	Ketcher an	1993	58350	Mollusca	7805	Bivalvia
Late Pleistocene	0.126	0.0117	Garrison et	2012	43635	Mollusca	7805	Bivalvia
Early Pleistocene	2.588	0.781	DuBar	1971	17556	Mollusca	7805	Bivalvia
Pliocene	5.333	2.588	DuBar	1971	17556	Mollusca	7805	Bivalvia
Late Pleistocene	0.126	0.0117	DuBar	1971	17556	Mollusca	7805	Bivalvia
Pliocene	5.333	2.588	DuBar	1971	17556	Mollusca	7805	Bivalvia
Pliocene	5.333	2.588	DuBar	1971	17556	Mollusca	7805	Bivalvia
Pliocene	5.333	2.588	DuBar	1971	17556	Mollusca	7805	Bivalvia
Pliocene	5.333	2.588	DuBar	1971	17556	Mollusca	7805	Bivalvia
Pliocene	5.333	2.588	DuBar	1971	17556	Mollusca	7805	Bivalvia
Pliocene	5.333	2.588	DuBar	1971	17556	Mollusca	7805	Bivalvia
Late Pleistocene	0.126	0.0117	DuBar	1971	17556	Mollusca	7805	Bivalvia
Late Pleistocene	0.126	0.0117	DuBar	1971	17556	Mollusca	7805	Bivalvia
Pliocene	5.333	2.588	DuBar	1971	17556	Mollusca	7805	Bivalvia
Pliocene	5.333	2.588	DuBar	1971	17556	Mollusca	7805	Bivalvia
Pliocene	5.333	2.588	DuBar	1971	17556	Mollusca	7805	Bivalvia
Pliocene	5.333	2.588	DuBar	1971	17556	Mollusca	7805	Bivalvia
Pliocene	5.333	2.588	DuBar	1971	17556	Mollusca	7805	Bivalvia
Late Pliocene	3.6	2.588	Campbell a	1995	17558	Mollusca	7805	Bivalvia
Late Pliocene	3.6	2.588	Campbell a	1995	17558	Mollusca	7805	Bivalvia
Late Pliocene	3.6	2.588	Campbell a	1995	17558	Mollusca	7805	Bivalvia
Late Pliocene	3.6	2.588	Campbell a	1995	17558	Mollusca	7805	Bivalvia
Late Pliocene	3.6	2.588	Campbell a	1995	17558	Mollusca	7805	Bivalvia
Late Pliocene	3.6	2.588	Campbell a	1995	17558	Mollusca	7805	Bivalvia
Late Pliocene	3.6	2.588	Campbell a	1995	17558	Mollusca	7805	Bivalvia
Late Pliocene	3.6	2.588	Campbell a	1995	17558	Mollusca	7805	Bivalvia
Zanclean	5.333	3.6	Campbell a	1995	17558	Mollusca	7805	Bivalvia
Zanclean	5.333	3.6	Campbell a	1995	17558	Mollusca	7805	Bivalvia
Zanclean	5.333	3.6	Campbell a	1995	17558	Mollusca	7805	Bivalvia
Zanclean	5.333	3.6	Campbell a	1995	17558	Mollusca	7805	Bivalvia
Zanclean	5.333	3.6	Campbell a	1995	17558	Mollusca	7805	Bivalvia
Late Pliocene	3.6	2.588	Campbell a	1995	17558	Mollusca	7805	Bivalvia
Early Pliocene	5.333	3.6	Campbell a	1995	17558	Mollusca	7805	Bivalvia
Zanclean	5.333	3.6	Campbell a	1995	17558	Mollusca	7805	Bivalvia
Zanclean	5.333	3.6	Campbell a	1995	17558	Mollusca	7805	Bivalvia
Zanclean	5.333	3.6	Campbell a	1995	17558	Mollusca	7805	Bivalvia
Zanclean	5.333	3.6	Campbell a	1995	17558	Mollusca	7805	Bivalvia
Zanclean	5.333	3.6	Campbell a	1995	17558	Mollusca	7805	Bivalvia

Early Pliocene		5.333	3.6	Campbell a	1995	17558	Mollusca	7805	Bivalvia
Late Pliocene		3.6	2.588	Campbell a	1995	17558	Mollusca	7805	Bivalvia
Pliocene		5.333	2.588	Gibson	1987	17902	Mollusca	7805	Bivalvia
Late Pleistocene		0.126	0.0117	Richards	1962	18974	Mollusca	7805	Bivalvia
Late Pleistocene		0.126	0.0117	Hendy et a	2008	19909	Mollusca	7805	Bivalvia
Pleistocene		2.588	0.0117	Hendy et a	2008	19909	Mollusca	7805	Bivalvia
Pleistocene		2.588	0.0117	Hendy et a	2008	19909	Mollusca	7805	Bivalvia
Early Pleistocene		2.588	0.781	Cooke	1936	31145	Mollusca	7805	Bivalvia
Early Pleistocene		2.588	0.781	Cooke	1936	31145	Mollusca	7805	Bivalvia
Early Pleistocene		2.588	0.781	Cooke	1936	31145	Mollusca	7805	Bivalvia
Early Pleistocene		2.588	0.781	Cooke	1936	31145	Mollusca	7805	Bivalvia
Early Pleistocene		2.588	0.781	Cooke	1936	31145	Mollusca	7805	Bivalvia
Early Pleistocene		2.588	0.781	Cooke	1936	31145	Mollusca	7805	Bivalvia
Late Pliocene		3.6	2.588	Cooke	1936	31145	Mollusca	7805	Bivalvia
Late Pliocene		3.6	2.588	Ward and	1987	16700	Mollusca	7805	Bivalvia
Late Pliocene		3.6	2.588	Ward and	1987	16700	Mollusca	7805	Bivalvia
Late Pliocene		3.6	2.588	Ward and	1987	16700	Mollusca	7805	Bivalvia
Early Pleistocene		2.588	0.781	Ward and	1987	16700	Mollusca	7805	Bivalvia
Early Pleistocene		2.588	0.781	Ward and	1987	16700	Mollusca	7805	Bivalvia
Early Pleistocene		2.588	0.781	Ward and	1987	16700	Mollusca	7805	Bivalvia
Early Pleistocene		2.588	0.781	Ward and	1987	16700	Mollusca	7805	Bivalvia
Early Pliocene		5.333	3.6	Ward	1992	16985	Mollusca	7805	Bivalvia
Early Pliocene		5.333	3.6	Ward	1992	16985	Mollusca	7805	Bivalvia
Late Pliocene		3.6	2.588	Ward	1992	16985	Mollusca	7805	Bivalvia
Late Pliocene		3.6	2.588	Gardner	1943	16984	Mollusca	7805	Bivalvia
Early Pleistocene		2.588	0.781	Gardner	1943	16984	Mollusca	7805	Bivalvia
Early Pleistocene		2.588	0.781	Gardner	1943	16984	Mollusca	7805	Bivalvia
Late Pliocene		3.6	2.588	Gardner	1943	16984	Mollusca	7805	Bivalvia
Early Plioc	Late Plioce	5.333	2.588	Gardner	1943	16984	Mollusca	7805	Bivalvia
Pleistocene		2.588	0.0117	Gardner	1943	16984	Mollusca	7805	Bivalvia
Early Pleistocene		2.588	0.781	Gardner	1943	16984	Mollusca	7805	Bivalvia
Late Pliocene		3.6	2.588	Gardner	1943	16984	Mollusca	7805	Bivalvia
Late Pliocene		3.6	2.588	Gardner	1943	16984	Mollusca	7805	Bivalvia
Late Pliocene		3.6	2.588	Gardner	1943	16984	Mollusca	7805	Bivalvia
Late Pliocene		3.6	2.588	Gardner	1943	16984	Mollusca	7805	Bivalvia
Late Pliocene		3.6	2.588	Gardner	1943	16984	Mollusca	7805	Bivalvia
Early Plioc	Late Plioce	5.333	2.588	Gardner	1943	16984	Mollusca	7805	Bivalvia
Late Pliocene		3.6	2.588	Gardner	1943	16984	Mollusca	7805	Bivalvia
Early Plioc	Late Plioce	5.333	2.588	Gardner	1943	16984	Mollusca	7805	Bivalvia
Early Plioc	Late Plioce	5.333	2.588	Gardner	1943	16984	Mollusca	7805	Bivalvia
Early Pleistocene		2.588	0.781	Gardner	1943	16984	Mollusca	7805	Bivalvia
Early Pleistocene		2.588	0.781	Gardner	1943	16984	Mollusca	7805	Bivalvia
Early Pleistocene		2.588	0.781	Gardner	1943	16984	Mollusca	7805	Bivalvia
Pleistocene		2.588	0.0117	Gardner	1943	16984	Mollusca	7805	Bivalvia
Early Pleistocene		2.588	0.781	Gardner	1943	16984	Mollusca	7805	Bivalvia
Early Pleistocene		2.588	0.781	Gardner	1943	16984	Mollusca	7805	Bivalvia
Early Pleistocene		2.588	0.781	Gardner	1943	16984	Mollusca	7805	Bivalvia

Late Pliocene	3.6	2.588	Gardner	1943	16984	Mollusca	7805	Bivalvia
Early Pleistocene	2.588	0.781	DuBar	1971	17556	Mollusca	7805	Bivalvia
Pliocene	5.333	2.588	DuBar	1971	17556	Mollusca	7805	Bivalvia
Pliocene	5.333	2.588	DuBar	1971	17556	Mollusca	7805	Bivalvia
Pliocene	5.333	2.588	DuBar	1971	17556	Mollusca	7805	Bivalvia
Early Pleistocene	2.588	0.781	Miller	1990	17561	Mollusca	7805	Bivalvia
Early Pleistocene	2.588	0.781	Miller	1990	17561	Mollusca	7805	Bivalvia
Early Pleistocene	2.588	0.781	Miller	1990	17561	Mollusca	7805	Bivalvia
Early Pleistocene	2.588	0.781	Miller	1990	17561	Mollusca	7805	Bivalvia
Pliocene	5.333	2.588	Gibson	1987	17902	Mollusca	7805	Bivalvia
Zanclean	5.333	3.6	Gibson	1987	17902	Mollusca	7805	Bivalvia
Zanclean	5.333	3.6	Gibson	1987	17902	Mollusca	7805	Bivalvia
Zanclean	5.333	3.6	Gibson	1987	17902	Mollusca	7805	Bivalvia
Zanclean	5.333	3.6	Gibson	1987	17902	Mollusca	7805	Bivalvia
Zanclean	5.333	3.6	Gibson	1987	17902	Mollusca	7805	Bivalvia
Zanclean	5.333	3.6	Gibson	1987	17902	Mollusca	7805	Bivalvia
Late Pleistocene	0.126	0.0117	Richards	1962	18974	Mollusca	7805	Bivalvia
Late Pleistocene	0.126	0.0117	Richards	1962	18974	Mollusca	7805	Bivalvia
Late Pleistocene	0.126	0.0117	Richards	1962	18974	Mollusca	7805	Bivalvia
Late Pleistocene	0.126	0.0117	Richards	1962	18974	Mollusca	7805	Bivalvia
Late Pleistocene	0.126	0.0117	Hendy et a	2008	19909	Mollusca	7805	Bivalvia
Gelasian	2.588	1.806	Bailey and	1986	23840	Mollusca	7805	Bivalvia
Early Pleistocene	2.588	0.781	Hendy et a	2008	19909	Mollusca	7805	Bivalvia
Early Pleistocene	2.588	0.781	Hendy et a	2008	19909	Mollusca	7805	Bivalvia
Late Pleistocene	0.126	0.0117	Hendy et a	2008	19909	Mollusca	7805	Bivalvia
Late Pliocene	3.6	2.588	Hendy et a	2008	19909	Mollusca	7805	Bivalvia
Late Pliocene	3.6	2.588	Hendy et a	2008	19909	Mollusca	7805	Bivalvia
Pliocene	5.333	2.588	Hendy et a	2008	19909	Mollusca	7805	Bivalvia
Early Pleistocene	2.588	0.781	Hendy et a	2008	19909	Mollusca	7805	Bivalvia
Late Pliocene	3.6	2.588	Hendy et a	2008	19909	Mollusca	7805	Bivalvia
Early Pleistocene	2.588	0.781	Hendy et a	2008	19909	Mollusca	7805	Bivalvia
Early Pleistocene	2.588	0.781	Hendy et a	2008	19909	Mollusca	7805	Bivalvia
Early Pleistocene	2.588	0.781	Hendy et a	2008	19909	Mollusca	7805	Bivalvia
Early Pleistocene	2.588	0.781	Hendy et a	2008	19909	Mollusca	7805	Bivalvia
Early Pleistocene	2.588	0.781	Hendy et a	2008	19909	Mollusca	7805	Bivalvia
Early Pleistocene	2.588	0.781	Hendy et a	2008	19909	Mollusca	7805	Bivalvia
Pliocene	5.333	2.588	Ward	2007	48009	Mollusca	7805	Bivalvia
Late Pleistocene	0.126	0.0117	Spencer ar	1987	15525	Mollusca	7805	Bivalvia
Late Pleistocene	0.126	0.0117	Spencer ar	1987	15525	Mollusca	7805	Bivalvia
Early Pliocene	5.333	3.6	Ward	1992	16985	Mollusca	7805	Bivalvia
Early Pliocene	5.333	3.6	Ward	1992	16985	Mollusca	7805	Bivalvia
Late Pliocene	3.6	2.588	Ward	1992	16985	Mollusca	7805	Bivalvia
Pliocene	5.333	2.588	Gardner	1943	16984	Mollusca	7805	Bivalvia
Pliocene	5.333	2.588	Gardner	1943	16984	Mollusca	7805	Bivalvia
Pliocene	5.333	2.588	Gardner	1943	16984	Mollusca	7805	Bivalvia
Pliocene	5.333	2.588	Gardner	1943	16984	Mollusca	7805	Bivalvia
Pliocene	5.333	2.588	Gardner	1943	16984	Mollusca	7805	Bivalvia

Late Pliocene		3.6	2.588	Ward	1985	17420	Mollusca	7805	Bivalvia
Late Pliocene		3.6	2.588	Ward	1985	17420	Mollusca	7805	Bivalvia
Late Pliocene		3.6	2.588	Edwards et	2005	17631	Mollusca	7805	Bivalvia
Zanclean	Piacenzian	5.333	2.588	Gibson	1987	17902	Mollusca	7805	Bivalvia
Pliocene		5.333	2.588	Gibson	1987	17902	Mollusca	7805	Bivalvia
Zanclean		5.333	3.6	Ward	1992	16985	Mollusca	7805	Bivalvia
Zanclean		5.333	3.6	Ward	1992	16985	Mollusca	7805	Bivalvia
Late Pliocene		3.6	2.588	Ward	1992	16985	Mollusca	7805	Bivalvia
Zanclean		5.333	3.6	Ward	1992	16985	Mollusca	7805	Bivalvia
Zanclean		5.333	3.6	Ward	1992	16985	Mollusca	7805	Bivalvia
Late Pliocene		3.6	2.588	Ward	1993	26351	Mollusca	7805	Bivalvia
Late Pliocene		3.6	2.588	Ward	1993	26351	Mollusca	7805	Bivalvia
Late Pliocene		3.6	2.588	Ward	1993	26351	Mollusca	7805	Bivalvia
Late Pliocene		3.6	2.588	Ward	1993	26351	Mollusca	7805	Bivalvia
Late Pliocene		3.6	2.588	Ward	1993	26351	Mollusca	7805	Bivalvia
Late Pliocene		3.6	2.588	Ward	1993	26351	Mollusca	7805	Bivalvia
Late Pliocene		3.6	2.588	Ward	1993	26351	Mollusca	7805	Bivalvia
Late Pliocene		3.6	2.588	Ward	1993	26351	Mollusca	7805	Bivalvia
Late Pliocene		3.6	2.588	Ward	1993	26351	Mollusca	7805	Bivalvia
Late Pliocene		3.6	2.588	Hendy et a	2008	19909	Mollusca	7805	Bivalvia
Late Pliocene		3.6	2.588	Hendy et a	2008	19909	Mollusca	7805	Bivalvia
Late Pliocene		3.6	2.588	Hendy et a	2008	19909	Mollusca	7805	Bivalvia
Late Pliocene		3.6	2.588	Hendy et a	2008	19909	Mollusca	7805	Bivalvia
Late Pliocene		3.6	2.588	Hendy et a	2008	19909	Mollusca	7805	Bivalvia
Late Pliocene		3.6	2.588	Hendy et a	2008	19909	Mollusca	7805	Bivalvia
Late Pliocene		3.6	2.588	Hendy et a	2008	19909	Mollusca	7805	Bivalvia
Late Pliocene		3.6	2.588	Hendy et a	2008	19909	Mollusca	7805	Bivalvia
Late Pliocene		3.6	2.588	Hendy et a	2008	19909	Mollusca	7805	Bivalvia
Late Pliocene		3.6	2.588	Hendy et a	2008	19909	Mollusca	7805	Bivalvia
Late Pliocene		3.6	2.588	Hendy et a	2008	19909	Mollusca	7805	Bivalvia
Late Pliocene		3.6	2.588	Hendy et a	2008	19909	Mollusca	7805	Bivalvia
Late Pliocene		3.6	2.588	Hendy et a	2008	19909	Mollusca	7805	Bivalvia
Late Pliocene		3.6	2.588	Hendy et a	2008	19909	Mollusca	7805	Bivalvia
Late Pliocene		3.6	2.588	Hendy et a	2008	19909	Mollusca	7805	Bivalvia
Late Pliocene		3.6	2.588	Hendy et a	2008	19909	Mollusca	7805	Bivalvia
Late Pliocene		3.6	2.588	Hendy et a	2008	19909	Mollusca	7805	Bivalvia
Late Pliocene		3.6	2.588	Hendy et a	2008	19909	Mollusca	7805	Bivalvia
Late Pliocene		3.6	2.588	Hendy et a	2008	19909	Mollusca	7805	Bivalvia
Late Pliocene		3.6	2.588	Hendy et a	2008	19909	Mollusca	7805	Bivalvia
Late Pleistocene		0.126	0.0117	DuBar	1971	17556	Mollusca	7805	Bivalvia
Late Pliocene		3.6	2.588	Ward and I	1987	16700	Mollusca	7805	Bivalvia
Late Pleistocene		0.126	0.0117	Spencer ar	1987	15525	Mollusca	7805	Bivalvia
Middle Pleistocene		0.781	0.126	Hoerle	1970	19536	Mollusca	7805	Bivalvia
Early Pleistocene		2.588	0.781	Olsson anc	1953	25463	Mollusca	7805	Bivalvia
Late Pliocene		3.6	2.588	Petuch anc	2007	26813	Mollusca	7805	Bivalvia
Late Pliocene		3.6	2.588	Vokes	1976	31307	Mollusca	7805	Bivalvia
Pliocene		5.333	2.588	Petuch	1986	47440	Mollusca	7805	Bivalvia

Late Pleistocene	0.126	0.0117	Richards	1962	18974	Mollusca	7805	Bivalvia
Late Pleistocene	0.126	0.0117	DuBar	1971	17556	Mollusca	7805	Bivalvia
Late Pleistocene	0.126	0.0117	Richards	1962	18974	Mollusca	7805	Bivalvia
Late Pleistocene	0.126	0.0117	Richards	1962	18974	Mollusca	7805	Bivalvia
Late Pleistocene	0.126	0.0117	Richards	1962	18974	Mollusca	7805	Bivalvia
Late Pleistocene	0.126	0.0117	Richards	1962	18974	Mollusca	7805	Bivalvia
Late Pleistocene	0.126	0.0117	Richards	1962	18974	Mollusca	7805	Bivalvia
Early Pleistocene	2.588	0.781	Ward and I	1987	16700	Mollusca	7805	Bivalvia
Pleistocene	2.588	0.0117	Gardner	1943	16984	Mollusca	7805	Bivalvia
Early Pleistocene	2.588	0.781	Gardner	1943	16984	Mollusca	7805	Bivalvia
Pleistocene	2.588	0.0117	Gardner	1943	16984	Mollusca	7805	Bivalvia
Early Pleistocene	2.588	0.781	Gardner	1943	16984	Mollusca	7805	Bivalvia
Late Pliocene	3.6	2.588	Gardner	1943	16984	Mollusca	7805	Bivalvia
Late Pliocene	3.6	2.588	Gardner	1943	16984	Mollusca	7805	Bivalvia
Late Pleistocene	0.126	0.0117	Richards	1962	18974	Mollusca	7805	Bivalvia
Late Pleistocene	0.126	0.0117	Spencer ar	1987	15525	Mollusca	7805	Bivalvia
Late Pleistocene	0.126	0.0117	Spencer ar	1987	15525	Mollusca	7805	Bivalvia
Late Pleistocene	0.126	0.0117	Spencer ar	1987	15525	Mollusca	7805	Bivalvia
Late Pliocene	3.6	2.588	Ward	1993	26351	Mollusca	7805	Bivalvia
Late Pliocene	3.6	2.588	Ward	1993	26351	Mollusca	7805	Bivalvia
Late Pliocene	3.6	2.588	Ward	1993	26351	Mollusca	7805	Bivalvia
Late Pliocene	3.6	2.588	Ward	1993	26351	Mollusca	7805	Bivalvia
Late Pliocene	3.6	2.588	Ward	1993	26351	Mollusca	7805	Bivalvia
Late Pliocene	3.6	2.588	Ward	1993	26351	Mollusca	7805	Bivalvia
Early Pleistocene	2.588	0.781	Portell et a	1992	17634	Mollusca	7805	Bivalvia
Middle Pleistocene	0.781	0.126	Hoerle	1970	19536	Mollusca	7805	Bivalvia
Late Pleistocene	0.126	0.0117	Hendy et a	2008	19909	Mollusca	7805	Bivalvia
Late Pliocene	3.6	2.588	Hendy et a	2008	19909	Mollusca	7805	Bivalvia
Late Pleistocene	0.126	0.0117	DuBar	1971	17556	Mollusca	7805	Bivalvia
Zanclean	5.333	3.6	Campbell a	1995	17558	Mollusca	7805	Bivalvia
Early Pliocene	5.333	3.6	Campbell a	1995	17558	Mollusca	7805	Bivalvia
Early Pleistocene	2.588	0.781	DuBar	1971	17556	Mollusca	7805	Bivalvia
Late Pleistocene	0.126	0.0117	Richards	1962	18974	Mollusca	7805	Bivalvia
Late Pleistocene	0.126	0.0117	Richards	1962	18974	Mollusca	7805	Bivalvia
Late Pleistocene	0.126	0.0117	Richards	1962	18974	Mollusca	7805	Bivalvia
Late Pleistocene	0.126	0.0117	Richards	1962	18974	Mollusca	7805	Bivalvia
Late Pleistocene	0.126	0.0117	Hendy et a	2008	19909	Mollusca	7805	Bivalvia
Late Pleistocene	0.126	0.0117	Hendy et a	2008	19909	Mollusca	7805	Bivalvia
Late Pleistocene	0.126	0.0117	Hendy et a	2008	19909	Mollusca	7805	Bivalvia
Late Pleistocene	0.126	0.0117	Hendy et a	2008	19909	Mollusca	7805	Bivalvia
Late Pleistocene	0.126	0.0117	Hendy et a	2008	19909	Mollusca	7805	Bivalvia
Late Pleistocene	0.126	0.0117	Hendy et a	2008	19909	Mollusca	7805	Bivalvia
Pleistocene	2.588	0.0117	Hendy et a	2008	19909	Mollusca	7805	Bivalvia
Late Pliocene	3.6	2.588	Ward and I	1987	16700	Mollusca	7805	Bivalvia
Late Pliocene	3.6	2.588	Ward and I	1987	16700	Mollusca	7805	Bivalvia
Late Pliocene	3.6	2.588	Ward and I	1987	16700	Mollusca	7805	Bivalvia
Early Pleistocene	2.588	0.781	Ward and I	1987	16700	Mollusca	7805	Bivalvia

Early Pleistocene	2.588	0.781	Miller	1990	17561	Mollusca	7805	Bivalvia
Late Pleistocene	0.126	0.0117	Richards	1962	18974	Mollusca	7805	Bivalvia
Late Pleistocene	0.126	0.0117	Richards	1962	18974	Mollusca	7805	Bivalvia
Late Pleistocene	0.126	0.0117	Richards	1962	18974	Mollusca	7805	Bivalvia
Late Pleistocene	0.126	0.0117	Richards	1962	18974	Mollusca	7805	Bivalvia
Late Pleistocene	0.126	0.0117	Richards	1962	18974	Mollusca	7805	Bivalvia
Late Pleistocene	0.126	0.0117	Richards	1962	18974	Mollusca	7805	Bivalvia
Late Pleistocene	0.126	0.0117	Richards	1962	18974	Mollusca	7805	Bivalvia
Late Pleistocene	0.126	0.0117	Richards	1962	18974	Mollusca	7805	Bivalvia
Late Pleistocene	0.126	0.0117	Richards	1962	18974	Mollusca	7805	Bivalvia
Gelasian	2.588	1.806	Bailey and	1986	23840	Mollusca	7805	Bivalvia
Early Pleistocene	2.588	0.781	Hendy et a	2008	19909	Mollusca	7805	Bivalvia
Late Pleistocene	0.126	0.0117	Hendy et a	2008	19909	Mollusca	7805	Bivalvia
Late Pliocene	3.6	2.588	Hendy et a	2008	19909	Mollusca	7805	Bivalvia
Late Pliocene	3.6	2.588	Hendy et a	2008	19909	Mollusca	7805	Bivalvia
Pliocene	5.333	2.588	Hendy et a	2008	19909	Mollusca	7805	Bivalvia
Pliocene	5.333	2.588	Hendy et a	2008	19909	Mollusca	7805	Bivalvia
Late Pliocene	3.6	2.588	Hendy et a	2008	19909	Mollusca	7805	Bivalvia
Late Pliocene	3.6	2.588	Hendy et a	2008	19909	Mollusca	7805	Bivalvia
Late Pliocene	3.6	2.588	Hendy et a	2008	19909	Mollusca	7805	Bivalvia
Early Pleistocene	2.588	0.781	Hendy et a	2008	19909	Mollusca	7805	Bivalvia
Early Pleistocene	2.588	0.781	Hendy et a	2008	19909	Mollusca	7805	Bivalvia
Early Pleistocene	2.588	0.781	Hendy et a	2008	19909	Mollusca	7805	Bivalvia
Early Pleistocene	2.588	0.781	Hendy et a	2008	19909	Mollusca	7805	Bivalvia
Middle Pleistocene	0.781	0.126	Miller	1985	30803	Mollusca	7805	Bivalvia
Middle Pleistocene	0.781	0.126	Miller	1985	30803	Mollusca	7805	Bivalvia
Pleistocene	2.588	0.0117	Coch	1971	441	Mollusca	7805	Bivalvia
Late Pleistocene	0.126	0.0117	Spencer ar	1987	15525	Mollusca	7805	Bivalvia
Late Pleistocene	0.126	0.0117	Spencer ar	1987	15525	Mollusca	7805	Bivalvia
Late Pleistocene	0.126	0.0117	Spencer ar	1987	15525	Mollusca	7805	Bivalvia
Late Pleistocene	0.126	0.0117	Hendy et a	2008	19909	Mollusca	7805	Bivalvia
Late Pliocene	3.6	2.588	Ward	1993	26351	Mollusca	7805	Bivalvia
Late Pliocene	3.6	2.588	Ward	1993	26351	Mollusca	7805	Bivalvia
Late Pliocene	3.6	2.588	Ward	1993	26351	Mollusca	7805	Bivalvia
Late Pleistocene	0.126	0.0117	Hendy et a	2008	19909	Mollusca	7805	Bivalvia
Middle Pleistocene	0.781	0.126	Hoerle	1970	19536	Mollusca	7805	Bivalvia
Early Pleistocene	2.588	0.781	Olsson anc	1953	25463	Mollusca	7805	Bivalvia
Late Plioce Early Pleist	3.6	0.781	Petuch anc	2007	26813	Mollusca	7805	Bivalvia
Early Pleistocene	2.588	0.781	Hendy et a	2008	19909	Mollusca	7805	Bivalvia
Late Pleistocene	0.126	0.0117	Kittle and I	2010	40884	Mollusca	7805	Bivalvia
Late Pleistocene	0.126	0.0117	Kittle and I	2010	40884	Mollusca	7805	Bivalvia
Zanclean	5.333	3.6	Campbell a	1995	17558	Mollusca	7805	Bivalvia
Late Pliocene	3.6	2.588	Campbell a	1995	17558	Mollusca	7805	Bivalvia
Late Pleistocene	0.126	0.0117	Richards	1962	18974	Mollusca	7805	Bivalvia
Late Pleistocene	0.126	0.0117	Richards	1962	18974	Mollusca	7805	Bivalvia
Late Pleistocene	0.126	0.0117	Richards	1962	18974	Mollusca	7805	Bivalvia
Late Pleistocene	0.126	0.0117	Richards	1962	18974	Mollusca	7805	Bivalvia

Late Pleistocene		0.126	0.0117	Richards	1962	18974	Mollusca	7805	Bivalvia
Late Pleistocene		0.126	0.0117	Richards	1962	18974	Mollusca	7805	Bivalvia
Late Pleistocene		0.126	0.0117	Richards	1962	18974	Mollusca	7805	Bivalvia
Late Pleistocene		0.126	0.0117	Hendy et a	2008	19909	Mollusca	7805	Bivalvia
Early Pleistocene		2.588	0.781	Cooke	1936	31145	Mollusca	7805	Bivalvia
Late Pliocene		3.6	2.588	Ward and I	1987	16700	Mollusca	7805	Bivalvia
Early Pleistocene		2.588	0.781	Gardner	1943	16984	Mollusca	7805	Bivalvia
Early Pleistocene		2.588	0.781	Gardner	1943	16984	Mollusca	7805	Bivalvia
Early Plioc	Late Plioce	5.333	2.588	Gardner	1943	16984	Mollusca	7805	Bivalvia
Late Pleistocene		0.126	0.0117	Richards	1962	18974	Mollusca	7805	Bivalvia
Late Pleistocene		0.126	0.0117	Richards	1962	18974	Mollusca	7805	Bivalvia
Late Pleistocene		0.126	0.0117	Richards	1962	18974	Mollusca	7805	Bivalvia
Middle Pleistocene		0.781	0.126	Miller	1985	30803	Mollusca	7805	Bivalvia
Pleistocene		2.588	0.0117	Coch	1971	441	Mollusca	7805	Bivalvia
Late Pleistocene		0.126	0.0117	Spencer ar	1987	15525	Mollusca	7805	Bivalvia
Late Pleistocene		0.126	0.0117	Spencer ar	1987	15525	Mollusca	7805	Bivalvia
Late Pleistocene		0.126	0.0117	Spencer ar	1987	15525	Mollusca	7805	Bivalvia
Late Pleistocene		0.126	0.0117	Spencer ar	1987	15525	Mollusca	7805	Bivalvia
Late Pliocene		3.6	2.588	Ward	1993	26351	Mollusca	7805	Bivalvia
Late Plioce	Early Pleist	3.6	0.781	Petuch anc	2007	26813	Mollusca	7805	Bivalvia
Early Pleistocene		2.588	0.781	Mansfield	1932	11083	Mollusca	7805	Bivalvia
Early Pleistocene		2.588	0.781	Mansfield	1932	11083	Mollusca	7805	Bivalvia
Early Pleistocene		2.588	0.781	DuBar	1962	11119	Mollusca	7805	Bivalvia
Early Pleistocene		2.588	0.781	DuBar	1962	11119	Mollusca	7805	Bivalvia
Late Pliocene		3.6	2.588	Mansfield	1932	17094	Mollusca	7805	Bivalvia
Early Pleistocene		2.588	0.781	Portell et a	1992	17634	Mollusca	7805	Bivalvia
Middle Pleistocene		0.781	0.126	Portell et a	1992	17634	Mollusca	7805	Bivalvia
Middle Pleistocene		0.781	0.126	McGinty	1970	19114	Mollusca	7805	Bivalvia
Early Pleistocene		2.588	0.781	Schroeder	1954	25303	Mollusca	7805	Bivalvia
Early Pleistocene		2.588	0.781	Olsson anc	1953	25463	Mollusca	7805	Bivalvia
Early Pleistocene		2.588	0.781	Hendy et a	2008	19909	Mollusca	7805	Bivalvia
Late Pleistocene		0.126	0.0117	Richards	1962	18974	Mollusca	7805	Bivalvia
Late Pleistocene		0.126	0.0117	Richards	1962	18974	Mollusca	7805	Bivalvia
Late Pleistocene		0.126	0.0117	Richards	1962	18974	Mollusca	7805	Bivalvia
Late Pleistocene		0.126	0.0117	Cooke	1936	31145	Mollusca	7805	Bivalvia
Pleistocene		2.588	0.0117	Holmes	1860	64712	Mollusca	7805	Bivalvia
Early Pleistocene		2.588	0.781	Hendy et a	2008	19909	Mollusca	7805	Bivalvia
Early Pleistocene		2.588	0.781	Hendy et a	2008	19909	Mollusca	7805	Bivalvia
Late Pleistocene		0.126	0.0117	Sellards	1917	9011	Mollusca	7805	Bivalvia
Early Pleistocene		2.588	0.781	Mansfield	1932	11083	Mollusca	7805	Bivalvia
Early Pleistocene		2.588	0.781	Mansfield	1932	11083	Mollusca	7805	Bivalvia
Early Pleistocene		2.588	0.781	Mansfield	1932	11083	Mollusca	7805	Bivalvia
Early Pleistocene		2.588	0.781	DuBar	1962	11119	Mollusca	7805	Bivalvia
Calabrian		1.806	0.781	DuBar	1962	11119	Mollusca	7805	Bivalvia
Calabrian		1.806	0.781	DuBar	1962	11119	Mollusca	7805	Bivalvia
Late Pleistocene		0.126	0.0117	DuBar	1962	11119	Mollusca	7805	Bivalvia
Early Pleistocene		2.588	0.781	DuBar	1962	11119	Mollusca	7805	Bivalvia

Late Pleistocene		0.126	0.0117	DuBar	1962	11119	Mollusca	7805	Bivalvia
Late Pliocene		3.6	2.588	Mansfield	1932	17094	Mollusca	7805	Bivalvia
Late Pliocene		3.6	2.588	Mansfield	1932	17094	Mollusca	7805	Bivalvia
Late Pliocene		3.6	2.588	Mansfield	1932	17094	Mollusca	7805	Bivalvia
Late Pliocene		3.6	2.588	Mansfield	1932	17094	Mollusca	7805	Bivalvia
Early Pleistocene		2.588	0.781	Portell et a	1992	17634	Mollusca	7805	Bivalvia
Middle Pleistocene		0.781	0.126	Portell et a	1992	17634	Mollusca	7805	Bivalvia
Late Pleistocene		0.126	0.0117	Portell et a	1992	17634	Mollusca	7805	Bivalvia
Middle Pleistocene		0.781	0.126	McGinty	1970	19114	Mollusca	7805	Bivalvia
Late Pleistocene		0.126	0.0117	Hendy et a	2008	19909	Mollusca	7805	Bivalvia
Early Pleistocene		2.588	0.781	Olsson anc	1953	25463	Mollusca	7805	Bivalvia
Late Pliocene		3.6	2.588	Hendy et a	2008	19909	Mollusca	7805	Bivalvia
Early Pleistocene		2.588	0.781	Hendy et a	2008	19909	Mollusca	7805	Bivalvia
Early Pleistocene		2.588	0.781	Hendy et a	2008	19909	Mollusca	7805	Bivalvia
Late Pliocene		3.6	2.588	Hendy et a	2008	19909	Mollusca	7805	Bivalvia
Late Pliocene		3.6	2.588	Hendy et a	2008	19909	Mollusca	7805	Bivalvia
Late Pliocene		3.6	2.588	Hendy et a	2008	19909	Mollusca	7805	Bivalvia
Middle Pleistocene		0.781	0.126	Hendy et a	2008	19909	Mollusca	7805	Bivalvia
Early Pleistocene		2.588	0.781	Hendy et a	2008	19909	Mollusca	7805	Bivalvia
Late Pliocene		3.6	2.588	Weisbord	1971	39743	Mollusca	7805	Bivalvia
Late Pleistocene		0.126	0.0117	Kittle and I	2010	40884	Mollusca	7805	Bivalvia
Pliocene		5.333	2.588	DuBar	1971	17556	Mollusca	7805	Bivalvia
Pliocene		5.333	2.588	DuBar	1971	17556	Mollusca	7805	Bivalvia
Late Pleistocene		0.126	0.0117	DuBar	1971	17556	Mollusca	7805	Bivalvia
Pliocene		5.333	2.588	DuBar	1971	17556	Mollusca	7805	Bivalvia
Zanclean		5.333	3.6	Campbell a	1995	17558	Mollusca	7805	Bivalvia
Late Pleistocene		0.126	0.0117	Richards	1962	18974	Mollusca	7805	Bivalvia
Late Pleistocene		0.126	0.0117	Hendy et a	2008	19909	Mollusca	7805	Bivalvia
Pleistocene		2.588	0.0117	Hendy et a	2008	19909	Mollusca	7805	Bivalvia
Pleistocene		2.588	0.0117	Hendy et a	2008	19909	Mollusca	7805	Bivalvia
Early Pleistocene		2.588	0.781	Cooke	1936	31145	Mollusca	7805	Bivalvia
Early Pleistocene		2.588	0.781	Cooke	1936	31145	Mollusca	7805	Bivalvia
Late Pliocene		3.6	2.588	Cooke	1936	31145	Mollusca	7805	Bivalvia
Late Pliocene		3.6	2.588	Ward and I	1987	16700	Mollusca	7805	Bivalvia
Late Pliocene		3.6	2.588	Ward and I	1987	16700	Mollusca	7805	Bivalvia
Late Pliocene		3.6	2.588	Ward and I	1987	16700	Mollusca	7805	Bivalvia
Early Plioc	Late Plioce	5.333	2.588	Gardner	1943	16984	Mollusca	7805	Bivalvia
Early Plioc	Late Plioce	5.333	2.588	Gardner	1943	16984	Mollusca	7805	Bivalvia
Early Plioc	Late Plioce	5.333	2.588	Gardner	1943	16984	Mollusca	7805	Bivalvia
Late Pliocene		3.6	2.588	Gardner	1943	16984	Mollusca	7805	Bivalvia
Late Pliocene		3.6	2.588	Gardner	1943	16984	Mollusca	7805	Bivalvia
Late Pliocene		3.6	2.588	Gardner	1943	16984	Mollusca	7805	Bivalvia
Late Pliocene		3.6	2.588	Gardner	1943	16984	Mollusca	7805	Bivalvia
Late Pliocene		3.6	2.588	Gardner	1943	16984	Mollusca	7805	Bivalvia
Early Pleistocene		2.588	0.781	Gardner	1943	16984	Mollusca	7805	Bivalvia
Early Pleistocene		2.588	0.781	Gardner	1943	16984	Mollusca	7805	Bivalvia
Early Pleistocene		2.588	0.781	Gardner	1943	16984	Mollusca	7805	Bivalvia

Early Pleistocene	2.588	0.781	DuBar	1971	17556	Mollusca	7805	Bivalvia
Late Pliocene	3.6	2.588	Hendy et a	2008	19909	Mollusca	7805	Bivalvia
Gelasian	2.588	1.806	Bailey and	1986	23840	Mollusca	7805	Bivalvia
Late Pliocene	3.6	2.588	Hendy et a	2008	19909	Mollusca	7805	Bivalvia
Pliocene	5.333	2.588	Hendy et a	2008	19909	Mollusca	7805	Bivalvia
Pliocene	5.333	2.588	Hendy et a	2008	19909	Mollusca	7805	Bivalvia
Late Pliocene	3.6	2.588	Hendy et a	2008	19909	Mollusca	7805	Bivalvia
Late Pliocene	3.6	2.588	Hendy et a	2008	19909	Mollusca	7805	Bivalvia
Early Pleistocene	2.588	0.781	Hendy et a	2008	19909	Mollusca	7805	Bivalvia
Early Pleistocene	2.588	0.781	Hendy et a	2008	19909	Mollusca	7805	Bivalvia
Late Pliocene	3.6	2.588	Hendy et a	2008	19909	Mollusca	7805	Bivalvia
Early Pleistocene	2.588	0.781	Hendy et a	2008	19909	Mollusca	7805	Bivalvia
Early Pleistocene	2.588	0.781	Hendy et a	2008	19909	Mollusca	7805	Bivalvia
Early Pleistocene	2.588	0.781	Hendy et a	2008	19909	Mollusca	7805	Bivalvia
Early Pleistocene	2.588	0.781	Hendy et a	2008	19909	Mollusca	7805	Bivalvia
Pliocene	5.333	2.588	Ward	2007	48009	Mollusca	7805	Bivalvia
Late Pliocene	3.6	2.588	Ward	1993	26351	Mollusca	7805	Bivalvia
Late Pliocene	3.6	2.588	Ward	1993	26351	Mollusca	7805	Bivalvia
Late Pliocene	3.6	2.588	Ward	1993	26351	Mollusca	7805	Bivalvia
Late Pliocene	3.6	2.588	Ward	1993	26351	Mollusca	7805	Bivalvia
Late Pliocene	3.6	2.588	Ward	1993	26351	Mollusca	7805	Bivalvia
Late Pliocene	3.6	2.588	Ward	1993	26351	Mollusca	7805	Bivalvia
Late Pliocene	3.6	2.588	Hendy et a	2008	19909	Mollusca	7805	Bivalvia
Late Pliocene	3.6	2.588	Hendy et a	2008	19909	Mollusca	7805	Bivalvia
Early Pleistocene	2.588	0.781	Olsson anc	1953	25463	Mollusca	7805	Bivalvia
Early Pleistocene	2.588	0.781	Hendy et a	2008	19909	Mollusca	7805	Bivalvia
Late Pliocene	3.6	2.588	Mansfield	1932	17094	Mollusca	7805	Bivalvia
Early Pleistocene	2.588	0.781	Hendy et a	2008	19909	Mollusca	7805	Bivalvia
Late Pliocene	3.6	2.588	Hendy et a	2008	19909	Mollusca	7805	Bivalvia
Late Pliocene	3.6	2.588	Hendy et a	2008	19909	Mollusca	7805	Bivalvia
Late Pliocene	3.6	2.588	Hendy et a	2008	19909	Mollusca	7805	Bivalvia
Early Pleistocene	2.588	0.781	DuBar	1962	11119	Mollusca	7805	Bivalvia
Late Pliocene	3.6	2.588	Mansfield	1932	17094	Mollusca	7805	Bivalvia
Late Pleistocene	0.126	0.0117	Portell et a	1992	17634	Mollusca	7805	Bivalvia
Middle Pleistocene	0.781	0.126	McGinty	1970	19114	Mollusca	7805	Bivalvia
Middle Pleistocene	0.781	0.126	McGinty	1970	19114	Mollusca	7805	Bivalvia
Late Pleistocene	0.126	0.0117	Hendy et a	2008	19909	Mollusca	7805	Bivalvia
Early Pleistocene	2.588	0.781	Olsson anc	1953	25463	Mollusca	7805	Bivalvia
Early Pleistocene	2.588	0.781	Olsson anc	1953	25463	Mollusca	7805	Bivalvia
Early Pleistocene	2.588	0.781	Hendy et a	2008	19909	Mollusca	7805	Bivalvia
Middle Pleistocene	0.781	0.126	Hendy et a	2008	19909	Mollusca	7805	Bivalvia
Late Pleistocene	0.126	0.0117	Kittle and I	2010	40884	Mollusca	7805	Bivalvia
Zanclean	5.333	3.6	Campbell a	1995	17558	Mollusca	7805	Bivalvia
Late Pleistocene	0.126	0.0117	Richards	1962	18974	Mollusca	7805	Bivalvia
Late Pleistocene	0.126	0.0117	Richards	1962	18974	Mollusca	7805	Bivalvia
Early Pleistocene	2.588	0.781	Hendy et a	2008	19909	Mollusca	7805	Bivalvia
Late Pliocene	3.6	2.588	Ward	1993	26351	Mollusca	7805	Bivalvia

Late Pleistocene		0.126	0.0117	Sellards	1917	9011	Mollusca	7805	Bivalvia
Late Pleistocene		0.126	0.0117	Sellards	1917	9011	Mollusca	7805	Bivalvia
Early Pleistocene		2.588	0.781	DuBar	1962	11119	Mollusca	7805	Bivalvia
Early Pleistocene		2.588	0.781	DuBar	1962	11119	Mollusca	7805	Bivalvia
Early Pleistocene		2.588	0.781	DuBar	1962	11119	Mollusca	7805	Bivalvia
Early Pleistocene		2.588	0.781	DuBar	1962	11119	Mollusca	7805	Bivalvia
Calabrian		1.806	0.781	DuBar	1962	11119	Mollusca	7805	Bivalvia
Calabrian		1.806	0.781	DuBar	1962	11119	Mollusca	7805	Bivalvia
Calabrian		1.806	0.781	DuBar	1962	11119	Mollusca	7805	Bivalvia
Calabrian		1.806	0.781	DuBar	1962	11119	Mollusca	7805	Bivalvia
Late Pleistocene		0.126	0.0117	DuBar	1962	11119	Mollusca	7805	Bivalvia
Late Pleistocene		0.126	0.0117	DuBar	1962	11119	Mollusca	7805	Bivalvia
Pliocene	Pleistocen	5.333	0.0117	DuBar	1962	11119	Mollusca	7805	Bivalvia
Late Pliocene		3.6	2.588	Mansfield	1932	17094	Mollusca	7805	Bivalvia
Late Pliocene		3.6	2.588	Mansfield	1932	17094	Mollusca	7805	Bivalvia
Late Pliocene		3.6	2.588	Mansfield	1932	17094	Mollusca	7805	Bivalvia
Late Pliocene		3.6	2.588	Mansfield	1932	17094	Mollusca	7805	Bivalvia
Late Pliocene		3.6	2.588	Mansfield	1932	17094	Mollusca	7805	Bivalvia
Late Pliocene		3.6	2.588	Mansfield	1932	17094	Mollusca	7805	Bivalvia
Late Pliocene		3.6	2.588	Mansfield	1932	17094	Mollusca	7805	Bivalvia
Late Pliocene		3.6	2.588	Mansfield	1932	17094	Mollusca	7805	Bivalvia
Late Pliocene		3.6	2.588	Mansfield	1932	17094	Mollusca	7805	Bivalvia
Late Pliocene		3.6	2.588	Mansfield	1932	17094	Mollusca	7805	Bivalvia
Late Pliocene		3.6	2.588	Mansfield	1932	17094	Mollusca	7805	Bivalvia
Early Pleistocene		2.588	0.781	Portell et a	1992	17634	Mollusca	7805	Bivalvia
Early Pleistocene		2.588	0.781	Portell et a	1992	17634	Mollusca	7805	Bivalvia
Early Pleistocene		2.588	0.781	Portell et a	1992	17634	Mollusca	7805	Bivalvia
Early Pleistocene		2.588	0.781	Portell et a	1992	17634	Mollusca	7805	Bivalvia
Middle Pleistocene		0.781	0.126	Portell et a	1992	17634	Mollusca	7805	Bivalvia
Middle Pleistocene		0.781	0.126	Portell et a	1992	17634	Mollusca	7805	Bivalvia
Middle Pleistocene		0.781	0.126	McGinty	1970	19114	Mollusca	7805	Bivalvia
Middle Pleistocene		0.781	0.126	Hoerle	1970	19536	Mollusca	7805	Bivalvia
Middle Pleistocene		0.781	0.126	Hoerle	1970	19536	Mollusca	7805	Bivalvia
Middle Pleistocene		0.781	0.126	Hoerle	1970	19536	Mollusca	7805	Bivalvia
Middle Pleistocene		0.781	0.126	Hoerle	1970	19536	Mollusca	7805	Bivalvia
Middle Pleistocene		0.781	0.126	Hoerle	1970	19536	Mollusca	7805	Bivalvia
Middle Pleistocene		0.781	0.126	Hoerle	1970	19536	Mollusca	7805	Bivalvia
Middle Pleistocene		0.781	0.126	Hoerle	1970	19536	Mollusca	7805	Bivalvia
Middle Pleistocene		0.781	0.126	Hoerle	1970	19536	Mollusca	7805	Bivalvia
Early Pleistocene		2.588	0.781	Schroeder	1954	25303	Mollusca	7805	Bivalvia
Early Pleistocene		2.588	0.781	Olsson anc	1953	25463	Mollusca	7805	Bivalvia
Early Pleistocene		2.588	0.781	Olsson anc	1953	25463	Mollusca	7805	Bivalvia
Early Pleistocene		2.588	0.781	Olsson anc	1953	25463	Mollusca	7805	Bivalvia
Early Pleistocene		2.588	0.781	Olsson anc	1953	25463	Mollusca	7805	Bivalvia
Early Pleistocene		2.588	0.781	Olsson anc	1953	25463	Mollusca	7805	Bivalvia
Early Pleistocene		2.588	0.781	Olsson anc	1953	25463	Mollusca	7805	Bivalvia
Early Pleistocene		2.588	0.781	Olsson anc	1953	25463	Mollusca	7805	Bivalvia

Early Pleistocene	2.588	0.781	Olsson anc	1953	25463	Mollusca	7805	Bivalvia
Early Pleistocene	2.588	0.781	Olsson anc	1953	25463	Mollusca	7805	Bivalvia
Early Pleistocene	2.588	0.781	Olsson anc	1953	25463	Mollusca	7805	Bivalvia
Early Pleistocene	2.588	0.781	Olsson anc	1953	25463	Mollusca	7805	Bivalvia
Middle Pleistocene	0.781	0.126	Petuch anc	2007	26813	Mollusca	7805	Bivalvia
Early Pleistocene	2.588	0.781	Hendy et a	2008	19909	Mollusca	7805	Bivalvia
Late Pliocene	3.6	2.588	Hendy et a	2008	19909	Mollusca	7805	Bivalvia
Late Pliocene	3.6	2.588	Hendy et a	2008	19909	Mollusca	7805	Bivalvia
Late Pliocene	3.6	2.588	Hendy et a	2008	19909	Mollusca	7805	Bivalvia
Late Pliocene	3.6	2.588	Hendy et a	2008	19909	Mollusca	7805	Bivalvia
Middle Pleistocene	0.781	0.126	Hendy et a	2008	19909	Mollusca	7805	Bivalvia
Middle Pleistocene	0.781	0.126	Hendy et a	2008	19909	Mollusca	7805	Bivalvia
Late Pleistocene	0.126	0.0117	Hendy et a	2008	19909	Mollusca	7805	Bivalvia
Late Pleistocene	0.126	0.0117	Hendy et a	2008	19909	Mollusca	7805	Bivalvia
Late Pleistocene	0.126	0.0117	Kittle and I	2010	40884	Mollusca	7805	Bivalvia
Late Pleistocene	0.126	0.0117	Kittle and I	2010	40884	Mollusca	7805	Bivalvia
Late Pleistocene	0.126	0.0117	Kittle and I	2010	40884	Mollusca	7805	Bivalvia
Late Pleistocene	0.126	0.0117	Kittle and I	2010	40884	Mollusca	7805	Bivalvia
Late Pleistocene	0.126	0.0117	Kittle and I	2010	40884	Mollusca	7805	Bivalvia
Late Pleistocene	0.126	0.0117	Hulbert an	1998	1962	Mollusca	7805	Bivalvia
Late Pleistocene	0.126	0.0117	Hulbert an	1998	1962	Mollusca	7805	Bivalvia
Late Pleistocene	0.126	0.0117	Rosenberg	2005	19419	Mollusca	7805	Bivalvia
Late Pleistocene	0.126	0.0117	DuBar	1971	17556	Mollusca	7805	Bivalvia
Late Pleistocene	0.126	0.0117	DuBar	1971	17556	Mollusca	7805	Bivalvia
Late Pleistocene	0.126	0.0117	Rosenberg	2005	19419	Mollusca	7805	Bivalvia
Late Pleistocene	0.126	0.0117	DuBar	1971	17556	Mollusca	7805	Bivalvia
Late Pleistocene	0.126	0.0117	Rosenberg	2005	19419	Mollusca	7805	Bivalvia
Late Pleistocene	0.126	0.0117	Rosenberg	2005	19419	Mollusca	7805	Bivalvia
Late Pleistocene	0.126	0.0117	DuBar	1971	17556	Mollusca	7805	Bivalvia
Zanclean	5.333	3.6	Campbell a	1995	17558	Mollusca	7805	Bivalvia
Early Pleistocene	2.588	0.781	Rosenberg	2005	19419	Mollusca	7805	Bivalvia
Late Pleistocene	0.126	0.0117	Richards	1962	18974	Mollusca	7805	Bivalvia
Late Pleistocene	0.126	0.0117	Richards	1962	18974	Mollusca	7805	Bivalvia
Late Pleistocene	0.126	0.0117	Richards	1962	18974	Mollusca	7805	Bivalvia
Late Pleistocene	0.126	0.0117	Richards	1962	18974	Mollusca	7805	Bivalvia
Late Pleistocene	0.126	0.0117	Richards	1962	18974	Mollusca	7805	Bivalvia
Late Pleistocene	0.126	0.0117	Richards	1962	18974	Mollusca	7805	Bivalvia
Late Pleistocene	0.126	0.0117	Richards	1962	18974	Mollusca	7805	Bivalvia
Late Pleistocene	0.126	0.0117	Richards	1962	18974	Mollusca	7805	Bivalvia
Late Pleistocene	0.126	0.0117	Richards	1962	18974	Mollusca	7805	Bivalvia
Late Pleistocene	0.126	0.0117	Richards	1962	18974	Mollusca	7805	Bivalvia
Late Pleistocene	0.126	0.0117	Rosenberg	2005	19419	Mollusca	7805	Bivalvia
Late Pleistocene	0.126	0.0117	Rosenberg	2005	19419	Mollusca	7805	Bivalvia
Late Pleistocene	0.126	0.0117	Rosenberg	2005	19419	Mollusca	7805	Bivalvia
Late Pleistocene	0.126	0.0117	Richards	1962	18974	Mollusca	7805	Bivalvia
Late Pleistocene	0.126	0.0117	Hendy et a	2008	19909	Mollusca	7805	Bivalvia
Late Pleistocene	0.126	0.0117	Hendy et a	2008	19909	Mollusca	7805	Bivalvia

Late Pleistocene		0.126	0.0117	Hendy et a	2008	19909	Mollusca	7805	Bivalvia
Late Pleistocene		0.126	0.0117	Hendy et a	2008	19909	Mollusca	7805	Bivalvia
Late Pleistocene		0.126	0.0117	Hendy et a	2008	19909	Mollusca	7805	Bivalvia
Late Pleistocene		0.126	0.0117	Hendy et a	2008	19909	Mollusca	7805	Bivalvia
Late Pleistocene		0.126	0.0117	Hendy et a	2008	19909	Mollusca	7805	Bivalvia
Late Pleistocene		0.126	0.0117	Hendy et a	2008	19909	Mollusca	7805	Bivalvia
Late Pleistocene		0.126	0.0117	Hendy et a	2008	19909	Mollusca	7805	Bivalvia
Late Pleistocene		0.126	0.0117	Hendy et a	2008	19909	Mollusca	7805	Bivalvia
Late Pleistocene		0.126	0.0117	Hendy et a	2008	19909	Mollusca	7805	Bivalvia
Late Pleistocene		0.126	0.0117	Hendy et a	2008	19909	Mollusca	7805	Bivalvia
Pleistocene		2.588	0.0117	Hendy et a	2008	19909	Mollusca	7805	Bivalvia
Late Pliocene		3.6	2.588	Cooke	1936	31145	Mollusca	7805	Bivalvia
Pleistocene		2.588	0.0117	Holmes	1860	64712	Mollusca	7805	Bivalvia
Pleistocene		2.588	0.0117	Holmes	1860	64712	Mollusca	7805	Bivalvia
Late Pliocene		3.6	2.588	Ward and I	1987	16700	Mollusca	7805	Bivalvia
Late Pliocene		3.6	2.588	Ward and I	1987	16700	Mollusca	7805	Bivalvia
Late Pliocene		3.6	2.588	Ward and I	1987	16700	Mollusca	7805	Bivalvia
Early Pleistocene		2.588	0.781	Ward and I	1987	16700	Mollusca	7805	Bivalvia
Early Pleistocene		2.588	0.781	Ward and I	1987	16700	Mollusca	7805	Bivalvia
Late Pliocene		3.6	2.588	Ward and I	1987	16700	Mollusca	7805	Bivalvia
Late Pliocene		3.6	2.588	Ward and I	1987	16700	Mollusca	7805	Bivalvia
Early Pleistocene		2.588	0.781	Ward and I	1987	16700	Mollusca	7805	Bivalvia
Late Pliocene		3.6	2.588	Gardner	1943	16984	Mollusca	7805	Bivalvia
Early Plioc	Late Plioce	5.333	2.588	Gardner	1943	16984	Mollusca	7805	Bivalvia
Early Pleistocene		2.588	0.781	Gardner	1943	16984	Mollusca	7805	Bivalvia
Early Pleistocene		2.588	0.781	Gardner	1943	16984	Mollusca	7805	Bivalvia
Pleistocene		2.588	0.0117	Gardner	1943	16984	Mollusca	7805	Bivalvia
Early Pleistocene		2.588	0.781	Gardner	1943	16984	Mollusca	7805	Bivalvia
Early Pleistocene		2.588	0.781	Gardner	1943	16984	Mollusca	7805	Bivalvia
Pleistocene		2.588	0.0117	Gardner	1943	16984	Mollusca	7805	Bivalvia
Early Pleistocene		2.588	0.781	Gardner	1943	16984	Mollusca	7805	Bivalvia
Late Pliocene		3.6	2.588	Gardner	1943	16984	Mollusca	7805	Bivalvia
Late Pliocene		3.6	2.588	Gardner	1943	16984	Mollusca	7805	Bivalvia
Late Pliocene		3.6	2.588	Gardner	1943	16984	Mollusca	7805	Bivalvia
Early Plioc	Late Plioce	5.333	2.588	Gardner	1943	16984	Mollusca	7805	Bivalvia
Early Plioc	Late Plioce	5.333	2.588	Gardner	1943	16984	Mollusca	7805	Bivalvia
Late Pliocene		3.6	2.588	Gardner	1943	16984	Mollusca	7805	Bivalvia
Late Pliocene		3.6	2.588	Gardner	1943	16984	Mollusca	7805	Bivalvia
Pleistocene		2.588	0.0117	Gardner	1943	16984	Mollusca	7805	Bivalvia
Early Pleistocene		2.588	0.781	Gardner	1943	16984	Mollusca	7805	Bivalvia
Early Pleistocene		2.588	0.781	Gardner	1943	16984	Mollusca	7805	Bivalvia
Early Pleistocene		2.588	0.781	Gardner	1943	16984	Mollusca	7805	Bivalvia
Late Pliocene		3.6	2.588	Gardner	1943	16984	Mollusca	7805	Bivalvia
Early Pleistocene		2.588	0.781	Gardner	1943	16984	Mollusca	7805	Bivalvia
Early Pleistocene		2.588	0.781	Gardner	1943	16984	Mollusca	7805	Bivalvia
Early Pleistocene		2.588	0.781	Miller	1990	17561	Mollusca	7805	Bivalvia
Early Pleistocene		2.588	0.781	Miller	1990	17561	Mollusca	7805	Bivalvia

Early Pleistocene	2.588	0.781	Miller	1990	17561	Mollusca	7805	Bivalvia
Early Pleistocene	2.588	0.781	Miller	1990	17561	Mollusca	7805	Bivalvia
Early Pleistocene	2.588	0.781	Miller	1990	17561	Mollusca	7805	Bivalvia
Early Pleistocene	2.588	0.781	Miller	1990	17561	Mollusca	7805	Bivalvia
Early Pleistocene	2.588	0.781	Miller	1990	17561	Mollusca	7805	Bivalvia
Early Pleistocene	2.588	0.781	Miller	1990	17561	Mollusca	7805	Bivalvia
Late Pleistocene	0.126	0.0117	Richards	1962	18974	Mollusca	7805	Bivalvia
Late Pleistocene	0.126	0.0117	Richards	1962	18974	Mollusca	7805	Bivalvia
Late Pleistocene	0.126	0.0117	Richards	1962	18974	Mollusca	7805	Bivalvia
Late Pleistocene	0.126	0.0117	Richards	1962	18974	Mollusca	7805	Bivalvia
Late Pleistocene	0.126	0.0117	Richards	1962	18974	Mollusca	7805	Bivalvia
Late Pleistocene	0.126	0.0117	Hendy et a	2008	19909	Mollusca	7805	Bivalvia
Late Pleistocene	0.126	0.0117	Hendy et a	2008	19909	Mollusca	7805	Bivalvia
Gelasian	2.588	1.806	Bailey and	1986	23840	Mollusca	7805	Bivalvia
Gelasian	2.588	1.806	Bailey and	1986	23840	Mollusca	7805	Bivalvia
Gelasian	2.588	1.806	Bailey and	1986	23840	Mollusca	7805	Bivalvia
Early Pleistocene	2.588	0.781	Hendy et a	2008	19909	Mollusca	7805	Bivalvia
Late Pleistocene	0.126	0.0117	Hendy et a	2008	19909	Mollusca	7805	Bivalvia
Late Pliocene	3.6	2.588	Hendy et a	2008	19909	Mollusca	7805	Bivalvia
Late Pliocene	3.6	2.588	Hendy et a	2008	19909	Mollusca	7805	Bivalvia
Late Pliocene	3.6	2.588	Hendy et a	2008	19909	Mollusca	7805	Bivalvia
Pliocene	5.333	2.588	Hendy et a	2008	19909	Mollusca	7805	Bivalvia
Pliocene	5.333	2.588	Hendy et a	2008	19909	Mollusca	7805	Bivalvia
Late Pliocene	3.6	2.588	Hendy et a	2008	19909	Mollusca	7805	Bivalvia
Late Pliocene	3.6	2.588	Hendy et a	2008	19909	Mollusca	7805	Bivalvia
Late Pliocene	3.6	2.588	Hendy et a	2008	19909	Mollusca	7805	Bivalvia
Late Pliocene	3.6	2.588	Hendy et a	2008	19909	Mollusca	7805	Bivalvia
Early Pleistocene	2.588	0.781	Hendy et a	2008	19909	Mollusca	7805	Bivalvia
Early Pleistocene	2.588	0.781	Hendy et a	2008	19909	Mollusca	7805	Bivalvia
Early Pleistocene	2.588	0.781	Hendy et a	2008	19909	Mollusca	7805	Bivalvia
Early Pleistocene	2.588	0.781	Hendy et a	2008	19909	Mollusca	7805	Bivalvia
Early Pleistocene	2.588	0.781	Hendy et a	2008	19909	Mollusca	7805	Bivalvia
Early Pleistocene	2.588	0.781	Hendy et a	2008	19909	Mollusca	7805	Bivalvia
Early Pleistocene	2.588	0.781	Hendy et a	2008	19909	Mollusca	7805	Bivalvia
Early Pleistocene	2.588	0.781	Hendy et a	2008	19909	Mollusca	7805	Bivalvia
Early Pleistocene	2.588	0.781	Hendy et a	2008	19909	Mollusca	7805	Bivalvia
Early Pleistocene	2.588	0.781	Miller and	1988	72890	Mollusca	7805	Bivalvia
Early Pleistocene	2.588	0.781	Miller and	1988	72890	Mollusca	7805	Bivalvia
Early Pleistocene	2.588	0.781	Miller and	1988	72890	Mollusca	7805	Bivalvia
Pleistocene	2.588	0.0117	Coch	1971	441	Mollusca	7805	Bivalvia
Late Pleistocene	0.126	0.0117	Spencer ar	1987	15525	Mollusca	7805	Bivalvia
Late Pleistocene	0.126	0.0117	Spencer ar	1987	15525	Mollusca	7805	Bivalvia
Late Pleistocene	0.126	0.0117	Spencer ar	1987	15525	Mollusca	7805	Bivalvia
Late Pleistocene	0.126	0.0117	Spencer ar	1987	15525	Mollusca	7805	Bivalvia
Late Pleistocene	0.126	0.0117	Spencer ar	1987	15525	Mollusca	7805	Bivalvia
Late Pleistocene	0.126	0.0117	Hendy et a	2008	19909	Mollusca	7805	Bivalvia
Late Pliocene	3.6	2.588	Ward	1993	26351	Mollusca	7805	Bivalvia

Late Pliocene	3.6	2.588	Ward	1993	26351	Mollusca	7805	Bivalvia
Late Pliocene	3.6	2.588	Ward	1993	26351	Mollusca	7805	Bivalvia
Late Pliocene	3.6	2.588	Ward	1993	26351	Mollusca	7805	Bivalvia
Late Pliocene	3.6	2.588	Hendy et a	2008	19909	Mollusca	7805	Bivalvia
Late Pliocene	3.6	2.588	Hendy et a	2008	19909	Mollusca	7805	Bivalvia
Early Pleistocene	2.588	0.781	DuBar	1962	11119	Mollusca	7805	Bivalvia
Calabrian	1.806	0.781	DuBar	1962	11119	Mollusca	7805	Bivalvia
Late Pliocene	3.6	2.588	Mansfield	1932	17094	Mollusca	7805	Bivalvia
Early Pleistocene	2.588	0.781	Portell et a	1992	17634	Mollusca	7805	Bivalvia
Middle Pleistocene	0.781	0.126	Hoerle	1970	19536	Mollusca	7805	Bivalvia
Middle Pleistocene	0.781	0.126	Hoerle	1970	19536	Mollusca	7805	Bivalvia
Late Pleistocene	0.126	0.0117	Hendy et a	2008	19909	Mollusca	7805	Bivalvia
Early Pleistocene	2.588	0.781	Olsson anc	1953	25463	Mollusca	7805	Bivalvia
Early Pleistocene	2.588	0.781	Olsson anc	1953	25463	Mollusca	7805	Bivalvia
Early Pleistocene	2.588	0.781	Olsson anc	1953	25463	Mollusca	7805	Bivalvia
Early Pleistocene	2.588	0.781	Olsson anc	1953	25463	Mollusca	7805	Bivalvia
Early Pleistocene	2.588	0.781	Olsson anc	1953	25463	Mollusca	7805	Bivalvia
Early Pleistocene	2.588	0.781	Olsson anc	1953	25463	Mollusca	7805	Bivalvia
Late Pleistocene	0.126	0.0117	Richards	1962	18974	Mollusca	7805	Bivalvia
Late Pleistocene	0.126	0.0117	Richards	1962	18974	Mollusca	7805	Bivalvia
Late Pleistocene	0.126	0.0117	Richards	1962	18974	Mollusca	7805	Bivalvia
Late Pleistocene	0.126	0.0117	Richards	1962	18974	Mollusca	7805	Bivalvia
Late Pleistocene	0.126	0.0117	Hendy et a	2008	19909	Mollusca	7805	Bivalvia
Early Pleistocene	2.588	0.781	Cooke	1936	31145	Mollusca	7805	Bivalvia
Early Pleistocene	2.588	0.781	Gardner	1943	16984	Mollusca	7805	Bivalvia
Pleistocene	2.588	0.0117	Gardner	1943	16984	Mollusca	7805	Bivalvia
Early Pleistocene	2.588	0.781	Gardner	1943	16984	Mollusca	7805	Bivalvia
Late Pleistocene	0.126	0.0117	Richards	1962	18974	Mollusca	7805	Bivalvia
Late Pleistocene	0.126	0.0117	Richards	1962	18974	Mollusca	7805	Bivalvia
Early Pleistocene	2.588	0.781	Hendy et a	2008	19909	Mollusca	7805	Bivalvia
Late Pliocene	3.6	2.588	Hendy et a	2008	19909	Mollusca	7805	Bivalvia
Late Pliocene	3.6	2.588	Hendy et a	2008	19909	Mollusca	7805	Bivalvia
Early Pleistocene	2.588	0.781	Hendy et a	2008	19909	Mollusca	7805	Bivalvia
Late Pleistocene	0.126	0.0117	Spencer ar	1987	15525	Mollusca	7805	Bivalvia
Late Pleistocene	0.126	0.0117	Spencer ar	1987	15525	Mollusca	7805	Bivalvia
Late Pliocene	3.6	2.588	Hendy et a	2008	19909	Mollusca	7805	Bivalvia
Late Pliocene	3.6	2.588	Hendy et a	2008	19909	Mollusca	7805	Bivalvia
Late Pleistocene	0.126	0.0117	Spencer ar	1987	15525	Mollusca	7805	Bivalvia
Late Pleistocene	0.126	0.0117	Richards	1962	18974	Mollusca	7805	Bivalvia
Early Pleistocene	2.588	0.781	Cooke	1936	31145	Mollusca	7805	Bivalvia
Pleistocene	2.588	0.0117	Gardner	1943	16984	Mollusca	7805	Bivalvia
Early Pleistocene	2.588	0.781	Hendy et a	2008	19909	Mollusca	7805	Bivalvia
Late Pliocene	3.6	2.588	Edwards et	2005	17631	Mollusca	7805	Bivalvia
Late Pliocene	3.6	2.588	Edwards et	2005	17631	Mollusca	7805	Bivalvia
Late Pleistocene	0.126	0.0117	Kittle and I	2010	40884	Mollusca	7805	Bivalvia
Pleistocene	2.588	0.0117	Auffenberg	2006	82817	Mollusca	7805	Bivalvia
Pleistocene	2.588	0.0117	Auffenberg	2006	82817	Mollusca	7805	Bivalvia

Late Pleistocene	0.126	0.0117	Guilday et	1977	35027	Mollusca	7805	Bivalvia
Late Pleistocene	0.126	0.0117	Guilday et	1977	35027	Mollusca	7805	Bivalvia
Early Pleistocene	2.588	0.781	Mansfield	1932	11083	Mollusca	7805	Bivalvia
Early Pleistocene	2.588	0.781	Mansfield	1932	11083	Mollusca	7805	Bivalvia
Early Pleistocene	2.588	0.781	DuBar	1962	11119	Mollusca	7805	Bivalvia
Calabrian	1.806	0.781	DuBar	1962	11119	Mollusca	7805	Bivalvia
Middle Pleistocene	0.781	0.126	McGinty	1970	19114	Mollusca	7805	Bivalvia
Early Pleistocene	2.588	0.781	Olsson anc	1953	25463	Mollusca	7805	Bivalvia
Early Pleistocene	2.588	0.781	Olsson anc	1953	25463	Mollusca	7805	Bivalvia
Early Pliocene	5.333	3.6	Olsson anc	1964	26836	Mollusca	7805	Bivalvia
Middle Pleistocene	0.781	0.126	Hendy et a	2008	19909	Mollusca	7805	Bivalvia
Pliocene	5.333	2.588	Petuch	1986	47440	Mollusca	7805	Bivalvia
Zanclean	5.333	3.6	Campbell a	1995	17558	Mollusca	7805	Bivalvia
Early Pleistocene	2.588	0.781	DuBar	1962	11119	Mollusca	7805	Bivalvia
Late Pliocene	3.6	2.588	Mansfield	1932	17094	Mollusca	7805	Bivalvia
Late Pliocene	3.6	2.588	Mansfield	1932	17094	Mollusca	7805	Bivalvia
Middle Pleistocene	0.781	0.126	McGinty	1970	19114	Mollusca	7805	Bivalvia
Middle Pleistocene	0.781	0.126	McGinty	1970	19114	Mollusca	7805	Bivalvia
Early Pleistocene	2.588	0.781	Olsson anc	1953	25463	Mollusca	7805	Bivalvia
Early Pleistocene	2.588	0.781	Olsson anc	1953	25463	Mollusca	7805	Bivalvia
Early Pleistocene	2.588	0.781	Olsson anc	1953	25463	Mollusca	7805	Bivalvia
Early Pleistocene	2.588	0.781	Olsson anc	1953	25463	Mollusca	7805	Bivalvia
Early Pleistocene	2.588	0.781	Olsson anc	1953	25463	Mollusca	7805	Bivalvia
Early Pleistocene	2.588	0.781	Olsson anc	1953	25463	Mollusca	7805	Bivalvia
Early Pleistocene	2.588	0.781	Olsson anc	1953	25463	Mollusca	7805	Bivalvia
Early Pleistocene	2.588	0.781	Olsson anc	1953	25463	Mollusca	7805	Bivalvia
Late Pleistocene	0.126	0.0117	Kittle and I	2010	40884	Mollusca	7805	Bivalvia
Late Pleistocene	0.126	0.0117	DuBar	1971	17556	Mollusca	7805	Bivalvia
Late Pleistocene	0.126	0.0117	Richards	1962	18974	Mollusca	7805	Bivalvia
Late Pleistocene	0.126	0.0117	Richards	1962	18974	Mollusca	7805	Bivalvia
Late Pleistocene	0.126	0.0117	Richards	1962	18974	Mollusca	7805	Bivalvia
Late Pleistocene	0.126	0.0117	Richards	1962	18974	Mollusca	7805	Bivalvia
Late Pleistocene	0.126	0.0117	Hendy et a	2008	19909	Mollusca	7805	Bivalvia
Late Pleistocene	0.126	0.0117	Hendy et a	2008	19909	Mollusca	7805	Bivalvia
Late Pliocene	3.6	2.588	Ward and I	1987	16700	Mollusca	7805	Bivalvia
Late Pliocene	3.6	2.588	Ward and I	1987	16700	Mollusca	7805	Bivalvia
Early Pleistocene	2.588	0.781	Ward and I	1987	16700	Mollusca	7805	Bivalvia
Early Pleistocene	2.588	0.781	Ward and I	1987	16700	Mollusca	7805	Bivalvia
Early Pleistocene	2.588	0.781	Ward and I	1987	16700	Mollusca	7805	Bivalvia
Pleistocene	2.588	0.0117	Gardner	1943	16984	Mollusca	7805	Bivalvia
Late Pliocene	3.6	2.588	Gardner	1943	16984	Mollusca	7805	Bivalvia
Pleistocene	2.588	0.0117	Gardner	1943	16984	Mollusca	7805	Bivalvia
Late Pliocene	3.6	2.588	Gardner	1943	16984	Mollusca	7805	Bivalvia
Pleistocene	2.588	0.0117	Gardner	1943	16984	Mollusca	7805	Bivalvia
Early Pleistocene	2.588	0.781	Gardner	1943	16984	Mollusca	7805	Bivalvia
Early Pleistocene	2.588	0.781	Gardner	1943	16984	Mollusca	7805	Bivalvia
Pleistocene	2.588	0.0117	Gardner	1943	16984	Mollusca	7805	Bivalvia

Early Pleistocene		2.588	0.781	Gardner	1943	16984	Mollusca	7805	Bivalvia
Pleistocene		2.588	0.0117	Gardner	1943	16984	Mollusca	7805	Bivalvia
Early Pleistocene		2.588	0.781	Miller	1990	17561	Mollusca	7805	Bivalvia
Early Pleistocene		2.588	0.781	Miller	1990	17561	Mollusca	7805	Bivalvia
Early Pleistocene		2.588	0.781	Miller	1990	17561	Mollusca	7805	Bivalvia
Early Pleistocene		2.588	0.781	Miller	1990	17561	Mollusca	7805	Bivalvia
Late Pleistocene		0.126	0.0117	Richards	1962	18974	Mollusca	7805	Bivalvia
Late Pleistocene		0.126	0.0117	Richards	1962	18974	Mollusca	7805	Bivalvia
Gelasian		2.588	1.806	Bailey and	1986	23840	Mollusca	7805	Bivalvia
Gelasian		2.588	1.806	Bailey and	1986	23840	Mollusca	7805	Bivalvia
Late Pliocene		3.6	2.588	Edwards e	2005	17631	Mollusca	7805	Bivalvia
Late Pleistocene		0.126	0.0117	Sellards	1917	9011	Mollusca	7805	Bivalvia
Late Pleistocene		0.126	0.0117	Sellards	1917	9011	Mollusca	7805	Bivalvia
Early Pleistocene		2.588	0.781	Mansfield	1932	11083	Mollusca	7805	Bivalvia
Early Pleistocene		2.588	0.781	DuBar	1962	11119	Mollusca	7805	Bivalvia
Early Pleistocene		2.588	0.781	DuBar	1962	11119	Mollusca	7805	Bivalvia
Early Pleistocene		2.588	0.781	DuBar	1962	11119	Mollusca	7805	Bivalvia
Early Pleistocene		2.588	0.781	DuBar	1962	11119	Mollusca	7805	Bivalvia
Early Pleistocene		2.588	0.781	DuBar	1962	11119	Mollusca	7805	Bivalvia
Early Pleistocene		2.588	0.781	DuBar	1962	11119	Mollusca	7805	Bivalvia
Early Pleistocene		2.588	0.781	DuBar	1962	11119	Mollusca	7805	Bivalvia
Early Pleistocene		2.588	0.781	DuBar	1962	11119	Mollusca	7805	Bivalvia
Early Pleistocene		2.588	0.781	DuBar	1962	11119	Mollusca	7805	Bivalvia
Early Pleistocene		2.588	0.781	DuBar	1962	11119	Mollusca	7805	Bivalvia
Early Pleistocene		2.588	0.781	DuBar	1962	11119	Mollusca	7805	Bivalvia
Early Pleistocene		2.588	0.781	DuBar	1962	11119	Mollusca	7805	Bivalvia
Early Pleistocene		2.588	0.781	DuBar	1962	11119	Mollusca	7805	Bivalvia
Calabrian		1.806	0.781	DuBar	1962	11119	Mollusca	7805	Bivalvia
Calabrian		1.806	0.781	DuBar	1962	11119	Mollusca	7805	Bivalvia
Calabrian		1.806	0.781	DuBar	1962	11119	Mollusca	7805	Bivalvia
Calabrian		1.806	0.781	DuBar	1962	11119	Mollusca	7805	Bivalvia
Calabrian		1.806	0.781	DuBar	1962	11119	Mollusca	7805	Bivalvia
Early Pleistocene		2.588	0.781	DuBar	1962	11119	Mollusca	7805	Bivalvia
Early Pleistocene		2.588	0.781	DuBar	1962	11119	Mollusca	7805	Bivalvia
Early Pleistocene		2.588	0.781	DuBar	1962	11119	Mollusca	7805	Bivalvia
Early Pleistocene		2.588	0.781	DuBar	1962	11119	Mollusca	7805	Bivalvia
Late Pleistocene		0.126	0.0117	DuBar	1962	11119	Mollusca	7805	Bivalvia
Late Pleistocene		0.126	0.0117	DuBar	1962	11119	Mollusca	7805	Bivalvia
Pliocene	Pleistocen	5.333	0.0117	DuBar	1962	11119	Mollusca	7805	Bivalvia
Pliocene	Pleistocen	5.333	0.0117	DuBar	1962	11119	Mollusca	7805	Bivalvia
Pliocene	Pleistocen	5.333	0.0117	DuBar	1962	11119	Mollusca	7805	Bivalvia
Pliocene	Pleistocen	5.333	0.0117	DuBar	1962	11119	Mollusca	7805	Bivalvia
Pliocene	Pleistocen	5.333	0.0117	DuBar	1962	11119	Mollusca	7805	Bivalvia
Late Pliocene		3.6	2.588	Mansfield	1932	17094	Mollusca	7805	Bivalvia
Late Pliocene		3.6	2.588	Mansfield	1932	17094	Mollusca	7805	Bivalvia
Late Pliocene		3.6	2.588	Mansfield	1932	17094	Mollusca	7805	Bivalvia
Late Pliocene		3.6	2.588	Mansfield	1932	17094	Mollusca	7805	Bivalvia

Late Pliocene	3.6	2.588	Mansfield	1932	17094	Mollusca	7805	Bivalvia
Late Pliocene	3.6	2.588	Mansfield	1932	17094	Mollusca	7805	Bivalvia
Late Pliocene	3.6	2.588	Mansfield	1932	17094	Mollusca	7805	Bivalvia
Late Pliocene	3.6	2.588	Mansfield	1932	17094	Mollusca	7805	Bivalvia
Late Pliocene	3.6	2.588	Mansfield	1932	17094	Mollusca	7805	Bivalvia
Late Pliocene	3.6	2.588	Mansfield	1932	17094	Mollusca	7805	Bivalvia
Late Pliocene	3.6	2.588	Mansfield	1932	17094	Mollusca	7805	Bivalvia
Late Pliocene	3.6	2.588	Mansfield	1932	17094	Mollusca	7805	Bivalvia
Late Pliocene	3.6	2.588	Mansfield	1932	17094	Mollusca	7805	Bivalvia
Early Pleistocene	2.588	0.781	Portell et a	1992	17634	Mollusca	7805	Bivalvia
Early Pleistocene	2.588	0.781	Portell et a	1992	17634	Mollusca	7805	Bivalvia
Early Pleistocene	2.588	0.781	Portell et a	1992	17634	Mollusca	7805	Bivalvia
Early Pleistocene	2.588	0.781	Portell et a	1992	17634	Mollusca	7805	Bivalvia
Early Pleistocene	2.588	0.781	Portell et a	1992	17634	Mollusca	7805	Bivalvia
Early Pleistocene	2.588	0.781	Portell et a	1992	17634	Mollusca	7805	Bivalvia
Middle Pleistocene	0.781	0.126	Portell et a	1992	17634	Mollusca	7805	Bivalvia
Middle Pleistocene	0.781	0.126	Portell et a	1992	17634	Mollusca	7805	Bivalvia
Middle Pleistocene	0.781	0.126	Portell et a	1992	17634	Mollusca	7805	Bivalvia
Middle Pleistocene	0.781	0.126	Portell et a	1992	17634	Mollusca	7805	Bivalvia
Middle Pleistocene	0.781	0.126	Portell et a	1992	17634	Mollusca	7805	Bivalvia
Late Pleistocene	0.126	0.0117	Portell et a	1992	17634	Mollusca	7805	Bivalvia
Late Pleistocene	0.126	0.0117	Portell et a	1992	17634	Mollusca	7805	Bivalvia
Middle Pleistocene	0.781	0.126	Hoerle	1970	19536	Mollusca	7805	Bivalvia
Middle Pleistocene	0.781	0.126	Hoerle	1970	19536	Mollusca	7805	Bivalvia
Middle Pleistocene	0.781	0.126	Hoerle	1970	19536	Mollusca	7805	Bivalvia
Middle Pleistocene	0.781	0.126	Hoerle	1970	19536	Mollusca	7805	Bivalvia
Middle Pleistocene	0.781	0.126	Hoerle	1970	19536	Mollusca	7805	Bivalvia
Middle Pleistocene	0.781	0.126	Hoerle	1970	19536	Mollusca	7805	Bivalvia
Middle Pleistocene	0.781	0.126	Hoerle	1970	19536	Mollusca	7805	Bivalvia
Middle Pleistocene	0.781	0.126	Hoerle	1970	19536	Mollusca	7805	Bivalvia
Middle Pleistocene	0.781	0.126	Hoerle	1970	19536	Mollusca	7805	Bivalvia
Middle Pleistocene	0.781	0.126	Hoerle	1970	19536	Mollusca	7805	Bivalvia
Middle Pleistocene	0.781	0.126	Hoerle	1970	19536	Mollusca	7805	Bivalvia
Middle Pleistocene	0.781	0.126	Hoerle	1970	19536	Mollusca	7805	Bivalvia
Middle Pleistocene	0.781	0.126	Hoerle	1970	19536	Mollusca	7805	Bivalvia
Middle Pleistocene	0.781	0.126	Hoerle	1970	19536	Mollusca	7805	Bivalvia
Middle Pleistocene	0.781	0.126	Hoerle	1970	19536	Mollusca	7805	Bivalvia
Middle Pleistocene	0.781	0.126	Hoerle	1970	19536	Mollusca	7805	Bivalvia
Middle Pleistocene	0.781	0.126	Hoerle	1970	19536	Mollusca	7805	Bivalvia
Late Pliocene	3.6	2.588	Mansfield	1939	19112	Mollusca	7805	Bivalvia
Late Pleistocene	0.126	0.0117	Hendy et a	2008	19909	Mollusca	7805	Bivalvia
Late Pleistocene	0.126	0.0117	Hendy et a	2008	19909	Mollusca	7805	Bivalvia
Late Pleistocene	0.126	0.0117	Hendy et a	2008	19909	Mollusca	7805	Bivalvia
Late Pleistocene	0.126	0.0117	Hendy et a	2008	19909	Mollusca	7805	Bivalvia
Late Pleistocene	0.126	0.0117	Hendy et a	2008	19909	Mollusca	7805	Bivalvia
Late Pleistocene	0.126	0.0117	Hendy et a	2008	19909	Mollusca	7805	Bivalvia

Late Pleistocene	0.126	0.0117	Hendy et a	2008	19909	Mollusca	7805	Bivalvia
Late Pleistocene	0.126	0.0117	Hendy et a	2008	19909	Mollusca	7805	Bivalvia
Late Pleistocene	0.126	0.0117	Hendy et a	2008	19909	Mollusca	7805	Bivalvia
Late Pleistocene	0.126	0.0117	Hendy et a	2008	19909	Mollusca	7805	Bivalvia
Late Pleistocene	0.126	0.0117	Hendy et a	2008	19909	Mollusca	7805	Bivalvia
Late Pleistocene	0.126	0.0117	Hendy et a	2008	19909	Mollusca	7805	Bivalvia
Late Pleistocene	0.126	0.0117	Hendy et a	2008	19909	Mollusca	7805	Bivalvia
Early Pleistocene	2.588	0.781	Schroeder	1954	25303	Mollusca	7805	Bivalvia
Early Pleistocene	2.588	0.781	Olsson anc	1953	25463	Mollusca	7805	Bivalvia
Early Pleistocene	2.588	0.781	Olsson anc	1953	25463	Mollusca	7805	Bivalvia
Early Pleistocene	2.588	0.781	Olsson anc	1953	25463	Mollusca	7805	Bivalvia
Early Pleistocene	2.588	0.781	Olsson anc	1953	25463	Mollusca	7805	Bivalvia
Early Pleistocene	2.588	0.781	Olsson anc	1953	25463	Mollusca	7805	Bivalvia
Early Pleistocene	2.588	0.781	Olsson anc	1953	25463	Mollusca	7805	Bivalvia
Early Pleistocene	2.588	0.781	Olsson anc	1953	25463	Mollusca	7805	Bivalvia
Early Pleistocene	2.588	0.781	Olsson anc	1953	25463	Mollusca	7805	Bivalvia
Early Pleistocene	2.588	0.781	Olsson anc	1953	25463	Mollusca	7805	Bivalvia
Early Pleistocene	2.588	0.781	Olsson anc	1953	25463	Mollusca	7805	Bivalvia
Early Pleistocene	2.588	0.781	Olsson anc	1953	25463	Mollusca	7805	Bivalvia
Early Pleistocene	2.588	0.781	Olsson anc	1953	25463	Mollusca	7805	Bivalvia
Early Pleistocene	2.588	0.781	Olsson anc	1953	25463	Mollusca	7805	Bivalvia
Early Pleistocene	2.588	0.781	Olsson anc	1953	25463	Mollusca	7805	Bivalvia
Early Pleistocene	2.588	0.781	Olsson anc	1953	25463	Mollusca	7805	Bivalvia
Early Pleistocene	2.588	0.781	Olsson anc	1953	25463	Mollusca	7805	Bivalvia
Early Pleistocene	2.588	0.781	Olsson anc	1953	25463	Mollusca	7805	Bivalvia
Early Pleistocene	2.588	0.781	Hendy et a	2008	19909	Mollusca	7805	Bivalvia
Early Pleistocene	2.588	0.781	Hendy et a	2008	19909	Mollusca	7805	Bivalvia
Early Pleistocene	2.588	0.781	Hendy et a	2008	19909	Mollusca	7805	Bivalvia
Early Pleistocene	2.588	0.781	Hendy et a	2008	19909	Mollusca	7805	Bivalvia
Early Pleistocene	2.588	0.781	Hendy et a	2008	19909	Mollusca	7805	Bivalvia
Early Pleistocene	2.588	0.781	Hendy et a	2008	19909	Mollusca	7805	Bivalvia
Late Pleistocene	0.126	0.0117	Hendy et a	2008	19909	Mollusca	7805	Bivalvia
Late Pleistocene	0.126	0.0117	Hendy et a	2008	19909	Mollusca	7805	Bivalvia
Late Pleistocene	0.126	0.0117	Hendy et a	2008	19909	Mollusca	7805	Bivalvia
Early Pleistocene	2.588	0.781	DuBar	1958	25514	Mollusca	7805	Bivalvia
Late Pleistocene	0.126	0.0117	DuBar	1958	25514	Mollusca	7805	Bivalvia
Late Pleistocene	0.126	0.0117	Hendy et a	2008	19909	Mollusca	7805	Bivalvia
Late Pleistocene	0.126	0.0117	Hendy et a	2008	19909	Mollusca	7805	Bivalvia
Late Pliocene	3.6	2.588	Hendy et a	2008	19909	Mollusca	7805	Bivalvia
Late Pliocene	3.6	2.588	Hendy et a	2008	19909	Mollusca	7805	Bivalvia
Early Pleistocene	2.588	0.781	Hendy et a	2008	19909	Mollusca	7805	Bivalvia
Early Pleistocene	2.588	0.781	Hendy et a	2008	19909	Mollusca	7805	Bivalvia
Early Pleistocene	2.588	0.781	Hendy et a	2008	19909	Mollusca	7805	Bivalvia
Early Pleistocene	2.588	0.781	Hendy et a	2008	19909	Mollusca	7805	Bivalvia
Early Pleistocene	2.588	0.781	Hendy et a	2008	19909	Mollusca	7805	Bivalvia
Early Pleistocene	2.588	0.781	Hendy et a	2008	19909	Mollusca	7805	Bivalvia
Pleistocene	2.588	0.0117	Hendy et a	2008	19909	Mollusca	7805	Bivalvia

Pleistocene	2.588	0.0117	Hendy et a	2008	19909	Mollusca	7805	Bivalvia
Pleistocene	2.588	0.0117	Hendy et a	2008	19909	Mollusca	7805	Bivalvia
Late Pliocene	3.6	2.588	Hendy et a	2008	19909	Mollusca	7805	Bivalvia
Late Pliocene	3.6	2.588	Hendy et a	2008	19909	Mollusca	7805	Bivalvia
Middle Pleistocene	0.781	0.126	Hendy et a	2008	19909	Mollusca	7805	Bivalvia
Late Pleistocene	0.126	0.0117	Hendy et a	2008	19909	Mollusca	7805	Bivalvia
Late Pleistocene	0.126	0.0117	Vokes	1972	31266	Mollusca	7805	Bivalvia
Late Pleistocene	0.126	0.0117	Vokes	1972	31266	Mollusca	7805	Bivalvia
Late Pleistocene	0.126	0.0117	Vokes	1972	31266	Mollusca	7805	Bivalvia
Pleistocene	2.588	0.0117	Vokes	1972	31266	Mollusca	7805	Bivalvia
Late Pleistocene	0.126	0.0117	Vokes	1972	31266	Mollusca	7805	Bivalvia
Early Pleistocene	2.588	0.781	Vokes	1972	31266	Mollusca	7805	Bivalvia
Late Pliocene	3.6	2.588	Vokes	1972	31266	Mollusca	7805	Bivalvia
Late Pliocene	3.6	2.588	Vokes	1972	31266	Mollusca	7805	Bivalvia
Late Pliocene	3.6	2.588	Vokes	1972	31266	Mollusca	7805	Bivalvia
Late Pleistocene	0.126	0.0117	Kittle and I	2010	40884	Mollusca	7805	Bivalvia
Late Pleistocene	0.126	0.0117	Kittle and I	2010	40884	Mollusca	7805	Bivalvia
Late Pleistocene	0.126	0.0117	DuBar	1971	17556	Mollusca	7805	Bivalvia
Late Pleistocene	0.126	0.0117	DuBar	1971	17556	Mollusca	7805	Bivalvia
Late Pleistocene	0.126	0.0117	DuBar	1971	17556	Mollusca	7805	Bivalvia
Late Pleistocene	0.126	0.0117	DuBar	1971	17556	Mollusca	7805	Bivalvia
Late Pleistocene	0.126	0.0117	DuBar	1971	17556	Mollusca	7805	Bivalvia
Late Pleistocene	0.126	0.0117	DuBar	1971	17556	Mollusca	7805	Bivalvia
Late Pleistocene	0.126	0.0117	DuBar	1971	17556	Mollusca	7805	Bivalvia
Zanclean	5.333	3.6	Campbell a	1995	17558	Mollusca	7805	Bivalvia
Early Pliocene	5.333	3.6	Campbell a	1995	17558	Mollusca	7805	Bivalvia
Early Pleistocene	2.588	0.781	DuBar	1971	17556	Mollusca	7805	Bivalvia
Late Pleistocene	0.126	0.0117	Richards	1962	18974	Mollusca	7805	Bivalvia
Late Pleistocene	0.126	0.0117	Richards	1962	18974	Mollusca	7805	Bivalvia
Late Pleistocene	0.126	0.0117	Richards	1962	18974	Mollusca	7805	Bivalvia
Late Pleistocene	0.126	0.0117	Richards	1962	18974	Mollusca	7805	Bivalvia
Late Pleistocene	0.126	0.0117	Richards	1962	18974	Mollusca	7805	Bivalvia
Late Pleistocene	0.126	0.0117	Richards	1962	18974	Mollusca	7805	Bivalvia
Late Pleistocene	0.126	0.0117	Richards	1962	18974	Mollusca	7805	Bivalvia
Late Pleistocene	0.126	0.0117	Richards	1962	18974	Mollusca	7805	Bivalvia
Late Pleistocene	0.126	0.0117	Richards	1962	18974	Mollusca	7805	Bivalvia
Late Pleistocene	0.126	0.0117	Richards	1962	18974	Mollusca	7805	Bivalvia
Late Pleistocene	0.126	0.0117	Richards	1962	18974	Mollusca	7805	Bivalvia
Late Pleistocene	0.126	0.0117	Richards	1962	18974	Mollusca	7805	Bivalvia
Late Pleistocene	0.126	0.0117	Richards	1962	18974	Mollusca	7805	Bivalvia
Late Pleistocene	0.126	0.0117	Richards	1962	18974	Mollusca	7805	Bivalvia
Late Pleistocene	0.126	0.0117	Richards	1962	18974	Mollusca	7805	Bivalvia
Late Pleistocene	0.126	0.0117	Hendy et a	2008	19909	Mollusca	7805	Bivalvia
Late Pleistocene	0.126	0.0117	Hendy et a	2008	19909	Mollusca	7805	Bivalvia
Late Pleistocene	0.126	0.0117	Hendy et a	2008	19909	Mollusca	7805	Bivalvia
Late Pleistocene	0.126	0.0117	Hendy et a	2008	19909	Mollusca	7805	Bivalvia
Late Pleistocene	0.126	0.0117	Hendy et a	2008	19909	Mollusca	7805	Bivalvia

Late Pleistocene		0.126	0.0117	Hendy et a	2008	19909	Mollusca	7805	Bivalvia
Late Pleistocene		0.126	0.0117	Hendy et a	2008	19909	Mollusca	7805	Bivalvia
Late Pleistocene		0.126	0.0117	Hendy et a	2008	19909	Mollusca	7805	Bivalvia
Late Pleistocene		0.126	0.0117	Hendy et a	2008	19909	Mollusca	7805	Bivalvia
Late Pleistocene		0.126	0.0117	Hendy et a	2008	19909	Mollusca	7805	Bivalvia
Pleistocene		2.588	0.0117	Hendy et a	2008	19909	Mollusca	7805	Bivalvia
Pleistocene		2.588	0.0117	Hendy et a	2008	19909	Mollusca	7805	Bivalvia
Pleistocene		2.588	0.0117	Hendy et a	2008	19909	Mollusca	7805	Bivalvia
Late Pliocene		3.6	2.588	Cooke	1936	31145	Mollusca	7805	Bivalvia
Late Pliocene		3.6	2.588	Cooke	1936	31145	Mollusca	7805	Bivalvia
Pleistocene		2.588	0.0117	Holmes	1860	64712	Mollusca	7805	Bivalvia
Late Pliocene		3.6	2.588	Ward and I	1987	16700	Mollusca	7805	Bivalvia
Late Pliocene		3.6	2.588	Ward and I	1987	16700	Mollusca	7805	Bivalvia
Early Pleistocene		2.588	0.781	Ward and I	1987	16700	Mollusca	7805	Bivalvia
Early Pleistocene		2.588	0.781	Ward and I	1987	16700	Mollusca	7805	Bivalvia
Pleistocene		2.588	0.0117	Gardner	1943	16984	Mollusca	7805	Bivalvia
Early Pleistocene		2.588	0.781	Gardner	1943	16984	Mollusca	7805	Bivalvia
Late Pliocene		3.6	2.588	Gardner	1943	16984	Mollusca	7805	Bivalvia
Late Pliocene		3.6	2.588	Gardner	1943	16984	Mollusca	7805	Bivalvia
Early Plioc	Late Plioce	5.333	2.588	Gardner	1943	16984	Mollusca	7805	Bivalvia
Late Pliocene		3.6	2.588	Gardner	1943	16984	Mollusca	7805	Bivalvia
Late Pliocene		3.6	2.588	Gardner	1943	16984	Mollusca	7805	Bivalvia
Early Pleistocene		2.588	0.781	Gardner	1943	16984	Mollusca	7805	Bivalvia
Late Pliocene		3.6	2.588	Gardner	1943	16984	Mollusca	7805	Bivalvia
Early Pleistocene		2.588	0.781	Gardner	1943	16984	Mollusca	7805	Bivalvia
Pleistocene		2.588	0.0117	Gardner	1943	16984	Mollusca	7805	Bivalvia
Early Pleistocene		2.588	0.781	Gardner	1943	16984	Mollusca	7805	Bivalvia
Early Pleistocene		2.588	0.781	Gardner	1943	16984	Mollusca	7805	Bivalvia
Early Pleistocene		2.588	0.781	Gardner	1943	16984	Mollusca	7805	Bivalvia
Early Plioc	Late Plioce	5.333	2.588	Gardner	1943	16984	Mollusca	7805	Bivalvia
Late Pliocene		3.6	2.588	Gardner	1943	16984	Mollusca	7805	Bivalvia
Late Pliocene		3.6	2.588	Gardner	1943	16984	Mollusca	7805	Bivalvia
Pleistocene		2.588	0.0117	Gardner	1943	16984	Mollusca	7805	Bivalvia
Late Pliocene		3.6	2.588	Gardner	1943	16984	Mollusca	7805	Bivalvia
Late Pliocene		3.6	2.588	Gardner	1943	16984	Mollusca	7805	Bivalvia
Early Pleistocene		2.588	0.781	Miller	1990	17561	Mollusca	7805	Bivalvia
Late Pleistocene		0.126	0.0117	Richards	1962	18974	Mollusca	7805	Bivalvia
Late Pleistocene		0.126	0.0117	Richards	1962	18974	Mollusca	7805	Bivalvia
Late Pleistocene		0.126	0.0117	Richards	1962	18974	Mollusca	7805	Bivalvia
Late Pleistocene		0.126	0.0117	Richards	1962	18974	Mollusca	7805	Bivalvia
Late Pleistocene		0.126	0.0117	Richards	1962	18974	Mollusca	7805	Bivalvia
Late Pleistocene		0.126	0.0117	Richards	1962	18974	Mollusca	7805	Bivalvia
Late Pleistocene		0.126	0.0117	Richards	1962	18974	Mollusca	7805	Bivalvia
Gelasian		2.588	1.806	Bailey and	1986	23840	Mollusca	7805	Bivalvia
Early Pleistocene		2.588	0.781	Hendy et a	2008	19909	Mollusca	7805	Bivalvia
Early Pleistocene		2.588	0.781	Hendy et a	2008	19909	Mollusca	7805	Bivalvia
Early Pleistocene		2.588	0.781	Hendy et a	2008	19909	Mollusca	7805	Bivalvia

Late Pleistocene	0.126	0.0117	Hendy et a	2008	19909	Mollusca	7805	Bivalvia
Late Pliocene	3.6	2.588	Hendy et a	2008	19909	Mollusca	7805	Bivalvia
Late Pliocene	3.6	2.588	Hendy et a	2008	19909	Mollusca	7805	Bivalvia
Late Pliocene	3.6	2.588	Hendy et a	2008	19909	Mollusca	7805	Bivalvia
Late Pliocene	3.6	2.588	Hendy et a	2008	19909	Mollusca	7805	Bivalvia
Late Pliocene	3.6	2.588	Hendy et a	2008	19909	Mollusca	7805	Bivalvia
Late Pliocene	3.6	2.588	Hendy et a	2008	19909	Mollusca	7805	Bivalvia
Late Pliocene	3.6	2.588	Hendy et a	2008	19909	Mollusca	7805	Bivalvia
Late Pliocene	3.6	2.588	Hendy et a	2008	19909	Mollusca	7805	Bivalvia
Late Pliocene	3.6	2.588	Hendy et a	2008	19909	Mollusca	7805	Bivalvia
Early Pleistocene	2.588	0.781	Hendy et a	2008	19909	Mollusca	7805	Bivalvia
Early Pleistocene	2.588	0.781	Hendy et a	2008	19909	Mollusca	7805	Bivalvia
Early Pleistocene	2.588	0.781	Hendy et a	2008	19909	Mollusca	7805	Bivalvia
Early Pleistocene	2.588	0.781	Hendy et a	2008	19909	Mollusca	7805	Bivalvia
Early Pleistocene	2.588	0.781	Hendy et a	2008	19909	Mollusca	7805	Bivalvia
Early Pleistocene	2.588	0.781	Hendy et a	2008	19909	Mollusca	7805	Bivalvia
Pleistocene	2.588	0.0117	Hendy et a	2008	19909	Mollusca	7805	Bivalvia
Late Pleistocene	0.126	0.0117	Spencer ar	1987	15525	Mollusca	7805	Bivalvia
Late Pleistocene	0.126	0.0117	Spencer ar	1987	15525	Mollusca	7805	Bivalvia
Late Pleistocene	0.126	0.0117	Spencer ar	1987	15525	Mollusca	7805	Bivalvia
Late Pleistocene	0.126	0.0117	Spencer ar	1987	15525	Mollusca	7805	Bivalvia
Late Pleistocene	0.126	0.0117	Spencer ar	1987	15525	Mollusca	7805	Bivalvia
Late Pleistocene	0.126	0.0117	Spencer ar	1987	15525	Mollusca	7805	Bivalvia
Late Pliocene	3.6	2.588	Edwards et	2005	17631	Mollusca	7805	Bivalvia
Late Pliocene	3.6	2.588	Edwards et	2005	17631	Mollusca	7805	Bivalvia
Late Pliocene	3.6	2.588	Edwards et	2005	17631	Mollusca	7805	Bivalvia
Late Pliocene	3.6	2.588	Edwards et	2005	17631	Mollusca	7805	Bivalvia
Late Pliocene	3.6	2.588	Ward	1993	26351	Mollusca	7805	Bivalvia
Late Pliocene	3.6	2.588	Ward	1993	26351	Mollusca	7805	Bivalvia
Late Pliocene	3.6	2.588	Ward	1993	26351	Mollusca	7805	Bivalvia
Late Pliocene	3.6	2.588	Ward	1993	26351	Mollusca	7805	Bivalvia
Late Pliocene	3.6	2.588	Ward	1993	26351	Mollusca	7805	Bivalvia
Late Pliocene	3.6	2.588	Ward	1993	26351	Mollusca	7805	Bivalvia
Late Pliocene	3.6	2.588	Ward	1993	26351	Mollusca	7805	Bivalvia
Late Pliocene	3.6	2.588	Hendy et a	2008	19909	Mollusca	7805	Bivalvia
Late Pliocene	3.6	2.588	Hendy et a	2008	19909	Mollusca	7805	Bivalvia
Late Pleistocene	0.126	0.0117	Hendy et a	2008	19909	Mollusca	7805	Bivalvia
Late Pleistocene	0.126	0.0117	Hendy et a	2008	19909	Mollusca	7805	Bivalvia
Late Pliocene	3.6	2.588	Hendy et a	2008	19909	Mollusca	7805	Bivalvia
Late Pliocene	3.6	2.588	Hendy et a	2008	19909	Mollusca	7805	Bivalvia
Late Pleistocene	0.126	0.0117	Hendy et a	2008	19909	Mollusca	7805	Bivalvia
Early Pleistocene	2.588	0.781	Hendy et a	2008	19909	Mollusca	7805	Bivalvia
Late Pliocene	3.6	2.588	Cooke	1936	31145	Mollusca	7805	Bivalvia
Late Pliocene	3.6	2.588	Gardner	1943	16984	Mollusca	7805	Bivalvia
Early Pliocene	5.333	3.6	Ward	1992	16985	Mollusca	7805	Bivalvia
Pliocene	5.333	2.588	Gardner	1943	16984	Mollusca	7805	Bivalvia
Zanclean	5.333	3.6	Ward	1992	16985	Mollusca	7805	Bivalvia

Zanclean	5.333	3.6	Ward	1992	16985	Mollusca	7805	Bivalvia
Late Pliocene	3.6	2.588	Ward	1993	26351	Mollusca	7805	Bivalvia
Late Pliocene	3.6	2.588	Ward	1993	26351	Mollusca	7805	Bivalvia
Late Pliocene	3.6	2.588	Ward	1993	26351	Mollusca	7805	Bivalvia
Late Pliocene	3.6	2.588	Ward	1993	26351	Mollusca	7805	Bivalvia
Late Pliocene	3.6	2.588	Hendy et a	2008	19909	Mollusca	7805	Bivalvia
Late Pliocene	3.6	2.588	Hendy et a	2008	19909	Mollusca	7805	Bivalvia
Late Pliocene	3.6	2.588	Hendy et a	2008	19909	Mollusca	7805	Bivalvia
Early Pleistocene	2.588	0.781	Mansfield	1932	11083	Mollusca	7805	Bivalvia
Late Pliocene	3.6	2.588	Mansfield	1939	19112	Mollusca	7805	Bivalvia
Late Pliocene	3.6	2.588	Mansfield	1939	19112	Mollusca	7805	Bivalvia
Middle Pleistocene	0.781	0.126	Hoerle	1970	19536	Mollusca	7805	Bivalvia
Late Pliocene	3.6	2.588	DuBar	1958	25514	Mollusca	7805	Bivalvia
Zanclean	5.333	3.6	Campbell a	1995	17558	Mollusca	7805	Bivalvia
Zanclean	5.333	3.6	Campbell a	1995	17558	Mollusca	7805	Bivalvia
Late Pliocene	3.6	2.588	Ward and I	1987	16700	Mollusca	7805	Bivalvia
Late Pliocene	3.6	2.588	Ward and I	1987	16700	Mollusca	7805	Bivalvia
Early Pleistocene	2.588	0.781	Ward and I	1987	16700	Mollusca	7805	Bivalvia
Late Pliocene	3.6	2.588	Gardner	1943	16984	Mollusca	7805	Bivalvia
Early Pleistocene	2.588	0.781	Gardner	1943	16984	Mollusca	7805	Bivalvia
Late Pliocene	3.6	2.588	Hendy et a	2008	19909	Mollusca	7805	Bivalvia
Late Pliocene	3.6	2.588	Hendy et a	2008	19909	Mollusca	7805	Bivalvia
Middle Pleistocene	0.781	0.126	McGinty	1970	19114	Mollusca	7805	Bivalvia
Late Pleistocene	0.126	0.0117	Hendy et a	2008	19909	Mollusca	7805	Bivalvia
Middle Pleistocene	0.781	0.126	Hoerle	1970	19536	Mollusca	7805	Bivalvia
Early Pleistocene	2.588	0.781	DuBar	1962	11119	Mollusca	7805	Bivalvia
Calabrian	1.806	0.781	DuBar	1962	11119	Mollusca	7805	Bivalvia
Pliocene Pleistocene	5.333	0.0117	DuBar	1962	11119	Mollusca	7805	Bivalvia
Late Pliocene	3.6	2.588	Mansfield	1932	17094	Mollusca	7805	Bivalvia
Late Pliocene	3.6	2.588	Mansfield	1932	17094	Mollusca	7805	Bivalvia
Late Pliocene	3.6	2.588	Mansfield	1932	17094	Mollusca	7805	Bivalvia
Late Pliocene	3.6	2.588	Mansfield	1932	17094	Mollusca	7805	Bivalvia
Early Pleistocene	2.588	0.781	Portell et a	1992	17634	Mollusca	7805	Bivalvia
Late Pleistocene	0.126	0.0117	Portell et a	1992	17634	Mollusca	7805	Bivalvia
Middle Pleistocene	0.781	0.126	McGinty	1970	19114	Mollusca	7805	Bivalvia
Middle Pleistocene	0.781	0.126	McGinty	1970	19114	Mollusca	7805	Bivalvia
Middle Pleistocene	0.781	0.126	McGinty	1970	19114	Mollusca	7805	Bivalvia
Late Pleistocene	0.126	0.0117	Hendy et a	2008	19909	Mollusca	7805	Bivalvia
Late Pleistocene	0.126	0.0117	Hendy et a	2008	19909	Mollusca	7805	Bivalvia
Early Pleistocene	2.588	0.781	Olsson anc	1953	25463	Mollusca	7805	Bivalvia
Early Pleistocene	2.588	0.781	Olsson anc	1953	25463	Mollusca	7805	Bivalvia
Early Pleistocene	2.588	0.781	Hendy et a	2008	19909	Mollusca	7805	Bivalvia
Early Pleistocene	2.588	0.781	Hendy et a	2008	19909	Mollusca	7805	Bivalvia
Early Pliocene	5.333	3.6	Petuch anc	2007	26813	Mollusca	7805	Bivalvia
Pleistocene	2.588	0.0117	Hendy et a	2008	19909	Mollusca	7805	Bivalvia
Late Pliocene	3.6	2.588	Smith	1941	32333	Mollusca	7805	Bivalvia
Late Pleistocene	0.126	0.0117	Kittle and I	2010	40884	Mollusca	7805	Bivalvia

Late Pleistocene	0.126	0.0117	Kittle and I	2010	40884	Mollusca	7805	Bivalvia
Late Pliocene	3.6	2.588	Ketcher an	1993	58350	Mollusca	7805	Bivalvia
Zanclean	5.333	3.6	Campbell a	1995	17558	Mollusca	7805	Bivalvia
Early Pleistocene	2.588	0.781	DuBar	1971	17556	Mollusca	7805	Bivalvia
Late Pleistocene	0.126	0.0117	Richards	1962	18974	Mollusca	7805	Bivalvia
Late Pleistocene	0.126	0.0117	Richards	1962	18974	Mollusca	7805	Bivalvia
Late Pleistocene	0.126	0.0117	Richards	1962	18974	Mollusca	7805	Bivalvia
Late Pleistocene	0.126	0.0117	Hendy et a	2008	19909	Mollusca	7805	Bivalvia
Late Pleistocene	0.126	0.0117	Hendy et a	2008	19909	Mollusca	7805	Bivalvia
Late Pleistocene	0.126	0.0117	Hendy et a	2008	19909	Mollusca	7805	Bivalvia
Late Pleistocene	0.126	0.0117	Hendy et a	2008	19909	Mollusca	7805	Bivalvia
Late Pleistocene	0.126	0.0117	Hendy et a	2008	19909	Mollusca	7805	Bivalvia
Pleistocene	2.588	0.0117	Hendy et a	2008	19909	Mollusca	7805	Bivalvia
Late Pliocene	3.6	2.588	Cooke	1936	31145	Mollusca	7805	Bivalvia
Late Pliocene	3.6	2.588	Cooke	1936	31145	Mollusca	7805	Bivalvia
Late Pliocene	3.6	2.588	Ward and I	1987	16700	Mollusca	7805	Bivalvia
Late Pliocene	3.6	2.588	Ward and I	1987	16700	Mollusca	7805	Bivalvia
Early Pleistocene	2.588	0.781	Ward and I	1987	16700	Mollusca	7805	Bivalvia
Early Pleistocene	2.588	0.781	Ward and I	1987	16700	Mollusca	7805	Bivalvia
Early Pleistocene	2.588	0.781	Ward and I	1987	16700	Mollusca	7805	Bivalvia
Early Pleistocene	2.588	0.781	Ward and I	1987	16700	Mollusca	7805	Bivalvia
Early Pleistocene	2.588	0.781	Gardner	1943	16984	Mollusca	7805	Bivalvia
Pleistocene	2.588	0.0117	Gardner	1943	16984	Mollusca	7805	Bivalvia
Late Pliocene	3.6	2.588	Gardner	1943	16984	Mollusca	7805	Bivalvia
Late Pliocene	3.6	2.588	Gardner	1943	16984	Mollusca	7805	Bivalvia
Early Pleistocene	2.588	0.781	Gardner	1943	16984	Mollusca	7805	Bivalvia
Early Pleistocene	2.588	0.781	Miller	1990	17561	Mollusca	7805	Bivalvia
Late Pleistocene	0.126	0.0117	Richards	1962	18974	Mollusca	7805	Bivalvia
Late Pleistocene	0.126	0.0117	Richards	1962	18974	Mollusca	7805	Bivalvia
Early Pleistocene	2.588	0.781	Hendy et a	2008	19909	Mollusca	7805	Bivalvia
Late Pleistocene	0.126	0.0117	Hendy et a	2008	19909	Mollusca	7805	Bivalvia
Late Pliocene	3.6	2.588	Hendy et a	2008	19909	Mollusca	7805	Bivalvia
Late Pliocene	3.6	2.588	Hendy et a	2008	19909	Mollusca	7805	Bivalvia
Early Pleistocene	2.588	0.781	Hendy et a	2008	19909	Mollusca	7805	Bivalvia
Early Pleistocene	2.588	0.781	Hendy et a	2008	19909	Mollusca	7805	Bivalvia
Early Pleistocene	2.588	0.781	Hendy et a	2008	19909	Mollusca	7805	Bivalvia
Early Pleistocene	2.588	0.781	Hendy et a	2008	19909	Mollusca	7805	Bivalvia
Early Pleistocene	2.588	0.781	Hendy et a	2008	19909	Mollusca	7805	Bivalvia
Early Pleistocene	2.588	0.781	Hendy et a	2008	19909	Mollusca	7805	Bivalvia
Early Pleistocene	2.588	0.781	Hendy et a	2008	19909	Mollusca	7805	Bivalvia
Late Pleistocene	0.126	0.0117	Spencer ar	1987	15525	Mollusca	7805	Bivalvia
Late Pliocene	3.6	2.588	Ward	1993	26351	Mollusca	7805	Bivalvia
Late Pliocene	3.6	2.588	Ward	1993	26351	Mollusca	7805	Bivalvia
Late Pliocene	3.6	2.588	Ward	1993	26351	Mollusca	7805	Bivalvia
Late Pliocene	3.6	2.588	Hendy et a	2008	19909	Mollusca	7805	Bivalvia
Late Pliocene	3.6	2.588	Hendy et a	2008	19909	Mollusca	7805	Bivalvia
Early Pleistocene	2.588	0.781	Hendy et a	2008	19909	Mollusca	7805	Bivalvia

Late Pleistocene	0.126	0.0117	Sellards	1917	9011	Mollusca	7805	Bivalvia
Late Pleistocene	0.126	0.0117	Sellards	1917	9011	Mollusca	7805	Bivalvia
Late Pleistocene	0.126	0.0117	Sellards	1917	9011	Mollusca	7805	Bivalvia
Late Pleistocene	0.126	0.0117	Sellards	1917	9011	Mollusca	7805	Bivalvia
Late Pleistocene	0.126	0.0117	Sellards	1917	9011	Mollusca	7805	Bivalvia
Late Pleistocene	0.126	0.0117	Sellards	1917	9011	Mollusca	7805	Bivalvia
Late Pleistocene	0.126	0.0117	Sellards	1917	9011	Mollusca	7805	Bivalvia
Late Pleistocene	0.126	0.0117	Sellards	1917	9011	Mollusca	7805	Bivalvia
Late Pleistocene	0.126	0.0117	Sellards	1917	9011	Mollusca	7805	Bivalvia
Late Pleistocene	0.126	0.0117	Sellards	1917	9011	Mollusca	7805	Bivalvia
Late Pleistocene	0.126	0.0117	Sellards	1917	9011	Mollusca	7805	Bivalvia
Late Pleistocene	0.126	0.0117	Sellards	1917	9011	Mollusca	7805	Bivalvia
Early Pleistocene	2.588	0.781	Mansfield	1932	11083	Mollusca	7805	Bivalvia
Early Pleistocene	2.588	0.781	Mansfield	1932	11083	Mollusca	7805	Bivalvia
Early Pleistocene	2.588	0.781	Mansfield	1932	11083	Mollusca	7805	Bivalvia
Early Pleistocene	2.588	0.781	Mansfield	1932	11083	Mollusca	7805	Bivalvia
Early Pleistocene	2.588	0.781	Mansfield	1932	11083	Mollusca	7805	Bivalvia
Early Pleistocene	2.588	0.781	Mansfield	1932	11083	Mollusca	7805	Bivalvia
Early Pleistocene	2.588	0.781	Mansfield	1932	11083	Mollusca	7805	Bivalvia
Early Pliocene	5.333	3.6	DuBar	1962	11119	Mollusca	7805	Bivalvia
Early Pleistocene	2.588	0.781	DuBar	1962	11119	Mollusca	7805	Bivalvia
Early Pleistocene	2.588	0.781	DuBar	1962	11119	Mollusca	7805	Bivalvia
Early Pleistocene	2.588	0.781	DuBar	1962	11119	Mollusca	7805	Bivalvia
Early Pleistocene	2.588	0.781	DuBar	1962	11119	Mollusca	7805	Bivalvia
Early Pleistocene	2.588	0.781	DuBar	1962	11119	Mollusca	7805	Bivalvia
Early Pleistocene	2.588	0.781	DuBar	1962	11119	Mollusca	7805	Bivalvia
Early Pleistocene	2.588	0.781	DuBar	1962	11119	Mollusca	7805	Bivalvia
Early Pleistocene	2.588	0.781	DuBar	1962	11119	Mollusca	7805	Bivalvia
Early Pleistocene	2.588	0.781	DuBar	1962	11119	Mollusca	7805	Bivalvia
Early Pleistocene	2.588	0.781	DuBar	1962	11119	Mollusca	7805	Bivalvia
Early Pleistocene	2.588	0.781	DuBar	1962	11119	Mollusca	7805	Bivalvia
Early Pleistocene	2.588	0.781	DuBar	1962	11119	Mollusca	7805	Bivalvia
Early Pleistocene	2.588	0.781	DuBar	1962	11119	Mollusca	7805	Bivalvia
Early Pleistocene	2.588	0.781	DuBar	1962	11119	Mollusca	7805	Bivalvia
Early Pleistocene	2.588	0.781	DuBar	1962	11119	Mollusca	7805	Bivalvia
Early Pleistocene	2.588	0.781	DuBar	1962	11119	Mollusca	7805	Bivalvia
Early Pleistocene	2.588	0.781	DuBar	1962	11119	Mollusca	7805	Bivalvia
Early Pleistocene	2.588	0.781	DuBar	1962	11119	Mollusca	7805	Bivalvia
Early Pleistocene	2.588	0.781	DuBar	1962	11119	Mollusca	7805	Bivalvia
Early Pleistocene	2.588	0.781	DuBar	1962	11119	Mollusca	7805	Bivalvia
Early Pleistocene	2.588	0.781	DuBar	1962	11119	Mollusca	7805	Bivalvia
Early Pleistocene	2.588	0.781	DuBar	1962	11119	Mollusca	7805	Bivalvia
Early Pleistocene	2.588	0.781	DuBar	1962	11119	Mollusca	7805	Bivalvia
Early Pleistocene	2.588	0.781	DuBar	1962	11119	Mollusca	7805	Bivalvia
Early Pleistocene	2.588	0.781	DuBar	1962	11119	Mollusca	7805	Bivalvia
Early Pleistocene	2.588	0.781	DuBar	1962	11119	Mollusca	7805	Bivalvia
Calabrian	1.806	0.781	DuBar	1962	11119	Mollusca	7805	Bivalvia

Calabrian		1.806	0.781	DuBar	1962	11119	Mollusca	7805	Bivalvia
Calabrian		1.806	0.781	DuBar	1962	11119	Mollusca	7805	Bivalvia
Calabrian		1.806	0.781	DuBar	1962	11119	Mollusca	7805	Bivalvia
Calabrian		1.806	0.781	DuBar	1962	11119	Mollusca	7805	Bivalvia
Calabrian		1.806	0.781	DuBar	1962	11119	Mollusca	7805	Bivalvia
Calabrian		1.806	0.781	DuBar	1962	11119	Mollusca	7805	Bivalvia
Calabrian		1.806	0.781	DuBar	1962	11119	Mollusca	7805	Bivalvia
Calabrian		1.806	0.781	DuBar	1962	11119	Mollusca	7805	Bivalvia
Calabrian		1.806	0.781	DuBar	1962	11119	Mollusca	7805	Bivalvia
Calabrian		1.806	0.781	DuBar	1962	11119	Mollusca	7805	Bivalvia
Calabrian		1.806	0.781	DuBar	1962	11119	Mollusca	7805	Bivalvia
Late Pleistocene		0.126	0.0117	DuBar	1962	11119	Mollusca	7805	Bivalvia
Early Pleistocene		2.588	0.781	DuBar	1962	11119	Mollusca	7805	Bivalvia
Early Pleistocene		2.588	0.781	DuBar	1962	11119	Mollusca	7805	Bivalvia
Early Pleistocene		2.588	0.781	DuBar	1962	11119	Mollusca	7805	Bivalvia
Early Pleistocene		2.588	0.781	DuBar	1962	11119	Mollusca	7805	Bivalvia
Early Pleistocene		2.588	0.781	DuBar	1962	11119	Mollusca	7805	Bivalvia
Early Pleistocene		2.588	0.781	DuBar	1962	11119	Mollusca	7805	Bivalvia
Early Pleistocene		2.588	0.781	DuBar	1962	11119	Mollusca	7805	Bivalvia
Early Pleistocene		2.588	0.781	DuBar	1962	11119	Mollusca	7805	Bivalvia
Early Pleistocene		2.588	0.781	DuBar	1962	11119	Mollusca	7805	Bivalvia
Late Pleistocene		0.126	0.0117	DuBar	1962	11119	Mollusca	7805	Bivalvia
Late Pleistocene		0.126	0.0117	DuBar	1962	11119	Mollusca	7805	Bivalvia
Late Pleistocene		0.126	0.0117	DuBar	1962	11119	Mollusca	7805	Bivalvia
Late Pleistocene		0.126	0.0117	DuBar	1962	11119	Mollusca	7805	Bivalvia
Late Pleistocene		0.126	0.0117	DuBar	1962	11119	Mollusca	7805	Bivalvia
Late Pleistocene		0.126	0.0117	DuBar	1962	11119	Mollusca	7805	Bivalvia
Late Pleistocene		0.126	0.0117	DuBar	1962	11119	Mollusca	7805	Bivalvia
Pliocene	Pleistocen	5.333	0.0117	DuBar	1962	11119	Mollusca	7805	Bivalvia
Pliocene	Pleistocen	5.333	0.0117	DuBar	1962	11119	Mollusca	7805	Bivalvia
Pliocene	Pleistocen	5.333	0.0117	DuBar	1962	11119	Mollusca	7805	Bivalvia
Pliocene	Pleistocen	5.333	0.0117	DuBar	1962	11119	Mollusca	7805	Bivalvia
Pliocene	Pleistocen	5.333	0.0117	DuBar	1962	11119	Mollusca	7805	Bivalvia
Pliocene	Pleistocen	5.333	0.0117	DuBar	1962	11119	Mollusca	7805	Bivalvia
Pliocene	Pleistocen	5.333	0.0117	DuBar	1962	11119	Mollusca	7805	Bivalvia
Pliocene	Pleistocen	5.333	0.0117	DuBar	1962	11119	Mollusca	7805	Bivalvia
Pliocene	Pleistocen	5.333	0.0117	DuBar	1962	11119	Mollusca	7805	Bivalvia
Pliocene	Pleistocen	5.333	0.0117	DuBar	1962	11119	Mollusca	7805	Bivalvia
Pliocene	Pleistocen	5.333	0.0117	DuBar	1962	11119	Mollusca	7805	Bivalvia
Irvingtonian		1.8	0.3	Hulbert an	1989	1960	Mollusca	7805	Bivalvia
Late Pliocene		3.6	2.588	Mansfield	1932	17094	Mollusca	7805	Bivalvia
Late Pliocene		3.6	2.588	Mansfield	1932	17094	Mollusca	7805	Bivalvia
Late Pliocene		3.6	2.588	Mansfield	1932	17094	Mollusca	7805	Bivalvia
Late Pliocene		3.6	2.588	Mansfield	1932	17094	Mollusca	7805	Bivalvia
Late Pliocene		3.6	2.588	Mansfield	1932	17094	Mollusca	7805	Bivalvia
Late Pliocene		3.6	2.588	Mansfield	1932	17094	Mollusca	7805	Bivalvia
Late Pliocene		3.6	2.588	Mansfield	1932	17094	Mollusca	7805	Bivalvia

Late Pliocene	3.6	2.588	Mansfield	1932	17094	Mollusca	7805	Bivalvia
Late Pliocene	3.6	2.588	Mansfield	1932	17094	Mollusca	7805	Bivalvia
Late Pliocene	3.6	2.588	Mansfield	1932	17094	Mollusca	7805	Bivalvia
Late Pliocene	3.6	2.588	Mansfield	1932	17094	Mollusca	7805	Bivalvia
Late Pliocene	3.6	2.588	Mansfield	1932	17094	Mollusca	7805	Bivalvia
Late Pliocene	3.6	2.588	Mansfield	1932	17094	Mollusca	7805	Bivalvia
Late Pliocene	3.6	2.588	Mansfield	1932	17094	Mollusca	7805	Bivalvia
Late Pliocene	3.6	2.588	Mansfield	1932	17094	Mollusca	7805	Bivalvia
Late Pliocene	3.6	2.588	Mansfield	1932	17094	Mollusca	7805	Bivalvia
Late Pliocene	3.6	2.588	Mansfield	1932	17094	Mollusca	7805	Bivalvia
Late Pliocene	3.6	2.588	Mansfield	1932	17094	Mollusca	7805	Bivalvia
Late Pliocene	3.6	2.588	Mansfield	1932	17094	Mollusca	7805	Bivalvia
Late Pliocene	3.6	2.588	Mansfield	1932	17094	Mollusca	7805	Bivalvia
Late Pliocene	3.6	2.588	Mansfield	1932	17094	Mollusca	7805	Bivalvia
Late Pliocene	3.6	2.588	Mansfield	1932	17094	Mollusca	7805	Bivalvia
Late Pliocene	3.6	2.588	Mansfield	1932	17094	Mollusca	7805	Bivalvia
Late Pliocene	3.6	2.588	Mansfield	1932	17094	Mollusca	7805	Bivalvia
Late Pliocene	3.6	2.588	Mansfield	1932	17094	Mollusca	7805	Bivalvia
Late Pliocene	3.6	2.588	Mansfield	1932	17094	Mollusca	7805	Bivalvia
Late Pliocene	3.6	2.588	Mansfield	1932	17094	Mollusca	7805	Bivalvia
Late Pliocene	3.6	2.588	Mansfield	1932	17094	Mollusca	7805	Bivalvia
Late Pliocene	3.6	2.588	Mansfield	1932	17094	Mollusca	7805	Bivalvia
Late Pliocene	3.6	2.588	Mansfield	1932	17094	Mollusca	7805	Bivalvia
Late Pliocene	3.6	2.588	Mansfield	1932	17094	Mollusca	7805	Bivalvia
Late Pliocene	3.6	2.588	Mansfield	1932	17094	Mollusca	7805	Bivalvia
Late Pliocene	3.6	2.588	Mansfield	1932	17094	Mollusca	7805	Bivalvia
Late Pliocene	3.6	2.588	Mansfield	1932	17094	Mollusca	7805	Bivalvia
Late Pliocene	3.6	2.588	Mansfield	1932	17094	Mollusca	7805	Bivalvia
Early Pleistocene	2.588	0.781	Portell et a	1992	17634	Mollusca	7805	Bivalvia
Early Pleistocene	2.588	0.781	Portell et a	1992	17634	Mollusca	7805	Bivalvia
Early Pleistocene	2.588	0.781	Portell et a	1992	17634	Mollusca	7805	Bivalvia
Early Pleistocene	2.588	0.781	Portell et a	1992	17634	Mollusca	7805	Bivalvia
Early Pleistocene	2.588	0.781	Portell et a	1992	17634	Mollusca	7805	Bivalvia
Early Pleistocene	2.588	0.781	Portell et a	1992	17634	Mollusca	7805	Bivalvia
Early Pleistocene	2.588	0.781	Portell et a	1992	17634	Mollusca	7805	Bivalvia
Early Pleistocene	2.588	0.781	Portell et a	1992	17634	Mollusca	7805	Bivalvia
Early Pleistocene	2.588	0.781	Portell et a	1992	17634	Mollusca	7805	Bivalvia
Early Pleistocene	2.588	0.781	Portell et a	1992	17634	Mollusca	7805	Bivalvia
Middle Pleistocene	0.781	0.126	Portell et a	1992	17634	Mollusca	7805	Bivalvia
Middle Pleistocene	0.781	0.126	Portell et a	1992	17634	Mollusca	7805	Bivalvia
Middle Pleistocene	0.781	0.126	Portell et a	1992	17634	Mollusca	7805	Bivalvia
Middle Pleistocene	0.781	0.126	Portell et a	1992	17634	Mollusca	7805	Bivalvia
Middle Pleistocene	0.781	0.126	Portell et a	1992	17634	Mollusca	7805	Bivalvia
Middle Pleistocene	0.781	0.126	Portell et a	1992	17634	Mollusca	7805	Bivalvia
Middle Pleistocene	0.781	0.126	Portell et a	1992	17634	Mollusca	7805	Bivalvia
Middle Pleistocene	0.781	0.126	Portell et a	1992	17634	Mollusca	7805	Bivalvia
Middle Pleistocene	0.781	0.126	Portell et a	1992	17634	Mollusca	7805	Bivalvia

Middle Pleistocene	0.781	0.126	Portell et a	1992	17634	Mollusca	7805	Bivalvia
Late Pleistocene	0.126	0.0117	Portell et a	1992	17634	Mollusca	7805	Bivalvia
Late Pleistocene	0.126	0.0117	Portell et a	1992	17634	Mollusca	7805	Bivalvia
Late Pleistocene	0.126	0.0117	Portell et a	1992	17634	Mollusca	7805	Bivalvia
Late Pleistocene	0.126	0.0117	Portell et a	1992	17634	Mollusca	7805	Bivalvia
Late Pleistocene	0.126	0.0117	Portell et a	1992	17634	Mollusca	7805	Bivalvia
Late Pleistocene	0.126	0.0117	Portell et a	1992	17634	Mollusca	7805	Bivalvia
Late Pleistocene	0.126	0.0117	Portell et a	1992	17634	Mollusca	7805	Bivalvia
Late Pleistocene	0.126	0.0117	Portell et a	1992	17634	Mollusca	7805	Bivalvia
Zanclean Piacenzian	5.333	2.588	Wilson	1983	17889	Mollusca	7805	Bivalvia
Zanclean Piacenzian	5.333	2.588	Wilson	1983	17889	Mollusca	7805	Bivalvia
Middle Pleistocene	0.781	0.126	McGinty	1970	19114	Mollusca	7805	Bivalvia
Middle Pleistocene	0.781	0.126	McGinty	1970	19114	Mollusca	7805	Bivalvia
Middle Pleistocene	0.781	0.126	McGinty	1970	19114	Mollusca	7805	Bivalvia
Middle Pleistocene	0.781	0.126	McGinty	1970	19114	Mollusca	7805	Bivalvia
Middle Pleistocene	0.781	0.126	McGinty	1970	19114	Mollusca	7805	Bivalvia
Middle Pleistocene	0.781	0.126	McGinty	1970	19114	Mollusca	7805	Bivalvia
Middle Pleistocene	0.781	0.126	McGinty	1970	19114	Mollusca	7805	Bivalvia
Middle Pleistocene	0.781	0.126	McGinty	1970	19114	Mollusca	7805	Bivalvia
Middle Pleistocene	0.781	0.126	McGinty	1970	19114	Mollusca	7805	Bivalvia
Middle Pleistocene	0.781	0.126	McGinty	1970	19114	Mollusca	7805	Bivalvia
Middle Pleistocene	0.781	0.126	McGinty	1970	19114	Mollusca	7805	Bivalvia
Middle Pleistocene	0.781	0.126	McGinty	1970	19114	Mollusca	7805	Bivalvia
Middle Pleistocene	0.781	0.126	McGinty	1970	19114	Mollusca	7805	Bivalvia
Middle Pleistocene	0.781	0.126	McGinty	1970	19114	Mollusca	7805	Bivalvia
Middle Pleistocene	0.781	0.126	McGinty	1970	19114	Mollusca	7805	Bivalvia
Middle Pleistocene	0.781	0.126	McGinty	1970	19114	Mollusca	7805	Bivalvia
Middle Pleistocene	0.781	0.126	McGinty	1970	19114	Mollusca	7805	Bivalvia
Middle Pleistocene	0.781	0.126	Hoerle	1970	19536	Mollusca	7805	Bivalvia
Late Pliocene	3.6	2.588	Mansfield	1939	19112	Mollusca	7805	Bivalvia
Late Pliocene	3.6	2.588	Mansfield	1939	19112	Mollusca	7805	Bivalvia
Late Pliocene	3.6	2.588	Mansfield	1939	19112	Mollusca	7805	Bivalvia
Late Pliocene	3.6	2.588	Mansfield	1939	19112	Mollusca	7805	Bivalvia
Late Pliocene	3.6	2.588	Mansfield	1939	19112	Mollusca	7805	Bivalvia
Late Pliocene	3.6	2.588	Mansfield	1939	19112	Mollusca	7805	Bivalvia
Late Pliocene	3.6	2.588	Mansfield	1939	19112	Mollusca	7805	Bivalvia
Late Pleistocene	0.126	0.0117	Hendy et a	2008	19909	Mollusca	7805	Bivalvia
Late Pleistocene	0.126	0.0117	Hendy et a	2008	19909	Mollusca	7805	Bivalvia
Late Pleistocene	0.126	0.0117	Hendy et a	2008	19909	Mollusca	7805	Bivalvia
Late Pleistocene	0.126	0.0117	Hendy et a	2008	19909	Mollusca	7805	Bivalvia
Late Pleistocene	0.126	0.0117	Hendy et a	2008	19909	Mollusca	7805	Bivalvia
Late Pleistocene	0.126	0.0117	Hendy et a	2008	19909	Mollusca	7805	Bivalvia
Late Pleistocene	0.126	0.0117	Hendy et a	2008	19909	Mollusca	7805	Bivalvia
Late Pleistocene	0.126	0.0117	Hendy et a	2008	19909	Mollusca	7805	Bivalvia
Late Pleistocene	0.126	0.0117	Hendy et a	2008	19909	Mollusca	7805	Bivalvia
Late Pleistocene	0.126	0.0117	Hendy et a	2008	19909	Mollusca	7805	Bivalvia
Late Pleistocene	0.126	0.0117	Hendy et a	2008	19909	Mollusca	7805	Bivalvia

Late Pleistocene	0.126	0.0117	Hendy et a	2008	19909	Mollusca	7805	Bivalvia
Late Pleistocene	0.126	0.0117	Hendy et a	2008	19909	Mollusca	7805	Bivalvia
Late Pleistocene	0.126	0.0117	Hendy et a	2008	19909	Mollusca	7805	Bivalvia
Late Pleistocene	0.126	0.0117	Hendy et a	2008	19909	Mollusca	7805	Bivalvia
Late Pleistocene	0.126	0.0117	Hendy et a	2008	19909	Mollusca	7805	Bivalvia
Late Pleistocene	0.126	0.0117	Hendy et a	2008	19909	Mollusca	7805	Bivalvia
Late Pleistocene	0.126	0.0117	Hendy et a	2008	19909	Mollusca	7805	Bivalvia
Early Pleistocene	2.588	0.781	Schroeder	1954	25303	Mollusca	7805	Bivalvia
Early Pleistocene	2.588	0.781	Schroeder	1954	25303	Mollusca	7805	Bivalvia
Early Pleistocene	2.588	0.781	Schroeder	1954	25303	Mollusca	7805	Bivalvia
Pliocene	5.333	2.588	Schroeder	1954	25303	Mollusca	7805	Bivalvia
Pliocene	5.333	2.588	Schroeder	1954	25303	Mollusca	7805	Bivalvia
Early Pleistocene	2.588	0.781	Schroeder	1954	25303	Mollusca	7805	Bivalvia
Early Pleistocene	2.588	0.781	Schroeder	1954	25303	Mollusca	7805	Bivalvia
Early Pleistocene	2.588	0.781	Schroeder	1954	25303	Mollusca	7805	Bivalvia
Early Pleistocene	2.588	0.781	Schroeder	1954	25303	Mollusca	7805	Bivalvia
Early Pleistocene	2.588	0.781	Schroeder	1954	25303	Mollusca	7805	Bivalvia
Pliocene	5.333	2.588	Schroeder	1954	25303	Mollusca	7805	Bivalvia
Pliocene	5.333	2.588	Schroeder	1954	25303	Mollusca	7805	Bivalvia
Pliocene	5.333	2.588	Schroeder	1954	25303	Mollusca	7805	Bivalvia
Pliocene	5.333	2.588	Schroeder	1954	25303	Mollusca	7805	Bivalvia
Pliocene	5.333	2.588	Schroeder	1954	25303	Mollusca	7805	Bivalvia
Pliocene	5.333	2.588	Schroeder	1954	25303	Mollusca	7805	Bivalvia
Pliocene	5.333	2.588	Schroeder	1954	25303	Mollusca	7805	Bivalvia
Pliocene	5.333	2.588	Schroeder	1954	25303	Mollusca	7805	Bivalvia
Pliocene	5.333	2.588	Schroeder	1954	25303	Mollusca	7805	Bivalvia
Pliocene	5.333	2.588	Schroeder	1954	25303	Mollusca	7805	Bivalvia
Early Pleistocene	2.588	0.781	Schroeder	1954	25303	Mollusca	7805	Bivalvia
Early Pleistocene	2.588	0.781	Schroeder	1954	25303	Mollusca	7805	Bivalvia
Early Pleistocene	2.588	0.781	Schroeder	1954	25303	Mollusca	7805	Bivalvia
Early Pleistocene	2.588	0.781	Schroeder	1954	25303	Mollusca	7805	Bivalvia
Early Pleistocene	2.588	0.781	Schroeder	1954	25303	Mollusca	7805	Bivalvia
Early Pleistocene	2.588	0.781	Schroeder	1954	25303	Mollusca	7805	Bivalvia
Pliocene	5.333	2.588	Schroeder	1954	25303	Mollusca	7805	Bivalvia
Pliocene	5.333	2.588	Schroeder	1954	25303	Mollusca	7805	Bivalvia
Pliocene	5.333	2.588	Schroeder	1954	25303	Mollusca	7805	Bivalvia
Pliocene	5.333	2.588	Schroeder	1954	25303	Mollusca	7805	Bivalvia
Pliocene	5.333	2.588	Schroeder	1954	25303	Mollusca	7805	Bivalvia
Pliocene	5.333	2.588	Schroeder	1954	25303	Mollusca	7805	Bivalvia
Pliocene	5.333	2.588	Schroeder	1954	25303	Mollusca	7805	Bivalvia
Pliocene	5.333	2.588	Schroeder	1954	25303	Mollusca	7805	Bivalvia
Pliocene	5.333	2.588	Schroeder	1954	25303	Mollusca	7805	Bivalvia
Pliocene	5.333	2.588	Schroeder	1954	25303	Mollusca	7805	Bivalvia
Early Pleistocene	2.588	0.781	Olsson anc	1953	25463	Mollusca	7805	Bivalvia
Early Pleistocene	2.588	0.781	Olsson anc	1953	25463	Mollusca	7805	Bivalvia
Early Pleistocene	2.588	0.781	Olsson anc	1953	25463	Mollusca	7805	Bivalvia
Early Pleistocene	2.588	0.781	Olsson anc	1953	25463	Mollusca	7805	Bivalvia

Early Pleistocene	2.588	0.781	Olsson anc	1953	25463	Mollusca	7805	Bivalvia
Early Pleistocene	2.588	0.781	Olsson anc	1953	25463	Mollusca	7805	Bivalvia
Early Pleistocene	2.588	0.781	Olsson anc	1953	25463	Mollusca	7805	Bivalvia
Early Pleistocene	2.588	0.781	Olsson anc	1953	25463	Mollusca	7805	Bivalvia
Early Pleistocene	2.588	0.781	Olsson anc	1953	25463	Mollusca	7805	Bivalvia
Early Pleistocene	2.588	0.781	Olsson anc	1953	25463	Mollusca	7805	Bivalvia
Early Pleistocene	2.588	0.781	Olsson anc	1953	25463	Mollusca	7805	Bivalvia
Early Pleistocene	2.588	0.781	Olsson anc	1953	25463	Mollusca	7805	Bivalvia
Early Pleistocene	2.588	0.781	Olsson anc	1953	25463	Mollusca	7805	Bivalvia
Early Pleistocene	2.588	0.781	Olsson anc	1953	25463	Mollusca	7805	Bivalvia
Early Pleistocene	2.588	0.781	Olsson anc	1953	25463	Mollusca	7805	Bivalvia
Early Pleistocene	2.588	0.781	Olsson anc	1953	25463	Mollusca	7805	Bivalvia
Early Pleistocene	2.588	0.781	Olsson anc	1953	25463	Mollusca	7805	Bivalvia
Early Pleistocene	2.588	0.781	Olsson anc	1953	25463	Mollusca	7805	Bivalvia
Late Pleistocene	0.126	0.0117	Lovejoy	1992	26806	Mollusca	7805	Bivalvia
Late Pleistocene	0.126	0.0117	Lovejoy	1992	26806	Mollusca	7805	Bivalvia
Late Pleistocene	0.126	0.0117	Petuch anc	2007	26813	Mollusca	7805	Bivalvia
Late Pleistocene	0.126	0.0117	Petuch anc	2007	26813	Mollusca	7805	Bivalvia
Late Pleistocene	0.126	0.0117	Petuch anc	2007	26813	Mollusca	7805	Bivalvia
Late Pleistocene	0.126	0.0117	Petuch anc	2007	26813	Mollusca	7805	Bivalvia
Late Pleistocene	0.126	0.0117	Petuch anc	2007	26813	Mollusca	7805	Bivalvia
Early Pleistocene	2.588	0.781	Petuch anc	2007	26813	Mollusca	7805	Bivalvia
Middle Pleistocene	0.781	0.126	Petuch anc	2007	26813	Mollusca	7805	Bivalvia
Late Pliocene	3.6	2.588	Olsson	1967	19826	Mollusca	7805	Bivalvia
Late Pliocene	3.6	2.588	Olsson anc	1964	26836	Mollusca	7805	Bivalvia
Late Pliocene	3.6	2.588	DuBar	1958	25514	Mollusca	7805	Bivalvia
Late Pliocene	3.6	2.588	DuBar	1958	25514	Mollusca	7805	Bivalvia
Late Plioce Pleistocen	3.6	0.0117	Petuch anc	2007	26813	Mollusca	7805	Bivalvia
Early Pleistocene	2.588	0.781	Hendy et a	2008	19909	Mollusca	7805	Bivalvia
Early Pleistocene	2.588	0.781	Hendy et a	2008	19909	Mollusca	7805	Bivalvia
Early Pleistocene	2.588	0.781	Hendy et a	2008	19909	Mollusca	7805	Bivalvia
Early Pleistocene	2.588	0.781	Hendy et a	2008	19909	Mollusca	7805	Bivalvia
Early Pleistocene	2.588	0.781	Hendy et a	2008	19909	Mollusca	7805	Bivalvia
Early Pleistocene	2.588	0.781	Hendy et a	2008	19909	Mollusca	7805	Bivalvia
Early Pleistocene	2.588	0.781	Hendy et a	2008	19909	Mollusca	7805	Bivalvia
Early Pleistocene	2.588	0.781	Hendy et a	2008	19909	Mollusca	7805	Bivalvia
Early Pleistocene	2.588	0.781	Hendy et a	2008	19909	Mollusca	7805	Bivalvia
Early Pleistocene	2.588	0.781	Hendy et a	2008	19909	Mollusca	7805	Bivalvia
Early Pleistocene	2.588	0.781	Hendy et a	2008	19909	Mollusca	7805	Bivalvia
Early Pleistocene	2.588	0.781	Hendy et a	2008	19909	Mollusca	7805	Bivalvia
Late Pleistocene	0.126	0.0117	Hendy et a	2008	19909	Mollusca	7805	Bivalvia
Late Pleistocene	0.126	0.0117	Hendy et a	2008	19909	Mollusca	7805	Bivalvia
Late Pleistocene	0.126	0.0117	Hendy et a	2008	19909	Mollusca	7805	Bivalvia
Late Pleistocene	0.126	0.0117	Hendy et a	2008	19909	Mollusca	7805	Bivalvia
Late Pleistocene	0.126	0.0117	Hendy et a	2008	19909	Mollusca	7805	Bivalvia
Late Pleistocene	0.126	0.0117	Hendy et a	2008	19909	Mollusca	7805	Bivalvia
Late Pleistocene	0.126	0.0117	Hendy et a	2008	19909	Mollusca	7805	Bivalvia

Late Pleistocene	0.126	0.0117	Hendy et a	2008	19909	Mollusca	7805	Bivalvia
Late Pliocene	3.6	2.588	Petuch anc	2007	26813	Mollusca	7805	Bivalvia
Late Pliocene	3.6	2.588	Petuch anc	2007	26813	Mollusca	7805	Bivalvia
Late Pliocene	3.6	2.588	Petuch anc	2007	26813	Mollusca	7805	Bivalvia
Late Pliocene	3.6	2.588	Petuch anc	2007	26813	Mollusca	7805	Bivalvia
Late Pliocene	3.6	2.588	Petuch anc	2007	26813	Mollusca	7805	Bivalvia
Late Pliocene	3.6	2.588	Petuch anc	2007	26813	Mollusca	7805	Bivalvia
Late Pliocene	3.6	2.588	Petuch anc	2007	26813	Mollusca	7805	Bivalvia
Late Pliocene	3.6	2.588	Petuch anc	2007	26813	Mollusca	7805	Bivalvia
Early Pleistocene	2.588	0.781	DuBar	1958	25514	Mollusca	7805	Bivalvia
Early Pleistocene	2.588	0.781	DuBar	1958	25514	Mollusca	7805	Bivalvia
Early Pleistocene	2.588	0.781	DuBar	1958	25514	Mollusca	7805	Bivalvia
Early Pleistocene	2.588	0.781	DuBar	1958	25514	Mollusca	7805	Bivalvia
Early Pleistocene	2.588	0.781	DuBar	1958	25514	Mollusca	7805	Bivalvia
Late Pleistocene	0.126	0.0117	DuBar	1958	25514	Mollusca	7805	Bivalvia
Late Pleistocene	0.126	0.0117	DuBar	1958	25514	Mollusca	7805	Bivalvia
Late Pleistocene	0.126	0.0117	DuBar	1958	25514	Mollusca	7805	Bivalvia
Late Pleistocene	0.126	0.0117	DuBar	1958	25514	Mollusca	7805	Bivalvia
Late Pleistocene	0.126	0.0117	DuBar	1958	25514	Mollusca	7805	Bivalvia
Late Pleistocene	0.126	0.0117	DuBar	1958	25514	Mollusca	7805	Bivalvia
Late Pleistocene	0.126	0.0117	Hendy et a	2008	19909	Mollusca	7805	Bivalvia
Late Pleistocene	0.126	0.0117	Hendy et a	2008	19909	Mollusca	7805	Bivalvia
Late Pleistocene	0.126	0.0117	Hendy et a	2008	19909	Mollusca	7805	Bivalvia
Late Pliocene	3.6	2.588	Hendy et a	2008	19909	Mollusca	7805	Bivalvia
Late Pliocene	3.6	2.588	Hendy et a	2008	19909	Mollusca	7805	Bivalvia
Late Pliocene	3.6	2.588	Hendy et a	2008	19909	Mollusca	7805	Bivalvia
Late Pliocene	3.6	2.588	Hendy et a	2008	19909	Mollusca	7805	Bivalvia
Early Pleistocene	2.588	0.781	Hendy et a	2008	19909	Mollusca	7805	Bivalvia
Early Pleistocene	2.588	0.781	Hendy et a	2008	19909	Mollusca	7805	Bivalvia
Early Pleistocene	2.588	0.781	Hendy et a	2008	19909	Mollusca	7805	Bivalvia
Early Pleistocene	2.588	0.781	Hendy et a	2008	19909	Mollusca	7805	Bivalvia
Early Pleistocene	2.588	0.781	Hendy et a	2008	19909	Mollusca	7805	Bivalvia
Early Pleistocene	2.588	0.781	Hendy et a	2008	19909	Mollusca	7805	Bivalvia
Early Pleistocene	2.588	0.781	Hendy et a	2008	19909	Mollusca	7805	Bivalvia
Early Pleistocene	2.588	0.781	Hendy et a	2008	19909	Mollusca	7805	Bivalvia
Early Pleistocene	2.588	0.781	Hendy et a	2008	19909	Mollusca	7805	Bivalvia
Early Pleistocene	2.588	0.781	Hendy et a	2008	19909	Mollusca	7805	Bivalvia
Early Pleistocene	2.588	0.781	Hendy et a	2008	19909	Mollusca	7805	Bivalvia
Early Pleistocene	2.588	0.781	Hendy et a	2008	19909	Mollusca	7805	Bivalvia
Early Pleistocene	2.588	0.781	Hendy et a	2008	19909	Mollusca	7805	Bivalvia
Early Pleistocene	2.588	0.781	Hendy et a	2008	19909	Mollusca	7805	Bivalvia
Early Pleistocene	2.588	0.781	Hendy et a	2008	19909	Mollusca	7805	Bivalvia
Early Pleistocene	2.588	0.781	Hendy et a	2008	19909	Mollusca	7805	Bivalvia
Early Pleistocene	2.588	0.781	Hendy et a	2008	19909	Mollusca	7805	Bivalvia
Early Pleistocene	2.588	0.781	Hendy et a	2008	19909	Mollusca	7805	Bivalvia
Early Pleistocene	2.588	0.781	Hendy et a	2008	19909	Mollusca	7805	Bivalvia

Early Pleistocene	2.588	0.781	Hendy et a	2008	19909	Mollusca	7805	Bivalvia
Early Pleistocene	2.588	0.781	Hendy et a	2008	19909	Mollusca	7805	Bivalvia
Late Pliocene	3.6	2.588	Hendy et a	2008	19909	Mollusca	7805	Bivalvia
Late Pliocene	3.6	2.588	Hendy et a	2008	19909	Mollusca	7805	Bivalvia
Late Pliocene	3.6	2.588	Hendy et a	2008	19909	Mollusca	7805	Bivalvia
Late Pliocene	3.6	2.588	Hendy et a	2008	19909	Mollusca	7805	Bivalvia
Late Pliocene	3.6	2.588	Hendy et a	2008	19909	Mollusca	7805	Bivalvia
Late Pliocene	3.6	2.588	Hendy et a	2008	19909	Mollusca	7805	Bivalvia
Late Pliocene	3.6	2.588	Hendy et a	2008	19909	Mollusca	7805	Bivalvia
Late Pliocene	3.6	2.588	Hendy et a	2008	19909	Mollusca	7805	Bivalvia
Late Pliocene	3.6	2.588	Hendy et a	2008	19909	Mollusca	7805	Bivalvia
Late Pliocene	3.6	2.588	Hendy et a	2008	19909	Mollusca	7805	Bivalvia
Late Pliocene	3.6	2.588	Hendy et a	2008	19909	Mollusca	7805	Bivalvia
Late Pliocene	3.6	2.588	Hendy et a	2008	19909	Mollusca	7805	Bivalvia
Late Pliocene	3.6	2.588	Hendy et a	2008	19909	Mollusca	7805	Bivalvia
Late Pliocene	3.6	2.588	Hendy et a	2008	19909	Mollusca	7805	Bivalvia
Late Pliocene	3.6	2.588	Hendy et a	2008	19909	Mollusca	7805	Bivalvia
Late Pliocene	3.6	2.588	Hendy et a	2008	19909	Mollusca	7805	Bivalvia
Middle Pleistocene	0.781	0.126	Hendy et a	2008	19909	Mollusca	7805	Bivalvia
Middle Pleistocene	0.781	0.126	Hendy et a	2008	19909	Mollusca	7805	Bivalvia
Middle Pleistocene	0.781	0.126	Hendy et a	2008	19909	Mollusca	7805	Bivalvia
Middle Pleistocene	0.781	0.126	Hendy et a	2008	19909	Mollusca	7805	Bivalvia
Middle Pleistocene	0.781	0.126	Hendy et a	2008	19909	Mollusca	7805	Bivalvia
Middle Pleistocene	0.781	0.126	Hendy et a	2008	19909	Mollusca	7805	Bivalvia
Middle Pleistocene	0.781	0.126	Hendy et a	2008	19909	Mollusca	7805	Bivalvia
Middle Pleistocene	0.781	0.126	Hendy et a	2008	19909	Mollusca	7805	Bivalvia
Middle Pleistocene	0.781	0.126	Hendy et a	2008	19909	Mollusca	7805	Bivalvia
Late Pleistocene	0.126	0.0117	Hendy et a	2008	19909	Mollusca	7805	Bivalvia
Late Pleistocene	0.126	0.0117	Hendy et a	2008	19909	Mollusca	7805	Bivalvia
Late Pleistocene	0.126	0.0117	Hendy et a	2008	19909	Mollusca	7805	Bivalvia
Late Pleistocene	0.126	0.0117	Hendy et a	2008	19909	Mollusca	7805	Bivalvia
Early Pleistocene	2.588	0.781	Hendy et a	2008	19909	Mollusca	7805	Bivalvia
Early Pleistocene	2.588	0.781	Hendy et a	2008	19909	Mollusca	7805	Bivalvia
Early Pleistocene	2.588	0.781	Hendy et a	2008	19909	Mollusca	7805	Bivalvia
Early Pleistocene	2.588	0.781	Hendy et a	2008	19909	Mollusca	7805	Bivalvia
Early Pleistocene	2.588	0.781	Hendy et a	2008	19909	Mollusca	7805	Bivalvia
Early Pleistocene	2.588	0.781	Hendy et a	2008	19909	Mollusca	7805	Bivalvia
Early Pleistocene	2.588	0.781	Hendy et a	2008	19909	Mollusca	7805	Bivalvia
Early Pleistocene	2.588	0.781	Hendy et a	2008	19909	Mollusca	7805	Bivalvia
Early Pleistocene	2.588	0.781	Hendy et a	2008	19909	Mollusca	7805	Bivalvia
Early Pleistocene	2.588	0.781	DuBar	1958	25514	Mollusca	7805	Bivalvia
Early Pleistocene	2.588	0.781	DuBar	1958	25514	Mollusca	7805	Bivalvia
Early Pleistocene	2.588	0.781	DuBar	1958	25514	Mollusca	7805	Bivalvia
Early Pleistocene	2.588	0.781	DuBar	1958	25514	Mollusca	7805	Bivalvia
Early Pleistocene	2.588	0.781	DuBar	1958	25514	Mollusca	7805	Bivalvia
Early Pleistocene	2.588	0.781	DuBar	1958	25514	Mollusca	7805	Bivalvia
Late Pleistocene	0.126	0.0117	DuBar	1958	25514	Mollusca	7805	Bivalvia

Early Pleistocene	2.588	0.781	DuBar	1958	25514	Mollusca	7805	Bivalvia
Early Pleistocene	2.588	0.781	DuBar	1958	25514	Mollusca	7805	Bivalvia
Late Pliocene	3.6	2.588	Smith	1941	32333	Mollusca	7805	Bivalvia
Late Pliocene	3.6	2.588	Weisbord	1971	39743	Mollusca	7805	Bivalvia
Late Pleistocene	0.126	0.0117	Kittle and F	2010	40884	Mollusca	7805	Bivalvia
Late Pleistocene	0.126	0.0117	Kittle and F	2010	40884	Mollusca	7805	Bivalvia
Late Pleistocene	0.126	0.0117	Kittle and F	2010	40884	Mollusca	7805	Bivalvia
Late Pleistocene	0.126	0.0117	Kittle and F	2010	40884	Mollusca	7805	Bivalvia
Late Pleistocene	0.126	0.0117	Kittle and F	2010	40884	Mollusca	7805	Bivalvia
Late Pleistocene	0.126	0.0117	Kittle and F	2010	40884	Mollusca	7805	Bivalvia
Late Pleistocene	0.126	0.0117	Kittle and F	2010	40884	Mollusca	7805	Bivalvia
Late Pleistocene	0.126	0.0117	Kittle and F	2010	40884	Mollusca	7805	Bivalvia
Late Pleistocene	0.126	0.0117	Kittle and F	2010	40884	Mollusca	7805	Bivalvia
Late Pleistocene	0.126	0.0117	Kittle and F	2010	40884	Mollusca	7805	Bivalvia
Late Pleistocene	0.126	0.0117	Kittle and F	2010	40884	Mollusca	7805	Bivalvia
Late Pleistocene	0.126	0.0117	Kittle and F	2010	40884	Mollusca	7805	Bivalvia
Late Pleistocene	0.126	0.0117	Hulbert an	1998	1962	Mollusca	7805	Bivalvia
Late Pleistocene	0.126	0.0117	Richards	1962	18974	Mollusca	7805	Bivalvia
Pliocene	5.333	2.588	Aldrich	1911	53829	Mollusca	7805	Bivalvia
Pliocene	5.333	2.588	Aldrich	1911	53829	Mollusca	7805	Bivalvia
Late Pleistocene	0.126	0.0117	DuBar	1971	17556	Mollusca	7805	Bivalvia
Late Pleistocene	0.126	0.0117	DuBar	1971	17556	Mollusca	7805	Bivalvia
Late Pleistocene	0.126	0.0117	DuBar	1971	17556	Mollusca	7805	Bivalvia
Late Pleistocene	0.126	0.0117	Hendy et a	2008	19909	Mollusca	7805	Bivalvia
Late Pleistocene	0.126	0.0117	DuBar	1971	17556	Mollusca	7805	Bivalvia
Late Pleistocene	0.126	0.0117	DuBar	1971	17556	Mollusca	7805	Bivalvia
Late Pleistocene	0.126	0.0117	DuBar	1971	17556	Mollusca	7805	Bivalvia
Late Pleistocene	0.126	0.0117	DuBar	1971	17556	Mollusca	7805	Bivalvia
Late Pleistocene	0.126	0.0117	DuBar	1971	17556	Mollusca	7805	Bivalvia
Late Pleistocene	0.126	0.0117	DuBar	1971	17556	Mollusca	7805	Bivalvia
Late Pleistocene	0.126	0.0117	DuBar	1971	17556	Mollusca	7805	Bivalvia
Late Pleistocene	0.126	0.0117	DuBar	1971	17556	Mollusca	7805	Bivalvia
Late Pleistocene	0.126	0.0117	DuBar	1971	17556	Mollusca	7805	Bivalvia
Late Pleistocene	0.126	0.0117	DuBar	1971	17556	Mollusca	7805	Bivalvia
Late Pleistocene	0.126	0.0117	DuBar	1971	17556	Mollusca	7805	Bivalvia
Late Pleistocene	0.126	0.0117	DuBar	1971	17556	Mollusca	7805	Bivalvia
Late Pleistocene	0.126	0.0117	DuBar	1971	17556	Mollusca	7805	Bivalvia
Pliocene	5.333	2.588	DuBar	1971	17556	Mollusca	7805	Bivalvia
Late Pliocene	3.6	2.588	Campbell a	1995	17558	Mollusca	7805	Bivalvia
Zanclean	5.333	3.6	Campbell a	1995	17558	Mollusca	7805	Bivalvia
Zanclean	5.333	3.6	Campbell a	1995	17558	Mollusca	7805	Bivalvia
Zanclean	5.333	3.6	Campbell a	1995	17558	Mollusca	7805	Bivalvia
Zanclean	5.333	3.6	Campbell a	1995	17558	Mollusca	7805	Bivalvia
Zanclean	5.333	3.6	Campbell a	1995	17558	Mollusca	7805	Bivalvia
Early Pliocene	5.333	3.6	Campbell a	1995	17558	Mollusca	7805	Bivalvia
Late Pliocene	3.6	2.588	Campbell a	1995	17558	Mollusca	7805	Bivalvia
Early Pleistocene	2.588	0.781	DuBar	1971	17556	Mollusca	7805	Bivalvia

Early Pleistocene	2.588	0.781	DuBar	1971	17556	Mollusca	7805	Bivalvia
Early Pleistocene	2.588	0.781	DuBar	1971	17556	Mollusca	7805	Bivalvia
Early Pleistocene	2.588	0.781	DuBar	1971	17556	Mollusca	7805	Bivalvia
Late Pleistocene	0.126	0.0117	Richards	1962	18974	Mollusca	7805	Bivalvia
Late Pleistocene	0.126	0.0117	Richards	1962	18974	Mollusca	7805	Bivalvia
Late Pleistocene	0.126	0.0117	Richards	1962	18974	Mollusca	7805	Bivalvia
Late Pleistocene	0.126	0.0117	Richards	1962	18974	Mollusca	7805	Bivalvia
Late Pleistocene	0.126	0.0117	Richards	1962	18974	Mollusca	7805	Bivalvia
Late Pleistocene	0.126	0.0117	Richards	1962	18974	Mollusca	7805	Bivalvia
Late Pleistocene	0.126	0.0117	Richards	1962	18974	Mollusca	7805	Bivalvia
Late Pleistocene	0.126	0.0117	Richards	1962	18974	Mollusca	7805	Bivalvia
Late Pleistocene	0.126	0.0117	Richards	1962	18974	Mollusca	7805	Bivalvia
Late Pleistocene	0.126	0.0117	Richards	1962	18974	Mollusca	7805	Bivalvia
Late Pleistocene	0.126	0.0117	Richards	1962	18974	Mollusca	7805	Bivalvia
Late Pleistocene	0.126	0.0117	Richards	1962	18974	Mollusca	7805	Bivalvia
Late Pleistocene	0.126	0.0117	Richards	1962	18974	Mollusca	7805	Bivalvia
Late Pleistocene	0.126	0.0117	Richards	1962	18974	Mollusca	7805	Bivalvia
Late Pleistocene	0.126	0.0117	Richards	1962	18974	Mollusca	7805	Bivalvia
Late Pleistocene	0.126	0.0117	Hendy et a	2008	19909	Mollusca	7805	Bivalvia
Late Pleistocene	0.126	0.0117	Hendy et a	2008	19909	Mollusca	7805	Bivalvia
Late Pleistocene	0.126	0.0117	Hendy et a	2008	19909	Mollusca	7805	Bivalvia
Late Pleistocene	0.126	0.0117	Hendy et a	2008	19909	Mollusca	7805	Bivalvia
Late Pleistocene	0.126	0.0117	Hendy et a	2008	19909	Mollusca	7805	Bivalvia
Late Pleistocene	0.126	0.0117	Hendy et a	2008	19909	Mollusca	7805	Bivalvia
Late Pleistocene	0.126	0.0117	Hendy et a	2008	19909	Mollusca	7805	Bivalvia
Late Pleistocene	0.126	0.0117	Hendy et a	2008	19909	Mollusca	7805	Bivalvia
Late Pleistocene	0.126	0.0117	Hendy et a	2008	19909	Mollusca	7805	Bivalvia
Pleistocene	2.588	0.0117	Hendy et a	2008	19909	Mollusca	7805	Bivalvia
Pleistocene	2.588	0.0117	Hendy et a	2008	19909	Mollusca	7805	Bivalvia
Pleistocene	2.588	0.0117	Hendy et a	2008	19909	Mollusca	7805	Bivalvia
Pleistocene	2.588	0.0117	Hendy et a	2008	19909	Mollusca	7805	Bivalvia
Pleistocene	2.588	0.0117	Hendy et a	2008	19909	Mollusca	7805	Bivalvia
Pleistocene	2.588	0.0117	Hendy et a	2008	19909	Mollusca	7805	Bivalvia
Pleistocene	2.588	0.0117	Hendy et a	2008	19909	Mollusca	7805	Bivalvia
Pleistocene	2.588	0.0117	Hendy et a	2008	19909	Mollusca	7805	Bivalvia
Pleistocene	2.588	0.0117	Hendy et a	2008	19909	Mollusca	7805	Bivalvia
Late Pleistocene	0.126	0.0117	Cooke	1936	31145	Mollusca	7805	Bivalvia
Early Pleistocene	2.588	0.781	Cooke	1936	31145	Mollusca	7805	Bivalvia
Early Pleistocene	2.588	0.781	Cooke	1936	31145	Mollusca	7805	Bivalvia
Early Pleistocene	2.588	0.781	Cooke	1936	31145	Mollusca	7805	Bivalvia
Early Pleistocene	2.588	0.781	Cooke	1936	31145	Mollusca	7805	Bivalvia
Early Pleistocene	2.588	0.781	Cooke	1936	31145	Mollusca	7805	Bivalvia
Early Pleistocene	2.588	0.781	Cooke	1936	31145	Mollusca	7805	Bivalvia
Early Pleistocene	2.588	0.781	Cooke	1936	31145	Mollusca	7805	Bivalvia
Early Pleistocene	2.588	0.781	Cooke	1936	31145	Mollusca	7805	Bivalvia
Early Pleistocene	2.588	0.781	Cooke	1936	31145	Mollusca	7805	Bivalvia
Early Pleistocene	2.588	0.781	Cooke	1936	31145	Mollusca	7805	Bivalvia

Early Pleistocene		2.588	0.781	Cooke	1936	31145	Mollusca	7805	Bivalvia
Early Pleistocene		2.588	0.781	Hendy et a	2008	19909	Mollusca	7805	Bivalvia
Early Pleistocene		2.588	0.781	Cooke	1936	31145	Mollusca	7805	Bivalvia
Late Pliocene		3.6	2.588	Cooke	1936	31145	Mollusca	7805	Bivalvia
Pleistocene		2.588	0.0117	Holmes	1860	64712	Mollusca	7805	Bivalvia
Late Pliocene		3.6	2.588	Ward and I	1987	16700	Mollusca	7805	Bivalvia
Late Pliocene		3.6	2.588	Ward and I	1987	16700	Mollusca	7805	Bivalvia
Late Pliocene		3.6	2.588	Ward and I	1987	16700	Mollusca	7805	Bivalvia
Late Pliocene		3.6	2.588	Ward and I	1987	16700	Mollusca	7805	Bivalvia
Late Pliocene		3.6	2.588	Ward and I	1987	16700	Mollusca	7805	Bivalvia
Early Pleistocene		2.588	0.781	Ward and I	1987	16700	Mollusca	7805	Bivalvia
Early Pleistocene		2.588	0.781	Ward and I	1987	16700	Mollusca	7805	Bivalvia
Early Pleistocene		2.588	0.781	Ward and I	1987	16700	Mollusca	7805	Bivalvia
Early Pleistocene		2.588	0.781	Ward and I	1987	16700	Mollusca	7805	Bivalvia
Early Pleistocene		2.588	0.781	Ward and I	1987	16700	Mollusca	7805	Bivalvia
Early Pleistocene		2.588	0.781	Ward and I	1987	16700	Mollusca	7805	Bivalvia
Early Pleistocene		2.588	0.781	Ward and I	1987	16700	Mollusca	7805	Bivalvia
Late Pliocene		3.6	2.588	Ward and I	1987	16700	Mollusca	7805	Bivalvia
Late Pliocene		3.6	2.588	Ward and I	1987	16700	Mollusca	7805	Bivalvia
Late Pliocene		3.6	2.588	Ward and I	1987	16700	Mollusca	7805	Bivalvia
Early Pleistocene		2.588	0.781	Ward and I	1987	16700	Mollusca	7805	Bivalvia
Early Pleistocene		2.588	0.781	Ward and I	1987	16700	Mollusca	7805	Bivalvia
Early Pleistocene		2.588	0.781	Ward and I	1987	16700	Mollusca	7805	Bivalvia
Early Pleistocene		2.588	0.781	Ward and I	1987	16700	Mollusca	7805	Bivalvia
Late Pliocene		3.6	2.588	Ward	1992	16985	Mollusca	7805	Bivalvia
Late Pliocene		3.6	2.588	Gardner	1943	16984	Mollusca	7805	Bivalvia
Early Plioc	Late Plioce	5.333	2.588	Gardner	1943	16984	Mollusca	7805	Bivalvia
Late Pliocene		3.6	2.588	Gardner	1943	16984	Mollusca	7805	Bivalvia
Pleistocene		2.588	0.0117	Gardner	1943	16984	Mollusca	7805	Bivalvia
Early Pleistocene		2.588	0.781	Gardner	1943	16984	Mollusca	7805	Bivalvia
Late Pliocene		3.6	2.588	Gardner	1943	16984	Mollusca	7805	Bivalvia
Late Pliocene		3.6	2.588	Gardner	1943	16984	Mollusca	7805	Bivalvia
Late Pliocene		3.6	2.588	Gardner	1943	16984	Mollusca	7805	Bivalvia
Pleistocene		2.588	0.0117	Gardner	1943	16984	Mollusca	7805	Bivalvia
Late Pliocene		3.6	2.588	Gardner	1943	16984	Mollusca	7805	Bivalvia
Early Pleistocene		2.588	0.781	Gardner	1943	16984	Mollusca	7805	Bivalvia
Early Pleistocene		2.588	0.781	Gardner	1943	16984	Mollusca	7805	Bivalvia
Late Pliocene		3.6	2.588	Gardner	1943	16984	Mollusca	7805	Bivalvia
Early Pleistocene		2.588	0.781	Gardner	1943	16984	Mollusca	7805	Bivalvia
Pleistocene		2.588	0.0117	Gardner	1943	16984	Mollusca	7805	Bivalvia
Early Pleistocene		2.588	0.781	Gardner	1943	16984	Mollusca	7805	Bivalvia
Early Pleistocene		2.588	0.781	Gardner	1943	16984	Mollusca	7805	Bivalvia
Late Pliocene		3.6	2.588	Gardner	1943	16984	Mollusca	7805	Bivalvia
Late Pliocene		3.6	2.588	Gardner	1943	16984	Mollusca	7805	Bivalvia
Early Plioc	Late Plioce	5.333	2.588	Gardner	1943	16984	Mollusca	7805	Bivalvia
Early Pleistocene		2.588	0.781	Gardner	1943	16984	Mollusca	7805	Bivalvia
Late Pliocene		3.6	2.588	Gardner	1943	16984	Mollusca	7805	Bivalvia

Early Plioc	Late Plioce	5.333	2.588	Gardner	1943	16984	Mollusca	7805	Bivalvia
Early Pleistocene		2.588	0.781	Gardner	1943	16984	Mollusca	7805	Bivalvia
Pleistocene		2.588	0.0117	Gardner	1943	16984	Mollusca	7805	Bivalvia
Late Pliocene		3.6	2.588	Gardner	1943	16984	Mollusca	7805	Bivalvia
Early Pleistocene		2.588	0.781	Gardner	1943	16984	Mollusca	7805	Bivalvia
Early Pleistocene		2.588	0.781	Gardner	1943	16984	Mollusca	7805	Bivalvia
Early Pleistocene		2.588	0.781	Gardner	1943	16984	Mollusca	7805	Bivalvia
Late Pliocene		3.6	2.588	Gardner	1943	16984	Mollusca	7805	Bivalvia
Late Pliocene		3.6	2.588	Gardner	1943	16984	Mollusca	7805	Bivalvia
Pleistocene		2.588	0.0117	Gardner	1943	16984	Mollusca	7805	Bivalvia
Early Pleistocene		2.588	0.781	Gardner	1943	16984	Mollusca	7805	Bivalvia
Early Pleistocene		2.588	0.781	Gardner	1943	16984	Mollusca	7805	Bivalvia
Early Plioc	Late Plioce	5.333	2.588	Gardner	1943	16984	Mollusca	7805	Bivalvia
Early Pleistocene		2.588	0.781	Gardner	1943	16984	Mollusca	7805	Bivalvia
Early Plioc	Late Plioce	5.333	2.588	Gardner	1943	16984	Mollusca	7805	Bivalvia
Early Plioc	Late Plioce	5.333	2.588	Gardner	1943	16984	Mollusca	7805	Bivalvia
Late Pliocene		3.6	2.588	Gardner	1943	16984	Mollusca	7805	Bivalvia
Late Pliocene		3.6	2.588	Gardner	1943	16984	Mollusca	7805	Bivalvia
Late Pliocene		3.6	2.588	Gardner	1943	16984	Mollusca	7805	Bivalvia
Late Pliocene		3.6	2.588	Gardner	1943	16984	Mollusca	7805	Bivalvia
Late Pliocene		3.6	2.588	Gardner	1943	16984	Mollusca	7805	Bivalvia
Pleistocene		2.588	0.0117	Gardner	1943	16984	Mollusca	7805	Bivalvia
Early Pleistocene		2.588	0.781	Gardner	1943	16984	Mollusca	7805	Bivalvia
Early Pleistocene		2.588	0.781	Gardner	1943	16984	Mollusca	7805	Bivalvia
Early Pleistocene		2.588	0.781	Gardner	1943	16984	Mollusca	7805	Bivalvia
Early Plioc	Late Plioce	5.333	2.588	Gardner	1943	16984	Mollusca	7805	Bivalvia
Early Pleistocene		2.588	0.781	Gardner	1943	16984	Mollusca	7805	Bivalvia
Early Pleistocene		2.588	0.781	Gardner	1943	16984	Mollusca	7805	Bivalvia
Early Pleistocene		2.588	0.781	Gardner	1943	16984	Mollusca	7805	Bivalvia
Early Pleistocene		2.588	0.781	Gardner	1943	16984	Mollusca	7805	Bivalvia
Early Pleistocene		2.588	0.781	Gardner	1943	16984	Mollusca	7805	Bivalvia
Late Pliocene		3.6	2.588	Gardner	1943	16984	Mollusca	7805	Bivalvia
Late Pliocene		3.6	2.588	Gardner	1943	16984	Mollusca	7805	Bivalvia
Late Pliocene		3.6	2.588	Gardner	1943	16984	Mollusca	7805	Bivalvia
Early Plioc	Late Plioce	5.333	2.588	Gardner	1943	16984	Mollusca	7805	Bivalvia
Early Plioc	Late Plioce	5.333	2.588	Gardner	1943	16984	Mollusca	7805	Bivalvia
Early Plioc	Late Plioce	5.333	2.588	Gardner	1943	16984	Mollusca	7805	Bivalvia
Pleistocene		2.588	0.0117	Gardner	1943	16984	Mollusca	7805	Bivalvia
Early Pleistocene		2.588	0.781	Gardner	1943	16984	Mollusca	7805	Bivalvia
Early Plioc	Late Plioce	5.333	2.588	Gardner	1943	16984	Mollusca	7805	Bivalvia
Early Pleistocene		2.588	0.781	Miller	1990	17561	Mollusca	7805	Bivalvia
Early Pleistocene		2.588	0.781	Miller	1990	17561	Mollusca	7805	Bivalvia
Early Pleistocene		2.588	0.781	Miller	1990	17561	Mollusca	7805	Bivalvia
Early Pleistocene		2.588	0.781	Miller	1990	17561	Mollusca	7805	Bivalvia
Early Pleistocene		2.588	0.781	Miller	1990	17561	Mollusca	7805	Bivalvia
Early Pleistocene		2.588	0.781	Miller	1990	17561	Mollusca	7805	Bivalvia
Piacenzian	Calabrian	3.6	0.781	Wilson	1983	17889	Mollusca	7805	Bivalvia

Zanclean	5.333	3.6	Wilson	1983	17889	Mollusca	7805	Bivalvia
Pliocene	5.333	2.588	Wilson	1983	17889	Mollusca	7805	Bivalvia
Pliocene	5.333	2.588	Wilson	1983	17889	Mollusca	7805	Bivalvia
Pliocene	5.333	2.588	Wilson	1983	17889	Mollusca	7805	Bivalvia
Pliocene	5.333	2.588	Wilson	1983	17889	Mollusca	7805	Bivalvia
Pliocene	5.333	2.588	Wilson	1983	17889	Mollusca	7805	Bivalvia
Pliocene	5.333	2.588	Wilson	1983	17889	Mollusca	7805	Bivalvia
Pliocene	5.333	2.588	Wilson	1983	17889	Mollusca	7805	Bivalvia
Pliocene	5.333	2.588	Wilson	1983	17889	Mollusca	7805	Bivalvia
Pliocene	5.333	2.588	Wilson	1983	17889	Mollusca	7805	Bivalvia
Late Pleistocene	0.126	0.0117	Richards	1962	18974	Mollusca	7805	Bivalvia
Late Pleistocene	0.126	0.0117	Richards	1962	18974	Mollusca	7805	Bivalvia
Late Pleistocene	0.126	0.0117	Richards	1962	18974	Mollusca	7805	Bivalvia
Late Pleistocene	0.126	0.0117	Richards	1962	18974	Mollusca	7805	Bivalvia
Late Pleistocene	0.126	0.0117	Richards	1962	18974	Mollusca	7805	Bivalvia
Late Pleistocene	0.126	0.0117	Richards	1962	18974	Mollusca	7805	Bivalvia
Late Pleistocene	0.126	0.0117	Richards	1962	18974	Mollusca	7805	Bivalvia
Late Pleistocene	0.126	0.0117	Richards	1962	18974	Mollusca	7805	Bivalvia
Late Pleistocene	0.126	0.0117	Richards	1962	18974	Mollusca	7805	Bivalvia
Late Pleistocene	0.126	0.0117	Richards	1962	18974	Mollusca	7805	Bivalvia
Late Pleistocene	0.126	0.0117	Richards	1962	18974	Mollusca	7805	Bivalvia
Late Pleistocene	0.126	0.0117	Richards	1962	18974	Mollusca	7805	Bivalvia
Late Pleistocene	0.126	0.0117	Richards	1962	18974	Mollusca	7805	Bivalvia
Late Pleistocene	0.126	0.0117	Richards	1962	18974	Mollusca	7805	Bivalvia
Late Pleistocene	0.126	0.0117	Richards	1962	18974	Mollusca	7805	Bivalvia
Late Pleistocene	0.126	0.0117	Richards	1962	18974	Mollusca	7805	Bivalvia
Late Pleistocene	0.126	0.0117	Richards	1962	18974	Mollusca	7805	Bivalvia
Late Pleistocene	0.126	0.0117	Richards	1962	18974	Mollusca	7805	Bivalvia
Late Pleistocene	0.126	0.0117	Richards	1962	18974	Mollusca	7805	Bivalvia
Late Pleistocene	0.126	0.0117	Richards	1962	18974	Mollusca	7805	Bivalvia
Late Pleistocene	0.126	0.0117	Richards	1962	18974	Mollusca	7805	Bivalvia
Late Pleistocene	0.126	0.0117	Richards	1962	18974	Mollusca	7805	Bivalvia
Late Pleistocene	0.126	0.0117	Richards	1962	18974	Mollusca	7805	Bivalvia
Late Pleistocene	0.126	0.0117	Richards	1962	18974	Mollusca	7805	Bivalvia
Late Pleistocene	0.126	0.0117	Richards	1962	18974	Mollusca	7805	Bivalvia
Late Pleistocene	0.126	0.0117	Richards	1962	18974	Mollusca	7805	Bivalvia
Late Pliocene	3.6	2.588	Hendy et a	2008	19909	Mollusca	7805	Bivalvia
Late Pleistocene	0.126	0.0117	Hendy et a	2008	19909	Mollusca	7805	Bivalvia
Late Pleistocene	0.126	0.0117	Hendy et a	2008	19909	Mollusca	7805	Bivalvia
Late Pleistocene	0.126	0.0117	Hendy et a	2008	19909	Mollusca	7805	Bivalvia
Late Pleistocene	0.126	0.0117	Hendy et a	2008	19909	Mollusca	7805	Bivalvia
Gelasian	2.588	1.806	Bailey and	1986	23840	Mollusca	7805	Bivalvia
Gelasian	2.588	1.806	Bailey and	1986	23840	Mollusca	7805	Bivalvia
Gelasian	2.588	1.806	Bailey and	1986	23840	Mollusca	7805	Bivalvia
Gelasian	2.588	1.806	Bailey and	1986	23840	Mollusca	7805	Bivalvia
Early Pleistocene	2.588	0.781	Hendy et a	2008	19909	Mollusca	7805	Bivalvia

Early Pleistocene	2.588	0.781	Hendy et a	2008	19909	Mollusca	7805	Bivalvia
Early Pleistocene	2.588	0.781	Hendy et a	2008	19909	Mollusca	7805	Bivalvia
Early Pleistocene	2.588	0.781	Hendy et a	2008	19909	Mollusca	7805	Bivalvia
Early Pleistocene	2.588	0.781	Hendy et a	2008	19909	Mollusca	7805	Bivalvia
Late Pleistocene	0.126	0.0117	Hendy et a	2008	19909	Mollusca	7805	Bivalvia
Late Pleistocene	0.126	0.0117	Hendy et a	2008	19909	Mollusca	7805	Bivalvia
Late Pleistocene	0.126	0.0117	Hendy et a	2008	19909	Mollusca	7805	Bivalvia
Late Pliocene	3.6	2.588	Hendy et a	2008	19909	Mollusca	7805	Bivalvia
Late Pliocene	3.6	2.588	Hendy et a	2008	19909	Mollusca	7805	Bivalvia
Late Pliocene	3.6	2.588	Hendy et a	2008	19909	Mollusca	7805	Bivalvia
Late Pliocene	3.6	2.588	Hendy et a	2008	19909	Mollusca	7805	Bivalvia
Late Pliocene	3.6	2.588	Hendy et a	2008	19909	Mollusca	7805	Bivalvia
Late Pliocene	3.6	2.588	Hendy et a	2008	19909	Mollusca	7805	Bivalvia
Late Pliocene	3.6	2.588	Hendy et a	2008	19909	Mollusca	7805	Bivalvia
Late Pliocene	3.6	2.588	Hendy et a	2008	19909	Mollusca	7805	Bivalvia
Late Pliocene	3.6	2.588	Hendy et a	2008	19909	Mollusca	7805	Bivalvia
Pliocene	5.333	2.588	Hendy et a	2008	19909	Mollusca	7805	Bivalvia
Pliocene	5.333	2.588	Hendy et a	2008	19909	Mollusca	7805	Bivalvia
Pliocene	5.333	2.588	Hendy et a	2008	19909	Mollusca	7805	Bivalvia
Pliocene	5.333	2.588	Hendy et a	2008	19909	Mollusca	7805	Bivalvia
Late Pliocene	3.6	2.588	Hendy et a	2008	19909	Mollusca	7805	Bivalvia
Late Pliocene	3.6	2.588	Hendy et a	2008	19909	Mollusca	7805	Bivalvia
Late Pliocene	3.6	2.588	Hendy et a	2008	19909	Mollusca	7805	Bivalvia
Late Pliocene	3.6	2.588	Hendy et a	2008	19909	Mollusca	7805	Bivalvia
Late Pliocene	3.6	2.588	Hendy et a	2008	19909	Mollusca	7805	Bivalvia
Late Pliocene	3.6	2.588	Hendy et a	2008	19909	Mollusca	7805	Bivalvia
Late Pliocene	3.6	2.588	Hendy et a	2008	19909	Mollusca	7805	Bivalvia
Late Pliocene	3.6	2.588	Hendy et a	2008	19909	Mollusca	7805	Bivalvia
Late Pliocene	3.6	2.588	Hendy et a	2008	19909	Mollusca	7805	Bivalvia
Early Pleistocene	2.588	0.781	Hendy et a	2008	19909	Mollusca	7805	Bivalvia
Early Pleistocene	2.588	0.781	Hendy et a	2008	19909	Mollusca	7805	Bivalvia
Early Pleistocene	2.588	0.781	Hendy et a	2008	19909	Mollusca	7805	Bivalvia
Early Pleistocene	2.588	0.781	Hendy et a	2008	19909	Mollusca	7805	Bivalvia
Early Pleistocene	2.588	0.781	Hendy et a	2008	19909	Mollusca	7805	Bivalvia
Early Pleistocene	2.588	0.781	Hendy et a	2008	19909	Mollusca	7805	Bivalvia
Early Pleistocene	2.588	0.781	Hendy et a	2008	19909	Mollusca	7805	Bivalvia
Early Pleistocene	2.588	0.781	Hendy et a	2008	19909	Mollusca	7805	Bivalvia
Late Pliocene	3.6	2.588	Hendy et a	2008	19909	Mollusca	7805	Bivalvia
Early Pleistocene	2.588	0.781	Hendy et a	2008	19909	Mollusca	7805	Bivalvia
Early Pleistocene	2.588	0.781	Hendy et a	2008	19909	Mollusca	7805	Bivalvia
Early Pleistocene	2.588	0.781	Hendy et a	2008	19909	Mollusca	7805	Bivalvia
Early Pleistocene	2.588	0.781	Hendy et a	2008	19909	Mollusca	7805	Bivalvia
Early Pleistocene	2.588	0.781	Hendy et a	2008	19909	Mollusca	7805	Bivalvia
Early Pleistocene	2.588	0.781	Hendy et a	2008	19909	Mollusca	7805	Bivalvia
Early Pleistocene	2.588	0.781	Hendy et a	2008	19909	Mollusca	7805	Bivalvia
Early Pleistocene	2.588	0.781	Hendy et a	2008	19909	Mollusca	7805	Bivalvia
Early Pleistocene	2.588	0.781	Hendy et a	2008	19909	Mollusca	7805	Bivalvia

Early Pleistocene		2.588	0.781	Hendy et a	2008	19909	Mollusca	7805	Bivalvia
Early Pleistocene		2.588	0.781	Hendy et a	2008	19909	Mollusca	7805	Bivalvia
Early Pleistocene		2.588	0.781	Hendy et a	2008	19909	Mollusca	7805	Bivalvia
Early Pleistocene		2.588	0.781	Hendy et a	2008	19909	Mollusca	7805	Bivalvia
Early Pleistocene		2.588	0.781	Hendy et a	2008	19909	Mollusca	7805	Bivalvia
Early Pleistocene		2.588	0.781	Hendy et a	2008	19909	Mollusca	7805	Bivalvia
Early Pleistocene		2.588	0.781	Hendy et a	2008	19909	Mollusca	7805	Bivalvia
Early Pleistocene		2.588	0.781	Hendy et a	2008	19909	Mollusca	7805	Bivalvia
Early Pleistocene		2.588	0.781	Hendy et a	2008	19909	Mollusca	7805	Bivalvia
Early Pleistocene		2.588	0.781	Hendy et a	2008	19909	Mollusca	7805	Bivalvia
Early Pleistocene		2.588	0.781	Hendy et a	2008	19909	Mollusca	7805	Bivalvia
Early Pleistocene		2.588	0.781	Hendy et a	2008	19909	Mollusca	7805	Bivalvia
Early Pleistocene		2.588	0.781	Hendy et a	2008	19909	Mollusca	7805	Bivalvia
Early Pleistocene		2.588	0.781	Hendy et a	2008	19909	Mollusca	7805	Bivalvia
Early Pleistocene		2.588	0.781	Hendy et a	2008	19909	Mollusca	7805	Bivalvia
Early Pleistocene		2.588	0.781	Hendy et a	2008	19909	Mollusca	7805	Bivalvia
Early Pleistocene		2.588	0.781	Hendy et a	2008	19909	Mollusca	7805	Bivalvia
Middle Pleistocene		0.781	0.126	Miller	1985	30824	Mollusca	7805	Bivalvia
Pliocene		5.333	2.588	Ward	2007	48009	Mollusca	7805	Bivalvia
Early Pleistocene		2.588	0.781	Miller and	1988	72890	Mollusca	7805	Bivalvia
Early Pleistocene		2.588	0.781	Miller and	1988	72890	Mollusca	7805	Bivalvia
Pleistocene		2.588	0.0117	Coch	1971	441	Mollusca	7805	Bivalvia
Pleistocene		2.588	0.0117	Coch	1971	441	Mollusca	7805	Bivalvia
Pleistocene		2.588	0.0117	Coch	1971	441	Mollusca	7805	Bivalvia
Pleistocene		2.588	0.0117	Coch	1971	441	Mollusca	7805	Bivalvia
Late Pleistocene		0.126	0.0117	Spencer ar	1987	15525	Mollusca	7805	Bivalvia
Late Pleistocene		0.126	0.0117	Spencer ar	1987	15525	Mollusca	7805	Bivalvia
Late Pleistocene		0.126	0.0117	Spencer ar	1987	15525	Mollusca	7805	Bivalvia
Late Pleistocene		0.126	0.0117	Spencer ar	1987	15525	Mollusca	7805	Bivalvia
Late Pleistocene		0.126	0.0117	Spencer ar	1987	15525	Mollusca	7805	Bivalvia
Late Pleistocene		0.126	0.0117	Spencer ar	1987	15525	Mollusca	7805	Bivalvia
Late Pleistocene		0.126	0.0117	Spencer ar	1987	15525	Mollusca	7805	Bivalvia
Late Pleistocene		0.126	0.0117	Spencer ar	1987	15525	Mollusca	7805	Bivalvia
Late Pleistocene		0.126	0.0117	Spencer ar	1987	15525	Mollusca	7805	Bivalvia
Early Pliocene		5.333	3.6	Ward	1992	16985	Mollusca	7805	Bivalvia
Pliocene		5.333	2.588	Gardner	1943	16984	Mollusca	7805	Bivalvia
Late Pliocene		3.6	2.588	Ward	1985	17420	Mollusca	7805	Bivalvia
Late Pliocene		3.6	2.588	Edwards et	2005	17631	Mollusca	7805	Bivalvia
Late Pliocene		3.6	2.588	Hendy et a	2008	19909	Mollusca	7805	Bivalvia
Late Pliocene		3.6	2.588	Edwards et	2005	17631	Mollusca	7805	Bivalvia
Late Pliocene		3.6	2.588	Edwards et	2005	17631	Mollusca	7805	Bivalvia
Late Pliocene		3.6	2.588	Edwards et	2005	17631	Mollusca	7805	Bivalvia
Late Pliocene		3.6	2.588	Edwards et	2005	17631	Mollusca	7805	Bivalvia
Pliocene		5.333	2.588	Wilson	1983	17889	Mollusca	7805	Bivalvia
Pliocene		5.333	2.588	Wilson	1983	17889	Mollusca	7805	Bivalvia
Zanclean	Piacenzian	5.333	2.588	Wilson	1983	17889	Mollusca	7805	Bivalvia
Zanclean	Piacenzian	5.333	2.588	Wilson	1983	17889	Mollusca	7805	Bivalvia

Zanclean	Piacenzian	5.333	2.588	Wilson	1983	17889	Mollusca	7805	Bivalvia
Zanclean	Piacenzian	5.333	2.588	Wilson	1983	17889	Mollusca	7805	Bivalvia
Zanclean	Piacenzian	5.333	2.588	Wilson	1983	17889	Mollusca	7805	Bivalvia
Zanclean	Piacenzian	5.333	2.588	Wilson	1983	17889	Mollusca	7805	Bivalvia
Zanclean	Piacenzian	5.333	2.588	Wilson	1983	17889	Mollusca	7805	Bivalvia
Zanclean	Piacenzian	5.333	2.588	Wilson	1983	17889	Mollusca	7805	Bivalvia
Zanclean	Piacenzian	5.333	2.588	Wilson	1983	17889	Mollusca	7805	Bivalvia
Zanclean	Piacenzian	5.333	2.588	Wilson	1983	17889	Mollusca	7805	Bivalvia
Late Pleistocene		0.126	0.0117	Hendy et a	2008	19909	Mollusca	7805	Bivalvia
Late Pleistocene		0.126	0.0117	Hendy et a	2008	19909	Mollusca	7805	Bivalvia
Late Pleistocene		0.126	0.0117	Hendy et a	2008	19909	Mollusca	7805	Bivalvia
Zanclean		5.333	3.6	Ward	1992	16985	Mollusca	7805	Bivalvia
Zanclean		5.333	3.6	Ward	1992	16985	Mollusca	7805	Bivalvia
Late Pliocene		3.6	2.588	Ward	1992	16985	Mollusca	7805	Bivalvia
Late Pliocene		3.6	2.588	Ward	1992	16985	Mollusca	7805	Bivalvia
Late Pliocene		3.6	2.588	Ward	1993	26351	Mollusca	7805	Bivalvia
Late Pliocene		3.6	2.588	Ward	1993	26351	Mollusca	7805	Bivalvia
Late Pliocene		3.6	2.588	Ward	1993	26351	Mollusca	7805	Bivalvia
Late Pliocene		3.6	2.588	Ward	1993	26351	Mollusca	7805	Bivalvia
Late Pliocene		3.6	2.588	Ward	1993	26351	Mollusca	7805	Bivalvia
Late Pliocene		3.6	2.588	Ward	1993	26351	Mollusca	7805	Bivalvia
Late Pliocene		3.6	2.588	Ward	1993	26351	Mollusca	7805	Bivalvia
Late Pliocene		3.6	2.588	Ward	1993	26351	Mollusca	7805	Bivalvia
Late Pliocene		3.6	2.588	Ward	1993	26351	Mollusca	7805	Bivalvia
Late Pliocene		3.6	2.588	Ward	1993	26351	Mollusca	7805	Bivalvia
Late Pliocene		3.6	2.588	Ward	1993	26351	Mollusca	7805	Bivalvia
Late Pliocene		3.6	2.588	Ward	1993	26351	Mollusca	7805	Bivalvia
Late Pliocene		3.6	2.588	Ward	1993	26351	Mollusca	7805	Bivalvia
Late Pliocene		3.6	2.588	Ward	1993	26351	Mollusca	7805	Bivalvia
Late Pliocene		3.6	2.588	Ward	1993	26351	Mollusca	7805	Bivalvia
Late Pliocene		3.6	2.588	Ward	1993	26351	Mollusca	7805	Bivalvia
Late Pliocene		3.6	2.588	Ward	1993	26351	Mollusca	7805	Bivalvia
Late Pliocene		3.6	2.588	Ward	1993	26351	Mollusca	7805	Bivalvia
Late Pliocene		3.6	2.588	Ward	1993	26351	Mollusca	7805	Bivalvia
Late Pliocene		3.6	2.588	Ward	1993	26351	Mollusca	7805	Bivalvia
Late Pliocene		3.6	2.588	Ward	1993	26351	Mollusca	7805	Bivalvia
Late Pliocene		3.6	2.588	Ward	1993	26351	Mollusca	7805	Bivalvia
Late Pliocene		3.6	2.588	Ward	1993	26351	Mollusca	7805	Bivalvia
Late Pliocene		3.6	2.588	Ward	1993	26351	Mollusca	7805	Bivalvia
Late Pliocene		3.6	2.588	Ward	1993	26351	Mollusca	7805	Bivalvia
Late Pliocene		3.6	2.588	Ward	1993	26351	Mollusca	7805	Bivalvia
Late Pliocene		3.6	2.588	Ward	1993	26351	Mollusca	7805	Bivalvia
Late Pliocene		3.6	2.588	Ward	1993	26351	Mollusca	7805	Bivalvia
Late Pliocene		3.6	2.588	Ward	1993	26351	Mollusca	7805	Bivalvia
Late Pliocene		3.6	2.588	Hendy et a	2008	19909	Mollusca	7805	Bivalvia
Late Pliocene		3.6	2.588	Hendy et a	2008	19909	Mollusca	7805	Bivalvia
Late Pliocene		3.6	2.588	Hendy et a	2008	19909	Mollusca	7805	Bivalvia

Late Pliocene		3.6	2.588	Hendy et a	2008	19909	Mollusca	7805	Bivalvia
Late Pliocene		3.6	2.588	Hendy et a	2008	19909	Mollusca	7805	Bivalvia
Late Pliocene		3.6	2.588	Hendy et a	2008	19909	Mollusca	7805	Bivalvia
Late Pliocene		3.6	2.588	Hendy et a	2008	19909	Mollusca	7805	Bivalvia
Late Pliocene		3.6	2.588	Hendy et a	2008	19909	Mollusca	7805	Bivalvia
Late Pliocene		3.6	2.588	Hendy et a	2008	19909	Mollusca	7805	Bivalvia
Late Pliocene		3.6	2.588	Hendy et a	2008	19909	Mollusca	7805	Bivalvia
Late Pleistocene		0.126	0.0117	Hendy et a	2008	19909	Mollusca	7805	Bivalvia
Late Pleistocene		0.126	0.0117	Hendy et a	2008	19909	Mollusca	7805	Bivalvia
Late Pliocene		3.6	2.588	Hendy et a	2008	19909	Mollusca	7805	Bivalvia
Late Pliocene		3.6	2.588	Hendy et a	2008	19909	Mollusca	7805	Bivalvia
Late Pliocene		3.6	2.588	Hendy et a	2008	19909	Mollusca	7805	Bivalvia
Late Pliocene		3.6	2.588	Hendy et a	2008	19909	Mollusca	7805	Bivalvia
Late Pliocene		3.6	2.588	Hendy et a	2008	19909	Mollusca	7805	Bivalvia
Late Pliocene		3.6	2.588	Hendy et a	2008	19909	Mollusca	7805	Bivalvia
Late Pleistocene		0.126	0.0117	Ray et al.	1967	25320	Mollusca	7805	Bivalvia
Middle Pleistocene		0.781	0.126	Hoerle	1970	19536	Mollusca	7805	Bivalvia
Early Pleistocene		2.588	0.781	Olsson anc	1953	25463	Mollusca	7805	Bivalvia
Early Pleistocene		2.588	0.781	Olsson anc	1953	25463	Mollusca	7805	Bivalvia
Zanclean		5.333	3.6	Campbell a	1995	17558	Mollusca	7805	Bivalvia
Late Pliocene		3.6	2.588	Ward and I	1987	16700	Mollusca	7805	Bivalvia
Early Pleistocene		2.588	0.781	Gardner	1943	16984	Mollusca	7805	Bivalvia
Late Pliocene		3.6	2.588	Gardner	1943	16984	Mollusca	7805	Bivalvia
Late Pliocene		3.6	2.588	Mansfield	1932	17094	Mollusca	7805	Bivalvia
Pleistocene		2.588	0.0117	Hendy et a	2008	19909	Mollusca	7805	Bivalvia
Late Pliocene		3.6	2.588	Hendy et a	2008	19909	Mollusca	7805	Bivalvia
Early Pleistocene		2.588	0.781	DuBar	1971	17556	Mollusca	7805	Bivalvia
Late Pleistocene		0.126	0.0117	Richards	1962	18974	Mollusca	7805	Bivalvia
Early Pleistocene		2.588	0.781	Cooke	1936	31145	Mollusca	7805	Bivalvia
Late Pliocene		3.6	2.588	Cooke	1936	31145	Mollusca	7805	Bivalvia
Early Plioc	Late Plioce	5.333	2.588	Gardner	1943	16984	Mollusca	7805	Bivalvia
Early Pleistocene		2.588	0.781	Gardner	1943	16984	Mollusca	7805	Bivalvia
Pleistocene		2.588	0.0117	Gardner	1943	16984	Mollusca	7805	Bivalvia
Late Pliocene		3.6	2.588	Gardner	1943	16984	Mollusca	7805	Bivalvia
Early Pleistocene		2.588	0.781	Hendy et a	2008	19909	Mollusca	7805	Bivalvia
Early Pleistocene		2.588	0.781	Miller	1990	17561	Mollusca	7805	Bivalvia
Early Pleistocene		2.588	0.781	Miller	1990	17561	Mollusca	7805	Bivalvia
Early Pleistocene		2.588	0.781	Miller	1990	17561	Mollusca	7805	Bivalvia
Late Pleistocene		0.126	0.0117	Richards	1962	18974	Mollusca	7805	Bivalvia
Late Pleistocene		0.126	0.0117	Richards	1962	18974	Mollusca	7805	Bivalvia
Late Pleistocene		0.126	0.0117	Richards	1962	18974	Mollusca	7805	Bivalvia
Gelasian		2.588	1.806	Bailey and	1986	23840	Mollusca	7805	Bivalvia
Early Pleistocene		2.588	0.781	Hendy et a	2008	19909	Mollusca	7805	Bivalvia
Late Pliocene		3.6	2.588	Edwards et	2005	17631	Mollusca	7805	Bivalvia
Late Pliocene		3.6	2.588	Edwards et	2005	17631	Mollusca	7805	Bivalvia
Late Pliocene		3.6	2.588	Edwards et	2005	17631	Mollusca	7805	Bivalvia
Late Pliocene		3.6	2.588	Edwards et	2005	17631	Mollusca	7805	Bivalvia

Late Pliocene	3.6	2.588	Ward	1993	26351	Mollusca	7805	Bivalvia
Late Pliocene	3.6	2.588	Ward	1993	26351	Mollusca	7805	Bivalvia
Late Pliocene	3.6	2.588	Ward	1993	26351	Mollusca	7805	Bivalvia
Late Pliocene	3.6	2.588	Ward	1993	26351	Mollusca	7805	Bivalvia
Late Pliocene	3.6	2.588	Ward	1993	26351	Mollusca	7805	Bivalvia
Late Pliocene	3.6	2.588	Hendy et a	2008	19909	Mollusca	7805	Bivalvia
Late Pleistocene	0.126	0.0117	Hendy et a	2008	19909	Mollusca	7805	Bivalvia
Calabrian	1.806	0.781	DuBar	1962	11119	Mollusca	7805	Bivalvia
Late Pleistocene	0.126	0.0117	Hendy et a	2008	19909	Mollusca	7805	Bivalvia
Late Pleistocene	0.126	0.0117	Hendy et a	2008	19909	Mollusca	7805	Bivalvia
Late Pleistocene	0.126	0.0117	Hendy et a	2008	19909	Mollusca	7805	Bivalvia
Late Pleistocene	0.126	0.0117	Hendy et a	2008	19909	Mollusca	7805	Bivalvia
Late Pliocene	3.6	2.588	Hendy et a	2008	19909	Mollusca	7805	Bivalvia
Early Pleistocene	2.588	0.781	Hendy et a	2008	19909	Mollusca	7805	Bivalvia
Early Pleistocene	2.588	0.781	Hendy et a	2008	19909	Mollusca	7805	Bivalvia
Early Pleistocene	2.588	0.781	Hendy et a	2008	19909	Mollusca	7805	Bivalvia
Late Pleistocene	0.126	0.0117	Hendy et a	2008	19909	Mollusca	7805	Bivalvia
Pleistocene	2.588	0.0117	Hendy et a	2008	19909	Mollusca	7805	Bivalvia
Early Pleistocene	2.588	0.781	Hendy et a	2008	19909	Mollusca	7805	Bivalvia
Pliocene	5.333	2.588	Hendy et a	2008	19909	Mollusca	7805	Bivalvia
Late Pliocene	3.6	2.588	Hendy et a	2008	19909	Mollusca	7805	Bivalvia
Late Pliocene	3.6	2.588	Hendy et a	2008	19909	Mollusca	7805	Bivalvia
Early Pleistocene	2.588	0.781	Hendy et a	2008	19909	Mollusca	7805	Bivalvia
Late Pliocene	3.6	2.588	Edwards et	2005	17631	Mollusca	7805	Bivalvia
Late Pliocene	3.6	2.588	Hendy et a	2008	19909	Mollusca	7805	Bivalvia
Late Pliocene	3.6	2.588	Hendy et a	2008	19909	Mollusca	7805	Bivalvia
Early Pleistocene	2.588	0.781	DuBar	1962	11119	Mollusca	7805	Gastropod
Late Pliocene	3.6	2.588	Mansfield	1930	17060	Mollusca	7805	Gastropod
Late Pliocene	3.6	2.588	Mansfield	1930	17060	Mollusca	7805	Gastropod
Early Pleistocene	2.588	0.781	Olsson anc	1953	25463	Mollusca	7805	Gastropod
Early Pleistocene	2.588	0.781	DuBar	1962	11119	Mollusca	7805	Gastropod
Late Pleistocene	0.126	0.0117	DuBar	1962	11119	Mollusca	7805	Gastropod
Late Pliocene	3.6	2.588	Mansfield	1930	17060	Mollusca	7805	Gastropod
Late Pliocene	3.6	2.588	Mansfield	1930	17060	Mollusca	7805	Gastropod
Pliocene	5.333	2.588	Schroeder	1954	25303	Mollusca	7805	Gastropod
Early Pleistocene	2.588	0.781	Olsson anc	1953	25463	Mollusca	7805	Gastropod
Early Pleistocene	2.588	0.781	Olsson anc	1953	25463	Mollusca	7805	Gastropod
Early Pleistocene	2.588	0.781	Olsson anc	1953	25463	Mollusca	7805	Gastropod
Late Pleistocene	0.126	0.0117	Kittle and I	2010	40884	Mollusca	7805	Gastropod
Late Pliocene	3.6	2.588	Ward	1993	26351	Mollusca	7805	Gastropod
Middle Pleistocene	0.781	0.126	Hoerle	1970	19536	Mollusca	7805	Gastropod
Early Pleistocene	2.588	0.781	Pilsbry	1953	27012	Mollusca	7805	Gastropod
Early Pleistocene	2.588	0.781	DuBar	1962	11119	Mollusca	7805	Gastropod
Early Pleistocene	2.588	0.781	DuBar	1962	11119	Mollusca	7805	Gastropod
Late Pliocene	3.6	2.588	Mansfield	1930	17060	Mollusca	7805	Gastropod
Late Pleistocene	0.126	0.0117	DuBar	1958	25514	Mollusca	7805	Gastropod
Early Pleistocene	2.588	0.781	DuBar	1958	25514	Mollusca	7805	Gastropod

Pleistocene		2.588	0.0117	Auffenberg	2006	82817	Mollusca	7805	Gastropod
Pleistocene		2.588	0.0117	Auffenberg	2006	82817	Mollusca	7805	Gastropod
Pliocene		5.333	2.588	Aldrich	1911	53829	Mollusca	7805	Gastropod
Pliocene		5.333	2.588	Aldrich	1911	53829	Mollusca	7805	Gastropod
Pliocene		5.333	2.588	Aldrich	1911	53829	Mollusca	7805	Gastropod
Late Pleistocene		0.126	0.0117	Kittle and I	2010	40884	Mollusca	7805	Gastropod
Pleistocene		2.588	0.0117	Auffenberg	2006	82817	Mollusca	7805	Gastropod
Pleistocene		2.588	0.0117	Auffenberg	2006	82817	Mollusca	7805	Gastropod
Pleistocene		2.588	0.0117	Auffenberg	2006	82817	Mollusca	7805	Gastropod
Pleistocene		2.588	0.0117	Auffenberg	2006	82817	Mollusca	7805	Gastropod
Pleistocene		2.588	0.0117	Auffenberg	2006	82817	Mollusca	7805	Gastropod
Pleistocene		2.588	0.0117	Auffenberg	2006	82817	Mollusca	7805	Gastropod
Late Pliocene		3.6	2.588	Petuch anc	2007	26813	Mollusca	7805	Gastropod
Pliocene	Pleistocene	5.333	0.0117	DuBar	1962	11119	Mollusca	7805	Gastropod
Late Pliocene		3.6	2.588	Mansfield	1930	17060	Mollusca	7805	Gastropod
Late Pliocene		3.6	2.588	Mansfield	1930	17060	Mollusca	7805	Gastropod
Late Pliocene		3.6	2.588	Mansfield	1930	17060	Mollusca	7805	Gastropod
Late Pleistocene		0.126	0.0117	Kittle and I	2010	40884	Mollusca	7805	Gastropod
Zanclean		5.333	3.6	Campbell a	1995	17558	Mollusca	7805	Gastropod
Late Pleistocene		0.126	0.0117	Richards	1962	18974	Mollusca	7805	Gastropod
Pleistocene		2.588	0.0117	Holmes	1860	64712	Mollusca	7805	Gastropod
Pleistocene		2.588	0.0117	Holmes	1860	64712	Mollusca	7805	Gastropod
Late Pliocene		3.6	2.588	Gardner	1948	17048	Mollusca	7805	Gastropod
Late Pliocene		3.6	2.588	Ward	1993	26351	Mollusca	7805	Gastropod
Middle Pleistocene		0.781	0.126	Hoerle	1970	19536	Mollusca	7805	Gastropod
Early Pleistocene		2.588	0.781	Olsson anc	1953	25463	Mollusca	7805	Gastropod
Late Pliocene		3.6	2.588	Ward and I	1987	16700	Mollusca	7805	Gastropod
Late Pliocene		3.6	2.588	Ward and I	1987	16700	Mollusca	7805	Gastropod
Late Pliocene		3.6	2.588	Ward and I	1987	16700	Mollusca	7805	Gastropod
Early Pleistocene		2.588	0.781	Ward and I	1987	16700	Mollusca	7805	Gastropod
Early Pleistocene		2.588	0.781	Gardner	1948	17048	Mollusca	7805	Gastropod
Early Pleistocene		2.588	0.781	Gardner	1948	17048	Mollusca	7805	Gastropod
Early Pleistocene		2.588	0.781	Gardner	1948	17048	Mollusca	7805	Gastropod
Gelasian		2.588	1.806	Bailey and	1986	23840	Mollusca	7805	Gastropod
Late Pliocene		3.6	2.588	Hendy et a	2008	19909	Mollusca	7805	Gastropod
Early Pleistocene		2.588	0.781	DuBar	1962	11119	Mollusca	7805	Gastropod
Early Pleistocene		2.588	0.781	DuBar	1962	11119	Mollusca	7805	Gastropod
Pliocene	Pleistocene	5.333	0.0117	DuBar	1962	11119	Mollusca	7805	Gastropod
Late Pleistocene		0.126	0.0117	Petuch	2004	18040	Mollusca	7805	Gastropod
Middle Pleistocene		0.781	0.126	McGinty	1970	19114	Mollusca	7805	Gastropod
Late Pleistocene		0.126	0.0117	Petuch anc	2007	26813	Mollusca	7805	Gastropod
Late Pleistocene		0.126	0.0117	Petuch anc	2007	26813	Mollusca	7805	Gastropod
Middle Pleistocene		0.781	0.126	Petuch anc	2007	26813	Mollusca	7805	Gastropod
Middle Pleistocene		0.781	0.126	Petuch anc	2007	26813	Mollusca	7805	Gastropod
Middle Pleistocene		0.781	0.126	Petuch anc	2007	26813	Mollusca	7805	Gastropod
Early Pleistocene		2.588	0.781	Olsson	1967	19826	Mollusca	7805	Gastropod
Early Pleistocene		2.588	0.781	Petuch anc	2007	26813	Mollusca	7805	Gastropod

Late Plioce	Early Pleist	3.6	0.781	Petuch anc	2007	26813	Mollusca	7805	Gastropod
Late Plioce	Early Pleist	3.6	0.781	Petuch anc	2007	26813	Mollusca	7805	Gastropod
Late Pliocene		3.6	2.588	Petuch anc	2007	26813	Mollusca	7805	Gastropod
Late Pliocene		3.6	2.588	Petuch anc	2007	26813	Mollusca	7805	Gastropod
Late Pliocene		3.6	2.588	Petuch anc	2007	26813	Mollusca	7805	Gastropod
Late Pliocene		3.6	2.588	Petuch anc	2007	26813	Mollusca	7805	Gastropod
Early Pleistocene		2.588	0.781	Olsson anc	1953	25463	Mollusca	7805	Gastropod
Early Pleistocene		2.588	0.781	Olsson anc	1953	25463	Mollusca	7805	Gastropod
Late Pliocene		3.6	2.588	Petuch	1988	31112	Mollusca	7805	Gastropod
Early Pleistocene		2.588	0.781	Petuch	1988	31112	Mollusca	7805	Gastropod
Middle Pleistocene		0.781	0.126	Petuch	1988	31112	Mollusca	7805	Gastropod
Middle Pleistocene		0.781	0.126	Petuch	1988	31112	Mollusca	7805	Gastropod
Late Pleistocene		0.126	0.0117	Kittle and I	2010	40884	Mollusca	7805	Gastropod
Late Pleistocene		0.126	0.0117	Kittle and I	2010	40884	Mollusca	7805	Gastropod
Late Pleistocene		0.126	0.0117	Kittle and I	2010	40884	Mollusca	7805	Gastropod
Late Pleistocene		0.126	0.0117	Kittle and I	2010	40884	Mollusca	7805	Gastropod
Early Pleistocene		2.588	0.781	DuBar	1962	11119	Mollusca	7805	Gastropod
Early Pleistocene		2.588	0.781	DuBar	1962	11119	Mollusca	7805	Gastropod
Pliocene	Pleistocene	5.333	0.0117	DuBar	1962	11119	Mollusca	7805	Gastropod
Pliocene	Pleistocene	5.333	0.0117	DuBar	1962	11119	Mollusca	7805	Gastropod
Pliocene	Pleistocene	5.333	0.0117	DuBar	1962	11119	Mollusca	7805	Gastropod
Pliocene	Pleistocene	5.333	0.0117	DuBar	1962	11119	Mollusca	7805	Gastropod
Late Pliocene		3.6	2.588	Mansfield	1930	17060	Mollusca	7805	Gastropod
Early Pleistocene		2.588	0.781	Portell et a	1992	17634	Mollusca	7805	Gastropod
Middle Pleistocene		0.781	0.126	Portell et a	1992	17634	Mollusca	7805	Gastropod
Middle Pleistocene		0.781	0.126	Portell et a	1992	17634	Mollusca	7805	Gastropod
Late Pleistocene		0.126	0.0117	Portell et a	1992	17634	Mollusca	7805	Gastropod
Middle Pleistocene		0.781	0.126	McGinty	1970	19114	Mollusca	7805	Gastropod
Middle Pleistocene		0.781	0.126	McGinty	1970	19114	Mollusca	7805	Gastropod
Pliocene		5.333	2.588	Schroeder	1954	25303	Mollusca	7805	Gastropod
Early Pleistocene		2.588	0.781	Olsson anc	1953	25463	Mollusca	7805	Gastropod
Early Pleistocene		2.588	0.781	Olsson anc	1953	25463	Mollusca	7805	Gastropod
Early Pleistocene		2.588	0.781	Olsson anc	1953	25463	Mollusca	7805	Gastropod
Early Pleistocene		2.588	0.781	Olsson anc	1953	25463	Mollusca	7805	Gastropod
Late Pleistocene		0.126	0.0117	Petuch anc	2007	26813	Mollusca	7805	Gastropod
Late Pliocene		3.6	2.588	Olsson	1967	19826	Mollusca	7805	Gastropod
Late Pliocene		3.6	2.588	Olsson	1967	19826	Mollusca	7805	Gastropod
Late Pliocene		3.6	2.588	Olsson	1967	19826	Mollusca	7805	Gastropod
Late Pliocene		3.6	2.588	Petuch anc	2007	26813	Mollusca	7805	Gastropod
Early Pleistocene		2.588	0.781	Olsson anc	1953	25463	Mollusca	7805	Gastropod
Early Pleistocene		2.588	0.781	Olsson anc	1953	25463	Mollusca	7805	Gastropod
Late Pliocene		3.6	2.588	Petuch anc	2007	26813	Mollusca	7805	Gastropod
Late Pliocene		3.6	2.588	Petuch anc	2007	26813	Mollusca	7805	Gastropod
Late Pliocene		3.6	2.588	Petuch anc	2007	26813	Mollusca	7805	Gastropod
Late Pliocene		3.6	2.588	Petuch anc	2007	26813	Mollusca	7805	Gastropod
Late Pliocene		3.6	2.588	Petuch anc	2007	26813	Mollusca	7805	Gastropod
Early Pleistocene		2.588	0.781	DuBar	1958	25514	Mollusca	7805	Gastropod

Early Pleistocene		2.588	0.781	Hendy et a	2008	19909	Mollusca	7805	Gastropod
Early Pleistocene		2.588	0.781	Hendy et a	2008	19909	Mollusca	7805	Gastropod
Late Pliocene		3.6	2.588	Hendy et a	2008	19909	Mollusca	7805	Gastropod
Late Pleistocene		0.126	0.0117	Hendy et a	2008	19909	Mollusca	7805	Gastropod
Early Pleistocene		2.588	0.781	Hendy et a	2008	19909	Mollusca	7805	Gastropod
Late Pliocene		3.6	2.588	Petuch	1988	31112	Mollusca	7805	Gastropod
Late Pleistocene		0.126	0.0117	Kittle and I	2010	40884	Mollusca	7805	Gastropod
Late Pleistocene		0.126	0.0117	Kittle and I	2010	40884	Mollusca	7805	Gastropod
Late Pleistocene		0.126	0.0117	Kittle and I	2010	40884	Mollusca	7805	Gastropod
Late Pleistocene		0.126	0.0117	Kittle and I	2010	40884	Mollusca	7805	Gastropod
Pliocene		5.333	2.588	Petuch	1986	47440	Mollusca	7805	Gastropod
Pliocene		5.333	2.588	Petuch	1986	47440	Mollusca	7805	Gastropod
Late Pleistocene		0.126	0.0117	DuBar	1971	17556	Mollusca	7805	Gastropod
Late Pleistocene		0.126	0.0117	DuBar	1971	17556	Mollusca	7805	Gastropod
Late Pliocene		3.6	2.588	Campbell a	1995	17558	Mollusca	7805	Gastropod
Late Pleistocene		0.126	0.0117	Richards	1962	18974	Mollusca	7805	Gastropod
Late Pleistocene		0.126	0.0117	Richards	1962	18974	Mollusca	7805	Gastropod
Late Pleistocene		0.126	0.0117	Richards	1962	18974	Mollusca	7805	Gastropod
Late Pleistocene		0.126	0.0117	Richards	1962	18974	Mollusca	7805	Gastropod
Late Pleistocene		0.126	0.0117	Richards	1962	18974	Mollusca	7805	Gastropod
Late Pliocene		3.6	2.588	Gardner	1948	17048	Mollusca	7805	Gastropod
Late Pliocene		3.6	2.588	Gardner	1948	17048	Mollusca	7805	Gastropod
Early Plioce	Late Plioce	5.333	2.588	Gardner	1948	17048	Mollusca	7805	Gastropod
Late Pliocene		3.6	2.588	Gardner	1948	17048	Mollusca	7805	Gastropod
Late Pliocene		3.6	2.588	Gardner	1948	17048	Mollusca	7805	Gastropod
Late Pliocene		3.6	2.588	Gardner	1948	17048	Mollusca	7805	Gastropod
Early Pleistocene		2.588	0.781	Gardner	1948	17048	Mollusca	7805	Gastropod
Late Pleistocene		0.126	0.0117	Richards	1962	18974	Mollusca	7805	Gastropod
Late Pleistocene		0.126	0.0117	Spencer ar	1987	15525	Mollusca	7805	Gastropod
Late Pleistocene		0.126	0.0117	Spencer ar	1987	15525	Mollusca	7805	Gastropod
Late Pleistocene		0.126	0.0117	Spencer ar	1987	15525	Mollusca	7805	Gastropod
Late Pliocene		3.6	2.588	Ward	1993	26351	Mollusca	7805	Gastropod
Late Pliocene		3.6	2.588	Ward	1993	26351	Mollusca	7805	Gastropod
Late Pliocene		3.6	2.588	Ward	1993	26351	Mollusca	7805	Gastropod
Late Pliocene		3.6	2.588	Hendy et a	2008	19909	Mollusca	7805	Gastropod
Late Pliocene		3.6	2.588	Hendy et a	2008	19909	Mollusca	7805	Gastropod
Late Pliocene		3.6	2.588	Hendy et a	2008	19909	Mollusca	7805	Gastropod
Late Pliocene		3.6	2.588	Hendy et a	2008	19909	Mollusca	7805	Gastropod
Late Pliocene		3.6	2.588	Hendy et a	2008	19909	Mollusca	7805	Gastropod
Pliocene		5.333	2.588	Petuch	1988	31112	Mollusca	7805	Gastropod
Early Pleistocene		2.588	0.781	DuBar	1962	11119	Mollusca	7805	Gastropod
Early Pleistocene		2.588	0.781	DuBar	1962	11119	Mollusca	7805	Gastropod
Calabrian		1.806	0.781	DuBar	1962	11119	Mollusca	7805	Gastropod
Late Pleistocene		0.126	0.0117	DuBar	1962	11119	Mollusca	7805	Gastropod
Early Pleistocene		2.588	0.781	DuBar	1962	11119	Mollusca	7805	Gastropod
Pliocene	Pleistocen	5.333	0.0117	DuBar	1962	11119	Mollusca	7805	Gastropod
Pliocene	Pleistocen	5.333	0.0117	DuBar	1962	11119	Mollusca	7805	Gastropod

Early Pleistocene	2.588	0.781	Portell et a	1992	17634	Mollusca	7805	Gastropod
Middle Pleistocene	0.781	0.126	Portell et a	1992	17634	Mollusca	7805	Gastropod
Late Pleistocene	0.126	0.0117	Portell et a	1992	17634	Mollusca	7805	Gastropod
Late Pleistocene	0.126	0.0117	Hendy et a	2008	19909	Mollusca	7805	Gastropod
Late Pleistocene	0.126	0.0117	Hendy et a	2008	19909	Mollusca	7805	Gastropod
Early Pleistocene	2.588	0.781	Olsson and	1953	25463	Mollusca	7805	Gastropod
Early Pleistocene	2.588	0.781	Olsson and	1953	25463	Mollusca	7805	Gastropod
Late Pleistocene	0.126	0.0117	Petuch and	2007	26813	Mollusca	7805	Gastropod
Late Pliocene	3.6	2.588	Petuch and	2007	26813	Mollusca	7805	Gastropod
Early Pleistocene	2.588	0.781	Hendy et a	2008	19909	Mollusca	7805	Gastropod
Early Pleistocene	2.588	0.781	Hendy et a	2008	19909	Mollusca	7805	Gastropod
Late Pleistocene	0.126	0.0117	Hendy et a	2008	19909	Mollusca	7805	Gastropod
Early Pleistocene	2.588	0.781	DuBar	1958	25514	Mollusca	7805	Gastropod
Late Pleistocene	0.126	0.0117	Hendy et a	2008	19909	Mollusca	7805	Gastropod
Early Pleistocene	2.588	0.781	Hendy et a	2008	19909	Mollusca	7805	Gastropod
Early Pleistocene	2.588	0.781	Hendy et a	2008	19909	Mollusca	7805	Gastropod
Early Pleistocene	2.588	0.781	Hendy et a	2008	19909	Mollusca	7805	Gastropod
Early Pleistocene	2.588	0.781	Hendy et a	2008	19909	Mollusca	7805	Gastropod
Late Pleistocene	0.126	0.0117	Petuch	1988	31112	Mollusca	7805	Gastropod
Late Pleistocene	0.126	0.0117	Kittle and I	2010	40884	Mollusca	7805	Gastropod
Late Pliocene	3.6	2.588	Beu	2010	30037	Mollusca	7805	Gastropod
Late Pliocene	3.6	2.588	Petuch and	2007	26813	Mollusca	7805	Gastropod
Late Pliocene	3.6	2.588	Beu	2010	30037	Mollusca	7805	Gastropod
Early Pleistocene	2.588	0.781	DuBar	1962	11119	Mollusca	7805	Gastropod
Early Pleistocene	2.588	0.781	DuBar	1962	11119	Mollusca	7805	Gastropod
Early Pleistocene	2.588	0.781	DuBar	1962	11119	Mollusca	7805	Gastropod
Early Pleistocene	2.588	0.781	DuBar	1962	11119	Mollusca	7805	Gastropod
Early Pleistocene	2.588	0.781	DuBar	1962	11119	Mollusca	7805	Gastropod
Early Pleistocene	2.588	0.781	DuBar	1962	11119	Mollusca	7805	Gastropod
Calabrian	1.806	0.781	DuBar	1962	11119	Mollusca	7805	Gastropod
Calabrian	1.806	0.781	DuBar	1962	11119	Mollusca	7805	Gastropod
Calabrian	1.806	0.781	DuBar	1962	11119	Mollusca	7805	Gastropod
Calabrian	1.806	0.781	DuBar	1962	11119	Mollusca	7805	Gastropod
Calabrian	1.806	0.781	DuBar	1962	11119	Mollusca	7805	Gastropod
Calabrian	1.806	0.781	DuBar	1962	11119	Mollusca	7805	Gastropod
Calabrian	1.806	0.781	DuBar	1962	11119	Mollusca	7805	Gastropod
Early Pleistocene	2.588	0.781	DuBar	1962	11119	Mollusca	7805	Gastropod
Early Pleistocene	2.588	0.781	DuBar	1962	11119	Mollusca	7805	Gastropod
Early Pleistocene	2.588	0.781	DuBar	1962	11119	Mollusca	7805	Gastropod
Late Pliocene	3.6	2.588	Mansfield	1930	17060	Mollusca	7805	Gastropod
Late Pliocene	3.6	2.588	Mansfield	1930	17060	Mollusca	7805	Gastropod
Late Pliocene	3.6	2.588	Mansfield	1930	17060	Mollusca	7805	Gastropod
Late Pliocene	3.6	2.588	Mansfield	1930	17060	Mollusca	7805	Gastropod
Late Pliocene	3.6	2.588	Mansfield	1930	17060	Mollusca	7805	Gastropod
Late Pliocene	3.6	2.588	Mansfield	1930	17060	Mollusca	7805	Gastropod
Middle Pleistocene	0.781	0.126	Portell et a	1992	17634	Mollusca	7805	Gastropod
Middle Pleistocene	0.781	0.126	Portell et a	1992	17634	Mollusca	7805	Gastropod

Middle Pleistocene		0.781	0.126	Portell et a	1992	17634	Mollusca	7805	Gastropod
Middle Pleistocene		0.781	0.126	Portell et a	1992	17634	Mollusca	7805	Gastropod
Early Pleistocene		2.588	0.781	Olsson anc	1953	25463	Mollusca	7805	Gastropod
Early Pleistocene		2.588	0.781	Olsson anc	1953	25463	Mollusca	7805	Gastropod
Early Pleistocene		2.588	0.781	Olsson anc	1953	25463	Mollusca	7805	Gastropod
Early Pleistocene		2.588	0.781	Olsson anc	1953	25463	Mollusca	7805	Gastropod
Early Pleistocene		2.588	0.781	Olsson anc	1953	25463	Mollusca	7805	Gastropod
Early Pleistocene		2.588	0.781	Olsson anc	1953	25463	Mollusca	7805	Gastropod
Early Pleistocene		2.588	0.781	Olsson anc	1953	25463	Mollusca	7805	Gastropod
Early Pleistocene		2.588	0.781	Olsson anc	1953	25463	Mollusca	7805	Gastropod
Early Pleistocene		2.588	0.781	Olsson anc	1953	25463	Mollusca	7805	Gastropod
Early Pleistocene		2.588	0.781	Olsson anc	1953	25463	Mollusca	7805	Gastropod
Early Pleistocene		2.588	0.781	Olsson anc	1953	25463	Mollusca	7805	Gastropod
Early Pleistocene		2.588	0.781	Olsson anc	1953	25463	Mollusca	7805	Gastropod
Late Pleistocene		0.126	0.0117	Kittle and I	2010	40884	Mollusca	7805	Gastropod
Late Pleistocene		0.126	0.0117	Kittle and I	2010	40884	Mollusca	7805	Gastropod
Late Pleistocene		0.126	0.0117	Kittle and I	2010	40884	Mollusca	7805	Gastropod
Late Pleistocene		0.126	0.0117	Kittle and I	2010	40884	Mollusca	7805	Gastropod
Early Pleistocene		2.588	0.781	DuBar	1971	17556	Mollusca	7805	Gastropod
Early Pleistocene		2.588	0.781	Cooke	1936	31145	Mollusca	7805	Gastropod
Early Pleistocene		2.588	0.781	Cooke	1936	31145	Mollusca	7805	Gastropod
Late Pliocene		3.6	2.588	Ward and I	1987	16700	Mollusca	7805	Gastropod
Late Pliocene		3.6	2.588	Ward and I	1987	16700	Mollusca	7805	Gastropod
Late Pliocene		3.6	2.588	Ward and I	1987	16700	Mollusca	7805	Gastropod
Late Pliocene		3.6	2.588	Ward and I	1987	16700	Mollusca	7805	Gastropod
Early Pleistocene		2.588	0.781	Ward and I	1987	16700	Mollusca	7805	Gastropod
Early Pleistocene		2.588	0.781	Ward and I	1987	16700	Mollusca	7805	Gastropod
Early Pleistocene		2.588	0.781	Ward and I	1987	16700	Mollusca	7805	Gastropod
Early Pleistocene		2.588	0.781	Gardner	1948	17048	Mollusca	7805	Gastropod
Early Pleistocene		2.588	0.781	Gardner	1948	17048	Mollusca	7805	Gastropod
Early Plioc	Late Plioce	5.333	2.588	Gardner	1948	17048	Mollusca	7805	Gastropod
Early Plioc	Late Plioce	5.333	2.588	Gardner	1948	17048	Mollusca	7805	Gastropod
Late Pliocene		3.6	2.588	Gardner	1948	17048	Mollusca	7805	Gastropod
Late Pleistocene		0.126	0.0117	Spencer ar	1987	15525	Mollusca	7805	Gastropod
Late Pleistocene		0.126	0.0117	Sellards	1917	9011	Mollusca	7805	Gastropod
Late Pleistocene		0.126	0.0117	Sellards	1917	9011	Mollusca	7805	Gastropod
Early Pleistocene		2.588	0.781	Mansfield	1932	11083	Mollusca	7805	Gastropod
Early Pleistocene		2.588	0.781	Mansfield	1932	11083	Mollusca	7805	Gastropod
Early Pleistocene		2.588	0.781	DuBar	1962	11119	Mollusca	7805	Gastropod
Early Pleistocene		2.588	0.781	DuBar	1962	11119	Mollusca	7805	Gastropod
Early Pleistocene		2.588	0.781	DuBar	1962	11119	Mollusca	7805	Gastropod
Early Pleistocene		2.588	0.781	DuBar	1962	11119	Mollusca	7805	Gastropod
Early Pleistocene		2.588	0.781	DuBar	1962	11119	Mollusca	7805	Gastropod
Early Pleistocene		2.588	0.781	DuBar	1962	11119	Mollusca	7805	Gastropod
Early Pleistocene		2.588	0.781	DuBar	1962	11119	Mollusca	7805	Gastropod
Early Pleistocene		2.588	0.781	DuBar	1962	11119	Mollusca	7805	Gastropod
Early Pleistocene		2.588	0.781	DuBar	1962	11119	Mollusca	7805	Gastropod

Early Pleistocene		2.588	0.781	DuBar	1962	11119	Mollusca	7805 Gastropod
Early Pleistocene		2.588	0.781	DuBar	1962	11119	Mollusca	7805 Gastropod
Early Pleistocene		2.588	0.781	DuBar	1962	11119	Mollusca	7805 Gastropod
Early Pleistocene		2.588	0.781	DuBar	1962	11119	Mollusca	7805 Gastropod
Early Pleistocene		2.588	0.781	DuBar	1962	11119	Mollusca	7805 Gastropod
Calabrian		1.806	0.781	DuBar	1962	11119	Mollusca	7805 Gastropod
Calabrian		1.806	0.781	DuBar	1962	11119	Mollusca	7805 Gastropod
Calabrian		1.806	0.781	DuBar	1962	11119	Mollusca	7805 Gastropod
Calabrian		1.806	0.781	DuBar	1962	11119	Mollusca	7805 Gastropod
Late Pleistocene		0.126	0.0117	DuBar	1962	11119	Mollusca	7805 Gastropod
Early Pleistocene		2.588	0.781	DuBar	1962	11119	Mollusca	7805 Gastropod
Early Pleistocene		2.588	0.781	DuBar	1962	11119	Mollusca	7805 Gastropod
Early Pleistocene		2.588	0.781	DuBar	1962	11119	Mollusca	7805 Gastropod
Late Pleistocene		0.126	0.0117	DuBar	1962	11119	Mollusca	7805 Gastropod
Pliocene	Pleistocen	5.333	0.0117	DuBar	1962	11119	Mollusca	7805 Gastropod
Pliocene	Pleistocen	5.333	0.0117	DuBar	1962	11119	Mollusca	7805 Gastropod
Pliocene	Pleistocen	5.333	0.0117	DuBar	1962	11119	Mollusca	7805 Gastropod
Pliocene	Pleistocen	5.333	0.0117	DuBar	1962	11119	Mollusca	7805 Gastropod
Pliocene	Pleistocen	5.333	0.0117	DuBar	1962	11119	Mollusca	7805 Gastropod
Late Pliocene		3.6	2.588	Mansfield	1930	17060	Mollusca	7805 Gastropod
Late Pliocene		3.6	2.588	Mansfield	1930	17060	Mollusca	7805 Gastropod
Late Pliocene		3.6	2.588	Mansfield	1930	17060	Mollusca	7805 Gastropod
Late Pliocene		3.6	2.588	Mansfield	1930	17060	Mollusca	7805 Gastropod
Late Pliocene		3.6	2.588	Mansfield	1930	17060	Mollusca	7805 Gastropod
Late Pliocene		3.6	2.588	Mansfield	1930	17060	Mollusca	7805 Gastropod
Late Pliocene		3.6	2.588	Mansfield	1930	17060	Mollusca	7805 Gastropod
Late Pliocene		3.6	2.588	Mansfield	1930	17060	Mollusca	7805 Gastropod
Late Pliocene		3.6	2.588	Mansfield	1930	17060	Mollusca	7805 Gastropod
Late Pliocene		3.6	2.588	Mansfield	1930	17060	Mollusca	7805 Gastropod
Late Pliocene		3.6	2.588	Mansfield	1930	17060	Mollusca	7805 Gastropod
Late Pliocene		3.6	2.588	Mansfield	1930	17060	Mollusca	7805 Gastropod
Late Pliocene		3.6	2.588	Mansfield	1930	17060	Mollusca	7805 Gastropod
Late Pliocene		3.6	2.588	Mansfield	1930	17060	Mollusca	7805 Gastropod
Late Pliocene		3.6	2.588	Mansfield	1930	17060	Mollusca	7805 Gastropod
Late Pliocene		3.6	2.588	Mansfield	1930	17060	Mollusca	7805 Gastropod
Late Pleistocene		0.126	0.0117	Portell et a	1992	17634	Mollusca	7805 Gastropod
Late Pleistocene		0.126	0.0117	Portell et a	1992	17634	Mollusca	7805 Gastropod
Late Pleistocene		0.126	0.0117	Portell et a	1992	17634	Mollusca	7805 Gastropod
Late Pleistocene		0.126	0.0117	Portell et a	1992	17634	Mollusca	7805 Gastropod
Early Pleistocene		2.588	0.781	Portell et a	1992	17634	Mollusca	7805 Gastropod
Early Pleistocene		2.588	0.781	Portell et a	1992	17634	Mollusca	7805 Gastropod
Early Pleistocene		2.588	0.781	Portell et a	1992	17634	Mollusca	7805 Gastropod
Early Pleistocene		2.588	0.781	Portell et a	1992	17634	Mollusca	7805 Gastropod
Middle Pleistocene		0.781	0.126	Portell et a	1992	17634	Mollusca	7805 Gastropod
Middle Pleistocene		0.781	0.126	Portell et a	1992	17634	Mollusca	7805 Gastropod
Middle Pleistocene		0.781	0.126	Portell et a	1992	17634	Mollusca	7805 Gastropod
Middle Pleistocene		0.781	0.126	Portell et a	1992	17634	Mollusca	7805 Gastropod

Middle Pleistocene	0.781	0.126	Portell et a	1992	17634	Mollusca	7805	Gastropod
Late Pleistocene	0.126	0.0117	Hendy et a	2008	19909	Mollusca	7805	Gastropod
Late Pleistocene	0.126	0.0117	Hendy et a	2008	19909	Mollusca	7805	Gastropod
Late Pleistocene	0.126	0.0117	Hendy et a	2008	19909	Mollusca	7805	Gastropod
Late Pleistocene	0.126	0.0117	Hendy et a	2008	19909	Mollusca	7805	Gastropod
Early Pleistocene	2.588	0.781	Schroeder	1954	25303	Mollusca	7805	Gastropod
Pliocene	5.333	2.588	Schroeder	1954	25303	Mollusca	7805	Gastropod
Late Pleistocene	0.126	0.0117	Lovejoy	1992	26806	Mollusca	7805	Gastropod
Late Pliocene	3.6	2.588	Petuch anc	2007	26813	Mollusca	7805	Gastropod
Early Pleistocene	2.588	0.781	Hendy et a	2008	19909	Mollusca	7805	Gastropod
Early Pleistocene	2.588	0.781	Hendy et a	2008	19909	Mollusca	7805	Gastropod
Early Pleistocene	2.588	0.781	Hendy et a	2008	19909	Mollusca	7805	Gastropod
Early Pleistocene	2.588	0.781	Hendy et a	2008	19909	Mollusca	7805	Gastropod
Late Pleistocene	0.126	0.0117	Hendy et a	2008	19909	Mollusca	7805	Gastropod
Late Pleistocene	0.126	0.0117	Hendy et a	2008	19909	Mollusca	7805	Gastropod
Late Pleistocene	0.126	0.0117	Hendy et a	2008	19909	Mollusca	7805	Gastropod
Early Pleistocene	2.588	0.781	Olsson anc	1953	25463	Mollusca	7805	Gastropod
Early Pleistocene	2.588	0.781	Olsson anc	1953	25463	Mollusca	7805	Gastropod
Early Pleistocene	2.588	0.781	DuBar	1958	25514	Mollusca	7805	Gastropod
Late Pleistocene	0.126	0.0117	DuBar	1958	25514	Mollusca	7805	Gastropod
Late Pliocene	3.6	2.588	Hendy et a	2008	19909	Mollusca	7805	Gastropod
Early Pleistocene	2.588	0.781	Hendy et a	2008	19909	Mollusca	7805	Gastropod
Early Pleistocene	2.588	0.781	Hendy et a	2008	19909	Mollusca	7805	Gastropod
Early Pleistocene	2.588	0.781	Hendy et a	2008	19909	Mollusca	7805	Gastropod
Early Pleistocene	2.588	0.781	Hendy et a	2008	19909	Mollusca	7805	Gastropod
Early Pleistocene	2.588	0.781	Hendy et a	2008	19909	Mollusca	7805	Gastropod
Early Pleistocene	2.588	0.781	Hendy et a	2008	19909	Mollusca	7805	Gastropod
Late Pliocene	3.6	2.588	Hendy et a	2008	19909	Mollusca	7805	Gastropod
Late Pliocene	3.6	2.588	Hendy et a	2008	19909	Mollusca	7805	Gastropod
Late Pliocene	3.6	2.588	Hendy et a	2008	19909	Mollusca	7805	Gastropod
Late Pliocene	3.6	2.588	Hendy et a	2008	19909	Mollusca	7805	Gastropod
Late Pliocene	3.6	2.588	Hendy et a	2008	19909	Mollusca	7805	Gastropod
Middle Pleistocene	0.781	0.126	Hendy et a	2008	19909	Mollusca	7805	Gastropod
Middle Pleistocene	0.781	0.126	Hendy et a	2008	19909	Mollusca	7805	Gastropod
Middle Pleistocene	0.781	0.126	Hendy et a	2008	19909	Mollusca	7805	Gastropod
Middle Pleistocene	0.781	0.126	Hendy et a	2008	19909	Mollusca	7805	Gastropod
Late Pleistocene	0.126	0.0117	Hendy et a	2008	19909	Mollusca	7805	Gastropod
Late Pleistocene	0.126	0.0117	Hendy et a	2008	19909	Mollusca	7805	Gastropod
Early Pleistocene	2.588	0.781	Hendy et a	2008	19909	Mollusca	7805	Gastropod
Early Pleistocene	2.588	0.781	Hendy et a	2008	19909	Mollusca	7805	Gastropod
Early Pleistocene	2.588	0.781	Hendy et a	2008	19909	Mollusca	7805	Gastropod
Early Pleistocene	2.588	0.781	Hendy et a	2008	19909	Mollusca	7805	Gastropod
Early Pleistocene	2.588	0.781	Hendy et a	2008	19909	Mollusca	7805	Gastropod
Early Pleistocene	2.588	0.781	Hendy et a	2008	19909	Mollusca	7805	Gastropod
Early Pleistocene	2.588	0.781	Hendy et a	2008	19909	Mollusca	7805	Gastropod
Early Pleistocene	2.588	0.781	Olsson anc	1953	25463	Mollusca	7805	Gastropod
Early Pleistocene	2.588	0.781	Olsson anc	1953	25463	Mollusca	7805	Gastropod

Early Pleistocene	2.588	0.781	Olsson anc	1953	25463	Mollusca	7805	Gastropod
Early Pleistocene	2.588	0.781	Olsson anc	1953	25463	Mollusca	7805	Gastropod
Early Pleistocene	2.588	0.781	Olsson anc	1953	25463	Mollusca	7805	Gastropod
Early Pleistocene	2.588	0.781	Olsson anc	1953	25463	Mollusca	7805	Gastropod
Early Pleistocene	2.588	0.781	Olsson anc	1953	25463	Mollusca	7805	Gastropod
Early Pleistocene	2.588	0.781	DuBar	1958	25514	Mollusca	7805	Gastropod
Early Pleistocene	2.588	0.781	DuBar	1958	25514	Mollusca	7805	Gastropod
Early Pleistocene	2.588	0.781	DuBar	1958	25514	Mollusca	7805	Gastropod
Late Plioce Early Pleist	3.6	0.781	Vokes	1975	31302	Mollusca	7805	Gastropod
Late Pliocene	3.6	2.588	Smith	1941	32333	Mollusca	7805	Gastropod
Late Pleistocene	0.126	0.0117	Kittle and I	2010	40884	Mollusca	7805	Gastropod
Late Pleistocene	0.126	0.0117	Kittle and I	2010	40884	Mollusca	7805	Gastropod
Late Pleistocene	0.126	0.0117	Kittle and I	2010	40884	Mollusca	7805	Gastropod
Late Pleistocene	0.126	0.0117	Kittle and I	2010	40884	Mollusca	7805	Gastropod
Late Pleistocene	0.126	0.0117	Kittle and I	2010	40884	Mollusca	7805	Gastropod
Late Pleistocene	0.126	0.0117	Kittle and I	2010	40884	Mollusca	7805	Gastropod
Late Pleistocene	0.126	0.0117	Kittle and I	2010	40884	Mollusca	7805	Gastropod
Pliocene	5.333	2.588	Petuch	1986	47440	Mollusca	7805	Gastropod
Pliocene	5.333	2.588	Petuch	1986	47440	Mollusca	7805	Gastropod
Pliocene	5.333	2.588	Petuch	1986	47440	Mollusca	7805	Gastropod
Pliocene	5.333	2.588	Petuch	1986	47440	Mollusca	7805	Gastropod
Pliocene	5.333	2.588	Petuch	1986	47440	Mollusca	7805	Gastropod
Pliocene	5.333	2.588	Petuch	1986	47440	Mollusca	7805	Gastropod
Pliocene	5.333	2.588	Petuch	1986	47440	Mollusca	7805	Gastropod
Pliocene	5.333	2.588	Petuch	1986	47440	Mollusca	7805	Gastropod
Pliocene	5.333	2.588	Petuch	1986	47440	Mollusca	7805	Gastropod
Pliocene	5.333	2.588	Petuch	1986	47440	Mollusca	7805	Gastropod
Pliocene	5.333	2.588	Petuch	1986	47440	Mollusca	7805	Gastropod
Piacenzian	3.6	2.588	Emslie et a	1996	50409	Mollusca	7805	Gastropod
Late Pleistocene	0.126	0.0117	Hulbert an	1998	1962	Mollusca	7805	Gastropod
Late Pleistocene	0.126	0.0117	Hulbert an	1998	1962	Mollusca	7805	Gastropod
Late Pleistocene	0.126	0.0117	DuBar	1971	17556	Mollusca	7805	Gastropod
Late Pleistocene	0.126	0.0117	DuBar	1971	17556	Mollusca	7805	Gastropod
Late Pleistocene	0.126	0.0117	DuBar	1971	17556	Mollusca	7805	Gastropod
Late Pleistocene	0.126	0.0117	DuBar	1971	17556	Mollusca	7805	Gastropod
Late Pleistocene	0.126	0.0117	DuBar	1971	17556	Mollusca	7805	Gastropod
Late Pleistocene	0.126	0.0117	DuBar	1971	17556	Mollusca	7805	Gastropod
Zanclean	5.333	3.6	Campbell a	1995	17558	Mollusca	7805	Gastropod
Zanclean	5.333	3.6	Campbell a	1995	17558	Mollusca	7805	Gastropod
Zanclean	5.333	3.6	Campbell a	1995	17558	Mollusca	7805	Gastropod
Early Pleistocene	2.588	0.781	DuBar	1971	17556	Mollusca	7805	Gastropod
Early Pleistocene	2.588	0.781	DuBar	1971	17556	Mollusca	7805	Gastropod
Late Pleistocene	0.126	0.0117	Richards	1962	18974	Mollusca	7805	Gastropod
Late Pleistocene	0.126	0.0117	Richards	1962	18974	Mollusca	7805	Gastropod
Late Pleistocene	0.126	0.0117	Richards	1962	18974	Mollusca	7805	Gastropod
Late Pleistocene	0.126	0.0117	Richards	1962	18974	Mollusca	7805	Gastropod
Late Pleistocene	0.126	0.0117	Richards	1962	18974	Mollusca	7805	Gastropod

Late Pleistocene	0.126	0.0117	Richards	1962	18974	Mollusca	7805	Gastropod
Late Pleistocene	0.126	0.0117	Hendy et a	2008	19909	Mollusca	7805	Gastropod
Late Pleistocene	0.126	0.0117	Hendy et a	2008	19909	Mollusca	7805	Gastropod
Late Pleistocene	0.126	0.0117	Hendy et a	2008	19909	Mollusca	7805	Gastropod
Late Pleistocene	0.126	0.0117	Hendy et a	2008	19909	Mollusca	7805	Gastropod
Late Pleistocene	0.126	0.0117	Hendy et a	2008	19909	Mollusca	7805	Gastropod
Late Pleistocene	0.126	0.0117	Hendy et a	2008	19909	Mollusca	7805	Gastropod
Late Pleistocene	0.126	0.0117	Hendy et a	2008	19909	Mollusca	7805	Gastropod
Late Pleistocene	0.126	0.0117	Hendy et a	2008	19909	Mollusca	7805	Gastropod
Pleistocene	2.588	0.0117	Hendy et a	2008	19909	Mollusca	7805	Gastropod
Pleistocene	2.588	0.0117	Hendy et a	2008	19909	Mollusca	7805	Gastropod
Pleistocene	2.588	0.0117	Hendy et a	2008	19909	Mollusca	7805	Gastropod
Pleistocene	2.588	0.0117	Hendy et a	2008	19909	Mollusca	7805	Gastropod
Early Pleistocene	2.588	0.781	Cooke	1936	31145	Mollusca	7805	Gastropod
Middle Pleistocene	0.781	0.126	Holmes	1860	64712	Mollusca	7805	Gastropod
Late Pleistocene	0.126	0.0117	Holmes	1860	64712	Mollusca	7805	Gastropod
Late Pliocene	3.6	2.588	Ward and I	1987	16700	Mollusca	7805	Gastropod
Late Pliocene	3.6	2.588	Ward and I	1987	16700	Mollusca	7805	Gastropod
Late Pliocene	3.6	2.588	Ward and I	1987	16700	Mollusca	7805	Gastropod
Late Pliocene	3.6	2.588	Ward and I	1987	16700	Mollusca	7805	Gastropod
Late Pliocene	3.6	2.588	Ward and I	1987	16700	Mollusca	7805	Gastropod
Early Pleistocene	2.588	0.781	Ward and I	1987	16700	Mollusca	7805	Gastropod
Early Pleistocene	2.588	0.781	Ward and I	1987	16700	Mollusca	7805	Gastropod
Early Pleistocene	2.588	0.781	Ward and I	1987	16700	Mollusca	7805	Gastropod
Early Pleistocene	2.588	0.781	Ward and I	1987	16700	Mollusca	7805	Gastropod
Early Pleistocene	2.588	0.781	Ward and I	1987	16700	Mollusca	7805	Gastropod
Pliocene	5.333	2.588	DuBar	1971	17556	Mollusca	7805	Gastropod
Pliocene	5.333	2.588	DuBar	1971	17556	Mollusca	7805	Gastropod
Early Pleistocene	2.588	0.781	Miller	1990	17561	Mollusca	7805	Gastropod
Early Pleistocene	2.588	0.781	Miller	1990	17561	Mollusca	7805	Gastropod
Early Pleistocene	2.588	0.781	Miller	1990	17561	Mollusca	7805	Gastropod
Early Pleistocene	2.588	0.781	Miller	1990	17561	Mollusca	7805	Gastropod
Early Pleistocene	2.588	0.781	Miller	1990	17561	Mollusca	7805	Gastropod
Early Pleistocene	2.588	0.781	Miller	1990	17561	Mollusca	7805	Gastropod
Early Pleistocene	2.588	0.781	Miller	1990	17561	Mollusca	7805	Gastropod
Early Pleistocene	2.588	0.781	Miller	1990	17561	Mollusca	7805	Gastropod
Early Pleistocene	2.588	0.781	Miller	1990	17561	Mollusca	7805	Gastropod
Early Pleistocene	2.588	0.781	Miller	1990	17561	Mollusca	7805	Gastropod
Early Pleistocene	2.588	0.781	Miller	1990	17561	Mollusca	7805	Gastropod
Early Pleistocene	2.588	0.781	Miller	1990	17561	Mollusca	7805	Gastropod
Early Pleistocene	2.588	0.781	Miller	1990	17561	Mollusca	7805	Gastropod
Late Pleistocene	0.126	0.0117	Richards	1962	18974	Mollusca	7805	Gastropod
Late Pleistocene	0.126	0.0117	Richards	1962	18974	Mollusca	7805	Gastropod
Late Pleistocene	0.126	0.0117	Richards	1962	18974	Mollusca	7805	Gastropod
Late Pleistocene	0.126	0.0117	Richards	1962	18974	Mollusca	7805	Gastropod
Late Pleistocene	0.126	0.0117	Richards	1962	18974	Mollusca	7805	Gastropod
Late Pleistocene	0.126	0.0117	Richards	1962	18974	Mollusca	7805	Gastropod

Late Pleistocene	0.126	0.0117	Richards	1962	18974	Mollusca	7805	Gastropod
Late Pleistocene	0.126	0.0117	Richards	1962	18974	Mollusca	7805	Gastropod
Late Pleistocene	0.126	0.0117	Richards	1962	18974	Mollusca	7805	Gastropod
Late Pleistocene	0.126	0.0117	Richards	1962	18974	Mollusca	7805	Gastropod
Late Pleistocene	0.126	0.0117	Richards	1962	18974	Mollusca	7805	Gastropod
Late Pleistocene	0.126	0.0117	Richards	1962	18974	Mollusca	7805	Gastropod
Late Pleistocene	0.126	0.0117	Richards	1962	18974	Mollusca	7805	Gastropod
Late Pleistocene	0.126	0.0117	Hendy et a	2008	19909	Mollusca	7805	Gastropod
Late Pleistocene	0.126	0.0117	Hendy et a	2008	19909	Mollusca	7805	Gastropod
Gelasian	2.588	1.806	Bailey and	1986	23840	Mollusca	7805	Gastropod
Gelasian	2.588	1.806	Bailey and	1986	23840	Mollusca	7805	Gastropod
Gelasian	2.588	1.806	Bailey and	1986	23840	Mollusca	7805	Gastropod
Gelasian	2.588	1.806	Bailey and	1986	23840	Mollusca	7805	Gastropod
Early Pleistocene	2.588	0.781	Hendy et a	2008	19909	Mollusca	7805	Gastropod
Late Pleistocene	0.126	0.0117	Hendy et a	2008	19909	Mollusca	7805	Gastropod
Late Pleistocene	0.126	0.0117	Hendy et a	2008	19909	Mollusca	7805	Gastropod
Late Pliocene	3.6	2.588	Hendy et a	2008	19909	Mollusca	7805	Gastropod
Late Pliocene	3.6	2.588	Hendy et a	2008	19909	Mollusca	7805	Gastropod
Late Pliocene	3.6	2.588	Hendy et a	2008	19909	Mollusca	7805	Gastropod
Late Pliocene	3.6	2.588	Hendy et a	2008	19909	Mollusca	7805	Gastropod
Late Pliocene	3.6	2.588	Hendy et a	2008	19909	Mollusca	7805	Gastropod
Pliocene	5.333	2.588	Hendy et a	2008	19909	Mollusca	7805	Gastropod
Pliocene	5.333	2.588	Hendy et a	2008	19909	Mollusca	7805	Gastropod
Pliocene	5.333	2.588	Hendy et a	2008	19909	Mollusca	7805	Gastropod
Late Pliocene	3.6	2.588	Hendy et a	2008	19909	Mollusca	7805	Gastropod
Late Pliocene	3.6	2.588	Hendy et a	2008	19909	Mollusca	7805	Gastropod
Late Pliocene	3.6	2.588	Hendy et a	2008	19909	Mollusca	7805	Gastropod
Late Pliocene	3.6	2.588	Hendy et a	2008	19909	Mollusca	7805	Gastropod
Late Pliocene	3.6	2.588	Hendy et a	2008	19909	Mollusca	7805	Gastropod
Late Pliocene	3.6	2.588	Hendy et a	2008	19909	Mollusca	7805	Gastropod
Early Pleistocene	2.588	0.781	Hendy et a	2008	19909	Mollusca	7805	Gastropod
Early Pleistocene	2.588	0.781	Hendy et a	2008	19909	Mollusca	7805	Gastropod
Early Pleistocene	2.588	0.781	Hendy et a	2008	19909	Mollusca	7805	Gastropod
Early Pleistocene	2.588	0.781	Hendy et a	2008	19909	Mollusca	7805	Gastropod
Early Pleistocene	2.588	0.781	Hendy et a	2008	19909	Mollusca	7805	Gastropod
Early Pleistocene	2.588	0.781	Hendy et a	2008	19909	Mollusca	7805	Gastropod
Early Pleistocene	2.588	0.781	Hendy et a	2008	19909	Mollusca	7805	Gastropod
Early Pleistocene	2.588	0.781	Hendy et a	2008	19909	Mollusca	7805	Gastropod
Early Pleistocene	2.588	0.781	Hendy et a	2008	19909	Mollusca	7805	Gastropod
Early Pleistocene	2.588	0.781	Hendy et a	2008	19909	Mollusca	7805	Gastropod
Early Pleistocene	2.588	0.781	Hendy et a	2008	19909	Mollusca	7805	Gastropod
Early Pleistocene	2.588	0.781	Hendy et a	2008	19909	Mollusca	7805	Gastropod
Early Pleistocene	2.588	0.781	Hendy et a	2008	19909	Mollusca	7805	Gastropod
Early Pleistocene	2.588	0.781	Hendy et a	2008	19909	Mollusca	7805	Gastropod
Early Pleistocene	2.588	0.781	Miller and	1988	72890	Mollusca	7805	Gastropod
Early Pleistocene	2.588	0.781	Miller and	1988	72890	Mollusca	7805	Gastropod
Early Pleistocene	2.588	0.781	Miller and	1988	72890	Mollusca	7805	Gastropod

Early Pleistocene	2.588	0.781	Miller and	1988	72890	Mollusca	7805	Gastropod
Pleistocene	2.588	0.0117	Coch	1971	441	Mollusca	7805	Gastropod
Late Pleistocene	0.126	0.0117	Spencer ar	1987	15525	Mollusca	7805	Gastropod
Late Pleistocene	0.126	0.0117	Spencer ar	1987	15525	Mollusca	7805	Gastropod
Late Pleistocene	0.126	0.0117	Spencer ar	1987	15525	Mollusca	7805	Gastropod
Late Pleistocene	0.126	0.0117	Spencer ar	1987	15525	Mollusca	7805	Gastropod
Late Pleistocene	0.126	0.0117	Spencer ar	1987	15525	Mollusca	7805	Gastropod
Late Pleistocene	0.126	0.0117	Spencer ar	1987	15525	Mollusca	7805	Gastropod
Late Pleistocene	0.126	0.0117	Spencer ar	1987	15525	Mollusca	7805	Gastropod
Late Pleistocene	0.126	0.0117	Spencer ar	1987	15525	Mollusca	7805	Gastropod
Late Pleistocene	0.126	0.0117	Spencer ar	1987	15525	Mollusca	7805	Gastropod
Late Pleistocene	0.126	0.0117	Spencer ar	1987	15525	Mollusca	7805	Gastropod
Late Pliocene	3.6	2.588	Edwards et	2005	17631	Mollusca	7805	Gastropod
Late Pleistocene	0.126	0.0117	Hendy et a	2008	19909	Mollusca	7805	Gastropod
Late Pleistocene	0.126	0.0117	Hendy et a	2008	19909	Mollusca	7805	Gastropod
Late Pliocene	3.6	2.588	Ward	1993	26351	Mollusca	7805	Gastropod
Late Pliocene	3.6	2.588	Ward	1993	26351	Mollusca	7805	Gastropod
Late Pliocene	3.6	2.588	Ward	1993	26351	Mollusca	7805	Gastropod
Late Pliocene	3.6	2.588	Ward	1993	26351	Mollusca	7805	Gastropod
Late Pliocene	3.6	2.588	Ward	1993	26351	Mollusca	7805	Gastropod
Late Pliocene	3.6	2.588	Ward	1993	26351	Mollusca	7805	Gastropod
Late Pliocene	3.6	2.588	Ward	1993	26351	Mollusca	7805	Gastropod
Late Pliocene	3.6	2.588	Ward	1993	26351	Mollusca	7805	Gastropod
Late Pliocene	3.6	2.588	Ward	1993	26351	Mollusca	7805	Gastropod
Late Pliocene	3.6	2.588	Ward	1993	26351	Mollusca	7805	Gastropod
Late Pliocene	3.6	2.588	Ward	1993	26351	Mollusca	7805	Gastropod
Late Pliocene	3.6	2.588	Ward	1993	26351	Mollusca	7805	Gastropod
Late Pliocene	3.6	2.588	Ward	1993	26351	Mollusca	7805	Gastropod
Late Pliocene	3.6	2.588	Ward	1993	26351	Mollusca	7805	Gastropod
Late Pliocene	3.6	2.588	Ward	1993	26351	Mollusca	7805	Gastropod
Late Pliocene	3.6	2.588	Ward	1993	26351	Mollusca	7805	Gastropod
Late Pliocene	3.6	2.588	Ward	1993	26351	Mollusca	7805	Gastropod
Late Pliocene	3.6	2.588	Ward	1993	26351	Mollusca	7805	Gastropod
Late Pliocene	3.6	2.588	Ward	1993	26351	Mollusca	7805	Gastropod
Late Pliocene	3.6	2.588	Ward	1993	26351	Mollusca	7805	Gastropod
Late Pliocene	3.6	2.588	Ward	1993	26351	Mollusca	7805	Gastropod
Late Pliocene	3.6	2.588	Ward	1993	26351	Mollusca	7805	Gastropod
Late Pliocene	3.6	2.588	Ward	1993	26351	Mollusca	7805	Gastropod
Late Pliocene	3.6	2.588	Ward	1993	26351	Mollusca	7805	Gastropod
Late Pliocene	3.6	2.588	Ward	1993	26351	Mollusca	7805	Gastropod
Late Pliocene	3.6	2.588	Ward	1993	26351	Mollusca	7805	Gastropod
Late Pliocene	3.6	2.588	Ward	1993	26351	Mollusca	7805	Gastropod
Late Pliocene	3.6	2.588	Ward	1993	26351	Mollusca	7805	Gastropod
Late Pliocene	3.6	2.588	Ward	1993	26351	Mollusca	7805	Gastropod
Late Pliocene	3.6	2.588	Ward	1993	26351	Mollusca	7805	Gastropod
Late Pliocene	3.6	2.588	Ward	1993	26351	Mollusca	7805	Gastropod
Late Pliocene	3.6	2.588	Ward	1993	26351	Mollusca	7805	Gastropod

Late Pliocene		3.6	2.588	Ward	1993	26351	Mollusca	7805	Gastropod
Late Pliocene		3.6	2.588	Ward	1993	26351	Mollusca	7805	Gastropod
Late Pliocene		3.6	2.588	Ward	1993	26351	Mollusca	7805	Gastropod
Late Pliocene		3.6	2.588	Ward	1993	26351	Mollusca	7805	Gastropod
Late Pliocene		3.6	2.588	Hendy et a	2008	19909	Mollusca	7805	Gastropod
Late Pliocene		3.6	2.588	Hendy et a	2008	19909	Mollusca	7805	Gastropod
Late Pliocene		3.6	2.588	Hendy et a	2008	19909	Mollusca	7805	Gastropod
Late Pliocene		3.6	2.588	Hendy et a	2008	19909	Mollusca	7805	Gastropod
Late Pliocene		3.6	2.588	Hendy et a	2008	19909	Mollusca	7805	Gastropod
Late Pliocene		3.6	2.588	Hendy et a	2008	19909	Mollusca	7805	Gastropod
Late Pliocene		3.6	2.588	Hendy et a	2008	19909	Mollusca	7805	Gastropod
Late Pliocene		3.6	2.588	Hendy et a	2008	19909	Mollusca	7805	Gastropod
Late Pliocene		3.6	2.588	Hendy et a	2008	19909	Mollusca	7805	Gastropod
Late Pliocene		3.6	2.588	Hendy et a	2008	19909	Mollusca	7805	Gastropod
Late Pliocene		3.6	2.588	Hendy et a	2008	19909	Mollusca	7805	Gastropod
Late Pliocene		3.6	2.588	Hendy et a	2008	19909	Mollusca	7805	Gastropod
Late Pliocene		3.6	2.588	Hendy et a	2008	19909	Mollusca	7805	Gastropod
Early Pleistocene		2.588	0.781	Hendy et a	2008	19909	Mollusca	7805	Gastropod
Early Pleistocene		2.588	0.781	Hendy et a	2008	19909	Mollusca	7805	Gastropod
Early Pleistocene		2.588	0.781	Olsson anc	1953	25463	Mollusca	7805	Gastropod
Early Pleistocene		2.588	0.781	Olsson anc	1953	25463	Mollusca	7805	Gastropod
Early Pleistocene		2.588	0.781	Olsson anc	1953	25463	Mollusca	7805	Gastropod
Early Pleistocene		2.588	0.781	Olsson anc	1953	25463	Mollusca	7805	Gastropod
Early Pleistocene		2.588	0.781	Olsson anc	1953	25463	Mollusca	7805	Gastropod
Early Pleistocene		2.588	0.781	Olsson anc	1953	25463	Mollusca	7805	Gastropod
Early Pleistocene		2.588	0.781	Olsson anc	1953	25463	Mollusca	7805	Gastropod
Early Pleistocene		2.588	0.781	Olsson anc	1953	25463	Mollusca	7805	Gastropod
Early Pleistocene		2.588	0.781	Olsson anc	1953	25463	Mollusca	7805	Gastropod
Early Pleistocene		2.588	0.781	Olsson anc	1953	25463	Mollusca	7805	Gastropod
Pliocene		5.333	2.588	Petuch	1986	47440	Mollusca	7805	Gastropod
Early Pleistocene		2.588	0.781	DuBar	1962	11119	Mollusca	7805	Gastropod
Early Pleistocene		2.588	0.781	DuBar	1962	11119	Mollusca	7805	Gastropod
Calabrian		1.806	0.781	DuBar	1962	11119	Mollusca	7805	Gastropod
Early Pleistocene		2.588	0.781	DuBar	1962	11119	Mollusca	7805	Gastropod
Pliocene	Pleistocene	5.333	0.0117	DuBar	1962	11119	Mollusca	7805	Gastropod
Pliocene	Pleistocene	5.333	0.0117	DuBar	1962	11119	Mollusca	7805	Gastropod
Pliocene	Pleistocene	5.333	0.0117	DuBar	1962	11119	Mollusca	7805	Gastropod
Pliocene	Pleistocene	5.333	0.0117	DuBar	1962	11119	Mollusca	7805	Gastropod
Late Pliocene		3.6	2.588	Mansfield	1930	17060	Mollusca	7805	Gastropod
Late Pliocene		3.6	2.588	Mansfield	1930	17060	Mollusca	7805	Gastropod
Late Pliocene		3.6	2.588	Mansfield	1930	17060	Mollusca	7805	Gastropod
Late Pliocene		3.6	2.588	Mansfield	1930	17060	Mollusca	7805	Gastropod
Late Pliocene		3.6	2.588	Mansfield	1930	17060	Mollusca	7805	Gastropod
Late Pliocene		3.6	2.588	Mansfield	1930	17060	Mollusca	7805	Gastropod
Late Pliocene		3.6	2.588	Mansfield	1932	17094	Mollusca	7805	Gastropod
Late Pliocene		3.6	2.588	Mansfield	1932	17094	Mollusca	7805	Gastropod
Middle Pleistocene		0.781	0.126	McGinty	1970	19114	Mollusca	7805	Gastropod

Late Pliocene	3.6	2.588	Mansfield	1939	19112	Mollusca	7805	Gastropod
Late Pliocene	3.6	2.588	Mansfield	1939	19112	Mollusca	7805	Gastropod
Late Pliocene	3.6	2.588	Mansfield	1939	19112	Mollusca	7805	Gastropod
Early Pleistocene	2.588	0.781	Schroeder	1954	25303	Mollusca	7805	Gastropod
Pliocene	5.333	2.588	Schroeder	1954	25303	Mollusca	7805	Gastropod
Early Pleistocene	2.588	0.781	Olsson and	1953	25463	Mollusca	7805	Gastropod
Early Pleistocene	2.588	0.781	Olsson and	1953	25463	Mollusca	7805	Gastropod
Early Pleistocene	2.588	0.781	Olsson and	1953	25463	Mollusca	7805	Gastropod
Early Pleistocene	2.588	0.781	Olsson and	1953	25463	Mollusca	7805	Gastropod
Early Pleistocene	2.588	0.781	Olsson and	1953	25463	Mollusca	7805	Gastropod
Early Pleistocene	2.588	0.781	Olsson and	1953	25463	Mollusca	7805	Gastropod
Early Pleistocene	2.588	0.781	Olsson and	1953	25463	Mollusca	7805	Gastropod
Early Pleistocene	2.588	0.781	Olsson and	1953	25463	Mollusca	7805	Gastropod
Early Pleistocene	2.588	0.781	Petuch and	2007	26813	Mollusca	7805	Gastropod
Late Pliocene	3.6	2.588	Olsson	1967	19826	Mollusca	7805	Gastropod
Late Pliocene	3.6	2.588	Olsson	1967	19826	Mollusca	7805	Gastropod
Late Pliocene	3.6	2.588	Olsson	1967	19826	Mollusca	7805	Gastropod
Late Pliocene	3.6	2.588	Olsson	1967	19826	Mollusca	7805	Gastropod
Late Pliocene	3.6	2.588	Olsson	1967	19826	Mollusca	7805	Gastropod
Early Pleistocene	2.588	0.781	Olsson	1967	19826	Mollusca	7805	Gastropod
Late Pliocene	3.6	2.588	Olsson and	1964	26836	Mollusca	7805	Gastropod
Late Pliocene	3.6	2.588	Olsson and	1964	26836	Mollusca	7805	Gastropod
Late Pliocene	3.6	2.588	Olsson and	1964	26836	Mollusca	7805	Gastropod
Early Pleistocene	2.588	0.781	Petuch and	2007	26813	Mollusca	7805	Gastropod
Early Pleistocene	2.588	0.781	Petuch and	2007	26813	Mollusca	7805	Gastropod
Late Plioce Early Pleist	3.6	0.781	Petuch and	2007	26813	Mollusca	7805	Gastropod
Late Plioce Early Pleist	3.6	0.781	Petuch and	2007	26813	Mollusca	7805	Gastropod
Late Plioce Pleistocene	3.6	0.0117	Petuch and	2007	26813	Mollusca	7805	Gastropod
Late Pliocene	3.6	2.588	Petuch and	2007	26813	Mollusca	7805	Gastropod
Late Pliocene	3.6	2.588	Petuch and	2007	26813	Mollusca	7805	Gastropod
Late Pliocene	3.6	2.588	Petuch and	2007	26813	Mollusca	7805	Gastropod
Late Pleistocene	0.126	0.0117	Hendy et a	2008	19909	Mollusca	7805	Gastropod
Late Pliocene	3.6	2.588	Petuch and	2007	26813	Mollusca	7805	Gastropod
Late Pliocene	3.6	2.588	Petuch and	2007	26813	Mollusca	7805	Gastropod
Early Pleistocene	2.588	0.781	Olsson and	1953	25463	Mollusca	7805	Gastropod
Late Pliocene	3.6	2.588	Petuch and	2007	26813	Mollusca	7805	Gastropod
Late Pliocene	3.6	2.588	Petuch and	2007	26813	Mollusca	7805	Gastropod
Late Pliocene	3.6	2.588	Petuch and	2007	26813	Mollusca	7805	Gastropod
Late Pliocene	3.6	2.588	Petuch and	2007	26813	Mollusca	7805	Gastropod
Late Pliocene	3.6	2.588	Petuch and	2007	26813	Mollusca	7805	Gastropod
Early Pleistocene	2.588	0.781	Hendy et a	2008	19909	Mollusca	7805	Gastropod
Early Pleistocene	2.588	0.781	Hendy et a	2008	19909	Mollusca	7805	Gastropod
Early Pleistocene	2.588	0.781	Hendy et a	2008	19909	Mollusca	7805	Gastropod
Early Pleistocene	2.588	0.781	Hendy et a	2008	19909	Mollusca	7805	Gastropod
Early Pleistocene	2.588	0.781	Hendy et a	2008	19909	Mollusca	7805	Gastropod
Late Pliocene	3.6	2.588	Hendy et a	2008	19909	Mollusca	7805	Gastropod
Late Pliocene	3.6	2.588	Weisbord	1974	28823	Mollusca	7805	Gastropod

Late Pliocene	3.6	2.588	Weisbord	1972	31274	Mollusca	7805	Gastropod
Late Pliocene	3.6	2.588	Petuch	1988	31112	Mollusca	7805	Gastropod
Late Pliocene	3.6	2.588	Petuch	1988	31112	Mollusca	7805	Gastropod
Late Pleistocene	0.126	0.0117	Kittle and F	2010	40884	Mollusca	7805	Gastropod
Pliocene	5.333	2.588	Petuch	1986	47440	Mollusca	7805	Gastropod
Pliocene	5.333	2.588	Petuch	1986	47440	Mollusca	7805	Gastropod
Pliocene	5.333	2.588	Petuch	1986	47440	Mollusca	7805	Gastropod
Pliocene	5.333	2.588	Petuch	1986	47440	Mollusca	7805	Gastropod
Pliocene	5.333	2.588	Petuch	1986	47440	Mollusca	7805	Gastropod
Pliocene	5.333	2.588	Petuch	1986	47440	Mollusca	7805	Gastropod
Pliocene	5.333	2.588	Petuch	1986	47440	Mollusca	7805	Gastropod
Late Pliocene	3.6	2.588	Olsson anc	1964	26836	Mollusca	7805	Gastropod
Late Pleistocene	0.126	0.0117	DuBar	1971	17556	Mollusca	7805	Gastropod
Late Pleistocene	0.126	0.0117	Richards	1962	18974	Mollusca	7805	Gastropod
Pleistocene	2.588	0.0117	Holmes	1860	64712	Mollusca	7805	Gastropod
Late Pliocene	3.6	2.588	Gardner	1948	17048	Mollusca	7805	Gastropod
Pliocene	5.333	2.588	Hendy et a	2008	19909	Mollusca	7805	Gastropod
Early Pleistocene	2.588	0.781	Hendy et a	2008	19909	Mollusca	7805	Gastropod
Late Pliocene	3.6	2.588	Ward	1993	26351	Mollusca	7805	Gastropod
Late Pliocene	3.6	2.588	Hendy et a	2008	19909	Mollusca	7805	Gastropod
Late Pliocene	3.6	2.588	Hendy et a	2008	19909	Mollusca	7805	Gastropod
Late Pliocene	3.6	2.588	Hendy et a	2008	19909	Mollusca	7805	Gastropod
Early Pleistocene	2.588	0.781	Hendy et a	2008	19909	Mollusca	7805	Gastropod
Late Pliocene	3.6	2.588	Mansfield	1930	17060	Mollusca	7805	Gastropod
Middle Pleistocene	0.781	0.126	McGinty	1970	19114	Mollusca	7805	Gastropod
Late Pleistocene	0.126	0.0117	Beu	2010	30037	Mollusca	7805	Gastropod
Middle Pleistocene	0.781	0.126	Petuch anc	2007	26813	Mollusca	7805	Gastropod
Middle Pleistocene	0.781	0.126	Petuch anc	2007	26813	Mollusca	7805	Gastropod
Middle Pleistocene	0.781	0.126	Petuch anc	2007	26813	Mollusca	7805	Gastropod
Late Pliocene	3.6	2.588	Petuch anc	2007	26813	Mollusca	7805	Gastropod
Late Pliocene	3.6	2.588	Petuch anc	2007	26813	Mollusca	7805	Gastropod
Late Pliocene	3.6	2.588	Petuch anc	2007	26813	Mollusca	7805	Gastropod
Late Pliocene	3.6	2.588	Petuch anc	2007	26813	Mollusca	7805	Gastropod
Late Pliocene	3.6	2.588	Beu	2010	30037	Mollusca	7805	Gastropod
Late Pliocene	3.6	2.588	Petuch	1988	31112	Mollusca	7805	Gastropod
Middle Pleistocene	0.781	0.126	Beu	2010	30037	Mollusca	7805	Gastropod
Early Pleistocene	2.588	0.781	Beu	2010	30037	Mollusca	7805	Gastropod
Middle Pleistocene	0.781	0.126	Beu	2010	30037	Mollusca	7805	Gastropod
Late Pleistocene	0.126	0.0117	Kittle and F	2010	40884	Mollusca	7805	Gastropod
Late Pleistocene	0.126	0.0117	Kittle and F	2010	40884	Mollusca	7805	Gastropod
Zanclean	5.333	3.6	Campbell a	1995	17558	Mollusca	7805	Gastropod
Late Pliocene	3.6	2.588	Gardner	1948	17048	Mollusca	7805	Gastropod
Late Pliocene	3.6	2.588	Gardner	1948	17048	Mollusca	7805	Gastropod
Late Pleistocene	0.126	0.0117	Sellards	1917	9011	Mollusca	7805	Gastropod
Late Pleistocene	0.126	0.0117	Sellards	1917	9011	Mollusca	7805	Gastropod
Late Pleistocene	0.126	0.0117	Sellards	1917	9011	Mollusca	7805	Gastropod
Early Pleistocene	2.588	0.781	DuBar	1962	11119	Mollusca	7805	Gastropod

Early Pleistocene		2.588	0.781	DuBar	1962	11119	Mollusca	7805	Gastropod
Early Pleistocene		2.588	0.781	DuBar	1962	11119	Mollusca	7805	Gastropod
Early Pleistocene		2.588	0.781	DuBar	1962	11119	Mollusca	7805	Gastropod
Early Pleistocene		2.588	0.781	DuBar	1962	11119	Mollusca	7805	Gastropod
Early Pleistocene		2.588	0.781	DuBar	1962	11119	Mollusca	7805	Gastropod
Early Pleistocene		2.588	0.781	DuBar	1962	11119	Mollusca	7805	Gastropod
Early Pleistocene		2.588	0.781	DuBar	1962	11119	Mollusca	7805	Gastropod
Early Pleistocene		2.588	0.781	DuBar	1962	11119	Mollusca	7805	Gastropod
Calabrian		1.806	0.781	DuBar	1962	11119	Mollusca	7805	Gastropod
Calabrian		1.806	0.781	DuBar	1962	11119	Mollusca	7805	Gastropod
Calabrian		1.806	0.781	DuBar	1962	11119	Mollusca	7805	Gastropod
Calabrian		1.806	0.781	DuBar	1962	11119	Mollusca	7805	Gastropod
Calabrian		1.806	0.781	DuBar	1962	11119	Mollusca	7805	Gastropod
Calabrian		1.806	0.781	DuBar	1962	11119	Mollusca	7805	Gastropod
Early Pleistocene		2.588	0.781	DuBar	1962	11119	Mollusca	7805	Gastropod
Early Pleistocene		2.588	0.781	DuBar	1962	11119	Mollusca	7805	Gastropod
Early Pleistocene		2.588	0.781	DuBar	1962	11119	Mollusca	7805	Gastropod
Early Pleistocene		2.588	0.781	DuBar	1962	11119	Mollusca	7805	Gastropod
Late Pleistocene		0.126	0.0117	DuBar	1962	11119	Mollusca	7805	Gastropod
Pliocene	Pleistocen	5.333	0.0117	DuBar	1962	11119	Mollusca	7805	Gastropod
Pliocene	Pleistocen	5.333	0.0117	DuBar	1962	11119	Mollusca	7805	Gastropod
Pliocene	Pleistocen	5.333	0.0117	DuBar	1962	11119	Mollusca	7805	Gastropod
Pliocene	Pleistocen	5.333	0.0117	DuBar	1962	11119	Mollusca	7805	Gastropod
Pliocene	Pleistocen	5.333	0.0117	DuBar	1962	11119	Mollusca	7805	Gastropod
Calabrian		1.806	0.781	DuBar	1962	11119	Mollusca	7805	Gastropod
Late Pliocene		3.6	2.588	Mansfield	1930	17060	Mollusca	7805	Gastropod
Late Pliocene		3.6	2.588	Mansfield	1930	17060	Mollusca	7805	Gastropod
Late Pleistocene		0.126	0.0117	Portell et a	1992	17634	Mollusca	7805	Gastropod
Late Pleistocene		0.126	0.0117	Portell et a	1992	17634	Mollusca	7805	Gastropod
Middle Pleistocene		0.781	0.126	Portell et a	1992	17634	Mollusca	7805	Gastropod
Middle Pleistocene		0.781	0.126	Portell et a	1992	17634	Mollusca	7805	Gastropod
Late Pleistocene		0.126	0.0117	Hendy et a	2008	19909	Mollusca	7805	Gastropod
Late Pleistocene		0.126	0.0117	Hendy et a	2008	19909	Mollusca	7805	Gastropod
Late Pleistocene		0.126	0.0117	Hendy et a	2008	19909	Mollusca	7805	Gastropod
Late Pleistocene		0.126	0.0117	Hendy et a	2008	19909	Mollusca	7805	Gastropod
Late Pleistocene		0.126	0.0117	Hendy et a	2008	19909	Mollusca	7805	Gastropod
Early Pleistocene		2.588	0.781	Schroeder	1954	25303	Mollusca	7805	Gastropod
Early Pleistocene		2.588	0.781	Schroeder	1954	25303	Mollusca	7805	Gastropod
Early Pleistocene		2.588	0.781	Schroeder	1954	25303	Mollusca	7805	Gastropod
Pliocene		5.333	2.588	Schroeder	1954	25303	Mollusca	7805	Gastropod
Pliocene		5.333	2.588	Schroeder	1954	25303	Mollusca	7805	Gastropod
Early Pleistocene		2.588	0.781	Schroeder	1954	25303	Mollusca	7805	Gastropod
Early Pleistocene		2.588	0.781	Schroeder	1954	25303	Mollusca	7805	Gastropod
Late Pleistocene		0.126	0.0117	Petuch an	2007	26813	Mollusca	7805	Gastropod
Late Pleistocene		0.126	0.0117	Petuch an	2007	26813	Mollusca	7805	Gastropod
Late Pleistocene		0.126	0.0117	Petuch an	2007	26813	Mollusca	7805	Gastropod
Late Plioce	Early Pleist	3.6	0.781	Petuch an	2007	26813	Mollusca	7805	Gastropod

Late Plioce Pleistocene	3.6	0.0117	Petuch and	2007	26813	Mollusca	7805	Gastropod
Early Pleistocene	2.588	0.781	Hendy et a	2008	19909	Mollusca	7805	Gastropod
Early Pleistocene	2.588	0.781	Hendy et a	2008	19909	Mollusca	7805	Gastropod
Late Pleistocene	0.126	0.0117	Hendy et a	2008	19909	Mollusca	7805	Gastropod
Late Pliocene	3.6	2.588	Petuch and	2007	26813	Mollusca	7805	Gastropod
Late Pliocene	3.6	2.588	Petuch and	2007	26813	Mollusca	7805	Gastropod
Late Pliocene	3.6	2.588	Petuch and	2007	26813	Mollusca	7805	Gastropod
Late Pliocene	3.6	2.588	Petuch and	2007	26813	Mollusca	7805	Gastropod
Early Pleistocene	2.588	0.781	DuBar	1958	25514	Mollusca	7805	Gastropod
Late Pleistocene	0.126	0.0117	DuBar	1958	25514	Mollusca	7805	Gastropod
Late Pleistocene	0.126	0.0117	Hendy et a	2008	19909	Mollusca	7805	Gastropod
Late Pliocene	3.6	2.588	Hendy et a	2008	19909	Mollusca	7805	Gastropod
Late Pliocene	3.6	2.588	Hendy et a	2008	19909	Mollusca	7805	Gastropod
Early Pleistocene	2.588	0.781	Hendy et a	2008	19909	Mollusca	7805	Gastropod
Early Pleistocene	2.588	0.781	Hendy et a	2008	19909	Mollusca	7805	Gastropod
Pleistocene	2.588	0.0117	Hendy et a	2008	19909	Mollusca	7805	Gastropod
Middle Pleistocene	0.781	0.126	Hendy et a	2008	19909	Mollusca	7805	Gastropod
Middle Pleistocene	0.781	0.126	Hendy et a	2008	19909	Mollusca	7805	Gastropod
Middle Pleistocene	0.781	0.126	Hendy et a	2008	19909	Mollusca	7805	Gastropod
Late Pleistocene	0.126	0.0117	Hendy et a	2008	19909	Mollusca	7805	Gastropod
Early Pleistocene	2.588	0.781	Olsson and	1953	25463	Mollusca	7805	Gastropod
Early Pleistocene	2.588	0.781	Olsson and	1953	25463	Mollusca	7805	Gastropod
Early Pleistocene	2.588	0.781	Olsson and	1953	25463	Mollusca	7805	Gastropod
Early Pleistocene	2.588	0.781	Olsson and	1953	25463	Mollusca	7805	Gastropod
Early Pleistocene	2.588	0.781	Olsson and	1953	25463	Mollusca	7805	Gastropod
Early Pleistocene	2.588	0.781	Olsson and	1953	25463	Mollusca	7805	Gastropod
Early Pleistocene	2.588	0.781	Olsson and	1953	25463	Mollusca	7805	Gastropod
Early Pleistocene	2.588	0.781	Olsson and	1953	25463	Mollusca	7805	Gastropod
Early Pleistocene	2.588	0.781	Olsson and	1953	25463	Mollusca	7805	Gastropod
Early Pleistocene	2.588	0.781	Olsson and	1953	25463	Mollusca	7805	Gastropod
Early Pleistocene	2.588	0.781	Olsson and	1953	25463	Mollusca	7805	Gastropod
Early Pleistocene	2.588	0.781	Olsson and	1953	25463	Mollusca	7805	Gastropod
Late Pleistocene	0.126	0.0117	Kittle and I	2010	40884	Mollusca	7805	Gastropod
Late Pleistocene	0.126	0.0117	Kittle and I	2010	40884	Mollusca	7805	Gastropod
Late Pleistocene	0.126	0.0117	Kittle and I	2010	40884	Mollusca	7805	Gastropod
Late Pleistocene	0.126	0.0117	Kittle and I	2010	40884	Mollusca	7805	Gastropod
Late Pleistocene	0.126	0.0117	Kittle and I	2010	40884	Mollusca	7805	Gastropod
Late Pleistocene	0.126	0.0117	Kittle and I	2010	40884	Mollusca	7805	Gastropod
Pliocene	5.333	2.588	Petuch	1986	47440	Mollusca	7805	Gastropod
Pliocene	5.333	2.588	Petuch	1986	47440	Mollusca	7805	Gastropod
Pliocene	5.333	2.588	Petuch	1986	47440	Mollusca	7805	Gastropod
Early Pleistocene	2.588	0.781	Ward and I	1987	16700	Mollusca	7805	Gastropod
Early Pleistocene	2.588	0.781	DuBar	1971	17556	Mollusca	7805	Gastropod
Late Pleistocene	0.126	0.0117	Hendy et a	2008	19909	Mollusca	7805	Gastropod
Late Pleistocene	0.126	0.0117	Spencer ar	1987	15525	Mollusca	7805	Gastropod
Early Pleistocene	2.588	0.781	Hendy et a	2008	19909	Mollusca	7805	Gastropod
Late Pleistocene	0.126	0.0117	DuBar	1962	11119	Mollusca	7805	Gastropod

Early Pleistocene	2.588	0.781	DuBar	1962	11119	Mollusca	7805	Gastropod
Late Pliocene	3.6	2.588	Mansfield	1930	17060	Mollusca	7805	Gastropod
Late Pliocene	3.6	2.588	Mansfield	1930	17060	Mollusca	7805	Gastropod
Late Pliocene	3.6	2.588	Mansfield	1930	17060	Mollusca	7805	Gastropod
Late Pliocene	3.6	2.588	Mansfield	1930	17060	Mollusca	7805	Gastropod
Late Pliocene	3.6	2.588	Mansfield	1930	17060	Mollusca	7805	Gastropod
Late Pleistocene	0.126	0.0117	Portell et a	1992	17634	Mollusca	7805	Gastropod
Early Pleistocene	2.588	0.781	Portell et a	1992	17634	Mollusca	7805	Gastropod
Middle Pleistocene	0.781	0.126	Hendy et a	2008	19909	Mollusca	7805	Gastropod
Middle Pleistocene	0.781	0.126	Portell et a	1992	17634	Mollusca	7805	Gastropod
Early Pleistocene	2.588	0.781	Olsson anc	1953	25463	Mollusca	7805	Gastropod
Early Pleistocene	2.588	0.781	Olsson anc	1953	25463	Mollusca	7805	Gastropod
Early Pleistocene	2.588	0.781	Olsson anc	1953	25463	Mollusca	7805	Gastropod
Early Pleistocene	2.588	0.781	Olsson anc	1953	25463	Mollusca	7805	Gastropod
Early Pleistocene	2.588	0.781	Olsson anc	1953	25463	Mollusca	7805	Gastropod
Early Pleistocene	2.588	0.781	Olsson anc	1953	25463	Mollusca	7805	Gastropod
Early Pleistocene	2.588	0.781	Olsson anc	1953	25463	Mollusca	7805	Gastropod
Early Pleistocene	2.588	0.781	Olsson anc	1953	25463	Mollusca	7805	Gastropod
Early Pleistocene	2.588	0.781	Hendy et a	2008	19909	Mollusca	7805	Gastropod
Early Pleistocene	2.588	0.781	Olsson anc	1953	25463	Mollusca	7805	Gastropod
Late Pleistocene	0.126	0.0117	Kittle and I	2010	40884	Mollusca	7805	Gastropod
Late Pleistocene	0.126	0.0117	Kittle and I	2010	40884	Mollusca	7805	Gastropod
Late Pleistocene	0.126	0.0117	DuBar	1971	17556	Mollusca	7805	Gastropod
Late Pleistocene	0.126	0.0117	DuBar	1971	17556	Mollusca	7805	Gastropod
Late Pleistocene	0.126	0.0117	DuBar	1971	17556	Mollusca	7805	Gastropod
Late Pleistocene	0.126	0.0117	Hendy et a	2008	19909	Mollusca	7805	Gastropod
Late Pleistocene	0.126	0.0117	Richards	1962	18974	Mollusca	7805	Gastropod
Early Pleistocene	2.588	0.781	Cooke	1936	31145	Mollusca	7805	Gastropod
Late Pliocene	3.6	2.588	Ward and I	1987	16700	Mollusca	7805	Gastropod
Late Pliocene	3.6	2.588	Ward and I	1987	16700	Mollusca	7805	Gastropod
Early Pleistocene	2.588	0.781	Ward and I	1987	16700	Mollusca	7805	Gastropod
Early Pleistocene	2.588	0.781	Ward and I	1987	16700	Mollusca	7805	Gastropod
Early Pleistocene	2.588	0.781	Hendy et a	2008	19909	Mollusca	7805	Gastropod
Late Pliocene	3.6	2.588	Hendy et a	2008	19909	Mollusca	7805	Gastropod
Early Pleistocene	2.588	0.781	Miller	1990	17561	Mollusca	7805	Gastropod
Early Pleistocene	2.588	0.781	Miller	1990	17561	Mollusca	7805	Gastropod
Early Pleistocene	2.588	0.781	Miller	1990	17561	Mollusca	7805	Gastropod
Early Pleistocene	2.588	0.781	Hendy et a	2008	19909	Mollusca	7805	Gastropod
Early Pleistocene	2.588	0.781	Miller	1990	17561	Mollusca	7805	Gastropod
Early Pleistocene	2.588	0.781	Miller	1990	17561	Mollusca	7805	Gastropod
Early Pleistocene	2.588	0.781	Rosenberg	2005	19419	Mollusca	7805	Gastropod
Early Pleistocene	2.588	0.781	Miller	1990	17561	Mollusca	7805	Gastropod
Late Pleistocene	0.126	0.0117	Hendy et a	2008	19909	Mollusca	7805	Gastropod
Gelasian	2.588	1.806	Bailey and	1986	23840	Mollusca	7805	Gastropod
Late Pleistocene	0.126	0.0117	Spencer ar	1987	15525	Mollusca	7805	Gastropod
Late Pleistocene	0.126	0.0117	Spencer ar	1987	15525	Mollusca	7805	Gastropod
Late Pleistocene	0.126	0.0117	Spencer ar	1987	15525	Mollusca	7805	Gastropod

Late Pliocene		3.6	2.588	Ward	1993	26351	Mollusca	7805 Gastropod
Late Pliocene		3.6	2.588	Ward	1993	26351	Mollusca	7805 Gastropod
Late Pliocene		3.6	2.588	Ward	1993	26351	Mollusca	7805 Gastropod
Late Pliocene		3.6	2.588	Ward	1993	26351	Mollusca	7805 Gastropod
Late Pliocene		3.6	2.588	Ward	1993	26351	Mollusca	7805 Gastropod
Late Pliocene		3.6	2.588	Ward	1993	26351	Mollusca	7805 Gastropod
Early Pleistocene		2.588	0.781	DuBar	1962	11119	Mollusca	7805 Gastropod
Early Pleistocene		2.588	0.781	DuBar	1962	11119	Mollusca	7805 Gastropod
Early Pleistocene		2.588	0.781	DuBar	1962	11119	Mollusca	7805 Gastropod
Late Pliocene		3.6	2.588	Mansfield	1930	17060	Mollusca	7805 Gastropod
Late Pliocene		3.6	2.588	Mansfield	1930	17060	Mollusca	7805 Gastropod
Early Pleistocene		2.588	0.781	Fargo	1953	27010	Mollusca	7805 Gastropod
Early Pleistocene		2.588	0.781	Fargo	1953	27010	Mollusca	7805 Gastropod
Early Pleistocene		2.588	0.781	Fargo	1953	27010	Mollusca	7805 Gastropod
Early Pleistocene		2.588	0.781	Fargo	1953	27010	Mollusca	7805 Gastropod
Early Pleistocene		2.588	0.781	Fargo	1953	27010	Mollusca	7805 Gastropod
Early Pleistocene		2.588	0.781	Fargo	1953	27010	Mollusca	7805 Gastropod
Early Pleistocene		2.588	0.781	Fargo	1953	27010	Mollusca	7805 Gastropod
Early Pleistocene		2.588	0.781	Fargo	1953	27010	Mollusca	7805 Gastropod
Pliocene		5.333	2.588	Petuch	1986	47440	Mollusca	7805 Gastropod
Late Pliocene		3.6	2.588	Gardner	1948	17048	Mollusca	7805 Gastropod
Late Pliocene		3.6	2.588	Gardner	1948	17048	Mollusca	7805 Gastropod
Early Pleistocene		2.588	0.781	Gardner	1948	17048	Mollusca	7805 Gastropod
Early Pleistocene		2.588	0.781	Gardner	1948	17048	Mollusca	7805 Gastropod
Pleistocene		2.588	0.0117	Gardner	1948	17048	Mollusca	7805 Gastropod
Late Pliocene		3.6	2.588	Gardner	1948	17048	Mollusca	7805 Gastropod
Early Pleistocene		2.588	0.781	DuBar	1962	11119	Mollusca	7805 Gastropod
Calabrian		1.806	0.781	DuBar	1962	11119	Mollusca	7805 Gastropod
Early Pleistocene		2.588	0.781	DuBar	1962	11119	Mollusca	7805 Gastropod
Early Pleistocene		2.588	0.781	Portell et a	1992	17634	Mollusca	7805 Gastropod
Early Pleistocene		2.588	0.781	Portell et a	1992	17634	Mollusca	7805 Gastropod
Late Pleistocene		0.126	0.0117	Portell et a	1992	17634	Mollusca	7805 Gastropod
Late Pleistocene		0.126	0.0117	Kittle and I	2010	40884	Mollusca	7805 Gastropod
Late Pleistocene		0.126	0.0117	Sellards	1917	9011	Mollusca	7805 Gastropod
Early Pleistocene		2.588	0.781	DuBar	1962	11119	Mollusca	7805 Gastropod
Early Pleistocene		2.588	0.781	DuBar	1962	11119	Mollusca	7805 Gastropod
Calabrian		1.806	0.781	DuBar	1962	11119	Mollusca	7805 Gastropod
Calabrian		1.806	0.781	DuBar	1962	11119	Mollusca	7805 Gastropod
Calabrian		1.806	0.781	DuBar	1962	11119	Mollusca	7805 Gastropod
Late Pleistocene		0.126	0.0117	DuBar	1962	11119	Mollusca	7805 Gastropod
Early Pleistocene		2.588	0.781	DuBar	1962	11119	Mollusca	7805 Gastropod
Late Pleistocene		0.126	0.0117	DuBar	1962	11119	Mollusca	7805 Gastropod
Late Pleistocene		0.126	0.0117	DuBar	1962	11119	Mollusca	7805 Gastropod
Pliocene	Pleistocen	5.333	0.0117	DuBar	1962	11119	Mollusca	7805 Gastropod
Pliocene	Pleistocen	5.333	0.0117	DuBar	1962	11119	Mollusca	7805 Gastropod
Late Pliocene		3.6	2.588	Mansfield	1930	17060	Mollusca	7805 Gastropod
Late Pliocene		3.6	2.588	Mansfield	1930	17060	Mollusca	7805 Gastropod

Late Pliocene	3.6	2.588	Mansfield	1930	17060	Mollusca	7805	Gastropod
Late Pliocene	3.6	2.588	Mansfield	1932	17094	Mollusca	7805	Gastropod
Late Pliocene	3.6	2.588	Mansfield	1932	17094	Mollusca	7805	Gastropod
Early Pleistocene	2.588	0.781	Portell et a	1992	17634	Mollusca	7805	Gastropod
Early Pleistocene	2.588	0.781	Portell et a	1992	17634	Mollusca	7805	Gastropod
Early Pleistocene	2.588	0.781	Portell et a	1992	17634	Mollusca	7805	Gastropod
Middle Pleistocene	0.781	0.126	Portell et a	1992	17634	Mollusca	7805	Gastropod
Middle Pleistocene	0.781	0.126	Portell et a	1992	17634	Mollusca	7805	Gastropod
Late Pleistocene	0.126	0.0117	Portell et a	1992	17634	Mollusca	7805	Gastropod
Late Pleistocene	0.126	0.0117	Portell et a	1992	17634	Mollusca	7805	Gastropod
Late Pleistocene	0.126	0.0117	Hendy et a	2008	19909	Mollusca	7805	Gastropod
Late Pleistocene	0.126	0.0117	Hendy et a	2008	19909	Mollusca	7805	Gastropod
Early Pleistocene	2.588	0.781	Schroeder	1954	25303	Mollusca	7805	Gastropod
Pliocene	5.333	2.588	Schroeder	1954	25303	Mollusca	7805	Gastropod
Early Pleistocene	2.588	0.781	Olsson anc	1953	25463	Mollusca	7805	Gastropod
Early Pleistocene	2.588	0.781	Olsson anc	1953	25463	Mollusca	7805	Gastropod
Early Pleistocene	2.588	0.781	Olsson anc	1953	25463	Mollusca	7805	Gastropod
Late Pleistocene	0.126	0.0117	Petuch anc	2007	26813	Mollusca	7805	Gastropod
Middle Pleistocene	0.781	0.126	Petuch anc	2007	26813	Mollusca	7805	Gastropod
Early Pleistocene	2.588	0.781	Olsson anc	1953	25463	Mollusca	7805	Gastropod
Early Pleistocene	2.588	0.781	Olsson anc	1953	25463	Mollusca	7805	Gastropod
Early Pleistocene	2.588	0.781	Olsson anc	1953	25463	Mollusca	7805	Gastropod
Early Pleistocene	2.588	0.781	Olsson anc	1953	25463	Mollusca	7805	Gastropod
Early Pleistocene	2.588	0.781	Olsson anc	1953	25463	Mollusca	7805	Gastropod
Early Pleistocene	2.588	0.781	Olsson anc	1953	25463	Mollusca	7805	Gastropod
Early Pleistocene	2.588	0.781	Olsson anc	1953	25463	Mollusca	7805	Gastropod
Early Pleistocene	2.588	0.781	Olsson anc	1953	25463	Mollusca	7805	Gastropod
Early Pleistocene	2.588	0.781	Olsson anc	1953	25463	Mollusca	7805	Gastropod
Early Pleistocene	2.588	0.781	Olsson anc	1953	25463	Mollusca	7805	Gastropod
Early Pleistocene	2.588	0.781	Olsson anc	1953	25463	Mollusca	7805	Gastropod
Early Pleistocene	2.588	0.781	Olsson anc	1953	25463	Mollusca	7805	Gastropod
Early Pleistocene	2.588	0.781	Olsson anc	1953	25463	Mollusca	7805	Gastropod
Late Pliocene	3.6	2.588	Petuch anc	2007	26813	Mollusca	7805	Gastropod
Late Pliocene	3.6	2.588	Petuch anc	2007	26813	Mollusca	7805	Gastropod
Late Pliocene	3.6	2.588	Petuch anc	2007	26813	Mollusca	7805	Gastropod
Early Pleistocene	2.588	0.781	DuBar	1958	25514	Mollusca	7805	Gastropod
Late Pleistocene	0.126	0.0117	DuBar	1958	25514	Mollusca	7805	Gastropod
Late Pliocene	3.6	2.588	Hendy et a	2008	19909	Mollusca	7805	Gastropod
Early Pleistocene	2.588	0.781	Hendy et a	2008	19909	Mollusca	7805	Gastropod
Pleistocene	2.588	0.0117	Hendy et a	2008	19909	Mollusca	7805	Gastropod
Late Pliocene	3.6	2.588	Hendy et a	2008	19909	Mollusca	7805	Gastropod
Middle Pleistocene	0.781	0.126	Hendy et a	2008	19909	Mollusca	7805	Gastropod
Middle Pleistocene	0.781	0.126	Petuch	1988	31112	Mollusca	7805	Gastropod
Early Pleistocene	2.588	0.781	Jung	1994	19070	Mollusca	7805	Gastropod
Early Pleistocene	2.588	0.781	Jung	1989	26828	Mollusca	7805	Gastropod
Late Pleistocene	0.126	0.0117	Kittle and I	2010	40884	Mollusca	7805	Gastropod
Late Pleistocene	0.126	0.0117	Kittle and I	2010	40884	Mollusca	7805	Gastropod

Late Pleistocene	0.126	0.0117	Kittle and I	2010	40884	Mollusca	7805	Gastropod
Late Pleistocene	0.126	0.0117	Kittle and I	2010	40884	Mollusca	7805	Gastropod
Late Pleistocene	0.126	0.0117	Kittle and I	2010	40884	Mollusca	7805	Gastropod
Late Pleistocene	0.126	0.0117	Kittle and I	2010	40884	Mollusca	7805	Gastropod
Pliocene	5.333	2.588	Petuch	1986	47440	Mollusca	7805	Gastropod
Late Pleistocene	0.126	0.0117	DuBar	1971	17556	Mollusca	7805	Gastropod
Late Pleistocene	0.126	0.0117	DuBar	1971	17556	Mollusca	7805	Gastropod
Late Pleistocene	0.126	0.0117	DuBar	1971	17556	Mollusca	7805	Gastropod
Late Pleistocene	0.126	0.0117	DuBar	1971	17556	Mollusca	7805	Gastropod
Late Pleistocene	0.126	0.0117	DuBar	1971	17556	Mollusca	7805	Gastropod
Late Pleistocene	0.126	0.0117	DuBar	1971	17556	Mollusca	7805	Gastropod
Late Pleistocene	0.126	0.0117	DuBar	1971	17556	Mollusca	7805	Gastropod
Early Pleistocene	2.588	0.781	DuBar	1971	17556	Mollusca	7805	Gastropod
Early Pleistocene	2.588	0.781	DuBar	1971	17556	Mollusca	7805	Gastropod
Late Pleistocene	0.126	0.0117	Richards	1962	18974	Mollusca	7805	Gastropod
Late Pleistocene	0.126	0.0117	Richards	1962	18974	Mollusca	7805	Gastropod
Late Pleistocene	0.126	0.0117	Richards	1962	18974	Mollusca	7805	Gastropod
Late Pleistocene	0.126	0.0117	Richards	1962	18974	Mollusca	7805	Gastropod
Late Pleistocene	0.126	0.0117	Richards	1962	18974	Mollusca	7805	Gastropod
Late Pleistocene	0.126	0.0117	Richards	1962	18974	Mollusca	7805	Gastropod
Late Pleistocene	0.126	0.0117	Richards	1962	18974	Mollusca	7805	Gastropod
Late Pleistocene	0.126	0.0117	Hendy et a	2008	19909	Mollusca	7805	Gastropod
Early Pleistocene	2.588	0.781	Cooke	1936	31145	Mollusca	7805	Gastropod
Late Pleistocene	0.126	0.0117	Holmes	1860	64712	Mollusca	7805	Gastropod
Pleistocene	2.588	0.0117	Holmes	1860	64712	Mollusca	7805	Gastropod
Late Pliocene	3.6	2.588	Ward and I	1987	16700	Mollusca	7805	Gastropod
Late Pliocene	3.6	2.588	Ward and I	1987	16700	Mollusca	7805	Gastropod
Late Pliocene	3.6	2.588	Ward and I	1987	16700	Mollusca	7805	Gastropod
Early Pleistocene	2.588	0.781	Ward and I	1987	16700	Mollusca	7805	Gastropod
Early Pleistocene	2.588	0.781	Ward and I	1987	16700	Mollusca	7805	Gastropod
Early Pleistocene	2.588	0.781	Ward and I	1987	16700	Mollusca	7805	Gastropod
Early Pleistocene	2.588	0.781	Ward and I	1987	16700	Mollusca	7805	Gastropod
Early Plioc Late Plioce	5.333	2.588	Gardner	1948	17048	Mollusca	7805	Gastropod
Late Pliocene	3.6	2.588	Gardner	1948	17048	Mollusca	7805	Gastropod
Late Pliocene	3.6	2.588	Gardner	1948	17048	Mollusca	7805	Gastropod
Pleistocene	2.588	0.0117	Gardner	1948	17048	Mollusca	7805	Gastropod
Early Pleistocene	2.588	0.781	Gardner	1948	17048	Mollusca	7805	Gastropod
Early Pleistocene	2.588	0.781	Gardner	1948	17048	Mollusca	7805	Gastropod
Early Pleistocene	2.588	0.781	Gardner	1948	17048	Mollusca	7805	Gastropod
Late Pliocene	3.6	2.588	Gardner	1948	17048	Mollusca	7805	Gastropod
Late Pliocene	3.6	2.588	Gardner	1948	17048	Mollusca	7805	Gastropod
Pleistocene	2.588	0.0117	Gardner	1948	17048	Mollusca	7805	Gastropod
Late Pliocene	3.6	2.588	Gardner	1948	17048	Mollusca	7805	Gastropod
Early Pleistocene	2.588	0.781	Gardner	1948	17048	Mollusca	7805	Gastropod
Early Pleistocene	2.588	0.781	Gardner	1948	17048	Mollusca	7805	Gastropod
Early Pleistocene	2.588	0.781	Gardner	1948	17048	Mollusca	7805	Gastropod
Early Plioc Late Plioce	5.333	2.588	Gardner	1948	17048	Mollusca	7805	Gastropod

Early Pleistocene	2.588	0.781	Gardner	1948	17048	Mollusca	7805	Gastropod
Late Pliocene	3.6	2.588	Gardner	1948	17048	Mollusca	7805	Gastropod
Early Pleistocene	2.588	0.781	Gardner	1948	17048	Mollusca	7805	Gastropod
Early Pleistocene	2.588	0.781	Gardner	1948	17048	Mollusca	7805	Gastropod
Late Pliocene	3.6	2.588	Gardner	1948	17048	Mollusca	7805	Gastropod
Early Pleistocene	2.588	0.781	DuBar	1971	17556	Mollusca	7805	Gastropod
Early Pleistocene	2.588	0.781	Miller	1990	17561	Mollusca	7805	Gastropod
Early Pleistocene	2.588	0.781	Miller	1990	17561	Mollusca	7805	Gastropod
Early Pleistocene	2.588	0.781	Miller	1990	17561	Mollusca	7805	Gastropod
Early Pleistocene	2.588	0.781	Miller	1990	17561	Mollusca	7805	Gastropod
Early Pleistocene	2.588	0.781	Miller	1990	17561	Mollusca	7805	Gastropod
Early Pleistocene	2.588	0.781	Miller	1990	17561	Mollusca	7805	Gastropod
Early Pleistocene	2.588	0.781	Miller	1990	17561	Mollusca	7805	Gastropod
Early Pleistocene	2.588	0.781	Miller	1990	17561	Mollusca	7805	Gastropod
Early Pleistocene	2.588	0.781	Miller	1990	17561	Mollusca	7805	Gastropod
Early Pleistocene	2.588	0.781	Miller	1990	17561	Mollusca	7805	Gastropod
Early Pleistocene	2.588	0.781	Miller	1990	17561	Mollusca	7805	Gastropod
Early Pleistocene	2.588	0.781	Miller	1990	17561	Mollusca	7805	Gastropod
Early Pleistocene	2.588	0.781	Miller	1990	17561	Mollusca	7805	Gastropod
Late Pleistocene	0.126	0.0117	Richards	1962	18974	Mollusca	7805	Gastropod
Late Pleistocene	0.126	0.0117	Richards	1962	18974	Mollusca	7805	Gastropod
Late Pleistocene	0.126	0.0117	Richards	1962	18974	Mollusca	7805	Gastropod
Late Pleistocene	0.126	0.0117	Richards	1962	18974	Mollusca	7805	Gastropod
Late Pleistocene	0.126	0.0117	Richards	1962	18974	Mollusca	7805	Gastropod
Late Pleistocene	0.126	0.0117	Richards	1962	18974	Mollusca	7805	Gastropod
Late Pleistocene	0.126	0.0117	Richards	1962	18974	Mollusca	7805	Gastropod
Late Pleistocene	0.126	0.0117	Richards	1962	18974	Mollusca	7805	Gastropod
Late Pleistocene	0.126	0.0117	Richards	1962	18974	Mollusca	7805	Gastropod
Gelasian	2.588	1.806	Bailey and	1986	23840	Mollusca	7805	Gastropod
Late Pliocene	3.6	2.588	Hendy et a	2008	19909	Mollusca	7805	Gastropod
Early Pleistocene	2.588	0.781	Hendy et a	2008	19909	Mollusca	7805	Gastropod
Early Pleistocene	2.588	0.781	Hendy et a	2008	19909	Mollusca	7805	Gastropod
Early Pleistocene	2.588	0.781	Hendy et a	2008	19909	Mollusca	7805	Gastropod
Early Pleistocene	2.588	0.781	Miller and	1988	72890	Mollusca	7805	Gastropod
Early Pleistocene	2.588	0.781	Miller and	1988	72890	Mollusca	7805	Gastropod
Early Pleistocene	2.588	0.781	Miller and	1988	72890	Mollusca	7805	Gastropod
Early Pleistocene	2.588	0.781	Miller and	1988	72890	Mollusca	7805	Gastropod
Early Pleistocene	2.588	0.781	Miller and	1988	72890	Mollusca	7805	Gastropod
Early Pleistocene	2.588	0.781	Miller and	1988	72890	Mollusca	7805	Gastropod
Early Pleistocene	2.588	0.781	Miller and	1988	72890	Mollusca	7805	Gastropod
Early Pleistocene	2.588	0.781	Miller and	1988	72890	Mollusca	7805	Gastropod
Late Pleistocene	0.126	0.0117	Spencer ar	1987	15525	Mollusca	7805	Gastropod
Late Pleistocene	0.126	0.0117	Spencer ar	1987	15525	Mollusca	7805	Gastropod
Late Pleistocene	0.126	0.0117	Spencer ar	1987	15525	Mollusca	7805	Gastropod
Late Pleistocene	0.126	0.0117	Spencer ar	1987	15525	Mollusca	7805	Gastropod
Late Pleistocene	0.126	0.0117	Hendy et a	2008	19909	Mollusca	7805	Gastropod
Late Pleistocene	0.126	0.0117	Hendy et a	2008	19909	Mollusca	7805	Gastropod

Late Pleistocene		0.126	0.0117	Hendy et a	2008	19909	Mollusca	7805	Gastropod
Late Pliocene		3.6	2.588	Ward	1993	26351	Mollusca	7805	Gastropod
Late Pliocene		3.6	2.588	Ward	1993	26351	Mollusca	7805	Gastropod
Late Pliocene		3.6	2.588	Ward	1993	26351	Mollusca	7805	Gastropod
Late Pliocene		3.6	2.588	Ward	1993	26351	Mollusca	7805	Gastropod
Late Pliocene		3.6	2.588	Ward	1993	26351	Mollusca	7805	Gastropod
Late Pliocene		3.6	2.588	Ward	1993	26351	Mollusca	7805	Gastropod
Late Pliocene		3.6	2.588	Ward	1993	26351	Mollusca	7805	Gastropod
Late Pliocene		3.6	2.588	Ward	1993	26351	Mollusca	7805	Gastropod
Late Pliocene		3.6	2.588	Ward	1993	26351	Mollusca	7805	Gastropod
Late Pliocene		3.6	2.588	Ward	1993	26351	Mollusca	7805	Gastropod
Late Pliocene		3.6	2.588	Ward	1993	26351	Mollusca	7805	Gastropod
Late Pliocene		3.6	2.588	Ward	1993	26351	Mollusca	7805	Gastropod
Late Pliocene		3.6	2.588	Ward	1993	26351	Mollusca	7805	Gastropod
Late Pliocene		3.6	2.588	Hendy et a	2008	19909	Mollusca	7805	Gastropod
Late Pliocene		3.6	2.588	Hendy et a	2008	19909	Mollusca	7805	Gastropod
Early Pleistocene		2.588	0.781	DuBar	1962	11119	Mollusca	7805	Gastropod
Early Pleistocene		2.588	0.781	DuBar	1962	11119	Mollusca	7805	Gastropod
Early Pleistocene		2.588	0.781	DuBar	1962	11119	Mollusca	7805	Gastropod
Early Pleistocene		2.588	0.781	DuBar	1962	11119	Mollusca	7805	Gastropod
Early Pleistocene		2.588	0.781	DuBar	1962	11119	Mollusca	7805	Gastropod
Calabrian		1.806	0.781	DuBar	1962	11119	Mollusca	7805	Gastropod
Late Pleistocene		0.126	0.0117	DuBar	1962	11119	Mollusca	7805	Gastropod
Early Pleistocene		2.588	0.781	DuBar	1962	11119	Mollusca	7805	Gastropod
Pliocene	Pleistocen	5.333	0.0117	DuBar	1962	11119	Mollusca	7805	Gastropod
Pliocene	Pleistocen	5.333	0.0117	DuBar	1962	11119	Mollusca	7805	Gastropod
Pliocene	Pleistocen	5.333	0.0117	DuBar	1962	11119	Mollusca	7805	Gastropod
Pliocene	Pleistocen	5.333	0.0117	DuBar	1962	11119	Mollusca	7805	Gastropod
Pliocene	Pleistocen	5.333	0.0117	DuBar	1962	11119	Mollusca	7805	Gastropod
Late Pliocene		3.6	2.588	Mansfield	1930	17060	Mollusca	7805	Gastropod
Late Pliocene		3.6	2.588	Mansfield	1930	17060	Mollusca	7805	Gastropod
Late Pliocene		3.6	2.588	Mansfield	1930	17060	Mollusca	7805	Gastropod
Late Pliocene		3.6	2.588	Mansfield	1930	17060	Mollusca	7805	Gastropod
Late Pliocene		3.6	2.588	Mansfield	1932	17094	Mollusca	7805	Gastropod
Late Pliocene		3.6	2.588	Hendricks	2008	29967	Mollusca	7805	Gastropod
Late Pliocene		3.6	2.588	Mansfield	1932	17094	Mollusca	7805	Gastropod
Early Pleistocene		2.588	0.781	Portell et a	1992	17634	Mollusca	7805	Gastropod
Middle Pleistocene		0.781	0.126	Portell et a	1992	17634	Mollusca	7805	Gastropod
Late Pleistocene		0.126	0.0117	Portell et a	1992	17634	Mollusca	7805	Gastropod
Late Pleistocene		0.126	0.0117	Petuch	2004	18040	Mollusca	7805	Gastropod
Late Pleistocene		0.126	0.0117	Hendy et a	2008	19909	Mollusca	7805	Gastropod
Late Pleistocene		0.126	0.0117	Hendy et a	2008	19909	Mollusca	7805	Gastropod
Pliocene		5.333	2.588	Schroeder	1954	25303	Mollusca	7805	Gastropod
Early Pleistocene		2.588	0.781	Schroeder	1954	25303	Mollusca	7805	Gastropod
Early Pleistocene		2.588	0.781	Olsson anc	1953	25463	Mollusca	7805	Gastropod
Early Pleistocene		2.588	0.781	Olsson anc	1953	25463	Mollusca	7805	Gastropod
Early Pleistocene		2.588	0.781	Olsson anc	1953	25463	Mollusca	7805	Gastropod

Early Pleistocene	2.588	0.781	Olsson anc	1953	25463	Mollusca	7805	Gastropod
Early Pleistocene	2.588	0.781	Olsson anc	1953	25463	Mollusca	7805	Gastropod
Early Pleistocene	2.588	0.781	Olsson anc	1953	25463	Mollusca	7805	Gastropod
Early Pleistocene	2.588	0.781	Olsson anc	1953	25463	Mollusca	7805	Gastropod
Early Pleistocene	2.588	0.781	Olsson anc	1953	25463	Mollusca	7805	Gastropod
Early Pleistocene	2.588	0.781	Olsson anc	1953	25463	Mollusca	7805	Gastropod
Early Pleistocene	2.588	0.781	Olsson anc	1953	25463	Mollusca	7805	Gastropod
Early Pleistocene	2.588	0.781	Olsson anc	1953	25463	Mollusca	7805	Gastropod
Early Pleistocene	2.588	0.781	Olsson anc	1953	25463	Mollusca	7805	Gastropod
Early Pleistocene	2.588	0.781	Olsson anc	1953	25463	Mollusca	7805	Gastropod
Early Pleistocene	2.588	0.781	Olsson anc	1953	25463	Mollusca	7805	Gastropod
Early Pleistocene	2.588	0.781	Olsson anc	1953	25463	Mollusca	7805	Gastropod
Early Pleistocene	2.588	0.781	Olsson anc	1953	25463	Mollusca	7805	Gastropod
Early Pleistocene	2.588	0.781	Olsson anc	1953	25463	Mollusca	7805	Gastropod
Early Pleistocene	2.588	0.781	Olsson anc	1953	25463	Mollusca	7805	Gastropod
Late Pleistocene	0.126	0.0117	Petuch anc	2007	26813	Mollusca	7805	Gastropod
Late Pleistocene	0.126	0.0117	Petuch anc	2007	26813	Mollusca	7805	Gastropod
Late Pleistocene	0.126	0.0117	Petuch anc	2007	26813	Mollusca	7805	Gastropod
Late Pleistocene	0.126	0.0117	Petuch anc	2007	26813	Mollusca	7805	Gastropod
Middle Pleistocene	0.781	0.126	Petuch anc	2007	26813	Mollusca	7805	Gastropod
Middle Pleistocene	0.781	0.126	Petuch anc	2007	26813	Mollusca	7805	Gastropod
Early Pleistocene	2.588	0.781	Petuch anc	2007	26813	Mollusca	7805	Gastropod
Middle Pleistocene	0.781	0.126	Petuch anc	2007	26813	Mollusca	7805	Gastropod
Middle Pleistocene	0.781	0.126	Petuch anc	2007	26813	Mollusca	7805	Gastropod
Middle Pleistocene	0.781	0.126	Petuch anc	2007	26813	Mollusca	7805	Gastropod
Middle Pleistocene	0.781	0.126	Petuch anc	2007	26813	Mollusca	7805	Gastropod
Middle Pleistocene	0.781	0.126	Petuch anc	2007	26813	Mollusca	7805	Gastropod
Middle Pleistocene	0.781	0.126	Petuch anc	2007	26813	Mollusca	7805	Gastropod
Late Pliocene	3.6	2.588	Olsson	1967	19826	Mollusca	7805	Gastropod
Early Pleistocene	2.588	0.781	Petuch anc	2007	26813	Mollusca	7805	Gastropod
Early Pleistocene	2.588	0.781	Petuch anc	2007	26813	Mollusca	7805	Gastropod
Early Pleistocene	2.588	0.781	Petuch anc	2007	26813	Mollusca	7805	Gastropod
Early Pleistocene	2.588	0.781	Petuch anc	2007	26813	Mollusca	7805	Gastropod
Early Pleistocene	2.588	0.781	Petuch anc	2007	26813	Mollusca	7805	Gastropod
Early Pleistocene	2.588	0.781	Petuch anc	2007	26813	Mollusca	7805	Gastropod
Early Pleistocene	2.588	0.781	Petuch anc	2007	26813	Mollusca	7805	Gastropod
Late Plioce Early Pleist	3.6	0.781	Petuch anc	2007	26813	Mollusca	7805	Gastropod
Late Plioce Early Pleist	3.6	0.781	Petuch anc	2007	26813	Mollusca	7805	Gastropod
Late Plioce Early Pleist	3.6	0.781	Petuch anc	2007	26813	Mollusca	7805	Gastropod
Late Plioce Early Pleist	3.6	0.781	Petuch anc	2007	26813	Mollusca	7805	Gastropod
Late Plioce Early Pleist	3.6	0.781	Petuch anc	2007	26813	Mollusca	7805	Gastropod
Late Plioce Pleistocen	3.6	0.0117	Petuch anc	2007	26813	Mollusca	7805	Gastropod
Late Plioce Pleistocen	3.6	0.0117	Petuch anc	2007	26813	Mollusca	7805	Gastropod
Late Pliocene	3.6	2.588	Petuch anc	2007	26813	Mollusca	7805	Gastropod
Late Pliocene	3.6	2.588	Petuch anc	2007	26813	Mollusca	7805	Gastropod
Late Pliocene	3.6	2.588	Petuch anc	2007	26813	Mollusca	7805	Gastropod
Late Pliocene	3.6	2.588	Petuch anc	2007	26813	Mollusca	7805	Gastropod

Late Pliocene	3.6	2.588	Petuch and	2007	26813	Mollusca	7805	Gastropod
Early Pleistocene	2.588	0.781	Hendy et a	2008	19909	Mollusca	7805	Gastropod
Early Pleistocene	2.588	0.781	Hendy et a	2008	19909	Mollusca	7805	Gastropod
Late Pliocene	3.6	2.588	Petuch and	2007	26813	Mollusca	7805	Gastropod
Late Pliocene	3.6	2.588	Petuch and	2007	26813	Mollusca	7805	Gastropod
Late Pliocene	3.6	2.588	Petuch and	2007	26813	Mollusca	7805	Gastropod
Late Pliocene	3.6	2.588	Petuch and	2007	26813	Mollusca	7805	Gastropod
Late Pliocene	3.6	2.588	Petuch and	2007	26813	Mollusca	7805	Gastropod
Late Pliocene	3.6	2.588	Petuch and	2007	26813	Mollusca	7805	Gastropod
Late Pliocene	3.6	2.588	Petuch and	2007	26813	Mollusca	7805	Gastropod
Late Pliocene	3.6	2.588	Petuch and	2007	26813	Mollusca	7805	Gastropod
Late Pliocene	3.6	2.588	Petuch and	2007	26813	Mollusca	7805	Gastropod
Late Pliocene	3.6	2.588	Petuch and	2007	26813	Mollusca	7805	Gastropod
Late Pliocene	3.6	2.588	Petuch and	2007	26813	Mollusca	7805	Gastropod
Late Pliocene	3.6	2.588	Petuch and	2007	26813	Mollusca	7805	Gastropod
Late Pliocene	3.6	2.588	Petuch and	2007	26813	Mollusca	7805	Gastropod
Late Pliocene	3.6	2.588	Petuch and	2007	26813	Mollusca	7805	Gastropod
Late Pliocene	3.6	2.588	Petuch and	2007	26813	Mollusca	7805	Gastropod
Late Pliocene	3.6	2.588	Petuch and	2007	26813	Mollusca	7805	Gastropod
Late Pliocene	3.6	2.588	Petuch and	2007	26813	Mollusca	7805	Gastropod
Late Pliocene	3.6	2.588	Petuch and	2007	26813	Mollusca	7805	Gastropod
Late Pleistocene	0.126	0.0117	Hendy et a	2008	19909	Mollusca	7805	Gastropod
Early Pleistocene	2.588	0.781	Hendy et a	2008	19909	Mollusca	7805	Gastropod
Early Pleistocene	2.588	0.781	Hendy et a	2008	19909	Mollusca	7805	Gastropod
Early Pleistocene	2.588	0.781	Hendy et a	2008	19909	Mollusca	7805	Gastropod
Middle Pleistocene	0.781	0.126	Hendy et a	2008	19909	Mollusca	7805	Gastropod
Middle Pleistocene	0.781	0.126	Hendy et a	2008	19909	Mollusca	7805	Gastropod
Late Pleistocene	0.126	0.0117	Hendy et a	2008	19909	Mollusca	7805	Gastropod
Early Pleistocene	2.588	0.781	DuBar	1958	25514	Mollusca	7805	Gastropod
Early Pleistocene	2.588	0.781	DuBar	1958	25514	Mollusca	7805	Gastropod
Late Pliocene	3.6	2.588	Weisbord	1974	28823	Mollusca	7805	Gastropod
Late Pliocene	3.6	2.588	Weisbord	1974	28823	Mollusca	7805	Gastropod
Late Pliocene	3.6	2.588	Weisbord	1972	31274	Mollusca	7805	Gastropod
Late Pliocene	3.6	2.588	Weisbord	1972	31274	Mollusca	7805	Gastropod
Late Pliocene	3.6	2.588	Petuch	1988	31112	Mollusca	7805	Gastropod
Late Pliocene	3.6	2.588	Petuch	1988	31112	Mollusca	7805	Gastropod
Early Pleistocene	2.588	0.781	Petuch	1988	31112	Mollusca	7805	Gastropod
Middle Pleistocene	0.781	0.126	Petuch	1988	31112	Mollusca	7805	Gastropod
Middle Pleistocene	0.781	0.126	Petuch	1988	31112	Mollusca	7805	Gastropod
Middle Pleistocene	0.781	0.126	Petuch	1988	31112	Mollusca	7805	Gastropod
Middle Pleistocene	0.781	0.126	Petuch	1988	31112	Mollusca	7805	Gastropod
Middle Pleistocene	0.781	0.126	Petuch	1988	31112	Mollusca	7805	Gastropod
Late Pliocene	3.6	2.588	Weisbord	1971	39743	Mollusca	7805	Gastropod
Late Pleistocene	0.126	0.0117	Kittle and I	2010	40884	Mollusca	7805	Gastropod
Late Pleistocene	0.126	0.0117	Kittle and I	2010	40884	Mollusca	7805	Gastropod
Late Pleistocene	0.126	0.0117	Kittle and I	2010	40884	Mollusca	7805	Gastropod
Pliocene	5.333	2.588	Petuch	1986	47440	Mollusca	7805	Gastropod

Pliocene	5.333	2.588	Petuch	1986	47440	Mollusca	7805	Gastropod
Pliocene	5.333	2.588	Petuch	1986	47440	Mollusca	7805	Gastropod
Pliocene	5.333	2.588	Petuch	1986	47440	Mollusca	7805	Gastropod
Late Pleistocene	0.126	0.0117	DuBar	1971	17556	Mollusca	7805	Gastropod
Zanclean	5.333	3.6	Campbell a	1995	17558	Mollusca	7805	Gastropod
Zanclean	5.333	3.6	Campbell a	1995	17558	Mollusca	7805	Gastropod
Zanclean	5.333	3.6	Campbell a	1995	17558	Mollusca	7805	Gastropod
Early Pleistocene	2.588	0.781	DuBar	1971	17556	Mollusca	7805	Gastropod
Late Pleistocene	0.126	0.0117	Hendy et a	2008	19909	Mollusca	7805	Gastropod
Early Pleistocene	2.588	0.781	Cooke	1936	31145	Mollusca	7805	Gastropod
Pliocene	5.333	2.588	Hendy et a	2008	19909	Mollusca	7805	Gastropod
Early Pleistocene	2.588	0.781	Hendy et a	2008	19909	Mollusca	7805	Gastropod
Early Pleistocene	2.588	0.781	Hendy et a	2008	19909	Mollusca	7805	Gastropod
Early Pliocene	5.333	3.6	Ward	1992	16985	Mollusca	7805	Gastropod
Late Pliocene	3.6	2.588	Ward	1993	26351	Mollusca	7805	Gastropod
Late Pliocene	3.6	2.588	Ward	1993	26351	Mollusca	7805	Gastropod
Pliocene	5.333	2.588	Petuch	1988	31112	Mollusca	7805	Gastropod
Late Pliocene	3.6	2.588	Mansfield	1930	17060	Mollusca	7805	Gastropod
Early Pleistocene	2.588	0.781	Olsson anc	1953	25463	Mollusca	7805	Gastropod
Early Pleistocene	2.588	0.781	Olsson anc	1953	25463	Mollusca	7805	Gastropod
Early Pleistocene	2.588	0.781	Olsson anc	1953	25463	Mollusca	7805	Gastropod
Early Pleistocene	2.588	0.781	Olsson anc	1953	25463	Mollusca	7805	Gastropod
Early Pleistocene	2.588	0.781	Olsson anc	1953	25463	Mollusca	7805	Gastropod
Late Plioce Early Pleist	3.6	0.781	Petuch anc	2007	26813	Mollusca	7805	Gastropod
Early Pleistocene	2.588	0.781	Cooke	1936	31145	Mollusca	7805	Gastropod
Pleistocene	2.588	0.0117	Holmes	1860	64712	Mollusca	7805	Gastropod
Late Pliocene	3.6	2.588	Ward and I	1987	16700	Mollusca	7805	Gastropod
Early Pleistocene	2.588	0.781	Ward and I	1987	16700	Mollusca	7805	Gastropod
Early Pleistocene	2.588	0.781	Hendy et a	2008	19909	Mollusca	7805	Gastropod
Early Pleistocene	2.588	0.781	DuBar	1962	11119	Mollusca	7805	Gastropod
Early Pleistocene	2.588	0.781	DuBar	1962	11119	Mollusca	7805	Gastropod
Early Pleistocene	2.588	0.781	DuBar	1962	11119	Mollusca	7805	Gastropod
Calabrian	1.806	0.781	DuBar	1962	11119	Mollusca	7805	Gastropod
Calabrian	1.806	0.781	DuBar	1962	11119	Mollusca	7805	Gastropod
Late Pleistocene	0.126	0.0117	DuBar	1962	11119	Mollusca	7805	Gastropod
Early Pleistocene	2.588	0.781	DuBar	1962	11119	Mollusca	7805	Gastropod
Late Pleistocene	0.126	0.0117	DuBar	1962	11119	Mollusca	7805	Gastropod
Late Pliocene	3.6	2.588	Mansfield	1930	17060	Mollusca	7805	Gastropod
Late Pliocene	3.6	2.588	Mansfield	1930	17060	Mollusca	7805	Gastropod
Late Pliocene	3.6	2.588	Mansfield	1930	17060	Mollusca	7805	Gastropod
Late Pliocene	3.6	2.588	Mansfield	1930	17060	Mollusca	7805	Gastropod
Late Pliocene	3.6	2.588	Mansfield	1930	17060	Mollusca	7805	Gastropod
Late Pliocene	3.6	2.588	Mansfield	1930	17060	Mollusca	7805	Gastropod
Late Pliocene	3.6	2.588	Mansfield	1930	17060	Mollusca	7805	Gastropod
Late Pliocene	3.6	2.588	Mansfield	1932	17094	Mollusca	7805	Gastropod
Early Pleistocene	2.588	0.781	Portell et a	1992	17634	Mollusca	7805	Gastropod
Late Pleistocene	0.126	0.0117	Portell et a	1992	17634	Mollusca	7805	Gastropod

Middle Pleistocene	0.781	0.126	Portell et a	1992	17634	Mollusca	7805	Gastropod
Middle Pleistocene	0.781	0.126	Portell et a	1992	17634	Mollusca	7805	Gastropod
Early Pleistocene	2.588	0.781	Schroeder	1954	25303	Mollusca	7805	Gastropod
Early Pleistocene	2.588	0.781	Olsson anc	1953	25463	Mollusca	7805	Gastropod
Early Pleistocene	2.588	0.781	Olsson anc	1953	25463	Mollusca	7805	Gastropod
Early Pleistocene	2.588	0.781	Olsson anc	1953	25463	Mollusca	7805	Gastropod
Early Pleistocene	2.588	0.781	Olsson anc	1953	25463	Mollusca	7805	Gastropod
Late Pleistocene	0.126	0.0117	DuBar	1958	25514	Mollusca	7805	Gastropod
Early Pleistocene	2.588	0.781	DuBar	1958	25514	Mollusca	7805	Gastropod
Early Pleistocene	2.588	0.781	DuBar	1958	25514	Mollusca	7805	Gastropod
Late Pleistocene	0.126	0.0117	DuBar	1958	25514	Mollusca	7805	Gastropod
Early Pleistocene	2.588	0.781	DuBar	1958	25514	Mollusca	7805	Gastropod
Late Pleistocene	0.126	0.0117	Kittle and I	2010	40884	Mollusca	7805	Gastropod
Late Pleistocene	0.126	0.0117	Kittle and I	2010	40884	Mollusca	7805	Gastropod
Late Pleistocene	0.126	0.0117	Richards	1962	18974	Mollusca	7805	Gastropod
Late Pleistocene	0.126	0.0117	DuBar	1971	17556	Mollusca	7805	Gastropod
Late Pleistocene	0.126	0.0117	DuBar	1971	17556	Mollusca	7805	Gastropod
Early Pleistocene	2.588	0.781	DuBar	1971	17556	Mollusca	7805	Gastropod
Late Pleistocene	0.126	0.0117	Richards	1962	18974	Mollusca	7805	Gastropod
Late Pleistocene	0.126	0.0117	Richards	1962	18974	Mollusca	7805	Gastropod
Late Pleistocene	0.126	0.0117	Hendy et a	2008	19909	Mollusca	7805	Gastropod
Late Pleistocene	0.126	0.0117	Hendy et a	2008	19909	Mollusca	7805	Gastropod
Late Pleistocene	0.126	0.0117	Hendy et a	2008	19909	Mollusca	7805	Gastropod
Early Pleistocene	2.588	0.781	Cooke	1936	31145	Mollusca	7805	Gastropod
Late Pliocene	3.6	2.588	Ward and I	1987	16700	Mollusca	7805	Gastropod
Early Pleistocene	2.588	0.781	Ward and I	1987	16700	Mollusca	7805	Gastropod
Early Pleistocene	2.588	0.781	Gardner	1948	17048	Mollusca	7805	Gastropod
Late Pliocene	3.6	2.588	Gardner	1948	17048	Mollusca	7805	Gastropod
Early Pleistocene	2.588	0.781	Miller	1990	17561	Mollusca	7805	Gastropod
Late Pleistocene	0.126	0.0117	Richards	1962	18974	Mollusca	7805	Gastropod
Late Pleistocene	0.126	0.0117	Richards	1962	18974	Mollusca	7805	Gastropod
Late Pleistocene	0.126	0.0117	Richards	1962	18974	Mollusca	7805	Gastropod
Late Pleistocene	0.126	0.0117	Richards	1962	18974	Mollusca	7805	Gastropod
Late Pleistocene	0.126	0.0117	Richards	1962	18974	Mollusca	7805	Gastropod
Late Pleistocene	0.126	0.0117	Richards	1962	18974	Mollusca	7805	Gastropod
Gelasian	2.588	1.806	Bailey and	1986	23840	Mollusca	7805	Gastropod
Late Pleistocene	0.126	0.0117	Spencer ar	1987	15525	Mollusca	7805	Gastropod
Late Pleistocene	0.126	0.0117	Spencer ar	1987	15525	Mollusca	7805	Gastropod
Late Pleistocene	0.126	0.0117	Spencer ar	1987	15525	Mollusca	7805	Gastropod
Late Pleistocene	0.126	0.0117	Spencer ar	1987	15525	Mollusca	7805	Gastropod
Late Pliocene	3.6	2.588	Edwards et	2005	17631	Mollusca	7805	Gastropod
Late Pliocene	3.6	2.588	Ward	1993	26351	Mollusca	7805	Gastropod
Late Pliocene	3.6	2.588	Ward	1993	26351	Mollusca	7805	Gastropod
Late Pliocene	3.6	2.588	Ward	1993	26351	Mollusca	7805	Gastropod
Late Pliocene	3.6	2.588	Ward	1993	26351	Mollusca	7805	Gastropod
Late Pliocene	3.6	2.588	Ward	1993	26351	Mollusca	7805	Gastropod
Late Pliocene	3.6	2.588	Ward	1993	26351	Mollusca	7805	Gastropod

Late Pliocene		3.6	2.588	Ward	1993	26351	Mollusca	7805	Gastropod
Late Pliocene		3.6	2.588	Ward	1993	26351	Mollusca	7805	Gastropod
Late Pliocene		3.6	2.588	Ward	1993	26351	Mollusca	7805	Gastropod
Early Pleistocene		2.588	0.781	DuBar	1962	11119	Mollusca	7805	Gastropod
Early Pleistocene		2.588	0.781	DuBar	1962	11119	Mollusca	7805	Gastropod
Pliocene	Pleistocene	5.333	0.0117	DuBar	1962	11119	Mollusca	7805	Gastropod
Late Pliocene		3.6	2.588	Mansfield	1930	17060	Mollusca	7805	Gastropod
Middle Pleistocene		0.781	0.126	McGinty	1970	19114	Mollusca	7805	Gastropod
Late Pliocene		3.6	2.588	Mansfield	1939	19112	Mollusca	7805	Gastropod
Late Pleistocene		0.126	0.0117	Petuch and	2007	26813	Mollusca	7805	Gastropod
Middle Pleistocene		0.781	0.126	Petuch and	2007	26813	Mollusca	7805	Gastropod
Middle Pleistocene		0.781	0.126	Petuch and	2007	26813	Mollusca	7805	Gastropod
Middle Pleistocene		0.781	0.126	Petuch and	2007	26813	Mollusca	7805	Gastropod
Middle Pleistocene		0.781	0.126	Petuch and	2007	26813	Mollusca	7805	Gastropod
Early Pleistocene		2.588	0.781	Olsson and	1964	26836	Mollusca	7805	Gastropod
Early Pleistocene		2.588	0.781	Petuch and	2007	26813	Mollusca	7805	Gastropod
Late Plioce	Early Pleist	3.6	0.781	Petuch and	2007	26813	Mollusca	7805	Gastropod
Late Plioce	Early Pleist	3.6	0.781	Petuch and	2007	26813	Mollusca	7805	Gastropod
Late Plioce	Early Pleist	3.6	0.781	Petuch and	2007	26813	Mollusca	7805	Gastropod
Early Pleistocene		2.588	0.781	Petuch and	2007	26813	Mollusca	7805	Gastropod
Early Pleistocene		2.588	0.781	Petuch and	2007	26813	Mollusca	7805	Gastropod
Early Pleistocene		2.588	0.781	Petuch and	2007	26813	Mollusca	7805	Gastropod
Late Plioce	Pleistocene	3.6	0.0117	Petuch and	2007	26813	Mollusca	7805	Gastropod
Late Plioce	Pleistocene	3.6	0.0117	Petuch and	2007	26813	Mollusca	7805	Gastropod
Late Pliocene		3.6	2.588	Petuch and	2007	26813	Mollusca	7805	Gastropod
Late Pliocene		3.6	2.588	Petuch and	2007	26813	Mollusca	7805	Gastropod
Late Pliocene		3.6	2.588	Petuch and	2007	26813	Mollusca	7805	Gastropod
Late Pliocene		3.6	2.588	Petuch and	2007	26813	Mollusca	7805	Gastropod
Late Pliocene		3.6	2.588	Petuch and	2007	26813	Mollusca	7805	Gastropod
Late Pliocene		3.6	2.588	Petuch and	2007	26813	Mollusca	7805	Gastropod
Late Pliocene		3.6	2.588	Petuch and	2007	26813	Mollusca	7805	Gastropod
Late Pliocene		3.6	2.588	Petuch and	2007	26813	Mollusca	7805	Gastropod
Late Pliocene		3.6	2.588	Petuch and	2007	26813	Mollusca	7805	Gastropod
Late Pliocene		3.6	2.588	Petuch and	2007	26813	Mollusca	7805	Gastropod
Late Pliocene		3.6	2.588	Petuch and	2007	26813	Mollusca	7805	Gastropod
Late Pliocene		3.6	2.588	Petuch and	2007	26813	Mollusca	7805	Gastropod
Late Pliocene		3.6	2.588	Petuch and	2007	26813	Mollusca	7805	Gastropod
Late Pliocene		3.6	2.588	Petuch and	2007	26813	Mollusca	7805	Gastropod
Late Pliocene		3.6	2.588	Petuch and	2007	26813	Mollusca	7805	Gastropod
Late Pliocene		3.6	2.588	Petuch and	2007	26813	Mollusca	7805	Gastropod
Late Pliocene		3.6	2.588	Petuch and	2007	26813	Mollusca	7805	Gastropod
Late Pliocene		3.6	2.588	Petuch and	2007	26813	Mollusca	7805	Gastropod
Late Pliocene		3.6	2.588	Petuch and	2007	26813	Mollusca	7805	Gastropod
Late Pliocene		3.6	2.588	Petuch and	2007	26813	Mollusca	7805	Gastropod
Late Pliocene		3.6	2.588	Petuch and	2007	26813	Mollusca	7805	Gastropod
Late Pliocene		3.6	2.588	Petuch and	2007	26813	Mollusca	7805	Gastropod
Late Pliocene		3.6	2.588	Petuch and	2007	26813	Mollusca	7805	Gastropod

Late Pliocene	3.6	2.588	Petuch anc	2007	26813	Mollusca	7805	Gastropod
Late Pliocene	3.6	2.588	Petuch anc	2007	26813	Mollusca	7805	Gastropod
Late Pliocene	3.6	2.588	Petuch anc	2007	26813	Mollusca	7805	Gastropod
Early Pleistocene	2.588	0.781	Olsson anc	1953	25463	Mollusca	7805	Gastropod
Late Pliocene	3.6	2.588	Petuch anc	2007	26813	Mollusca	7805	Gastropod
Late Pliocene	3.6	2.588	Petuch anc	2007	26813	Mollusca	7805	Gastropod
Late Pliocene	3.6	2.588	Petuch anc	2007	26813	Mollusca	7805	Gastropod
Late Pliocene	3.6	2.588	Petuch anc	2007	26813	Mollusca	7805	Gastropod
Late Pliocene	3.6	2.588	Petuch anc	2007	26813	Mollusca	7805	Gastropod
Late Pliocene	3.6	2.588	Petuch anc	2007	26813	Mollusca	7805	Gastropod
Late Pliocene	3.6	2.588	Petuch anc	2007	26813	Mollusca	7805	Gastropod
Late Pliocene	3.6	2.588	Petuch anc	2007	26813	Mollusca	7805	Gastropod
Late Pliocene	3.6	2.588	Petuch anc	2007	26813	Mollusca	7805	Gastropod
Late Pliocene	3.6	2.588	Petuch anc	2007	26813	Mollusca	7805	Gastropod
Late Pliocene	3.6	2.588	Petuch	1996	26971	Mollusca	7805	Gastropod
Late Pliocene	3.6	2.588	Petuch	1996	26971	Mollusca	7805	Gastropod
Late Pliocene	3.6	2.588	Petuch	1996	26971	Mollusca	7805	Gastropod
Late Pliocene	3.6	2.588	Petuch	1996	26971	Mollusca	7805	Gastropod
Late Pliocene	3.6	2.588	Petuch	1988	31112	Mollusca	7805	Gastropod
Late Pliocene	3.6	2.588	Petuch	1988	31112	Mollusca	7805	Gastropod
Late Pliocene	3.6	2.588	Petuch	1988	31112	Mollusca	7805	Gastropod
Late Pliocene	3.6	2.588	Petuch	1988	31112	Mollusca	7805	Gastropod
Early Pleistocene	2.588	0.781	Petuch	1988	31112	Mollusca	7805	Gastropod
Middle Pleistocene	0.781	0.126	Petuch	1988	31112	Mollusca	7805	Gastropod
Late Pleistocene	0.126	0.0117	Kittle and I	2010	40884	Mollusca	7805	Gastropod
Pliocene	5.333	2.588	Petuch	1986	47440	Mollusca	7805	Gastropod
Pliocene	5.333	2.588	Petuch	1986	47440	Mollusca	7805	Gastropod
Late Pliocene	3.6	2.588	Petuch	2003	58594	Mollusca	7805	Gastropod
Late Pliocene	3.6	2.588	Petuch	2003	58594	Mollusca	7805	Gastropod
Early Plioc Late Plioce	5.333	2.588	Gardner	1948	17048	Mollusca	7805	Gastropod
Late Pliocene	3.6	2.588	Gardner	1948	17048	Mollusca	7805	Gastropod
Late Pliocene	3.6	2.588	Gardner	1948	17048	Mollusca	7805	Gastropod
Late Pliocene	3.6	2.588	Gardner	1948	17048	Mollusca	7805	Gastropod
Late Pliocene	3.6	2.588	Gardner	1948	17048	Mollusca	7805	Gastropod
Late Pliocene	3.6	2.588	Gardner	1948	17048	Mollusca	7805	Gastropod
Zanclean	5.333	3.6	Ingram	1939	59298	Mollusca	7805	Gastropod
Early Pleistocene	2.588	0.781	DuBar	1962	11119	Mollusca	7805	Gastropod
Early Pleistocene	2.588	0.781	DuBar	1962	11119	Mollusca	7805	Gastropod
Early Pleistocene	2.588	0.781	DuBar	1962	11119	Mollusca	7805	Gastropod
Calabrian	1.806	0.781	DuBar	1962	11119	Mollusca	7805	Gastropod
Late Pliocene	3.6	2.588	Mansfield	1930	17060	Mollusca	7805	Gastropod
Early Pleistocene	2.588	0.781	Olsson anc	1953	25463	Mollusca	7805	Gastropod
Early Pleistocene	2.588	0.781	Olsson anc	1953	25463	Mollusca	7805	Gastropod
Early Pleistocene	2.588	0.781	Olsson anc	1953	25463	Mollusca	7805	Gastropod
Early Pleistocene	2.588	0.781	DuBar	1958	25514	Mollusca	7805	Gastropod
Late Pleistocene	0.126	0.0117	Kittle and I	2010	40884	Mollusca	7805	Gastropod
Late Pliocene	3.6	2.588	Ward and I	1987	16700	Mollusca	7805	Gastropod

Early Pleistocene		2.588	0.781	Ward and I	1987	16700	Mollusca	7805	Gastropod
Late Pliocene		3.6	2.588	Gardner	1948	17048	Mollusca	7805	Gastropod
Gelasian		2.588	1.806	Bailey and	1986	23840	Mollusca	7805	Gastropod
Late Pleistocene		0.126	0.0117	Spencer ar	1987	15525	Mollusca	7805	Gastropod
Late Pliocene		3.6	2.588	Ward	1993	26351	Mollusca	7805	Gastropod
Late Pliocene		3.6	2.588	Ward	1993	26351	Mollusca	7805	Gastropod
Late Pliocene		3.6	2.588	Ward	1993	26351	Mollusca	7805	Gastropod
Late Pleistocene		0.126	0.0117	Guilday et	1977	35027	Mollusca	7805	Gastropod
Late Pleistocene		0.126	0.0117	Guilday et	1977	35027	Mollusca	7805	Gastropod
Late Pleistocene		0.126	0.0117	Guilday	1962	35381	Mollusca	7805	Gastropod
Early Pleistocene		2.588	0.781	DuBar	1962	11119	Mollusca	7805	Gastropod
Early Pleistocene		2.588	0.781	DuBar	1962	11119	Mollusca	7805	Gastropod
Early Pleistocene		2.588	0.781	DuBar	1962	11119	Mollusca	7805	Gastropod
Early Pleistocene		2.588	0.781	DuBar	1962	11119	Mollusca	7805	Gastropod
Early Pleistocene		2.588	0.781	DuBar	1962	11119	Mollusca	7805	Gastropod
Early Pleistocene		2.588	0.781	DuBar	1962	11119	Mollusca	7805	Gastropod
Early Pleistocene		2.588	0.781	DuBar	1962	11119	Mollusca	7805	Gastropod
Early Pleistocene		2.588	0.781	DuBar	1962	11119	Mollusca	7805	Gastropod
Pliocene	Pleistocene	5.333	0.0117	DuBar	1962	11119	Mollusca	7805	Gastropod
Pliocene	Pleistocene	5.333	0.0117	DuBar	1962	11119	Mollusca	7805	Gastropod
Pliocene	Pleistocene	5.333	0.0117	DuBar	1962	11119	Mollusca	7805	Gastropod
Pliocene	Pleistocene	5.333	0.0117	DuBar	1962	11119	Mollusca	7805	Gastropod
Late Pliocene		3.6	2.588	Mansfield	1930	17060	Mollusca	7805	Gastropod
Late Pliocene		3.6	2.588	Mansfield	1930	17060	Mollusca	7805	Gastropod
Late Pliocene		3.6	2.588	Mansfield	1930	17060	Mollusca	7805	Gastropod
Late Pliocene		3.6	2.588	Mansfield	1930	17060	Mollusca	7805	Gastropod
Late Pliocene		3.6	2.588	Mansfield	1930	17060	Mollusca	7805	Gastropod
Late Pliocene		3.6	2.588	Mansfield	1930	17060	Mollusca	7805	Gastropod
Late Pliocene		3.6	2.588	Mansfield	1930	17060	Mollusca	7805	Gastropod
Late Pliocene		3.6	2.588	Mansfield	1930	17060	Mollusca	7805	Gastropod
Late Pliocene		3.6	2.588	Mansfield	1932	17094	Mollusca	7805	Gastropod
Late Pliocene		3.6	2.588	Mansfield	1932	17094	Mollusca	7805	Gastropod
Middle Pleistocene		0.781	0.126	Portell et a	1992	17634	Mollusca	7805	Gastropod
Early Pleistocene		2.588	0.781	Schroeder	1954	25303	Mollusca	7805	Gastropod
Pliocene		5.333	2.588	Schroeder	1954	25303	Mollusca	7805	Gastropod
Middle Pleistocene		0.781	0.126	Petuch and	2007	26813	Mollusca	7805	Gastropod
Late Pliocene		3.6	2.588	Olsson	1967	19826	Mollusca	7805	Gastropod
Late Pliocene		3.6	2.588	Olsson	1967	19826	Mollusca	7805	Gastropod
Late Pliocene		3.6	2.588	Olsson	1967	19826	Mollusca	7805	Gastropod
Late Pliocene		3.6	2.588	Olsson and	1964	26836	Mollusca	7805	Gastropod
Late Plioce	Early Pleist	3.6	0.781	Petuch and	2007	26813	Mollusca	7805	Gastropod
Late Pliocene		3.6	2.588	Petuch and	2007	26813	Mollusca	7805	Gastropod
Early Pleistocene		2.588	0.781	Hendy et a	2008	19909	Mollusca	7805	Gastropod
Early Pleistocene		2.588	0.781	Hendy et a	2008	19909	Mollusca	7805	Gastropod
Early Pleistocene		2.588	0.781	Hendy et a	2008	19909	Mollusca	7805	Gastropod
Late Pleistocene		0.126	0.0117	Hendy et a	2008	19909	Mollusca	7805	Gastropod
Early Pleistocene		2.588	0.781	Fargo	1953	27010	Mollusca	7805	Gastropod

Early Pleistocene	2.588	0.781	Fargo	1953	27010	Mollusca	7805	Gastropod
Early Pleistocene	2.588	0.781	Fargo	1953	27010	Mollusca	7805	Gastropod
Early Pleistocene	2.588	0.781	Fargo	1953	27010	Mollusca	7805	Gastropod
Late Pliocene	3.6	2.588	Weisbord	1971	39743	Mollusca	7805	Gastropod
Pliocene	5.333	2.588	Petuch	1986	47440	Mollusca	7805	Gastropod
Pliocene	5.333	2.588	Petuch	1986	47440	Mollusca	7805	Gastropod
Pliocene	5.333	2.588	Petuch	1986	47440	Mollusca	7805	Gastropod
Pliocene	5.333	2.588	Petuch	1986	47440	Mollusca	7805	Gastropod
Late Pliocene	3.6	2.588	Ward and I	1987	16700	Mollusca	7805	Gastropod
Early Pleistocene	2.588	0.781	Ward and I	1987	16700	Mollusca	7805	Gastropod
Early Pleistocene	2.588	0.781	Gardner	1948	17048	Mollusca	7805	Gastropod
Pleistocene	2.588	0.0117	Gardner	1948	17048	Mollusca	7805	Gastropod
Late Pliocene	3.6	2.588	Gardner	1948	17048	Mollusca	7805	Gastropod
Late Pliocene	3.6	2.588	Gardner	1948	17048	Mollusca	7805	Gastropod
Late Pliocene	3.6	2.588	Gardner	1948	17048	Mollusca	7805	Gastropod
Late Pliocene	3.6	2.588	Gardner	1948	17048	Mollusca	7805	Gastropod
Early Pleistocene	2.588	0.781	Gardner	1948	17048	Mollusca	7805	Gastropod
Late Pliocene	3.6	2.588	Gardner	1948	17048	Mollusca	7805	Gastropod
Pleistocene	2.588	0.0117	Gardner	1948	17048	Mollusca	7805	Gastropod
Early Pleistocene	2.588	0.781	Gardner	1948	17048	Mollusca	7805	Gastropod
Late Pliocene	3.6	2.588	Gardner	1948	17048	Mollusca	7805	Gastropod
Gelasian	2.588	1.806	Bailey and	1986	23840	Mollusca	7805	Gastropod
Early Pleistocene	2.588	0.781	Hendy et a	2008	19909	Mollusca	7805	Gastropod
Late Pliocene	3.6	2.588	Ward	1993	26351	Mollusca	7805	Gastropod
Late Pliocene	3.6	2.588	Ward	1993	26351	Mollusca	7805	Gastropod
Late Pliocene	3.6	2.588	Ward	1993	26351	Mollusca	7805	Gastropod
Late Pliocene	3.6	2.588	Ward	1993	26351	Mollusca	7805	Gastropod
Late Pliocene	3.6	2.588	Ward	1993	26351	Mollusca	7805	Gastropod
Late Pliocene	3.6	2.588	Ward	1993	26351	Mollusca	7805	Gastropod
Late Pliocene	3.6	2.588	Hendy et a	2008	19909	Mollusca	7805	Gastropod
Late Pleistocene	0.126	0.0117	Petuch anc	2007	26813	Mollusca	7805	Gastropod
Late Pleistocene	0.126	0.0117	Petuch anc	2007	26813	Mollusca	7805	Gastropod
Late Pleistocene	0.126	0.0117	Kittle and I	2010	40884	Mollusca	7805	Gastropod
Late Pleistocene	0.126	0.0117	Kittle and I	2010	40884	Mollusca	7805	Gastropod
Late Pleistocene	0.126	0.0117	Kittle and I	2010	40884	Mollusca	7805	Gastropod
Late Pleistocene	0.126	0.0117	Kittle and I	2010	40884	Mollusca	7805	Gastropod
Late Pleistocene	0.126	0.0117	Richards	1962	18974	Mollusca	7805	Gastropod
Late Pleistocene	0.126	0.0117	Guilday et	1977	35027	Mollusca	7805	Gastropod
Late Pleistocene	0.126	0.0117	Guilday et	1977	35027	Mollusca	7805	Gastropod
Late Pleistocene	0.126	0.0117	Guilday et	1977	35027	Mollusca	7805	Gastropod
Late Pleistocene	0.126	0.0117	Guilday	1962	35381	Mollusca	7805	Gastropod
Late Pleistocene	0.126	0.0117	Sellards	1917	9011	Mollusca	7805	Gastropod
Late Pleistocene	0.126	0.0117	Sellards	1917	9011	Mollusca	7805	Gastropod
Early Pleistocene	2.588	0.781	DuBar	1962	11119	Mollusca	7805	Gastropod
Early Pleistocene	2.588	0.781	DuBar	1962	11119	Mollusca	7805	Gastropod
Early Pleistocene	2.588	0.781	DuBar	1962	11119	Mollusca	7805	Gastropod
Late Pliocene	3.6	2.588	Mansfield	1930	17060	Mollusca	7805	Gastropod

Late Pleistocene	0.126	0.0117	Portell et a	1992	17634	Mollusca	7805	Gastropod
Early Pleistocene	2.588	0.781	Portell et a	1992	17634	Mollusca	7805	Gastropod
Middle Pleistocene	0.781	0.126	Portell et a	1992	17634	Mollusca	7805	Gastropod
Middle Pleistocene	0.781	0.126	Hoerle	1970	19536	Mollusca	7805	Gastropod
Middle Pleistocene	0.781	0.126	Hoerle	1970	19536	Mollusca	7805	Gastropod
Middle Pleistocene	0.781	0.126	Hoerle	1970	19536	Mollusca	7805	Gastropod
Middle Pleistocene	0.781	0.126	Hoerle	1970	19536	Mollusca	7805	Gastropod
Middle Pleistocene	0.781	0.126	Hoerle	1970	19536	Mollusca	7805	Gastropod
Middle Pleistocene	0.781	0.126	Hoerle	1970	19536	Mollusca	7805	Gastropod
Middle Pleistocene	0.781	0.126	Hoerle	1970	19536	Mollusca	7805	Gastropod
Middle Pleistocene	0.781	0.126	Hoerle	1970	19536	Mollusca	7805	Gastropod
Middle Pleistocene	0.781	0.126	Hoerle	1970	19536	Mollusca	7805	Gastropod
Early Pleistocene	2.588	0.781	Schroeder	1954	25303	Mollusca	7805	Gastropod
Early Pleistocene	2.588	0.781	Schroeder	1954	25303	Mollusca	7805	Gastropod
Late Pliocene	3.6	2.588	Petuch and	2007	26813	Mollusca	7805	Gastropod
Pleistocene	2.588	0.0117	Hendy et a	2008	19909	Mollusca	7805	Gastropod
Early Pleistocene	2.588	0.781	Olsson and	1953	25463	Mollusca	7805	Gastropod
Early Pleistocene	2.588	0.781	Olsson and	1953	25463	Mollusca	7805	Gastropod
Early Pleistocene	2.588	0.781	Olsson and	1953	25463	Mollusca	7805	Gastropod
Early Pleistocene	2.588	0.781	Olsson and	1953	25463	Mollusca	7805	Gastropod
Early Pleistocene	2.588	0.781	Olsson and	1953	25463	Mollusca	7805	Gastropod
Early Pleistocene	2.588	0.781	Olsson and	1953	25463	Mollusca	7805	Gastropod
Early Pleistocene	2.588	0.781	Olsson and	1953	25463	Mollusca	7805	Gastropod
Early Pleistocene	2.588	0.781	Olsson and	1953	25463	Mollusca	7805	Gastropod
Late Pleistocene	0.126	0.0117	Kittle and I	2010	40884	Mollusca	7805	Gastropod
Late Pleistocene	0.126	0.0117	Hulbert an	1998	1962	Mollusca	7805	Gastropod
Late Pleistocene	0.126	0.0117	Hulbert an	1998	1962	Mollusca	7805	Gastropod
Late Pleistocene	0.126	0.0117	DuBar	1971	17556	Mollusca	7805	Gastropod
Late Pleistocene	0.126	0.0117	DuBar	1971	17556	Mollusca	7805	Gastropod
Zanclean	5.333	3.6	Campbell a	1995	17558	Mollusca	7805	Gastropod
Early Pleistocene	2.588	0.781	DuBar	1971	17556	Mollusca	7805	Gastropod
Early Pleistocene	2.588	0.781	DuBar	1971	17556	Mollusca	7805	Gastropod
Early Pleistocene	2.588	0.781	DuBar	1971	17556	Mollusca	7805	Gastropod
Late Pleistocene	0.126	0.0117	Richards	1962	18974	Mollusca	7805	Gastropod
Late Pleistocene	0.126	0.0117	Richards	1962	18974	Mollusca	7805	Gastropod
Late Pleistocene	0.126	0.0117	Richards	1962	18974	Mollusca	7805	Gastropod
Late Pleistocene	0.126	0.0117	Richards	1962	18974	Mollusca	7805	Gastropod
Late Pleistocene	0.126	0.0117	Richards	1962	18974	Mollusca	7805	Gastropod
Late Pleistocene	0.126	0.0117	Richards	1962	18974	Mollusca	7805	Gastropod
Late Pleistocene	0.126	0.0117	Richards	1962	18974	Mollusca	7805	Gastropod
Late Pleistocene	0.126	0.0117	Richards	1962	18974	Mollusca	7805	Gastropod
Late Pleistocene	0.126	0.0117	Hendy et a	2008	19909	Mollusca	7805	Gastropod
Late Pliocene	3.6	2.588	Cooke	1936	31145	Mollusca	7805	Gastropod
Pleistocene	2.588	0.0117	Holmes	1860	64712	Mollusca	7805	Gastropod
Late Pliocene	3.6	2.588	Ward and I	1987	16700	Mollusca	7805	Gastropod
Late Pliocene	3.6	2.588	Ward and I	1987	16700	Mollusca	7805	Gastropod
Late Pliocene	3.6	2.588	Ward and I	1987	16700	Mollusca	7805	Gastropod

Early Pleistocene	2.588	0.781	Ward and I	1987	16700	Mollusca	7805	Gastropod
Late Pliocene	3.6	2.588	Gardner	1948	17048	Mollusca	7805	Gastropod
Late Pliocene	3.6	2.588	Gardner	1948	17048	Mollusca	7805	Gastropod
Late Pliocene	3.6	2.588	Gardner	1948	17048	Mollusca	7805	Gastropod
Late Pliocene	3.6	2.588	Gardner	1948	17048	Mollusca	7805	Gastropod
Early Pleistocene	2.588	0.781	Gardner	1948	17048	Mollusca	7805	Gastropod
Late Pliocene	3.6	2.588	Gardner	1948	17048	Mollusca	7805	Gastropod
Late Pliocene	3.6	2.588	Gardner	1948	17048	Mollusca	7805	Gastropod
Early Pleistocene	2.588	0.781	Miller	1990	17561	Mollusca	7805	Gastropod
Early Pleistocene	2.588	0.781	Miller	1990	17561	Mollusca	7805	Gastropod
Early Pleistocene	2.588	0.781	Miller	1990	17561	Mollusca	7805	Gastropod
Early Pleistocene	2.588	0.781	Miller	1990	17561	Mollusca	7805	Gastropod
Late Pleistocene	0.126	0.0117	Richards	1962	18974	Mollusca	7805	Gastropod
Late Pleistocene	0.126	0.0117	Richards	1962	18974	Mollusca	7805	Gastropod
Late Pleistocene	0.126	0.0117	Richards	1962	18974	Mollusca	7805	Gastropod
Late Pleistocene	0.126	0.0117	Richards	1962	18974	Mollusca	7805	Gastropod
Gelasian	2.588	1.806	Bailey and	1986	23840	Mollusca	7805	Gastropod
Early Pleistocene	2.588	0.781	Hendy et a	2008	19909	Mollusca	7805	Gastropod
Late Pleistocene	0.126	0.0117	Spencer ar	1987	15525	Mollusca	7805	Gastropod
Late Pleistocene	0.126	0.0117	Spencer ar	1987	15525	Mollusca	7805	Gastropod
Late Pleistocene	0.126	0.0117	Spencer ar	1987	15525	Mollusca	7805	Gastropod
Late Pleistocene	0.126	0.0117	Spencer ar	1987	15525	Mollusca	7805	Gastropod
Late Pleistocene	0.126	0.0117	Spencer ar	1987	15525	Mollusca	7805	Gastropod
Late Pleistocene	0.126	0.0117	Spencer ar	1987	15525	Mollusca	7805	Gastropod
Late Pleistocene	0.126	0.0117	Spencer ar	1987	15525	Mollusca	7805	Gastropod
Late Pleistocene	0.126	0.0117	Spencer ar	1987	15525	Mollusca	7805	Gastropod
Late Pleistocene	0.126	0.0117	Spencer ar	1987	15525	Mollusca	7805	Gastropod
Late Pleistocene	0.126	0.0117	Spencer ar	1987	15525	Mollusca	7805	Gastropod
Late Pleistocene	0.126	0.0117	Spencer ar	1987	15525	Mollusca	7805	Gastropod
Late Pliocene	3.6	2.588	Ward	1993	26351	Mollusca	7805	Gastropod
Late Pliocene	3.6	2.588	Ward	1993	26351	Mollusca	7805	Gastropod
Late Pliocene	3.6	2.588	Ward	1993	26351	Mollusca	7805	Gastropod
Late Pliocene	3.6	2.588	Ward	1993	26351	Mollusca	7805	Gastropod
Late Pliocene	3.6	2.588	Ward	1993	26351	Mollusca	7805	Gastropod
Late Pliocene	3.6	2.588	Ward	1993	26351	Mollusca	7805	Gastropod
Late Pliocene	3.6	2.588	Ward	1993	26351	Mollusca	7805	Gastropod
Late Pliocene	3.6	2.588	Ward	1993	26351	Mollusca	7805	Gastropod
Early Pleistocene	2.588	0.781	DuBar	1962	11119	Mollusca	7805	Gastropod
Early Pleistocene	2.588	0.781	DuBar	1962	11119	Mollusca	7805	Gastropod
Calabrian	1.806	0.781	DuBar	1962	11119	Mollusca	7805	Gastropod
Calabrian	1.806	0.781	DuBar	1962	11119	Mollusca	7805	Gastropod
Late Pliocene	3.6	2.588	Mansfield	1930	17060	Mollusca	7805	Gastropod
Late Pliocene	3.6	2.588	Mansfield	1930	17060	Mollusca	7805	Gastropod
Late Pliocene	3.6	2.588	Mansfield	1930	17060	Mollusca	7805	Gastropod
Late Pliocene	3.6	2.588	Mansfield	1930	17060	Mollusca	7805	Gastropod
Late Pliocene	3.6	2.588	Mansfield	1930	17060	Mollusca	7805	Gastropod
Late Pliocene	3.6	2.588	Mansfield	1930	17060	Mollusca	7805	Gastropod

Late Pliocene	3.6	2.588	Mansfield	1930	17060	Mollusca	7805	Gastropod
Late Pleistocene	0.126	0.0117	Portell et a	1992	17634	Mollusca	7805	Gastropod
Early Pleistocene	2.588	0.781	Portell et a	1992	17634	Mollusca	7805	Gastropod
Early Pleistocene	2.588	0.781	Portell et a	1992	17634	Mollusca	7805	Gastropod
Middle Pleistocene	0.781	0.126	Portell et a	1992	17634	Mollusca	7805	Gastropod
Middle Pleistocene	0.781	0.126	Hoerle	1970	19536	Mollusca	7805	Gastropod
Middle Pleistocene	0.781	0.126	Hoerle	1970	19536	Mollusca	7805	Gastropod
Middle Pleistocene	0.781	0.126	Hendy et a	2008	19909	Mollusca	7805	Gastropod
Middle Pleistocene	0.781	0.126	Hoerle	1970	19536	Mollusca	7805	Gastropod
Early Pleistocene	2.588	0.781	Olsson anc	1953	25463	Mollusca	7805	Gastropod
Early Pleistocene	2.588	0.781	Olsson anc	1953	25463	Mollusca	7805	Gastropod
Early Pleistocene	2.588	0.781	Olsson anc	1953	25463	Mollusca	7805	Gastropod
Early Pleistocene	2.588	0.781	Olsson anc	1953	25463	Mollusca	7805	Gastropod
Early Pleistocene	2.588	0.781	Olsson anc	1953	25463	Mollusca	7805	Gastropod
Early Pleistocene	2.588	0.781	Olsson anc	1953	25463	Mollusca	7805	Gastropod
Early Pleistocene	2.588	0.781	Olsson anc	1953	25463	Mollusca	7805	Gastropod
Early Pleistocene	2.588	0.781	Olsson anc	1953	25463	Mollusca	7805	Gastropod
Early Pleistocene	2.588	0.781	Olsson anc	1953	25463	Mollusca	7805	Gastropod
Early Pleistocene	2.588	0.781	Olsson anc	1953	25463	Mollusca	7805	Gastropod
Early Pleistocene	2.588	0.781	Olsson anc	1953	25463	Mollusca	7805	Gastropod
Early Pleistocene	2.588	0.781	Olsson anc	1953	25463	Mollusca	7805	Gastropod
Late Pleistocene	0.126	0.0117	DuBar	1971	17556	Mollusca	7805	Gastropod
Late Pliocene	3.6	2.588	Campbell a	1995	17558	Mollusca	7805	Gastropod
Late Pleistocene	0.126	0.0117	Richards	1962	18974	Mollusca	7805	Gastropod
Late Pleistocene	0.126	0.0117	Richards	1962	18974	Mollusca	7805	Gastropod
Late Pliocene	3.6	2.588	Ward and I	1987	16700	Mollusca	7805	Gastropod
Early Pleistocene	2.588	0.781	Ward and I	1987	16700	Mollusca	7805	Gastropod
Early Pleistocene	2.588	0.781	Ward and I	1987	16700	Mollusca	7805	Gastropod
Early Pleistocene	2.588	0.781	Ward and I	1987	16700	Mollusca	7805	Gastropod
Early Pleistocene	2.588	0.781	Ward and I	1987	16700	Mollusca	7805	Gastropod
Late Pliocene	3.6	2.588	Gardner	1948	17048	Mollusca	7805	Gastropod
Early Pleistocene	2.588	0.781	Gardner	1948	17048	Mollusca	7805	Gastropod
Early Pleistocene	2.588	0.781	Gardner	1948	17048	Mollusca	7805	Gastropod
Late Pliocene	3.6	2.588	Gardner	1948	17048	Mollusca	7805	Gastropod
Late Pliocene	3.6	2.588	Gardner	1948	17048	Mollusca	7805	Gastropod
Early Plioc‹ Late Plioce	5.333	2.588	Gardner	1948	17048	Mollusca	7805	Gastropod
Late Pliocene	3.6	2.588	Gardner	1948	17048	Mollusca	7805	Gastropod
Late Pliocene	3.6	2.588	Gardner	1948	17048	Mollusca	7805	Gastropod
Early Pleistocene	2.588	0.781	Gardner	1948	17048	Mollusca	7805	Gastropod
Early Pleistocene	2.588	0.781	Miller	1990	17561	Mollusca	7805	Gastropod
Early Pleistocene	2.588	0.781	Miller	1990	17561	Mollusca	7805	Gastropod
Early Pleistocene	2.588	0.781	Hendy et a	2008	19909	Mollusca	7805	Gastropod
Early Pleistocene	2.588	0.781	Miller	1990	17561	Mollusca	7805	Gastropod
Early Pleistocene	2.588	0.781	Hendy et a	2008	19909	Mollusca	7805	Gastropod
Early Pleistocene	2.588	0.781	Miller	1990	17561	Mollusca	7805	Gastropod
Late Pleistocene	0.126	0.0117	Hendy et a	2008	19909	Mollusca	7805	Gastropod
Late Pleistocene	0.126	0.0117	Hendy et a	2008	19909	Mollusca	7805	Gastropod

Gelasian		2.588	1.806	Bailey and	1986	23840	Mollusca	7805	Gastropod
Early Pleistocene		2.588	0.781	Hendy et a	2008	19909	Mollusca	7805	Gastropod
Early Pleistocene		2.588	0.781	Miller and	1988	72890	Mollusca	7805	Gastropod
Late Pliocene		3.6	2.588	Edwards et	2005	17631	Mollusca	7805	Gastropod
Late Pliocene		3.6	2.588	Edwards et	2005	17631	Mollusca	7805	Gastropod
Late Pliocene		3.6	2.588	Ward	1993	26351	Mollusca	7805	Gastropod
Late Pliocene		3.6	2.588	Ward	1993	26351	Mollusca	7805	Gastropod
Late Pliocene		3.6	2.588	Ward	1993	26351	Mollusca	7805	Gastropod
Late Pliocene		3.6	2.588	Ward	1993	26351	Mollusca	7805	Gastropod
Late Pliocene		3.6	2.588	Ward	1993	26351	Mollusca	7805	Gastropod
Late Pliocene		3.6	2.588	Ward	1993	26351	Mollusca	7805	Gastropod
Late Pliocene		3.6	2.588	Ward	1993	26351	Mollusca	7805	Gastropod
Late Pliocene		3.6	2.588	Ward	1993	26351	Mollusca	7805	Gastropod
Late Pliocene		3.6	2.588	Ward	1993	26351	Mollusca	7805	Gastropod
Late Pleistocene		0.126	0.0117	Sellards	1917	9011	Mollusca	7805	Gastropod
Early Pleistocene		2.588	0.781	Mansfield	1932	11083	Mollusca	7805	Gastropod
Early Pleistocene		2.588	0.781	Mansfield	1932	11083	Mollusca	7805	Gastropod
Early Pleistocene		2.588	0.781	DuBar	1962	11119	Mollusca	7805	Gastropod
Early Pleistocene		2.588	0.781	DuBar	1962	11119	Mollusca	7805	Gastropod
Early Pleistocene		2.588	0.781	DuBar	1962	11119	Mollusca	7805	Gastropod
Early Pleistocene		2.588	0.781	DuBar	1962	11119	Mollusca	7805	Gastropod
Early Pleistocene		2.588	0.781	DuBar	1962	11119	Mollusca	7805	Gastropod
Calabrian		1.806	0.781	DuBar	1962	11119	Mollusca	7805	Gastropod
Calabrian		1.806	0.781	DuBar	1962	11119	Mollusca	7805	Gastropod
Early Pleistocene		2.588	0.781	DuBar	1962	11119	Mollusca	7805	Gastropod
Early Pleistocene		2.588	0.781	DuBar	1962	11119	Mollusca	7805	Gastropod
Late Pleistocene		0.126	0.0117	DuBar	1962	11119	Mollusca	7805	Gastropod
Late Pleistocene		0.126	0.0117	DuBar	1962	11119	Mollusca	7805	Gastropod
Pliocene	Pleistocene	5.333	0.0117	DuBar	1962	11119	Mollusca	7805	Gastropod
Pliocene	Pleistocene	5.333	0.0117	DuBar	1962	11119	Mollusca	7805	Gastropod
Pliocene	Pleistocene	5.333	0.0117	DuBar	1962	11119	Mollusca	7805	Gastropod
Pliocene	Pleistocene	5.333	0.0117	DuBar	1962	11119	Mollusca	7805	Gastropod
Pliocene	Pleistocene	5.333	0.0117	DuBar	1962	11119	Mollusca	7805	Gastropod
Late Pliocene		3.6	2.588	Mansfield	1930	17060	Mollusca	7805	Gastropod
Late Pliocene		3.6	2.588	Mansfield	1930	17060	Mollusca	7805	Gastropod
Late Pliocene		3.6	2.588	Mansfield	1930	17060	Mollusca	7805	Gastropod
Late Pliocene		3.6	2.588	Mansfield	1932	17094	Mollusca	7805	Gastropod
Late Pliocene		3.6	2.588	Mansfield	1932	17094	Mollusca	7805	Gastropod
Late Pliocene		3.6	2.588	Mansfield	1932	17094	Mollusca	7805	Gastropod
Late Pliocene		3.6	2.588	Mansfield	1932	17094	Mollusca	7805	Gastropod
Early Pleistocene		2.588	0.781	Portell et a	1992	17634	Mollusca	7805	Gastropod
Early Pleistocene		2.588	0.781	Portell et a	1992	17634	Mollusca	7805	Gastropod
Middle Pleistocene		0.781	0.126	Portell et a	1992	17634	Mollusca	7805	Gastropod
Middle Pleistocene		0.781	0.126	Portell et a	1992	17634	Mollusca	7805	Gastropod
Middle Pleistocene		0.781	0.126	McGinty	1970	19114	Mollusca	7805	Gastropod
Middle Pleistocene		0.781	0.126	McGinty	1970	19114	Mollusca	7805	Gastropod
Middle Pleistocene		0.781	0.126	McGinty	1970	19114	Mollusca	7805	Gastropod

Middle Pleistocene	0.781	0.126	McGinty	1970	19114	Mollusca	7805	Gastropod
Late Pleistocene	0.126	0.0117	Hendy et a	2008	19909	Mollusca	7805	Gastropod
Early Pleistocene	2.588	0.781	Olsson anc	1953	25463	Mollusca	7805	Gastropod
Early Pleistocene	2.588	0.781	Olsson anc	1953	25463	Mollusca	7805	Gastropod
Early Pleistocene	2.588	0.781	Olsson anc	1953	25463	Mollusca	7805	Gastropod
Early Pleistocene	2.588	0.781	Olsson anc	1953	25463	Mollusca	7805	Gastropod
Early Pleistocene	2.588	0.781	Olsson anc	1953	25463	Mollusca	7805	Gastropod
Early Pleistocene	2.588	0.781	Olsson anc	1953	25463	Mollusca	7805	Gastropod
Early Pleistocene	2.588	0.781	Olsson anc	1953	25463	Mollusca	7805	Gastropod
Late Pleistocene	0.126	0.0117	Petuch anc	2007	26813	Mollusca	7805	Gastropod
Late Pleistocene	0.126	0.0117	Petuch anc	2007	26813	Mollusca	7805	Gastropod
Late Pleistocene	0.126	0.0117	Petuch anc	2007	26813	Mollusca	7805	Gastropod
Middle Pleistocene	0.781	0.126	Petuch anc	2007	26813	Mollusca	7805	Gastropod
Middle Pleistocene	0.781	0.126	Petuch anc	2007	26813	Mollusca	7805	Gastropod
Early Pleistocene	2.588	0.781	Petuch anc	2007	26813	Mollusca	7805	Gastropod
Early Pleistocene	2.588	0.781	Petuch anc	2007	26813	Mollusca	7805	Gastropod
Middle Pleistocene	0.781	0.126	Petuch anc	2007	26813	Mollusca	7805	Gastropod
Middle Pleistocene	0.781	0.126	Petuch anc	2007	26813	Mollusca	7805	Gastropod
Middle Pleistocene	0.781	0.126	Petuch anc	2007	26813	Mollusca	7805	Gastropod
Middle Pleistocene	0.781	0.126	Petuch anc	2007	26813	Mollusca	7805	Gastropod
Middle Pleistocene	0.781	0.126	Petuch anc	2007	26813	Mollusca	7805	Gastropod
Early Pleistocene	2.588	0.781	Olsson anc	1964	26836	Mollusca	7805	Gastropod
Early Pleistocene	2.588	0.781	Olsson anc	1964	26836	Mollusca	7805	Gastropod
Middle Pleistocene	0.781	0.126	Olsson anc	1964	26836	Mollusca	7805	Gastropod
Middle Pleistocene	0.781	0.126	Olsson anc	1964	26836	Mollusca	7805	Gastropod
Early Pleistocene	2.588	0.781	Petuch anc	2007	26813	Mollusca	7805	Gastropod
Late Plioce Early Pleist	3.6	0.781	Petuch anc	2007	26813	Mollusca	7805	Gastropod
Late Plioce Early Pleist	3.6	0.781	Petuch anc	2007	26813	Mollusca	7805	Gastropod
Late Plioce Early Pleist	3.6	0.781	Petuch anc	2007	26813	Mollusca	7805	Gastropod
Late Plioce Early Pleist	3.6	0.781	Petuch anc	2007	26813	Mollusca	7805	Gastropod
Late Plioce Early Pleist	3.6	0.781	Petuch anc	2007	26813	Mollusca	7805	Gastropod
Late Plioce Early Pleist	3.6	0.781	Petuch anc	2007	26813	Mollusca	7805	Gastropod
Early Pleistocene	2.588	0.781	Petuch anc	2007	26813	Mollusca	7805	Gastropod
Early Pleistocene	2.588	0.781	Petuch anc	2007	26813	Mollusca	7805	Gastropod
Early Pleistocene	2.588	0.781	Petuch anc	2007	26813	Mollusca	7805	Gastropod
Late Plioce Pleistocen	3.6	0.0117	Petuch anc	2007	26813	Mollusca	7805	Gastropod
Late Plioce Pleistocen	3.6	0.0117	Petuch anc	2007	26813	Mollusca	7805	Gastropod
Late Plioce Early Pleist	3.6	0.781	Petuch anc	2007	26813	Mollusca	7805	Gastropod
Late Pliocene	3.6	2.588	Petuch anc	2007	26813	Mollusca	7805	Gastropod
Late Pliocene	3.6	2.588	Petuch anc	2007	26813	Mollusca	7805	Gastropod
Late Pliocene	3.6	2.588	Petuch anc	2007	26813	Mollusca	7805	Gastropod
Late Pliocene	3.6	2.588	Petuch anc	2007	26813	Mollusca	7805	Gastropod
Late Pliocene	3.6	2.588	Petuch anc	2007	26813	Mollusca	7805	Gastropod
Late Pliocene	3.6	2.588	Petuch anc	2007	26813	Mollusca	7805	Gastropod
Late Pliocene	3.6	2.588	Petuch anc	2007	26813	Mollusca	7805	Gastropod
Late Pliocene	3.6	2.588	Petuch anc	2007	26813	Mollusca	7805	Gastropod
Early Pleistocene	2.588	0.781	Hendy et a	2008	19909	Mollusca	7805	Gastropod

Early Pleistocene	2.588	0.781	Hendy et a	2008	19909	Mollusca	7805	Gastropod
Early Pleistocene	2.588	0.781	Hendy et a	2008	19909	Mollusca	7805	Gastropod
Late Pleistocene	0.126	0.0117	Hendy et a	2008	19909	Mollusca	7805	Gastropod
Late Pliocene	3.6	2.588	Petuch and	2007	26813	Mollusca	7805	Gastropod
Late Pliocene	3.6	2.588	Petuch and	2007	26813	Mollusca	7805	Gastropod
Late Pliocene	3.6	2.588	Petuch and	2007	26813	Mollusca	7805	Gastropod
Late Pliocene	3.6	2.588	Petuch and	2007	26813	Mollusca	7805	Gastropod
Late Pliocene	3.6	2.588	Petuch and	2007	26813	Mollusca	7805	Gastropod
Late Pliocene	3.6	2.588	Petuch and	2007	26813	Mollusca	7805	Gastropod
Late Pliocene	3.6	2.588	Petuch and	2007	26813	Mollusca	7805	Gastropod
Late Pliocene	3.6	2.588	Petuch and	2007	26813	Mollusca	7805	Gastropod
Late Pliocene	3.6	2.588	Petuch and	2007	26813	Mollusca	7805	Gastropod
Late Pliocene	3.6	2.588	Petuch and	2007	26813	Mollusca	7805	Gastropod
Late Pliocene	3.6	2.588	Petuch and	2007	26813	Mollusca	7805	Gastropod
Late Pliocene	3.6	2.588	Petuch and	2007	26813	Mollusca	7805	Gastropod
Late Pliocene	3.6	2.588	Petuch and	2007	26813	Mollusca	7805	Gastropod
Late Pleistocene	0.126	0.0117	Hendy et a	2008	19909	Mollusca	7805	Gastropod
Early Pleistocene	2.588	0.781	Hendy et a	2008	19909	Mollusca	7805	Gastropod
Early Pleistocene	2.588	0.781	Hendy et a	2008	19909	Mollusca	7805	Gastropod
Early Pleistocene	2.588	0.781	Hendy et a	2008	19909	Mollusca	7805	Gastropod
Early Pleistocene	2.588	0.781	Hendy et a	2008	19909	Mollusca	7805	Gastropod
Late Pliocene	3.6	2.588	Hendy et a	2008	19909	Mollusca	7805	Gastropod
Late Pliocene	3.6	2.588	Hendy et a	2008	19909	Mollusca	7805	Gastropod
Middle Pleistocene	0.781	0.126	Hendy et a	2008	19909	Mollusca	7805	Gastropod
Middle Pleistocene	0.781	0.126	Hendy et a	2008	19909	Mollusca	7805	Gastropod
Middle Pleistocene	0.781	0.126	Hendy et a	2008	19909	Mollusca	7805	Gastropod
Late Pleistocene	0.126	0.0117	Hendy et a	2008	19909	Mollusca	7805	Gastropod
Early Pleistocene	2.588	0.781	Hendy et a	2008	19909	Mollusca	7805	Gastropod
Late Pliocene	3.6	2.588	Petuch	1988	31112	Mollusca	7805	Gastropod
Late Pliocene	3.6	2.588	Petuch	1988	31112	Mollusca	7805	Gastropod
Early Pleistocene	2.588	0.781	Petuch	1988	31112	Mollusca	7805	Gastropod
Early Pleistocene	2.588	0.781	Petuch	1988	31112	Mollusca	7805	Gastropod
Middle Pleistocene	0.781	0.126	Petuch	1988	31112	Mollusca	7805	Gastropod
Middle Pleistocene	0.781	0.126	Petuch	1988	31112	Mollusca	7805	Gastropod
Late Pleistocene	0.126	0.0117	Kittle and I	2010	40884	Mollusca	7805	Gastropod
Late Pleistocene	0.126	0.0117	Kittle and I	2010	40884	Mollusca	7805	Gastropod
Late Pleistocene	0.126	0.0117	Kittle and I	2010	40884	Mollusca	7805	Gastropod
Late Pleistocene	0.126	0.0117	Kittle and I	2010	40884	Mollusca	7805	Gastropod
Pliocene	5.333	2.588	Petuch	1986	47440	Mollusca	7805	Gastropod
Late Pliocene	3.6	2.588	Campbell a	1995	17558	Mollusca	7805	Gastropod
Zanclean	5.333	3.6	Campbell a	1995	17558	Mollusca	7805	Gastropod
Zanclean	5.333	3.6	Campbell a	1995	17558	Mollusca	7805	Gastropod
Zanclean	5.333	3.6	Campbell a	1995	17558	Mollusca	7805	Gastropod
Early Pliocene	5.333	3.6	Campbell a	1995	17558	Mollusca	7805	Gastropod
Early Pliocene	5.333	3.6	Campbell a	1995	17558	Mollusca	7805	Gastropod
Late Pleistocene	0.126	0.0117	Richards	1962	18974	Mollusca	7805	Gastropod
Late Pleistocene	0.126	0.0117	Richards	1962	18974	Mollusca	7805	Gastropod

Late Pleistocene	0.126	0.0117	Richards	1962	18974	Mollusca	7805	Gastropod
Late Pleistocene	0.126	0.0117	Richards	1962	18974	Mollusca	7805	Gastropod
Late Pleistocene	0.126	0.0117	Richards	1962	18974	Mollusca	7805	Gastropod
Late Pleistocene	0.126	0.0117	Hendy et a	2008	19909	Mollusca	7805	Gastropod
Late Pleistocene	0.126	0.0117	Hendy et a	2008	19909	Mollusca	7805	Gastropod
Late Pliocene	3.6	2.588	Gardner	1948	17048	Mollusca	7805	Gastropod
Early Pleistocene	2.588	0.781	Gardner	1948	17048	Mollusca	7805	Gastropod
Late Pliocene	3.6	2.588	Gardner	1948	17048	Mollusca	7805	Gastropod
Late Pleistocene	0.126	0.0117	Hendy et a	2008	19909	Mollusca	7805	Gastropod
Late Pliocene	3.6	2.588	Hendy et a	2008	19909	Mollusca	7805	Gastropod
Early Pleistocene	2.588	0.781	Hendy et a	2008	19909	Mollusca	7805	Gastropod
Early Pleistocene	2.588	0.781	Hendy et a	2008	19909	Mollusca	7805	Gastropod
Early Pleistocene	2.588	0.781	Hendy et a	2008	19909	Mollusca	7805	Gastropod
Early Pleistocene	2.588	0.781	Hendy et a	2008	19909	Mollusca	7805	Gastropod
Late Pliocene	3.6	2.588	Petuch	1988	31112	Mollusca	7805	Gastropod
Pliocene	5.333	2.588	Ward	2007	48009	Mollusca	7805	Gastropod
Late Pliocene	3.6	2.588	Ward	1993	26351	Mollusca	7805	Gastropod
Late Pliocene	3.6	2.588	Ward	1993	26351	Mollusca	7805	Gastropod
Late Pliocene	3.6	2.588	Ward	1993	26351	Mollusca	7805	Gastropod
Late Pliocene	3.6	2.588	Ward	1993	26351	Mollusca	7805	Gastropod
Late Pliocene	3.6	2.588	Ward	1993	26351	Mollusca	7805	Gastropod
Late Pliocene	3.6	2.588	Ward	1993	26351	Mollusca	7805	Gastropod
Late Pliocene	3.6	2.588	Ward	1993	26351	Mollusca	7805	Gastropod
Late Pliocene	3.6	2.588	Ward	1993	26351	Mollusca	7805	Gastropod
Late Pliocene	3.6	2.588	Ward	1993	26351	Mollusca	7805	Gastropod
Late Pliocene	3.6	2.588	Ward	1993	26351	Mollusca	7805	Gastropod
Late Pliocene	3.6	2.588	Ward	1993	26351	Mollusca	7805	Gastropod
Late Pliocene	3.6	2.588	Ward	1993	26351	Mollusca	7805	Gastropod
Late Pliocene	3.6	2.588	Hendy et a	2008	19909	Mollusca	7805	Gastropod
Late Pliocene	3.6	2.588	Hendy et a	2008	19909	Mollusca	7805	Gastropod
Late Pliocene	3.6	2.588	Hendy et a	2008	19909	Mollusca	7805	Gastropod
Late Pliocene	3.6	2.588	Hendy et a	2008	19909	Mollusca	7805	Gastropod
Calabrian	1.806	0.781	DuBar	1962	11119	Mollusca	7805	Gastropod
Late Pliocene	3.6	2.588	Mansfield	1930	17060	Mollusca	7805	Gastropod
Early Pleistocene	2.588	0.781	Portell et a	1992	17634	Mollusca	7805	Gastropod
Late Pliocene	3.6	2.588	Petuch anc	2007	26813	Mollusca	7805	Gastropod
Early Pleistocene	2.588	0.781	Olsson anc	1953	25463	Mollusca	7805	Gastropod
Early Pleistocene	2.588	0.781	Olsson anc	1953	25463	Mollusca	7805	Gastropod
Early Pleistocene	2.588	0.781	Olsson anc	1953	25463	Mollusca	7805	Gastropod
Early Pleistocene	2.588	0.781	Olsson anc	1953	25463	Mollusca	7805	Gastropod
Late Pliocene	3.6	2.588	Petuch anc	2007	26813	Mollusca	7805	Gastropod
Middle Pleistocene	0.781	0.126	Hendy et a	2008	19909	Mollusca	7805	Gastropod
Late Pleistocene	0.126	0.0117	Kittle and I	2010	40884	Mollusca	7805	Gastropod
Zanclean	5.333	3.6	Campbell a	1995	17558	Mollusca	7805	Gastropod
Early Pleistocene	2.588	0.781	Gardner	1948	17048	Mollusca	7805	Gastropod
Early Pleistocene	2.588	0.781	Hendy et a	2008	19909	Mollusca	7805	Gastropod
Early Pleistocene	2.588	0.781	Hendy et a	2008	19909	Mollusca	7805	Gastropod

Late Pleistocene	0.126	0.0117	Sellards	1917	9011	Mollusca	7805	Gastropod
Early Pleistocene	2.588	0.781	DuBar	1962	11119	Mollusca	7805	Gastropod
Early Pleistocene	2.588	0.781	DuBar	1962	11119	Mollusca	7805	Gastropod
Calabrian	1.806	0.781	DuBar	1962	11119	Mollusca	7805	Gastropod
Calabrian	1.806	0.781	DuBar	1962	11119	Mollusca	7805	Gastropod
Early Pleistocene	2.588	0.781	DuBar	1962	11119	Mollusca	7805	Gastropod
Late Pliocene	3.6	2.588	Mansfield	1930	17060	Mollusca	7805	Gastropod
Middle Pleistocene	0.781	0.126	Portell et a	1992	17634	Mollusca	7805	Gastropod
Middle Pleistocene	0.781	0.126	Portell et a	1992	17634	Mollusca	7805	Gastropod
Middle Pleistocene	0.781	0.126	Portell et a	1992	17634	Mollusca	7805	Gastropod
Middle Pleistocene	0.781	0.126	Portell et a	1992	17634	Mollusca	7805	Gastropod
Middle Pleistocene	0.781	0.126	Portell et a	1992	17634	Mollusca	7805	Gastropod
Late Pleistocene	0.126	0.0117	Portell et a	1992	17634	Mollusca	7805	Gastropod
Early Pleistocene	2.588	0.781	Portell et a	1992	17634	Mollusca	7805	Gastropod
Middle Pleistocene	0.781	0.126	Hoerle	1970	19536	Mollusca	7805	Gastropod
Middle Pleistocene	0.781	0.126	Hoerle	1970	19536	Mollusca	7805	Gastropod
Middle Pleistocene	0.781	0.126	Hoerle	1970	19536	Mollusca	7805	Gastropod
Middle Pleistocene	0.781	0.126	Hoerle	1970	19536	Mollusca	7805	Gastropod
Middle Pleistocene	0.781	0.126	Hoerle	1970	19536	Mollusca	7805	Gastropod
Middle Pleistocene	0.781	0.126	Hoerle	1970	19536	Mollusca	7805	Gastropod
Middle Pleistocene	0.781	0.126	Hoerle	1970	19536	Mollusca	7805	Gastropod
Early Pleistocene	2.588	0.781	Hendy et a	2008	19909	Mollusca	7805	Gastropod
Late Pliocene	3.6	2.588	Petuch anc	2007	26813	Mollusca	7805	Gastropod
Late Pliocene	3.6	2.588	Hendy et a	2008	19909	Mollusca	7805	Gastropod
Early Pleistocene	2.588	0.781	Hendy et a	2008	19909	Mollusca	7805	Gastropod
Late Pleistocene	0.126	0.0117	Hendy et a	2008	19909	Mollusca	7805	Gastropod
Early Pleistocene	2.588	0.781	Olsson anc	1953	25463	Mollusca	7805	Gastropod
Early Pleistocene	2.588	0.781	Olsson anc	1953	25463	Mollusca	7805	Gastropod
Early Pleistocene	2.588	0.781	Olsson anc	1953	25463	Mollusca	7805	Gastropod
Early Pleistocene	2.588	0.781	Olsson anc	1953	25463	Mollusca	7805	Gastropod
Early Pleistocene	2.588	0.781	Olsson anc	1953	25463	Mollusca	7805	Gastropod
Early Pleistocene	2.588	0.781	Olsson anc	1953	25463	Mollusca	7805	Gastropod
Early Pleistocene	2.588	0.781	Olsson anc	1953	25463	Mollusca	7805	Gastropod
Early Pleistocene	2.588	0.781	Olsson anc	1953	25463	Mollusca	7805	Gastropod
Early Pleistocene	2.588	0.781	Olsson anc	1953	25463	Mollusca	7805	Gastropod
Early Pleistocene	2.588	0.781	Olsson anc	1953	25463	Mollusca	7805	Gastropod
Early Pleistocene	2.588	0.781	Olsson anc	1953	25463	Mollusca	7805	Gastropod
Late Pleistocene	0.126	0.0117	Kittle and I	2010	40884	Mollusca	7805	Gastropod
Late Pleistocene	0.126	0.0117	Kittle and I	2010	40884	Mollusca	7805	Gastropod
Pliocene	5.333	2.588	Petuch	1986	47440	Mollusca	7805	Gastropod
Pliocene	5.333	2.588	Petuch	1986	47440	Mollusca	7805	Gastropod
Pliocene	5.333	2.588	Petuch	1986	47440	Mollusca	7805	Gastropod
Pliocene	5.333	2.588	Petuch	1986	47440	Mollusca	7805	Gastropod
Late Pleistocene	0.126	0.0117	DuBar	1971	17556	Mollusca	7805	Gastropod
Late Pleistocene	0.126	0.0117	DuBar	1971	17556	Mollusca	7805	Gastropod
Zanclean	5.333	3.6	Campbell a	1995	17558	Mollusca	7805	Gastropod
Early Pleistocene	2.588	0.781	DuBar	1971	17556	Mollusca	7805	Gastropod

Late Pleistocene		0.126	0.0117	Richards	1962	18974	Mollusca	7805	Gastropod
Late Pleistocene		0.126	0.0117	Richards	1962	18974	Mollusca	7805	Gastropod
Late Pleistocene		0.126	0.0117	Richards	1962	18974	Mollusca	7805	Gastropod
Late Pleistocene		0.126	0.0117	Richards	1962	18974	Mollusca	7805	Gastropod
Late Pleistocene		0.126	0.0117	Hendy et a	2008	19909	Mollusca	7805	Gastropod
Early Pleistocene		2.588	0.781	Cooke	1936	31145	Mollusca	7805	Gastropod
Late Pliocene		3.6	2.588	Cooke	1936	31145	Mollusca	7805	Gastropod
Late Pleistocene		0.126	0.0117	Holmes	1860	64712	Mollusca	7805	Gastropod
Late Pliocene		3.6	2.588	Ward and	1987	16700	Mollusca	7805	Gastropod
Late Pliocene		3.6	2.588	Ward and	1987	16700	Mollusca	7805	Gastropod
Late Pliocene		3.6	2.588	Ward and	1987	16700	Mollusca	7805	Gastropod
Early Pleistocene		2.588	0.781	Ward and	1987	16700	Mollusca	7805	Gastropod
Early Plioc	Late Plioce	5.333	2.588	Gardner	1948	17048	Mollusca	7805	Gastropod
Early Plioc	Late Plioce	5.333	2.588	Gardner	1948	17048	Mollusca	7805	Gastropod
Early Plioc	Late Plioce	5.333	2.588	Gardner	1948	17048	Mollusca	7805	Gastropod
Late Pliocene		3.6	2.588	Gardner	1948	17048	Mollusca	7805	Gastropod
Late Pliocene		3.6	2.588	Hendy et a	2008	19909	Mollusca	7805	Gastropod
Early Pleistocene		2.588	0.781	Hendy et a	2008	19909	Mollusca	7805	Gastropod
Early Pleistocene		2.588	0.781	Hendy et a	2008	19909	Mollusca	7805	Gastropod
Early Pleistocene		2.588	0.781	Hendy et a	2008	19909	Mollusca	7805	Gastropod
Late Pliocene		3.6	2.588	Edwards et	2005	17631	Mollusca	7805	Gastropod
Late Pliocene		3.6	2.588	Ward	1993	26351	Mollusca	7805	Gastropod
Late Pliocene		3.6	2.588	Ward	1993	26351	Mollusca	7805	Gastropod
Late Pliocene		3.6	2.588	Ward	1993	26351	Mollusca	7805	Gastropod
Late Pliocene		3.6	2.588	Ward	1993	26351	Mollusca	7805	Gastropod
Late Pliocene		3.6	2.588	Hendy et a	2008	19909	Mollusca	7805	Gastropod
Late Pliocene		3.6	2.588	Hendy et a	2008	19909	Mollusca	7805	Gastropod
Late Pliocene		3.6	2.588	Hendy et a	2008	19909	Mollusca	7805	Gastropod
Late Pliocene		3.6	2.588	Hendy et a	2008	19909	Mollusca	7805	Gastropod
Late Pleistocene		0.126	0.0117	Kittle and	2010	40884	Mollusca	7805	Gastropod
Late Pleistocene		0.126	0.0117	Kittle and	2010	40884	Mollusca	7805	Gastropod
Late Pleistocene		0.126	0.0117	Kittle and	2010	40884	Mollusca	7805	Gastropod
Pleistocene		2.588	0.0117	Auffenberg	2006	82817	Mollusca	7805	Gastropod
Pleistocene		2.588	0.0117	Auffenberg	2006	82817	Mollusca	7805	Gastropod
Pleistocene		2.588	0.0117	Auffenberg	2006	82817	Mollusca	7805	Gastropod
Late Pleistocene		0.126	0.0117	Guilday et	1977	35027	Mollusca	7805	Gastropod
Late Pleistocene		0.126	0.0117	Guilday	1962	35381	Mollusca	7805	Gastropod
Early Pleistocene		2.588	0.781	DuBar	1962	11119	Mollusca	7805	Gastropod
Calabrian		1.806	0.781	DuBar	1962	11119	Mollusca	7805	Gastropod
Calabrian		1.806	0.781	DuBar	1962	11119	Mollusca	7805	Gastropod
Late Pleistocene		0.126	0.0117	Hendy et a	2008	19909	Mollusca	7805	Gastropod
Early Pleistocene		2.588	0.781	Olsson and	1953	25463	Mollusca	7805	Gastropod
Late Pliocene		3.6	2.588	Hendy et a	2008	19909	Mollusca	7805	Gastropod
Middle Pleistocene		0.781	0.126	McGinty	1970	19114	Mollusca	7805	Gastropod
Early Pleistocene		2.588	0.781	Petuch and	2007	26813	Mollusca	7805	Gastropod
Late Plioce	Early Pleist	3.6	0.781	Petuch and	2007	26813	Mollusca	7805	Gastropod
Late Plioce	Early Pleist	3.6	0.781	Petuch and	2007	26813	Mollusca	7805	Gastropod

Early Pleistocene		2.588	0.781	Petuch	1988	31112	Mollusca	7805	Gastropod
Middle Pleistocene		0.781	0.126	Vokes	1998	18707	Mollusca	7805	Gastropod
Early Pleistocene		2.588	0.781	Jung	1994	19070	Mollusca	7805	Gastropod
Late Pleistocene		0.126	0.0117	Guilday et	1977	35027	Mollusca	7805	Gastropod
Late Pleistocene		0.126	0.0117	Guilday	1962	35381	Mollusca	7805	Gastropod
Late Pliocene		3.6	2.588	Petuch anc	2007	26813	Mollusca	7805	Gastropod
Early Pleistocene		2.588	0.781	Olsson anc	1953	25463	Mollusca	7805	Gastropod
Early Pleistocene		2.588	0.781	Fargo	1953	27010	Mollusca	7805	Gastropod
Early Pleistocene		2.588	0.781	Fargo	1953	27010	Mollusca	7805	Gastropod
Pliocene		5.333	2.588	Petuch	1986	47440	Mollusca	7805	Gastropod
Early Pleistocene		2.588	0.781	DuBar	1962	11119	Mollusca	7805	Gastropod
Calabrian		1.806	0.781	DuBar	1962	11119	Mollusca	7805	Gastropod
Early Pleistocene		2.588	0.781	DuBar	1962	11119	Mollusca	7805	Gastropod
Middle Pleistocene		0.781	0.126	Portell et a	1992	17634	Mollusca	7805	Gastropod
Late Pleistocene		0.126	0.0117	Kittle and I	2010	40884	Mollusca	7805	Gastropod
Pliocene		5.333	2.588	Aldrich	1911	53829	Mollusca	7805	Gastropod
Late Pleistocene		0.126	0.0117	Spencer ar	1987	15525	Mollusca	7805	Gastropod
Late Pliocene		3.6	2.588	Mansfield	1930	17060	Mollusca	7805	Gastropod
Middle Pleistocene		0.781	0.126	Hoerle	1970	19536	Mollusca	7805	Gastropod
Early Pleistocene		2.588	0.781	Pilsbry	1953	27012	Mollusca	7805	Gastropod
Early Pleistocene		2.588	0.781	Pilsbry	1953	27012	Mollusca	7805	Gastropod
Early Pleistocene		2.588	0.781	Pilsbry	1953	27012	Mollusca	7805	Gastropod
Early Pleistocene		2.588	0.781	Cooke	1936	31145	Mollusca	7805	Gastropod
Late Pliocene		3.6	2.588	Ward and I	1987	16700	Mollusca	7805	Gastropod
Late Pliocene		3.6	2.588	Ward and I	1987	16700	Mollusca	7805	Gastropod
Early Pleistocene		2.588	0.781	Ward and I	1987	16700	Mollusca	7805	Gastropod
Pleistocene		2.588	0.0117	Gardner	1948	17048	Mollusca	7805	Gastropod
Early Pleistocene		2.588	0.781	DuBar	1971	17556	Mollusca	7805	Gastropod
Late Pleistocene		0.126	0.0117	Sellards	1917	9011	Mollusca	7805	Gastropod
Late Pliocene		3.6	2.588	Olsson	1967	19826	Mollusca	7805	Gastropod
Late Plioce	Early Pleist	3.6	0.781	Petuch anc	2007	26813	Mollusca	7805	Gastropod
Late Plioce	Early Pleist	3.6	0.781	Petuch anc	2007	26813	Mollusca	7805	Gastropod
Late Plioce	Pleistocen	3.6	0.0117	Petuch anc	2007	26813	Mollusca	7805	Gastropod
Late Pliocene		3.6	2.588	Petuch anc	2007	26813	Mollusca	7805	Gastropod
Early Pleistocene		2.588	0.781	Olsson anc	1953	25463	Mollusca	7805	Gastropod
Late Pleistocene		0.126	0.0117	Kittle and I	2010	40884	Mollusca	7805	Gastropod
Late Pleistocene		0.126	0.0117	Richards	1962	18974	Mollusca	7805	Gastropod
Late Pleistocene		0.126	0.0117	Richards	1962	18974	Mollusca	7805	Gastropod
Late Pleistocene		0.126	0.0117	Richards	1962	18974	Mollusca	7805	Gastropod
Late Pleistocene		0.126	0.0117	Richards	1962	18974	Mollusca	7805	Gastropod
Late Pleistocene		0.126	0.0117	Richards	1962	18974	Mollusca	7805	Gastropod
Late Pleistocene		0.126	0.0117	Hendy et a	2008	19909	Mollusca	7805	Gastropod
Early Pleistocene		2.588	0.781	Cooke	1936	31145	Mollusca	7805	Gastropod
Pleistocene		2.588	0.0117	Holmes	1860	64712	Mollusca	7805	Gastropod
Pleistocene		2.588	0.0117	Holmes	1860	64712	Mollusca	7805	Gastropod
Early Pleistocene		2.588	0.781	Gardner	1948	17048	Mollusca	7805	Gastropod
Early Pleistocene		2.588	0.781	Gardner	1948	17048	Mollusca	7805	Gastropod

Early Pleistocene	2.588	0.781	Gardner	1948	17048	Mollusca	7805	Gastropod
Late Pliocene	3.6	2.588	Gardner	1948	17048	Mollusca	7805	Gastropod
Pleistocene	2.588	0.0117	Gardner	1948	17048	Mollusca	7805	Gastropod
Late Pleistocene	0.126	0.0117	Richards	1962	18974	Mollusca	7805	Gastropod
Late Pleistocene	0.126	0.0117	Richards	1962	18974	Mollusca	7805	Gastropod
Early Pleistocene	2.588	0.781	Hendy et a	2008	19909	Mollusca	7805	Gastropod
Early Pleistocene	2.588	0.781	Hendy et a	2008	19909	Mollusca	7805	Gastropod
Late Pleistocene	0.126	0.0117	Spencer ar	1987	15525	Mollusca	7805	Gastropod
Middle Pleistocene	0.781	0.126	Hoerle	1970	19536	Mollusca	7805	Gastropod
Late Pliocene	3.6	2.588	Petuch and	2007	26813	Mollusca	7805	Gastropod
Late Pliocene	3.6	2.588	Hendy et a	2008	19909	Mollusca	7805	Gastropod
Late Pleistocene	0.126	0.0117	Kittle and I	2010	40884	Mollusca	7805	Gastropod
Late Pleistocene	0.126	0.0117	Kittle and I	2010	40884	Mollusca	7805	Gastropod
Pleistocene	2.588	0.0117	Auffenberg	2006	82817	Mollusca	7805	Gastropod
Pleistocene	2.588	0.0117	Auffenberg	2006	82817	Mollusca	7805	Gastropod
Pleistocene	2.588	0.0117	Auffenberg	2006	82817	Mollusca	7805	Gastropod
Pleistocene	2.588	0.0117	Auffenberg	2006	82817	Mollusca	7805	Gastropod
Pleistocene	2.588	0.0117	Auffenberg	2006	82817	Mollusca	7805	Gastropod
Late Pleistocene	0.126	0.0117	Guilday	1962	35381	Mollusca	7805	Gastropod
Late Pliocene	3.6	2.588	Petuch and	2007	26813	Mollusca	7805	Gastropod
Early Pleistocene	2.588	0.781	DuBar	1962	11119	Mollusca	7805	Gastropod
Early Pleistocene	2.588	0.781	DuBar	1962	11119	Mollusca	7805	Gastropod
Early Pleistocene	2.588	0.781	DuBar	1962	11119	Mollusca	7805	Gastropod
Early Pleistocene	2.588	0.781	DuBar	1962	11119	Mollusca	7805	Gastropod
Early Pleistocene	2.588	0.781	DuBar	1962	11119	Mollusca	7805	Gastropod
Early Pleistocene	2.588	0.781	DuBar	1962	11119	Mollusca	7805	Gastropod
Early Pleistocene	2.588	0.781	DuBar	1962	11119	Mollusca	7805	Gastropod
Pliocene Pleistocene	5.333	0.0117	DuBar	1962	11119	Mollusca	7805	Gastropod
Early Pleistocene	2.588	0.781	DuBar	1962	11119	Mollusca	7805	Gastropod
Early Pleistocene	2.588	0.781	DuBar	1962	11119	Mollusca	7805	Gastropod
Late Pliocene	3.6	2.588	Mansfield	1930	17060	Mollusca	7805	Gastropod
Late Pliocene	3.6	2.588	Mansfield	1930	17060	Mollusca	7805	Gastropod
Late Pliocene	3.6	2.588	Mansfield	1930	17060	Mollusca	7805	Gastropod
Late Pliocene	3.6	2.588	Mansfield	1930	17060	Mollusca	7805	Gastropod
Late Pliocene	3.6	2.588	Mansfield	1930	17060	Mollusca	7805	Gastropod
Late Pliocene	3.6	2.588	Mansfield	1930	17060	Mollusca	7805	Gastropod
Late Pliocene	3.6	2.588	Mansfield	1930	17060	Mollusca	7805	Gastropod
Middle Pleistocene	0.781	0.126	Portell et a	1992	17634	Mollusca	7805	Gastropod
Late Pleistocene	0.126	0.0117	Portell et a	1992	17634	Mollusca	7805	Gastropod
Early Pleistocene	2.588	0.781	Fargo	1953	27010	Mollusca	7805	Gastropod
Early Pleistocene	2.588	0.781	Fargo	1953	27010	Mollusca	7805	Gastropod
Early Pleistocene	2.588	0.781	Fargo	1953	27010	Mollusca	7805	Gastropod
Early Pleistocene	2.588	0.781	Fargo	1953	27010	Mollusca	7805	Gastropod
Early Pleistocene	2.588	0.781	Fargo	1953	27010	Mollusca	7805	Gastropod
Early Pleistocene	2.588	0.781	Fargo	1953	27010	Mollusca	7805	Gastropod
Early Pleistocene	2.588	0.781	Fargo	1953	27010	Mollusca	7805	Gastropod
Early Pleistocene	2.588	0.781	Fargo	1953	27010	Mollusca	7805	Gastropod

Early Pleistocene	2.588	0.781	Fargo	1953	27010	Mollusca	7805	Gastropod
Early Pleistocene	2.588	0.781	Fargo	1953	27010	Mollusca	7805	Gastropod
Early Pleistocene	2.588	0.781	Fargo	1953	27010	Mollusca	7805	Gastropod
Early Pleistocene	2.588	0.781	Fargo	1953	27010	Mollusca	7805	Gastropod
Early Pleistocene	2.588	0.781	Fargo	1953	27010	Mollusca	7805	Gastropod
Early Pleistocene	2.588	0.781	Fargo	1953	27010	Mollusca	7805	Gastropod
Early Pleistocene	2.588	0.781	Fargo	1953	27010	Mollusca	7805	Gastropod
Early Pleistocene	2.588	0.781	Fargo	1953	27010	Mollusca	7805	Gastropod
Early Pleistocene	2.588	0.781	Fargo	1953	27010	Mollusca	7805	Gastropod
Late Pleistocene	0.126	0.0117	Kittle and I	2010	40884	Mollusca	7805	Gastropod
Late Pleistocene	0.126	0.0117	Kittle and I	2010	40884	Mollusca	7805	Gastropod
Pliocene	5.333	2.588	Petuch	1986	47440	Mollusca	7805	Gastropod
Late Pleistocene	0.126	0.0117	Richards	1962	18974	Mollusca	7805	Gastropod
Late Pleistocene	0.126	0.0117	Richards	1962	18974	Mollusca	7805	Gastropod
Pleistocene	2.588	0.0117	Holmes	1860	64712	Mollusca	7805	Gastropod
Early Pleistocene	2.588	0.781	Gardner	1948	17048	Mollusca	7805	Gastropod
Late Pliocene	3.6	2.588	Gardner	1948	17048	Mollusca	7805	Gastropod
Early Pleistocene	2.588	0.781	Gardner	1948	17048	Mollusca	7805	Gastropod
Early Pleistocene	2.588	0.781	Gardner	1948	17048	Mollusca	7805	Gastropod
Early Pleistocene	2.588	0.781	Gardner	1948	17048	Mollusca	7805	Gastropod
Early Pleistocene	2.588	0.781	Gardner	1948	17048	Mollusca	7805	Gastropod
Pleistocene	2.588	0.0117	Gardner	1948	17048	Mollusca	7805	Gastropod
Late Pleistocene	0.126	0.0117	Richards	1962	18974	Mollusca	7805	Gastropod
Late Pleistocene	0.126	0.0117	Richards	1962	18974	Mollusca	7805	Gastropod
Late Pleistocene	0.126	0.0117	Richards	1962	18974	Mollusca	7805	Gastropod
Gelasian	2.588	1.806	Bailey and	1986	23840	Mollusca	7805	Gastropod
Late Pleistocene	0.126	0.0117	Spencer ar	1987	15525	Mollusca	7805	Gastropod
Late Pleistocene	0.126	0.0117	Spencer ar	1987	15525	Mollusca	7805	Gastropod
Late Pleistocene	0.126	0.0117	Sellards	1917	9011	Mollusca	7805	Gastropod
Late Pleistocene	0.126	0.0117	Sellards	1917	9011	Mollusca	7805	Gastropod
Early Pleistocene	2.588	0.781	DuBar	1962	11119	Mollusca	7805	Gastropod
Early Pleistocene	2.588	0.781	DuBar	1962	11119	Mollusca	7805	Gastropod
Early Pleistocene	2.588	0.781	DuBar	1962	11119	Mollusca	7805	Gastropod
Early Pleistocene	2.588	0.781	DuBar	1962	11119	Mollusca	7805	Gastropod
Early Pleistocene	2.588	0.781	DuBar	1962	11119	Mollusca	7805	Gastropod
Early Pleistocene	2.588	0.781	DuBar	1962	11119	Mollusca	7805	Gastropod
Early Pleistocene	2.588	0.781	DuBar	1962	11119	Mollusca	7805	Gastropod
Early Pleistocene	2.588	0.781	DuBar	1962	11119	Mollusca	7805	Gastropod
Early Pleistocene	2.588	0.781	DuBar	1962	11119	Mollusca	7805	Gastropod
Early Pleistocene	2.588	0.781	DuBar	1962	11119	Mollusca	7805	Gastropod
Early Pleistocene	2.588	0.781	DuBar	1962	11119	Mollusca	7805	Gastropod
Calabrian	1.806	0.781	DuBar	1962	11119	Mollusca	7805	Gastropod
Calabrian	1.806	0.781	DuBar	1962	11119	Mollusca	7805	Gastropod
Calabrian	1.806	0.781	DuBar	1962	11119	Mollusca	7805	Gastropod
Calabrian	1.806	0.781	DuBar	1962	11119	Mollusca	7805	Gastropod
Late Pleistocene	0.126	0.0117	DuBar	1962	11119	Mollusca	7805	Gastropod
Late Pleistocene	0.126	0.0117	DuBar	1962	11119	Mollusca	7805	Gastropod

Early Pleistocene		2.588	0.781	DuBar	1962	11119	Mollusca	7805	Gastropod
Early Pleistocene		2.588	0.781	DuBar	1962	11119	Mollusca	7805	Gastropod
Early Pleistocene		2.588	0.781	DuBar	1962	11119	Mollusca	7805	Gastropod
Late Pleistocene		0.126	0.0117	DuBar	1962	11119	Mollusca	7805	Gastropod
Pliocene	Pleistocen	5.333	0.0117	DuBar	1962	11119	Mollusca	7805	Gastropod
Pliocene	Pleistocen	5.333	0.0117	DuBar	1962	11119	Mollusca	7805	Gastropod
Pliocene	Pleistocen	5.333	0.0117	DuBar	1962	11119	Mollusca	7805	Gastropod
Pliocene	Pleistocen	5.333	0.0117	DuBar	1962	11119	Mollusca	7805	Gastropod
Pliocene	Pleistocen	5.333	0.0117	DuBar	1962	11119	Mollusca	7805	Gastropod
Pliocene	Pleistocen	5.333	0.0117	DuBar	1962	11119	Mollusca	7805	Gastropod
Late Pliocene		3.6	2.588	Mansfield	1930	17060	Mollusca	7805	Gastropod
Late Pliocene		3.6	2.588	Mansfield	1930	17060	Mollusca	7805	Gastropod
Late Pliocene		3.6	2.588	Mansfield	1930	17060	Mollusca	7805	Gastropod
Late Pliocene		3.6	2.588	Mansfield	1930	17060	Mollusca	7805	Gastropod
Late Pliocene		3.6	2.588	Mansfield	1930	17060	Mollusca	7805	Gastropod
Late Pliocene		3.6	2.588	Mansfield	1930	17060	Mollusca	7805	Gastropod
Late Pliocene		3.6	2.588	Mansfield	1930	17060	Mollusca	7805	Gastropod
Early Pleistocene		2.588	0.781	Portell et a	1992	17634	Mollusca	7805	Gastropod
Early Pleistocene		2.588	0.781	Portell et a	1992	17634	Mollusca	7805	Gastropod
Early Pleistocene		2.588	0.781	Portell et a	1992	17634	Mollusca	7805	Gastropod
Early Pleistocene		2.588	0.781	Portell et a	1992	17634	Mollusca	7805	Gastropod
Early Pleistocene		2.588	0.781	Portell et a	1992	17634	Mollusca	7805	Gastropod
Middle Pleistocene		0.781	0.126	Portell et a	1992	17634	Mollusca	7805	Gastropod
Middle Pleistocene		0.781	0.126	Portell et a	1992	17634	Mollusca	7805	Gastropod
Middle Pleistocene		0.781	0.126	Portell et a	1992	17634	Mollusca	7805	Gastropod
Late Pleistocene		0.126	0.0117	Portell et a	1992	17634	Mollusca	7805	Gastropod
Late Pleistocene		0.126	0.0117	Hendy et a	2008	19909	Mollusca	7805	Gastropod
Late Pleistocene		0.126	0.0117	Hendy et a	2008	19909	Mollusca	7805	Gastropod
Late Pleistocene		0.126	0.0117	Hendy et a	2008	19909	Mollusca	7805	Gastropod
Late Pleistocene		0.126	0.0117	Hendy et a	2008	19909	Mollusca	7805	Gastropod
Early Pleistocene		2.588	0.781	Schroeder	1954	25303	Mollusca	7805	Gastropod
Early Pleistocene		2.588	0.781	Schroeder	1954	25303	Mollusca	7805	Gastropod
Early Pleistocene		2.588	0.781	Schroeder	1954	25303	Mollusca	7805	Gastropod
Pliocene		5.333	2.588	Schroeder	1954	25303	Mollusca	7805	Gastropod
Pliocene		5.333	2.588	Schroeder	1954	25303	Mollusca	7805	Gastropod
Pliocene		5.333	2.588	Schroeder	1954	25303	Mollusca	7805	Gastropod
Pliocene		5.333	2.588	Schroeder	1954	25303	Mollusca	7805	Gastropod
Pliocene		5.333	2.588	Schroeder	1954	25303	Mollusca	7805	Gastropod
Early Pleistocene		2.588	0.781	Schroeder	1954	25303	Mollusca	7805	Gastropod
Early Pleistocene		2.588	0.781	Olsson anc	1953	25463	Mollusca	7805	Gastropod
Early Pleistocene		2.588	0.781	Olsson anc	1953	25463	Mollusca	7805	Gastropod
Early Pleistocene		2.588	0.781	Olsson anc	1953	25463	Mollusca	7805	Gastropod
Early Pleistocene		2.588	0.781	Olsson anc	1953	25463	Mollusca	7805	Gastropod
Early Pleistocene		2.588	0.781	Olsson anc	1953	25463	Mollusca	7805	Gastropod
Early Pleistocene		2.588	0.781	Olsson anc	1953	25463	Mollusca	7805	Gastropod
Early Pleistocene		2.588	0.781	Olsson anc	1953	25463	Mollusca	7805	Gastropod
Early Pleistocene		2.588	0.781	Olsson anc	1953	25463	Mollusca	7805	Gastropod

Early Pleistocene	2.588	0.781	Olsson anc	1953	25463	Mollusca	7805	Gastropod
Early Pleistocene	2.588	0.781	Olsson anc	1953	25463	Mollusca	7805	Gastropod
Early Pleistocene	2.588	0.781	Olsson anc	1953	25463	Mollusca	7805	Gastropod
Early Pleistocene	2.588	0.781	Olsson anc	1953	25463	Mollusca	7805	Gastropod
Early Pleistocene	2.588	0.781	Olsson anc	1953	25463	Mollusca	7805	Gastropod
Late Pliocene	3.6	2.588	Olsson	1967	19826	Mollusca	7805	Gastropod
Late Pliocene	3.6	2.588	Olsson	1967	19826	Mollusca	7805	Gastropod
Early Pleistocene	2.588	0.781	Hendy et a	2008	19909	Mollusca	7805	Gastropod
Early Pleistocene	2.588	0.781	Hendy et a	2008	19909	Mollusca	7805	Gastropod
Early Pleistocene	2.588	0.781	DuBar	1958	25514	Mollusca	7805	Gastropod
Late Pleistocene	0.126	0.0117	DuBar	1958	25514	Mollusca	7805	Gastropod
Late Pleistocene	0.126	0.0117	Hendy et a	2008	19909	Mollusca	7805	Gastropod
Early Pleistocene	2.588	0.781	Hendy et a	2008	19909	Mollusca	7805	Gastropod
Early Pleistocene	2.588	0.781	Hendy et a	2008	19909	Mollusca	7805	Gastropod
Early Pleistocene	2.588	0.781	Hendy et a	2008	19909	Mollusca	7805	Gastropod
Middle Pleistocene	0.781	0.126	Hendy et a	2008	19909	Mollusca	7805	Gastropod
Late Pleistocene	0.126	0.0117	Hendy et a	2008	19909	Mollusca	7805	Gastropod
Early Pleistocene	2.588	0.781	Hendy et a	2008	19909	Mollusca	7805	Gastropod
Early Pleistocene	2.588	0.781	Hendy et a	2008	19909	Mollusca	7805	Gastropod
Early Pleistocene	2.588	0.781	DuBar	1958	25514	Mollusca	7805	Gastropod
Late Pleistocene	0.126	0.0117	Kittle and I	2010	40884	Mollusca	7805	Gastropod
Late Pleistocene	0.126	0.0117	Kittle and I	2010	40884	Mollusca	7805	Gastropod
Late Pleistocene	0.126	0.0117	Kittle and I	2010	40884	Mollusca	7805	Gastropod
Late Pleistocene	0.126	0.0117	Kittle and I	2010	40884	Mollusca	7805	Gastropod
Late Pleistocene	0.126	0.0117	DuBar	1971	17556	Mollusca	7805	Gastropod
Early Pleistocene	2.588	0.781	DuBar	1971	17556	Mollusca	7805	Gastropod
Late Pleistocene	0.126	0.0117	Richards	1962	18974	Mollusca	7805	Gastropod
Late Pleistocene	0.126	0.0117	Richards	1962	18974	Mollusca	7805	Gastropod
Late Pleistocene	0.126	0.0117	Richards	1962	18974	Mollusca	7805	Gastropod
Late Pleistocene	0.126	0.0117	Hendy et a	2008	19909	Mollusca	7805	Gastropod
Pleistocene	2.588	0.0117	Hendy et a	2008	19909	Mollusca	7805	Gastropod
Early Pleistocene	2.588	0.781	Cooke	1936	31145	Mollusca	7805	Gastropod
Early Pleistocene	2.588	0.781	Cooke	1936	31145	Mollusca	7805	Gastropod
Early Pleistocene	2.588	0.781	Cooke	1936	31145	Mollusca	7805	Gastropod
Late Pliocene	3.6	2.588	Ward and I	1987	16700	Mollusca	7805	Gastropod
Late Pliocene	3.6	2.588	Ward and I	1987	16700	Mollusca	7805	Gastropod
Late Pliocene	3.6	2.588	Ward and I	1987	16700	Mollusca	7805	Gastropod
Late Pliocene	3.6	2.588	Ward and I	1987	16700	Mollusca	7805	Gastropod
Late Pliocene	3.6	2.588	Ward and I	1987	16700	Mollusca	7805	Gastropod
Early Pleistocene	2.588	0.781	Ward and I	1987	16700	Mollusca	7805	Gastropod
Early Pleistocene	2.588	0.781	Ward and I	1987	16700	Mollusca	7805	Gastropod
Early Pleistocene	2.588	0.781	Ward and I	1987	16700	Mollusca	7805	Gastropod
Late Pliocene	3.6	2.588	Gardner	1948	17048	Mollusca	7805	Gastropod
Late Pliocene	3.6	2.588	Gardner	1948	17048	Mollusca	7805	Gastropod
Late Pliocene	3.6	2.588	Gardner	1948	17048	Mollusca	7805	Gastropod
Early Pleistocene	2.588	0.781	Miller	1990	17561	Mollusca	7805	Gastropod
Early Pleistocene	2.588	0.781	Miller	1990	17561	Mollusca	7805	Gastropod

Early Pleistocene	2.588	0.781	Miller	1990	17561	Mollusca	7805	Gastropod
Early Pleistocene	2.588	0.781	Miller	1990	17561	Mollusca	7805	Gastropod
Early Pleistocene	2.588	0.781	Miller	1990	17561	Mollusca	7805	Gastropod
Early Pleistocene	2.588	0.781	Miller	1990	17561	Mollusca	7805	Gastropod
Late Pleistocene	0.126	0.0117	Richards	1962	18974	Mollusca	7805	Gastropod
Late Pliocene	3.6	2.588	Hendy et a	2008	19909	Mollusca	7805	Gastropod
Late Pleistocene	0.126	0.0117	Hendy et a	2008	19909	Mollusca	7805	Gastropod
Gelasian	2.588	1.806	Bailey and	1986	23840	Mollusca	7805	Gastropod
Gelasian	2.588	1.806	Bailey and	1986	23840	Mollusca	7805	Gastropod
Late Pliocene	3.6	2.588	Hendy et a	2008	19909	Mollusca	7805	Gastropod
Late Pliocene	3.6	2.588	Hendy et a	2008	19909	Mollusca	7805	Gastropod
Pliocene	5.333	2.588	Hendy et a	2008	19909	Mollusca	7805	Gastropod
Late Pliocene	3.6	2.588	Hendy et a	2008	19909	Mollusca	7805	Gastropod
Late Pliocene	3.6	2.588	Hendy et a	2008	19909	Mollusca	7805	Gastropod
Early Pleistocene	2.588	0.781	Hendy et a	2008	19909	Mollusca	7805	Gastropod
Early Pleistocene	2.588	0.781	Hendy et a	2008	19909	Mollusca	7805	Gastropod
Early Pleistocene	2.588	0.781	Hendy et a	2008	19909	Mollusca	7805	Gastropod
Early Pleistocene	2.588	0.781	Miller and	1988	72890	Mollusca	7805	Gastropod
Pleistocene	2.588	0.0117	Coch	1971	441	Mollusca	7805	Gastropod
Late Pleistocene	0.126	0.0117	Spencer ar	1987	15525	Mollusca	7805	Gastropod
Late Pleistocene	0.126	0.0117	Spencer ar	1987	15525	Mollusca	7805	Gastropod
Late Pleistocene	0.126	0.0117	Hendy et a	2008	19909	Mollusca	7805	Gastropod
Late Pleistocene	0.126	0.0117	Hendy et a	2008	19909	Mollusca	7805	Gastropod
Late Pliocene	3.6	2.588	Ward	1993	26351	Mollusca	7805	Gastropod
Late Pliocene	3.6	2.588	Ward	1993	26351	Mollusca	7805	Gastropod
Late Pliocene	3.6	2.588	Ward	1993	26351	Mollusca	7805	Gastropod
Late Pliocene	3.6	2.588	Ward	1993	26351	Mollusca	7805	Gastropod
Late Pliocene	3.6	2.588	Ward	1993	26351	Mollusca	7805	Gastropod
Late Pliocene	3.6	2.588	Ward	1993	26351	Mollusca	7805	Gastropod
Late Pliocene	3.6	2.588	Ward	1993	26351	Mollusca	7805	Gastropod
Late Pliocene	3.6	2.588	Ward	1993	26351	Mollusca	7805	Gastropod
Late Pliocene	3.6	2.588	Ward	1993	26351	Mollusca	7805	Gastropod
Late Pliocene	3.6	2.588	Ward	1993	26351	Mollusca	7805	Gastropod
Late Pliocene	3.6	2.588	Hendy et a	2008	19909	Mollusca	7805	Gastropod
Late Pleistocene	0.126	0.0117	Sellards	1917	9011	Mollusca	7805	Gastropod
Late Pleistocene	0.126	0.0117	Sellards	1917	9011	Mollusca	7805	Gastropod
Late Pleistocene	0.126	0.0117	Sellards	1917	9011	Mollusca	7805	Gastropod
Early Pleistocene	2.588	0.781	DuBar	1962	11119	Mollusca	7805	Gastropod
Early Pleistocene	2.588	0.781	DuBar	1962	11119	Mollusca	7805	Gastropod
Early Pleistocene	2.588	0.781	DuBar	1962	11119	Mollusca	7805	Gastropod
Early Pleistocene	2.588	0.781	DuBar	1962	11119	Mollusca	7805	Gastropod
Early Pleistocene	2.588	0.781	DuBar	1962	11119	Mollusca	7805	Gastropod
Early Pleistocene	2.588	0.781	DuBar	1962	11119	Mollusca	7805	Gastropod
Early Pleistocene	2.588	0.781	DuBar	1962	11119	Mollusca	7805	Gastropod
Calabrian	1.806	0.781	DuBar	1962	11119	Mollusca	7805	Gastropod
Calabrian	1.806	0.781	DuBar	1962	11119	Mollusca	7805	Gastropod
Calabrian	1.806	0.781	DuBar	1962	11119	Mollusca	7805	Gastropod

Late Pleistocene		0.126	0.0117	DuBar	1962	11119	Mollusca	7805	Gastropod
Early Pleistocene		2.588	0.781	DuBar	1962	11119	Mollusca	7805	Gastropod
Early Pleistocene		2.588	0.781	DuBar	1962	11119	Mollusca	7805	Gastropod
Early Pleistocene		2.588	0.781	DuBar	1962	11119	Mollusca	7805	Gastropod
Late Pleistocene		0.126	0.0117	DuBar	1962	11119	Mollusca	7805	Gastropod
Late Pleistocene		0.126	0.0117	DuBar	1962	11119	Mollusca	7805	Gastropod
Late Pleistocene		0.126	0.0117	DuBar	1962	11119	Mollusca	7805	Gastropod
Pliocene	Pleistocen	5.333	0.0117	DuBar	1962	11119	Mollusca	7805	Gastropod
Pliocene	Pleistocen	5.333	0.0117	DuBar	1962	11119	Mollusca	7805	Gastropod
Pliocene	Pleistocen	5.333	0.0117	DuBar	1962	11119	Mollusca	7805	Gastropod
Pliocene	Pleistocen	5.333	0.0117	DuBar	1962	11119	Mollusca	7805	Gastropod
Pliocene	Pleistocen	5.333	0.0117	DuBar	1962	11119	Mollusca	7805	Gastropod
Late Pliocene		3.6	2.588	Mansfield	1932	17094	Mollusca	7805	Gastropod
Late Pliocene		3.6	2.588	Mansfield	1932	17094	Mollusca	7805	Gastropod
Late Pliocene		3.6	2.588	Mansfield	1932	17094	Mollusca	7805	Gastropod
Early Pleistocene		2.588	0.781	Portell et a	1992	17634	Mollusca	7805	Gastropod
Early Pleistocene		2.588	0.781	Portell et a	1992	17634	Mollusca	7805	Gastropod
Early Pleistocene		2.588	0.781	Portell et a	1992	17634	Mollusca	7805	Gastropod
Middle Pleistocene		0.781	0.126	Portell et a	1992	17634	Mollusca	7805	Gastropod
Middle Pleistocene		0.781	0.126	Portell et a	1992	17634	Mollusca	7805	Gastropod
Middle Pleistocene		0.781	0.126	Portell et a	1992	17634	Mollusca	7805	Gastropod
Late Pleistocene		0.126	0.0117	Portell et a	1992	17634	Mollusca	7805	Gastropod
Middle Pleistocene		0.781	0.126	McGinty	1970	19114	Mollusca	7805	Gastropod
Late Pleistocene		0.126	0.0117	Hendy et a	2008	19909	Mollusca	7805	Gastropod
Late Pleistocene		0.126	0.0117	Hendy et a	2008	19909	Mollusca	7805	Gastropod
Late Pleistocene		0.126	0.0117	Hendy et a	2008	19909	Mollusca	7805	Gastropod
Late Pleistocene		0.126	0.0117	Hendy et a	2008	19909	Mollusca	7805	Gastropod
Early Pleistocene		2.588	0.781	Schroeder	1954	25303	Mollusca	7805	Gastropod
Early Pleistocene		2.588	0.781	Olsson and	1953	25463	Mollusca	7805	Gastropod
Early Pleistocene		2.588	0.781	Olsson and	1953	25463	Mollusca	7805	Gastropod
Early Pleistocene		2.588	0.781	Olsson and	1953	25463	Mollusca	7805	Gastropod
Early Pleistocene		2.588	0.781	Olsson and	1953	25463	Mollusca	7805	Gastropod
Early Pleistocene		2.588	0.781	Olsson and	1953	25463	Mollusca	7805	Gastropod
Early Pleistocene		2.588	0.781	Olsson and	1953	25463	Mollusca	7805	Gastropod
Early Pleistocene		2.588	0.781	Olsson and	1953	25463	Mollusca	7805	Gastropod
Late Pleistocene		0.126	0.0117	Lovejoy	1992	26806	Mollusca	7805	Gastropod
Late Pleistocene		0.126	0.0117	Petuch and	2007	26813	Mollusca	7805	Gastropod
Late Pleistocene		0.126	0.0117	Petuch and	2007	26813	Mollusca	7805	Gastropod
Late Pleistocene		0.126	0.0117	Petuch and	2007	26813	Mollusca	7805	Gastropod
Late Pleistocene		0.126	0.0117	Petuch and	2007	26813	Mollusca	7805	Gastropod
Late Pleistocene		0.126	0.0117	Petuch and	2007	26813	Mollusca	7805	Gastropod
Late Pleistocene		0.126	0.0117	Petuch and	2007	26813	Mollusca	7805	Gastropod
Middle Pleistocene		0.781	0.126	Petuch and	2007	26813	Mollusca	7805	Gastropod
Middle Pleistocene		0.781	0.126	Petuch and	2007	26813	Mollusca	7805	Gastropod
Middle Pleistocene		0.781	0.126	Petuch and	2007	26813	Mollusca	7805	Gastropod
Middle Pleistocene		0.781	0.126	Petuch and	2007	26813	Mollusca	7805	Gastropod
Early Pleistocene		2.588	0.781	Petuch and	2007	26813	Mollusca	7805	Gastropod

Early Pleistocene	2.588	0.781	Petuch anc	2007	26813	Mollusca	7805	Gastropod
Early Pleistocene	2.588	0.781	Petuch anc	2007	26813	Mollusca	7805	Gastropod
Early Pleistocene	2.588	0.781	Petuch anc	2007	26813	Mollusca	7805	Gastropod
Early Pleistocene	2.588	0.781	Petuch anc	2007	26813	Mollusca	7805	Gastropod
Early Pleistocene	2.588	0.781	Petuch anc	2007	26813	Mollusca	7805	Gastropod
Middle Pleistocene	0.781	0.126	Petuch anc	2007	26813	Mollusca	7805	Gastropod
Middle Pleistocene	0.781	0.126	Petuch anc	2007	26813	Mollusca	7805	Gastropod
Middle Pleistocene	0.781	0.126	Petuch anc	2007	26813	Mollusca	7805	Gastropod
Middle Pleistocene	0.781	0.126	Petuch anc	2007	26813	Mollusca	7805	Gastropod
Middle Pleistocene	0.781	0.126	Petuch anc	2007	26813	Mollusca	7805	Gastropod
Middle Pleistocene	0.781	0.126	Petuch anc	2007	26813	Mollusca	7805	Gastropod
Late Pliocene	3.6	2.588	Olsson	1967	19826	Mollusca	7805	Gastropod
Late Pliocene	3.6	2.588	Olsson	1967	19826	Mollusca	7805	Gastropod
Late Pliocene	3.6	2.588	Olsson	1967	19826	Mollusca	7805	Gastropod
Middle Pleistocene	0.781	0.126	Olsson anc	1964	26836	Mollusca	7805	Gastropod
Early Pleistocene	2.588	0.781	Petuch anc	2007	26813	Mollusca	7805	Gastropod
Early Pleistocene	2.588	0.781	Petuch anc	2007	26813	Mollusca	7805	Gastropod
Early Pleistocene	2.588	0.781	Petuch anc	2007	26813	Mollusca	7805	Gastropod
Early Pleistocene	2.588	0.781	Petuch anc	2007	26813	Mollusca	7805	Gastropod
Early Pleistocene	2.588	0.781	Petuch anc	2007	26813	Mollusca	7805	Gastropod
Late Plioce Early Pleist	3.6	0.781	Petuch anc	2007	26813	Mollusca	7805	Gastropod
Late Plioce Early Pleist	3.6	0.781	Petuch anc	2007	26813	Mollusca	7805	Gastropod
Late Plioce Early Pleist	3.6	0.781	Petuch anc	2007	26813	Mollusca	7805	Gastropod
Early Pleistocene	2.588	0.781	Petuch anc	2007	26813	Mollusca	7805	Gastropod
Early Pleistocene	2.588	0.781	Petuch anc	2007	26813	Mollusca	7805	Gastropod
Early Pleistocene	2.588	0.781	Petuch anc	2007	26813	Mollusca	7805	Gastropod
Late Plioce Early Pleist	3.6	0.781	Petuch anc	2007	26813	Mollusca	7805	Gastropod
Late Plioce Early Pleist	3.6	0.781	Petuch anc	2007	26813	Mollusca	7805	Gastropod
Late Plioce Early Pleist	3.6	0.781	Petuch anc	2007	26813	Mollusca	7805	Gastropod
Late Plioce Early Pleist	3.6	0.781	Petuch anc	2007	26813	Mollusca	7805	Gastropod
Late Plioce Early Pleist	3.6	0.781	Petuch anc	2007	26813	Mollusca	7805	Gastropod
Late Plioce Pleistocen	3.6	0.0117	Petuch anc	2007	26813	Mollusca	7805	Gastropod
Late Pliocene	3.6	2.588	Petuch anc	2007	26813	Mollusca	7805	Gastropod
Late Pliocene	3.6	2.588	Petuch anc	2007	26813	Mollusca	7805	Gastropod
Late Pliocene	3.6	2.588	Petuch anc	2007	26813	Mollusca	7805	Gastropod
Late Pliocene	3.6	2.588	Petuch anc	2007	26813	Mollusca	7805	Gastropod
Late Pliocene	3.6	2.588	Petuch anc	2007	26813	Mollusca	7805	Gastropod
Late Pliocene	3.6	2.588	Petuch anc	2007	26813	Mollusca	7805	Gastropod
Late Pliocene	3.6	2.588	Petuch anc	2007	26813	Mollusca	7805	Gastropod
Late Pliocene	3.6	2.588	Petuch anc	2007	26813	Mollusca	7805	Gastropod
Late Pliocene	3.6	2.588	Petuch anc	2007	26813	Mollusca	7805	Gastropod
Late Pliocene	3.6	2.588	Petuch anc	2007	26813	Mollusca	7805	Gastropod
Late Pliocene	3.6	2.588	Petuch anc	2007	26813	Mollusca	7805	Gastropod
Early Pleistocene	2.588	0.781	Hendy et a	2008	19909	Mollusca	7805	Gastropod
Early Pleistocene	2.588	0.781	Hendy et a	2008	19909	Mollusca	7805	Gastropod
Early Pleistocene	2.588	0.781	Hendy et a	2008	19909	Mollusca	7805	Gastropod
Early Pleistocene	2.588	0.781	Hendy et a	2008	19909	Mollusca	7805	Gastropod

Late Pliocene	3.6	2.588	Petuch and	2007	26813	Mollusca	7805	Gastropod
Late Pliocene	3.6	2.588	Petuch and	2007	26813	Mollusca	7805	Gastropod
Late Pliocene	3.6	2.588	Petuch and	2007	26813	Mollusca	7805	Gastropod
Late Pliocene	3.6	2.588	Petuch and	2007	26813	Mollusca	7805	Gastropod
Late Pliocene	3.6	2.588	Petuch and	2007	26813	Mollusca	7805	Gastropod
Late Pliocene	3.6	2.588	Petuch and	2007	26813	Mollusca	7805	Gastropod
Late Pliocene	3.6	2.588	Petuch and	2007	26813	Mollusca	7805	Gastropod
Late Pliocene	3.6	2.588	Petuch and	2007	26813	Mollusca	7805	Gastropod
Late Pliocene	3.6	2.588	Petuch and	2007	26813	Mollusca	7805	Gastropod
Late Pliocene	3.6	2.588	Petuch and	2007	26813	Mollusca	7805	Gastropod
Late Pliocene	3.6	2.588	Petuch and	2007	26813	Mollusca	7805	Gastropod
Late Pliocene	3.6	2.588	Petuch and	2007	26813	Mollusca	7805	Gastropod
Late Pliocene	3.6	2.588	Petuch and	2007	26813	Mollusca	7805	Gastropod
Late Pliocene	3.6	2.588	Petuch and	2007	26813	Mollusca	7805	Gastropod
Late Pliocene	3.6	2.588	Petuch and	2007	26813	Mollusca	7805	Gastropod
Late Pliocene	3.6	2.588	Petuch and	2007	26813	Mollusca	7805	Gastropod
Late Pliocene	3.6	2.588	Petuch and	2007	26813	Mollusca	7805	Gastropod
Late Pliocene	3.6	2.588	Petuch and	2007	26813	Mollusca	7805	Gastropod
Late Pliocene	3.6	2.588	Petuch and	2007	26813	Mollusca	7805	Gastropod
Late Pliocene	3.6	2.588	Petuch and	2007	26813	Mollusca	7805	Gastropod
Late Pliocene	3.6	2.588	Petuch and	2007	26813	Mollusca	7805	Gastropod
Late Pliocene	3.6	2.588	Petuch and	2007	26813	Mollusca	7805	Gastropod
Late Pliocene	3.6	2.588	Petuch and	2007	26813	Mollusca	7805	Gastropod
Late Pliocene	3.6	2.588	Petuch and	2007	26813	Mollusca	7805	Gastropod
Late Pliocene	3.6	2.588	Petuch and	2007	26813	Mollusca	7805	Gastropod
Late Pliocene	3.6	2.588	Petuch and	2007	26813	Mollusca	7805	Gastropod
Late Pleistocene	0.126	0.0117	Hendy et a	2008	19909	Mollusca	7805	Gastropod
Early Pleistocene	2.588	0.781	Hendy et a	2008	19909	Mollusca	7805	Gastropod
Early Pleistocene	2.588	0.781	Hendy et a	2008	19909	Mollusca	7805	Gastropod
Early Pleistocene	2.588	0.781	Hendy et a	2008	19909	Mollusca	7805	Gastropod
Early Pleistocene	2.588	0.781	Hendy et a	2008	19909	Mollusca	7805	Gastropod
Middle Pleistocene	0.781	0.126	Hendy et a	2008	19909	Mollusca	7805	Gastropod
Middle Pleistocene	0.781	0.126	Hendy et a	2008	19909	Mollusca	7805	Gastropod
Middle Pleistocene	0.781	0.126	Hendy et a	2008	19909	Mollusca	7805	Gastropod
Late Pleistocene	0.126	0.0117	Hendy et a	2008	19909	Mollusca	7805	Gastropod
Early Pleistocene	2.588	0.781	Hendy et a	2008	19909	Mollusca	7805	Gastropod
Late Pliocene	3.6	2.588	Petuch	1988	31112	Mollusca	7805	Gastropod
Late Pliocene	3.6	2.588	Petuch	1988	31112	Mollusca	7805	Gastropod
Late Pliocene	3.6	2.588	Petuch	1988	31112	Mollusca	7805	Gastropod
Late Pliocene	3.6	2.588	Petuch	1988	31112	Mollusca	7805	Gastropod
Late Pliocene	3.6	2.588	Petuch	1988	31112	Mollusca	7805	Gastropod
Late Pliocene	3.6	2.588	Petuch	1988	31112	Mollusca	7805	Gastropod
Late Pliocene	3.6	2.588	Petuch	1988	31112	Mollusca	7805	Gastropod
Late Pliocene	3.6	2.588	Petuch	1988	31112	Mollusca	7805	Gastropod
Late Pliocene	3.6	2.588	Petuch	1988	31112	Mollusca	7805	Gastropod
Late Pliocene	3.6	2.588	Petuch	1988	31112	Mollusca	7805	Gastropod
Early Pleistocene	2.588	0.781	Petuch	1988	31112	Mollusca	7805	Gastropod

Late Pliocene	3.6	2.588	Petuch	1988	31112	Mollusca	7805	Gastropod
Middle Pleistocene	0.781	0.126	Petuch	1988	31112	Mollusca	7805	Gastropod
Middle Pleistocene	0.781	0.126	Petuch	1988	31112	Mollusca	7805	Gastropod
Middle Pleistocene	0.781	0.126	Petuch	1988	31112	Mollusca	7805	Gastropod
Middle Pleistocene	0.781	0.126	Petuch	1988	31112	Mollusca	7805	Gastropod
Middle Pleistocene	0.781	0.126	Petuch	1988	31112	Mollusca	7805	Gastropod
Late Pleistocene	0.126	0.0117	Petuch	1988	31112	Mollusca	7805	Gastropod
Late Pleistocene	0.126	0.0117	Kittle and I	2010	40884	Mollusca	7805	Gastropod
Late Pleistocene	0.126	0.0117	Kittle and I	2010	40884	Mollusca	7805	Gastropod
Late Pleistocene	0.126	0.0117	Kittle and I	2010	40884	Mollusca	7805	Gastropod
Late Pleistocene	0.126	0.0117	Kittle and I	2010	40884	Mollusca	7805	Gastropod
Late Pleistocene	0.126	0.0117	Kittle and I	2010	40884	Mollusca	7805	Gastropod
Late Pleistocene	0.126	0.0117	Hulbert an	1998	1962	Mollusca	7805	Gastropod
Late Pleistocene	0.126	0.0117	Hulbert an	1998	1962	Mollusca	7805	Gastropod
Late Pleistocene	0.126	0.0117	Hulbert an	1998	1962	Mollusca	7805	Gastropod
Zanclean	5.333	3.6	Campbell a	1995	17558	Mollusca	7805	Gastropod
Zanclean	5.333	3.6	Campbell a	1995	17558	Mollusca	7805	Gastropod
Zanclean	5.333	3.6	Campbell a	1995	17558	Mollusca	7805	Gastropod
Zanclean	5.333	3.6	Campbell a	1995	17558	Mollusca	7805	Gastropod
Early Pliocene	5.333	3.6	Campbell a	1995	17558	Mollusca	7805	Gastropod
Early Pleistocene	2.588	0.781	DuBar	1971	17556	Mollusca	7805	Gastropod
Late Pleistocene	0.126	0.0117	Richards	1962	18974	Mollusca	7805	Gastropod
Late Pleistocene	0.126	0.0117	Richards	1962	18974	Mollusca	7805	Gastropod
Late Pleistocene	0.126	0.0117	Richards	1962	18974	Mollusca	7805	Gastropod
Late Pleistocene	0.126	0.0117	Richards	1962	18974	Mollusca	7805	Gastropod
Late Pleistocene	0.126	0.0117	Richards	1962	18974	Mollusca	7805	Gastropod
Late Pleistocene	0.126	0.0117	Richards	1962	18974	Mollusca	7805	Gastropod
Late Pleistocene	0.126	0.0117	Richards	1962	18974	Mollusca	7805	Gastropod
Late Pleistocene	0.126	0.0117	Richards	1962	18974	Mollusca	7805	Gastropod
Late Pleistocene	0.126	0.0117	Richards	1962	18974	Mollusca	7805	Gastropod
Early Pleistocene	2.588	0.781	Cooke	1936	31145	Mollusca	7805	Gastropod
Pleistocene	2.588	0.0117	Holmes	1860	64712	Mollusca	7805	Gastropod
Pleistocene	2.588	0.0117	Holmes	1860	64712	Mollusca	7805	Gastropod
Late Pliocene	3.6	2.588	Ward and I	1987	16700	Mollusca	7805	Gastropod
Early Pleistocene	2.588	0.781	Ward and I	1987	16700	Mollusca	7805	Gastropod
Early Pleistocene	2.588	0.781	Ward and I	1987	16700	Mollusca	7805	Gastropod
Early Pleistocene	2.588	0.781	Ward and I	1987	16700	Mollusca	7805	Gastropod
Pleistocene	2.588	0.0117	Gardner	1948	17048	Mollusca	7805	Gastropod
Late Pliocene	3.6	2.588	Gardner	1948	17048	Mollusca	7805	Gastropod
Early Pleistocene	2.588	0.781	Gardner	1948	17048	Mollusca	7805	Gastropod
Late Pliocene	3.6	2.588	Gardner	1948	17048	Mollusca	7805	Gastropod
Late Pliocene	3.6	2.588	Gardner	1948	17048	Mollusca	7805	Gastropod
Late Pliocene	3.6	2.588	Gardner	1948	17048	Mollusca	7805	Gastropod
Pleistocene	2.588	0.0117	Gardner	1948	17048	Mollusca	7805	Gastropod
Early Pleistocene	2.588	0.781	Gardner	1948	17048	Mollusca	7805	Gastropod
Pleistocene	2.588	0.0117	Gardner	1948	17048	Mollusca	7805	Gastropod
Early Pleistocene	2.588	0.781	Gardner	1948	17048	Mollusca	7805	Gastropod

Early Pleistocene	2.588	0.781	Gardner	1948	17048	Mollusca	7805	Gastropod
Pleistocene	2.588	0.0117	Gardner	1948	17048	Mollusca	7805	Gastropod
Early Pleistocene	2.588	0.781	Miller	1990	17561	Mollusca	7805	Gastropod
Early Pleistocene	2.588	0.781	Miller	1990	17561	Mollusca	7805	Gastropod
Early Pleistocene	2.588	0.781	Miller	1990	17561	Mollusca	7805	Gastropod
Early Pleistocene	2.588	0.781	Miller	1990	17561	Mollusca	7805	Gastropod
Late Pleistocene	0.126	0.0117	Richards	1962	18974	Mollusca	7805	Gastropod
Late Pleistocene	0.126	0.0117	Richards	1962	18974	Mollusca	7805	Gastropod
Late Pleistocene	0.126	0.0117	Richards	1962	18974	Mollusca	7805	Gastropod
Late Pleistocene	0.126	0.0117	Richards	1962	18974	Mollusca	7805	Gastropod
Late Pleistocene	0.126	0.0117	Richards	1962	18974	Mollusca	7805	Gastropod
Late Pleistocene	0.126	0.0117	Richards	1962	18974	Mollusca	7805	Gastropod
Late Pleistocene	0.126	0.0117	Richards	1962	18974	Mollusca	7805	Gastropod
Late Pleistocene	0.126	0.0117	Richards	1962	18974	Mollusca	7805	Gastropod
Late Pleistocene	0.126	0.0117	Richards	1962	18974	Mollusca	7805	Gastropod
Late Pleistocene	0.126	0.0117	Richards	1962	18974	Mollusca	7805	Gastropod
Late Pleistocene	0.126	0.0117	Richards	1962	18974	Mollusca	7805	Gastropod
Late Pleistocene	0.126	0.0117	Richards	1962	18974	Mollusca	7805	Gastropod
Late Pleistocene	0.126	0.0117	Hendy et a	2008	19909	Mollusca	7805	Gastropod
Gelasian	2.588	1.806	Bailey and	1986	23840	Mollusca	7805	Gastropod
Gelasian	2.588	1.806	Bailey and	1986	23840	Mollusca	7805	Gastropod
Late Pleistocene	0.126	0.0117	Hendy et a	2008	19909	Mollusca	7805	Gastropod
Late Pliocene	3.6	2.588	Hendy et a	2008	19909	Mollusca	7805	Gastropod
Pliocene	5.333	2.588	Hendy et a	2008	19909	Mollusca	7805	Gastropod
Early Pleistocene	2.588	0.781	Hendy et a	2008	19909	Mollusca	7805	Gastropod
Early Pleistocene	2.588	0.781	Hendy et a	2008	19909	Mollusca	7805	Gastropod
Early Pleistocene	2.588	0.781	Hendy et a	2008	19909	Mollusca	7805	Gastropod
Early Pleistocene	2.588	0.781	Hendy et a	2008	19909	Mollusca	7805	Gastropod
Early Pleistocene	2.588	0.781	Hendy et a	2008	19909	Mollusca	7805	Gastropod
Middle Pleistocene	0.781	0.126	Miller	1985	30803	Mollusca	7805	Gastropod
Middle Pleistocene	0.781	0.126	Miller	1985	30803	Mollusca	7805	Gastropod
Late Pliocene	3.6	2.588	Petuch	1988	31112	Mollusca	7805	Gastropod
Pliocene	5.333	2.588	Ward	2007	48009	Mollusca	7805	Gastropod
Early Pleistocene	2.588	0.781	Miller and	1988	72890	Mollusca	7805	Gastropod
Pleistocene	2.588	0.0117	Coch	1971	441	Mollusca	7805	Gastropod
Pleistocene	2.588	0.0117	Coch	1971	441	Mollusca	7805	Gastropod
Pleistocene	2.588	0.0117	Coch	1971	441	Mollusca	7805	Gastropod
Pleistocene	2.588	0.0117	Coch	1971	441	Mollusca	7805	Gastropod
Late Pleistocene	0.126	0.0117	Spencer ar	1987	15525	Mollusca	7805	Gastropod
Late Pleistocene	0.126	0.0117	Spencer ar	1987	15525	Mollusca	7805	Gastropod
Late Pleistocene	0.126	0.0117	Spencer ar	1987	15525	Mollusca	7805	Gastropod
Late Pleistocene	0.126	0.0117	Spencer ar	1987	15525	Mollusca	7805	Gastropod
Late Pleistocene	0.126	0.0117	Spencer ar	1987	15525	Mollusca	7805	Gastropod
Late Pleistocene	0.126	0.0117	Spencer ar	1987	15525	Mollusca	7805	Gastropod
Late Pleistocene	0.126	0.0117	Spencer ar	1987	15525	Mollusca	7805	Gastropod
Late Pleistocene	0.126	0.0117	Hendy et a	2008	19909	Mollusca	7805	Gastropod
Late Pleistocene	0.126	0.0117	Hendy et a	2008	19909	Mollusca	7805	Gastropod

Late Pliocene		3.6	2.588	Ward	1993	26351	Mollusca	7805 Gastropod
Late Pliocene		3.6	2.588	Ward	1993	26351	Mollusca	7805 Gastropod
Late Pliocene		3.6	2.588	Ward	1993	26351	Mollusca	7805 Gastropod
Late Pliocene		3.6	2.588	Ward	1993	26351	Mollusca	7805 Gastropod
Late Pliocene		3.6	2.588	Ward	1993	26351	Mollusca	7805 Gastropod
Late Pliocene		3.6	2.588	Ward	1993	26351	Mollusca	7805 Gastropod
Late Pliocene		3.6	2.588	Ward	1993	26351	Mollusca	7805 Gastropod
Late Pliocene		3.6	2.588	Ward	1993	26351	Mollusca	7805 Gastropod
Late Pliocene		3.6	2.588	Ward	1993	26351	Mollusca	7805 Gastropod
Late Pliocene		3.6	2.588	Ward	1993	26351	Mollusca	7805 Gastropod
Late Pliocene		3.6	2.588	Ward	1993	26351	Mollusca	7805 Gastropod
Late Pliocene		3.6	2.588	Ward	1993	26351	Mollusca	7805 Gastropod
Late Pliocene		3.6	2.588	Ward	1993	26351	Mollusca	7805 Gastropod
Late Pliocene		3.6	2.588	Ward	1993	26351	Mollusca	7805 Gastropod
Pliocene		5.333	2.588	Petuch	1988	31112	Mollusca	7805 Gastropod
Pliocene		5.333	2.588	Petuch	1988	31112	Mollusca	7805 Gastropod
Pliocene		5.333	2.588	Petuch	1988	31112	Mollusca	7805 Gastropod
Early Pleistocene		2.588	0.781	DuBar	1962	11119	Mollusca	7805 Gastropod
Pliocene	Pleistocene	5.333	0.0117	DuBar	1962	11119	Mollusca	7805 Gastropod
Late Pliocene		3.6	2.588	Mansfield	1932	17094	Mollusca	7805 Gastropod
Late Pliocene		3.6	2.588	Mansfield	1932	17094	Mollusca	7805 Gastropod
Early Pleistocene		2.588	0.781	Schroeder	1954	25303	Mollusca	7805 Gastropod
Early Pleistocene		2.588	0.781	Olsson and	1953	25463	Mollusca	7805 Gastropod
Early Pleistocene		2.588	0.781	Olsson and	1953	25463	Mollusca	7805 Gastropod
Early Pleistocene		2.588	0.781	Olsson and	1953	25463	Mollusca	7805 Gastropod
Late Plioce	Early Pleist	3.6	0.781	Petuch and	2007	26813	Mollusca	7805 Gastropod
Late Pleistocene		0.126	0.0117	Hendy et a	2008	19909	Mollusca	7805 Gastropod
Late Pliocene		3.6	2.588	Petuch and	2007	26813	Mollusca	7805 Gastropod
Late Pliocene		3.6	2.588	Petuch and	2007	26813	Mollusca	7805 Gastropod
Early Pleistocene		2.588	0.781	Hendy et a	2008	19909	Mollusca	7805 Gastropod
Early Pleistocene		2.588	0.781	Hendy et a	2008	19909	Mollusca	7805 Gastropod
Late Pliocene		3.6	2.588	Petuch	1988	31112	Mollusca	7805 Gastropod
Late Pleistocene		0.126	0.0117	Kittle and I	2010	40884	Mollusca	7805 Gastropod
Pliocene		5.333	2.588	Petuch	1986	47440	Mollusca	7805 Gastropod
Early Pleistocene		2.588	0.781	Cooke	1936	31145	Mollusca	7805 Gastropod
Late Pliocene		3.6	2.588	Petuch	1988	31112	Mollusca	7805 Gastropod
Early Pleistocene		2.588	0.781	DuBar	1962	11119	Mollusca	7805 Gastropod
Calabrian		1.806	0.781	DuBar	1962	11119	Mollusca	7805 Gastropod
Early Pleistocene		2.588	0.781	Fargo	1953	27010	Mollusca	7805 Gastropod
Late Pleistocene		0.126	0.0117	Sellards	1917	9011	Mollusca	7805 Gastropod
Early Pleistocene		2.588	0.781	DuBar	1962	11119	Mollusca	7805 Gastropod
Early Pleistocene		2.588	0.781	DuBar	1962	11119	Mollusca	7805 Gastropod
Early Pleistocene		2.588	0.781	DuBar	1962	11119	Mollusca	7805 Gastropod
Pliocene	Pleistocene	5.333	0.0117	DuBar	1962	11119	Mollusca	7805 Gastropod
Late Pliocene		3.6	2.588	Mansfield	1930	17060	Mollusca	7805 Gastropod
Middle Pleistocene		0.781	0.126	Portell et a	1992	17634	Mollusca	7805 Gastropod
Late Pleistocene		0.126	0.0117	Hendy et a	2008	19909	Mollusca	7805 Gastropod

Late Pleistocene		0.126	0.0117	Hendy et a	2008	19909	Mollusca	7805	Gastropod
Late Pleistocene		0.126	0.0117	Hendy et a	2008	19909	Mollusca	7805	Gastropod
Late Pleistocene		0.126	0.0117	Petuch and	2007	26813	Mollusca	7805	Gastropod
Late Pleistocene		0.126	0.0117	Petuch and	2007	26813	Mollusca	7805	Gastropod
Late Plioce	Early Pleist	3.6	0.781	Petuch and	2007	26813	Mollusca	7805	Gastropod
Early Pleistocene		2.588	0.781	Hendy et a	2008	19909	Mollusca	7805	Gastropod
Late Pliocene		3.6	2.588	Petuch and	2007	26813	Mollusca	7805	Gastropod
Late Pleistocene		0.126	0.0117	Hendy et a	2008	19909	Mollusca	7805	Gastropod
Early Pleistocene		2.588	0.781	Hendy et a	2008	19909	Mollusca	7805	Gastropod
Early Pleistocene		2.588	0.781	Hendy et a	2008	19909	Mollusca	7805	Gastropod
Early Pleistocene		2.588	0.781	Olsson and	1953	25463	Mollusca	7805	Gastropod
Early Pleistocene		2.588	0.781	Olsson and	1953	25463	Mollusca	7805	Gastropod
Early Pleistocene		2.588	0.781	Olsson and	1953	25463	Mollusca	7805	Gastropod
Early Pleistocene		2.588	0.781	Olsson and	1953	25463	Mollusca	7805	Gastropod
Late Pleistocene		0.126	0.0117	Petuch	1988	31112	Mollusca	7805	Gastropod
Late Pleistocene		0.126	0.0117	Kittle and I	2010	40884	Mollusca	7805	Gastropod
Late Pleistocene		0.126	0.0117	Kittle and I	2010	40884	Mollusca	7805	Gastropod
Pliocene		5.333	2.588	Petuch	1986	47440	Mollusca	7805	Gastropod
Late Pleistocene		0.126	0.0117	Sellards	1917	9011	Mollusca	7805	Gastropod
Late Pleistocene		0.126	0.0117	Sellards	1917	9011	Mollusca	7805	Gastropod
Late Pleistocene		0.126	0.0117	Sellards	1917	9011	Mollusca	7805	Gastropod
Late Pleistocene		0.126	0.0117	Sellards	1917	9011	Mollusca	7805	Gastropod
Early Pleistocene		2.588	0.781	DuBar	1962	11119	Mollusca	7805	Gastropod
Early Pleistocene		2.588	0.781	DuBar	1962	11119	Mollusca	7805	Gastropod
Early Pleistocene		2.588	0.781	DuBar	1962	11119	Mollusca	7805	Gastropod
Early Pleistocene		2.588	0.781	DuBar	1962	11119	Mollusca	7805	Gastropod
Early Pleistocene		2.588	0.781	DuBar	1962	11119	Mollusca	7805	Gastropod
Early Pleistocene		2.588	0.781	DuBar	1962	11119	Mollusca	7805	Gastropod
Early Pleistocene		2.588	0.781	DuBar	1962	11119	Mollusca	7805	Gastropod
Early Pleistocene		2.588	0.781	DuBar	1962	11119	Mollusca	7805	Gastropod
Early Pleistocene		2.588	0.781	DuBar	1962	11119	Mollusca	7805	Gastropod
Early Pleistocene		2.588	0.781	DuBar	1962	11119	Mollusca	7805	Gastropod
Calabrian		1.806	0.781	DuBar	1962	11119	Mollusca	7805	Gastropod
Calabrian		1.806	0.781	DuBar	1962	11119	Mollusca	7805	Gastropod
Calabrian		1.806	0.781	DuBar	1962	11119	Mollusca	7805	Gastropod
Calabrian		1.806	0.781	DuBar	1962	11119	Mollusca	7805	Gastropod
Early Pleistocene		2.588	0.781	DuBar	1962	11119	Mollusca	7805	Gastropod
Early Pleistocene		2.588	0.781	DuBar	1962	11119	Mollusca	7805	Gastropod
Pliocene	Pleistocene	5.333	0.0117	DuBar	1962	11119	Mollusca	7805	Gastropod
Pliocene	Pleistocene	5.333	0.0117	DuBar	1962	11119	Mollusca	7805	Gastropod
Pliocene	Pleistocene	5.333	0.0117	DuBar	1962	11119	Mollusca	7805	Gastropod
Pliocene	Pleistocene	5.333	0.0117	DuBar	1962	11119	Mollusca	7805	Gastropod
Pliocene	Pleistocene	5.333	0.0117	DuBar	1962	11119	Mollusca	7805	Gastropod
Pliocene	Pleistocene	5.333	0.0117	DuBar	1962	11119	Mollusca	7805	Gastropod
Late Pliocene		3.6	2.588	Mansfield	1930	17060	Mollusca	7805	Gastropod
Late Pliocene		3.6	2.588	Mansfield	1930	17060	Mollusca	7805	Gastropod
Late Pliocene		3.6	2.588	Mansfield	1930	17060	Mollusca	7805	Gastropod

Late Pliocene	3.6	2.588	Mansfield	1930	17060	Mollusca	7805	Gastropod
Early Pleistocene	2.588	0.781	Portell et a	1992	17634	Mollusca	7805	Gastropod
Early Pleistocene	2.588	0.781	Portell et a	1992	17634	Mollusca	7805	Gastropod
Early Pleistocene	2.588	0.781	Portell et a	1992	17634	Mollusca	7805	Gastropod
Early Pleistocene	2.588	0.781	Portell et a	1992	17634	Mollusca	7805	Gastropod
Middle Pleistocene	0.781	0.126	Portell et a	1992	17634	Mollusca	7805	Gastropod
Middle Pleistocene	0.781	0.126	Portell et a	1992	17634	Mollusca	7805	Gastropod
Middle Pleistocene	0.781	0.126	Portell et a	1992	17634	Mollusca	7805	Gastropod
Middle Pleistocene	0.781	0.126	Portell et a	1992	17634	Mollusca	7805	Gastropod
Middle Pleistocene	0.781	0.126	Portell et a	1992	17634	Mollusca	7805	Gastropod
Middle Pleistocene	0.781	0.126	Portell et a	1992	17634	Mollusca	7805	Gastropod
Middle Pleistocene	0.781	0.126	Portell et a	1992	17634	Mollusca	7805	Gastropod
Late Pleistocene	0.126	0.0117	Portell et a	1992	17634	Mollusca	7805	Gastropod
Late Pleistocene	0.126	0.0117	Portell et a	1992	17634	Mollusca	7805	Gastropod
Late Pleistocene	0.126	0.0117	Portell et a	1992	17634	Mollusca	7805	Gastropod
Middle Pleistocene	0.781	0.126	McGinty	1970	19114	Mollusca	7805	Gastropod
Middle Pleistocene	0.781	0.126	McGinty	1970	19114	Mollusca	7805	Gastropod
Middle Pleistocene	0.781	0.126	McGinty	1970	19114	Mollusca	7805	Gastropod
Middle Pleistocene	0.781	0.126	McGinty	1970	19114	Mollusca	7805	Gastropod
Late Pleistocene	0.126	0.0117	Hendy et a	2008	19909	Mollusca	7805	Gastropod
Late Pleistocene	0.126	0.0117	Hendy et a	2008	19909	Mollusca	7805	Gastropod
Late Pleistocene	0.126	0.0117	Petuch anc	2007	26813	Mollusca	7805	Gastropod
Late Pleistocene	0.126	0.0117	Petuch anc	2007	26813	Mollusca	7805	Gastropod
Middle Pleistocene	0.781	0.126	Petuch anc	2007	26813	Mollusca	7805	Gastropod
Middle Pleistocene	0.781	0.126	Petuch anc	2007	26813	Mollusca	7805	Gastropod
Middle Pleistocene	0.781	0.126	Petuch anc	2007	26813	Mollusca	7805	Gastropod
Middle Pleistocene	0.781	0.126	Petuch anc	2007	26813	Mollusca	7805	Gastropod
Middle Pleistocene	0.781	0.126	Petuch anc	2007	26813	Mollusca	7805	Gastropod
Early Pleistocene	2.588	0.781	Petuch anc	2007	26813	Mollusca	7805	Gastropod
Early Pleistocene	2.588	0.781	Petuch anc	2007	26813	Mollusca	7805	Gastropod
Middle Pleistocene	0.781	0.126	Petuch anc	2007	26813	Mollusca	7805	Gastropod
Late Pliocene	3.6	2.588	Olsson anc	1964	26836	Mollusca	7805	Gastropod
Late Pliocene	3.6	2.588	Olsson anc	1964	26836	Mollusca	7805	Gastropod
Early Pleistocene	2.588	0.781	Petuch anc	2007	26813	Mollusca	7805	Gastropod
Late Plioce Early Pleist	3.6	0.781	Petuch anc	2007	26813	Mollusca	7805	Gastropod
Late Plioce Early Pleist	3.6	0.781	Petuch anc	2007	26813	Mollusca	7805	Gastropod
Late Plioce Early Pleist	3.6	0.781	Petuch anc	2007	26813	Mollusca	7805	Gastropod
Late Plioce Early Pleist	3.6	0.781	Petuch anc	2007	26813	Mollusca	7805	Gastropod
Late Plioce Early Pleist	3.6	0.781	Petuch anc	2007	26813	Mollusca	7805	Gastropod
Early Pleistocene	2.588	0.781	Petuch anc	2007	26813	Mollusca	7805	Gastropod
Early Pleistocene	2.588	0.781	Petuch anc	2007	26813	Mollusca	7805	Gastropod
Late Plioce Early Pleist	3.6	0.781	Petuch anc	2007	26813	Mollusca	7805	Gastropod
Late Plioce Early Pleist	3.6	0.781	Petuch anc	2007	26813	Mollusca	7805	Gastropod
Late Plioce Pleistocen	3.6	0.0117	Petuch anc	2007	26813	Mollusca	7805	Gastropod
Late Plioce Pleistocen	3.6	0.0117	Petuch anc	2007	26813	Mollusca	7805	Gastropod
Late Pliocene	3.6	2.588	Petuch anc	2007	26813	Mollusca	7805	Gastropod
Late Pliocene	3.6	2.588	Petuch anc	2007	26813	Mollusca	7805	Gastropod

Late Pliocene	3.6	2.588	Petuch anc	2007	26813	Mollusca	7805	Gastropod
Late Pliocene	3.6	2.588	Petuch anc	2007	26813	Mollusca	7805	Gastropod
Late Pliocene	3.6	2.588	Petuch anc	2007	26813	Mollusca	7805	Gastropod
Late Pliocene	3.6	2.588	Petuch anc	2007	26813	Mollusca	7805	Gastropod
Late Pliocene	3.6	2.588	Petuch anc	2007	26813	Mollusca	7805	Gastropod
Late Pliocene	3.6	2.588	Petuch anc	2007	26813	Mollusca	7805	Gastropod
Late Pliocene	3.6	2.588	Petuch anc	2007	26813	Mollusca	7805	Gastropod
Early Pleistocene	2.588	0.781	Vermeij an	2004	26879	Mollusca	7805	Gastropod
Early Pleistocene	2.588	0.781	Hendy et a	2008	19909	Mollusca	7805	Gastropod
Early Pleistocene	2.588	0.781	Hendy et a	2008	19909	Mollusca	7805	Gastropod
Late Pleistocene	0.126	0.0117	Hendy et a	2008	19909	Mollusca	7805	Gastropod
Early Pliocene	5.333	3.6	Petuch anc	2007	26813	Mollusca	7805	Gastropod
Early Pliocene	5.333	3.6	Petuch anc	2007	26813	Mollusca	7805	Gastropod
Early Pliocene	5.333	3.6	Petuch anc	2007	26813	Mollusca	7805	Gastropod
Early Pliocene	5.333	3.6	Petuch anc	2007	26813	Mollusca	7805	Gastropod
Late Pliocene	3.6	2.588	Petuch anc	2007	26813	Mollusca	7805	Gastropod
Late Pliocene	3.6	2.588	Petuch anc	2007	26813	Mollusca	7805	Gastropod
Late Pliocene	3.6	2.588	Petuch anc	2007	26813	Mollusca	7805	Gastropod
Late Pliocene	3.6	2.588	Petuch anc	2007	26813	Mollusca	7805	Gastropod
Early Pleistocene	2.588	0.781	Olsson anc	1953	25463	Mollusca	7805	Gastropod
Early Pleistocene	2.588	0.781	Olsson anc	1953	25463	Mollusca	7805	Gastropod
Early Pleistocene	2.588	0.781	Olsson anc	1953	25463	Mollusca	7805	Gastropod
Early Pleistocene	2.588	0.781	Olsson anc	1953	25463	Mollusca	7805	Gastropod
Early Pleistocene	2.588	0.781	Olsson anc	1953	25463	Mollusca	7805	Gastropod
Early Pleistocene	2.588	0.781	Olsson anc	1953	25463	Mollusca	7805	Gastropod
Early Pleistocene	2.588	0.781	Olsson anc	1953	25463	Mollusca	7805	Gastropod
Early Pleistocene	2.588	0.781	Olsson anc	1953	25463	Mollusca	7805	Gastropod
Early Pleistocene	2.588	0.781	Olsson anc	1953	25463	Mollusca	7805	Gastropod
Early Pleistocene	2.588	0.781	Olsson anc	1953	25463	Mollusca	7805	Gastropod
Early Pleistocene	2.588	0.781	Olsson anc	1953	25463	Mollusca	7805	Gastropod
Early Pleistocene	2.588	0.781	Olsson anc	1953	25463	Mollusca	7805	Gastropod
Early Pleistocene	2.588	0.781	Olsson anc	1953	25463	Mollusca	7805	Gastropod
Early Pleistocene	2.588	0.781	Olsson anc	1953	25463	Mollusca	7805	Gastropod
Early Pleistocene	2.588	0.781	Olsson anc	1953	25463	Mollusca	7805	Gastropod
Early Pleistocene	2.588	0.781	Olsson anc	1953	25463	Mollusca	7805	Gastropod
Late Pliocene	3.6	2.588	Petuch anc	2007	26813	Mollusca	7805	Gastropod
Late Pliocene	3.6	2.588	Petuch anc	2007	26813	Mollusca	7805	Gastropod
Late Pliocene	3.6	2.588	Petuch anc	2007	26813	Mollusca	7805	Gastropod
Late Pliocene	3.6	2.588	Petuch anc	2007	26813	Mollusca	7805	Gastropod
Late Pliocene	3.6	2.588	Petuch anc	2007	26813	Mollusca	7805	Gastropod
Late Pliocene	3.6	2.588	Petuch anc	2007	26813	Mollusca	7805	Gastropod
Late Pliocene	3.6	2.588	Petuch anc	2007	26813	Mollusca	7805	Gastropod
Late Pliocene	3.6	2.588	Petuch anc	2007	26813	Mollusca	7805	Gastropod
Late Pliocene	3.6	2.588	Petuch anc	2007	26813	Mollusca	7805	Gastropod
Late Pliocene	3.6	2.588	Petuch anc	2007	26813	Mollusca	7805	Gastropod
Late Pliocene	3.6	2.588	Petuch anc	2007	26813	Mollusca	7805	Gastropod
Late Pliocene	3.6	2.588	Petuch anc	2007	26813	Mollusca	7805	Gastropod

Late Pliocene	3.6	2.588	Petuch and	2007	26813	Mollusca	7805	Gastropod
Late Pliocene	3.6	2.588	Hendy et a	2008	19909	Mollusca	7805	Gastropod
Late Pliocene	3.6	2.588	Hendy et a	2008	19909	Mollusca	7805	Gastropod
Early Pleistocene	2.588	0.781	Hendy et a	2008	19909	Mollusca	7805	Gastropod
Early Pleistocene	2.588	0.781	Hendy et a	2008	19909	Mollusca	7805	Gastropod
Early Pleistocene	2.588	0.781	Hendy et a	2008	19909	Mollusca	7805	Gastropod
Early Pleistocene	2.588	0.781	Hendy et a	2008	19909	Mollusca	7805	Gastropod
Early Pleistocene	2.588	0.781	Hendy et a	2008	19909	Mollusca	7805	Gastropod
Late Pliocene	3.6	2.588	Hendy et a	2008	19909	Mollusca	7805	Gastropod
Middle Pleistocene	0.781	0.126	Hendy et a	2008	19909	Mollusca	7805	Gastropod
Middle Pleistocene	0.781	0.126	Hendy et a	2008	19909	Mollusca	7805	Gastropod
Late Pleistocene	0.126	0.0117	Hendy et a	2008	19909	Mollusca	7805	Gastropod
Late Pleistocene	0.126	0.0117	Hendy et a	2008	19909	Mollusca	7805	Gastropod
Late Pleistocene	0.126	0.0117	Hendy et a	2008	19909	Mollusca	7805	Gastropod
Early Pleistocene	2.588	0.781	Hendy et a	2008	19909	Mollusca	7805	Gastropod
Late Pliocene	3.6	2.588	Weisbord	1974	28823	Mollusca	7805	Gastropod
Late Pliocene	3.6	2.588	Vokes	1965	17604	Mollusca	7805	Gastropod
Early Pleistocene	2.588	0.781	Vokes	1965	17604	Mollusca	7805	Gastropod
Late Pliocene	3.6	2.588	Vokes	1965	17604	Mollusca	7805	Gastropod
Pleistocene	2.588	0.0117	Vokes	1965	17604	Mollusca	7805	Gastropod
Early Pleistocene	2.588	0.781	Vokes	1965	17604	Mollusca	7805	Gastropod
Early Pleistocene	2.588	0.781	Vokes	1965	17604	Mollusca	7805	Gastropod
Late Pliocene	3.6	2.588	Vokes	1965	17604	Mollusca	7805	Gastropod
Late Pliocene	3.6	2.588	Vokes	1965	17604	Mollusca	7805	Gastropod
Early Pleistocene	2.588	0.781	Vokes	1965	17604	Mollusca	7805	Gastropod
Late Pliocene	3.6	2.588	Vokes	1965	17604	Mollusca	7805	Gastropod
Pleistocene	2.588	0.0117	Vokes	1965	17604	Mollusca	7805	Gastropod
Early Pleistocene	2.588	0.781	Vokes	1965	17604	Mollusca	7805	Gastropod
Late Pliocene	3.6	2.588	Vokes	1965	17604	Mollusca	7805	Gastropod
Late Plioce Early Pleist	3.6	0.781	Vokes	1965	17604	Mollusca	7805	Gastropod
Late Pliocene	3.6	2.588	Vokes	1965	17604	Mollusca	7805	Gastropod
Early Pleistocene	2.588	0.781	Vokes	1965	17604	Mollusca	7805	Gastropod
Early Pleistocene	2.588	0.781	Vokes	1965	17604	Mollusca	7805	Gastropod
Early Pleistocene	2.588	0.781	Vokes	1965	17604	Mollusca	7805	Gastropod
Early Pleistocene	2.588	0.781	Vokes	1965	17604	Mollusca	7805	Gastropod
Late Plioce Early Pleist	3.6	0.781	Vokes	1965	17604	Mollusca	7805	Gastropod
Early Pleistocene	2.588	0.781	Vokes	1965	17604	Mollusca	7805	Gastropod
Early Pleistocene	2.588	0.781	Vokes	1965	17604	Mollusca	7805	Gastropod
Early Pleistocene	2.588	0.781	Vokes	1965	17604	Mollusca	7805	Gastropod
Early Pleistocene	2.588	0.781	Vokes	1965	17604	Mollusca	7805	Gastropod
Late Pleistocene	0.126	0.0117	Vokes	1965	17604	Mollusca	7805	Gastropod
Late Pleistocene	0.126	0.0117	Vokes	1965	17604	Mollusca	7805	Gastropod
Late Pleistocene	0.126	0.0117	Vokes	1965	17604	Mollusca	7805	Gastropod
Late Pleistocene	0.126	0.0117	Vokes	1965	17604	Mollusca	7805	Gastropod
Late Pleistocene	0.126	0.0117	Vokes	1963	17605	Mollusca	7805	Gastropod
Early Pleistocene	2.588	0.781	Vokes	1963	17605	Mollusca	7805	Gastropod
Early Pleistocene	2.588	0.781	Vokes	1963	17605	Mollusca	7805	Gastropod

Early Pleistocene	2.588	0.781	Vokes	1963	17605	Mollusca	7805	Gastropod
Late Plioce Early Pleist	3.6	0.781	Vokes	1963	17605	Mollusca	7805	Gastropod
Early Pleistocene	2.588	0.781	Vokes	1963	17605	Mollusca	7805	Gastropod
Early Pleistocene	2.588	0.781	Vokes	1963	17605	Mollusca	7805	Gastropod
Early Pleistocene	2.588	0.781	Vokes	1963	17605	Mollusca	7805	Gastropod
Early Pleistocene	2.588	0.781	Vokes	1963	17605	Mollusca	7805	Gastropod
Pleistocene	2.588	0.0117	Vokes	1963	17605	Mollusca	7805	Gastropod
Pleistocene	2.588	0.0117	Vokes	1963	17605	Mollusca	7805	Gastropod
Pleistocene	2.588	0.0117	Vokes	1963	17605	Mollusca	7805	Gastropod
Early Pleistocene	2.588	0.781	Vokes	1963	17605	Mollusca	7805	Gastropod
Late Pleistocene	0.126	0.0117	Vokes	1963	17605	Mollusca	7805	Gastropod
Late Pliocene	3.6	2.588	Gertman	1969	31231	Mollusca	7805	Gastropod
Late Pliocene	3.6	2.588	Gertman	1969	31231	Mollusca	7805	Gastropod
Late Pliocene	3.6	2.588	Gertman	1969	31231	Mollusca	7805	Gastropod
Late Pliocene	3.6	2.588	Gertman	1969	31231	Mollusca	7805	Gastropod
Late Pliocene	3.6	2.588	Gertman	1969	31231	Mollusca	7805	Gastropod
Late Plioce Early Pleist	3.6	0.781	Gertman	1969	31231	Mollusca	7805	Gastropod
Late Pliocene	3.6	2.588	Gertman	1969	31231	Mollusca	7805	Gastropod
Late Pliocene	3.6	2.588	Gertman	1969	31231	Mollusca	7805	Gastropod
Late Pliocene	3.6	2.588	Gertman	1969	31231	Mollusca	7805	Gastropod
Pleistocene	2.588	0.0117	Gertman	1969	31231	Mollusca	7805	Gastropod
Pleistocene	2.588	0.0117	Gertman	1969	31231	Mollusca	7805	Gastropod
Early Pleistocene	2.588	0.781	Gertman	1969	31231	Mollusca	7805	Gastropod
Late Pliocene	3.6	2.588	Gertman	1969	31231	Mollusca	7805	Gastropod
Late Pliocene	3.6	2.588	Gertman	1969	31231	Mollusca	7805	Gastropod
Late Pliocene	3.6	2.588	Gertman	1969	31231	Mollusca	7805	Gastropod
Late Pliocene	3.6	2.588	Gertman	1969	31231	Mollusca	7805	Gastropod
Early Pleistocene	2.588	0.781	Gertman	1969	31231	Mollusca	7805	Gastropod
Early Pleistocene	2.588	0.781	Gertman	1969	31231	Mollusca	7805	Gastropod
Early Pleistocene	2.588	0.781	Gertman	1969	31231	Mollusca	7805	Gastropod
Early Pleistocene	2.588	0.781	Gertman	1969	31231	Mollusca	7805	Gastropod
Late Plioce Early Pleist	3.6	0.781	Gertman	1969	31231	Mollusca	7805	Gastropod
Early Pleistocene	2.588	0.781	Gertman	1969	31231	Mollusca	7805	Gastropod
Early Pleistocene	2.588	0.781	Gertman	1969	31231	Mollusca	7805	Gastropod
Late Plioce Early Pleist	3.6	0.781	Gertman	1969	31231	Mollusca	7805	Gastropod
Early Pleistocene	2.588	0.781	Gertman	1969	31231	Mollusca	7805	Gastropod
Early Pleistocene	2.588	0.781	Gertman	1969	31231	Mollusca	7805	Gastropod
Early Pleistocene	2.588	0.781	Gertman	1969	31231	Mollusca	7805	Gastropod
Late Pliocene	3.6	2.588	Gertman	1969	31231	Mollusca	7805	Gastropod
Pleistocene	2.588	0.0117	Gertman	1969	31231	Mollusca	7805	Gastropod
Early Pleistocene	2.588	0.781	Gertman	1969	31231	Mollusca	7805	Gastropod
Early Pleistocene	2.588	0.781	Gertman	1969	31231	Mollusca	7805	Gastropod
Late Plioce Early Pleist	3.6	0.781	Gertman	1969	31231	Mollusca	7805	Gastropod
Late Pleistocene	0.126	0.0117	Gertman	1969	31231	Mollusca	7805	Gastropod
Late Pleistocene	0.126	0.0117	Gertman	1969	31231	Mollusca	7805	Gastropod
Late Pliocene	3.6	2.588	Vokes	1970	31194	Mollusca	7805	Gastropod
Late Pliocene	3.6	2.588	Vokes	1970	31194	Mollusca	7805	Gastropod

Late Pliocene	3.6	2.588	Vokes	1970	31194	Mollusca	7805	Gastropod
Late Pliocene	3.6	2.588	Vokes	1970	31194	Mollusca	7805	Gastropod
Late Pliocene	3.6	2.588	Weisbord	1972	31274	Mollusca	7805	Gastropod
Late Pliocene	3.6	2.588	Vokes	1974	31296	Mollusca	7805	Gastropod
Late Pliocene	3.6	2.588	Vokes	1975	31301	Mollusca	7805	Gastropod
Late Pliocene	3.6	2.588	Vokes	1975	31301	Mollusca	7805	Gastropod
Late Pleistocene	0.126	0.0117	Vokes	1975	31301	Mollusca	7805	Gastropod
Early Pleistocene	2.588	0.781	Vokes	1975	31301	Mollusca	7805	Gastropod
Late Pleistocene	0.126	0.0117	Vokes	1975	31301	Mollusca	7805	Gastropod
Late Pleistocene	0.126	0.0117	Vokes	1975	31301	Mollusca	7805	Gastropod
Early Pleistocene	2.588	0.781	Vokes	1975	31301	Mollusca	7805	Gastropod
Early Pleistocene	2.588	0.781	Vokes	1975	31301	Mollusca	7805	Gastropod
Early Pleistocene	2.588	0.781	Vokes	1975	31301	Mollusca	7805	Gastropod
Early Pleistocene	2.588	0.781	Vokes	1975	31301	Mollusca	7805	Gastropod
Late Pleistocene	0.126	0.0117	Vokes	1975	31301	Mollusca	7805	Gastropod
Late Pleistocene	0.126	0.0117	Vokes	1975	31301	Mollusca	7805	Gastropod
Pleistocene	2.588	0.0117	Vokes	1975	31301	Mollusca	7805	Gastropod
Pleistocene	2.588	0.0117	Vokes	1975	31301	Mollusca	7805	Gastropod
Late Pliocene	3.6	2.588	Vokes	1975	31301	Mollusca	7805	Gastropod
Pleistocene	2.588	0.0117	Vokes	1975	31301	Mollusca	7805	Gastropod
Early Pleistocene	2.588	0.781	Vokes	1975	31301	Mollusca	7805	Gastropod
Early Pleistocene	2.588	0.781	Vokes	1975	31301	Mollusca	7805	Gastropod
Pleistocene	2.588	0.0117	Vokes	1975	31301	Mollusca	7805	Gastropod
Early Pleistocene	2.588	0.781	Vokes	1975	31301	Mollusca	7805	Gastropod
Early Pleistocene	2.588	0.781	Vokes	1975	31301	Mollusca	7805	Gastropod
Early Pleistocene	2.588	0.781	Vokes	1975	31301	Mollusca	7805	Gastropod
Early Pleistocene	2.588	0.781	Vokes	1975	31301	Mollusca	7805	Gastropod
Early Pleistocene	2.588	0.781	Vokes	1975	31301	Mollusca	7805	Gastropod
Late Pliocene	3.6	2.588	Vokes	1975	31301	Mollusca	7805	Gastropod
Pleistocene	2.588	0.0117	Vokes	1975	31301	Mollusca	7805	Gastropod
Late Pliocene	3.6	2.588	Vokes	1975	31301	Mollusca	7805	Gastropod
Late Plioce Early Pleist	3.6	0.781	Vokes	1975	31301	Mollusca	7805	Gastropod
Late Pliocene	3.6	2.588	Vokes	1975	31301	Mollusca	7805	Gastropod
Late Plioce Early Pleist	3.6	0.781	Vokes	1975	31301	Mollusca	7805	Gastropod
Late Plioce Early Pleist	3.6	0.781	Vokes	1975	31301	Mollusca	7805	Gastropod
Late Pleistocene	0.126	0.0117	Vokes	1975	31301	Mollusca	7805	Gastropod
Late Pliocene	3.6	2.588	Vokes	1975	31301	Mollusca	7805	Gastropod
Late Pleistocene	0.126	0.0117	Vokes	1975	31301	Mollusca	7805	Gastropod
Late Pleistocene	0.126	0.0117	Vokes	1975	31301	Mollusca	7805	Gastropod
Late Pleistocene	0.126	0.0117	Vokes	1975	31301	Mollusca	7805	Gastropod
Pleistocene	2.588	0.0117	Vokes	1976	31304	Mollusca	7805	Gastropod
Late Pliocene	3.6	2.588	Vokes	1976	31304	Mollusca	7805	Gastropod
Early Pleistocene	2.588	0.781	Vokes	1976	31304	Mollusca	7805	Gastropod
Early Pleistocene	2.588	0.781	Vokes	1976	31304	Mollusca	7805	Gastropod
Pleistocene	2.588	0.0117	Vokes	1976	31304	Mollusca	7805	Gastropod
Late Plioce Early Pleist	3.6	0.781	Vokes	1976	31304	Mollusca	7805	Gastropod
Late Pliocene	3.6	2.588	Vokes	1976	31304	Mollusca	7805	Gastropod

Late Pliocene	3.6	2.588	Vokes	1976	31304	Mollusca	7805	Gastropod
Early Pleistocene	2.588	0.781	Vokes	1976	31304	Mollusca	7805	Gastropod
Early Pleistocene	2.588	0.781	Vokes	1976	31304	Mollusca	7805	Gastropod
Late Plioce Early Pleist	3.6	0.781	Vokes	1976	31304	Mollusca	7805	Gastropod
Early Pleistocene	2.588	0.781	Vokes	1976	31304	Mollusca	7805	Gastropod
Late Pleistocene	0.126	0.0117	Vokes	1976	31304	Mollusca	7805	Gastropod
Early Pleistocene	2.588	0.781	Vokes	1976	31304	Mollusca	7805	Gastropod
Early Pleistocene	2.588	0.781	Vokes	1976	31304	Mollusca	7805	Gastropod
Late Pleistocene	0.126	0.0117	Vokes	1976	31304	Mollusca	7805	Gastropod
Early Pleistocene	2.588	0.781	Vokes	1976	31304	Mollusca	7805	Gastropod
Late Pliocene	3.6	2.588	Vokes	1976	31304	Mollusca	7805	Gastropod
Late Pliocene	3.6	2.588	Vokes	1976	31304	Mollusca	7805	Gastropod
Late Pleistocene	0.126	0.0117	Vokes	1976	31304	Mollusca	7805	Gastropod
Late Pliocene	3.6	2.588	Vokes	1976	31304	Mollusca	7805	Gastropod
Late Pliocene	3.6	2.588	Vokes	1976	31304	Mollusca	7805	Gastropod
Early Pleistocene	2.588	0.781	Vokes	1976	31304	Mollusca	7805	Gastropod
Pleistocene	2.588	0.0117	Vokes	1976	31304	Mollusca	7805	Gastropod
Late Pliocene	3.6	2.588	Vokes	1976	31304	Mollusca	7805	Gastropod
Late Plioce Early Pleist	3.6	0.781	Vokes	1976	31304	Mollusca	7805	Gastropod
Late Pliocene	3.6	2.588	Vokes	1976	31304	Mollusca	7805	Gastropod
Late Pliocene	3.6	2.588	Vokes	1976	31304	Mollusca	7805	Gastropod
Late Pleistocene	0.126	0.0117	Vokes	1976	31304	Mollusca	7805	Gastropod
Late Pliocene	3.6	2.588	Vokes	1976	31304	Mollusca	7805	Gastropod
Late Pliocene	3.6	2.588	Vokes	1976	31304	Mollusca	7805	Gastropod
Late Pliocene	3.6	2.588	Vokes	1976	31304	Mollusca	7805	Gastropod
Late Pliocene	3.6	2.588	Vokes	1976	31304	Mollusca	7805	Gastropod
Late Pleistocene	0.126	0.0117	Vokes	1976	31304	Mollusca	7805	Gastropod
Late Pleistocene	0.126	0.0117	Vokes	1976	31304	Mollusca	7805	Gastropod
Late Pliocene	3.6	2.588	Vokes	1976	31304	Mollusca	7805	Gastropod
Late Pliocene	3.6	2.588	Vokes	1976	31304	Mollusca	7805	Gastropod
Late Pliocene	3.6	2.588	Vokes	1976	31304	Mollusca	7805	Gastropod
Early Pleistocene	2.588	0.781	Vokes	1976	31304	Mollusca	7805	Gastropod
Late Pliocene	3.6	2.588	Vokes	1976	31304	Mollusca	7805	Gastropod
Early Pleistocene	2.588	0.781	Vokes	1976	31304	Mollusca	7805	Gastropod
Early Pleistocene	2.588	0.781	Vokes	1976	31304	Mollusca	7805	Gastropod
Pleistocene	2.588	0.0117	Vokes	1976	31304	Mollusca	7805	Gastropod
Late Pleistocene	0.126	0.0117	Vokes	1976	31304	Mollusca	7805	Gastropod
Late Pleistocene	0.126	0.0117	Vokes	1976	31304	Mollusca	7805	Gastropod
Late Pleistocene	0.126	0.0117	Vokes	1976	31304	Mollusca	7805	Gastropod
Late Pleistocene	0.126	0.0117	Vokes	1976	31304	Mollusca	7805	Gastropod
Late Pleistocene	0.126	0.0117	Vokes	1976	31304	Mollusca	7805	Gastropod
Late Pliocene	3.6	2.588	Vokes	1976	31304	Mollusca	7805	Gastropod
Late Pliocene	3.6	2.588	Vokes	1976	31304	Mollusca	7805	Gastropod
Late Pleistocene	0.126	0.0117	Vokes	1976	31304	Mollusca	7805	Gastropod
Early Pleistocene	2.588	0.781	Vokes	1976	31304	Mollusca	7805	Gastropod
Late Pleistocene	0.126	0.0117	Vokes	1976	31304	Mollusca	7805	Gastropod
Late Pleistocene	0.126	0.0117	Vokes	1976	31304	Mollusca	7805	Gastropod

Late Pliocene	3.6	2.588	Vokes	1976	31304	Mollusca	7805	Gastropod
Late Pliocene	3.6	2.588	Vokes	1976	31304	Mollusca	7805	Gastropod
Late Pliocene	3.6	2.588	Vokes	1968	31169	Mollusca	7805	Gastropod
Late Pliocene	3.6	2.588	Vokes	1968	31169	Mollusca	7805	Gastropod
Late Pleistocene	0.126	0.0117	Vokes	1968	31169	Mollusca	7805	Gastropod
Late Pliocene	3.6	2.588	Vokes	1968	31169	Mollusca	7805	Gastropod
Late Pliocene	3.6	2.588	Vokes	1968	31169	Mollusca	7805	Gastropod
Late Pliocene	3.6	2.588	Vokes	1968	31169	Mollusca	7805	Gastropod
Late Pliocene	3.6	2.588	Vokes	1968	31169	Mollusca	7805	Gastropod
Late Pliocene	3.6	2.588	Vokes	1968	31169	Mollusca	7805	Gastropod
Late Pliocene	3.6	2.588	Vokes	1968	31169	Mollusca	7805	Gastropod
Pleistocene	2.588	0.0117	Vokes	1968	31169	Mollusca	7805	Gastropod
Pleistocene	2.588	0.0117	Vokes	1968	31169	Mollusca	7805	Gastropod
Early Pleistocene	2.588	0.781	Vokes	1968	31169	Mollusca	7805	Gastropod
Early Pleistocene	2.588	0.781	Vokes	1968	31169	Mollusca	7805	Gastropod
Pleistocene	2.588	0.0117	Vokes	1968	31169	Mollusca	7805	Gastropod
Early Pleistocene	2.588	0.781	Vokes	1968	31169	Mollusca	7805	Gastropod
Early Pleistocene	2.588	0.781	Vokes	1968	31169	Mollusca	7805	Gastropod
Early Pleistocene	2.588	0.781	Vokes	1968	31169	Mollusca	7805	Gastropod
Early Pleistocene	2.588	0.781	Vokes	1968	31169	Mollusca	7805	Gastropod
Late Pliocene	3.6	2.588	Vokes	1968	31169	Mollusca	7805	Gastropod
Late Pliocene	3.6	2.588	Vokes	1968	31169	Mollusca	7805	Gastropod
Late Pliocene	3.6	2.588	Vokes	1968	31169	Mollusca	7805	Gastropod
Early Pleistocene	2.588	0.781	Vokes	1968	31169	Mollusca	7805	Gastropod
Pleistocene	2.588	0.0117	Vokes	1968	31169	Mollusca	7805	Gastropod
Late Pliocene	3.6	2.588	Vokes	1968	31169	Mollusca	7805	Gastropod
Late Plioce Early Pleist	3.6	0.781	Vokes	1968	31169	Mollusca	7805	Gastropod
Late Pliocene	3.6	2.588	Vokes	1968	31169	Mollusca	7805	Gastropod
Early Pleistocene	2.588	0.781	Vokes	1968	31169	Mollusca	7805	Gastropod
Late Pleistocene	0.126	0.0117	Vokes	1968	31169	Mollusca	7805	Gastropod
Late Pleistocene	0.126	0.0117	Vokes	1968	31169	Mollusca	7805	Gastropod
Early Pleistocene	2.588	0.781	Vokes	1968	31169	Mollusca	7805	Gastropod
Early Pleistocene	2.588	0.781	Vokes	1968	31169	Mollusca	7805	Gastropod
Late Pleistocene	0.126	0.0117	Vokes	1968	31169	Mollusca	7805	Gastropod
Late Pleistocene	0.126	0.0117	Vokes	1968	31169	Mollusca	7805	Gastropod
Pleistocene	2.588	0.0117	Vokes	1968	31169	Mollusca	7805	Gastropod
Late Pleistocene	0.126	0.0117	Vokes	1968	31169	Mollusca	7805	Gastropod
Late Pleistocene	0.126	0.0117	Vokes	1968	31169	Mollusca	7805	Gastropod
Late Pliocene	3.6	2.588	Vokes	1968	31169	Mollusca	7805	Gastropod
Late Pliocene	3.6	2.588	Vokes	1968	31169	Mollusca	7805	Gastropod
Late Pliocene	3.6	2.588	Vokes	1968	31169	Mollusca	7805	Gastropod
Late Pliocene	3.6	2.588	Vokes	1968	31169	Mollusca	7805	Gastropod
Late Pliocene	3.6	2.588	Vokes	1968	31169	Mollusca	7805	Gastropod
Late Pliocene	3.6	2.588	Vokes	1968	31169	Mollusca	7805	Gastropod
Late Pliocene	3.6	2.588	Vokes	1968	31169	Mollusca	7805	Gastropod
Late Pliocene	3.6	2.588	Vokes	1968	31169	Mollusca	7805	Gastropod
Late Plioce Early Pleist	3.6	0.781	Vokes	1968	31169	Mollusca	7805	Gastropod

Late Pliocene	3.6	2.588	Vokes	1968	31169	Mollusca	7805	Gastropod
Late Pliocene	3.6	2.588	Vokes	1968	31169	Mollusca	7805	Gastropod
Pleistocene	2.588	0.0117	Vokes	1968	31169	Mollusca	7805	Gastropod
Pleistocene	2.588	0.0117	Vokes	1968	31169	Mollusca	7805	Gastropod
Pleistocene	2.588	0.0117	Vokes	1968	31169	Mollusca	7805	Gastropod
Early Pleistocene	2.588	0.781	Vokes	1968	31169	Mollusca	7805	Gastropod
Early Pleistocene	2.588	0.781	Vokes	1968	31169	Mollusca	7805	Gastropod
Early Pleistocene	2.588	0.781	Vokes	1968	31169	Mollusca	7805	Gastropod
Early Pleistocene	2.588	0.781	Vokes	1968	31169	Mollusca	7805	Gastropod
Early Pleistocene	2.588	0.781	Vokes	1968	31169	Mollusca	7805	Gastropod
Early Pleistocene	2.588	0.781	Vokes	1968	31169	Mollusca	7805	Gastropod
Early Pleistocene	2.588	0.781	Vokes	1968	31169	Mollusca	7805	Gastropod
Early Pleistocene	2.588	0.781	Vokes	1968	31169	Mollusca	7805	Gastropod
Early Pleistocene	2.588	0.781	Vokes	1968	31169	Mollusca	7805	Gastropod
Early Pleistocene	2.588	0.781	Vokes	1968	31169	Mollusca	7805	Gastropod
Pleistocene	2.588	0.0117	Vokes	1968	31169	Mollusca	7805	Gastropod
Late Plioce Early Pleist	3.6	0.781	Vokes	1968	31169	Mollusca	7805	Gastropod
Late Pliocene	3.6	2.588	Vokes	1968	31169	Mollusca	7805	Gastropod
Pleistocene	2.588	0.0117	Vokes	1968	31169	Mollusca	7805	Gastropod
Late Pliocene	3.6	2.588	Vokes	1968	31169	Mollusca	7805	Gastropod
Early Pleistocene	2.588	0.781	Vokes	1968	31169	Mollusca	7805	Gastropod
Early Pleistocene	2.588	0.781	Vokes	1968	31169	Mollusca	7805	Gastropod
Late Pliocene	3.6	2.588	Vokes	1968	31169	Mollusca	7805	Gastropod
Early Pleistocene	2.588	0.781	Vokes	1968	31169	Mollusca	7805	Gastropod
Early Pleistocene	2.588	0.781	Vokes	1968	31169	Mollusca	7805	Gastropod
Early Pleistocene	2.588	0.781	Vokes	1968	31169	Mollusca	7805	Gastropod
Late Pliocene	3.6	2.588	Vokes	1968	31169	Mollusca	7805	Gastropod
Late Pliocene	3.6	2.588	Vokes	1968	31169	Mollusca	7805	Gastropod
Early Pleistocene	2.588	0.781	Vokes	1968	31169	Mollusca	7805	Gastropod
Pleistocene	2.588	0.0117	Vokes	1968	31169	Mollusca	7805	Gastropod
Late Plioce Early Pleist	3.6	0.781	Vokes	1968	31169	Mollusca	7805	Gastropod
Late Pliocene	3.6	2.588	Vokes	1968	31169	Mollusca	7805	Gastropod
Late Pliocene	3.6	2.588	Petuch	1988	31112	Mollusca	7805	Gastropod
Late Pliocene	3.6	2.588	Petuch	1988	31112	Mollusca	7805	Gastropod
Late Pliocene	3.6	2.588	Petuch	1988	31112	Mollusca	7805	Gastropod
Late Pliocene	3.6	2.588	Petuch	1988	31112	Mollusca	7805	Gastropod
Late Pliocene	3.6	2.588	Petuch	1988	31112	Mollusca	7805	Gastropod
Late Pliocene	3.6	2.588	Petuch	1988	31112	Mollusca	7805	Gastropod
Late Pliocene	3.6	2.588	Petuch	1988	31112	Mollusca	7805	Gastropod
Late Pliocene	3.6	2.588	Petuch	1988	31112	Mollusca	7805	Gastropod
Early Pleistocene	2.588	0.781	Petuch	1988	31112	Mollusca	7805	Gastropod
Late Pliocene	3.6	2.588	Petuch	1988	31112	Mollusca	7805	Gastropod
Middle Pleistocene	0.781	0.126	Petuch	1988	31112	Mollusca	7805	Gastropod
Middle Pleistocene	0.781	0.126	Petuch	1988	31112	Mollusca	7805	Gastropod
Late Pleistocene	0.126	0.0117	Petuch	1988	31112	Mollusca	7805	Gastropod
Late Pliocene	3.6	2.588	Weisbord	1971	39743	Mollusca	7805	Gastropod
Early Pleistocene	2.588	0.781	Vokes	1998	18707	Mollusca	7805	Gastropod

Early Pleistocene	2.588	0.781	Vokes	1998	18707	Mollusca	7805	Gastropod
Middle Pleistocene	0.781	0.126	Vokes	1998	18707	Mollusca	7805	Gastropod
Middle Pleistocene	0.781	0.126	Vokes	1998	18707	Mollusca	7805	Gastropod
Middle Pleistocene	0.781	0.126	Vokes	1998	18707	Mollusca	7805	Gastropod
Early Pleistocene	2.588	0.781	Vokes	1998	18707	Mollusca	7805	Gastropod
Early Pleistocene	2.588	0.781	Vokes	1998	18707	Mollusca	7805	Gastropod
Late Pliocene	3.6	2.588	Vokes	1998	18707	Mollusca	7805	Gastropod
Late Pleistocene	0.126	0.0117	Kittle and I	2010	40884	Mollusca	7805	Gastropod
Late Pleistocene	0.126	0.0117	Kittle and I	2010	40884	Mollusca	7805	Gastropod
Late Pleistocene	0.126	0.0117	Kittle and I	2010	40884	Mollusca	7805	Gastropod
Late Pleistocene	0.126	0.0117	Kittle and I	2010	40884	Mollusca	7805	Gastropod
Late Pleistocene	0.126	0.0117	Kittle and I	2010	40884	Mollusca	7805	Gastropod
Late Pleistocene	0.126	0.0117	Kittle and I	2010	40884	Mollusca	7805	Gastropod
Late Pleistocene	0.126	0.0117	Kittle and I	2010	40884	Mollusca	7805	Gastropod
Late Pleistocene	0.126	0.0117	Kittle and I	2010	40884	Mollusca	7805	Gastropod
Pliocene	5.333	2.588	Petuch	1986	47440	Mollusca	7805	Gastropod
Pliocene	5.333	2.588	Petuch	1986	47440	Mollusca	7805	Gastropod
Pliocene	5.333	2.588	Petuch	1986	47440	Mollusca	7805	Gastropod
Pliocene	5.333	2.588	Petuch	1986	47440	Mollusca	7805	Gastropod
Pliocene	5.333	2.588	Petuch	1986	47440	Mollusca	7805	Gastropod
Pliocene	5.333	2.588	Petuch	1986	47440	Mollusca	7805	Gastropod
Pliocene	5.333	2.588	Petuch	1986	47440	Mollusca	7805	Gastropod
Pliocene	5.333	2.588	Petuch	1986	47440	Mollusca	7805	Gastropod
Late Pliocene	3.6	2.588	Petuch	2003	58594	Mollusca	7805	Gastropod
Late Pliocene	3.6	2.588	Petuch	2003	58594	Mollusca	7805	Gastropod
Late Pleistocene	0.126	0.0117	Hulbert an	1998	1962	Mollusca	7805	Gastropod
Late Pleistocene	0.126	0.0117	Hulbert an	1998	1962	Mollusca	7805	Gastropod
Late Pleistocene	0.126	0.0117	Richards	1962	18974	Mollusca	7805	Gastropod
Late Pleistocene	0.126	0.0117	DuBar	1971	17556	Mollusca	7805	Gastropod
Late Pleistocene	0.126	0.0117	DuBar	1971	17556	Mollusca	7805	Gastropod
Late Pleistocene	0.126	0.0117	DuBar	1971	17556	Mollusca	7805	Gastropod
Late Pliocene	3.6	2.588	Campbell a	1995	17558	Mollusca	7805	Gastropod
Late Pliocene	3.6	2.588	Campbell a	1995	17558	Mollusca	7805	Gastropod
Zanclean	5.333	3.6	Campbell a	1995	17558	Mollusca	7805	Gastropod
Zanclean	5.333	3.6	Campbell a	1995	17558	Mollusca	7805	Gastropod
Zanclean	5.333	3.6	Campbell a	1995	17558	Mollusca	7805	Gastropod
Late Pleistocene	0.126	0.0117	Richards	1962	18974	Mollusca	7805	Gastropod
Late Pleistocene	0.126	0.0117	Richards	1962	18974	Mollusca	7805	Gastropod
Late Pleistocene	0.126	0.0117	Richards	1962	18974	Mollusca	7805	Gastropod
Late Pleistocene	0.126	0.0117	Richards	1962	18974	Mollusca	7805	Gastropod
Late Pleistocene	0.126	0.0117	Richards	1962	18974	Mollusca	7805	Gastropod
Late Pleistocene	0.126	0.0117	Richards	1962	18974	Mollusca	7805	Gastropod
Late Pleistocene	0.126	0.0117	Richards	1962	18974	Mollusca	7805	Gastropod
Late Pleistocene	0.126	0.0117	Richards	1962	18974	Mollusca	7805	Gastropod
Late Pleistocene	0.126	0.0117	Hendy et a	2008	19909	Mollusca	7805	Gastropod
Late Pleistocene	0.126	0.0117	Hendy et a	2008	19909	Mollusca	7805	Gastropod
Early Pleistocene	2.588	0.781	Cooke	1936	31145	Mollusca	7805	Gastropod

Early Pleistocene	2.588	0.781	Vokes	1965	17604	Mollusca	7805	Gastropod
Early Pleistocene	2.588	0.781	Gertman	1969	31231	Mollusca	7805	Gastropod
Early Pleistocene	2.588	0.781	Vokes	1968	31169	Mollusca	7805	Gastropod
Early Pleistocene	2.588	0.781	Vokes	1968	31169	Mollusca	7805	Gastropod
Early Pleistocene	2.588	0.781	Vokes	1968	31169	Mollusca	7805	Gastropod
Early Pleistocene	2.588	0.781	Vokes	1968	31169	Mollusca	7805	Gastropod
Late Pliocene	3.6	2.588	Ward and I	1987	16700	Mollusca	7805	Gastropod
Early Pleistocene	2.588	0.781	Ward and I	1987	16700	Mollusca	7805	Gastropod
Early Pleistocene	2.588	0.781	Ward and I	1987	16700	Mollusca	7805	Gastropod
Early Pleistocene	2.588	0.781	Gardner	1948	17048	Mollusca	7805	Gastropod
Late Pliocene	3.6	2.588	Gardner	1948	17048	Mollusca	7805	Gastropod
Pleistocene	2.588	0.0117	Gardner	1948	17048	Mollusca	7805	Gastropod
Early Pleistocene	2.588	0.781	Gardner	1948	17048	Mollusca	7805	Gastropod
Early Plioc(Late Plioce	5.333	2.588	Gardner	1948	17048	Mollusca	7805	Gastropod
Late Pliocene	3.6	2.588	Gardner	1948	17048	Mollusca	7805	Gastropod
Late Pliocene	3.6	2.588	Gardner	1948	17048	Mollusca	7805	Gastropod
Early Pleistocene	2.588	0.781	Gardner	1948	17048	Mollusca	7805	Gastropod
Early Pleistocene	2.588	0.781	Gardner	1948	17048	Mollusca	7805	Gastropod
Late Pliocene	3.6	2.588	Gardner	1948	17048	Mollusca	7805	Gastropod
Late Pliocene	3.6	2.588	Gardner	1948	17048	Mollusca	7805	Gastropod
Late Pliocene	3.6	2.588	Gardner	1948	17048	Mollusca	7805	Gastropod
Pleistocene	2.588	0.0117	Gardner	1948	17048	Mollusca	7805	Gastropod
Late Pliocene	3.6	2.588	Gardner	1948	17048	Mollusca	7805	Gastropod
Early Pleistocene	2.588	0.781	Gardner	1948	17048	Mollusca	7805	Gastropod
Pleistocene	2.588	0.0117	Gardner	1948	17048	Mollusca	7805	Gastropod
Late Pliocene	3.6	2.588	Gardner	1948	17048	Mollusca	7805	Gastropod
Early Pleistocene	2.588	0.781	Gardner	1948	17048	Mollusca	7805	Gastropod
Late Pliocene	3.6	2.588	Gardner	1948	17048	Mollusca	7805	Gastropod
Early Pleistocene	2.588	0.781	Miller	1990	17561	Mollusca	7805	Gastropod
Early Pleistocene	2.588	0.781	Miller	1990	17561	Mollusca	7805	Gastropod
Early Pleistocene	2.588	0.781	Miller	1990	17561	Mollusca	7805	Gastropod
Early Pleistocene	2.588	0.781	Miller	1990	17561	Mollusca	7805	Gastropod
Late Pleistocene	0.126	0.0117	Richards	1962	18974	Mollusca	7805	Gastropod
Late Pleistocene	0.126	0.0117	Richards	1962	18974	Mollusca	7805	Gastropod
Late Pleistocene	0.126	0.0117	Richards	1962	18974	Mollusca	7805	Gastropod
Late Pleistocene	0.126	0.0117	Richards	1962	18974	Mollusca	7805	Gastropod
Late Pleistocene	0.126	0.0117	Richards	1962	18974	Mollusca	7805	Gastropod
Late Pleistocene	0.126	0.0117	Hendy et a	2008	19909	Mollusca	7805	Gastropod
Late Pleistocene	0.126	0.0117	Hendy et a	2008	19909	Mollusca	7805	Gastropod
Gelasian	2.588	1.806	Bailey and	1986	23840	Mollusca	7805	Gastropod
Gelasian	2.588	1.806	Bailey and	1986	23840	Mollusca	7805	Gastropod
Gelasian	2.588	1.806	Bailey and	1986	23840	Mollusca	7805	Gastropod
Early Pleistocene	2.588	0.781	Hendy et a	2008	19909	Mollusca	7805	Gastropod
Late Pleistocene	0.126	0.0117	Hendy et a	2008	19909	Mollusca	7805	Gastropod
Pliocene	5.333	2.588	Hendy et a	2008	19909	Mollusca	7805	Gastropod
Pliocene	5.333	2.588	Hendy et a	2008	19909	Mollusca	7805	Gastropod
Late Pliocene	3.6	2.588	Hendy et a	2008	19909	Mollusca	7805	Gastropod

Early Pleistocene	2.588	0.781	Hendy et a	2008	19909	Mollusca	7805	Gastropod
Early Pleistocene	2.588	0.781	Vokes	1976	31304	Mollusca	7805	Gastropod
Early Pleistocene	2.588	0.781	Hendy et a	2008	19909	Mollusca	7805	Gastropod
Early Pleistocene	2.588	0.781	Hendy et a	2008	19909	Mollusca	7805	Gastropod
Late Pliocene	3.6	2.588	Gertman	1969	31231	Mollusca	7805	Gastropod
Early Pleistocene	2.588	0.781	Vokes	1976	31304	Mollusca	7805	Gastropod
Early Pleistocene	2.588	0.781	Miller and	1988	72890	Mollusca	7805	Gastropod
Early Pleistocene	2.588	0.781	Miller and	1988	72890	Mollusca	7805	Gastropod
Pleistocene	2.588	0.0117	Coch	1971	441	Mollusca	7805	Gastropod
Pleistocene	2.588	0.0117	Coch	1971	441	Mollusca	7805	Gastropod
Late Pleistocene	0.126	0.0117	Spencer ar	1987	15525	Mollusca	7805	Gastropod
Late Pleistocene	0.126	0.0117	Spencer ar	1987	15525	Mollusca	7805	Gastropod
Late Pleistocene	0.126	0.0117	Spencer ar	1987	15525	Mollusca	7805	Gastropod
Late Pleistocene	0.126	0.0117	Spencer ar	1987	15525	Mollusca	7805	Gastropod
Late Pleistocene	0.126	0.0117	Spencer ar	1987	15525	Mollusca	7805	Gastropod
Late Pleistocene	0.126	0.0117	Spencer ar	1987	15525	Mollusca	7805	Gastropod
Late Pleistocene	0.126	0.0117	Spencer ar	1987	15525	Mollusca	7805	Gastropod
Early Pliocene	5.333	3.6	Ward	1992	16985	Mollusca	7805	Gastropod
Late Pleistocene	0.126	0.0117	Hendy et a	2008	19909	Mollusca	7805	Gastropod
Zanclean	5.333	3.6	Ward	1992	16985	Mollusca	7805	Gastropod
Zanclean	5.333	3.6	Ward	1992	16985	Mollusca	7805	Gastropod
Late Pliocene	3.6	2.588	Ward	1992	16985	Mollusca	7805	Gastropod
Late Pliocene	3.6	2.588	Ward	1993	26351	Mollusca	7805	Gastropod
Late Pliocene	3.6	2.588	Ward	1993	26351	Mollusca	7805	Gastropod
Late Pliocene	3.6	2.588	Ward	1993	26351	Mollusca	7805	Gastropod
Late Pliocene	3.6	2.588	Ward	1993	26351	Mollusca	7805	Gastropod
Late Pliocene	3.6	2.588	Ward	1993	26351	Mollusca	7805	Gastropod
Late Pliocene	3.6	2.588	Ward	1993	26351	Mollusca	7805	Gastropod
Late Pliocene	3.6	2.588	Ward	1993	26351	Mollusca	7805	Gastropod
Late Pliocene	3.6	2.588	Ward	1993	26351	Mollusca	7805	Gastropod
Late Pliocene	3.6	2.588	Ward	1993	26351	Mollusca	7805	Gastropod
Late Pliocene	3.6	2.588	Ward	1993	26351	Mollusca	7805	Gastropod
Late Pliocene	3.6	2.588	Ward	1993	26351	Mollusca	7805	Gastropod
Late Pliocene	3.6	2.588	Ward	1993	26351	Mollusca	7805	Gastropod
Late Pliocene	3.6	2.588	Ward	1993	26351	Mollusca	7805	Gastropod
Late Pliocene	3.6	2.588	Ward	1993	26351	Mollusca	7805	Gastropod
Late Pliocene	3.6	2.588	Hendy et a	2008	19909	Mollusca	7805	Gastropod
Late Pliocene	3.6	2.588	Hendy et a	2008	19909	Mollusca	7805	Gastropod
Late Pliocene	3.6	2.588	Hendy et a	2008	19909	Mollusca	7805	Gastropod
Late Pliocene	3.6	2.588	Hendy et a	2008	19909	Mollusca	7805	Gastropod
Late Pliocene	3.6	2.588	Hendy et a	2008	19909	Mollusca	7805	Gastropod
Late Pliocene	3.6	2.588	Hendy et a	2008	19909	Mollusca	7805	Gastropod
Late Pliocene	3.6	2.588	Hendy et a	2008	19909	Mollusca	7805	Gastropod
Late Pliocene	3.6	2.588	Hendy et a	2008	19909	Mollusca	7805	Gastropod
Late Pliocene	3.6	2.588	Hendy et a	2008	19909	Mollusca	7805	Gastropod
Late Pleistocene	0.126	0.0117	Hendy et a	2008	19909	Mollusca	7805	Gastropod
Late Pleistocene	0.126	0.0117	Hendy et a	2008	19909	Mollusca	7805	Gastropod

Pliocene		5.333	2.588	Petuch	1988	31112	Mollusca	7805	Gastropod
Pliocene		5.333	2.588	Petuch	1988	31112	Mollusca	7805	Gastropod
Pliocene		5.333	2.588	Petuch	1988	31112	Mollusca	7805	Gastropod
Late Pleistocene		0.126	0.0117	Sellards	1917	9011	Mollusca	7805	Gastropod
Early Pleistocene		2.588	0.781	DuBar	1962	11119	Mollusca	7805	Gastropod
Early Pleistocene		2.588	0.781	DuBar	1962	11119	Mollusca	7805	Gastropod
Early Pleistocene		2.588	0.781	DuBar	1962	11119	Mollusca	7805	Gastropod
Early Pleistocene		2.588	0.781	DuBar	1962	11119	Mollusca	7805	Gastropod
Early Pleistocene		2.588	0.781	DuBar	1962	11119	Mollusca	7805	Gastropod
Early Pleistocene		2.588	0.781	DuBar	1962	11119	Mollusca	7805	Gastropod
Early Pleistocene		2.588	0.781	DuBar	1962	11119	Mollusca	7805	Gastropod
Calabrian		1.806	0.781	DuBar	1962	11119	Mollusca	7805	Gastropod
Calabrian		1.806	0.781	DuBar	1962	11119	Mollusca	7805	Gastropod
Calabrian		1.806	0.781	DuBar	1962	11119	Mollusca	7805	Gastropod
Calabrian		1.806	0.781	DuBar	1962	11119	Mollusca	7805	Gastropod
Calabrian		1.806	0.781	DuBar	1962	11119	Mollusca	7805	Gastropod
Early Pleistocene		2.588	0.781	DuBar	1962	11119	Mollusca	7805	Gastropod
Early Pleistocene		2.588	0.781	DuBar	1962	11119	Mollusca	7805	Gastropod
Late Pleistocene		0.126	0.0117	DuBar	1962	11119	Mollusca	7805	Gastropod
Late Pleistocene		0.126	0.0117	DuBar	1962	11119	Mollusca	7805	Gastropod
Late Pleistocene		0.126	0.0117	DuBar	1962	11119	Mollusca	7805	Gastropod
Pliocene	Pleistocene	5.333	0.0117	Landau and	2010	36423	Mollusca	7805	Gastropod
Pliocene	Pleistocene	5.333	0.0117	DuBar	1962	11119	Mollusca	7805	Gastropod
Late Pliocene		3.6	2.588	Landau and	2010	36423	Mollusca	7805	Gastropod
Late Pliocene		3.6	2.588	Mansfield	1930	17060	Mollusca	7805	Gastropod
Late Pliocene		3.6	2.588	Mansfield	1930	17060	Mollusca	7805	Gastropod
Late Pliocene		3.6	2.588	Mansfield	1930	17060	Mollusca	7805	Gastropod
Late Pliocene		3.6	2.588	Mansfield	1930	17060	Mollusca	7805	Gastropod
Late Pliocene		3.6	2.588	Mansfield	1930	17060	Mollusca	7805	Gastropod
Late Pliocene		3.6	2.588	Mansfield	1930	17060	Mollusca	7805	Gastropod
Late Pliocene		3.6	2.588	Mansfield	1930	17060	Mollusca	7805	Gastropod
Late Pliocene		3.6	2.588	Mansfield	1930	17060	Mollusca	7805	Gastropod
Late Pliocene		3.6	2.588	Landau and	2010	36423	Mollusca	7805	Gastropod
Early Pleistocene		2.588	0.781	Portell et a	1992	17634	Mollusca	7805	Gastropod
Early Pleistocene		2.588	0.781	Portell et a	1992	17634	Mollusca	7805	Gastropod
Middle Pleistocene		0.781	0.126	Portell et a	1992	17634	Mollusca	7805	Gastropod
Middle Pleistocene		0.781	0.126	Portell et a	1992	17634	Mollusca	7805	Gastropod
Middle Pleistocene		0.781	0.126	Portell et a	1992	17634	Mollusca	7805	Gastropod
Late Pleistocene		0.126	0.0117	Portell et a	1992	17634	Mollusca	7805	Gastropod
Late Pleistocene		0.126	0.0117	Portell et a	1992	17634	Mollusca	7805	Gastropod
Late Pliocene		3.6	2.588	Landau and	2010	36423	Mollusca	7805	Gastropod
Late Pleistocene		0.126	0.0117	Hendy et a	2008	19909	Mollusca	7805	Gastropod
Early Pleistocene		2.588	0.781	Olsson and	1953	25463	Mollusca	7805	Gastropod
Early Pleistocene		2.588	0.781	Olsson and	1953	25463	Mollusca	7805	Gastropod
Early Pleistocene		2.588	0.781	Olsson and	1953	25463	Mollusca	7805	Gastropod
Early Pleistocene		2.588	0.781	Olsson and	1953	25463	Mollusca	7805	Gastropod
Early Pleistocene		2.588	0.781	Olsson and	1953	25463	Mollusca	7805	Gastropod

Early Pleistocene	2.588	0.781	Olsson and	1953	25463	Mollusca	7805	Gastropod
Early Pleistocene	2.588	0.781	Olsson and	1953	25463	Mollusca	7805	Gastropod
Early Pleistocene	2.588	0.781	Olsson and	1953	25463	Mollusca	7805	Gastropod
Late Pleistocene	0.126	0.0117	Petuch and	2007	26813	Mollusca	7805	Gastropod
Late Pliocene	3.6	2.588	Olsson	1967	19826	Mollusca	7805	Gastropod
Late Pliocene	3.6	2.588	Olsson	1967	19826	Mollusca	7805	Gastropod
Late Pliocene	3.6	2.588	Olsson	1967	19826	Mollusca	7805	Gastropod
Late Pliocene	3.6	2.588	Olsson	1967	19826	Mollusca	7805	Gastropod
Late Pliocene	3.6	2.588	Landau and	2010	36423	Mollusca	7805	Gastropod
Late Plioce Early Pleist	3.6	0.781	Petuch and	2007	26813	Mollusca	7805	Gastropod
Late Plioce Early Pleist	3.6	0.781	Petuch and	2007	26813	Mollusca	7805	Gastropod
Late Plioce Early Pleist	3.6	0.781	Petuch and	2007	26813	Mollusca	7805	Gastropod
Late Pliocene	3.6	2.588	Petuch and	2007	26813	Mollusca	7805	Gastropod
Late Pliocene	3.6	2.588	Petuch and	2007	26813	Mollusca	7805	Gastropod
Early Pleistocene	2.588	0.781	Hendy et a	2008	19909	Mollusca	7805	Gastropod
Early Pleistocene	2.588	0.781	Hendy et a	2008	19909	Mollusca	7805	Gastropod
Late Pleistocene	0.126	0.0117	Hendy et a	2008	19909	Mollusca	7805	Gastropod
Early Pleistocene	2.588	0.781	DuBar	1958	25514	Mollusca	7805	Gastropod
Late Pleistocene	0.126	0.0117	DuBar	1958	25514	Mollusca	7805	Gastropod
Late Pleistocene	0.126	0.0117	DuBar	1958	25514	Mollusca	7805	Gastropod
Late Pleistocene	0.126	0.0117	Hendy et a	2008	19909	Mollusca	7805	Gastropod
Late Pliocene	3.6	2.588	Hendy et a	2008	19909	Mollusca	7805	Gastropod
Early Pleistocene	2.588	0.781	Hendy et a	2008	19909	Mollusca	7805	Gastropod
Early Pleistocene	2.588	0.781	Hendy et a	2008	19909	Mollusca	7805	Gastropod
Early Pleistocene	2.588	0.781	Hendy et a	2008	19909	Mollusca	7805	Gastropod
Early Pleistocene	2.588	0.781	Hendy et a	2008	19909	Mollusca	7805	Gastropod
Early Pleistocene	2.588	0.781	Hendy et a	2008	19909	Mollusca	7805	Gastropod
Late Pliocene	3.6	2.588	Hendy et a	2008	19909	Mollusca	7805	Gastropod
Late Pleistocene	0.126	0.0117	Hendy et a	2008	19909	Mollusca	7805	Gastropod
Early Pleistocene	2.588	0.781	DuBar	1958	25514	Mollusca	7805	Gastropod
Early Pleistocene	2.588	0.781	DuBar	1958	25514	Mollusca	7805	Gastropod
Late Pliocene	3.6	2.588	Vokes	1969	31166	Mollusca	7805	Gastropod
Late Plioce Early Pleist	3.6	0.781	Vokes	1969	31166	Mollusca	7805	Gastropod
Late Pliocene	3.6	2.588	Vokes	1969	31166	Mollusca	7805	Gastropod
Late Pliocene	3.6	2.588	Vokes	1969	31166	Mollusca	7805	Gastropod
Late Pliocene	3.6	2.588	Vokes	1969	31166	Mollusca	7805	Gastropod
Late Pliocene	3.6	2.588	Vokes	1969	31166	Mollusca	7805	Gastropod
Late Pliocene	3.6	2.588	Vokes	1969	31166	Mollusca	7805	Gastropod
Late Pliocene	3.6	2.588	Petuch	1988	31112	Mollusca	7805	Gastropod
Late Pliocene	3.6	2.588	Petuch	1988	31112	Mollusca	7805	Gastropod
Late Pliocene	3.6	2.588	Petuch	1988	31112	Mollusca	7805	Gastropod
Early Pleistocene	2.588	0.781	Petuch	1988	31112	Mollusca	7805	Gastropod
Late Pleistocene	0.126	0.0117	Kittle and I	2010	40884	Mollusca	7805	Gastropod
Late Pleistocene	0.126	0.0117	Kittle and I	2010	40884	Mollusca	7805	Gastropod
Late Pleistocene	0.126	0.0117	Kittle and I	2010	40884	Mollusca	7805	Gastropod
Late Pleistocene	0.126	0.0117	Kittle and I	2010	40884	Mollusca	7805	Gastropod
Pliocene	5.333	2.588	Petuch	1986	47440	Mollusca	7805	Gastropod

Late Pleistocene	0.126	0.0117	Richards	1962	18974	Mollusca	7805	Gastropod
Zanclean	5.333	3.6	Campbell a	1995	17558	Mollusca	7805	Gastropod
Early Pleistocene	2.588	0.781	DuBar	1971	17556	Mollusca	7805	Gastropod
Late Pleistocene	0.126	0.0117	Richards	1962	18974	Mollusca	7805	Gastropod
Late Pleistocene	0.126	0.0117	Richards	1962	18974	Mollusca	7805	Gastropod
Late Pleistocene	0.126	0.0117	Richards	1962	18974	Mollusca	7805	Gastropod
Late Pleistocene	0.126	0.0117	Richards	1962	18974	Mollusca	7805	Gastropod
Late Pleistocene	0.126	0.0117	Richards	1962	18974	Mollusca	7805	Gastropod
Late Pleistocene	0.126	0.0117	Richards	1962	18974	Mollusca	7805	Gastropod
Late Pleistocene	0.126	0.0117	Richards	1962	18974	Mollusca	7805	Gastropod
Late Pleistocene	0.126	0.0117	Richards	1962	18974	Mollusca	7805	Gastropod
Late Pleistocene	0.126	0.0117	Richards	1962	18974	Mollusca	7805	Gastropod
Late Pleistocene	0.126	0.0117	Richards	1962	18974	Mollusca	7805	Gastropod
Late Pleistocene	0.126	0.0117	Hendy et a	2008	19909	Mollusca	7805	Gastropod
Late Pleistocene	0.126	0.0117	Hendy et a	2008	19909	Mollusca	7805	Gastropod
Late Pleistocene	0.126	0.0117	Hendy et a	2008	19909	Mollusca	7805	Gastropod
Pleistocene	2.588	0.0117	Hendy et a	2008	19909	Mollusca	7805	Gastropod
Early Pleistocene	2.588	0.781	Cooke	1936	31145	Mollusca	7805	Gastropod
Late Pliocene	3.6	2.588	Ward and I	1987	16700	Mollusca	7805	Gastropod
Late Pliocene	3.6	2.588	Ward and I	1987	16700	Mollusca	7805	Gastropod
Early Pleistocene	2.588	0.781	Ward and I	1987	16700	Mollusca	7805	Gastropod
Early Pleistocene	2.588	0.781	Ward and I	1987	16700	Mollusca	7805	Gastropod
Late Pliocene	3.6	2.588	Gardner	1948	17048	Mollusca	7805	Gastropod
Late Pliocene	3.6	2.588	Gardner	1948	17048	Mollusca	7805	Gastropod
Early Pleistocene	2.588	0.781	Gardner	1948	17048	Mollusca	7805	Gastropod
Late Pliocene	3.6	2.588	Gardner	1948	17048	Mollusca	7805	Gastropod
Pleistocene	2.588	0.0117	Gardner	1948	17048	Mollusca	7805	Gastropod
Late Pliocene	3.6	2.588	Gardner	1948	17048	Mollusca	7805	Gastropod
Pleistocene	2.588	0.0117	Gardner	1948	17048	Mollusca	7805	Gastropod
Early Pleistocene	2.588	0.781	Gardner	1948	17048	Mollusca	7805	Gastropod
Early Pleistocene	2.588	0.781	Gardner	1948	17048	Mollusca	7805	Gastropod
Early Plioce Late Plioce	5.333	2.588	Gardner	1948	17048	Mollusca	7805	Gastropod
Early Pleistocene	2.588	0.781	Gardner	1948	17048	Mollusca	7805	Gastropod
Early Pleistocene	2.588	0.781	Gardner	1948	17048	Mollusca	7805	Gastropod
Late Pliocene	3.6	2.588	Gardner	1948	17048	Mollusca	7805	Gastropod
Pleistocene	2.588	0.0117	Gardner	1948	17048	Mollusca	7805	Gastropod
Late Pliocene	3.6	2.588	Gardner	1948	17048	Mollusca	7805	Gastropod
Early Pleistocene	2.588	0.781	Gardner	1948	17048	Mollusca	7805	Gastropod
Early Plioce Late Plioce	5.333	2.588	Gardner	1948	17048	Mollusca	7805	Gastropod
Late Pliocene	3.6	2.588	Gardner	1948	17048	Mollusca	7805	Gastropod
Late Pliocene	3.6	2.588	Gardner	1948	17048	Mollusca	7805	Gastropod
Late Pliocene	3.6	2.588	Gardner	1948	17048	Mollusca	7805	Gastropod
Late Pliocene	3.6	2.588	Gardner	1948	17048	Mollusca	7805	Gastropod
Early Pleistocene	2.588	0.781	Gardner	1948	17048	Mollusca	7805	Gastropod
Late Pliocene	3.6	2.588	Gardner	1948	17048	Mollusca	7805	Gastropod
Pleistocene	2.588	0.0117	Gardner	1948	17048	Mollusca	7805	Gastropod
Early Pleistocene	2.588	0.781	Gardner	1948	17048	Mollusca	7805	Gastropod

Pleistocene	2.588	0.0117	Gardner	1948	17048	Mollusca	7805	Gastropod
Late Pliocene	3.6	2.588	Gardner	1948	17048	Mollusca	7805	Gastropod
Late Pliocene	3.6	2.588	Gardner	1948	17048	Mollusca	7805	Gastropod
Late Pliocene	3.6	2.588	Gardner	1948	17048	Mollusca	7805	Gastropod
Late Pliocene	3.6	2.588	Gardner	1948	17048	Mollusca	7805	Gastropod
Early Pleistocene	2.588	0.781	Gardner	1948	17048	Mollusca	7805	Gastropod
Early Pleistocene	2.588	0.781	Gardner	1948	17048	Mollusca	7805	Gastropod
Pleistocene	2.588	0.0117	Gardner	1948	17048	Mollusca	7805	Gastropod
Late Pliocene	3.6	2.588	Gardner	1948	17048	Mollusca	7805	Gastropod
Late Pliocene	3.6	2.588	Gardner	1948	17048	Mollusca	7805	Gastropod
Late Pliocene	3.6	2.588	Gardner	1948	17048	Mollusca	7805	Gastropod
Late Pliocene	3.6	2.588	Gardner	1948	17048	Mollusca	7805	Gastropod
Late Pliocene	3.6	2.588	Gardner	1948	17048	Mollusca	7805	Gastropod
Early Pleistocene	2.588	0.781	Gardner	1948	17048	Mollusca	7805	Gastropod
Late Pliocene	3.6	2.588	Gardner	1948	17048	Mollusca	7805	Gastropod
Late Pliocene	3.6	2.588	Gardner	1948	17048	Mollusca	7805	Gastropod
Pleistocene	2.588	0.0117	Gardner	1948	17048	Mollusca	7805	Gastropod
Early Pleistocene	2.588	0.781	Gardner	1948	17048	Mollusca	7805	Gastropod
Early Pleistocene	2.588	0.781	Gardner	1948	17048	Mollusca	7805	Gastropod
Late Pliocene	3.6	2.588	Gardner	1948	17048	Mollusca	7805	Gastropod
Late Pliocene	3.6	2.588	Gardner	1948	17048	Mollusca	7805	Gastropod
Early Pleistocene	2.588	0.781	Gardner	1948	17048	Mollusca	7805	Gastropod
Pleistocene	2.588	0.0117	Gardner	1948	17048	Mollusca	7805	Gastropod
Early Pleistocene	2.588	0.781	Gardner	1948	17048	Mollusca	7805	Gastropod
Late Pliocene	3.6	2.588	Gardner	1948	17048	Mollusca	7805	Gastropod
Late Pliocene	3.6	2.588	Gardner	1948	17048	Mollusca	7805	Gastropod
Early Pleistocene	2.588	0.781	DuBar	1971	17556	Mollusca	7805	Gastropod
Early Pleistocene	2.588	0.781	Miller	1990	17561	Mollusca	7805	Gastropod
Early Pleistocene	2.588	0.781	Miller	1990	17561	Mollusca	7805	Gastropod
Early Pleistocene	2.588	0.781	Miller	1990	17561	Mollusca	7805	Gastropod
Early Pleistocene	2.588	0.781	Miller	1990	17561	Mollusca	7805	Gastropod
Late Pleistocene	0.126	0.0117	Richards	1962	18974	Mollusca	7805	Gastropod
Late Pleistocene	0.126	0.0117	Richards	1962	18974	Mollusca	7805	Gastropod
Late Pleistocene	0.126	0.0117	Richards	1962	18974	Mollusca	7805	Gastropod
Late Pleistocene	0.126	0.0117	Richards	1962	18974	Mollusca	7805	Gastropod
Late Pleistocene	0.126	0.0117	Richards	1962	18974	Mollusca	7805	Gastropod
Late Pleistocene	0.126	0.0117	Richards	1962	18974	Mollusca	7805	Gastropod
Late Pleistocene	0.126	0.0117	Richards	1962	18974	Mollusca	7805	Gastropod
Late Pleistocene	0.126	0.0117	Richards	1962	18974	Mollusca	7805	Gastropod
Late Pleistocene	0.126	0.0117	Richards	1962	18974	Mollusca	7805	Gastropod
Late Pleistocene	0.126	0.0117	Richards	1962	18974	Mollusca	7805	Gastropod
Late Pleistocene	0.126	0.0117	Richards	1962	18974	Mollusca	7805	Gastropod
Late Pleistocene	0.126	0.0117	Richards	1962	18974	Mollusca	7805	Gastropod
Late Pleistocene	0.126	0.0117	Richards	1962	18974	Mollusca	7805	Gastropod
Late Pleistocene	0.126	0.0117	Richards	1962	18974	Mollusca	7805	Gastropod
Late Pleistocene	0.126	0.0117	Richards	1962	18974	Mollusca	7805	Gastropod
Late Pleistocene	0.126	0.0117	Richards	1962	18974	Mollusca	7805	Gastropod

Late Pleistocene	0.126	0.0117	Richards	1962	18974	Mollusca	7805	Gastropod
Late Pleistocene	0.126	0.0117	Richards	1962	18974	Mollusca	7805	Gastropod
Late Pleistocene	0.126	0.0117	Richards	1962	18974	Mollusca	7805	Gastropod
Late Pleistocene	0.126	0.0117	Richards	1962	18974	Mollusca	7805	Gastropod
Late Pleistocene	0.126	0.0117	Richards	1962	18974	Mollusca	7805	Gastropod
Late Pleistocene	0.126	0.0117	Richards	1962	18974	Mollusca	7805	Gastropod
Late Pleistocene	0.126	0.0117	Richards	1962	18974	Mollusca	7805	Gastropod
Late Pleistocene	0.126	0.0117	Hendy et a	2008	19909	Mollusca	7805	Gastropod
Late Pleistocene	0.126	0.0117	Hendy et a	2008	19909	Mollusca	7805	Gastropod
Gelasian	2.588	1.806	Bailey and	1986	23840	Mollusca	7805	Gastropod
Gelasian	2.588	1.806	Bailey and	1986	23840	Mollusca	7805	Gastropod
Gelasian	2.588	1.806	Bailey and	1986	23840	Mollusca	7805	Gastropod
Late Pleistocene	0.126	0.0117	Hendy et a	2008	19909	Mollusca	7805	Gastropod
Late Pleistocene	0.126	0.0117	Hendy et a	2008	19909	Mollusca	7805	Gastropod
Late Pliocene	3.6	2.588	Hendy et a	2008	19909	Mollusca	7805	Gastropod
Early Pleistocene	2.588	0.781	Hendy et a	2008	19909	Mollusca	7805	Gastropod
Late Pliocene	3.6	2.588	Hendy et a	2008	19909	Mollusca	7805	Gastropod
Early Pleistocene	2.588	0.781	Hendy et a	2008	19909	Mollusca	7805	Gastropod
Early Pleistocene	2.588	0.781	Hendy et a	2008	19909	Mollusca	7805	Gastropod
Early Pleistocene	2.588	0.781	Hendy et a	2008	19909	Mollusca	7805	Gastropod
Early Pleistocene	2.588	0.781	Hendy et a	2008	19909	Mollusca	7805	Gastropod
Early Pleistocene	2.588	0.781	Hendy et a	2008	19909	Mollusca	7805	Gastropod
Early Pleistocene	2.588	0.781	Hendy et a	2008	19909	Mollusca	7805	Gastropod
Early Pleistocene	2.588	0.781	Hendy et a	2008	19909	Mollusca	7805	Gastropod
Early Pleistocene	2.588	0.781	Hendy et a	2008	19909	Mollusca	7805	Gastropod
Early Pleistocene	2.588	0.781	Hendy et a	2008	19909	Mollusca	7805	Gastropod
Early Pleistocene	2.588	0.781	Hendy et a	2008	19909	Mollusca	7805	Gastropod
Late Pliocene	3.6	2.588	Vokes	1969	31166	Mollusca	7805	Gastropod
Early Pleistocene	2.588	0.781	Miller and	1988	72890	Mollusca	7805	Gastropod
Early Pleistocene	2.588	0.781	Miller and	1988	72890	Mollusca	7805	Gastropod
Early Pleistocene	2.588	0.781	Miller and	1988	72890	Mollusca	7805	Gastropod
Early Pleistocene	2.588	0.781	Miller and	1988	72890	Mollusca	7805	Gastropod
Pleistocene	2.588	0.0117	Coch	1971	441	Mollusca	7805	Gastropod
Pleistocene	2.588	0.0117	Coch	1971	441	Mollusca	7805	Gastropod
Pleistocene	2.588	0.0117	Coch	1971	441	Mollusca	7805	Gastropod
Late Pleistocene	0.126	0.0117	Spencer ar	1987	15525	Mollusca	7805	Gastropod
Late Pleistocene	0.126	0.0117	Spencer ar	1987	15525	Mollusca	7805	Gastropod
Late Pliocene	3.6	2.588	Edwards et	2005	17631	Mollusca	7805	Gastropod
Late Pleistocene	0.126	0.0117	Hendy et a	2008	19909	Mollusca	7805	Gastropod
Late Pleistocene	0.126	0.0117	Hendy et a	2008	19909	Mollusca	7805	Gastropod
Late Pleistocene	0.126	0.0117	Hendy et a	2008	19909	Mollusca	7805	Gastropod
Late Pliocene	3.6	2.588	Ward	1993	26351	Mollusca	7805	Gastropod
Late Pliocene	3.6	2.588	Ward	1993	26351	Mollusca	7805	Gastropod
Late Pliocene	3.6	2.588	Ward	1993	26351	Mollusca	7805	Gastropod
Late Pliocene	3.6	2.588	Ward	1993	26351	Mollusca	7805	Gastropod
Late Pliocene	3.6	2.588	Ward	1993	26351	Mollusca	7805	Gastropod
Late Pliocene	3.6	2.588	Ward	1993	26351	Mollusca	7805	Gastropod

Late Pliocene		3.6	2.588	Ward	1993	26351	Mollusca	7805	Gastropod
Late Pliocene		3.6	2.588	Ward	1993	26351	Mollusca	7805	Gastropod
Late Pliocene		3.6	2.588	Ward	1993	26351	Mollusca	7805	Gastropod
Late Pliocene		3.6	2.588	Ward	1993	26351	Mollusca	7805	Gastropod
Late Pliocene		3.6	2.588	Hendy et a	2008	19909	Mollusca	7805	Gastropod
Pliocene		5.333	2.588	Petuch	1988	31112	Mollusca	7805	Gastropod
Pliocene		5.333	2.588	Petuch	1988	31112	Mollusca	7805	Gastropod
Late Pleistocene		0.126	0.0117	Sellards	1917	9011	Mollusca	7805	Gastropod
Late Pleistocene		0.126	0.0117	Sellards	1917	9011	Mollusca	7805	Gastropod
Early Pleistocene		2.588	0.781	DuBar	1962	11119	Mollusca	7805	Gastropod
Early Pleistocene		2.588	0.781	DuBar	1962	11119	Mollusca	7805	Gastropod
Early Pleistocene		2.588	0.781	DuBar	1962	11119	Mollusca	7805	Gastropod
Early Pleistocene		2.588	0.781	DuBar	1962	11119	Mollusca	7805	Gastropod
Early Pleistocene		2.588	0.781	DuBar	1962	11119	Mollusca	7805	Gastropod
Early Pleistocene		2.588	0.781	DuBar	1962	11119	Mollusca	7805	Gastropod
Early Pleistocene		2.588	0.781	DuBar	1962	11119	Mollusca	7805	Gastropod
Early Pleistocene		2.588	0.781	DuBar	1962	11119	Mollusca	7805	Gastropod
Early Pleistocene		2.588	0.781	DuBar	1962	11119	Mollusca	7805	Gastropod
Calabrian		1.806	0.781	DuBar	1962	11119	Mollusca	7805	Gastropod
Calabrian		1.806	0.781	DuBar	1962	11119	Mollusca	7805	Gastropod
Late Pleistocene		0.126	0.0117	DuBar	1962	11119	Mollusca	7805	Gastropod
Late Pleistocene		0.126	0.0117	DuBar	1962	11119	Mollusca	7805	Gastropod
Early Pleistocene		2.588	0.781	DuBar	1962	11119	Mollusca	7805	Gastropod
Late Pleistocene		0.126	0.0117	DuBar	1962	11119	Mollusca	7805	Gastropod
Pliocene	Pleistocene	5.333	0.0117	DuBar	1962	11119	Mollusca	7805	Gastropod
Pliocene	Pleistocene	5.333	0.0117	DuBar	1962	11119	Mollusca	7805	Gastropod
Pliocene	Pleistocene	5.333	0.0117	DuBar	1962	11119	Mollusca	7805	Gastropod
Pliocene	Pleistocene	5.333	0.0117	DuBar	1962	11119	Mollusca	7805	Gastropod
Late Pliocene		3.6	2.588	Mansfield	1930	17060	Mollusca	7805	Gastropod
Late Pliocene		3.6	2.588	Mansfield	1930	17060	Mollusca	7805	Gastropod
Late Pliocene		3.6	2.588	Mansfield	1930	17060	Mollusca	7805	Gastropod
Late Pliocene		3.6	2.588	Mansfield	1930	17060	Mollusca	7805	Gastropod
Late Pliocene		3.6	2.588	Mansfield	1930	17060	Mollusca	7805	Gastropod
Late Pliocene		3.6	2.588	Mansfield	1930	17060	Mollusca	7805	Gastropod
Late Pliocene		3.6	2.588	Mansfield	1930	17060	Mollusca	7805	Gastropod
Late Pliocene		3.6	2.588	Mansfield	1930	17060	Mollusca	7805	Gastropod
Late Pliocene		3.6	2.588	Mansfield	1930	17060	Mollusca	7805	Gastropod
Late Pliocene		3.6	2.588	Mansfield	1930	17060	Mollusca	7805	Gastropod
Late Pliocene		3.6	2.588	Mansfield	1930	17060	Mollusca	7805	Gastropod
Early Pleistocene		2.588	0.781	Portell et a	1992	17634	Mollusca	7805	Gastropod
Early Pleistocene		2.588	0.781	Portell et a	1992	17634	Mollusca	7805	Gastropod
Early Pleistocene		2.588	0.781	Portell et a	1992	17634	Mollusca	7805	Gastropod
Middle Pleistocene		0.781	0.126	Portell et a	1992	17634	Mollusca	7805	Gastropod
Middle Pleistocene		0.781	0.126	Portell et a	1992	17634	Mollusca	7805	Gastropod
Middle Pleistocene		0.781	0.126	Portell et a	1992	17634	Mollusca	7805	Gastropod
Late Pleistocene		0.126	0.0117	Portell et a	1992	17634	Mollusca	7805	Gastropod
Late Pleistocene		0.126	0.0117	Portell et a	1992	17634	Mollusca	7805	Gastropod

Late Pleistocene	0.126	0.0117	Hendy et a	2008	19909	Mollusca	7805	Gastropod
Late Pleistocene	0.126	0.0117	Hendy et a	2008	19909	Mollusca	7805	Gastropod
Late Pleistocene	0.126	0.0117	Hendy et a	2008	19909	Mollusca	7805	Gastropod
Pliocene	5.333	2.588	Schroeder	1954	25303	Mollusca	7805	Gastropod
Early Pleistocene	2.588	0.781	Schroeder	1954	25303	Mollusca	7805	Gastropod
Pliocene	5.333	2.588	Schroeder	1954	25303	Mollusca	7805	Gastropod
Late Plioce Early Pleist	3.6	0.781	Petuch anc	2007	26813	Mollusca	7805	Gastropod
Early Pleistocene	2.588	0.781	Hendy et a	2008	19909	Mollusca	7805	Gastropod
Early Pleistocene	2.588	0.781	Hendy et a	2008	19909	Mollusca	7805	Gastropod
Early Pleistocene	2.588	0.781	Olsson anc	1953	25463	Mollusca	7805	Gastropod
Early Pleistocene	2.588	0.781	Olsson anc	1953	25463	Mollusca	7805	Gastropod
Early Pleistocene	2.588	0.781	Olsson anc	1953	25463	Mollusca	7805	Gastropod
Early Pleistocene	2.588	0.781	Olsson anc	1953	25463	Mollusca	7805	Gastropod
Early Pleistocene	2.588	0.781	Olsson anc	1953	25463	Mollusca	7805	Gastropod
Early Pleistocene	2.588	0.781	Olsson anc	1953	25463	Mollusca	7805	Gastropod
Early Pleistocene	2.588	0.781	Olsson anc	1953	25463	Mollusca	7805	Gastropod
Early Pleistocene	2.588	0.781	Olsson anc	1953	25463	Mollusca	7805	Gastropod
Early Pleistocene	2.588	0.781	Olsson anc	1953	25463	Mollusca	7805	Gastropod
Early Pleistocene	2.588	0.781	Olsson anc	1953	25463	Mollusca	7805	Gastropod
Early Pleistocene	2.588	0.781	Olsson anc	1953	25463	Mollusca	7805	Gastropod
Early Pleistocene	2.588	0.781	Olsson anc	1953	25463	Mollusca	7805	Gastropod
Late Pliocene	3.6	2.588	Petuch anc	2007	26813	Mollusca	7805	Gastropod
Early Pleistocene	2.588	0.781	Hendy et a	2008	19909	Mollusca	7805	Gastropod
Early Pleistocene	2.588	0.781	Hendy et a	2008	19909	Mollusca	7805	Gastropod
Early Pleistocene	2.588	0.781	Hendy et a	2008	19909	Mollusca	7805	Gastropod
Early Pleistocene	2.588	0.781	Hendy et a	2008	19909	Mollusca	7805	Gastropod
Early Pleistocene	2.588	0.781	Hendy et a	2008	19909	Mollusca	7805	Gastropod
Late Pleistocene	0.126	0.0117	Hendy et a	2008	19909	Mollusca	7805	Gastropod
Early Pleistocene	2.588	0.781	DuBar	1958	25514	Mollusca	7805	Gastropod
Late Pleistocene	0.126	0.0117	Kittle and I	2010	40884	Mollusca	7805	Gastropod
Late Pleistocene	0.126	0.0117	Kittle and I	2010	40884	Mollusca	7805	Gastropod
Late Pleistocene	0.126	0.0117	Kittle and I	2010	40884	Mollusca	7805	Gastropod
Late Pleistocene	0.126	0.0117	Kittle and I	2010	40884	Mollusca	7805	Gastropod
Late Pleistocene	0.126	0.0117	Kittle and I	2010	40884	Mollusca	7805	Gastropod
Late Pleistocene	0.126	0.0117	Kittle and I	2010	40884	Mollusca	7805	Gastropod
Late Pliocene	3.6	2.588	Ketcher an	1993	58350	Mollusca	7805	Gastropod
Late Pleistocene	0.126	0.0117	Hulbert an	1998	1962	Mollusca	7805	Gastropod
Late Pleistocene	0.126	0.0117	Hulbert an	1998	1962	Mollusca	7805	Gastropod
Late Pleistocene	0.126	0.0117	Hulbert an	1998	1962	Mollusca	7805	Gastropod
Late Pleistocene	0.126	0.0117	Richards	1962	18974	Mollusca	7805	Gastropod
Pliocene	5.333	2.588	Aldrich	1911	53829	Mollusca	7805	Gastropod
Late Pleistocene	0.126	0.0117	DuBar	1971	17556	Mollusca	7805	Gastropod
Late Pleistocene	0.126	0.0117	DuBar	1971	17556	Mollusca	7805	Gastropod
Late Pleistocene	0.126	0.0117	DuBar	1971	17556	Mollusca	7805	Gastropod
Late Pleistocene	0.126	0.0117	DuBar	1971	17556	Mollusca	7805	Gastropod
Zanclean	5.333	3.6	Campbell a	1995	17558	Mollusca	7805	Gastropod
Zanclean	5.333	3.6	Campbell a	1995	17558	Mollusca	7805	Gastropod

Zanclean	5.333	3.6	Campbell a	1995	17558	Mollusca	7805	Gastropod
Zanclean	5.333	3.6	Campbell a	1995	17558	Mollusca	7805	Gastropod
Early Pleistocene	2.588	0.781	DuBar	1971	17556	Mollusca	7805	Gastropod
Early Pleistocene	2.588	0.781	DuBar	1971	17556	Mollusca	7805	Gastropod
Late Pleistocene	0.126	0.0117	Richards	1962	18974	Mollusca	7805	Gastropod
Late Pleistocene	0.126	0.0117	Richards	1962	18974	Mollusca	7805	Gastropod
Late Pleistocene	0.126	0.0117	Richards	1962	18974	Mollusca	7805	Gastropod
Late Pleistocene	0.126	0.0117	Richards	1962	18974	Mollusca	7805	Gastropod
Late Pleistocene	0.126	0.0117	Richards	1962	18974	Mollusca	7805	Gastropod
Late Pleistocene	0.126	0.0117	Richards	1962	18974	Mollusca	7805	Gastropod
Late Pleistocene	0.126	0.0117	Richards	1962	18974	Mollusca	7805	Gastropod
Late Pleistocene	0.126	0.0117	Richards	1962	18974	Mollusca	7805	Gastropod
Late Pleistocene	0.126	0.0117	Richards	1962	18974	Mollusca	7805	Gastropod
Late Pleistocene	0.126	0.0117	Hendy et a	2008	19909	Mollusca	7805	Gastropod
Late Pleistocene	0.126	0.0117	Hendy et a	2008	19909	Mollusca	7805	Gastropod
Late Pleistocene	0.126	0.0117	Hendy et a	2008	19909	Mollusca	7805	Gastropod
Late Pleistocene	0.126	0.0117	Hendy et a	2008	19909	Mollusca	7805	Gastropod
Late Pleistocene	0.126	0.0117	Hendy et a	2008	19909	Mollusca	7805	Gastropod
Pleistocene	2.588	0.0117	Hendy et a	2008	19909	Mollusca	7805	Gastropod
Early Pleistocene	2.588	0.781	Cooke	1936	31145	Mollusca	7805	Gastropod
Early Pleistocene	2.588	0.781	Cooke	1936	31145	Mollusca	7805	Gastropod
Late Pliocene	3.6	2.588	Ward and	1987	16700	Mollusca	7805	Gastropod
Late Pliocene	3.6	2.588	Ward and	1987	16700	Mollusca	7805	Gastropod
Late Pliocene	3.6	2.588	Ward and	1987	16700	Mollusca	7805	Gastropod
Early Pleistocene	2.588	0.781	Ward and	1987	16700	Mollusca	7805	Gastropod
Early Pleistocene	2.588	0.781	Ward and	1987	16700	Mollusca	7805	Gastropod
Early Pleistocene	2.588	0.781	Miller	1990	17561	Mollusca	7805	Gastropod
Early Pleistocene	2.588	0.781	Miller	1990	17561	Mollusca	7805	Gastropod
Early Pleistocene	2.588	0.781	Miller	1990	17561	Mollusca	7805	Gastropod
Late Pleistocene	0.126	0.0117	Richards	1962	18974	Mollusca	7805	Gastropod
Late Pleistocene	0.126	0.0117	Richards	1962	18974	Mollusca	7805	Gastropod
Late Pleistocene	0.126	0.0117	Richards	1962	18974	Mollusca	7805	Gastropod
Late Pleistocene	0.126	0.0117	Richards	1962	18974	Mollusca	7805	Gastropod
Late Pleistocene	0.126	0.0117	Richards	1962	18974	Mollusca	7805	Gastropod
Late Pleistocene	0.126	0.0117	Richards	1962	18974	Mollusca	7805	Gastropod
Late Pleistocene	0.126	0.0117	Richards	1962	18974	Mollusca	7805	Gastropod
Late Pleistocene	0.126	0.0117	Richards	1962	18974	Mollusca	7805	Gastropod
Late Pleistocene	0.126	0.0117	Richards	1962	18974	Mollusca	7805	Gastropod
Late Pleistocene	0.126	0.0117	Richards	1962	18974	Mollusca	7805	Gastropod
Late Pleistocene	0.126	0.0117	Richards	1962	18974	Mollusca	7805	Gastropod
Late Pliocene	3.6	2.588	Hendy et a	2008	19909	Mollusca	7805	Gastropod
Late Pleistocene	0.126	0.0117	Hendy et a	2008	19909	Mollusca	7805	Gastropod
Late Pleistocene	0.126	0.0117	Hendy et a	2008	19909	Mollusca	7805	Gastropod
Gelasian	2.588	1.806	Bailey and	1986	23840	Mollusca	7805	Gastropod
Gelasian	2.588	1.806	Bailey and	1986	23840	Mollusca	7805	Gastropod
Early Pleistocene	2.588	0.781	Hendy et a	2008	19909	Mollusca	7805	Gastropod
Late Pleistocene	0.126	0.0117	Hendy et a	2008	19909	Mollusca	7805	Gastropod

Pliocene	5.333	2.588	Hendy et a	2008	19909	Mollusca	7805	Gastropod
Late Pliocene	3.6	2.588	Hendy et a	2008	19909	Mollusca	7805	Gastropod
Late Pliocene	3.6	2.588	Hendy et a	2008	19909	Mollusca	7805	Gastropod
Late Pliocene	3.6	2.588	Hendy et a	2008	19909	Mollusca	7805	Gastropod
Late Pliocene	3.6	2.588	Hendy et a	2008	19909	Mollusca	7805	Gastropod
Early Pleistocene	2.588	0.781	Hendy et a	2008	19909	Mollusca	7805	Gastropod
Early Pleistocene	2.588	0.781	Hendy et a	2008	19909	Mollusca	7805	Gastropod
Early Pleistocene	2.588	0.781	Hendy et a	2008	19909	Mollusca	7805	Gastropod
Early Pleistocene	2.588	0.781	Hendy et a	2008	19909	Mollusca	7805	Gastropod
Early Pleistocene	2.588	0.781	Hendy et a	2008	19909	Mollusca	7805	Gastropod
Early Pleistocene	2.588	0.781	Hendy et a	2008	19909	Mollusca	7805	Gastropod
Early Pleistocene	2.588	0.781	Hendy et a	2008	19909	Mollusca	7805	Gastropod
Middle Pleistocene	0.781	0.126	Miller	1985	30803	Mollusca	7805	Gastropod
Middle Pleistocene	0.781	0.126	Miller	1985	30803	Mollusca	7805	Gastropod
Early Pleistocene	2.588	0.781	Miller and	1988	72890	Mollusca	7805	Gastropod
Pleistocene	2.588	0.0117	Coch	1971	441	Mollusca	7805	Gastropod
Pleistocene	2.588	0.0117	Coch	1971	441	Mollusca	7805	Gastropod
Late Pleistocene	0.126	0.0117	Spencer ar	1987	15525	Mollusca	7805	Gastropod
Late Pleistocene	0.126	0.0117	Spencer ar	1987	15525	Mollusca	7805	Gastropod
Late Pliocene	3.6	2.588	Edwards et	2005	17631	Mollusca	7805	Gastropod
Late Pliocene	3.6	2.588	Edwards et	2005	17631	Mollusca	7805	Gastropod
Late Pliocene	3.6	2.588	Edwards et	2005	17631	Mollusca	7805	Gastropod
Late Pleistocene	0.126	0.0117	Hendy et a	2008	19909	Mollusca	7805	Gastropod
Late Pleistocene	0.126	0.0117	Hendy et a	2008	19909	Mollusca	7805	Gastropod
Late Pleistocene	0.126	0.0117	Hendy et a	2008	19909	Mollusca	7805	Gastropod
Late Pliocene	3.6	2.588	Ward	1993	26351	Mollusca	7805	Gastropod
Late Pliocene	3.6	2.588	Ward	1993	26351	Mollusca	7805	Gastropod
Late Pliocene	3.6	2.588	Ward	1993	26351	Mollusca	7805	Gastropod
Late Pliocene	3.6	2.588	Ward	1993	26351	Mollusca	7805	Gastropod
Late Pliocene	3.6	2.588	Ward	1993	26351	Mollusca	7805	Gastropod
Late Pliocene	3.6	2.588	Ward	1993	26351	Mollusca	7805	Gastropod
Late Pliocene	3.6	2.588	Ward	1993	26351	Mollusca	7805	Gastropod
Late Pliocene	3.6	2.588	Ward	1993	26351	Mollusca	7805	Gastropod
Late Pliocene	3.6	2.588	Ward	1993	26351	Mollusca	7805	Gastropod
Late Pliocene	3.6	2.588	Ward	1993	26351	Mollusca	7805	Gastropod
Late Pliocene	3.6	2.588	Ward	1993	26351	Mollusca	7805	Gastropod
Late Pliocene	3.6	2.588	Ward	1993	26351	Mollusca	7805	Gastropod
Late Pliocene	3.6	2.588	Ward	1993	26351	Mollusca	7805	Gastropod
Late Pliocene	3.6	2.588	Ward	1993	26351	Mollusca	7805	Gastropod
Late Pliocene	3.6	2.588	Hendy et a	2008	19909	Mollusca	7805	Gastropod
Late Pliocene	3.6	2.588	Hendy et a	2008	19909	Mollusca	7805	Gastropod
Late Pliocene	3.6	2.588	Hendy et a	2008	19909	Mollusca	7805	Gastropod
Late Pliocene	3.6	2.588	Hendy et a	2008	19909	Mollusca	7805	Gastropod
Late Pliocene	3.6	2.588	Hendy et a	2008	19909	Mollusca	7805	Gastropod
Middle Pleistocene	0.781	0.126	Hoerle	1970	19536	Mollusca	7805	Gastropod
Late Pliocene	3.6	2.588	Petuch anc	2007	26813	Mollusca	7805	Gastropod
Early Pleistocene	2.588	0.781	Olsson anc	1953	25463	Mollusca	7805	Gastropod

Early Pleistocene		2.588	0.781	Olsson anc	1953	25463	Mollusca	7805	Gastropod
Late Pliocene		3.6	2.588	Petuch	1988	31112	Mollusca	7805	Gastropod
Late Pleistocene		0.126	0.0117	Kittle and I	2010	40884	Mollusca	7805	Gastropod
Late Pleistocene		0.126	0.0117	Kittle and I	2010	40884	Mollusca	7805	Gastropod
Late Pleistocene		0.126	0.0117	Kittle and I	2010	40884	Mollusca	7805	Gastropod
Pliocene		5.333	2.588	Aldrich	1911	53829	Mollusca	7805	Gastropod
Gelasian		2.588	1.806	Bailey and	1986	23840	Mollusca	7805	Gastropod
Early Pleistocene		2.588	0.781	Hendy et a	2008	19909	Mollusca	7805	Gastropod
Late Pleistocene		0.126	0.0117	Sellards	1917	9011	Mollusca	7805	Gastropod
Late Pleistocene		0.126	0.0117	Sellards	1917	9011	Mollusca	7805	Gastropod
Late Pleistocene		0.126	0.0117	Sellards	1917	9011	Mollusca	7805	Gastropod
Early Pleistocene		2.588	0.781	DuBar	1962	11119	Mollusca	7805	Gastropod
Early Pleistocene		2.588	0.781	DuBar	1962	11119	Mollusca	7805	Gastropod
Early Pleistocene		2.588	0.781	DuBar	1962	11119	Mollusca	7805	Gastropod
Early Pleistocene		2.588	0.781	DuBar	1962	11119	Mollusca	7805	Gastropod
Calabrian		1.806	0.781	DuBar	1962	11119	Mollusca	7805	Gastropod
Calabrian		1.806	0.781	DuBar	1962	11119	Mollusca	7805	Gastropod
Late Pleistocene		0.126	0.0117	DuBar	1962	11119	Mollusca	7805	Gastropod
Early Pleistocene		2.588	0.781	DuBar	1962	11119	Mollusca	7805	Gastropod
Early Pleistocene		2.588	0.781	DuBar	1962	11119	Mollusca	7805	Gastropod
Late Pleistocene		0.126	0.0117	DuBar	1962	11119	Mollusca	7805	Gastropod
Pliocene	Pleistocene	5.333	0.0117	Landau and	2010	36423	Mollusca	7805	Gastropod
Pliocene	Pleistocene	5.333	0.0117	DuBar	1962	11119	Mollusca	7805	Gastropod
Late Pliocene		3.6	2.588	Mansfield	1930	17060	Mollusca	7805	Gastropod
Late Pliocene		3.6	2.588	Mansfield	1930	17060	Mollusca	7805	Gastropod
Late Pliocene		3.6	2.588	Mansfield	1930	17060	Mollusca	7805	Gastropod
Late Pliocene		3.6	2.588	Mansfield	1930	17060	Mollusca	7805	Gastropod
Late Pliocene		3.6	2.588	Mansfield	1930	17060	Mollusca	7805	Gastropod
Late Pliocene		3.6	2.588	Mansfield	1930	17060	Mollusca	7805	Gastropod
Late Pliocene		3.6	2.588	Mansfield	1930	17060	Mollusca	7805	Gastropod
Late Pliocene		3.6	2.588	Mansfield	1930	17060	Mollusca	7805	Gastropod
Early Pleistocene		2.588	0.781	Portell et a	1992	17634	Mollusca	7805	Gastropod
Early Pleistocene		2.588	0.781	Portell et a	1992	17634	Mollusca	7805	Gastropod
Early Pleistocene		2.588	0.781	Portell et a	1992	17634	Mollusca	7805	Gastropod
Early Pleistocene		2.588	0.781	Portell et a	1992	17634	Mollusca	7805	Gastropod
Middle Pleistocene		0.781	0.126	Portell et a	1992	17634	Mollusca	7805	Gastropod
Middle Pleistocene		0.781	0.126	Portell et a	1992	17634	Mollusca	7805	Gastropod
Middle Pleistocene		0.781	0.126	Portell et a	1992	17634	Mollusca	7805	Gastropod
Middle Pleistocene		0.781	0.126	Portell et a	1992	17634	Mollusca	7805	Gastropod
Late Pleistocene		0.126	0.0117	Portell et a	1992	17634	Mollusca	7805	Gastropod
Late Pleistocene		0.126	0.0117	Portell et a	1992	17634	Mollusca	7805	Gastropod
Late Pleistocene		0.126	0.0117	Portell et a	1992	17634	Mollusca	7805	Gastropod
Late Pleistocene		0.126	0.0117	Hendy et a	2008	19909	Mollusca	7805	Gastropod
Late Pleistocene		0.126	0.0117	Hendy et a	2008	19909	Mollusca	7805	Gastropod
Late Pleistocene		0.126	0.0117	Hendy et a	2008	19909	Mollusca	7805	Gastropod
Early Pleistocene		2.588	0.781	Schroeder	1954	25303	Mollusca	7805	Gastropod
Pliocene		5.333	2.588	Schroeder	1954	25303	Mollusca	7805	Gastropod

Pliocene	5.333	2.588	Schroeder	1954	25303	Mollusca	7805	Gastropod
Pliocene	5.333	2.588	Schroeder	1954	25303	Mollusca	7805	Gastropod
Pliocene	5.333	2.588	Schroeder	1954	25303	Mollusca	7805	Gastropod
Early Pleistocene	2.588	0.781	Schroeder	1954	25303	Mollusca	7805	Gastropod
Pliocene	5.333	2.588	Schroeder	1954	25303	Mollusca	7805	Gastropod
Early Pleistocene	2.588	0.781	Schroeder	1954	25303	Mollusca	7805	Gastropod
Early Pleistocene	2.588	0.781	Schroeder	1954	25303	Mollusca	7805	Gastropod
Pliocene	5.333	2.588	Schroeder	1954	25303	Mollusca	7805	Gastropod
Pliocene	5.333	2.588	Schroeder	1954	25303	Mollusca	7805	Gastropod
Pliocene	5.333	2.588	Schroeder	1954	25303	Mollusca	7805	Gastropod
Early Pleistocene	2.588	0.781	Olsson anc	1953	25463	Mollusca	7805	Gastropod
Early Pleistocene	2.588	0.781	Olsson anc	1953	25463	Mollusca	7805	Gastropod
Early Pleistocene	2.588	0.781	Olsson anc	1953	25463	Mollusca	7805	Gastropod
Early Pleistocene	2.588	0.781	Olsson anc	1953	25463	Mollusca	7805	Gastropod
Early Pleistocene	2.588	0.781	Olsson anc	1953	25463	Mollusca	7805	Gastropod
Early Pleistocene	2.588	0.781	Olsson anc	1953	25463	Mollusca	7805	Gastropod
Early Pleistocene	2.588	0.781	Olsson anc	1953	25463	Mollusca	7805	Gastropod
Early Pleistocene	2.588	0.781	Olsson anc	1953	25463	Mollusca	7805	Gastropod
Early Pleistocene	2.588	0.781	Olsson anc	1953	25463	Mollusca	7805	Gastropod
Late Pleistocene	0.126	0.0117	Lovejoy	1992	26806	Mollusca	7805	Gastropod
Late Pleistocene	0.126	0.0117	Petuch anc	2007	26813	Mollusca	7805	Gastropod
Middle Pleistocene	0.781	0.126	Petuch anc	2007	26813	Mollusca	7805	Gastropod
Middle Pleistocene	0.781	0.126	Petuch anc	2007	26813	Mollusca	7805	Gastropod
Middle Pleistocene	0.781	0.126	Petuch anc	2007	26813	Mollusca	7805	Gastropod
Middle Pleistocene	0.781	0.126	Petuch anc	2007	26813	Mollusca	7805	Gastropod
Middle Pleistocene	0.781	0.126	Petuch anc	2007	26813	Mollusca	7805	Gastropod
Middle Pleistocene	0.781	0.126	Petuch anc	2007	26813	Mollusca	7805	Gastropod
Early Pleistocene	2.588	0.781	Petuch anc	2007	26813	Mollusca	7805	Gastropod
Early Pleistocene	2.588	0.781	Petuch anc	2007	26813	Mollusca	7805	Gastropod
Early Pleistocene	2.588	0.781	Petuch anc	2007	26813	Mollusca	7805	Gastropod
Middle Pleistocene	0.781	0.126	Petuch anc	2007	26813	Mollusca	7805	Gastropod
Middle Pleistocene	0.781	0.126	Petuch anc	2007	26813	Mollusca	7805	Gastropod
Middle Pleistocene	0.781	0.126	Petuch anc	2007	26813	Mollusca	7805	Gastropod
Middle Pleistocene	0.781	0.126	Petuch anc	2007	26813	Mollusca	7805	Gastropod
Middle Pleistocene	0.781	0.126	Petuch anc	2007	26813	Mollusca	7805	Gastropod
Middle Pleistocene	0.781	0.126	Petuch anc	2007	26813	Mollusca	7805	Gastropod
Middle Pleistocene	0.781	0.126	Petuch anc	2007	26813	Mollusca	7805	Gastropod
Late Pliocene	3.6	2.588	Olsson anc	1964	26836	Mollusca	7805	Gastropod
Late Pliocene	3.6	2.588	Olsson anc	1964	26836	Mollusca	7805	Gastropod
Late Pliocene	3.6	2.588	Olsson anc	1964	26836	Mollusca	7805	Gastropod
Middle Pleistocene	0.781	0.126	Olsson anc	1964	26836	Mollusca	7805	Gastropod
Early Pleistocene	2.588	0.781	Petuch anc	2007	26813	Mollusca	7805	Gastropod
Late Plioce Early Pleist	3.6	0.781	Petuch anc	2007	26813	Mollusca	7805	Gastropod
Late Plioce Early Pleist	3.6	0.781	Petuch anc	2007	26813	Mollusca	7805	Gastropod
Late Plioce Early Pleist	3.6	0.781	Petuch anc	2007	26813	Mollusca	7805	Gastropod
Late Plioce Pleistocen	3.6	0.0117	Petuch anc	2007	26813	Mollusca	7805	Gastropod
Early Pleistocene	2.588	0.781	Hendy et a	2008	19909	Mollusca	7805	Gastropod

Late Pliocene	3.6	2.588	Petuch anc	2007	26813	Mollusca	7805	Gastropod
Late Pliocene	3.6	2.588	Petuch anc	2007	26813	Mollusca	7805	Gastropod
Late Pliocene	3.6	2.588	Petuch anc	2007	26813	Mollusca	7805	Gastropod
Late Pliocene	3.6	2.588	Petuch anc	2007	26813	Mollusca	7805	Gastropod
Early Pleistocene	2.588	0.781	DuBar	1958	25514	Mollusca	7805	Gastropod
Late Pleistocene	0.126	0.0117	DuBar	1958	25514	Mollusca	7805	Gastropod
Late Pleistocene	0.126	0.0117	Hendy et a	2008	19909	Mollusca	7805	Gastropod
Late Pleistocene	0.126	0.0117	Hendy et a	2008	19909	Mollusca	7805	Gastropod
Late Pliocene	3.6	2.588	Hendy et a	2008	19909	Mollusca	7805	Gastropod
Late Pliocene	3.6	2.588	Hendy et a	2008	19909	Mollusca	7805	Gastropod
Early Pleistocene	2.588	0.781	Hendy et a	2008	19909	Mollusca	7805	Gastropod
Early Pleistocene	2.588	0.781	Hendy et a	2008	19909	Mollusca	7805	Gastropod
Early Pleistocene	2.588	0.781	Hendy et a	2008	19909	Mollusca	7805	Gastropod
Middle Pleistocene	0.781	0.126	Hendy et a	2008	19909	Mollusca	7805	Gastropod
Middle Pleistocene	0.781	0.126	Hendy et a	2008	19909	Mollusca	7805	Gastropod
Pleistocene	2.588	0.0117	Drez	1981	31281	Mollusca	7805	Gastropod
Late Pliocene	3.6	2.588	Petuch	1988	31112	Mollusca	7805	Gastropod
Middle Pleistocene	0.781	0.126	Petuch	1988	31112	Mollusca	7805	Gastropod
Middle Pleistocene	0.781	0.126	Petuch	1988	31112	Mollusca	7805	Gastropod
Middle Pleistocene	0.781	0.126	Petuch	1988	31112	Mollusca	7805	Gastropod
Middle Pleistocene	0.781	0.126	Petuch	1988	31112	Mollusca	7805	Gastropod
Late Pliocene	3.6	2.588	Weisbord	1971	39743	Mollusca	7805	Gastropod
Late Pleistocene	0.126	0.0117	Kittle and I	2010	40884	Mollusca	7805	Gastropod
Late Pleistocene	0.126	0.0117	Kittle and I	2010	40884	Mollusca	7805	Gastropod
Late Pleistocene	0.126	0.0117	Kittle and I	2010	40884	Mollusca	7805	Gastropod
Late Pleistocene	0.126	0.0117	Kittle and I	2010	40884	Mollusca	7805	Gastropod
Late Pleistocene	0.126	0.0117	Hulbert an	1998	1962	Mollusca	7805	Gastropod
Late Pleistocene	0.126	0.0117	Hulbert an	1998	1962	Mollusca	7805	Gastropod
Late Pleistocene	0.126	0.0117	Hulbert an	1998	1962	Mollusca	7805	Gastropod
Late Pleistocene	0.126	0.0117	DuBar	1971	17556	Mollusca	7805	Gastropod
Late Pleistocene	0.126	0.0117	DuBar	1971	17556	Mollusca	7805	Gastropod
Late Pleistocene	0.126	0.0117	DuBar	1971	17556	Mollusca	7805	Gastropod
Late Pleistocene	0.126	0.0117	DuBar	1971	17556	Mollusca	7805	Gastropod
Late Pleistocene	0.126	0.0117	DuBar	1971	17556	Mollusca	7805	Gastropod
Late Pleistocene	0.126	0.0117	DuBar	1971	17556	Mollusca	7805	Gastropod
Late Pleistocene	0.126	0.0117	DuBar	1971	17556	Mollusca	7805	Gastropod
Late Pleistocene	0.126	0.0117	DuBar	1971	17556	Mollusca	7805	Gastropod
Zanclean	5.333	3.6	Campbell a	1995	17558	Mollusca	7805	Gastropod
Early Pleistocene	2.588	0.781	DuBar	1971	17556	Mollusca	7805	Gastropod
Late Pleistocene	0.126	0.0117	Richards	1962	18974	Mollusca	7805	Gastropod
Late Pleistocene	0.126	0.0117	Richards	1962	18974	Mollusca	7805	Gastropod
Late Pleistocene	0.126	0.0117	Richards	1962	18974	Mollusca	7805	Gastropod
Late Pleistocene	0.126	0.0117	Richards	1962	18974	Mollusca	7805	Gastropod
Late Pleistocene	0.126	0.0117	Richards	1962	18974	Mollusca	7805	Gastropod
Late Pleistocene	0.126	0.0117	Hendy et a	2008	19909	Mollusca	7805	Gastropod
Late Pleistocene	0.126	0.0117	Hendy et a	2008	19909	Mollusca	7805	Gastropod
Late Pleistocene	0.126	0.0117	Hendy et a	2008	19909	Mollusca	7805	Gastropod

Late Pleistocene	0.126	0.0117	Hendy et a	2008	19909	Mollusca	7805	Gastropod
Late Pleistocene	0.126	0.0117	Hendy et a	2008	19909	Mollusca	7805	Gastropod
Late Pleistocene	0.126	0.0117	Hendy et a	2008	19909	Mollusca	7805	Gastropod
Late Pleistocene	0.126	0.0117	Hendy et a	2008	19909	Mollusca	7805	Gastropod
Pleistocene	2.588	0.0117	Hendy et a	2008	19909	Mollusca	7805	Gastropod
Pleistocene	2.588	0.0117	Hendy et a	2008	19909	Mollusca	7805	Gastropod
Pleistocene	2.588	0.0117	Hendy et a	2008	19909	Mollusca	7805	Gastropod
Late Pleistocene	0.126	0.0117	Cooke	1936	31145	Mollusca	7805	Gastropod
Early Pleistocene	2.588	0.781	Cooke	1936	31145	Mollusca	7805	Gastropod
Early Pleistocene	2.588	0.781	Cooke	1936	31145	Mollusca	7805	Gastropod
Early Pleistocene	2.588	0.781	Cooke	1936	31145	Mollusca	7805	Gastropod
Early Pleistocene	2.588	0.781	Cooke	1936	31145	Mollusca	7805	Gastropod
Late Pliocene	3.6	2.588	Ward and I	1987	16700	Mollusca	7805	Gastropod
Late Pliocene	3.6	2.588	Ward and I	1987	16700	Mollusca	7805	Gastropod
Late Pliocene	3.6	2.588	Ward and I	1987	16700	Mollusca	7805	Gastropod
Late Pliocene	3.6	2.588	Ward and I	1987	16700	Mollusca	7805	Gastropod
Early Pleistocene	2.588	0.781	Ward and I	1987	16700	Mollusca	7805	Gastropod
Late Pliocene	3.6	2.588	Gardner	1948	17048	Mollusca	7805	Gastropod
Late Pliocene	3.6	2.588	Gardner	1948	17048	Mollusca	7805	Gastropod
Early Pleistocene	2.588	0.781	Miller	1990	17561	Mollusca	7805	Gastropod
Late Pleistocene	0.126	0.0117	Richards	1962	18974	Mollusca	7805	Gastropod
Late Pleistocene	0.126	0.0117	Richards	1962	18974	Mollusca	7805	Gastropod
Late Pleistocene	0.126	0.0117	Richards	1962	18974	Mollusca	7805	Gastropod
Late Pleistocene	0.126	0.0117	Richards	1962	18974	Mollusca	7805	Gastropod
Late Pleistocene	0.126	0.0117	Richards	1962	18974	Mollusca	7805	Gastropod
Late Pleistocene	0.126	0.0117	Richards	1962	18974	Mollusca	7805	Gastropod
Late Pleistocene	0.126	0.0117	Richards	1962	18974	Mollusca	7805	Gastropod
Late Pleistocene	0.126	0.0117	Richards	1962	18974	Mollusca	7805	Gastropod
Late Pleistocene	0.126	0.0117	Richards	1962	18974	Mollusca	7805	Gastropod
Late Pleistocene	0.126	0.0117	Richards	1962	18974	Mollusca	7805	Gastropod
Late Pleistocene	0.126	0.0117	Richards	1962	18974	Mollusca	7805	Gastropod
Late Pleistocene	0.126	0.0117	Richards	1962	18974	Mollusca	7805	Gastropod
Late Pleistocene	0.126	0.0117	Richards	1962	18974	Mollusca	7805	Gastropod
Late Pleistocene	0.126	0.0117	Hendy et a	2008	19909	Mollusca	7805	Gastropod
Late Pleistocene	0.126	0.0117	Hendy et a	2008	19909	Mollusca	7805	Gastropod
Gelasian	2.588	1.806	Bailey and	1986	23840	Mollusca	7805	Gastropod
Late Pleistocene	0.126	0.0117	Hendy et a	2008	19909	Mollusca	7805	Gastropod
Late Pliocene	3.6	2.588	Hendy et a	2008	19909	Mollusca	7805	Gastropod
Late Pliocene	3.6	2.588	Hendy et a	2008	19909	Mollusca	7805	Gastropod
Late Pliocene	3.6	2.588	Hendy et a	2008	19909	Mollusca	7805	Gastropod
Pliocene	5.333	2.588	Hendy et a	2008	19909	Mollusca	7805	Gastropod
Pliocene	5.333	2.588	Hendy et a	2008	19909	Mollusca	7805	Gastropod
Late Pliocene	3.6	2.588	Hendy et a	2008	19909	Mollusca	7805	Gastropod
Late Pliocene	3.6	2.588	Hendy et a	2008	19909	Mollusca	7805	Gastropod
Late Pliocene	3.6	2.588	Hendy et a	2008	19909	Mollusca	7805	Gastropod
Early Pleistocene	2.588	0.781	Hendy et a	2008	19909	Mollusca	7805	Gastropod
Early Pleistocene	2.588	0.781	Hendy et a	2008	19909	Mollusca	7805	Gastropod

Pleistocene		2.588	0.0117	Coch	1971	441	Mollusca	7805	Gastropod
Late Pleistocene		0.126	0.0117	Spencer ar	1987	15525	Mollusca	7805	Gastropod
Late Pleistocene		0.126	0.0117	Spencer ar	1987	15525	Mollusca	7805	Gastropod
Late Pliocene		3.6	2.588	Edwards et	2005	17631	Mollusca	7805	Gastropod
Late Pleistocene		0.126	0.0117	Hendy et a	2008	19909	Mollusca	7805	Gastropod
Late Pliocene		3.6	2.588	Ward	1993	26351	Mollusca	7805	Gastropod
Late Pliocene		3.6	2.588	Ward	1993	26351	Mollusca	7805	Gastropod
Late Pliocene		3.6	2.588	Ward	1993	26351	Mollusca	7805	Gastropod
Late Pliocene		3.6	2.588	Ward	1993	26351	Mollusca	7805	Gastropod
Late Pliocene		3.6	2.588	Ward	1993	26351	Mollusca	7805	Gastropod
Late Pliocene		3.6	2.588	Ward	1993	26351	Mollusca	7805	Gastropod
Late Pliocene		3.6	2.588	Ward	1993	26351	Mollusca	7805	Gastropod
Late Pliocene		3.6	2.588	Ward	1993	26351	Mollusca	7805	Gastropod
Late Pleistocene		0.126	0.0117	Hendy et a	2008	19909	Mollusca	7805	Gastropod
Late Pliocene		3.6	2.588	Hendy et a	2008	19909	Mollusca	7805	Gastropod
Middle Pleistocene		0.781	0.126	McGinty	1970	19114	Mollusca	7805	Gastropod
Middle Pleistocene		0.781	0.126	Petuch anc	2007	26813	Mollusca	7805	Gastropod
Middle Pleistocene		0.781	0.126	Petuch anc	2007	26813	Mollusca	7805	Gastropod
Late Plioce	Early Pleist	3.6	0.781	Petuch anc	2007	26813	Mollusca	7805	Gastropod
Late Plioce	Pleistocene	3.6	0.0117	Petuch anc	2007	26813	Mollusca	7805	Gastropod
Late Pliocene		3.6	2.588	Petuch anc	2007	26813	Mollusca	7805	Gastropod
Late Pliocene		3.6	2.588	Petuch anc	2007	26813	Mollusca	7805	Gastropod
Late Pliocene		3.6	2.588	Petuch anc	2007	26813	Mollusca	7805	Gastropod
Late Pliocene		3.6	2.588	Petuch anc	2007	26813	Mollusca	7805	Gastropod
Late Pliocene		3.6	2.588	Petuch anc	2007	26813	Mollusca	7805	Gastropod
Middle Pleistocene		0.781	0.126	Petuch	1988	31112	Mollusca	7805	Gastropod
Late Pleistocene		0.126	0.0117	Kittle and I	2010	40884	Mollusca	7805	Gastropod
Late Pleistocene		0.126	0.0117	Kittle and I	2010	40884	Mollusca	7805	Gastropod
Pliocene		5.333	2.588	Petuch	1986	47440	Mollusca	7805	Gastropod
Pliocene		5.333	2.588	Petuch	1986	47440	Mollusca	7805	Gastropod
Pliocene		5.333	2.588	Petuch	1986	47440	Mollusca	7805	Gastropod
Early Pleistocene		2.588	0.781	Dolin	1991	57818	Mollusca	7805	Gastropod
Late Pleistocene		0.126	0.0117	Richards	1962	18974	Mollusca	7805	Gastropod
Late Pleistocene		0.126	0.0117	Richards	1962	18974	Mollusca	7805	Gastropod
Middle Pleistocene		0.781	0.126	Hoerle	1970	19536	Mollusca	7805	Gastropod
Early Pleistocene		2.588	0.781	Olsson anc	1953	25463	Mollusca	7805	Gastropod
Early Pleistocene		2.588	0.781	DuBar	1962	11119	Mollusca	7805	Gastropod
Early Pleistocene		2.588	0.781	DuBar	1962	11119	Mollusca	7805	Gastropod
Pliocene	Pleistocene	5.333	0.0117	DuBar	1962	11119	Mollusca	7805	Gastropod
Irvingtonian		1.8	0.3	Hulbert an	1989	1960	Mollusca	7805	Gastropod
Middle Pleistocene		0.781	0.126	Portell et a	1992	17634	Mollusca	7805	Gastropod
Late Pleistocene		0.126	0.0117	Portell et a	1992	17634	Mollusca	7805	Gastropod
Early Pleistocene		2.588	0.781	DuBar	1958	25514	Mollusca	7805	Gastropod
Late Pleistocene		0.126	0.0117	DuBar	1958	25514	Mollusca	7805	Gastropod
Late Pleistocene		0.126	0.0117	Kittle and I	2010	40884	Mollusca	7805	Gastropod
Early Pleistocene		2.588	0.781	Cooke	1936	31145	Mollusca	7805	Gastropod
Late Pleistocene		0.126	0.0117	Guilday	1962	35381	Mollusca	7805	Gastropod

Late Pliocene	3.6	2.588	Mansfield	1930	17060	Mollusca	7805	Gastropod
Late Pliocene	3.6	2.588	Ward	1993	26351	Mollusca	7805	Gastropod
Late Pliocene	3.6	2.588	Ward	1993	26351	Mollusca	7805	Gastropod
Late Pliocene	3.6	2.588	Ward	1993	26351	Mollusca	7805	Gastropod
Late Pliocene	3.6	2.588	Ward	1993	26351	Mollusca	7805	Gastropod
Early Pleistocene	2.588	0.781	DuBar	1962	11119	Mollusca	7805	Gastropod
Early Pleistocene	2.588	0.781	DuBar	1962	11119	Mollusca	7805	Gastropod
Calabrian	1.806	0.781	DuBar	1962	11119	Mollusca	7805	Gastropod
Early Pleistocene	2.588	0.781	DuBar	1962	11119	Mollusca	7805	Gastropod
Early Pleistocene	2.588	0.781	DuBar	1962	11119	Mollusca	7805	Gastropod
Early Pleistocene	2.588	0.781	DuBar	1962	11119	Mollusca	7805	Gastropod
Pliocene Pleistocen	5.333	0.0117	DuBar	1962	11119	Mollusca	7805	Gastropod
Irvingtonian	1.8	0.3	Hulbert an	1989	1960	Mollusca	7805	Gastropod
Early Pleistocene	2.588	0.781	Portell et a	1992	17634	Mollusca	7805	Gastropod
Early Pleistocene	2.588	0.781	Portell et a	1992	17634	Mollusca	7805	Gastropod
Middle Pleistocene	0.781	0.126	Portell et a	1992	17634	Mollusca	7805	Gastropod
Middle Pleistocene	0.781	0.126	Portell et a	1992	17634	Mollusca	7805	Gastropod
Middle Pleistocene	0.781	0.126	Portell et a	1992	17634	Mollusca	7805	Gastropod
Late Pleistocene	0.126	0.0117	Portell et a	1992	17634	Mollusca	7805	Gastropod
Late Pleistocene	0.126	0.0117	Portell et a	1992	17634	Mollusca	7805	Gastropod
Late Pleistocene	0.126	0.0117	DuBar	1958	25514	Mollusca	7805	Gastropod
Early Pleistocene	2.588	0.781	Hendy et a	2008	19909	Mollusca	7805	Gastropod
Early Pleistocene	2.588	0.781	DuBar	1958	25514	Mollusca	7805	Gastropod
Late Pleistocene	0.126	0.0117	DuBar	1958	25514	Mollusca	7805	Gastropod
Early Pleistocene	2.588	0.781	DuBar	1958	25514	Mollusca	7805	Gastropod
Late Pleistocene	0.126	0.0117	Kittle and I	2010	40884	Mollusca	7805	Gastropod
Late Pleistocene	0.126	0.0117	Kittle and I	2010	40884	Mollusca	7805	Gastropod
Late Pleistocene	0.126	0.0117	Kittle and I	2010	40884	Mollusca	7805	Gastropod
Late Pleistocene	0.126	0.0117	Kittle and I	2010	40884	Mollusca	7805	Gastropod
Late Pleistocene	0.126	0.0117	Kittle and I	2010	40884	Mollusca	7805	Gastropod
Late Pleistocene	0.126	0.0117	Kittle and I	2010	40884	Mollusca	7805	Gastropod
Late Pleistocene	0.126	0.0117	Kittle and I	2010	40884	Mollusca	7805	Gastropod
Late Pleistocene	0.126	0.0117	Kittle and I	2010	40884	Mollusca	7805	Gastropod
Late Pleistocene	0.126	0.0117	Kittle and I	2010	40884	Mollusca	7805	Gastropod
Late Pleistocene	0.126	0.0117	Kittle and I	2010	40884	Mollusca	7805	Gastropod
Late Pleistocene	0.126	0.0117	Kittle and I	2010	40884	Mollusca	7805	Gastropod
Pleistocene	2.588	0.0117	Auffenberg	2006	82817	Mollusca	7805	Gastropod
Pleistocene	2.588	0.0117	Auffenberg	2006	82817	Mollusca	7805	Gastropod
Pleistocene	2.588	0.0117	Auffenberg	2006	82817	Mollusca	7805	Gastropod
Pleistocene	2.588	0.0117	Auffenberg	2006	82817	Mollusca	7805	Gastropod
Pleistocene	2.588	0.0117	Auffenberg	2006	82817	Mollusca	7805	Gastropod
Pleistocene	2.588	0.0117	Auffenberg	2006	82817	Mollusca	7805	Gastropod
Pleistocene	2.588	0.0117	Auffenberg	2006	82817	Mollusca	7805	Gastropod
Pleistocene	2.588	0.0117	Auffenberg	2006	82817	Mollusca	7805	Gastropod
Pleistocene	2.588	0.0117	Auffenberg	2006	82817	Mollusca	7805	Gastropod
Pleistocene	2.588	0.0117	Auffenberg	2006	82817	Mollusca	7805	Gastropod
Pleistocene	2.588	0.0117	Auffenberg	2006	82817	Mollusca	7805	Gastropod

Pleistocene	2.588	0.0117	Auffenberg	2006	82817	Mollusca	7805	Gastropod
Pleistocene	2.588	0.0117	Auffenberg	2006	82817	Mollusca	7805	Gastropod
Pleistocene	2.588	0.0117	Auffenberg	2006	82817	Mollusca	7805	Gastropod
Pleistocene	2.588	0.0117	Auffenberg	2006	82817	Mollusca	7805	Gastropod
Pleistocene	2.588	0.0117	Auffenberg	2006	82817	Mollusca	7805	Gastropod
Pleistocene	2.588	0.0117	Auffenberg	2006	82817	Mollusca	7805	Gastropod
Pleistocene	2.588	0.0117	Auffenberg	2006	82817	Mollusca	7805	Gastropod
Pleistocene	2.588	0.0117	Auffenberg	2006	82817	Mollusca	7805	Gastropod
Pleistocene	2.588	0.0117	Auffenberg	2006	82817	Mollusca	7805	Gastropod
Pleistocene	2.588	0.0117	Auffenberg	2006	82817	Mollusca	7805	Gastropod
Pleistocene	2.588	0.0117	Auffenberg	2006	82817	Mollusca	7805	Gastropod
Pleistocene	2.588	0.0117	Auffenberg	2006	82817	Mollusca	7805	Gastropod
Pleistocene	2.588	0.0117	Auffenberg	2006	82817	Mollusca	7805	Gastropod
Pliocene	5.333	2.588	Aldrich	1911	53829	Mollusca	7805	Gastropod
Early Pleistocene	2.588	0.781	Cooke	1936	31145	Mollusca	7805	Gastropod
Middle Pleistocene	0.781	0.126	Portell et a	1992	17634	Mollusca	7805	Gastropod
Late Pleistocene	0.126	0.0117	Kittle and I	2010	40884	Mollusca	7805	Gastropod
Late Pleistocene	0.126	0.0117	Kittle and I	2010	40884	Mollusca	7805	Gastropod
Late Pleistocene	0.126	0.0117	Kittle and I	2010	40884	Mollusca	7805	Gastropod
Pleistocene	2.588	0.0117	Auffenberg	2006	82817	Mollusca	7805	Gastropod
[truncated: 3,437,069 more chars]
